# Analysis of the Political Viewpoint of Policy Statements From Professional Medical Organizations Using ChatGPT With GPT-4: Cross-Sectional Study

**DOI:** 10.2196/66204

**Published:** 2025-06-13

**Authors:** Ben Knudsen, Amr Madkour, Preetam Cholli, Alyson Haslam, Vinay Prasad

**Affiliations:** 1 The George Washington University School of Medicine and Health Sciences Washington, DC United States; 2 Department of Obstetrics and Gynecology The George Washington University Washington, DC United States; 3 Department of Internal Medicine Emory University Atlanta, GA United States; 4 Department of Epidemiology and Biostatistics University of California San Francisco San Francisco, CA United States

**Keywords:** medical organizations, health policy, policy statements, public health, ChatGPT, AI, chatbot, political viewpoint, political alignment, political ideology

## Abstract

**Background:**

Professional medical organizations publish policy statements that are used to impact legislation or address societal issues. Many organizations are nonpartisan, yet it is uncertain whether their policy statements balance liberal and conservative values.

**Objective:**

This study aims to evaluate the political viewpoint of policy statements from 6 influential medical organizations, including the American Academy of Pediatrics, American College of Surgeons, American Psychiatric Association, American College of Obstetricians and Gynecologists, American College of Physicians, and American Academy of Family Physicians.

**Methods:**

Between December 2023 and February 2024, policy statements from the 6 organizations were identified and evaluated using ChatGPT with GPT-4 to reduce bias. Each statement was pasted into a new ChatGPT session following the phrase “Does this text align with a liberal or conservative viewpoint?” Two authors reviewed each response and categorized the statement as liberal, probably liberal, neutral, probably conservative, or conservative.

**Results:**

One-third of policy statements (529/1592, 33.2%) were found to be aligned with a political ideology. Among these 529 statements, 516 (97.5%) were liberal or probably liberal and 13 (2.5%) were conservative or probably conservative. For each organization, among policy statements with a political leaning, the percentage of liberal or probably liberal statements was as follows: 100% (69/69) for the American Academy of Pediatrics, 100% (24/24) for the American College of Obstetricians and Gynecologists, 100% (12/12) for the American College of Surgeons, 99% (72/73) for the American Psychiatric Association, 97% (174/180) for the American Academy of Family Physicians, and 96% (165/171) for the American College of Physicians.

**Conclusions:**

One in 3 policy statements from these 6 professional organizations align with a partisan political viewpoint. Among these, positions are 40 times more likely to be liberal or probably liberal than conservative or probably conservative. Whether or not organizations are politically neutral and seek viewpoint diversity warrants further exploration.

## Introduction

### Background

Professional medical organizations have guided the medical community for well over a century and continue to evolve in size, function, and influence [1]. Currently, in addition to creating clinical guidelines, holding conferences [2], and publishing research [3], organizations develop policy and position statements that are used to advocate for legislative changes [4]. Political advocacy often occurs in the form of lobbying [5] and, to a lesser extent, contributions to political candidates or parties [6].

In 2023, among lobbyists for health professionals, the American Medical Association (AMA) was the top donor at US $21,215,000, followed by the American Academy of Family Physicians (AAFP) with US $3,152,917 in contributions [5]; thus, some medical organizations have leverage to impact legislation and engage in political activities.

Although some overlap between politics and medicine is unavoidable, there have been growing concerns, most notably during the COVID-19 pandemic [7-9], about clinical and public health recommendations becoming associated with a political party. Global polls suggest that 2 in 3 people worry about the politicization of medical science [10]. This has resulted in widespread effects, ranging from growing distrust among the public toward federal and other public health institutions, as well as scientific experts, to increased emotional burden and stress among practicing physicians during patient encounters [11].

Several major medical organizations, such as the AMA [12], American Academy of Pediatrics (AAP) [13,14], American College of Surgeons (ACS) [15], American College of Physicians (ACP) [16], American College of Obstetricians and Gynecologists (ACOG) [17], and others, label themselves as nonpartisan entities while simultaneously publishing policy statements that center on political issues. It is uncertain whether these policy statements are nonpartisan or bipartisan, given that some medical organizations have recently (and historically) [18] taken stances on important political issues [19], including climate change [20-24] and universal healthcare coverage [25-27]. This may indicate a preference for policies favored by one political party.

### Objectives

Given the influence and involvement of medical organizations in politics, their endorsement of political issues, and the negative effects of the politicization of medicine, we sought to characterize the political viewpoint (liberal vs conservative) of all policy statements from the following 6 prominent medical organizations using ChatGPT with GPT-4 (OpenAI): AAP, ACS, ACOG, ACP, AAFP, and American Psychiatric Association (APA). Our analysis seeks to provide the first descriptive analysis of the political alignment of policy preferences of medical societies. To reduce potential bias and more objectively ascertain the political leaning of each statement, a third-party method—ChatGPT—was used to code the political valence of all statements.

## Methods

### Overview

We used ChatGPT with GPT-4 to analyze the political alignment of policy and position statements (referred to as policy statements) from 6 medical organizations, including AAP, ACS, APA, ACOG, ACP, and AAFP. ChatGPT is a chatbot designed to understand input presented by users and provide a humanlike text response.

We limited our analysis to flagship organizations for medical specialties that are considered core rotations in medical school (pediatrics, surgery, psychiatry, obstetrics and gynecology, internal medicine, and family medicine). This cross-sectional study was performed in accordance with the STROBE (Strengthening the Reporting of Observational Studies in Epidemiology) statement.

### Policy Statement Inclusion Criteria

We identified all policy statements listed on the websites of the 6 organizations from December 2023 to February 2024. The following bulleted list describes the digital location of the policy statements that were retrieved for each medical organization:

- For the AAP, policy statements available under the Policy Statements section [28] through December 2023 were included. Documents under the following headings were not included: Clinical Reports, Clinical Practice Guidelines, AAP News, Statements of Endorsement, Technical Reports, or AAP Policy Books.
- For the ACS, statements available under the Statements heading [29] through December 2023 were included.
- For the APA, position statements under the Policy Finder section [30] through December 2023 were included.
- For the ACOG, reports under the headings Statements of Policy and Position Statements [31] through December 2023 were included.
- For the ACP, policy statements located in the Policy Compendium [32] through December 2023 were included. The Policy Compendium is a collection of “policy statements of a national scope adopted by the ACP Board of Regents” and listed as an “up-to-date summary of ACP’s policy positions.” Other policy statements or papers from the ACP were not considered.
- For the AAFP, policies, position statements, and position papers located in the full policy listing [33] through February 2024 were included.

Policy statements that were duplicates, old versions, or unable to be viewed or evaluated were excluded. For the AAP, definitions, prevention guidelines, AAP retired or reaffirmed, and vaccine schedule updates were excluded. The name, date (most recent), and status of each policy statement were obtained. Many policy statements were a revision of a prior statement. In these cases, the most recent version was used. Older versions were not readily available.

### Political Viewpoint Determination

The whole text of each policy statement (excluding references and acknowledgments) was pasted into a new ChatGPT session (with prior queries having been deleted) following the phrase “Does this text align with a liberal or conservative viewpoint?” The full ChatGPT response was recorded. Safeguards were used to prevent ChatGPT from being influenced by prior queries. Specifically, the first ChatGPT output was always used; the option to regenerate a response was never selected; previous ChatGPT sessions were always deleted and never present when performing the analysis for each subsequent policy statement; and after each policy statement evaluation, the ChatGPT session was deleted, and a new session was initiated.

ChatGPT was used to evaluate political alignment to reduce bias that may be present in human reviewers and for its ability to quickly analyze long and detailed documents. In addition, ChatGPT has performed highly on various professional and academic examinations [34] and is growing in popularity in medical research [35]. It is reported that ChatGPT may have a left-leaning political bias [36-38]. However, newer versions of ChatGPT [38,39], specifically GPT 3.5-turbo and GPT 4, have shown less political bias than earlier models. Despite ChatGPT’s observed political bias, our methodology did not prompt ChatGPT to provide its own opinion; rather, we queried ChatGPT to match ideas in a text to well-established political values, which likely bypassed ChatGPT’s inherent political preferences. Motoki et al [36] showed that ChatGPT was capable of correctly identifying liberal and conservative values, which is the foundation of our study.

ChatGPT responses did not always definitively label a statement as either liberal or conservative. Thus, each ChatGPT response was analyzed independently and separately by 2 authors (BK and AM), and the policy statement was determined to be one of the following: liberal, probably liberal, neutral, probably conservative, or conservative. If both authors agreed, the political classification was assigned to the policy statement. If the 2 authors disagreed, a third author (AH) independently analyzed and assigned a political label to the statement. Then, the third author’s political classification was compared to that of the first 2 authors (BK and AM). The final classification was determined to be the political category that received the most votes. For example, if BK labeled a statement as liberal and AM labeled a statement as probably liberal, a third author, AH, evaluated the statement. If AH found the statement to be liberal, then the liberal category had received 2 votes, and the final classification for that policy statement was liberal.

Full transcripts of the ChatGPT results are available in Multimedia Appendix 1. The following bullet points provide further explanation regarding the criteria used to categorize the ChatGPT outputs:

- Liberal or conservative—the ChatGPT output clearly defined the political alignment of the policy statement. ChatGPT described principles in the policy statement that were associated with either liberal or conservative views. The explanation was nonambiguous. The explanation did not indicate that any part of the policy statement aligned with the opposing political perspective.
- Probably liberal or probably conservative—the ChatGPT output was nuanced and did not obviously indicate that the policy statement was strictly liberal or conservative. The ChatGPT explanation indicated that some, but not all, ideas in the policy statement were associated with a political ideology. ChatGPT recognized that some ideas in the policy statement aligned with both liberal and conservative views while also suggesting that 1 ideology was more apparent and discussed with more importance.
- Neutral—ChatGPT clearly indicated that the policy statement was neutral, and its contents were not political in nature. The ChatGPT output described that the policy statement contained both liberal and conservative ideologies but did not report that 1 set of ideas was more prominent.

As a quality assurance check for ChatGPT outputs, random policy statements were read by the authors after the ChatGPT query to ensure the ChatGPT responses were representative of the policy statement.

Textbox 1 contains examples of parts of the ChatGPT output that were key to categorizing the statement into a political category. Policy statements were not evaluated based on their original content but were assessed based on their associated ChatGPT output.

**Examples of key elements of ChatGPT output that were used for classifying the political alignment of policy statements.**

**Liberal**

- “The text you provided appears to align more with a liberal viewpoint. This conclusion is based on several key aspects of the text...”
- “Key aspects that suggest a liberal alignment include...”
- “The text provided primarily aligns with a liberal viewpoint...”
- “The text you provided expresses a viewpoint that is generally aligned with liberal or progressive ideologies. This alignment is evident in several key aspects...”
- “The text you provided presents a viewpoint that is more commonly associated with liberal ideologies, although it’s important to note that such categorizations can vary and are not always clear-cut. Here are some key reasons why this text aligns more closely with a liberal viewpoint...”
- “Conservative viewpoints, in contrast, might emphasize...”

**Probably liberal**

- “Overall, while the text does not explicitly align with a strictly liberal or conservative viewpoint, its emphasis on community involvement, addressing social determinants of health, and advocating for systemic changes in public health policy might resonate more with liberal perspectives...”
- “In summary, while the text itself is focused on educational criteria and does not explicitly endorse a political ideology, some of the values it promotes—such as a focus on underserved communities, inclusivity, and a comprehensive approach to healthcare—might resonate more with ideologies typically associated with progressive or liberal viewpoints...”
- “Thus, while the text aligns with principles often associated with liberal viewpoints, the call for transparency in healthcare transcends traditional political boundaries and can be supported by individuals with a wide range of political beliefs...”
- “Therefore, while some elements of the text may align more closely with liberal viewpoints, particularly in terms of regulatory and public health perspectives, the overall concern for reducing the impact of tobacco use is a bipartisan issue that transcends traditional liberal-conservative divides...”

**Neutral**

- “The text you provided doesn’t align distinctly with either a liberal or conservative viewpoint...”
- “The content is neutral and objective, presenting facts and guidelines without any discernible bias towards a liberal or conservative viewpoint...”
- “However, the text you’ve provided is centered around the structure and philosophy of care rather than explicit political arguments or policy prescriptions...”
- “The text you provided primarily aligns with a viewpoint that emphasizes patient autonomy, informed consent, and ethical considerations in healthcare, which are principles that can be appreciated across the political spectrum...”
- “In summary, the text discusses healthcare and legal reform in a way that doesn’t neatly fit into a liberal or conservative box. It seems to prioritize practical solutions and systemic improvement over ideological positions...”
- “In essence, while the text does not neatly fit into a single political ideology, elements of it can be appreciated or critiqued from both liberal and conservative perspectives...”

**Probably conservative**

- “The text primarily aligns with principles that could be appreciated by both sides for different reasons but leans more towards a conservative viewpoint...”
- “The ACP’s stance against mandatory Medicare assignment by states might not align neatly with traditional liberal views, as it advocates for less state control over Medicare policies...”
- “In essence, the ACP’s position might be seen as aligning more with a conservative viewpoint on regulatory issues, advocating for professional discretion over government mandates...”

**Conservative**

- “The text you’ve provided appears to align with a more conservative viewpoint...”
- “The text reflects a conservative viewpoint towards healthcare professional roles and scope of practice...”
- “This stance aligns more closely with a perspective that values structured oversight, professional standards, and integration within traditional healthcare frameworks, which can be seen as conservative in the context of healthcare policy...”
- “The text you provided represents a viewpoint that is generally aligned with conservative principles...”

Here is an example of how an ambiguous case was evaluated. The following is the ChatGPT output for an ACP policy statement titled “Proper Use of Accepted Drugs”:

The text expresses a viewpoint advocating for physician autonomy and clinical judgment in determining the proper usage of approved drugs, rather than strict legislative or administrative controls. This perspective aligns more with principles typically associated with a liberal viewpoint, which often emphasizes individual professional judgment and less restrictive government intervention in professional practices. However, some aspects of this stance could be embraced by conservatives who support limited government regulation in professional affairs. Ultimately, the alignment might vary based on the broader context of healthcare policies and the specific beliefs of individuals within each ideological group.

This statement was classified as probably liberal. There is a blurry and unclear delineation between liberal and conservative values. The output suggests that both viewpoints may align with parts of the policy statement depending on specific beliefs. However, given the emphasis on liberal values, such as individual professional judgment and less restrictive government intervention in professional practices, it was determined that overall, the policy statement was probably liberal.

### Data Analysis

Among all medical organizations and for each individual organization, the percentage of statements categorized as liberal, probably liberal, neutral, probably conservative, or conservative was calculated.

Descriptive statistics are provided. Graphs were created using Excel (Microsoft Corporation). The κ coefficient was found using R (version 4.2.2; R Foundation for Statistical Computing) to determine the degree of agreement between the political categories assigned to policy statements by the 2 reviewers.

### Secondary Analysis: ChatGPT Reliability

Identical queries and the words used to prompt ChatGPT have been shown to influence its output; thus, a secondary analysis was performed from December 2024 to January 2025 using the following scenarios to evaluate ChatGPT’s reliability. In total, 150 random policy statements were reanalyzed using ChatGPT or Grok (xAI) with either the same prompt or an alternative prompt. This is approximately 10% of all policy statements, and this number was chosen for feasibility purposes. Grok is a chatbot with capabilities similar to ChatGPT but differs in various ways that include the developer, philosophy, and integration. In addition to its popularity and accessibility, Grok was chosen due to the possibility that it has a conservative bias, as its founder, Elon Musk, has recently been associated with the Republican party. However, this is speculative, and studies have indicated that Grok has less political bias than ChatGPT. Nonetheless, Grok is a chatbot that may exhibit a conservative leaning and thus was selected as a comparator to ChatGPT. The following list describes 3 scenarios used in the secondary analysis to evaluate ChatGPT reliability:

1. Policy statements were requeried in ChatGPT with the original prompt (“Does this text align with a liberal or conservative viewpoint?”).
2. Policy statements were requeried in ChatGPT with an alternative prompt (“Please describe if you think the following statement leans toward a politically liberal or conservative stance.”). This entire scenario was repeated twice with the same policy statements to gather more data.
3. Policy statements were requeried in Grok with the original prompt.

All safeguards to protect against ChatGPT bias as described in the Political Viewpoint Determination section were used. In addition, memory (this is a feature within ChatGPT settings that allows the program to learn from user responses) was cleared and disabled to prevent any prior queries within ChatGPT from influencing the output for the policy statement evaluations. A political category (liberal, probably liberal, neutral, probably conservative, or conservative) was then assigned to the policy statement by BK (classification from the original analysis was hidden). The political label was then compared to the original analysis.

The percentage of policy statements with the same political label was calculated. For statements with discordant results, the statement from the secondary analysis was characterized as either more liberal, less liberal, more conservative, or less conservative compared to the original analysis by BK. For example, if a statement was labeled neutral in the original analysis and liberal or probably liberal in the secondary analysis, it was placed in the more liberal category. Alternatively, if a statement was labeled as liberal in the original analysis and probably liberal or neutral in the secondary analysis, it was placed in the less liberal category.

### Ethical Considerations

In accordance with 45 Code of Federal Regulations §46.102 (f), this study was not submitted for institutional review board approval because it involved publicly available data and did not involve individual patient data. This study did not involve individual patients, and therefore, informed consent was not required.

## Results

### Primary Analysis

Collectively, there were 1672 statements across the 6 high-profile medical organizations evaluated (AAP, ACS, APA, ACOG, ACP, and AAFP). After duplicates, old versions, statements that were unable to be viewed, and others (from AAP) were excluded, 95% (1592/1672) of the policy statements were included in the final analysis, which were either revised or originally published between 1986 and 2023.

For AAP, 91.6% (294/321) of the policy statements published or revised between 2010 and 2023 were included. Of the 294 policy statements, there were 225 (76.5%) neutral and 69 (23.5%) liberal or probably liberal policy statements (Table 1). The κ coefficient between reviewers was 0.841 (*P*<.001). Of liberal or probably liberal policy statements, the most common topics were related to health care (11/69, 16%), social justice (10/69, 15%), and public health (chemical exposure, immunization, nutrition, smoking, etc; 10/69, 15%; Table 2).

**Table 1:** Policy statements from each medical organization stratified by political categorization (liberal, probably liberal, neutral, probably conservative, or conservative).

Medical organization and all combined	Liberal, n (%)	Probably liberal, n (%)	Neutral, n (%)	Probably conservative, n (%)	Conservative, n (%)
American Academy of Pediatrics (n=294)	53 (18)	16 (5.4)	225 (76.5)	0 (0)	0 (0)
American College of Surgeons (n=106)	7 (6.6)	5 (4.7)	94 (88.7)	0 (0)	0 (0)
American Psychiatric Association (n=222)	55 (24.8)	17 (7.7)	149 (67.1)	0 (0)	1 (0.5)
American College of Obstetricians and Gynecologists (n=37)	22 (59)	2 (5)	13 (35)	0 (0)	0 (0)
American College of Physicians (n=524)	142 (27.1)	23 (4.4)	353 (67.4)	2 (0.4)	4 (0.8)
American Academy of Family Physicians (n=409)	143 (35)	31 (7.6)	229 (55)	2 (0.5)	4 (1)
All organizations combined (N=1592)	422 (26.5)	94 (5.9)	1063 (66.8)	4 (0.3)	9 (0.6)

**Table 2:** Themes of liberal and probably liberal policy statements. Policy statements were categorized by political theme based on their content. The number of liberal or probably liberal policy statements from each medical organization is presented, categorized by political topic. A few policy statements did not fall under these categories, and thus, they were excluded from the figure.

Political theme	AAP^a^	ACS^b^	APA^c^	ACOG^d^	ACP^e^	AAFP^f^
Health care (access, financing, efficiency, reform, etc)	11	2	7	5	38	27
Social justice (racial equity, inclusion, diversity, and LGBTQ^g^ equity)	10	2	16	2	13	45
Public health	10	1	6	2	25	20
Reproductive rights	4	1	4	7	4	10
Education and medical training	5	—^h^	—	—	6	12
Criminal justice and crime	—	—	12	2	3	5
Immigration	2	—	5	1	5	5
Substance use	3	2	6	—	2	2
Pharmaceuticals	—	—	—	—	12	1
Firearms	2	1	4	1	2	2
Mental health	1	—	4	—	1	4
Social determinants of health	3	—	1	—	4	2
Clinical care	3	—	1	—	1	4
Climate and environment	2	—	1	1	2	2
COVID-19	—	—	1	1	5	1
Domestic violence	—	1	—	—	1	4
Employee rights	1	1	1	1	—	2
Poverty, welfare, and homelessness	2	—	1	—	—	2
Alternative treatments	—	—	—	—	1	2
Foreign policy and national security	—	—	—	—	3	1

^a^AAP: American Academy of Pediatrics.

^b^ACS: American College of Surgeons.

^c^APA: American Psychiatric Association.

^d^ACOG: American College of Obstetricians and Gynecologists.

^e^ACP: American College of Physicians.

^f^AAFP: American Academy of Family Physicians.

^g^LGBTQ: lesbian, gay, bisexual, transgender, queer.

^h^Not applicable.

For the ACS, 93.8% (106/113) of the policy statements published or revised between 1986 and 2023 were included. Of the 106 policy statements, there were 94 (88.7%) neutral and 12 (11.3%) liberal or probably liberal policy statements (Table 1). The κ coefficient between reviewers was 0.716 (*P*<.001). The topics of some of the liberal or probably liberal policy statements were health care (2/12, 17%), social justice (2/12, 17%), substance use (2/12, 17%), public health (1/12, 8%), reproductive rights (1/12, 8%), firearms (1/12, 8%), and employee rights (1/12, 8%; Table 2).

For the APA, 92.9% (222/239) of the policy statements published or revised between 1993 and 2023 were included. Of the 222 policy statements, there were 149 (67.1%) neutral, 72 (32.4%) liberal or probably liberal, and 1 (0.5%) conservative policy statement (Table 1). The κ coefficient between reviewers was 0.861 (*P*<.001). The conservative policy statement was about opposing cannabis as a treatment for posttraumatic stress disorder. The most common topics among liberal or probably liberal policy statements were social justice (16/72, 22%), criminal justice and crime (12/72, 17%), and health care (7/72, 10%; Table 2).

For the ACOG, 100% (37/37) of the policy statements published or revised between 2016 and 2023 were included. Of the 37 policy statements, there were 13 (35%) neutral and 24 (65%) liberal or probably liberal policy statements (Table 1). The κ coefficient between reviewers was 0.89 (*P*<.001). Among liberal or probably liberal statements, the most common topics were reproductive rights (7/24, 29%) and health care (5/24, 21%; Table 2).

For the ACP, 95.1% (524/551) of the policy statements published or revised between 2008 and 2023 were included. Of the 524 policy statements, there were 353 (67.4%) neutral, 165 (31.5%) liberal or probably liberal, and 6 (1.1%) conservative or probably conservative policy statements (Table 1). The κ coefficient between reviewers was 0.847 (*P*<.001). The most common political issues discussed among liberal or probably liberal policy statements were health care (38/165, 23%), public health (25/165, 15.1%), social justice (13/165, 7.9%), and pharmaceuticals (12/165, 7.3%; Table 2).

For the AAFP, 99.5% (409/411) of the policy statements published or revised between 2008 and 2023 were included. Of the 409 policy statements, there were 229 (55.0%) neutral, 174 (42.5%) liberal or probably liberal, and 6 (1.5%) conservative or probably conservative policy statements (Table 1). The κ coefficient between reviewers was 0.855 (*P*<.001). The most common political issues discussed among liberal or probably liberal policy statements were social justice (45/174, 25.9%), health care (27/174, 15.5%), and public health (20/174, 11.5%; Table 2).

Overall, of the 1592 policy statements, 1063 (66.8%) were neutral, 422 (26.5%) were liberal, and 94 (5.9%) were probably liberal. Of the 1592 policy statements, there were only 9 (0.6%) and 4 (0.3%) conservative and probably conservative policy statements, respectively. Examples of liberal and conservative policy statements with a selection of their ChatGPT output are shown in Multimedia Appendix 2.

### Secondary Analysis

In total, 150 random policy statements were reanalyzed using an identical prompt, an alternative prompt, or Grok and compared to the original analysis (Table 3). Some of the policy statements (50/150, 33.3%) were analyzed twice using the alternative prompt (scenario 3); thus, the analysis included 200 total data points. Overall, the percentage agreement between the 2 analyses ranged from 54% to 66%. Among the policy statements coded differently, the majority (72/80, 90%) were categorized as more liberal in the secondary analysis. There were 0 policy statements that moved from either liberal or probably liberal to conservative or probably conservative or vice versa. Among statements within the “more liberal” group, most policy statements (58/72, 81%) changed from neutral (original analysis) to liberal (secondary analysis). There were 0 neutral statements in the original analysis that changed to conservative or probably conservative. There were 0 statements that were less conservative in the secondary analysis.

**Table 3:** Results from the secondary analysis. Scenarios describe the prompt and chatbot used to reevaluate policy statements. The percentage of policy statements coded with either the same, more liberal, less liberal, or more conservative political label is described. Footnotes explain the prompts and the types of political category change within the groups: more liberal, less liberal, and more conservative.

Scenario	Classification of policy statements relative to the original analysis, n/N (%)			
	Same	More liberal^a^	Less liberal^b^	More conservative^c^
Original prompt^d^—original prompt (ChatGPT)	33/50 (66)	14/17 (82)	3/17 (18)	0/17 (0)
Original prompt—new prompt^e^ (ChatGPT)	30/50 (60)	19/20 (95)	0/20 (0)	1/20 (5)
Original prompt—new prompt (×2; ChatGPT)	27/50 (54)	20/23 (87)	2/23 (9)	1/23 (4)
Original prompt—original prompt (Grok)	30/50 (60)	19/20 (95)	1/20 (5)	0/20 (0)

^a^More liberal: neutral to liberal; neutral to probably liberal; probably liberal to liberal.

^b^Less liberal: liberal to neutral; liberal to probably liberal; probably liberal to neutral.

^c^More conservative: probably conservative to conservative.

^d^Original prompt: Does this text align with a liberal or conservative viewpoint?

^e^New prompt: Please describe if you think the following statement leans toward a politically liberal or conservative stance.

Among 71 policy statements originally coded as liberal or conservative (the most distinct categories), 67 (94%) were coded the same in the secondary analysis. In contrast, among 119 statements originally coded as neutral, 51 (42.9%) were coded the same in the secondary analysis.

## Discussion

### Principal Findings

We found that 33.2% (529/1592) of policy statements from 6 highly regarded medical organizations were nonneutral and correlated with a set of political values. Of these statements, the majority (516/529, 97.5%) were aligned with a liberal or probably liberal viewpoint, whereas only 2.5% (13/529) aligned with a conservative or probably conservative viewpoint. This equates to 40 times more liberal than conservative policy statements.

**Figure 1:**
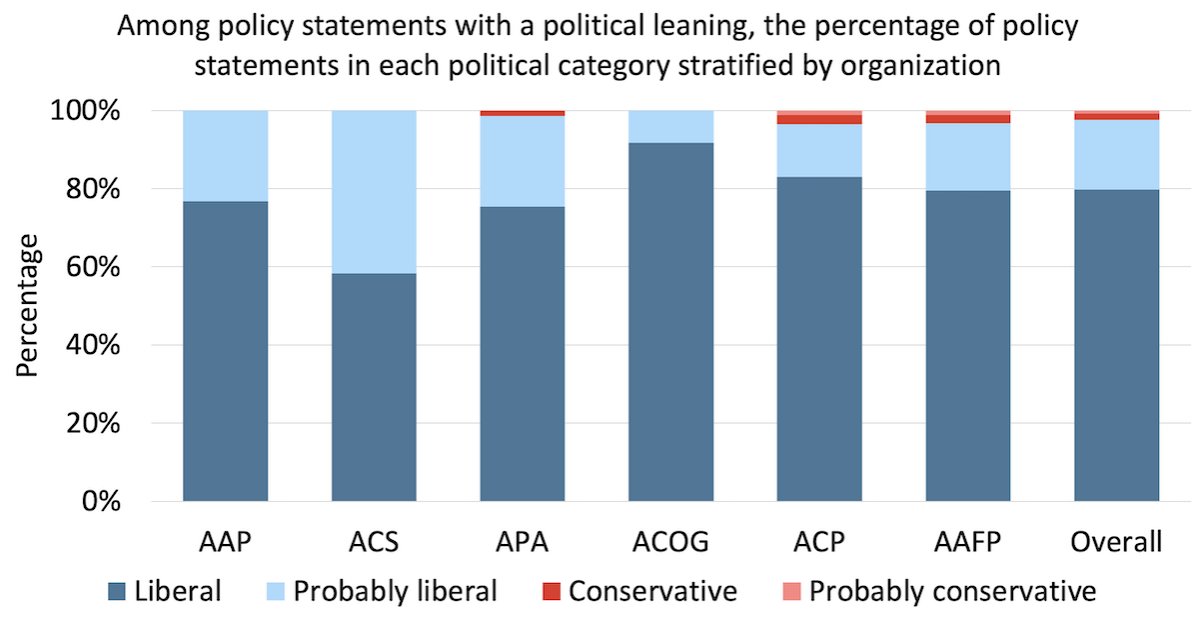
Policy statements that are liberal, probably liberal, conservative, or probably conservative for each medical organization among only policy statements found to have a political alignment. Dark blue depicts liberal, light blue depicts probably liberal, dark red depicts conservative, and light red depicts probably conservative. Blue and red were chosen due to the association with liberal and conservative political ideology, respectively, as represented in modern US politics. AAFP: American Academy of Family Physicians; AAP: American Academy of Pediatrics; ACOG: American College of Obstetricians and Gynecologists; ACP: American College of Physicians; ACS: American College of Surgeons; APA: American Psychiatric Association.

For each organization, among policy statements with a political leaning, the percentage of liberal or probably liberal statements was 100% (69/69) for the AAP, 100% (24/24) for the ACOG, 100% (12/12) for the ACS, 99% (72/73) for the APA, 97% (174/180) for the AAFP, 96% (165/171) for the ACP (Figure 1). The number of conservative or probably conservative policy statements, which totaled 13, were from only 3 of the 6 organizations: the APA, ACP, and AAFP.

Our results may be unexpected, given that physicians from both major political parties are represented within each medical specialty [40,41]. Our results differ and represent an imbalance in favor of liberal values. For example, data published in 2016 in the *New York Times* [41] reported that about 47% of obstetrician and gynecologist physicians are registered as Republican; however, 100% (24/24) of the nonneutral policy statements published by ACOG, the largest organization for this specialty, lean liberal. Similarly, the same report showed that 52% of family medicine physicians are registered as Republican; however, the AAFP published 29 times more liberal or probably liberal than conservative or probably conservative policy statements. The same trend is true for the other organizations included in this study.

Across organizations, there were different proportions of liberal policy statements. For example, the ACOG had the highest (22/37, 59% liberal), while the ACS had the lowest (7/106, 6.6% liberal). There are a few explanations that may account for this difference. First, particular fields may attract individuals with a common political ideology. For example, psychiatry may recruit individuals with a liberal political preference due to the focus on mental health. Second, the nature of the profession may impact and alter the political ideology of an individual. People training and practicing obstetrics and gynecology may become proponents of medical abortion after witnessing the devastating complications of unsafe abortions. Finally, members of the medical organizations or individuals that write policy statements may have a common political ideology that does not capture alternative viewpoints in the field. This reflects limited viewpoint diversity, which some have identified as a problem within the field of public health [42]. Future research could seek to evaluate if causal relationships exist between individuals’ political orientation and policy positions. This could be evaluated within and between medical organizations. Moreover, it may be valuable to understand how political preferences of individuals within specialties change over time and measure how this affects favorability toward policies. As noted earlier, demographics, such as age, gender, background, socioeconomic status, and professional experiences, among others, may influence policy preferences, and additional research exploring these relationships would be an important contribution.

Medical organizations have an extensive history of involvement in politics [43]; however, it is unclear how their politicization has changed over time. A cross-sectional analysis in 2024 found that 64% (35/55) of medical organizations had statements related to gender-affirming care, and 97% of the statements were supportive [44]. Furthermore, a report in 2022 found that 45% (50/111) of the US medical organizations had at least 1 type of content related to climate change on their website [45]. Finally, in our analysis, we found that 67% (4/6) of the organizations had policy statements related to COVID-19 that aligned with a liberal ideology. Although outside the scope of this analysis, it appears that medical organizations may be becoming more politicized, especially because of the COVID-19 pandemic. Research expanding on this work could aim to evaluate the political alignment of policy statements over time to explore how viewpoints have changed.

There are potential upsides and downsides to having medical societies issue policy statements. As a potential benefit, these can catalyze important policy discussions that may lead to improved advocacy efforts and outcomes. For example, pediatricians may rightfully believe that they have a duty to advocate against firearm shootings, given the negative effects on children and society. Psychiatrists may feel compelled by a responsibility to advocate for an improved criminal justice system and legal procedures, given the vulnerability of those with psychiatric disorders and their overrepresentation within the criminal justice system.

For potential downsides, it is unknown whether these statements achieve tangible change, and the effect on public trust, particularly among conservatives, may be impacted. Surveys show a major decline in public confidence in medical experts and organizations over the past 5 decades [46], and specifically in recent years, this decline has been particularly pronounced among Republicans [47,48].

Some may argue that our findings are explained merely by the fact that conservative viewpoints do not seek to maximize or promote public health, and ergo, it is natural that organizations’ policy preferences are liberal. However, we contend that this point of view itself lacks empirical support and may represent the underlying bias present in organizational views. Fundamentally, conservative and liberal policy positions are typically disagreements about the solutions to societal problems, rather than the enumeration of the problems themselves. Indeed, one striking example of this is the Affordable Care Act, which addresses the problems of uninsured Americans with a marketplace solution and was originally offered by the conservative Heritage Foundation. Similar to the Affordable Care Act, there may be other potentially valuable conservative proposals, but excluding these views from professional organizations may result in missed opportunities to advance medicine and public health. Ultimately, our paper merely documents the political leaning of policy statements from high-profile medical societies and does not seek to adjudicate a reason for their alignment or their purpose.

The secondary analysis showed that ChatGPT has variable reliability depending on the content of the policy statement. For example, policy statements coded as liberal or conservative in the original analysis had 94% agreement in the secondary analysis when using the original prompt, an alternative prompt, or a different chatbot, Grok (Table 3). Liberal and conservative values represent opposite ends of the spectrum and embody principles that have minimal overlap. Thus, we found that ChatGPT had minimal variability when reanalyzing policy statements with clear political ideology. In contrast, neutral policy statements had 43% agreement in political coding. Ultimately, this is unsurprising given that policy statements without a uniform ideology often contained numerous political ideas that could be interpreted through a liberal or conservative lens based on the reader. ChatGPT frequently responded with this disclaimer.

Notably, among policy statements with discordant results, 90% (72/80) were coded as more liberal compared to the original analysis. There were 0 neutral statements in the original analysis that were coded as conservative or probably conservative in the secondary analysis. Thus, it is likely that the net vector of bias in these political statements lies in the liberal direction, which aligns with our main findings. Although these findings strengthen the study results, we do not have a concrete answer for why statements in the secondary analysis almost always shifted in the liberal direction relative to the original classification. Admittedly, we are not experts in artificial intelligence and are unfamiliar with the internal coding and processes within ChatGPT or Grok. Thus, it is hard to speculate about the reason for the results. It is possible that this represents an inherent bias that exists within various chatbots. Although the same version of ChatGPT was used in the secondary analysis, it is possible that there were minor changes to the code or internal algorithm that influenced our results. Considering these results, it is possible that our original analysis may have undercounted the number of liberal leaning statements and captured only the lower bound of the total number of politically biased statements.

### Strengths and Limitations

Our study has at least 3 strengths and 5 limitations. To our knowledge, our work is the first to comprehensively evaluate most policy statements from these 6 professional organizations for political alignment. As opposed to selectively picking policy statements to analyze, we sampled most or all (in some cases) policy statements that the organizations published on their website. Second, the use of ChatGPT allowed us to reduce bias that may have been present in human reviewers. Finally, at least 2 independent authors reviewed each output from ChatGPT to determine the final political alignment, and their agreement was highly correlated, indicating high reliability in our classification.

For limitations, we evaluated policy statements from only 6 medical organizations; thus, our results may not be generalizable to other professional groups. Second, we were unable to analyze the trend in the political alignment of policy statements over time. Many policy statements were revisions (or updates) of established policy statements, and the original text was unavailable. Third, we used a single prompt for all ChatGPT sessions and used the first ChatGPT response for our analysis. ChatGPT responses can be variable depending on the text used in each session and can change with repeated, identical queries [36]. Thus, our results are limited to a single prompt and do not account for variability in ChatGPT’s output; however, using a single prompt and only the first ChatGPT output, we maintained a uniform process for all policy statement evaluations. Fourth, most policy statements were long documents that discussed various ideas and covered many topics. It is uncertain if ChatGPT considered all points in each policy statement equally or selectively focused on a few. If the latter is true, then our results may not represent the entire scope of each policy statement. However, there is no reason to suspect that ChatGPT was selective in its analysis. Moreover, some policy statements were read in full by the authors after the ChatGPT query, and the ChatGPT outputs were representative of the policy statements. Finally, some reports have indicated that ChatGPT has a left-leaning bias, which may have influenced our results. However, when ChatGPT’s output was compared to an alternative chatbot, Grok, of all the statements categorized as liberal by ChatGPT, Grok agreed 95% of the time. Although it is possible that Grok is also politically biased, it is unlikely that separate chatbots with different developers would produce almost identical results. Moreover, the secondary analysis showed that ChatGPT and Grok interpreted many more policy statements to be aligned with a liberal ideology than in the main analysis. Thus, we are more confident that political bias in our study was not overestimated.

### Conclusions

One-third of the policy statements from 6 medical organizations champion a political view. When this occurs, the organization’s statement is 40 times more likely to espouse a liberal rather than conservative viewpoint. Policy may inherently be political in nature, but our study shows a strong preference for liberal viewpoints in medical organizations. Notably, many of these organizations explicitly state that they are nonpartisan. It remains to be explored whether these positions primarily reflect alignment with evidence-based policies or instead indicate a lack of viewpoint diversity within the organizations.

## Appendix

Multimedia Appendix 1 Full ChatGPT output for all policy statements analyzed.

Multimedia Appendix 2 Examples of part of the ChatGPT output for liberal and conservative policy statements.

## Abbreviations

AAFP: American Academy of Family Physicians
AAP: American Academy of Pediatrics
ACOG: American College of Obstetricians and Gynecologists
ACP: American College of Physicians
ACS: American College of Surgeons
APA: American Psychiatric Association
STROBE: Strengthening the Reporting of Observational Studies in Epidemiology

## Data Availability

All data generated or analyzed during this study are included in this published article and its supplementary information files. The ChatGPT output for all policy statements analyzed in this study is available in Multimedia Appendix 1.
