# Supplementary material for: Analysis of the Political Viewpoint of Policy Statements From Professional Medical Organizations Using ChatGPT With GPT-4: Cross-Sectional Study
Source: JMIR Form Res. 2025 Jun 13;9:e66204. doi: 10.2196/66204 (PMC12205260; doi:10.2196/66204)
Supplement: Multimedia Appendix 1 [file formative_v9i1e66204_app1.pdf]

Organization	Policy Statement Name	Date	ChatGPT Output	Grade
AAP	Addressing Early Education and Child Care Expulsion	10/30/2023	The text you provided appears to align more with a liberal viewpoint. This conclusion is based on several key aspects of the text:<br><br>1. **Emphasis on Social Equity and Inclusion**: The text highlights issues like systemic racism, implicit bias, and the disproportionate effects of preschool expulsion on children of color, males, children with disabilities, and socioeconomically disadvantaged populations. These concerns are typically more emphasized in liberal ideologies, which tend to focus on social justice and equality.<br><br>2. **Focus on Mental Health and Emotional Well-being**: The text advocates for addressing the emotional and behavioral concerns of young children through the involvement of mental health professionals and emphasizes the importance of nurturing environments. This approach aligns with liberal perspectives that often prioritize mental health and emotional support in education.<br><br>3. **Advocacy for Government and Community Involvement**: The recommendations involve significant roles for pediatricians, government policies, and community resources in addressing the issue of preschool expulsion. This approach is consistent with liberal viewpoints that support active government involvement in social issues and public education.<br><br>4. **Addressing Structural Issues**: The text discusses structural issues like systemic racism and the need for training in early childhood mental health, which is more aligned with a liberal perspective that acknowledges and seeks to address systemic inequalities and structural problems in society.<br><br>In contrast, conservative viewpoints might emphasize more individualistic approaches, personal responsibility, and limited government intervention in such issues. The text, however, leans towards systemic analysis and community-based solutions, which are more characteristic of liberal ideologies.	liberal
AAP	Preventing Excessive Noise Exposure in Infants, Children, and Adolescents	10/21/2023	The text you provided doesn't align distinctly with either a liberal or conservative viewpoint. It is primarily a factual, informational piece focused on the health impacts of noise, particularly noise-induced hearing loss, and its effects on various age groups, including children and adolescents. It discusses the physical and psychological effects of noise exposure and emphasizes the need for greater public awareness and protective measures, especially for children.<br><br>This type of content is more scientific and health-oriented, without clear political or ideological biases. It concentrates on raising awareness about a public health issue, advocating for preventive measures and interventions to reduce noise exposure and protect hearing, particularly in vulnerable populations like children. The emphasis on public health, preventive care, and attention to underserved communities can be of interest to both liberal and conservative audiences, as these are universal health concerns.	neutral

AAP	Medicaid and the Children’s Health Insurance Program: Optimization to Promote Equity in Child and Young Adult Health	10/20/2023	The text from the American Academy of Pediatrics (AAP) regarding Medicaid and the Children’s Health Insurance Program (CHIP) primarily aligns with a liberal viewpoint, given its emphasis on universal access to healthcare, equity, and government-supported health care programs. It advocates for comprehensive, family-centered, and equitable healthcare, with a specific focus on addressing the needs of racial and ethnic minoritized populations and those with complex medical conditions. The text emphasizes the importance of government programs like Medicaid and CHIP in providing healthcare and calls for reforms to enhance these programs, reduce disparities, and improve health outcomes.<br><br>Key aspects that suggest a liberal alignment include:<br><br>1. **Advocacy for Universal Healthcare Access**: The text supports universal eligibility for children's healthcare coverage, a concept more commonly associated with liberal policies.<br><br>2. **Focus on Equity**: Emphasizing equity in healthcare, particularly for racial and ethnic minoritized populations, aligns with liberal values which often prioritize social justice and addressing systemic inequalities.<br><br>3. **Government Involvement in Healthcare**: The liberal viewpoint typically supports stronger government intervention in healthcare, as evidenced by the text's call for foundational changes in Medicaid and CHIP.<br><br>4. **Reducing Structural Racism**: The text’s emphasis on dismantling structural racism within healthcare systems aligns with liberal values that prioritize addressing systemic social issues.<br><br>5. **Standardization of Services and Quality of Care**: The advocacy for national standards in healthcare services and quality is more in line with a liberal perspective that often favors federal oversight for uniformity and equity.<br><br>Conservative viewpoints, on the other hand, generally favor less government intervention in healthcare, more market-based solutions, and individual responsibility for health insurance. The ideas presented in this text are less aligned with such conservative principles.<br><br>It's important to note that these are generalizations and the spectrum of political beliefs is broad and diverse. Individuals may hold a mix of liberal and conservative views on different issues.	liberal
AAP	Recommendations for Prevention and Control of Influenza in Children, 2023–2024	8/29/2023	The text you've provided is a detailed and comprehensive policy statement regarding the recommendations for influenza vaccination and antiviral treatment for children in the 2023-2024 influenza season. It is from the American Academy of Pediatrics and covers various aspects of influenza prevention and management in children, including vaccine composition, high-risk groups, vaccination strategies, and treatment guidelines.<br><br>This text aligns with a scientific and medical perspective rather than a political one. It focuses on public health guidelines based on evidence and research, aiming to provide the best possible care and prevention strategies for children regarding influenza. The recommendations are rooted in clinical evidence and studies, emphasizing the importance of vaccination and treatment for specific groups, and recognizing disparities in health outcomes among different populations.<br><br>The content is neutral and objective, presenting facts and guidelines without any discernible bias towards a liberal or conservative viewpoint. Public health policies like these are generally designed to be inclusive and beneficial for the entire population, regardless of political affiliations. Their primary goal is to protect and improve the health of the community, especially vulnerable groups like children.	neutral

AAP	Principles of Child Health Care Financing	8/28/2023	The text from the American Academy of Pediatrics (AAP) regarding child health financing policies aligns more closely with a liberal viewpoint. This alignment is evident in several key aspects of the text:<br><br>1. **Universal Health Coverage**: The emphasis on ensuring that all children, adolescents, and young adults have affordable access to high-quality comprehensive health care is a principle commonly associated with liberal policies. The idea of universal health coverage, regardless of income, family composition, or immigration status, is a hallmark of progressive health policy.<br><br>2. **Addressing Health Inequities**: The focus on addressing historic and present inequities in health care, and ensuring equitable access to health care for all families, reflects a liberal concern for social justice and equality.<br><br>3. **Government Involvement and Regulation**: The recommendations for strong government involvement in health care, including maintaining Medicaid as an entitlement program, ensuring adequate funding for CHIP, and enforcing national standards for health care benefits, are typical of liberal viewpoints that favor more government regulation and intervention in health care.<br><br>4. **Comprehensive Coverage and Services**: The emphasis on comprehensive health care services, including preventive and wellness services, mental health services, and gender-affirming care, aligns with liberal priorities for inclusive and extensive health care services.<br><br>5. **Focus on Social Determinants of Health (SDOHs)**: Acknowledging and addressing social determinants of health, such as income, family environment, and social conditions, is a principle commonly advocated in liberal health policies.<br><br>6. **Equity in Health Care**: The strong emphasis on promoting equity and addressing longstanding health and health care disparities, especially concerning children of color and marginalized populations, is consistent with liberal values of equality and inclusivity.<br><br>In summary, the AAP's position on child health financing, with its focus on universal coverage, equity, comprehensive services, and significant government involvement, is more aligned with liberal viewpoints. Conservative viewpoints generally advocate for less government intervention, more market-based solutions, and individual responsibility in health care.	liberal
AAP	Corporal Punishment in Schools	8/21/2023	The text provided primarily aligns with a liberal viewpoint. It emphasizes the ineffectiveness and ethical concerns associated with corporal punishment in schools, advocates for its abolition, and promotes nonviolent, alternative forms of behavior management. These perspectives are often associated with liberal ideologies, which tend to emphasize individual rights, equality, and social welfare. The focus on the disproportionate impact of corporal punishment on marginalized groups such as Black students and children with disabilities further aligns with liberal concerns about social justice and equity. The reliance on scientific evidence and recommendations from professional organizations like the American Academy of Pediatrics also reflects a liberal tendency to base policy decisions on expert advice and empirical data. However, it's important to recognize that viewpoints on such issues can vary within political spectrums, and not all individuals or groups within a political ideology will necessarily agree with every aspect of this perspective.	liberal

AAP	The Management of Children and Youth With Pediatric Mental and Behavioral Health Emergencies	8/16/2023	The text you provided focuses on the challenges and recommendations for improving care for children and youth with mental and behavioral health (MBH) emergencies, particularly in the context of emergency departments (EDs). It does not neatly align with a traditional liberal or conservative viewpoint as it is primarily a clinical and policy-oriented discussion.<br><br>The content addresses the following issues:<br><br>1. **Increasing Incidence of MBH Emergencies in Youths:** It notes the rising rates of mental health emergencies among children and youth, including issues like depression, suicide, and behavioral disorders.<br><br>2. **Challenges in Current Health Care Infrastructure:** The text highlights the inadequacies in community and institutional resources for managing such emergencies, leading to a reliance on EDs.<br><br>3. **Disparities in Mental Health Care:** It acknowledges racial and socioeconomic disparities in mental health outcomes and access to care, emphasizing the need for behavioral health equity.<br><br>4. **Strategies for Improvement:** The text suggests several strategies to improve MBH care, including systems-level coordination, better training for ED personnel, and the development of community-based care models.<br><br>5. **Focus on a Multidisciplinary Approach:** The need for a comprehensive, patient-centered approach that includes education, prevention, acute care, and post-discharge follow-up is emphasized.<br><br>The approach is comprehensive and multifaceted, cutting across various aspects of healthcare policy and practice. While some elements, like the focus on disparities and equitable access, may resonate more with liberal viewpoints, the overall discussion is centered on healthcare management and policy rather than political ideology. The emphasis is on improving healthcare delivery and outcomes, a concern that transcends political affiliations.	probably liberal
AAP	Pediatric Organ Donation and Transplantation: Across the Care Continuum	8/16/2023	The text provided appears to be a comprehensive and factual report on mental and behavioral health (MBH) emergencies in children and youth, particularly focusing on the challenges and strategies within emergency departments and the broader healthcare system. This text does not align explicitly with either a liberal or conservative viewpoint. Instead, it approaches the subject from a medical and healthcare perspective, emphasizing the need for improved care, resources, and strategies to address MBH emergencies in pediatric populations.<br><br>The report covers various aspects such as the increasing prevalence of mental health issues in children and youth, the challenges faced by emergency departments in providing adequate care, the disparities in mental health outcomes based on race and other factors, and the need for systemic improvements in healthcare delivery for MBH conditions. These include better training for healthcare professionals, improved infrastructure, more equitable access to care, and enhanced community and institutional support systems.<br><br>Rather than advocating a particular political ideology, the text focuses on the medical, social, and systemic aspects of pediatric MBH care. It highlights the importance of addressing these issues from a healthcare perspective, underlining the need for comprehensive, evidence-based, and equitable approaches to improve outcomes for children and youth facing mental and behavioral health emergencies.	neutral

AAP	Child Pedestrian Safety	6/20/2023	The text provided primarily aligns with a public health and safety perspective rather than a strictly liberal or conservative viewpoint. It focuses on the advancement of pedestrian safety, especially for children, and discusses various strategies and policies aimed at reducing traffic fatalities and injuries. This includes the implementation of pedestrian education, the risks of distracted walking, the benefits of safe routes to school programs, and the Vision Zero strategy.<br><br>The emphasis is on evidence-based approaches to enhance safety and promote equitable mobility, which can be a concern across the political spectrum. However, some aspects, such as the focus on urban design, investment in public infrastructure, and addressing disparities that affect underprivileged and minority communities, might resonate more with a liberal perspective that often emphasizes social equity and public investment in community well-being.<br><br>The advocacy for legislative changes, such as implementing Complete Streets policies and adopting safety technology in vehicles, reflects a proactive approach to public health and safety, which is a universal concern but might be approached differently by different political ideologies. Liberals might focus more on regulatory and infrastructural solutions, while conservatives might emphasize personal responsibility and private sector solutions.<br><br>Overall, the text presents a nuanced approach that intersects with public health, urban planning, and social equity, areas that are of interest to both liberal and conservative viewpoints, but with different emphases and preferred solutions.	neutral
AAP	Protecting Children and Adolescents From Tobacco and Nicotine	4/17/2023	The text you provided appears to be a comprehensive and evidence-based policy statement on tobacco and nicotine use, particularly focusing on the impact on children and adolescents. It outlines various public health recommendations and policy actions to mitigate the harm caused by tobacco and nicotine products.<br><br>This text does not align strictly with either a liberal or conservative viewpoint, as public health concerns, especially those affecting children and adolescents, tend to transcend traditional political boundaries. The emphasis is on scientific evidence, public health, and the welfare of children and adolescents, which are concerns shared across the political spectrum.<br><br>The recommendations focus on regulation, prevention, and treatment related to tobacco and nicotine use, which can be seen as a public health approach rather than a politically motivated one. Both conservative and liberal viewpoints may support aspects of these recommendations, such as the importance of protecting youth and the need for effective public health strategies.<br><br>In summary, the text is aligned more with public health perspectives and evidence-based policy rather than with a specific political ideology.	neutral

AAP	AAP Perspective: Supporting Our Pediatric Advocates	2/27/2023	The text you provided expresses a viewpoint that is generally aligned with liberal or progressive ideologies. This alignment is evident in several key aspects:<br><br>1. **Advocacy for Public Health Policies**: The emphasis on advocating for public health measures such as vaccines, air quality, and seatbelt safety is a common theme in liberal ideologies, which often prioritize government intervention for the sake of public welfare.<br><br>2. **Gun Violence Prevention**: The focus on advancing gun violence prevention legislation, especially in response to mass shootings, aligns with liberal positions which generally advocate for stricter gun control laws.<br><br>3. **Collaboration Across Political Parties**: The text's mention of working with lawmakers from any political party suggests a bipartisan approach, but the context of advancing public health and safety initiatives often leans more towards liberal policies.<br><br>4. **Focus on Health Disparities and Social Determinants of Health**: The acknowledgment of the role of policy and politics in driving health disparities is a perspective more commonly associated with liberal thought, which tends to emphasize the social determinants of health.<br><br>5. **Child Advocacy and Protection**: The emphasis on child advocacy, especially in the context of preventing exploitation and ensuring safety, is a priority typically associated with liberal values, which often focus on protecting vulnerable populations.<br><br>6. **Emphasis on Science and Rational Debate**: The text's emphasis on science, reason, and rational debate in policy-making is a hallmark of liberal ideologies, which often advocate for evidence-based policies.<br><br>7. **Historical Context of Pediatric Advocacy**: The historical overview of pediatric advocacy, especially in the context of social activism for children's rights and labor law reforms, aligns with progressive movements.<br><br>8. **Resilience in Advocacy Despite Setbacks**: The narrative of enduring through legislative and judicial setbacks to achieve long-term goals is reflective of a progressive mindset, emphasizing perseverance in social reform.<br><br>It's important to note that while these themes align more closely with liberal viewpoints, the text also shows a commitment to working across party lines and a focus on the broader goal of improving children's health, which can resonate across the political spectrum.	liberal
AAP	Direct Admission to Hospital for Children in the United States	2/27/2023	The text provided does not align distinctly with either a liberal or conservative viewpoint. It is a factual and technical discussion about hospital admission processes, specifically focusing on the practice of direct admissions for children and adolescents in hospitals. The text presents data, research findings, and recommendations for optimizing the quality and safety of direct admissions in pediatric healthcare settings.<br><br>Such discussions are typically non-partisan and are aimed at improving healthcare practices based on evidence and expert opinion, rather than being influenced by political ideologies. The focus is on practical and logistical aspects of hospital care, patient safety, and efficiency, which are concerns shared across the political spectrum.	neutral
AAP	Access to Critical Health Information for Children During Emergencies: Emergency Information Forms and Beyond	2/20/2023	The text you've provided appears to be a detailed and comprehensive policy statement regarding the Emergency Information Form (EIF) and its use in pediatric emergency medicine. This document seems to focus on the importance of having accessible, up-to-date medical information for children, especially those with chronic or complex medical conditions. It discusses the integration of such information with electronic health records (EHRs) and emphasizes the benefits of improved information sharing in emergency situations, including disaster management.<br><br>This statement does not align explicitly with a liberal or conservative political viewpoint. Instead, it focuses on practical, medical, and technological aspects of healthcare delivery. The emphasis is on improving patient outcomes and care efficiency through better information management and accessibility. This approach is generally aligned with public health and medical best practices, transcending specific political ideologies.<br><br>In healthcare policy, discussions often revolve around how to implement such systems, who bears the cost, and issues of privacy and data security. Different political viewpoints might have varying approaches to these aspects (e.g., government-led initiatives versus	neutral

			private sector solutions, extent of regulation, etc.), but the core idea of improving emergency medical care through better information accessibility is a broadly accepted goal across the political spectrum.	
AAP	Crowding in the Emergency Department: Challenges and Recommendations for the Care of Children	2/20/2023	The text provided does not align specifically with either a liberal or conservative viewpoint in a political sense. It is a factual and analytical piece discussing the issue of emergency department (ED) crowding, particularly in the context of pediatric care. It addresses the causes, effects, and potential solutions for ED crowding without delving into ideological or political arguments.<br><br>The focus is on improving healthcare delivery, patient safety, and experience, as well as reducing healthcare costs. These topics are generally of interest across the political spectrum, although the methods of addressing them can vary based on political views. However, this text stays neutral and focuses on practical and systemic solutions to the problem of ED crowding, rather than advocating for a specific political approach.	neutral
AAP	Drinking Water From Private Wells and Risks to Children	1/30/2023	The text you've provided is a detailed and informative piece on the issues surrounding private wells, water quality, and public health in the United States. It doesn't align explicitly with a liberal or conservative viewpoint. Instead, it focuses on presenting factual information about water sources, potential contaminants, and the responsibilities of well owners, as well as public health recommendations.<br><br>The content is scientific and neutral, aiming to educate and inform about environmental health issues, particularly as they relate to children. It discusses the natural and anthropogenic factors that can affect well water quality, the types of contaminants that can be found in well water, and the health risks associated with these contaminants. It also highlights the limitations of current regulations and the responsibilities of private well owners.<br><br>This approach is characteristic of public health and environmental health literature, which typically focuses on evidence-based information and recommendations rather than advocating for a specific political stance. However, the emphasis on environmental justice, the impacts of climate change, and the need for more comprehensive regulations could be seen as aligning more closely with viewpoints typically associated with liberal ideologies, which often prioritize environmental protection and public health initiatives.<br><br>On the other hand, the text's focus on individual responsibility for well maintenance and water quality testing could resonate with conservative viewpoints that emphasize personal responsibility and limited government intervention.<br><br>In summary, while certain aspects of the text might be more consistent with one political ideology or the other, the overall content is factual and neutral, primarily concerned with public health and safety.	neutral

AAP	The Unique Value Proposition of Pediatric Health Care	1/23/2023	The text you've provided is centered around pediatric health care and does not align strictly with a liberal or conservative viewpoint in the traditional political sense. It focuses on the importance of early childhood health and its long-term effects on individual well-being and societal outcomes. The emphasis is on the value of pediatric care, the impact of social determinants of health, and the role of preventive measures in pediatric health.<br><br>The principles and ideas presented in this text could be interpreted through different political lenses. For instance:<br><br>1. **Liberal Viewpoint**: The emphasis on social determinants of health, the need for high-quality care for all children (including those from diverse racial and ethnic backgrounds and those with special needs), and the focus on upstream prevention and addressing issues like poverty and racism could resonate with liberal ideologies. Liberals often emphasize the role of social factors in health and advocate for comprehensive, accessible, and inclusive health care.<br><br>2. **Conservative Viewpoint**: The discussion of value-based care (outcomes relative to costs), the focus on effective use of resources, and the potential for long-term societal benefits, including economic benefits, might appeal to conservative ideologies. Conservatives often emphasize efficient use of resources, personal responsibility, and long-term fiscal responsibility.<br><br>In summary, the text presents a multifaceted approach to pediatric health care that incorporates elements which can be appreciated across the political spectrum. It doesn't squarely fit into a liberal or conservative category but rather addresses a broad range of issues important to the health and well-being of children and society.	neutral
AAP	The Hospitalized Adolescent	1/23/2023	The text you provided appears to be a comprehensive and detailed policy statement focused on the care of hospitalized adolescents. It covers various aspects such as the effects of hospitalization on adolescents, the importance of confidentiality, legal and ethical issues, and considerations for adolescents facing unique challenges such as those with disabilities, mental health issues, or those involved in the juvenile justice system.<br><br>This statement seems to align more with a liberal viewpoint, primarily due to its emphasis on comprehensive, inclusive care, and attention to issues like confidentiality, legal/ethical matters, and systemic biases including racism. Liberal perspectives often prioritize inclusivity, individual rights, and a focus on addressing systemic issues. The detailed attention to the unique needs of diverse adolescent populations, including LGBTQ+ adolescents and those from various racial and ethnic backgrounds, aligns with liberal values of equality and social justice.<br><br>Moreover, the policy's emphasis on the importance of privacy and the rights of adolescents to participate in their own healthcare decisions, as well as the focus on addressing issues like implicit bias and racism in healthcare, are consistent with liberal priorities of individual autonomy and social equity.<br><br>It's important to note that such a policy statement is primarily focused on providing the best possible care and support for hospitalized adolescents and is grounded in evidence-based medical practices and legal/ethical considerations. While it may align more closely with liberal values, its primary goal is to ensure high-quality, equitable healthcare for adolescents.	probably liberal

AAP	Firearm-Related Injuries and Deaths in Children and Youth: Injury Prevention and Harm Reduction	11/28/2022	The text you provided takes a public health approach to addressing firearm injuries and deaths among children and youth in the United States. It emphasizes harm reduction, anticipatory guidance, community-level interventions, firearm safety regulations, and the need for legislation and research funding. This perspective aligns more closely with a liberal viewpoint for several reasons:<br><br>1. **Focus on Gun Safety and Regulation:** The text advocates for stronger safety regulations for firearms, including safe storage practices, "smart" gun technology, and consumer product regulations. This perspective is more commonly associated with liberal viewpoints, which often support increased regulation and safety measures for firearms.<br><br>2. **Advocacy for Legislation:** The call for legislation, including universal background checks, child access prevention laws, and bans on semiautomatic military-style weapons, aligns with liberal positions that typically favor stricter gun control laws.<br><br>3. **Public Health and Community Approach:** The text approaches firearm injuries and deaths as a public health issue and emphasizes community-level interventions and addressing social determinants of health. This holistic approach to health and safety issues is more characteristic of liberal ideologies, which often emphasize social and community factors in policy making.<br><br>4. **Emphasis on Research and Data:** The text highlights the importance of funding for research on firearm injury prevention. The focus on evidence-based policy and the acknowledgment of the need for more data align with a liberal perspective, which often values scientific research as a basis for policy decisions.<br><br>In summary, the text's emphasis on gun safety regulations, legislative action, a public health approach, and reliance on research and data aligns it more with a liberal viewpoint. Conservative viewpoints, in contrast, often emphasize Second Amendment rights, less regulation on firearms, and may prioritize individual responsibility over community or government interventions in matters of public safety.	liberal
AAP	Optimizing Pediatric Patient Safety in the Emergency Care Setting	10/3/2022	The text you've provided does not inherently align with a liberal or conservative viewpoint. It is a policy statement focused on patient safety in emergency care settings, particularly for pediatric patients. This statement discusses various factors affecting patient safety, such as environmental and human factors, and emphasizes the importance of adopting safety principles, supporting a culture of safety, and implementing best practices in emergency departments.<br><br>The content of this policy statement is primarily focused on healthcare practices and safety protocols, which are generally neutral in terms of political ideology. It addresses practical and technical aspects of medical care rather than ideological positions. The emphasis is on improving patient outcomes, minimizing medical errors, and ensuring effective communication and procedures in emergency settings.<br><br>Such topics usually transcend political lines as they are based on medical research and best practices aimed at improving patient care, rather than being influenced by political beliefs or ideologies.	neutral
AAP	American Academy of Pediatrics Recommendations for the Prevention of Pediatric ATV-Related Deaths and Injuries	9/26/2022	The text you've provided appears to align more with a liberal viewpoint, particularly in its emphasis on regulation and public health considerations. The focus is on documenting the risks associated with all-terrain vehicles (ATVs), especially for children and adolescents, and advocating for stringent safety measures and regulations. This perspective often aligns with liberal ideologies that prioritize government intervention for public safety and health. Conservative viewpoints, in contrast, might emphasize personal responsibility and freedom of choice over government regulations. However, it's important to note that such issues can transcend simple political categorizations, as concerns for child safety and public health are shared across the political spectrum.	liberal
AAP	Immunization Information Systems	9/19/2022	The text provided does not align distinctly with either a liberal or conservative viewpoint. It is a factual and technical exposition on Immunization Information Systems (IISs), focusing on their development, utility, and challenges. The document emphasizes the importance of IISs in tracking vaccination histories, supporting public health efforts, and enhancing the effectiveness of immunization programs. It discusses various aspects such as the history of IIS development, their role in society, the challenges faced by pediatric practices in implementing IIS, and the evolution of IIS in response to public health needs.<br><br>This content is more about public health administration and medical technology rather than political ideology. It addresses practical and operational aspects of immunization programs and does not delve into ideological debates typically associated with liberal or conservative viewpoints. The focus is on improving healthcare quality, data management, and public health outcomes, which are concerns that transcend political lines.	neutral

AAP	Recommendations for Prevention and Control of Influenza in Children, 2022–2023	9/6/2022	The text you provided is a comprehensive policy statement from the American Academy of Pediatrics regarding influenza vaccination and treatment recommendations for the 2022-2023 season. It is a scientific and medical document, focusing on evidence-based practices for the prevention and treatment of influenza in children.<br><br>This statement does not align strictly with either a liberal or conservative viewpoint as it is based on medical research and public health principles rather than political ideology. It is a professional guideline aimed at improving child health outcomes by recommending vaccination and appropriate use of antiviral medications.<br><br>Policy statements like these are typically guided by scientific evidence and best practices in the field of medicine, rather than by political considerations. They are intended to inform healthcare providers, public health practitioners, and policy makers about effective strategies for disease prevention and management, based on the latest available evidence and expert consensus.	neutral
AAP	The Adolescent's Right to Confidential Care When Considering Abortion	8/29/2022	The text presents a viewpoint that aligns more closely with liberal perspectives, particularly in its approach to adolescent reproductive rights and abortion access. Key indicators of this alignment include:<br><br>1. **Advocacy for Confidential Care**: The emphasis on the rights of adolescents to access confidential medical care, including abortion, without mandatory parental involvement is a stance commonly associated with liberal viewpoints. This perspective prioritizes individual autonomy and privacy in health care decisions.<br><br>2. **Opposition to Restrictive Abortion Laws**: The text critiques restrictive abortion laws and the negative impacts these laws have on adolescents, a stance typically associated with liberal ideologies. It highlights the risks and barriers these laws create for young individuals seeking abortion services.<br><br>3. **Support for Judicial Bypass**: The support for judicial bypass procedures, which allow adolescents to seek court approval for an abortion without parental consent, aligns with liberal perspectives that seek to protect individual rights and access to health care services.<br><br>4. **Focus on Comprehensive Health Education**: The advocacy for comprehensive health and sexuality education and the promotion of effective contraception align with liberal approaches to teen pregnancy prevention, emphasizing education and access to resources.<br><br>5. **Acknowledgment of Structural Inequities**: The text discusses the disproportionate impact of restrictive abortion laws on marginalized communities, reflecting a liberal concern for social justice and equity in health care access.<br><br>6. **Professional Societies' Support**: The reference to support from various professional societies, such as the American Medical Association and the American Academy of Pediatrics, for adolescent access to confidential reproductive health care aligns with more progressive views on health care policy.<br><br>This analysis is based on the themes and positions presented in the text, which generally align with liberal ideologies, particularly in the context of reproductive rights, individual autonomy in health care, and social justice considerations.	liberal

AAP	Options Counseling for the Pregnant Adolescent Patient	8/29/2022	The text you provided presents a comprehensive and non-partisan approach to adolescent pregnancy and its management, aligning with principles of medical ethics and patient autonomy. It doesn't neatly fit into a strictly liberal or conservative viewpoint. Instead, it emphasizes the importance of providing adolescents with unbiased, factually accurate information about all pregnancy options, including continuing the pregnancy, adoption, and abortion.<br><br>Key points supporting this neutral and professional stance include:<br><br>1. **Nonjudgmental Counseling:** The emphasis is on providing adolescents with nonjudgmental counseling that respects their autonomy and decision-making capacity.<br><br>2. **Full Range of Options:** The text advocates for informing adolescents about all available options, reflecting a commitment to comprehensive healthcare without favoring one option over others.<br><br>3. **Legal and Ethical Considerations:** It acknowledges the legal and policy landscapes surrounding adolescent pregnancy, particularly regarding access to abortion, without taking a political stance.<br><br>4. **Professional Responsibility:** The focus is on the responsibilities of healthcare professionals, like pediatricians, to provide appropriate care and referrals, irrespective of their personal beliefs.<br><br>5. **Public Health Perspective:** The text approaches teen pregnancy from a public health perspective, highlighting the importance of effective contraception and the impact of societal and economic factors on adolescent pregnancy rates.<br><br>6. **Equity and Access:** There is an emphasis on ensuring equitable access to care for all adolescents, including marginalized groups.<br><br>7. **Adherence to Medical Standards:** The guidelines and recommendations are based on the policies of established medical authorities like the American Academy of Pediatrics.<br><br>In summary, the text balances medical, ethical, and legal considerations, aiming to provide comprehensive and unbiased care to adolescents. It does not advocate for a particular political ideology but rather focuses on the best practices in adolescent health care.	neutral
AAP	Protecting Children From Sexual Abuse by Health Care Professionals and in the Health Care Setting	8/29/2022	The text provided does not align specifically with either a liberal or conservative viewpoint. Rather, it reflects a professional and ethical stance on child protection and the conduct of healthcare professionals, particularly pediatricians. The focus is on the unacceptability of sexual abuse or exploitation of children, especially by healthcare professionals who hold a position of trust. The text emphasizes the need for appropriate training, screening, and procedures within medical institutions to prevent and address sexual abuse, alongside the responsibility of healthcare professionals to maintain proper boundaries with patients. This approach is consistent with general ethical standards and professional responsibilities in healthcare, transcending political viewpoints.	neutral

AAP	Scope of Health Care Benefits for Neonates, Infants, Children, Adolescents, and Young Adults Through Age 26	8/29/2022	The text provided is a comprehensive policy statement focused on updating recommendations for a health care benefit package specifically designed for children through age 26. This statement is based on the fourth edition of Bright Futures guidelines and includes recommendations developed through a private-public partnership between the National Business Group on Health and the Health Resources and Services Administration.<br><br>The policy statement does not straightforwardly align with a purely liberal or conservative viewpoint. However, its emphasis on comprehensive health care coverage for children, including preventive care, facility-based care, and coverage across both public and private health plans, suggests a leaning towards principles often associated with liberal viewpoints. These include a strong role for government in ensuring broad access to healthcare, focus on preventive care, and support for public health initiatives.<br><br>Additionally, the statement references historical context and developments, such as the enactment of Medicaid in 1965 and the Patient Protection and Affordable Care Act of 2010, which are often seen as milestones of progressive healthcare policy. The inclusion of various forms of care, like preventive services, mental health services, and a broad range of pediatric-specific services, reflects a comprehensive approach to healthcare, a principle more commonly associated with liberal policies.<br><br>However, it's important to note that healthcare policy, especially when it concerns children, can transcend traditional partisan lines. The focus on children's health and well-being can be a unifying aspect that garners support across the political spectrum. The text also acknowledges the challenges and complexities of implementing such comprehensive healthcare policies, including state-level variations and the financial implications, which are concerns typically raised in conservative viewpoints.<br><br>In summary, while the statement has elements that align with liberal viewpoints, especially in its comprehensive and inclusive approach to healthcare, it also addresses practical and financial considerations that are often central to conservative perspectives. This blend of approaches underscores the complexity and multifaceted nature of healthcare policy.	probably liberal
AAP	Considerations in the Determination of Medical Necessity in Children: Application to Contractual Language	8/22/2022	The text you've provided is a comprehensive policy statement from the American Academy of Pediatrics. It advocates for consistent medical necessity definitions in health care insurance that reflect the specific needs of children at various developmental stages. The statement emphasizes the importance of considering pediatric-specific factors in medical necessity decisions and the need for health care contracts to address the full spectrum of children's health care needs.<br><br>Regarding its alignment with liberal or conservative viewpoints, the statement is primarily technical and professional, focusing on medical and health care practices for children. It doesn't explicitly align with a political ideology. However, certain aspects of the statement might be interpreted differently based on one's political viewpoint:<br><br>1. **Liberal Viewpoint**: The emphasis on comprehensive health care coverage for children, including those with special needs or from diverse backgrounds, and the call for transparent and equitable health care practices, might resonate more with liberal ideologies. Liberals generally advocate for inclusive and comprehensive health care policies and might support the policy's focus on the unique needs of children.<br><br>2. **Conservative Viewpoint**: Conservatives might appreciate the emphasis on evidence-based and cost-effective medical interventions. They often prioritize fiscal responsibility and might support aspects of the policy that advocate for interventions providing meaningful benefits relative to their costs.<br><br>However, it's important to note that health care policies, especially those concerning children's health, often transcend traditional political divisions. The focus here is on the health and well-being of children, which is a universal concern regardless of political affiliation.	neutral
AAP	Creating Work and Learning Environments Free of Gender-Based Harassment in Pediatric Health Care	8/22/2022	The text you provided aligns more with a liberal viewpoint. It emphasizes the importance of acknowledging and addressing sexual and gender-based harassment, including more subtle forms such as microaggressions, in the medical profession. The focus on inclusivity, recognition of the unique experiences of gender and sexual minorities, and advocacy for zero-tolerance policies against harassment are typically associated with liberal perspectives, which often prioritize social justice, equity, and the protection of marginalized groups. The text also underscores the need for systemic change and accountability, hallmarks of progressive approaches to social issues.<br><br>Conservative viewpoints, on the other hand, might place a greater emphasis on traditional values and personal responsibility, and may be less focused on systemic change or the specific challenges faced by minority groups. While there is recognition across the political spectrum of the need to address harassment, the approach and emphasis can vary significantly.	liberal

AAP	Helmet Use in Preventing Head Injuries in Bicycling, Snow Sports, and Other Recreational Activities and Sports	8/15/2022	The text provided does not inherently align with a strictly liberal or conservative viewpoint. It focuses on the importance of helmet use in recreational sports to prevent head injuries, including traumatic brain injuries (TBIs), among children and youth. The approach recommended is evidence-based and multi-faceted, involving legislation, enforcement, public education, and guidance from clinicians.<br><br>In the context of political viewpoints:<br><br>- **Liberal Viewpoint**: Liberals might support the recommendations as they align with a proactive approach to public health and safety, especially for children. Emphasis on legislation, public education, and government involvement in health initiatives are typically supported by liberal ideologies.<br><br>- **Conservative Viewpoint**: Conservatives might support aspects of the recommendations that focus on personal responsibility, like choosing to wear helmets and parental guidance. However, they might be more skeptical about the need for increased legislation and government involvement, preferring less regulatory approaches.<br><br>Overall, the text is more centered on public health and safety rather than political ideology. It presents a pragmatic approach to a health issue, supported by research and statistics, rather than aligning with a specific political stance.	neutral
AAP	Guiding Principles for Managed Care Arrangements for the Health of Newborns, Infants, Children, Adolescents and Young Adults	7/25/2022	The text you provided does not neatly align with a traditionally liberal or conservative viewpoint in a political sense. It addresses managed care arrangements in healthcare, focusing on effective strategies for delivering and financing healthcare services, especially for children and young adults. The emphasis is on improving quality, access, and cost-efficiency in healthcare, which are concerns shared across the political spectrum.<br><br>The text discusses detailed principles and recommendations for managing healthcare delivery, including the importance of primary and specialty care, transparent administrative processes, and the integration of value-based payment models. These topics are generally more about effective healthcare management and less about political ideology.<br><br>Both liberal and conservative viewpoints might find elements to agree with in this text. For example, conservatives might appreciate the focus on cost-efficiency and value-based payment models, which can align with principles of fiscal responsibility and market-driven solutions. Liberals might support the emphasis on access to quality care for all, including vulnerable populations like children with special health care needs, which aligns with principles of social equity and comprehensive healthcare access.<br><br>Ultimately, the text seems to prioritize effective healthcare management and the well-being of patients, particularly children, over a strictly political stance.	neutral
AAP	Vision and Concussion: Symptoms, Signs, Evaluation, and Treatment	7/18/2022	The text provided is a detailed, factual, and medically oriented discussion about the effects of concussion on vision in children and adolescents, diagnostic methods, and recommended treatments. It does not align with a specific political viewpoint, whether liberal or conservative. This type of content is more focused on medical science and healthcare practice, rather than political ideology.<br><br>It provides a thorough overview of how concussions can impact visual systems in pediatric patients, the importance of early detection and management of these symptoms, and the possible need for referral to specialists. The emphasis is on clinical best practices, patient care, and the implications of these medical conditions on children's daily lives and activities, including their return to school and sports. This focus on healthcare and scientific understanding transcends political viewpoints and is geared towards medical professionals and caregivers.	neutral

AAP	Eliminating Race-Based Medicine	6/27/2022	The text you've provided discusses the problematic nature of race-based medicine and argues for its elimination in favor of a more equitable approach that considers social determinants of health and the impact of racism. It challenges the historical misuse of race as a biological proxy and highlights the need for an evidence-informed approach in clinical decision-making that acknowledges the social construction of race and the systemic inequities leading to health disparities.<br><br>This perspective aligns more closely with a liberal viewpoint, as it emphasizes the need for social justice, equity, and systemic change in healthcare. It recognizes the role of social determinants and structural inequities in health outcomes and advocates for a shift away from traditional race-based medical practices.<br><br>Liberal viewpoints often prioritize addressing systemic inequalities and recognize the complex interplay of social, economic, and environmental factors in individual and community health. This perspective is consistent with those values, advocating for a more holistic and equitable approach to healthcare that moves beyond simplistic and biologically reductionist views of race.<br><br>Conservative viewpoints, on the other hand, might focus more on individual responsibility and less on systemic change. While there is a spectrum of opinions within conservatism, a typical conservative approach might be less inclined to emphasize systemic racism or social determinants of health as primary factors in healthcare disparities.<br><br>It's important to note, however, that these are broad generalizations, and individuals' views may vary widely within these ideological spectrums.	liberal
AAP	Improving Substance Use Prevention, Assessment, and Treatment Financing to Enhance Equity and Improve Outcomes Among Children, Adolescents, and Young Adults	6/27/2022	The text you've shared presents a comprehensive and detailed view on the challenges and recommendations regarding substance use prevention, assessment, and treatment services for children, adolescents, and young adults. It does not neatly align with either a strictly liberal or conservative viewpoint, as the focus is primarily on public health, evidence-based policy, and equitable access to healthcare services.<br><br>Key aspects of this text that are typically associated with more liberal viewpoints include:<br>1. Emphasis on health equity and addressing racial/ethnic disparities in treatment access.<br>2. Advocacy for expanded public insurance coverage, including Medicaid and Children's Health Insurance Program (CHIP), particularly for marginalized groups.<br>3. Support for comprehensive substance use services, including prevention, early intervention, and treatment, as part of health insurance coverage.<br>4. Recognition of social determinants of health and the need for integrated, community-based approaches.<br><br>Elements that might resonate across the political spectrum include:<br>1. Focus on evidence-based interventions and the effectiveness of early prevention and treatment.<br>2. Recognition of the societal and economic impacts of substance use disorders.<br>3. Attention to the need for better integration of behavioral health care in primary care settings.<br>4. Emphasis on efficient use of healthcare resources and funding, including alternative payment models.<br><br>The text primarily revolves around public health concerns, efficient use of healthcare resources, and the need for evidence-based, equitable access to care. It reflects a holistic approach to health policy that seeks to balance the needs of individuals, communities, and the healthcare system.	probably liberal
AAP	Individual Medical Emergencies Occurring at School	6/27/2022	The text provided doesn't align exclusively with either a liberal or conservative viewpoint. It is primarily a factual, policy-oriented statement focusing on emergency medical preparedness in schools, particularly for children with special health care needs and chronic medical conditions. The emphasis is on the importance of schools being equipped and prepared to handle medical emergencies, highlighting the roles of various stakeholders like school health teams, pediatricians, and emergency medical services.<br><br>The content is centered around ensuring the safety and well-being of students in schools, a concern that transcends political ideologies. While different political groups might have varying approaches to funding, staffing, and implementing such policies, the underlying goal of student safety and health is a universal one. The text doesn't advocate for specific political ideologies or policies but rather focuses on practical measures and guidelines to improve emergency medical care in school settings.	neutral

AAP	Policy Statement: Breastfeeding and the Use of Human Milk	6/27/2022	The text you've provided is primarily a policy statement regarding breastfeeding, emphasizing its benefits and recommending practices to support it. This type of content is generally aligned with public health perspectives and is not inherently political in nature. It focuses on the medical, developmental, and public health aspects of breastfeeding, advocating for practices based on scientific research and health outcomes.<br><br>The document does not explicitly align with either a liberal or conservative viewpoint. It is centered around health recommendations from reputable organizations like the American Academy of Pediatrics (AAP) and the World Health Organization (WHO), which base their guidelines on extensive research and evidence.<br><br>However, certain aspects of the policy, such as advocating for societal changes to support breastfeeding (like paid maternity leave or workplace accommodations for breastfeeding), can be interpreted through a political lens. These aspects might be more resonant with liberal viewpoints that typically advocate for more robust social support systems and workplace accommodations. But it's important to note that the core content of the statement – promoting breastfeeding based on its health benefits – is a public health issue rather than a partisan one.	probably liberal
AAP	Sleep-Related Infant Deaths: Updated 2022 Recommendations for Reducing Infant Deaths in the Sleep Environment	6/21/2022	The text you've provided is a comprehensive and factual report on sleep-related infant deaths, including sudden infant death syndrome (SIDS) and other causes. It focuses on presenting data, research findings, and recommendations from the American Academy of Pediatrics for reducing the risk of these tragic occurrences.<br><br>This text does not explicitly align with either a liberal or conservative viewpoint. It is a scientific and medical document aimed at informing and guiding healthcare professionals, parents, and caregivers about best practices for infant sleep safety. The recommendations and data presented are based on research and evidence, not on political or ideological beliefs.<br><br>Public health and safety topics like SIDS prevention often transcend traditional political divides, focusing instead on evidence-based strategies to protect the well-being of individuals, in this case, infants. The report is structured to provide clear, actionable advice to reduce the risk of sleep-related infant deaths, grounded in scientific research and expert consensus.	neutral
AAP	Postnatal Corticosteroids to Prevent or Treat Chronic Lung Disease Following Preterm Birth	5/30/2022	The text you provided appears to be a detailed, evidence-based medical guideline or review regarding the use of postnatal corticosteroids in the treatment of chronic lung disease in preterm infants. It discusses various aspects of corticosteroid therapy, including types of corticosteroids, dosages, routes of administration, and the benefits and risks associated with their use. It also refers to various studies, clinical trials, and policy statements from the American Academy of Pediatrics.<br><br>This text is purely scientific and medical in nature, focusing on neonatology and pediatric care. It does not align with either a liberal or conservative viewpoint in the political sense. Medical guidelines like these are generally based on scientific evidence and clinical trials, and they aim to provide the best possible care based on current knowledge, irrespective of political ideologies.<br><br>The document provides a neutral, fact-based perspective intended to guide healthcare professionals in making informed decisions about the care of preterm infants with chronic lung disease. It emphasizes the importance of individualized patient care and shared decision-making with parents, which are hallmarks of evidence-based medical practice.	neutral
AAP	Guidance for Structuring a Pediatric Intermediate Care Unit	4/18/2022	The text provided does not specifically align with a liberal or conservative viewpoint. It is a detailed policy statement and clinical report update from the American Academy of Pediatrics, focusing on the development and operation of pediatric intermediate care units (IMCUs). This document addresses advances in pediatric medical, surgical, and critical care, and provides guidance for various healthcare professionals involved in pediatric care.<br><br>The content is technical and medically focused, dealing with the management of intermediate care units, their staffing, the patient populations they serve, and payment models for such care. It discusses improvements in patient care, staffing recommendations, and operational guidelines for IMCUs, which are crucial in a healthcare setting but not inherently political or ideologically driven.<br><br>Therefore, it can be said that the document is neutral in terms of political ideology, focusing instead on medical best practices and patient care standards.	neutral

AAP	Health Care Clinicians and Product Promotion by Industry	3/21/2022	The text you've provided appears to be a balanced and nuanced discussion on the relationship between healthcare providers and the medical industry, particularly pharmaceutical and medical device manufacturers. It does not align strictly with either a liberal or conservative viewpoint. Instead, it focuses on the ethical considerations and potential conflicts of interest that may arise in the interactions between healthcare providers and the industry.<br><br>Key points include:<br><br>1. **Importance of Industry Collaboration**: The text acknowledges the necessity of collaboration between healthcare providers and the medical industry in developing and testing medical products.<br><br>2. **Potential Conflicts of Interest**: It highlights the potential divergence of interests between healthcare providers and the industry. The text suggests that even minor relationships with the industry can influence healthcare providers' decisions, which may not always benefit the patient.<br><br>3. **Ethical Responsibility of Healthcare Providers**: There is a strong emphasis on the ethical duty of healthcare providers to recognize and guard against situations and marketing strategies designed to influence their decision-making, especially regarding diagnostic and therapeutic options for patients.<br><br>4. **Awareness and Education**: The text recommends that healthcare providers educate themselves about the ways they might be influenced by the industry and remain vigilant, particularly regarding industry-sponsored continuing medical education and potential biases in journal articles.<br><br>5. **Transparency and Disclosure**: It underscores the importance of disclosing conflicts of interest and calls for clear, detailed descriptions of industry relationships in education and research.<br><br>Overall, the text seems to aim for a balanced perspective, advocating for the benefits of industry collaboration in advancing medical care while cautioning against the risks of undue influence and conflicts of interest. This approach transcends typical political categorizations and focuses more on ethical considerations in the healthcare field.	neutral
AAP	Parental Leave for Residents and Pediatric Training Programs	2/28/2022	The text provided appears to approach the topic of parental leave for medical residents and fellows from a perspective that emphasizes the importance of work-life balance, the health and well-being of trainees and their families, and the need for inclusive and supportive policies. This viewpoint aligns more closely with liberal ideals, which often prioritize social welfare, inclusive policies, and worker rights.<br><br>Liberal viewpoints typically advocate for policies that support the well-being of individuals, including parental leave, and emphasize the importance of accommodating diverse family structures and life circumstances. The text's focus on the benefits of parental leave for both parents and children, the need for equitable and inclusive policies (including for same-sex families and various types of family structures), and the acknowledgment of challenges faced by pregnant and postpartum women in the medical profession are indicative of a liberal stance.<br><br>It's important to note, however, that the concepts of liberal and conservative can vary greatly depending on cultural, national, and individual contexts, and the interpretation of these labels can differ widely. The text seems to be more focused on pragmatic and empathetic considerations rather than adhering strictly to a specific political ideology.	liberal
AAP	Vitamin K and the Newborn Infant	2/22/2022	The text you've provided is a medical statement discussing the use of vitamin K for the prevention of vitamin K deficiency bleeding (VKDB) in newborns. This statement is primarily medical and scientific in nature, focusing on clinical practices, research findings, and public health recommendations. It does not inherently align with a specific political viewpoint, whether liberal or conservative.<br><br>Medical and scientific texts like this one are typically designed to be neutral and fact-based, aiming to provide information based on research and clinical evidence rather than political or ideological beliefs. The discussion about vitamin K in this text relates to its medical effectiveness and public health implications, rather than political or social ideologies.<br><br>It's important to recognize that while health policies and practices can sometimes become politicized or be influenced by political views, the underlying medical science, as presented in this text, is intended to be objective and based on empirical evidence.	neutral

AAP	Pediatric Primary Health Care: The Central Role of Pediatricians in Maintaining Children's Health in Evolving Health Care Models	1/24/2022	The text you provided does not inherently align with a specific liberal or conservative viewpoint. It focuses on the importance of pediatric primary health care (PPHC) and the central role of pediatricians in delivering and leading this care. The text emphasizes the need for continuous, comprehensive, and coordinated care for children, advocating for a patient- and family-centered medical home approach. It also highlights the evolving models of health care delivery, including the integration of telehealth, and the importance of pediatricians in guiding policy innovations in this field.<br><br>The text's emphasis is on the quality and comprehensiveness of health care for children, a topic that generally transcends political leanings. Both liberal and conservative viewpoints can support the importance of effective health care for children, although they might differ in their approaches to healthcare policy and funding. The text does not delve into these political aspects but rather focuses on the medical and care aspects of pediatric health.<br><br>It's important to note that the focus on evidence-based care, holistic health approaches, and inclusion of new technologies like telehealth are modern medical practices and not necessarily indicative of a political stance.	neutral
AAP	COVID-19 Vaccines in Children and Adolescents	12/20/2021	The text you've provided primarily presents factual information and recommendations from authoritative medical organizations like the American Academy of Pediatrics (AAP), the US Food and Drug Administration (FDA), and the Centers for Disease Control and Prevention (CDC). It focuses on the safety and effectiveness of vaccines, particularly COVID-19 vaccines, in children and adolescents, and outlines specific recommendations and guidelines from these organizations.<br><br>This text aligns more with a science-based, public health perspective rather than a specific political viewpoint, whether liberal or conservative. It emphasizes the importance of vaccines in protecting against infectious diseases and the rigorous process involved in evaluating their safety and efficacy. Additionally, it addresses the role of pediatricians in promoting vaccination and providing guidance to patients and their families.<br><br>In the contemporary political landscape, attitudes towards vaccines can sometimes be influenced by political beliefs, but the content of this text is consistent with the consensus in the medical and scientific community, which transcends partisan viewpoints. It is focused on public health and the well-being of children and adolescents, based on evidence and expert recommendations, rather than on political ideologies.	neutral
AAP	Preventing Home Medication Administration Errors	12/1/2021	The text provided offers a detailed analysis of medication administration errors in the home, particularly concerning pediatric patients. It doesn't explicitly align with a traditional liberal or conservative viewpoint as it focuses on healthcare quality improvement and patient safety rather than political ideologies.<br><br>The text addresses various aspects of medication errors, including challenges in health literacy, the complexity of medication schedules, and the risks associated with liquid medications and caregivers with limited English proficiency. It offers comprehensive strategies for reducing medication administration errors, such as enhancing health literacy, improving provider prescribing practices, and leveraging technology.<br><br>In terms of political alignment, the content could be interpreted in various ways depending on the reader's perspective. For instance:<br><br>1. **Liberal Viewpoint**: Liberals might appreciate the emphasis on addressing health literacy disparities and providing support for caregivers with limited English proficiency. The focus on regulatory measures and the role of technology and standardized practices in healthcare aligns with a more interventionist approach to public health, which is often associated with liberal politics.<br><br>2. **Conservative Viewpoint**: Conservatives might focus on the aspects of individual responsibility, such as the importance of caregiver education and the role of personal diligence in medication administration. The emphasis on improving prescribing practices and the potential for technology to streamline healthcare might also resonate with conservative ideals of efficiency and self-reliance in healthcare.<br><br>Ultimately, the text is more centered on medical and healthcare quality improvement practices, transcending simple political categorization. It aims to address a critical public health issue through a multifaceted approach involving healthcare providers, caregivers, and systemic changes in healthcare practices.	neutral

AAP	School-Based Health Centers and Pediatric Practice	10/1/2021	The text provided doesn't explicitly align with either a liberal or conservative viewpoint. It presents factual information and policy recommendations about School-Based Health Centers (SBHCs) without overt political bias. The focus is on the benefits and challenges of SBHCs, their role in improving access to healthcare for school-aged children and adolescents, and how they integrate with the broader healthcare system.<br><br>This approach is typical in scientific and policy discussions, where the goal is to inform and provide evidence-based recommendations rather than to advocate for a specific political stance. The emphasis on providing healthcare access to underserved populations and addressing health disparities could be interpreted as aligning with more progressive or liberal values that prioritize social equity and public health initiatives. However, the text does not explicitly endorse a political ideology. It aims to present a comprehensive view of SBHCs based on research and best practices in healthcare.	neutral
AAP	Recommendations for Prevention and Control of Influenza in Children, 2021–2022	10/1/2021	The text provided is a comprehensive and factual update on the recommendations of the American Academy of Pediatrics regarding influenza vaccine and antiviral medications for children. It's primarily focused on public health guidelines and evidence-based medical practices. The statement does not inherently align with a specific liberal or conservative viewpoint.<br><br>It focuses on:<br>- The routine use of influenza vaccines and antiviral medications in children.<br>- Specific recommendations for various groups, such as high-risk children.<br>- Information on vaccine types, dosages, and schedules.<br>- Considerations regarding co-administration with COVID-19 vaccines.<br><br>Such medical and health guidelines typically transcend political ideologies, aiming instead to provide scientifically-backed recommendations for the health and safety of the population, particularly children in this case. The emphasis is on preventing and treating influenza effectively, rather than promoting a political stance.	neutral
AAP	Telehealth: Improving Access to and Quality of Pediatric Health Care	9/1/2021	The text you provided presents a viewpoint that is more commonly associated with liberal ideologies, although it's important to note that such categorizations can vary and are not always clear-cut. Here are some key reasons why this text aligns more closely with a liberal viewpoint:<br><br>1. **Emphasis on Equity and Access**: The text focuses heavily on ensuring equitable access to healthcare for all children, regardless of their race/ethnicity, financial resources, or geographic location. This aligns with liberal ideologies that prioritize social justice and equal access to resources.<br><br>2. **Addressing Disparities**: The text acknowledges and seeks to address disparities in healthcare, particularly those based on economic and racial factors. This reflects a liberal concern for addressing systemic inequalities and advocating for marginalized communities.<br><br>3. **Support for Telehealth and Innovation**: The text advocates for the use of telehealth as a means to improve access to healthcare, especially for under-resourced populations. Liberals often support the integration of technology and innovation in public services to improve accessibility and efficiency.<br><br>4. **Government Involvement in Healthcare**: The text implies a role for government and policy in ensuring access to healthcare and in supporting telehealth initiatives. Liberal viewpoints typically endorse more government involvement in healthcare to ensure that basic needs are met for all citizens.<br><br>5. **Focus on Children and Adolescents**: The emphasis on the healthcare needs of children and adolescents aligns with liberal priorities on welfare and protection of vulnerable populations.<br><br>It's important to recognize that healthcare policy can be a complex and multifaceted issue, and different aspects of it might align with different political ideologies. Additionally, perspectives on healthcare are evolving, and there is increasing recognition across the political spectrum of the importance of access to healthcare.	liberal

AAP	COVID-19 Vaccines in Children and Adolescents	8/1/2021	The text you provided focuses on the safety and effectiveness of vaccines, as well as recommendations for COVID-19 vaccination in children and adolescents by reputable health organizations like the American Academy of Pediatrics (AAP), the US Food and Drug Administration, and the Centers for Disease Control and Prevention (CDC). This information is presented in a factual and scientific manner, aligning with established medical and scientific consensus.<br><br>As such, it doesn't inherently align with a specific political viewpoint, whether liberal or conservative. It is centered around public health and scientific evidence rather than political ideology. Both liberal and conservative viewpoints can support vaccination based on scientific evidence, though individuals within these groups might have varying opinions on the subject.<br><br>The perception of the text as liberal or conservative might vary among different audiences, depending on their personal beliefs, the political climate, and how they view the role of government and public health authorities in healthcare decisions. However, the content itself is primarily scientific and medical, not political.	neutral
AAP	Trauma-Informed Care in Child Health Systems	8/1/2021	The text you provided does not inherently align with either a liberal or conservative viewpoint as it is primarily a scientific and clinical discussion on Trauma-Informed Care (TIC) in pediatric health. It focuses on understanding and addressing the impacts of early childhood adversities and trauma, and how this knowledge can be applied in healthcare settings to support children, adolescents, and families.<br><br>This approach is rooted in evidence-based research and clinical practice, emphasizing the biological, psychological, and social aspects of trauma and resilience. The text discusses the integration of TIC into pediatric healthcare systems, the importance of understanding the effects of trauma on long-term health, and the need for comprehensive strategies that involve policymakers, legislators, and healthcare organizations.<br><br>While different political ideologies might have varied approaches to healthcare policy and funding, the core content of this text is based on medical and psychological research and practice, which transcends political leanings. It's more about improving healthcare delivery and outcomes for children and adolescents affected by trauma, rather than promoting a specific political ideology.	neutral
AAP	Preventing Childhood Toxic Stress: Partnering With Families and Communities to Promote Relational Health	8/1/2021	The text you've provided appears to be a comprehensive and detailed examination of pediatric care, with a specific focus on the concept of Safe, Stable, and Nurturing Relationships (SSNRs) and their role in mitigating toxic stress and promoting resilience in children. It discusses how early life experiences, both positive and negative, are biologically embedded and impact health, education, and economic outcomes across a person's life span.<br><br>This text can be seen as aligning with viewpoints that are generally supported across a broad political spectrum, as it emphasizes the importance of early childhood development, public health approaches, family and community involvement, and science-based policy formation. However, it does touch on topics that might be more closely associated with certain political viewpoints:<br><br>1. **Emphasis on Community and Public Health:** The focus on public health approaches, including the importance of community involvement and addressing social determinants of health, could be seen as aligning more with liberal viewpoints, which often prioritize community-based solutions and public health initiatives.<br><br>2. **Consideration of Socioeconomic and Racial Factors:** The acknowledgment of widening economic inequities and deeply embedded structural racism as current threats to child well-being, and the discussion of issues like poverty, racism, and social isolation, might resonate more with liberal perspectives, which tend to emphasize systemic issues and social justice.<br><br>3. **Focus on Early Intervention and Prevention:** The emphasis on early intervention, prevention, and a holistic approach to child well-being, including the consideration of mental health and relational aspects, aligns with viewpoints that support comprehensive healthcare and social services.<br><br>4. **Policy Recommendations:** The call for policy changes, public health approaches, and the integration of services across sectors might be more aligned with liberal policy approaches, which often advocate for systemic changes and government-led initiatives in healthcare and social services.<br><br>Overall, while the text does not explicitly align with a strictly liberal or conservative viewpoint, its emphasis on community involvement, addressing social determinants of health, and advocating for systemic changes in public health policy might resonate more with liberal perspectives. However, its focus on family, early childhood development, and prevention could find support across the political spectrum.	probably liberal

AAP	Immunizing Against Hate: Overcoming Asian American and Pacific Islander Racism	7/1/2021	The text you've provided doesn't neatly align with a traditional liberal or conservative viewpoint. It addresses a specific issue - the rise in racism and xenophobic attacks against Asian Americans during the COVID-19 pandemic - in a factual and compassionate manner. The focus is on public health, social justice, and the historical context of racism against Asian Americans, rather than on political ideology.<br><br>It's important to note that discussions about racism and public health can intersect with political views, but the text itself appears more focused on raising awareness about a specific social issue and its impact on health, rather than promoting a partisan viewpoint. It emphasizes the importance of understanding and addressing the effects of racism on health, which is a concern that transcends political boundaries.	neutral
AAP	Promoting Healthy Sexuality for Children and Adolescents With Disabilities	7/1/2021	The text provided appears to be a comprehensive clinical report focused on the sexuality of children and adolescents with developmental disabilities, offering updated information and guidance for pediatric health care providers. It emphasizes the importance of appropriate education, the absence of coercion and violence, and the developmental acquisition of skills to navigate feelings, desires, relationships, and social pressures.<br><br>This text does not align strictly with either a liberal or conservative viewpoint. Instead, it takes a medically informed, inclusive, and evidence-based approach to address the sexual health needs of children and adolescents with disabilities. The report prioritizes the provision of resources and tools for clinical practice, aiming to promote competence in achieving healthy sexuality regardless of physical, cognitive, or socioemotional limitations. It discusses the role of pediatric health care providers in delivering anticipatory guidance and education to all children and youth, including those with disabilities, to help them understand their changing bodies, feelings, and behaviors.<br><br>The approach taken in this report is consistent with contemporary health care principles that prioritize patient-centered care, inclusivity, evidence-based practice, and the rights of individuals with disabilities to receive comprehensive and appropriate health education and services. It reflects a broader, non-partisan commitment to improving the health and well-being of all individuals, particularly those with special health care needs.	neutral
AAP	Transition to a Safe Home Sleep Environment for the NICU Patient	7/1/2021	The text you provided is a detailed and comprehensive medical report focused on the care and management of infants in Neonatal Intensive Care Units (NICUs), with a particular emphasis on safe sleep practices. It does not inherently align with a specific liberal or conservative viewpoint as it is a scientific and medical document. The content is factual, based on medical research and guidelines, and aimed at improving healthcare practices for newborns, especially those with special medical needs.<br><br>This type of text is typical of technical reports or guidelines produced by medical bodies or healthcare institutions. It is designed to inform and guide healthcare professionals in providing the best care for their patients, in this case, infants in NICUs. The report is not political in nature; instead, it focuses on health care standards, practices, and recommendations based on research and clinical evidence.	neutral
AAP	Transition to a Safe Home Sleep Environment for the NICU Patient	7/1/2021	The text provided is a clinical report discussing various aspects of neonatal intensive care, particularly focusing on safe sleep environments for infants, especially those born preterm or with low birth weight. It details the medical and clinical practices recommended for ensuring the safety and health of these infants, including the positions they should be placed in for sleep and the environments that are conducive to their well-being.<br><br>This text is primarily medical and scientific in nature, focusing on healthcare guidelines and practices. It does not align with a specific political viewpoint, whether liberal or conservative. The content is based on medical research and recommendations from healthcare bodies like the American Academy of Pediatrics. It aims to inform healthcare professionals about best practices in neonatal care and does not delve into political or ideological discussions.<br><br>Medical and scientific texts like this are typically designed to be neutral, focusing solely on evidence-based practices and research findings. They are intended for a professional audience, such as healthcare providers, and are not written to advocate for or against any political ideologies.	neutral
AAP	Integrating Web Services/Applications to Improve Pediatric Functionalities in Electronic Health Records	7/1/2021	The text provided is neutral and informative, focusing on the technical and developmental aspects of electronic health records (EHRs) and their use in pediatric care. It does not align explicitly with either a liberal or conservative viewpoint. Instead, it discusses the challenges and opportunities in integrating pediatric functionalities into EHR systems, emphasizing the need for improvements in EHR systems to support pediatric care effectively. The text also highlights the role of web services and applications in enhancing EHR functionalities and the importance of privacy, security, accuracy, and compliance with standards. This approach is more aligned with healthcare policy and technology development rather than being influenced by a specific political ideology.	neutral

AAP	Sudden Death in the Young: Information for the Primary Care Provider	7/1/2021	The text you've provided is a detailed and comprehensive medical article focused on the prevention of sudden cardiac arrest (SCA) and sudden cardiac death (SCD) in children, including athletes and non-athletes. It outlines various strategies for screening, evaluation, and management of the risk of SCA and SCD in young individuals.<br><br>This article does not inherently align with a specific political viewpoint, whether liberal or conservative. It is a medical and scientific document that discusses health policy and medical practices based on research and evidence. The emphasis is on clinical guidelines, medical recommendations, and strategies for prevention of cardiac issues in the pediatric population. Such topics are generally considered non-partisan and are focused on public health rather than political ideology.<br><br>However, if the implementation of these guidelines or the funding of such programs were to be discussed in a political context, different viewpoints might emerge. For example, a liberal viewpoint might advocate for more government involvement and funding in implementing widespread cardiac screening programs in schools, while a conservative viewpoint might emphasize individual responsibility and private sector solutions. But the text itself, as it stands, is neutral and focused on medical science and health care practice.	neutral
AAP	Early Childhood Caries in Indigenous Communities	6/1/2021	The text provided appears to be a comprehensive and factual account of oral health issues in Indigenous children in Canada and the United States, focusing on Early Childhood Caries (ECC). It does not overtly align with a specific liberal or conservative viewpoint. Instead, it presents detailed information on the prevalence, causes, and prevention strategies for ECC among Indigenous populations. The text emphasizes the need for better oral health care and access to dental services for these communities, as well as the importance of early intervention and community-based health promotion initiatives.<br><br>This approach is characteristic of a public health perspective that prioritizes health equity and access to care. While it may resonate more with those who advocate for increased social support and healthcare access – often associated with liberal viewpoints – the emphasis on effective healthcare intervention and community-based solutions can also align with certain conservative perspectives that favor community-level over federal-level solutions.<br><br>Overall, the text appears to be more focused on addressing a significant health issue through a variety of strategies and recommendations rather than promoting a specific political ideology.	neutral
AAP	Ambient Air Pollution: Health Hazards to Children	6/1/2021	The text provided seems to be a comprehensive review of the health impacts of ambient air pollution, focusing on its sources, effects on health (particularly in children), and policy recommendations for mitigating these effects. It presents a factual, scientific viewpoint rather than aligning strictly with a liberal or conservative political stance.<br><br>The focus on environmental health, public policy for air quality improvement, and the detailed discussion of the health effects of air pollution, including on vulnerable populations like children, might be more commonly associated with liberal viewpoints, which typically emphasize environmental protection and public health measures. However, the text also delves into regulatory and economic aspects, topics that can be of interest across the political spectrum.<br><br>It's important to note that while environmental issues can sometimes be politicized, the fundamental concerns about air pollution and public health are universal and not inherently political. This text appears to prioritize scientific understanding and public health implications over any specific political ideology.	probably liberal
AAP	Recommended Essential Equipment for Basic Life Support and Advanced Life Support Ground Ambulances 2020: A Joint Position Statement	6/1/2021	The text you provided appears to be a detailed and comprehensive guideline focused on the standards, protocols, and equipment necessary for high-quality Emergency Medical Services (EMS). It discusses the importance of provider credentialing, evidence-supported clinical guidelines, and proper equipment for effective patient care in EMS settings. The text also addresses the evolution of EMS medicine, the need for periodic review and revision of equipment standards, and the importance of competency in using the equipment.<br><br>This text doesn't explicitly align with a liberal or conservative viewpoint. It is a professional, medically-oriented document that focuses on best practices in the field of emergency medical care. The emphasis is on evidence-based medicine, standardization of care, and ensuring high-quality outcomes for patients in emergency situations.<br><br>Political viewpoints, whether liberal or conservative, often focus on broader issues like healthcare policy, funding, and governance. This document, however, concentrates on operational and clinical guidelines rather than policy or ideological debates. Therefore, it's more appropriate to view this text as a professional and clinical guideline rather than a political statement.	neutral

AAP	Access to Optimal Emergency Care for Children	5/1/2021	The text provided presents a detailed, factual analysis of the challenges and barriers faced in accessing optimal pediatric emergency care in the United States. It does not align explicitly with either a liberal or conservative viewpoint, as it focuses more on outlining the systemic issues and possible improvements in the healthcare system rather than advocating for a specific political stance.<br><br>The text highlights various systemic issues, such as barriers to accessing emergency care, differences in readiness and quality of emergency services, financial concerns, and the need for better coordination and education among healthcare providers and the public. It also emphasizes the importance of addressing these challenges to ensure that all children, regardless of their background or circumstances, have access to high-quality emergency medical care.<br><br>This approach is more aligned with a public health perspective, emphasizing evidence-based policy and system improvements to enhance access and quality of care. It advocates for collaborative efforts among healthcare professionals, government agencies, and other stakeholders to address the outlined challenges. While there are elements that could be interpreted in line with liberal or conservative values, such as the emphasis on systemic change (often associated with liberal perspectives) or the focus on efficient and effective use of resources (which can align with conservative values), the text itself remains neutral and focused on the healthcare issue at hand.	neutral
AAP	Caring for American Indian and Alaska Native Children and Adolescents	4/1/2021	The text you provided does not align explicitly with either a liberal or conservative viewpoint. Instead, it focuses on specific health disparities and challenges faced by American Indian and Alaska Native (AI/AN) children and adolescents. The document is a policy statement that aims to raise awareness and provide recommendations for pediatric care providers, as well as suggestions for public policy advocacy.<br><br>Key points include:<br>- Acknowledging the substantial health inequities faced by AI/AN populations.<br>- The importance of pediatric care providers in addressing these inequities.<br>- The need for culturally sensitive family-centered care.<br>- Discussion of various health issues such as obesity, mental health, substance use disorder, and the impacts of Adverse Childhood Experiences (ACEs).<br>- Advocacy for improved health policies and research specific to AI/AN communities.<br><br>The text is primarily informative and focused on healthcare policy rather than political ideology. It aims to present facts and recommendations based on research and the specific health needs of AI/AN children and adolescents. The emphasis is on understanding and addressing the unique health challenges faced by these populations, promoting better health outcomes, and advocating for policy changes to support these goals.	neutral
AAP	Child Life Services	1/1/2021	The text you've provided doesn't align strictly with either a liberal or conservative viewpoint. It focuses on child life programs in pediatric hospital care, emphasizing the role of child life specialists in addressing the psychological needs of children during hospital stays and medical treatments. This approach is centered on pediatric psychology and child development, advocating for therapeutic play, family involvement, and tailored interventions to reduce anxiety and promote coping in children facing medical procedures.<br><br>Both liberal and conservative ideologies generally support the well-being and healthcare needs of children, although they might differ in their approaches to healthcare policy and funding. The text primarily describes clinical practices and research findings related to child life programs, which are a specialized area within pediatric care. This is a healthcare and child development issue, not inherently political or ideological in nature. It focuses on evidence-based practices to improve the hospital experience for children and their families, which is a universally acknowledged goal across various political spectrums.	neutral
AAP	Antibiotic Stewardship in Pediatrics	1/1/2021	The text you provided does not align specifically with either a liberal or conservative viewpoint. It is a scientific and medical statement focused on the issue of antibiotic overuse and the practice of antibiotic stewardship. This topic is generally considered a public health concern rather than a partisan issue. The text emphasizes the importance of using antibiotics responsibly to prevent antibiotic resistance, a problem that affects the health of the general population regardless of political affiliation. The approach is based on medical evidence and best practices in healthcare, aiming to optimize patient outcomes while minimizing the unintended consequences of antibiotic use, especially in pediatric populations.	neutral
AAP	Recommendations for Prevention and Control of Influenza in Children, 2020–2021	10/1/2020	The statement from the American Academy of Pediatrics (AAP) about the routine use of influenza vaccine and antiviral medications in children does not align with a specific political viewpoint, whether liberal or conservative. It is a medical recommendation based on public health and scientific research. The AAP's guidelines are aimed at protecting children's health by recommending vaccination and treatment strategies for influenza, particularly in the context of the COVID-19 pandemic. These recommendations are grounded in medical science and are intended to reduce the burden of respiratory illnesses and protect vulnerable populations. The focus of such statements is on public health and safety, rather than political ideology.	neutral

AAP	Participation of Children and Adolescents in Live Crisis Drills and Exercises	9/1/2020	The text you provided appears to balance concerns commonly associated with both liberal and conservative viewpoints. It doesn't neatly align with one specific political ideology, instead offering a nuanced approach to a complex issue. Here's a breakdown:<br><br>**Liberal Viewpoint Aspects:**<br>1. **Emphasis on Psychological Well-being:** The concern for the potential psychological impacts on children participating in high-intensity drills reflects a liberal tendency to prioritize emotional and mental health in policy considerations.<br>2. **Advocacy for Evidence-based Practices:** The call for research and evidence-based approaches to crisis preparedness aligns with a typically liberal emphasis on scientific evidence in policymaking.<br>3. **Focus on Accommodations for Vulnerable Groups:** The attention to the unique needs of children, especially those with disabilities or past traumas, aligns with liberal values of inclusivity and accommodation for vulnerable populations.<br><br>**Conservative Viewpoint Aspects:**<br>1. **Crisis Preparedness and Security Measures:** The discussion about active shooter drills in schools, even with the recommended limitations, can resonate with conservative priorities on security and preparedness.<br>2. **Involvement of Law Enforcement and First Responders:** The text acknowledges the role of law enforcement and other first responders in school safety, which aligns with a conservative emphasis on law and order and the value of these professions.<br><br>**Neutral or Overarching Themes:**<br>1. **Child Safety and Welfare:** The primary focus on the safety and well-being of children transcends political ideologies.<br>2. **Balanced Approach:** The text suggests a careful, balanced approach to crisis drills in schools, considering both the benefits of preparedness and the potential psychological impact on children.<br><br>In summary, the text reflects a multidimensional view that incorporates concerns and perspectives often found in both liberal and conservative ideologies, with a central focus on the safety and well-being of children in the context of crisis preparedness.	neutral
AAP	Truth, Reconciliation, and Transformation: Continuing on the Path to Equity	9/1/2020	The text provided does not neatly align with a singular political viewpoint, whether liberal or conservative. It primarily focuses on historical and current efforts by the American Academy of Pediatrics (AAP) to address and rectify past racial discriminations and biases within its organization and the broader medical community. The text highlights:<br><br>1. **Historical Context**: It acknowledges the challenges faced by Drs. Alonzo deGrate Smith and Roland Boyd Scott, two pioneering Black pediatricians, in gaining membership to the AAP due to systemic racism.<br><br>2. **Racial Discrimination in the Medical Field**: The text details the discriminatory practices of the early 20th century, such as the segregation policies of the American Medical Association and the hurdles faced by Black physicians in obtaining hospital privileges.<br><br>3. **AAP's Evolution**: It notes the AAP's efforts to evolve its equity agenda, including recognizing and addressing past injustices and implementing policies to ensure non-discrimination.<br><br>4. **Broader Societal Issues**: The text touches on the broader societal challenges of racism and the need for historical acknowledgement and reconciliation.<br><br>5. **AAP's Current Stance**: It emphasizes the AAP's current commitment to diversity, inclusion, and combating bias and discrimination, including its resolution to apologize for past racism and amend its bylaws to explicitly prohibit discrimination.<br><br>This content can be interpreted through various political lenses:<br><br>- **Liberal Viewpoint**: Might focus on the recognition of systemic racism, the importance of diversity and inclusion, and the active steps taken to address past injustices, aligning with liberal values of social justice and equality.<br><br>- **Conservative Viewpoint**: Some conservative perspectives might appreciate the focus on historical context and the evolution of institutions over time, though they might have differing views on the extent and nature of systemic racism and the methods of addressing it.<br><br>In summary, the text reflects a complex interplay of historical acknowledgment, institutional evolution, and contemporary efforts to	neutral

			address racial discrimination, themes that can resonate across different political spectrums depending on the focus and interpretation.	
AAP	Barrier Protection Use by Adolescents During Sexual Activity	8/1/2020	The text you've provided is a factual and informative statement regarding sexual health, particularly focusing on adolescents. It appears to be a balanced and evidence-based approach to sexual education and health, emphasizing the importance of barrier methods in preventing sexually transmitted infections (STIs) and unintended pregnancies.<br><br>This text does not explicitly align with either a liberal or conservative viewpoint. Instead, it focuses on providing medically accurate information and emphasizes the role of pediatricians in educating and supporting adolescents in making informed decisions about their sexual health. The acknowledgment of various sexual orientations, behaviors, and the inclusion of all adolescents, including those with intellectual and physical differences, suggests an inclusive and comprehensive approach to sexual education, which is generally more aligned with liberal viewpoints.<br><br>However, the emphasis on evidence-based practices, the importance of parental communication, and the promotion of sexual abstinence as the most effective way to prevent STIs and unintended pregnancies could be interpreted as elements that align with more conservative perspectives on sexual education.<br><br>Overall, the text seems to aim for a balanced approach, integrating aspects that could be appreciated by both liberal and conservative viewpoints, emphasizing health, education, and informed choices.	neutral
AAP	Electronic Documentation in Pediatrics: The Rationale and Functionality Requirements	7/1/2020	The text provided does not align distinctly with either a liberal or conservative viewpoint. It is a neutral, technical discussion focused on clinical documentation, specifically in the context of electronic health records (EHRs) and health information technology. The document addresses the need for specialized documentation approaches in pediatric care, challenges associated with electronic documentation, and suggestions for improvement in these systems. It emphasizes the need for research, development of best practices, and stakeholder involvement in the evolution of electronic documentation, particularly in pediatric healthcare. The text is grounded in professional and technical concerns rather than political ideology.	neutral

AAP	Digital Advertising to Children	7/1/2020	The text provided does not strictly align with either a liberal or conservative viewpoint. Instead, it focuses on the multifaceted issue of advertising to children and teenagers, presenting concerns and recommendations from a public health and developmental perspective. The text reviews various forms of advertising, including digital marketing strategies like sponsored content, influencer marketing, and behavioral targeting through machine learning. It emphasizes the vulnerability of children and teenagers to these marketing tactics due to their developing critical thinking and impulse control.<br><br>The statement advocates for awareness among parents and pediatric health care providers about the influence of marketing on children. It highlights the negative impacts of advertising on children's health behaviors, including unhealthy food choices, tobacco and e-cigarette use, alcohol and marijuana consumption, and indoor tanning.<br><br>The text also discusses the challenges in identifying modern, integrated forms of advertising and the inadequacy of current regulations to protect children in the digital age. It calls for increased digital literacy and responsibility from parents, health care providers, industry, and policymakers to protect children from potentially harmful marketing practices.<br><br>Overall, the text presents a balanced, research-based view concerned with child welfare and public health rather than aligning with a specific political ideology.	neutral
AAP	Advocacy and Collaborative Health Care for Justice-Involved Youth	7/1/2020	The text you provided does not clearly align with a specific political viewpoint, such as liberal or conservative, as it focuses on a factual and comprehensive overview of issues related to children and adolescents involved in the justice system. It discusses the complexities of their needs, including medical, mental health, developmental, social, and legal aspects, and the impact of childhood trauma and adversity on their involvement in the justice system and overall well-being.<br><br>The text advocates for a better understanding and response to these needs by pediatricians, emphasizing their role in promoting health and advocating for juvenile justice reform. It acknowledges racial biases and the overrepresentation of youth of color in the justice system and highlights various aspects of the system, including health and mental health status of justice-involved youth and opportunities for advocacy.<br><br>Overall, the content is more focused on providing a detailed, evidence-based analysis of the juvenile justice system, the challenges faced by justice-involved youth, and the potential roles of pediatricians in addressing these challenges. It does not explicitly promote a particular political ideology but rather underscores the importance of a multidisciplinary and informed approach to juvenile justice.	neutral
AAP	Children With Intellectual and Developmental Disabilities as Organ Transplantation Recipients	5/1/2020	The text you've provided doesn't neatly align with a traditional liberal or conservative viewpoint. It primarily discusses ethical and medical considerations regarding organ transplant eligibility, focusing on individuals with intellectual and developmental disabilities (IDDs). The main themes are:<br><br>1. **Organ Transplant Demand and Allocation**: It acknowledges the scarcity of transplantable solid organs and the need for careful selection criteria, which includes both medical and psychosocial factors.<br><br>2. **Historical Exclusion of IDD Individuals**: It highlights that historically, individuals with IDDs have often been excluded from being potential organ transplant recipients.<br><br>3. **Legal and Ethical Considerations**: The text argues that excluding IDD individuals might constitute discrimination under laws like the Americans with Disabilities Act. It emphasizes the need for fair and unbiased evaluation processes.<br><br>4. **Transplant Success and IDD**: It discusses studies and examples where IDD individuals have successfully undergone transplants, challenging assumptions about their post-transplant compliance and quality of life.<br><br>5. **Recommendations for Policy and Practice**: The text calls for standardized, equitable, and transparent criteria in transplant evaluations, stressing the importance of considering both cognitive and adaptive skills of IDD individuals.<br><br>While these points don't fit squarely into a political ideology, they touch upon principles like equality, fairness, and non-discrimination, which are generally emphasized in liberal thought. However, the focus on practicality, effective resource allocation, and individualized assessment can also resonate with conservative values. In essence, the text blends ethical, legal, and medical perspectives rather than being overtly political.	neutral

AAP	Providing Care for Infants Born at Home	5/1/2020	The text from the American Academy of Pediatrics (AAP) regarding home birth primarily aligns with a scientific and medical viewpoint rather than a distinctly liberal or conservative political stance. It focuses on presenting evidence-based recommendations and guidelines for safe childbirth practices, emphasizing the importance of hospital or accredited birth center settings for reducing risks associated with childbirth. The text acknowledges the increasing trend of home births and the variety of reasons why some women may choose this option. It also recognizes the right of women to make informed decisions about their childbirth plans.<br><br>This perspective is primarily rooted in medical and scientific evidence about the safety and risks associated with different birthing options. It does not explicitly endorse or oppose any political ideology but rather aims to inform healthcare providers and expectant mothers about the safest practices for childbirth based on current medical research and standards.<br><br>The discussion of home birth can intersect with political views regarding healthcare access, women's rights, and individual choice. However, the AAP's statement itself is more focused on medical safety and best practices rather than promoting a specific political ideology.	neutral
AAP	Nickel Allergic Contact Dermatitis: Identification, Treatment, and Prevention	5/1/2020	The text provided does not align strictly with either a liberal or conservative viewpoint. It is a scientific and medical discussion about nickel allergic contact dermatitis (Ni-ACD), its epidemiology, symptoms, and the impact of legislation on its prevalence. The focus is on the medical and public health aspects of Ni-ACD, particularly in children, and the effectiveness of regulatory measures in reducing its incidence.<br><br>The statement discusses the history of nickel use, its prevalence as an allergen, the symptoms and diagnosis of Ni-ACD, and the role of legislation in reducing exposure and incidence. It cites the European Union's directive to limit nickel release from consumer items as an effective measure in reducing Ni-ACD cases. The advocacy for similar legislation in the United States by the American Academy of Pediatrics is presented as a public health measure, not from a partisan political perspective.<br><br>In essence, the text is informational and aimed at raising awareness about Ni-ACD, its impacts, and potential preventive measures. It approaches the topic from a scientific and public health standpoint rather than a political one.	neutral
AAP	Abusive Head Trauma in Infants and Children	4/1/2020	The text you provided doesn't align strictly with a liberal or conservative viewpoint. It is a medical and scientific statement focusing on Abusive Head Trauma (AHT) in children. This statement, from a medical perspective, addresses the diagnosis, evaluation, and management of AHT, along with legal and preventive aspects. It is primarily concerned with improving medical practice and child welfare, rather than promoting a particular political ideology.<br><br>Medical statements like these are typically guided by evidence-based research and clinical best practices, aiming to provide healthcare professionals with information to enhance patient care and safety. The discussion about AHT spans various aspects, including its diagnosis, clinical presentation, evaluation procedures, implications for legal proceedings, and prevention strategies, which are universal concerns transcending political affiliations.<br><br>In the broader context, child welfare and protection can be of interest to both liberal and conservative platforms, but the approach and emphasis might differ. Liberals might focus more on systemic and social support measures, while conservatives might emphasize family responsibility and legal repercussions. However, this text doesn't advocate for a specific policy or approach from a political standpoint; rather, it focuses on the medical and factual aspects of AHT.	neutral
AAP	Chemical-Biological Terrorism and Its Impact on Children	2/1/2020	The text provided does not explicitly align with a traditional liberal or conservative viewpoint. It focuses on public health preparedness, particularly in relation to chemical and biological events, and their impact on children. The text emphasizes the need for preparedness at the pediatrician level, the role of government agencies, and the importance of funding for state and local initiatives. It is a factual and informative piece centered on healthcare and emergency preparedness, rather than a political argument or opinion piece. Therefore, it doesn't strongly align with the typical ideological stances of either liberalism or conservatism but rather addresses a public health concern that can be of interest to people across the political spectrum.	neutral

AAP	Pediatric Readiness in Emergency Medical Services Systems	1/1/2020	The text you've provided is primarily a factual and policy-oriented statement regarding prehospital emergency care, particularly focused on the needs of pediatric patients in emergency medical services (EMS). It discusses the importance of specific training, equipment, protocols, and oversight in ensuring effective EMS care for children.<br><br>This text does not align distinctly with either a liberal or conservative viewpoint in the typical political sense. It is more about best practices in healthcare, particularly in emergency medical services for children, than about political ideology. The recommendations and conclusions focus on improving healthcare outcomes, a goal generally shared across the political spectrum.<br><br>However, the emphasis on systematic planning, collaboration among various stakeholders (including public health experts and family advocates), and adherence to national guidelines could be interpreted as aligning with a more progressive or liberal approach to healthcare, which often emphasizes comprehensive, evidence-based, and collaborative strategies. On the other hand, there isn't a clear indication of conservative principles such as reduced government intervention or market-based solutions in healthcare.<br><br>In summary, while the text does lean towards a systematic and collaborative approach to healthcare, typical of more progressive views, it is fundamentally centered on healthcare policy and best practices rather than political ideology.	probably liberal
AAP	Principles of Financing the Medical Home for Children	1/1/2020	The text you provided seems to align more with a liberal viewpoint, particularly in its emphasis on comprehensive healthcare coverage, the absence of cost-sharing for preventive services, and the focus on a uniform definition of medical necessity that includes a wide range of services. These concepts are generally more aligned with liberal ideologies, which often advocate for broader healthcare coverage and accessibility. Additionally, the text supports the idea of payment models that discourage inappropriate use of medical services while promoting necessary care, a principle that is in line with liberal views on healthcare efficiency and effectiveness.<br><br>The emphasis on population health and the specific focus on pediatric care also align with liberal priorities, which often include a strong emphasis on public health and preventive care. The liberal viewpoint typically stresses the importance of healthcare access for all segments of the population, including children, and the prevention of health issues through comprehensive care.<br><br>Overall, the approach described in the text advocates for an inclusive, comprehensive healthcare system that minimizes out-of-pocket costs for patients, aligns closely with liberal healthcare policies, and contrasts with conservative approaches that often emphasize market-based solutions and personal responsibility in healthcare.	liberal
AAP	Pediatric Metabolic and Bariatric Surgery: Evidence, Barriers, and Best Practices	12/1/2019	The text provided is primarily factual and informative in nature, detailing the prevalence, implications, and treatment options for severe obesity in youth. It does not explicitly align with a liberal or conservative viewpoint. Instead, it focuses on medical and scientific information about severe obesity among young people, including statistical data, treatment methods like bariatric surgery, and considerations for practitioners and policy makers.<br><br>This text appears to be from a medical or health policy report. It discusses the health risks associated with severe obesity in youth, various surgical treatments, and the importance of accessibility and best practices in treatment. The language is neutral and objective, aimed at informing readers about the current state of knowledge and practice in this area of healthcare. The emphasis is on evidence-based recommendations and a thorough understanding of the benefits and risks of different treatments, rather than on promoting any particular ideological stance.	neutral
AAP	Unique Needs of the Adolescent	12/1/2019	The text provided does not align strictly with either a liberal or conservative viewpoint. It is a factual, evidence-based statement focusing on adolescent health and development, addressing the unique challenges faced by adolescents and the need for comprehensive health services. The statement emphasizes the importance of recognizing and addressing the specific health needs of adolescents to ensure healthy development into adulthood. It discusses various aspects of adolescent health, including physical, mental, and sexual health, as well as the impact of social and environmental factors. The approach is scientific and medically oriented, advocating for policies and practices that are informed by research and focused on the well-being of adolescents. This type of content is typically found in medical and health policy literature, aiming to inform healthcare providers, policymakers, and the public about the specific needs and challenges of adolescent health.	neutral

AAP	Emergency Contraception	12/1/2019	The text provided appears to be an objective, fact-based presentation of information regarding emergency contraception (EC) and its use among teenagers. It does not explicitly align with a specific political viewpoint, whether liberal or conservative. Instead, it focuses on the medical aspects of EC, including its types, efficacy, safety, and indications for use. The text aims to educate healthcare providers about EC methods and encourage routine counseling and advance prescription as a public health strategy to reduce teenage pregnancy.<br><br>This approach is consistent with a public health perspective, emphasizing evidence-based medical practices and the importance of access to reproductive health services. It addresses the high rates of teen pregnancy in the United States compared to other high-income nations and discusses various EC methods approved by the U.S. Food and Drug Administration, as well as their appropriate uses and considerations.<br><br>While the topic of contraception, including emergency contraception, can be politically charged and subject to differing opinions based on political or personal beliefs, the text itself presents information in a neutral, informative manner without advocating for a specific political stance.	neutral
AAP	The Use of Nonnutritive Sweeteners in Children	11/1/2019	The text provided does not align with a specific political viewpoint, whether liberal or conservative. Instead, it is a detailed scientific and medical examination of nonnutritive sweeteners (NNSs), their history, usage, potential health impacts, and the regulatory status. It discusses various aspects of NNSs, including their introduction into the food supply, safety evaluations by the FDA, potential health concerns (such as cancer risks and impact on metabolic syndrome), and their use in different populations (like children, those with obesity, or diabetes).<br><br>This kind of scientific literature aims to inform and guide healthcare professionals, particularly pediatricians, about NNSs. It is based on research findings and health policy rather than political ideology. The document's focus is on public health, safety, and nutrition, areas that typically transcend political boundaries and are of interest to a wide audience regardless of political affiliation.	neutral
AAP	Mental Health Competencies for Pediatric Practice	11/1/2019	The text you provided does not neatly align with a traditional liberal or conservative viewpoint. Instead, it primarily focuses on pediatric healthcare, particularly mental health care for children and adolescents. The themes discussed, such as the importance of addressing mental health in pediatric care, the integration of mental health services in primary care settings, and the acknowledgment of social determinants on mental health, are generally non-partisan and concern public health and medical practice.<br><br>The emphasis on evidence-based practices, awareness of the impact of social and environmental factors on mental health, and the need for systemic changes in healthcare delivery are common in modern medical discourse and do not inherently signal a political ideology. These aspects reflect a focus on comprehensive healthcare, acknowledging the multifaceted nature of health issues, which is a perspective shared across various political and professional spectrums.<br><br>However, the approach to healthcare policy, funding, and the role of government in providing or regulating healthcare can be influenced by political views. For example, a more liberal viewpoint might advocate for greater government involvement in ensuring access to mental health services, while a conservative viewpoint might emphasize private sector solutions and individual responsibility. But the text itself, centered on pediatric mental health care practices and competencies, remains neutral and focused on medical and healthcare professional standards.	neutral
AAP	Executive Summary: Criteria for Critical Care of Infants and Children: PICU Admission, Discharge, and Triage Practice Statement and Levels of Care Guidance	10/1/2019	The text you provided is an executive summary of the 2019 update to the 2004 guidelines for levels of care in Pediatric Intensive Care Units (PICU). It outlines advancements in pediatric critical care medicine and the evolution of resources and training in PICUs. The summary discusses the revised guidelines designed to aid hospitals, institutions, and individuals in developing appropriate PICU structures for their community needs. It also details the methodology used for the guideline update, including a comprehensive literature review and a modified Delphi process for developing consensus-based recommendations.<br><br>This text is primarily technical and medical in nature, focusing on improving pediatric critical care. It does not align distinctly with either a liberal or conservative viewpoint as it is a scientific and medical document. Its aim is to provide updated guidelines for the care of critically ill children, based on the latest research and expert consensus, rather than to promote a political agenda. The content is specialized and directed towards professionals in pediatric critical care, emphasizing the importance of evidence-based practice and multidisciplinary teamwork in the PICU.	neutral

AAP	Pediatric Application of Coding and Valuation Systems	10/1/2019	The text provided primarily focuses on technical and policy aspects related to healthcare payment systems, particularly in the context of pediatric care. It discusses the Resource-Based Relative Value Scale (RBRVS), Current Procedural Terminology (CPT), and International Classification of Diseases, 10th Revision, Clinical Modification (ICD-10-CM) codes, as well as recommendations for coding, valuation, and quality measures in pediatric healthcare.<br><br>This text does not align distinctly with either a liberal or conservative political viewpoint. Instead, it is a detailed, professional document likely intended for healthcare providers, policy makers, and administrators. It focuses on the mechanics of healthcare payment systems, advocating for fair and transparent payment processes, and ensuring adequate coverage and valuation of pediatric services.<br><br>The content is more technical and administrative rather than ideological. While different political viewpoints may have varying stances on healthcare policy, this document does not explicitly advocate for or against any particular political ideology. Instead, it appears to aim for improving and refining existing healthcare payment systems to better serve pediatric healthcare needs.	neutral
AAP	Recommendations for Prevention and Control of Influenza in Children, 2019–2020	10/1/2019	The statement you provided is a comprehensive update from the American Academy of Pediatrics on the use of influenza vaccines and antiviral medications for the prevention and treatment of influenza in children during the 2019–2020 season. This statement is primarily medical and scientific in nature, focusing on public health recommendations and does not inherently align with a specific political ideology, whether liberal or conservative.<br><br>It is important to note that public health guidelines and recommendations, such as those related to vaccines and medications, are typically based on scientific evidence and research. They aim to promote the health and well-being of the population and are generally not designed to reflect or align with political viewpoints. However, public perception and acceptance of such guidelines can vary and might be influenced by political, cultural, or personal beliefs.	neutral
AAP	Providing Care for Children in Immigrant Families	9/1/2019	The text you provided does not align strictly with either a liberal or conservative viewpoint in a political sense. Instead, it presents a detailed, factual, and compassionate overview of the health care challenges and considerations for children in immigrant families (CIF) in the United States. The focus is on understanding the unique needs of this demographic, enhancing care through cultural humility and safety, and advocating for policies that promote health equity for CIF.<br><br>Key aspects of the text include:<br><br>1. **Demographic Information**: It provides statistical data on the population of immigrant children and CIF in the U.S., highlighting their growing presence.<br><br>2. **Diverse Reasons for Immigration**: The text acknowledges various reasons families immigrate, including economic needs, educational pursuits, and fleeing persecution.<br><br>3. **Health Challenges and Inequities**: It discusses the unique health challenges faced by CIF, such as access to care, social determinants of health, and potential inequities that threaten their well-being.<br><br>4. **Cultural Considerations**: The importance of cultural humility and safety in providing care to CIF is emphasized, recognizing the dynamic nature of culture and its impact on health.<br><br>5. **Recommendations for Care**: The text outlines practice-level recommendations for pediatricians to promote health equity for CIF, including attention to core competencies in clinical care, community engagement, and system-level support.<br><br>6. **Advocacy and Policy**: It suggests advocacy and policy changes to improve the health and well-being of CIF, such as providing legal representation in immigration proceedings and ensuring access to health care irrespective of immigration status.<br><br>The approach is comprehensive and holistic, focusing on the health and well-being of CIF as a vulnerable population rather than taking a partisan stance. It advocates for understanding, compassion, and equitable treatment in the healthcare system, which are principles that can resonate across the political spectrum.	neutral

AAP	The Impact of Racism on Child and Adolescent Health	8/1/2019	The text you've provided appears to align more closely with a liberal viewpoint. It focuses on addressing racism as a social determinant of health, emphasizing the need for systemic change and acknowledging the historical and ongoing impacts of racism on health outcomes, particularly for children and adolescents. This perspective is often associated with liberal ideologies, which tend to advocate for social justice, equity, and systemic reforms to address social inequalities.<br><br>Conservative viewpoints, on the other hand, might focus more on individual responsibility, limited government intervention in social issues, and may be less inclined to view systemic racism as a primary factor in health disparities.<br><br>It's important to note that these are generalizations and that viewpoints can vary widely within both liberal and conservative ideologies. However, the emphasis on systemic change and equity in the text aligns more closely with traditional liberal perspectives.	liberal
AAP	Electronic Communication of the Health Record and Information With Pediatric Patients and Their Guardians	7/1/2019	The text you've provided is a detailed and comprehensive examination of the challenges and evolving landscape of electronic health records (EHRs), especially in the context of pediatric and adolescent patients. It addresses the complexities of privacy, confidentiality, legal guardianship, and the technological capabilities and limitations of EHRs. The text also delves into the nuances of communication between healthcare teams, patients, and guardians, and the legal and regulatory environment surrounding electronic health data.<br><br>This text does not align explicitly with either a liberal or conservative viewpoint. Instead, it takes a neutral, informative stance, focusing on the technical, ethical, and practical aspects of EHRs and health information technology. It discusses the need for collaboration among healthcare providers, patients, policymakers, and technology vendors to optimize the use of EHRs for effective and safe communication of health data.<br><br>The content is more aligned with healthcare policy and management, technology in healthcare, and patient privacy rights. It seeks to address the challenges in the field without advocating for a specific political ideology or viewpoint. The primary concern here is improving healthcare delivery and patient outcomes through better use of technology, while ensuring privacy and legal compliance.	neutral
AAP	Alcohol Use by Youth	7/1/2019	The text provided does not align explicitly with either a liberal or conservative viewpoint. It presents a factual and research-based approach to the topic of alcohol use among youth and young adults, highlighting the potential adverse impacts on the developing brain, the prevalence of underage drinking, and the associated risks.<br><br>The statement and accompanying data emphasize the importance of understanding the neurobiology of adolescent development and the hazards of alcohol use. It discusses various aspects such as binge drinking, the Diagnostic and Statistical Manual of Mental Disorders' criteria for alcohol use disorders, the impact of alcohol on the brain and behavior, and factors contributing to hazardous alcohol use.<br><br>Additionally, the text outlines the role of pediatricians in addressing this issue and provides recommendations from the American Academy of Pediatrics, advocating for policies and practices aimed at reducing alcohol use among adolescents and young adults.<br><br>This approach is more scientific and medically oriented rather than being politically charged. It focuses on health and safety considerations, which are typically a concern across the political spectrum, though the emphasis on policy recommendations and public health interventions might resonate more with those who advocate for proactive government involvement in health issues.	neutral
AAP	Improving Health and Safety at Camp	7/1/2019	The text provided doesn't align specifically with a liberal or conservative viewpoint. It is a set of recommendations and guidelines from the American Academy of Pediatrics (AAP) concerning health and safety practices at camps for children. These recommendations cover a wide range of topics, including the roles of parents and guardians, primary care providers, and camp staff, as well as specific health considerations like immunizations, mental health, nutrition, and management of chronic conditions.<br><br>The focus of this text is on ensuring a safe and healthy environment for children at camp, emphasizing preparedness, prevention, and collaboration among parents, health care providers, and camp staff. It's more centered around pediatric health care and best practices in a camp setting rather than any political ideology.	neutral
AAP	Transporting Children With Special Health Care Needs	5/1/2019	The text you've provided is a comprehensive policy statement on the safe transportation of children with special health care needs. It does not align specifically with either a liberal or conservative viewpoint; rather, it focuses on practical, safety-oriented recommendations and guidelines for the proper transportation of children with specific medical conditions. The statement emphasizes the importance of accessibility to appropriate transportation resources and safety devices for all children, including those with special health care needs.	neutral

			This emphasis on equal access and safety for children with special needs is a universal concern that transcends traditional political divides. The detailed guidelines and recommendations are grounded in research and expert opinion, highlighting a commitment to child safety and well-being that is generally supported across the political spectrum.	
AAP	Institutional Ethics Committees	5/1/2019	The text provided appears to be a detailed and comprehensive overview of the roles, functions, and ethical considerations of Institutional Ethics Committees (IECs) in healthcare settings, particularly in the United States. This text does not explicitly align with a specific political viewpoint, whether liberal or conservative. Instead, it focuses on the practical and ethical aspects of IECs in the context of healthcare, discussing their evolution, roles in clinical ethics consultation, education in ethics, policy review and development, and their role in organizational ethics.<br><br>The content is largely factual and technical, outlining standards, procedures, and recommendations for the effective functioning of IECs. It addresses the need for ethical oversight and consultation in healthcare settings, the importance of interdisciplinary representation in these committees, and the various tasks they undertake, such as case consultation, policy development, and education on ethical issues in patient care.<br><br>Such a focus on ethics, best practices, and procedural guidelines in healthcare transcends typical political categorizations and is more aligned with professional standards and ethical considerations in medical practice. The text aims to provide guidance and information relevant to healthcare professionals and administrators rather than advocating for a specific political ideology.	neutral
AAP	Prevention of Drowning	5/1/2019	The text you provided doesn't inherently align with either a liberal or conservative viewpoint. It is a factual and informative piece, focused on the issue of drowning as a cause of injury-related death in children, and discusses various strategies for prevention. The emphasis is on public health and safety, particularly for children, and it includes statistical data, recommendations for parents, caregivers, and pediatricians, as well as policy implications.<br><br>Political viewpoints often come into play when discussing how to implement safety measures, fund public health initiatives, or regulate community and private facilities. For instance, conservatives might emphasize personal responsibility and limited government intervention, while liberals might advocate for more government-led initiatives and regulations for public safety. However, the text itself is neutral and focused on presenting information and recommendations based on research and statistics, without advocating for a specific political approach.	neutral
AAP	Public Policies to Reduce Sugary Drink Consumption in Children and Adolescents	4/1/2019	The text provided doesn't align strictly with either a liberal or conservative viewpoint, as it focuses primarily on public health concerns related to the consumption of added sugars, particularly in sugary drinks, and its impact on children and adolescents. However, the emphasis on public policy interventions, such as excise taxes on sugary drinks and regulations to reduce their consumption, is more characteristic of liberal viewpoints, which generally support a more active role for government in addressing public health and social issues.<br><br>Liberal perspectives often advocate for government intervention to address public health concerns, especially when these issues disproportionately affect vulnerable populations, such as children in minority and low-income communities. The text's focus on using public policy to mitigate health risks associated with sugary drinks aligns with this approach.<br><br>Conservative viewpoints, on the other hand, tend to emphasize personal responsibility and limited government intervention in individual choices, including dietary habits. From this perspective, the emphasis might be more on individual education and choice rather than on regulatory or tax-based approaches.<br><br>It's important to note that public health issues like the ones discussed in the text can transcend traditional political boundaries, and there can be a range of opinions within both liberal and conservative circles on the best approaches to addressing such issues.	probably liberal

AAP	Understanding Liability Risks and Protections for Pediatric Providers During Disasters	3/1/2019	The text provided does not align distinctly with either a liberal or conservative viewpoint in a traditional political sense. It is primarily focused on healthcare policy, specifically regarding disaster preparedness and the management of legal liabilities for pediatric healthcare providers during disasters. The recommendations and policy suggestions are centered around ensuring that pediatric health care providers are educated, prepared, and legally protected when responding to disasters. This focus on preparedness, education, and legal protection is not inherently political or indicative of a specific political ideology.<br><br>The policy statement emphasizes the importance of:<br>1. Educating and preparing pediatric healthcare providers for disaster response.<br>2. Strengthening legal protections to ensure providers can offer care during disasters without fear of legal repercussions.<br>3. Advocating for better laws and policies at both state and federal levels to address these issues.<br><br>These points are generally aimed at improving healthcare response in emergencies and do not directly address broader political or ideological debates typically associated with liberal or conservative viewpoints. The focus is on practical, logistical, and legal aspects of healthcare delivery in crisis situations, which can be of concern to people across the political spectrum.	neutral
AAP	E-Cigarettes and Similar Devices	2/1/2019	The text you provided presents a viewpoint that is primarily concerned with public health, specifically the health risks associated with e-cigarette use among youth and the need for regulation to address these risks. This perspective is focused on the evidence of health harms related to e-cigarettes and the need for public policy interventions.<br><br>In terms of political alignment, it doesn't strictly adhere to traditional liberal or conservative viewpoints as understood in the United States. However, the emphasis on regulation and public health intervention could be more aligned with liberal viewpoints, which often support the role of government in regulating industries to protect public health and safety. Liberals typically advocate for stronger regulatory measures in matters related to public health.<br><br>Conversely, conservative viewpoints often emphasize less government intervention in businesses and personal choices, advocating for individual responsibility and market-driven solutions. However, it is important to note that public health issues, especially those affecting children and adolescents, can sometimes transcend typical liberal-conservative divides, with individuals from both sides showing concern and advocating for measures to protect vulnerable populations.<br><br>It's key to understand that such issues are complex and may not neatly fit into a single political ideology. Different aspects of the issue might be emphasized differently by liberals and conservatives.	probably liberal
AAP	Principles of Pediatric Patient Safety: Reducing Harm Due to Medical Care	2/1/2019	The text you've provided is centered on the topic of pediatric patient safety, emphasizing the importance of reducing harm due to medical care, improving the quality of care, and advocating for best practices and policies unique to pediatric healthcare. This focus aligns with general healthcare improvement principles, which are typically not bound by political ideology.<br><br>However, if we were to analyze it through the lens of traditional liberal and conservative viewpoints in the context of American politics, some aspects might align more closely with one viewpoint or the other:<br><br>1. **Liberal Viewpoint Alignment**:<br>- Emphasis on Systemic Changes and Regulations: The text advocates for systemic changes in healthcare practices and policies to improve patient safety. Liberals generally support the idea of systemic reforms and the implementation of regulations to ensure safety and quality in healthcare.<br>- Focus on Collaborative Efforts and Education: The text highlights the importance of education, training, and collaborative efforts to improve pediatric patient safety. Liberals often favor approaches that involve community and collective actions for societal benefits.<br><br>2. **Conservative Viewpoint Alignment**:<br>- Emphasis on Individual Responsibility and Accountability: The text also discusses the importance of individual healthcare providers being responsible for patient safety and knowledgeable about best practices. Conservatives generally value individual responsibility and accountability in various sectors, including healthcare.<br>- Potential Concerns about Overregulation: While not explicitly stated in the text, the implementation of extensive safety protocols and systems might be viewed by some conservatives as overregulation, potentially leading to increased bureaucracy in healthcare.<br><br>It's important to note that patient safety and healthcare quality are widely considered non-partisan issues, with a shared goal across	neutral

			the political spectrum of ensuring effective and safe medical care for all patients. The text primarily focuses on these universally acknowledged healthcare goals rather than on partisan politics.	
AAP	The Link Between School Attendance and Good Health	2/1/2019	The text provided is a comprehensive and informative exploration of the issue of chronic absenteeism in schools, particularly in the United States. It delves into various aspects of the problem, including its definition, underlying causes, associated risks, and potential interventions. The text does not align explicitly with either a liberal or conservative viewpoint. Instead, it takes an educational and health-centered approach, focusing on the impacts of absenteeism on children's health, educational outcomes, and long-term well-being.<br><br>The discussion encompasses factors such as poverty, unstable housing, racial and ethnic disparities, mental health, and the role of pediatricians and medical professionals in addressing these issues. It emphasizes the importance of understanding absenteeism in a broader social and health context, suggesting that solutions should be multidisciplinary and community-focused.<br><br>In terms of political alignment, the text could resonate with viewpoints across the spectrum. For instance, those with liberal perspectives might appreciate the focus on social determinants of health, racial and income disparities, and the call for comprehensive community and health-based interventions. On the other hand, those with conservative views might value the emphasis on personal responsibility, family involvement, and local solutions over federal interventions.<br><br>Overall, the text's primary alignment is with educational, medical, and public health perspectives, rather than being distinctly political. It advocates for a holistic understanding of chronic absenteeism, recognizing the complex interplay of health, social, economic, and educational factors.	neutral
AAP	Incorporating Recognition and Management of Perinatal Depression Into Pediatric Practice	1/1/2019	The text you've provided seems to be a comprehensive and clinical overview of perinatal depression (PND), including its impact, prevalence, screening, treatment, and policy recommendations, particularly from the perspective of pediatric primary care. It does not align explicitly with either a liberal or conservative political viewpoint. Instead, it focuses on medical and public health aspects of PND, emphasizing the importance of screening, early intervention, and coordinated care.<br><br>The text approaches PND from a medical and public health perspective, highlighting the need for systemic changes in healthcare practices to better address this issue. It advocates for integrated care approaches, training for healthcare providers, and public policy support for PND screening and treatment.<br><br>While different political ideologies might vary in their approach to healthcare policy and funding, the information in this text is presented in a way that is focused on health outcomes and best practices rather than political ideology. It could be supported by those on both ends of the political spectrum who recognize the importance of mental health care, particularly as it relates to mothers and infants.	neutral

AAP	Effective Discipline to Raise Healthy Children	12/1/2018	The text you provided seems to align more with a liberal viewpoint, primarily due to its emphasis on non-physical and non-verbal forms of discipline in child-rearing, its reliance on recent scientific research to guide parenting practices, and its advocacy for the abandonment of corporal punishment. This viewpoint aligns with more progressive stances that prioritize psychological and emotional well-being, non-violent forms of communication, and evidence-based practices in child development and parenting.<br><br>Liberal perspectives often emphasize the importance of understanding and addressing the underlying causes of behavioral issues in children rather than using punitive measures. The text's focus on educating parents about positive and effective parenting strategies, and its reference to evidence linking corporal punishment to negative outcomes, are consistent with this approach.<br><br>Conservative viewpoints, on the other hand, might place a higher emphasis on traditional discipline methods, including the use of corporal punishment as a tool for teaching respect and obedience. They might argue for parental autonomy in deciding how to discipline their children, emphasizing values like family privacy and the preservation of traditional parenting practices.<br><br>However, it's important to note that these categorizations can be oversimplified and that viewpoints on child discipline can vary widely within both liberal and conservative circles. There are conservatives who oppose corporal punishment, just as there are liberals who might support certain traditional aspects of child-rearing. The distinction here is based on general tendencies and predominant views within these political ideologies.	liberal
AAP	The Effects of Armed Conflict on Children	12/1/2018	The text provided seems to address the issue of armed conflict and its impact on children from a humanitarian and child welfare perspective, rather than aligning explicitly with either a liberal or conservative viewpoint. The focus is on the rights of children, their health and well-being, and the need for a collaborative, rights-based approach to address the effects of armed conflict on children.<br><br>This approach is more centered on humanitarian principles and the protection of children's rights, which can transcend traditional political divisions. It advocates for the involvement of child health professionals and organizations like the American Academy of Pediatrics in addressing these issues globally, emphasizing the importance of a multi-faceted response encompassing clinical care, systems development, and policy formulation.<br><br>Overall, the text aligns more with universal principles of child welfare and human rights rather than with a specific political ideology.	neutral
AAP	Pediatric Considerations Before, During, and After Radiological or Nuclear Emergencies	12/1/2018	The text you provided appears to be a comprehensive and factual report or policy statement concerning radiation emergencies, specifically focusing on the impact on children. It doesn't inherently align with a liberal or conservative viewpoint. Instead, it is centered on health and safety recommendations, and the information is presented in a scientific and objective manner.<br><br>The text discusses various aspects of radiation exposure risks for children, citing examples from historical nuclear incidents and providing guidelines for health professionals and government entities. It emphasizes the need for preparedness and education in the face of potential radiological emergencies, without advocating for a specific political stance or policy beyond those related to public health and safety.<br><br>Such a focus on health and safety, independent of political views, is crucial in ensuring that the information remains accessible and useful to a broad audience, regardless of their political leanings.	neutral
AAP	Targeted Reforms in Health Care Financing to Improve the Care of Adolescents and Young Adults	12/1/2018	The text you provided is a detailed and comprehensive policy statement discussing various aspects of health care financing for adolescents and young adults (AYAs), particularly in the context of the Patient Protection and Affordable Care Act (ACA) of 2010 and the Paul Wellstone and Pete Domenici Mental Health Parity and Addiction Equity Act of 2008. It addresses issues such as insurance coverage, barriers to health care access, cost sharing, and the need for confidentiality in health care services for AYAs.<br><br>This text does not explicitly align with a traditional liberal or conservative viewpoint. Instead, it presents a factual analysis of the health care situation for AYAs in the United States, focusing on policy implications, challenges, and potential solutions within the framework of existing legislation. The text emphasizes the importance of health care access and equity, discusses the limitations and challenges of current health care policies, and suggests improvements and reforms.<br><br>In the context of American politics, support for the ACA (also known as Obamacare) and policies aimed at expanding health care coverage and reducing costs are typically associated with liberal viewpoints, which prioritize wider access to health care and governmental involvement in ensuring health care equity. Conservative viewpoints, on the other hand, often emphasize limited government intervention in health care, market-based solutions, and personal responsibility for health care decisions and costs.<br><br>However, this text primarily focuses on specific policy aspects, challenges, and data related to health care for AYAs without	neutral

			advocating a specific political ideology. It aims to present an informed perspective on health care policy issues, particularly those affecting young individuals, and suggests practical steps for improvement within the existing legislative framework.	
AAP	The Role of Pediatricians in Global Health	12/1/2018	The text provided does not neatly align with either a liberal or conservative viewpoint in the traditional sense of American political ideologies. It focuses on global health, specifically child health, and discusses the importance of pediatricians in addressing health disparities and promoting equitable health care worldwide.<br><br>The key points emphasized in the text are:<br><br>1. **Global Health Focus:** The text highlights the importance of global health, defining it as a discipline that prioritizes equity in health care and requires collective action across national borders.<br><br>2. **Child Health in Low- and Middle-Income Countries:** It notes that a significant portion of the world's children live in countries where barriers to health contribute to high rates of morbidity and mortality.<br><br>3. **Role of Pediatricians:** The text underscores the role of pediatricians in improving global child health, not only through direct patient care but also through teaching, research, and advocacy.<br><br>4. **Pediatric Education and Training:** There is a call for pediatricians to receive training in global health, with a focus on a range of topics from immigrant and refugee health to ethical considerations in international medical practice.<br><br>5. **Advocacy and Collaboration:** The text encourages pediatricians to advocate for policies supporting global child health and to collaborate with international partners.<br><br>This approach is generally humanitarian and globally oriented, focusing on health equity and the well-being of children worldwide. It doesn't specifically advocate for policies typically associated with either liberal or conservative platforms in the U.S. political context. Instead, it seems to transcend political affiliations, aiming for a universal goal of improved child health on a global scale.	neutral
AAP	Screening Examination of Premature Infants for Retinopathy of Prematurity	12/1/2018	The text you provided is a detailed and comprehensive policy statement on the screening and treatment of Retinopathy of Prematurity (ROP), a medical condition affecting the eyes of preterm infants. It does not align explicitly with either a liberal or conservative viewpoint, as it is primarily a medical and scientific document.<br><br>Liberal and conservative viewpoints generally relate to political ideologies and may influence policies in areas such as economics, social issues, and governance. However, in the realm of medical science and healthcare, the focus is predominantly on evidence-based practice, clinical guidelines, and scientific research, which are intended to be non-partisan and not inherently aligned with any specific political ideology.<br><br>This document presents clinical guidelines and recommendations based on scientific research and evidence in the field of ophthalmology, specifically concerning the treatment and management of ROP in preterm infants. It discusses the importance of timely screening and intervention to prevent blindness and visual impairment in these infants. Such guidelines are typically developed by medical professionals and researchers based on empirical evidence and are aimed at improving patient outcomes, regardless of political leanings.	neutral

AAP	Pediatric Readiness in the Emergency Department	11/1/2018	The text provided appears to be a comprehensive and detailed policy statement related to pediatric emergency care. It outlines guidelines and recommendations for emergency departments (EDs) to effectively care for children in emergency situations. The content is focused on ensuring that EDs are properly equipped and staffed to handle the unique physical and psychosocial needs of children in emergencies.<br><br>This text doesn't align specifically with either a liberal or conservative viewpoint. It is primarily a technical and professional document, aimed at improving healthcare practices in emergency departments. The focus is on practical and medical considerations rather than political ideologies. The recommendations are based on research and expert opinion, and they aim to enhance the quality of pediatric emergency care without delving into partisan perspectives or policy debates typically associated with liberal or conservative viewpoints.<br><br>In healthcare policy, political viewpoints might come into play in discussions about funding, access to care, or the role of government in healthcare. However, this document is centered on clinical guidelines and best practices, which are generally considered neutral in the context of political ideology.	neutral
AAP	Child Passenger Safety	11/1/2018	The text you provided is primarily focused on child passenger safety and is presented in a factual, evidence-based manner. It doesn't inherently align with a specific political viewpoint, whether liberal or conservative. The emphasis is on best practices for child restraint systems in vehicles, based on scientific research and safety data.<br><br>This approach is characteristic of public health and safety recommendations, which typically aim to be non-partisan and based on empirical evidence. The recommendations, such as using rear-facing car seats for as long as possible and keeping children under 13 in the rear seats, are based on studies showing their effectiveness in reducing injuries and fatalities in car accidents.<br><br>The American Academy of Pediatrics, which is cited as the source of these recommendations, is a professional organization that focuses on the health and well-being of children, and its guidelines are generally informed by scientific research rather than political ideologies.<br><br>Therefore, it's more accurate to categorize this text as a public health guideline rather than as aligning with a specific political viewpoint.	neutral
AAP	Ensuring Comprehensive Care and Support for Transgender and Gender-Diverse Children and Adolescents	10/1/2018	The text you've provided presents a detailed and comprehensive overview of the American Academy of Pediatrics' (AAP) stance and recommendations regarding the health care of children and youth who identify as transgender and gender diverse (TGD). It outlines various aspects such as definitions, epidemiology, mental health implications, gender-affirmative care, medical management, health disparities, family acceptance, and recommendations for pediatric care and education.<br><br>Regarding whether this text aligns with a liberal or conservative viewpoint, it's important to understand that such classifications can vary based on individual perspectives and the political context. However, in general terms:<br><br>1. **Liberal Viewpoint**: The text's emphasis on inclusivity, recognition of gender diversity, advocacy for equal rights and healthcare access, and opposition to discriminatory practices and conversion therapy are more commonly associated with liberal ideologies. Liberal perspectives typically prioritize individual rights, social equality, and support for marginalized communities, including LGBTQ+ individuals.<br><br>2. **Conservative Viewpoint**: Traditional conservative ideologies tend to emphasize traditional values and may be less supportive of changes that challenge established norms, including those related to gender identity. However, it's important to note that there's a spectrum within conservative views, and some conservatives may support or accept policies aimed at inclusivity and anti-discrimination based on a belief in individual freedoms and rights.<br><br>The AAP's policy statement, as described, seems to align more closely with liberal viewpoints, particularly in its advocacy for inclusive and affirmative care for TGD youth, its stance against discrimination and conversion therapy, and its emphasis on the need for societal and systemic changes to better accommodate and support TGD individuals. This alignment is reflective of a broader shift in many professional medical organizations towards recognizing and addressing the specific needs of the LGBTQ+ community.	liberal

AAP	The Teen Driver	10/1/2018	The text you've provided appears to be a comprehensive and factual overview of the risks and challenges associated with teenage driving. It doesn't explicitly align with either a liberal or conservative viewpoint. Instead, it focuses on presenting statistical data, research findings, and public health considerations related to adolescent driving behavior, risks, and policies.<br><br>The text discusses various factors that contribute to the high rate of motor vehicle crashes among teen drivers, such as inexperience, distraction, alcohol and drug use, and the impact of passengers. It also covers the effectiveness of graduated driver licensing (GDL) programs, the role of parents in influencing teen driving habits, and various risk factors specific to adolescent drivers.<br><br>This neutral, evidence-based approach is typical of public health and safety discussions, where the primary focus is on reducing risk and improving outcomes based on empirical evidence, rather than advocating for a particular political ideology.	neutral
AAP	Recommendations for Prevention and Control of Influenza in Children, 2018–2019	10/1/2018	The text you provided is a comprehensive and detailed set of guidelines and recommendations from the American Academy of Pediatrics (AAP) regarding influenza vaccination and treatment in children. This document is primarily medical and scientific in nature, focusing on best practices for influenza prevention and treatment based on research and clinical data.<br><br>It does not inherently align with a liberal or conservative political viewpoint. The focus of the text is on public health and pediatric care, emphasizing the importance of vaccination and antiviral treatments for children. It provides detailed recommendations based on age, health status, and risk factors, and includes information about vaccine types, dosing, and precautions for specific populations, such as pregnant women and healthcare workers.<br><br>Public health and medical recommendations like these are typically based on scientific evidence and clinical expertise rather than political ideologies. While individual attitudes towards vaccination can vary and may be influenced by personal beliefs or political viewpoints, the text itself is neutral and aims to provide guidance for the best medical care of children in relation to influenza.	neutral
AAP	Food Additives and Child Health	8/1/2018	Determining whether a text aligns with a liberal or conservative viewpoint can be complex, especially when it involves technical and scientific topics like food additives and health regulations. The text you've provided focuses on the regulation and health implications of food additives and chemicals in food packaging, emphasizing child health, public safety, and the need for regulatory reform at the FDA.<br><br>This text primarily reflects concerns typically associated with public health, environmental safety, and consumer protection. These issues are often more closely aligned with liberal viewpoints, which tend to emphasize the role of government in regulating industries to protect public health and the environment. The emphasis on the potential disproportionate impact of these additives on minority and low-income populations also aligns with liberal concerns about social justice and equity.<br><br>However, it's important to note that public health and safety are universal concerns that can transcend traditional political boundaries. Both liberal and conservative viewpoints might agree on the importance of ensuring the safety of food additives, although they might differ in their approaches to regulation and the role of government agencies like the FDA.<br><br>In summary, while the text leans more towards concerns typically highlighted by liberal viewpoints, aspects of it could be supported or contested by individuals across the political spectrum, depending on their specific views about public health, government regulation, and industry practices.	probably liberal
AAP	Advocating for Life Support Training of Children, Parents, Caregivers, School Personnel, and the Public	6/1/2018	The text provided appears to be primarily factual and educational, focusing on the importance of out-of-hospital cardiac arrest (OHCA) awareness, the benefits of bystander cardiopulmonary resuscitation (CPR), and the use of automated external defibrillators (AEDs), especially in pediatric cases. It doesn't explicitly align with either a liberal or conservative viewpoint, as it primarily discusses medical practices and recommendations without delving into political ideologies or policies.<br><br>The emphasis is on the importance of training and equipping individuals, including children, parents, caregivers, and school personnel, with life-saving skills and tools. It advocates for the implementation of life support training and AED placement in schools, which could be seen as a public health and safety issue rather than a partisan one.<br><br>While some aspects of public health policy can become politicized, the text itself is neutral and focuses on medical recommendations and the benefits of public education and preparedness in emergency medical situations. The approach to public health and safety, including education and resource allocation, can be influenced by political views, but the text doesn't explicitly advocate for a specific political stance.	neutral

AAP	School Bus Transportation of Children With Special Health Care Needs	5/1/2018	The text you provided is neutral and factual, focusing on the specific needs and safety requirements for transporting children with special needs in school buses. It does not align explicitly with either a liberal or conservative viewpoint. Instead, it addresses practical and safety considerations, legal requirements, and recommendations based on medical and educational expertise. This approach is typical of professional and academic texts, which aim to inform and guide practices based on research and established standards rather than advocate for a particular political viewpoint.	neutral
AAP	AAP Diversity and Inclusion Statement	4/1/2018	The text provided aligns more closely with a liberal viewpoint, particularly in its emphasis on diversity, inclusiveness, and opposition to intolerance, bigotry, bias, and discrimination. The focus on celebrating the uniqueness of each child, regardless of their background or identity, and the commitment to fostering a diverse and inclusive environment in the medical field, are key themes often associated with liberal perspectives.<br><br>Liberal viewpoints generally advocate for social equality, support for marginalized groups, and the promotion of diversity and inclusiveness. The American Academy of Pediatrics (AAP)'s statement reflects these values by emphasizing the importance of diversity in race, ethnicity, language, religion, disability, socioeconomic status, sexual orientation, and gender identity, both in their patient care and within their organization.<br><br>Conservative viewpoints, on the other hand, might place a greater emphasis on traditional values and might be more focused on individual responsibility and less on systemic issues like social diversity and inclusivity. However, it's important to note that these are generalizations and there can be a wide range of beliefs within both liberal and conservative spectrums.	liberal
AAP	Pediatric Medication Safety in the Emergency Department	3/1/2018	The text you've provided doesn't inherently align with a specific liberal or conservative viewpoint. It's a detailed, factual discussion about the challenges and potential strategies for improving medication safety in pediatric emergency departments. The focus is on technical and medical aspects of healthcare, particularly on reducing medication errors in children in emergency settings.<br><br>Political viewpoints, liberal or conservative, often involve debates over broader healthcare policy issues like healthcare funding, access to healthcare services, and the role of government in healthcare. This text, however, is centered around specific clinical practices and recommendations to enhance patient safety and doesn't delve into broader policy discussions that typically distinguish liberal and conservative viewpoints.	neutral
AAP	Advocacy for Improving Nutrition in the First 1000 Days to Support Childhood Development and Adult Health	2/1/2018	The text you provided is a comprehensive and factual discussion on the importance of maternal prenatal nutrition and the child's nutrition in the first 1000 days of life for neurodevelopment and lifelong mental health. It emphasizes the role of various nutrients in brain growth and development and the potential long-term consequences of nutritional deficiencies during this critical period. Furthermore, it highlights the need for public policies and healthcare providers to prioritize early-life nutrition and support programs like WIC and SNAP.<br><br>This text does not explicitly align with a traditional liberal or conservative viewpoint. Instead, it focuses on scientific and medical evidence regarding the importance of early childhood nutrition. However, the emphasis on public policy and support programs could resonate more with a liberal viewpoint, which often advocates for government intervention and support in public health and welfare programs. Conversely, conservative viewpoints might focus more on personal responsibility and less on government-led initiatives, but they can also support such programs when they align with broader goals like reducing long-term healthcare costs and promoting family health.<br><br>It's important to note that issues like childhood nutrition and public health can transcend political lines, with advocates from both sides recognizing the importance of these issues. The text seems to prioritize scientific evidence and public health perspectives over political ideologies.	probably liberal
AAP	Pediatricians and Public Health: Optimizing the Health and Well-Being of the Nation's Children	2/1/2018	The text you provided is focused on public health and pediatric care, emphasizing the importance of integrating clinical practice with public health initiatives to improve children's health at both individual and population levels. This approach advocates for collaboration between pediatricians and public health professionals to address a wide range of children's health issues, including chronic diseases, obesity, communicable diseases, injury prevention, and mental health.<br><br>The viewpoint presented in the text aligns more closely with a public health perspective, which typically emphasizes population-based approaches and the social determinants of health. This perspective is generally associated with liberal viewpoints, which tend to emphasize the role of government and public policy in addressing societal health issues and inequalities. However, the focus on collaboration, community engagement, and prevention can also resonate with some conservative viewpoints, especially those that recognize the role of community and local initiatives in public health.<br><br>It's important to note that public health as a discipline often transcends traditional political categorizations, aiming to combine a	neutral

			variety of approaches and strategies to improve health outcomes for all. The text focuses on practical and evidence-based strategies to improve children's health, which is a goal that can be supported across the political spectrum.	
AAP	Global Human Trafficking and Child Victimization	12/1/2017	The text provided presents a comprehensive and factual view on the issue of child trafficking, addressing its various dimensions including health impacts, public policy, medical education, research, and collaboration. This viewpoint aligns with a humanitarian and human rights perspective, focusing on the protection of children's rights and well-being. It does not explicitly align with a specific political ideology, such as liberal or conservative, as it transcends political boundaries in favor of a universal concern for children's safety and health. The emphasis is on a multidisciplinary approach to combat child trafficking, advocating for policies and practices that protect children and support victims, which is a universally acknowledged concern across various political spectrums.	neutral
AAP	Stigma Experienced by Children and Adolescents With Obesity	12/1/2017	The text provided doesn't neatly align with a typical liberal or conservative viewpoint, as it primarily focuses on the issues of weight stigma and obesity, particularly in youth, and the role of healthcare professionals in addressing these concerns. The perspective presented is more medical and social than political. However, some aspects of the viewpoint could be interpreted in the context of broader political ideologies:<br><br>1. **Liberal Viewpoint Alignment**:<br>- Emphasis on Social Justice and Inclusion: The text's focus on combating stigma and promoting inclusivity aligns with liberal values that emphasize social justice and equality.<br>- Advocacy for Systemic Change: The call for systemic changes in healthcare, education, and media to address weight stigma resonates with a liberal approach that often advocates for systemic reforms to address social issues.<br>- Focus on Empathy and Non-Discrimination: The emphasis on empathetic communication, understanding the complex causes of obesity, and avoiding judgment aligns with liberal values of tolerance and understanding diversity.<br><br>2. **Conservative Viewpoint Considerations**:<br>- Personal Responsibility: While the text emphasizes the harmful effects of weight stigma and the need for societal change, some conservative viewpoints might emphasize personal responsibility in matters of health and obesity.<br>- Limited Role of Government: Conservatives often advocate for a limited role of government in personal affairs. Therefore, they might support interventions at the individual or community level rather than broad systemic changes.<br><br>In conclusion, the text primarily presents a medical and social perspective on obesity and weight stigma, focusing on the negative impacts of stigma and the need for compassionate approaches in healthcare and society. While certain aspects may resonate more with liberal values, such as social justice and systemic change, the text doesn't explicitly align with a traditional political ideology. It's more centered around public health and social welfare considerations.	probably liberal
AAP	Infection Prevention and Control in Pediatric Ambulatory Settings	11/1/2017	The text provided is a comprehensive and detailed statement on infection prevention and control in pediatric ambulatory settings. It does not inherently align with either a liberal or conservative viewpoint as it is focused on medical practices, health care policies, and guidelines based on scientific research and evidence. The statement addresses a range of topics including hand hygiene, immunization, sterilization, and communication with health care facilities, emphasizing the importance of infection control measures in pediatric care settings.<br><br>This type of content is generally considered neutral in the context of political ideology, as it is focused on medical best practices and public health recommendations rather than on political issues or ideological beliefs. The primary aim of such statements is to provide guidance to health care professionals to ensure patient safety and effective infection control, irrespective of political affiliations or beliefs.	neutral

AAP	The Care of Children With Congenital Heart Disease in Their Primary Medical Home	11/1/2017	The text provided does not inherently align with a liberal or conservative viewpoint. It is a medical and policy statement regarding the management of congenital heart disease (CHD), focusing on clinical practices and guidelines for primary care providers. The statement emphasizes the importance of coordinated care, early detection, family support, nutritional guidance, and the need for addressing psychosocial and neurodevelopmental issues associated with CHD. It also discusses the role of electronic health records and advocacy for better CHD care.<br><br>This kind of medical policy statement typically transcends political leanings, focusing instead on the best practices for patient care based on current research and knowledge in the field. The emphasis is on improving patient outcomes, providing comprehensive care, and ensuring that patients with CHD receive the appropriate attention throughout their life stages, rather than pushing a particular political agenda.	neutral
AAP	Cord Blood Banking for Potential Future Transplantation	11/1/2017	The text you've provided is a policy statement on cord blood banking. It's primarily a scientific and medical document and does not explicitly align with either a liberal or conservative viewpoint in the typical political sense.<br><br>This document focuses on providing detailed information about cord blood banking, including its purposes, the differences between public and private banking, quality control, costs, and recommendations for parents and medical professionals. It discusses the medical benefits of cord blood transplantation and the operational standards for cord blood banks.<br><br>Such policy statements are generally aimed at informing medical practice and public understanding rather than advocating for a specific political ideology. It's more about medical ethics, healthcare practices, and patient education than about politics. However, indirectly, discussions around healthcare access, costs, and regulations can intersect with broader political viewpoints about healthcare systems and public health policy. For instance, discussions about public versus private healthcare services and their regulation might resonate differently with liberal and conservative audiences, given their typical stances on healthcare policy. But this text itself remains neutral and informative, primarily concerned with medical and ethical considerations.	neutral
AAP	Recommendations for Prevention and Control of Influenza in Children, 2017 – 2018	10/1/2017	The text you provided is a detailed and comprehensive set of recommendations and guidelines from the American Academy of Pediatrics regarding influenza vaccination and treatment, particularly for children. It is a scientific and medical document, primarily focused on public health and clinical practice.<br><br>This document does not inherently align with either a liberal or conservative viewpoint. It is a technical and medically-oriented text, based on scientific research and public health principles. It aims to provide evidence-based guidelines for healthcare professionals to optimize the prevention and treatment of influenza, particularly in children.<br><br>Political viewpoints (liberal or conservative) typically involve interpretations or perspectives on policies and their social implications. This document, however, is primarily focused on medical and scientific recommendations without delving into political ideologies or policy debates.<br><br>In general, public health recommendations such as these are intended to transcend political affiliations and are based on the best available scientific evidence to promote the health and well-being of the population.	neutral
AAP	Metric Units and the Preferred Dosing of Orally Administered Liquid Medications	9/1/2017	The text you provided is a comprehensive and detailed policy statement focusing on medical error prevention, specifically regarding the dosing of liquid medications for children. It advocates for the use of milliliter-based dosing and the provision of appropriate dosing devices to reduce medication overdoses and improve precision in drug administration.<br><br>This text is predominantly technical and medical in nature, centering on improving patient safety and reducing medical errors. It does not align explicitly with either a liberal or conservative political viewpoint. Instead, it focuses on evidence-based practices and public health recommendations to improve medication safety. The emphasis is on standardizing medication dosing to minimize errors, which is a universal concern that transcends political ideologies.<br><br>Issues like medication safety and error prevention are typically seen as non-partisan, aimed at improving health outcomes and patient care efficiency. While different political groups might have varied approaches to healthcare policy and management, the specific issue of medication dosing precision is generally a matter of scientific and medical consensus, rather than political debate.	neutral

AAP	Guidance on Forgoing Life-Sustaining Medical Treatment	9/1/2017	The text you've provided doesn't align specifically with either a liberal or conservative viewpoint. It focuses on ethical considerations and guidelines in pediatric health care, particularly regarding life-sustaining medical treatment (LSMT). The content is centered around medical ethics and best practices, aiming to provide balanced and compassionate care for children in critical conditions. It discusses the importance of considering the best interests of the child, balancing benefits and burdens of treatment, involving families in decision-making, and respecting legal and ethical guidelines.<br><br>Such discussions typically transcend traditional political divides, focusing instead on medical, ethical, and legal considerations. The emphasis is on patient-centered care, ethical decision-making, and the involvement of families and interdisciplinary teams in the process. These principles are universally relevant across different political or ideological perspectives.	neutral
AAP	Early Childhood Home Visiting	9/1/2017	The text provided aligns more closely with a liberal viewpoint, primarily because it advocates for extensive public health programs, federal funding, and systemic approaches to address issues related to family welfare, child development, and poverty alleviation. Key aspects that suggest a liberal orientation include:<br><br>1. **Support for Public Health Programs and Federal Funding:** The text emphasizes the importance of high-quality home-visiting services and unwavering federal funding for these initiatives. The call for robust, state-supported, and federally-funded programs aligns with liberal perspectives that often advocate for strong government involvement in social welfare and public health initiatives.<br><br>2. **Focus on Comprehensive, Systemic Solutions:** The text discusses the integration of home visiting within a broader early childhood system, emphasizing a coordinated and systemic approach. This holistic perspective is typical of liberal viewpoints, which often prioritize systemic changes and integrated solutions to social issues.<br><br>3. **Emphasis on Social Justice and Equity:** The history and development of home visiting in the United States are rooted in social justice movements. The focus on supporting high-risk groups, such as adolescent mothers, single-parent families, recently incarcerated adolescents, and immigrants, reflects a commitment to social equity and justice, hallmarks of liberal ideology.<br><br>4. **Advocacy for Evidence-Based Programs and National Evaluation:** The text supports the expansion of evidence-based programs and a coordinated national evaluation to confirm best practices and cost-efficiency. This focus on evidence-based policy-making and accountability in public spending is consistent with modern liberal approaches to governance.<br><br>5. **Multigenerational Interventions and Poverty Alleviation:** The text highlights the role of home visiting in improving child health and family well-being across multiple domains, including economic self-sufficiency. This approach to tackling poverty through multigenerational interventions reflects a liberal viewpoint that seeks to address the root causes of poverty through comprehensive services.<br><br>6. **Global Perspective on Home Visiting:** The inclusion of information about home visiting programs in other industrialized nations, many of which provide these services as part of a comprehensive maternal and child health system, aligns with a liberal inclination toward learning from and implementing international best practices in social welfare.<br><br>While the text may find some support among conservatives, especially those who prioritize family values and early childhood development, its strong emphasis on federal funding, systemic change, and social justice initiatives is more characteristic of liberal perspectives.	liberal
AAP	Age Limit of Pediatrics	9/1/2017	The text you've provided doesn't align strictly with a liberal or conservative viewpoint in the traditional political sense. It is a policy statement focused on pediatric care, addressing the age limits and guidelines for pediatric services. This statement emphasizes the individualization of care, suggesting that decisions about when to transition from pediatric to adult healthcare should be based on the unique needs of each patient and family, rather than on arbitrary age limits.<br><br>This approach is more about healthcare policy and best practices within the field of pediatrics, rather than a reflection of a specific political ideology. It promotes a patient-centered and flexible approach to healthcare, which is a common perspective in the medical community, irrespective of political leanings.<br><br>In the context of broader political debates, both liberal and conservative viewpoints might find aspects of this statement to agree with. For instance, conservatives might appreciate the emphasis on individual decision-making between patients, families, and physicians. Liberals might support the focus on accommodating the needs of young adults and children with special health care	neutral

			needs. However, these interpretations would depend heavily on the broader context of each political viewpoint and their stances on healthcare policy.	
AAP	Principles of Child Health Care Financing	9/1/2017	The text you've provided generally aligns with a liberal viewpoint. It emphasizes the importance of the Patient Protection and Affordable Care Act (ACA) in increasing health insurance coverage for children and young adults, advocates for comprehensive and affordable health care for all children and adolescents, and supports the maintenance and expansion of Medicaid and the Children's Health Insurance Program (CHIP). These positions are typically associated with liberal or progressive political views, which often prioritize extensive government involvement in providing social services, including health care.<br><br>The emphasis on universal health care coverage, the expansion of government-funded programs, and the focus on reducing health disparities are characteristic of liberal policy positions. Additionally, the text's advocacy for maintaining health care as an entitlement and opposing funding cuts or structural changes to Medicaid that would reduce benefits or access aligns with liberal priorities of protecting and expanding social welfare programs.<br><br>Conservative viewpoints, on the other hand, often emphasize limited government intervention, fiscal conservatism, and the promotion of private sector solutions for health care. They might advocate for less government spending on health care, more market-based solutions, and personal responsibility in health care decisions.<br><br>This text specifically addresses pediatric health care, emphasizing the importance of ensuring access and coverage for children and young adults, which is a focal area of concern in liberal health care policies.	liberal
AAP	Elimination of Perinatal Hepatitis B: Providing the First Vaccine Dose Within 24 Hours of Birth	9/1/2017	The text you've provided is a medical policy statement on hepatitis B vaccination. It presents factual information and updated guidelines based on scientific research and clinical findings. This type of medical guideline is typically neutral and focused on public health rather than aligning with a specific political viewpoint, whether liberal or conservative.<br><br>The emphasis is on evidence-based practices for reducing the incidence of perinatal hepatitis B, which is a non-partisan issue concerning public health and safety. It discusses the effectiveness of hepatitis B vaccine, the importance of early vaccination, and strategies for preventing perinatal transmission of hepatitis B.<br><br>In general, public health guidelines and medical recommendations are based on scientific evidence and are intended to inform healthcare professionals and the public about the best practices for health and safety. They are not typically framed within the context of political ideologies.	neutral

AAP	Guiding Principles for Team-Based Pediatric Care	8/1/2017	The text provided appears to be a policy statement or guideline from the American Academy of Pediatrics (AAP) regarding team-based care for children. It emphasizes the importance of a holistic approach to pediatric care that involves a variety of stakeholders, including medical providers, community partners, and importantly, children and their families.<br><br>This text aligns with principles that are generally supported across the political spectrum but may resonate more strongly with a liberal viewpoint due to its emphasis on collaborative, community-oriented approaches and the integration of a wide range of services and supports. Key aspects of this text that might align with a liberal perspective include:<br><br>1. **Holistic and Inclusive Care**: The focus on addressing the needs of children within the context of their families and communities aligns with a more progressive view of healthcare, which often emphasizes the importance of social determinants of health and community-based support systems.<br><br>2. **Team-Based Approach**: The advocacy for a collaborative, team-based approach to care, which includes teachers, state agencies, and other community members, aligns with liberal views that often stress the importance of collective effort and community involvement in addressing social issues, including healthcare.<br><br>3. **Focus on Vulnerable Populations**: The attention to the needs of children, who are considered a vulnerable population, and the emphasis on adapting care to their changing needs, resonates with liberal principles of social justice and equity.<br><br>4. **Investment in Infrastructure and Technology**: The call for investment in infrastructure and technology to support team-based care aligns with a liberal perspective that often advocates for proactive government investment in public services and infrastructure to improve societal outcomes.<br><br>However, the text does not strictly adhere to a purely liberal or conservative viewpoint. It focuses on improving healthcare for children, a goal that can be shared across the political spectrum. The emphasis on team-based care, quality of service, and the inclusion of a diverse range of stakeholders are principles that could be endorsed by conservatives as well, especially those who emphasize community engagement and efficient healthcare delivery models.<br><br>In summary, while certain aspects of the text may align more closely with liberal viewpoints, especially its emphasis on community involvement and holistic care, the fundamental goal of improving pediatric healthcare is a non-partisan issue and could find support across the political spectrum.	probably liberal
AAP	Quality Early Education and Child Care From Birth to Kindergarten	8/1/2017	The text provided appears to be a policy statement or a detailed review of the importance and benefits of high-quality early education and child care. It discusses the positive outcomes of early childhood education, the barriers to accessing high-quality care, and the role of pediatricians in promoting such education. It also outlines steps taken to improve the quality of early education and child care and offers recommendations for pediatricians and policymakers.<br><br>Regarding its alignment with political viewpoints:<br><br>- **Liberal Viewpoint**: The text may align more closely with liberal viewpoints in several respects. It emphasizes the role of government and public policy in improving access to and quality of early education and child care, which is typically a stance more associated with liberal politics. It also discusses investment in early childhood education as a means to address inequalities, a concern often highlighted in liberal agendas. Furthermore, the emphasis on regulation, state involvement, and the need for funding for these programs aligns with liberal perspectives that advocate for a stronger role of the government in social welfare and education.<br><br>- **Conservative Viewpoint**: While the text does not explicitly align with conservative viewpoints, certain aspects might be agreeable to a conservative audience. The focus on the long-term benefits and return on investment of early childhood education can resonate with fiscal responsibility, a common conservative value. However, the conservative approach typically favors less government intervention and regulation, which contrasts with the text's emphasis on increasing funding and regulatory measures.<br><br>In conclusion, while the text touches on themes that could find resonance across the political spectrum, its overall focus on government intervention, regulation, and public investment in early childhood education is more in line with liberal viewpoints.	liberal

AAP	Fruit Juice in Infants, Children, and Adolescents: Current Recommendations	6/1/2017	The text you provided is a comprehensive and factual overview of the nutritional aspects and health implications of fruit juice consumption in infants, children, and adolescents. It doesn't align explicitly with a liberal or conservative viewpoint as it focuses on scientific and health-related information rather than political or ideological perspectives.<br><br>This text covers various aspects such as the nutritional composition of fruit juice, its historical and current recommendations by pediatricians, the effects of juice consumption on health (like dental caries, weight gain, and juice-drug interactions), and guidelines for juice consumption. It also addresses regulatory definitions and standards for fruit juice labeling.<br><br>The approach in the text is evidence-based and aims to inform readers about the benefits and risks associated with fruit juice consumption in a pediatric context. It emphasizes the importance of balanced nutrition and the potential health risks of excessive juice consumption, without leaning towards any political or ideological stance.	neutral
AAP	Detention of Immigrant Children	5/1/2017	The text you provided focuses on the treatment and care of immigrant children, particularly those who are unaccompanied or in family units, and emphasizes the importance of their health and well-being. This perspective aligns more closely with liberal viewpoints for several reasons:<br><br>1. **Emphasis on Humanitarian Treatment and Rights of Children:** The text emphasizes the dignity, respect, and humane treatment of immigrant and refugee children. This focus on humanitarian values and the rights of vulnerable groups, including children, is generally more associated with liberal ideologies.<br><br>2. **Concern for the Conditions of Detention:** The text expresses concern about the conditions in which children are detained and calls for the improvement of these conditions. This aligns with liberal perspectives that often prioritize the welfare and humane treatment of immigrants and refugees.<br><br>3. **Advocacy for Comprehensive Health Care and Support Services:** The text advocates for comprehensive health care, legal services, and support services for immigrant children. The focus on extensive social support services is typically more aligned with liberal policies.<br><br>4. **Critique of Current Government Policies:** The text criticizes certain aspects of the Department of Homeland Security's handling of immigrant children, suggesting that these facilities do not meet basic standards for the care of children in residential settings. This critique of existing government policies, particularly those related to immigration enforcement, is often a feature of liberal viewpoints.<br><br>In summary, the text's focus on humanitarian treatment, concern for the conditions of detention, advocacy for comprehensive support services, and critique of current policies aligns more with liberal viewpoints. Conservative viewpoints, on the other hand, might emphasize stricter immigration controls and policies, and might have a different approach to the treatment and processing of immigrant children.	liberal
AAP	Nonemergency Acute Care: When It's Not the Medical Home	5/1/2017	The text you've provided is a detailed policy statement from the American Academy of Pediatrics (AAP) focusing on the optimal care for children in acute, nonemergency situations. It emphasizes the concept of a "medical home" as the best setting for such care, advocating for comprehensive, continuous, coordinated, and family-centered care. The statement also acknowledges the growth of alternative acute care entities like urgent care facilities, retail-based clinics, and telemedicine services. It emphasizes the importance of high-quality, safe, and coordinated care, regardless of where it is accessed, and urges integration and communication with the medical home to ensure continuous and coordinated care.<br><br>This text doesn't neatly align with traditional liberal or conservative political viewpoints. Instead, it focuses on pediatric healthcare best practices, emphasizing the importance of accessible, comprehensive, and coordinated healthcare for children. The statement addresses healthcare delivery from a medical and ethical standpoint rather than a political one.<br><br>The AAP's position is rooted in professional healthcare standards and practices, prioritizing the welfare of children and the effectiveness of healthcare delivery. It doesn't directly engage with political debates about healthcare systems, funding, or management, which are often where liberal and conservative viewpoints diverge significantly. Instead, it's more concerned with the practical, day-to-day realities of providing optimal healthcare to children, regardless of the broader political or economic context.	neutral

AAP	Needs of Kinship Care Families and Pediatric Practice	4/1/2017	The text provided seems to be a comprehensive and detailed exploration of kinship care arrangements and their impact on children in the United States. It appears to be a policy statement or a scholarly article that discusses various aspects of kinship care, including the pathways to such care, legal implications, federal legislation, and the needs of families and children involved in these arrangements.<br><br>Regarding its alignment with a liberal or conservative viewpoint, it's important to note that the text is primarily informative and focuses on presenting data and insights into kinship care rather than advocating a specific political ideology. However, certain aspects of the content can be interpreted in the context of broader political perspectives:<br><br>1. **Emphasis on Kinship Over Nonrelative Foster Care**: The text's support for kinship care over nonrelative foster care can be seen as aligning with a more liberal viewpoint, which often emphasizes the importance of family bonds and community support.<br><br>2. **Discussion of Federal and State Legislation**: The acknowledgment and support of federal and state laws to aid kinship care arrangements might align more with a liberal perspective, which typically advocates for government intervention and support in social welfare issues.<br><br>3. **Focus on the Needs of Vulnerable Populations**: The detailed discussion about the needs of children in kinship care and the challenges faced by their caregivers might resonate more with liberal ideologies, which often prioritize social justice and support for marginalized groups.<br><br>4. **Healthcare and Public Policy**: The emphasis on healthcare needs and public policy advocacy could be seen as a liberal standpoint, as it involves advocating for systemic change and support through legislation and public services.<br><br>In summary, while the text does not explicitly endorse a political ideology, certain elements of its content and focus on social welfare, family bonds, and government support in the context of kinship care might be more closely aligned with liberal viewpoints. However, it's important to recognize that topics like child welfare and family support can find relevance and support across the political spectrum.	probably liberal
AAP	The Child Witness in the Courtroom	3/1/2017	The text you provided appears to be a policy statement from the American Academy of Pediatrics (AAP) concerning the role of children as witnesses in court proceedings. It does not align strictly with either a liberal or conservative viewpoint. Instead, it focuses on a factual and research-based approach to the psychological and legal implications of children testifying in court. The statement addresses various aspects such as the history of child testimony in courts, psychological impacts, legal considerations, and recommendations for supporting child witnesses.<br><br>The focus is on the welfare of children and how to minimize their trauma when involved in legal proceedings, whether as victims or witnesses. This approach is more about child advocacy and protection rather than adhering to a specific political ideology. However, one could argue that the emphasis on protection and support for vulnerable individuals, like children, may resonate more with certain liberal social policies focused on welfare and protection of vulnerable groups. Conversely, the detailed discussion of legal procedures and reforms might appeal to conservative perspectives on law and order.<br><br>Overall, the statement is a comprehensive, nonpartisan review of the issues surrounding children in legal settings, grounded in research and clinical experience rather than political ideology.	neutral
AAP	Financing of Pediatric Home Health Care	3/1/2017	The text you provided seems to align more closely with a liberal viewpoint. It emphasizes the importance of comprehensive healthcare coverage, particularly for children with complex medical needs or developmental disabilities. The focus is on increasing access to pediatric home health care services, advocating for sufficient payment to providers, and ensuring that these services are recognized and supported by both public and private insurance plans.<br><br>Liberal viewpoints typically prioritize expanded healthcare access and coverage, especially for vulnerable populations. This text supports these ideas by advocating for policy changes and increased funding for pediatric home health care. It also emphasizes the role of government programs like Medicaid and the Affordable Care Act in providing essential health benefits, which is a common stance in liberal perspectives.<br><br>The text does not align with conservative viewpoints, which often emphasize reduced government involvement in healthcare, privatization, and personal responsibility over expanded public healthcare programs. The advocacy for increased government regulation, oversight, and funding in healthcare, as presented in your text, is more characteristic of liberal viewpoints.	liberal

AAP	Expert Witness Participation in Civil and Criminal Proceedings	3/1/2017	The text provided is primarily a policy statement from the American Academy of Pediatrics (AAP) concerning expert witness testimony, particularly in the context of pediatric cases. This statement emphasizes the importance of scientifically sound, unbiased, and ethical expert testimony in both civil and criminal proceedings. It doesn't neatly align with a traditional liberal or conservative political viewpoint. Instead, it focuses on professional standards and ethical obligations within the medical community, particularly among pediatricians.<br><br>- **Liberal Viewpoints:** Generally, liberal perspectives prioritize social justice, equality, and community solutions. They often advocate for change in social structures and support government intervention in addressing social issues.<br><br>- **Conservative Viewpoints:** Conservative perspectives typically emphasize tradition, personal responsibility, and limited government intervention in social affairs. They often prioritize individual liberty and market-based solutions to social problems.<br><br>The AAP's statement is more aligned with professional ethics and standards in medicine rather than a specific political ideology. It advocates for responsible, ethical, and well-informed participation of medical professionals in legal processes, which is a matter of professional conduct rather than a political stance. The recommendations focus on ensuring that medical expert testimony is accurate, unbiased, and based on current medical standards and knowledge, which transcends typical liberal or conservative viewpoints.	neutral
AAP	A Public Health Response to Opioid Use in Pregnancy	3/1/2017	The text provided appears to align more closely with a liberal viewpoint. This can be inferred from several key aspects:<br><br>1. **Public Health Approach vs. Punitive Measures:** The text emphasizes a public health response to the issue of opioid use during pregnancy, rather than punitive measures such as criminal prosecution and incarceration of pregnant women. This approach typically aligns with liberal perspectives, which often prioritize healthcare and rehabilitative strategies over punitive or criminal justice responses.<br><br>2. **Focus on Comprehensive Care and Treatment:** The emphasis on providing comprehensive obstetric care, opioid-replacement therapy, gender-specific substance use treatment programs, and improved funding for social services and child welfare systems reflects a liberal inclination. Liberals often advocate for more inclusive and comprehensive healthcare policies, especially in addressing complex social issues like substance abuse.<br><br>3. **Support from Professional Organizations:** The reference to the support of the American College of Obstetricians and Gynecologists and other healthcare organizations also suggests a liberal viewpoint. These organizations often advocate for evidence-based, patient-centered approaches that prioritize individual health and well-being, which aligns more closely with liberal ideologies.<br><br>4. **Emphasis on Prevention and Education:** The focus on preventing unintended pregnancies, improving access to contraception, and universal screening for substance use further indicates a liberal stance. Liberals typically support such preventative measures and educational approaches in healthcare.<br><br>5. **Equal Application of Healthcare Practices:** The advocacy for equal application of screening and treatment regardless of age, race, ethnicity, or socioeconomic status is in line with liberal values that emphasize equality and inclusivity in healthcare access.<br><br>Overall, the text's focus on public health responses, comprehensive care, support from medical organizations, preventative measures, and equality in healthcare aligns it more with liberal viewpoints.	liberal

AAP	The Adolescent’s Right to Confidential Care When Considering Abortion	2/1/2017	The text provided is a statement from the American Academy of Pediatrics (AAP) that discusses the rights of adolescents in relation to confidential care when considering abortion. It covers various aspects including the importance of adolescent access to healthcare, the role of parental involvement in abortion decisions, the impact of legislation mandating parental involvement, and the AAP's stance on these issues.<br><br>The viewpoint of this statement aligns more closely with a liberal perspective for several reasons:<br><br>1. **Emphasis on Individual Rights and Confidential Care**: The statement strongly supports the rights of adolescents to confidential care when considering abortion. This emphasis on individual rights and autonomy, particularly in reproductive health decisions, is typically associated with liberal viewpoints.<br><br>2. **Critique of Mandatory Parental Involvement Laws**: The AAP argues against legislation that mandates parental involvement in an adolescent's decision to obtain an abortion, stating that such laws do not promote family communication and can delay access to appropriate medical care. This criticism of restrictive laws aligns with liberal perspectives that often advocate for fewer restrictions on abortion access.<br><br>3. **Support for Comprehensive Health and Sexuality Education**: The AAP's support for comprehensive health and sexuality education, including the use of effective contraception, is a stance more commonly found in liberal ideologies, which tend to emphasize education and preventive measures in addressing issues like teen pregnancy.<br><br>4. **Recognition of the Diversity of Family Structures**: The statement acknowledges the complexity and diversity of modern family structures, suggesting a more inclusive approach that is characteristic of liberal perspectives.<br><br>5. **Focus on Adolescent Well-being Over Legislative Intent**: The AAP prioritizes the well-being and health of the adolescent over the intended benefits of legislation requiring parental involvement. This approach, which puts individual welfare at the forefront, is more in line with liberal principles.<br><br>It's important to note that the distinction between liberal and conservative viewpoints can vary based on specific issues, cultural contexts, and individual interpretations. The AAP's statement is primarily focused on the health and well-being of adolescents, guided by medical and ethical considerations, rather than political ideology.	liberal
AAP	Donor Human Milk for the High-Risk Infant: Preparation, Safety, and Usage Options in the United States	1/1/2017	The text provided primarily focuses on medical and health care considerations regarding the use of donor human milk for high-risk infants, particularly those with low birth weight or severe intestinal disorders. It discusses the benefits and risks associated with pasteurized and nonpasteurized donor milk, as well as issues related to the collection, pasteurization, and distribution of donor milk. Additionally, it addresses concerns about safety, nutrient loss during pasteurization, and the importance of prioritizing the mother’s milk, with donor milk as a supplement.<br><br>This text does not inherently align with a specifically liberal or conservative viewpoint. It is predominantly a medical and health policy-related document, presenting information based on scientific research and health care practices rather than political ideology. The focus is on the safety, efficacy, and accessibility of donor human milk in neonatal care, which are topics generally considered within the realm of public health and medical science rather than partisan politics.<br><br>However, aspects of the text that discuss the development of public policy to improve and expand access to pasteurized donor milk, including governmental and private financial support for donor milk banks, could be interpreted within a political context. Views on government involvement in public health initiatives and financial support for health services can vary between liberal and conservative ideologies. Liberals might be more supportive of increased government intervention and funding in public health matters, while conservatives might favor more private-sector solutions and less government involvement. Nonetheless, these are general tendencies and may not apply universally. The text itself remains focused on health care and medical practice, without explicit political advocacy.	neutral

AAP	Off-Label Use of Medical Devices in Children	1/1/2017	The text you've provided doesn't align explicitly with a liberal or conservative viewpoint. It is primarily a factual and policy-oriented discussion about the use of medical and surgical devices in pediatric care, specifically addressing the challenges and regulatory aspects of off-label use of devices in children. This discussion is more about medical ethics, regulatory policy, and healthcare practices rather than a political ideology.<br><br>The text outlines the difficulties in developing and approving medical devices specifically for children, the necessity of off-label use due to these challenges, and the efforts by organizations like the American Academy of Pediatrics (AAP) to improve the situation. It emphasizes the need for specific design, testing, labeling, and market availability of devices for pediatric medical and surgical conditions.<br><br>Such issues are generally addressed from a healthcare and patient welfare perspective, rather than being framed within a political ideological context. The focus is on improving pediatric healthcare and addressing regulatory and market challenges, which can be of concern to people across the political spectrum.	neutral
AAP	A New Era in Quality Measurement: The Development and Application of Quality Measures	1/1/2017	The text provided does not align strictly with either a liberal or conservative viewpoint. It is a comprehensive and neutral exposition on the topic of quality measures in pediatric healthcare, discussing their development, application, and challenges without favoring a particular political ideology.<br><br>The focus is on improving healthcare quality and patient safety through various measures and programs, addressing both the practical aspects of measure development and the specific challenges in pediatric care. It emphasizes the importance of evidence-based practices, stakeholder involvement, and continuous improvement in healthcare quality, which are generally non-partisan issues in healthcare policy.<br><br>The text also highlights the need for increased investment in pediatric healthcare quality measures and the alignment of these measures with broader healthcare goals, suggesting a commitment to systemic improvements in healthcare that transcend political leanings.	neutral
AAP	Responsible Innovation in Children's Surgical Care	1/1/2017	The text provided does not explicitly align with a liberal or conservative viewpoint. It is primarily a scientific and ethical discussion about innovation in surgery, particularly in the context of pediatric care. The focus is on the challenges and considerations involved in implementing new surgical techniques and devices, the role of regulatory bodies like the U.S. Food and Drug Administration (FDA), and the ethical dimensions of innovation in surgical practices.<br><br>The text emphasizes the importance of patient safety, informed consent, and ethical conduct in the process of introducing new surgical therapies. It discusses the balance between encouraging innovation and ensuring patient protection and transparency. It also highlights the need for cultural competence and respectful approaches in patient care, and the unique challenges in pediatric surgery, including issues related to consent and the limited role of the FDA in regulating devices for children.<br><br>This approach is more aligned with a scientific and professional perspective rather than being situated within a political ideology. It addresses the complexities of medical innovation from a standpoint of patient care, ethics, and regulatory compliance, rather than from a political standpoint.	neutral
AAP	Addressing Early Childhood Emotional and Behavioral Problems	12/1/2016	The text you've provided seems to adopt a neutral, clinical approach rather than aligning explicitly with a liberal or conservative viewpoint. It focuses on the clinical and evidence-based aspects of diagnosing and treating emotional, behavioral, and relationship problems in young children, emphasizing the importance of early intervention and access to care.<br><br>This perspective is rooted in scientific research and medical best practices, rather than being driven by a particular political ideology. The emphasis is on practical, evidence-based solutions and the importance of addressing these issues early in a child's life, which is a common stance in the field of pediatric healthcare and mental health.<br><br>Both liberal and conservative viewpoints may find elements to support within this text. For instance, conservatives might appreciate the focus on family involvement in treatment, while liberals might support the call for increased access to healthcare services and the importance of addressing community and societal factors. However, the text itself does not explicitly endorse or reject either political ideology, instead focusing on the medical and scientific aspects of pediatric mental health care.	neutral

AAP	Disclosure of Adverse Events in Pediatrics	12/1/2016	The text provided doesn't neatly align with a typical liberal or conservative viewpoint. It primarily focuses on the issues of patient safety, medical errors, and the ethical and practical considerations surrounding the disclosure of adverse events (AEs) in the healthcare system. These topics are generally non-partisan and concern systemic improvements in healthcare quality and transparency.<br><br>Key points discussed include:<br><br>1. **Importance of Patient Safety**: The text highlights the significance of patient safety as a crucial aspect of healthcare quality. This focus is generally universal and not specific to any political ideology.<br><br>2. **Ethical Obligation of Disclosure**: The mention of an ethical obligation to inform patients and families about preventable AEs and medical errors aligns with a broader commitment to transparency and patient rights, values that are typically emphasized across the political spectrum.<br><br>3. **Barriers to Disclosure**: The discussion about barriers such as fear of liability, while not explicitly political, touches upon the broader debate around medical malpractice laws and the legal system, which can have different interpretations from liberal or conservative perspectives.<br><br>4. **Legal and Systemic Changes**: Suggested changes to the legal system and the development of policies and programs for better disclosure practices are aimed at systemic improvements. These kinds of recommendations often find support across political lines, though the specifics of such changes might be debated differently by liberals and conservatives.<br><br>5. **Economic and Emotional Impact**: The text also touches on the economic costs of AEs and the emotional impact on healthcare providers, which are issues of concern to both liberal and conservative viewpoints, although the approaches to addressing these impacts might vary.<br><br>In summary, while the text deals with topics that are relevant to healthcare policy, a field that often involves political debate, the specific content here is more centered on quality improvement, ethical considerations, and systemic challenges in healthcare, rather than on partisan issues.	neutral
AAP	Media and Young Minds	11/1/2016	The text you provided does not align explicitly with either a liberal or conservative viewpoint. It is a policy statement focused on the impact of media on the health and development of children aged 0 to 5 years. The statement is scientific and evidence-based, discussing various aspects of media use, including its potential educational benefits, health concerns, and the importance of parental guidance in media consumption.<br><br>The text emphasizes the need for high-quality programming, parental involvement in media use, and the importance of balancing digital media with other activities crucial for healthy development. It also addresses concerns like obesity, sleep disruption, and developmental delays associated with excessive media use.<br><br>This approach is characteristic of public health and child development research, focusing on evidence-based recommendations rather than political ideologies. The emphasis is on guiding parents and pediatric providers in managing young children's media use based on current research and best practices in child health and development.	neutral
AAP	SIDS and Other Sleep-Related Infant Deaths: Updated 2016 Recommendations for a Safe Infant Sleeping Environment	11/1/2016	The text provided presents a scientific and medical perspective on sleep-related infant deaths, including sudden infant death syndrome (SIDS) and other sleep-related causes. It does not align explicitly with either a liberal or conservative viewpoint. Instead, it focuses on providing evidence-based recommendations for reducing the risk of these deaths, which is a public health concern rather than a politically partisan issue.<br><br>The document details various risk factors for SIDS and other sleep-related infant deaths, offers definitions of relevant terms, and outlines recommendations for safe infant sleep practices. These recommendations are based on extensive research and are aimed at guiding healthcare providers, parents, and caregivers in reducing the risk of sleep-related infant deaths.<br><br>The content is factual and oriented towards health and safety, devoid of political or ideological bias. It's a reflection of the consensus in the medical community regarding best practices for infant care, particularly in relation to sleep. The American Academy of Pediatrics, a respected authority in pediatric health, is cited, emphasizing the scientific and medical basis of the information.	neutral

AAP	Media Use in School-Aged Children and Adolescents	11/1/2016	The text you provided appears to be a comprehensive and balanced examination of the impact of media on children and adolescents. It does not strictly align with either a liberal or conservative viewpoint. Instead, it provides an objective analysis of both the benefits and risks associated with media use among young people.<br><br>Key points include:<br><br>1. **Benefits of Media Use**: Exposure to new ideas and information, opportunities for social contact and support, and access to health-promotion messages.<br><br>2. **Risks of Media Use**: Negative health effects like impact on weight and sleep, exposure to inappropriate content, and risks to privacy and confidentiality.<br><br>3. **Parental Challenges**: Monitoring children's media use and serving as positive role models.<br><br>4. **Approach to Media Use**: The statement suggests a personalized approach to media use that considers a child's developmental stage, rather than a one-size-fits-all strategy.<br><br>5. **Role of Pediatricians and Parents**: Collaboration to develop a Family Media Use Plan tailored to individual children's needs.<br><br>This policy statement seems to prioritize a nuanced and evidence-based approach to managing children's media use, focusing on health and development rather than aligning with a specific political ideology.	neutral
AAP	Handoffs: Transitions of Care for Children in the Emergency Department	11/1/2016	The text provided does not inherently align with either a liberal or conservative viewpoint. It is a detailed and objective discussion on the topic of transitions of care (ToCs) in healthcare settings, particularly in emergency medicine. The focus is on improving patient safety and care quality through structured transition processes, drawing upon practices from high-risk industries like aerospace and nuclear power.<br><br>This text emphasizes the importance of standardization and the need for further research to identify the most effective models for ToCs. It discusses various aspects of patient care transitions, including challenges and strategies for effective communication among healthcare providers, and the impact of such transitions on patient outcomes. The text also highlights the need for training healthcare professionals in structured ToC processes and incorporating technology like electronic health records (EHRs) to improve ToC accuracy.<br><br>Overall, the text is a technical and professional examination of ToCs in healthcare, focusing on improving patient outcomes and reducing errors in a highly specialized field. It does not promote or critique any political ideology or policy, which are typically the markers of a text being aligned with a liberal or conservative viewpoint.	neutral
AAP	Recommendations for Prevention and Control of Influenza in Children, 2016–2017	10/1/2016	The text you provided is a detailed, evidence-based health guideline from the American Academy of Pediatrics (AAP) regarding the prevention and treatment of influenza in children. This document focuses on recommending the annual influenza vaccine for everyone aged 6 months and older and provides specific guidance on the types of vaccines available, their effectiveness, and the usage of antiviral medications in treatment.<br><br>This text does not align specifically with either a liberal or conservative viewpoint as it is a scientific and medical guideline. It is based on research and clinical evidence rather than political or ideological beliefs. Health recommendations like these are typically grounded in scientific research and clinical evidence, aiming to provide the best care and prevention strategies for the population, in this case, children and adolescents. The focus is on public health and safety, which transcends political affiliations.<br><br>It's important to note that public health guidelines are generally designed to be non-partisan and are based on scientific evidence and the consensus of medical experts. Their primary goal is to protect and improve the health of the population, regardless of political leanings.	neutral
AAP	The Pediatrician's Role in Optimizing School Readiness	9/1/2016	The text provided does not align strictly with either a liberal or conservative viewpoint in the traditional political sense. Instead, it focuses on a comprehensive understanding of school readiness, emphasizing the importance of physical health, social and emotional development, language skills, cognitive abilities, and family and community involvement in children's early education. This perspective is largely educational and developmental, rather than political, and seeks to address the multifaceted nature of preparing children for school. It highlights the role of pediatricians, families, communities, and schools in supporting children's development, suggesting a holistic approach to education and child development that crosses traditional political lines.	neutral

AAP	Medication-Assisted Treatment of Adolescents With Opioid Use Disorders	9/1/2016	The text provided does not explicitly align with either a liberal or conservative viewpoint. It primarily presents factual information and research findings about opioid use disorders among US youth, treatment options, and the need for improved access to these treatments. It focuses on the medical and public health aspects of opioid addiction, including the effectiveness of medications and the importance of substance use disorder counseling.<br><br>Political viewpoints, whether liberal or conservative, might interpret or emphasize different aspects of this issue. For example:<br><br>- A liberal viewpoint might focus on the need for increased funding for public health programs, advocating for broader access to healthcare, and addressing social and economic factors contributing to addiction.<br>- A conservative viewpoint might emphasize personal responsibility, the role of family and community support in addressing addiction, and concerns about potential misuse of opioid replacement therapies.<br><br>However, the text itself remains neutral and informative, aiming to provide a comprehensive understanding of the opioid crisis among youth and the available treatments without endorsing a specific political stance.	neutral
AAP	Recommendations for Serogroup B Meningococcal Vaccine for Persons 10 Years and Older	9/1/2016	The text you provided is a detailed policy statement regarding recommendations for the prevention of serogroup B meningococcal disease using two specific vaccines. This statement is focused on public health and medical recommendations and does not explicitly align with a liberal or conservative viewpoint.<br><br>Public health policy, especially regarding vaccination, often intersects with broader political and social discussions. However, the content of this statement is primarily scientific and medical in nature, centered around the efficacy, safety, and recommended use of the vaccines MenB-FHbp and MenB-4C. It discusses the conditions under which these vaccines are recommended, the age groups for which they are approved, and other relevant clinical data.<br><br>In the current political landscape, issues surrounding public health and vaccinations can sometimes be interpreted through a partisan lens, but the statement itself is neutral and focused on medical advice and evidence. It represents a position based on scientific research and public health expertise, rather than a political ideology.<br><br>Public health policies, such as vaccination recommendations, aim to address the health needs of the population based on scientific evidence and epidemiological data, and they generally transcend traditional political divides of conservative and liberal viewpoints.	neutral
AAP	Medical Versus Nonmedical Immunization Exemptions for Child Care and School Attendance	9/1/2016	The text provided presents a factual, evidence-based perspective on routine childhood immunizations, emphasizing their importance in public health and the concept of community immunity. It outlines the rationale behind immunization requirements for school attendance, discusses medical and nonmedical exemptions, and addresses legal and ethical considerations.<br><br>This approach aligns more closely with what is typically considered a liberal viewpoint, especially in the current socio-political context of the United States. The liberal perspective generally emphasizes the role of government in protecting public health and the welfare of the community, including through vaccination policies. This viewpoint tends to support the idea that individual choices, such as the decision to vaccinate, should sometimes be guided or regulated by public health concerns, especially when those choices can have significant impacts on others.<br><br>In contrast, a conservative viewpoint often emphasizes individual liberties and personal choice, and may be more supportive of nonmedical exemptions to vaccination on the basis of personal or religious beliefs. Conservatives may argue for less government intervention in personal health decisions and might be more inclined to support policies that allow for a wider range of exemptions to vaccination requirements.<br><br>However, it's important to note that these generalizations do not apply to all liberals or conservatives, and viewpoints on vaccinations can vary widely within each group. Additionally, the political alignment of public health issues like vaccination can differ across countries and over time.	liberal

AAP	Management of Pediatric Trauma	8/1/2016	The text provided doesn't align distinctly with a liberal or conservative viewpoint. It's primarily factual and focuses on the public health issue of childhood injury in the United States. The content emphasizes the prevalence and impact of injuries among children and adolescents, the costs associated with these injuries, and the importance of an effective trauma system. It highlights the need for comprehensive care, including prehospital, hospital, and rehabilitation services, and stresses the importance of injury prevention and preparedness for mass casualty events.<br><br>The text is largely apolitical and doesn't advocate for specific policies that are traditionally associated with either liberal or conservative ideologies. Instead, it presents information in a way that underscores the significance of childhood injury as a public health concern, the necessity of an inclusive trauma system, and the importance of research and continuous improvement in pediatric trauma care. This approach is more aligned with public health and medical best practices rather than a particular political ideology.	neutral
AAP	Achieving Quality Health Services for Adolescents	8/1/2016	The text provided does not specifically align with a liberal or conservative viewpoint. It is a comprehensive and objective analysis of the patient-centered medical home (PCMH) model and its application in adolescent healthcare, presented by the American Academy of Pediatrics. The focus is on improving the quality and efficiency of health care for adolescents through this model. It addresses various aspects of adolescent health care, including developmentally appropriate care, confidentiality, the transition to adult care, and the use of electronic health records. The text emphasizes evidence-based approaches, quality measures, and the importance of research in advancing adolescent health care. It does not advocate for a specific political ideology but rather focuses on medical best practices and patient-centered approaches.	neutral
AAP	Virtual Violence	8/1/2016	The text you've provided aligns more closely with a liberal viewpoint, particularly in its approach to addressing the issue of virtual violence and its impact on children. The focus on scientific research, public health concerns, and the advocacy for legislative and policy changes to protect children from exposure to media violence are characteristics often associated with liberal perspectives.<br><br>Liberal viewpoints generally emphasize the role of government and public policy in addressing societal issues and protecting vulnerable populations, such as children. The text's emphasis on the need for pediatricians, parents, and policymakers to be actively involved in mitigating the effects of virtual violence reflects this approach. Additionally, the call for more regulation of media content and the development of a robust, reliable rating system overseen by the federal government aligns with liberal tendencies to advocate for increased government oversight in media and public health issues.<br><br>On the other hand, conservative viewpoints often prioritize individual responsibility, parental control over children's media consumption, and limited government intervention in private industries, such as entertainment and media. Conservatives might argue that it is primarily the responsibility of parents to monitor and control their children's exposure to media violence, rather than relying on government legislation or industry regulation.<br><br>However, it's important to note that the distinction between liberal and conservative viewpoints is not always clear-cut, and there can be considerable overlap or variation within these broad categories. Additionally, individuals and groups may hold different positions on specific issues that do not always align neatly with generalized liberal or conservative ideologies.	liberal
AAP	Informed Consent in Decision-Making in Pediatric Practice	8/1/2016	The text you provided seems to align with neither a strictly liberal nor conservative viewpoint, but rather focuses on ethical considerations in healthcare, specifically in pediatric care. It emphasizes the importance of informed consent, including the evolving role of children and adolescents in medical decision-making. The principles discussed are rooted in ethical theories and legal precedents, focusing on patient autonomy, beneficence, and justice. This perspective is more aligned with bioethical principles than with any particular political ideology.<br><br>The text highlights the American Academy of Pediatrics' (AAP) stance on informed consent in pediatric practice, discussing the historical development and the current understanding of this concept. It explains the differences in decision-making processes between adults and children, acknowledging children's developing capacity for decision-making and the importance of parental permission and child assent. The document also considers various standards for surrogate decision-making in pediatrics, recognizing the role of family in the decision-making process and the unique needs of pediatric patients.<br><br>Overall, the text is more focused on the ethical dimensions of pediatric healthcare and informed consent, rather than on any political or ideological standpoint.	neutral

AAP	Substance Use Screening, Brief Intervention, and Referral to Treatment	7/1/2016	The text provided primarily aligns with a non-partisan, medical and public health viewpoint. It emphasizes the importance of adolescent substance use prevention, detection, assessment, and intervention in healthcare, particularly within pediatric care. The focus is on the American Academy of Pediatrics' recommendations and policies regarding substance use in adolescents, highlighting the role of pediatricians in addressing this issue.<br><br>This approach is more centered on medical best practices and public health concerns rather than being distinctly liberal or conservative. Both liberal and conservative viewpoints might agree on the significance of addressing substance use in adolescents, although they may differ in their preferred methods of implementation or funding. The text underlines the need for research, universal screening, intervention strategies, and the involvement of healthcare providers in combating substance use disorders among adolescents, which is a generally accepted public health perspective.	neutral
AAP	Prevention of Childhood Lead Toxicity	7/1/2016	The text you've provided doesn't align with a strictly liberal or conservative viewpoint. It's primarily a factual and scientific discourse on lead exposure and its effects, especially in children. The focus is on the environmental and health impacts of lead, the historical reduction in lead exposure due to policy changes, and the continuing challenges and strategies for managing lead exposure in children.<br><br>This type of information typically transcends political ideologies, as it is based on scientific research and public health data. Both liberal and conservative viewpoints might converge on the importance of addressing lead exposure, although they might propose different methods or policies to address the issue. Liberals might emphasize stricter environmental regulations and government-funded programs for lead abatement, while conservatives might focus on individual responsibility and private sector solutions.<br><br>The text's emphasis on evidence-based approaches, historical data, and the need for ongoing public health measures to reduce lead exposure can be seen as a universal concern, rather than one confined to a specific political ideology.	neutral
AAP	Role of the School Nurse in Providing School Health Services	6/1/2016	The text provided primarily aligns with a neutral, professional viewpoint rather than a specifically liberal or conservative political stance. It focuses on the role of school nurses in promoting health and wellness among school-aged children, emphasizing the importance of their collaboration with pediatricians and the school community. The content is largely factual, addressing the historical development of the school nursing role, its expansion over time, and its impact on student health and academic success.<br><br>Key elements include:<br><br>1. **Historical Background**: It outlines the evolution of the school nursing role since its inception in 1902, highlighting its expansion from reducing absenteeism to encompassing a wider range of health services, including chronic disease management and mental health support.<br><br>2. **Interdisciplinary Collaboration**: The text emphasizes the importance of collaboration between school nurses, pediatricians, families, and the wider school community. This collaboration is viewed as essential for addressing the complex health needs of students.<br><br>3. **Legal and Medical Context**: It discusses legal and medical changes that have influenced the need for professional nurses in schools, such as disability rights laws and advancements in medical care for chronic conditions.<br><br>4. **Societal Impact**: The role of school nurses in addressing broader societal issues like obesity prevention, mental health, and public health initiatives is highlighted.<br><br>5. **Policy and Practice Recommendations**: The text concludes with specific recommendations for pediatricians to support and collaborate with school nurses.<br><br>The perspective presented is primarily educational and health-focused, with an emphasis on best practices in school health services. It does not delve into political ideologies but rather concentrates on the professional and practical aspects of school nursing and its integration within the broader health and educational systems.	neutral

AAP	Financing Graduate Medical Education to Meet the Needs of Children and the Future Pediatrician Workforce	4/1/2016	The text presented primarily aligns with a liberal viewpoint, particularly in its emphasis on increased public funding and support for graduate medical education (GME) in pediatrics. It advocates for expanded government involvement and financial support in healthcare education, which is generally consistent with liberal policy preferences. The text emphasizes the importance of ensuring adequate numbers of trained pediatricians and specialists, which reflects a concern for public welfare and access to healthcare, key components of liberal ideology.<br><br>The recommendations for broadening the scope of funding sources, including government agencies, hospitals, and the healthcare industry, align with liberal perspectives on shared responsibility and the role of the public sector in addressing societal needs. The focus on ensuring that all children, especially those with special healthcare needs, have access to optimal healthcare resonates with liberal values of equity and social justice.<br><br>However, it's important to note that while the text may align more closely with liberal viewpoints, issues of healthcare education and workforce development can transcend traditional political boundaries. Both liberal and conservative viewpoints might agree on the importance of a well-trained healthcare workforce, even if they differ on the methods to achieve this goal.	liberal
AAP	Poverty and Child Health in the United States	4/1/2016	The text you've provided appears to align more with a liberal viewpoint. It emphasizes the importance of government intervention and social support programs to mitigate child poverty and its effects. The text advocates for public policies that support vulnerable children and families, suggesting a belief in the positive role of government in addressing social issues. This perspective is generally associated with liberal ideology, which often prioritizes social welfare, government-funded programs, and interventions to address inequalities and improve social outcomes. In contrast, conservative viewpoints typically emphasize limited government intervention, personal responsibility, and private sector solutions to social problems.	liberal
AAP	Prevention and Management of Procedural Pain in the Neonate: An Update	2/1/2016	The text provided doesn't neatly align with a typical liberal or conservative viewpoint. It focuses on the clinical and ethical aspects of neonatal pain management in a medical context. The emphasis is on evidence-based care, ethical considerations, and improving health outcomes for neonates, especially preterm infants, in the NICU (Neonatal Intensive Care Unit).<br><br>The content is largely apolitical, addressing issues of neonatal pain assessment and management from a medical and scientific perspective. It discusses various methods for pain assessment and management, including both pharmacologic and nonpharmacologic strategies, and emphasizes the need for ongoing research and education in this area.<br><br>Political ideologies, whether liberal or conservative, typically concern themselves with broader societal, economic, and governmental policies. This text, on the other hand, is specific to pediatric healthcare practices and is more aligned with medical ethics and best practices in healthcare rather than with political ideologies.	neutral
AAP	Medical Countermeasures for Children in Public Health Emergencies, Disasters, or Terrorism	2/1/2016	The text provided does not align strictly with either a liberal or conservative viewpoint. It focuses on public health policy, specifically regarding medical countermeasures (MCMs) for children in the context of disasters and public health emergencies. The emphasis is on collaboration among various federal and health agencies, highlighting the need for preparedness in addressing children's unique health requirements during disasters.<br><br>This policy-oriented text is largely non-partisan, addressing practical and logistical issues in public health preparedness rather than advocating for a specific political ideology. It emphasizes the importance of federal agencies, such as the Department of Health and Human Services and the Centers for Disease Control and Prevention, in preparing for and responding to public health emergencies, particularly those that affect children. The focus is on the need for adequate medical countermeasures for children, recognizing their unique vulnerabilities in disaster situations.<br><br>Such discussions are common in public health policy and are generally supported across the political spectrum, though the extent and methods of government involvement in public health can be points of ideological debate. The text advocates for increased attention to and investment in public health preparedness, especially for vulnerable populations like children, which is a concern typically shared by both liberal and conservative viewpoints, albeit often approached differently.	neutral
AAP	Visual System Assessment in Infants, Children, and Young Adults by Pediatricians	1/1/2016	The text you provided is a detailed and factual exposition on the importance and methods of regular visual system assessments and eye examinations in children. It does not align specifically with either a liberal or conservative viewpoint. Instead, it focuses on medical best practices and guidelines established by professional organizations like the American Academy of Ophthalmology, the American Association for Pediatric Ophthalmology and Strabismus, and the American Association of Certified Orthoptists.<br><br>The text emphasizes the significance of early and regular eye examinations in detecting and treating various ocular and systemic conditions. Such a scientific and health-focused perspective is generally non-partisan and aimed at improving public health outcomes rather than promoting a particular political ideology.	neutral

AAP	Critical Elements for the Pediatric Perioperative Anesthesia Environment	12/1/2015	The text provided doesn't align with a traditionally liberal or conservative viewpoint. It is a technical and professional document focusing on medical standards and guidelines proposed by the American Academy of Pediatrics for pediatric perioperative anesthesia care. The content is centered on medical practices, patient safety, and healthcare quality, which are generally viewed as non-partisan, professional matters rather than subjects of political debate. The emphasis is on improving medical outcomes and ensuring the safety of infants and children in perioperative environments, addressing issues such as facility requirements, staff qualifications, and procedural guidelines. This focus on medical professionalism and patient care standards transcends political leanings.	neutral
AAP	Electronic Nicotine Delivery Systems	12/1/2015	The text you've provided presents a detailed and factual account of the risks and implications associated with the use of Electronic Nicotine Delivery Systems (ENDS), especially among youth. It does not directly align with either a liberal or conservative viewpoint in a political sense. Instead, it focuses on public health concerns, presenting data and calling for regulatory actions to protect youth from the potential harms of ENDS.<br><br>The text's emphasis is on the health risks associated with ENDS, including addiction, exposure to toxic substances, and the potential for poisoning, especially in children. It also highlights the need for regulation and legislation to address these issues.<br><br>From a political perspective, different individuals or groups might interpret or emphasize various aspects of this information according to their own viewpoints. For instance:<br><br>- A conservative viewpoint might focus on personal responsibility, parental control, and the freedom of the market, perhaps arguing against heavy regulation.<br>- A liberal viewpoint might emphasize the need for government intervention and strict regulations to protect public health, particularly the health of minors.<br><br>However, the text itself is primarily informative and health-focused, rather than politically oriented. It aims to provide a comprehensive understanding of the issues surrounding ENDS and their impact on public health, particularly among youth.	neutral
AAP	Ensuring the Health of Children in Disasters	11/1/2015	The text you've provided appears to be an academic or professional document focused on disaster preparedness, specifically addressing the unique needs of children in such scenarios. It emphasizes the role of pediatricians and healthcare professionals in preparing for, responding to, and recovering from disasters. It advocates for specialized planning and resources to ensure the safety and well-being of children during these events.<br><br>This text does not neatly align with traditional political viewpoints like liberal or conservative. Instead, it's more focused on public health and safety, particularly for children. Such topics generally transcend partisan lines, emphasizing the universal need for effective disaster management and healthcare preparedness. The document is detailed, evidence-based, and professionally oriented, suggesting it's meant for a specialized audience like healthcare providers, policymakers, and disaster response planners.	neutral
AAP	Promoting Food Security for All Children	11/1/2015	The text provided seems to be a comprehensive and detailed analysis of food insecurity in the United States, particularly focusing on how it affects children. It addresses various aspects including the demographics of food-insecure households, the effects of food insecurity on children's health and development, the relationship between poverty and food insecurity, and the various programs and interventions aimed at mitigating this issue.<br><br>This text does not inherently align with a specific political viewpoint, such as liberal or conservative. Instead, it presents factual information and research findings related to food insecurity, its impacts, and potential solutions. It discusses the role of federal programs like SNAP (Supplemental Nutrition Assistance Program) and WIC (Women, Infants, and Children), the importance of pediatricians in identifying and addressing food insecurity, and the broader societal implications of this issue.<br><br>The approach taken in the text is more aligned with a public health and welfare perspective, emphasizing the need for societal and governmental intervention to address food insecurity and its consequences. It advocates for evidence-based policies and programs to ensure adequate nutrition and health for all children, regardless of their economic background.<br><br>While different political ideologies might have varying opinions on the best ways to address food insecurity (such as the extent of government involvement or specific policies), the text itself presents a neutral, informative view focused on the issue's impacts and potential solutions.	neutral

AAP	Global Climate Change and Children's Health	11/1/2015	The text you've provided aligns more closely with a liberal viewpoint. It emphasizes the scientific consensus on human-driven climate change, highlights the vulnerability of children to climate change impacts, and advocates for substantial action to mitigate these effects. This perspective typically resonates more with liberal ideologies, which often prioritize environmental protection, acknowledge the role of human activity in climate change, and support proactive governmental and societal responses to address these issues.<br><br>Conservative viewpoints, in contrast, may vary widely on this topic. Some conservatives recognize and address the reality of climate change and its human-driven factors, advocating for market-based solutions or technological innovation to address the issue. However, other segments within conservative circles might downplay the human role in climate change, question the extent of the problem, or oppose extensive government intervention in addressing the issue, focusing instead on economic impacts and personal responsibility.<br><br>The emphasis in the text on the need for a "paradigm shift in production and consumption of energy," the need for substantial policy and societal action, and the highlighting of children's vulnerability and injustice towards them if action is not taken, are all themes commonly found in liberal discourse on climate change.	liberal
AAP	Tackling in Youth Football	11/1/2015	The text you've provided appears to be a detailed and scholarly analysis of injuries, particularly head and neck injuries, in youth football (American football). It discusses the incidence and nature of these injuries, the debate over the safety of football, the potential long-term consequences of concussions and subconcussive blows, and various strategies for reducing injuries in the sport.<br><br>This text does not align neatly with either a liberal or conservative viewpoint in a political sense. Instead, it adopts a scientific and medical perspective, focusing on empirical data and research findings. It presents a balanced view, acknowledging both the risks associated with football, particularly related to head injuries and concussions, and the benefits of the sport, such as regular exercise, social, and academic outcomes.<br><br>The discussion includes various viewpoints and studies, covering arguments for reducing contact in practices, delaying the age at which tackling is introduced, and improving safety measures like protective gear and proper tackling techniques. The text seems to aim for a comprehensive understanding of the issue, rather than advocating for a particular political or ideological stance.<br><br>In summary, the text is more scientific and medical in nature, dealing with sports medicine and injury prevention, and does not fit squarely into a liberal or conservative political framework.	neutral
AAP	Clinical Practice Policy to Protect Children From Tobacco, Nicotine, and Tobacco Smoke	11/1/2015	The text provided does not inherently align with a liberal or conservative viewpoint. Instead, it focuses on the health impacts of tobacco use and exposure, particularly in children, and outlines clinical recommendations for pediatricians. The emphasis is on the medical and health aspects of tobacco use, such as the effects of secondhand and thirdhand smoke, the risks associated with in utero exposure, and the importance of treating tobacco dependence. It addresses the role of pediatricians in screening for tobacco use, providing guidance to prevent smoking initiation, and reducing tobacco smoke exposure.<br><br>The text also discusses the importance of addressing tobacco dependence in caregivers, the need for healthcare systems to facilitate effective tobacco dependence prevention and treatment, and the inclusion of tobacco dependence prevention and treatment in medical education. These recommendations are grounded in health care and public health concerns, rather than being framed within a political ideology.<br><br>In general, public health issues like tobacco use and its effects on children can be a concern across the political spectrum, though the specific approaches to addressing these issues might vary based on political beliefs. However, this text focuses primarily on medical guidelines and health care practices rather than on political or policy debates.	neutral

AAP	Health Care Issues for Children and Adolescents in Foster Care and Kinship Care	10/1/2015	The text provided presents a comprehensive overview of the health needs, challenges, and recommended care approaches for children and adolescents in foster care. It does not align explicitly with either a liberal or conservative viewpoint. Instead, it focuses on factual information, professional guidelines, and best practices for addressing the complex medical, mental health, developmental, and educational needs of this vulnerable population.<br><br>This text appears to be from a policy or guidance document, likely produced by a professional organization such as the American Academy of Pediatrics, given its detailed discussion of health care recommendations and coordination for children in foster care. It emphasizes the importance of a pediatric medical home, coordinated health care, and the role of pediatricians in ensuring high-quality health services for these children. The document also highlights the various challenges faced by children in foster care, including trauma, disrupted attachments, and high rates of medical and psychological needs, as well as systemic issues like fragmented care and the difficulty in coordinating services among various agencies and caregivers.<br><br>The approach is pragmatic and centered on the best interests of children in foster care, drawing on research and expert consensus rather than ideological positions. It recognizes the complexity of the foster care system and the special health care needs of children within this system, advocating for comprehensive, trauma-informed, and coordinated care.	neutral
AAP	Influenza Immunization for All Health Care Personnel: Keep It Mandatory	10/1/2015	The text provided primarily aligns with a liberal viewpoint, particularly in its emphasis on public health measures and the importance of mandatory vaccinations for healthcare personnel to protect patient safety and public health. The focus on mandatory immunization policies, backed by scientific evidence and concern for community welfare, is consistent with liberal perspectives that often prioritize collective health and safety measures. The text emphasizes the ethical and just nature of mandatory influenza immunization for healthcare personnel, highlighting its necessity for improving patient safety and reducing healthcare-associated influenza infections. This approach aligns with liberal values that often support strong public health policies, even if they require mandating certain behaviors for the greater good.<br><br>Conservative viewpoints, on the other hand, often emphasize individual rights and personal choice, including in healthcare decisions. While conservatives may also recognize the importance of vaccinations, they are more likely to support voluntary vaccination programs and may have concerns about mandatory policies infringing on individual liberties or autonomy.<br><br>It's important to note, however, that public health is a complex issue that can transcend simple political categorizations, and individuals with conservative viewpoints may also support certain public health measures, including vaccinations, under specific circumstances.	liberal
AAP	The Apgar Score	10/1/2015	The text you provided is a detailed and factual description of the Apgar score, its purpose, limitations, and applications in neonatal care. It does not align with either a liberal or conservative viewpoint as it is a scientific and medical document. It focuses on providing accurate, evidence-based information about the Apgar score, a tool used to quickly assess the health of newborns immediately after birth. The text emphasizes the importance of understanding the limitations of the Apgar score, such as its inability to predict individual neonatal mortality or neurologic outcomes, and the inappropriateness of using it to diagnose asphyxia.<br><br>This type of text is characteristic of medical literature, which aims to inform and guide healthcare professionals based on current research and best practices. It does not engage in political or ideological debates, but rather focuses on clinical facts and guidelines to improve patient care. The information is presented objectively, without any apparent bias or inclination towards a political ideology.	neutral
AAP	Recommendations for Prevention and Control of Influenza in Children, 2015–2016	10/1/2015	The text provided is primarily a technical and medical guideline regarding the vaccination and treatment of influenza in children, and it doesn't inherently align with either a liberal or conservative viewpoint. It focuses on recommendations from the American Academy of Pediatrics (AAP) for the routine use of influenza vaccines and antiviral medications in children for the 2015–2016 season. The document includes detailed information about the different strains of influenza vaccines, dosing recommendations, the importance of vaccination for various at-risk groups, and strategies for vaccination and treatment.<br><br>Health policies like vaccination recommendations are often based on scientific research and public health objectives rather than political ideologies. While vaccination policies can sometimes become politicized, the content of this document itself is neutral and focuses on medical and public health guidance rather than promoting a specific political stance.	neutral

AAP	The Use of Telemedicine to Address Access and Physician Workforce Shortages	7/1/2015	The text provided does not explicitly align with a traditional liberal or conservative viewpoint. Instead, it focuses on the practical and policy aspects of telemedicine in pediatric care. The text discusses how telemedicine can improve access to care, enhance the quality of healthcare, address physician workforce shortages, and potentially reduce healthcare costs. It advocates for the integration of telemedicine into pediatric healthcare systems and discusses various aspects such as patient access, physician capacity, quality and efficiencies of care, costs, graduate and continuing medical education, payment and funding, and the support of the patient-centered medical home (PCMH) model.<br><br>This approach is primarily pragmatic and is centered around improving healthcare delivery and outcomes. It does not delve into political or ideological debates that typically distinguish liberal and conservative viewpoints. Instead, it focuses on healthcare innovation, efficiency, and effectiveness, themes that can find support across the political spectrum.	neutral
AAP	Hospital Stay for Healthy Term Newborn Infants	5/1/2015	The text you provided focuses on policy recommendations regarding the hospital stay length for a mother and her newborn. It emphasizes individualized care, considering factors like the health of the mother and infant, the mother's ability to care for the newborn, and the support system at home. It advocates for decision-making that involves input from the mother and her healthcare providers, ensuring readiness for discharge based on a variety of health and social criteria.<br><br>This text does not align strictly with either a liberal or conservative viewpoint in the traditional political sense. Its focus is on medical and health policy, emphasizing individualized care and patient-centered decision-making. Such approaches are generally supported across the political spectrum, although the extent to which they emphasize individual choice, government regulation, or the role of insurance providers might be interpreted differently by different political ideologies.<br><br>For instance, a conservative viewpoint might emphasize the importance of individual choice and minimizing government intervention in healthcare decisions. In contrast, a liberal viewpoint might focus on ensuring access to comprehensive postnatal care for all, regardless of socio-economic status, and might support regulations ensuring a minimum length of hospital stay.<br><br>The recommendations in your text appear to balance these perspectives by advocating for personalized care based on medical and social needs while also acknowledging the role of legislation and institutional guidelines in ensuring quality care. This suggests a more centrist or pragmatic approach to healthcare policy.	neutral
AAP	Point-of-Care Ultrasonography by Pediatric Emergency Medicine Physicians	4/1/2015	The text you provided does not align specifically with either a liberal or conservative viewpoint. It is a professional and clinical policy statement focused on the use of point-of-care ultrasonography, particularly in pediatric emergency medicine (PEM). The text discusses the importance of adequate training for PEM physicians in point-of-care ultrasonography, the need for credentialing and quality assurance programs, and the development of standards for training and competency assessment.<br><br>This kind of document is typically neutral and non-partisan, aiming to provide guidelines and standards for medical practice rather than advocating for a specific political ideology. It focuses on improving patient care, enhancing diagnostic accuracy, reducing unnecessary radiation exposure, and ensuring that medical professionals are properly trained and credentialed in the use of ultrasonography in emergency settings. Such objectives are generally considered important across the political spectrum and are more about medical best practices than political ideology.	neutral

AAP	Adolescent Drug Testing Policies in Schools	4/1/2015	The text provided appears to be a balanced and nuanced examination of the issue of school-based drug testing for substance abuse, presenting both sides of the argument without clearly aligning with either a liberal or conservative viewpoint. Here's a breakdown of perspectives from both sides:<br><br>1. **Conservative Viewpoint Indicators**:<br>- **Support for Drug Testing**: The text acknowledges the potential benefits of school-based drug testing. It cites a study by DuPont et al. which found lower rates of drug use among students subjected to such testing. This aligns with a more conservative viewpoint that often emphasizes law and order and the use of preventive measures to curb drug use.<br><br>2. **Liberal Viewpoint Indicators**:<br>- **Concerns About Effectiveness and Privacy**: The text raises concerns about the effectiveness of these programs and the potential breach of privacy, which are more aligned with liberal viewpoints that often prioritize individual rights and question the efficacy of punitive or invasive measures.<br>- **Emphasis on Alternative Methods**: The suggestion of using school resources for counseling and treatment, as well as the American Academy of Pediatrics' opposition to widespread drug testing due to lack of solid evidence, aligns more with liberal perspectives that often focus on rehabilitation and prevention over punitive measures.<br><br>3. **Neutral and Balanced Presentation**:<br>- The text presents both the potential benefits and drawbacks of school-based drug testing.<br>- It cites scientific studies and recommendations from the American Academy of Pediatrics, indicating a reliance on expert opinion and research rather than ideological positions.<br>- The focus is on the effectiveness and impact of these programs rather than ideological arguments.<br><br>In conclusion, the text seems to strive for a balanced presentation, highlighting both the potential benefits of school-based drug testing (a viewpoint more commonly associated with conservative perspectives) and the concerns about its effectiveness and potential for privacy breaches (aligning more with liberal perspectives). The emphasis on evidence and expert recommendations indicates an approach that prioritizes a comprehensive understanding of the issue over a strictly ideological stance.	neutral
AAP	The Impact of Marijuana Policies on Youth: Clinical, Research, and Legal Update	3/1/2015	The text from the American Academy of Pediatrics (AAP) policy statement on marijuana legalization aligns more closely with a liberal viewpoint, though it's important to note that the focus is primarily on public health rather than political ideology. The statement emphasizes:<br><br>1. **Opposition to Marijuana Use in Children and Adolescents:** The AAP stands against the use of marijuana by individuals under the age of 21 due to its negative health and brain development effects. This is consistent with a more liberal perspective on prioritizing public health concerns, especially for vulnerable populations like children and adolescents.<br><br>2. **Support for Medical Research and Re-classification:** The AAP advocates for the re-classification of marijuana from a Schedule I to a Schedule II drug to facilitate medical research. This position aligns with a more progressive stance on drug policy, emphasizing the need for evidence-based approaches and acknowledging the potential medical benefits of marijuana.<br><br>3. **Support for Decriminalization:** The AAP strongly supports the decriminalization of marijuana use for both minors and young adults, focusing on treatment rather than criminal penalties. This approach aligns with a liberal viewpoint that prioritizes public health and social justice over punitive measures.<br><br>4. **Emphasis on Regulation and Public Health Campaigns:** In states where marijuana is legal, the AAP recommends strict regulations similar to those for tobacco and alcohol, and supports public health campaigns to communicate the risks associated with marijuana use. This perspective aligns with a liberal approach that seeks to balance legalization with public health and safety concerns.<br><br>5. **Acknowledgment of Social Justice Issues:** The policy recognizes the disproportionate impact of marijuana criminalization on minority youth and advocates for policies that address these disparities. This awareness and advocacy for social justice is typically associated with a more liberal viewpoint.<br><br>Overall, while the AAP's statement is rooted in medical and public health expertise, its emphasis on research, regulation, decriminalization, and social justice issues generally aligns more with liberal viewpoints on drug policy and public health.	liberal

AAP	Snacks, Sweetened Beverages, Added Sugars, and Schools	3/1/2015	The text provided appears to present a balanced, evidence-based perspective on school nutrition and childhood obesity, incorporating elements that could align with both liberal and conservative viewpoints. It emphasizes the importance of nutritious school meals and the role of various food sources in schools, including federal meal programs, competitive items like vending machines, and informal sources like packed meals and fundraisers. The text also acknowledges the efforts made by federal, state, and local governments, as well as industry, in improving school nutrition.<br><br>Key points that might align with a liberal viewpoint include the emphasis on federal and state regulation and policies to improve nutrition in schools, and the acknowledgment of socioeconomic factors affecting nutrition, like food insecurity and economic downturns. Liberals typically support government intervention and regulation to address public health issues and to ensure equal access to resources like nutritious foods.<br><br>On the other hand, aspects of the text could also resonate with conservative viewpoints. The mention of industry's role in developing alternative products and the idea of gradual improvement in nutrition rather than outright prohibition of certain foods align with conservative preferences for market solutions and gradual, less intrusive government policies.<br><br>Overall, the text seems to prioritize evidence-based, practical approaches to improving nutrition in schools, focusing on a balanced diet and the importance of educating students and parents about healthy food choices. This balanced approach suggests an effort to address a public health issue in a way that could find support across the political spectrum.	neutral
AAP	Race, Ethnicity, and Socioeconomic Status in Research on Child Health	1/1/2015	The text you provided is academically neutral and does not align strictly with either a liberal or conservative viewpoint. It presents a comprehensive, research-based analysis of child health disparities influenced by race, ethnicity, and socioeconomic status (SES). This text reflects an evidence-based approach common in public health and medical research, focusing on documenting disparities, understanding their causes, and suggesting areas for further research and policy development.<br><br>The content emphasizes the complex interplay of biological, social, and environmental factors in determining health outcomes and disparities among children. It acknowledges the importance of considering both biological and social mechanisms when studying health disparities. The text also recognizes the importance of accurate measurement and analysis of race, ethnicity, and SES in research, highlighting the challenges and nuances involved in these processes.<br><br>While the focus on social determinants of health and the call for action to address health disparities might resonate more with liberal perspectives, which often emphasize social equity and systemic factors in health, the text itself remains grounded in scientific research and data, presenting a balanced view that does not explicitly advocate for a particular political ideology. It aims to inform and guide researchers, policymakers, and healthcare providers in understanding and addressing health disparities in a comprehensive and evidence-based manner.	probably liberal
AAP	Maintaining and Improving the Oral Health of Young Children	12/1/2014	The text provided primarily focuses on oral health, specifically dental caries, in children and the role of pediatricians in preventing and managing this condition. It doesn't explicitly align with either a liberal or conservative viewpoint as it is a scientific and medical discourse rather than a political one. The text covers the epidemiology of dental caries, risk factors, preventive strategies, and the importance of pediatricians in oral health care. It emphasizes evidence-based medical practices and public health perspectives, which are generally non-partisan and focused on the overall well-being of children regardless of political ideology.	neutral
AAP	Immunization for Streptococcus pneumoniae Infections in High-Risk Children	12/1/2014	The text provided primarily focuses on medical and public health recommendations regarding pneumococcal vaccines (PCV7, PCV13, and PPSV23) and their efficacy in preventing invasive pneumococcal disease (IPD) in various age groups, particularly in children with specific health conditions. This content is scientific and factual in nature, detailing the use, effectiveness, and recommendations for pneumococcal vaccines.<br><br>This type of information is generally not aligned with a specific political viewpoint, whether liberal or conservative. It is rooted in scientific research and public health policy, aimed at preventing disease and promoting health in the population. The recommendations mentioned in the text are based on evidence and studies conducted in the field of infectious diseases and are made by authoritative health bodies like the Advisory Committee on Immunization Practices of the Centers for Disease Control and Prevention and the American Academy of Pediatrics.<br><br>Public health recommendations, particularly those related to vaccination, can sometimes become politicized or controversial in public discourse. However, the content of this text itself is neutral and focuses on medical facts and guidelines rather than political opinions or ideologies.	neutral

AAP	Recommendations for Prevention and Control of Influenza in Children, 2014–2015	11/1/2014	The text you've provided is a detailed, factual summary of influenza vaccination recommendations for children, provided by the American Academy of Pediatrics for the 2014–2015 influenza season. It discusses vaccine compositions, recommendations for different age groups and health conditions, and guidelines for the use of antiviral medications in conjunction with vaccines.<br><br>This text primarily presents medical guidelines and recommendations based on scientific research and public health policy. It is neutral and does not explicitly align with a liberal or conservative viewpoint. Public health guidelines like these are generally based on scientific evidence and are aimed at promoting the health and well-being of the population, regardless of political affiliation.<br><br>It's important to note that while public health policies can sometimes become politicized, the underlying scientific recommendations and medical guidelines are typically based on evidence and research, not political ideology.	neutral
AAP	Contraception for Adolescents	10/1/2014	The text presented primarily aligns with a liberal viewpoint. It emphasizes the importance of contraception in reducing adolescent pregnancy rates and advocates for pediatricians to have a working knowledge of various contraceptive methods. The focus on educating adolescents about contraception, including the support for minor consent and confidentiality in sexual health care, generally reflects liberal values that prioritize individual autonomy, access to comprehensive sexual health education, and reproductive rights.<br><br>Conservative viewpoints, on the other hand, might emphasize abstinence education over contraceptive use, focus more on parental involvement in adolescents' health care decisions, and may have reservations about the broad access to contraception for minors without parental consent.<br><br>However, it's important to note that views on contraception can vary within both liberal and conservative circles, and the distinction is not always clear-cut. The text reflects a public health perspective that prioritizes reducing unintended adolescent pregnancies and promoting sexual health through informed choices and access to contraception.	liberal
AAP	Equipment for Ground Ambulances	9/1/2014	The text you provided does not align with a specifically liberal or conservative viewpoint. It is a factual and technical description of the standards and practices for equipment on ambulances and emergency medical services (EMS). This document outlines the evolution of these standards over the years, the involvement of various professional organizations in their development, and the specifics of the equipment required for different levels of EMS.<br><br>Such texts are typically neutral and are focused on ensuring the safety and effectiveness of medical services rather than promoting a political viewpoint. The emphasis is on the practical aspects of medical care in emergency situations, including the types of equipment used, the definitions of various age groups for treatment, and the guidelines for out-of-hospital care. This is more about public health and safety standards than about political ideology.	neutral
AAP	School Start Times for Adolescents	9/1/2014	The text provided does not clearly align with either a liberal or conservative viewpoint. It is a factual, evidence-based exposition on the importance of sufficient sleep for adolescents, particularly in relation to school start times. The focus is on public health, safety, and academic success, emphasizing the biological and social factors contributing to sleep deprivation in teenagers. The recommendations and findings are rooted in scientific research and medical expertise, presented by the American Academy of Pediatrics, and do not inherently favor a specific political ideology. The text is more aligned with medical and educational concerns, advocating for policy changes based on health research rather than political leanings.	neutral
AAP	The Pediatrician's Role in the Evaluation and Preparation of Pediatric Patients Undergoing Anesthesia	9/1/2014	The text provided does not inherently align with either a liberal or conservative viewpoint. It is a medical and procedural document focused on the role of pediatricians in preparing patients and families for anesthesia and surgery. The content is centered around clinical practices, medical evaluations, and the importance of communication among healthcare providers to ensure safe and effective perioperative care. It discusses various aspects of preoperative preparation, including assessing the patient's medical condition, educating families about the surgery process, and addressing special medical considerations like cardiac disease, respiratory disorders, and diabetes mellitus.<br><br>This type of document is typically non-partisan and is aimed at providing information and guidelines to medical professionals for the best possible patient care. It does not delve into political or ideological debates but rather focuses on medical protocols and best practices.	neutral

AAP	Standards for Pediatric Cancer Centers	8/1/2014	The text you've provided does not explicitly align with either a liberal or conservative viewpoint. It is a technical and medical document focused on the standards and guidelines for pediatric cancer centers. This text emphasizes the importance of specialized medical personnel and facilities in the treatment of pediatric cancer, advocating for high standards of care and the involvement of multidisciplinary teams.<br><br>The content is centered around medical best practices and standards, rather than political or ideological perspectives. It discusses the essential components of effective pediatric cancer treatment, such as board-certified pediatric hematologists/oncologists, specialized nursing staff, advanced diagnostic and treatment facilities, and a comprehensive, multidisciplinary approach to patient care.<br><br>Such guidelines and recommendations are typically based on scientific research and clinical evidence rather than political ideology. Their primary goal is to improve patient outcomes and provide the best possible care for children, adolescents, and young adults with cancer, irrespective of any political considerations.	neutral
AAP	Literacy Promotion: An Essential Component of Primary Care Pediatric Practice	8/1/2014	The text provided doesn't align specifically with either a liberal or conservative viewpoint as traditionally defined in political terms. Instead, it focuses on early childhood development, literacy, and health policy. The emphasis is on the importance of reading to young children for their cognitive and emotional development and the role pediatricians can play in promoting this practice. This perspective is rooted in scientific research and public health policy rather than political ideology.<br><br>The text discusses the benefits of early literacy and the disparities in language and literacy skills among children from different socioeconomic backgrounds. It also outlines strategies for pediatricians to encourage reading in families and advocates for policy support in this area. The viewpoint expressed in the text is consistent with contemporary understandings of child development and education, emphasizing equal opportunity and access to resources for all children, which can be seen as aligning with broader social equity concerns. However, it doesn't explicitly advocate for a particular political stance but rather focuses on health and educational outcomes for children.	neutral
AAP	Updated Recommendations on the Use of Meningococcal Vaccines	8/1/2014	The text you provided is an informational piece focusing on the medical and scientific aspects of meningococcal vaccines, including various types of vaccines, their intended age groups, and specific health conditions that necessitate different vaccination strategies. It is largely technical and clinical in nature, detailing the recommendations for vaccination against meningococcal disease, a serious bacterial infection.<br><br>This text does not align clearly with either a liberal or conservative viewpoint as it is a scientific and medical document. It focuses on public health recommendations, vaccine types, and vaccination schedules without delving into political or ideological discussions. Public health guidelines and vaccine recommendations like these are typically based on scientific evidence and epidemiological data, and they aim to provide guidance for healthcare providers and policy makers for the prevention of diseases.<br><br>In general, discussions about vaccines can sometimes become politicized, but the information in this text is strictly medical and does not advocate for a specific political stance. It simply provides updated information on meningococcal vaccines and their usage based on the latest medical and scientific research.	neutral
AAP	Updated Guidance for Palivizumab Prophylaxis Among Infants and Young Children at Increased Risk of Hospitalization for Respiratory Syncytial Virus Infection	8/1/2014	The text you've provided is a detailed and evidence-based medical policy statement about the use of palivizumab, a drug for the prevention of serious respiratory infections in certain children at high risk. This type of statement is primarily focused on medical science and clinical evidence, and it is not inherently aligned with either liberal or conservative political viewpoints.<br><br>The policy is centered on providing the best possible care based on current scientific understanding and is an example of evidence-based medicine. It discusses indications for the drug's use, considerations based on different medical conditions (such as preterm birth, chronic lung disease, congenital heart disease, etc.), and updates to previous guidelines based on new research and data.<br><br>In general, medical guidelines like this are developed by experts in the field and are intended to inform healthcare professionals about the best practices for patient care. They are typically based on a thorough review of scientific literature and are revised as new evidence becomes available.<br><br>It's important to note that while healthcare policies can sometimes become topics of political debate, the text you've provided is strictly medical in nature and does not advocate for any particular political ideology or policy. It's an example of how medical recommendations are made based on current evidence and understanding of a particular health issue.	neutral

AAP	Death of a Child in the Emergency Department	7/1/2014	The text provided does not inherently align with a liberal or conservative viewpoint. It is a professional and medical policy statement focused on the care of children, families, and staff in the event of a child's death in an emergency department. This document, issued jointly by the American Academy of Pediatrics, American College of Emergency Physicians, and Emergency Nurses Association, primarily deals with clinical practices, ethical considerations, and support mechanisms in a highly sensitive and challenging medical situation.<br><br>The statement emphasizes patient-centered, family-focused, and team-oriented approaches, compassionate support respecting cultural diversity, effective palliative care, coordination with primary care providers, and detailed protocols for handling such situations. It also highlights the importance of education and training for emergency medicine personnel in these matters.<br><br>Such guidelines are typically developed based on clinical evidence and best practices to improve care quality and are not typically associated with political ideologies. They aim to address the practical, emotional, and ethical complexities of a tragic situation like the death of a child in a medical setting, focusing on the well-being of the patients, their families, and the healthcare professionals involved.	neutral
AAP	Screening for Nonviral Sexually Transmitted Infections in Adolescents and Young Adults	7/1/2014	The text you provided is primarily informative and factual, presenting data and recommendations regarding nonviral sexually transmitted infection (STI) screening in adolescents. It does not align explicitly with either a liberal or conservative viewpoint. The focus is on public health policy and medical practice, emphasizing the importance of early detection and treatment of STIs to prevent health complications and further transmission. The guidelines and recommendations cited are from reputable health organizations like the US Preventive Services Task Force and the Centers for Disease Control and Prevention, reflecting a scientific and medical approach to STI management and prevention in adolescents.	neutral
AAP	Reducing Injury Risk From Body Checking in Boys’ Youth Ice Hockey	6/1/2014	The text you provided does not align distinctly with either a liberal or conservative viewpoint. It is a factual, evidence-based discussion focused on the risks associated with body checking in youth ice hockey, particularly for boys. The emphasis is on injury prevention and safety recommendations based on research and statistics, rather than on political or ideological arguments. The text primarily concerns public health and sports medicine, areas that typically transcend traditional political divides.	neutral
AAP	Iodine Deficiency, Pollutant Chemicals, and the Thyroid: New Information on an Old Problem	6/1/2014	The text you provided is predominantly factual and focuses on the topic of iodine deficiency, particularly in pregnant and lactating women, and the related health implications. It discusses the necessity of iodine intake for proper thyroid function and brain development in children, the prevalence of iodine deficiency in the United States, the role of environmental pollutants like nitrate, thiocyanate, and perchlorate, and recommendations from health organizations regarding iodine supplementation.<br><br>This text doesn't explicitly align with a traditional liberal or conservative viewpoint. Instead, it presents scientific and health-related information with an emphasis on public health concerns and regulatory recommendations. The discussion on government regulation, public health policy, and the role of agencies like the FDA and EPA is more aligned with policy-making and public health administration rather than partisan politics.<br><br>Issues related to public health, environmental pollutants, and government regulation can sometimes be interpreted through political lenses, with different emphasis placed on regulatory approaches or individual freedom. However, the primary aim of this text appears to be to inform and recommend best practices for health, not to advocate for a specific political ideology.	neutral

AAP	Pediatric Care Recommendations for Freestanding Urgent Care Facilities	5/1/2014	The text provided is a comprehensive and detailed policy statement regarding the treatment of children at freestanding urgent care facilities. It does not explicitly align with a liberal or conservative viewpoint. Instead, it focuses on medical and operational aspects of pediatric care in urgent care settings.<br><br>The key points of this statement are:<br><br>1. **Growth and Scope of Pediatric Urgent Care:** It notes the increasing number of urgent care facilities and their varied roles, emphasizing the importance of these facilities in providing acute care to children outside of traditional hospital-based emergency departments.<br><br>2. **Standards and Regulations:** The text stresses the importance of maintaining high standards of care, including proper training for staff, appropriate scope of care, and adherence to regulations. It highlights the need for urgent care facilities to be well-integrated into the wider medical community and to work in conjunction with other healthcare providers.<br><br>3. **Emergency Preparedness and Scope of Care:** It recommends that urgent care facilities should be prepared for emergencies, including having the right equipment and training for staff. The scope of care should be clearly defined and communicated to the public.<br><br>4. **Role of the Medical Home:** The statement emphasizes the importance of urgent care facilities supporting, but not replacing, the medical home model. It advocates for collaboration with primary care providers.<br><br>5. **Staffing and Education:** There's a focus on the need for well-trained staff and the development of educational opportunities related to pediatric urgent care.<br><br>6. **Participation in Systems of Care and Quality Assessment:** The statement encourages urgent care facilities to be part of broader healthcare systems and to have plans for disaster preparedness. It also calls for regular reviews and assessments to ensure quality care.<br><br>7. **Research and Accreditation:** It points out the need for more research on pediatric urgent care and for meaningful accreditation processes.<br><br>This document appears to be more oriented towards healthcare professionals and policymakers, focusing on practical and operational guidelines rather than political ideologies. It advocates for high-quality, efficient, and integrated urgent care services for children, a viewpoint generally supported across various political perspectives.	neutral
AAP	Addendum—Adolescent Pregnancy: Current Trends and Issues	5/1/2014	The text you've provided presents a factual, research-based overview of adolescent sexuality, contraceptive use, and teen pregnancy in the United States. It focuses on statistical trends, the efficacy of contraceptive methods, and initiatives for pregnancy prevention among adolescents.<br><br>This text does not explicitly align with either a liberal or conservative viewpoint. It provides data and research findings without advocating for a specific political stance or policy. The information is presented in a neutral, informative manner, aiming to update professionals on the subject matter based on recent research and statistics.<br><br>Such an approach is characteristic of scientific and medical literature, where the emphasis is on evidence and data rather than on promoting a particular political ideology.	neutral

AAP	Promoting Education, Mentorship, and Support for Pediatric Research	5/1/2014	The text you provided does not neatly align with a traditional liberal or conservative viewpoint, as it primarily focuses on the importance of pediatric research and the role of pediatricians in advancing child health. The topics discussed, such as the need for specialized pediatric research, the broad scope of pediatric research (including basic science, translational, community-based, health services, and policy research), and the importance of research education and mentorship in pediatrics, are generally apolitical and pertain to public health and medical education.<br><br>The text emphasizes the need for evidence-based medicine, the critical evaluation of scientific literature, and the advocacy for evidence-based child health policy. These themes are universally recognized in the medical community and do not inherently align with a specific political ideology.<br><br>Furthermore, the text discusses the challenges and recommendations for pediatric research and training, including the necessity of early exposure to research, the integration of research training in medical and postgraduate education, and the support for junior faculty in pediatric research. These topics are more related to educational policy and healthcare research rather than being indicative of a political ideology.<br><br>In summary, the text is more centered around medical education, pediatric healthcare, and research, rather than being an expression of a liberal or conservative political stance.	neutral
AAP	High-Deductible Health Plans	5/1/2014	The text provided presents a balanced and comprehensive analysis of High-Deductible Health Plans (HDHPs), detailing both the benefits and drawbacks of these plans. It does not strictly align with either a liberal or conservative viewpoint but rather offers a nuanced perspective that acknowledges the complexities and trade-offs involved in health care policy.<br><br>Key points that suggest a more conservative viewpoint include:<br><br>1. **Market-Based Approach**: HDHPs represent a market-based solution to health care, which is typically more aligned with conservative values. They promote consumer responsibility and choice, encouraging individuals to make more cost-conscious health care decisions.<br><br>2. **Reduced Premiums and Employer Costs**: The emphasis on lower premium costs for both individuals and employers aligns with conservative preferences for lower business costs and individual financial responsibility.<br><br>However, the text also presents aspects that align with a more liberal viewpoint:<br><br>1. **Concerns About Access and Equity**: The discussion highlights concerns about the impact of HDHPs on access to care, especially for families with limited resources and children with special health care needs. This focus on equity and access is more aligned with liberal perspectives.<br><br>2. **Potential Negative Impact on Primary Care and Preventive Services**: The emphasis on the importance of primary care and preventive services, and the concern that HDHPs might discourage their use, aligns with liberal values that prioritize comprehensive health coverage and preventative care.<br><br>3. **Consideration of Governmental Involvement**: The suggestion that government and policy makers should explore alternative strategies for reducing health care costs reflects a liberal inclination towards governmental involvement in health care.<br><br>Overall, the text provides a thorough examination of HDHPs without overtly favoring a liberal or conservative stance. It highlights the importance of understanding the multifaceted impacts of health insurance policies on different segments of the population and calls for more research and consideration of various policy approaches.	neutral

AAP	Withholding or Termination of Resuscitation in Pediatric Out-of-Hospital Traumatic Cardiopulmonary Arrest	4/1/2014	The text provided is a detailed, multi-organizational literature review focused on the topic of out-of-hospital termination of resuscitation in pediatric patients, particularly those who are victims of traumatic cardiopulmonary arrest. This review does not align with a typical liberal or conservative viewpoint as it is a scientific and medical document. Its primary aim is to provide an evidence base for medical guidelines and practices, rather than to promote a political ideology.<br><br>The content revolves around clinical criteria, ethical considerations, and medical evidence regarding the resuscitation of pediatric patients who have suffered traumatic cardiopulmonary arrest outside of hospital settings. It discusses the complexities of making resuscitation decisions in the field, especially for children, and the impact of such decisions on survival rates, neurological outcomes, and family and healthcare provider experiences.<br><br>In summary, this text is an objective, evidence-based medical review, devoid of political or ideological bias, focused on improving medical practice and patient outcomes in a highly specific and sensitive area of healthcare.	neutral
AAP	AAP Principles Concerning Retail-Based Clinics	3/1/2014	The text provided does not align strictly with either a liberal or conservative viewpoint in the traditional political sense. It is a policy statement from the American Academy of Pediatrics (AAP) regarding retail-based clinics (RBCs) as sources of primary care for pediatric patients. The AAP's stance is based on medical and healthcare principles rather than political ideologies.<br><br>Key points in the text reflect concerns about healthcare quality, continuity, and coordination, especially for children:<br><br>1. **Opposition to Fragmented Care**: The AAP emphasizes the importance of integrated, continuous healthcare, which they feel is compromised by the use of RBCs. This concern transcends typical liberal-conservative divides and focuses on healthcare quality and patient safety.<br><br>2. **Emphasis on the Medical Home Model**: The medical home model, advocated by the AAP, stresses comprehensive, continuous, and coordinated care. This model is a healthcare approach rather than a political stance.<br><br>3. **Concerns Over Quality of Care in RBCs**: The AAP expresses concerns that RBCs, often located in retail settings, may not provide the level of specialized pediatric care necessary, especially for children with chronic conditions or special needs.<br><br>4. **Financial Aspects and Accessibility**: While the AAP acknowledges the convenience and potential cost benefits of RBCs, they prioritize the medical home model for its comprehensive approach to care, which can be seen as a stance prioritizing healthcare quality over cost-saving measures.<br><br>In summary, the text reflects a professional medical organization's viewpoint on pediatric healthcare delivery systems, focusing on quality of care, continuity, and the medical home model. It does not directly align with political ideologies but rather addresses healthcare-specific concerns and standards.	neutral
AAP	Off-Label Use of Drugs in Children	3/1/2014	The text you've provided appears to be a policy statement from a medical or pediatric organization, likely the American Academy of Pediatrics, discussing the off-label use of medications in children. It addresses the implications of the Best Pharmaceuticals for Children Act (BPCA) and the Pediatric Research Equity Act (PREA), and how these have influenced drug labeling and prescribing practices for pediatric populations.<br><br>The content of this statement is primarily factual and technical, focusing on medical practices, regulations, and legislation related to pediatric medication. It does not explicitly align with either a liberal or conservative viewpoint as it is centered on medical and regulatory issues rather than political ideologies.<br><br>Medical and healthcare policies can sometimes intersect with political discussions, particularly in areas related to healthcare access, funding, and regulatory oversight. However, the focus of this text is on ensuring safe and effective medication use in children, which is a concern that generally transcends political divisions. Its emphasis is on evidence-based practice, regulatory compliance, and the importance of ongoing research and adjustments in drug labeling and use for pediatric patients.<br><br>In summary, the statement is more aligned with medical best practices and regulatory compliance than with a specific political ideology.	neutral

AAP	Children’s Health Insurance Program (CHIP): Accomplishments, Challenges, and Policy Recommendations	3/1/2014	The text provided presents a detailed review of the Children's Health Insurance Program (CHIP) and its evolution over time, including its legislative background, funding mechanisms, effects on insurance coverage, access to care, and health status of participants. It also addresses challenges, the relationship between CHIP and the Affordable Care Act (ACA), and offers recommendations for future policy.<br><br>From a political viewpoint, this text aligns more closely with a liberal perspective. This can be inferred from its emphasis on expanding and strengthening public health insurance programs for children, particularly those from low-income families. The focus on federal assistance, block grants for states, and the need for comprehensive funding and coverage aligns with liberal principles of providing social safety nets and government intervention to ensure health care access for all, especially vulnerable populations like children.<br><br>The text's detailed discussion of the program's benefits, such as increased insurance coverage, improved access to care, and reduction in health disparities, further supports a viewpoint favoring government-sponsored healthcare programs. Additionally, the recommendations for extending funding, enhancing federal matching rates, and ensuring comprehensive coverage are indicative of a liberal approach to health policy, which typically prioritizes expanded government involvement in healthcare to ensure equitable access and coverage.	liberal
AAP	Referral to Pediatric Surgical Specialists	2/1/2014	The text provided is a detailed set of recommendations and guidelines for pediatric healthcare, particularly focusing on when and how to refer pediatric patients for specialized surgical care. This text primarily aligns with a professional, medical viewpoint rather than a distinctly liberal or conservative political viewpoint.<br><br>The content is technical and focuses on best practices in pediatric healthcare, emphasizing the importance of specialized care for children with various health conditions. It advocates for the involvement of pediatric specialists in cases such as congenital heart disease, dental care, neurologic surgery, ophthalmology, orthopedic surgery, otolaryngology (ear, nose, and throat), plastic surgery, general pediatric surgery, and urology.<br><br>The guidelines also address the logistical and practical challenges in accessing specialized pediatric care, recognizing that geographic and resource disparities can affect care. These aspects of the text might indirectly touch on broader social issues like healthcare access and equity, which can be politicized. However, the text itself remains focused on medical guidelines and does not advocate for specific political policies or positions.<br><br>In summary, the text is a professional medical document intended to guide pediatricians and healthcare providers in referring patients to specialized pediatric care, and it does not directly align with either a liberal or conservative political viewpoint.	neutral
AAP	Consumption of Raw or Unpasteurized Milk and Milk Products by Pregnant Women and Children	1/1/2014	The text you've provided seems to align more with a science-based, public health perspective rather than fitting neatly into a liberal or conservative viewpoint. It focuses on the risks associated with the consumption of raw or unpasteurized milk and milk products, citing data and studies to support its stance. The emphasis is on the health risks posed by pathogens found in raw milk and the benefits of pasteurization.<br><br>In the context of American politics, where issues often get polarized along liberal-conservative lines, this topic can be seen through different lenses:<br><br>1. **Conservative Viewpoint**: Some conservatives might view regulations restricting raw milk sales as governmental overreach, arguing in favor of personal choice and the rights of farmers to sell raw milk. They might also support the use of natural or less processed foods.<br><br>2. **Liberal Viewpoint**: Liberals might focus on the public health aspect, supporting regulations that ensure food safety. They might advocate for pasteurization as a means to protect public health, especially vulnerable populations like children, pregnant women, and immunocompromised individuals.<br><br>However, it's important to note that the issue of raw milk sales doesn't strictly fall along traditional liberal-conservative lines. People across the political spectrum can have various opinions on this matter, often influenced by their views on food safety, personal freedom, government regulation, and natural health practices. The text itself is primarily informative and scientific, without explicit advocacy for a political viewpoint.	neutral

AAP	Respiratory Support in Preterm Infants at Birth	1/1/2014	The text you've provided is a scientific and medical discussion focused on the treatment of preterm infants with respiratory distress syndrome. It discusses various approaches to respiratory support, such as the administration of surfactant, the use of continuous positive airway pressure (CPAP), and the implications of different treatment strategies on outcomes like bronchopulmonary dysplasia and mortality rates.<br><br>This text does not align with either a liberal or conservative viewpoint as understood in a political context. It is a neutral, evidence-based discussion relevant to neonatology and medical practice, concentrating on the best treatment options for preterm infants based on recent research and clinical trials. Such scientific and medical discussions are typically independent of political ideologies and are focused on medical evidence and patient outcomes.	neutral
AAP	Guiding Principles for Managed Care Arrangements for the Health Care of Newborns, Infants, Children, Adolescents, and Young Adults	11/1/2013	The text you provided appears to align more with a liberal viewpoint, particularly in the context of American politics. It emphasizes the importance of accessibility, comprehensive coverage, and quality improvement in healthcare, especially for children. The focus on managed care, access to a range of pediatric services, and a strong emphasis on government programs like Medicaid and the State Children's Health Insurance Program (CHIP) aligns with liberal values that prioritize inclusive, accessible healthcare and government intervention in ensuring healthcare access for all, especially vulnerable populations like children.<br><br>Conservative viewpoints typically emphasize less government intervention in healthcare, favoring market-based solutions and limited government regulation. The text's support for detailed government policies, regulations, and involvement in healthcare delivery contrasts with these conservative principles.<br><br>It's important to note that the distinction between liberal and conservative viewpoints can vary based on the specific context and issues at hand, and that these labels might not fully capture the nuances of the positions discussed in the text.	liberal
AAP	Children, Adolescents, and the Media	11/1/2013	The text you provided seems to align with a more liberal viewpoint, focusing on public health, child development, and the role of media in influencing these areas. It emphasizes concerns about the potential harmful effects of media on children and teenagers, while also recognizing the positive aspects of media use. The approach is characterized by advocating for responsible media consumption, promoting media literacy, and suggesting interventions involving pediatricians, parents, schools, and policymakers.<br><br>This perspective is consistent with liberal viewpoints in several ways:<br><br>1. **Focus on Public Health and Child Development**: The emphasis on the health and well-being of children and adolescents is a hallmark of liberal social policies, which often prioritize public health initiatives.<br><br>2. **Role of Media in Society**: The text's concern with the impact of media on society reflects a liberal inclination to critically analyze the role of powerful institutions, like the media, in shaping public opinion and behavior.<br><br>3. **Advocacy for Intervention and Regulation**: The recommendations for pediatricians, parents, schools, and even the government to take active roles in managing and monitoring media consumption align with a liberal preference for societal and governmental involvement in addressing social issues.<br><br>4. **Emphasis on Education and Prevention**: The focus on media literacy education and the promotion of positive media usage are in line with liberal strategies that prioritize education and preventive measures over punitive approaches.<br><br>It's important to note that the classification of viewpoints as strictly 'liberal' or 'conservative' can oversimplify complex issues. Different aspects of this text might resonate with individuals across the political spectrum, especially those concerned with child welfare and healthy development.	liberal
AAP	Pediatric Palliative Care and Hospice Care Commitments, Guidelines, and Recommendations	11/1/2013	The text you've provided is primarily factual and educational in nature, discussing the principles, policies, and practices of pediatric palliative care and hospice care (PPC-PHC). It does not explicitly align with a liberal or conservative viewpoint. Instead, it focuses on clinical guidelines, ethical considerations, and the provision of comprehensive care to children with life-threatening conditions and their families. The text outlines best practices for patient-centered care, respect for patient and family wishes, quality and equitable access to care, and the importance of research and continuous improvement in this field.<br><br>Political viewpoints, whether liberal or conservative, often come into play when discussing healthcare policies, especially regarding funding, access, and regulation. However, this text stays neutral, focusing on the medical and ethical aspects of PPC-PHC without delving into politically charged debates. It aims to inform healthcare professionals and policymakers about effective and	neutral

			compassionate care practices for seriously ill children, emphasizing the importance of interdisciplinary teams, family support, and integrated care models.	
AAP	Conflicts Between Religious or Spiritual Beliefs and Pediatric Care: Informed Refusal, Exemptions, and Public Funding	11/1/2013	The text provided presents a viewpoint that can be considered more aligned with a liberal perspective. The central themes include advocating for state intervention in cases of medical neglect, even if the parents' refusal of medical treatment is based on religious or spiritual beliefs. The emphasis is on the protection of children's health and future autonomy, suggesting that the state has a responsibility to intervene in certain situations to ensure the well-being of minors.<br><br>This perspective is typically associated with liberal ideologies that prioritize individual rights and welfare, especially of vulnerable populations like children. It also challenges the idea of religious exemptions in child abuse and neglect laws, arguing that such exemptions do not protect all children equally and may lead to harm. This stance often aligns with liberal views that advocate for the separation of religion and state affairs and emphasize the importance of evidence-based medical practices over religious beliefs in healthcare.<br><br>Furthermore, the text argues against public health care funds being used for alternative, unproven religious or spiritual healing practices. This position aligns with a liberal viewpoint that favors the allocation of public funds to scientifically proven and effective medical treatments rather than unverified alternative methods.<br><br>It's important to note, however, that the distinction between liberal and conservative viewpoints is not always clear-cut, and there can be overlaps and variations within these broad categories. Additionally, individual perspectives may vary, and not all liberals or conservatives may agree with every aspect of the views expressed in this text.	liberal
AAP	Guiding Principles for Pediatric Hospital Medicine Programs	10/1/2013	The text provided does not align strictly with either a liberal or conservative viewpoint. It is a professional and clinical document that focuses on the roles, responsibilities, and practices of pediatric hospital medicine programs. The document discusses various aspects of pediatric hospital medicine, such as program setup, leadership, transitions of care, advocacy, and recommendations for pediatric hospital medicine programs.<br><br>This kind of text is typical in medical and academic literature, aiming to provide information and guidelines based on research and professional consensus rather than promoting a political ideology. It is centered on improving patient care and the functioning of pediatric hospital medicine within the healthcare system, rather than engaging in political discourse.	neutral

AAP	Enhancing Pediatric Workforce Diversity and Providing Culturally Effective Pediatric Care: Implications for Practice, Education, and Policy Making	10/1/2013	The text you provided presents a policy statement from the American Academy of Pediatrics (AAP) that focuses on culturally effective health care (CEHC) and workforce diversity in the context of pediatric care. It emphasizes the importance of understanding and respecting cultural differences in healthcare delivery, addressing health disparities, and promoting diversity within the healthcare workforce.<br><br>This policy statement aligns more closely with a liberal viewpoint, based on several key aspects:<br><br>1. **Emphasis on Diversity and Inclusion**: The statement's strong focus on cultural diversity, inclusion, and addressing the specific needs of various cultural groups is a hallmark of liberal perspectives, which often prioritize social equality and inclusiveness.<br><br>2. **Affirmative Action and Workforce Diversity**: The support for affirmative action and efforts to increase diversity in the healthcare workforce align with liberal policies, which often advocate for measures to address historical inequalities and increase representation of underrepresented groups.<br><br>3. **Health Disparities and Social Determinants of Health**: The acknowledgement of health disparities and the need to address them, including the consideration of social determinants of health, is consistent with liberal approaches that emphasize holistic and equitable healthcare.<br><br>4. **Educational Interventions and Advocacy**: The call for educational interventions to ensure culturally effective care and the focus on advocacy for policy changes are in line with liberal viewpoints, which often emphasize education and policy reform as tools for social change.<br><br>It's important to note that while these points align more with a liberal perspective, the principles of cultural effectiveness and diversity in healthcare can be supported across a broad political spectrum due to their overarching goal of improving health outcomes for all children.	liberal
AAP	Recommendations for Prevention and Control of Influenza in Children, 2013–2014	10/1/2013	The text provided does not directly align with either a liberal or conservative viewpoint. It is a detailed, factual statement from a medical perspective, outlining recommendations for the routine use of seasonal influenza vaccine and antiviral medications in children. The focus is on public health and preventive medicine, particularly regarding the influenza season of 2013–2014.<br><br>This statement discusses the types of influenza vaccines available, including trivalent and quadrivalent vaccines, and the specific virus strains they contain. It also covers vaccine recommendations for various groups, such as children with certain medical conditions, pregnant women, and healthcare personnel. Additionally, it addresses the use of antiviral medications in the treatment and prevention of influenza.<br><br>The information is presented in a neutral, scientific tone, emphasizing the importance of vaccination and antiviral treatment for reducing the morbidity and mortality associated with influenza. It does not advocate for or against any political ideologies or policies but focuses on providing healthcare guidelines based on medical research and evidence.	neutral
AAP	Pediatrician Workforce Policy Statement	8/1/2013	The text you provided appears to be a policy statement from the American Academy of Pediatrics (AAP). It focuses on trends and factors affecting the pediatrician workforce and the provision of pediatric health care in the United States. This includes discussions on shortages in pediatric medical and surgical specialists, the distribution of primary care pediatricians, and the impact of these issues on the quality of pediatric care, especially in rural and underserved areas.<br><br>This statement doesn't neatly align with traditional liberal or conservative political viewpoints as it primarily addresses healthcare workforce issues from a professional and technical perspective. It emphasizes the need for adequate numbers of trained pediatricians and specialists, discusses the impact of health care reforms, and calls for policies to address workforce shortages and distribution issues. The emphasis on ensuring access to comprehensive, patient- and family-centered care for all children, addressing disparities in healthcare, and advocating for equitable payment systems could resonate more with liberal viewpoints, which often prioritize healthcare accessibility and equity. However, the statement's focus on practical and professional aspects of pediatric healthcare workforce development is generally non-partisan.	neutral

AAP	Essential Contractual Language for Medical Necessity in Children	8/1/2013	The text you provided discusses the American Academy of Pediatrics' policy statement on "Model Contractual Language for Medical Necessity in Children" and related issues in health care, particularly in the context of pediatric care. It emphasizes the unique health care needs of children, the concept of medical necessity, the impact of the Patient Protection and Affordable Care Act (ACA), and the role of pediatricians in influencing health care models and contracts.<br><br>This text does not align explicitly with either a liberal or conservative viewpoint in the traditional political sense. Instead, it focuses on advocating for pediatric health care needs and ensuring that health care plans and standards, including those influenced by the ACA, are appropriately tailored to children's needs. It stresses the importance of understanding and addressing the unique medical necessities of children, which might transcend typical partisan divides.<br><br>The discussion centers on the necessity of specialized health care standards for children, the variability of essential health benefits under the ACA, the definition of medical necessity for pediatric care, and the value of pediatric services. It also touches on the need for transparency in health care delivery and decision-making processes regarding medical necessity.<br><br>In summary, the text is more aligned with health policy and pediatric care advocacy than with a specific political ideology. Its focus is on enhancing the understanding and implementation of health care standards for children, which is a concern that generally crosses political boundaries.	neutral
AAP	Transitioning HIV-Infected Youth Into Adult Health Care	7/1/2013	The text you provided is a comprehensive and detailed medical and public health perspective on the transition of HIV-infected youth to adult health care. It doesn't neatly align with a specific political viewpoint, whether liberal or conservative, but rather focuses on the medical, social, and psychological aspects of HIV care in adolescents transitioning to adulthood.<br><br>This text emphasizes the importance of a structured and supportive transition process from pediatric to adult health care for HIV-infected youth. It discusses the challenges and needs specific to this population, including medical management, psychosocial support, and continuity of care. The approach is holistic, considering not just the medical aspects of HIV treatment but also the broader social and psychological factors that affect adherence to treatment and overall well-being.<br><br>This kind of text is typically found in medical journals or public health literature, where the primary concern is the health and welfare of patients rather than the promotion of a particular political ideology. The focus is on evidence-based practices and the recognition of the unique challenges faced by HIV-infected youth, which transcends political boundaries.<br><br>The specific inclusion of details about racial disparities in HIV infection rates, the social determinants of health, and the need for comprehensive, culturally sensitive care might resonate more with viewpoints that emphasize social justice and health equity. However, these are also recognized as important factors in public health and medical practice, regardless of political affiliation.	neutral
AAP	Office-Based Care for Lesbian, Gay, Bisexual, Transgender, and Questioning Youth	7/1/2013	The text provided appears to be a comprehensive and scientific statement focused on the health and well-being of LGBTQ youth. It aligns more closely with a liberal viewpoint, primarily due to its emphasis on understanding, acceptance, and support for LGBTQ individuals, which are typically more emphasized in liberal ideologies. The text discusses the challenges faced by LGBTQ youth, including health disparities, mental health issues, and the effects of homophobia and heterosexism. It advocates for inclusive and non-judgmental healthcare practices, the avoidance of "conversion" or "reparative" therapies, and the importance of supportive environments for these individuals.<br><br>Liberal viewpoints often prioritize the rights and well-being of marginalized groups, including LGBTQ individuals, and advocate for policies and practices that promote equality and inclusivity. This text's focus on evidence-based care, acknowledgment of the unique challenges faced by LGBTQ youth, and recommendations for supportive and affirming healthcare practices are in line with such liberal values.<br><br>On the other hand, conservative viewpoints might focus more on traditional values and might be less supportive of the normalization and integration of LGBTQ identities, especially in contexts like healthcare or education. However, it's important to note that views on LGBTQ issues can vary widely within both liberal and conservative circles, and there are conservatives who support LGBTQ rights and liberals who may have reservations.	liberal

AAP	Scope of Practice Issues in the Delivery of Pediatric Health Care	6/1/2013	The text provided is a policy statement from the American Academy of Pediatrics (AAP) on pediatric health care. It doesn't align strictly with either a liberal or conservative viewpoint as traditionally understood in the political spectrum. The focus of the statement is on professional standards, the role of pediatricians, and the scope of practice within pediatric healthcare.<br><br>The text emphasizes the importance of a team-based approach to pediatric care led by a physician, ideally a pediatrician. It acknowledges the contributions of non-physician clinicians like nurse practitioners and physician assistants, but also raises concerns about the expansion of their scope of practice without proper supervision.<br><br>In general, the document can be interpreted as advocating for maintaining high professional standards in pediatric care, ensuring the safety and quality of healthcare for children, and recognizing the unique qualifications of pediatricians. It might be seen as conservative in its emphasis on traditional roles and standards within the medical profession, but it also includes progressive elements, such as the call for team-based care and the recognition of the roles of various healthcare professionals.<br><br>This kind of policy statement is more about professional guidelines and standards within a specific field of healthcare rather than a clear cut political stance. It reflects the AAP's priorities in advocating for optimal pediatric care and ensuring patient safety, rather than aligning with a specific political ideology.	neutral
AAP	Providing Care for Children and Adolescents Facing Homelessness and Housing Insecurity	6/1/2013	The text you've provided seems to align more with a liberal viewpoint. It emphasizes social issues such as child health, housing insecurity, homelessness, and the role of healthcare providers, particularly pediatricians, in addressing these issues. The focus is on understanding and mitigating the effects of homelessness on children, advocating for social support systems, and recognizing the multifaceted nature of homelessness, including its ties to health, education, and family dynamics.<br><br>Liberal viewpoints often prioritize social welfare, healthcare access, and support for vulnerable populations, including children affected by homelessness. This perspective is evident in the emphasis on the role of pediatricians in not just treating medical issues but also understanding and addressing the social determinants of health, such as housing and nutrition.<br><br>Conservative viewpoints, on the other hand, might focus more on individual responsibility, economic factors leading to homelessness, and the role of private sectors or limited government intervention in addressing social issues. This text, however, seems to advocate for a more systemic and community-oriented approach, which is more aligned with liberal ideologies.	liberal
AAP	Providing Care for Immigrant, Migrant, and Border Children	6/1/2013	The text provided appears to align more closely with a liberal viewpoint. This determination is based on several key aspects:<br><br>1. **Emphasis on Support for Vulnerable Populations:** The policy statement prioritizes the health and well-being of immigrant children, particularly those from low-income and vulnerable backgrounds. This focus on aiding marginalized groups is often associated with liberal ideologies.<br><br>2. **Advocacy for Comprehensive Healthcare Access:** The text advocates for expanded access to high-quality healthcare, including culturally and linguistically effective care. This aligns with liberal perspectives that often emphasize the importance of inclusive and comprehensive healthcare for all.<br><br>3. **Recognition of Diversity and Inclusivity:** The statement acknowledges and respects the diversity within immigrant populations, which is a hallmark of liberal values that emphasize inclusivity and respect for different cultures and backgrounds.<br><br>4. **Focus on Social Determinants of Health:** The text discusses how factors like poverty, lack of insurance, and educational barriers impact the health of immigrant children. This holistic approach to health, considering social and economic factors, is more typical of liberal viewpoints.<br><br>5. **Recommendations for Policy Changes:** The recommendations include advocating for policy changes that support immigrant child health, such as health insurance coverage for all children in the U.S., regardless of immigration status. Such policy advocacy aligns with liberal principles that often push for systemic change to address social inequities.<br><br>Overall, while the statement focuses on health care strategies and does not explicitly align with a political ideology, its emphasis on inclusivity, support for marginalized populations, and advocacy for systemic change in healthcare policies aligns more with liberal viewpoints.	liberal

AAP	Planned Home Birth	5/1/2013	The text you've provided primarily aligns with a neutral, professional viewpoint focused on medical and health care practices rather than a specific political ideology. It emphasizes the importance of safe birthing practices, informed decision-making for women, and high standards of care for newborn infants, regardless of their birth location. This approach is consistent with the professional and evidence-based perspectives of medical organizations like the American Academy of Pediatrics and the American College of Obstetricians and Gynecologists.<br><br>The text does not explicitly advocate for or against political positions typically associated with liberal or conservative ideologies. Instead, it focuses on providing guidelines and recommendations based on medical research and best practices in perinatal and neonatal care. The emphasis on patient rights, informed choice, and accessibility to safe and professional care can resonate with values across the political spectrum, as these are fundamental principles in health care.	neutral
AAP	Medicaid Policy Statement	5/1/2013	The text provided appears to be a comprehensive, factual overview of the history, policies, and impacts of the Medicaid program in the United States, particularly in relation to children's health care. It does not explicitly align with either a liberal or conservative viewpoint but instead focuses on presenting information about Medicaid's evolution, its role in children's health care, and the implications of various policy changes, including the Affordable Care Act (ACA).<br><br>The text includes details about Medicaid's funding, eligibility criteria, and the impact of the ACA and Supreme Court decisions on the program. It also discusses the American Academy of Pediatrics' recommendations for Medicaid policies, emphasizing the need for adequate coverage, financing, and access to quality care for children.<br><br>In summary, the text seems to be oriented towards providing detailed information about Medicaid and its significance in pediatric health care rather than promoting a specific political viewpoint. It highlights the importance of Medicaid in ensuring healthcare access for a significant portion of the pediatric population in the U.S. and discusses various policy recommendations to improve the program.	neutral
AAP	Ethical Controversies in Organ Donation After Circulatory Death	5/1/2013	The text provided discusses ethical and practical considerations regarding controlled Donation After Circulatory Death (DCD), touching on topics like the "dead donor rule," waiting periods before declaring death, conflicts of interest, and the role of informed consent.<br><br>This text does not neatly align with traditional liberal or conservative viewpoints as typically understood in political contexts. Instead, it addresses complex medical ethics and policy issues, focusing on maintaining integrity in organ donation and transplantation processes. The considerations involve balancing ethical obligations to both donors and recipients, ensuring that organ donation is conducted under rigorous ethical standards, and respecting both legal and moral norms.<br><br>The discussion about DCD and organ donation crosses various ethical, medical, and societal domains. It delves into nuanced debates like the definition and criteria of death, the ethical implications of organ retrieval, and the importance of informed consent, particularly in sensitive cases involving incapacitated patients or children. These issues are typically addressed within the medical and bioethical communities, rather than being central to liberal or conservative political ideologies.<br><br>In summary, the text reflects a discourse grounded in medical ethics and policy, focusing on specific practices and principles in organ donation, rather than aligning with a particular political ideology.	neutral
AAP	Promoting the Well-Being of Children Whose Parents Are Gay or Lesbian	4/1/2013	The text you've provided represents a viewpoint that aligns more closely with liberal ideologies. It emphasizes the importance of equal rights and opportunities for all individuals, regardless of their sexual orientation. This includes support for civil marriage rights, adoption, and foster care opportunities for same-gender couples, which is a stance typically associated with liberal viewpoints.<br><br>Liberal ideologies often prioritize inclusivity, equality, and social justice, advocating for the rights of marginalized groups. The focus on ensuring that children have access to nurturing environments, irrespective of the parents' sexual orientation, aligns with these liberal principles. The text also discusses the importance of legal and social support for diverse family structures, which is another key aspect of liberal social policy.<br><br>Conservative viewpoints, on the other hand, might place a stronger emphasis on traditional family structures and might be more cautious or opposed to changing established norms, especially regarding marriage and parenting rights for same-gender couples. While there are conservatives who support LGBTQ+ rights, the general trend in conservative thought has historically been more reserved in terms of embracing these changes compared to liberal ideologies.<br><br>However, it's important to note that these are general trends and there can be a wide range of beliefs within both liberal and	liberal

			conservative circles. Not all individuals who identify as liberal or conservative will necessarily align perfectly with these general descriptions.	
AAP	Electronic Prescribing in Pediatrics: Toward Safer and More Effective Medication Management	4/1/2013	The text you've provided is a policy statement from the American Academy of Pediatrics (AAP) advocating for the adoption of electronic prescribing (e-prescribing) systems with pediatric functionality. This statement is primarily focused on improving the quality and safety of medication administration in pediatric healthcare. It does not align directly with a traditional liberal or conservative political viewpoint as it is more centered around medical best practices and patient safety.<br><br>The emphasis is on the practical aspects of healthcare, particularly the need for improved e-prescribing systems in pediatric care to reduce medication errors and improve outcomes. The AAP's position is informed by data and legislative context, seeking to align with federal statutes that encourage the use of such systems. This approach is typical of professional medical organizations, which tend to focus on evidence-based recommendations for healthcare practices rather than political ideology.<br><br>However, one could argue that the advocacy for federal incentives and the emphasis on standards and regulations might be more aligned with a liberal viewpoint, which generally supports more regulation and government involvement in healthcare. Conversely, the focus on innovation, efficiency, and the use of technology to improve healthcare outcomes could be seen as aligning with conservative principles that favor market-based solutions and technological advancement.<br><br>Overall, the statement is more about practical healthcare improvements than political ideology.	neutral
AAP	Out-of-School Suspension and Expulsion	3/1/2013	The text you provided appears to align with a more liberal viewpoint. The focus on alternatives to traditional punitive measures like out-of-school suspension and expulsion, the emphasis on understanding and addressing the broader social and emotional needs of students, and the advocacy for inclusive and equitable treatment of all students, including those with disabilities, are characteristic of liberal perspectives. These viewpoints often prioritize understanding the root causes of behavior, promoting restorative practices, and ensuring equal educational opportunities.<br><br>Conservative viewpoints, in contrast, might emphasize more traditional disciplinary approaches, maintaining order and discipline through stricter enforcement of rules, and might be less inclined to focus extensively on the broader societal and emotional factors influencing student behavior. They generally support the idea of personal responsibility and consequences for actions.<br><br>However, it's important to note that these are broad generalizations, and there can be considerable variation within each political spectrum. Education policies can attract a range of opinions that don't always align neatly with traditional political categories.	liberal

AAP	Community Pediatrics: Navigating the Intersection of Medicine, Public Health, and Social Determinants of Children's Health	3/1/2013	The text provided appears to align more with a liberal viewpoint than a conservative one. This alignment can be inferred from several key aspects:<br><br>1. **Emphasis on Social Determinants of Health**: The text places significant emphasis on the social determinants of health, which is a concept typically more emphasized in liberal viewpoints. It acknowledges the impact of economic, social, cultural, and environmental factors on children's health, suggesting a holistic approach to healthcare that goes beyond traditional clinical treatments.<br><br>2. **Advocacy and Community Involvement**: The text advocates for pediatricians to be involved in child advocacy at local, state, and federal levels, emphasizing the need for broader societal engagement to address child health issues. This advocacy for collective action and community-based approaches is more aligned with liberal ideologies, which often focus on the role of societal structures and community support in addressing individual and public health issues.<br><br>3. **Focus on Equity and Access to Healthcare**: There is a clear focus on eliminating health disparities and ensuring universal access to high-quality medical care for all children, regardless of their background. This concern for equity and universal healthcare access is typically more associated with liberal ideologies.<br><br>4. **Interdisciplinary and Collaborative Approaches**: The statement encourages pediatricians to merge traditional clinical skills with public health approaches, emphasizing interdisciplinary collaboration. This approach aligns with liberal perspectives that often advocate for integrated, collaborative solutions to societal problems.<br><br>5. **Policy and Legislative Advocacy**: The text mentions the importance of influencing policies and laws affecting children and families, reflecting a belief in the role of government and policy in ensuring the well-being of children. This belief in the positive role of government intervention is more characteristic of liberal viewpoints.<br><br>Overall, the text reflects a comprehensive, community-oriented approach to child health, emphasizing social equity, government involvement, and the broader social context of health, which are hallmarks of a liberal perspective.	liberal
AAP	Ethical and Policy Issues in Genetic Testing and Screening of Children	3/1/2013	The text you provided does not clearly align with a traditional liberal or conservative viewpoint, as it primarily focuses on the ethical, clinical, and psychosocial aspects of genetic testing and screening in children. It presents a balanced approach, emphasizing the best interest of the child, informed consent, the importance of genetic counseling, and considerations for various types of genetic tests (diagnostic, carrier, predictive, etc.). The recommendations are developed collaboratively by the American Academy of Pediatrics and the American College of Medical Genetics and Genomics, indicating a scientific and medical perspective rather than a political one.<br><br>The content is largely informative and neutral, aiming to provide guidance based on current scientific understanding and ethical considerations. It does not delve into political or ideological debates that typically characterize liberal or conservative viewpoints. Instead, it focuses on best practices and recommendations for healthcare providers, parents, and guardians in the context of genetic testing and screening in children.	neutral

AAP	Parental Leave for Residents and Pediatric Training Programs	2/1/2013	The text you've provided does not align strictly with either a liberal or conservative viewpoint as defined in the traditional political sense. Instead, it focuses on practical, equitable, and legal aspects of parental leave policies for medical residents. It emphasizes the importance of these policies in supporting the well-being of pediatric residents and their families, including developing infants, and advocates for fair and effective implementation of such policies in residency programs.<br><br>Key points include:<br><br>1. **Support for Family and Medical Leave Act (FMLA)**: Advocating for FMLA benefits to be extended to all residents and interns from the beginning of their pediatric residency training. This is a position that can be interpreted as valuing work-life balance and family well-being, which can resonate with broader social welfare principles often associated with liberal viewpoints.<br><br>2. **Gender-neutral Parental Leave**: The recommendation for a minimum of 6 to 8 weeks of paid parental leave, regardless of gender, aligns with modern principles of gender equality and family support.<br><br>3. **Flexibility and Individual Consideration**: Emphasizing flexibility in scheduling and the need for policies to be sensitive to individual circumstances, including pregnancy and adoption scenarios, reflects a person-centered approach.<br><br>4. **Professional and Educational Balance**: The text also considers the balance between meeting the educational requirements of residency programs and supporting residents' personal and family needs.<br><br>5. **Cultural Sensitivity**: The call for policies to be communicated in a culturally sensitive manner suggests an awareness of diversity and inclusivity, which are often key components of more progressive viewpoints.<br><br>Overall, while the text doesn't squarely fit into a traditional political ideology, it leans towards a compassionate, inclusive approach that considers the needs of individuals and families, which can be associated with more liberal social policies. However, it also emphasizes legal compliance and the practical aspects of implementing these policies in a professional setting, which can be appealing from a more conservative standpoint of structured, rule-based governance.	neutral
AAP	Infant Feeding and Transmission of Human Immunodeficiency Virus in the United States	2/1/2013	The text you've provided is primarily factual and medical in nature, focusing on the guidelines and recommendations for managing the risk of HIV transmission through breastfeeding, particularly in the United States. It is not aligned with a specific political viewpoint, whether liberal or conservative.<br><br>The content mainly revolves around clinical practices and public health policies based on scientific research and evidence. It discusses the risks associated with breastfeeding by HIV-infected mothers, the benefits of breastfeeding in general, and the different recommendations based on resource availability and the mother's HIV status. The recommendations from the American Academy of Pediatrics and the Centers for Disease Control and Prevention are based on medical evidence and public health considerations rather than political ideologies.<br><br>This approach to public health and medical care—basing decisions and guidelines on scientific evidence and the specific context of each case (such as the availability of resources like clean water and affordable feeding alternatives)—is a hallmark of medical professionalism and ethics, transcending political affiliations.	neutral
AAP	Role of the School Physician	1/1/2013	The text provided doesn't align clearly with either a liberal or conservative viewpoint in the traditional political sense. It focuses on the role of physicians in schools and the importance of integrating health and education for the well-being of children. This subject, by its nature, is more centered on public health and education policy rather than on partisan political ideologies.<br><br>The text emphasizes the importance of having school physicians and the various roles they can play in supporting the health and educational outcomes of children. It discusses the history of physicians in schools, the current lack of uniformity in laws regarding school physicians, the necessary knowledge base for physicians in this setting, and the roles and relationships that these physicians can have with schools.<br><br>The recommendations made by the American Academy of Pediatrics (AAP) focus on advocating for the presence of school physicians, the need for pediatricians to support school health programs, and the importance of school physicians being well-informed about legal and medical issues pertinent to their role.	neutral

			Overall, the text is more aligned with public health advocacy and education reform, areas which can be supported by individuals across the political spectrum. It does not explicitly endorse or oppose any specific political ideology or party.	
AAP	The Crucial Role of Recess in School	1/1/2013	The text provided appears to be an academic or policy-oriented discussion about the importance of recess in schools. It emphasizes the cognitive, social, emotional, and physical benefits of recess for children, advocating for its inclusion in the school day. This perspective aligns more closely with a liberal viewpoint, particularly in its focus on holistic child development, the value of unstructured play, and the importance of addressing physical and emotional needs alongside academic learning.<br><br>Liberal viewpoints often prioritize the development of the whole individual, including emotional and social aspects, and tend to support educational approaches that encompass more than just traditional academics. The emphasis on equity (noting the lesser availability of recess for children of lower socioeconomic status and in urban settings) and the importance of play for emotional and social development are characteristic of a more liberal perspective on education.<br><br>Conservative viewpoints, on the other hand, might focus more on traditional academic achievement and structure in education, potentially viewing recess as a secondary priority compared to core academic subjects. However, it's important to note that views on education can vary widely within both liberal and conservative spectrums, and there can be overlaps in certain aspects.	liberal
AAP	Clostridium difficile Infection in Infants and Children	1/1/2013	The text you provided is a factual, medical statement and doesn't align with a specific liberal or conservative viewpoint. It focuses on the medical and scientific aspects of Clostridium difficile infections in hospitalized children, discussing epidemiological trends, diagnostic methods, and treatment protocols. This type of content is typically neutral, aiming to inform and educate about a specific health issue rather than promote a particular political stance.<br><br>In general, scientific and medical discussions, especially those centered on disease treatment and prevention, aim to be based on evidence and research findings, avoiding partisan biases. Political viewpoints are more likely to be evident in discussions about healthcare policy, funding, and systemic approaches to health issues, rather than in the specific medical details of disease management.	neutral
AAP	Health Care of Youth Aging Out of Foster Care	12/1/2012	The text provided seems to align more closely with a liberal viewpoint, primarily due to its emphasis on government intervention and support for vulnerable populations, in this case, youth transitioning out of foster care. The text highlights the significant medical and mental health care needs of these youth and underscores the fact that they often do not receive necessary services due to a lack of health insurance. It also details the steps taken by the federal government, through various policies and programs, to support these individuals.<br><br>The mention of acts like the Fostering Connections to Success and Increasing Adoptions Act of 2008 and the Patient Protection and Affordable Care Act of 2010, which aim to provide health insurance and support to these youth, reflects a belief in the positive role of government in addressing social issues. This perspective is typically associated with liberal ideology, which often advocates for government action to address societal problems and supports social safety nets.<br><br>In addition, the text focuses on the role of pediatricians and the health care system in collaborating with child welfare agencies to meet the needs of these youth, which aligns with a liberal viewpoint that emphasizes comprehensive healthcare access and the responsibility of the society to care for its vulnerable members.<br><br>It's important to note that while the text aligns more with a liberal viewpoint, it does not explicitly endorse a political ideology. It focuses on specific policies and their impact on a particular group, rather than advocating for a broader ideological stance.	liberal

AAP	Pesticide Exposure in Children	12/1/2012	The text you provided aligns more closely with a liberal viewpoint, primarily because of its emphasis on public health concerns, especially regarding children, and its advocacy for more stringent regulatory actions and improved safety measures related to pesticides. It reflects a perspective that prioritizes environmental and health protections over commercial or agricultural interests often associated with pesticide use.<br><br>Key aspects of this liberal viewpoint include:<br><br>1. **Focus on Health and Safety**: The text underscores the risks of pesticides to children's health, including acute poisoning and chronic health issues like cancer, cognitive impairment, and behavioral problems. This focus on public health and safety is a hallmark of liberal perspectives, which often prioritize these concerns over economic or business interests.<br><br>2. **Advocacy for Stricter Regulations**: The text calls for more rigorous regulatory action on pesticides, including better labeling, marketing practices, and consideration of child health in policy-making. Liberals generally advocate for stronger government oversight and regulations, especially in areas impacting public health and safety.<br><br>3. **Emphasis on Research and Education**: The text advocates for ongoing research and better education in medical training about the effects of pesticides. This approach aligns with liberal values that stress the importance of science and education in policy-making.<br><br>4. **Integrated Pest Management (IPM)**: The recommendation for policies that promote IPM, which minimizes the use of chemical pesticides, reflects a more environmentally conscious approach typical of liberal viewpoints.<br><br>5. **Global Perspective**: The text suggests restricting the exportation of harmful pesticides and promoting safer alternatives internationally. This broader, global perspective on health and environmental issues is more aligned with liberal ideologies.<br><br>In contrast, a conservative viewpoint might emphasize the economic benefits of pesticides in agriculture and pest control, argue for less governmental regulation, and prioritize business interests alongside public health considerations.	liberal
AAP	Standards for Health Information Technology to Ensure Adolescent Privacy	11/1/2012	The text provided appears to be a policy statement or a report focused on the privacy and security of health information, specifically concerning minors and adolescents. It doesn't align strictly with a traditional liberal or conservative viewpoint as understood in the political context. Instead, it addresses a specific issue within the healthcare system - the need for enhanced privacy and security in electronic health records (EHRs) for adolescents.<br><br>This text discusses the challenges and requirements for protecting the privacy of minors in health information systems. It reviews state and federal laws related to minors' health care consent and the privacy of their health information. The document emphasizes the need for electronic health records and other health information technologies to have the capability to protect minors' health information, especially in areas protected by federal and state laws.<br><br>The text also includes a series of recommendations aimed at ensuring the privacy and security of health information for adolescents. These include the creation and implementation of criteria for EHR systems that meet privacy standards for adolescents, accommodate consent from minors, and protect sensitive information.<br><br>Overall, the focus is on health policy and patient privacy rights rather than a broader political ideology. This type of policy discussion can be found across the political spectrum, as it deals with the intersection of healthcare, technology, and legal rights.	neutral
AAP	Human Embryonic Stem Cell (hESC) and Human Embryo Research	11/1/2012	The text provided appears to align more closely with a liberal viewpoint, especially in the context of American politics. It supports continued research and funding for human embryonic stem cell (hESC) research, citing its potential benefits in treating pediatric diseases. This perspective is typically associated with liberal or progressive stances, which often emphasize scientific research and its potential for medical advancements.<br><br>Conservative viewpoints, particularly those influenced by religious or moral considerations, often raise ethical concerns about the use of embryonic stem cells, especially when it involves the destruction of human embryos. This ethical concern is mentioned in the text, but the overall stance of the document is in favor of continuing such research, suggesting a more liberal perspective.<br><br>It's important to note that the categorization of viewpoints as 'liberal' or 'conservative' can vary based on the specific context and	liberal

			the broader political and cultural environment. In some contexts or regions, there may be a mix of opinions on such issues that don't neatly align with traditional political labels.	
AAP	Instrument-Based Pediatric Vision Screening Policy Statement	11/1/2012	The text you provided is a policy statement describing the use of automated vision screening technology in children. It is primarily technical and medical in nature, focusing on the effectiveness, methods, and challenges of using instrument-based vision screening, such as photoscreening and autorefraction, for detecting vision problems in children.<br><br>This statement does not align explicitly with either a liberal or conservative viewpoint as it is focused on medical practice and technology rather than political ideology. It discusses the benefits of using these technologies, particularly for children who are preverbal, preliterate, or developmentally delayed. It also addresses the barriers to adoption of these technologies, such as cost and third-party payment policies.<br><br>In terms of political perspectives, different aspects of the statement could be interpreted to align with various viewpoints:<br><br>1. **Focus on Innovation and Technology (Potentially Neutral):** The emphasis on adopting new technologies for health benefits doesn't inherently align with a specific political ideology.<br><br>2. **Consideration of Cost and Third-Party Payment (Potentially Mixed):** Concerns about the cost of new technology and reliance on third-party payers could be seen in different lights. A more conservative viewpoint might focus on the cost implications for private practices and the need for market solutions, while a more liberal viewpoint might focus on the need for broader access to such technologies regardless of cost and the role of government or insurance in funding healthcare advancements.<br><br>3. **Healthcare Accessibility (Potentially Liberal):** The emphasis on making advanced healthcare accessible to all children, including those with developmental delays, could align with more liberal viewpoints that prioritize healthcare access and equity.<br><br>4. **Research and Evidence-Based Practice (Potentially Neutral):** Advocacy for ongoing research and validation of these technologies is a scientific stance that is generally neutral in terms of political ideology.<br><br>Overall, the statement is more focused on medical and technological issues rather than political ideology. Any alignment with political viewpoints would be indirect and dependent on how different aspects of healthcare policy and funding are interpreted.	neutral
AAP	Firearm-Related Injuries Affecting the Pediatric Population	11/1/2012	The text you provided aligns more closely with a liberal viewpoint. It emphasizes the need for gun control, the dangers of firearm availability, particularly in homes with children and adolescents, and supports specific measures such as the regulation of firearms, a ban on semiautomatic assault weapons, and strong regulations on handguns for civilian use. These perspectives are generally associated with liberal or progressive political stances, which often advocate for stricter gun control laws as a means to reduce gun violence and increase public safety.<br><br>In contrast, a conservative viewpoint typically emphasizes Second Amendment rights, individual liberty, and may prioritize the right to own firearms with fewer restrictions. Conservatives often argue that responsible gun ownership can contribute to personal safety and security, and may oppose stricter gun control measures as infringements on individual freedoms.<br><br>The text's focus on firearm-related injuries and deaths among children and adolescents, the economic costs of firearm-related injuries, and the advocacy for specific measures to reduce gun violence, including physician counseling and public health approaches, are indicative of a public health perspective often associated with liberal viewpoints.	liberal

AAP	Cheerleading Injuries: Epidemiology and Recommendations for Prevention	11/1/2012	The text provided doesn't align explicitly with either a liberal or conservative viewpoint. It's a factual and neutral account of the evolution of cheerleading, the epidemiology of cheerleading injuries, and recommendations for injury prevention. The text focuses on historical changes, statistical data, and safety guidelines in cheerleading, without delving into ideological or political discussions. The content is more oriented towards informing about cheerleading as a sport, its risks, and the measures to mitigate these risks, rather than advocating for a particular political stance.	neutral
AAP	Trampoline Safety in Childhood and Adolescence	10/1/2012	The text you provided appears to be a policy statement from a medical or pediatric organization, specifically the American Academy of Pediatrics (AAP), regarding the use of trampolines. This statement focuses on the safety concerns associated with trampoline use, especially in the home setting. It references various studies and safety recommendations, as well as injury rates and patterns, to support its guidance.<br><br>This text does not inherently align with a liberal or conservative viewpoint in the political sense. Instead, it's grounded in public health and safety concerns, based on scientific research and data. The emphasis is on minimizing risk and preventing injuries, particularly in children and adolescents, who are the primary users of trampolines in a recreational context.<br><br>Policy statements like this one are typically based on empirical evidence and are intended to guide best practices in health and safety. They are not designed to propagate a political ideology but rather to inform and protect the public based on current understanding and research findings in a particular field.	neutral
AAP	Recommendations for Prevention and Control of Influenza in Children, 2012–2013	10/1/2012	The text provided is a detailed and informative statement on the recommendations for influenza vaccination and antiviral medications for children, primarily focused on the 2012-2013 influenza season. This statement is scientific and medical in nature, discussing the specifics of the trivalent seasonal influenza vaccine, its constituents, and the guidelines for its administration to various groups, including children, pregnant women, healthcare personnel, and others at high risk for influenza complications.<br><br>This text does not explicitly align with a liberal or conservative viewpoint as it is based on medical and scientific data and recommendations from authoritative health organizations like the American Academy of Pediatrics and the Centers for Disease Control and Prevention. It focuses on public health and safety, emphasizing the importance of vaccination and antiviral treatments to prevent and manage influenza, particularly in children.<br><br>The approach taken in this statement is typical of public health communications, which prioritize evidence-based recommendations for the wellbeing of the population, without delving into political ideologies. The emphasis is on the importance of vaccination and treatment to reduce the risk of influenza and its complications, which is a common perspective in the field of public health and medicine.	neutral
AAP	Circumcision Policy Statement	9/1/2012	The text you've provided, primarily discussing the health benefits and risks of male circumcision, seems to be a factual, medically-oriented statement rather than one that aligns explicitly with a liberal or conservative viewpoint. It outlines the findings of a systematic evaluation conducted by the American Academy of Pediatrics and endorsed by the American College of Obstetricians and Gynecologists.<br><br>The statement focuses on the health aspects of male circumcision, including its benefits in preventing urinary tract infections, penile cancer, and the transmission of certain sexually transmitted infections, including HIV. It also mentions that while the health benefits do not warrant recommending routine circumcision for all male newborns, they are significant enough to justify access to the procedure for those families who choose it.<br><br>In terms of political alignment, health topics like male circumcision can be viewed differently across the political spectrum, but this specific text appears to be presenting medical findings without a clear lean towards either liberal or conservative viewpoints. It emphasizes informed choice, medical evidence, and the importance of parental decision-making based on a variety of factors, including medical, religious, ethical, and cultural considerations.	neutral

AAP	Levels of Neonatal Care	9/1/2012	The text provided seems to be a policy statement or a scholarly review concerning the provision of risk-appropriate care for newborn infants and mothers, focusing on the importance of a tiered system of care, consistent standards, and regionalized systems for neonatal and perinatal care. This text is primarily scientific and technical in nature, relating to healthcare policy and practice, rather than being aligned with a specific political ideology such as liberal or conservative.<br><br>It discusses the evolution of care levels for newborns, emphasizing the need for uniform definitions and standards in neonatal care. The objective is to review and update the designations for newborn care in the United States, based on the latest data and research. The background highlights the importance of neonatal intensive care and its impact on high-risk infants, while also noting the challenges and changes in the field over time.<br><br>The content is largely factual and data-driven, focused on improving health outcomes for newborns and their mothers. It does not explicitly advocate for a particular political viewpoint but rather aims to inform and guide medical practice and policy based on scientific evidence and healthcare needs.<br><br>In summary, this text is more aligned with healthcare policy and medical practice, focusing on evidence-based approaches to neonatal care, rather than fitting neatly into a liberal or conservative political viewpoint.	neutral
AAP	Sensory Integration Therapies for Children With Developmental and Behavioral Disorders	6/1/2012	The text provided doesn't explicitly align with a traditional liberal or conservative viewpoint. It is a detailed, clinical discussion about sensory-based therapies, their application in occupational therapy, and the challenges associated with diagnosing and treating sensory processing disorders, especially in children.<br><br>This kind of text is more scientific and medical in nature, focusing on the understanding, diagnosis, and treatment of sensory processing issues in the context of developmental disorders like autism spectrum disorders, ADHD, and others. It delves into the history, theory, and current practices in sensory integration therapy, discussing the evidence (or lack thereof) for its effectiveness, and offers recommendations for healthcare providers.<br><br>The content is more aligned with medical and therapeutic best practices, evidence-based medicine, and the complexities of pediatric developmental and behavioral health care. It is not centered around political or ideological beliefs, but rather on clinical research, therapy practices, and the challenges of diagnosing and treating complex developmental disorders.	neutral
AAP	Pediatric Sudden Cardiac Arrest	4/1/2012	The text you provided is a comprehensive and detailed discussion on pediatric sudden cardiac arrest (SCA), its causes, prevention strategies, and management approaches. It doesn't align specifically with either a liberal or conservative viewpoint. The focus of the text is on medical and health policy, emphasizing the importance of understanding, diagnosing, preventing, and treating pediatric SCA. It addresses a broad range of topics including the incidence of pediatric SCA, genetic factors, warning signs and symptoms, screening techniques, and recommendations for pediatric care providers.<br><br>The text is factual and evidence-based, presenting information that is relevant to healthcare professionals, policymakers, and potentially affected families. It does not delve into political or ideological arguments but rather stays focused on medical science and public health. The endorsement of the statement by prominent medical organizations like the American College of Cardiology, the American Heart Association, and the Heart Rhythm Society further underscores its medical and scientific orientation rather than a political one.	neutral
AAP	HPV Vaccine Recommendations	3/1/2012	The text you provided is a factual, medical policy statement regarding the use of human papillomavirus (HPV) vaccines. It primarily focuses on the scientific and health-related aspects of HPV vaccination, recommending routine immunization for both males and females to prevent HPV-related diseases, including various types of cancer.<br><br>This statement does not inherently align with a specific liberal or conservative viewpoint. It is a medical recommendation based on scientific research and public health considerations. In the context of political viewpoints, discussions about HPV vaccines can sometimes intersect with broader debates on public health policies, sexual education, and individual rights, which can be viewed differently by liberal and conservative perspectives.<br><br>However, the text itself, as a scientific and medical guideline, aims to be neutral and focuses on health outcomes rather than political ideology.	neutral

AAP	Baseball and Softball	3/1/2012	The text you've provided does not align strictly with either a liberal or conservative viewpoint. It is a factual, research-based statement focusing on the safety and health aspects of youth participation in baseball and softball. The text emphasizes the importance of safety measures, injury prevention, and appropriate medical guidance in these sports. It discusses various aspects such as the epidemiology of injuries, the impact of equipment, the role of pediatricians in advising young athletes, and guidelines for safe participation.<br><br>This kind of statement is generally non-partisan and focuses on public health and safety rather than political ideology. It's more aligned with medical, athletic, and child welfare concerns, which are areas typically addressed across the political spectrum. The emphasis on research, data-driven conclusions, and recommendations for safe practices reflects a scientific and practical approach to sports safety rather than a political stance.	neutral
AAP	Patient- and Family-Centered Care and the Pediatrician's Role	2/1/2012	The text you provided aligns with neither a distinctly liberal nor conservative viewpoint in the traditional political sense. It is a comprehensive and detailed statement on patient- and family-centered care in pediatrics, focusing on the principles and benefits of this approach. The text emphasizes the importance of collaboration, respect for family and cultural values, effective communication, and the active involvement of families in the health care process.<br><br>It discusses the historical development of patient- and family-centered care, outlines its core principles, and provides evidence of its effectiveness in improving health outcomes and satisfaction among patients and families. The statement also makes recommendations for implementing this approach in various healthcare settings.<br><br>This approach to healthcare is not inherently political or ideologically driven but rather focuses on improving patient care and outcomes through partnership and collaboration with families. It is more about best practices in healthcare than about political ideology.	neutral
AAP	Endorsement of Health and Human Services Recommendation for Pulse Oximetry Screening for Critical Congenital Heart Disease	1/1/2012	The text you've provided focuses on medical and health policy recommendations rather than political ideology. It discusses the incorporation of pulse oximetry in newborn screening to detect critical congenital heart disease (CCHD), a move supported by the American Academy of Pediatrics (AAP) and the Secretary of Health and Human Services (HHS). The emphasis is on improving healthcare outcomes for infants through early detection and intervention.<br><br>This text doesn't align clearly with either a liberal or conservative viewpoint in the traditional political sense. Health and medical recommendations, such as the use of pulse oximetry for newborns, are typically based on scientific evidence and clinical expertise rather than political ideology.<br><br>The advocacy for implementing this screening in a cost-effective, practical manner and ensuring adequate funding and support may resonate with both liberal and conservative perspectives, as it encompasses elements of public health improvement (often a liberal focus) and cost-effectiveness/practical implementation (which can align with conservative viewpoints on fiscal responsibility). However, the text itself is more centered on medical best practices and public health policy than on political ideology.	neutral
AAP	Health Care for Youth in the Juvenile Justice System	12/1/2011	The text you've shared seems to present a balanced, comprehensive view of the health needs of youth in the juvenile correctional system. It doesn't explicitly align with a strictly liberal or conservative viewpoint. Instead, it focuses on empirical data and the health and welfare of a vulnerable population, which is a concern that can be shared across the political spectrum.<br><br>The text emphasizes the importance of understanding and addressing the physical, mental, and developmental health needs of youth in the juvenile correctional system. It acknowledges the disparities and challenges faced by these individuals, including higher rates of health issues compared to the general adolescent population, inconsistent healthcare access, and the need for better care continuity between community and correctional facilities.<br><br>This approach aligns with a more holistic and reformative view of the justice system, which can be supported by individuals across various political beliefs. It advocates for equal access to healthcare and emphasizes the importance of understanding the social determinants of health. The focus on evidence-based interventions, the need for advocacy in various areas (like healthcare access and continuity), and the importance of addressing systemic issues like socioeconomic status and educational needs, are elements that can be endorsed by both liberal and conservative viewpoints, albeit for different reasons.<br><br>Liberals might emphasize the social justice and equity aspects of these issues, focusing on the systemic inequalities that lead to higher rates of incarceration among certain demographics. Conservatives might focus on the need for effective rehabilitation and the importance of personal responsibility facilitated by access to proper healthcare and education. Both viewpoints can converge on the importance of addressing these needs for the betterment of individuals and society.	neutral

AAP	The Pediatrician's Role in Family Support and Family Support Programs	12/1/2011	The text provided does not neatly align with either a strictly liberal or conservative viewpoint. Instead, it encompasses aspects that might be embraced by both sides of the political spectrum.<br><br>Key elements of the text include:<br><br>1. **Emphasis on Child Development and Family Support:** The focus on children's social, emotional, and physical health, and the role of family relationships in early childhood development, is a concern shared across political lines. Both liberal and conservative viewpoints generally acknowledge the importance of a supportive family environment for child development.<br><br>2. **Acknowledgment of Contemporary Stressors:** The text notes the challenges contemporary American families face, such as economic pressures and changes in family structures. This recognition of societal stressors and their impact on families can be a point of discussion in both liberal and conservative circles, though the proposed solutions might differ.<br><br>3. **Role of Pediatricians and Community:** The text advocates for pediatricians to partner with families and community providers to support children's well-being. This approach suggests a community-oriented perspective, which is often more aligned with liberal viewpoints that emphasize communal support and social responsibility. However, the focus on family well-being and resilience could also resonate with conservative values that emphasize family integrity and self-reliance.<br><br>4. **Importance of Family Structure and Support Programs:** The discussion about changes in family structures (e.g., single parenting, divorce rates) and the need for family support programs is another area where the text intersects with both liberal and conservative perspectives. Conservatives often emphasize traditional family structures, while liberals might focus more on supporting diverse family configurations and providing community-based assistance.<br><br>5. **Non-Political, Professional Focus:** The text appears to be from a professional, possibly medical, perspective, focusing on the well-being of children and families rather than advocating a specific political agenda. It emphasizes evidence-based approaches and the role of healthcare professionals in supporting families.<br><br>In summary, while the text touches on topics relevant to both liberal and conservative viewpoints, it does so in a way that transcends a simple political categorization, focusing instead on the health and development of children within the context of family and community.	neutral
AAP	Meningococcal Conjugate Vaccines Policy Update: Booster Dose Recommendations	12/1/2011	The text you provided is a detailed medical guideline regarding the use of quadrivalent meningococcal conjugate vaccines. It presents recommendations from the Advisory Committee on Immunization Practices of the Centers for Disease Control and Prevention and the American Academy of Pediatrics. These recommendations are based on scientific data and research regarding the effectiveness, immunogenicity, and epidemiology of meningococcal disease and its vaccines.<br><br>This text does not inherently align with a liberal or conservative viewpoint. It is a scientific and medical document focused on public health guidelines and does not delve into political or ideological discussions. The recommendations are based on empirical data and aim to inform healthcare providers and public health officials about best practices in immunizing adolescents and individuals at high risk of meningococcal disease.<br><br>Public health guidelines like these are typically developed with the goal of protecting and improving the health of the population, regardless of political affiliations or ideologies. They are based on scientific research and evidence, and their primary concern is the safety and well-being of individuals in the context of infectious diseases.	neutral
AAP	Adolescents and HIV Infection: The Pediatrician's Role in Promoting Routine Testing	11/1/2011	The text provided is a detailed medical and public health perspective on HIV testing and prevention among adolescents and young adults. It does not explicitly align with a traditional liberal or conservative political viewpoint. Instead, it focuses on medical and epidemiological facts, public health policies, and recommendations for healthcare providers, particularly pediatricians, in the context of HIV/AIDS prevention and control.<br><br>The text emphasizes the importance of routine HIV testing, risk assessment, and counseling as crucial components of adolescent healthcare. It outlines the epidemiological data on HIV/AIDS among young people, the risk factors associated with HIV transmission, and the disparities in infection rates among different demographic groups. It also discusses the barriers to HIV testing and the need for nonjudgmental, confidential healthcare environments to encourage testing and counseling.<br><br>This approach is grounded in public health principles and evidence-based medicine rather than in a specific political ideology. The	neutral

			emphasis on routine screening, access to healthcare, and addressing health disparities can be seen as aligning with broader public health goals that transcend political affiliations.	
AAP	Media Use by Children Younger Than 2 Years	11/1/2011	The text from the American Academy of Pediatrics (AAP) policy statement on media use in children doesn't align specifically with either a liberal or conservative viewpoint. It's a scientific and health-oriented perspective focused on the developmental impacts of media exposure on children, particularly those under the age of two.<br><br>The AAP's stance is based on research findings and aims to provide guidance for parents and pediatricians regarding the potential negative effects of media on young children's development. The statement emphasizes the lack of evidence for educational benefits from media use in very young children and highlights concerns about potential adverse effects on health, development, and parent-child interaction.<br><br>This approach is characteristic of public health and pediatric policy, which prioritizes evidence-based recommendations for child health and development, rather than adhering to a particular political ideology. The focus is on scientific research and the wellbeing of children, rather than on promoting a liberal or conservative agenda.	neutral
AAP	Poliovirus	10/1/2011	"The text provided is a medical and public health policy statement regarding polio vaccination schedules and does not align with a traditional liberal or conservative viewpoint. It is a scientific and medical discussion focused on the immunization schedule for poliovirus to maintain high immunity levels in children in the United States and to prevent outbreaks. The text discusses the history and rationale behind the use of inactivated poliovirus vaccine (IPV) in the U.S., the effectiveness of different vaccination schedules, and recommendations for immunization against polio.<br><br>The information is presented in a factual manner, emphasizing the importance of IPV in preventing polio and maintaining public health safety. It outlines the benefits and necessity of following a specific vaccination schedule to ensure effective immunization against polio, especially considering the global context of polio eradication and the risk of polio importation into the U.S.<br><br>The text's focus on public health and safety, evidence-based medical practices, and the importance of vaccination for disease prevention places it within the realm of scientific discourse rather than political ideology. Therefore, it does not specifically align with either a liberal or conservative viewpoint but rather represents a perspective grounded in medical science and public health policy."	neutral
AAP	Additional Recommendations for Use of Tetanus Toxoid, Reduced-Content Diphtheria Toxoid, and Acellular Pertussis Vaccine (Tdap)	10/1/2011	The text provided appears to be a scientific and medical recommendation regarding the use of Tdap (tetanus, diphtheria, and acellular pertussis) vaccine, formulated by reputable health organizations such as the American Academy of Pediatrics and the Centers for Disease Control and Prevention. This text primarily focuses on updating guidelines based on clinical trials and safety data, and it is not explicitly aligned with either a liberal or conservative viewpoint.<br><br>The recommendations are based on scientific evidence and public health considerations, rather than political ideologies. The aim is to ensure effective immunization against certain diseases while considering safety and reactogenicity data. Such health recommendations typically transcend political lines, focusing instead on the welfare and health of the public.<br><br>In summary, the text is a scientific guideline on vaccination and does not inherently align with any specific political ideology.	neutral

AAP	Recommendations for Administering Hepatitis A Vaccine to Contacts of International Adoptees	10/1/2011	The text provided is a factual summary of recommendations and policies regarding the administration of hepatitis A vaccine, particularly in the context of international adoptions. It is primarily medical and public health-oriented, focusing on reducing the risk of hepatitis A infection. This text does not explicitly align with either a liberal or conservative political viewpoint. It is centered around health recommendations from established medical authorities like the Advisory Committee on Immunization Practices of the Centers for Disease Control and Prevention and the American Academy of Pediatrics.<br><br>Such health guidelines are typically based on epidemiological data and public health concerns rather than political ideologies. They aim to protect public health and individual well-being, regardless of political leanings. The focus is on preventing disease transmission in specific situations (like adoption from countries with high hepatitis A rates) and ensuring the health and safety of both the adoptees and their contacts in the receiving country.<br><br>In today's highly polarized environment, public health policies can sometimes be interpreted through political lenses, but this text itself is neutral and focuses on medical recommendations and data-driven practices.	neutral
AAP	Recommendations for Prevention and Control of Influenza in Children, 2011–2012	10/1/2011	The text provided is a detailed and informative statement on influenza vaccination and antiviral medication recommendations for children. It is largely a scientific and medical statement, primarily focused on public health and evidence-based medicine. This kind of text is typically not aligned with a specific political viewpoint, whether liberal or conservative, as it is grounded in medical research and public health principles.<br><br>The recommendations are from the American Academy of Pediatrics and are based on data and studies about influenza, its impact on children, and the effectiveness of vaccines and antiviral medications. The emphasis is on protecting children and communities from influenza, reducing the risk of complications, and promoting overall public health safety.<br><br>Such texts are usually crafted based on scientific evidence and medical expertise, aiming to provide guidance to healthcare professionals and inform public health policy. They are generally intended to transcend political affiliations and focus on the wellbeing and health of the population, especially vulnerable groups like children.	neutral
AAP	Prevention of Varicella: Update of Recommendations for Use of Quadrivalent and Monovalent Varicella Vaccines in Children	9/1/2011	The text provided is a factual, scientific report focused on the efficacy and safety of varicella-containing vaccines, including information about the associated risk of febrile seizures. It does not inherently align with either a liberal or conservative viewpoint.<br><br>This type of text is characteristic of medical and scientific literature, aiming to present data and evidence-based recommendations rather than opinions or ideological perspectives. The focus is on providing information to healthcare providers and the public about the benefits and risks of different vaccination strategies, based on clinical studies and statistical data.<br><br>In the context of public discourse, vaccine policies and their discussion can sometimes be influenced by political viewpoints. However, the text itself remains neutral, focusing solely on the medical and scientific aspects of vaccine administration.	neutral
AAP	Climatic Heat Stress and Exercising Children and Adolescents	9/1/2011	The text provided is a scientific and medical overview focusing on the risks and prevention of exertional heat illness in children and adolescents during sports and physical activities in hot environments. It does not align with a specific liberal or conservative viewpoint. Instead, it presents research findings and guidelines from the American Academy of Pediatrics, emphasizing the importance of proper preparation, hydration, acclimatization, and monitoring to prevent heat-related illnesses in youth. The text is factual and neutral, dealing with health and safety issues rather than political ideology.	neutral
AAP	Professional Liability Insurance and Medicolegal Education for Pediatric Residents and Fellows	9/1/2011	The text you've provided from the American Academy of Pediatrics (AAP) about professional liability insurance for pediatric residents and fellows doesn't align directly with either a liberal or conservative political viewpoint. Instead, it focuses on practical and professional considerations regarding medical liability, insurance coverage, and risk management for medical trainees in pediatrics.<br><br>This type of information is generally non-partisan and is aimed at informing and guiding medical professionals, irrespective of their political affiliations or ideologies. It deals with the realities of medical practice, emphasizing the importance of being well-informed about legal responsibilities and insurance coverage as part of professional development in the medical field.<br><br>Political viewpoints (liberal or conservative) typically come into play with broader policy discussions about healthcare systems, insurance regulations, and medical liability laws, rather than specific professional guidelines like these.	neutral

AAP	Boxing Participation by Children and Adolescents	9/1/2011	The text provided presents a neutral, fact-based analysis of the risks associated with boxing for children and adolescents. It does not align strictly with either a liberal or conservative viewpoint. Instead, it focuses on presenting medical data and recommendations from various health organizations regarding the physical risks of boxing, particularly related to head injuries and concussions. The text outlines the positions of medical bodies like the American Academy of Pediatrics and the Canadian Paediatric Society, emphasizing their opposition to boxing for youth due to health risks, and contrasts this with the benefits that supporters of amateur boxing claim, such as exercise, self-discipline, and self-confidence.<br><br>This approach is characteristic of a scientific and medical perspective, prioritizing data and health outcomes over political or ideological stances. The text aims to inform and educate about the potential dangers of boxing for young participants, highlighting the need for careful consideration and possibly the exploration of alternative sports.	neutral
AAP	Recommendations for the Prevention of Perinatal Group B Streptococcal (GBS) Disease	9/1/2011	The text you provided focuses on the clinical and public health aspects of perinatal Group B Streptococcal (GBS) disease and the guidelines issued by the Centers for Disease Control and Prevention (CDC) and endorsed by the American Academy of Pediatrics (AAP). This text is primarily concerned with medical and scientific information related to the prevention, diagnosis, and treatment of GBS in neonates. It discusses the evolution of these guidelines, the methodology behind them, and their impact on reducing the incidence of early-onset GBS disease in neonates.<br><br>The content of this text is not aligned with a specific political viewpoint, whether liberal or conservative. It is a factual, evidence-based discussion of medical guidelines and practices. The focus is on clinical recommendations, evidence grading, and the rationale behind various medical interventions in the context of perinatal care. Such scientific and medical discussions typically transcend political ideologies, aiming instead to provide the best possible care based on current evidence and research.<br><br>In summary, this text is neutral in terms of political alignment and is focused on medical and scientific information related to GBS in neonates.	neutral
AAP	Consent for Emergency Medical Services for Children and Adolescents	8/1/2011	The text you provided is a policy statement from the American Academy of Pediatrics (AAP) regarding the medical treatment of minors, especially in emergency situations where parental consent may not be available. It outlines the legal and ethical considerations for healthcare professionals when treating minors, including situations where minors can legally consent to their own treatment, such as in cases of emancipation or when they meet the criteria of the mature minor doctrine.<br><br>This text does not explicitly align with a liberal or conservative viewpoint. Instead, it focuses on legal and ethical guidelines within the healthcare context. It emphasizes the importance of ensuring the welfare and safety of pediatric patients in emergency situations, even in the absence of parental consent, while also considering legal statutes and principles. The statement aims to provide guidance to healthcare professionals on how to navigate complex situations involving the treatment of minors, balancing the need for urgent medical care with legal and ethical considerations.<br><br>In terms of political viewpoints, both liberal and conservative perspectives might find elements in this text that align with their broader principles. Liberals might support the emphasis on the rights of minors and the provision of care regardless of ability to pay, while conservatives might appreciate the respect for legal statutes and the recognition of parental rights in most situations. However, the primary focus of this statement is on medical ethics and legal compliance, rather than on promoting a specific political ideology.	neutral
AAP	Children, Adolescents, Obesity, and the Media	7/1/2011	The text presented does not inherently align with a specific liberal or conservative viewpoint. Instead, it focuses on the public health issue of obesity, particularly in children and adolescents, and examines the role of media and advertising in its development. The text discusses the correlation between screen time, advertising of unhealthy foods, and obesity, advocating for measures like banning junk-food advertising in children's programming and asking key questions about media use during pediatric visits.<br><br>This approach is primarily centered on public health and scientific research rather than political ideology. It emphasizes the importance of understanding and addressing the factors contributing to obesity, including media influence and lifestyle habits. The recommendations given, such as limiting screen time and controlling advertising directed at children, are based on research findings and public health perspectives rather than political beliefs. Therefore, it's more accurate to classify this text as a public health discussion rather than aligning it with a particular political ideology.	neutral

AAP	Use of Chaperones During the Physical Examination of the Pediatric Patient	5/1/2011	The text you've provided does not align distinctly with either a liberal or conservative viewpoint. Instead, it focuses on ethical and practical considerations in pediatric healthcare, specifically regarding the use of chaperones during physical examinations. The content emphasizes the importance of communication, respect for patient comfort and privacy, the role of chaperones, and adherence to medical ethics and state regulations.<br><br>These principles are generally accepted across the medical profession and are not inherently political or ideologically driven. They reflect a commitment to patient care, privacy, and professionalism that transcends political affiliations. The focus on patient-centered care, shared decision-making, and respect for privacy and confidentiality are universal healthcare values.	neutral
AAP	Health Information Technology and the Medical Home	5/1/2011	The text you've provided appears to align more closely with a liberal viewpoint, particularly in its emphasis on comprehensive and universal healthcare strategies, which are typically supported by liberal ideologies. The focus on developing a thorough electronic infrastructure to manage pediatric health information, ensuring its portability and accessibility across various healthcare settings, and advocating for financial incentives to support these developments are characteristic of a progressive approach to healthcare.<br><br>Liberal perspectives often prioritize the implementation of systemic solutions to enhance the quality, efficiency, and accessibility of healthcare services. The text's emphasis on creating an interconnected health information system, which would facilitate better care coordination and patient outcomes, reflects this approach. Additionally, the support for financial incentives to promote the adoption of advanced health information technologies aligns with the liberal view of actively using governmental and institutional policies to drive positive changes in healthcare.<br><br>In contrast, conservative viewpoints typically favor less government intervention in healthcare, focusing more on market-driven solutions and individual choice. The comprehensive and coordinated approach outlined in the text, emphasizing systemic changes and the use of incentives to encourage adoption of certain practices, is less aligned with these conservative principles.<br><br>However, it's important to note that healthcare policy can transcend traditional political divisions, and elements of the approach described in the text could receive support from individuals across the political spectrum, especially those concerned with improving pediatric healthcare outcomes.	liberal
AAP	Chemical-Management Policy: Prioritizing Children's Health	5/1/2011	The text provided appears to reflect a perspective that emphasizes environmental and public health concerns, particularly regarding the effects of chemical exposure on children and pregnant women. This viewpoint aligns more closely with a liberal perspective, especially in its advocacy for stronger government regulation and oversight of chemicals in commerce, a focus on preventive health measures, and the emphasis on protecting vulnerable populations like children and pregnant women.<br><br>Liberal viewpoints typically support the idea of government intervention to protect public health and the environment, and the text's criticism of the Toxic Substance Control Act (TSCA) for its ineffectiveness in this regard aligns with such views. It calls for more stringent regulations and responsibilities for chemical manufacturers, including premarket testing and postmarket follow-up, which are consistent with a liberal approach to environmental policy and public health.<br><br>Conservative viewpoints, on the other hand, often prioritize less government intervention in business and commerce and might argue for more market-based or voluntary approaches to managing chemical safety. The text's criticism of voluntary programs as inadequate and its advocacy for rewriting chemical-management policy to impose more obligations on manufacturers diverge from typical conservative positions.<br><br>It's important to note that these alignments are not absolute and there can be overlap in viewpoints, but based on the predominant themes of government intervention for public health and environmental protection, the text leans more towards a liberal viewpoint.	liberal

AAP	Rabies-Prevention Policy Update: New Reduced-Dose Schedule	4/1/2011	The text provided is a factual and scientific statement from the Advisory Committee on Immunization Practices of the Centers for Disease Control and Prevention regarding the adjustment of doses for rabies postexposure prophylaxis. It focuses on public health policy and medical evidence related to rabies vaccination and does not inherently align with a specific liberal or conservative viewpoint.<br><br>The nature of this text is primarily medical and scientific, presenting data and recommendations based on research and clinical evidence. It addresses the efficacy and safety of rabies vaccines and the appropriate dosing regimen for different populations. Such information is typically neutral and aims to inform medical practice and public health policy, rather than promote a political agenda.<br><br>In the context of public health and medicine, recommendations like these are usually based on evidence from clinical trials, observational studies, and expert consensus. They aim to optimize patient care and public health outcomes based on the best available evidence, irrespective of political ideologies.<br><br>However, it's important to recognize that the interpretation of such statements can sometimes be influenced by an individual's political beliefs, especially when it comes to public health interventions. For instance, views on vaccine policies and public health recommendations can vary among different political groups. But the statement itself, as presented, is neutral and focused on medical practice and public health rather than political ideology.	neutral
AAP	Creating Healthy Camp Experiences	4/1/2011	The text provided doesn't specifically align with a traditional liberal or conservative viewpoint. Instead, it appears to be a neutral, health-oriented statement focused on ensuring the safety and well-being of children attending camps. It outlines recommendations for health appraisals and preparations for children participating in day or resident camps, emphasizing the importance of collaboration among parents, healthcare providers, and camp staff. The statement addresses various aspects of camp health and safety, including physical and psychological preparedness, camp health policies, management of medical conditions, and emergency preparedness. This approach is typical of professional health organizations like the American Academy of Pediatrics and the American Camp Association, which focus on evidence-based practices and child welfare rather than political ideologies.	neutral
AAP	Ultraviolet Radiation: A Hazard to Children and Adolescents	3/1/2011	The text you provided is a comprehensive and detailed discussion on the health impacts of ultraviolet radiation (UVR), skin cancer, and sun protection measures. It focuses on medical and scientific facts about UVR's effects on skin, the risks of skin cancer, the importance of sun protection, and the role of vitamin D in human health. This text does not explicitly align with a liberal or conservative viewpoint.<br><br>It presents factual, evidence-based information that is relevant to public health and medical understanding, irrespective of political leanings. The emphasis on scientific evidence, public health recommendations, and the risks associated with UVR exposure and artificial tanning are consistent with a neutral, medically informed perspective.<br><br>Political viewpoints (liberal or conservative) typically come into play more clearly in discussions about policy responses to such health information, public health funding, regulation of industries (like tanning salons), or education programs, rather than in the presentation of the medical facts themselves.	neutral
AAP	Principles of Health Care Financing	11/1/2010	The text you provided aligns more closely with a liberal viewpoint. It emphasizes the need for comprehensive, affordable health insurance coverage for children and adolescents, including those from low-income families. The focus on ensuring access to a wide range of health services, including preventive and mental health care, and the advocacy for government involvement in providing and regulating health insurance, are typical of liberal perspectives. These views generally prioritize social welfare, equity in healthcare access, and a significant role for government in ensuring these outcomes. Conservative viewpoints, on the other hand, often emphasize limited government intervention, personal responsibility, and market-based solutions to healthcare. The text's emphasis on broad access to healthcare services, regardless of income, and the detailed recommendations for government and employer involvement in health insurance provision, are more in line with liberal ideologies.	liberal

AAP	Media Education	11/1/2010	The text provided appears to align more closely with a liberal viewpoint. This assessment is based on several key aspects of the content:<br><br>1. **Emphasis on Public Health and Education**: The text focuses heavily on the public health risks associated with media exposure, especially for children and adolescents. It advocates for media education as a means to mitigate these risks. This approach is typically more aligned with liberal perspectives, which often prioritize public health initiatives and educational reforms.<br><br>2. **Concerns about Media Violence and Sexual Content**: The discussion about the harmful effects of media violence and sexual content on young people, and the call for more responsible media portrayal, is a concern often voiced in liberal circles. Liberals generally support measures to protect children from potentially harmful content.<br><br>3. **Focus on Regulation and Government Involvement**: The recommendations for increased government involvement in media education and regulation, including the suggestion that Congress should mandate and fund media education, align with liberal viewpoints that endorse a more active role for government in addressing social issues.<br><br>4. **Attention to Specific Issues like Tobacco, Alcohol, and Drug Use**: The text's emphasis on the portrayal of tobacco, alcohol, and illicit drugs in the media, and the impact this has on youth, reflects a liberal concern for public health and the influence of corporate advertising on consumer behavior.<br><br>5. **Advocacy for Early Childhood Development and Education**: The focus on the importance of early childhood interactions over media exposure, and the suggestion that pediatricians and schools take active roles in this area, aligns with liberal values that emphasize early education and development.<br><br>Overall, the text's emphasis on public health, education, regulation, and specific social issues leans towards a liberal viewpoint. However, it's important to note that the categorization of viewpoints as strictly "liberal" or "conservative" can sometimes oversimplify complex issues, and different aspects of these topics might be championed or contested by individuals across the political spectrum.	liberal
AAP	Postnatal Corticosteroids to Prevent or Treat Bronchopulmonary Dysplasia	10/1/2010	The text provided doesn't align with a specific liberal or conservative viewpoint. It is a medical and scientific discussion regarding the use of postnatal glucocorticoids for the treatment or prevention of bronchopulmonary dysplasia (BPD) in preterm infants. The focus is on reviewing current research, evaluating the efficacy and safety of various glucocorticoid treatments (like dexamethasone and hydrocortisone), and making recommendations based on the available evidence.<br><br>The text discusses various studies and trials, their findings, and implications for clinical practice. It emphasizes the need for balanced judgment in clinical decisions, considering the potential benefits and risks of treatments. The approach is empirical and evidence-based, characteristic of scientific and medical literature, rather than aligned with a political ideology.	neutral
AAP	Children, Adolescents, Substance Abuse, and the Media	10/1/2010	The text provided doesn't align strictly with a liberal or conservative viewpoint; rather, it takes a public health perspective on the issue of adolescent substance use. The focus is on the multifactorial causes of substance use in adolescents, with a particular emphasis on the role of media and advertising in promoting tobacco and alcohol use among this age group.<br><br>The text cites extensive research to argue that the portrayal of tobacco and alcohol in media, as well as direct advertising, significantly influences adolescent behavior. It discusses the effectiveness of advertising in influencing children and adolescents, the portrayal of drugs in entertainment media, and the impact of new digital media technologies on drug exposure.<br><br>The recommendations and guidelines provided, such as banning tobacco advertising and limiting alcohol advertising, are aimed at protecting public health, particularly the health of children and adolescents. These suggestions are based on research findings and public health principles rather than political ideologies.<br><br>While some aspects of the text, such as advocating for regulatory measures on advertising, may resonate more with those holding liberal viewpoints who generally support regulatory interventions for public welfare, the core focus of the text is on health and safety, transcending a simple liberal-conservative dichotomy. The recommendations are aimed at reducing substance use among adolescents and mitigating its negative health impacts, which is a concern that can be shared across the political spectrum.	neutral

AAP	Recommendations for Prevention and Control of Influenza in Children, 2010–2011	10/1/2010	The text provided is a detailed, evidence-based medical guideline on the use and recommendations of the trivalent seasonal influenza vaccine and antiviral medications for children. It outlines specific recommendations for different age groups, high-risk groups, healthcare personnel, and pregnant women. The guideline emphasizes the importance of vaccination and rapid treatment of influenza to reduce morbidity and mortality in children. It also discusses the use of antiviral medications, contraindications, and precautions related to the influenza vaccine.<br><br>This text does not inherently align with a liberal or conservative viewpoint. It is a scientific and medical document focused on public health and pediatric care, specifically regarding influenza vaccination and treatment. The recommendations are based on medical research and public health principles rather than political ideologies. The primary focus is on the safety, efficacy, and public health implications of influenza vaccination and antiviral use in children. Political viewpoints typically do not influence such medical guidelines, which are based on clinical evidence and expert consensus.	neutral
AAP	Sexuality, Contraception, and the Media	9/1/2010	The text provided doesn't align strictly with either a liberal or conservative viewpoint. Instead, it takes a public health approach to the issue of adolescent sexual activity and media influence. The focus is on the potential health risks associated with early sexual activity, such as pregnancy and sexually transmitted infections (STIs), and how media exposure influences adolescent behavior and attitudes towards sex.<br><br>The text acknowledges the reality of adolescent sexual activity and the pervasive sexual content in media, critiquing the disconnect between media portrayals of sex and the need for realistic sexual education. It points out the ineffectiveness of abstinence-only education, suggesting a need for comprehensive sex education that includes information about contraception and sexual responsibility. This perspective is often associated with a more liberal viewpoint, which tends to support comprehensive sex education and acknowledges the role of media in shaping behaviors and attitudes.<br><br>However, the text also emphasizes the importance of parental guidance and media literacy, a perspective that can resonate with more conservative viewpoints that emphasize family involvement and personal responsibility in addressing social issues.<br><br>Overall, the text seems to strive for a balanced view, advocating for responsible media portrayals of sex and the importance of comprehensive sex education, while also acknowledging the role of parents and the need for media literacy. This approach suggests a focus on practical solutions to public health issues rather than adherence to a strictly liberal or conservative ideology.	neutral
AAP	Child Fatality Review	9/1/2010	The text provided primarily aligns with a public health perspective rather than fitting neatly into a liberal or conservative political viewpoint. It focuses on the importance of systematic reviews of child deaths to identify preventable causes and improve public health strategies. The emphasis is on evidence-based approaches to reduce child mortality, including accurate data collection and analysis, interagency collaboration, and legislative support for enhanced child fatality review processes. While public health initiatives can be supported by political ideologies across the spectrum, the text itself does not advocate for a specific liberal or conservative agenda but rather promotes a comprehensive and systematic approach to understanding and preventing child fatalities.	neutral

AAP	Cochlear Implants in Children: Surgical Site Infections and Prevention and Treatment of Acute Otitis Media and Meningitis	8/1/2010	The text provided is a detailed, scientific discussion of cochlear implants, including their use, potential complications, and recommended preventative and treatment strategies for associated conditions like bacterial meningitis and otitis media. It primarily focuses on medical and technical aspects of cochlear implants, without directly addressing any political or ideological viewpoints.<br><br>However, if one were to consider the broader implications or perspectives that could be inferred, it might touch on several areas that are often associated with liberal or conservative viewpoints:<br><br>1. **Healthcare and Accessibility**: The emphasis on medical advancements and accessibility to such technology might align more with liberal viewpoints, which often advocate for broader access to healthcare and advancements in medical technology.<br><br>2. **Preventative Healthcare**: The focus on vaccination and preventative measures could be seen as aligning with a public health perspective, often more emphasized in liberal policies.<br><br>3. **Scientific Approach**: The text's reliance on scientific data and evidence-based recommendations aligns with a viewpoint that prioritizes scientific understanding and research, a stance typically more associated with liberal perspectives.<br><br>4. **Individual Care and Treatment**: The detailed recommendations for individualized patient care might resonate with conservative viewpoints that emphasize individual choice and responsibility in healthcare decisions.<br><br>Overall, the text is more scientific and medical in nature rather than political. Its content doesn't inherently align with a strictly liberal or conservative viewpoint but could be interpreted through the lens of broader ideological stances on healthcare, science, and individual care.	neutral
AAP	Recommendations for the Prevention of Streptococcus pneumoniae Infections in Infants and Children: Use of 13-Valent Pneumococcal Conjugate Vaccine (PCV13) and Pneumococcal Polysaccharide Vaccine (PPSV23)	7/1/2010	The text you've provided is a scientific and medical statement regarding the use of pneumococcal vaccines. It discusses the introduction and recommendation of a 13-valent pneumococcal conjugate vaccine (PCV13) following the observed efficacy and limitations of the 7-valent pneumococcal conjugate vaccine (PCV7). This statement includes information on vaccine safety, immunogenicity, recommended schedules for vaccination, and its importance in public health, particularly for children.<br><br>This text does not inherently align with a specific political viewpoint, whether liberal or conservative. It is a factual, evidence-based discussion focused on medical science and public health. The content is centered around the effectiveness of vaccines in preventing diseases, the importance of updating vaccine formulations to cover a broader range of disease-causing strains, and recommendations for vaccine administration.<br><br>Political viewpoints (liberal or conservative) typically come into play when discussing policy decisions, funding, or public health strategies around such medical interventions, rather than the scientific facts themselves. However, this text stays within the realm of scientific discussion and medical recommendations, not extending into policy or political ideology.	neutral
AAP	Ritual Genital Cutting of Female Minors	7/1/2010	The text you've provided aligns primarily with a human rights and ethical medical practice viewpoint, focusing on the welfare and rights of children. The American Academy of Pediatrics (AAP) is expressing a firm stance against female genital cutting (FGC), a practice widely recognized as a violation of the rights of girls and women and harmful to their health. This position is based on medical, ethical, and human rights considerations, rather than a strictly political or ideological viewpoint.<br><br>In the context of liberal versus conservative viewpoints, this statement may not neatly align with either side as traditionally defined in American politics. Issues of human rights, child welfare, and medical ethics often transcend traditional political boundaries. Both liberals and conservatives might find common ground in opposing practices that harm children or infringe upon basic human rights, although they might approach these issues from different philosophical or moral perspectives.	neutral

AAP	Increasing Immunization Coverage	6/1/2010	The text you provided does not explicitly align with a traditional liberal or conservative viewpoint. It focuses on factual information and policy recommendations regarding immunization, vaccine coverage, and challenges in vaccine delivery and administration. These include:<br><br>1. **Historical and Current Immunization Efforts**: The text outlines the history of immunization efforts, starting from the American Academy of Pediatrics' 1977 statement advocating universal immunization, through the policy developments in 1995 and 2003, to recent progress and challenges.<br><br>2. **Immunization Rates and Goals**: It discusses immunization coverage rates, the success in meeting certain targets, and areas where goals (like those set in Healthy People 2010) have not been met.<br><br>3. **Challenges to Immunization Efforts**: The text addresses various challenges, including financial barriers, vaccine shortages, rising costs, manufacturing issues, and the impact of the anti-vaccination movement.<br><br>4. **Public Health Policy Recommendations**: It offers detailed recommendations for improving immunization coverage, such as advocating for reforms in vaccine distribution and payment systems, increasing public awareness, and addressing misinformation about vaccines.<br><br>5. **Role of Pediatricians and Health Professionals**: The text emphasizes the role of pediatricians and health organizations in advocating for and ensuring timely immunization, countering anti-vaccination misinformation, and supporting the medical home model for vaccine delivery.<br><br>This approach is characteristic of a public health perspective, which generally transcends political affiliations and focuses on evidence-based strategies to improve health outcomes. The emphasis is on factual, scientific information and practical policy solutions, rather than ideological or partisan positions.	neutral
AAP	Athletic Participation by Children and Adolescents Who Have Systemic Hypertension	6/1/2010	The text provided primarily presents a medical and health-based viewpoint rather than aligning specifically with liberal or conservative political viewpoints. It focuses on updating guidelines and recommendations for the participation of athletes with hypertension, based on established medical reports and research findings.<br><br>The information is rooted in scientific and medical research, such as the "36th Bethesda Conference: Eligibility Recommendations for Competitive Athletes with Cardiovascular Abnormalities," the "Fourth Report on the Diagnosis, Evaluation, and Treatment of High Blood Pressure in Children and Adolescents," and the "Seventh Report of the Joint National Committee on Prevention, Detection, Evaluation, and Treatment of High Blood Pressure."<br><br>The guidelines address concerns about hypertension in children and adolescents, especially those engaged in competitive sports, and suggest lifestyle changes and medical evaluations for managing this condition. This includes recommendations for different stages of hypertension and considerations for special populations such as obese athletes or those with spinal cord injuries.<br><br>Such content is typically seen as non-partisan, focusing on health and safety rather than promoting a particular political ideology. It aims to provide evidence-based guidance for healthcare providers and those involved in sports medicine, emphasizing the importance of monitoring and managing high blood pressure in young athletes to prevent potential health complications.	neutral

AAP	Honoring Do-Not-Attempt-Resuscitation Requests in Schools	5/1/2010	The text you've provided presents a nuanced and complex discussion about the ethical, legal, and medical aspects of implementing Do-Not-Attempt-Resuscitation (DNAR) orders for children and adolescents with complex chronic conditions, especially in the school environment. This topic does not neatly align with traditional liberal or conservative viewpoints as it involves a blend of ethical considerations, healthcare policy, legal implications, and educational accommodations.<br><br>Liberal viewpoints typically emphasize individual rights, comprehensive healthcare access, and supportive social services. In this context, a liberal perspective might focus on ensuring that the rights and wishes of the students and their families are respected and that appropriate healthcare and support services are available in schools.<br><br>On the other hand, conservative viewpoints often emphasize personal responsibility, limited government intervention, and traditional values. From this perspective, there might be a focus on the role of family decision-making in healthcare, concerns about the implications of such policies for school and medical staff, and potentially the cost implications of implementing these policies in educational settings.<br><br>However, it's important to note that issues surrounding end-of-life care, especially for minors, transcend simple political categorizations. They often bring together a diverse range of stakeholders with different priorities, including healthcare providers, educators, legal experts, and ethicists, along with the affected families and individuals. The text reflects this complexity and doesn't explicitly advocate for a political stance, instead focusing on the practical and ethical challenges of honoring DNAR requests in schools.	neutral
AAP	Ritual Genital Cutting of Female Minors	5/1/2020	The text provided is primarily a medical and ethical discussion on female genital cutting (FGC) or female genital mutilation (FGM), rather than a political statement aligned strictly with liberal or conservative viewpoints. It focuses on the medical, cultural, ethical, and legal aspects of the practice, highlighting the health risks and the need for cultural sensitivity and legal regulation.<br><br>Key aspects of the text include:<br><br>1. **Medical Perspective**: It presents detailed information on the different types of FGC, their prevalence, and the associated health risks, emphasizing the position of medical organizations like the American Academy of Pediatrics, the World Health Organization, and others against FGC due to its health risks.<br><br>2. **Cultural Sensitivity**: The text acknowledges the cultural, religious, and traditional underpinnings of FGC in various communities. It suggests that while working to end harmful practices, there should be a respectful understanding of the cultural contexts in which these practices are rooted.<br><br>3. **Legal and Ethical Issues**: It discusses the legality of FGC in various countries, including the United States, and touches on ethical considerations, such as the principle of nonmaleficence and the rights of children.<br><br>4. **Educational Approach**: The text emphasizes the importance of education and counseling for families and communities that practice FGC, advocating for a culturally sensitive approach.<br><br>The text does not overtly support or oppose any political ideology but instead presents a nuanced view of a complex issue, balancing medical and ethical considerations with cultural sensitivity. It leans towards a human rights and health-focused perspective, which can resonate with both liberal and conservative viewpoints in different ways. Liberals might focus more on the human rights and gender equality aspects, while conservatives might emphasize the importance of cultural traditions and family decisions, although these are not strict or exclusive alignments.	neutral
AAP	Health Equity and Children's Rights	4/1/2010	The text provided doesn't align strictly with either a liberal or conservative viewpoint as it primarily focuses on child health equity, which is a topic that can transcend traditional political divides. However, the emphasis on social determinants of health, the role of public policy in addressing health disparities, and the focus on social justice and children's rights are themes more commonly associated with liberal viewpoints. Liberal perspectives often prioritize social equity, public health initiatives, and systemic approaches to address inequalities.<br><br>Conservative viewpoints, on the other hand, might emphasize individual responsibility, limited government intervention in healthcare, and private sector solutions to health issues. This text leans more towards advocating for systemic change and public policy interventions, which aligns more closely with liberal ideologies.	liberal

			It's important to note that issues of child health and well-being can be and often are addressed by individuals and groups across the political spectrum, and the approaches can vary significantly depending on the broader ideological and policy frameworks they are situated within.	
AAP	Prevention of Choking Among Children	3/1/2010	The text you've provided is a comprehensive and factual discussion on the topic of choking hazards for children, with a focus on prevention and safety measures. It doesn't specifically align with either a liberal or conservative viewpoint, as it is primarily educational and informative in nature, addressing public health concerns.<br><br>The content is centered around the importance of regulatory measures, safety standards, and public education to prevent choking incidents among children. It highlights the roles of various federal agencies, like the US Consumer Product Safety Commission (CPSC) and the US Food and Drug Administration (FDA), in enhancing safety standards and regulatory measures for toys and food products. Additionally, it emphasizes the need for improved surveillance, hazard evaluation, enforcement, and public education activities to mitigate the risks of choking in children.<br><br>This kind of approach to public health and safety transcends typical political divisions, focusing instead on practical and evidence-based strategies to protect a vulnerable population (children) from a well-documented risk (choking). While different political ideologies might vary in their preferred methods of regulation and the role of government in public safety, the text itself does not advocate for a specific political stance but rather for effective and comprehensive measures to address a public health issue.	neutral
AAP	Children as Hematopoietic Stem Cell Donors	2/1/2010	The text you provided is a detailed, technical discussion of hematopoietic stem cell transplantation, including its history, medical processes, ethical considerations, and guidelines, particularly concerning minors as donors. This text primarily aligns with a scientific and medical viewpoint, focusing on clinical practices, ethical considerations in medicine, and the policies of medical organizations like the American Academy of Pediatrics.<br><br>It does not explicitly align with a liberal or conservative political viewpoint. The discussion is centered on medical facts, clinical experiences, and ethical considerations in pediatric healthcare, which are topics typically addressed from a neutral, scientific standpoint rather than a political one.<br><br>The text addresses complex ethical issues in medicine, such as the involvement of minors in stem cell donation, balancing risks and benefits for donors and recipients, and the considerations of using siblings as donors. These topics are approached from a medical ethics perspective, emphasizing patient welfare, informed consent, and the ethical implications of medical decisions, rather than from a political standpoint.	neutral
ACS	Statement on Bicycle Safety and the Promotion of Bicycle Helmet Use	3/15/2023	The text provided does not inherently align with a specific liberal or conservative viewpoint. It is primarily a factual and policy-oriented statement from the American College of Surgeons Committee on Trauma, focusing on bicycle safety and helmet use. The statement emphasizes the importance of injury prevention, the effectiveness of helmet use in reducing injuries, the impact of bicycle injuries on healthcare costs, and the importance of equitable enforcement of helmet laws.<br><br>The content is more centered around public health and safety concerns rather than political ideology. Both liberal and conservative viewpoints can find aspects to support within this statement. Liberals might focus on the emphasis on equitable enforcement and public health, while conservatives might appreciate the focus on personal responsibility and prevention measures. However, the statement itself is neutral and aimed at promoting safety and health, rather than advocating for a political stance.	neutral

ACS	Statement on Supreme Court Decision in Dobbs v. Jackson Women's Health Case	6/24/2022	The statement from the American College of Surgeons (ACS) regarding the U.S. Supreme Court decision in Dobbs v. Jackson Women's Health aligns more closely with a liberal viewpoint. Key aspects of this alignment include:<br><br>1. **Opposition to Governmental Interference**: The ACS emphasizes its long-standing opposition to governmental interference in the clinical practice of medicine and the physician-patient relationship. This perspective often aligns with liberal viewpoints that advocate for personal freedoms in healthcare decisions.<br><br>2. **Support for Reproductive Services**: The statement expresses concern about the impact of the Supreme Court's decision on the availability of comprehensive and safe reproductive healthcare services. The liberal viewpoint typically supports access to reproductive services, including abortion.<br><br>3. **Autonomy in Medical Decisions**: The ACS underscores the importance of patients and physicians being primarily in control of medical decisions, without government interference. This reflects a liberal stance on healthcare autonomy, particularly in the context of reproductive choices.<br><br>4. **Protection of Physicians**: The statement advocates for the freedom of physicians to practice medicine based on medical education, experience, and scientific evidence, without fear of criminalization. This is in line with liberal views that emphasize evidence-based medicine and the protection of healthcare providers.<br><br>5. **Access to Healthcare as Essential**: The ACS points out that restricted access to healthcare negatively impacts patient health, especially those in underserved communities. This is consistent with liberal perspectives that prioritize healthcare accessibility for all, including marginalized groups.<br><br>6. **Advocacy for Evidence-Based Care**: The ACS's commitment to evidence-based care and opposition to interference in the patient-physician relationship aligns with liberal values that emphasize science and evidence in policymaking.<br><br>In summary, the ACS's statement reflects a liberal viewpoint through its emphasis on personal and professional autonomy in healthcare, support for reproductive services, and advocacy for evidence-based, accessible healthcare for all.	liberal
ACS	Support of the FSMB Policy on Physician Illness and Impairment	4/1/2022	The statement from the American College of Surgeons (ACS) reflects a viewpoint that is primarily centered around healthcare policy and physician well-being, rather than aligning strictly with traditional liberal or conservative political ideologies. It focuses on:<br><br>1. **Addressing Surgeon Shortage and Well-being**: Acknowledging the projected shortage of surgeons and the impact of the COVID-19 pandemic on surgeon well-being. This concern transcends political boundaries, as it deals with the practical aspects of healthcare provision and the health of healthcare providers.<br><br>2. **Advocacy for Supportive Pathways**: Advocating for pathways that allow physicians to seek help proactively for any impairment or illness. This approach aligns with a general emphasis on mental health and well-being, which can be seen as a universal healthcare issue rather than a politically partisan one.<br><br>3. **Confidentiality and Reduction of Stigma**: Emphasizing the importance of confidentiality in treatment and reducing stigma around seeking help, which aligns with broader healthcare initiatives to improve mental health support.<br><br>4. **Support for FSMB's Policy**: Supporting the Federation of State Medical Boards' (FSMB) policies on physician illness and impairment, focusing on patient safety and physician health. This again is more about practical healthcare policy rather than political ideology.<br><br>5. **Inclusivity and Non-Discrimination**: The statement emphasizes non-discrimination and equal access to Physician Health Programs (PHPs), which aligns with a more inclusive approach to healthcare.<br><br>6. **Financial Support and Emergency Interventions**: Highlighting the need for financial support and emergency interventions, focusing on the systemic aspects of healthcare support systems.<br><br>Overall, the statement is more aligned with healthcare policy and the specific needs of physicians, particularly surgeons, rather than	neutral

			fitting neatly into a liberal or conservative political framework. It appears to prioritize the well-being and support of medical professionals, which is a concern that cuts across political lines.	
ACS	Surgeon Well-Being	12/9/2021	The text you've provided doesn't align explicitly with either a liberal or conservative viewpoint in the traditional political sense. It focuses on the well-being, resilience, and work-life integration of surgeons, emphasizing the importance of addressing the holistic needs of individuals in the surgical profession. This includes considerations of emotional, occupational, physical, intellectual, financial, social, spiritual, and environmental well-being.<br><br>The approach is more centered on healthcare professionals' welfare and doesn't delve into politically charged topics that typically distinguish liberal and conservative ideologies. Both political spectrums could potentially support such initiatives as they align with broader goals of healthcare quality and professional well-being, which are important across political lines.<br><br>Moreover, advocating for systems-level change, work-life balance, and mental health support doesn't inherently fall into a specific political ideology but rather addresses universal concerns in the healthcare profession. The focus is on practical, supportive measures rather than ideological stances.	neutral
ACS	The American College of Surgeons Stands Against Interference in Doctor/Patient Relationship	10/22/2012	The statement from the American College of Surgeons (ACS) reflects a viewpoint that emphasizes the autonomy of patients and physicians in making healthcare decisions, opposition to legislative interference in the physician-patient relationship, and the importance of medical decisions being informed by education, experience, and scientific evidence.<br><br>This viewpoint aligns with both liberal and conservative principles in different ways:<br><br>1. **Liberal Viewpoint**: The emphasis on patient autonomy and opposition to legislative interference in personal health decisions aligns with liberal principles of individual rights and freedoms. Liberals often advocate for minimal government involvement in personal healthcare decisions, stressing the importance of personal choice and the right to privacy.<br><br>2. **Conservative Viewpoint**: The statement also resonates with conservative values, particularly the emphasis on professional autonomy and opposition to government overreach. Conservatives typically support the idea of limited government intervention in professional practices and personal affairs. The focus on the importance of medical education and experience aligns with a conservative respect for professional expertise and traditional practices.<br><br>Overall, the ACS's statement reflects a blend of principles that can be found in both liberal and conservative ideologies, focusing on the importance of professional autonomy, patient rights, and evidence-based practice, while opposing excessive government intervention in the physician-patient relationship.	neutral

ACS	Restrictive Covenants	10/1/2021	The text you've provided doesn't explicitly align with a traditional liberal or conservative viewpoint as understood in the context of political ideologies. Instead, it focuses on professional and legal advice for surgeons regarding employment contracts and restrictive covenants.<br><br>This text is primarily concerned with the following:<br><br>1. **Legal Caution and Advice:** Advising surgeons to have their employment contracts reviewed by a legal expert. This is a pragmatic approach focused on professional well-being and legal safety, rather than a political stance.<br><br>2. **Concerns About Restrictive Covenants:** Highlighting the potential negative impacts of restrictive covenants on the physician-patient relationship and access to care. While one could argue that concern for uninterrupted healthcare delivery and patient access aligns with a more liberal viewpoint valuing wider access to healthcare services, the text doesn't explicitly endorse a political ideology.<br><br>3. **Balancing Interests:** Acknowledging the reasons why employers might include restrictive covenants, suggesting a balanced view of the issue. This approach doesn't favor a particular political ideology but rather aims at finding a middle ground that is fair to both the physician and the employer.<br><br>Overall, the text seems to be more about professional and legal advice for a specific group (surgeons) rather than advocating a particular political stance. The focus is on ensuring that surgeons understand and negotiate the terms of their employment contracts in a way that is legally sound and considers the impact on healthcare delivery.	neutral
ACS	Trauma Center Designation Based upon System Need and the Economic Drivers Impacting Trauma Systems	10/1/2021	The text you provided is primarily factual and policy-oriented, focusing on the management and optimization of trauma care systems. It does not explicitly align with a traditional liberal or conservative viewpoint. Instead, it addresses practical and administrative aspects of healthcare, specifically trauma system management, and emphasizes the need for effective regional planning, equitable resource allocation, and patient-centered care.<br><br>The text discusses the importance of matching trauma center distribution to population needs, challenges in designating trauma centers, and the impact of healthcare economics on trauma care. It advocates for a systematic, needs-based approach to trauma center designation, and for governmental agencies to take a lead role in this process.<br><br>Such a focus on system efficiency, equitable resource allocation, and government oversight might resonate with viewpoints that prioritize structured, equitable access to healthcare. However, the text is more about effective healthcare management rather than promoting a specific political ideology.	neutral
ACS	Resident Access to Personal Protective Equipment	9/11/2021	The text you've provided does not neatly align with either a liberal or conservative viewpoint in the traditional sense of political ideology. Instead, it focuses on practical, ethical, and safety concerns related to the provision of personal protective equipment (PPE) for healthcare professionals, particularly surgical residents, during the COVID-19 pandemic.<br><br>The American College of Surgeons (ACS) statement emphasizes the importance of adequate PPE to ensure the safety of healthcare workers and patients and to prevent the spread of contagious diseases. It also addresses issues such as the mental health impact of PPE shortages, the need for prioritizing PPE for those at highest risk, and the condemnation of practices that restrict access to necessary PPE.<br><br>These concerns transcend typical political divides and are more about ensuring the well-being and safety of healthcare professionals and patients. They align with general public health principles and the ethical responsibility of medical institutions to protect their staff and patients.<br><br>However, it's important to note that individuals with different political views might interpret and prioritize these issues differently. For example, a more conservative viewpoint might emphasize individual responsibility and institutional autonomy, while a more liberal viewpoint might focus on systemic solutions and government intervention to ensure safety and equity in healthcare. But the statement itself is centered on professional and ethical standards in healthcare, rather than on political ideology.	neutral

ACS	Importance of Pregnancy, Parental Leave, and Workplace Accommodations for Surgical Residents and Fellows	7/29/2021	The text you've provided outlines the American College of Surgeons' (ACS) stance on parental leave and accommodations for surgeons and surgical trainees who are new parents. It doesn't align neatly with a traditionally liberal or conservative viewpoint, as it encompasses aspects that could be interpreted in various ways depending on individual perspectives.<br><br>1. **Support for Parental Leave and Family Values**: The emphasis on supporting surgeons who choose to become parents and ensuring they do not face punitive repercussions could resonate with conservative values that often prioritize family and parental roles. Additionally, advocating for equitable treatment of those who choose to have children aligns with family-centric values often found in conservative viewpoints.<br><br>2. **Advocacy for Workplace Equality and Accommodations**: The call for explicit parental leave policies, support for pregnancy and breastfeeding accommodations, and ensuring these choices do not impact professional progression or opportunities could be seen as aligning with liberal values. These aspects emphasize workplace equality, support for diverse family structures (including surrogacy and adoption), and women's rights, particularly in traditionally male-dominated fields like surgery.<br><br>3. **Overlap with Both Viewpoints**: The text also reflects a blend of values that can be appreciated across the political spectrum. It addresses practical workplace policies (like parental leave), which is a concern often shared by people regardless of their political leanings. The focus on fair and equitable treatment of all surgical trainees and surgeons, regardless of their parental choices, speaks to a broader theme of equality and fairness.<br><br>In summary, the text embodies a pragmatic approach to addressing the challenges faced by surgical trainees and surgeons who are also parents. It doesn't squarely fit into a liberal or conservative framework but rather addresses specific professional and personal needs that can be appreciated and supported across the political spectrum.	neutral
ACS	The Importance of Workplace Accommodations for Pregnancy, Parental Leave, and Lactation Support for Practicing Surgeons	7/23/2021	The text provided reflects a viewpoint that is typically associated with liberal or progressive ideologies. This alignment is evident in several key aspects:<br><br>1. **Emphasis on Equality and Inclusivity**: The text advocates for equal treatment and support for surgeons who are parents, irrespective of how they became parents (pregnancy, surrogacy, fostering, or adoption). This inclusive approach is often aligned with liberal values that emphasize equality and non-discrimination.<br><br>2. **Support for Parental Leave**: The advocacy for explicit inclusion of parental leave in employment contracts and the detailed guidelines for such leave resonate with liberal views. Liberals generally support policies that provide work-life balance and protect workers' rights, including parental leave.<br><br>3. **Workplace Accommodations and Flexibility**: The text's focus on workplace accommodations for pregnant surgeons and support for breastfeeding aligns with liberal perspectives on workers' rights and women's rights in particular.<br><br>4. **Health and Safety Considerations**: The emphasis on the health and safety of surgeons, including during pregnancy, and the provision of facilities for expressing breast milk, are consistent with liberal priorities for healthcare and worker welfare.<br><br>5. **Legal and Policy Advocacy**: The reference to federal and state laws and the encouragement of voluntary compliance with the Family Medical Leave Act (FMLA) by institutions exempt from it, demonstrates a liberal approach to policy and legal frameworks for worker protection.<br><br>In summary, the text's emphasis on equality, inclusivity, worker protection, and health and safety align it more closely with liberal ideologies. It's important to note that while these issues may be more prominently advocated by liberal ideologies, they are not exclusively liberal concerns and can be supported by individuals across the political spectrum.	liberal

ACS	Suicide Prevention	6/11/2021	The text you provided appears to be a factual summary of statistics and recommendations related to suicide prevention, with a specific focus on the role of firearms in suicide and the importance of healthcare intervention. It does not align explicitly with either a liberal or conservative viewpoint as it primarily presents data and health policy recommendations.<br><br>The viewpoint or stance in such a text can be subject to interpretation based on the reader's perspective. For instance:<br><br>- **Liberal Viewpoint Alignment**: Some may see the focus on firearm-related suicides and the advocacy for electronic health records and proactive healthcare measures as aligning more with liberal viewpoints, particularly if they associate these approaches with gun control advocacy or a more active role for healthcare in societal issues.<br><br>- **Conservative Viewpoint Consideration**: Conversely, others might argue that the emphasis on personal healthcare responsibility and the potential implications for individual gun ownership rights could be of interest to conservative viewpoints, especially if they focus on the individual rights and responsibilities aspects.<br><br>Ultimately, the text seems to be centered around public health and safety, aiming to address a significant societal issue (suicide) with data-driven and healthcare-focused solutions, which can transcend simple political categorization.	neutral
ACS	International Safety Center Releases Consensus Statement on Sharps Safety	6/1/2020	The text you provided primarily focuses on healthcare worker safety, particularly in relation to sharps injuries and exposure to bloodborne pathogens. It doesn't explicitly align with a liberal or conservative viewpoint as it is centered around occupational health and safety, a topic that generally transcends partisan lines.<br><br>The emphasis is on the effectiveness of regulatory measures (like the Occupational Safety and Health Administration's Bloodborne Pathogens Standard and the Needlestick Safety and Prevention Act) and the ongoing efforts to improve safety in healthcare settings. Such issues are usually considered matters of public health and worker safety, rather than being inherently political or ideological.<br><br>The text mentions a consensus statement aimed at further reducing sharps injuries, indicating an ongoing commitment to worker safety and public health, which are universal concerns. Political leanings typically become more evident in discussions about the role of government regulation versus market-based solutions, funding levels for public health initiatives, or the broader context of healthcare policy. This text, however, is more technical and specific in nature, dealing with occupational safety standards and practices.	neutral
ACS	Older Adult Falls and Falls Prevention	9/1/2019	The text provided from the ACS (American College of Surgeons, presumably) regarding falls in older adults and the promotion of fall prevention strategies does not align specifically with either a liberal or conservative viewpoint. It is primarily a health and safety issue focused on older adults.<br><br>The content of the text is centered around acknowledging the risks of falls in older adults, the consequences of these falls, and the importance of implementing comprehensive fall prevention programs. These measures include partnerships with community-based centers, exercise and physical therapy programs, home-based risk assessments, and collaboration with medical services and institutions. The focus is on practical, evidence-based strategies to improve the health and safety of older adults, which transcends political affiliations.<br><br>Healthcare and public safety are areas that can be addressed by policymakers across the political spectrum. While the implementation and funding of such programs might be influenced by political ideologies, the core issue of preventing falls in older adults is a common concern that is not inherently political.	neutral

ACS	Patient Safety Principles for Office-Based Surgery Utilizing Moderate Sedation/Analgesia	9/1/2019	The text provided outlines a set of guidelines and standards for patient safety in surgical procedures, particularly those involving deep sedation, analgesia, or general anesthesia in accredited surgical centers or office-based settings. This text primarily focuses on the following:<br><br>1. **Accreditation and Licensing:** It emphasizes the importance of performing surgical procedures in accredited surgical centers and having facilities that utilize moderate sedation or analgesia accredited by national or state accrediting organizations. It also mentions the necessity for physicians to be state-licensed.<br><br>2. **Patient Selection and Evaluation:** The use of the American Society of Anesthesiologists (ASA) Physical Status Classification System for selecting patients for office-based procedures is highlighted. It also stresses the need for thorough pre-procedure patient evaluation and documentation.<br><br>3. **Informed Consent:** The text underscores the importance of obtaining written informed consent from patients after discussing the risks, benefits, and alternatives of the surgery and anesthesia.<br><br>4. **Professional Competence and Scope of Practice:** Surgeons are advised to perform procedures in line with their board certification, training, experience, and state-recognized scope of practice.<br><br>5. **Anesthesia Administration and Monitoring:** It outlines the qualifications for individuals administering anesthesia and the necessity of having trained personnel present during the procedure. It also emphasizes patient monitoring and discharge criteria.<br><br>6. **Protocols for Complications:** Guidelines for transferring patients to acute care facilities in case of complications are provided, along with the requirement for regular review of patient transfers and adverse events.<br><br>7. **Record Keeping and Professional Practice Evaluation:** The text highlights the responsibility of the governing body of the office-based surgery practice in maintaining records of physician credentialing and licensure and participating in ongoing professional practice evaluation.<br><br>This text does not explicitly align with a liberal or conservative viewpoint. Instead, it focuses on medical professionalism, patient safety, and quality of care in surgical practices. These guidelines reflect a commitment to high standards in medical care, irrespective of political ideology. They are based on professional medical standards and practices designed to ensure the safety and well-being of patients undergoing surgical procedures.	neutral
ACS	Harassment, Bullying, and Discrimination	6/12/2019	The text you've provided primarily aligns with a viewpoint that emphasizes respect, tolerance, and inclusivity in the professional medical environment, particularly in the context of surgery. It doesn't neatly fit into a traditional "liberal" or "conservative" political framework but rather focuses on ethical and professional standards within the healthcare sector.<br><br>The principles outlined in the text – such as treating all individuals with respect, fostering a culture of equality, and having zero tolerance for harassment, bullying, and discrimination – are generally supported across a wide range of political and ideological spectrums, particularly within professional and institutional contexts. These principles are foundational to creating a safe and effective work environment, ensuring quality patient care, and promoting a healthy and inclusive workplace culture.<br><br>While some aspects of these principles, like the emphasis on inclusivity and combating implicit bias, might be more prominently highlighted in liberal viewpoints, the fundamental ideas of professionalism, respect, and fair treatment in the workplace are broadly accepted values.	neutral
ACS	Guidelines for the Ethical Use of Social Media by Surgeons	5/1/2019	The text you provided appears to be a set of guidelines or recommendations for surgeons on how to ethically and professionally use social media in relation to their practice and interactions with patients, colleagues, and the public. It does not align specifically with either a liberal or conservative political viewpoint. Instead, it focuses on ethical considerations, professional conduct, and the implications of social media use in a healthcare context. The guidelines address various aspects such as maintaining boundaries in the surgeon-patient relationship, privacy concerns, patient education, surgeon education, the surgeon's online persona, and professional representation.<br><br>The content is more centered around medical ethics, professionalism, and the responsible use of social media in a healthcare setting than it is about political ideology. It aims to provide surgeons with a framework to navigate the complexities of digital communication and online presence while maintaining professional standards and patient confidentiality. This approach is typical of	neutral

			professional guidelines and is intended to be applicable and relevant regardless of the personal political beliefs of individual surgeons.	
ACS	Maintaining Surgical Access with a Locum Tenens Surgeon	9/21/2018	The text you provided does not clearly align with either a liberal or conservative viewpoint. It is primarily a professional and procedural document focused on the medical field, specifically relating to the standards and guidelines for locum tenens surgeons. This type of content is generally apolitical and focused on ensuring quality, safety, and ethical practices in a specific area of healthcare.<br><br>The text outlines criteria for locum tenens surgeons, procedures for host practice groups, and principles for locum tenens agencies. These include requirements for state medical licensure, certification, credentialing processes, maintenance of surgical logs, patient care handoffs, and performance evaluations. The emphasis is on maintaining high standards of medical care and ensuring seamless integration of locum tenens surgeons into healthcare settings, rather than promoting any particular political ideology.<br><br>Political ideologies typically involve views on governance, economics, social issues, and public policy, which are not the focus of this text. The content here is centered on operational and professional standards within the healthcare industry, making it a neutral document in terms of political leanings.	neutral
ACS	Recommendations for Surgeons Caring for Patients Who Are Jehovah's Witnesses	9/1/2018	The text provided does not neatly align with a typical liberal or conservative viewpoint as understood in a political sense. It is primarily a medical and ethical discussion focused on respecting patient autonomy, particularly in the context of Jehovah's Witnesses (JWs) and their views on blood transfusions. This discussion transcends typical political categorizations and instead addresses the following key areas:<br><br>1. **Patient Autonomy and Individualized Care**: The emphasis on respecting each patient's unique values and decisions, especially regarding blood transfusions, reflects a universal medical ethic that prioritizes patient autonomy. This approach is not inherently political but is a standard practice in modern medicine.<br><br>2. **Avoidance of Generalizations and Prescriptive Guidelines**: The text advocates against blanket guidelines for JW patients, emphasizing the importance of individualized care and decision-making. This approach aligns with a patient-centered care model, which is a broadly accepted principle in healthcare.<br><br>3. **Ethical Considerations in Medical Practice**: The discussion about balancing respect for patient beliefs with the necessity of providing effective medical care, especially in life-threatening situations, reflects ethical considerations that are central to medical practice. These considerations are largely independent of political ideologies.<br><br>4. **Legal and Ethical Complexities in Treating Minors**: The text addresses the complexities involved in making medical decisions for minors, especially when their guardians' religious beliefs may conflict with medical recommendations. This aspect touches on legal and ethical issues that are again more medical and ethical in nature than political.<br><br>5. **Promotion of Bloodless Surgery**: The advocacy for bloodless surgery techniques, which benefit all patients, not just JWs, points to a focus on medical advancement and patient safety. This aspect is more about medical best practices than a political stance.<br><br>Overall, the text is more aligned with medical ethics and patient-centered care principles than with any specific political ideology. It emphasizes respect for patient beliefs and autonomy, individualized treatment plans, and ethical decision-making in healthcare, which are key tenets of modern medical practice.	neutral

ACS	Intimate Partner Violence	6/1/2018	The text you've shared presents a fact-based, educational perspective on Intimate Partner Violence (IPV) and does not align explicitly with either a liberal or conservative viewpoint. It focuses on defining IPV, acknowledging its impact across various demographics, and emphasizing the importance of recognizing, preventing, and treating IPV, especially in the medical community.<br><br>The approach is neutral and aims to raise awareness about IPV's seriousness, its physical and psychological effects, and the importance of screening and intervention. It advocates for education and support systems within the medical community, particularly among surgeons, to address IPV effectively.<br><br>Moreover, the text supports legislative and policy measures to combat IPV, which is a stance that both liberal and conservative viewpoints might agree with, albeit possibly for different reasons or with different emphasis. The focus on IPV as a major public health issue transcends typical political categorizations, aiming instead to address a critical social and health concern.	neutral
ACS	Patient Safety in the Operating Room: Team Care	6/1/2018	The text you provided focuses primarily on patient safety protocols, team-based approaches in the operating room, the use of technology for enhanced safety, and the responsibilities of surgeons. It aligns neither explicitly with a liberal nor a conservative viewpoint. Instead, it is a professional, medical guideline focused on best practices in healthcare, particularly in surgical settings.<br><br>Political viewpoints, whether liberal or conservative, often concern broader policy issues, ideological beliefs, and approaches to governance. This text, however, is centered on practical, operational procedures in healthcare, aiming to improve patient outcomes and safety. It's more about clinical governance and quality assurance in healthcare services, which are typically non-partisan issues.<br><br>Both liberal and conservative ideologies might support such measures in the interest of public health and safety, albeit for different underlying reasons or with varying emphasis on aspects like funding, regulation, and implementation. However, the text itself does not advocate for a specific political ideology or policy perspective. It's more aligned with medical professionalism and standard healthcare practices.	neutral
ACS	Medical Student Use of the Electronic Health Record	4/1/2018	The text you provided is primarily focused on the topic of medical education, particularly regarding the use of Electronic Health Records (EHR) by medical students. It outlines various viewpoints and recommendations from medical organizations like the American Medical Association (AMA), the Association of American Medical Colleges (AAMC), and others on how medical students should be trained in and allowed to use EHRs.<br><br>This text does not align clearly with either a liberal or conservative viewpoint in the traditional political sense. Instead, it addresses educational and professional standards within the field of medicine, which are generally considered non-partisan issues. The main concerns expressed in the text are about ensuring proper training for medical students, maintaining the integrity and usefulness of medical records, and addressing the potential risks and benefits of allowing medical students to access and contribute to EHRs.<br><br>The focus on educational standards, patient safety, and the evolving nature of medical training and documentation practices transcends typical political ideologies and instead reflects the ongoing efforts within the medical community to adapt to technological advancements and maintain high standards of patient care and medical education.	neutral
ACS	Credentialing and Privileging and Volume Performance Issues	4/1/2018	The text you provided is a detailed explanation of the processes and criteria involved in the credentialing and privileging of surgeons. It focuses on the qualifications, training, experience, and ongoing evaluation of surgeons, emphasizing the importance of ensuring safety and quality in surgical procedures.<br><br>This text does not inherently align with a specific political viewpoint, whether liberal or conservative. It is a technical and procedural description relevant to the healthcare field, particularly surgery. The emphasis is on professional standards, patient safety, institutional policies, and the need for rigorous assessment and continuous improvement in surgical practice.<br><br>Such content is generally non-partisan and focused on best practices in medical care, without delving into political ideologies or policies. It addresses the operational and professional aspects of healthcare, which are typically universal concerns across different political spectrums.	neutral

ACS	Recommendations for Operating Room Attire	2/27/2018	The text you provided does not inherently align with a liberal or conservative viewpoint. It is a factual account of a meeting among medical and surgical associations to discuss recommendations for operating room attire, specifically focusing on the practicality and evidence supporting the use of ear and hair coverings in surgical settings.<br><br>The conclusions drawn in the text are based on evidence and practical considerations in the medical field, particularly regarding the effectiveness and necessity of certain types of surgical attire in preventing surgical site infections (SSIs). This is a technical and professional matter, not one that typically falls within the scope of political ideologies.<br><br>The focus on evidence-based recommendations and the questioning of previously accepted practices based on new data reflects a scientific approach to policy and procedure formulation in the medical field. It emphasizes the importance of reassessing and updating practices based on the latest available evidence, which is a principle that transcends political affiliations.	neutral
ACS	Cannabis Regulation and Risk of Injury	2/1/2018	The text you provided seems to be a balanced and factual statement regarding cannabis use, its legal status, and associated risks, especially in the context of driving. It doesn't explicitly align with a liberal or conservative viewpoint but rather focuses on public health and safety concerns. Here's an analysis of how it might be perceived from both perspectives:<br><br>1. **Liberal Viewpoint Considerations**:<br>- **Recognition of Increased Usage**: Liberals might appreciate the acknowledgment of increased cannabis use and the evolution of its potency, as this can lead to more informed public discussions and policies.<br>- **Legalization in States**: The mention of over 25 states legalizing marijuana to some degree aligns with liberal advocacy for decriminalization and legalization of cannabis.<br>- **Focus on Public Safety**: Emphasizing the need for public education and research into safe cannabis use, especially in relation to driving, aligns with a liberal focus on harm reduction and informed decision-making.<br><br>2. **Conservative Viewpoint Considerations**:<br>- **Highlighting Risks and Abuse**: The text's emphasis on cannabis as a commonly abused substance and its association with driving-related incidents may resonate with conservative concerns about drug abuse and public safety.<br>- **Challenges in Regulation and Enforcement**: Mentioning the difficulties in determining cannabis-related intoxication and the need for more effective legislation and testing methods aligns with conservative emphasis on law and order.<br>- **Federal Classification as a Controlled Substance**: The reference to cannabis's classification as a Schedule I Controlled Substance may align with conservative views favoring stricter drug regulations.<br><br>Overall, the text appears to take a more neutral, fact-based approach, highlighting both the prevalence and risks of cannabis use, as well as the challenges in regulating and managing its impact, especially in relation to driving. It doesn't strongly advocate for or against legalization but rather focuses on public health and safety considerations, which can be important to both liberal and conservative viewpoints.	neutral
ACS	Post-Traumatic Stress Disorder in Pediatric Trauma Patients	2/1/2018	The text you provided focuses on recognizing and addressing Post-Traumatic Stress Disorder (PTSD), particularly in children and adolescents, and does not align distinctly with either a liberal or conservative viewpoint in the traditional political sense. The content is largely factual and medical in nature, discussing the symptoms, epidemiology, and treatment of PTSD, especially in the context of pediatric trauma. It emphasizes the importance of screening, intervention, and research funding for PTSD, and acknowledges the impact of trauma on both children and their parents.<br><br>Political ideologies like liberalism and conservatism generally differ in their approaches to healthcare, social services, and government intervention. Liberals typically advocate for more government involvement in healthcare and social services, emphasizing the need for accessible and comprehensive care for all, including mental health services. Conservatives, on the other hand, might emphasize individual responsibility and private sector solutions, with less government intervention in healthcare.<br><br>However, the recognition and treatment of PTSD, as described in your text, is a healthcare issue that transcends political ideologies. Both liberal and conservative viewpoints can support the need for effective mental health care, especially for vulnerable populations like children affected by trauma. The focus here is more on public health and medical best practices rather than on a specific political ideology.	neutral

ACS	Post-Traumatic Stress Disorder in Adults	2/1/2018	The text you've provided appears to be a factual and informative statement regarding PTSD (Post-Traumatic Stress Disorder), its prevalence, and approaches to its management and research, rather than a statement aligned with a specific political viewpoint, either liberal or conservative. It focuses on medical facts and public health strategies, rather than political ideologies.<br><br>In general, topics related to healthcare, including mental health issues like PTSD, can be viewed through various lenses, including political ones. However, the approach to PTSD, as outlined in your text, emphasizes evidence-based practices, public health strategies, and research, which are typically nonpartisan issues. The emphasis on scientific evidence and practical solutions to health issues is a common ground that transcends political divisions.<br><br>While it's true that different political ideologies may have varying approaches to healthcare policy, the information provided in your text is centered on medical facts and best practices, which are typically supported across the political spectrum. The focus is on understanding and addressing a health issue, not on promoting a particular political stance.	neutral
ACS	Social Media	1/1/2018	The text you provided does not align strictly with either a liberal or conservative viewpoint. Instead, it focuses on ethical and professional guidelines for physicians using social media. The recommendations emphasize the importance of maintaining patient confidentiality, professionalism, and awareness of the lasting nature of online content. These guidelines are more about best practices in a professional context, specifically for healthcare professionals, rather than reflecting a particular political ideology. They address the use of social media in a way that is mindful of professional responsibilities and ethical standards, which is a concern that transcends political affiliations.	neutral
ACS	The Uniform Emergency Volunteer Health Practitioners Act	1/1/2018	The text provided does not distinctly align with a traditional liberal or conservative viewpoint. Rather, it focuses on practical and humanitarian concerns related to healthcare provision in emergency situations. The Uniform Emergency Volunteer Health Practitioners Act (UEVHPA) aims to address legal and licensing barriers that impede volunteer healthcare practitioners from providing care during declared emergencies, especially across state lines.<br><br>This approach is primarily concerned with improving disaster response efficiency and ensuring that qualified healthcare professionals, including surgeons, can offer their services where needed most, regardless of state licensure. The emphasis on facilitating cross-state medical assistance and enhancing disaster response capabilities is a pragmatic and non-partisan issue, focusing on public health and safety rather than ideological differences.<br><br>Both liberal and conservative viewpoints generally support measures that enhance public safety and health, especially in times of crisis. Thus, the support for the UEVHPA can be seen as a reflection of a broader, bipartisan consensus on the importance of effective disaster response and healthcare accessibility in emergency situations.	neutral
ACS	Diversity	1/1/2018	The text provided reflects a viewpoint that is generally associated with liberal ideologies. Key aspects of this viewpoint include:<br><br>1. **Emphasis on Diversity and Multiculturalism**: The text highlights a strong commitment to multiculturalism and diversity. Liberal viewpoints typically stress the importance of including people from diverse backgrounds and ensuring equal opportunities for all, regardless of their race, ethnicity, or other demographic characteristics.<br><br>2. **Active Promotion of Inclusion in Leadership**: The mention of ensuring that leadership positions are held by individuals from diverse groups aligns with a liberal perspective, which often advocates for representation and inclusivity in decision-making roles.<br><br>3. **Focus on Underrepresented Groups**: The acknowledgment of the need for specific recruitment and development of individuals from underrepresented groups is a hallmark of liberal approaches, which prioritize social justice and equity.<br><br>4. **Merit-based Nominations with an Inclusive Approach**: The combination of basing nominations on individual qualifications and the active effort to include diverse backgrounds is reflective of a liberal viewpoint that seeks to balance meritocracy with inclusivity.<br><br>5. **Development of Measures for Progress in Diversity and Inclusion**: The commitment to developing metrics for evaluating progress in diversity and inclusion initiatives is consistent with liberal ideologies, which often emphasize the need for measurable outcomes in social policies.<br><br>While these points align more closely with liberal ideologies, it's important to note that aspects of diversity and inclusion can also be valued across the political spectrum. The emphasis in this text, however, leans more towards a liberal perspective.	liberal

ACS	Lithium Batteries	10/4/2017	The text provided primarily presents factual information about the safety concerns and risks associated with lithium-ion batteries, as recognized by the American Chemical Society (ACS). It does not inherently align with a liberal or conservative viewpoint. The content focuses on:<br><br>1. The unique risks of lithium-ion batteries due to flammable organic solvents.<br>2. Causes of short-circuits in these batteries, such as manufacturing defects, overcharging, exposure to high voltage, physical damage, and mechanical failure.<br>3. The prevalence of incidents involving lithium-ion batteries in various consumer products.<br>4. Actions taken by the U.S. Consumer Product Safety Commission and the Federal Aviation Administration regarding the safety of these batteries.<br>5. Rising injuries related to e-cigarettes powered by lithium batteries.<br>6. The ACS's support for safer manufacturing processes, regulations for safe use and storage, and injury tracking to improve safety and prevention.<br><br>This text leans towards a safety and public health perspective, emphasizing the need for better manufacturing practices, regulatory oversight, and data collection to prevent accidents and injuries. These issues are generally of concern to both liberals and conservatives, though the specific policy approaches and regulatory measures might be viewed differently based on political ideologies. Liberals might focus more on regulatory measures and consumer safety, while conservatives might emphasize market solutions and innovation in battery technology. However, the text itself remains neutral and factual, without advocating for a specific political stance.	neutral
ACS	Opioids and Motor Vehicle Crash Prevention	10/4/2017	The text you provided presents a viewpoint that aligns more closely with a public health perspective than strictly with liberal or conservative political ideologies. It focuses on acknowledging the risks associated with opioid use, especially in relation to driving and the use of other psychoactive substances. This approach is primarily concerned with patient safety and public health, rather than being rooted in a particular political stance.<br><br>However, the emphasis on education, regulation, and legislation could be interpreted differently based on political leanings:<br><br>1. **Liberal Viewpoint**: Liberals might support this stance as it advocates for increased education and regulation, aspects often favored by liberal ideologies. They typically endorse more government involvement in health and safety matters, believing that such regulations can protect public welfare.<br><br>2. **Conservative Viewpoint**: Conservatives might have mixed reactions. While they generally support personal responsibility and might agree with educating patients about the risks of opioids, they often favor less government regulation. Thus, the call for new legislation and policies might be seen as excessive government intervention.<br><br>Overall, the statement seems more aligned with a non-partisan, public health perspective, emphasizing safety and education over political ideology.	probably liberal

ACS	The Rationale for Emergency Surgical Call	8/2/2017	The text provided reflects a blend of viewpoints that could align with both liberal and conservative perspectives, but it primarily centers around professional ethics in healthcare, particularly in emergency surgical care. It doesn't explicitly favor a political ideology but instead focuses on the challenges faced by surgeons and the healthcare system. Here's a breakdown:<br><br>1. **Liberal Viewpoint Aspects**:<br>- Emphasis on universal access to emergency surgical care, regardless of patients' socio-economic status.<br>- Recognition of the challenges faced by indigent patients and the aging population, suggesting a concern for vulnerable groups which is typically associated with liberal values.<br>- Support for government regulations (like EMTALA) that ensure patient care, which aligns with more regulatory and supportive roles of government often advocated by liberals.<br><br>2. **Conservative Viewpoint Aspects**:<br>- Concern over the fiscal viability and competitive nature of surgical practices, which aligns with conservative views emphasizing free market principles and financial sustainability in healthcare.<br>- The mention of practical considerations for surgeons in the face of increasing demands, which can be interpreted as a call for efficient management and resource utilization – concepts often emphasized in conservative thought.<br><br>3. **Professional Ethics and Practical Considerations**:<br>- The text heavily focuses on the ethical obligation of surgeons to provide care to all individuals, a viewpoint that transcends political ideologies.<br>- It discusses the practical challenges in providing emergency surgical care, such as the need for adequate compensation, resource allocation, and maintaining a work-life balance for surgeons.<br><br>In summary, while the text includes elements that could be interpreted as aligning with both liberal and conservative viewpoints, its primary focus is on the ethical and practical aspects of providing emergency surgical care, rather than on promoting a specific political ideology.	neutral
ACS	The Development and Use of Proprietary Guidelines for Accountable Patient-Centered Care	8/2/2017	The text you provided outlines the American College of Surgeons (ACS) stance on health care guidelines, patient care, physician roles, and government involvement in health care. It doesn't align strictly with either a liberal or conservative viewpoint as traditionally defined in American politics. Instead, it blends elements that could be appreciated by both sides:<br><br>1. **Patient-Centered Care**: The emphasis on patient health and well-being, patient freedom to choose physicians, and shared decision-making are principles that resonate across the political spectrum. This focus on individual choice and patient autonomy is a value often emphasized in conservative thought.<br><br>2. **Evidence-Based Practice**: The insistence on evidence-based guidelines and the reduction of unnecessary variation in care reflects a scientific and pragmatic approach to medicine. This emphasis on evidence and efficiency can appeal to both liberal and conservative viewpoints, though the methodical, data-driven approach is often more associated with liberal thinking.<br><br>3. **Physician Autonomy**: The emphasis on the physician's role in health care, including their involvement in creating and implementing guidelines, aligns with a more conservative view that values professional autonomy and expertise.<br><br>4. **Government Role**: The text acknowledges a role for government in health care, particularly in ensuring patient access to care and in shared decision-making. This recognition of a government role can be seen as aligning with more liberal perspectives, which often advocate for government involvement in ensuring healthcare access. However, it also emphasizes that government guidelines should follow nationally accepted, evidence-based standards and not interfere with physician responsibility, which can resonate with conservative views favoring limited government interference in professional practice.<br><br>In summary, the ACS's position blends elements of both liberal and conservative viewpoints, focusing on patient autonomy, evidence-based practice, physician involvement, and a balanced role for government in health care. This approach seems to strive for a pragmatic balance that prioritizes patient care and physician autonomy, within a framework that acknowledges the role of government in supporting health care access and standards.	neutral

ACS	Gender Salary Equity	8/2/2017	The text you've provided seems to align more with a liberal viewpoint. This perspective is characterized by a focus on addressing systemic inequalities and promoting policies that foster fairness and inclusivity. The emphasis on gender pay equity, transparency in salary determination, and the provision of training to address implicit bias are hallmarks of a more progressive or liberal approach.<br><br>Liberal viewpoints generally advocate for active measures to ensure equal opportunities and outcomes across different groups, in this case, addressing gender disparities in the medical field. The suggestions for routine reviews of performance and compensation, as well as educational programs to empower individuals to negotiate equitable salaries, reflect a commitment to systemic change and equal treatment, which are key aspects of liberal ideology.<br><br>In contrast, a conservative viewpoint might focus more on individual responsibility and merit-based systems, potentially arguing that market forces should dictate salaries without as much direct intervention or regulation. Conservatives might emphasize personal initiative and negotiation skills as the primary tools for addressing pay disparities, rather than systemic reforms or oversight.	liberal
ACS	The Opioid Abuse Epidemic	8/2/2017	The text provided does not align strictly with either a liberal or conservative viewpoint. It presents a balanced and practical approach to a public health issue, focusing on the responsible use of opioids and the prevention of abuse. The text acknowledges the need for pain management in certain patient populations while also recognizing the risks associated with opioid use and the importance of monitoring and regulating prescriptions. The emphasis on evidence-based education, patient safety, and the improvement of prescription drug monitoring programs (PDMPs) reflects a comprehensive and multi-faceted approach that transcends traditional political lines. The strategies suggested, such as enhancing PDMPs, providing training for safe prescribing practices, and addressing the specific needs of military veterans, indicate a focus on practical solutions rather than ideological positions.	neutral
ACS	Health Care Reform	5/1/2017	The text from the American College of Surgeons (ACS) mission statement and policy positions reflects a mix of viewpoints that do not squarely align with traditional liberal or conservative ideologies. Instead, it embodies a professional and pragmatic approach focused on improving healthcare quality, patient safety, and accessibility. Key points include:<br><br>1. **Emphasis on Quality and Safety**: The ACS prioritizes quality and safety in surgical care, advocating for clinical registries and educational efforts to drive improvements. This focus on evidence-based care and continuous improvement is a common theme in healthcare policy, not specific to any political ideology.<br><br>2. **Universal Access to Healthcare**: The organization's support for universal access to patient-centered, timely, affordable, and appropriate healthcare aligns more closely with liberal viewpoints, which generally advocate for broader healthcare access.<br><br>3. **Cost Reduction and Medical Liability Reform**: Their stance on reducing healthcare costs and reforming medical liability, including support for measures like the Medical Injury Compensation Reform Act (MICRA), may resonate more with conservative perspectives, which often emphasize cost control and reforming malpractice laws to reduce unnecessary expenditures.<br><br>4. **Professional Autonomy and Education**: ACS's emphasis on maintaining professional standards, the importance of surgical education, and the role of surgeons suggests a focus on professional autonomy and high standards of practice. This is a viewpoint generally shared across the healthcare profession, irrespective of political leanings.<br><br>5. **Support for Specific Healthcare Programs and Policies**: The ACS's positions on issues like Medicaid expansion, children's health insurance, and physician ownership rights do not neatly fit into a single political ideology but reflect a nuanced approach considering various aspects of healthcare system improvement.<br><br>6. **Advocacy for Evidence-Based Guidelines and Performance Measurement**: Their support for evidence-based guidelines and performance measurement in healthcare aligns with a data-driven, outcome-focused approach that transcends political ideologies.<br><br>Overall, the ACS's mission and policy positions represent a blend of ideals focusing on professional standards, patient care quality, healthcare accessibility, and system sustainability. The mix of these elements doesn't neatly categorize into a traditional liberal or conservative viewpoint but rather shows a commitment to advancing the field of surgery and healthcare in a holistic manner.	neutral

ACS	The use of general anesthetics and sedation drugs in children and pregnant women	4/1/2017	The text you provided presents a nuanced perspective that doesn't align strictly with either a liberal or conservative viewpoint. Instead, it focuses on medical and ethical considerations regarding the use of general anesthesia in young children and pregnant women. Key points include:<br><br>1. **FDA Warning on Anesthesia Risks**: The U.S. Food and Drug Administration (FDA) issued a warning about potential neurological risks associated with the use of general anesthesia in children under three years of age and pregnant women in their final trimester. This warning is based on animal studies, with unclear applicability to humans.<br><br>2. **Lack of Conclusive Human Data**: Despite years of study, there is no definitive human data supporting the conclusion that anesthesia poses these risks. Some epidemiological studies suggest a potential association between anesthesia exposure and adverse neurodevelopmental outcomes, but it's unclear if these are due to anesthesia, the surgical procedure, the patient's medical condition, or other factors.<br><br>3. **Surgical Decision-making and Informed Consent**: The American College of Surgeons (ACS) emphasizes the importance of informed consent discussions, especially in urgent or emergent procedures for children. Surgeons prioritize a child's health and well-being and consider the necessity of surgery and anesthesia despite potential risks.<br><br>4. **Different Perspective for Elective Procedures**: For elective procedures, the ACS advises that the FDA's communication should be discussed with patients and their families. Timing of surgery can be critical, and delaying surgery can sometimes adversely affect outcomes.<br><br>5. **Pediatric Surgeons' Practices**: Pediatric surgeons have historically led practice changes to safely delay or avoid surgery and general anesthesia when possible. There's an ongoing professional debate about delaying elective surgical procedures due to potential anesthesia risks.<br><br>6. **Shared Decision-making and Balancing Risks**: The ACS recommends informed discussions based on current evidence about the potential adverse effects of anesthesia on young children. However, it also stresses the importance of shared decision-making that considers the consequences of delaying time-sensitive operations.<br><br>This text reflects a medically-informed perspective prioritizing patient safety, informed consent, and the careful consideration of risks versus benefits in surgical decision-making. It transcends political categorization, focusing instead on healthcare ethics and evidence-based medicine.	neutral
ACS	Sharps Safety	10/1/2016	The text you provided does not align with either a liberal or conservative viewpoint in the traditional political sense. It is a technical and factual discussion focused on healthcare practices, specifically addressing the prevention of sharps injuries and glove tears in surgical settings. This type of content is centered on medical safety and occupational health, rather than political ideology.<br><br>It discusses various measures and recommendations for reducing the risk of infection due to needlestick injuries and glove perforations in surgical environments. The text cites studies and recommendations from authoritative medical bodies like the American College of Surgeons (ACS) and mentions specific techniques like double gloving, using blunt-tip suture needles, and hands-free techniques. The focus is on improving safety protocols in healthcare settings to protect both healthcare workers and patients from infection risks.<br><br>Such discussions are generally rooted in scientific research and clinical best practices, and they typically transcend political affiliations or ideologies. The primary concern here is the health and safety of medical professionals and patients, which is a universal concern across all political spectrums.	neutral
ACS	Statement on Documentation and Reporting of Accidental Punctures and Lacerations During Surgery	10/1/2016	The text you've provided is primarily technical and procedural, focusing on guidelines for documenting and reporting accidental punctures and lacerations in medical settings. It discusses the Agency for Healthcare Research and Quality (AHRQ) patient safety indicator (PSI) and emphasizes the importance of accurate coding and documentation in surgical procedures.<br><br>This text does not inherently align with a liberal or conservative viewpoint. It is a factual, medical guideline aimed at improving patient safety and the accuracy of medical records. Such technical and professional guidelines generally transcend political leanings and are focused on practical, clinical outcomes rather than ideological positions.<br><br>Political viewpoints, liberal or conservative, typically involve opinions on governance, social issues, economic policies, and similar	neutral

			matters. In contrast, the text you've provided is centered on healthcare quality measures and best practices, which are areas more related to medical professionalism and standards than to political ideology.	
ACS	The Prevention of Unintentionally Retained Surgical Items After Surgery	10/1/2016	The text you've provided outlines a series of recommendations and guidelines for surgical procedures, focusing primarily on patient safety and the prevention of unintended retention of surgical items. This content is primarily medical and procedural in nature, and does not inherently align with a specific political viewpoint, whether liberal or conservative.<br><br>The focus here is on best practices in a medical setting, emphasizing the importance of safety, communication, standardized processes, and technology in surgical procedures. These recommendations are aimed at improving patient outcomes and ensuring the safety of surgical processes, which is a universal concern in healthcare regardless of political affiliation.<br><br>Political viewpoints typically involve interpretations and opinions on social, economic, and political issues. In contrast, the text you've provided is centered on medical practices and safety protocols, which are generally guided by clinical evidence and professional standards rather than political ideology.	neutral
ACS	Health Care Industry Representatives in the Operating Room	10/1/2016	The text provided doesn't align strictly with either a liberal or conservative viewpoint as it primarily focuses on technical and procedural guidelines within a healthcare setting. The content is centered around the introduction of policies and practices for Health Care Industry Representatives (HCIRs) in the operating room and perioperative environment. It emphasizes the need for structured systems for education, training, and the introduction of new procedures, technologies, and equipment.<br><br>This type of text is more oriented towards healthcare management and policy, rather than political ideology. It discusses standards for patient safety, privacy, and confidentiality, as well as the roles and limitations of HCIRs in surgical settings. These guidelines are aimed at ensuring optimal surgical outcomes and maintaining high standards of care, which are universal concerns in the healthcare industry regardless of political affiliation.<br><br>The focus on patient safety, effective use of technology, and adherence to legal and ethical standards transcends typical liberal-conservative divides, fitting more into healthcare policy and administration.	neutral
ACS	Surgical Technology Training and Certification	10/1/2016	The text you provided is primarily factual and educational in nature, focusing on the roles, education, and certification of surgical technologists. It does not inherently align with either a liberal or conservative viewpoint. The content is centered on professional standards and practices in the field of surgical technology, detailing the accreditation of educational programs and the certification of graduates. Such topics typically fall outside the usual spectrum of political ideologies and are more aligned with professional and educational standards in healthcare.	neutral
ACS	Safe Surgery Checklists, and Ensuring Correct Patient, Correct Site, and Correct Procedure Surgery	10/1/2016	The text provided focuses on medical safety protocols and guidelines recommended by the American College of Surgeons (ACS) to prevent wrong site surgeries. These guidelines emphasize patient safety, verification of procedures, consent, and coordination among surgical teams.<br><br>This text does not inherently align with a liberal or conservative viewpoint. It is a professional and technical document aimed at improving patient care and safety in a medical context. The emphasis is on practical, procedural measures rather than ideological or political considerations.<br><br>In general, issues of patient safety and medical best practices tend to be non-partisan and are supported across the political spectrum. The focus in such texts is usually on evidence-based medicine, clinical outcomes, and patient welfare, rather than on political ideologies.	neutral

ACS	Distractions in the Operating Room	9/1/2016	The text provided doesn't inherently align with a specific liberal or conservative viewpoint. It's primarily focused on addressing practical concerns related to distractions in the operating room (OR), particularly regarding the use of technology and noise levels. These concerns are related to patient safety, efficient healthcare delivery, and the optimal performance of medical professionals, issues that transcend political ideologies.<br><br>The text discusses the importance of mitigating distractions from various sources, both intrinsic (like alarms and noise from surgical devices) and extrinsic (such as cell phones and other electronic devices). It also touches upon the adoption of structured communication protocols and the concept of the "sterile cockpit" to limit distractions during critical periods in the OR. These points are grounded in operational efficiency and patient safety, which are universal concerns in healthcare regardless of political affiliation.<br><br>In summary, the text is more aligned with healthcare management and patient safety perspectives rather than being indicative of a specific political ideology.	neutral
ACS	Statement on General Helmet Use	9/1/2016	The text you've provided focuses primarily on safety and health concerns related to helmet use in various activities. It does not explicitly align with either a liberal or conservative viewpoint as it is centered around scientific findings and health recommendations rather than political ideology.<br><br>Issues like helmet safety and the promotion of their use are typically considered matters of public health and safety, rather than partisan political issues. The statements emphasize the importance of research, evidence-based policy, and the adaptation of safety measures to specific activities and demographics, which are approaches generally favored in public health discourse.<br><br>While it's possible that discussions around mandatory helmet laws could intersect with political viewpoints (with some conservatives potentially viewing them as overreach of government regulation and liberals potentially supporting them as necessary for public safety), the text you've provided does not delve into these aspects and remains neutral, focusing on the scientific and health perspectives of helmet use.	neutral
ACS	Physician-Led Team-Based Surgical Care	8/1/2016	The text you provided outlines principles and practices for perioperative care, focusing on patient-centered, efficient, safe, and high-quality medical care. It emphasizes the importance of multidisciplinary teams, patient involvement, evidence-based care, and effective coordination among healthcare providers.<br><br>This text does not neatly align with a specific political viewpoint, such as liberal or conservative, in the traditional sense. Instead, it focuses on healthcare practices and principles that aim to improve patient outcomes and efficiency in medical care. The emphasis on evidence-based care, multidisciplinary teams, and patient involvement is consistent with general principles of modern healthcare, which transcend specific political ideologies.<br><br>In healthcare discussions, liberal and conservative viewpoints often differ on issues like healthcare funding, the role of government in healthcare, and healthcare policy. However, the principles of efficient, patient-centered care, as described in your text, are generally accepted across the spectrum as desirable goals in healthcare.<br><br>Thus, the text is more about best practices in healthcare rather than being aligned with a specific political ideology.	neutral
ACS	The Principles of Patient Education	5/2/2016	The text you provided focuses on the importance of patient education and involvement in surgical care, emphasizing the need for patient-centered decision-making and access to educational materials. This perspective aligns more closely with a liberal viewpoint in its emphasis on patient rights, inclusivity (taking into account factors like culture, gender, and health literacy), and collaborative decision-making.<br><br>Liberal viewpoints often prioritize individual rights, inclusivity, and comprehensive access to resources and information. The text's focus on empowering patients to participate actively in their healthcare decisions reflects these principles. It highlights the importance of understanding diverse patient needs and ensuring that patients are well-informed and involved in their healthcare.<br><br>However, it's important to note that the promotion of patient rights and the emphasis on education and inclusivity in healthcare are not exclusively liberal ideas. These concepts can be supported across the political spectrum as part of a broader commitment to high-quality healthcare. The text does not explicitly align itself with a specific political ideology but instead focuses on best practices in patient care and medical education.	liberal

ACS	The Aging Surgeon	1/1/2016	The text you've provided seems to be a policy statement from the American College of Surgeons (ACS) regarding aging surgeons. It addresses the challenges and recommendations concerning the competency and performance of surgeons as they age. This statement does not align strictly with a liberal or conservative viewpoint as understood in a political context. Instead, it focuses on professional standards, patient safety, and the well-being of surgeons, which are concerns that transcend political ideologies.<br><br>The statement highlights the importance of wellness, acknowledges the variability of age-related decline, and suggests voluntary assessments rather than mandatory retirement ages. These points suggest a balanced approach, emphasizing individual assessment and responsibility over broad regulatory measures. This kind of approach can be appreciated across the political spectrum, as it combines elements of personal autonomy (often emphasized in conservative viewpoints) with a commitment to community welfare and safety (common in liberal viewpoints).<br><br>In summary, the statement appears to be more aligned with professional and ethical considerations within the medical field rather than with a specific political ideology.	neutral
ACS	Prevention of Non-Traffic Vehicle-Related Injuries in Children	1/1/2016	The text you've provided appears to be primarily factual and informative, focusing on the issue of child safety around motor vehicles. It presents data and statistics about injuries and deaths of children in non-traffic vehicle-related incidents and suggests certain prevention activities and legislative measures.<br><br>The content of this text doesn't align explicitly with a traditional liberal or conservative viewpoint as understood in the context of U.S. politics. Issues of child safety and public health, like the ones discussed here, are generally of concern across the political spectrum. Both liberal and conservative viewpoints might support the prevention of child injuries, although they might differ in their approaches to legislation and regulation.<br><br>- A more liberal viewpoint might focus on the need for government intervention and regulation, such as supporting legislation to impose fines or mandate safety devices in vehicles.<br>- A conservative viewpoint might emphasize personal responsibility and education of parents and caregivers, possibly resisting increased government regulation.<br><br>However, the text itself is primarily presenting data and suggesting safety measures, without advocating for a specific political approach to the issue. It's important to note that issues like child safety can transcend traditional political boundaries, with agreement possible from multiple perspectives on the importance of addressing the problem.	neutral
ACS	Safety Belt Laws and Enforcement	1/1/2016	The text provided appears to be a factual statement regarding motor vehicle crashes, injuries, deaths, and the effectiveness of safety belts, rather than aligning explicitly with either a liberal or conservative viewpoint. It presents statistical data and findings on the impact of safety belt laws and their effectiveness in reducing fatalities and injuries. This kind of information is generally considered objective and evidence-based, focusing on public health and safety rather than political ideology.<br><br>However, the support for strong legislation and effective enforcement of primary safety belt laws could be interpreted differently depending on one's political viewpoint:<br><br>1. **Liberal Viewpoint**: Those with a liberal perspective might agree with this statement as it advocates for government intervention and regulation to ensure public safety. Liberals often support the role of government in protecting citizens, which in this case involves enacting and enforcing safety belt laws.<br><br>2. **Conservative Viewpoint**: On the other hand, conservatives might be wary of increased government regulation and enforcement, preferring personal responsibility and freedom of choice. However, conservatives also value safety and the rule of law, so there could be conservative support for safety belt laws based on their proven effectiveness in saving lives.<br><br>In summary, while the text is primarily factual and centered on public health, its implications can be interpreted through different ideological lenses.	neutral

ACS	Older Adult Burn Prevention	1/1/2016	The text you've provided appears to be primarily factual and informational, focusing on the risks and prevention of burns in elderly populations. It outlines various physiological and environmental factors that increase the risk of burns and suggests preventive measures. This content does not inherently align with a specific liberal or conservative viewpoint, as it is centered on medical and health-related information rather than political ideology.<br><br>Liberal and conservative viewpoints typically differ in their approaches to policy, economics, social issues, and governance, but the information in your text is more aligned with public health and safety, which are generally non-partisan issues. Both liberal and conservative platforms can support public health initiatives, although their methods of implementation or emphasis on certain aspects might vary.<br><br>In this case, the focus on education, prevention, and awareness in geriatric care is a universal concern that transcends political divisions, aiming to improve health outcomes for a vulnerable population. It's important to note that while health policies can be influenced by political beliefs, the underlying medical facts and safety recommendations are typically based on scientific research and clinical experience, not political ideology.	neutral
ACS	Support of Motorcycle Helmet Laws	5/1/2015	The text you've shared aligns more closely with a public health and safety perspective rather than being strictly liberal or conservative in a political sense. It emphasizes the importance of safety measures (in this case, the use of helmets by motorcyclists) to prevent injuries and fatalities. This viewpoint is grounded in empirical data and public health research.<br><br>In terms of political alignment, different people might interpret it differently based on their ideological leanings:<br><br>1. **Liberal Viewpoint**: Liberals might support this stance as it aligns with a broader commitment to public health and safety regulations. They often advocate for government intervention and policies aimed at protecting individual well-being and public health.<br><br>2. **Conservative Viewpoint**: Conservatives might have mixed views. Some conservatives might support helmet laws as a measure to reduce healthcare costs and protect individual safety. However, others might oppose such regulations, viewing them as an overreach of government authority and an infringement on personal freedom.<br><br>It's important to note that the interpretation of such policies can vary widely even within these broad political categories, and the support or opposition to such measures might be influenced by factors beyond mere political ideology, such as cultural values, personal experiences, and specific contexts.	neutral
ACS	Physician Tiering and Narrow Network Programs	5/1/2015	The text provided presents a viewpoint that emphasizes the importance of quality in healthcare, particularly in the context of physician tiering and narrow network programs. It advocates for healthcare protocols and programs that are based primarily on quality, not just cost, and calls for transparency, reliability, and validity in the methods used to evaluate healthcare providers. This perspective aligns more closely with a liberal viewpoint in the following ways:<br><br>1. **Emphasis on Quality Over Cost**: The text prioritizes quality of care over cost reduction, which is a common stance in liberal ideologies. Liberals often advocate for policies that focus on the quality and accessibility of services, rather than just efficiency or cost-cutting measures.<br><br>2. **Call for Government and Organizational Oversight**: The suggestion that government agencies, hospitals, and other organizations develop policies to ensure high-quality care aligns with a liberal perspective, which typically supports the role of government and regulatory bodies in overseeing and ensuring the quality of healthcare services.<br><br>3. **Support for Transparency and Accountability**: The text's call for transparent methods and standards, as well as involvement of physicians and stakeholders in the development of healthcare protocols, reflects a liberal viewpoint that values openness, accountability, and participatory decision-making in public services.<br><br>4. **Concern for Patient Access and Equity**: The emphasis on ensuring ongoing access for higher-risk patients and adjusting for socioeconomic status indicates a concern for equity in healthcare, a key principle in liberal thought.<br><br>5. **Opposition to Cost-Only Measures**: The explicit rejection of cost as the sole metric for healthcare decision-making aligns with liberal values, which often prioritize the well-being and needs of individuals over purely economic considerations.	liberal

			It's important to note that these interpretations can vary, and different individuals or groups might see different aspects of this text aligning with their own views, whether liberal, conservative, or otherwise. The assessment here is based on the general principles typically associated with liberal ideologies in the context of healthcare policy.	
ACS	Medical Liability Reform	3/1/2015	The text you provided appears to discuss issues surrounding the medical liability system, patient safety, quality of healthcare, and various reforms. It doesn't explicitly align with a traditionally liberal or conservative viewpoint. Instead, it focuses on specific problems and potential solutions within the healthcare system.<br><br>- **Nonpartisan Focus on Systemic Issues**: The text highlights systemic issues in the medical liability system, such as inefficiencies and the high cost, which is a concern irrespective of political leanings.<br><br>- **Reform Oriented**: The emphasis on reforming the medical liability system, including alternative solutions like health courts, enterprise liability, and alternative dispute resolution, doesn't necessarily align with a specific political ideology. Both liberals and conservatives may agree on the need for reform, although they might differ on the specifics.<br><br>- **Patient-Centered Approach**: The focus on patient safety, quality healthcare, and provider accountability is a universal concern that transcends traditional political boundaries.<br><br>- **Economic Concerns**: The mention of the financial impact and cost (e.g., $55.6 billion annually) could resonate with fiscally conservative viewpoints that emphasize cost-efficiency. However, the emphasis on patient welfare and systemic improvement can also align with liberal values.<br><br>In summary, the text discusses healthcare and legal reform in a way that doesn't neatly fit into a liberal or conservative box. It seems to prioritize practical solutions and systemic improvement over ideological positions.	neutral

ACS	The Effects of Tobacco Use on Surgical Complications and the Utility of Smoking Cessation Counseling	8/1/2014	The text provided primarily presents factual information and statistics about the health impacts of smoking, the benefits of smoking cessation, especially in the context of surgical procedures, and the role surgeons can play in smoking cessation counseling. It also highlights the economic costs associated with smoking-related diseases and the low rates of smoking cessation counseling by surgeons.<br><br>This text does not explicitly align with a strictly liberal or conservative viewpoint. Instead, it focuses on public health concerns, medical facts, and the importance of medical intervention in smoking cessation. Such topics can be of interest and concern to individuals across the political spectrum.<br><br>Both liberal and conservative viewpoints might converge on the importance of addressing public health issues like smoking, though they may differ in their approaches to regulation, healthcare policy, and the role of government in public health initiatives. The text's emphasis on the need for more robust smoking cessation counseling and support for government regulation of tobacco products might be more aligned with a public health perspective, which is often associated with liberal views on healthcare. However, the focus on the economic burden and the potential for improving health outcomes through individual behavior change can resonate with conservative values as well.<br><br>In summary, the text seems to take a neutral stance, focused more on health and economic aspects of smoking rather than advocating a specific political ideology.	neutral
ACS	Peak Performance and Management of Fatigue	8/1/2014	The text you provided doesn't align explicitly with either a liberal or conservative viewpoint as it primarily focuses on professional standards and practices in the field of surgery. It emphasizes the importance of recognizing and mitigating fatigue in surgical practice, the unique aspects of surgical proficiency, the significance of teamwork, and the role of the surgeon in patient care.<br><br>The content is more centered around medical professionalism, patient care, and the operational aspects of surgical practice rather than political ideology. It discusses the steps taken by the American College of Surgeons (ACS) to enhance surgical performance through the understanding and management of fatigue, which is a subject of universal concern in healthcare irrespective of political leanings. The establishment of the Committee to Enhance Peak Performance in Surgery through Recognition and Mitigation of Fatigue demonstrates a commitment to improving medical practice based on scientific understanding and expert consensus, rather than on a political agenda.	neutral
ACS	Advance Directives by Patients: "Do Not Resuscitate" in the Operating Room	1/3/2014	The text you provided doesn't align specifically with either a liberal or conservative viewpoint as it is primarily focused on medical ethics and patient care practices, particularly in the context of surgical procedures for patients with "Do Not Resuscitate" (DNR) orders.<br><br>This text discusses the importance of respecting patients' wishes and rights to self-determination in medical decision-making, especially when they have a DNR order and are undergoing surgery. It emphasizes the need for surgeons and medical staff to engage in meaningful discussions with patients or their surrogates about the risks and benefits of surgical procedures, the potential impact on their DNR status, and the alignment of medical care with the patient's values and preferences.<br><br>The concepts of patient autonomy, informed consent, and ethical decision-making in healthcare are central themes in this text, which are broadly relevant across the political spectrum. They are core principles in medical ethics rather than issues that typically divide along partisan lines.<br><br>In summary, the text is more about medical ethics and patient-centered care than about political ideology.	neutral
ACS	Optimal Access	4/1/2013	The text provided does not explicitly align with either a liberal or conservative viewpoint. Instead, it focuses on factual and healthcare-related issues, emphasizing the importance of access to surgical care and its impact on patient outcomes. It addresses various factors that affect access to surgical care, such as socioeconomic status, age, gender, education, race, ethnicity, healthcare availability, geographic distance, and insurance status. The emphasis is on the equality of access to care and its role in reducing disparities in surgical outcomes.<br><br>Political ideologies like liberalism or conservatism might interpret and use these facts differently, but the text itself is neutral and focused on presenting healthcare issues and advocating for equal access to care. Liberals might emphasize the need for systemic changes to ensure equal access and address disparities, while conservatives might focus on individual responsibility and market-based solutions. However, these interpretations are external to the text itself, which remains factual and neutral in its presentation.	neutral

ACS	Firearm Injuries	1/16/2013	The text you've provided aligns more closely with a liberal viewpoint. This alignment is based on the specific policies and approaches it advocates for in relation to gun control and firearm regulation. Key points that align with liberal perspectives include:<br><br>1. **Support for Legislation Restricting Firearm Access**: Advocating for bans on assault weapons, large ammunition clips, and certain munitions, which is a common stance in liberal policies focused on reducing gun violence.<br><br>2. **Enhanced Background Checks**: The call for more comprehensive background checks, including at gun shows and auctions, is another hallmark of liberal approaches to gun control, aiming to restrict access to firearms for individuals who may pose a risk.<br><br>3. **Role of Health Care Professionals**: Emphasizing the role of health care professionals in preventing firearm injuries, including through patient counseling and mental health referrals, aligns with a more holistic, public health-oriented approach often found in liberal viewpoints.<br><br>4. **Safe Gun Storage and Non-Violent Conflict Resolution**: Promoting safe gun storage practices and non-violent conflict resolution education reflects a preventive approach to gun violence, typical of liberal perspectives.<br><br>5. **Research and Data Collection on Firearm Injuries**: Advocating for evidence-based research and the creation of a national firearm injury database to inform policy is consistent with a liberal emphasis on data-driven decision-making in public policy.<br><br>It's important to note that political viewpoints can be complex and multifaceted, and while these points align with general liberal viewpoints, there can be a range of opinions and beliefs within any political orientation.	liberal
ACS	Concussion and Brain Injury	12/4/2012	The statement from the American College of Surgeons (ACS) Committee on Trauma (COT) regarding concussion and brain injury prevention in athletes does not align strictly with either a liberal or conservative viewpoint. Instead, it reflects a medical and public health perspective focused on injury prevention and safety in sports.<br><br>The statement emphasizes the importance of legislative efforts to improve care for injured athletes, promote head protection, educate relevant personnel about concussion recognition and management, and implement policies for the safe participation of athletes in sports. These recommendations are grounded in medical evidence and concern for athlete safety rather than being aligned with a particular political ideology.<br><br>Health and safety issues, such as those addressed in this statement, often transcend traditional political boundaries. While there may be variations in how different political groups approach the implementation of such recommendations (for example, through legislation or private sector initiatives), the underlying concern for athlete health and safety is a generally nonpartisan issue.	neutral
ACS	Joint Statement by the ACS and SAGES on FLS Completion for General Surgeons Who Perform Laparoscopy	3/7/2012	The text you've provided is primarily informative and educational, focusing on the Fundamentals of Laparoscopic Surgery™ Program (FLS) and its role in surgical education and certification. It does not inherently align with a liberal or conservative viewpoint. The content is centered around medical education, surgical skills, and professional standards in healthcare, particularly laparoscopic surgery.<br><br>Discussions on educational standards, certification requirements, and professional competency in medicine are typically neutral and based on scientific and educational principles. They are generally apart from political ideologies unless they explicitly intersect with policy debates or sociopolitical issues, which is not the case in the text provided.	neutral
ACS	The Physician Acting as an Expert Witness	4/1/2011	The text you've provided doesn't inherently align with a specific liberal or conservative viewpoint. It is primarily a professional and ethical guideline issued by the American College of Surgeons regarding the role and responsibilities of physicians who act as expert witnesses in malpractice litigation. These guidelines emphasize the importance of qualifications, impartiality, honesty, and ethical conduct in providing expert testimony.<br><br>The focus on maintaining high professional standards, ethical behavior, and a commitment to truth and impartiality in the legal process is a matter of professional ethics rather than political ideology. Both liberal and conservative viewpoints generally support the principles of professional integrity and ethical conduct in legal proceedings, although the specific details or applications of these principles might be viewed differently across the political spectrum.<br><br>In summary, this text is more about professional conduct within the medical and legal fields than about political ideology.	neutral

ACS	Revised Recommendations for Use of Real-Time Ultrasound Guidance for Placement of Central Venous Catheters	2/1/2011	The text you've provided is focused on medical practices and evidence-based recommendations for the placement of central venous catheters (CVCs). It discusses the use of ultrasound guidance in CVC placement, citing studies, guidelines, and recommendations from healthcare organizations.<br><br>This text aligns neither with a liberal nor a conservative viewpoint in the political sense. It is a medical and scientific discourse, presenting data and recommendations based on clinical studies and expert opinions. The focus is on improving patient care and safety in medical procedures, which is a matter of healthcare practice and research rather than political ideology.<br><br>In general, discussions about medical procedures and best practices, especially those based on research and clinical trials, are not typically categorized along political lines, as they aim to provide objective, evidence-based information to improve healthcare outcomes.	neutral
ACS	Health Care Disparities	11/16/2010	The text you've provided doesn't align strictly with either a liberal or conservative viewpoint; rather, it focuses on healthcare disparities, particularly in surgical care, and suggests a more universal concern about equitable access to healthcare. It discusses the disparities in healthcare outcomes among different racial and ethnic groups, emphasizing the need for improvements in access to high-quality care and specialists.<br><br>While some aspects of the text might resonate more with liberal ideologies, such as the emphasis on systemic inequalities and the call for broad-based interventions to address these disparities, the overall tone and content aim to present a factual and research-based perspective on healthcare disparities without overt political bias. The text highlights the importance of addressing these disparities as a means of improving the overall quality of healthcare and advocates for policy changes that are inclusive and equitable. The focus on ethical and moral considerations in medical practice transcends typical political categorizations.	probably liberal
ACS	High-Performance Teams	2/1/2010	The text you've provided doesn't align distinctly with either a liberal or conservative viewpoint as typically defined in the context of American politics. It focuses on healthcare, emphasizing the importance of teamwork, effective leadership, respectful behavior, and structured communication in achieving the best patient outcomes. These principles are generally considered best practices in medical and organizational contexts, rather than being associated with a specific political ideology.<br><br>The emphasis on collaboration, respect, and structured systems for improving patient care transcends traditional political divides. Both liberal and conservative ideologies in the U.S. tend to agree on the importance of effective healthcare systems, although they may differ in their approaches to healthcare policy and funding. This text, however, is apolitical and concentrates on the operational and interpersonal aspects of healthcare delivery, which are universally acknowledged as crucial for successful patient outcomes in the medical field.	neutral
ACS	Medical and Surgical Tourism	4/1/2019	The text provided does not align explicitly with either a liberal or conservative viewpoint. Instead, it presents a factual and comprehensive overview of medical tourism, discussing both the reasons why people might seek medical care outside the U.S. and the potential risks and considerations involved. The stance of the American College of Surgeons (ACS) as outlined in the text is focused on patient safety, quality of care, informed decision-making, and the avoidance of mandatory referrals by insurers without patient consent. This approach is more centered on medical ethics and patient rights rather than political ideology.<br><br>In general, liberal and conservative viewpoints often differ in their approach to healthcare policy, with liberals typically advocating for broader access to healthcare and government involvement in ensuring this access, and conservatives often supporting more market-based solutions and less government intervention. However, the issue of medical tourism, as discussed in this text, is more nuanced and does not neatly fit into a liberal-conservative spectrum. It focuses more on the practicalities and ethics of seeking medical care internationally rather than on broader healthcare policy debates.	neutral
ACS	Guidelines for Collaboration of Industry and Surgical Organizations in Support of Continuing Education	2/1/2009	The text you provided seems to be primarily focused on establishing ethical guidelines for the collaboration between surgical organizations and the medical industry, particularly regarding the development and implementation of Continuing Medical Education (CME) programs. It emphasizes the importance of maintaining impartiality and autonomy in surgical organizations, managing conflicts of interest, and ensuring that commercial support does not influence the content or delivery of CME activities.<br><br>This text does not align clearly with a liberal or conservative viewpoint as understood in the typical political sense. Instead, it addresses professional and ethical standards within the medical field, which are concerns that transcend political ideologies. The focus here is on maintaining ethical standards, transparency, and the integrity of medical education and patient care, which are priorities that can be shared across the political spectrum.<br><br>Political ideologies, like liberalism and conservatism, often involve perspectives on government intervention, individual rights, economic policies, and social issues. This text, however, is more concerned with professional conduct and ethical considerations in	neutral

			the medical and educational aspects of the healthcare industry, making it more of a professional guideline than a politically charged statement.	
ACS	All-Terrain Vehicle Injuries	2/1/2009	The statement from the American College of Surgeons' Committee on Trauma regarding all-terrain vehicle (ATV) injuries and prevention doesn't neatly align with a specific political viewpoint such as liberal or conservative. Instead, it focuses on public health and safety concerns, advocating for legislative and manufacturing efforts to improve ATV safety.<br><br>The key points in the statement, such as advocating for safety design, rider training and licensure, and special consideration for children under 16, are primarily concerned with reducing injury and death rates associated with ATV use. These concerns transcend typical liberal or conservative ideologies and are more aligned with public health and safety advocacy.<br><br>In the broader political landscape, both liberal and conservative viewpoints can support such measures, albeit for different reasons. Liberals might emphasize the role of government in ensuring public safety and protecting children, while conservatives might focus on the importance of responsible use of vehicles and the role of personal responsibility in training and licensure. However, these are generalizations and do not necessarily apply to all individuals or groups within these ideological categories. The emphasis here is on practical, evidence-based approaches to reduce harm, which can be a point of agreement across different political perspectives.	neutral
ACS	The Surgical Workforce	8/1/2007	This text primarily presents a perspective focused on healthcare policy and workforce challenges, rather than aligning distinctly with either liberal or conservative viewpoints. It addresses the concerns of the American College of Surgeons about the accessibility of surgical care in the United States, highlighting issues such as aging population, geographical disparities in liability climate, technological changes, subspecialization trends, and the limited growth in the number of surgeons. These points are generally apolitical and pertain to the healthcare system's functionality and resource allocation.<br><br>However, the text does touch upon topics that can be interpreted through different political lenses:<br><br>1. **Expansion of Medical Education and Training**: The call for expanding U.S. medical school graduates and residencies, and removing caps on federally supported residents, can be seen as aligning with more progressive or liberal policies that emphasize increased government investment in public health infrastructure.<br><br>2. **Federal Support for Health Specialties**: Advocating for federal support for specialties and for physicians in rural areas might be interpreted differently by different political viewpoints. Liberals may view this as necessary government intervention to address healthcare disparities, while conservatives might be concerned about the extent of government involvement in healthcare.<br><br>3. **Liability and Practice Environment**: The mention of creating a less hostile practice environment and addressing liability issues could resonate with conservative viewpoints that often emphasize tort reform and reducing litigation risks for healthcare professionals.<br><br>4. **Safety Net Services for Uninsured Patients**: Providing financial support for specialists serving uninsured patients might be more aligned with liberal policies focusing on healthcare access for all, including marginalized groups.<br><br>Overall, while the text does not strongly align with a specific political viewpoint, different aspects of the discussion might be emphasized or supported differently by liberal or conservative perspectives based on their broader policy priorities and beliefs about the role of government in healthcare.	neutral

ACS	Emergency Surgical Care	6/1/2007	The text you provided does not align explicitly with either a liberal or conservative viewpoint in the traditional political sense. It focuses on professional standards and practices within the field of surgery, specifically regarding emergency surgical care. The points emphasized include:<br><br>1. **Qualifications of Surgeons**: The requirement for surgeons to be well-trained, accredited, and board-certified.<br>2. **Patient-Centered Care**: The emphasis on humane, responsive, and accessible care for all patients.<br>3. **Collaboration Among Specialists**: The acknowledgment of the value of consulting other medical and surgical specialists to enhance patient care.<br>4. **Local Credentialing and Coordination**: The idea that credentialing criteria should be developed locally and that treating surgeons should coordinate and provide continuity of care.<br>5. **Resource Commitment and Reimbursement**: The belief that optimal care requires commitment from healthcare organizations, including necessary resources and support, and appropriate reimbursement from insurers and agencies.<br><br>These points are more aligned with professional ethics and standards in healthcare rather than being indicative of a political stance. The focus is on ensuring high standards of care and the proper administration of emergency surgical services, which is a matter of professional practice rather than political ideology.	neutral
ACS	Surgery Using Lasers, Pulsed Light, Radiofrequency Devices, or Other Techniques	4/1/2007	The text you provided does not inherently align with a specific political viewpoint, be it liberal or conservative. It is a detailed and technical description of surgical procedures, including the use of lasers, pulsed light, and radiofrequency devices. It discusses the scope and regulation of surgical practices, the role of different healthcare practitioners in performing surgeries, and standards set by the American College of Surgeons for performing these procedures.<br><br>Political viewpoints, whether liberal or conservative, typically relate to opinions or stances on social, economic, or political issues. The text you provided is more of a medical or procedural nature, focused on the practices and standards of surgical procedures, rather than expressing any political stance or opinion.<br><br>However, aspects of healthcare policy, such as the regulation of medical practices, licensing of healthcare practitioners, and the scope of medical procedures, can be influenced by political beliefs. Different political groups might have varying opinions on these aspects, but the text itself is neutral and focused on medical practice rather than politics.	neutral
ACS	Insurance, Alcohol-Related Injuries, and Trauma Centers	9/1/2006	The text you've provided doesn't align strictly with a liberal or conservative viewpoint in a traditional political sense. Instead, it focuses on public health policy, specifically regarding alcohol-related injuries and trauma care. The perspective presented emphasizes the importance of screening for alcohol problems in trauma patients, the effectiveness of interventions at trauma centers, and the role of insurance policies in these scenarios.<br><br>Key points include:<br><br>1. **Acknowledgement of Alcohol's Role in Traumatic Injuries**: The text recognizes the significant percentage of trauma cases involving alcohol. This is more of a public health observation than a political statement.<br><br>2. **Insurance Policy on Alcohol-Related Injuries**: The reference to the Uniform Accident and Sickness Policy Provision Law (UPPL) and its implications for insurance coverage for patients injured while intoxicated could be seen as a critique of policies that penalize patients for alcohol use. This could be interpreted as a more progressive stance, advocating for equitable healthcare access regardless of personal behavior.<br><br>3. **Support for Trauma Center Interventions**: The emphasis on the effectiveness and cost-efficiency of alcohol interventions in trauma centers aligns with a public health approach that values preventive care and holistic treatment.<br><br>4. **Legislative Advocacy**: The support for the 2001 NAIC model law, which prohibits insurers from denying coverage based on patient intoxication, suggests an advocacy for policies that ensure equal access to medical care. This could be seen as aligning with more progressive health policy views.<br><br>Overall, while the text doesn't fit neatly into a "liberal" or "conservative" category, it does reflect certain principles such as equitable access to healthcare and the importance of public health initiatives, which can sometimes align more closely with progressive or liberal viewpoints. However, it's important to note that public health policies often transcend traditional political divides, focusing more on the wellbeing of the population than on ideological principles.	probably liberal

ACS	Statement in Support of Legislation Regarding Fire-Safe Cigarettes	2/1/2006	The text you've provided seems to be primarily focused on public health and safety rather than aligning clearly with a specific political viewpoint, be it liberal or conservative. It discusses the hazards of cigarette smoking, specifically in relation to fires caused by cigarettes, and advocates for fire-safe cigarette legislation. This focus on public health and safety can be a concern across the political spectrum.<br><br>However, the emphasis on regulation and advocacy for legislative change can sometimes be more associated with liberal viewpoints, which often support the use of government intervention to address public health issues. Conservative viewpoints, on the other hand, tend to favor less government intervention in personal choices and business practices, but they also value safety and public health.<br><br>In summary, while the text may have elements that align with a liberal approach due to its advocacy for regulation and legislative action, its primary focus on public health and safety is a concern shared across the political spectrum.	probably liberal
ACS	Statement of Principles of Palliative Care	8/1/2005	The text you provided about palliative care does not align strictly with either a liberal or conservative viewpoint in the political sense. Rather, it focuses on ethical principles in medical care, specifically within the context of palliative and surgical care. The principles mentioned – such as respect for patient autonomy, beneficence, nonmaleficence, justice, and duty – are universally recognized in the medical and ethical communities, transcending political ideologies.<br><br>The emphasis on patient-centered care, respecting patients' values and choices, and addressing a broad range of needs (physical, psychological, social, and spiritual) reflects a holistic approach to healthcare. This approach is widely accepted in modern medical practice and ethics, without specific ties to a political ideology.<br><br>The principles outlined in the text align with general ethical standards in healthcare, focusing on patient welfare and the professional responsibilities of healthcare providers, rather than advocating for a particular political stance.	neutral
ACS	Prevention of Nontraffic Vehicle-Related Injuries in Children	1/1/2005	The text you provided doesn't inherently align with a traditional liberal or conservative viewpoint in the political sense. It focuses on public health and safety, specifically regarding children's safety around motor vehicles. The American College of Surgeons' position and recommendations are based on factual data about injuries and fatalities involving children and vehicles. The emphasis is on preventive measures and legislative support to enhance child safety.<br><br>Political viewpoints might diverge on how to address these issues. For example:<br><br>- A **liberal viewpoint** might focus on the role of government in enforcing safety measures, supporting legislation for fines, and mandating the installation of safety devices in vehicles.<br>- A **conservative viewpoint** might emphasize personal responsibility and parental education, possibly resisting increased government regulation or mandates.<br><br>However, the text itself is primarily informational and oriented towards public health rather than being explicitly political. It advocates for specific safety measures and legislative actions based on data about child injuries and fatalities related to motor vehicles.	neutral
ACS	The Surgeon and HIV Infection	5/1/2004	The text you provided doesn't align strictly with either a liberal or conservative viewpoint in a political sense. Instead, it presents a medically and scientifically informed perspective on HIV/AIDS, focusing on its implications for surgical practices and healthcare worker safety. The text emphasizes the importance of stringent infection control measures, acknowledges the low risk of HIV transmission in surgical settings, and advocates for the rights and responsibilities of surgeons, both HIV-infected and uninfected, in accordance with scientific evidence and ethical considerations.<br><br>This stance is more reflective of a public health and medical ethics viewpoint rather than a politically driven one. It balances concerns about patient safety with the rights and professional capabilities of healthcare workers, without delving into the politically charged aspects often associated with HIV/AIDS discussions, such as sexuality, drug use, or other social issues. The text appears to be grounded in a commitment to evidence-based medicine, patient care, and the well-being of healthcare professionals.	neutral

ACS	The Surgeon and Hepatitis	4/1/2004	The text provided is a factual and technical discussion about the risks and management of blood-borne pathogens, specifically hepatitis B virus (HBV) and hepatitis C virus (HCV), in healthcare settings. It primarily focuses on the transmission risks between patients and healthcare workers, particularly surgeons, and outlines preventive measures and protocols for handling such risks.<br><br>This text doesn't align explicitly with either a liberal or conservative viewpoint. It is a medically and scientifically oriented piece, presenting information that is relevant to public health and occupational safety within the healthcare field. The approach is neutral, emphasizing evidence-based practices, public health guidelines, and the importance of safety measures in medical environments.<br><br>Political viewpoints, whether liberal or conservative, typically involve interpretations or opinions about policies, governance, and social issues. In contrast, this text is centered around medical facts and standard practices in healthcare, which transcend political affiliations and are based on scientific research and public health principles.	neutral
ACS	Universal Health Insurance	3/1/2004	The text you've provided reflects a mix of viewpoints, some aligning with liberal and others with conservative perspectives.<br><br>1. **Universal Access to Care**: Endorsing universal access to care is typically associated with a liberal viewpoint. Liberals often advocate for healthcare as a right and support efforts to make it universally accessible.<br><br>2. **Incremental Achievement and State-by-State Implementation**: The idea of achieving universal health insurance coverage incrementally and allowing states to implement it according to local priorities is more in line with a conservative approach. Conservatives generally favor gradual change and giving more autonomy to states in policy implementation.<br><br>3. **Investment in Information Technology**: Promoting information technology to improve patient care is a viewpoint that doesn't strongly align with traditional liberal or conservative ideologies. Both sides might support this for different reasons: liberals for improving healthcare quality and conservatives for efficiency and cost reduction.<br><br>4. **Reducing Healthcare Costs Through Efficiency Rather Than Rationing**: Preferring to reduce healthcare costs through administrative reforms and efficiency improvements, rather than by rationing care, can be seen as a conservative viewpoint. Conservatives often emphasize cost reduction through efficiency and market-based solutions rather than limiting services.<br><br>5. **Administrative Reforms Like Standard Health Insurance Claim Forms**: Advocating for administrative reforms such as standardizing claim forms can be seen as a practical, bipartisan issue, but the emphasis on administrative efficiency over broader systemic changes might be more appealing to conservative ideals.<br><br>Overall, this text appears to blend elements from both liberal and conservative ideologies, suggesting a pragmatic approach that seeks to find a middle ground between the two. This type of approach is common in professional organizations that aim to influence policy in a way that garners broad support.	neutral
ACS	Statement in Support of a Sustainable, Competent, and Diverse Nursing Workforce	1/1/2004	The text you've provided is focused on the topic of nursing supply and demand in the U.S., particularly highlighting the challenges and trends affecting the nursing workforce. This topic, in itself, is generally neutral and can be of interest to both liberal and conservative viewpoints. However, the manner in which different political ideologies might interpret or respond to these challenges can vary.<br><br>1. **Liberal Viewpoint:** From a liberal perspective, emphasis might be placed on the need for increased government investment in healthcare education and training, advocating for better working conditions and higher wages for nurses, and highlighting the importance of diversity in the nursing workforce. Liberals may also focus on the need for more comprehensive healthcare policies that address the root causes of nurse shortages and the impact on patient care.<br><br>2. **Conservative Viewpoint:** Conservatives might approach the issue by emphasizing the role of market forces in addressing the nursing shortage. They might advocate for less government intervention and more private sector solutions, such as incentives for private hospitals to invest in nursing education and training. They could also focus on reducing regulatory barriers that might impede the growth of the nursing workforce or the efficiency of healthcare delivery.<br><br>Overall, the text presents factual information about the nursing shortage and its implications, which can be interpreted through various ideological lenses. The solutions or policy recommendations that one might infer from the text can align more closely with either liberal or conservative ideologies, depending on the emphasis on government intervention versus market-based solutions.	neutral

ACS	Disaster and Mass Casualty Management	8/1/2003	The text provided does not inherently align with either a liberal or conservative viewpoint. It is a factual and procedural statement regarding the role of surgeons and medical professionals in disaster response. The focus is on the importance of training, preparedness, and integrated response strategies in managing mass casualties following disasters. This type of content typically transcends political affiliations, focusing instead on public health, safety, and emergency response best practices.<br><br>It emphasizes the need for comprehensive disaster planning, triage, medical management, and the integration of various community resources, which are generally considered non-partisan issues. The perspective is more aligned with professional and emergency management standards rather than being influenced by a particular political ideology.	neutral
ACS	Scope of Practice	11/1/2002	The text you've provided is primarily a statement of professional standards and guidelines from the American College of Surgeons. It outlines the qualifications required for surgeons, the importance of continuing education, adherence to specialty board guidelines, and the need for honesty and transparency in public education and advertising about surgical qualifications.<br><br>This text does not explicitly align with a liberal or conservative viewpoint as it is focused on professional standards within the medical field. It addresses the qualifications and ethical responsibilities of surgeons, which are generally considered to be matters of professional practice rather than political ideology.<br><br>In general, discussions about professional standards and qualifications in medicine tend to revolve around ensuring patient safety, maintaining high levels of care, and upholding ethical standards. These concerns transcend political affiliations and are typically shared across the political spectrum.	neutral
ACS	The Use of Animals in Research, Education, and Teaching	10/1/2002	The statement you've provided from the American College of Surgeons regarding the use of laboratory animals in research doesn't neatly align with either a strictly liberal or conservative viewpoint. It reflects a stance that values the responsible and humane treatment of animals in research, which can be a concern across the political spectrum.<br><br>Key points to consider:<br><br>1. **Ethical Treatment of Animals**: The emphasis on humane treatment and minimization of pain and distress aligns with values that are often associated with animal rights and welfare advocacy. This concern for animal welfare can be found among people with a range of political views.<br><br>2. **Scientific Research**: The acknowledgment of the necessity of animal use in current biomedical research may align more with a pragmatic or scientific viewpoint, which doesn't necessarily fall strictly into liberal or conservative categories. It recognizes the current limitations of alternatives to animal testing in certain areas of research.<br><br>3. **Legislation and Regulation Compliance**: The mention of adherence to local, state, and federal laws indicates a respect for existing legal frameworks, which is a perspective that can be shared by individuals with diverse political beliefs.<br><br>4. **Search for Alternatives**: The statement’s encouragement to develop and employ alternatives to the use of live animals can resonate with more progressive or reformist viewpoints, which are often about seeking change or improvement in existing practices.<br><br>It's important to note that issues like animal welfare in research often transcend simple political categorizations, as they involve ethical, scientific, and practical considerations that people from different political backgrounds can view differently.	neutral
ACS	Residency Work Hours	9/1/2002	The text you've provided focuses on the principles of surgical residency education, emphasizing patient safety, resident well-being, and the quality of medical training. It does not neatly align with either a liberal or conservative viewpoint in the traditional political sense. Instead, it addresses issues related to medical education, work conditions for medical residents, and patient care quality.<br><br>The concerns raised are universal in the field of healthcare education and do not inherently favor a specific political ideology. Discussions about resident work hours, patient safety, and the need for adequate support and educational resources are prevalent in medical education debates, transcending political boundaries.<br><br>The emphasis on safe and well-supervised patient care, resident well-being, and the importance of a supportive educational environment reflect general principles in the healthcare sector rather than political ideologies. Therefore, it would be more accurate to categorize this text as focused on medical education and healthcare policy rather than aligning it with a specific political viewpoint.	neutral

ACS	Recommendations for Facilities Performing Bariatric Surgery	9/1/2000	The text provided presents a factual and medical perspective on the issue of obesity, particularly focusing on the consequences of morbid obesity and the efficacy of bariatric surgery. It doesn't explicitly align with either a liberal or conservative viewpoint, as it primarily discusses medical data, surgical procedures, and health care recommendations rather than political or ideological arguments.<br><br>The content focuses on:<br>1. The health implications and costs associated with obesity in the U.S.<br>2. Definitions and health risks of morbid obesity.<br>3. The effectiveness and specifics of bariatric surgical procedures.<br>4. Recommendations for surgical practices, team composition, and hospital facilities.<br><br>In the context of political viewpoints:<br>- A conservative viewpoint might emphasize personal responsibility, the role of individual lifestyle choices in obesity, and concerns about the cost of healthcare.<br>- A liberal viewpoint might focus on the societal factors contributing to obesity, advocate for broader access to healthcare, and emphasize the role of government in addressing public health crises.<br><br>However, the text itself is neutral and sticks to medical facts and guidelines, without delving into these broader social or political discussions.	neutral
ACS	ACS Statement on Domestic Violence	2/1/2000	The text provided presents a perspective focused on public health and social welfare, particularly in the context of addressing and preventing domestic violence. It's important to recognize that issues like domestic violence can be approached from various political viewpoints, and the framing of such issues might align differently across the political spectrum.<br><br>1. **Liberal Viewpoint**: Generally, liberals tend to emphasize the importance of social programs, healthcare accessibility, and the role of public institutions in addressing societal issues. From a liberal perspective, the emphasis on recognizing domestic violence as a major public health issue, the need for medical professionals to be involved in identifying and preventing it, and the call for community leadership might resonate well. This aligns with the liberal focus on societal responsibility and proactive public health measures.<br><br>2. **Conservative Viewpoint**: Conservatives typically prioritize individual responsibility, limited government intervention, and a focus on traditional family values. The text's emphasis on the role of surgeons and medical professionals in addressing domestic violence could be seen as an individual and professional responsibility rather than a call for expansive government programs. However, conservatives might be more hesitant about the extent of medical professionals' involvement in social issues, preferring a more limited role for them that focuses primarily on medical care rather than societal leadership.<br><br>In conclusion, while the text primarily aligns with a public health perspective, its emphasis on professional responsibility and community leadership can resonate to varying degrees with both liberal and conservative viewpoints, depending on how these roles are interpreted in the context of societal and governmental responsibilities.	probably liberal
ACS	Ultrasound Examinations by Surgeons	6/1/1998	The text you've provided is a technical and educational document about the use of ultrasonography in surgical practices. It outlines standards, guidelines, and training requirements for surgeons using ultrasound technology. This text is strictly professional and medical in nature, focusing on the verification process for surgeons, the educational criteria, the use of ultrasound in various medical scenarios, and the maintenance of quality standards in ultrasound facilities.<br><br>This content does not align with a specific political viewpoint, whether liberal or conservative. It is a neutral, factual document concerned with medical practice and education, rather than political ideology or policy. The text is concerned with ensuring that medical professionals are properly trained and facilities are adequately equipped to provide high-quality medical care, which is a nonpartisan issue.	neutral

ACS	Physician Qualifications for Stereotactic Breast Biopsy: A Revised Statement	5/1/1998	The text you've provided primarily focuses on the qualifications, training, and professional standards for physicians performing stereotactic breast biopsies. It outlines the requirements for both radiologists and surgeons in collaborative and independent practice settings. The text is technical and specific to medical practice standards and does not inherently align with a specific liberal or conservative viewpoint.<br><br>Liberal and conservative viewpoints typically relate to political or social issues and ideologies. Discussions on medical qualifications and procedures, such as the one in this text, are generally neutral and based on scientific, medical, and educational standards. They aim to ensure patient safety, quality of care, and professional competence, rather than reflect political ideologies.<br><br>In summary, this text is neutral and professional, pertaining to medical standards and practices, without any direct alignment to liberal or conservative viewpoints.	neutral
ACS	Verification by the American College of Surgeons for the Use of Emerging Technologies	5/1/1998	The text you've provided outlines procedural guidelines by the American College of Surgeons for verifying surgeons in new technological procedures. It's focused on technical and professional standards rather than political ideology, so it doesn't neatly align with a liberal or conservative viewpoint.<br><br>The content primarily emphasizes the importance of rigorous evaluation, education, and continuous improvement in the medical field, particularly in adopting new technologies. These themes are generally non-partisan and are more about ensuring medical competency and patient safety than about advancing any specific political agenda.<br><br>In the broader context, both liberal and conservative groups may have differing views on aspects of healthcare, such as regulation, access, and funding. However, this specific text is more centered on professional standards within the medical community and doesn't directly address those broader political issues.	neutral
ACS	Approval of Courses in New Skills	3/1/1998	The text you've provided is primarily informational and technical in nature, focusing on the standards and methodologies for surgical training and education, particularly in the context of new technologies. It does not inherently align with a specific liberal or conservative viewpoint. The content is centered around:<br><br>1. **Educational Practices in Surgery**: It discusses informal and formal ways of learning new surgical techniques, emphasizing the need for high-quality, evidence-based education.<br><br>2. **Role of the American College of Surgeons**: The text mentions how this institution has responded to the need for educating surgeons in new technologies.<br><br>3. **Standards for Advanced Courses in New Technology**: It outlines detailed standards for educational courses, including program goals, content, assessment of cognitive and procedural skills, and documentation requirements.<br><br>4. **Documentation by Course Directors**: The levels of verification provided by course directors for courses sponsored by the American College of Surgeons are described.<br><br>This content is more aligned with professional and educational standards in the medical field rather than political ideologies. It reflects concerns about the quality of surgical education, the importance of evidence-based learning, and the need for standardized training methods, all of which are topics generally outside the scope of typical liberal-conservative political discourse.	neutral
ACS	Principles Underlying Perioperative Responsibility	9/1/1996	The text you've provided outlines the responsibilities and ethical considerations for a surgeon in relation to patient care. This includes confirming diagnoses, presenting treatment options, obtaining informed consent, ensuring proper preoperative preparation, performing the operation safely and competently, providing postoperative care, and disclosing information to the patient.<br><br>This text does not inherently align with a specific political viewpoint, whether liberal or conservative. It focuses on medical ethics and best practices in the field of surgery, which are generally guided by principles of patient care, safety, and informed consent rather than by political ideologies.<br><br>Ethical medical practice emphasizes the importance of patient autonomy, informed decision-making, and the physician's duty of care, which are principles upheld across various political spectrums. The emphasis on informed consent and patient choice in this text is consistent with general ethical guidelines in medicine, rather than being indicative of a specific political ideology.	neutral

ACS	Indications for the Use of Permanently Implanted Cardiac Pacemakers	2/1/1996	The text you provided does not inherently align with a liberal or conservative viewpoint. It is a factual and professional statement regarding medical guidelines and practices related to the implantation of cardiac pacemakers and antiarrhythmia devices. The content focuses on the recommendations for medical practitioners in this specific field and encourages continual education and skill maintenance. It does not delve into political ideologies or policies but instead addresses medical standards and practices. In the context of political viewpoints, liberal and conservative stances typically relate to social, economic, and political issues rather than specific medical practices or guidelines.	neutral
ACS	Issues to Be Considered Before New Surgical Technology is Applied to the Care of Patients	9/1/1995	The text you provided doesn't clearly align with a specific liberal or conservative viewpoint. It focuses on a pragmatic and scientific approach to the introduction and assessment of new biomedical technologies. This approach emphasizes patient safety, efficacy, qualifications of medical practitioners, and cost-effectiveness.<br><br>Liberal and conservative viewpoints often differ in their emphasis on regulation, government involvement in healthcare, and the speed of implementing new technologies. Liberals might favor more regulation and thorough testing to ensure safety and efficacy, while conservatives might emphasize market-driven solutions and faster implementation to encourage innovation.<br><br>However, the text seems neutral and focuses more on the practical aspects of technology adoption in healthcare rather than political ideologies. It stresses the importance of evidence-based decision-making, rigorous testing, and thorough evaluation, which are principles that can be endorsed by people across the political spectrum.	neutral
ACS	Managed Care and the Trauma System	1/1/1995	The text provided primarily outlines the challenges and proposed solutions in the context of trauma care systems and managed care in the United States. It does not align strictly with either a liberal or conservative viewpoint as traditionally defined in U.S. politics. Instead, it focuses on specific issues related to healthcare delivery, patient care, and insurance practices.<br><br>Liberal and conservative viewpoints typically differ on healthcare policy, but this text does not explicitly advocate for a particular political ideology. Instead, it discusses the practical and ethical considerations in trauma care and managed care systems, such as the need for rapid treatment authorization, appropriate patient triage, reimbursement issues, and the importance of trauma centers in providing emergency care.<br><br>Both liberal and conservative perspectives might find elements of agreement or disagreement with various aspects of the text. For example:<br><br>1. **Liberal Viewpoint:** Generally advocates for more comprehensive healthcare coverage and might support the idea of minimizing barriers to emergency care and ensuring prompt treatment without bureaucratic hurdles. However, they might also emphasize the need for broader healthcare reforms beyond just trauma care.<br><br>2. **Conservative Viewpoint:** Typically emphasizes personal responsibility and free-market solutions in healthcare. Conservatives might agree with the need for efficient and effective trauma care, but they may also focus on the potential costs and regulatory impacts of the proposed changes.<br><br>Overall, the text is more technical and policy-oriented rather than politically ideological. It presents issues and recommendations from a healthcare and patient care perspective, which can be relevant to policymakers and stakeholders across the political spectrum.	neutral

ACS	Statement of Recommendations to Ensure Quality of Surgical Services in Managed Care Environments	12/1/1994	The text you've provided is a set of recommendations from the American College of Surgeons regarding the quality of surgical care within managed care systems. It focuses on ensuring that patients receive timely, appropriate, and high-quality surgical care, irrespective of financial incentives or constraints.<br><br>This text doesn't neatly align with a traditional liberal or conservative viewpoint as understood in a political context. Instead, it primarily addresses healthcare quality and management, emphasizing patient care standards, the role of surgeons in healthcare decision-making, and the importance of data and outcomes in evaluating care.<br><br>In the broader context of U.S. politics, aspects of these recommendations might resonate with both liberal and conservative perspectives:<br><br>1. **Liberal Viewpoint Alignment**: Emphasis on equal access to high-quality care and advocacy for patients could align with liberal values, which often prioritize equitable access to healthcare and safeguarding patient rights.<br><br>2. **Conservative Viewpoint Alignment**: The focus on cost-effectiveness and the involvement of surgeons in decision-making can align with conservative values, which often emphasize efficient healthcare spending and the autonomy of medical professionals.<br><br>However, it's important to note that healthcare policy is a complex field, and these recommendations are primarily professional and practical in nature, not explicitly political. They aim to balance quality of care with cost-effectiveness, which is a common goal across various healthcare policies, regardless of political affiliation.	neutral
ACS	Emerging Surgical Technologies and the Evaluation of Credentials	6/1/1994	The statement you've provided on "Emerging Surgical Technologies" is primarily technical and procedural in nature, focusing on the steps and guidelines for introducing, evaluating, and implementing new surgical technologies. It emphasizes the importance of safety, efficacy, education, and ethical considerations in the development and application of new surgical technologies.<br><br>This statement doesn't explicitly align with a traditional liberal or conservative viewpoint as understood in political terms. Instead, it reflects a scientific and professional approach to healthcare and technology implementation. It focuses on ensuring that new surgical technologies are introduced in a responsible and effective manner, which is a concern that transcends political ideologies.<br><br>In general, liberal viewpoints tend to emphasize the importance of access to healthcare and may support the advancement of medical technologies as a means to improve healthcare outcomes and accessibility. Conservatives might focus on the cost-effectiveness and efficiency of new technologies, as well as the importance of individual choice and market-driven solutions in healthcare.<br><br>However, the text you provided is more about best practices in the field of surgery rather than a statement on broader healthcare policy, which is where more distinct liberal or conservative viewpoints typically emerge.	neutral
ACS	Statement Regarding Clinical Trials	5/1/1994	The text provided does not clearly align with either a liberal or conservative viewpoint as it primarily focuses on clinical and medical ethics, research integrity, and patient care in the context of breast cancer trials. Issues like the integrity of clinical trials, patient safety, and scientific accuracy transcend typical political categorizations. The emphasis is on maintaining trust in clinical trials, ensuring the quality of medical research, and addressing patient concerns, which are topics generally considered non-partisan and not tied to a specific political ideology.	neutral
ACS	Surgical Residencies and the Educational Environment	1/1/1994	The text provided primarily presents an objective, factual overview of the development and current state of surgical residency programs in the United States, particularly focusing on the American College of Surgeons, the American Board of Surgery, and various Residency Review Committees. It doesn't explicitly align with a liberal or conservative viewpoint. Instead, it discusses educational standards, the evolution of surgical training, duty hours, the resident work environment, and the importance of a balanced educational and working environment for surgical residents.<br><br>The issues discussed are more related to professional standards, educational quality, and healthcare administration rather than political ideology. While there may be underlying policy implications related to healthcare education and work conditions, the text itself does not advocate for specific political ideologies or policies typically associated with liberal or conservative platforms.	neutral

ACS	Laparoscopic and Thoracoscopic Procedures	9/1/1993	The text you've provided appears to be a professional guideline or standard for surgeons, particularly focusing on laparoscopic and thoracoscopic surgical procedures. It emphasizes the importance of adequate training and experience in comparable open surgeries, along with the ability to manage potential complications, including the judgment and capability to switch to an open surgical procedure if needed.<br><br>This statement doesn't directly align with a typical liberal or conservative viewpoint as it is primarily focused on medical professionalism and standards of care. It represents a position on best practices in surgery, which is more of a professional and technical matter than a political one.<br><br>Political viewpoints, whether liberal or conservative, usually pertain to issues like government policy, social justice, economic systems, etc., rather than specific technical standards in professional fields such as medicine or surgery. The emphasis in this text on high standards and qualifications for surgeons is a matter of professional ethics and practice, not inherently tied to a political ideology.	neutral
ACS	Certificates of Special or Added Qualifications	4/1/1992	The text provided does not inherently align with a traditional liberal or conservative viewpoint as it pertains to the professional standards and qualifications within the field of surgery, rather than political or ideological matters. It discusses the expectations and criteria set by the American College of Surgeons for the practice and certification of surgeons, focusing on the importance of training, experience, and performance over the possession of additional qualifications. This topic is more aligned with professional and medical standards rather than political ideologies.	neutral
ACS	Laparoscopic Cholecystectomy	6/1/1990	The text you've provided appears to be focused on medical standards and practices rather than political viewpoints. It emphasizes the importance of surgeons being qualified in both laparoscopic and open cholecystectomy for optimal patient care, suggesting that a comprehensive skill set is crucial for handling various surgical situations and potential complications.<br><br>This perspective is more aligned with professional medical standards and best practices in healthcare rather than a specific political ideology, liberal or conservative. In healthcare, discussions about qualifications and procedures are typically grounded in clinical evidence and professional consensus, rather than political ideology.	neutral
ACS	Statement for the Advisory Council for General Surgery to the Board of Regents of the American College of Surgeons	6/1/1989	The text you've provided is largely apolitical and focuses on the standards and practices within the field of General Surgery. It outlines the definition of General Surgery, the scope of practice, training standards, and the role of technology in surgery. This text does not explicitly align with a liberal or conservative viewpoint as it addresses professional standards and practices in a medical specialty rather than political ideologies or policies.<br><br>Issues concerning medical education, practice standards, and professional definitions are generally considered neutral and are more about maintaining quality and consistency in the healthcare field. They typically do not fall under political discussions unless they intersect with broader policy issues like healthcare reform, which is not the case in this text.	neutral
ACS	Disclosure of Commercial Interest	1/1/1989	The text you've provided does not inherently align with a liberal or conservative viewpoint. It appears to be a neutral, ethical guideline or policy statement concerning the conduct of Fellows, possibly in a professional or academic organization. The focus is on maintaining transparency and honesty in communications, especially in the context of commercial advertising or promotional activities.<br><br>The text emphasizes the importance of disclosure of financial interests or remuneration to prevent misleading or deceptive communications. This stance is more about professional ethics and integrity rather than political ideology. Both liberal and conservative viewpoints generally support ethical standards in professional conduct, although they might differ in their approaches to regulation and oversight in such matters.	neutral
ACS	Fundamental Characteristics of Surgical Residency Programs	8/1/1988	The text provided appears to be a balanced discussion on the topic of resident working hours in medical settings, particularly in surgical education. It does not explicitly align with either a liberal or conservative viewpoint. Instead, it focuses on the practical and educational aspects of residency training, emphasizing the importance of continuity of care, the need for supervision, and the varying requirements of different specialties.<br><br>The text discusses the drawbacks of strictly limiting working hours without considering the impact on educational opportunities and patient care. It also mentions the importance of supervision, the structure of residency programs, and the need for appropriate work schedules that prioritize education and patient care.<br><br>It's worth noting that the text does criticize the use of residents for non-educational activities and points out the need for adequate support services. This aspect might be interpreted as a call for better working conditions, which can be a concern shared across the political spectrum.	neutral

			Overall, the text seems more focused on practical considerations and educational standards in the medical field rather than advocating for a specific political ideology.	
ACS	Statement in Response to the Clinical Alert from the National Cancer Institute	7/1/1988	The text you provided is primarily a scientific and clinical discussion about the early release of information from the National Cancer Institute (NCI) regarding breast cancer treatment trials. It does not inherently align with a liberal or conservative viewpoint as it focuses on medical practice, the timing of information release, and the implications for patient care and physician decision-making.<br><br>Political viewpoints, such as liberal or conservative ideologies, typically relate to opinions and policies on government, economics, social issues, and public policy. The text in question is centered around medical practice and ethics, particularly the dissemination of clinical trial results and its impact on both physicians and patients.<br><br>While healthcare policy can be a topic of political debate, the specific content of this text is more aligned with medical ethics and clinical practice rather than a specific political ideology.	neutral
ACS	Interprofessional Relations with Doctors of Chiropractic	11/1/1987	The text you provided is a statement from the American College of Surgeons regarding the professional relationship and cooperation between doctors of chiropractic and medical physicians. This statement does not inherently align with a liberal or conservative viewpoint in the typical political sense. It is primarily focused on healthcare practice and ethics, emphasizing collaboration and mutual respect between different types of healthcare professionals for the benefit of patient care.<br><br>The text advocates for legal and ethical cooperation, highlighting individual choice and judgment in professional associations. It also mentions various forms of collaboration like referrals, consultations, group practices, participation in training programs, research, and educational seminars.<br><br>It's important to note that healthcare policies and practices can sometimes be influenced by political viewpoints, but this statement itself is neutral and focused on medical ethics and professional conduct rather than political ideology.	neutral
ACS	Billing of Surgical Fees for Extracorporeal Shock Wave Lithotripsy (ESWL) Treatment of Patients with Calculous Disease	4/1/1986	The text you've provided doesn't align explicitly with either a liberal or conservative viewpoint in the traditional political sense. Instead, it addresses ethical considerations in healthcare billing practices, specifically regarding extracorporeal shock wave lithotripsy (ESWL).<br><br>The focus of the text is on the transparency and ethics of fee arrangements between healthcare providers and patients. It emphasizes the importance of patients being fully informed about the fees for the services they receive and who is receiving payments for these services. This concern with transparency and ethical billing practices in healthcare transcends typical political categorizations and is more closely related to medical ethics and healthcare policy.<br><br>The American College of Surgeons' stance, as described in the text, advocates for patient awareness and the ethical handling of fee distributions. Such a stance can be supported by individuals across the political spectrum, as it deals with fairness, transparency, and the integrity of medical professionals, which are values widely upheld in various political ideologies.	neutral

APA	Position Statement on Peer Support Services	12/2023	The text provided doesn't align strictly with either a liberal or conservative viewpoint, as it focuses on a healthcare issue rather than a political ideology. The perspective presented here emphasizes the importance of peer support in psychiatric care and recovery-oriented systems. This viewpoint is more aligned with general healthcare best practices and mental health advocacy rather than being distinctly liberal or conservative.<br><br>The American Psychiatric Association's (APA) stance on the value of peer support services and their integration into recovery-oriented systems of care is grounded in clinical and recovery-focused principles. Their emphasis on appropriate training and reimbursement for peer support personnel reflects a commitment to professional standards and effective patient care, which transcends typical political categorizations.<br><br>Healthcare policies can be viewed through a political lens, with different parties having varying approaches to funding, management, and delivery of healthcare services. However, this particular issue – the advocacy for peer support in mental health care – is more about healthcare best practices and less about a specific political ideology.	neutral
APA	Position Statement on Contingency Management for the Treatment of Stimulant Use Disorder	12/2023	The text you've provided discusses a specific approach to addressing stimulant use disorder (StUD) and doesn't neatly align with a traditional liberal or conservative viewpoint. Instead, it focuses on evidence-based practices in healthcare and addiction treatment. Let's break it down:<br><br>1. **Emphasis on Evidence-Based Practices**: The text highlights the importance of evidence-based practices like contingency management (CM) in treating StUD. This approach is consistent with a more general scientific and medical perspective that values empirical research and data-driven solutions.<br><br>2. **Regulatory and Legislative Changes**: The call for policy changes to facilitate the implementation of CM could be interpreted differently by liberal and conservative viewpoints. Liberals might focus on the aspect of expanding healthcare options and addressing public health crises, while conservatives might emphasize the need for efficient and effective solutions, possibly with concerns about regulatory overreach or the cost of such programs.<br><br>3. **Psychosocial Interventions**: The focus on psychosocial interventions like CM, community reinforcement therapy, or cognitive-behavioral therapy might be seen as a more holistic approach to addiction treatment. This could resonate with both liberal and conservative viewpoints, albeit for different reasons.<br><br>4. **Funding for Research**: The suggestion to increase funding for research into these treatments might be more closely aligned with liberal viewpoints that generally support increased funding for public health initiatives. However, the focus on research and data could also appeal to conservative perspectives that emphasize effective and proven solutions.<br><br>In summary, while certain aspects of the text might resonate more with one political ideology over the other, the overall content is centered around a medical and research-based approach to treating StUD. It doesn't squarely fit into a political category but rather crosses over multiple aspects that can be interpreted through different ideological lenses.	neutral

APA	Position Statement on Harm Reduction	12/2023	The text you've provided outlines a perspective that is generally aligned with a liberal viewpoint, particularly in its emphasis on harm reduction strategies for addressing substance use disorders (addiction). This viewpoint is characterized by the following key aspects:<br><br>1. **Focus on Public Health and Harm Reduction:** The text emphasizes reducing the harms associated with substance use through evidence-based harm reduction strategies. This approach is typically more aligned with liberal viewpoints, which often prioritize public health measures and view addiction as a health issue rather than solely a criminal justice problem.<br><br>2. **Support for Policy and Legislative Changes:** The call for policy, legislation, and funding to enhance harm reduction interventions aligns with a liberal approach, advocating for systemic changes to improve health outcomes.<br><br>3. **Equitable Access to Treatment:** The emphasis on ensuring equitable access to harm reduction interventions for all patients reflects a liberal concern for social justice and equality in healthcare.<br><br>4. **Emphasis on Education and Research:** The advocacy for increased training in harm reduction practices and research into effective modalities is consistent with liberal values that prioritize education and evidence-based approaches.<br><br>5. **Healthcare System Integration:** The support for policies that encourage the healthcare system's uptake of harm reduction strategies indicates a preference for integrating these approaches into mainstream healthcare, a perspective more commonly associated with liberal ideologies.<br><br>It's important to note that while these points align more closely with a liberal viewpoint, the issue of substance use disorder treatment and harm reduction can transcend traditional political boundaries, and there may be conservatives who also support these measures. The delineation between liberal and conservative viewpoints on such complex issues is not always clear-cut and can vary depending on individual beliefs and the specific context of the discussion.	liberal
APA	Position Statement on Housing, Homelessness, and Mental Health	12/2023	The text you've shared aligns more closely with a liberal viewpoint. Here's a breakdown of why:<br><br>1. **Emphasis on Housing as a Human Right**: The perspective that housing is a universal human right aligns with liberal ideologies that prioritize social welfare and human rights. Conservative viewpoints tend to focus more on individual responsibility and less on housing as a right.<br><br>2. **Focus on Economic Causes of Homelessness**: Highlighting economic factors like job loss and debt as primary triggers of homelessness is typical of liberal perspectives. Conservatives might emphasize individual responsibility and decision-making in discussions of homelessness.<br><br>3. **Avoiding Conflation of Mental Health with Homelessness**: The text argues against conflating mental health disorders with homelessness. This approach, which emphasizes understanding and addressing the root causes of social issues, is more aligned with liberal ideologies. Conservative viewpoints might differ in their approach to mental health and homelessness, potentially focusing more on individual behavioral change.<br><br>4. **Support for Large-scale Housing Interventions**: Advocacy for state and federal housing initiatives and the Housing First approach is consistent with liberal policies that support government intervention in social issues. Conservatives might be more inclined to support private sector solutions or limited government involvement.<br><br>5. **Positions of the APA (American Psychological Association)**: The APA's positions mentioned in the text, such as the importance of safe and stable housing for mental health and not making housing contingent on treatment, align with liberal views that prioritize social support systems and oppose mandatory treatments.<br><br>In summary, the text presents a view that is more aligned with liberal perspectives, particularly in its emphasis on housing as a human right, focus on economic causes of homelessness, and support for government-led housing interventions.	liberal

APA	Position Statement on the Role of Psychiatrists in Reducing Physical Health Disparities in Patients with Mental Illness	12/2023	The text you've shared presents a viewpoint that is primarily focused on healthcare and mental health, rather than aligning strictly with a liberal or conservative political viewpoint. The issues and positions it discusses are centered around the role of psychiatrists in monitoring and treating patients with mental illness and substance use disorders, and the importance of addressing their medical needs.<br><br>This perspective is more about healthcare policy and the role of mental health professionals in comprehensive patient care. It emphasizes the importance of integrated care, where psychiatrists are involved not only in psychiatric treatment but also in monitoring general health and coordinating with other healthcare services.<br><br>Both liberal and conservative viewpoints might agree on the importance of addressing the healthcare needs of individuals with mental illness and substance use disorders, though they might differ in their approaches to healthcare policy and funding. This text, however, doesn't specifically advocate for a particular funding model or healthcare system structure, which are often the points of divergence between liberal and conservative policies in healthcare. It focuses more on professional standards and roles within the existing healthcare framework.	neutral
APA	Position Statement on Firearms Access: Inquiries in Clinical Settings	12/2023	The text presented reflects a viewpoint more commonly associated with liberal ideologies in the United States. The key aspects of this position include:<br><br>1. **Emphasis on Public Health Concerns**: Recognizing firearm-related deaths and injuries as a major public health issue aligns with a liberal perspective, which often prioritizes public health and safety over individual gun rights.<br><br>2. **Opposition to Restrictions on Physicians**: The stance against legislation that limits physicians' ability to inquire about firearms in patient care contexts is in line with liberal values that support comprehensive healthcare, including mental health, and the belief that healthcare professionals should have the freedom to address all aspects of a patient's health and safety.<br><br>3. **Role of Healthcare in Firearm Safety**: Advocating for the involvement of healthcare professionals in discussions about firearm access and safety reflects a liberal viewpoint that considers gun control a matter of public health and safety, rather than solely a matter of individual rights.<br><br>This position contrasts with conservative viewpoints, which typically emphasize individual gun rights and may view such inquiries as an infringement on personal freedoms. Conservatives often prioritize the Second Amendment rights and may oppose what they perceive as governmental or institutional overreach into private matters, including firearm ownership.<br><br>It's important to note that these generalizations may not apply to every individual's beliefs, as there is a wide range of perspectives within both liberal and conservative circles.	liberal
APA	Position Statement Police Interactions with Persons with Mental Illness	12/2023	The text you've provided outlines a position regarding the training and deployment of law enforcement officers in situations involving individuals with mental illness. It doesn't align strictly with either a liberal or conservative viewpoint as traditionally defined in American politics. Instead, it focuses on a practical, policy-based approach to a specific issue.<br><br>The position advocates for specialized training for law enforcement officers to recognize and handle mental health crises. This perspective emphasizes the need for deescalation and the avoidance of physical force, which could be seen as aligning with more progressive or liberal ideas about police reform and mental health awareness.<br><br>However, it also acknowledges the role of law enforcement in certain situations where safety is a concern, which might resonate with more conservative viewpoints that emphasize the importance of law and order.<br><br>Overall, this position by the American Psychiatric Association (APA) reflects a nuanced approach that transcends simple political categorization, focusing instead on practical solutions to complex issues at the intersection of law enforcement and mental health care.	neutral

APA	Position Statement on Utilization of Measurement Based Care	12/2023	The text provided appears to be neutral and focused on a specific issue within the healthcare field, specifically the use of Measurement-Based Care (MBC) in psychiatry. It does not explicitly align with a traditional liberal or conservative viewpoint, as it is more focused on clinical practice and evidence-based treatment in mental health care rather than broader political or ideological issues.<br><br>The emphasis on evidence-based treatment, efficiency, and improvement of patient outcomes through standardized measures is generally a professional and clinical perspective. It aligns with a focus on improving healthcare quality and effectiveness, which can be a point of interest across various political spectra. Both liberal and conservative viewpoints often agree on the importance of effective healthcare, although they may differ in their approaches to healthcare policy and funding.<br><br>In summary, the text is more about a specific approach within psychiatric care and less about broader political ideologies.	neutral
APA	Position Statement on Abortion, Family Planning, Legislative Intrusion, and Reproductive Decisions	12/2023	The text you've provided aligns more closely with a liberal viewpoint. It emphasizes the importance of women's rights, reproductive freedom, and the autonomy of patient-physician relationships in making healthcare decisions, including abortion. These principles are typically associated with liberal perspectives, which often advocate for personal freedoms in reproductive choices and oppose government interference in these decisions. The text's focus on ensuring that abortion remains accessible and is considered a part of standard healthcare is also a position more commonly held by those with liberal views. In contrast, conservative viewpoints generally emphasize more restrictive policies on abortion, focusing on moral and ethical considerations and often advocating for greater governmental involvement in limiting access to abortion services.	liberal
APA	Position Statement on Regulatory Oversight of Data, Apps, and Novel Technologies in Mental Health	10/2023	The text provided presents a nuanced perspective on the use of technology in mental health services and the associated privacy concerns. It doesn't neatly align with traditional liberal or conservative viewpoints, as the issues addressed span across concerns typically associated with both political orientations.<br><br>1. **Privacy and Regulation Concerns**: The emphasis on data privacy and the need for updated patient protection frameworks can resonate with a conservative viewpoint, which often values personal privacy and is cautious about data collection by corporations. However, the call for increased regulation and oversight of technology companies and their handling of health data aligns more with a liberal perspective, which generally supports stronger regulation to protect consumer rights and privacy.<br><br>2. **Healthcare Accessibility and Quality**: The acknowledgment of consumer-facing, treatment-focused apps as a means to improve health outcomes through convenient access aligns with liberal ideals of increasing healthcare accessibility and leveraging technology for public good. However, the emphasis on maintaining high standards of evidence-based practice and the statement that these apps are not a substitute for professional care might be more in line with conservative views emphasizing traditional healthcare delivery methods and caution in adopting new, unregulated technologies.<br><br>3. **Role of Government and Regulation**: The position of the APA, advocating for clear and stringent data privacy standards and the need for these standards to apply broadly, suggests a belief in the active role of government or regulatory bodies in safeguarding consumer rights and privacy. This perspective is typically more aligned with a liberal viewpoint, which generally supports the idea of government intervention for public welfare and consumer protection.<br><br>Overall, the text reflects a balanced approach, addressing concerns typically associated with both liberal (regulation for consumer protection, accessibility of healthcare) and conservative (privacy, reliance on traditional healthcare methods) viewpoints. The focus is more on practical and ethical considerations in the intersection of technology and mental health, rather than on a strictly ideological stance.	neutral
APA	Position Statement on Assessing Driving Ability	7/2023	The text you've provided outlines an issue related to psychiatric disorders and driving safety, and presents the American Psychiatric Association's (APA) position on the matter. This issue is more about medical ethics and public safety rather than aligning with traditional liberal or conservative political viewpoints.<br><br>The focus here is on the balance between patient confidentiality and public safety. The APA's position supports the idea that psychiatrists should be protected from liability when reporting in good faith about a patient's ability to drive safely. This position can be seen as prioritizing public safety, which is a concern that transcends typical political lines.<br><br>In terms of liberal vs. conservative viewpoints, it's important to note that such issues often do not fit neatly into these categories. Instead, they involve complex considerations of individual rights, public health, legal responsibilities, and ethical obligations of healthcare providers.	neutral

			Therefore, it would be more accurate to view this text as addressing an ethical and public health issue rather than as strictly aligning with either a liberal or conservative viewpoint.	
APA	Position Statement on the Endorsement of the U.S. Ratification of the Convention on the Rights of the Child	7/2023	The issue of the United Nations Convention on the Rights of the Child (CRC) and the position of the American Psychiatric Association (APA) in supporting its principles can be aligned with different political viewpoints, but it's not strictly limited to a liberal or conservative perspective. Instead, it encompasses broader human rights and child welfare considerations. Here's a breakdown:<br><br>**Liberal Viewpoint Alignment:**<br>1. **Emphasis on Human Rights and Welfare:** The CRC's focus on safeguarding children's rights and welfare aligns with liberal ideologies that prioritize human rights, social welfare, and the role of international cooperation in addressing these issues.<br>2. **Support for International Agreements:** Liberals generally support active participation in international treaties and agreements, seeing them as vital for global cooperation and progress.<br><br>**Conservative Viewpoint Considerations:**<br>1. **Sovereignty and Domestic Policy Preference:** Conservatives might be cautious about ratifying international treaties like the CRC, preferring domestic solutions to issues related to children's rights and welfare. The concern often revolves around the potential loss of national sovereignty and the imposition of external standards.<br>2. **Diverse Conservative Perspectives:** It's important to note that conservatism is not a monolith, and there are conservative viewpoints that also prioritize children's welfare and rights, though they might differ in approaches and emphasis on international vs. national strategies.<br><br>**APA's Position:**<br>The APA’s support for legislation consistent with the CRC, focusing on children’s access to health, education, protection from abuse, and participation in decisions, is grounded in a commitment to children's welfare and mental health. This stance is more aligned with a humanistic and welfare-oriented viewpoint, transcending traditional political divisions.<br><br>In summary, while the CRC and APA’s support for it may have aspects that align with liberal ideologies, especially regarding human rights and international cooperation, the issue also intersects with conservative considerations about national sovereignty and policy-making. The topic of children's rights and welfare is complex and can be viewed through multiple political lenses.	neutral

APA	Position Statement Lengthy Sentences without Parole for Juveniles	7/2023	The text presented aligns more closely with a liberal viewpoint. Here's the reasoning:<br><br>1. **Focus on Rehabilitation and Developmental Considerations:** The emphasis on the developmental immaturity and potential for rehabilitation of youth offenders reflects a liberal perspective, which often prioritizes rehabilitation over punitive measures, especially for young offenders. This view is grounded in the belief that individuals, particularly the youth, can change and should be given opportunities for reform and reintegration into society.<br><br>2. **Opposition to Harsh Sentences for Minors:** The stance against long-term sentences without the possibility of parole for minors, especially for crimes committed when they were under 18, is in line with liberal ideologies. Liberals generally advocate for more lenient sentencing, especially for juveniles, based on the understanding that their cognitive and moral faculties are not fully developed.<br><br>3. **Supreme Court Decisions Referenced:** The mention of U.S. Supreme Court decisions since 2005, which have recognized the lesser culpability of youth and imposed restrictions on life without parole sentences for minors, aligns with liberal judicial philosophies that favor rehabilitative justice over strict punitive measures for juveniles.<br><br>4. **APA Position:** The American Psychological Association's (APA) position mentioned here reflects a scientifically informed perspective on juvenile justice. This approach, which considers psychological development and the potential for rehabilitation, is more commonly associated with liberal viewpoints.<br><br>In summary, the text's emphasis on the developmental aspects of youth offenders, advocacy for rehabilitative justice, and opposition to harsh sentencing for minors, aligns it more closely with liberal perspectives on juvenile justice.	liberal
APA	Position Statement on Restrictive Housing of Incarcerated Adults with Serious Mental Illness	7/2023	The text you've provided outlines a position on the use of restrictive housing (solitary confinement) in prisons, particularly for individuals with serious mental illness. It doesn't align strictly with a liberal or conservative viewpoint as traditionally defined, but rather addresses a specific issue within the criminal justice system from a mental health and human rights perspective.<br><br>Generally speaking:<br><br>- **Liberal Viewpoint**: Liberals tend to emphasize rehabilitation and mental health care in the criminal justice system. They often advocate for reducing the use of solitary confinement, especially for vulnerable populations like those with mental illness, citing human rights concerns and the potential for psychological harm.<br><br>- **Conservative Viewpoint**: Conservatives traditionally focus on law and order and might be more inclined to support the use of restrictive housing for disciplinary reasons and institutional safety. However, there's growing recognition among many conservatives of the need for prison reform, including the treatment of mental health issues in prisons.<br><br>The American Psychological Association's (APA) position, as outlined in your text, emphasizes the potential harm of restrictive housing to individuals with serious mental illness. This perspective is more closely aligned with reformist views, which can be found across the political spectrum, but are more typically associated with liberal viewpoints in the context of criminal justice reform. The focus here is on the well-being and rights of the incarcerated, and the recognition that solitary confinement can exacerbate mental health issues, which aligns with a more humanitarian and rehabilitative approach to corrections.	probably liberal

APA	Position Statement on Promoting Health and Protecting Vulnerable Populations from Social Media and Online Harms	7/2023	The text you provided presents an issue and a position by the American Psychiatric Association (APA) regarding the challenges of social media and online engagement in psychiatric practice. This statement does not align strictly with either a liberal or conservative viewpoint as traditionally defined in political terms. Instead, it focuses on health policy and research, addressing concerns about privacy, mental health, and the impact of technology on well-being.<br><br>The APA's position advocates for:<br><br>1. Federal oversight of security and privacy standards to protect personal information on online platforms.<br>2. Prevention of harmful content such as the promotion of self-harm, addiction-like behaviors, deceptive content, and unlawful marketing.<br>3. Culturally informed research funding on the impact of social media on health and the nature of social media consumption.<br><br>These points reflect concerns common to public health and privacy advocates, which can be supported by individuals across the political spectrum. They emphasize the need for regulation and research to protect vulnerable populations and understand the broader impacts of social media. This approach is generally issue-focused and pragmatic, seeking to address specific challenges rather than aligning with a broader ideological stance.	neutral
APA	Position Statement on Studying the Decriminalization of Illicit Substance Possession and Use	7/2023	The text provided appears to align more closely with a liberal viewpoint. It focuses on the need for reform in drug policy, emphasizing the reduction or elimination of criminal penalties for drug possession, increased access to treatment, and addressing racial disparities in the criminal justice system. These positions are typically associated with liberal or progressive perspectives, which often advocate for social reform, public health approaches to drug use, and addressing systemic inequalities.<br><br>In contrast, a conservative viewpoint generally emphasizes law and order, strict enforcement of drug laws, and may be less inclined to support decriminalization or a shift towards treatment-focused approaches. Conservatives often prioritize maintaining traditional legal frameworks and may view drug use more as a criminal issue rather than a public health concern.<br><br>The American Psychiatric Association's (APA) position, as outlined, aligns with these liberal perspectives by advocating for incremental policy changes, focusing on decriminalization complemented by increased access to treatment and support services, and acknowledging the disproportionate impact of drug laws on communities of color. This approach reflects a more holistic and socially conscious viewpoint commonly associated with liberal ideologies.	liberal
APA	Position Statement on Assessing the Risk for Violence	7/2023	The text you've provided seems to be a balanced, professional statement rather than one that aligns clearly with either a liberal or conservative viewpoint. It focuses on the complexities and limitations inherent in psychiatric assessments regarding the prediction of violent behavior.<br><br>Key points in the text indicate a focus on facts and professional understanding rather than political ideology:<br><br>1. **Clinical Assessment and Structured Risk Assessments**: The text acknowledges the use of clinical and structured methods to identify risk factors for violence, which is a practice accepted across various political spectrums.<br><br>2. **Difficulty in Prediction**: The acknowledgment of the difficulty in definitively predicting violent behavior reflects an understanding of the complexities in mental health, a perspective that transcends political leanings.<br><br>3. **APA Position**: The American Psychiatric Association (APA) is a professional organization focused on psychiatric knowledge and practice, and its position as described here emphasizes the practical and ethical aspects of psychiatric assessment without delving into political ideology.<br><br>4. **Realistic View of Risk Assessment**: The statement recognizes that risk assessment is not infallible, a view that is consistent with a professional, rather than a political, approach to the topic.<br><br>In summary, the text appears to be a professionally-oriented statement that doesn't clearly align with either a liberal or conservative viewpoint. It focuses on the realities and limitations of psychiatric practice in assessing the risk of violence.	neutral

APA	Position Statement on Addressing Discriminatory Policies That Prevent Access to Housing and Employment	7/2023	The text you provided outlines a position on housing and employment for persons with mental disorders, especially those with a criminal background related to their disorders. It is aligned more closely with a liberal viewpoint, primarily due to its emphasis on social justice, equal opportunities, and protection against discrimination for vulnerable populations. Liberal perspectives often prioritize social welfare, anti-discrimination measures, and support for marginalized groups, which is reflected in this position.<br><br>Key aspects of this viewpoint that align with liberal ideologies include:<br><br>1. **Opposition to Discriminatory Laws and Policies**: The stance against laws and policies that impede access to housing and employment for persons with mental disorders, including those with criminal histories, aligns with liberal views on social justice and equality.<br><br>2. **Support for Robust Protection Laws**: Advocating for laws that provide strong protection for persons with mental disorders to access housing and employment reflects a liberal emphasis on safeguarding the rights of vulnerable and marginalized groups.<br><br>3. **Focus on Reducing Hospitalizations and Recidivism**: The recognition of housing and employment as critical social determinants of health, and their role in reducing hospitalizations, symptom burden, and criminal recidivism, aligns with the liberal focus on comprehensive social welfare measures.<br><br>It's important to note that while these positions align more closely with a liberal viewpoint, mental health policy can be a bipartisan issue, with support from both liberal and conservative sides for different aspects of mental health care and support.	liberal
APA	Position Statement on Mental Health and Climate Change	3/2023	The text you've provided appears to align more with a liberal viewpoint, primarily because of its emphasis on climate change, mental health, and social action. Here's a breakdown:<br><br>1. **Focus on Climate Change**: The acknowledgment of climate change as a significant and urgent issue aligns more closely with liberal viewpoints. Liberals tend to emphasize the importance of addressing climate change and its wide-ranging impacts.<br><br>2. **Mental Health Awareness**: The text underscores the mental health impacts of climate change, which reflects a liberal perspective that often stresses the importance of mental health issues and the need for comprehensive healthcare, including mental health services.<br><br>3. **Support for Research and Intervention**: The call for research funding and the development of interventions to mitigate the effects of climate change on mental health align with liberal priorities, which often advocate for scientific research and evidence-based policy.<br><br>4. **Advocacy for Vulnerable Populations**: The emphasis on protecting underrepresented and marginalized communities, and those with mental illness, resonates with liberal values that prioritize social justice and equity.<br><br>5. **Environmental Policy Reform**: Advocating for pro-environmental policy reform and reducing the carbon footprint in medical practices aligns with liberal environmental policies focused on sustainability and reducing carbon emissions.<br><br>Conservative viewpoints, in contrast, might be less inclined to emphasize climate change as a pressing concern, might prioritize different aspects of healthcare, and may focus more on economic and regulatory impacts when considering environmental policies.<br><br>However, it's important to recognize that these are generalizations, and there is a spectrum of beliefs within both liberal and conservative ideologies. Additionally, there can be overlap in certain areas, and not all individuals or groups within these categories will fully align with these general trends.	liberal

APA	Position Statement on Intimate Partner Violence	3/2023	The text you provided appears to be a statement or position from the American Psychiatric Association (APA) on intimate partner violence (IPV) and its intersection with mental health and substance use disorders. It does not align explicitly with either a liberal or conservative political viewpoint. Instead, it focuses on public health, mental health, and victim support, areas that can be of concern to individuals across the political spectrum.<br><br>The APA's position emphasizes the need for:<br>- Guidelines for responding to IPV in clinical settings.<br>- Psychiatric education and training on IPV.<br>- Research to improve mental health care for IPV victims.<br>- Increased funding for IPV prevention and services.<br>- Increased access to culturally relevant and trauma-informed mental health and substance use disorder treatment.<br>- IPV-informed education and training to strengthen systems for survivors.<br><br>These points are generally aligned with public health and welfare concerns, advocating for improved medical education, better patient care, and increased resources for IPV victims. Such issues are often supported by a range of political ideologies, as they address fundamental concerns about health care, education, and victim support.	neutral
APA	Position Statement on Abortion and Women's Reproductive Health Care Rights	3/2023	The text you've provided aligns more closely with a liberal viewpoint. The key points in the text that suggest this alignment are:<br><br>1. **Emphasis on Evidence-Based Approach**: The text cites "ample literature evidence" to argue that first trimester abortions do not negatively impact women's mental health. This reliance on scientific and medical literature is a trait often associated with liberal perspectives, which typically emphasize evidence-based policy decisions.<br><br>2. **Opposition to Restrictions on Abortion and Family Planning**: The text mentions that restrictive abortion and contraception policies can lead to a variety of mental health problems and negatively impact women's health. Liberals generally support the right to access abortion and family planning services and oppose legislation that restricts these rights.<br><br>3. **APA's Position Against Curtailing Family Planning and Abortion Services**: The American Psychiatric Association's stance, as described in the text, is to oppose amendments, legislation, and regulations that curtail family planning and abortion services. This position aligns with liberal values that advocate for reproductive rights and healthcare access.<br><br>4. **Support for Patient Autonomy and Choice in Medical Procedures**: The text emphasizes the right of patients to make choices about their medical care, including the decision to have an abortion. This respect for individual choice and autonomy is a hallmark of liberal thinking.<br><br>5. **Viewing Access to Abortion as a Mental Health Imperative**: The text argues that the freedom to interrupt pregnancy is a mental health imperative with significant social implications. This perspective is more aligned with liberal viewpoints that consider reproductive rights as integral to mental and social wellbeing.<br><br>Overall, the text's focus on supporting reproductive rights, relying on scientific evidence, and advocating for patient autonomy in healthcare aligns it more with liberal viewpoints.	liberal
APA	Position Statement on Human Rights	3/2023	The text you've provided outlines the position of the American Psychiatric Association (APA) regarding the impact of human rights abuses on mental health. It does not align explicitly with either a liberal or conservative viewpoint in the traditional political sense. Instead, it focuses on a human rights and mental health perspective.<br><br>The APA's stance is centered on the acknowledgment of the mental health consequences resulting from human rights abuses like unjust incarceration, cruel and unusual punishment, torture, and human trafficking. It emphasizes the negative impact of the denial or cover-up of these abuses by governments and institutions on the mental health of victims and their families. This position is more about advocating for the psychological well-being of individuals affected by human rights violations and less about a specific political ideology.<br><br>In general, human rights and mental health are issues that can be championed by individuals across the political spectrum. While certain aspects of the approach to these issues might vary between liberal and conservative viewpoints, the core concern for the well-being of those affected by human rights abuses is a universally relevant issue.	neutral

APA	Position Statement Engaging Law Enforcement Personnel and Correctional Staff to Address Mental Health and Racial Inequities in Jails and Prisons	3/2023	The text you've provided aligns more with a liberal viewpoint. Liberal perspectives typically emphasize social justice, equality, and reform, especially concerning systemic issues like racial inequality and mental health care. This text focuses on:<br><br>1. **Disparities in the U.S. Incarceration System**: It highlights the overrepresentation of racial minorities in the U.S. prison population, a concern often raised in liberal discourse about systemic racism and inequality.<br><br>2. **Mental Health in the Criminal Legal System**: The emphasis on the high prevalence of mental health diagnoses among incarcerated individuals and the need for better mental health care aligns with liberal concerns about health care reform and the treatment of vulnerable populations.<br><br>3. **Reform Proposals**: Suggestions for reform, such as education on implicit bias, hiring diverse law enforcement personnel, and adopting transparency measures, are typical of liberal approaches that focus on systemic change and social equity.<br><br>4. **Focus on Systemic Issues and Cultural Sensitivity**: The recognition of systemic problems and the call for culturally sensitive and anti-racist training are indicative of a liberal viewpoint, which often prioritizes addressing root causes of social issues.<br><br>In summary, the text's emphasis on racial and mental health disparities, systemic reform, and cultural sensitivity aligns it more closely with liberal viewpoints, which prioritize social justice, equity, and systemic change.	liberal
APA	Position Statement on Vaping Products and Electronic Cigarettes	2022	The text provided does not align strictly with either a liberal or conservative viewpoint. It focuses on public health concerns related to the use of electronic cigarettes (e-cigarettes) and vaping, especially among youth and vulnerable populations. The emphasis is on the potential health risks, the need for ongoing research, and the regulation of marketing strategies targeting youth.<br><br>This approach can be associated with public health and regulatory perspectives, which are not inherently political. However, one might observe that:<br><br>- **Liberal Viewpoint:** Generally, liberals might support stricter regulations on e-cigarettes, particularly concerning marketing to youth, as it aligns with their tendency to favor regulations for public health and safety. The emphasis on research and protection of vulnerable groups, including those with mental illnesses, also resonates with liberal values of social welfare and public health.<br><br>- **Conservative Viewpoint:** Conservatives might be cautious about excessive regulation, prioritizing personal freedom and market freedom. They might argue for individual responsibility and less government intervention in private choices, including the use of e-cigarettes. However, the focus on youth protection might resonate with some conservative values, especially those related to family and community welfare.<br><br>In summary, the text reflects a public health perspective that prioritizes research, regulation, and protection of vulnerable populations, aspects that can find support across a wide political spectrum but may align more closely with liberal viewpoints due to the emphasis on regulation and protection of public health.	probably liberal
APA	Position Statement on The Need to Monitor and Assess the Public Health and Safety Consequences of Legalizing Cannabis	2022	The position statement you provided from the American Psychological Association (APA) regarding marijuana legalization and its impact on health and safety is not explicitly aligned with either a liberal or conservative viewpoint. Instead, it focuses on a public health and policy perspective, emphasizing the need for careful monitoring, research, and regulation of marijuana use, especially in states where it's legalized for medical or recreational purposes.<br><br>Key aspects of this position include:<br><br>- Advocacy for coordinated efforts by federal agencies to monitor health and safety consequences of marijuana legalization.<br>- Emphasis on research funding to understand the impact of legalization, particularly on children and adolescents.<br>- Suggestions for modifications to federal laws and policies to ensure proper oversight and to safeguard public health and safety.<br><br>This approach represents a scientifically-informed perspective that seeks to balance the implications of legalization with public health concerns, rather than adhering strictly to typical liberal or conservative ideologies. The APA's focus is on evidence-based policy recommendations, which can sometimes intersect with political viewpoints but are not inherently partisan.	neutral

APA	Position Statement on Direct-to-Consumer Pharmaceutical Advertising	7/2022	The text you've provided outlines various perspectives on direct-to-consumer pharmaceutical advertising (DTCA) and doesn't align strictly with either a liberal or conservative viewpoint. Instead, it presents a nuanced view of the complexities associated with DTCA, including its impact on patient and physician behaviors, the potential benefits of increased awareness about health conditions and treatment options, as well as the concerns about the profit motives of pharmaceutical companies and the importance of evidence-based medical practice.<br><br>The American Psychiatric Association (APA) and the American Medical Association (AMA) are cited, expressing concerns about the impact of DTCA and advocating for a more evidence-based approach to patient care, free from commercial influences. The APA specifically opposes direct-to-consumer advertising of medications prescribed for psychiatric disorders and any prescription medications.<br><br>Overall, the text seems to prioritize patient well-being and informed decision-making in healthcare, advocating for a more cautious and research-informed approach to DTCA. This perspective transcends simple political categorization and reflects a more medically and ethically driven viewpoint.	neutral
APA	Position Statement on Level of Care Criteria for Acute Psychiatric Treatment	7/2022	The text you've provided outlines concerns and positions regarding the improvement of inpatient psychiatric care and utilization review (UR) processes. It doesn't align strictly with a traditional liberal or conservative viewpoint as understood in a political sense. Instead, it focuses on healthcare policy and patient care improvement, which can be supported by individuals across the political spectrum.<br><br>The key issues addressed – such as enhancing patient care, improving access to psychiatric services, and modifying UR processes – are generally healthcare management and policy matters. These issues are more about practical and efficient healthcare delivery rather than ideological positions.<br><br>In the U.S. political context, liberals might be more inclined to support such measures due to their general advocacy for improved healthcare access and quality, and focus on social determinants of health. However, conservatives who are interested in efficient healthcare systems, cost reduction, and improved outcomes might also support these measures. The emphasis on evidence-based criteria, transparency, and patient-centered care can appeal to viewpoints across the political spectrum.<br><br>It's important to note that the approach to healthcare policy can vary significantly within each political ideology, and there are often overlaps in support for specific healthcare improvements.	neutral
APA	Position Statement on the Use of Psychedelic and Empathogenic Agents for Mental Health Conditions	7/2022	The text you've provided doesn't align distinctly with either a liberal or conservative viewpoint. Rather, it presents a scientific and regulatory perspective on the use of psychedelics for psychiatric treatment. The key points in the text are:<br><br>1. **Interest in Psychedelics for Treatment**: It acknowledges the growing interest in psychedelics for treating conditions like treatment-resistant depression and PTSD, stemming from promising preliminary research.<br><br>2. **Classification and Effects of Psychedelics**: The text describes different classes of psychedelics, their effects, and examples of each class, without showing a political bias.<br><br>3. **Regulatory and Research Status**: It highlights that these agents are currently investigational and not yet fully reviewed or approved by the FDA for clinical use. The text emphasizes the importance of regulatory standards and scientific evidence in evaluating these treatments.<br><br>4. **FDA's Role and Breakthrough Therapy Status**: It notes that the FDA has granted breakthrough therapy status to some of these agents, indicating a process of ongoing research and regulatory review.<br><br>5. **Public and Commercial Interest Risks**: The text mentions the risks associated with the use of psychedelics outpacing evidence-based research and regulatory approval, reflecting concerns about public safety and scientific integrity.<br><br>6. **APA's Position**: The American Psychiatric Association (APA) does not endorse the use of psychedelics outside of approved studies and supports research with the same rigor as other medical therapies, a stance focusing on scientific evidence and regulatory compliance rather than a political viewpoint.<br><br>The overall tone is cautious, emphasizing the need for rigorous scientific research and adherence to regulatory standards. This	neutral

			approach is typical of professional and scientific organizations like the APA, which tend to focus on evidence-based practice rather than political ideologies.	
APA	Position Statement on Police Interactions with Children and Adolescents in Mental Health Crisis	7/2022	The text you provided outlines concerns about the safety of children and adolescents in interactions with police, particularly in the context of mental health crises and the disproportionate impact on minority youth. It also includes the American Psychological Association's (APA) position advocating for reforms in law enforcement's response to youth, emphasizing health care and mental health workers as first responders, limits on the use of force against children, mandatory training for law enforcement, and resource allocation to underserved communities.<br><br>This text primarily aligns with a liberal viewpoint for several reasons:<br><br>1. **Emphasis on Mental Health and De-escalation:** The focus on mental health workers as first responders and the call for de-escalation techniques in police interactions with youth is more aligned with liberal perspectives, which often prioritize social services and mental health support over traditional law enforcement methods.<br><br>2. **Concern for Minority Groups:** The attention to the disproportionate impact of police violence on Black and Hispanic children reflects a concern for social justice and racial equality, themes more commonly associated with liberal ideologies.<br><br>3. **Advocacy for Reform and Regulation:** The call for national standards and reform, including limits on police use of force and mandatory training, aligns with a liberal approach that often advocates for increased regulation and reform in various social areas, especially in relation to law enforcement.<br><br>4. **Resource Allocation to Underserved Communities:** The emphasis on breaking the cycle of poverty and criminalization in racial minority communities through resource allocation is in line with liberal policies that focus on equity, social welfare, and addressing systemic inequalities.<br><br>However, it's important to note that while these positions are more typically associated with liberal viewpoints, the concern for the safety and well-being of children and adolescents is a universal one, and there can be conservative perspectives that also support some of these reforms, especially those that emphasize community safety and effective law enforcement practices. The political alignment of such a text can vary depending on the broader context and how these issues are framed within different political ideologies.	liberal

APA	Position Statement on the Impact of Structural Racism on Substance Use and Substance Use Disorders	7/2022	The text you've provided outlines the position of the American Psychiatric Association (APA) regarding substance use disorders (SUDs), focusing on addressing racial inequities and promoting equitable treatment and access to healthcare. This position aligns more closely with a liberal viewpoint for several reasons:<br><br>1. **Emphasis on Equity and Social Justice**: The APA's focus on acknowledging and rectifying past injustices, especially regarding structural racism, aligns with liberal values that prioritize social justice and equity.<br><br>2. **Support for Policy Change**: The support for legislation and policies that improve social and structural determinants of health, and opposition to those perpetuating racial inequities, is a stance typically associated with liberal ideologies, which often advocate for systemic change to address social issues.<br><br>3. **Critique of Existing Drug Policies**: The recognition of racial discrimination in law enforcement and the call for the decriminalization of drug use, along with the elimination of policies leading to disproportionate arrest and incarceration rates, align with liberal perspectives that often critique the criminal justice system and advocate for reform.<br><br>4. **Focus on Multidisciplinary Education and Culturally Informed Research**: The emphasis on education and research that incorporates historical and cultural contexts, particularly regarding substance use and SUDs, is consistent with liberal approaches that value diverse perspectives and inclusivity.<br><br>5. **Advocacy for Equitable Healthcare Access**: Urging healthcare systems to ensure equitable access to treatments, especially for marginalized communities, resonates with liberal viewpoints that emphasize the importance of accessible and fair healthcare for all.<br><br>Overall, the APA's position, as outlined in the text, reflects liberal values of equity, social justice, systemic reform, and inclusivity, particularly in the context of healthcare and the treatment of substance use disorders.	liberal
APA	Position Statement on the Mental Health Needs of Immigrants and People Affected by Forced Displacement	7/2022	The text you've provided is centered around the issue of immigration, with a focus on the mental health needs of immigrants and the stance of the American Psychiatric Association (APA) on treating immigrants with dignity and respect. This viewpoint tends to align more with a liberal perspective, particularly in its emphasis on:<br><br>1. **Humanitarian Approach**: The text emphasizes the need for compassionate treatment of immigrants, asylum seekers, and refugees, underscoring the importance of dignity and respect. This is a hallmark of more liberal viewpoints, which often prioritize humanitarian values in immigration policies.<br><br>2. **Healthcare Access**: Advocating for accessible healthcare, including mental health services, for all immigrants, regardless of their legal status, is more commonly associated with liberal policies. Conservatives might be more inclined to limit such access based on legal status.<br><br>3. **Opposition to For-Profit Detention**: The text's opposition to the for-profit detention of migrants, citing concerns about quality and safety, aligns with liberal critiques of privatization in areas impacting human rights.<br><br>4. **Research and Education**: The call for more research and education on immigrant mental health is consistent with liberal emphases on science and education to inform policy.<br><br>5. **Criticism of Restrictive Policies**: The opposition to laws and practices that are seen as eroding the dignity and respect of immigrants, such as prolonged or inhumane detention conditions, aligns with liberal critiques of conservative immigration policies.<br><br>It's important to note that these categorizations are not absolute, and there can be overlap in viewpoints across the political spectrum. However, based on the key themes and approaches outlined in the text, it leans more towards a liberal viewpoint.	liberal

APA	Position Statement on the Role of Psychiatry in HIV	7/2022	The text you've provided presents an issue and a position statement from the American Psychiatric Association (APA) regarding the comprehensive care of individuals with HIV. It doesn't align strictly with either a liberal or conservative viewpoint as traditionally defined in politics. Instead, it focuses on a public health perspective, emphasizing the need for comprehensive treatment and care for individuals with HIV. The key points include:<br><br>- Collaboration between various health specialists,<br>- Addressing structural racism, bias, stigma, and structural competency,<br>- Acknowledging the interrelation between mental illness and HIV,<br>- Promoting HIV prevention and treatment, including routine testing and risk behavior evaluation,<br>- Continuing research into the effects of HIV on the brain.<br><br>These points are grounded in medical and public health principles rather than a political ideology. They emphasize the importance of a multifaceted approach to HIV treatment and care, recognizing the complex social and medical factors involved. The focus on structural racism, stigma, and the need for collaborative care could be seen as aligning with more progressive or liberal views that emphasize social justice and comprehensive healthcare. However, these are also increasingly recognized as essential elements of effective public health strategies across the political spectrum.	probably liberal
APA	Position Statement on Need to Train Psychiatrists in Provision of Care and Support to Individuals with Disorders of Sex Development and Their Families	2022	The text provided doesn't strictly align with either a liberal or conservative viewpoint in the traditional political sense. It focuses on the medical and psychological aspects of Differences of Sex Development (DSDs) and advocates for a multidisciplinary, patient-centered approach.<br><br>This approach, which emphasizes comprehensive and interdisciplinary care, respect for patient autonomy, and sensitivity to cultural and individual differences, can be seen as aligning with more progressive, patient-centered healthcare philosophies. This perspective values the complexity and diversity of human experiences and conditions, advocating for personalized care rather than a one-size-fits-all approach.<br><br>However, it's important to note that medical and psychological best practices are generally developed based on scientific research and clinical experience, rather than political ideologies. The recommendations made in the text for the treatment of individuals with DSDs, including integrated interdisciplinary teams and increased training and research, are rooted in a desire to improve patient outcomes and well-being, which is a universal concern across the political spectrum.<br><br>In summary, while the text may resonate more with progressive or liberal values of inclusivity, individualized care, and scientific research, it is primarily a reflection of contemporary medical and psychological best practices rather than a political stance.	probably liberal
APA	Position Statement on Misuse of Psychiatric Examinations and Disclosure of Psychiatric Records in Sexual Harassment Litigation	2022	The issue and the American Psychological Association (APA)'s position on mandatory disclosure of psychiatric records or compulsory psychiatric examinations in sexual harassment litigation don't align strictly with either a liberal or conservative viewpoint in a traditional political sense. Instead, they focus on the intersection of legal, ethical, and psychological considerations.<br><br>From a broader perspective, one might argue that the concern for individual privacy, the protection of victims, and the opposition to potential abuse of psychiatric assessments in legal proceedings can be associated with values often emphasized in liberal ideologies. These values include the protection of individual rights, particularly for vulnerable groups, and a cautious approach towards invasive legal practices.<br><br>Conversely, conservative viewpoints often emphasize the importance of established legal procedures and might argue for thorough investigation methods in legal cases, including sexual harassment claims. However, the conservative perspective also values individual privacy and might be concerned about potential overreach in legal processes.<br><br>It's important to note that such issues often transcend simple political categorization, as they involve nuanced considerations of ethics, law, and individual rights that can be valued across the political spectrum. The APA's position seems primarily rooted in a concern for ethical standards in psychological practice and the protection of individual privacy rights, rather than aligning with a specific political ideology.	probably liberal

APA	Position Statement on Health Care	2022	The statement "The United States needs to move towards seeing health care, including mental health care, as a basic human right," and the APA's position that health care, inclusive of mental health care, is a human right, generally aligns more closely with a liberal viewpoint.<br><br>In the United States, liberal perspectives often emphasize the importance of universal access to health care, including mental health services, as a fundamental human right. This viewpoint advocates for the government's role in ensuring that all individuals have access to necessary health care services, regardless of their financial status.<br><br>Conservative viewpoints, on the other hand, tend to prioritize individual responsibility and market-based solutions for health care. They often emphasize limited government intervention in health care markets and may be more inclined to support private sector solutions rather than government-provided health care.<br><br>It's important to note, however, that these are general trends and there can be considerable variation within each political ideology. Additionally, the definitions of 'liberal' and 'conservative' can vary in different countries and political contexts.	liberal
APA	Position Statement Legislative Attempts Permitting Pharmacists to Alter Prescriptions	2022	The text you provided outlines a specific healthcare policy issue in Arkansas, focusing on the modification of laws governing pharmacists' authority to substitute medications within the same therapeutic class without prior explicit approval from the prescriber. It also touches upon concerns related to patient informed consent.<br><br>To determine whether this text aligns more with a liberal or conservative viewpoint, it's essential to understand the typical stances of these political ideologies in the context of healthcare:<br><br>1. **Conservative Viewpoint**: Generally, conservatives favor less government intervention in healthcare, advocating for market-driven solutions and individual choice. They often emphasize the role of private entities and personal responsibility in healthcare decisions. In the context of this issue, a conservative viewpoint might favor the law as it could be seen as reducing regulatory barriers and giving pharmacists more autonomy, potentially leading to more efficient healthcare delivery and reduced costs.<br><br>2. **Liberal Viewpoint**: Liberals typically advocate for more government involvement in healthcare to ensure equity, quality, and accessibility for all. They often support regulations that protect patient rights and safety. Regarding this issue, a liberal perspective might oppose the law, arguing that it could compromise patient safety and informed consent by reducing the direct oversight of prescribing physicians.<br><br>The text seems to align more closely with a liberal viewpoint, as it raises concerns about patient safety, informed consent, and the potential risks of allowing pharmacists to substitute medications without prior approval from the prescriber. These concerns reflect a preference for more regulated and physician-centered approaches to medication management, which is more in line with liberal perspectives on healthcare regulation and patient rights.	liberal
APA	Position Statement on Use of Jails to Hold Persons with Criminal Charges Who Are Awaiting Civil Psychiatric Hospital Beds	2022	The text you've provided represents a position statement from the American Psychiatric Association (APA) regarding the housing of individuals in the process of being civilly committed. It does not align strictly with either a liberal or conservative viewpoint in the traditional political sense. Instead, it reflects a stance based on medical and ethical considerations specific to mental health care.<br><br>The issue at hand is the placement of patients, who are undergoing civil commitment procedures, in jails or correctional facilities instead of therapeutic environments. The APA's position emphasizes that such individuals should be admitted to mental health facilities that are properly staffed and equipped to handle their needs.<br><br>This stance is grounded in concerns about the standard of care for psychiatric patients and the importance of appropriate clinical settings for their treatment. It prioritizes the health and well-being of these individuals over other considerations, such as those related to criminal justice or correctional systems.<br><br>While this position could be seen as aligning with broader progressive values that emphasize humane treatment and healthcare access, it's primarily a professional and ethical stance rather than a political one. It speaks to the best practices in psychiatric care and the rights of patients, rather than aligning with a specific political ideology.	neutral

APA	Position Statement on Remuneration for Psychiatrists' Time Performing Utilization Review	2022	The text provided appears to be a neutral, factual description of the challenges faced by psychiatrists regarding prior authorizations and their impact on the practice of psychiatry. It does not explicitly align with either a liberal or conservative viewpoint. Instead, it focuses on the practical aspects of healthcare administration and the implications for psychiatrists in different work settings.<br><br>The American Psychiatric Association (APA) position stated at the end advocates for fair compensation for physicians for the time spent on case management, supervisory services, and prior authorizations. This stance is centered on ensuring that mental health professionals are adequately compensated for their work, which can be seen as a general healthcare policy issue rather than one inherently aligned with a specific political ideology.<br><br>Overall, the issue and position are more closely related to healthcare administration and practice management, rather than being indicative of a particular political stance.	neutral
APA	Position Statement on Trial and Sentencing of Juveniles in the Criminal Justice System	12/2021	The text provided presents a viewpoint that aligns more closely with liberal ideologies than conservative ones. This alignment is based on several key aspects:<br><br>1. **Emphasis on Rehabilitation and Developmental Understanding**: The text highlights the importance of neurobehavioral understanding of adolescent development and the effectiveness of developmentally grounded interventions. This approach, focusing on rehabilitation and the unique developmental stage of adolescents, is more typical of liberal viewpoints, which often prioritize rehabilitation over punishment, especially for juveniles.<br><br>2. **Reference to Landmark Legal Decisions**: The mention of Supreme Court decisions that ban the juvenile death penalty and restrict life without parole sentences for juveniles reflects a liberal stance on criminal justice, emphasizing the need for age-appropriate sentencing and consideration of developmental factors.<br><br>3. **Opposition to Trying Juveniles as Adults**: The position against trying adolescents in adult criminal courts unless certain stringent conditions are met is indicative of a liberal approach. This perspective values the distinct nature of juvenile courts and the rehabilitation focus they typically embody, as opposed to the more punitive nature of adult criminal courts.<br><br>4. **American Psychiatric Association’s Position**: The APA’s position, as described in the text, advocates for keeping youth within the jurisdiction of the juvenile court system, with an emphasis on treatment and rehabilitation. This approach is more aligned with liberal ideologies that prioritize rehabilitation and treatment over punitive measures, especially for youth offenders.<br><br>In contrast, conservative viewpoints on juvenile justice might emphasize stricter punitive measures, less flexibility in sentencing, and a greater tendency to try juveniles as adults in cases of serious crimes. The focus in conservative approaches is often more on the nature of the crime and public safety rather than on the developmental stage of the offender.	liberal
APA	Position Statement on Telemedicine in Psychiatry	12/2021	The text provided outlines the American Psychiatric Association's (APA) position on telepsychiatry and its potential to enhance access to psychiatric care, particularly for underserved and marginalized communities. It emphasizes the importance of telemedicine in promoting health equity, especially during the COVID-19 pandemic, and outlines recommendations for its effective and ethical implementation.<br><br>This text doesn't neatly align with a traditional liberal or conservative viewpoint, as it primarily focuses on healthcare policy and practice rather than political ideology. However, different aspects of it might resonate more with one side or the other.<br><br>**Liberal Viewpoint Alignment:**<br>1. **Emphasis on Equity and Access:** The focus on increasing access to psychiatric care for underserved populations, including rural, low-income, and LGBTQ+ communities, aligns with liberal values that prioritize social equity and healthcare access.<br>2. **Telehealth Expansion:** Liberals generally support the expansion of telehealth services as part of broader healthcare reforms aimed at increasing accessibility and affordability.<br><br>**Conservative Viewpoint Alignment:**<br>1. **Telehealth as Cost-Efficient:** Conservatives might appreciate the cost-efficiency and convenience of telemedicine, which can reduce overhead costs and improve service delivery.<br>2. **State Licensing and Regulation:** The suggestion to reduce barriers to interstate telepsychiatry practice might resonate with conservative values favoring state autonomy and reduced federal regulation.	neutral

			In summary, while the text does not explicitly align with a specific political ideology, elements of its focus on health equity, technology use, and regulatory recommendations could find support across the political spectrum. The nuanced nature of healthcare policy often transcends simple liberal-conservative dichotomies.	
APA	Position Statement on Sexual Harassment	2021	The text provided appears to be a position statement from the American Psychiatric Association (APA) regarding sexual harassment. It outlines the issue of sexual harassment, provides data on its prevalence, discusses its impact, and states the APA's stance on addressing the issue. The text does not explicitly align with either a liberal or conservative viewpoint; rather, it takes a stance against sexual harassment based on ethical, medical, and social considerations.<br><br>The APA's position is focused on acknowledging the seriousness of sexual harassment, its widespread nature, and the need for effective measures to combat it. This includes encouraging reporting, improving recognition and decreasing incidence through training and education, and facilitating treatment for victims. The approach is grounded in a concern for individual rights, well-being, and a healthy work and learning environment, which are values that can be endorsed across a wide political spectrum.<br><br>In general, discussions around sexual harassment and measures to combat it can be supported by various political ideologies for different reasons. Liberals might focus on the empowerment of marginalized groups and promoting equality, while conservatives might emphasize the importance of maintaining professional and respectful environments in accordance with traditional values. However, the specific text you've shared is more focused on the issue from a professional and health perspective rather than a political one.	neutral
APA	Position Statement on Psychiatric Services in Adult Correctional Facilities	12/2021	The text provided outlines a position on mental health services in the criminal justice system, specifically in U.S. adult correctional facilities. It doesn't strictly align with either a liberal or conservative viewpoint as it addresses a public health and criminal justice issue that can be of concern to both sides of the political spectrum. However, its emphasis on comprehensive mental health services, quality assurance, and the balance between security and treatment could be seen as aligning more closely with certain principles typically associated with liberal viewpoints:<br><br>1. **Focus on Rehabilitation and Mental Health**: Liberals often emphasize rehabilitation and mental health care in the criminal justice system, advocating for treatment over punishment, especially for individuals with mental illnesses.<br><br>2. **Healthcare Accessibility**: The emphasis on accessible and quality mental health care is a hallmark of liberal healthcare policy.<br><br>3. **Human Rights and Dignity**: Advocating for humane treatment and an environment optimized by support and guidance reflects a liberal concern for the rights and dignity of all individuals, including those incarcerated.<br><br>Conversely, conservative viewpoints might focus more on the aspects of security and effective management of correctional facilities, although this does not imply a lack of concern for mental health issues among conservatives. It's important to note that mental health care in prisons can be a bipartisan issue, with both liberals and conservatives recognizing the importance of addressing the mental health needs of the incarcerated population to improve overall public safety and reduce recidivism rates.	probably liberal

APA	Position Statement on Off-Label Treatments	12/2021	The text you've provided discusses the off-label use of FDA-approved drugs and the position of the American Psychological Association (APA) on this issue. It's important to understand that classifying this viewpoint as strictly "liberal" or "conservative" is not straightforward, as the issue intersects with several areas that can be relevant to both political ideologies.<br><br>1. **Patient Autonomy and Physician Authority**: The support for physician autonomy and the ability to prescribe FDA-approved drugs for off-label uses could be seen as aligning with conservative principles, which often emphasize individual choice and less regulatory interference in professional practices.<br><br>2. **Healthcare Regulation and Safety**: The emphasis on safety and effective therapy in off-label drug use can align with liberal perspectives, which often advocate for comprehensive healthcare policies and regulations to ensure patient safety.<br><br>3. **Healthcare Coverage and Reimbursement**: The stance that off-label drug use should be reimbursed by third-party payers, including public ones, aligns more with a liberal viewpoint, which often supports broader healthcare coverage and access.<br><br>4. **Medical Innovation and Research**: The recognition of new uses for drugs through ongoing medical research and practice might be seen as a non-partisan issue, appealing to both sides in terms of promoting scientific advancement and practical healthcare improvements.<br><br>In summary, the viewpoint expressed in the text integrates elements that could resonate with both liberal and conservative ideologies. It addresses physician autonomy (more conservative), patient safety and broad healthcare coverage (more liberal), and the importance of medical innovation (cross-partisan). Therefore, it's a complex issue that doesn't neatly fit into a single political category.	neutral
APA	Position Statement on Location of Civil Commitment Hearings	12/2021	The text you've provided outlines the American Psychiatric Association's (APA) position on the location of civil commitment hearings for psychiatric patients. It's important to note that the text doesn't align strictly with either a liberal or conservative viewpoint in the traditional political sense. Instead, it focuses on practical, ethical, and legal considerations in the treatment and rights of psychiatric patients.<br><br>Here are some key points:<br><br>1. **Patient Dignity and Rights**: Emphasizing the dignity and rights of psychiatric patients aligns with a human rights perspective, which can be embraced by both liberal and conservative viewpoints, albeit for different reasons. Liberals may focus on the rights of vulnerable populations, while conservatives might emphasize the legal and ethical obligations of the state towards individuals in its care.<br><br>2. **Practical Considerations**: The discussion about the practicality of in-hospital versus courthouse hearings doesn't neatly fall into a liberal or conservative category. It's more about logistical efficiency, patient safety, and resource allocation.<br><br>3. **Technological Solutions**: The mention of using technology like video conferencing as a solution is a modern approach that transcends traditional political divides, focusing instead on innovative and cost-effective solutions.<br><br>In summary, the text presents a position based on a blend of ethical, legal, and practical considerations rather than aligning with a specific political ideology. It reflects a multidimensional approach that considers the well-being of psychiatric patients, the legal process, and the efficient use of resources.	neutral

APA	Position Statement on Core Principles for Alternative Payment Models for Behavioral Health	12/2021	The text you provided discusses various aspects of healthcare policy, specifically focusing on mental health and substance use disorders. It's important to note that the concepts and recommendations mentioned don't neatly align with a single political viewpoint, as both liberal and conservative viewpoints may share concerns about healthcare, mental health, and equitable access to treatment.<br><br>However, some elements of the text could be interpreted in light of typical liberal or conservative positions:<br><br>1. **Liberal Viewpoint Aspects**:<br>- Emphasis on health equity and eliminating disparities, particularly among minorities.<br>- Advocacy for improved access to care for underserved populations.<br>- Support for alternative payment models and value-based payments.<br>- Inclusion of telepsychiatry and electronic health record technology, which aligns with a progressive approach to healthcare delivery.<br>- Focus on broad, inclusive, and flexible healthcare policies.<br><br>2. **Conservative Viewpoint Aspects**:<br>- Concerns about government regulations and mandates (e.g., caution about the Medicare Access and CHIP Reauthorization Act of 2015 (MACRA)).<br>- Advocacy for voluntary rather than mandatory participation in Behavioral Health Alternative Payment Models (APMs).<br>- Emphasis on covering the true costs of care, which may resonate with conservative views on fiscal responsibility and efficiency.<br><br>In summary, while some aspects of the text may resonate more with liberal viewpoints, particularly the focus on health equity and broad access, other aspects, such as concerns about government mandates and the emphasis on fiscal responsibility, could align with conservative principles. The text presents a nuanced view that incorporates elements relevant to both perspectives.	neutral
APA	Position Statement on College and University Mental Health	12/2021	The text provided outlines the need for improved mental health services on college and university campuses and presents the American Psychological Association's (APA) position on this issue. It doesn't align specifically with either a liberal or conservative viewpoint. Instead, it addresses a universal concern regarding mental health among college students, an issue that transcends traditional political divisions.<br><br>Mental health, in general, is a topic that receives attention across the political spectrum, though the approaches to addressing it may vary. Liberals often advocate for increased funding for mental health services, broader insurance coverage, and more inclusive policies, while conservatives may focus on personal responsibility, family and community support systems, and private sector solutions. However, the emphasis in this text on the need for accessible mental health services, comprehensive health insurance coverage, and the integration of mental health care into the educational system can be seen as aligning with principles commonly associated with more progressive or liberal viewpoints, particularly in the emphasis on systemic solutions and insurance coverage.<br><br>However, it's important to note that mental health issues, especially in the context of young adults and educational settings, are increasingly viewed as non-partisan, with a growing consensus on the importance of addressing these concerns for the betterment of society as a whole.	probably liberal

APA	Position Statement on Any Willing Physician	12/2021	The text you've provided outlines a position by the American Psychological Association (APA) regarding healthcare policy, specifically related to "any willing physician" legislation. It's important to understand that issues related to healthcare policy can be complex and do not always align neatly with traditional liberal or conservative viewpoints. However, I can provide some insights into how aspects of this position might be viewed from both perspectives:<br><br>1. **Liberal Viewpoint:**<br>- Liberals generally support increased access to healthcare and patient freedom of choice. The APA's recognition of the importance of patient freedom-of-choice aligns with this perspective.<br>- They often advocate for more inclusive healthcare policies, which could be seen in the APA's support for legislation requiring payers to contract with any licensed physician willing to meet their terms.<br>- However, liberals also tend to support more regulation in healthcare, which could conflict with the idea of "any willing physician" if it is perceived as reducing the oversight on the quality of care.<br><br>2. **Conservative Viewpoint:**<br>- Conservatives typically emphasize free market principles in healthcare. The concept of "any willing physician" could align with this by potentially increasing competition among providers.<br>- They often favor less government intervention in healthcare, which might make them supportive of the idea that payers should not be overly restricted in whom they contract with.<br>- However, conservatives might also be concerned about the implications of such legislation on healthcare costs and the quality of medical care.<br><br>In summary, the APA's position has elements that could be supported by both liberal and conservative viewpoints, depending on how one prioritizes aspects like patient choice, market freedom, healthcare access, and regulation. It's essential to recognize that healthcare policies often transcend simple political categorizations and can be supported or opposed for various reasons by individuals across the political spectrum.	neutral
APA	Position Statement on Immigration, Children, Adolescents, and their Families	12/2021	The text you provided discusses the psychosocial impact of migration, specifically focusing on the mental health and well-being of child and adolescent migrants, and critiques certain U.S. immigration policies. This perspective aligns more closely with a liberal viewpoint for several reasons:<br><br>1. **Emphasis on Humanitarian Concerns**: The text highlights the detrimental psychological and developmental impacts of migration policies on children and adolescents. This focus on the humanitarian aspect, especially concerning vulnerable populations like children, is often associated with liberal viewpoints.<br><br>2. **Critique of Strict Immigration Enforcement**: The text criticizes the "zero-tolerance" policy and the forcible separation of children from their parents. Such criticism is typically a feature of liberal perspectives, which often advocate for more compassionate and less punitive approaches to immigration.<br><br>3. **Support for Comprehensive Healthcare and Asylum Policies**: The APA's position, as outlined in the text, supports healthcare access, pathways to asylum, and measures to ensure the safety and dignity of families fleeing danger. These policy recommendations are in line with liberal ideologies that emphasize inclusivity, healthcare access, and protection of rights for asylum seekers and immigrants.<br><br>4. **Opposition to Prolonged Detention and Inhumane Treatment**: The stance against prolonged detention and inhumane treatment of migrants, especially children, aligns with liberal views that often advocate for humane treatment and criticize detention practices.<br><br>It's important to note that while these points align with a liberal viewpoint, the issues of immigration and migrant welfare can be complex and may be viewed through various lenses across the political spectrum. The focus here is on the well-being and rights of migrants, particularly children, which is typically a concern more emphasized in liberal discourses.	liberal

APA	Position Statement on Mental Health Impact of Public Health Emergencies on Young People	12/2021	The text you've provided seems to focus on the impact of public health emergencies on children and adolescents, especially those from underserved and marginalized backgrounds, and the need for increased screening and support for these groups. This perspective aligns more closely with a liberal viewpoint, as it emphasizes:<br><br>1. **Social Equity and Vulnerable Populations**: It highlights the disproportionate impact of public health emergencies on underserved populations, a concern typically associated with liberal ideologies, which often focus on social equity and support for marginalized groups.<br><br>2. **Structural Factors**: The mention of addressing structural racism and its effects, such as housing instability and food insecurity, reflects a liberal approach that looks at systemic issues and their impact on individuals and communities.<br><br>3. **Public Health and Mental Health Initiatives**: The emphasis on public health initiatives, like tele-mental health and school-based mental health services, aligns with a more liberal viewpoint that advocates for broad-based public health solutions and the importance of mental health care.<br><br>4. **Childhood Development and Trauma**: The focus on adverse childhood experiences (ACEs) and their long-term impact on mental and physical health is a concern that is often more emphasized in liberal policy discussions.<br><br>However, it's important to note that the distinction between liberal and conservative viewpoints is not always clear-cut, and there can be overlap in concerns and solutions proposed by both sides. The focus on children's wellbeing and public health can be a point of bipartisan agreement, though the methods and emphasis might differ between ideologies.	liberal
APA	Position Statement on Racism and Racial Discrimination in the Psychiatric Workplace	12/2021	The text you've provided seems to align more closely with a liberal viewpoint, primarily due to its focus on acknowledging and addressing systemic racism, especially in healthcare settings and workplaces. The emphasis on recognizing and addressing hate speech and discriminatory treatment aligns with liberal perspectives that typically advocate for social justice, equity, and the active addressing of systemic issues in society.<br><br>Conservative viewpoints, while they may recognize the existence of racism, often focus more on individual responsibility and merit-based systems, and might be less inclined to emphasize systemic racism as a pervasive issue. They may also prioritize different approaches to addressing such problems, focusing more on individual actions and less on systemic changes.<br><br>It's important to note that these are general trends and there can be considerable variation within each political spectrum. Political viewpoints are complex and multifaceted, and individuals may hold a mix of liberal and conservative views on different issues.	liberal
APA	Position Statement on Moral Injury Among Healthcare Workers During a Public Health Crisis	12/2021	The text you've shared presents a nuanced perspective on the challenges faced by healthcare workers during the COVID-19 pandemic and advocates for specific policies to support them. This perspective and the proposed solutions do not align strictly with either a liberal or conservative viewpoint, as they encompass aspects that could be supported by both sides.<br><br>1. **Emphasis on Public Health Measures**: The call for implementing public health strategies like contact tracing, testing, travel restrictions, and social distancing mandates is typically more aligned with a liberal viewpoint, which often prioritizes government intervention in public health crises.<br><br>2. **Public Funding for Healthcare Workers**: Advocating for public funding to support healthcare workers can also be seen as more liberal, as it suggests government responsibility for individual healthcare professionals' well-being.<br><br>3. **Expanding Grant Funding for Healthcare Institutions**: This point could be seen as more neutral or bipartisan, as both liberal and conservative viewpoints can support funding healthcare institutions, albeit for different reasons.<br><br>4. **Reaffirming Scientific Evidence in Public Health Measures**: The emphasis on science and evidence-based approaches in policymaking is often associated with liberal viewpoints, although it is not exclusively liberal.<br><br>5. **Support for Healthcare Clinicians through Public Messaging**: This aspect is quite neutral and can be endorsed by both liberals and conservatives, as it emphasizes the importance of public support for healthcare workers.<br><br>Overall, while some aspects of the text align more with liberal viewpoints (particularly the emphasis on public health measures and government funding), the overall focus on supporting healthcare workers and ensuring public health can be considered a broadly	probably liberal

			bipartisan or centrist concern. The text does not strongly advocate for ideological positions typically associated with conservative viewpoints, such as reduced government intervention or prioritizing economic considerations over public health measures.	
APA	Position Statement on Medical Supervision of Psychiatry Residents and Fellows	12/2021	The text you provided presents a position statement from the American Psychiatric Association (APA) regarding the supervision of psychiatry residents and fellows. This statement emphasizes the APA's stance that psychiatry residents and fellows should be supervised by psychiatrists, particularly in areas like psychiatric diagnostic evaluations, managing psychiatric emergencies, and medication management, rather than solely by advanced practice providers such as nurse practitioners or physician assistants.<br><br>This position doesn't neatly align with traditional liberal or conservative viewpoints as understood in a political context. Instead, it's more focused on professional standards and training in the field of psychiatry. The concern here is primarily about ensuring appropriate training and supervision for psychiatric residents and fellows, which is more of a professional and educational issue rather than a political one.<br><br>However, if one were to try and fit this into a political framework, it might touch upon broader themes that each political side could interpret differently:<br><br>1. **Conservative Viewpoint**: Conservatives might support this statement as it emphasizes traditional hierarchies and structures in medical training and professionalism. It could be seen as an endorsement of established medical professionals (psychiatrists) over newer roles (nurse practitioners, physician assistants).<br><br>2. **Liberal Viewpoint**: Liberals might focus on the implications for healthcare accessibility and the role of advanced practice providers in expanding access to mental health care. They might argue for the value and efficacy of diverse healthcare professionals in various roles, including supervision and training.<br><br>However, it's important to note that these interpretations are quite broad and might not accurately represent the nuanced views within each political spectrum, especially as this issue is more about medical education and professional standards than about political ideology.	neutral

APA	Position Statement on Civil Commitment of Minors	12/2021	The issue described in the text revolves around the American Psychiatric Association's (APA) Position Statement on the hospitalization of minors with mental illness, particularly in situations where consent is either not given or cannot be given. The position advocates for a similar approach to both minors and adults regarding voluntary and involuntary hospitalization, with an emphasis on aligning commitment standards and procedures for both groups.<br><br>Determining whether this text aligns with a liberal or conservative viewpoint is not straightforward, as it intersects with various aspects of both political ideologies:<br><br>1. **Liberal Viewpoint**: Liberals generally emphasize individual rights and protections, especially for vulnerable populations like minors. They might support such a position if they believe it protects the rights and wellbeing of minors with mental illness, ensuring they receive necessary care even in the absence of consent. Additionally, liberals often advocate for equitable treatment across different age groups, which aligns with the APA's stance of not significantly differentiating between minors and adults in this context.<br><br>2. **Conservative Viewpoint**: Conservatives typically prioritize family rights and parental authority in decision-making for minors. Some conservatives might oppose the APA's position if they view it as an overreach that diminishes parental control or interferes with family autonomy. However, if the emphasis is on maintaining societal order and ensuring the wellbeing of individuals who cannot make decisions for themselves, some conservatives might agree with the approach, especially in cases where the lack of intervention could lead to harm.<br><br>The overlap in these perspectives indicates that this issue is complex and can be interpreted differently depending on one's emphasis on individual rights, family authority, and the need for societal intervention in healthcare. This complexity makes it challenging to categorically align the statement with either a liberal or conservative viewpoint without additional context or interpretation.	neutral
APA	Position Statement on Discharge from Hospital-Based Care to the Community for Substance Use Disorders	12/2021	The text provided outlines a series of recommendations and positions regarding the treatment of individuals with substance use disorders (SUDs), particularly focusing on the importance of transitional care plans, evidence-based treatment options, and comprehensive care that extends beyond detoxification. This approach is generally aligned with a healthcare perspective that emphasizes evidence-based, patient-centered treatment and the integration of medical, psychosocial, and support services.<br><br>In terms of political alignment, this approach may not neatly fit into a traditional liberal or conservative viewpoint. However, elements of this approach can be associated with certain political perspectives:<br><br>1. **Liberal Viewpoint**: The emphasis on comprehensive, evidence-based treatment and the integration of various support services (such as peer-support and patient navigators) could align with liberal perspectives, which often advocate for extensive healthcare services and support systems. Liberals typically support increased funding for healthcare services and the expansion of treatment options for individuals with health issues, including SUDs.<br><br>2. **Conservative Viewpoint**: Some aspects of this approach could align with conservative values, such as the emphasis on informed decision-making and autonomy for patients. Conservatives often value personal responsibility and might support approaches that empower individuals to make informed choices about their treatment. However, conservatives might be more cautious about the extent of government involvement or funding required to implement such comprehensive care models.<br><br>It's important to note that the approach described in the text is primarily clinical and focuses on what is considered effective and evidence-based in the field of addiction treatment. The alignment with political viewpoints might be more about how such programs are funded and implemented rather than the clinical recommendations themselves.	neutral

APA	Position Statement on Orchiectomy or Treatment with Anti-Androgen Medications as a Condition of Release from Incarceration	7/2021	Determining whether a particular viewpoint aligns with a liberal or conservative ideology can be complex, as it often depends on the specific aspects of the issue and the context in which it is being discussed. Here's an analysis from both perspectives:<br><br>### Liberal Viewpoint<br>- **Human Rights and Ethical Concerns:** Liberals often prioritize human rights and individual autonomy. The opposition to mandatory orchiectomy or anti-androgen treatments as a condition for release from incarceration might be seen as a stance that aligns with liberal values. Liberals might argue that such policies infringe on personal rights and bodily autonomy.<br>- **Medical and Ethical Best Practices:** Liberals generally advocate for decisions based on scientific and medical expertise. The concern that the law does not adequately consider medical indications and contraindications, and lacks provisions for psychiatric assessments, could align with a liberal viewpoint that emphasizes evidence-based policy.<br>- **Opposition to Coercive Treatment:** Liberals are likely to oppose coercive medical treatments, especially those linked to criminal justice decisions, as they may view these practices as unethical and a violation of civil liberties.<br><br>### Conservative Viewpoint<br>- **Public Safety and Law Enforcement:** Conservatives often emphasize law and order and public safety. Some conservatives might support the use of orchiectomy or anti-androgen treatments for certain sex offenders as a means to protect the public, especially from repeat offenders.<br>- **Tough on Crime Approach:** A conservative stance might favor stringent conditions for the release of individuals convicted of serious offenses, viewing such measures as necessary for the protection of society.<br>- **Cost Considerations:** The aspect of individuals covering the cost of their treatment might align with conservative views on personal responsibility and reducing state expenditures.<br><br>### Neutral Considerations<br>- **Medical Autonomy vs. Public Safety:** This issue presents a complex interplay between medical ethics, human rights, and public safety concerns. Both liberals and conservatives might have varied opinions within their groups based on how they prioritize these factors.<br>- **Legal and Ethical Precedents:** The comparison with practices in other Western countries introduces an international perspective, which might influence viewpoints across the political spectrum.<br><br>In conclusion, while aspects of the issue might align more closely with traditional liberal or conservative viewpoints, the complexity of the matter allows for a range of opinions within each ideological group. The APA's position, focusing on medical expertise, informed consent, and ethical practice, can resonate with elements of both liberal and conservative philosophies, particularly where they overlap with concerns for human rights and ethical medical practice.	neutral
APA	Position Statement on Misogyny and Gender Bias and Their Adverse Effects on the Health of Women	3/2021	The text provided appears to align more closely with a liberal viewpoint. Liberal perspectives often emphasize social equality, support for marginalized groups, and a focus on addressing systemic issues like gender bias and discrimination. The points mentioned in the text, such as recognizing the disproportionate impact of misogyny and gender bias on women, particularly those from marginalized groups, and advocating for equitable treatment in healthcare and social institutions, are consistent with liberal values that prioritize social justice and equality.<br><br>Conservative viewpoints, on the other hand, might focus more on traditional social structures and might not emphasize systemic change to the same extent. However, it's important to note that views on gender equality and combating misogyny can vary within both liberal and conservative circles, and the text does not represent all individuals within these groups.<br><br>The American Psychiatric Association's (APA) position, as outlined, advocates for condemnation of misogyny, opposition to gender bias, recognition of the impacts of discrimination, and commitment to equity and justice. These stances align with a progressive approach to social issues, emphasizing the importance of acknowledging and addressing systemic inequalities and biases.	liberal

APA	Position Statement on the Primacy of the Treating Psychiatrist's Responsibility to the Patient	4/2021	The text provided is primarily focused on ethical principles in medical and psychiatric practice, specifically highlighting the importance of prioritizing patient care and addressing potential conflicts of interest. It does not explicitly align with either a liberal or conservative viewpoint as it centers on professional ethics and responsibilities within the medical field.<br><br>The principles emphasized — respect for persons, beneficence, fairness, trust, humility, honesty, intellectual integrity, compassion, and competence — are universal in the context of healthcare and transcend political ideologies. The emphasis is on the physician's duty to prioritize patient care over other interests, including those that may arise from dual agency or institutional roles.<br><br>The American Psychiatric Association's position reinforces the idea that a psychiatrist's primary responsibility is to their patient, even when facing conflicting obligations from other entities or individuals. This stance is rooted in the ethical practice of medicine and psychiatry rather than in political ideology.<br><br>In summary, the text outlines ethical standards and professional responsibilities in healthcare, focusing on patient-centered care and conflict resolution in the physician-patient relationship, without aligning with a specific political viewpoint.	neutral
APA	PositionStatement on Psychiatrist Input into the Design and Construction of Psychiatric Evaluation and Treatment Facilities	7/2020	The text provided outlines a position on including psychiatrists in the planning and design of psychiatric facilities, as advocated by the American Psychiatric Association (APA). This position focuses on improving patient safety and care quality in psychiatric settings.<br><br>Regarding its alignment with political viewpoints:<br><br>1. **Liberal Viewpoint**: This text could align with a liberal viewpoint insofar as it emphasizes patient safety, quality of care, and the inclusion of professional expertise in healthcare planning. Liberals often advocate for comprehensive healthcare reforms that prioritize patient welfare and involve experts in policy and facility design. The focus on vulnerable populations, like those with major mental disorders, is also a common feature of liberal healthcare policies.<br><br>2. **Conservative Viewpoint**: The text might not align as closely with traditional conservative viewpoints, which often emphasize less regulation and more market-driven approaches to healthcare. However, conservatives do value effective management and could support the notion of including relevant expertise (psychiatrists in this case) in facility design to enhance efficiency and quality of care. The emphasis on safety and quality can appeal to conservative values of efficiency and practicality in healthcare delivery.<br><br>In summary, while the text may more directly resonate with liberal perspectives on healthcare, particularly in its focus on patient welfare and expert involvement, aspects of it can also appeal to conservative values of efficiency and quality in healthcare systems. It's important to note that healthcare issues often transcend simple left-right political divisions, with various aspects appealing to different ideologies.	probably liberal

APA	Position Statement on Xenophobia, Immigration, and Mental Health	7/2020	The text you've provided is a position statement from the American Psychiatric Association (APA) regarding xenophobia and its consequences. It's important to note that classifying such statements as strictly "liberal" or "conservative" can be reductive and overlook the complexity of the issues and viewpoints.<br><br>However, we can analyze the text for themes that are often associated with liberal or conservative viewpoints:<br><br>1. **Emphasis on the Rights of Immigrants and Refugees**: The text's focus on protecting the rights of immigrants, refugees, and asylum seekers, as well as promoting cultural diversity and competence, is more commonly associated with liberal viewpoints. Liberals typically advocate for inclusive policies towards immigrants and emphasize the importance of cultural diversity.<br><br>2. **Condemnation of Xenophobia**: The strong stance against xenophobia and the call for public education on cultural competence and diversity aligns with liberal ideologies that often prioritize social justice and anti-discrimination efforts.<br><br>3. **Policy Recommendations Based on Objective Data**: The call for rational, data-driven policy debates, especially in areas like immigration and foreign relations, is a principle that can be embraced by both liberal and conservative viewpoints. However, the specific context of advocating for objective debates in the context of national and ethnic diversity may be more aligned with liberal perspectives.<br><br>4. **Mental Health and Public Health Consequences**: The emphasis on the mental health and public health consequences of xenophobia and prejudice, including the specific mention of adverse effects on immigrants and marginalized populations, tends to align with liberal viewpoints, which often prioritize public health and welfare concerns.<br><br>In summary, while the APA's position incorporates aspects that could resonate with both liberal and conservative viewpoints, the overall emphasis on protecting the rights of immigrants and refugees, condemning xenophobia, and promoting cultural diversity and competence is more typically associated with liberal ideologies.	liberal
APA	Position Statement on Voluntary and Involuntary Hospitalization of Adults with Mental Illness	7/2020	The text you've provided appears to be a balanced and comprehensive policy statement from the American Psychiatric Association (APA) regarding psychiatric hospitalization. It doesn't align strictly with either a liberal or conservative viewpoint as it encompasses aspects that both sides might support or have concerns about.<br><br>1. **Liberal Viewpoint Alignment**:<br>- The emphasis on recovery-oriented, person-centered, trauma-informed, and community-based treatment aligns with liberal values that focus on individual rights and comprehensive, compassionate care.<br>- The insistence on legal representation and periodic judicial reviews for those involuntarily hospitalized respects civil liberties, a principle often championed by liberals.<br>- The principle of the least restrictive alternative and the provision of care regardless of ability to pay also align with more progressive social policies.<br><br>2. **Conservative Viewpoint Alignment**:<br>- The acknowledgment of the need for involuntary hospitalization in certain cases aligns with conservative views emphasizing public safety and order.<br>- The emphasis on clear and convincing evidence for continued hospitalization and the involvement of multiple medical professionals might be seen as aligning with a more conservative approach to institutional authority and procedural rigor.<br>- The focus on mental disorders as defined in the Diagnostic and Statistical Manual of the American Psychiatric Association (not including solely Intellectual Developmental Disorder or Substance Use Disorder) might align with a conservative preference for clear, established criteria in policy.<br><br>In summary, this statement seems to be a carefully considered, ethically informed approach that seeks to balance individual rights with public safety and clinical necessity. It reflects a professional and nuanced understanding of the complexities involved in psychiatric care, rather than a straightforward alignment with a political ideology.	neutral

APA	Position Statement on Use of the Terms Client and Provider	12/2020	The text provided does not align strictly with a traditional liberal or conservative viewpoint. Instead, it addresses a matter of terminology in the healthcare sector, specifically in the context of the patient-physician relationship. The issue at hand is more about professional and ethical considerations within the medical community rather than a matter of political ideology.<br><br>The focus of the statement is on the perceived inaccuracies and implications of using the terms "provider" and "client" in healthcare. It argues that these terms might lead to misunderstandings about the nature of the healthcare professional's training and the relationship between physicians and patients, suggesting a more commercial rather than medical perspective.<br><br>The American Psychiatric Association's (APA) position, as described, advocates for discontinuing the use of these terms in their communications, reflecting a preference for terminology that more accurately represents the medical and professional nature of their work. This viewpoint is more aligned with concerns about professional standards, language precision, and patient perception in healthcare, rather than with political leanings.	neutral
APA	Position Statement on Universal Health Care in the United States	7/2020	The text you provided reflects a viewpoint that aligns more closely with liberal ideologies. This alignment is evident in the emphasis on universal access to healthcare, including mental health and substance use disorder treatment, and the assertion that healthcare is a human right. These positions are typically associated with liberal or progressive perspectives, which often prioritize expanded access to healthcare services, inclusivity, and the reduction of disparities in healthcare access.<br><br>Key aspects of this viewpoint that align with liberal ideologies include:<br><br>1. **Universal Health Care Access**: Advocating for universal health care coverage is a hallmark of liberal policy, emphasizing the right of every individual to receive healthcare services.<br><br>2. **Mental Health Parity**: The emphasis on equal treatment for mental health and substance use disorders as compared to physical illnesses reflects a progressive approach to healthcare, focusing on holistic treatment and the elimination of stigma associated with mental health.<br><br>3. **Opposition to Utilization Management Protocols**: The stance against restrictive protocols such as lifetime limits and pre-existing condition denials is consistent with liberal positions that seek to reduce barriers to healthcare access.<br><br>4. **Emphasis on Patient-Centered Care**: The focus on patient and family-centered, culturally sensitive, and community-based care aligns with liberal values that prioritize individual rights, diversity, and community welfare.<br><br>5. **Addressing Disparities**: The call to address disparities in access and quality of care for ethnic and racial minorities is a key component of progressive agendas that emphasize equality and social justice.<br><br>6. **Privacy and Confidentiality in Electronic Health Records**: The balance between the benefits of electronic health records and the protection of patient privacy reflects a nuanced approach to healthcare technology, consistent with liberal values of individual rights and privacy.<br><br>It's important to note that while these positions are more commonly associated with liberal ideologies, healthcare is a complex issue that can transcend traditional political boundaries. Different aspects of healthcare policy can find support across the political spectrum for various reasons.	liberal

APA	Position Statement on Treatment of Transgender (Trans) and Gender Diverse Youth	7/2020	The text you've provided outlines various aspects of gender-affirming treatment for transgender and gender non-conforming youth, including the use of puberty blockers and hormone therapy. It also mentions the stance of the American Psychiatric Association (APA) on these treatments.<br><br>The viewpoint expressed in the text aligns more closely with a liberal viewpoint. Liberal perspectives generally emphasize the importance of individual rights and freedoms, including the rights of transgender and gender diverse individuals to receive gender-affirming care. This viewpoint often supports the use of medical interventions for transgender youth, recognizing the benefits of such treatments for mental health and overall well-being.<br><br>In contrast, a conservative viewpoint might emphasize more traditional or biological understandings of gender and might be more cautious or skeptical about the use of medical interventions, especially for minors. Conservative perspectives often raise concerns about the long-term effects of such treatments and the ability of young people to make decisions about gender-affirming care.<br><br>It's important to note that these are general trends, and there can be a wide range of opinions within both liberal and conservative circles. The issue of gender-affirming care for transgender youth is complex and often involves considerations of medical ethics, children's rights, parental rights, and public policy.	liberal
APA	Position Statement on the Use of Antipsychotic Medication in Patients with Major Neurocognitive Disorder	7/2020	The text you've provided is a medical and ethical discussion regarding the treatment of Major Neurocognitive Disorder (MNCD), specifically addressing the use of antipsychotic medications versus non-pharmacologic approaches. This issue primarily revolves around medical, ethical, and patient care considerations rather than aligning directly with liberal or conservative political viewpoints.<br><br>Liberal and conservative viewpoints in politics typically differ on issues like government intervention, social welfare, economic policies, and individual freedoms. In contrast, the text you've provided is focused on clinical practices, patient safety, and the efficacy of treatment options in a specific medical context. It discusses the necessity of balancing the benefits and risks of antipsychotic medications in treating MNCD, emphasizes the importance of informed consent, and acknowledges the limitations of current treatment options.<br><br>In summary, the text is more aligned with medical ethics and best practices in healthcare rather than being indicative of a particular political ideology.	neutral
APA	Position Statement on the Impact of Cannabis on Children and Adolescents	12/2020	The text presents a nuanced view that incorporates aspects often associated with both liberal and conservative viewpoints, making it difficult to classify it strictly under one category.<br><br>From a **liberal perspective**:<br>1. Acknowledgement of the potential medical benefits of cannabis, especially for life-threatening and severely debilitating conditions.<br>2. Support for further research into the therapeutic components of cannabis.<br>3. Concern about the risks associated with cannabis use, especially in young people.<br>4. Advocacy for comprehensive policies to regulate marketing and advertising to protect children and adolescents.<br><br>From a **conservative perspective**:<br>1. Opposition to the use of cannabis by children, adolescents, and young adults up to age 25, based on the lack of scientific support for safe use and known adverse impacts.<br>2. Caution against the rapid expansion of medical marijuana use without FDA approval and regulation.<br>3. Emphasis on the risks of addiction and the potential for neuropsychiatric symptoms, including psychosis.<br>4. Recommendation for strict enforcement of regulations and accountability for manufacturers.<br><br>Overall, the statement appears to strive for a balanced approach, recognizing the potential benefits of cannabis while also highlighting the risks and advocating for regulatory oversight and further research. This approach is typical of professional organizations like the American Psychiatric Association (APA), which often aim to base their positions on evidence and concern for public health rather than aligning strictly with political ideologies.	neutral

APA	Position Statement on Suicide Among Black Youth in the United States	12/2020	The text provided doesn't align strictly with either a liberal or conservative viewpoint. Rather, it presents factual information and research findings regarding the rates of suicide among Black American youth, along with recommendations from the American Psychiatric Association (APA) on addressing this public health concern.<br><br>The focus of the text is on:<br>1. Highlighting the alarming increase in suicide rates among Black American youth.<br>2. Identifying the need for further research and effective interventions tailored to this group.<br>3. Emphasizing the importance of addressing health disparities, structural barriers in accessing treatment, and the impact of social determinants on mental health.<br><br>Such content is primarily concerned with public health, mental health, and racial disparities in these areas. It is factual and research-based, aimed at raising awareness and prompting action regarding a specific public health issue. While discussions of health disparities and structural barriers might resonate more with liberal perspectives that often emphasize systemic issues and social determinants of health, the focus on public health and community well-being transcends political boundaries and is relevant to both conservative and liberal viewpoints.<br><br>The APA's position, as stated, is grounded in a scientific and health-focused approach rather than a political one. Their recommendations for increased funding, removing barriers to treatment, and exploring social determinants are aimed at improving health outcomes and addressing a specific crisis. This approach is in line with public health principles and does not inherently align with a specific political ideology.	neutral
APA	Position Statement on Sexual Abuse of Migrants in ICE Custody	12/2020	The text you've provided doesn't explicitly align with a specific liberal or conservative viewpoint. Instead, it focuses on reporting facts and statements from various sources, including media outlets, U.S. government agencies, and the American Psychiatric Association (APA). The information presented includes:<br><br>1. **Statistics on Allegations of Sexual Abuse**: It details the number of allegations of sexual abuse of migrants while in ICE custody, citing specific figures and time frames from sources like the New York Times and the Office of Refugee Resettlement.<br><br>2. **Mental Health Impact**: The text discusses the higher rates of mental health conditions such as post-traumatic stress disorder and depression among migrant children and their families, attributing these to pre-migration trauma and risks faced during migration.<br><br>3. **APA's Position**: The American Psychiatric Association's stance is clearly stated, opposing detention practices that increase the risk of sexual abuse and supporting measures to ensure the safety and humane treatment of migrant families.<br><br>Each of these points is presented as factual information or the stance of a professional organization, rather than being framed within a political ideology. However, the topics discussed—such as the treatment of migrants, the conditions in detention centers, and the mental health of vulnerable populations—are often subjects of political debate, with different aspects emphasized by liberal and conservative viewpoints. Liberals typically focus on humanitarian concerns and the rights of migrants, while conservatives may emphasize border security and immigration law enforcement. The text you've provided, however, remains neutral and factual in its presentation.	neutral

APA	Position Statement on Second Generation Antipsychotic Medications	7/2020	The text you provided is a position statement related to healthcare, specifically focusing on the availability of second-generation antipsychotic medications for treating psychosis spectrum disorders. It does not clearly align with a traditional liberal or conservative viewpoint as it primarily addresses a healthcare issue rather than a political ideology.<br><br>In the context of American politics:<br><br>- **Liberal Viewpoint**: Generally, liberals advocate for comprehensive healthcare access and might support such a position as it emphasizes the importance of making effective treatments widely available, including in public institutions like jails and youth facilities. They often focus on public health and equitable access to healthcare.<br><br>- **Conservative Viewpoint**: Conservatives might support this position from the perspective of efficient healthcare financing and the effectiveness of treatments. However, they might also have concerns about the cost implications for private insurers and the mandate for such widespread availability across all systems, including correctional facilities, depending on their views about government involvement in healthcare.<br><br>It's important to note that healthcare issues can transcend simple liberal-conservative divides, especially when they are focused on clinical outcomes and evidence-based practices. The statement you provided is more centered around medical consensus and best practices in healthcare rather than being directly tied to a political ideology.	neutral
APA	Position Statement on Screening and Treatment of Mood and Anxiety Disorders During Pregnancy and Postpartum	12/2020	The text you've shared primarily presents a clinical and public health perspective on perinatal mental health disorders. It focuses on the incidence, impact, and recommendations for screening and treatment of psychiatric illnesses during pregnancy and the postpartum period. This text aligns more with a public health and medical viewpoint rather than a strictly political one.<br><br>However, its implications and recommendations can be interpreted differently based on liberal or conservative viewpoints:<br><br>1. **Liberal Viewpoint**: A liberal perspective might emphasize the need for comprehensive healthcare policies, including mental health support for pregnant and postpartum women. Liberals may advocate for increased funding for public health programs, greater access to healthcare services, and support for research in this area. They might also stress the importance of addressing social determinants of health, like socioeconomic status and domestic violence, as factors contributing to these mental health issues.<br><br>2. **Conservative Viewpoint**: Conservatives might focus on the role of individual responsibility and the importance of family and community support systems in addressing these health issues. They may advocate for solutions that involve less government intervention and more reliance on private healthcare providers. There might also be a focus on the role of traditional family structures in providing support for women during the perinatal period.<br><br>Both viewpoints would likely agree on the importance of addressing these mental health issues but might differ in their approaches to policy and funding. The text itself, however, is neutral and focused on clinical facts and recommendations for healthcare providers.	neutral

APA	Position Statement on Reducing the Burden of Treatment Plan Documentation	12/2020	The text provided reflects a viewpoint that prioritizes reducing administrative burdens for healthcare professionals, particularly psychiatrists, and emphasizes the impact of excessive documentation on patient care quality. This stance aligns more closely with liberal viewpoints in several ways:<br><br>1. **Emphasis on Healthcare Access and Quality**: The liberal viewpoint often focuses on ensuring access to quality healthcare and reducing barriers to it. The text's concern about the negative impact of excessive documentation on access to quality psychiatric care resonates with this liberal emphasis.<br><br>2. **Critique of Overregulation**: While conservative viewpoints also criticize overregulation, the specific context here is important. The criticism is leveled at regulations that are seen as disproportionately affecting mental health services compared to other medical services. This reflects a liberal concern for the unique challenges in mental health care, a field that often receives less attention and resources.<br><br>3. **Support for Mental Health Services**: Liberals typically advocate for strong support of mental health services, recognizing the importance of mental health in overall healthcare. The text's call to align the documentation requirements of psychiatric visits with those of other medical settings reflects a desire to normalize and integrate mental health care into the broader healthcare system.<br><br>However, it's important to note that the viewpoint also touches on themes commonly found in conservative perspectives, such as reducing unnecessary regulations. The call to eliminate what is seen as bureaucratic overreach could resonate with conservative principles of efficiency and minimal government intervention in professional practices.<br><br>Overall, while the text aligns more closely with liberal viewpoints due to its emphasis on healthcare access and quality, as well as mental health advocacy, it also incorporates elements that could be agreeable to conservative perspectives, particularly regarding the reduction of perceived excessive regulations.	liberal
APA	Position Statement on Recognition and Management of HIV-Related Neuropsychiatric Findings and Associated Impairments	7/2020	The text you've provided does not align clearly with either a liberal or conservative viewpoint in a traditional political sense. Instead, it focuses on medical and psychiatric aspects related to HIV/AIDS and offers guidelines for psychiatrists in treating patients with these conditions.<br><br>This text emphasizes understanding and addressing the neuropsychiatric manifestations of HIV/AIDS, promoting nondiscrimination, and advocating for the rights of individuals living with HIV under the Americans with Disabilities Act. These points are generally aligned with a compassionate, science-based approach to healthcare, which is a perspective that can be embraced by individuals across various political spectrums.<br><br>The guidelines encourage a holistic and individualized approach to patient care, caution against making assumptions based on HIV status alone, and underscore the importance of legal protections for those living with HIV. These elements reflect a commitment to patient-centered care, nondiscrimination, and legal protections for marginalized groups. These principles are broadly accepted in the medical and psychiatric communities and do not necessarily fall into a strictly liberal or conservative category.	neutral
APA	Position Statement on Recognition and Management of Addictive Disorders and Other Mental Illnesses Multimorbid with HIV	7/2020	The text provided appears to be a balanced, evidence-based position from a medical or psychiatric perspective, rather than aligning strictly with a liberal or conservative political viewpoint. The focus is on integrated care for individuals with co-occurring psychiatric and addictive disorders, particularly in the context of HIV treatment and prevention. This approach emphasizes:<br><br>1. **Evidence-based treatment**: Advocating for the use of proven methods in treating substance use disorders and psychiatric conditions.<br>2. **Integrated care**: Emphasizing the importance of combining medical and psychiatric care to improve clinical outcomes.<br>3. **Reduction of stigma**: Highlighting the need to decrease stigma associated with HIV and mental illness.<br>4. **Patient-centered approach**: Encouraging individualized care that addresses both psychiatric and medical needs.<br><br>This position aligns with general healthcare best practices that prioritize patient welfare, evidence-based treatment, and a holistic approach to care. It doesn't specifically endorse any political ideology but rather focuses on medical and psychiatric best practices.	neutral

APA	Position Statement on Pharmaceutical Marketing to Justice Entities regarding Medication Treatment for Substance Use Disorders	7/2020	The text you've provided presents a nuanced perspective on the treatment of substance use disorders within the criminal justice system. It doesn't neatly align with traditional liberal or conservative viewpoints as it encompasses aspects that might resonate with both sides.<br><br>**Liberal Perspective Alignment:**<br>- Emphasis on treatment over incarceration: This aligns with a more liberal viewpoint that often advocates for rehabilitation rather than punitive measures.<br>- Concern about pharmaceutical lobbying and marketing: Liberals typically express concerns about corporate influence in public policy, including healthcare.<br>- Advocacy for comprehensive medical evaluations and decision-making by medical professionals: This aligns with liberal values of relying on expert opinions and evidence-based practices in policy-making.<br><br>**Conservative Perspective Alignment:**<br>- The text does not outright reject the role of the criminal justice system in addressing substance use disorders, which might resonate with more conservative views that emphasize law and order.<br>- It also discusses the importance of medication-assisted treatment, which some conservatives might support as a practical solution to a pressing problem.<br><br>However, the text primarily leans towards a public health and evidence-based approach rather than a strictly ideological stance. It emphasizes the importance of medical expertise, patient-centered care, and concerns about non-medical influence on treatment options, which can transcend traditional political divides.	neutral
APA	Position Statement on Medical Necessity	12/2020	The text you've provided outlines the position of the American Psychiatric Association (APA) on the issue of medical necessity determinations in healthcare. It does not strictly align with a liberal or conservative viewpoint in the traditional political sense. Instead, it focuses on healthcare policy and ethics, emphasizing the need for fairness, accuracy, and transparency in medical necessity determinations.<br><br>The APA's position advocates for:<br><br>1. Defining "medical necessity" in a way that prioritizes patient care and aligns with the standards of medical practice.<br>2. Developing medical necessity criteria jointly by professional societies, payers, and regulatory agencies based on evidence-based clinical standards.<br>3. Ensuring consistency in the criteria used for similar services across different payers.<br>4. Making criteria for determination of medical necessity easily and publicly available to healthcare providers and patients.<br>5. Advocating for transparency in the decision-making processes of government agencies, managed care organizations, third-party payers, and healthcare accreditation organizations.<br><br>This stance is centered around improving the healthcare system in terms of transparency and patient care quality. It addresses issues such as the influence of financial incentives in healthcare decisions and the need for more inclusive and transparent guideline development. These concerns can resonate with individuals across the political spectrum, as they relate more to healthcare policy and ethics than to partisan political ideologies.	neutral

APA	Position Statement on Medicaid Coverage for Maternal Postpartum Care	12/2020	The text provided outlines a health issue concerning postpartum mental health and substance use disorders and recommends extending maternal postpartum Medicaid coverage to one year post-delivery. This issue can be viewed through both liberal and conservative lenses, but it might align more closely with a liberal viewpoint for several reasons:<br><br>1. **Emphasis on Healthcare Access and Expansion**: The suggestion to extend Medicaid coverage reflects a liberal perspective that often advocates for expanded access to healthcare services. Liberals typically support government intervention to provide social safety nets and healthcare for vulnerable populations.<br><br>2. **Focus on Women's Health Issues**: The text's focus on women's health, specifically during the postpartum period, aligns with liberal priorities that often emphasize women's health rights and access to comprehensive healthcare services.<br><br>3. **Addressing Mental Health and Substance Use**: The liberal viewpoint often includes a strong focus on mental health and substance use treatment, seeing these as crucial public health issues that require attention and resources.<br><br>However, it's important to note that the concern for maternal and infant health can transcend political boundaries. Conservatives may also support aspects of this initiative, particularly if framed in terms of family values, the well-being of children, and efficient use of healthcare resources. The division isn't always clear-cut, and there can be overlap in the support for such healthcare policies across the political spectrum.	probably liberal
APA	Position Statement on Leadership of Academic Departments of Psychiatry	7/2020	The text provided doesn't align strictly with a traditional liberal or conservative viewpoint as it primarily addresses a specific issue within the field of medical education and psychiatry. The focus is on the professional standards and qualifications required for leaders in academic departments of psychiatry.<br><br>The position taken here advocates for the requirement that leaders of academic departments and training programs in psychiatry be board-certified psychiatrists. This stance is based on the belief that such qualifications ensure the integrity of training programs and provide appropriate role models and mentorship for psychiatric trainees.<br><br>The issue revolves around professional standards within a specific medical field, rather than broader political or ideological beliefs typically associated with liberal or conservative viewpoints. It reflects a perspective within the medical community concerning the qualifications and roles of professionals in psychiatric education and training.	neutral
APA	Position Statement on Issues Related to Sexual Orientation and Gender Minority Status	7/2020	The text provided aligns more closely with a liberal viewpoint. This assessment is based on the emphasis on inclusivity, support for diverse sexual orientations and gender identities, opposition to practices like conversion therapy, and advocacy for equal rights in various aspects of life such as employment, military service, and family rights. These positions are typically associated with liberal or progressive ideologies, which often prioritize the protection and recognition of minority groups' rights, inclusivity, and social justice.<br><br>Conservative viewpoints, on the other hand, might place a greater emphasis on traditional values and may be less supportive of such extensive measures to promote and protect the rights of sexual orientation and gender identity minorities. It's important to note, however, that viewpoints can vary widely within both liberal and conservative circles, and there are conservatives who support LGBTQ+ rights as well.	liberal

APA	Position Statement on Issues Pertaining to Capital Sentencing and the Death Penalty	7/2020	The text presents a position that is generally aligned with a liberal viewpoint. Liberal perspectives often emphasize the protection of human rights, the need for due process, and the humane treatment of individuals, especially those with mental disorders or disabilities. The American Psychiatric Association's (APA) position, as outlined, advocates for a moratorium on capital punishment and emphasizes the need for fair and impartial administration of justice, particularly concerning individuals with mental disorders or intellectual disabilities.<br><br>This position includes several key elements that are typically associated with liberal viewpoints:<br><br>1. **Moratorium on Capital Punishment:** The call for a moratorium on capital punishment reflects a concern for human rights and the potential for systemic injustices in the application of the death penalty, which are concerns often raised by liberal ideologies.<br><br>2. **Concern for Individuals with Mental Disorders or Disabilities:** The emphasis on the rights and treatment of individuals with mental disorders or disabilities during the legal process aligns with liberal values that prioritize the protection and fair treatment of vulnerable populations.<br><br>3. **Due Process and Fairness:** The focus on ensuring that capital punishment, if used, is administered fairly and in accordance with due process is consistent with liberal perspectives on criminal justice, which often stress the importance of fairness and equity in legal proceedings.<br><br>4. **Reduction of Punishment in Cases of Impaired Capacity:** The position that individuals who were unable to understand or control their actions due to mental disorders or disabilities should not be subject to the death penalty aligns with liberal views on rehabilitation, compassion, and the ethical treatment of individuals with mental health issues.<br><br>It's important to note that while this position aligns more closely with liberal viewpoints, the issues of capital punishment and the treatment of individuals with mental disorders in the criminal justice system can also be of concern to individuals across the political spectrum, including some conservatives who may have concerns about the fairness and efficacy of the death penalty.	liberal
APA	Position Statement on Involuntary Outpatient Commitment and Related Programs of Assisted Outpatient Treatment	12/2020	The position statement from the American Psychiatric Association (APA) on involuntary outpatient commitment doesn't neatly align with traditional liberal or conservative viewpoints. Instead, it reflects a blend of concerns and principles that can be found in both political spectrums.<br><br>1. **Liberal Viewpoint Alignment**:<br>- Emphasis on ethical concerns, individual autonomy, and the rights of patients align with liberal values.<br>- Focus on due process and non-discriminatory application of involuntary outpatient commitment.<br>- Advocacy for resources to be dedicated to voluntary treatment before resorting to involuntary measures.<br>- Attention to the disproportionate use of involuntary commitment among minority and disenfranchised groups.<br><br>2. **Conservative Viewpoint Alignment**:<br>- The use of court-ordered treatment to improve adherence to treatment and prevent dangerous behavior can resonate with a conservative emphasis on law and order, and public safety.<br>- The notion of involuntary commitment as a preventive measure to avoid more severe outcomes like hospitalization or incarceration could align with conservative views on proactive measures for community safety.<br>- The structured approach and systematic implementation of these programs might appeal to conservative preferences for organized, rule-based systems.<br><br>Overall, the APA's position seeks a balance between respecting individual rights and addressing public health and safety concerns. It acknowledges the complexity of mental health treatment, the importance of ethical considerations, and the need for resource allocation and effective program implementation. This blend of concerns shows an attempt to integrate aspects valued by both liberal and conservative perspectives, rather than aligning strictly with one political ideology.	neutral

APA	Position Statement on HIV Risk Reduction	7/2020	The text provided does not align strictly with either a liberal or conservative viewpoint in a political sense. Instead, it focuses on public health policies and medical recommendations regarding HIV prevention and treatment. The recommendations are based on scientific research and public health data, as evidenced by references to the Centers for Disease Control and Prevention (CDC) and the American Psychiatric Association (APA).<br><br>The emphasis is on evidence-based strategies for HIV prevention and treatment, including regular testing, antiretroviral treatment, pre-exposure prophylaxis (PrEP), post-exposure prophylaxis (PEP), syringe exchange programs, and addressing social determinants of health. These approaches are generally supported by the public health community irrespective of political leanings, as they are based on empirical evidence of effectiveness in reducing HIV transmission and improving health outcomes.<br><br>However, it's worth noting that some of the strategies mentioned, such as syringe exchange programs and comprehensive treatment for substance use disorders, may be more politically contentious and could be viewed differently across the political spectrum. Liberals might typically be more supportive of these harm reduction strategies, considering them essential for addressing public health issues. Conservatives might be more critical, possibly favoring more traditional or punitive approaches to drug use.<br><br>In summary, while the text is centered on public health and medical perspectives on HIV prevention and treatment, individual aspects of the recommendations might be viewed differently depending on one's political stance.	probably liberal
APA	Position Statement on Growing Fear over Coronavirus Spread and Mental Health Impact in ICE Detention Centers	12/2020	The text you've shared presents a viewpoint that is generally more aligned with liberal perspectives, especially in its emphasis on the humanitarian and mental health concerns related to the detention of immigrants during the coronavirus pandemic.<br><br>Liberal viewpoints often prioritize human rights, health and welfare, and a focus on the potential negative impacts of detention on mental health, as well as the risks of spreading COVID-19 in detention facilities, aligns with these values. The call for supervised release of detainees or finding alternatives for those charged with immigration violations, particularly vulnerable groups like the elderly and pregnant women, also reflects a liberal inclination towards more compassionate immigration policies.<br><br>Conservative viewpoints, on the other hand, tend to emphasize stricter immigration controls and may prioritize national security and legal procedures over the concerns highlighted in this text. They might argue for maintaining the detention of immigrants as a matter of law and order, even in the face of a pandemic.<br><br>It's important to note, however, that these are generalizations and individuals' views can vary widely within these broad political categories. Additionally, viewpoints on complex issues like immigration and public health can intersect with many different facets of political ideology.	liberal
APA	Position Statement on Ensuring Access to, and Appropriate Utilization of, Psychiatric Services for the Elderly	12/2020	The text you've provided outlines the position of the American Psychiatric Association (APA) regarding the treatment of older Americans with psychiatric illnesses. It's focused on advocating for better access to psychiatric care for the aging population and emphasizes the importance of ethical standards, compassion, and the effectiveness of treatment.<br><br>This text does not align distinctly with a liberal or conservative viewpoint in the traditional political sense. It is primarily a professional stance focused on healthcare, particularly mental healthcare for older adults. The emphasis is on equal access to care, respect, and the need for ongoing research, which are concerns typically shared across a wide range of political and social viewpoints.<br><br>Healthcare policies, including those related to mental health, can be interpreted differently based on political viewpoints. However, this specific text is more centered around medical ethics and best practices rather than a political ideology. It's important to recognize that while healthcare policy can become a political discussion, the APA's position here is focused on medical and ethical considerations.	neutral

APA	Position Statement on Consent to Mental Health Treatment by Guardians, Health Care Agents or other Legally Designated Surrogate Decision-making for Adults with Mental Illness	7/2020	The text you provided outlines various legal and ethical considerations for decision-making in mental health care, specifically concerning individuals deemed incompetent or incapable. It addresses the role of guardians, health care agents, and surrogate decision-makers in authorizing treatment, including the variability of these roles and authorities across different states. The American Psychiatric Association's position emphasizes the need for timely access to treatment while protecting vulnerable populations and outlines principles for authorization of health care decisions.<br><br>This text does not align explicitly with either a liberal or conservative viewpoint as traditionally defined in political terms. Instead, it focuses on ethical and legal aspects of mental health care, advocating for a balance between patient autonomy and the need for treatment in cases where individuals cannot make decisions for themselves. The principles it advocates for – respecting individual rights, ensuring informed consent, and providing flexibility and oversight in decision-making – are generally aligned with broader ethical standards in healthcare, rather than being specific to a political ideology.<br><br>In the context of U.S. politics, liberals generally emphasize individual rights and protections, including for vulnerable populations, while conservatives might emphasize personal responsibility and limited government intervention. However, the text mainly addresses legal and ethical considerations in healthcare, which can be important to both liberal and conservative viewpoints, albeit potentially for different reasons.<br><br>Therefore, it's more accurate to say that this text reflects a healthcare and legal perspective on mental health treatment and patient rights, rather than a strictly political one.	neutral
APA	Position Statement on Concerns About Use of the Term Excited Delirium and Appropriate Medical	12/2020	The text from the APA's Position Statement does not strictly align with a traditional liberal or conservative viewpoint. Instead, it primarily reflects a medical and ethical perspective on the issue of how law enforcement and medical personnel respond to individuals experiencing mental health crises.<br><br>The statement addresses concerns about the use of the term "excited delirium" and the administration of sedatives like ketamine in law enforcement contexts. These concerns are framed in terms of the need for clear diagnostic criteria, the risks of medication misuse, and the importance of evidence-based protocols.<br><br>However, the emphasis on investigating potential racial disparities in the application of the term "excited delirium" and the call for evidence-based, medically appropriate treatment protocols could be seen as aligning with viewpoints that prioritize social justice, equity, and rigorous scientific standards. These priorities are often associated with more liberal perspectives, particularly those concerned with systemic inequalities and the ethical implications of law enforcement practices.<br><br>On the other hand, the focus on scrutinizing and potentially regulating law enforcement practices might be viewed skeptically by some conservative viewpoints, especially those that prioritize law enforcement autonomy and resist what they see as external interference in police procedures.<br><br>Overall, the statement is best understood as a professional, medical position focused on patient care, ethical standards, and evidence-based practices, with implications that might resonate differently across the political spectrum depending on how they intersect with broader views on law enforcement, mental health, and racial equity.	neutral
APA	Position Statement on Competence Evaluation and Restoration Services and the Interface with Criminal Justice and Mental Health Systems	7/2020	The text you provided outlines a position on the treatment of individuals with mental illness or intellectual disabilities within the criminal justice system in the United States. It doesn't strictly align with either a liberal or conservative viewpoint as it addresses concerns about the intersection of mental health and criminal justice, topics that can be approached from multiple political perspectives.<br><br>However, the focus on reforming the system to ensure timely and appropriate treatment for individuals with mental illness, intellectual disabilities, and neurobehavioral conditions, and the emphasis on community-based services, jail diversion, and adequate funding for mental health services could be seen as more aligned with liberal viewpoints. Liberals often advocate for comprehensive healthcare, including mental health services, and for reforms in the criminal justice system that address systemic issues and support vulnerable populations.<br><br>On the other hand, conservatives might also support aspects of this approach, especially if it can be shown to be effective in reducing crime rates, recidivism, or costs associated with the criminal justice system. However, they might have different opinions on the methods of implementing such reforms, focusing perhaps more on personal responsibility, the role of families and communities, and efficient use of resources.	probably liberal

			Overall, the text represents a nuanced view that intersects with several key areas of public policy and can find support across the political spectrum depending on the specific aspects emphasized and the proposed methods of implementation.	
APA	Position Statement on Banning of Pharmacy Benefit Management Policies that Require the Provision of Dangerous Quantities of Medications	7/2020	The text you provided seems to be focused on a specific healthcare policy issue related to Pharmacy Benefit Management (PBM) and its impact on patient safety, particularly for those at risk of suicide. It does not align strictly with either a liberal or conservative viewpoint as traditionally defined in politics. Instead, it addresses a practical and ethical concern in healthcare:<br><br>1. **Healthcare Management and Patient Safety:** The main issue is about balancing the risks of providing large quantities of medication (which could be misused in cases of suicide attempts) with the benefits of cost savings and convenience for patients who need long-term medication.<br><br>2. **Physician's Discretion vs. Cost Management:** The position suggests that the treating physician's judgment about the quantity of medication should be prioritized over cost considerations. This perspective emphasizes individualized patient care and the professional autonomy of physicians.<br><br>3. **Policy Flexibility:** The suggested policy is for PBM companies to offer more flexibility in dispensing medications, allowing for smaller, more frequent shipments without additional cost. This approach aims to enhance patient safety while still recognizing the economic realities of medication management.<br><br>In summary, the text focuses on a specific healthcare policy issue, advocating for patient safety and physician autonomy in prescription practices. It does not neatly fit into a conventional liberal or conservative political framework but rather addresses a nuanced area of healthcare management and ethics.	neutral
APA	Position Statement on Antiretroviral-Based Therapy for HIV Prevention	7/2020	The text you've provided presents a factual, evidence-based approach to preventing HIV infection, primarily focusing on the use of pre-exposure prophylaxis (PrEP) and post-exposure prophylaxis (PEP). It does not explicitly align with either a liberal or conservative viewpoint. Instead, it appears to be a medical or public health perspective, emphasizing the effectiveness of PrEP and PEP in reducing the risk of HIV transmission and encouraging their use in combination with safer sexual and other behavioral practices.<br><br>The stance of the American Psychiatric Association (APA) supports the use of these prophylaxes and acknowledges the role of mental health professionals in this context. This position is consistent with a public health perspective that prioritizes evidence-based strategies for disease prevention, irrespective of political leanings.<br><br>Healthcare policy can sometimes be interpreted through a political lens, but the focus here is on medical efficacy and public health strategy, not political ideology.	neutral

APA	Position Statement on Use of Opioid Medications with Terminally Ill Patients	7/2019	The text you've provided outlines the American Psychiatric Association's (APA) position on the use of opioid analgesics in terminally ill patients. It focuses on the need for careful evaluation and monitoring of patients receiving opioid therapy to balance effective pain management with the risks of substance use disorders (SUDs) and diversion.<br><br>This position does not straightforwardly align with traditional liberal or conservative viewpoints as defined in political terms. Instead, it represents a medical and ethical stance that emphasizes patient care, the importance of managing pain effectively in terminally ill patients, and the need for caution due to the risks associated with opioid use.<br><br>- **Liberal Viewpoint**: Typically, liberal viewpoints in healthcare emphasize patient rights, comprehensive healthcare access, and often advocate for evidence-based approaches to treatment. In this context, the APA's position could align with a liberal perspective in terms of advocating for effective pain management and considering the patient's overall well-being, including mental health factors like depression and anxiety.<br><br>- **Conservative Viewpoint**: Conservative viewpoints in healthcare might focus more on personal responsibility, caution in prescribing potentially addictive substances, and strict regulation of drugs. The APA's stance on careful monitoring, evaluation for SUD and diversion risk, and consideration of non-opioid treatments might resonate with these conservative concerns.<br><br>Overall, the APA's position seems to integrate aspects that could be valued by both liberal and conservative perspectives: the importance of patient care and effective pain management (more liberal) and the emphasis on caution, monitoring, and consideration of non-opioid alternatives (more conservative). The focus here is more on medical ethics and best practices than on a clear-cut political ideology.	neutral
APA	Position Statement on Transitional Aged Youth	12/2019	The text you've provided focuses on mental health issues, specifically in transitional aged youth (TAY), and outlines the American Psychiatric Association's (APA) position on how to better serve this population. It doesn't align explicitly with a liberal or conservative viewpoint as these concepts typically relate to political ideologies. Instead, the text is centered around medical, psychological, and social perspectives on mental health care.<br><br>The emphasis is on recognizing the unique challenges faced by transitional aged youth and the importance of effective mental health services during this period. The APA's position advocates for early discussions about healthcare transitions, informed treatment that takes into account the developmental needs of TAY, educational opportunities for trainees in mental health, and policies that reduce financial barriers to accessing mental health care.<br><br>While certain elements of this position could be supported more by one political ideology than another (for instance, liberal viewpoints might more strongly emphasize the reduction of financial barriers to healthcare), the text as a whole represents a specialized, professional perspective focused on mental health care rather than a general political stance.	neutral
APA	Position Statement on The Role of Psychiatrists in Nursing Facilities	7/2019	The text you've provided appears to be a position statement from the American Psychiatric Association (APA) on the care of psychiatric disorders in nursing home settings. This text does not align clearly with either a liberal or conservative viewpoint in the traditional political sense. Instead, it focuses on medical, ethical, and practical aspects of psychiatric care for the elderly in nursing homes.<br><br>The key points highlighted in the APA position focus on the need for specialized psychiatric care, the importance of interprofessional collaboration, and the ethical considerations in treating psychiatric disorders in nursing home residents. These points emphasize the complexity of care required for this population and the need for adequate training and resources in geriatric psychiatry.<br><br>The mention of diversity in the workforce and the need for understanding cultural differences could be interpreted as aligning with more progressive or liberal views on inclusivity and cultural competence. However, these aspects are presented in the context of improving patient care and outcomes, which is a universally important goal in healthcare.<br><br>In summary, the text is primarily clinical and ethical in nature, focusing on best practices in the field of geriatric psychiatry rather than on political ideologies.	neutral

APA	Position Statement on the Insanity Defense	12/2019	The position statement from the American Psychiatric Association (APA) on the insanity defense doesn't neatly align with a purely liberal or conservative viewpoint. Instead, it reflects a perspective grounded in the principles of mental health and legal fairness. Here's a breakdown of how it might be perceived from different viewpoints:<br><br>1. **Liberal Viewpoint**:<br>- **Alignment**: Liberals often emphasize rehabilitation over punishment, especially for individuals with mental health issues. The APA's support for the insanity defense aligns with this perspective, as it acknowledges the importance of considering mental health in the context of criminal responsibility.<br>- **Focus on Fairness**: The statement's emphasis on the unfairness of punishing those with significant mental impairments at the time of their crime resonates with liberal values of compassion and understanding for marginalized groups, including those with mental health disorders.<br><br>2. **Conservative Viewpoint**:<br>- **Potential Agreement**: Some conservatives might agree with the notion that individuals who are genuinely incapable of understanding or controlling their actions due to mental disorders should not be held to the same legal standards as others.<br>- **Concerns about Misuse**: However, conservatives might be concerned about the potential for misuse or overextension of the insanity defense, fearing it could be used to avoid just punishment.<br><br>3. **General Legal and Ethical Perspective**:<br>- The APA's position is more aligned with ethical and legal principles of justice and individual assessment than with a specific political ideology. It underscores the importance of considering an individual's mental state and capacity for rational decision-making in legal proceedings.<br><br>In summary, the APA's position on the insanity defense transcends traditional political categorizations, focusing instead on a blend of legal, ethical, and mental health considerations.	neutral
APA	Position Statement on the Care of Medically Vulnerable Migrants in the United States	12/2019	The text you've provided discusses the "medical deferred action" policy of the United States Citizenship and Immigration Services and takes a position on the treatment of individuals with severe medical needs. It advocates for allowing these individuals to remain in the United States for treatment without the fear of deportation and stresses the importance of mental health screening for this population.<br><br>This viewpoint aligns more closely with a liberal perspective. Generally, liberal viewpoints emphasize the protection of vulnerable populations, including immigrants with medical needs. They often advocate for policies that provide humanitarian aid and support, as well as access to healthcare, regardless of immigration status. The emphasis on mental health and the holistic approach to the well-being of recently immigrated children and individuals with severe medical conditions is consistent with liberal values that prioritize public health, empathy, and inclusivity.<br><br>On the other hand, conservative viewpoints tend to emphasize stricter immigration controls and may prioritize the interests of citizens over non-citizens. They might argue that resources should be focused on citizens and legal residents, and that leniency in immigration policy could lead to abuses or strain on the healthcare system.<br><br>It's important to note that these are generalizations and individuals' views may vary. Also, the distinction between liberal and conservative viewpoints can sometimes be complex and nuanced, depending on the specific issue and context.	liberal

APA	Position Statement on Supporting Implementation of the Mental Health Parity and Addiction Equity Act	7/2019	The text you've provided outlines the American Psychiatric Association's (APA) position on the Mental Health Parity and Addiction Equity Act of 2008 (MHPAEA). This legislation and the APA's stance on it don't neatly align with traditional conservative or liberal viewpoints as they are understood in a broad political sense.<br><br>1. **Liberal Viewpoint Tendencies**: The MHPAEA, by advocating for equal treatment of mental health and substance use disorders in health insurance plans, aligns with principles often associated with liberal ideologies, such as equality, nondiscrimination, and comprehensive healthcare access. The emphasis on regulatory oversight and the need for health plans to demonstrate compliance also aligns with a more regulatory and interventionist approach typically favored by liberal viewpoints.<br><br>2. **Conservative Viewpoint Considerations**: On the other hand, aspects of the MHPAEA could also align with certain conservative principles. Ensuring that all individuals receive fair treatment under their insurance plans can be seen as upholding the principles of fairness and justice, which are also important in conservative ideologies. However, the increased regulation and oversight required by the MHPAEA might not align as closely with conservative preferences for smaller government and less regulation.<br><br>3. **Bipartisan Support for Mental Health Issues**: Mental health and substance use disorder treatment have become increasingly recognized as critical healthcare issues, garnering support across the political spectrum. While there may be differences in how each side approaches these issues, there is a growing consensus on the importance of addressing mental health needs.<br><br>4. **Complexity Beyond Political Dichotomies**: The MHPAEA's focus on parity in mental health and substance use disorder coverage transcends simple liberal-conservative dichotomies. It addresses complex health care and social justice issues that have relevance to, and impact on, a wide range of political beliefs and policies.<br><br>In summary, while certain aspects of the MHPAEA and the APA's position on it might resonate more with liberal ideologies, particularly in terms of advocacy for healthcare equality and regulatory oversight, these issues also touch on values and concerns that are relevant across the political spectrum.	neutral
APA	Position Statement on Substance Use Disorders	7/2019	The position statement you've provided from the American Psychiatric Association (APA) on the treatment and management of substance use disorders doesn't neatly align with traditional liberal or conservative viewpoints in a political sense. Rather, it reflects a medical and scientific approach to dealing with substance use disorders.<br><br>However, different aspects of this statement might resonate more with certain political ideologies:<br><br>1. **Emphasis on Medical Treatment and Access to Care**: The focus on recognizing substance use disorders as a medical issue, ensuring access to treatment, and advocating for parity in insurance coverage might align more closely with liberal viewpoints, which generally emphasize healthcare access and parity.<br><br>2. **Evidence-Based Treatment**: The insistence on evidence-based treatment is a scientific approach and doesn't inherently align with a particular political viewpoint. Both liberal and conservative viewpoints generally support the use of empirical evidence in healthcare, although there may be differences in the application or emphasis.<br><br>3. **Training and Education in Psychiatry**: The focus on education and training in the medical field is a universal concern, although the approach to funding and structuring such training may vary between political ideologies.<br><br>4. **Integrated Treatment for Co-occurring Disorders**: This approach is consistent with a comprehensive healthcare perspective, which can be associated with liberal views on healthcare, emphasizing the interconnectedness of mental and physical health.<br><br>5. **Regulation and Law Enforcement**: The statement's support for continued efforts to limit access to substances with high potential for abuse and to reduce the positive depiction of substance use in media might find support across the political spectrum, but the methods and extent of such regulation can be a point of divergence between liberal and conservative viewpoints.<br><br>6. **Research and Public Policy**: The advocacy for ongoing research into substance use disorders and their social consequences, as well as the involvement in public policy, is a scientifically oriented stance. The degree to which this research should influence public policy can be interpreted differently by liberal and conservative perspectives.<br><br>In summary, while the APA's position on substance use disorders is primarily medical and scientific in nature, different elements of	neutral

			the statement might resonate more with certain political ideologies, depending on how these issues are framed within the broader context of healthcare, regulation, and public policy.	
APA	Position Statement on Relationship Between Treatment and Mutual Support	12/2019	The text you've provided doesn't explicitly align with a traditional liberal or conservative viewpoint as understood in the context of U.S. politics. Instead, it focuses on professional guidelines and recommendations regarding addiction treatment, emphasizing the importance of professional treatment alongside the value of mutual support groups.<br><br>This perspective is grounded in medical and psychiatric expertise and practice, rather than political ideology. It advocates for a balanced approach to addiction treatment, recognizing the role of both professional medical treatment and mutual support groups. This approach is more about medical best practices and patient care rather than a political stance.<br><br>In the broader political context, liberals and conservatives might interpret or emphasize different aspects of these recommendations based on their own viewpoints. For instance:<br><br>- Liberals might focus on the importance of accessible healthcare, including mental health and addiction services, and might advocate for policies that support these services.<br>- Conservatives might focus on the role of community-based initiatives, like mutual support groups, and might emphasize personal responsibility in the context of addiction treatment.<br><br>However, these are generalizations and the text itself remains neutral, oriented towards medical best practices rather than political ideology.	neutral

APA	Position Statement on Psychiatric Participation in Interrogation of Detainees	12/2019	The position statement from the American Psychiatric Association (APA) regarding the participation of psychiatrists in the interrogation of detainees and their stance on torture doesn't align strictly with a traditional liberal or conservative viewpoint. Instead, it reflects ethical principles and professional standards in the field of psychiatry.<br><br>The APA's stance emphasizes several key points:<br>- Opposition to torture and the duty of psychiatrists to report torture.<br>- The right of all detainees to appropriate medical care.<br>- The primary obligation of psychiatrists to the well-being of their patients, including advocating for them, and not participating in their interrogation.<br>- Confidentiality of patient medical records and information.<br>- The prohibition of direct participation of psychiatrists in interrogations, but allowing them to train law enforcement on relevant psychiatric topics.<br><br>This position is rooted in medical ethics and human rights, which transcend political ideologies. It is focused on ensuring the humane treatment of detainees and the ethical conduct of psychiatrists, aligning with principles of human dignity and professional integrity rather than a specific political viewpoint.	neutral
APA	Position Statement on Prescription Drug Monitoring Programs	12/2019	The text provided does not inherently align with either a liberal or conservative viewpoint. Instead, it presents a factual and policy-focused perspective on the issue of opioid overdose and the use of Prescription Drug Monitoring Programs (PDMPs) in the United States.<br><br>The points outlined in the text are primarily focused on public health, patient safety, privacy, and the effective use of PDMPs to tackle prescription drug misuse and overdose. These issues are generally approached from a healthcare and public policy standpoint rather than being distinctly partisan.<br><br>Both liberal and conservative viewpoints might find different aspects of these recommendations agreeable. For example:<br><br>- Liberals might support the emphasis on patient privacy, healthcare access, and the use of public health agencies in addressing the opioid crisis.<br>- Conservatives might appreciate the focus on controlled substance regulation, the potential involvement of law enforcement (albeit with court orders), and the accountability of healthcare providers.<br><br>It's important to note that issues like the opioid crisis often transcend traditional political boundaries, with both liberals and conservatives recognizing the severity of the issue and the need for effective solutions, albeit potentially differing in the methods of implementation.	neutral
APA	Position Statement on Peer Review of Expert Testimony	12/2019	The text from the American Psychiatric Association (APA) is a position statement on peer review of psychiatric expert testimony. It does not neatly align with a liberal or conservative viewpoint as it is a professional, not political, statement. It focuses on the quality and improvement of psychiatric expertise in legal settings, advocating for voluntary education and the development of peer review models. This is more about professional standards and practices within psychiatry rather than a political stance. It emphasizes the importance of systematic evaluation to enhance the effectiveness of these models. Overall, the statement reflects a commitment to maintaining high professional standards and the integrity of psychiatric testimony in legal contexts.	neutral
APA	Position Statement on Neuroscience-based Nomenclature (NbN) Project	7/2019	The text you've provided doesn't clearly align with either a liberal or conservative viewpoint in the traditional political sense. It focuses on the technical and scientific aspects of psychiatric medication nomenclature and classification, specifically discussing the benefits of the Neuroscience-based Nomenclature (NbN) for psychotropic drugs.<br><br>This issue is more scientific and medical rather than political. It addresses the need for a more precise and updated classification system for psychotropic drugs based on their pharmacological mechanisms and developments in neuroscience, rather than their traditional, broader categories. The American Psychiatric Association (APA)'s position supports the integration of NbN into scientific publications and encourages pilot trials to assess its effectiveness in improving patient care and communication.<br><br>Such topics typically transcend typical political categorizations as they are rooted in scientific development and healthcare practices. However, the emphasis on evidence-based practice, patient-centered care, and advancing medical knowledge through updated scientific terminologies might be more resonant with ideologies that prioritize scientific advancement and healthcare improvements. This doesn't necessarily make it exclusively liberal or conservative, as these are goals that can be shared across a wide spectrum of political beliefs.	neutral

APA	Position Statement on Need to Maintain Intermediate and Long-Term Inpatient Care Access for Persons with Serious Mental Illness	7/2019	The text you've provided outlines the American Psychiatric Association's (APA) position on the care and treatment of persons with serious mental illness. It emphasizes the importance of a balanced approach to mental health care, advocating for both comprehensive community resources and the availability of intermediate- and long-term inpatient psychiatric care.<br><br>This position does not neatly align with traditional liberal or conservative viewpoints. Instead, it represents a nuanced perspective on mental health care, highlighting the complexity of mental illness treatment and the need for a range of services to adequately support individuals with serious mental illness.<br><br>- The emphasis on community resources and comprehensive support for individuals in their communities might resonate with more liberal viewpoints, which often prioritize community-based solutions and deinstitutionalization.<br>- The acknowledgment of the need for intermediate- and long-term inpatient care, along with sufficient funding for such services, might align with certain conservative perspectives that emphasize structured, hierarchical approaches to healthcare and the importance of institutional care for certain populations.<br><br>However, it's important to note that mental health policy often transcends traditional political divides, as it deals with complex, multifaceted issues that require a blend of approaches for effective solutions. The APA's position appears to be aimed at finding a balance that ensures both effective community integration and the availability of necessary inpatient care for those who need it.	neutral
APA	Position Statement on Leadership of State Behavioral Health Services	12/2019	The statement from the American Psychological Association (APA) you're referring to primarily focuses on the management and administration of mental health, addiction, and developmental disability services. It advocates for the involvement of qualified psychiatrists in leading or being part of the senior management of state mental health and addiction authorities.<br><br>This viewpoint doesn't neatly align with traditional liberal or conservative political ideologies. Instead, it reflects a professional and healthcare-oriented perspective. The emphasis is on the qualifications and expertise necessary for managing mental health and addiction services, rather than on a political stance.<br><br>In general:<br><br>1. **Liberal Viewpoints** tend to focus on expanding access to healthcare, including mental health services, and advocating for comprehensive, community-based systems of care. They often support increased public funding for these services.<br><br>2. **Conservative Viewpoints** may emphasize individual responsibility and private sector solutions for healthcare. They might advocate for limited government intervention in healthcare, which can include mental health services.<br><br>The APA's position, however, is centered on the professional qualifications required for effective management of mental health services. This is more about setting standards for healthcare administration than about a specific political ideology. It could be seen as compatible with both liberal and conservative views in different aspects: Liberals might agree with the emphasis on professional standards in public health services, while conservatives might support the principle of ensuring qualified leadership in any public service, including mental health care.	neutral
APA	Position Statement on Joint Resolution Against Torture	12/2019	The text from the American Psychiatric Association (APA) doesn't align directly with a liberal or conservative viewpoint in the traditional political sense. It's more focused on ethical principles in the medical and psychological professions, emphasizing human dignity, respect for individual rights, and opposition to torture.<br><br>These principles are grounded in the medical ethics of providing competent, compassionate care and protecting human rights. The condemnation of torture and support for the UN Declaration and Convention against Torture transcend typical political categorizations, as they are based on universal human rights and medical ethics.<br><br>While different political ideologies might have varied stances on related issues, the APA's position here is primarily based on professional ethics and humanitarian principles rather than a specific political alignment.	neutral

APA	Position Statement on Hospital Privileges for Psychologists and Other Non-Psychiatrist Mental Health Professionals	7/2019	The text you've provided outlines a position regarding hospital treatment for complex psychiatric conditions and the roles of different professionals in patient care. It does not explicitly align with a traditional liberal or conservative viewpoint as it primarily focuses on medical practice and healthcare policy rather than political ideology.<br><br>Here's a breakdown:<br><br>1. **Emphasis on Interdisciplinary Teams**: The text advocates for psychiatrists leading interdisciplinary teams, which includes psychologists and other mental health professionals. This approach to healthcare could be seen as aligning with more progressive or liberal ideas that often emphasize collaborative and holistic approaches to healthcare.<br><br>2. **Role of Psychiatrists**: The statement that psychiatrists should be primarily responsible for mental health and substance use disorder care in hospital settings might not have a clear political alignment. It could be interpreted as a professional stance based on medical expertise rather than political ideology.<br><br>3. **Role of Psychologists**: The text suggests that psychologists are critical team members but should not be the attending of record. This could be viewed as a stance on professional roles and competencies rather than a political statement.<br><br>Overall, the text seems more focused on healthcare management and the roles of specific professionals in a medical setting, which doesn't neatly fit into a liberal or conservative framework. It's important to note that interpretations can vary, and others might see different political implications in this statement.	neutral
APA	Position Statement on Diversity and Inclusion in the Physician Workforce	12/2019	The text presented primarily aligns with a liberal viewpoint, characterized by its emphasis on diversity, inclusion, and affirmative action within the medical profession, specifically in psychiatry. This perspective is reflected in several key aspects:<br><br>1. **Focus on Diversity and Inclusion**: The text strongly advocates for increasing the representation of underrepresented groups in medicine (URM), reflecting a commitment to diversity. This is a hallmark of liberal perspectives, which often prioritize diversity and representation in all sectors of society.<br><br>2. **Systemic Change and Support for Affirmative Action**: The text supports systemic changes in recruitment, retention, and development of URM individuals in psychiatry. This approach, including advocacy for affirmative action policies, is typically associated with liberal ideologies, which tend to support proactive measures to address historical and systemic inequalities.<br><br>3. **Alignment with Major Medical Organizations**: The position is in alignment with statements and policies of major medical organizations like the American Psychiatric Association (APA), American Medical Association (AMA), and others. These organizations often advocate for progressive changes in healthcare, which are generally consistent with liberal values.<br><br>4. **Research and Best Practices for Inclusivity**: The call for research and the adoption of best practices to improve diversity and inclusivity in the medical workforce further aligns with liberal values, which often emphasize evidence-based approaches to social reform.<br><br>5. **Emphasis on Serving Diverse Populations**: The text highlights the importance of serving diverse and underserved populations, addressing disparities in mental health care. This focus on equity and serving marginalized groups is a key aspect of liberal ideology.<br><br>It's important to note that while these viewpoints align more closely with liberal ideologies, issues of diversity and inclusion can transcend traditional political boundaries, and there may be elements that resonate with individuals across the political spectrum.	liberal

APA	Position Statement on Discriminatory Disability Insurance Coverage	12/2019	The position of the American Psychological Association (APA) as described in your text aligns more closely with a liberal viewpoint. This perspective is based on several key principles typically associated with liberal ideology:<br><br>1. **Equality in Healthcare**: The APA's stance that coverage for disability due to psychiatric disorders should be equivalent to coverage for non-psychiatric medical conditions reflects a liberal emphasis on equality and non-discrimination in healthcare access.<br><br>2. **Opposition to Restrictions Based on Diagnosis**: The APA's opposition to diagnosis-based contracts and limiting coverage for non-psychiatric conditions due to co-morbid psychiatric diagnoses aligns with liberal values of inclusivity and fairness in medical treatment.<br><br>3. **Focus on Reducing Stigma**: The concern about perpetuating stigma against mental illness and its impact on access to care is consistent with a liberal focus on social justice and reducing discrimination against marginalized groups, including those with mental health issues.<br><br>4. **Advocacy for Comprehensive Healthcare Coverage**: Liberals generally advocate for comprehensive healthcare coverage and are opposed to policies that restrict or limit access to necessary medical treatments, including psychiatric care.<br><br>It's important to note that while these points align with liberal values, mental health advocacy and the push for equitable healthcare coverage can also find support across the political spectrum, as these issues increasingly become recognized as essential components of overall health and wellbeing.	liberal
APA	Position Statement on Discrimination Against Persons with Previous Psychiatric Treatment	12/2019	The position expressed in the text from the American Psychiatric Association (APA) does not align strictly with either a liberal or conservative viewpoint in the traditional political sense. It primarily reflects a perspective focused on mental health advocacy, anti-discrimination, and the promotion of equal opportunities for individuals with psychiatric diagnoses or a history of psychiatric treatment.<br><br>This viewpoint emphasizes the following key points:<br><br>1. **Opposition to Discrimination Based on Mental Health**: The APA strongly opposes discrimination based on mental disorder or a history of psychiatric treatment, highlighting the unfounded and irrational nature of such biases.<br><br>2. **Individual Variability**: The statement recognizes the diversity and variability among individuals with the same diagnosis or treatment, arguing against categorical distinctions and stereotypes.<br><br>3. **Impact on Employment and Opportunities**: It discusses how discrimination in employment and other areas can be detrimental, both to individuals with mental health histories and to society at large, by discouraging people from seeking necessary treatment.<br><br>4. **Inadequacy of Psychiatric History as a Predictor of Job Performance**: The APA criticizes the use of psychiatric history as a screening tool in employment, pointing out the lack of a substantiated causal link between psychiatric history and job performance.<br><br>5. **Support for Anti-Discrimination Legislation**: The position strongly supports the Americans with Disabilities Act (ADA) and other legislation that provides protection for persons with mental impairments, advocating for their rights and inclusion in society.<br><br>6. **Focus on Functional Capacity**: The APA emphasizes that decisions related to employment and other opportunities should be based on an individual's functional capacity rather than their diagnosis or disability.<br><br>This stance is primarily driven by a commitment to mental health advocacy, human rights, and the promotion of equal opportunities, rather than by a specific political ideology. It focuses on challenging stigma, promoting understanding of mental health issues, and advocating for policies that ensure fair treatment and equal opportunities for those with mental health histories.	neutral

APA	Position Statement on Disaster Preparedness and Response for Older Americans	12/2019	The text you've provided presents a nuanced view on the impact of geoclimatic disasters on older Americans, particularly focusing on their mental health and the social, economic, and political factors that exacerbate their vulnerability. This text doesn't align strictly with a traditional liberal or conservative viewpoint, as it touches on various aspects that could be emphasized differently by each ideology.<br><br>1. **Liberal Viewpoint Alignment**: The text emphasizes the role of social, economic, and political institutions in mitigating the effects of disasters, highlighting the need for systemic solutions and support. This perspective aligns with liberal ideologies that often stress the importance of government and community intervention in addressing societal issues, especially for vulnerable populations.<br><br>2. **Conservative Viewpoint Considerations**: A conservative viewpoint might focus more on individual and community resilience, personal responsibility, and the role of local organizations (like nursing homes and community groups) rather than broader systemic changes. While the text does mention the resilience of older individuals and the importance of local support networks, its emphasis on broader institutional failures and the need for systemic solutions might be less resonant with conservative ideologies.<br><br>3. **Non-partisan Aspects**: The text's focus on the specific needs of older adults in disasters, such as their unique health challenges and the importance of addressing these through targeted interventions, is a non-partisan issue. Both liberal and conservative perspectives generally recognize the importance of caring for vulnerable populations in crisis situations, though they might propose different approaches to doing so.<br><br>In summary, while certain elements of the text could be seen as aligning more with liberal ideologies, especially its emphasis on systemic solutions and the role of institutions, the overall message about the importance of supporting older adults in disaster situations is a broadly non-partisan issue.	probably liberal
APA	Position Statement on Cultural Psychiatry as a Specific Field of Study Relevant to the Assessment and Care of All Patients	7/2019	The statement from the American Psychiatric Association (APA) regarding Cultural Psychiatry does not align directly with a liberal or conservative political viewpoint. Instead, it reflects a professional and medical perspective focused on improving psychiatric care.<br><br>Cultural Psychiatry as described here emphasizes understanding patient preferences and values, acknowledging unconscious biases in clinicians, and promoting mental health equity. These points are generally aligned with principles of inclusivity and cultural sensitivity, which can be associated with socially progressive values often found in liberal viewpoints. However, the focus on evidence-based medicine and patient-centered care is a universal goal in the medical field, transcending political ideologies.<br><br>In summary, while the statement incorporates elements that might resonate more with liberal values, such as emphasis on cultural competence and addressing disparities, its primary focus is on improving healthcare outcomes, which is a non-partisan objective.	probably liberal

APA	Position Statement on Controlling Drug Prices	12/2019	The text provided, outlining the American Psychiatric Association's (APA) position on drug pricing legislation and regulations, doesn't strictly align with a traditional liberal or conservative viewpoint. Instead, it focuses on practical and specific policy recommendations aimed at addressing the issue of drug pricing and availability, particularly in the context of mental health and substance use disorders. Here's a breakdown of how the recommendations may align with different political ideologies:<br><br>1. **Emphasis on Generic Medications and Market Entry**: The APA advocates for faster and more widespread entry of generic medications, which can be seen as a cost-effective approach. This could align with conservative principles of market efficiency and reducing regulatory barriers. However, it also aligns with liberal viewpoints that prioritize making healthcare more affordable and accessible.<br><br>2. **Maintaining and Facilitating Generic Medications**: This recommendation could be seen as a balance between market forces and regulatory oversight, aligning partially with both liberal and conservative perspectives.<br><br>3. **Stability in Drug Formularies**: The suggestion to keep medications on drug formularies for a reasonable amount of time and allow for new medications could be seen as a consumer protection measure, which is more aligned with liberal views. However, it also acknowledges the importance of innovation in medication, which can be a more conservative viewpoint.<br><br>4. **Transparency in the Prescription Drug Supply Chain**: Advocating for increased transparency in pricing aligns with both liberal and conservative views, as both sides generally support transparency, albeit for different reasons.<br><br>5. **Opposition to Utilization Management Tools for Cost Control**: The APA opposes the use of prior authorization and step therapy to control drug costs. This stance might align more closely with liberal ideologies, which often emphasize direct access to healthcare services without excessive administrative barriers.<br><br>In summary, the APA's position is centered on pragmatic solutions to specific issues in drug pricing and availability, particularly concerning mental health. This approach incorporates elements that could resonate with both liberal and conservative ideologies, making it a more bipartisan or centrist viewpoint rather than strictly aligning with one side.	neutral
APA	Position Statement on Civil Commitment for Adults with Substance Use Disorders	9/2019	The text you've provided outlines the American Psychiatric Association's (APA) position on laws related to civil commitment for substance use disorders (SUDs). This position is neither strictly liberal nor conservative but reflects a nuanced, evidence-based approach to a complex health issue. Here's a breakdown:<br><br>1. **Liberal Viewpoints:**<br>- Emphasis on Access to Treatment: The APA's stance on ensuring access to high-quality treatment services aligns with liberal views prioritizing healthcare access and comprehensive treatment options.<br>- Focus on Rights and Quality of Care: The insistence on conforming to medical standards, protecting legal rights, and integrating seamless linkages to care during and after commitment aligns with liberal values emphasizing individual rights and quality healthcare.<br><br>2. **Conservative Viewpoints:**<br>- Civil Commitment: The idea of civil commitment for SUDs, even with stipulated conditions, can resonate with more conservative approaches that favor structured interventions for drug use. However, the APA does not fully endorse civil commitment due to a lack of evidence on its effectiveness, showing a more cautious stance.<br><br>3. **Balanced/Evidence-Based Approach:**<br>- The APA's position is heavily based on the current evidence and research, emphasizing the need for more substantial evidence of effectiveness before taking a definitive stance on SUD commitment statutes.<br>- The focus on evidence-based practices and quality monitoring shows a commitment to data-driven policy decisions, a feature that transcends traditional political divides.<br><br>In summary, the APA's position reflects a blend of values and considerations, with a strong emphasis on evidence-based practice, quality care, and protecting individual rights. It cannot be neatly categorized into a strictly liberal or conservative viewpoint but rather represents a thoughtful, balanced approach to a complex issue.	neutral

APA	Position Statement on Carve Outs and Discrimination	7/2019	The text you provided reflects a viewpoint that is more aligned with liberal perspectives, particularly in its focus on the integration of mental health services with general healthcare and its opposition to the separation (carve-outs) of mental health and substance abuse services from general medical services.<br><br>Liberal viewpoints often emphasize the importance of comprehensive healthcare, including mental health, and advocate for policies that reduce stigmatization and improve access to care for all individuals, including those with mental illnesses. The text's emphasis on the detrimental effects of carve-outs on the quality of care, access to services, and the potential for stigmatization aligns with these liberal values.<br><br>Conservative viewpoints, on the other hand, might focus more on cost-efficiency, the role of private sector solutions in healthcare, or the minimization of government involvement in healthcare. While these aspects are not directly addressed in the text, the opposition to carve-outs due to their negative impact on patient care and equality in access to services does not typically align with conservative priorities in healthcare policy.<br><br>It's important to note that the distinction between liberal and conservative viewpoints can vary depending on the specific context and issue, and there can be overlapping concerns between the two ideologies. However, based on the focus and arguments presented in this text, it aligns more closely with a liberal perspective.	liberal
APA	Position Statement on Assuring the Appropriate Care of Pregnant and Newly Delivered Women with Substance Use Disorders	12/2019	The text provided reflects a liberal viewpoint rather than a conservative one. This assessment is based on the following observations:<br><br>1. **Emphasis on Public Health Over Legal Punishment:** The text emphasizes a public health approach to substance use in pregnant women rather than punitive legal measures. This aligns more closely with liberal perspectives, which often prioritize healthcare and rehabilitative approaches over criminalization, especially in matters of public health and substance abuse.<br><br>2. **Focus on Evidence-Based Treatment:** The advocacy for evidence-based treatments, comprehensive care, and harm reduction strategies is typically associated with liberal viewpoints, which often prioritize scientific research and data-driven policies in addressing social issues.<br><br>3. **Opposition to Criminalization of Substance Use in Pregnancy:** The text's opposition to criminalizing pregnant women for substance use aligns with a liberal stance. Conservatives are generally more likely to support punitive measures as a deterrent, whereas liberals tend to view such issues through a lens of public health and social welfare.<br><br>4. **Stress on Non-Discrimination and Inclusivity:** The call for consistent, non-discriminatory screening, and the emphasis on avoiding stigmatization and marginalization, reflects a liberal inclination towards inclusivity and equal treatment in healthcare.<br><br>5. **Advocacy for Social Support Services:** The text's support for social supports and inclusion, as well as comprehensive, culturally congruent care, aligns with liberal policies that often emphasize the social determinants of health and the need for a holistic approach to healthcare, including mental health.<br><br>6. **Opposition to Involuntary Commitment Laws:** The opposition to discriminatory implementation of involuntary commitment laws, especially in a manner that differs from their application to non-pregnant individuals, reflects a liberal concern for individual rights and civil liberties.<br><br>7. **Collaboration with a Range of Medical Professionals:** The support for ongoing collaboration across various medical fields for an integrated care approach is in line with a liberal perspective on interdisciplinary and holistic healthcare.<br><br>In summary, the text's emphasis on public health, evidence-based treatment, inclusivity, and opposition to punitive legal measures aligns it more closely with liberal viewpoints.	liberal

APA	Position Statement on Addressing Racial and Ethnic Health Disparities in Substance Use	12/2019	The text provided aligns more closely with a liberal viewpoint. It emphasizes issues such as racial and ethnic disparities in the criminal justice system, particularly regarding substance use and substance use disorders (SUDs). The focus on systemic inequities, the need for evidence-based treatment, and the criticism of existing judicial and correctional systems are characteristic of liberal perspectives, which often prioritize social justice, equity, and reform in institutional practices.<br><br>Key points indicating a liberal viewpoint include:<br><br>1. **Highlighting Racial and Ethnic Disparities**: The text underscores the disproportionate incarceration rates of racial and ethnic minority groups for substance-related offenses, a concern typically associated with liberal views on criminal justice reform.<br><br>2. **Emphasis on Evidence-Based Treatment**: Advocating for evidence-based treatment for SUDs, both within and outside the criminal justice system, aligns with liberal perspectives that prioritize science and research in policy-making.<br><br>3. **Diversion Programs Over Incarceration**: The suggestion to offer SUD treatment as an alternative to incarceration, especially for non-violent drug-related crimes, reflects a liberal approach towards criminal justice, focusing on rehabilitation over punishment.<br><br>4. **Addressing Systemic Inequities**: The call for national legislation to improve access to SUD treatment for all racial and ethnic backgrounds indicates a liberal inclination towards systemic change and government intervention to address social inequalities.<br><br>5. **Monitoring and Improving Access to Treatment**: The proposal for institutions to monitor equitable access to SUD treatment and to implement measures for increasing retention in these treatments is in line with liberal advocacy for transparent and equitable healthcare practices.<br><br>Overall, these points suggest a focus on systemic reform, healthcare equity, and social justice, which are key components of liberal ideologies.	liberal
APA	Position Statement on Abuse and Misuse of Psychiatry	7/2019	The text you've provided outlines a position regarding the ethical use of psychiatry, focusing on prioritizing individual needs and outcomes over organizational, social, personal, or political objectives. This stance does not align distinctly with either a liberal or conservative viewpoint in the traditional sense of American politics.<br><br>Instead, it reflects a professional and ethical perspective specific to the field of psychiatry. The American Psychiatric Association's emphasis on ethical evaluation, treatment, research, and education, as well as its caution against the abuse and misuse of psychiatry for non-clinical objectives, is more about maintaining professional integrity and patient welfare rather than aligning with a particular political ideology.<br><br>This approach is centered on ethical principles in medicine, such as beneficence, non-maleficence, and respect for patient autonomy, rather than political leanings. It highlights the importance of using psychiatric knowledge responsibly and ethically, irrespective of broader political contexts or beliefs.	neutral
APA	Position Statement In Opposition to Cannabis as Medicine	7/2019	The text provided reflects a cautious and evidence-based approach to the medical use of cannabis, aligning with neither a strictly liberal nor conservative viewpoint exclusively. It emphasizes the need for scientific evidence and FDA approval before endorsing cannabis for psychiatric treatments. This stance can be seen as conservative in its emphasis on regulation, controlled studies, and FDA approval processes. However, the call for more research and potential openness to future evidence-based uses could align with more liberal views on medical research and cannabis use.<br><br>The American Psychiatric Association's (APA) position is primarily concerned with the lack of scientific evidence supporting the use of cannabis for psychiatric disorders and the risks associated with its use, especially in adolescents. They advocate for more research and caution against state initiatives that authorize medical cannabis use through ballot initiatives, stressing the need for medical treatment to be evidence-based and meet professional standards of care.<br><br>This position is more aligned with a cautious, scientific, and regulatory approach than with a specific political ideology. It reflects a professional medical organization's focus on evidence-based practice, patient safety, and the need for rigorous clinical trials and FDA approval processes, which can be values appreciated across the political spectrum.	neutral

APA	Position Statement Against the Use of Cannabis for PTSD	7/2019	The text you've provided appears to align with a more conservative viewpoint. This assessment is based on the following observations:<br><br>1. Emphasis on Lack of Credible Studies: The statement highlights the absence of credible studies supporting the clinical effectiveness of medical cannabis for PTSD. This cautious approach to adopting new medical treatments without substantial evidence is often associated with conservative perspectives, which typically emphasize traditional methods and rigorous scientific validation.<br><br>2. Non-endorsement of Medical Cannabis: The American Psychiatric Association's (APA) position of not endorsing medical cannabis for PTSD treatment reflects a conservative stance. Conservative viewpoints often express skepticism or caution regarding the legalization and medical use of cannabis, preferring established treatments and further research.<br><br>3. Focus on Quality Care and Research: The involvement of councils such as the Council on Addiction Psychiatry, the Council on Research, and the Council on Quality Care, and their emphasis on the need for high-quality evidence, aligns with a conservative approach that prioritizes established medical practices and thorough scientific research.<br><br>However, it's important to note that the categorization of viewpoints as strictly "liberal" or "conservative" can be overly simplistic. Views on medical cannabis vary widely within both liberal and conservative groups, and the scientific and medical communities prioritize evidence-based approaches that may transcend typical political categorizations.	conservative
APA	Position on Sexually Transmitted Infections Including HIV Infection Among Older Adults	7/2019	The text provided does not align distinctly with either a liberal or conservative viewpoint. Instead, it focuses on a public health issue concerning the sexual health of older adults. It emphasizes the importance of acknowledging sexual activity in older adults, the associated risks of sexually transmitted infections (STIs), and the need for healthcare providers to address these issues as part of comprehensive care.<br><br>This approach is grounded in public health and medical perspectives, advocating for improved healthcare practices to address the needs of a specific population segment. It prioritizes the health and wellbeing of older adults, encouraging healthcare providers to include sexual health as a part of regular health screenings and discussions. This perspective transcends traditional political alignments and focuses on evidence-based healthcare practices.	neutral
APA	Position Statement on Weapons Use in Hospitals and Patient Safety	7/2018	The text you've provided discusses the position of the American Psychiatric Association (APA) on the use of weapons in hospitals, particularly in the context of managing patient behavior. The APA advocates for a clinical approach to managing patient violence, emphasizing the use of psychological and interpersonal interventions, and opposes the use of weapons as a clinical response.<br><br>This issue, as presented, doesn't align neatly with traditional liberal or conservative viewpoints as typically understood in the context of American politics. Instead, it focuses on professional and ethical considerations in a healthcare setting. However, different aspects of this issue might resonate differently with liberal and conservative ideologies:<br><br>1. **Liberal Viewpoint**: Liberals might support the APA's position as it emphasizes non-violent, clinical approaches to managing patient behavior and discourages the use of weapons. This aligns with a broader liberal perspective that often prioritizes healthcare, mental health, and non-violent solutions.<br><br>2. **Conservative Viewpoint**: Conservatives might have a mixed response. Some might agree with the emphasis on safety and the proper training of law enforcement personnel within hospitals. Others might argue for more stringent security measures, including the use of weapons, as part of maintaining overall safety and order, especially in high-risk situations.<br><br>It's important to note that such issues can transcend simple political categorization, as they involve complex considerations of public health, safety, professional ethics, and the rights and treatment of individuals with mental health issues.	neutral

APA	Position Statement on Use of the Principles of Recovery	12/2018	The position statement from the American Psychiatric Association (APA) regarding the application of recovery principles in the treatment of mental illness does not align strictly with either a liberal or conservative viewpoint. Instead, it reflects a medical and humanistic approach to mental health care.<br><br>Key aspects of the statement include:<br><br>1. **Emphasis on Recovery**: The APA highlights the importance of recovery, focusing on hope, meaningful life, and the integration of treatment with an individual's life goals and cultural, spiritual, and personal ideals.<br><br>2. **Person-Centered Approach**: The statement advocates for treating individuals with respect, meeting them at their level of awareness and readiness, and using a strengths-based approach. It stresses the importance of collaborative decision-making that involves the individual in the treatment process.<br><br>3. **Inclusion of Family and Supporters**: The APA suggests including family and close supporters as essential partners in the recovery process, especially for children.<br><br>4. **Comprehensive Care**: The principles support a comprehensive approach to care that values autonomy, dignity, self-respect, and full community integration. They focus on adapting to life's challenges and managing symptoms to improve functioning and health.<br><br>5. **Commitment to Resources**: The statement asserts that recovery should not lead to a reduction in resources or access to skilled professionals and high-quality care. It is based on a partnership among the individual, psychiatrist, and other practitioners.<br><br>The APA's position is rooted in a holistic view of mental health treatment that prioritizes individual needs, strengths, and involvement in the care process. This approach is generally consistent with modern psychiatric practices and is focused on patient welfare and empowerment rather than fitting into a specific political ideology.	neutral
APA	Position Statement on the Risk of Adolescents' Online Activity	7/2018	The text you provided discusses the risks associated with adolescent online activity, including exposure to cyberbullying, legal consequences, and negative impacts on mental health. It also mentions the American Psychiatric Association's position on the importance of acknowledging these risks and the role of psychiatrists in promoting safe online engagement.<br><br>This text does not align squarely with a traditional liberal or conservative viewpoint as understood in a political context. Instead, it focuses on public health and psychological perspectives. The concerns raised about online safety, mental health, and the need for education and protective measures can be seen as non-partisan issues that are important across the political spectrum.<br><br>Liberal viewpoints generally emphasize the importance of social welfare and public health initiatives, and might support the idea of increased education and resources for mental health and online safety. Conservatives, while traditionally focusing on individual responsibility and parental rights, might also see value in highlighting the risks of online activity for adolescents and the need for parental awareness and involvement.<br><br>In summary, the text seems to be more aligned with public health and child welfare perspectives, which can be supported by both liberal and conservative viewpoints, depending on the specific policies and measures proposed to address the issues raised.	neutral
APA	Position Statement on Solitary Confinement (Restricted Housing) of Juveniles	7/2018	The position expressed in the text aligns more closely with a liberal viewpoint. Liberal perspectives typically emphasize the protection of individual rights and the well-being of vulnerable populations, such as juveniles. The focus on the potential psychological harm of solitary confinement, the need for mental health and medical care, and the emphasis on using solitary confinement only as a last resort are consistent with liberal values that prioritize rehabilitation and humane treatment over punitive measures.<br><br>Conservative viewpoints, on the other hand, might emphasize stricter disciplinary approaches and could be more inclined to support the use of solitary confinement as a disciplinary tool. However, it's important to note that views on issues like juvenile justice can vary within both liberal and conservative circles, and there may be conservatives who also oppose the use of solitary confinement for juveniles based on concerns about its effectiveness or ethical implications.	liberal

APA	Position Statement on Screening and Treatment of Mood and Anxiety Disorders During Pregnancy and Postpartum	12/2018	The text you've provided outlines a significant public health issue regarding mood and anxiety disorders in perinatal women in the United States, along with recommendations from the American Psychiatric Association (APA) for addressing these issues. This text does not align strictly with a liberal or conservative viewpoint as traditionally defined in political terms. Rather, it focuses on medical and mental health concerns, emphasizing the need for screening, education, and treatment for perinatal psychiatric disorders.<br><br>Issues surrounding healthcare, mental health, and access to medical services can be viewed through various political lenses, but the text itself is presented in a neutral, medical, and factual manner. It addresses a public health concern and proposes solutions based on medical evidence and best practices, not political ideology.<br><br>In the broader context of political discourse, liberals might focus on the need for universal healthcare access, including mental health services, as a way to address these issues, while conservatives might emphasize individual responsibility, private healthcare solutions, or limited government involvement. However, the text itself remains neutral and focused on the medical aspects of the issue.	neutral
APA	Position Statement on Safe Prescribing	7/2018	The text from the APA (American Psychological Association) position statement you provided focuses on the treatment of patients with mental illness using medication and emphasizes the importance of medical education, training, supervision, and patient safety. This text is primarily centered around medical and ethical considerations rather than political ideology. It doesn't explicitly align with either a liberal or conservative viewpoint, as it is more focused on healthcare standards and patient safety, which are concerns that cross political boundaries.<br><br>Both liberal and conservative viewpoints can agree on the importance of properly educating and training healthcare professionals and ensuring patient safety. However, the approach to healthcare policy and regulation may vary between political ideologies, with liberal perspectives often emphasizing broader access and regulatory oversight, and conservative perspectives typically focusing on individual choice and less government intervention.<br><br>This APA position, however, does not delve into these political aspects of healthcare policy. It's more about setting a standard for the treatment of mental illness that is based on medical expertise and patient care considerations.	neutral
APA	Position Statement on Role of Psychiatrists in Palliative Care	12/2018	The issue and the APA position on palliative care do not inherently align with a strictly liberal or conservative viewpoint. Palliative care, as a comprehensive approach to treating serious illnesses and addressing physical, emotional, social, and spiritual needs of patients, transcends traditional political divides. It focuses on improving the quality of life for patients and their families through symptom management and support.<br><br>In the context of U.S. politics, both liberal and conservative perspectives might find value in different aspects of palliative care:<br><br>1. **Liberal Viewpoint**: Liberals generally advocate for comprehensive healthcare services, emphasizing the importance of mental health and the need for holistic approaches. The emphasis on integrating psychiatric care into palliative care, as well as the focus on individual patient needs and preferences, aligns with liberal values of individual rights and comprehensive healthcare access.<br><br>2. **Conservative Viewpoint**: Conservatives might appreciate the aspect of palliative care that involves careful resource management, focusing on patient comfort rather than extensive, costly medical interventions at the end of life. The respect for patient preferences and family involvement in decision-making can also resonate with conservative values around family and individual autonomy.<br><br>The American Psychiatric Association's (APA) position on integrating palliative care into psychiatric practice is focused on improving patient care and is not inherently political. Their advocacy for increased training in palliative care for psychiatrists and the integration of specialized psychiatric care into palliative services is a professional stance aimed at enhancing the quality of healthcare, rather than a political statement.	neutral

APA	Position Statement on Resolution Against Racism and Racial Discrimination and Their Adverse Impacts on Mental Health	7/2018	The text provided outlines the American Psychiatric Association's (APA) position on racism and racial discrimination and their impact on mental health. This text doesn't align strictly with a conservative or liberal viewpoint in the traditional political sense, as it is a professional and medical perspective.<br><br>However, if we examine the content in terms of common themes associated with liberal and conservative viewpoints in the United States, we can draw some connections:<br><br>- **Liberal Viewpoint**: The text aligns more closely with themes often associated with liberal ideologies. Liberals typically emphasize the importance of recognizing and addressing systemic issues like racism and discrimination. The APA's focus on the mental health impacts of racism, its advocacy for multiculturalism and diversity, and the call for equitable mental health services align with these themes.<br><br>- **Conservative Viewpoint**: Traditionally, conservative viewpoints may place a stronger emphasis on individual responsibility and might be less focused on systemic analyses of issues like racism. However, recognizing the mental health impacts of racism does not inherently conflict with conservative values. It's important to note that modern conservative viewpoints can vary widely, and some conservatives may indeed support these perspectives on mental health and racism.<br><br>It's crucial to understand that such topics as mental health and racism transcend simple political categorization and are relevant to broader human and societal issues. The APA's position is rooted in clinical and scientific research, emphasizing the health and well-being of individuals and communities, which can be a concern across the political spectrum.	neutral
APA	Position Statement on Residency Training Needs in Addiction Psychiatry for the General Psychiatrist	12/2018	The position statement from the American Psychiatric Association (APA) regarding general psychiatry residency training programs doesn't neatly align with a traditional liberal or conservative viewpoint. Instead, it focuses on medical and psychiatric education, emphasizing the importance of comprehensive training in handling substance use disorders.<br><br>Key elements of this position include:<br><br>1. **Screening and Brief Intervention:** This is a universal approach that doesn't inherently align with a specific political viewpoint. It's about identifying and addressing substance use issues early.<br><br>2. **Management of Intoxication and Withdrawal, and Pharmacotherapy:** These are clinical practices based on medical evidence, not political ideology.<br><br>3. **Co-occurring Substance Use and Psychiatric Disorders:** The focus here is on comprehensive care, which is a healthcare approach rather than a political stance.<br><br>4. **Exposure to Evidence-based Psychotherapy and Psychosocial Interventions:** Techniques like motivational interviewing and cognitive-behavioral therapy are grounded in scientific research, not political beliefs.<br><br>While some aspects of healthcare policy can be politicized, the APA's statement is primarily centered on medical education and best practices in psychiatric care, reflecting a focus on evidence-based treatment rather than aligning with a specific political ideology.	neutral

APA	Position Statement on Research with Involuntary Psychiatric Patients	7/2018	The text provided reflects a balanced, ethical perspective that doesn't clearly align with traditional liberal or conservative viewpoints. Instead, it focuses on the ethical considerations and practical aspects of conducting clinical research on psychiatric patients, particularly those who are involuntarily committed.<br><br>Key points in the text include:<br><br>1. **Emphasis on Existing Guidelines and Oversight**: The text supports the use of existing guidelines and regulatory frameworks to protect psychiatric patients in research settings. This is a neutral stance, advocating for the use of established systems rather than proposing radical changes.<br><br>2. **Role of Institutional Review Boards (IRBs)**: The text suggests that IRBs are best suited to make decisions about research risks and benefits, including considerations of participant vulnerability. This reflects a trust in existing institutional structures and processes.<br><br>3. **Participation of Hospitalized Psychiatric Patients in Research**: The text argues for allowing involuntarily committed psychiatric patients to participate in research with appropriate safeguards and informed consent. This view respects patient autonomy and supports the inclusion of marginalized groups in research, which can be seen as progressive but also aligns with ethical research practices broadly accepted across the political spectrum.<br><br>4. **Concerns about Stigma and Research Progress**: The position against barring involuntarily committed patients from research participation due to presumptions about their capacity highlights a concern for both scientific progress and the social implications of such exclusions, particularly stigma.<br><br>Overall, the stance taken in this text is primarily focused on ethical research practices and patient rights, transcending a simple liberal-conservative dichotomy. It advocates for a nuanced, case-by-case approach to research participation by psychiatric patients, emphasizing the importance of safeguards and informed consent while cautioning against broad exclusions that could hinder research progress and reinforce stigma.	neutral
APA	Position Statement on Religious Persecution and Genocide	7/2018	The text you've provided appears to be aligned with a humanitarian rather than a strictly liberal or conservative viewpoint. It focuses on the mental health implications of religious persecution and genocide, emphasizing the need for awareness, condemnation, and research on the impacts of these atrocities. This approach is generally consistent with humanitarian and human rights perspectives, which are concerned with the welfare and rights of all individuals, regardless of political affiliations.<br><br>The positions outlined, such as condemning acts of religious persecution and genocide, urging psychiatrists to speak out, and calling for further research, do not neatly align with a specific political ideology. They are more aligned with universal principles of human rights and mental health advocacy.<br><br>While different political ideologies might approach the issue of religious persecution and genocide differently, the emphasis on mental health, research, and advocacy transcends traditional political divides and is more about addressing human suffering and promoting well-being.	neutral
APA	Position Statement on Police Brutality and Black Males	12/2018	The text you provided aligns more closely with a liberal viewpoint. Liberal perspectives generally emphasize social justice, racial equality, and the reform of systems that perpetuate discrimination and inequality. This viewpoint is evident in the emphasis on the negative impact of police brutality on the mental health of black males and the broader black community, the call for reforms in law enforcement practices, and the focus on collaboration between law enforcement and mental health professionals to address these issues.<br><br>Conservative viewpoints, on the other hand, often prioritize law and order, and may emphasize the role of individual responsibility over systemic factors. While not opposed to justice or reform, conservative perspectives might focus more on supporting law enforcement and addressing issues within the context of maintaining social order and security.<br><br>The text's focus on systemic issues, the impact of police brutality on mental health, and the call for broad reforms and collaborative efforts between law enforcement and mental health professionals is more aligned with liberal ideologies that prioritize systemic change and address issues of racial inequality and justice.	liberal

APA	Position Statement on Physician Health Programs in the Treatment of Addiction and Substance Use Disorders	12/2018	The text provided does not align strictly with a liberal or conservative viewpoint. It is primarily a factual and clinical discussion about substance use disorders (SUDs) among physicians, their treatment, and the role of Physician Health Programs (PHPs). This issue transcends traditional political divides, focusing more on public health, medical ethics, and occupational health rather than partisan politics.<br><br>The American Psychiatric Association's (APA) position reflects a commitment to evidence-based treatment, reducing stigma, and ensuring equitable access to care for physicians with SUDs. These goals are generally consistent with public health-oriented approaches and do not inherently align with a specific political ideology.<br><br>In terms of political viewpoints:<br><br>- **Liberal Perspective:** A liberal viewpoint might emphasize the importance of comprehensive healthcare access, reducing stigma associated with mental health and substance use disorders, and supporting rehabilitative over punitive approaches. The APA's commitment to equitable and confidential treatment and research into SUDs among physicians aligns with these values.<br><br>- **Conservative Perspective:** A conservative viewpoint might focus on personal responsibility, the importance of maintaining high professional standards among physicians, and the need for effective monitoring to ensure patient safety. The aspect of the APA's position that emphasizes the importance of uncompromised patient care by physicians in PHPs could resonate with this perspective.<br><br>Overall, the text and the APA's position are more aligned with a healthcare and public health perspective than a specifically political one. They address the need for effective, evidence-based approaches to a significant issue in the medical community, which is a concern shared across the political spectrum.	neutral
APA	Position Statement on Neuroscience Training in Psychiatric Residency Training	12/2018	The text provided appears to be neutral and focuses on professional and educational standards in the field of psychiatry, rather than aligning with a specific political viewpoint, whether liberal or conservative. It emphasizes the importance of incorporating neuroscience knowledge into psychiatric education and training, advocating for enhanced curricula to better prepare future psychiatrists. This perspective is centered on advancing medical education and improving mental health care, which are generally considered non-partisan issues. The emphasis is on the quality of medical education and patient care rather than on political ideology.	neutral
APA	Position Statement on Mental Health Equity and the Social and Structural Determinants of Mental Health	12/2018	The text provided aligns more closely with a liberal viewpoint. This is evident from the focus on addressing social and structural determinants of mental health, promoting health equity, and advocating for policies that support marginalized or under-resourced groups. These themes are consistent with liberal ideologies that emphasize social justice, the importance of addressing systemic inequalities, and the role of public policy in improving societal welfare.<br><br>Liberal perspectives often prioritize the role of social factors in contributing to individual outcomes and advocate for systemic changes to address these issues. The emphasis on mental health equity, the need for supportive policies, and the focus on the broader social and structural determinants of health are in line with these principles.<br><br>The American Psychiatric Association's (APA) position, as outlined in the text, advocates for legislation and policies that promote mental health equity, emphasizes the importance of understanding and addressing the social and structural determinants of mental health, and calls for increased funding for research in these areas. This advocacy for systemic change and support for marginalized groups is characteristic of a liberal viewpoint.<br><br>Conservative viewpoints, on the other hand, might focus more on individual responsibility and less on systemic or structural changes. They might also prioritize different areas of public policy, such as fiscal conservatism or smaller government, which are not the focus of this text.<br><br>It's important to note that these are generalizations and that there can be a wide range of beliefs within both liberal and conservative ideologies. The text primarily aligns with liberal ideals due to its focus on social justice, equity, and systemic change in the context of mental health.	liberal

APA	Position Statement on Inquiries about Diagnosis and Treatment of Mental Disorders in Connection with Professional Credentialing and Licensing	7/2018	The text you provided outlines the American Psychological Association (APA)'s position on the evaluation of professional licensing applicants, particularly in relation to mental health. This position statement is focused on ensuring fairness and non-discrimination in the licensing process, emphasizing current functional ability rather than past mental health diagnoses or treatments.<br><br>This perspective aligns more closely with a liberal viewpoint for several reasons:<br><br>1. **Emphasis on Non-Discrimination:** The APA's stance against using past mental health diagnoses and treatments as criteria for professional licensing aligns with liberal values, which often prioritize non-discrimination and equal treatment, especially concerning mental health issues.<br><br>2. **Focus on Current Capacity:** The recommendation to focus on an individual's current capacity rather than past conditions reflects a progressive approach to mental health, recognizing that people can recover or manage their mental health effectively. This is in line with liberal views that advocate for the rights and capabilities of individuals with mental health challenges.<br><br>3. **Privacy and Confidentiality:** The insistence on the confidentiality of personal health information and the tailored approach to inquiries about mental health aligns with liberal values that emphasize the importance of privacy and individual rights.<br><br>4. **Responsive and Adaptive Approach:** The APA's suggestions for a nuanced and tailored approach to each applicant's situation reflect a more adaptive and individual-focused perspective, which is more commonly associated with liberal ideologies.<br><br>However, it's important to recognize that such viewpoints, while they can be broadly categorized, are not strictly confined to one political ideology. Different individuals or groups within both liberal and conservative spectrums might agree or disagree with these principles for various reasons. The text primarily reflects a concern for fairness, non-discrimination, and respect for individual privacy, values that can be appreciated across a wide range of political beliefs.	liberal
APA	Position Statement on Human Trafficking	12/2018	The text you've shared does not align specifically with a liberal or conservative viewpoint. Rather, it reflects a humanitarian and health-focused perspective on the issue of human trafficking. The emphasis is on the recognition of human trafficking as a public health issue with significant mental health consequences. The position calls for:<br><br>1. Increased education of mental health providers to identify and assist trafficking victims.<br>2. Collaborative efforts across disciplines to address the multifaceted impacts of human trafficking.<br>3. Advocacy for research into addressing the mental health needs of trafficking victims.<br>4. Legislative focus on prevention, protection of victims, and access to mental health care.<br><br>These points suggest a focus on healthcare, education, and legal reform, addressing human rights and public health rather than aligning with a specific political ideology. It reflects a comprehensive approach to a complex social issue, emphasizing multidisciplinary collaboration and systemic change.	neutral
APA	Position Statement on Elder Abuse, Neglect and Exploitation	7/2018	The text provided does not neatly align with either a liberal or conservative viewpoint in a traditional political sense. Rather, it focuses on addressing elder abuse, neglect, and exploitation as significant public health concerns. This issue transcends typical political divides, as it deals with the well-being and protection of a vulnerable population, which is a concern shared across the political spectrum.<br><br>The statement emphasizes the importance of recognizing and addressing various forms of elder abuse, the need for comprehensive assessments, and the role of mental health professionals in identifying, reporting, and treating the effects of such abuse. These points are generally consistent with both liberal and conservative values, as they focus on public health, the protection of vulnerable individuals, and the involvement of professionals and legal systems in safeguarding the rights and well-being of older adults.<br><br>Therefore, the text is more accurately described as a professional stance on a public health issue, aligning with the American Psychiatric Association's mission to ensure humane care and effective treatment for all persons with mental disorders, rather than being specifically liberal or conservative in nature.	neutral

APA	Position Statement on Discrimination of Religious Minorities	7/2018	The text you provided discusses the impact of discrimination against religious minorities on mental health, citing specific studies and findings. The position it advocates for is aligned with advocating for the well-being of minority groups and recognizing the detrimental effects of discrimination.<br><br>This perspective is generally more aligned with liberal viewpoints, which often emphasize social justice, the importance of addressing inequality, and the protection of minority rights. Liberal ideologies typically support the idea that societal structures and institutions should actively work to prevent discrimination and promote inclusivity.<br><br>However, it's important to note that the concern for the well-being of minority groups and the acknowledgment of the negative impacts of discrimination can also be shared by individuals with conservative viewpoints, especially those who prioritize individual rights and ethical treatment of all individuals.<br><br>The text's focus on empirical research (e.g., studies on the impact of discrimination on mental health) and its call for further research and education in the field suggests a scientific and evidence-based approach, which is a characteristic that transcends political ideology.<br><br>In summary, while the text's focus on minority rights and social justice aligns more closely with liberal viewpoints, aspects of its approach can resonate across the political spectrum, particularly its emphasis on evidence-based findings and concern for individual well-being.	liberal
APA	Position Statement on Discrimination Against Transgender and Gender Diverse Individuals	7/2018	The text you've provided outlines a position statement from the American Psychiatric Association (APA) regarding transgender and gender diverse individuals. It emphasizes the need for civil rights protections, equal treatment in various societal aspects, and advocacy against discrimination.<br><br>This position can be considered more aligned with a liberal viewpoint for several reasons:<br><br>1. **Emphasis on Equality and Civil Rights:** Liberal perspectives often prioritize civil rights and equality for marginalized groups, including the LGBTQ+ community. The text's focus on the need for legal protections and the repeal of discriminatory laws aligns with this.<br><br>2. **Opposition to Discrimination:** The text strongly opposes discrimination in areas such as health care, employment, and housing. This stance is typically associated with liberal ideologies, which often advocate for inclusive policies and practices.<br><br>3. **Advocacy for Marginalized Groups:** The APA's commitment to speaking out against discrimination and advocating for transgender and gender diverse individuals reflects a liberal inclination towards activism and social justice.<br><br>It's important to note that political viewpoints can be complex, and not all individuals or organizations fit neatly into "liberal" or "conservative" categories. However, based on the content and values expressed in the text, it aligns more closely with liberal perspectives, particularly in its advocacy for the rights and fair treatment of transgender and gender diverse individuals.	liberal

APA	Position Statement on Conversion Therapy and LGBTQ Patients	12/2018	The text you provided reflects a viewpoint that aligns more closely with liberal perspectives, particularly in the context of contemporary American politics and social attitudes. The key aspects of this viewpoint include:<br><br>1. **Opposition to Conversion Therapy**: The text clearly opposes "reparative" or conversion therapies aimed at changing an individual's sexual orientation or gender identity. This opposition is a stance more commonly associated with liberal ideologies, which generally emphasize the acceptance and rights of LGBTQ+ individuals.<br><br>2. **Affirmation of LGBTQ+ Identities**: The American Psychiatric Association (APA) recommends affirming individuals' sexual orientations and gender identities, which is a principle often advocated by liberal groups. This contrasts with more conservative viewpoints, which might either question or not fully support such affirmations, especially when linked to policy and legislative recommendations.<br><br>3. **Legislative Action**: The encouragement of legislation to prohibit conversion therapies is also more aligned with liberal policies. Such legislative actions are often supported by those advocating for greater protection and rights for LGBTQ+ communities.<br><br>4. **Evolution of Psychiatric Understanding**: The recognition of the historical evolution in the understanding of sexual orientation and gender identity – from being seen as mental illnesses to being recognized as part of the human condition – aligns with a liberal perspective that often emphasizes social progress and the reevaluation of past attitudes based on new understanding and inclusivity.<br><br>It's important to note that terms like "liberal" and "conservative" can vary in meaning depending on the cultural and political context. However, within the context of contemporary American sociopolitical discourse, the positions outlined in this text are more closely aligned with liberal viewpoints.	liberal
APA	Position Statement on Consistent Treatment of All Applicants for State Medical Licensure	12/2018	The text you provided from the APA (American Psychological Association) regarding the endorsement of equitable, fair, and consistent treatment for state medical licensure for various medical school graduates does not neatly align with a traditional liberal or conservative viewpoint in the political sense. It focuses more on professional standards and equity in the medical field rather than political ideologies.<br><br>From a professional standpoint, the statement advocates for equal treatment and opportunities for medical graduates, regardless of where they completed their education. This perspective aligns with principles of fairness and equal opportunity, which are generally endorsed across a broad spectrum of political views.<br><br>In terms of political ideologies:<br><br>- **Liberal Viewpoint**: Liberals typically advocate for equal opportunities and inclusivity, which aligns with the APA's position on equitable treatment for all medical school graduates.<br><br>- **Conservative Viewpoint**: Conservatives often emphasize merit and qualifications. If they view the APA's position as ensuring that all qualified candidates, regardless of their educational background, have a fair chance at licensure, they might also support this stance.<br><br>Thus, the APA's position could be seen as compatible with values from both sides of the political spectrum, as it emphasizes professional equity and standards rather than a specific political ideology.	neutral

APA	Position Statement on Access to Care for Transgender and Gender Diverse Individuals	7/2018	The text provided represents a viewpoint that aligns more closely with liberal ideologies, particularly in its emphasis on inclusivity, health care equity, and the recognition of the rights and needs of transgender and gender diverse individuals. This perspective is characterized by:<br><br>1. **Advocacy for Inclusive Health Care**: The call for removal of barriers to care and support for both public and private health insurance coverage for gender transition treatment reflects a liberal stance on health care, emphasizing the importance of inclusive and accessible health services for all individuals, regardless of gender identity.<br><br>2. **Recognition of Gender Diversity**: The acknowledgment of the benefits of medical and surgical gender-affirming treatments for transgender and gender diverse individuals aligns with liberal values that prioritize the recognition and support of diverse gender identities.<br><br>3. **Opposition to Discriminatory Practices**: The opposition to categorical exclusions of coverage for medically necessary treatment prescribed by a physician is indicative of a liberal viewpoint that challenges discriminatory practices and seeks to ensure equal rights and treatment for all individuals.<br><br>4. **Emphasis on Evidence-Based Care**: The support for evidence-based coverage of all gender-affirming procedures is in line with a liberal perspective that values scientific principles and data-driven approaches in policy and health care decisions.<br><br>5. **Patient-Focused Treatment Decisions**: The focus on patient-focused treatment decisions and advocating for patients' rights and well-being reflects a liberal approach to health care, where individual needs and choices are given priority.<br><br>Overall, the text supports the principles of equality, inclusivity, and the right to accessible health care, which are key components of liberal ideologies.	liberal
APA	Position Statement on Firearm Access, Acts of Violence, and the Relationship to Mental Illness and Mental Health Services	5/2018	The text you've provided generally aligns more closely with a liberal viewpoint. It emphasizes public health approaches to gun violence, including measures like background checks, safe storage requirements, regulation of firearm characteristics, restrictions on high-capacity magazines, and banning firearms in certain locations. It also advocates for research and training on firearm violence, reasonable restrictions on gun access not solely based on mental health diagnoses, and improved identification and access to mental health care.<br><br>Liberal viewpoints often prioritize public health and safety, advocating for regulatory measures to control gun violence, and emphasizing the importance of research and evidence-based policy. The focus on not stigmatizing mental illness in the context of gun violence and advocating for fair processes in gun ownership rights is also more in line with liberal perspectives.<br><br>Conservative viewpoints, on the other hand, often emphasize Second Amendment rights, minimal regulation of firearms, and may be more inclined to focus on mental health issues as a primary factor in gun violence, rather than broader public health approaches.<br><br>It's important to note that these are generalizations and there can be a wide range of opinions within each political ideology. The American Psychiatric Association, from which this statement originates, is focusing on public health and safety while also considering the rights of individuals, which can intersect with both liberal and conservative viewpoints in different ways.	liberal
APA	Resolution Opposing Any Restriction on the Number of International Medical Graduates (IMG) Entering Graduate Medical Training	7/2017	The text provided doesn't align strictly with either a liberal or conservative viewpoint in the traditional sense of U.S. politics. Instead, it focuses on a specific issue within the medical and psychiatric fields, particularly the role of International Medical Graduates (IMGs) in providing psychiatric care, especially in underserved areas such as inner cities, state hospitals, and prisons.<br><br>The American Psychiatric Association's (APA) position, as stated, opposes restrictions on the number of IMGs entering graduate medical training. This stance is based on the belief that such restrictions would negatively impact the delivery of mental health care. This viewpoint is primarily grounded in concerns about healthcare accessibility and quality, rather than a clear-cut liberal or conservative ideology.<br><br>In general, discussions about healthcare workforce policies can intersect with broader political ideologies, but they often involve specific considerations that aren't neatly categorized as "liberal" or "conservative." For instance, concerns about healthcare accessibility, quality of care, and workforce diversity might be shared across the political spectrum, though the proposed solutions might vary.	neutral

APA	Position Statement on Use of Stigma as a Political Tactic	7/2017	The text you've shared seems to align more closely with a liberal viewpoint, though it primarily reflects a nonpartisan, medical perspective. Here's the reasoning:<br><br>1. **Emphasis on Mental Health Equality:** The statement advocates for equal treatment of mental health issues compared to physical health issues. This approach, focusing on equality and destigmatization of mental health, is often associated with liberal viewpoints, which tend to emphasize social equality and the reduction of stigma in healthcare.<br><br>2. **Non-Discrimination and Inclusivity:** The position taken against using mental health history as a political weapon aligns with liberal values of inclusivity and non-discrimination. Liberals typically advocate for the rights and fair treatment of individuals with various backgrounds and conditions, including mental health.<br><br>3. **American Psychiatric Association's Stance:** The APA's position as stated here is focused on medical ethics and the fair treatment of individuals, which is a nonpartisan issue. However, the emphasis on removing stigma and not discriminating based on mental health history can resonate more with liberal ideologies, which often prioritize these aspects in policy discussions.<br><br>4. **Critique of Exploiting Prejudices:** The condemnation of using mental health for political gain by exploiting prejudices may be seen as a liberal standpoint because it challenges the status quo of political campaigning and advocates for a more empathetic, understanding approach to mental health.<br><br>It's important to note that while these points align more closely with liberal ideologies, mental health advocacy and the fight against stigma are increasingly recognized as nonpartisan issues, with support across the political spectrum. The primary focus of the text is on ethical considerations and medical professionalism rather than on a specific political ideology.	liberal
APA	Position Statement on the Prevention of Violence	12/2017	The text you provided appears to focus on public health and the role of psychiatry in the prevention and treatment of violence, including both victims and perpetrators. It advocates for the American Psychiatric Association (APA) to support various levels of violence prevention and to educate psychiatrists about violence prevention.<br><br>This stance does not align clearly with a traditional "liberal" or "conservative" viewpoint as understood in the context of American politics. Instead, it focuses on public health and professional standards in psychiatry. The emphasis on prevention, intervention, and education in dealing with violence is generally consistent with a public health approach.<br><br>In the American political context, liberal viewpoints often emphasize the importance of public health interventions, social services, and education, which this text aligns with to some extent. However, the text does not explicitly address broader political or social issues that typically differentiate liberal and conservative viewpoints, such as gun control, criminal justice reform, or the role of government in healthcare.<br><br>Conservative viewpoints might support aspects of this statement as well, particularly if they see the role of psychiatrists in violence prevention as part of a broader approach to public safety and individual responsibility.<br><br>Overall, the text is more closely aligned with professional and public health perspectives than with a specific political ideology.	neutral
APA	Position Statement on Discrimination Against International Medical Graduates	7/2017	The text you've provided doesn't neatly align with a traditional liberal or conservative viewpoint in the United States' political spectrum. It focuses on a policy from the American Psychiatric Association (APA) regarding non-discrimination against International Medical Graduates (IMGs) based on the country of their medical education. This policy emphasizes equality and non-discrimination in the professional field, which are principles that can be endorsed by individuals across various political ideologies.<br><br>In the U.S. political context, liberal viewpoints generally emphasize equality, inclusiveness, and anti-discrimination, which aligns with the sentiment of this policy. On the other hand, conservative viewpoints traditionally emphasize merit-based systems and may also support such a policy if it is viewed as promoting fairness and equal opportunity based on individual merit rather than nationality.<br><br>Therefore, the policy can be seen as aligning with broader principles of fairness and equality, which are values that can transcend specific political ideologies.	neutral

APA	Position Statement on Affirmative Action	7/2017	The position described in the text aligns more closely with a liberal viewpoint. It emphasizes affirmative action, the recruitment and training of minority psychiatrists, and the recognition of the special mental health needs of minority populations. These priorities are typically associated with liberal ideologies, which often prioritize diversity, equity, and inclusion in various sectors, including healthcare and education.<br><br>Conservative viewpoints, on the other hand, might emphasize merit-based systems and may be more critical of affirmative action policies, viewing them as potentially unfair or as a form of reverse discrimination. They often focus on individual responsibility and qualifications rather than group representation.<br><br>However, it's important to note that these are general trends and there can be a range of opinions within each political ideology. Also, the interpretation of this position can vary based on the context and the specific values of individuals within these broader political categories.	liberal
APA	Position Statement No Dangerous Patient Exception to Federal Psychotherapist Patient Testimonial Privilege	7/2017	The position stated regarding the American Psychiatric Association's view on the "dangerous patient" exception to the federal psychotherapist-patient privilege doesn't neatly align with traditional liberal or conservative viewpoints. This is because it intersects with multiple domains: mental health care, patient confidentiality, legal rights, and public safety, which are concerns of both liberal and conservative ideologies, albeit approached differently.<br><br>1. **Liberal Viewpoint**: Liberals generally advocate for strong patient rights and confidentiality, emphasizing the importance of trust in the therapist-patient relationship. They might support this position as it protects patient privacy and encourages individuals to seek help without fear of legal consequences. However, liberals also emphasize public safety and might have concerns about the potential risks to society if potentially dangerous individuals are not reported.<br><br>2. **Conservative Viewpoint**: Conservatives typically prioritize public safety and may have reservations about blanket protections that could hinder law enforcement and public protection. They might argue that exceptions are necessary in cases where a patient poses a clear danger. However, conservatives also value individual rights and might support aspects of this position that protect personal liberties and confidentiality.<br><br>This position reflects a complex balance between ethical considerations in mental health practice, legal rights, and public safety concerns. It's a nuanced issue that doesn't align perfectly with either ideological viewpoint, as it contains elements that both sides might agree or disagree with.	neutral
APA	Position Statement on Segregation of Prisoners with Mental Illness	12/2017	The text you provided expresses a position on the treatment of inmates with serious mental illness, particularly regarding their segregation and access to mental health services. This position doesn't neatly align with traditional conservative or liberal viewpoints as it encompasses aspects that both political ideologies might support or contest.<br><br>- **Liberal Viewpoint**: Liberals generally advocate for prison reform, emphasizing rehabilitation over punishment, and improved mental health care for inmates. This viewpoint aligns with the idea of avoiding prolonged segregation for inmates with mental illnesses and providing them with adequate mental health treatment and recreational activities. It reflects a focus on human rights and the well-being of marginalized populations.<br><br>- **Conservative Viewpoint**: Conservatives traditionally prioritize law and order, often favoring stricter prison regimes. However, there's a growing recognition among conservatives of the need for prison reform, including better mental health care, to reduce recidivism and improve public safety. From this perspective, the text could align with a more modern, reform-minded conservative viewpoint that acknowledges the importance of mental health care in corrections.<br><br>In summary, while the text might more closely align with liberal principles of prison reform and mental health advocacy, it also fits into a broader, bipartisan context of prison reform that includes elements agreeable to some conservative perspectives, especially those focused on effective rehabilitation and public safety.	neutral
APA	Position Statement on Homicide Prevention and Gun Control	12/2017	The text provided appears to align with a liberal viewpoint. It advocates for strong controls on the availability of firearms to private citizens, emphasizing the relationship between firearms and increased rates of homicide and personal injury. This position is generally more aligned with liberal or progressive ideologies, which often support stricter gun control measures as a means of reducing gun violence and enhancing public safety. Conservative viewpoints, on the other hand, typically emphasize Second Amendment rights and may oppose stricter gun control measures, advocating instead for the rights of individuals to own and carry firearms with fewer restrictions.	liberal

APA	Position Statement on Assessing the Risk of Violence	12/2017	The text you provided is a position statement related to the assessment of violence risk by psychiatrists. It does not align strictly with either a liberal or conservative viewpoint as defined in political terms. Instead, it focuses on the professional and clinical aspects of psychiatric practice. The statement emphasizes the complexity and uncertainty inherent in assessing the risk of violence, acknowledging that psychiatrists can identify risk factors but cannot predict dangerousness with absolute certainty. This perspective is grounded in clinical practice and research, rather than in political ideology.<br><br>In general, discussions about mental health and risk assessment can intersect with political views, especially in debates about public policy, healthcare, and legal issues. However, the specific text you've shared is more centered on professional guidelines and the realities of psychiatric assessment rather than political ideology.	neutral
APA	Accountability for Persons with Serious Mental Illness	7/2016	The text provided outlines a comprehensive approach to addressing the needs of individuals with serious mental illness and emphasizes the importance of accessible, inclusive, and integrated healthcare. This perspective can align with both liberal and conservative viewpoints in different ways:<br><br>1. **Liberal Viewpoint Alignment**:<br>- The emphasis on inclusive and accessible healthcare for all, regardless of sociocultural background, is a common theme in liberal ideologies.<br>- The focus on government responsibility and accountability for public health aligns with liberal views on the role of government in providing social services.<br>- The comprehensive approach to mental health, including the integration of services and support for marginalized groups, resonates with liberal values of equality and social justice.<br><br>2. **Conservative Viewpoint Alignment**:<br>- The text also places importance on personal responsibility and recovery-oriented services, which can align with conservative values emphasizing individual responsibility.<br>- The involvement of family and community in the treatment process can resonate with conservative views on the importance of traditional social structures.<br>- The emphasis on efficient care and treatment, and possibly the idea of reforming social security eligibility, might appeal to conservative preferences for fiscal responsibility and efficient use of resources.<br><br>In summary, while the text contains elements that could align with both liberal and conservative viewpoints, it primarily focuses on comprehensive, inclusive, and effective mental health care, which is a universal concern beyond political affiliations.	neutral
APA	Position Statement on Treatment of Substance Use Disorders in the Criminal Justice System	12/2016	The text you've provided represents a viewpoint that could be associated with either liberal or conservative ideologies, depending on the specific aspects being considered. The focus on evidence-based treatment for substance-related disorders, the emphasis on comprehensive care including mental health and substance abuse treatment, and the advocacy for continued care and support post-release align with principles commonly found in liberal viewpoints. These principles include an emphasis on rehabilitation, addressing systemic issues in the criminal justice system, and prioritizing public health and comprehensive care.<br><br>However, certain aspects of the statement, such as the support for drug courts and the use of sanctions for relapses, might also resonate with more conservative viewpoints that emphasize personal responsibility, the rule of law, and structured interventions within the criminal justice system.<br><br>In contemporary political discourse, issues surrounding criminal justice reform and public health are often complex and can cross traditional ideological boundaries. Both liberal and conservative viewpoints may converge on the need for effective interventions in the criminal justice system, albeit with differing approaches and emphases. This statement from the American Psychiatric Association reflects a multifaceted approach that incorporates elements which could be endorsed by people across the political spectrum.	neutral

APA	Position Statement on Screening and Testing for HIV Infection	12/2016	The text you've provided is primarily focused on public health guidelines and recommendations regarding HIV testing and treatment. It aligns more with a scientific and medical perspective rather than a specific political viewpoint.<br><br>The guidelines emphasize the importance of early diagnosis and treatment of HIV, and the benefits of antiretroviral medications in reducing transmission and improving health outcomes. These points are generally supported by a wide range of public health experts and organizations, and they reflect a consensus in the medical community about best practices in HIV prevention and care.<br><br>While public health policies can sometimes become entangled in political debates, the information presented here is based on scientific research and public health expertise. It focuses on practical measures to manage and prevent HIV, rather than advocating for a particular political ideology.<br><br>In conclusion, this text is more aligned with a public health perspective, emphasizing evidence-based practices for HIV testing and treatment, rather than aligning clearly with either a liberal or conservative political viewpoint.	neutral
APA	Position Statement on Recognition and Management of HIV-Associated Neurocognitive Impairment and Disorders (HAND)	12/2016	The text you've provided outlines a position on the management and accommodation of HIV-associated neurocognitive impairment and disorder (HAND) in a clinical and legal context. It doesn't neatly align with traditional liberal or conservative viewpoints as understood in a political sense. Instead, it focuses on medical and legal best practices for treating individuals with HAND.<br><br>The points emphasize:<br><br>1. Legal protections for HIV-infected individuals under the Americans with Disabilities Act.<br>2. The importance of thorough assessment and referral for HAND diagnosis.<br>3. The inappropriateness of using HIV status as an indicator of cognitive impairment.<br>4. Awareness of neurocognitive manifestations of HIV.<br>5. Participation in treatment and prevention of HAND.<br><br>These positions are centered around medical ethics, patient rights, and public health considerations rather than political ideology. They advocate for fair treatment, non-discrimination, and thorough medical care for individuals with HAND, aligning with principles of human rights and medical ethics that can be supported across a wide political spectrum.	neutral
APA	Position Statement on Psychiatric Hospitalization of Children and Adolescents	7/2016	The text provided reflects a viewpoint that prioritizes healthcare access, particularly mental health care for children and adolescents, and does not strictly align with traditional conservative or liberal viewpoints. Instead, it focuses on public health, emphasizing the need for increased access to inpatient psychiatric services, better treatment facilities, family involvement in treatment, and addressing the shortage of specialized psychiatrists.<br><br>While it's true that discussions about healthcare can often be politicized, this text seems more centered on healthcare policy and public health concerns than on ideological divisions. Both liberal and conservative viewpoints might find aspects to support in this statement, such as the emphasis on improving healthcare services and addressing public health needs, although they might differ in approaches to funding, implementation, and the role of government in healthcare.<br><br>In summary, the text is more healthcare-focused and oriented towards improving mental health services for a vulnerable population, rather than being aligned with a specific political ideology.	neutral
APA	Position Statement on Out of Network Restriction of Psychiatrists	12/2016	The text provided does not neatly align with a strictly liberal or conservative viewpoint as these terms are traditionally understood in the context of political ideologies. Instead, it addresses a specific issue in healthcare policy, particularly related to psychiatry, insurance, and access to care.<br><br>The position of the American Psychiatric Association (APA) as described here focuses on ensuring that patients have access to necessary medical care regardless of the network status of their healthcare provider. This stance emphasizes patient rights and access to care, which can be a concern across the political spectrum.<br><br>In general, debates about healthcare often involve a range of viewpoints that can't be easily categorized as purely liberal or conservative. Different aspects of healthcare policy, such as insurance coverage, physician reimbursement, and patient access to care, can be viewed and interpreted through various ideological lenses. The APA's position, as outlined, seems to prioritize patient access and equity in healthcare, values that are often emphasized in a variety of political and policy discussions.	neutral

APA	Position Statement on Medical Euthanasia	12/2016	The statement you provided reflects a position on medical euthanasia, specifically relating to non-terminally ill persons, as held by the American Psychiatric Association and aligned with the American Medical Association's stance. This position emphasizes that psychiatrists should not prescribe or administer interventions to non-terminally ill individuals for the purpose of causing death.<br><br>Labeling this viewpoint as strictly "liberal" or "conservative" can be challenging because views on medical euthanasia often intersect with various ethical, moral, and religious beliefs, and do not always align neatly with political ideologies. In general, conservative viewpoints tend to emphasize traditional values and may be more inclined to oppose euthanasia, citing moral and ethical concerns. On the other hand, liberal viewpoints might be more open to considering euthanasia, especially in the context of personal autonomy and the right to choose in matters of life and death.<br><br>However, it's important to note that within both liberal and conservative circles, there can be a wide range of opinions on this issue. The stance mentioned in your text seems to be more focused on ethical considerations in medical practice rather than a direct alignment with a political ideology. It emphasizes the responsibility of medical professionals, particularly psychiatrists, in the care of non-terminally ill patients, suggesting a cautious and ethical approach to the issue of life-ending interventions.	neutral
APA	Position Statement on Integrated Care	7/2016	The text you've provided is a position statement from the American Psychiatric Association (APA) regarding integrated care in mental health services. It doesn't align explicitly with either a liberal or conservative viewpoint in the traditional political sense. Instead, it focuses on healthcare policy and practice, advocating for evidence-based, patient-centered approaches in psychiatric care.<br><br>Key elements of the statement include:<br><br>1. **Promotion of Integrated Care Models**: Emphasizing team-based, population-based, measurement-based, evidence-based, and accountable care. This approach is about enhancing healthcare quality and efficiency, which can be a priority across the political spectrum.<br><br>2. **Advocacy for Mental Health Parity**: The APA's support for sustainable reimbursement strategies in line with the Mental Health Parity and Addiction Equity Act (MHPAEA) shows a commitment to ensuring equal treatment for mental health in healthcare policies.<br><br>3. **Focus on Evidence and Quality Improvement**: The emphasis on using evidence-based practices and continuous quality improvement in care models is a healthcare-specific approach that transcends political ideologies.<br><br>4. **Attention to Health Outcomes and Cost Containment**: The statement addresses both improving health outcomes and containing costs, which are concerns common to many stakeholders in the healthcare system.<br><br>In summary, the statement is more aligned with healthcare policy and best practices in psychiatry rather than fitting neatly into a liberal or conservative political framework. It emphasizes evidence-based, patient-centered care, quality improvement, and parity in mental health treatment, which are issues that can be championed by advocates across the political spectrum.	neutral

APA	Position Statement on Equitable Access to Quality Medical Care for Substance Related Disorders	7/2016	The text provided presents a viewpoint that aligns more closely with liberal perspectives, particularly in the context of health care policy and mental health. Here's a breakdown of how it aligns with liberal viewpoints:<br><br>1. **Advocacy for Equal Coverage**: The stance that substance related disorders should be covered equally by insurance, similar to other medical disorders, reflects a liberal perspective on health care. Liberals often advocate for comprehensive health care coverage and parity in insurance, emphasizing the importance of treating mental health disorders on equal footing with physical health disorders.<br><br>2. **Emphasis on Accessible Treatment**: The call for expanded access to effective prevention and treatment, including medication assisted treatment, is in line with liberal values of ensuring broad access to health care services. Liberals typically support policies that increase accessibility to health care, especially for underserved or marginalized populations.<br><br>3. **Opposition to Discrimination in Health Care**: The position against discriminatory practices in health care coverage for those with substance related disorders aligns with liberal ideals of equality and fairness. Liberals often focus on eliminating discrimination in all forms, including within the health care system.<br><br>4. **Recognition of Substance Related Disorders as Mental Illnesses**: The acknowledgement that all substance related disorders are diagnosable mental illnesses and the advocacy for their treatment reflects a progressive approach to mental health, which is more commonly associated with liberal perspectives. This viewpoint emphasizes the need to understand and treat mental health issues with the same seriousness as physical health problems.<br><br>It's important to note that while these positions align more closely with liberal perspectives, issues of health care and mental health can transcend traditional political boundaries. There can be conservative viewpoints that also support some of these measures, particularly when they are framed in terms of overall societal benefit or cost-effectiveness. However, based on the principles outlined in the text, it leans more towards a liberal viewpoint.	liberal
APA	Position Statement on Emergency Boarding of Patients with Acute Mental Illness	7/2016	The text you've provided outlines a position on the treatment of individuals with acute mental illness in emergency departments, emphasizing the need for better care and resources. This position does not align strictly with a traditional liberal or conservative viewpoint as understood in contemporary American politics. Instead, it touches on issues that are relevant and potentially of interest to both sides:<br><br>1. **Liberal Viewpoint Alignment**: The emphasis on increased investment in preventive mental health services, better care for vulnerable populations, and the call for more humane treatment align with typical liberal values, which often prioritize social welfare and public health initiatives.<br><br>2. **Conservative Viewpoint Alignment**: The concern over the inefficiency of current systems, which leads to more expensive emergency care, and the potential interest in finding cost-effective solutions, can resonate with conservative values, which often emphasize fiscal responsibility and efficient government spending.<br><br>It's important to note that mental health care is increasingly seen as a bipartisan issue, with both liberals and conservatives recognizing the importance of effective mental health services. The emphasis on practical solutions to systemic problems in mental health care crosses traditional political divides.	neutral

APA	Position Statement on Adolescent Substance Abuse	12/2016	The position outlined here doesn't neatly align with a traditional "liberal" or "conservative" viewpoint as these terms are commonly understood in the political spectrum. It appears to be a comprehensive, medically and scientifically informed approach to adolescent substance use, emphasizing early intervention, education, research, and treatment.<br><br>Key aspects of this position include:<br><br>1. Acknowledging the harm of early and regular substance use in adolescents.<br>2. Emphasizing the importance of routine screening for substance use in psychiatric and medical assessments.<br>3. Balancing confidentiality with family involvement in treatment and prevention.<br>4. Addressing attitudes and beliefs surrounding substance use.<br>5. Prioritizing early identification and prevention programs.<br>6. Supporting ongoing research in all aspects of substance use.<br>7. Advocating for responsible media portrayal of substance use.<br>8. Encouraging policy advocacy for the treatment of substance use disorders and parity in health insurance coverage.<br>9. Fostering professional training and education on adolescent substance use.<br>10. Including comprehensive education about substance use in medical and psychiatric training programs.<br><br>This approach combines elements that might be supported by both liberal and conservative viewpoints. For instance, the focus on research, prevention, and treatment aligns with a public health perspective often associated with liberal policies. Conversely, the emphasis on family involvement and responsible media portrayal can resonate with more conservative views on community and societal values. However, the primary focus here is on public health and clinical best practices rather than political ideology.	neutral
APA	Position Statement on Use of Animals in Research	7/2015	The text you've provided appears to be a position statement supporting the use of animals in psychiatric and neuroscience research, emphasizing the necessity of such research for advancing human health while advocating for the humane treatment of animals.<br><br>This statement doesn't neatly align with a traditional liberal or conservative viewpoint as these terms are commonly understood in a political context. Instead, it addresses a specific scientific and ethical issue that can cross political lines. Here's why:<br><br>1. **Science and Health Focus**: The primary focus is on the importance of animal research in advancing psychiatric medicine and neuroscience. This is a scientific stance rather than a political one.<br><br>2. **Ethical Considerations**: The statement acknowledges ethical concerns about animal welfare, a topic that can resonate with individuals across the political spectrum. Liberals might appreciate the emphasis on humane treatment and ethical responsibility, while conservatives might support the necessity of such research for human health and scientific advancement.<br><br>3. **Regulatory Compliance**: The call for strict adherence to standards and regulations can be seen as a balanced approach, recognizing the need for oversight (which may align more with a liberal viewpoint advocating for regulation) and the importance of the research itself (which can align with a conservative viewpoint prioritizing scientific and medical advancement).<br><br>It's important to note that the interpretation of this statement can vary among individuals with different political beliefs. Some liberals might oppose animal research on ethical grounds, while some conservatives might support it strongly for its contributions to science and health. Others in both groups might find the balanced approach of humane treatment combined with scientific advancement to be agreeable.	neutral

APA	Position Statement on Tobacco Use Disorder	12/2015	The text you've provided seems to align more with a liberal viewpoint, though it's important to recognize that public health issues like tobacco use and addiction can be viewed through various lenses and don't always fit neatly into political categories.<br><br>Here's why it might be seen as leaning liberal:<br><br>1. **Emphasis on Public Health and Prevention**: The focus on preventive measures, public education, and the development of policies to reduce tobacco use aligns with a liberal approach that often prioritizes public health initiatives.<br><br>2. **Advocacy for Comprehensive Healthcare**: The call for adequate health insurance coverage for treatments, including behavioral and pharmacological interventions, is in line with liberal views that advocate for broad and inclusive healthcare coverage.<br><br>3. **Focus on Vulnerable Populations**: The text's emphasis on the impact of tobacco on people with mental illnesses and substance use disorders, and the call for targeted interventions for these groups, reflects a liberal tendency to focus on the needs of vulnerable populations.<br><br>4. **Support for Institutional Policies**: The advocacy for tobacco-free policies in healthcare facilities and the broader society is typical of a liberal approach, which often supports institutional and governmental involvement in health-related issues.<br><br>However, it's important to note that issues like smoking cessation and public health can garner support across the political spectrum, and the strategies and policies proposed can vary based on different ideological viewpoints. The American Psychiatric Association (APA), from which the position is stated, generally aims to base its recommendations on scientific evidence and clinical best practices, which can transcend simple political categorization.	liberal
APA	Position Statement on Substance Use Disorders in Older Adults	12/2015	The text presented outlines a comprehensive position on substance use disorders among older adults, specifically addressing the need for diagnosis, treatment, and integration of services within the healthcare system. It emphasizes the importance of recognizing and treating substance use disorders in older adults as part of general medical and psychiatric care, promoting screening for co-occurring disorders, and paying attention to psychosocial stressors. Additionally, it calls for sensitivity to the unique needs of older adults, integration of mental health services into primary care, access to comprehensive mental health services, training for healthcare providers, research on cannabis use, and policy development to support these initiatives.<br><br>This position can be seen as aligning with both liberal and conservative viewpoints in different aspects:<br><br>1. **Liberal Viewpoint**:<br>- Emphasis on comprehensive healthcare access: The call for affordable and comprehensive range of mental health services, including substance use disorder services, aligns with a typically liberal viewpoint which often advocates for universal healthcare access.<br>- Focus on integration of services: The push for integration of mental health services into primary care and community-based service systems reflects a liberal approach to healthcare, emphasizing holistic, community-centered care.<br>- Policy changes: Advocating for policy changes to improve healthcare coverage, such as eliminating exclusions based on pre-existing conditions and ensuring parity in coverage, is a stance more commonly associated with liberal politics.<br><br>2. **Conservative Viewpoint**:<br>- Emphasis on personal responsibility: The position encourages older adults to be educated about their condition and seek appropriate treatment, which can align with a conservative emphasis on personal responsibility in healthcare.<br>- Training and competence in healthcare providers: The focus on training for healthcare providers at various levels could be seen as a conservative approach, emphasizing professional competence and quality of care.<br>- Research and evidence-based practices: The call for more research, especially on the effects of substances like cannabis, aligns with a conservative preference for policies and practices grounded in research and evidence.<br><br>Overall, this text represents a balanced approach, focusing on practical solutions and improvements in healthcare for older adults with substance use disorders. It reflects a blend of ideals that can appeal to both liberal and conservative viewpoints, emphasizing both individual responsibility and the need for systemic changes in healthcare.	neutral

APA	Position Statement on Recognition and Management of Substance Use Disorders and Other Mental Illnesses Comorbid with HIV	12/2015	The text you've provided outlines a position from the American Psychiatric Association (APA) regarding the treatment and care of HIV-infected individuals, particularly addressing the co-morbidity of substance abuse and psychiatric disorders. This position does not align strictly with a liberal or conservative viewpoint. Instead, it is a professional and medical stance focused on best practices and evidence-based treatment strategies.<br><br>The APA's recommendations emphasize the need for comprehensive and integrated care, encouraging psychiatrists to stay informed about effective treatments for substance use disorders, collaborate with medical colleagues for holistic patient care, and consider a wide range of potential causes when evaluating changes in mental status in HIV-infected patients.<br><br>This approach is grounded in medical science and patient care rather than political ideology. It focuses on practical and effective treatment strategies for individuals with complex medical needs, which is a hallmark of professional medical advice rather than political positioning.	neutral
APA	Position Statement on Publication of Findings from Clinical Trials	7/2015	The text provided does not align strictly with either a liberal or conservative viewpoint in the traditional political sense. It represents a position of the American Psychiatric Association (APA) on issues related to the publication rights of researchers in clinical trials and the importance of a comprehensive clinical trials registry. These positions are more closely related to scientific and ethical standards in research rather than political ideologies.<br><br>The APA's stance emphasizes the importance of transparency, the unrestricted publication of trial results regardless of the outcome, and the value of a public registry for clinical trials. These principles are geared towards ensuring research integrity, improving patient care, and promoting informed decision-making in the field of mental health. This approach is generally consistent with the broader scientific community's commitment to ethical research practices and transparency, rather than being representative of a specific political ideology.	neutral
APA	Position Statement on Psychotherapy as an Essential Skill of Psychiatrists	12/2015	The text you've provided is a position statement related to the field of psychiatry, particularly focusing on the importance of integrating psychotherapy and medication management in psychiatric care. It does not inherently align with a specific political viewpoint, whether liberal or conservative, as it is more focused on medical practice and standards within the field of psychiatry.<br><br>The statement emphasizes the value of comprehensive treatment by psychiatrists, including both medication and psychotherapy, and advocates for reimbursement policies that support integrated care rather than separating biological treatments from psychosocial interventions. It also stresses the importance of thorough training for psychiatry residents in evidence-based psychotherapies and collaborative treatment models.<br><br>This perspective is rooted in clinical practice and medical education standards rather than political ideology. It addresses the complexities of psychiatric care and the importance of training and reimbursement policies that support effective, integrated treatment for patients. Such positions typically transcend traditional political divides and are more concerned with medical efficacy and patient care standards.	neutral
APA	Position Statement on HCV Coinfection	7/2015	The text you provided appears to be a position statement or guideline from the American Psychiatric Association (APA) regarding the management and treatment of individuals with co-morbid HIV and Hepatitis C Virus (HCV) infections. It outlines the role of psychiatrists in treating these conditions, emphasizing the need for staying updated on medical knowledge, encouraging HIV and HCV testing, addressing mood disorders before HCV treatment, advocating for access to HCV treatment, collaborating with other healthcare providers, and monitoring for drug interactions and toxicities.<br><br>This text does not align explicitly with either a liberal or conservative viewpoint in the traditional political sense. It is a medical and psychiatric guideline focused on the clinical management of specific health conditions. The content is grounded in medical best practices and patient care, rather than political ideology. It addresses the medical, psychological, and social complexities associated with HIV and HCV, particularly in patients with co-morbid psychiatric conditions, and advocates for comprehensive, integrated care. This approach is typical of medical guidelines, which are generally based on scientific evidence and clinical expertise rather than political perspectives.	neutral

APA	Position Statement on Posttraumatic Stress Disorder and Traumatic Brain Injury	12/2015	The text you've provided appears to be more aligned with a non-partisan or issue-specific viewpoint rather than strictly liberal or conservative. It focuses on the importance of research, education, and care for individuals with Posttraumatic Stress Disorder (PTSD) and Traumatic Brain Injury (TBI), which are concerns that transcend traditional political divisions.<br><br>Both liberal and conservative viewpoints can support the need for medical research and improved care for veterans and victims of violence. The emphasis on mental health and the care of individuals with PTSD and TBI might be more traditionally associated with liberal policies, which often prioritize healthcare and social services. However, the focus on veterans' health is also a key concern in conservative circles, particularly given the conservative emphasis on national defense and support for the military.<br><br>Therefore, the statement from the American Psychiatric Association (APA) advocating for neuropsychiatry research and training to address these issues can be seen as a broadly humanitarian and health-oriented stance rather than one rooted in a specific political ideology.	neutral
APA	Position Statement on Medication Substitutions	7/2015	The text you've provided seems to focus on a specific policy issue related to healthcare and medication practices, rather than a broad ideological stance. It addresses the American Psychiatric Association's opposition to the practice of therapeutic interchange of psychoactive medication, including the interchangeability of generic and brand medications, without the consent of the prescribing psychiatrist.<br><br>This issue intersects with several broader topics, such as healthcare policy, patient autonomy, and pharmaceutical practices, which can be viewed through different ideological lenses. Here's how each viewpoint might approach it:<br><br>- **Liberal Viewpoint**: A liberal perspective might focus on the importance of patient access to affordable medication, hence supporting the use of generic drugs. However, they might also emphasize patient autonomy and the right to be informed and consent to changes in their medication.<br><br>- **Conservative Viewpoint**: A conservative viewpoint might prioritize the sanctity of the doctor-patient relationship and the autonomy of medical professionals in making decisions about patient care, including medication prescriptions. This could align with opposing mandatory therapeutic interchange if it's seen as infringing on this autonomy.<br><br>However, it's important to note that such healthcare issues can transcend traditional liberal-conservative divides, with opinions varying based on different facets of the issue like economic implications, patient rights, and medical ethics. The stance described in your text can be supported or opposed by individuals across the political spectrum depending on which aspect of the issue they prioritize.	neutral
APA	Position Statement on Hypnosis	12/2015	The text you've provided outlines a position on the use of hypnosis in clinical settings, as stated by the American Psychological Association (APA). This position focuses on ensuring that hypnosis is practiced by appropriately licensed and trained professionals within the scope of their expertise, alongside a thorough medical and psychiatric evaluation. It emphasizes evidence-based practice, clear structuring of the trance state, and professional credentials for training in hypnosis.<br><br>This position is primarily clinical and professional in nature and doesn't align clearly with either a liberal or conservative political viewpoint. Instead, it reflects a concern for patient safety, professional standards, and evidence-based practice in psychology and medicine. These aspects are generally considered to be non-partisan and focused on healthcare quality and ethics, rather than political ideology.	neutral
APA	Position Statement on High Volume Psychiatric Practice and Quality of Patient Care	12/2015	The statement from the American Psychiatric Association (APA) focuses on maintaining the quality and safety of psychiatric treatment, emphasizing that financial, organizational, or administrative pressures should not compromise patient care. This statement does not inherently align with a strictly liberal or conservative viewpoint. Instead, it reflects professional and ethical standards relevant to psychiatric practice.<br><br>Both liberal and conservative perspectives generally agree on the importance of high-quality healthcare and patient safety, although they might differ in their approaches to healthcare policy and administration. The APA statement centers on the ethical responsibility of psychiatrists to prioritize patient care over non-clinical pressures, which is a principle that transcends political ideologies. It emphasizes the need for clinical decisions to be based on medical standards rather than external administrative or financial pressures. This focus on professional ethics and patient welfare is a universal concern in the healthcare field, making it non-partisan in nature.	neutral

APA	Position Statement on Electroconvulsive Therapy (ECT)	7/2015	The statement about Electroconvulsive Therapy (ECT) being a safe and effective evidence-based medical treatment, and its endorsement by the American Psychiatric Association (APA) for use by qualified psychiatrists on appropriately selected patients, does not inherently align with a specific political viewpoint, either liberal or conservative.<br><br>This statement is primarily medical and scientific in nature, focusing on the efficacy and safety of ECT as a treatment, as well as the importance of its administration by qualified professionals. Such statements are generally based on clinical evidence and medical consensus rather than political ideologies.<br><br>Both liberal and conservative viewpoints can support evidence-based medical treatments, although the degree and manner of their support might vary based on broader political beliefs about healthcare systems, medical ethics, and government involvement in healthcare. However, the statement itself is neutral and focuses on medical facts and professional standards rather than political beliefs.	neutral
APA	Position Statement on Confidentiality of Electronic Health Information	7/2015	The provided text addresses the issue of electronic health records (EHRs) and health information exchanges (HIEs), focusing on the benefits of these technologies for health care delivery and quality, as well as the challenges related to security, confidentiality, and privacy. The stance presented does not align exclusively with either a liberal or conservative viewpoint in the traditional political sense. Instead, it encompasses concerns that are relevant to both perspectives:<br><br>1. **Privacy and Confidentiality:** The emphasis on protecting patients' privacy and confidentiality aligns with values that are important across the political spectrum. Both liberal and conservative viewpoints generally agree on the importance of safeguarding personal information, although the specific approaches to achieving this might differ.<br><br>2. **Advancement of Technology in Healthcare:** The recognition of the benefits of computerized medical records for improving healthcare delivery is a viewpoint that transcends political ideologies. Both liberals and conservatives can support technological advancements that enhance healthcare efficiency and effectiveness.<br><br>3. **Government Oversight and Security:** The concern over government agencies potentially accessing private health information relates to broader debates about government surveillance and individual privacy. Liberals might focus more on the protection of individual rights and freedoms, while conservatives might emphasize the need for security measures, though these concerns are not exclusive to either side.<br><br>4. **Patient Autonomy and Consent:** The idea that patients should have control over their health information and be able to give informed consent aligns with principles of individual autonomy and self-determination, which are valued by both liberal and conservative philosophies.<br><br>In summary, the text presents a multifaceted viewpoint that incorporates elements relevant to both liberal and conservative perspectives, focusing on the balance between technological advancement in healthcare and the protection of individual privacy and autonomy.	neutral
APA	Position Statement on Bias-Related Incidents	12/2015	The text you provided is primarily focused on addressing bias-related incidents, their effects, and the position of the American Psychiatric Association (APA) in opposing them. This issue, while deeply relevant to both liberal and conservative viewpoints, isn't inherently aligned with either side. Instead, it addresses a broad, non-partisan concern about social justice, mental health, and equity.<br><br>The description of bias-related incidents and their impacts on individuals and society reflects a concern for social justice and equality, themes often associated with liberal viewpoints. Liberals typically emphasize the importance of recognizing and addressing various forms of discrimination and bias to promote inclusivity and equality.<br><br>However, the emphasis on the well-being of individuals and communities can also resonate with certain conservative values, particularly those that prioritize social stability and the health of the community. Conservatives may also recognize the detrimental effects of such incidents, although they might differ in their approaches to addressing them.<br><br>In summary, while the text touches on issues often discussed within liberal circles, its focus on the well-being of individuals and communities can be relevant across the political spectrum. The APA's position of opposing bias-related incidents and encouraging active responses to prevent and address them is a stance that transcends simple political categorization, aiming instead at broader humanistic and societal goals.	probably liberal

APA	Position Statement on Opioid Overdose Education and Naloxone Distribution	12/2015	The text you provided presents an issue and a position that don't align strictly with either a liberal or conservative viewpoint as traditionally understood in U.S. politics. The issue of increasing mortality from prescription drug overdoses and the response to it, as suggested by the American Psychiatric Association and the American Academy of Addiction Psychiatry, transcends typical partisan lines and focuses more on public health and safety measures.<br><br>The emphasis on expanded access to naloxone, training for various individuals (including law enforcement and family members of those at risk), and the support for Good Samaritan laws and broader distribution of naloxone, are primarily public health-oriented responses. They aim to reduce harm and provide emergency assistance in the case of opioid overdoses. These measures are generally supported across the political spectrum, as they address a public health crisis rather than a partisan issue.<br><br>However, the broader context of how opioid addiction and drug policy are addressed can sometimes fall into more traditionally liberal or conservative viewpoints. For example, conservatives might emphasize law enforcement and border control to stop drug trafficking, while liberals might focus on healthcare access and treatment for addiction. But the specific measures described in your text, particularly around naloxone access and overdose response, are more universally recognized as necessary public health interventions.	neutral
APA	Position Statement on the Provision of Psychotherapy for Psychiatric Residents	12/2014	The text you provided from the American Psychiatric Association (APA) focuses on advocating for the mental health and wellbeing of psychiatric residents, emphasizing the importance of access to affordable and confidential psychiatric services, including individual psychotherapy. This stance primarily reflects a concern for healthcare accessibility and professional ethics in medical training rather than aligning directly with a specific political ideology.<br><br>However, its emphasis on healthcare access, mental health, and the importance of non-discriminatory practices could be seen as aligning with liberal viewpoints, which often prioritize healthcare accessibility, mental health awareness, and anti-discrimination measures. Liberals generally advocate for the rights and well-being of all individuals, including those in professional settings, and support policies that promote equitable access to healthcare services.<br><br>Conservatives, on the other hand, might not necessarily oppose these ideas, but their focus tends to be more on personal responsibility, limited government intervention in healthcare, and the autonomy of private institutions. They might view such guidelines as overreach or unnecessary regulation, depending on their perspective on healthcare policy and professional training standards.<br><br>In conclusion, while the APA's statement reflects a commitment to healthcare accessibility and ethical standards that could be more closely associated with liberal values, it doesn't exclusively align with a liberal or conservative viewpoint. It represents a professional stance focused on the wellbeing and ethical treatment of psychiatric residents.	probably liberal

APA	Rights and Regulations	12/2014	The text you've provided outlines several position statements and guidelines related to psychiatrists practicing in managed care environments. These statements focus on the rights and regulations of psychiatrists, the definition and importance of medical necessity, the relationship between psychiatrists and their patients, and the interaction with managed care organizations.<br><br>Determining whether this text aligns with a liberal or conservative viewpoint is not straightforward, as the issues presented here can be interpreted through different ideological lenses. However, some general observations can be made:<br><br>1. **Emphasis on Professional Autonomy and Patient Advocacy**: The text emphasizes the autonomy of psychiatrists to practice according to their training and the best interests of their patients. This stance, advocating for professional discretion and patient-centered care, can align with both liberal and conservative viewpoints. Liberals might support it due to its focus on individual healthcare rights, while conservatives might appreciate the emphasis on professional autonomy and less interference from managed care organizations.<br><br>2. **Medical Necessity Defined by Professional Bodies**: The insistence that medical necessity be defined by professional bodies like the AMA and APA, rather than solely by insurance or managed care entities, can also be seen in both liberal and conservative lights. Liberals may view this as protecting patients from cost-cutting measures that might compromise care, while conservatives might see it as ensuring decisions are made by medical professionals rather than bureaucrats.<br><br>3. **Transparency and Fair Practices in Managed Care**: The call for transparency, fair peer review, and reasonable termination practices in managed care might be universally appreciated across the political spectrum. It represents a desire for fair and ethical business practices, which is a concern shared by many, regardless of political affiliation.<br><br>4. **Regulatory Oversight and Standards**: The reference to standards set by organizations like NCQA and URAC and the call for reasonable fees and prompt payment might be more aligned with a liberal perspective that often advocates for regulatory oversight to ensure fairness and quality in healthcare.<br><br>In summary, while certain aspects of this text might be more resonant with one political ideology than another, the overall content reflects concerns and principles that could be supported by individuals across the political spectrum. It focuses more on professional ethics, quality of care, and fair practices in healthcare, which are issues of general concern rather than strictly partisan.	neutral
APA	Position Statement on Prior Authorizations for Psychotropic Medications	7/2014	The text you provided reflects a position on the issue of requiring prior authorization for psychotropic medications by insurance companies or other entities. It's important to understand that the classification of viewpoints as "liberal" or "conservative" can be overly simplistic, especially when discussing healthcare policies, which often involve complex considerations of public health, economics, and regulatory practices.<br><br>This position statement advocates for minimizing bureaucratic obstacles in prescribing psychotropic medications, emphasizing the need for timely patient access to necessary treatments and reducing the administrative burden on psychiatrists. It argues against prior authorization except in specific, justified cases, and suggests that any such decisions should be made by a Board Certified Psychiatrist.<br><br>This viewpoint could be interpreted in different ways:<br><br>1. **Liberal Perspective**: It might align with a more liberal viewpoint in that it advocates for fewer restrictions on access to healthcare and emphasizes the importance of patient care over cost-saving measures by insurance companies. The focus on patient rights and reducing administrative burdens on healthcare providers can be seen as aligning with liberal values of individual healthcare rights and skepticism of corporate (insurance companies) control over healthcare decisions.<br><br>2. **Conservative Perspective**: Conversely, one could argue that the emphasis on professional autonomy and decision-making by psychiatrists, rather than by insurance companies or government entities, aligns with conservative values favoring professional discretion and reduced regulatory oversight.<br><br>3. **Beyond Political Dichotomies**: This position could also be seen as a professional stance by a medical association, focused on best practices and patient care quality, which transcends typical liberal-conservative dichotomies. It reflects a specialized, practical viewpoint based on the experiences and expertise of psychiatric professionals, rather than a political ideology.	neutral

			In conclusion, while different aspects of this position can align with both liberal and conservative viewpoints, it is primarily a professional stance focused on healthcare practices rather than a straightforward political position.	
APA	Position Statement on Federal Exemption from the Medicaid Institutions for Mental Diseases (IMD) Exclusion	12/2014	The text you provided discusses a policy proposal related to mental health and substance abuse treatment, specifically regarding Federal exemptions for certain healthcare facilities from the IMD (Institutions for Mental Diseases) Exclusion. The IMD Exclusion is a policy that restricts federal Medicaid funding for adults in mental health and substance abuse residential treatment facilities with more than 16 beds.<br><br>Analyzing this text from a political viewpoint is not straightforward, as mental health policy can cross traditional political lines. However, here are some general observations:<br><br>1. **Liberal Viewpoint**: Liberals generally advocate for increased funding and support for mental health services. This proposal, which seems to aim at expanding access to federal funding for larger mental health and substance abuse treatment facilities, could align with liberal priorities. Additionally, the emphasis on maintaining or increasing state expenditures in this area might also resonate with liberal views on social welfare and public health.<br><br>2. **Conservative Viewpoint**: Conservatives typically emphasize state autonomy and might support aspects of this proposal that allow states more flexibility in managing their mental health services. However, conservatives are also generally cautious about expanding federal programs or spending. The requirement for states to maintain or increase their mental health spending could be viewed as federal overreach, which might not align with conservative principles.<br><br>3. **Bipartisan Elements**: Mental health policy is often a bipartisan issue, with both liberals and conservatives recognizing the importance of addressing mental health and substance abuse. This proposal, by offering states more options and potentially improving mental health services, might find support across the political spectrum.<br><br>In summary, the proposal contains elements that might appeal to both liberal and conservative viewpoints, though its acceptance would depend on how individual policymakers prioritize aspects of healthcare funding, state autonomy, and federal involvement in public health.	neutral

APA	Position Statement on Therapies Focused on Memories of Childhood Physical and Sexual Abuse	7/2013	The text you've provided appears to be a position statement regarding the treatment of individuals with memories of childhood abuse, specifically focusing on the challenges and complexities of eliciting and interpreting such memories in therapy. It does not align strictly with either a liberal or conservative viewpoint in the traditional political sense. Instead, it addresses a nuanced and specialized area of mental health practice.<br><br>The statement emphasizes a balanced and cautious approach to dealing with memories of childhood abuse in therapy, recognizing the potential for both true and false memories, and the importance of a neutral, empathetic stance in psychiatric evaluation and treatment. It acknowledges the complexities involved in memory recall, especially under stress, and the influence that therapy can have on shaping memories.<br><br>This approach aligns with general principles of professional ethics and evidence-based practice in mental health care, rather than with a specific political ideology. It stresses the importance of corroborating evidence, the avoidance of prejudgment, and the need for further research and education on these topics.<br><br>The statement's focus on professional responsibility, ethical treatment, and the need for research might resonate with values commonly associated with both liberal and conservative perspectives, such as the importance of scientific inquiry (often emphasized in liberal ideologies) and the concern for individual rights and the risks of false accusations (which can align with conservative viewpoints). However, it primarily reflects clinical and ethical considerations rather than political ones.	neutral
APA	Position Statement on Somatic Cell Nuclear Transfer	12/2013	The statement regarding the American Psychiatric Association's support for stem cell research, including the use of somatic cell nuclear transfer (SCNT) for therapeutic but not reproductive purposes, tends to align more with a liberal viewpoint.<br><br>Liberal perspectives generally advocate for scientific and medical research, including stem cell research, as they often prioritize advancements in healthcare and treatments for diseases. The support for therapeutic applications of SCNT, which could lead to significant medical breakthroughs, aligns with this approach.<br><br>Conservative viewpoints, especially those influenced by religious or ethical considerations, may be more cautious or opposed to certain types of stem cell research, particularly if it involves embryonic stem cells or cloning techniques. The distinction made in the statement between therapeutic and reproductive purposes might be an attempt to address some of these ethical concerns.<br><br>However, it's important to note that the dichotomy of 'liberal' versus 'conservative' can oversimplify complex issues. Views on stem cell research can vary widely within both political spectrums, influenced by ethical, religious, and scientific perspectives.	liberal
APA	Position Statement on School-Based Health Clinics	12/2013	The statement about the American Psychiatric Association (APA) supporting the allocation of resources for the participation of child and adolescent psychiatrists with School-Based Health Centers (SBHCs) doesn't neatly align with a strictly liberal or conservative viewpoint. It's more focused on mental health care in an educational setting, emphasizing the need for resources and expertise in this area. This issue transcends traditional political lines, as it deals with mental health and the well-being of children and adolescents, concerns that are generally shared across the political spectrum.<br><br>However, the nuances of how it's perceived could vary based on political beliefs:<br><br>1. **Liberal Viewpoint**: A liberal perspective might emphasize the importance of accessible mental health care for all, including in schools. They might see this as a part of broader social services and support the investment in mental health professionals in educational settings.<br><br>2. **Conservative Viewpoint**: A conservative perspective might also recognize the value of mental health services but may have differing opinions on the method of implementation and funding. They might support such initiatives but prefer solutions that involve private sector participation or local control rather than federal involvement.<br><br>Overall, the statement is more about public health policy and the well-being of children and adolescents than about a particular political ideology.	neutral

APA	Position Statement on Newborn Infant Adoptions	12/2013	The text you've provided outlines guidelines and recommendations regarding decision-making in the newborn adoptive process, focusing on the best interest of the child, the competence and needs of the biological parents, and the importance of secure attachments and stable relationships in the adoptive family. It emphasizes the need for timely finalization of adoptions and treats the disruption of adoptive family relationships with the same seriousness as those in biologically intact families.<br><br>This text does not neatly align with a specific liberal or conservative viewpoint. Instead, it reflects a child-centric, ethical approach to adoption, emphasizing the well-being of the child and the responsibilities of both biological and adoptive parents. The focus on the child's best interest, the importance of stable family relationships, and the careful consideration of adoption finalization and disruption are principles that can resonate across the political spectrum.<br><br>In general, liberal viewpoints often emphasize individual rights, social equality, and support for social services, while conservative viewpoints typically emphasize traditional values, individual responsibility, and limited government intervention. However, in the context of adoption and child welfare, these lines can blur, as both liberals and conservatives can agree on the paramount importance of the child's well-being and the need for responsible, ethical practices in the adoption process.	neutral
APA	Position Statement on Legal Proceedings and Access to Psychiatric Care for Juvenile Offenders	7/2013	The text you've provided appears to be a balanced and nuanced discussion on the topic of juvenile adjudication, blending aspects that could align with both liberal and conservative viewpoints.<br><br>1. **Liberal Viewpoint Elements**:<br>- **Emphasis on Rehabilitation and Mental Health**: The focus on rehabilitation, particularly for juveniles with mental illness and neurodevelopmental disorders, aligns with liberal perspectives that often prioritize treatment and rehabilitation over punishment.<br>- **Opposition to Automatic Transfer to Adult Court**: Liberals typically oppose policies that treat juvenile offenders as adults without considering individual circumstances and developmental factors.<br>- **Support for Judicial Discretion and Individualized Assessment**: The emphasis on individualized judicial decisions and the importance of comprehensive psychiatric assessments reflect a liberal inclination towards personalized justice rather than one-size-fits-all solutions.<br><br>2. **Conservative Viewpoint Elements**:<br>- **Acknowledgment of Public Safety and Serious Offenses**: The mention of state statutes that consider the seriousness of the offense and public safety risks can align with conservative views that emphasize law and order and the protection of society.<br>- **Use of Judicial and Prosecutorial Discretion**: While the text supports discretion, it also suggests a framework where judges and prosecutors have significant authority to make decisions, which can resonate with conservative principles favoring a strong judicial system.<br><br>3. **Balanced Approach**:<br>- The text appears to strive for a balance between protecting society (a more conservative concern) and rehabilitating young offenders (a more liberal concern).<br>- It acknowledges the complexity of juvenile adjudication and the need for a multifaceted approach that considers the severity of the offense, the individual's mental health, and the potential for rehabilitation.<br><br>In summary, the text presents a comprehensive view that incorporates elements from both liberal and conservative ideologies, focusing on a balanced approach to juvenile justice that considers individual circumstances, public safety, and the potential for rehabilitation.	neutral
APA	Position Statement on Issues Related to Homosexuality	12/2013	The text you provided reflects a viewpoint that is generally more aligned with liberal ideologies. The emphasis on the equality and normalcy of same-sex attraction, opposition to discrimination based on sexual orientation, support for same-sex marriage, and the recognition of the harmful effects of societal stigma are typically associated with liberal perspectives. These viewpoints prioritize individual rights, social equality, and the acknowledgement of diverse sexual orientations as normal aspects of human behavior, which are key elements often found in liberal ideology.<br><br>Conservative viewpoints, in contrast, might emphasize more traditional views on family and marriage, and may be less supportive of changes to established social norms regarding sexual orientation. However, it's important to note that not all conservatives or liberals adhere strictly to these generalized positions, as there is a wide range of beliefs within each political ideology.	liberal

APA	Position Statement on Syringe Exchange Programs	7/2012	The text you provided aligns more closely with a liberal viewpoint. Liberal perspectives generally emphasize public health approaches to drug use, focusing on harm reduction strategies. Syringe exchange programs, as described in your text, are a hallmark of this approach. They aim to reduce the transmission of diseases like HIV and hepatitis among intravenous drug users (IDUs) without necessarily addressing the legality of drug use itself.<br><br>In contrast, conservative viewpoints typically emphasize law enforcement and abstinence-based approaches to drug use. These perspectives often prioritize criminal justice responses over public health strategies and may view syringe exchange programs as tacitly endorsing or facilitating illegal drug use.<br><br>The emphasis in the provided text on providing comprehensive healthcare services, including counseling, medical care, and referrals to treatment, alongside syringe exchange, aligns with a holistic public health approach often advocated by liberal viewpoints. The support for removing government restrictions on sterile syringes and broadening the availability of needle exchange programs also reflects a liberal stance focused on harm reduction and public health rather than criminalization.	liberal
APA	Protocols and Protections	7/2012	The text you've provided focuses on guidelines and policies for health care workers regarding the occupational risk of HIV infection. It emphasizes the importance of education, the implementation of safety protocols, confidentiality, non-discrimination, and access to medical and mental health resources. This text does not align strictly with a conservative or liberal viewpoint. Instead, it presents a factual, medical, and ethical approach to handling occupational HIV risks in health care settings. The guidelines prioritize the safety and rights of health care workers, emphasizing informed consent, confidentiality, and appropriate medical response, which are principles that transcend political affiliations.	neutral
APA	Position Statement on HIV-Infected Psychiatrists	7/2012	The text you provided does not align strictly with either a liberal or conservative viewpoint in the traditional political sense. Instead, it focuses on ethical and professional guidelines related to the treatment and management of HIV among psychiatrists. Key points include:<br><br>1. **Compassionate Treatment and Privacy:** Advocating for compassion, respect for rights, privacy, and confidentiality for individuals with HIV, which can be seen as aligning with broader human rights and healthcare privacy concerns.<br><br>2. **Professional Obligations and Guidelines:** Emphasizing the physician's duty to do no harm and adherence to CDC guidelines, which is more about professional ethics and standards in healthcare.<br><br>3. **Non-Discrimination:** The stance against discrimination based on HIV status, especially in licensing, hospital privileges, or admission to medical training programs, aligns with anti-discrimination principles found in laws like the Americans with Disabilities Act of 1990.<br><br>4. **Confidentiality and Clinical Competence:** Highlighting the importance of confidentiality in reporting HIV/AIDS cases and addressing clinical competence impairments due to any illness, not just HIV.<br><br>These points primarily reflect medical ethics and legal considerations rather than a specific political ideology. They are centered on fair treatment, non-discrimination, and adherence to established medical guidelines and legal frameworks, which are principles that can be endorsed by individuals across the political spectrum.	neutral

APA	Position Statement on HIV Infection and Pregnant Women	12/2012	The text you provided appears to focus on medical and ethical guidelines for the treatment and prevention of HIV transmission from pregnant women to their children. It doesn't align strictly with a liberal or conservative viewpoint in the political sense, as it is primarily a medical issue.<br><br>The recommendations emphasize the importance of HIV testing, counseling, and treatment for pregnant women, which is a public health approach rather than a partisan issue. Both liberal and conservative viewpoints generally support public health measures, although the methods and extent of implementation might vary based on political beliefs.<br><br>For example, the emphasis on "opt-out" testing and not requiring separate consent for HIV testing could be seen as a public health priority, aiming to maximize testing and prevention efforts. This approach might be more aligned with a liberal perspective that emphasizes collective welfare and public health measures. On the other hand, conservatives might support these measures too, especially if they are seen as effective ways to reduce healthcare costs and prevent disease spread.<br><br>However, aspects like personal choice and the extent of government involvement in healthcare can be points of divergence between liberal and conservative viewpoints. Liberals might focus more on the availability and accessibility of such healthcare services, while conservatives might emphasize personal responsibility and limited government intervention.<br><br>In summary, the text is primarily medical in nature, addressing a significant public health issue, and does not neatly align with a specific political ideology. The focus is on effective management and prevention strategies for HIV transmission, which can be supported across the political spectrum, albeit with different emphases.	neutral
APA	Position Statement on Proposed Legislation on Guns on College and University Campuses	7/2011	The text you've provided aligns more closely with a liberal viewpoint. It emphasizes public health and safety concerns regarding firearms on educational campuses and opposes legislation that would allow guns to be carried on campus. This position is generally associated with liberal perspectives, which often advocate for stricter gun control measures to enhance public safety and reduce gun violence. Conservative viewpoints, in contrast, tend to emphasize Second Amendment rights and may support legislation that allows for more freedom in carrying firearms, including on educational campuses.	liberal
APA	Position Statement on Physician Wellness	12/2011	The statement "Psychiatrists are important role models for their patients and peers, making their own personal health an influential factor in effective health promotion and disease prevention practices" does not neatly align with a specific liberal or conservative viewpoint. It is a professional and health-related perspective, emphasizing the role of psychiatrists in health promotion, disease prevention, and wellness.<br><br>This viewpoint centers on the idea that psychiatrists, by maintaining their own health, can positively influence their patients and peers. It touches on topics like mental health, addiction, and occupational stress, which are generally considered important across various political spectrums. However, the emphasis on personal responsibility for health and the role of professionals in setting an example might resonate more with conservative ideals that focus on individual responsibility. At the same time, the acknowledgment of issues like addiction and mental illness and the need for treatment could align with liberal perspectives that often advocate for comprehensive healthcare and mental health services.<br><br>Overall, the statement is more aligned with medical and professional ethics rather than being distinctly political.	neutral
APA	Position Statement on Homosexuality (Call for International Action to Repeal Discriminatory Legislation)	7/2011	The text you've shared aligns more closely with a liberal viewpoint. It emphasizes the non-pathological nature of homosexuality and advocates for the repeal of laws that penalize homosexual acts among consenting adults. Additionally, it calls for efforts to reduce the stigma associated with homosexuality. This perspective is typically associated with liberal ideologies, which often prioritize individual rights, social equality, and oppose discrimination based on sexual orientation. Conservative viewpoints, on the other hand, have historically been more likely to support traditional social norms and may have differing views on the legislation and social acceptance of homosexuality. However, it's important to note that viewpoints on such issues can vary widely within both liberal and conservative circles.	liberal

APA	AACAP Position Statement on Mental health, Children and Disasters	12/2011	The text you provided does not explicitly align with either a liberal or conservative viewpoint. It focuses on the support of the American Psychiatric Association (APA) and the American Academy of Child and Adolescent Psychiatry (AACAP) for the recommendations of the National Commission on Children and Disasters in 2010. This statement emphasizes the importance of mental health preparedness for children and families in the context of disasters, advocating for research, training, and collaborative efforts with various governmental organizations.<br><br>This approach is centered on public health and disaster preparedness, areas that typically transcend traditional political divisions. Both liberal and conservative viewpoints might agree on the importance of addressing mental health needs, especially in the context of disasters and emergencies. However, the specific policies or methods for implementing these recommendations could be interpreted or supported differently by individuals with different political beliefs.	neutral
APA	APA Position Statement on Bullying-Related Morbidity and Mortality	3/2011	The text you provided primarily presents a professional and scientific perspective on bullying, as outlined by the American Academy of Child and Adolescent Psychiatry (AACAP) and the American Psychiatric Association (APA). It focuses on the psychiatric, medical, and public health risks associated with bullying, emphasizing its widespread occurrence and the need for prevention and intervention.<br><br>This text does not align explicitly with either a liberal or conservative political viewpoint. Instead, it is based on scientific evidence and professional expertise in psychiatry and mental health. The emphasis is on the need for awareness, prevention, intervention, and treatment of bullying, which are issues that transcend political lines and are generally considered important in public health and education, regardless of political affiliation.<br><br>In terms of political viewpoints, liberal perspectives often prioritize social welfare, public health initiatives, and educational programs, which might align with the advocacy for bullying prevention and intervention mentioned in the text. On the other hand, conservative viewpoints might also support such initiatives, especially if they align with values of community safety and individual responsibility.<br><br>Overall, the text is more aligned with professional and scientific perspectives rather than being distinctly liberal or conservative in nature.	neutral
APA	With Special Interest for Clinical Practice and Research	12/2010	The text you've provided does not straightforwardly align with either a liberal or conservative viewpoint as it primarily focuses on ethical practices in the medical field. The emphasis is on evidence-based practice, ethical research, and managing conflicts of interest in medical research and practice. These principles are generally considered essential in the medical community, regardless of political orientation.<br><br>Liberal and conservative viewpoints typically differ in areas such as social policy, economic policy, and the role of government, rather than on the principles of medical ethics and practice. The text emphasizes the importance of scientific validity, ethical conduct, and the avoidance of conflicts of interest, which are universal professional standards in medicine.<br><br>Thus, the text reflects professional and ethical standards in the medical field rather than a specific political ideology.	neutral
APA	Position Statement Regarding Hypnosis	9/2009	The text provided seems to be a neutral, informative overview of hypnosis as a medical and psychiatric practice rather than a statement aligning with a specific political ideology. It discusses the use, effectiveness, and training associated with hypnosis without advocating for or against it from a policy perspective.<br><br>The text focuses on the clinical and therapeutic aspects of hypnosis, emphasizing its role in treatment and the importance of proper training for those who practice it. It also touches on the potential dangers of improper use. This information is presented in a factual manner, likely intended for an audience interested in the medical and psychological fields.<br><br>There isn't an explicit liberal or conservative viewpoint present. Both liberal and conservative ideologies might support the use of hypnosis in clinical settings for different reasons, such as improving mental health care or offering alternative treatments to reduce healthcare costs. However, the text itself doesn't delve into these broader political or ideological discussions. Instead, it stays focused on the clinical and educational aspects of hypnosis.	neutral

APA	Position Statement on Tobacco Dependence	9/2009	The text you've provided regarding the American Psychiatric Association's encouragement for psychiatrists to diagnose and treat nicotine dependence, recognize the role of nicotine in psychiatric disorders, and participate in advocacy and research concerning tobacco use does not inherently align with either a liberal or conservative viewpoint. It is primarily a health-focused statement, addressing a medical issue (nicotine dependence and its relation to psychiatric disorders) from a clinical and scientific perspective.<br><br>Healthcare and medical guidelines like these are typically based on scientific research and clinical evidence, aiming to improve patient care and outcomes. They usually do not align directly with political ideologies but are focused on best practices in medicine and public health. However, the interpretation of such guidelines can sometimes be influenced by individual or political beliefs, especially when it comes to topics like public health policy and advocacy.	neutral
APA	Position Statement on Interference with Scientific Research and Medical Care	9/2009	The text you provided appears to align with a viewpoint that prioritizes the protection of scientific research, medical care, and patient rights. This stance can be seen as a blend of both liberal and conservative viewpoints:<br><br>1. **Liberal Viewpoint**: The emphasis on safeguarding scientific research and the provision of legal medical care, as well as the strong stance against stereotyping patients, aligns with liberal values. Liberals often advocate for the protection and advancement of scientific research, particularly in areas like medical psychotherapy, and are generally supportive of measures that ensure equal access to healthcare. Additionally, opposing stereotypes and ensuring the dignity of patients, especially in the context of mental health, is a position frequently associated with liberal ideology.<br><br>2. **Conservative Viewpoint**: The text also could resonate with conservative values, especially those related to the rule of law and the protection of legal medical practices. Conservatives often emphasize the importance of respecting legal institutions and practices, which includes the lawful conduct of scientific research and medical treatment. The objection to violence and intimidation in impeding legal activities could also align with conservative principles.<br><br>Overall, the text seems to reflect a non-partisan, professional standpoint more than a strictly political one. It focuses on upholding the integrity of medical treatment and research, as well as the rights and dignity of patients, which are concerns that can be shared across the political spectrum.	neutral
APA	AIDS and Confidentiality, Disclosure and Protection of Others	9/2009	The text you've provided outlines ethical guidelines for psychiatrists regarding confidentiality, informed consent, and notification in the context of HIV risk and infection. It does not align strictly with either a liberal or conservative viewpoint. Instead, it reflects ethical considerations in medical practice, particularly in psychiatry.<br><br>The guidelines balance the need for patient confidentiality with the responsibility to prevent harm to others. This approach is consistent with general ethical principles in healthcare, such as beneficence (doing good), nonmaleficence (not causing harm), and respect for autonomy.<br><br>In a political context, liberals typically advocate for individual rights and protections, including privacy and informed consent in healthcare. Conservatives often emphasize personal responsibility and the protection of society, which can include measures to prevent harm to others.<br><br>This text, however, focuses on ethical considerations within the medical field rather than political ideologies. It aims to guide psychiatrists in making decisions that respect patient confidentiality while also considering the welfare of others, particularly in cases where there is a risk of HIV transmission. These guidelines are designed to help navigate complex situations where ethical principles may be in tension with each other.	neutral
APA	Position Statement on HIV and Outpatient Psychiatric Services	9/2009	The text you've provided appears to focus on guidelines and ethical considerations for psychiatrists in treating patients with HIV/AIDS, particularly in the context of psychiatric services. It emphasizes the importance of respecting patient confidentiality, providing appropriate care regardless of HIV status, and ensuring both patient and staff safety.<br><br>This text doesn't neatly align with a strictly liberal or conservative viewpoint as these terms are typically understood in political discourse. Instead, it reflects a professional and ethical stance that prioritizes patient care, confidentiality, and public health concerns, which are principles generally upheld across various political spectrums.<br><br>The guidelines emphasize the importance of non-discrimination, patient education, and the responsible management of HIV/AIDS in a psychiatric setting. These are principles that can be supported by a range of political ideologies, as they are grounded in medical ethics and public health, rather than in political doctrine.	neutral

			Therefore, it would be more accurate to classify this text as aligning with medical ethics and public health principles rather than with a specific political viewpoint.	
APA	Position Statement on HIV and Inpatient Psychiatric Services	9/2009	The text you've provided appears to be a set of guidelines or position statements regarding the care and management of psychiatric patients with HIV or at risk of HIV. These guidelines focus on non-discriminatory care, the importance of universal precautions, the need for comprehensive counseling and education, and the respect for patient privacy and rights.<br><br>The viewpoints expressed in these guidelines do not align squarely with traditional liberal or conservative political ideologies. Instead, they reflect a medically and ethically informed approach to patient care. The emphasis on equal treatment, patient education, and respect for privacy can be seen as aligning with liberal values that prioritize individual rights and social justice. On the other hand, the focus on responsible management of patient behavior to prevent disease transmission could be seen as aligning with conservative values emphasizing personal responsibility and safety.<br><br>In summary, while some aspects of these guidelines might resonate more with one political ideology than the other, the overall approach is primarily guided by medical ethics and best practices rather than a specific political viewpoint.	neutral
APA	Position Statement on HIV and Adolescents	9/2009	The text you provided does not align strictly with either a liberal or conservative viewpoint. It focuses on medical and psychiatric guidelines and best practices for dealing with adolescents at risk of or affected by HIV. These guidelines emphasize education, cultural competency, understanding legal aspects, and providing comprehensive care.<br><br>Key points in the text include:<br>- The importance of psychiatrists being educated about HIV, especially as it relates to youth.<br>- The need for psychiatry training programs to include HIV-related training.<br>- The comprehensive evaluation of adolescent sexual behavior and substance use, with a focus on HIV risk assessment.<br>- Emphasizing culturally competent family assessments and involving families and other community organizations in education and intervention.<br>- Tailoring prevention strategies to individuals, focusing on those at higher risk of HIV exposure.<br>- Understanding legal aspects of HIV testing, sexual activity, and medical treatment for minors.<br>- Addressing the psychological impact of HIV on adolescents, including issues related to family members or peers who are HIV positive.<br><br>These guidelines are grounded in medical ethics and public health principles, aiming to provide the best possible care and prevention strategies for adolescents at risk of or affected by HIV. They do not inherently lean towards a specific political ideology but rather focus on the health and wellbeing of the targeted population.	neutral

APA	Position Statement on Psychiatric Hospitalization of Children and Adolescents	2008	The text provided doesn't neatly align with a specific liberal or conservative viewpoint. Rather, it focuses on ethical and professional standards in the psychiatric treatment of children and adolescents. Key aspects of this statement include:<br><br>1. **Emphasis on Medical Necessity and Ethics**: The text underscores the importance of medical necessity and ethics in admitting young patients for psychiatric care. This approach is consistent with a general healthcare philosophy that prioritizes patient well-being over financial considerations.<br><br>2. **Concern About Under-Treatment**: The statement highlights the issue of many children and adolescents not receiving necessary psychiatric care. This concern transcends political lines and focuses on a gap in healthcare provision.<br><br>3. **Role of Psychiatrists**: The text stresses the role of psychiatrists, particularly child and adolescent specialists, in making admission and treatment decisions. This approach promotes professional judgment and expertise in healthcare, a view generally supported across the political spectrum.<br><br>4. **Recognition of Systemic Issues**: The statement acknowledges systemic issues like the shortage of treatment facilities and trained staff, and the challenges posed by insurance and managed care systems. These concerns are typically addressed in broader healthcare policy debates, which can have both liberal and conservative perspectives.<br><br>5. **Call for Comprehensive Care**: There's an advocacy for a full spectrum of psychiatric services for children and adolescents, indicating a desire for a robust healthcare system that can address various needs. This is a healthcare-focused view that may resonate with both liberal and conservative ideologies, depending on the context of the discussion around healthcare funding and management.<br><br>In summary, the statement is more aligned with healthcare professionalism and ethics than with a specific political ideology. It addresses concerns that are relevant to the field of psychiatric care for children and adolescents, transcending traditional political divisions.	neutral
APA	Position Statement on HIV and Crystal Methamphetamine	12/2008	The text provided outlines a series of guidelines for psychiatrists working with patients who use or are at risk of using methamphetamine. It's important to note that the content is primarily clinical and focuses on medical best practices rather than aligning with a specific political ideology. However, aspects of the approach can be seen as aligning with certain liberal or conservative viewpoints:<br><br>1. **Integrated Interdisciplinary Treatment Model**: This approach, emphasizing collaboration among different healthcare professionals, is generally consistent with a more progressive or liberal viewpoint that values comprehensive, holistic care.<br><br>2. **Addressing Family and Domestic Violence Issues**: This aspect aligns more with liberal viewpoints, which often emphasize the importance of addressing social and familial factors in healthcare.<br><br>3. **Treating Co-occurring Psychiatric Disorders**: This is a standard medical practice and doesn't align distinctly with a political viewpoint. Both liberal and conservative perspectives generally support the treatment of co-occurring disorders in the context of substance abuse.<br><br>4. **Promoting a Recovery Model with Evidence-Based Practices**: The emphasis on evidence-based practices is a hallmark of modern medical practice and does not align strongly with a political viewpoint. However, the inclusion of harm reduction approaches might be more favored in liberal circles, as conservatives tend to emphasize abstinence-based approaches.<br><br>5. **Addressing Safer Sex Practices**: This tends to align with a liberal viewpoint, which generally supports comprehensive sexual education and harm reduction strategies.<br><br>6. **Interaction with the Legal System**: This is a practical aspect of treatment for those mandated to treatment and doesn't inherently align with a specific political ideology, though the degree and manner of collaboration with legal systems might vary between more liberal or conservative jurisdictions.<br><br>In summary, while the guidelines are primarily medical and not explicitly political, elements of the approach have more in common	probably liberal

			with liberal viewpoints, especially in their emphasis on holistic, evidence-based, and harm reduction strategies. However, it's crucial to recognize that medical best practices often transcend simple political categorization.	
APA	Position Statement on Denial of Human Rights Abuses	2008	The text you've provided doesn't align strictly with a liberal or conservative viewpoint in the traditional sense of political ideology. Instead, it addresses a broader human rights and ethical issue that transcends specific political ideologies.<br><br>The statement focuses on the impact of denying or covering up human rights abuses, emphasizing that such actions not only compound the initial violation but also negatively affect the mental health of victims and their families. This perspective is generally aligned with universal human rights principles, which are foundational to various international agreements and declarations, rather than being specific to a particular political ideology.<br><br>Both liberal and conservative viewpoints can, in principle, agree on the importance of acknowledging and addressing human rights abuses, although they may differ in their approaches to these issues. Liberals might emphasize the role of international institutions and norms in addressing these abuses, while conservatives might focus on national sovereignty and the responsibilities of individual states. However, the core idea of acknowledging and addressing human rights abuses can find resonance across the political spectrum.	neutral
APA	Adoption of the American Medical Association (AMA) Statement on Capital Punishment (Policy E-2.06)	7/2008	The text you've provided primarily presents an ethical perspective on physician participation in capital punishment, particularly concerning lethal injection executions. It doesn't align strictly with a liberal or conservative viewpoint as it is more centered on the ethical and professional considerations for physicians in the context of capital punishment.<br><br>However, the stance against physician participation in executions could be interpreted in different ways by liberal and conservative viewpoints:<br><br>1. **Liberal Viewpoint**: Liberals generally advocate for human rights and ethical standards in all professions, including medicine. The text's emphasis on preserving life, ethical boundaries for physicians, and the detailed delineation of what constitutes participation in an execution might resonate with liberal values. Liberals typically oppose capital punishment, so the stance against physician involvement in executions would align with this view.<br><br>2. **Conservative Viewpoint**: Conservatives might have varied opinions on this issue. While some conservatives strongly support capital punishment as a criminal justice tool, others might agree with the ethical stance of not involving physicians, who are traditionally seen as healers, in the process of execution. However, conservatives focusing on law and order might view the involvement of physicians as a means to ensure the lawful and humane carrying out of capital punishment.	neutral

			In summary, the text primarily focuses on the ethical implications for physicians in the context of capital punishment, which might be interpreted differently by individuals with liberal or conservative viewpoints, depending on their stance on capital punishment and the role of medical professionals in such procedures.	
APA	Resolution Condemning the Role of Psychiatrist Radovan Karadzic in Human Rights Abuses in the Former Yugoslavia	2007	The text from the American Psychiatric Association condemning Dr. Karadzic for his actions as the Bosnian Serb leader doesn't align strictly with either a liberal or conservative viewpoint in the traditional political sense. Instead, it focuses on ethical and humanitarian concerns.<br><br>The statement emphasizes the inhumanity of Dr. Karadzic's actions, the betrayal of the values of medicine and psychiatry, and the importance of defending human rights. This focus on human rights, ethical practice in medicine, and the condemnation of inhumane actions transcends typical political categorizations. It is more aligned with universal principles of human rights and professional ethics in healthcare, which are embraced by individuals across the political spectrum.	neutral
APA	A Call for Action From Medicine, Nursing, and Public Health	2007	The text you provided leans more towards a liberal viewpoint. It emphasizes the need for health professionals to engage in violence prevention, collaboration with various sectors (education, law, social work, etc.), and a focus on community and family health. Key liberal themes include:<br><br>1. **Emphasis on Prevention and Community Health**: The text's focus on prevention, rather than punitive measures, aligns with liberal views that often prioritize social and community-based solutions over strict law enforcement or punitive approaches.<br><br>2. **Collaborative and Multi-sector Approach**: The call for collaboration among various professionals and community members is a hallmark of liberal approaches, which often emphasize collective action and community involvement.<br><br>3. **Health and Mental Health Care Accessibility**: The advocacy for increased access to health and mental health care services aligns with liberal policies that emphasize healthcare accessibility and mental health as critical components of public welfare.<br><br>4. **Gun Control**: The point about reducing access to guns for children and youth reflects a liberal stance on gun control, advocating for stricter regulations as a means to prevent violence.<br><br>5. **Reduced Exposure to Media Violence**: This point, along with the overall focus on societal and environmental factors in violence, is consistent with liberal perspectives on the influence of societal structures on individual behavior.<br><br>6. **Support for Research, Policy, and Legislation**: The call for national support and advocacy through research, public policy, and legislation is typical of liberal approaches, which often rely on government intervention and policy changes to address social issues.<br><br>Overall, the text's focus on prevention, community health, collaboration, and policy intervention aligns more with liberal viewpoints, which generally emphasize social welfare, community solutions, and government involvement in addressing societal issues.	liberal

APA	Position Statement on Treatment of Substance Use Disorders in the Criminal Justice System	7/2007	The text you provided outlines a perspective on handling substance use disorders and mental health issues in the criminal justice system, with a focus on treatment rather than punitive measures. This viewpoint aligns more closely with liberal ideologies, which typically emphasize rehabilitation and comprehensive healthcare, including mental health and addiction treatment.<br><br>Liberal perspectives often advocate for the integration of health services, including mental health and substance abuse treatment, into the criminal justice system. They tend to support policies that view addiction and mental health issues as healthcare problems rather than purely criminal matters. This approach includes providing treatment for substance use disorders in jails and prisons, ensuring continuity of care, and using diversion courts like mental health and drug courts to address underlying issues rather than imposing traditional criminal penalties.<br><br>Conservative viewpoints, on the other hand, traditionally emphasize more punitive approaches to criminal justice and may place a greater focus on law enforcement and incarceration. However, it's important to note that perspectives on criminal justice reform are diverse and can vary widely within both conservative and liberal circles. In recent years, there has been a growing recognition across the political spectrum of the need for criminal justice reform, including addressing the treatment needs of individuals with substance use disorders.	liberal
APA	Position Statement on Right to Privacy	2007	The statement you provided reflects a liberal viewpoint. It emphasizes individual rights and freedoms in matters of personal choice, particularly regarding birth control, reproductive choice, and adult consensual sexual relations. These topics are often associated with liberal ideologies, which typically advocate for personal autonomy, especially in areas related to sexuality and reproductive health. Liberal perspectives generally support legislative and judicial actions that protect these personal freedoms.<br><br>Conservative viewpoints, on the other hand, might place more emphasis on traditional values, potentially favoring more regulation or restrictions in these areas. They often prioritize community standards and moral values, which can lead to different stances on issues like birth control and reproductive rights.<br><br>It's important to note that these are generalizations and that individuals' views can vary widely within these broad categories.	liberal
APA	Position Statement on Release of Patients' Records to State Medical Boards	12/2007	The text you've provided outlines principles endorsed by the American Psychiatric Association (APA) regarding the handling of psychiatric and medical records in medical licensure board investigations. These principles primarily focus on patient privacy, the integrity of the investigation process, and the appropriate use of medical records.<br><br>This text does not straightforwardly align with a traditional liberal or conservative viewpoint as understood in the political context. Rather, it addresses professional ethics and legal standards in the medical and psychiatric fields. The emphasis is on balancing the rights of patients (privacy and consent) with the need for regulatory oversight and investigation in cases of potential medical misconduct.<br><br>Both liberal and conservative viewpoints might find aspects of these principles agreeable. Liberals might appreciate the strong emphasis on patient privacy and rights, ensuring that patients have a say in how their medical records are used. Conservatives might value the principles for their potential to uphold professional standards and accountability in the medical profession.<br><br>Ultimately, these principles reflect a focus on ethical medical practice, patient rights, and regulatory responsibilities, transcending simple political categorization.	neutral
APA	A Commitment from Health Professional	2007	The text you provided does not align strictly with either a liberal or conservative viewpoint in the traditional sense of American political ideology. Instead, it focuses on public health and violence prevention, emphasizing community involvement, education, and advocacy. These principles could be embraced by individuals across a wide political spectrum, as they relate more to health and social welfare than to specific partisan policies.<br><br>However, certain aspects of the statement, such as the emphasis on community-based approaches, the mention of firearms in the context of violence, and advocacy for increased public resources, may resonate more with liberal viewpoints, which often prioritize community-oriented solutions and public health initiatives. Conservative viewpoints might focus more on individual responsibility, private sector solutions, or less government intervention in these areas.<br><br>It's important to note that public health and violence prevention are complex issues that can be approached from multiple ideological perspectives, and this text reflects a multi-faceted approach that does not neatly fit into a single political category.	neutral

APA	Position Statement on Patient Access to Treatments Prescribed by Their Physicians	7/2007	The text you provided outlines the American Psychiatric Association's (APA) positions on various topics related to the use of FDA-approved drugs and medical devices, including their use for unlabeled indications, the reimbursement of such uses by third-party payers, and the dissemination of information about these uses.<br><br>The content of this text doesn't align straightforwardly with a typical liberal or conservative viewpoint as understood in a political sense. Instead, it reflects a professional and clinical perspective on medical practice and policy. The focus is on evidence-based medicine, the autonomy of medical practitioners, patient care, and the role of regulatory bodies and pharmaceutical companies in supporting these goals.<br><br>The APA's stance emphasizes the importance of scientific evidence and medical judgment in making clinical decisions, which is a principle that transcends political ideologies. Additionally, the support for coverage of off-label drug uses by insurance providers, including Medicare, is more related to healthcare policy and practice than to a specific political ideology.<br><br>It's important to note that healthcare policies and practices can be influenced by a variety of factors, including but not limited to political beliefs. Therefore, while different political groups might have varying views on specific aspects of these policies (like the role of government in healthcare, regulation of pharmaceuticals, etc.), the text itself is primarily professional and clinical in nature, rather than political.	neutral
APA	Position Statement on Minimum Necessary Guidelines for Third-Party Payers for Psychiatric Treatment	2007	The text you provided is a detailed and technical document focused on the "minimum necessary" guidelines for third-party payers in psychiatric treatment. It outlines the American Psychiatric Association's (APA) position on what information should be disclosed to insurance companies and other third-party payers when processing claims for psychiatric services. The document is structured to respect patient privacy while ensuring that necessary information for claim processing is provided.<br><br>This text does not align explicitly with either a liberal or conservative viewpoint in the traditional political sense. Instead, it addresses healthcare privacy and administrative practices within the context of psychiatric treatment. It reflects concerns about patient confidentiality, the necessity of disclosing certain information for insurance purposes, and the professional standards and judgments of healthcare providers.<br><br>The document's focus is more on the intersection of healthcare policy, patient rights, and medical ethics rather than on a specific political ideology. Its primary concern is balancing the need for privacy in mental health treatment with the requirements of insurance providers and regulatory bodies, a topic that can be of interest to parties across the political spectrum.	neutral
APA	Position Statement on Use of the Concept of Recovery	7/2005	The text you provided is a statement from the American Psychiatric Association regarding their endorsement of the concept of recovery in the treatment of mental illness. This statement does not align explicitly with either a liberal or conservative viewpoint in the traditional political sense. Instead, it focuses on medical and therapeutic approaches to mental health care.<br><br>The principles outlined in the statement, such as emphasizing patient autonomy, dignity, integration into community life, and the partnership between psychiatrist and patient, are broadly consistent with a person-centered approach to healthcare. Such an approach is generally supported across various political and ideological spectrums, although the emphasis on certain aspects might resonate differently with different groups.<br><br>For example:<br>- **Liberal Viewpoints**: Might particularly appreciate the emphasis on patient autonomy, dignity, and integration into the community. Liberals often advocate for person-centered care and the empowerment of individuals in their healthcare decisions.<br>- **Conservative Viewpoints**: Might support the focus on personal responsibility and resilience, as well as the idea of patients actively participating in their care. Conservatives often emphasize the importance of individual initiative and self-reliance in various aspects of life, including healthcare.<br><br>However, it's important to note that healthcare and medical treatment philosophies can transcend traditional political categorizations. The concept of recovery in mental health, as described here, is more a reflection of contemporary psychiatric and psychological practice rather than a statement of political ideology.	neutral

APA	Position Statement on Pharmacy Benefit Managers (PBM)	2002	The text provided appears to focus on specific policy positions related to the field of psychiatry and pharmaceutical management, particularly as advocated by the American Psychiatric Association (APA). These positions include:<br><br>1. Opposition to therapeutic substitution without the approval of the treating physician.<br>2. Opposition to fail-first policies in medication prescription.<br>3. Advocacy for an "easy pass" system for specialists prescribing medications within their field.<br>4. Advocacy for the inclusion of psychiatrists on formulary review boards, especially those reviewing psychotropic agents.<br>5. Requirement for full disclosure of any conflict of interest by members of formulary review boards.<br><br>These positions do not neatly align with traditional liberal or conservative viewpoints. Instead, they are more directly related to healthcare policy, particularly in the realms of mental health and pharmacy management. The APA's stances seem primarily focused on ensuring patient safety, physician autonomy, and transparency in the medication prescribing process, rather than adhering to a specific political ideology.<br><br>Liberal and conservative viewpoints in politics often deal with broader issues like economic policy, social issues, and government's role in individual lives. Healthcare policies can sometimes intersect with these ideologies, particularly on issues like healthcare funding and access. However, the specific points mentioned here are more technical and professional in nature, focusing on best practices in psychiatric treatment and pharmaceutical management rather than broader ideological principles.	neutral
APA	Position Statement on Medicine's Social Contract with Humanity	6/2002	The text you provided does not align strictly with either a liberal or conservative viewpoint as traditionally defined in political terms. It focuses primarily on ethical principles and responsibilities in the medical and healthcare field, emphasizing the universal challenges faced by humanity, the importance of caring for the sick and suffering, and the commitment to medical ethics and human dignity.<br><br>The themes in this text are more humanitarian and professional in nature rather than politically partisan. It stresses the importance of global cooperation, respect for human life, competence in medical treatment, and advocacy for social changes that improve human well-being. These principles are generally upheld across a wide spectrum of political ideologies.<br><br>It's important to note that while certain aspects of the text, like advocating for social changes or emphasizing global cooperation, might resonate more with certain political ideologies, the overall message is one of professional ethics and humanitarian concern, which transcends specific political alignments.	neutral
APA	Vision, Mission, Values, and Goals of the American Psychiatric Association	12/2001	The text you've provided outlines the mission, values, and goals of the American Psychiatric Association (APA). This text does not explicitly align with either a liberal or conservative political viewpoint. Instead, it focuses on professional and ethical standards in the field of psychiatry. The key points emphasized in the text include:<br><br>- Compassionate and patient-focused care.<br>- Evidence-based treatment principles.<br>- Advocacy for patients.<br>- Commitment to lifelong learning and professional development.<br>- Respect for diverse views and collaboration with other health professionals.<br>- Goals related to improving access to quality psychiatric services, research, education, and fostering collaboration in mental health fields.<br>- Promoting high clinical and ethical standards in psychiatry.<br><br>These points are generally aligned with professional medical and ethical standards rather than specific political ideologies. The APA, as a professional organization, aims to promote the best interests of patients and the field of psychiatry, which can involve a range of activities that do not neatly fit into a single political perspective.	neutral

APA	Position Statement on Identification of Abuse and Misuse of Psychiatry	12/1998	The text you provided is a position statement by the American Psychiatric Association (APA) from 1994, focusing on the ethical use of psychiatric knowledge and practice. It emphasizes the use of psychiatric skills and knowledge primarily for patient benefit, upholding confidentiality, and resisting misuse of psychiatry for non-medical objectives such as political or organizational gains.<br><br>This statement doesn't clearly align with a traditional liberal or conservative viewpoint as understood in political terms. Instead, it aligns with professional and ethical standards in psychiatry. The principles outlined are focused on medical ethics, patient care, and the responsibility of psychiatrists to resist external pressures that may compromise these ethics.<br><br>Liberal and conservative viewpoints in politics often differ in their approach to healthcare, government intervention, and individual rights, but the APA's statement is more about professional conduct within psychiatry rather than a commentary on broader political ideologies. It reflects universal principles in medical ethics, which transcend specific political affiliations.	neutral
APA	Position Statement on Codification of Medical Evaluation and Management Services in Psychotherapy	12/1997	The text you provided is primarily a professional or technical description of the roles and capabilities of psychiatrists in the context of healthcare policy. It focuses on the unique aspects of psychiatric practice, especially the blend of psychotherapy and medical evaluation and management that psychiatrists provide.<br><br>This text does not inherently align with a specific political viewpoint, whether liberal or conservative. It's more centered around medical practice, healthcare policy, and the recognition of the comprehensive care provided by psychiatrists. The discussion about the value of psychiatric services and how they are classified and recognized within healthcare systems could be related to broader healthcare policy debates, which can have political dimensions. However, the text itself is neutral and focuses on the specifics of psychiatric practice rather than advocating for a particular political stance.	neutral
APA	Position Statement on TV Violence	12/1993	The text you've provided seems to align with a viewpoint that emphasizes public health and safety, particularly concerning children and adolescents. This viewpoint advocates for regulatory action to decrease television violence, citing its potential harm to young people and families.<br><br>In terms of political alignment, it might be interpreted in various ways:<br><br>1. **Liberal Viewpoint**: The focus on government regulation to protect public health and the well-being of children could be seen as aligning with liberal values. Liberals often support the idea of government intervention to address social issues and protect vulnerable populations.<br><br>2. **Conservative Viewpoint**: However, some aspects of this viewpoint might also resonate with certain conservative principles. Conservatives often prioritize family values and the protection of children. The concern about the impact of TV violence on family stability could align with this.<br><br>3. **Bipartisan/Non-partisan**: It's also possible to interpret this stance as non-partisan or bipartisan. The concern for children's health and well-being transcends political boundaries, and both liberals and conservatives might find common ground in advocating for measures to protect young viewers from harmful content.<br><br>Ultimately, the interpretation can depend on the broader context in which these statements are made and how they align with other policies and principles advocated by specific political groups.	neutral
ACOG	Paid Parental Leave	12/2023	The text aligns more closely with a liberal viewpoint. It emphasizes the importance of paid parental leave and its positive impact on health, economic benefits, and workplace equality. The detailed policy recommendations advocate for comprehensive support for new parents, including maintaining full benefits and salary, promoting equitable access to parental leave, and improving child and parent health outcomes. These points generally resonate with liberal values that prioritize social welfare, equity, and supportive labor policies. Conservatives might support some aspects of family leave but are typically more reserved about government-mandated policies and extensive workplace regulations, favoring market-based solutions and limited intervention. This text, with its emphasis on structured guidelines and benefits for all workers, leans towards a liberal perspective on social support and labor rights.	

ACOG	Midwifery Education and Certification	7/2023	The text primarily focuses on promoting access to safe and high-quality obstetric and gynecologic healthcare by ensuring that clinicians, specifically midwives, meet certain educational and professional standards. It does not explicitly align with a traditional liberal or conservative viewpoint. Instead, it emphasizes the importance of certification and standards in healthcare, particularly in midwifery, and supports choice in healthcare providers as long as they meet these established standards.<br><br>From a broader perspective:<br>- **Liberal Viewpoint**: Liberals generally support comprehensive healthcare access and might favor the emphasis on ensuring high-quality and safe services, the recognition of international standards, and the support for patient choice in healthcare providers.<br>- **Conservative Viewpoint**: Conservatives might appreciate the focus on professional standards and regulations to ensure quality healthcare. They might also support the notion of upholding high standards for healthcare professionals.<br><br>However, both viewpoints could find aspects of the statement to agree with, as it focuses on improving healthcare quality and safety, which are generally bipartisan issues. The text does not delve into more contentious areas like healthcare financing or broader policy debates, which are typically where more distinct liberal or conservative positions emerge.	
ACOG	Violence and Racism in the Criminal Legal System: A Women's Health Crisis	7/2023	ChatGPT<br>The text provided aligns more closely with a liberal viewpoint. It emphasizes opposition to racism and systemic inequity, particularly in healthcare and law enforcement, and advocates for comprehensive reforms. These include decarceration, community safety investments focused on marginalized populations, and anti-racism education. The focus on systemic change, equity, and social justice are themes commonly associated with liberal ideologies, which tend to support proactive government intervention to address societal inequalities and promote civil rights.	
ACOG	Virginity Testing	7/2023	The text reflects a viewpoint that primarily aligns with liberal ideologies. It emphasizes the health and human rights of adolescents and women, a common focus within liberal perspectives which often prioritize individual rights and ethical concerns in healthcare. The American College of Obstetricians and Gynecologists (ACOG)'s stance against "virginity testing" is based on the lack of medical validity and the ethical, psychological, and physical harm it can cause. This stance aligns with broader liberal values of individual autonomy, the protection of human rights, and evidence-based medicine. Conservative viewpoints might vary on this issue, with some emphasizing traditional or cultural practices, but the text itself leans more towards a liberal viewpoint, advocating for the elimination of a practice considered harmful and unethical.	
ACOG	Gun Violence and Safety	2/2023	The text provided presents arguments and policy positions that are generally associated with a liberal viewpoint. Here are some aspects that align it with liberal perspectives:<br><br>1. **Focus on Gun Violence Impact**: The emphasis on the negative impacts of gun violence on health and safety is a concern commonly highlighted in liberal discourse. Liberals often advocate for stricter gun control measures as a way to address public health and safety issues.<br><br>2. **Advocacy for Research and Education**: The call for more research and education on gun violence, as well as opposition to legislative interference in scientific research, aligns with a liberal emphasis on evidence-based policy and the importance of science in informing public health initiatives.<br><br>3. **Support for Specific Gun Control Measures**: The support for laws or regulations limiting firearm access to individuals with a history of violence, particularly intimate partner violence, is a stance more commonly supported by liberals. Conservatives tend to resist firearm restrictions and emphasize Second Amendment rights.<br><br>4. **Mental Health Care Access**: The call for improved access to mental health care services is a typical liberal stance, reflecting a broader liberal agenda on healthcare accessibility and the role of mental health in public safety.<br><br>5. **Protection of Doctor-Patient Relationship**: Opposing government restrictions on conversations between doctors and patients, particularly regarding gun safety, aligns with a liberal viewpoint that emphasizes personal privacy and the importance of professional medical advice.<br><br>6. **Focus on Disproportionate Effects on Marginalized Communities**: Highlighting how gun violence disproportionately affects communities of color and other marginalized groups is a perspective often associated with liberal policies, which tend to emphasize social justice and the need to address systemic inequalities.	

			It's important to note that while these positions align more closely with a liberal viewpoint, political beliefs are complex, and individuals might hold a mix of liberal and conservative views on different issues. Additionally, the interpretation of these positions can vary based on individual perspectives and the political context.	
ACOG	Joint Statement of Practice Relations Between Obstetrician–Gynecologists and Certified Nurse-Midwives/Certified Midwives	11/2022	The text you've provided is a joint statement from the American College of Obstetricians and Gynecologists (ACOG) and the American College of Nurse-Midwives (ACNM). It primarily focuses on promoting evidence-based models of health care, advocating for team-based care, enhancing the quality of care through professional relationships, addressing shortages of healthcare professionals, and advocating for policies that ensure access to care for all. It also emphasizes the importance of education, certification, and collaboration among healthcare professionals to optimize patient outcomes and support health equity.<br><br>This statement does not align strictly with a liberal or conservative viewpoint; rather, it focuses on medical professionalism, evidence-based practice, and improving healthcare outcomes. Both liberal and conservative individuals or groups might find aspects of the statement they agree with. For instance:<br><br>- **Liberal viewpoints** might resonate with the emphasis on health equity, addressing racism and oppression in healthcare, and ensuring broad access to care for all individuals, which align with values of inclusiveness and social justice.<br>- **Conservative viewpoints** might appreciate the focus on professional standards, licensure, and the promotion of a collaborative, team-based approach to healthcare, which align with values of personal responsibility, professionalism, and quality of care.<br><br>Overall, the statement aims to present a balanced, non-partisan perspective focused on improving healthcare outcomes and ensuring the safety and quality of care provided by obstetricians, gynecologists, and nurse-midwives. It's more about professional standards and healthcare policy than political ideology.	
ACOG	Abortion Policy	5/2022	The text you've provided aligns more closely with a liberal viewpoint. It emphasizes the importance of comprehensive, evidence-based health care, which includes access to abortion services. The liberal perspective typically supports the idea of broad access to a range of healthcare services, including reproductive rights and the protection of patient-clinician relationships free from governmental or external interference. The text's strong opposition to efforts that impede access to abortion, its advocacy for privacy and dignity in healthcare decisions, and its condemnation of stigma and violence against healthcare providers are all stances generally associated with liberal ideologies.<br><br>On the other hand, conservative viewpoints often prioritize more restrictive approaches to abortion, focusing on moral and ethical considerations, and may advocate for increased regulation and limitations on access to abortion services. The text's stance against such restrictions and its advocacy for the integration of abortion into mainstream medical care are not in line with typical conservative positions.	

ACOG	Inclusive Language	2/2022	The text provided seems to align more closely with a liberal viewpoint. Liberal perspectives often emphasize the importance of recognizing and respecting diverse identities, including those related to gender, and advocate for inclusive language and policies. This approach is evident in the emphasis on valuing and respecting individual identities, affirming the identities of all individuals regardless of gender, and the commitment to using inclusive language in medical communications and guidance. The focus on improving health outcomes through respect and inclusivity, as well as the call for research that includes gender-expansive participants, aligns with liberal values that prioritize diversity, equity, and inclusivity. However, it's important to note that viewpoints on such issues can vary widely within both liberal and conservative circles, and not all individuals within a particular political ideology may agree with the stance outlined in the text.	
ACOG	Racism in Obstetrics and Gynecology	2/2022	The text provided primarily aligns with a liberal viewpoint. It emphasizes the social construction of race, acknowledges systemic racism and its impact on health disparities, and calls for systemic changes to address these issues. This approach is generally associated with liberal or progressive ideologies, which tend to focus on social justice, systemic inequalities, and the need for active reform to address historical and ongoing injustices.<br><br>Conservative viewpoints, on the other hand, might focus more on individual responsibility, question the extent of systemic racism, or prioritize different methods for addressing racial disparities. They might also emphasize different aspects of policy and societal change.<br><br>It's important to note, however, that these are broad generalizations and individuals' beliefs can vary widely within these ideological spectrums. Additionally, the focus on empirical evidence and the call for systemic reform to improve health outcomes can resonate across a wide range of political beliefs, highlighting the multifaceted nature of these issues.	
ACOG	Female Genital Mutilation	7/2022	The text provided primarily presents factual information about female genital mutilation (FGM) and doesn't explicitly align with either a liberal or conservative viewpoint. It describes what FGM is, its prevalence, the legal status in the U.S., and positions of various medical and international organizations. The opposition to FGM is widely considered a human rights issue rather than a partisan one. Both liberal and conservative factions generally agree that FGM is a violation of human rights and an extreme form of discrimination against women. Therefore, the text is more aligned with a human rights perspective, focusing on health, legal, and ethical considerations rather than a particular political ideology.	
ACOG	Sexual Misconduct	6/2023	The text you've provided doesn't align specifically with liberal or conservative viewpoints as traditionally defined in politics. Instead, it focuses on professional ethics, specifically within the context of the physician-patient relationship and the medical community. The text outlines the ethical standards and responsibilities expected of physicians, especially concerning sexual misconduct and the importance of maintaining a professional boundary to ensure trust and integrity in healthcare.<br><br>Both liberal and conservative ideologies generally support ethical standards in professional fields, although they might approach regulation and enforcement differently. Liberals might emphasize systemic solutions and broad protections for victims, while conservatives might focus on individual responsibility and stricter punitive measures for violators. However, the core subject of this text — advocating for ethical behavior, condemning sexual misconduct, and promoting a safe and professional working environment in medicine — is a concern that cuts across the political spectrum and is shared by individuals with various political beliefs.	

ACOG	Access to Obstetric and Gynecologic Health Care	7/2022	The text you've provided generally aligns more closely with a liberal viewpoint. Here are the key aspects that suggest this alignment:<br><br>1. **Emphasis on Universal Healthcare**: The text supports universal coverage and expanding health coverage to all Americans, which is a position more commonly associated with liberal politics. It stresses the importance of everyone having access to healthcare without financial, cultural, or legal barriers.<br><br>2. **Non-discrimination**: The statement from the American College of Obstetricians and Gynecologists (ACOG) emphasizes non-discrimination in healthcare based on a wide range of factors, including race, color, national origin, disability, age, religion, marital status, sexual orientation, and gender identity. The inclusive approach to healthcare aligns with liberal values that focus on equality and civil rights.<br><br>3. **Holistic Approach to Health**: The text's emphasis on the long-term physical, intellectual, social, and economic well-being of society and the recognition of healthcare as a basic determinant of future generations' health are perspectives often associated with liberal viewpoints. They tend to favor comprehensive, preventative, and community-oriented healthcare approaches.<br><br>4. **Advocacy for Quality and Accessible Healthcare**: The call for quality healthcare appropriate to every person's needs and the mention of the responsibility of healthcare providers to improve health status not just through patient-physician relationships but also by working within communities and at various government levels reflect a proactive stance on healthcare reform. Such advocacy is typically more aligned with liberal ideologies that support active government involvement in ensuring health equity.<br><br>It's important to note that while these points suggest a liberal leaning, healthcare is a complex issue, and individuals or organizations may hold specific views that don't neatly fit into a liberal or conservative category. Also, political views can be fluid and vary widely within the broad categories of "liberal" and "conservative."	
ACOG	Targeted Marketing of Tobacco and Nicotine Products	7/2022	This text aligns more closely with a liberal viewpoint. Liberal perspectives often emphasize public health, social justice, and corporate responsibility, particularly regarding marginalized groups. The statement from the American College of Obstetricians and Gynecologists focuses on the disproportionate health risks of tobacco use among women and marginalized communities like the LGBTQ+ population. It criticizes corporate practices that target vulnerable populations and demands responsible behavior from those corporations. The emphasis on protecting public health, vulnerable groups, and advocating for corporate responsibility are typical of liberal viewpoints, which prioritize collective welfare and critique practices that may exploit or harm disadvantaged groups.	
ACOG	Vaccines and Nonmedical Exemptions	7/2022	The text provided aligns more with a liberal viewpoint. It strongly supports the use of vaccines as a critical public health tool and emphasizes their safety and efficacy in preventing infectious diseases, including for pregnant individuals. The text cites authoritative health organizations like the Centers for Disease Control and Prevention and the American College of Obstetricians and Gynecologists to support its stance. It also expresses concern over the decline in vaccination rates due to nonmedical exemptions and characterizes vaccine hesitancy as a significant global health threat. The emphasis on community health, reliance on scientific consensus, and opposition to non-medical exemptions are positions generally associated with liberal public health policies. Additionally, the focus on protecting vulnerable populations and advocating for evidence-based medicine over personal, philosophical, or religious exemptions aligns with a more liberal viewpoint on public health and preventive care.	
ACOG	Legislative Interference with Patient Care, Medical Decisions, and the Patient-Physician Relationship	8/2021	The text provided appears to align more closely with a liberal viewpoint. It emphasizes the protection of the patient-physician relationship, opposes governmental interference in personal medical decisions, and supports evidence-based medical care. This stance is typically associated with liberal ideologies, which often prioritize individual rights and freedoms, especially concerning personal health decisions and reproductive rights. The text explicitly opposes laws that restrict medical practices such as abortion and gender-affirming care, which are issues often supported by liberal agendas. It also critiques the imposition of outdated or unsupported medical regulations, advocating instead for policies based on current medical evidence and best practices. This focus on individual rights, evidence-based practice, and opposition to restrictive laws on medical care aligns more with liberal principles than conservative ones, which might emphasize more restrictive approaches to these issues.	
ACOG	AAFP ACOG Joint Statement on Cooperative Practice and Hospital Privileges	7/2021	The text you've provided doesn't align strictly with a liberal or conservative viewpoint. It's more of a professional and procedural perspective on healthcare, focusing on the importance of collaborative, high-quality care in maternity services. It emphasizes the need for cooperation among various healthcare providers, shared standards of care, and ongoing professional development and competence. This approach is central to improving patient outcomes and healthcare services in general, rather than reflecting a specific political ideology. Both liberals and conservatives might support these principles from their respective viewpoints, with liberals possibly focusing on the accessibility and equality aspect of care, and conservatives perhaps emphasizing the professional autonomy and high standards of medical practice.	

ACOG	Certification and Procedural Credentialing	7/2021	The text provided primarily outlines the standards, training, certification, and maintenance processes for obstetrics and gynecology in the United States as governed by various professional and accrediting bodies such as the American Board of Obstetrics and Gynecology (ABOG) and the American College of Obstetricians and Gynecologists (ACOG). It does not inherently align with a liberal or conservative viewpoint. Rather, it focuses on the professional and medical standards necessary for practice in this field, including the importance of ongoing education, certification, and quality assurance in patient care.<br><br>Both liberal and conservative perspectives might value different aspects of this system, such as the emphasis on high standards of care, the importance of specialized training, or the need for continuous learning and assessment. However, political viewpoints might diverge on issues related to healthcare policy, access, and regulation, which are not specifically addressed in this text. Therefore, it's best understood as a neutral, informative description of medical practice standards rather than a statement with political alignment.	
ACOG	Supporting Member Involvement in Ob-GynPAC	7/2021	The text seems to advocate for involvement in legislative and political processes to influence decisions related to women's health, particularly in the field of obstetrics and gynecology. This advocacy is positioned within the context of a professional organization, the American College of Obstetricians and Gynecologists, encouraging its members to engage actively in politics and policy-making.<br><br>While the text itself doesn't explicitly align with a liberal or conservative viewpoint, the focus on women's health and proactive engagement in legislative advocacy could be interpreted differently depending on one's political orientation:<br><br>- **Liberal viewpoint**: Individuals with liberal leanings might view this advocacy as crucial for protecting and advancing women's health rights, especially in the context of reproductive rights and access to healthcare. They might see it as a positive step towards ensuring that medical professionals are directly involved in shaping policies that affect their practice and patients.<br><br>- **Conservative viewpoint**: On the other hand, conservatives might have varied reactions. Some might support the idea of medical professionals being more involved in the legislative process, seeing it as a way to ensure that policies are informed by expert opinions. However, others might be wary if they believe this advocacy supports policies they disagree with, such as those expanding access to abortion or other reproductive services.<br><br>In general, the text promotes active engagement and advocacy in political processes by medical professionals to influence women's health policies. The perception of whether this aligns more with a liberal or conservative viewpoint would largely depend on the individual's broader political beliefs and their stance on women's health issues.	
ACOG	Opposition to Immigration Practices that Are Detrimental to the Well-Being of Women and Children	7/2021	The text provided seems to align more closely with a liberal viewpoint. This is primarily because it emphasizes the protection of human rights, the well-being of immigrants, and opposition to strict immigration practices that harm women and children. These stances are typically associated with liberal ideologies, which often prioritize social justice, individual rights, and comprehensive healthcare access. Additionally, the focus on the specific needs of women and girls in healthcare and the acknowledgment of their unique vulnerabilities align with liberal values concerning equality and support for marginalized groups. Conservative viewpoints, on the other hand, might prioritize stricter immigration controls and may not emphasize the same level of concern for the individual rights of immigrants or the specific health needs of women and girls to the same extent.	
ACOG	The Importance of Preserving Access to Care Through the Federal Title X Program	7/2021	The text provided primarily aligns with a liberal viewpoint. It advocates for comprehensive reproductive and preventive healthcare services for low-income women through Title X of the Public Health Services Act, emphasizing the importance of access to a full range of FDA-approved contraceptive methods, cancer screenings, STI testing, and other essential health services. The passage also expresses opposition to efforts that would undermine the Title X program, restrict provider-patient communication, or limit the availability of medically approved contraceptive methods. These positions generally align with liberal perspectives, which often prioritize broad access to healthcare, reproductive rights, and evidence-based medical practices. The text supports policies that ensure women's health and reproductive autonomy, which are typically more emphasized in liberal ideologies.	
ACOG	Global Women's Health and Rights	7/2021	The text predominantly aligns with a liberal viewpoint. It emphasizes the importance of women's rights, autonomy, and health as fundamental aspects of human rights and societal well-being. The support for comprehensive healthcare access, protection against gender-based violence, and equality in education, work, and decision-making are typically associated with liberal ideologies. These ideologies often prioritize social equality, individual rights, and support for social justice initiatives. Conservatives might support some aspects of these rights but often have different approaches to policy and implementation, focusing more on traditional values and less on government intervention. The text's focus on international resolutions and broad societal responsibilities further aligns it with a liberal perspective, emphasizing global cooperation and comprehensive rights for women.	

ACOG	The Role of the Obstetrician-Gynecologist in Aesthetic Services	7/2021	The text you've provided doesn't align distinctly with a liberal or conservative viewpoint. Instead, it addresses professional and ethical considerations in the medical field, specifically relating to obstetrician-gynecologists providing aesthetic services. It discusses the expanding scope of women's healthcare beyond reproductive services, the potential benefits of offering aesthetic services within a medical context, and the ethical responsibilities of physicians to prioritize patient well-being and informed consent.<br><br>The text is primarily concerned with medical ethics, patient safety, and the broadening scope of health services. These topics are generally neutral and pertain to professional standards rather than political ideologies. While different political viewpoints might have varying opinions on healthcare provision and regulation, the text itself does not advocate for a specific political stance but rather focuses on the responsibilities and ethical considerations of healthcare providers in a changing medical landscape.	
ACOG	Opposition to Criminalization of Individuals During Pregnancy and the Postpartum Period	12/2020	The text presents a viewpoint that aligns more closely with liberal principles. It emphasizes the importance of patient autonomy, bodily autonomy, and opposes criminalizing behaviors during pregnancy, which are typically associated with progressive or liberal stances on healthcare and reproductive rights. The stance against punitive measures for pregnant individuals, especially concerning substance use and refusal of care, reflects a focus on public health, social justice, and individual rights over strict legal enforcement, which are hallmarks of liberal ideology. Furthermore, the text's concern with the disproportionate impact of such laws on marginalized communities and the advocacy for harm reduction and education rather than punishment are consistent with liberal values emphasizing equality, social support, and comprehensive healthcare access.	
ACOG	Practice Considerations for Rural and Low-Volume Obstetric Settings	7/2023	The text you've provided outlines strategies for maintaining quality obstetric care in rural and low-volume settings. It doesn't align explicitly with a liberal or conservative viewpoint; rather, it focuses on practical, medical, and organizational strategies to ensure safe and effective health care. The suggestions made, such as maximizing health care provider attendance at deliveries, rotating staff for broader experience, providing structured orientation and training, and integrating with regional health systems, are aimed at improving patient care and safety.<br><br>However, the text could be interpreted differently depending on one's political perspective:<br><br>1. **Liberal Viewpoint:**<br>- Liberals might support the text's emphasis on comprehensive training, integrated health systems, and government and community support for health care providers. They may see these strategies as part of a broader commitment to ensuring equitable access to quality health care, particularly in underserved areas.<br>- The suggestion of shared responsibility among community, government, and payers aligns with liberal views on collective action and public investment in health care.<br><br>2. **Conservative Viewpoint:**<br>- Conservatives might appreciate the focus on practical and localized solutions to health care issues and the emphasis on maximizing current resources.<br>- The ideas about rotating staff to gain experience and leveraging telemedicine could be seen as innovative and efficient ways to improve care without necessarily expanding government programs.<br><br>In summary, while the text itself is neutral and focused on medical best practices, different aspects of the strategies presented could resonate with both liberal and conservative values depending on how one prioritizes aspects of health care policy and rural support.	
ACOG	COVID-19 Public Health Considerations for Pregnant and Postpartum People Who Are Incarcerated	7/25/2023	The text provided discusses the heightened risks and recommendations for pregnant individuals in incarceration settings during the COVID-19 pandemic, with a focus on reducing exposure and improving care. It does not explicitly align with a liberal or conservative viewpoint. Instead, it focuses on health, safety, and ethical considerations, reflecting concerns common to public health and human rights perspectives.<br><br>Liberal viewpoints typically emphasize the protection of individual rights, advocating for reforms that reduce incarceration rates and improve conditions for vulnerable populations, such as pregnant women. The text's emphasis on reducing incarceration, improving healthcare access, and acknowledging systemic inequities aligns with these values.<br><br>Conservative viewpoints often prioritize law and order and may have a less reform-oriented approach to incarceration. However, conservatives also value efficient use of resources and might support measures that improve health outcomes and reduce long-term costs.<br><br>The text’s focus on evidence-based healthcare, reducing COVID-19 risks, and acknowledging racial disparities transcends simple	

			political categorization. It represents a multifaceted approach to a complex issue, incorporating elements that might resonate with a broad spectrum of political beliefs focused on public health, ethical treatment, and systemic reform.	
ACOG	Qualifications for Performing and Interpreting Imaging Procedures in Obstetrics and Gynecology	5/24/2023	The text you've provided outlines standards and guidelines for physicians, particularly obstetrician-gynecologists, regarding the performance, evaluation, and interpretation of gynecologic and obstetric ultrasound examinations. It emphasizes the need for proper training, experience, competence, and an understanding of ultrasound safety, along with patient care responsibilities.<br><br>This text is primarily professional and technical, focusing on medical standards and best practices rather than political ideology. However, if one were to interpret it through a political lens:<br><br>1. **Liberal viewpoint**: The text might align with liberal values in the sense that it advocates for patient access to optimal care and opposes policies that unnecessarily restrict qualified practitioners. Liberals often emphasize the importance of access to healthcare and might support the idea of allowing well-trained and competent physicians to perform and interpret imaging without additional, potentially burdensome certification requirements.<br><br>2. **Conservative viewpoint**: On the other hand, conservatives might appreciate the text's emphasis on ensuring that physicians have proper training and demonstrated competence, reflecting a concern for maintaining high standards and quality in healthcare. The focus on individual certification and responsibility might also resonate with conservative values regarding professional autonomy and accountability.<br><br>Ultimately, the text is more about professional medical standards than political ideology, and different aspects of it might resonate with different ideological perspectives for various reasons. It's important to note that interpretations can vary widely, and what seems liberal to one person might seem conservative to another, depending on their personal views and the specific political context.	
ACOG	ACOG Statement on “Personhood” Measures	11/9/2022	The text aligns more closely with a liberal viewpoint. It emphasizes opposition to "personhood" laws, which are often supported by conservative groups, as they confer legal rights to a fertilized egg, embryo, or fetus, potentially restricting access to abortion and other reproductive health services. The American College of Obstetricians and Gynecologists (ACOG) argues against these measures, advocating for comprehensive reproductive health care, including access to contraception, abortion, and in vitro fertilization, which are positions generally associated with liberal ideologies. ACOG emphasizes the importance of science in public health policies and medical decision-making, advocating for the rights and health of women and people seeking reproductive health care. This stance is typically aligned with liberal values that prioritize individual rights and health care access over more conservative positions that might prioritize fetal rights from conception.	
ACOG	Opposition to the Criminalization of Self-Managed Abortion	7/6/2022	The text you've provided aligns more closely with a liberal viewpoint. It discusses the criminalization and prosecution of individuals for actions related to their pregnancies, including substance use, declining medical procedures, and self-managed abortions. The text cites studies and statistics to highlight the prevalence and reasons behind self-managed abortions, particularly focusing on the barriers to accessing safe, clinic-based care and the impacts of restrictive abortion laws. The American College of Obstetricians and Gynecologists (ACOG)'s stance against the criminalization of pregnant individuals and the opposition to policies mandating reporting of self-managed abortion are indicative of a perspective that values patient autonomy, confidentiality, and the right to access healthcare without fear of legal repercussions.<br><br>This position is typically associated with liberal politics, which often emphasize reproductive rights, access to healthcare, and the protection of patient privacy. The text's focus on the negative impacts of criminalization and the importance of access to safe abortion services aligns with liberal values of individual rights and opposition to government intrusion into personal health decisions.	

ACOG	Addressing Climate Change	11/2021	The text provided aligns more closely with a liberal viewpoint. It emphasizes the urgency and negative impacts of climate change, a stance typically associated with liberal politics. The focus on the disproportionate effects on women's health, the call for national and international action to curb greenhouse gas emissions, and the support for environmentally responsible practices and policies are all consistent with liberal priorities. Additionally, the advocacy for research and mitigation of climate change's health-related harms, especially for vulnerable populations, aligns with liberal concerns for social justice and equity. Conservative viewpoints might be more skeptical of the urgency or extent of climate change's impacts or prioritize economic considerations over environmental regulations.	
ACOG	Addressing Health Equity During the COVID-19 Pandemic	5/11/2020	The text you've provided doesn't align strictly with a single political viewpoint, liberal or conservative, as it addresses public health and ethical considerations, which are of universal concern. However, the focus on addressing health disparities, structural racism, and social determinants of health, as well as advocating for equitable healthcare access and outcomes, tends to resonate more with liberal ideologies. Liberal perspectives often emphasize social justice, equity, and the role of systemic factors in individual health outcomes.<br><br>The emphasis on data collection, collaboration with community leaders, and the acknowledgment of the history of discrimination and exploitation in communities of color also align with a more progressive approach to healthcare. Liberals typically support policies and efforts aimed at reducing inequalities and addressing the needs of marginalized communities.<br><br>Conservatives might support aspects of the text that emphasize efficient resource allocation and personal responsibility in healthcare. However, the conservative viewpoint generally focuses more on individual liberties, market-based solutions, and less government intervention, which is not the central focus of this text.<br><br>Overall, while the text does not strictly belong to a single political ideology, its emphasis on equity, acknowledgment of structural factors, and call for systemic changes align more closely with liberal viewpoints.	
ACOG	Surgical Assistants	4/2023	The text you've provided focuses on the standards for surgical assistance in obstetric and gynecologic operations, emphasizing the importance of having competent surgical assistants, often physicians, available for complex surgeries to ensure patient safety. It also addresses the autonomy of the primary surgeon in determining the necessary number of assistants and their qualifications, as well as the fair compensation for such assistants.<br><br>This statement doesn't align strictly with liberal or conservative viewpoints as it primarily addresses medical best practices and the autonomy of medical professionals rather than a political ideology. Both liberals and conservatives might agree on the importance of high-quality medical care, patient safety, and professional autonomy. However, the aspects concerning compensation and the role of third-party payers might be interpreted differently based on individual political beliefs:<br><br>1. **Liberal Viewpoint**: A liberal perspective might focus on the importance of access to quality healthcare and might support the statement as advocating for necessary resources and personnel to ensure patient safety. They might also emphasize the need for third-party payers (like insurance companies) to support these necessary medical practices without imposing restrictive cost-cutting measures.<br><br>2. **Conservative Viewpoint**: A conservative might agree with the emphasis on professional autonomy and the implication that regulatory bodies or insurers shouldn't unduly influence medical decisions. They might also support the notion of fair compensation for services rendered as part of a market-driven approach to healthcare.<br><br>In summary, while the text doesn't inherently align with one political ideology over the other, aspects of it can be interpreted differently depending on one's liberal or conservative leanings, particularly regarding healthcare regulation, professional autonomy, and the role of insurance companies.	

ACOG	Improving Access to Mifepristone for Reproductive Health Indications	3/2021	The text provided aligns more closely with a liberal viewpoint, particularly in the context of American politics. The key themes supporting this include:<br><br>1. **Advocacy for Access to Women's Health Care:** The statement supports improving access to women’s health care and opposes regulations that impede access to evidence-based care. This is typically associated with liberal politics, which often emphasize the protection and expansion of healthcare access.<br><br>2. **Opposition to Restrictive Measures:** The text criticizes the FDA's Risk Evaluation and Mitigation Strategy (REMS) and Elements to Assure Safe Use (ETASU) for mifepristone as outdated and unnecessarily restrictive. Liberal viewpoints often challenge excessive regulations on healthcare services, arguing they can limit personal freedom and choice, particularly in the context of reproductive rights.<br><br>3. **Support for Evidence-Based Medicine:** The statement emphasizes the importance of evidence-based medicine in guiding healthcare policies. While both liberals and conservatives can support scientific evidence, the context here is about increasing access to reproductive services, which is more commonly a liberal stance.<br><br>4. **Focus on Reproductive Rights:** The text specifically discusses medication-induced abortion and the need to remove barriers to accessing mifepristone for this purpose. The liberal viewpoint in the U.S. typically supports the right to choose and access to abortion services, contrasting with the conservative viewpoint, which more often seeks to restrict access to abortion.<br><br>5. **Opposition to Unique Restrictions for Abortion Services:** The text notes the inconsistency of the regulations on mifepristone compared to other medications and opposes regulations that are uniquely restrictive to abortion services. This is in line with a liberal perspective that argues for reproductive health services, including abortion, to be treated like other medical services without additional barriers.<br><br>While the text aligns more closely with a liberal viewpoint, it's important to recognize that views on healthcare and reproductive rights can vary widely within each political spectrum, and individuals may hold nuanced positions that don't fully align with one side or the other.	
ACOG	Access to Genetic Testing	3/2023	The text you've provided critiques barriers to genetic testing proposed by health care insurers and the Centers for Medicare and Medicaid Services (CMS), particularly the requirements for certified genetic counseling before testing and the implications of preauthorization requirements. The American College of Obstetricians and Gynecologists (ACOG) opposes these barriers as they impose unnecessary delays and restrictions on patient care, especially for women.<br><br>This perspective aligns more closely with a liberal viewpoint for several reasons:<br><br>1. **Emphasis on Access to Healthcare**: The liberal viewpoint often prioritizes the removal of barriers to healthcare access. The text argues against additional requirements and certifications that could hinder timely access to genetic testing, especially for women, which resonates with liberal values of healthcare accessibility and equity.<br><br>2. **Opposition to Over-Regulation**: The text opposes what it sees as over-regulation by health insurers and governmental bodies, arguing that such measures are unnecessary and obstructive. Liberals generally support regulation that protects consumers but may oppose excessive bureaucracy that impedes access to care.<br><br>3. **Support for Professional Autonomy**: The ACOG's stance supports the professional judgment and qualifications of obstetrician–gynecologists. Liberals often advocate for the rights of professionals to operate without undue restriction, trusting their expertise and advocating for policies that support their autonomy in serving patients' best interests.<br><br>However, it's important to note that healthcare policy can cross traditional liberal-conservative lines, and individuals from either side may have various reasons for supporting or opposing specific healthcare policies. Additionally, conservatives may also support aspects of this viewpoint, especially if they emphasize the reduction of government intervention and support for professional autonomy. Thus, while the text leans more towards a liberal viewpoint due to its focus on accessibility and opposition to regulation, it doesn't exclusively represent the ideology of one political spectrum.	

ACOG	Definition of “Procedures” Related to Obstetrics and Gynecology	3/2023	The text provided appears to be focused on healthcare policy and practice, specifically on the definitions and classifications of medical interventions. It doesn't explicitly align with a liberal or conservative viewpoint, as it is more technical and procedural in nature. However, some aspects could be interpreted differently based on ideological leanings:<br><br>1. **Liberal Viewpoint**:<br>- **Team-Based Care**: Liberals might favor the emphasis on team-based care, as it can suggest a more inclusive, accessible healthcare system with a variety of professionals contributing to patient care.<br>- **Non-Physician Providers**: Liberals might support the idea of expanding roles for non-physician providers to increase access to care, reduce costs, and improve efficiency within the healthcare system.<br><br>2. **Conservative Viewpoint**:<br>- **Regulation and Classification**: Conservatives might appreciate the focus on clearly defining medical procedures and surgeries, as it could be seen as a way to maintain high standards of patient care and ensure that only qualified professionals perform certain tasks.<br>- **Risk and Safety**: The emphasis on safety and risk might resonate with a conservative focus on individual responsibility and minimizing potential for litigation or unnecessary government intervention in healthcare.<br><br>In essence, while the text itself is neutral and focused on healthcare practices, different aspects of it could be supported or critiqued by either side depending on their broader healthcare policies and beliefs. It's also possible that both liberals and conservatives might find certain parts agreeable or concerning, depending on their perspectives on healthcare regulation, professional roles, and patient safety.	
ACOG	Restrictions to Comprehensive Reproductive Health Care	4/2023	The text presents a viewpoint more closely aligned with liberal ideologies. It emphasizes the importance of comprehensive reproductive health care, women's autonomy and rights, and the potential dangers of restrictive policies based on non-scientific grounds. The focus on ensuring access to a full range of reproductive services, including contraception and pregnancy termination, and advocating against policies that limit such access, are positions more commonly associated with liberal perspectives. The text also criticizes hospital mergers and policies that restrict reproductive health services, which aligns with a liberal concern for maintaining broad access to healthcare and upholding individual rights over institutional or religious restrictions.	
ACOG	Decriminalization of Self-Induced Abortion	12/2017	The text presents a viewpoint that aligns more closely with liberal perspectives. It discusses the criminalization and prosecution of women for actions during pregnancy, including self-induced abortions, and highlights the opposition of the American College of Obstetricians and Gynecologists (ACOG) to these practices. The focus on women's reproductive rights, the criticism of legal restrictions on abortion, and the emphasis on patient autonomy and confidentiality are positions typically associated with liberal viewpoints. The text argues against criminalizing women's health choices and advocates for access to care, reflecting a stance that prioritizes individual rights and healthcare access, which are key issues in liberal ideologies.	

ACOG	Counseling Patients With Zika Infection	12/2016	The text you've provided outlines guidelines and considerations for obstetric care providers in counseling pregnant women exposed to or infected with Zika virus, focusing on the need for comprehensive counseling and access to a full range of reproductive options, including termination. It emphasizes individualized counseling, informed consent, and non-imposition of personal beliefs by healthcare providers.<br><br>This text might align with different political viewpoints in various ways:<br><br>**Liberal viewpoint:**<br>- **Pro-choice stance**: The emphasis on full access to reproductive options, including termination of pregnancy, aligns with liberal and pro-choice viewpoints, which advocate for women's rights to make decisions about their own bodies and pregnancies.<br>- **Scientific approach**: The focus on evolving scientific understanding and the need for individualized, informed counseling may resonate with liberal ideologies, which often prioritize science and personal autonomy in healthcare.<br><br>**Conservative viewpoint:**<br>- **Focus on informed consent and individualized care**: Conservatives might appreciate the emphasis on informed consent and the provision of comprehensive information to the patient, as it respects individual decision-making and personal responsibility.<br>- **Non-imposition of beliefs**: The directive for healthcare providers not to impose their personal beliefs could be seen as a nod to professional ethics and respect for diverse opinions, which some conservatives might support.<br><br>However, the text's explicit support for access to abortion as part of comprehensive care is more commonly associated with liberal viewpoints, especially in the context of the current political climate in many places where reproductive rights are a divisive issue. It's essential to note that individuals' beliefs can't always be neatly categorized, and people may hold a mix of liberal and conservative views on different aspects of this issue.	
ACP	Protective Head Gear for Bicycle and Moped Riders	2016	The statement "ACP recommends bicycle helmets for all moped and bicycle riders and encourages state legislatures to pass laws requiring bicycle and moped riders to wear protective head gear (helmets) on all county, state, and national highways" does not neatly align with a traditional liberal or conservative viewpoint as understood in the context of American politics. It is primarily a public health and safety recommendation.<br><br>- **Liberal Viewpoint:** Traditionally, liberals in the U.S. tend to support government intervention in public health and safety issues. From this perspective, advocating for helmet laws could be seen as an effort to protect individuals and reduce healthcare costs resulting from accidents.<br><br>- **Conservative Viewpoint:** Conservatives often emphasize personal freedom and limited government intervention. In this light, mandating helmet use could be viewed as an overreach of government authority, imposing on individual freedoms.<br><br>However, issues like helmet laws often transcend conventional political ideologies. They can be seen as pragmatic, focusing on public health and safety rather than strictly political principles. The American College of Physicians (ACP), as a medical organization, would likely base its recommendations on medical and safety data rather than political ideologies.	probably liberal
ACP	Drunk Driving	2016	The statement from the American College of Physicians (ACP) regarding alcoholism and driving under the influence (DUI) doesn't strictly align with either a liberal or conservative viewpoint in the traditional sense. Instead, it reflects a blend of perspectives that emphasize public health, responsibility, and law enforcement.<br><br>1. **Public Health Focus**: The call to recognize alcoholism as a disease and to require evaluation and treatment for DUI offenders reflects a public health approach. This perspective is often associated with more liberal viewpoints, which tend to emphasize treatment and rehabilitation over punishment.<br><br>2. **Personal Responsibility**: The statement that having the disease of alcoholism should not exempt DUI offenders from being responsible for their actions aligns with a more conservative viewpoint, which emphasizes personal accountability and responsibility for one's actions.<br><br>3. **Law Enforcement and Public Safety**: Supporting stringent enforcement of laws against drunk driving and the enactment of more effective laws could be seen as aligning with both liberal and conservative viewpoints. Conservatives often support strong law enforcement measures, while liberals may support such measures in the context of public safety and health.	neutral

			In summary, the ACP's position is multidimensional, addressing the issue of DUI from both a public health and a law enforcement perspective. It underscores the importance of addressing the underlying causes of DUI (like alcoholism) while still holding individuals accountable for their actions, a stance that transcends simple political categorization.	
ACP	Provision of Clean Needles/Syringes to Drug Addicts	2016	The statement about needle and syringe exchange programs being warranted as a means of AIDS control aligns more closely with a liberal viewpoint. Generally, liberal perspectives prioritize public health strategies that include harm reduction approaches. Needle exchange programs are typically seen as part of harm reduction strategies, aiming to reduce the spread of infectious diseases like HIV/AIDS among drug users.<br><br>Conservative viewpoints, on the other hand, might be less supportive of such programs, often emphasizing abstinence, law enforcement, and drug prevention approaches over harm reduction strategies. There can be concerns among some conservatives about needle exchange programs enabling or condoning drug use.<br><br>However, it's important to note that these are general trends and there can be a range of opinions within both liberal and conservative circles. Public health issues like AIDS control sometimes transcend traditional political lines, with focus on pragmatic solutions over ideological positions.	liberal
ACP	Mandatory Testing for All Physicians	2016	The statement "ACP vigorously opposes mandatory HIV testing of all physicians" does not align distinctly with either a liberal or conservative viewpoint in a traditional political sense. Instead, it reflects a stance on a specific healthcare policy issue.<br><br>The American College of Physicians (ACP), as a medical professional organization, bases its positions more on medical ethics, patient care standards, and public health considerations rather than on political ideologies. Their opposition to mandatory HIV testing of all physicians likely stems from considerations such as medical privacy, the effectiveness of such measures in preventing HIV transmission, and the rights of healthcare workers.<br><br>Both liberal and conservative viewpoints might find aspects to support or oppose in this statement, depending on their focus on individual rights, public health concerns, privacy issues, and the role of government in healthcare. Therefore, categorizing this statement strictly as liberal or conservative oversimplifies the complex nature of healthcare policy discussions.	neutral
ACP	Nurse Practitioners in Primary Care	2022	The text provided seems to express a viewpoint that is neither strictly liberal nor conservative but rather focuses on pragmatic and professional considerations in the healthcare field. It emphasizes the distinct but complementary roles of physicians and nurse practitioners, advocating for collaboration and clear communication between these professionals to provide the best possible patient care. This perspective is centered on improving healthcare delivery through teamwork, recognizing the unique contributions of each profession, and ensuring patients are informed and receive care from the most appropriate healthcare provider.<br><br>Such a stance is more reflective of a professional and sector-specific viewpoint, prioritizing effective healthcare delivery and patient outcomes over any particular political ideology.	neutral

ACP	Integration of Care for Mental Health, Substance Abuse, and Other Behavioral Health Concerns into Primary Care	2015	The text you provided outlines positions of the American College of Physicians (ACP) on integrating behavioral health care into primary care. It's important to note that such policy recommendations might not align neatly with traditional liberal or conservative viewpoints as they primarily focus on healthcare practices and policies. However, we can analyze them from a political spectrum perspective:<br><br>1. **Support for Integration of Behavioral Health in Primary Care**: This is a healthcare-centric approach, focusing on holistic patient care. It doesn't align clearly with liberal or conservative ideologies but reflects a more progressive approach to healthcare.<br><br>2. **Removing Payment Barriers for Integrated Care**: Advocating for the removal of financial barriers in healthcare can be seen as a more liberal stance, as it suggests increased regulation or intervention in the healthcare market to promote accessibility.<br><br>3. **Addressing Insurance Coverage Gaps and Strengthening Nondiscrimination Laws**: These recommendations align more with liberal policies, emphasizing healthcare equity and regulation to ensure comprehensive coverage.<br><br>4. **Support for Increased Research**: This position is neutral and focuses on evidence-based practice, a key aspect of modern medical practice, rather than a specific political ideology.<br><br>5. **Encouraging Government Efforts for Adequate Workforce Training**: This could be seen as leaning towards a liberal viewpoint as it involves government intervention in education and training for healthcare.<br><br>6. **Initiatives to Reduce Stigma Associated with Behavioral Health**: This recommendation is more aligned with a liberal perspective, focusing on social issues and the role of government and stakeholders in addressing these issues.<br><br>Overall, while these points focus primarily on healthcare policy and practice, they tend to align more with liberal viewpoints due to their emphasis on government intervention, equity, and comprehensive coverage in healthcare. However, it's crucial to recognize that healthcare policies often transcend simple political binaries and are influenced by a wide range of factors including ethics, economics, and social needs.	probably liberal
ACP	Blood Donations by Donors Over 65 Years of Age	2017	The statement "ACP supports and encourages healthy adults of all ages to be active blood donors" does not align strongly with either a liberal or conservative viewpoint. It reflects a general health and welfare perspective, advocating for blood donation, which is a universal healthcare need and does not typically fall along political lines. Both liberal and conservative viewpoints tend to support public health initiatives like blood donation, as it is a non-partisan issue that benefits the broader community. This statement is more aligned with a public health perspective rather than a political ideology.	neutral
ACP	Amended Recommendation on Appropriate Patient Age for Internal Medicine	2010	The text you've provided doesn't directly align with a typical liberal or conservative viewpoint. It's a statement about medical practice, specifically regarding the age at which internists might choose to accept patients based on their training, experience, and comfort with adolescent and/or pediatric medicine. The focus is on professional standards and individual physician discretion rather than on a political ideology.<br><br>In general, liberal and conservative viewpoints often differ on issues like government intervention in healthcare, reproductive rights, and public health policies, but the specifics of medical practice, such as the age at which a doctor chooses to see patients, are usually not directly tied to these political ideologies. This text seems more related to professional guidelines and individual practitioner preferences within the medical field.	neutral
ACP	Relation of the Physician to Government	2018	The text you provided is a statement on medical ethics, specifically addressing the role of physicians in relation to human rights, torture, executions, interrogations, and healthcare in correctional systems. It doesn't align strictly with a liberal or conservative viewpoint in the typical political sense. Instead, it reflects ethical principles that are generally accepted within the medical profession.<br><br>These principles emphasize the importance of human rights, the duty of physicians to speak out against torture and abuse, and the ethical boundaries in their involvement in certain government or institutional actions. Such stances are rooted in the broader humanitarian and ethical guidelines that govern medical practice, rather than being specifically tied to a particular political ideology.<br><br>While different political groups might have varying stances on issues like the death penalty or prison healthcare, the text itself is focused on the ethical obligations of physicians rather than advocating for a specific political position. It's more about establishing a standard of conduct for medical professionals in situations that involve human rights and ethical dilemmas.	neutral

ACP	Medicine and the Law	2018	The text you provided discusses the relationship between ethical standards in medicine and legal obligations, focusing on how physicians should navigate conflicts between these two realms. It does not explicitly align with a liberal or conservative viewpoint as it is primarily concerned with professional ethics and legal responsibilities in the medical field.<br><br>However, it does touch on themes that both liberal and conservative viewpoints might interpret differently:<br><br>1. **Liberal Viewpoint**: Liberals might focus on the emphasis on equal treatment and legal rights, regardless of illness. This aligns with liberal values of equality, individual rights, and justice. The acknowledgment of ethical considerations sometimes being at odds with the law might also resonate with liberal perspectives that advocate for progressive changes in legal and social systems.<br><br>2. **Conservative Viewpoint**: Conservatives might focus on the respect for the law and the caution advised against violating legal boundaries. This respect for established legal frameworks and the importance of consulting with legal counsel or colleagues before taking action could align with conservative values that emphasize law and order, and respect for traditional structures.<br><br>Overall, the text presents a nuanced view that highlights the complexity of decision-making in the medical field, where legal, ethical, and professional considerations often intersect. It advocates for careful consideration and consultation in cases of conflict, a stance that can be appreciated across a broad spectrum of political ideologies.	neutral
ACP	Health Professionals and the Health Effects of Economic Sanctions and Embargoes	2022	The text you've provided outlines the position of the American College of Physicians (ACP) regarding economic sanctions and humanitarian aid. It emphasizes the importance of exempting humanitarian goods from sanctions, empowering agencies to address humanitarian needs, providing medical supplies to mitigate the health impacts of sanctions, and monitoring the delivery of medical and health-related materials.<br><br>This position aligns more closely with a liberal viewpoint. Liberal ideologies generally prioritize humanitarian concerns, advocate for the protection of civilian populations during conflicts or sanctions, and emphasize the role of international cooperation and agencies in addressing humanitarian crises. The focus on ensuring the delivery of medical and health-related supplies, even in sanctioned areas, reflects a commitment to human rights and global health, which are key aspects of liberal thought.<br><br>Conservative viewpoints might be more cautious about such exemptions, focusing on the importance of maintaining the full impact of sanctions as a tool of foreign policy. They might argue that exceptions can be exploited or might weaken the intended effect of the sanctions.<br><br>However, it's important to note that humanitarian issues can sometimes transcend traditional political divides, with both liberals and conservatives recognizing the need for humanitarian aid in certain circumstances. The ACP's stance is primarily focused on medical ethics and the health and well-being of civilians, which can be a point of bipartisan agreement.	liberal
ACP	Equal Opportunity	2019	The text you've provided appears to align with a liberal viewpoint. It emphasizes inclusivity and non-discrimination based on gender, gender identity, race, religion, nationality, or sexual orientation. This approach is generally more associated with liberal ideologies, which often prioritize diversity, equality, and anti-discrimination policies. Conservative viewpoints, while they can also support non-discrimination, often place a higher emphasis on tradition and may not prioritize these issues in the same way. It's important to note, however, that political views can be complex and diverse within any ideology.	liberal

ACP	Environmental Health	2022	The text you've provided aligns more closely with a liberal viewpoint. Here's a breakdown of how the statements align with typical liberal values:<br><br>1. **Climate Change and Global Warming**: Acknowledging climate change as a crisis and advocating for immediate action to limit global temperature rise is a position more commonly associated with liberal viewpoints. Conservatives may acknowledge climate change but often differ in the urgency and type of response they support.<br><br>2. **Environmental Justice**: The focus on environmental justice and the specific mention of communities of color, low-income groups, and marginalized populations is a hallmark of liberal policies, which often emphasize social equity and addressing disparities.<br><br>3. **Support for the Clean Air Act and EPA Regulations**: Liberals typically support robust environmental regulations and the empowerment of agencies like the EPA. Conservatives might support some level of regulation but often with greater emphasis on economic impact and less regulatory burden.<br><br>4. **Safe Drinking Water and Clean Water Acts**: Advocacy for strong water quality laws aligns with a liberal approach to environmental policy, emphasizing public health and environmental protection.<br><br>5. **Protection from Toxic Exposures**: The focus on protecting vulnerable populations from toxic substances, including children and pregnant people, is consistent with liberal priorities in public health and environmental policy.<br><br>6. **Funding for Environmental Health Agencies**: Advocating for sustainable funding for federal agencies with an environmental health mission is typically a liberal stance, emphasizing the role of government in safeguarding public health and the environment.<br><br>Overall, the text reflects a liberal perspective, particularly in its emphasis on environmental protection, social equity, and proactive government intervention.	liberal
ACP	Climate Change and Health	2016	The text provided aligns more closely with a liberal viewpoint. Here's why:<br><br>1. **Emphasis on Global Efforts and Leadership in Climate Change**: The call for a global effort to reduce greenhouse emissions and the United States taking a leadership role is a stance typically associated with liberal ideologies. Liberals often advocate for proactive, international collaboration on climate issues.<br><br>2. **Healthcare Sector's Role in Climate Change**: The focus on the healthcare sector implementing environmentally sustainable practices and preparing for climate impacts is consistent with liberal priorities of integrating environmental sustainability into various sectors.<br><br>3. **Physician Advocacy for Climate Policies**: Encouraging physicians to advocate for climate change policies and to communicate its health benefits to the community and policymakers reflects a liberal perspective that values science-based policy-making and public education on environmental issues.<br><br>4. **Education Emphasis**: The emphasis on educating physicians about climate change and incorporating related coursework in medical education is in line with liberal values that stress the importance of education and science in addressing societal challenges.<br><br>5. **Government Commitment to Climate Research Funding**: The call for governments to provide substantial funding for climate change research, adaptation, and mitigation, especially regarding human health, is a stance commonly seen in liberal politics, where there is a greater advocacy for government intervention and funding in addressing environmental and public health issues.<br><br>Overall, the text's focus on proactive, collaborative, and education-driven approaches to addressing climate change, with a significant role for government and healthcare sectors, is more aligned with liberal viewpoints. Conservative viewpoints, while they may also acknowledge the importance of addressing climate change, often differ in the approach, typically emphasizing market-based solutions, less government intervention, and a more cautious approach to international collaboration.	liberal

ACP	Coding for Lab Services	2017	The text you provided appears to be related to healthcare policy, specifically regarding changes to the Current Procedural Terminology (CPT) coding system. These changes involve the restructuring of codes for automated, multichannel tests and the maintenance of organ-oriented laboratory panels with input from experienced consultants.<br><br>Determining whether these positions align with liberal or conservative viewpoints is not straightforward, as they deal with specific medical coding practices rather than broader ideological issues. However, I can provide a general perspective:<br><br>1. **Liberal Viewpoint**: Liberals generally advocate for efficient and accessible healthcare systems. The proposal to replace codes with a smaller, more defined number of panels might be seen as a step towards efficiency and better healthcare management. However, liberals also emphasize the importance of individualized care, so the encouragement to add or delete specific tests based on medical appropriateness could align with this view.<br><br>2. **Conservative Viewpoint**: Conservatives typically emphasize cost-efficiency and the reduction of regulatory burdens in healthcare. The idea of maintaining organ-oriented laboratory panels and reconstructing them with the help of experienced consultants could be seen as an approach to ensure quality and efficiency in healthcare, which may align with conservative values. Conservatives might also support the idea of allowing physicians to tailor tests based on medical appropriateness, as it could be viewed as reducing unnecessary regulatory constraints.<br><br>It's important to note that such policy details can cross ideological lines, and different individuals or groups within each political spectrum might have varying opinions on these specific healthcare policy proposals. The alignment with liberal or conservative viewpoints could depend on the broader context of how these changes impact healthcare access, efficiency, costs, and regulation.	neutral
ACP	Cognitive/Evaluation and Management Services	2019	The text you've provided outlines positions of the American College of Physicians (ACP) regarding Evaluation and Management (E&M) Current Procedural Terminology (CPT) codes and their interpretation, application, and reimbursement in medical practice. It doesn't neatly align with traditional liberal or conservative political viewpoints as understood in a broader political context. Instead, these statements are more focused on healthcare policy and practice, particularly in the context of medical billing and reimbursement.<br><br>1. **Clarification of CPT Codes**: This point is about making CPT codes clearer and more relevant for day-to-day medical practice. It's a technical stance, primarily concerned with operational efficiency and clarity in healthcare, which doesn't inherently align with a political ideology.<br><br>2. **Uniform Interpretation of CPT Codes**: Advocating for uniform interpretation by Medicare and other payers is also a technical position, aiming for consistency in medical billing practices. This could be seen as appealing to principles of fairness and standardization, which are not exclusive to any one political ideology.<br><br>3. **Opposition to Compression of Codes for Cognitive Services**: This is about opposing the simplification of codes for cognitive services, which might affect how these services are billed and reimbursed. It's a stance focused on the specifics of medical coding, without a clear liberal or conservative leaning.<br><br>4. **Adequate Recognition and Reimbursement for Complex Evaluations**: This point advocates for appropriate reimbursement for the evaluation of complex patients. It could be interpreted as a stance supporting the financial sustainability and recognition of medical practitioners' expertise, but it doesn't directly align with a liberal or conservative viewpoint.<br><br>In summary, these points are more about specific healthcare policy and administrative practices rather than broad political ideologies. They reflect concerns about operational clarity, fairness, and adequate compensation in healthcare, which are issues that can be important to professionals across the political spectrum.	neutral

ACP	Provider Based Billing	2023	The text you provided does not align strictly with either a liberal or conservative viewpoint in the traditional political sense. Instead, it reflects a perspective on healthcare economics and policy that focuses on efficiency, transparency, and the equitable delivery of healthcare services.<br><br>1. **Efficiency in Care Delivery**: The text supports the idea of delivering care in the most efficient setting while maintaining quality. This approach is not inherently political but is concerned with healthcare system efficiency and cost-effectiveness.<br><br>2. **Transparency in Billing**: Advocating for transparency in billing policies by hospitals and hospital-owned outpatient practices is a matter of patient rights and consumer protection, which can be supported across the political spectrum.<br><br>3. **Provider-Based Billing**: The text's opposition to using provider-based billing as a mechanism for hospitals to recoup funding or ensure access to care suggests a preference for alternative healthcare funding and access strategies. This position could be interpreted as a critique of certain market-driven healthcare practices, which might be more closely aligned with liberal perspectives that favor increased regulation and oversight in healthcare. However, the focus on efficiency and cost-effectiveness can also resonate with conservative views that emphasize fiscal responsibility.<br><br>4. **Alternative Support Means**: The advocacy for increased/improved health insurance coverage, strengthened workforce policies, and delivery system reforms indicates a holistic approach to healthcare policy that does not neatly fit into a conservative/liberal dichotomy. Instead, it suggests a pragmatic approach to addressing healthcare system issues.<br><br>In summary, the text represents a pragmatic and policy-oriented perspective on healthcare economics, focusing on efficiency, patient rights, and systemic reform, rather than aligning with a specific liberal or conservative ideological viewpoint.	neutral
ACP	Reimbursing Physicians for Telephone Care	2023	The text provided appears to align more closely with a viewpoint that doesn't strictly adhere to traditional liberal or conservative labels but incorporates aspects that could be appealing to both sides. It focuses on pragmatic solutions to healthcare reimbursement, particularly concerning telehealth services, a topic that has gained significance across the political spectrum.<br><br>1. **Support for Adequate Reimbursement**: Advocating for adequate reimbursement by Medicare and other payers for telephone consultations and health-related communications is a viewpoint that could be seen as aligning with liberal values. Liberals often advocate for comprehensive healthcare coverage and might support measures ensuring that physicians are adequately compensated for telehealth services.<br><br>2. **Collaboration with the Physician Community**: The recommendation for Medicare and other payers to work with the physician community to develop reimbursement guidelines could be seen as a balanced approach. It reflects a collaborative stance, seeking to involve healthcare professionals directly in the decision-making process, which might appeal to both conservative and liberal viewpoints.<br><br>3. **Ensuring No Reduction in Separate Payments**: The emphasis on ensuring that payment for telephone communications does not reduce separate payments for evaluation and management (E/M) services might resonate more with conservative values. Conservatives often advocate for free-market principles and might support the idea of fair compensation without reducing other service payments.<br><br>In summary, the recommendations are focused on practical aspects of healthcare reimbursement, particularly in the context of telehealth, and incorporate ideas that could be supported by both liberal and conservative viewpoints. They emphasize adequate compensation, collaboration between payers and physicians, and fair reimbursement practices without clear adherence to a strictly liberal or conservative agenda.	neutral

ACP	ACP Recommendations for Achieving an Interoperable National Healthcare Information System	2019	The text you've provided outlines a set of recommendations for developing and implementing a national interoperable healthcare information infrastructure. It's important to understand that aligning a policy or recommendation with a specific political viewpoint like "liberal" or "conservative" can be subjective and complex, as different aspects of the policy may resonate differently with each ideology. However, I'll attempt to analyze key elements of the text in relation to typical liberal and conservative viewpoints.<br><br>1. **Support for Interoperable Health Information Networks**: This point suggests a significant role for federal involvement and leadership in healthcare, which often aligns more with liberal viewpoints that favor governmental intervention in healthcare systems. Conservatives typically prefer less federal involvement and more market-driven solutions.<br><br>2. **Leadership and Advocacy Role of ACP**: The proactive stance in advocating for federal initiatives and participating in public-private partnerships can be seen in both liberal and conservative lights. Liberals may focus on the advocacy for federal initiatives, while conservatives might appreciate the involvement of private sector initiatives.<br><br>3. **Avoidance of Unfunded Mandates**: This point, emphasizing that these healthcare technologies should not become unfunded mandates, can resonate with conservative principles, which often stress fiscal responsibility and caution against imposing financial burdens without providing necessary funding.<br><br>4. **Support for Voluntary Standards**: The recommendation for voluntary standards over federal mandates could align with conservative principles favoring less federal regulation and more autonomy for private entities.<br><br>5. **Practicality and Testing of Technologies**: This aspect, emphasizing practicality and thorough testing before national implementation, doesn't distinctly align with either liberal or conservative viewpoints, as it's a broadly accepted principle in policy implementation.<br><br>6. **Involvement of Physicians and Stakeholders**: The emphasis on involving physicians and stakeholders in the development process is a universal approach and doesn't distinctly align with a specific political ideology.<br><br>7. **Resource Allocation for Technology and Training**: The call for adequate time and financial resources aligns somewhat with liberal views, which often advocate for well-funded public initiatives and support for professionals adapting to new systems.<br><br>8. **Preservation of Clinical Autonomy**: The emphasis on preserving the physician's decision-making autonomy could resonate more with conservative values, which often prioritize individual professional judgment and freedom from overbearing regulations.<br><br>9. **Access to Health Records**: This point, ensuring clinician and patient access to health records, aligns with a general healthcare improvement goal rather than a specific political ideology.<br><br>10. **Efficiency in Systems Integration**: This is a practical recommendation, focusing on efficiency and doesn't distinctly align with either liberal or conservative viewpoints.<br><br>11. **Insurance Company Responsibilities**: This recommendation for insurance companies to provide clear information could be seen as a form of regulation, which might lean slightly towards liberal views favoring consumer protection and transparency.<br><br>12. **Avoidance of Mandatory Use of EHR Functions**: The emphasis on not mandating the use of each EHR function could resonate more with conservative views that are wary of overregulation.<br><br>13. **E-Prescribing Systems Specifications**: The detailed specifications for e-prescribing systems emphasize practicality, efficiency, and privacy, which are generally non-partisan issues.<br><br>In summary, the recommendations contain elements that could align with both liberal and conservative viewpoints. The emphasis on federal involvement, advocacy, and resource allocation may lean more towards liberal ideals, while the stress on avoiding unfunded mandates, preserving clinical autonomy, and favoring voluntary standards over federal mandates might resonate more with conservative principles. However, many points are practical, efficiency-oriented, and focused on improving healthcare systems, transcending simple political categorization.	neutral

ACP	e-Prescribing	2019	The text you provided discusses the adoption and implementation of e-prescribing technology within the healthcare system, focusing on its benefits, challenges, and efforts at various levels to facilitate its adoption. This text can align with viewpoints from both liberal and conservative perspectives, but with different emphases:<br><br>1. **Liberal Viewpoint Alignment**:<br>- **Support for Regulation and Standards**: Liberals often advocate for government involvement in setting standards and regulations for healthcare to ensure quality and safety. The text's support for developing foundation standards for e-prescribing functions aligns with this view.<br>- **Promotion of Technology for Equity**: Liberals may emphasize the role of technology in improving healthcare accessibility and quality, particularly for under-served communities like small, medium, and rural practices.<br>- **Advocacy for Government Funding**: The mention of loans, grants, and increased payment to facilitate e-prescribing adoption could resonate with liberal policies favoring government investment in healthcare infrastructure.<br><br>2. **Conservative Viewpoint Alignment**:<br>- **Emphasis on Efficiency and Cost-Savings**: Conservatives often focus on reducing healthcare costs and increasing efficiency. The text's mention of administrative efficiencies and lower costs associated with e-prescribing technology aligns with this perspective.<br>- **Support for Private Sector Involvement**: The acknowledgment of efforts at the private sector level and the creation of safe harbors to the Medicare Anti-kickback Act suggest a favorable view towards private sector participation in healthcare, a typical conservative stance.<br>- **Concerns about Implementation Costs and Technical Challenges**: Highlighting the challenges and costs of implementing e-prescribing, especially for smaller practices, aligns with a conservative viewpoint that is cautious about new regulations or technologies that might impose financial burdens on businesses.<br><br>In summary, the text presents a balanced view that recognizes the benefits of e-prescribing technology in healthcare while acknowledging the challenges and barriers to its implementation. It contains elements that could be endorsed by both liberal and conservative viewpoints, depending on the specific aspects emphasized.	neutral
ACP	Electronic Prescribing of Controlled Substances	9/2021	The text you provided appears to be a statement on healthcare policy, specifically regarding the use of electronic prescribing for controlled substances and the recommendation for a backup system. This statement doesn't align strictly with either a liberal or conservative viewpoint as it focuses more on practical aspects of healthcare management rather than ideological principles.<br><br>Liberal and conservative viewpoints in the context of healthcare often differ on broader issues such as the extent of government involvement in healthcare, healthcare affordability and accessibility, and regulations on healthcare providers and pharmaceutical companies. However, the statement you provided is more about the implementation of a specific practice (electronic prescribing) and ensuring reliability (through a backup system). These concerns are operational and can be shared by individuals across the political spectrum, as they focus on efficiency and safety rather than ideological beliefs.	neutral
ACP	Downcoding	2019	The text you've provided seems to reflect a professional and technical perspective rather than a strictly political one. It's focused on the operational and administrative aspects of healthcare, particularly in relation to Medicare and Medicaid services, coding practices, and documentation standards.<br><br>The American College of Physicians (ACP) is primarily concerned with the practicalities of healthcare delivery, especially in terms of coding and billing practices, which is a significant issue for healthcare providers. Their emphasis on working with various bodies like the Centers for Medicare and Medicaid Services, Congress, and the Medicare Payment Advisory Commission (MedPAC) indicates a desire for collaborative and practical solutions to these issues.<br><br>This text does not explicitly align with traditional liberal or conservative viewpoints as understood in a political context. Instead, it is more focused on pragmatic healthcare administration, aiming to improve efficiency, clarity, and fairness in medical billing and coding practices. This kind of focus is common in professional organizations that work to represent the interests of their members in specific industries or fields, in this case, the medical profession.	neutral

ACP	Coding	2016	The text you provided, "ACP opposes burdensome coding and record-keeping requirements unless patient care benefits result from their implementation. (HoD 89; reaffirmed BoR 04; reaffirmed BoR 16)", appears to be a statement from a healthcare organization (presumably the American College of Physicians, ACP) regarding their stance on administrative procedures in healthcare.<br><br>This statement does not clearly align with a traditional liberal or conservative viewpoint as understood in a political context. Rather, it focuses on practical and administrative aspects of healthcare, emphasizing the importance of patient care over bureaucratic processes. The opposition to burdensome coding and record-keeping is rooted in a concern for efficiency and effectiveness in healthcare, rather than a specific political ideology.<br><br>Both liberal and conservative viewpoints might find aspects of this statement agreeable. Liberals might support the emphasis on patient care and the skepticism towards potentially unnecessary bureaucracy. Conservatives might agree with the idea of reducing what they could see as excessive government or administrative involvement in healthcare. However, the statement is more about healthcare management and policy than about broader political ideologies.	neutral
ACP	Support for AMA/CPT	2016	The text you provided appears to be a position statement related to the American College of Physicians (ACP) approving and supporting the American Medical Association (AMA) Current Procedural Terminology (CPT) coding and nomenclature. It doesn't directly align with a traditional liberal or conservative political viewpoint as it focuses on medical coding and professional standards within the medical field.<br><br>The ACP's support for CPT coding and the AMA's efforts suggests an endorsement of standardized practices and formalized nomenclature in medical services, which is more about professional and industry standards rather than political ideology.<br><br>Liberal and conservative viewpoints typically differ on issues like healthcare policy, government involvement in healthcare, and funding, rather than on the technical aspects of medical practice like coding and nomenclature. This statement is more indicative of the medical community's internal governance and professional standards.	neutral
ACP	Third-Party Manipulation of Terminology	2016	The text you've provided appears to be a policy statement from a medical or health-related organization, specifically addressing the modification of procedural descriptions or terminologies by third parties without professional medical consultation. It emphasizes that any use of modified data in terminology systems is considered invalid for various purposes like reimbursement, peer review, and utilization review.<br><br>This statement doesn't align clearly with a traditional liberal or conservative viewpoint as understood in a political context. Instead, it reflects a stance on professional standards and practices within the medical or healthcare field. The focus is on maintaining accuracy and professional oversight in medical documentation and data usage, which is more of a professional and ethical issue than a political one.<br><br>Both liberal and conservative viewpoints in politics may have opinions on such matters, particularly in the context of healthcare management, insurance, and regulatory policies. However, this specific statement is neutral and focused on professional standards within the healthcare industry, rather than being aligned with a specific political ideology.	neutral
ACP	Timely Release of New CPT/CMS Common Procedural Coding System Codes	2016	The text you've provided reflects a viewpoint that aligns more closely with moderate or centrist positions, rather than distinctly liberal or conservative ideologies. It focuses on practical aspects of healthcare administration, specifically advocating for the timely release of codes for new medical treatments, procedures, and medications, as well as fair and prompt reimbursement by the Centers for Medicare & Medicaid Services (CMS).<br><br>This perspective emphasizes efficiency and clarity in healthcare administration, which can be seen as a non-partisan issue. It does not directly touch upon more ideologically divisive topics typically associated with liberal or conservative viewpoints, such as the extent of government involvement in healthcare or broader social and economic policies.<br><br>The American College of Physicians (ACP), as suggested by the text, appears to be focusing on practical improvements within the existing healthcare system rather than advocating for large-scale reforms or policy shifts that might be more clearly aligned with a specific political ideology.	neutral

ACP	Controlling Health Care Costs: Options for Controlling Administrative Costs	2022	The text you've provided seems to align more closely with a liberal viewpoint. Here's a breakdown of why:<br><br>1. **Comprehensive Assessment of Administrative Burdens on Physicians**: This reflects a liberal perspective focused on analyzing and potentially reducing the regulatory burden on healthcare providers. It suggests a government-led approach to streamline and optimize healthcare administration, which is more in line with liberal ideology that supports active government intervention in healthcare.<br><br>2. **Legislation Requirements and Funding**: The call for new regulatory requirements to be accompanied by funding, and a moratorium on unfunded mandates, aligns with liberal perspectives that favor comprehensive, funded government regulations rather than imposing unfunded mandates that could stress small practices.<br><br>3. **Uniform Health Insurance Forms**: The suggestion for uniformity in health insurance forms to simplify processes also leans liberal, emphasizing standardization and simplification in healthcare administration, possibly leading to greater government involvement in setting these standards.<br><br>4. **Online Platform for Benefit Information**: Establishing an online platform for easy access to benefit information and forms suggests a preference for modernizing and streamlining healthcare administration, which is a typical liberal stance that favors using technology to enhance public services.<br><br>5. **Standard Physician Credentialing**: The idea of standardizing processes with a focus on efficiency and less disruption aligns with liberal values of effective government intervention to streamline healthcare administration.<br><br>6. **Transparency in Health Insurance Companies**: The requirement for health insurance companies to disclose how healthcare premiums are spent is in line with liberal principles of transparency, accountability, and consumer protection in the healthcare industry.<br><br>Overall, these points suggest a preference for structured, government-led reform in healthcare administration, with an emphasis on efficiency, transparency, and funding. This approach is generally more consistent with liberal viewpoints, which often advocate for government involvement in ensuring equitable and efficient healthcare systems.	liberal
ACP	Efficiency Benchmarks for Health Insurance Companies	2022	The text you provided seems to be a policy statement related to healthcare, specifically about the American College of Physicians (ACP) working with the American Medical Association (AMA) to establish performance benchmarks for health insurance companies. It's focused on furnishing this information to various stakeholders including providers, purchasers, patients, and policymakers.<br><br>Determining whether this statement aligns with a liberal or conservative viewpoint isn't straightforward, as it involves various aspects of healthcare policy that can be interpreted differently depending on one's political perspective. However, I can provide some insights into how it might be viewed from both perspectives:<br><br>1. **Liberal Viewpoint**: Liberals generally advocate for greater transparency in healthcare and support efforts to make health insurance companies more accountable. The emphasis on providing information to patients and policymakers aligns with the liberal focus on consumer rights and informed decision-making in healthcare. Therefore, this statement could be seen as aligning with liberal principles of transparency and accountability in the health insurance sector.<br><br>2. **Conservative Viewpoint**: Conservatives typically support market-driven solutions in healthcare and may view the establishment of performance benchmarks as a way to promote competition and efficiency among insurance companies. This can align with conservative values of free-market principles and limited government intervention. However, some conservatives might be cautious about the potential for increased regulation or government oversight implied in such a process.<br><br>In summary, the statement touches on aspects of healthcare policy that both liberals and conservatives might find agreeable, but for different reasons. Liberals might focus on the consumer protection and transparency aspects, while conservatives might appreciate the potential for market efficiency and competition. It's a complex issue and doesn't neatly align with a single political viewpoint.	neutral

ACP	Physicians and Joint Negotiations	2022	The text you provided is a position statement regarding the rights and practices of physicians in negotiating with health insurance plans and addressing work conditions. It's not straightforward to categorize this statement as strictly aligning with either a liberal or conservative viewpoint because it touches on a mix of issues that could resonate with values traditionally associated with both political spectrums.<br><br>1. **Advocacy for Collective Negotiation and Worker Rights**: The support for physicians to jointly negotiate with insurance plans and address issues like payment policies, working conditions, and patient care quality is a stance that often aligns with liberal viewpoints. This perspective emphasizes the empowerment of workers (in this case, physicians) in their professional environment.<br><br>2. **Opposition to Actions Harming Patients and Anticompetitive Practices**: The statement's opposition to strikes, slow-downs, and other actions that could harm patient care, as well as its stance against price fixing and anticompetitive behavior, can be seen as aligning with conservative principles that prioritize market competition and patient care over collective action that might disrupt services.<br><br>3. **Physician Autonomy and Voluntary Association**: The emphasis on the voluntary nature of organization membership and the right of physicians to choose whether to join groups that represent them in negotiations is a principle that resonates with conservative values, particularly those emphasizing individual choice and freedom from compulsory association.<br><br>4. **Exclusion of Non-Physician Providers from Bargaining Units**: This aspect might be seen as a more conservative stance, focusing on the distinct professional status of physicians compared to other healthcare providers.<br><br>5. **Conflict-Resolution and Educational Content Oversight**: The call for established conflict-resolution mechanisms and the stipulation that educational content remains under the control of specific committees and directors may be seen as a neutral or administrative stance, not particularly aligned with a political ideology.<br><br>Overall, the statement reflects a blend of principles, advocating for worker rights and collective negotiation (more liberal) while also emphasizing individual choice, market competition, and professional autonomy (more conservative). It's an example of a position that doesn't fit neatly into a single political category but rather crosses over different aspects of both liberal and conservative ideologies.	neutral
ACP	Comparative Effectiveness	2020	The positions outlined by the American College of Physicians (ACP) regarding the improvement of access to information comparing clinical management strategies and the role of the federal government in funding and maintaining a comparative effectiveness entity, like the Patient-Centered Outcomes Research Institute (PCORI), align with both liberal and conservative viewpoints in different aspects.<br><br>1. **Liberal Viewpoints**:<br>- The emphasis on the federal government playing a significant role in healthcare, including funding and maintaining comparative effectiveness entities, aligns with a more liberal perspective, which typically supports a robust role for government in healthcare.<br>- The focus on transparency, stakeholder involvement (including diverse representation), and the development of evidence-based healthcare aligns with liberal values of inclusivity, transparency, and reliance on scientific expertise.<br><br>2. **Conservative Viewpoints**:<br>- The insistence on the entity's independence from undue governmental and private sector influence may resonate with conservative ideals of limiting government overreach and ensuring market freedom.<br>- The recommendation that the entity should not be directly involved in healthcare decisions but rather provide information for such decisions aligns with conservative preferences for less government intervention in individual healthcare choices.<br><br>3. **Bipartisan/Non-partisan Elements**:<br>- The focus on quality, efficiency, and evidence-based practice in healthcare transcends typical partisan lines and can be seen as a non-partisan approach to improving healthcare systems.<br>- The emphasis on the need for healthcare decisions to be patient-centered and considerate of diverse needs and values is a principle that can garner support across the political spectrum.<br><br>In summary, the ACP's positions reflect a blend of ideals that can be found in both liberal and conservative ideologies, focusing on	neutral

			the role of government, scientific evidence, inclusivity, and efficient healthcare, while maintaining an emphasis on independence from government and private sector influence.	
ACP	Health Care During Incarceration: A Policy Position Paper	2022	The text you provided outlines a series of positions and recommendations from the American College of Physicians (ACP) regarding correctional medicine and the engagement of clinicians in the U.S. justice system. These positions advocate for:<br><br>1. Ensuring that the quality of care in jails and prisons is consistent with community standards.<br>2. Providing incarcerated individuals with timely access to evidence-based medical care.<br>3. Offering nutritious food, opportunities for physical activity, and preventive health care.<br>4. Ensuring quality care for chronic noncommunicable and infectious diseases.<br>5. Supporting treatment over incarceration for substance use disorders.<br>6. Providing comprehensive behavioral health care.<br>7. Ensuring comprehensive reproductive health services and respectful treatment for women and LGBTQ+ individuals.<br>8. Providing dignified and appropriate care for aging, disabled, and terminally ill incarcerated individuals.<br>9. Treating immigrant detainees with dignity and providing oversight of their healthcare.<br>10. Facilitating community reentry and supporting continuity of healthcare and social services.<br><br>These recommendations generally align more with a liberal viewpoint, as they emphasize comprehensive healthcare, equity, and humane treatment within the correctional system. Liberal perspectives often prioritize healthcare access, the dignity and rights of marginalized groups, and a rehabilitative approach to criminal justice.<br><br>Conservative viewpoints, on the other hand, might focus more on the aspects of personal responsibility, cost-effectiveness, and the primary role of incarceration as a means of punishment and deterrence. While there can be overlap (such as in the support for evidence-based approaches to healthcare), conservative perspectives may place less emphasis on expanding services and rights within the correctional system.<br><br>It's important to note, however, that perspectives on these issues can vary widely within both liberal and conservative camps, and there can be significant overlap in some areas. These positions by the ACP represent a healthcare-focused approach that prioritizes the well-being and rights of incarcerated individuals, which is generally more aligned with contemporary liberal values.	liberal

ACP	Controlling Health Care Costs: Enhance and Coordinate Technology Assessments	2022	The text presents a balanced viewpoint, incorporating elements from both liberal and conservative perspectives. Here's a breakdown:<br><br>1. **Liberal Viewpoint Elements:**<br>- Advocates for an independent and evidence-based assessment process for medical technologies, aligning with a more liberal perspective that emphasizes regulatory oversight and the importance of evidence in policymaking.<br>- Calls for comparative effectiveness research and incorporating cost-benefit analysis into healthcare decisions, reflecting a desire for systematic and rational healthcare management, a principle often supported by liberal ideologies.<br>- Suggests dissemination of information to educate the public, a notion that aligns with liberal values of transparency and informed decision-making.<br><br>2. **Conservative Viewpoint Elements:**<br>- Emphasizes the need to balance research and regulation with the potential to stifle innovation, reflecting a conservative concern about over-regulation and its impact on market dynamics and technological advancement.<br>- States that coverage should not be denied based solely on cost-effectiveness, which aligns with conservative priorities of individual choice and skepticism towards purely economic rationales for healthcare decisions.<br><br>3. **Centrist or Non-Partisan Elements:**<br>- The focus on ensuring that new technologies of value are not hindered and that coverage decisions reflect clinical effectiveness can be seen as broadly appealing, not strictly adhering to one political ideology.<br>- The emphasis on appropriate utilization and outcomes of technology reflects a practical, outcome-focused approach that both sides might agree on.<br><br>Overall, the text proposes a nuanced approach that incorporates regulatory oversight and effectiveness research (more liberal) while also considering the importance of innovation and not solely relying on cost-effectiveness for decisions (more conservative). It reflects a centrist or pragmatic stance, aiming to balance various priorities and concerns in healthcare policy.	neutral
ACP	Controlling Health Care Costs: Pay Appropriately for Health Care Services, and Encourage Adoption of the Patient-Centered Medical Home and Other Innovative Models of Health Care Delivery	2022	The text presents a viewpoint that can be interpreted as aligning with aspects of both liberal and conservative ideologies, though it leans more towards a liberal perspective due to its focus on systemic healthcare reform and the expansion of coordinated care through models like the Patient-Centered Medical Home (PCMH).<br><br>1. **Liberal Viewpoint Indicators:**<br>- Advocating for an acceleration of the PCMH model, which emphasizes comprehensive, coordinated care, is a progressive approach to healthcare reform.<br>- The suggestion for severity-adjusted monthly bundled payments and performance-based incentives reflects a desire for a more equitable, outcome-oriented healthcare system, a common liberal stance.<br>- Calling for increased payments to primary care physicians to ensure a sufficient supply and thus improve overall healthcare efficiency and cost-effectiveness aligns with liberal priorities of investing in preventative and primary care.<br><br>2. **Conservative Viewpoint Considerations:**<br>- The mention of fee-for-service payments and the introduction of various payment models indicates a willingness to maintain some aspects of the traditional market-driven healthcare system, which might resonate with more conservative ideologies favoring less drastic overhauls and market solutions.<br>- The focus on efficiency and cost-saving measures can also be appealing from a fiscal conservative standpoint.<br><br>Overall, while there are elements that might be seen as conservative, the emphasis on systemic reform, care coordination, and investment in primary care leans towards a liberal viewpoint focused on comprehensive healthcare changes. The specific reference to "evidence-based quality, patient satisfaction, and efficiency measures" indicates a nuanced approach that aims to balance quality and cost, reflecting a pragmatic aspect that might transcend the simple liberal-conservative dichotomy.	probably liberal

ACP	Envisioning a Better U.S. Health Care System for All: Coverage and Cost of Care – Universal Coverage	None listed	The text you've provided from the American College of Physicians outlines various policies and supports aimed at improving the efficiency and cost-effectiveness of healthcare. It doesn't explicitly align with a traditional liberal or conservative viewpoint as it focuses on pragmatic health system reforms rather than ideological positions. However, different elements might resonate more with one side or the other.<br><br>1. **Reducing Excessive Pricing and Improving Efficiency**: Both liberals and conservatives are generally concerned with reducing healthcare costs, but they might disagree on methods. Liberals might favor direct regulation and oversight, while conservatives might prefer market-based solutions. However, both could agree on the need for transparency and efficiency.<br><br>2. **Overtreatment, Low-Value Care, and Preventable Diseases**: Efforts to reduce low-value care and preventable diseases are typically bipartisan, though the approaches might differ. Liberals might support more government intervention, while conservatives might look for market-based incentives.<br><br>3. **Global Budgets and All-Payer Rate Setting**: These are often associated with more liberal policies. They suggest a structured and possibly more regulated approach to healthcare financing, which conservatives might oppose unless it's designed to enhance market efficiency.<br><br>4. **Reference Pricing and Cost-Effectiveness Analyses (CEAs)**: Reference pricing and CEAs can be seen as tools to introduce more market principles into healthcare by making pricing more competitive and transparent, which might appeal to conservatives. However, the actual implementation might require regulatory frameworks more typically supported by liberals.<br><br>In summary, while the text doesn't neatly fit into a liberal or conservative box, different aspects of it might be more appealing to one side or the other based on how they perceive the role of government versus the market in healthcare. The overall aim of improving efficiency and reducing costs is a universal one, but the means of achieving it reflect different ideological stances.	neutral
ACP	Joint Statement in Support of COVID-19 Vaccine Mandates for All Workers in Health and Long-Term Care	7/24/2021	The text primarily aligns with a liberal viewpoint. It advocates for mandatory COVID-19 vaccinations for healthcare and long-term care workers, emphasizing the ethical commitment to patient health and the importance of vaccination in controlling the pandemic. This perspective is often associated with a public health and community welfare approach, emphasizing the collective responsibility and the need for stringent measures to protect public health, which are more commonly associated with liberal ideologies.<br><br>The text supports vaccine mandates in healthcare settings, discusses the danger of contagious variants, and highlights the role of vaccination in returning to normalcy, all of which align with a proactive public health stance. It also acknowledges the need to engage with marginalized populations and address vaccine hesitancy, indicating a recognition of social inequalities and the importance of inclusivity in healthcare, which are values typically emphasized in liberal viewpoints.<br><br>However, it's important to note that public health and safety can be a bipartisan concern, and there might be individuals with conservative views who also support vaccination efforts due to the priority of protecting lives and restoring economic stability. Political viewpoints can be complex, and individuals may hold a mix of beliefs that don't neatly fit into one category or the other.	liberal
ACP	Statement on Global COVID-19 Vaccine Distribution and Allocation: On Being Ethical and Practical	6/8/2021	The text primarily aligns with a liberal viewpoint. It emphasizes ethical obligations, global cooperation, and equitable access to healthcare resources, which are themes commonly associated with liberal perspectives. The focus on advocating for the health and well-being of patients and communities worldwide, addressing social inequities, and the ethical imperative to distribute COVID-19 vaccines equitably, especially to countries and populations most in need, resonates with liberal values that prioritize social justice, human rights, and international solidarity.<br><br>The text also discusses the importance of coordinated global responses, equitable vaccine allocation, and the necessity of prioritizing marginalized and underserved populations. These elements reflect a commitment to principles of equity and justice, as well as a belief in the collective responsibility for public health, which are more typically associated with liberal ideologies.<br><br>Furthermore, the reference to previous global health initiatives like PEPFAR and the call for similar cooperative efforts indicate a preference for multilateral approaches and international cooperation in solving global issues, which is another hallmark of liberal thinking.<br><br>Conservative viewpoints, on the other hand, might focus more on individual responsibility, national interests, and less on global cooperative efforts or addressing systemic inequities. While the text does not directly oppose any conservative principles, its	liberal

			emphasis on collective action, global solidarity, and ethical obligations to address inequities is more closely aligned with liberal ideals.	
ACP	Policy Statement on the Provision, Distribution, and Payment of COVID-19 Vaccines	2021	The text provided is a policy statement from the American College of Physicians (ACP) on the ethical allocation of vaccines during pandemics, including COVID-19. It does not neatly align with a strictly liberal or conservative viewpoint as it primarily focuses on public health and ethical considerations. Here's a breakdown of its components in terms of how they might align with general political viewpoints:<br><br>1. **Support for state-licensed clinicians to administer vaccines and physician involvement in counseling**: This approach is generally non-partisan, emphasizing the role of medical professionals in vaccine distribution and patient education.<br><br>2. **Wide distribution of vaccine-related materials and support for a national campaign**: This might be seen as more aligned with liberal viewpoints that often emphasize the importance of broad public health initiatives and government involvement in healthcare education.<br><br>3. **Coordination among healthcare professionals**: The call for collaboration among healthcare providers is a practical approach to healthcare that does not inherently align with a specific political ideology.<br><br>4. **Integration of health IT systems and support for public health registries**: Advocating for improved health IT and data systems could be seen as technocratic and forward-thinking, which does not inherently align with a particular political spectrum. However, the emphasis on systematic data collection and public health infrastructure might resonate more with those holding liberal views, who often support stronger government roles in healthcare.<br><br>5. **Insurance reimbursement and funding for vaccination programs**: Requiring insurers to provide adequate reimbursement and supporting funding for vaccination of uninsured adults might be more aligned with liberal viewpoints that advocate for broader healthcare coverage and support for underprivileged populations.<br><br>Overall, while some aspects might resonate more with one political ideology than another, the statement is primarily centered around public health and ethical considerations rather than partisan politics. It advocates for practical, informed, and ethical approaches to vaccine distribution, emphasizing the importance of scientific guidelines, healthcare collaboration, and public education, all of which are generally considered essential components of effective public health strategies.	probably liberal

ACP	ACP Policy Statement on Wearing Masks in Community Settings	1/11/2021	The text from the American College of Physicians outlines a series of measures and supports related to mask-wearing and other preventative strategies against COVID-19. It doesn't align exclusively with a liberal or conservative viewpoint as traditionally defined in U.S. politics. Instead, it focuses on public health and safety measures based on scientific understanding and epidemiological data.<br><br>In the U.S. political context, liberals (often associated with the Democratic Party) generally favor more government intervention in public health matters and might be more supportive of mandates and public health campaigns like those described. Conservatives (often associated with the Republican Party), especially in recent years, might be more skeptical of government mandates and emphasize personal freedom and responsibility.<br><br>However, public health is inherently a non-partisan issue, and there are individuals and groups across the political spectrum who support or oppose various measures based on a wide range of beliefs and values. The text is primarily scientific and health-focused rather than politically motivated. It aims to address a public health crisis with comprehensive strategies, which can be supported by people with various political beliefs who prioritize public health and evidence-based policies.	neutral
ACP	Policy Statement on the Ethical Allocation of Vaccines During Pandemics Including COVID-19	11/23/2020	The text primarily aligns with a liberal viewpoint. It emphasizes the equitable allocation of COVID-19 vaccines, prioritizing high-risk groups and those most vulnerable, including health care workers, older adults, individuals with disabilities, and people with underlying health conditions. The support for a phased allocation based on risk and the focus on equity, including prioritization based on the Social Vulnerability Index, align with liberal values of social justice, equality, and collective responsibility.<br><br>The text's support for the National Academies of Sciences, Engineering, and Medicine (NASEM) report and its emphasis on ethical principles such as maximizing public health benefit, promoting equity, and mitigating health inequities also resonate with a liberal perspective. These principles reflect a focus on the well-being of the community as a whole and a commitment to ensuring that vulnerable and marginalized groups receive fair access to vaccines.<br><br>Moreover, the text's recommendation for open communication, transparency, and evidence-based decision-making aligns with liberal values of openness, accountability, and reliance on scientific expertise. The approach of adapting the allocation scheme as more evidence becomes available and as the pandemic circumstances change reflects a progressive mindset open to evolving strategies based on new information.<br><br>While the focus on individual rights and autonomy might find some resonance with conservative values, the overall emphasis on collective well-being, social justice, and prioritizing the most vulnerable groups in society aligns more closely with liberal principles.	liberal
ACP	MCAT Position Statement	8/3/2020	The text provided appears to align more closely with a liberal viewpoint. Here's the analysis:<br><br>- **Emphasis on Diversity and Inclusion**: The call for holistic reviews of applicants in medical school admissions, particularly to account for challenges faced by applicants of color and those from lower socioeconomic backgrounds, aligns with liberal values that prioritize diversity, equity, and inclusion.<br><br>- **Response to Public Health Crisis**: The recognition of the COVID-19 pandemic as a significant disruptor for pre-medical students, especially those from marginalized communities, and the suggestion to waive MCAT requirements during this period reflects a progressive approach to education and public health.<br><br>- **Advocacy for Change in Standard Practices**: The push to alter traditional admission processes in light of current challenges aligns with a more liberal stance that often seeks to reform existing systems to be more inclusive and responsive to societal needs.<br><br>It's important to note that while these points suggest a liberal leaning, the text is primarily focused on addressing specific, practical issues related to medical school admissions during a public health crisis. The viewpoint is more about ensuring fairness and access to medical education during an unprecedented time rather than pushing a broader ideological agenda.	liberal
ACP	Racism and Health: A Position Statement from the American College of Physicians	6/8/2020	The text primarily aligns with a liberal viewpoint. It emphasizes the condemnation of systemic racism and discrimination, advocating for equitable access to healthcare, and calling for comprehensive reforms in law enforcement practices to ensure equality and reduce violence and discrimination, particularly against Black, indigenous, and other people of color. The focus on social justice, systemic change, and the protection of minority rights are themes commonly associated with liberal ideologies, which typically emphasize equality, civil rights, and the need for systemic reforms to address social inequalities. The text's detailed policy recommendations and its commitment to addressing the impacts of racism in various societal aspects reflect a progressive approach to social issues, aligning with liberal values of inclusivity, equity, and justice.	liberal

ACP	*ACP and AMA Joint Statement on Non-Discrimination in Health Care: A Wake-Up Call for American Health Care: Emerging Ethical Lessons from Covid-19*	6/8/2020	The text presents a viewpoint that aligns more closely with liberal ideologies. It emphasizes the need for equitable health care, the recognition of systemic and structural barriers affecting marginalized communities, and the ethical implications of resource allocation during the Covid-19 pandemic. The focus on addressing health inequities, advocating for fairness in treatment access, and urging for systemic changes in health care delivery reflects principles commonly associated with liberal thought, such as social justice, equality, and a collective responsibility to support vulnerable populations. The text calls for fundamental reforms to the health care system, aligning with liberal values of progress and inclusivity.	liberal
ACP	Partial Resumption of Economic, Health Care and Other Activities While Mitigating COVID-19 Risk and Expanding System Capacity: A Clinical and Public Policy Guidance from the American College of Physicians	5/6/2020	The text you've provided outlines recommendations from the American College of Physicians (ACP) on resuming economic and social activities safely during the COVID-19 pandemic, focusing on testing, contact tracing, privacy, public health authority collaboration, and resource allocation. It doesn't align strictly with liberal or conservative viewpoints as it is primarily a public health and scientific discourse. However, aspects of it may resonate differently with various political ideologies:<br><br>1. **Liberal Viewpoint Resonance:**<br>- **Emphasis on Public Health and Safety**: Liberals generally prioritize public health measures and may support the detailed, science-based approach to resuming activities safely.<br>- **Focus on Civil Liberties and Privacy in Contact Tracing**: The emphasis on privacy, non-discrimination, and civil liberties in the use of technology for public health surveillance aligns with liberal concerns about individual rights and government overreach.<br>- **Support for Vulnerable Populations**: The attention to older adults, those with underlying conditions, and communities with pronounced healthcare disparities may resonate with liberal values of equity and social justice.<br><br>2. **Conservative Viewpoint Resonance:**<br>- **Gradual Resumption of Economic Activities**: Conservatives might appreciate the phased approach to reopening the economy, as there is often a conservative emphasis on economic stability and minimizing restrictions.<br>- **Local and State Authority Empowerment**: Recommendations for state and local officials to tailor their approaches might appeal to conservative principles of smaller federal government and more localized decision-making.<br>- **Informed Consent and Opt-in for Surveillance**: The requirement for informed consent and the option to opt-in for public health surveillance technologies may align with conservative values of personal freedom and skepticism of government intrusion.<br><br>3. **Points of Potential Conflict or Debate:**<br>- **Government's Role in Healthcare**: The recommendation for government-provided resources and funding might be contentious, with liberals generally more supportive of government intervention in healthcare and conservatives preferring limited government involvement.<br>- **Balancing Public Health and Economic Interests**: There might be disagreements on the timing and extent of resuming activities, with some conservatives advocating for quicker reopening to boost the economy and liberals emphasizing caution and extended public health measures.<br><br>In summary, the text is a comprehensive set of health-focused recommendations that do not strictly adhere to a liberal or conservative viewpoint but contain elements that could be supported or contested by either side depending on their priorities and interpretations of public health and government roles.	neutral

ACP	Statement on Identifying “Essential Elements” to Ease Social Distancing Protocols, Address White House Guidance to “Re-open” the U.S.	4/17/2020	The text provided outlines a position from the American College of Physicians (ACP) regarding the U.S. Administration's "Guidelines for Opening Up America Again" and expresses a cautious approach to easing social distancing measures. It emphasizes an evidence-based, phased approach to reopening, the necessity of robust testing and contact tracing, and the potential risks of easing restrictions prematurely. This perspective aligns with key principles typically associated with a liberal viewpoint:<br><br>1. **Evidence-based decision-making:** The text emphasizes the importance of making decisions based on up-to-date data and scientific evidence, a stance commonly associated with liberal viewpoints that prioritize empirical evidence and expert opinion in policy-making.<br><br>2. **Public health over economic or political expedience:** The caution against prematurely easing social distancing reflects a prioritization of public health and safety over economic pressures or political agendas. This emphasis on the collective well-being is often more aligned with liberal ideologies.<br><br>3. **Support for robust government intervention:** The call for comprehensive testing, contact tracing, and necessary resources indicates support for active government involvement in managing the crisis, a principle more commonly found in liberal thought, which tends to support stronger government roles in addressing societal issues.<br><br>However, it's important to note that public health and safety are universal concerns, and there may be conservatives who also agree with the cautious, evidence-based approach outlined in the text. The pandemic has created a complex scenario where traditional political alignments might not always predict individual or group stances accurately. The ACP's position, as presented, is primarily focused on public health and safety based on scientific evidence, which transcends simple political categorization.	liberal
ACP	Protecting Patient and Physician Health and Safety During the COVID-19 Pandemic	4/1/2020	The text you've provided outlines various positions regarding the supply and use of personal protection equipment (PPE) for clinicians and health care workers, as well as advocating for the rights of physicians to speak out about safety and patient care concerns. This text doesn't align strictly with a liberal or conservative viewpoint as traditionally defined in U.S. politics; rather, it focuses on practical, ethical, and professional standards within the healthcare industry. Here's how the points relate to broader political ideologies:<br><br>1. **Advocating for adequate supply and distribution of PPE**: This can be seen as a public health and safety issue, which is generally a nonpartisan concern. However, the emphasis on government intervention to ensure supply might be more aligned with liberal views favoring government involvement in healthcare.<br><br>2. **Physicians bringing their own PPE and speaking out against unsafe conditions**: This emphasizes individual rights and responsibilities, which could resonate with conservative ideals of personal liberty and freedom of speech. Yet, the call for institutional support and the right to speak out without fear of retribution might also align with liberal values related to worker protection and whistleblowing.<br><br>3. **Improving care and patient safety through collaboration with health care systems**: This is a broadly supported goal across the political spectrum, as it focuses on improving outcomes and safety for patients, a universal concern.<br><br>4. **Protection against employment termination for speaking out**: This aspect emphasizes worker rights and protections, which is traditionally more aligned with liberal policies focused on labor rights and protections.<br><br>Overall, the text represents a blend of concerns and values that can resonate with both liberal and conservative viewpoints, depending on how the issues are framed and prioritized. It's more centered on professional ethics and standards in healthcare rather than partisan politics.	neutral

ACP	Statement on Nonurgent In-Person Medical Care	3/23/2020	The text you've provided outlines a perspective on healthcare delivery, particularly in the context of the COVID-19 pandemic, focusing on the shift to telehealth and the importance of safe and accessible patient care. It doesn't explicitly align with a traditional liberal or conservative viewpoint as it primarily addresses practical considerations for patient care during a public health crisis. Here's how it relates to both viewpoints:<br><br>1. **Liberal Viewpoint Considerations**:<br>- **Emphasis on Access and Safety**: Liberals generally advocate for broad access to healthcare and may support the proactive measures suggested in the text to maintain patient safety and care continuity.<br>- **Support for Telehealth**: Liberals might favor the expansion of telehealth as a means to increase access to healthcare, especially for underserved communities.<br>- **Regulatory Adaptation**: The call for insurance and government programs to adapt quickly and cover telehealth services, including "audio-only" phone calls, might resonate with liberal ideologies that often support stronger healthcare protections and adaptations for patient needs.<br><br>2. **Conservative Viewpoint Considerations**:<br>- **Efficient Use of Resources**: Conservatives might appreciate the focus on preventing unnecessary emergency room visits and hospital care, as it relates to efficient resource use and potentially reducing healthcare costs.<br>- **Adaptation to Crisis**: The pragmatic approach to adjusting healthcare delivery during a crisis might align with a conservative perspective that values flexibility and adaptability in private sectors, including healthcare.<br>- **Concerns Over Expanding Coverage**: Some conservatives might be cautious about rapidly expanding insurance coverage and payments for telehealth, concerned about the long-term implications for costs and the healthcare system.<br><br>Ultimately, the text is centered around practical and pragmatic healthcare delivery rather than ideological principles. Its emphasis is on ensuring patient safety, adapting to new circumstances, and addressing the immediate needs of the healthcare system in a crisis. As such, individuals from both liberal and conservative backgrounds might find aspects of the approach agreeable or might have concerns based on their broader views on healthcare policy and government intervention.	neutral
ACP	Autopsies	2016	The statement from the American College of Physicians (ACP) about encouraging autopsies while respecting cultural differences and not supporting financial remuneration for obtaining consent for autopsies doesn't align strictly with either a liberal or conservative viewpoint. It's more a matter of professional and ethical standards in the medical field. The emphasis on respecting cultural differences might be more aligned with liberal values, which often prioritize inclusivity and cultural sensitivity. On the other hand, the opposition to financial incentives for obtaining consent could be seen as a stance against market-driven healthcare, a view that can be found in both liberal and conservative ideologies, depending on the context. Overall, this statement seems to prioritize ethical medical practices and respect for diversity, transcending a simple liberal-conservative spectrum.	neutral
ACP	AComprehensive Policy Framework to Understand and Address Disparities and Discrimination in Health and Health Care	2020	The text presented aligns more closely with a liberal viewpoint. It emphasizes addressing healthcare disparities through comprehensive policies that consider race, ethnicity, religion, cultural identity, and other personal characteristics. The focus is on understanding and mitigating the impacts of racism, discrimination, and social determinants of health, such as poverty and education. It advocates for systemic changes in health care delivery, a diverse and inclusive healthcare workforce, and policies addressing broader social issues like criminal justice reform. The approach is holistic, emphasizing equity, inclusivity, and social justice, which are typically associated with liberal ideologies.	liberal

ACP	Understanding and Addressing Disparities and Discriminations Affecting the Health and Health Care of Persons and Populations at Highest Risk	2020	The text you've provided leans towards a liberal viewpoint. Here's why:<br><br>1. **Focus on Discrimination and Social Determinants of Health**: The emphasis on addressing discrimination, hate, and social drivers like poverty is a hallmark of liberal thought, which often prioritizes social justice and systemic change.<br><br>2. **Universal and Inclusive Health Coverage**: Advocating for universal health coverage, expanding Medicaid, and exploring coverage for unauthorized immigrants align with liberal policies focused on inclusivity and the belief that healthcare is a right.<br><br>3. **Acknowledgment of Historical Injustices**: Recognizing and committing to address the long history of racism and other injustices, particularly towards Indigenous people, reflects a liberal inclination to confront and correct historical and systemic inequalities.<br><br>4. **Cultural Competency and Linguistic Access**: The push for culturally and linguistically sensitive healthcare services is consistent with liberal values of diversity and inclusion.<br><br>5. **Environmental and Occupational Health**: Addressing environmental factors, climate change, and protecting the health of workers, especially during pandemics, are typically aligned with liberal priorities.<br><br>6. **Maternal Health and Research**: The focus on eliminating disparities in maternal mortality and advocating for more research into health disparities is in line with liberal priorities of evidence-based policy and healthcare equity.<br><br>Overall, the text's emphasis on addressing health disparities, discrimination, and social determinants of health, along with advocating for universal and culturally competent healthcare, reflects a liberal perspective on public policy and healthcare.	liberal
ACP	Understanding and Addressing Disparities and Discriminations in Law Enforcement and Criminal Justice Affecting the Health of At-Risk Persons and Populations		The text you've provided leans towards a liberal viewpoint. It focuses on systemic racism, discrimination, and violence within criminal justice and law enforcement, emphasizing the need for evidence-based solutions. The policies it supports include deploying social workers and mental health professionals in certain law enforcement situations, addressing socioeconomic factors related to crime, and implementing reforms to reduce racial and ethnic disparities in the criminal justice system. These points typically align with liberal ideals, which often prioritize social justice, equity, and systemic reforms to address societal issues.	liberal
ACP	Understanding and Addressing Disparities and Discriminations in Education and in the Physician Workforce		The text provided aligns more closely with a liberal viewpoint. It emphasizes the need for systemic change and equality in the healthcare system, focusing on addressing implicit biases, promoting diversity, equity, and inclusion in the medical workforce, and advocating for policies that ensure a healthcare system free of discrimination. These ideas are typically associated with progressive or liberal ideologies that prioritize social justice, equal opportunity, and comprehensive reform to address systemic inequalities. The text's support for federal and state intervention to enforce these changes further aligns with a liberal perspective, which often advocates for governmental action to achieve social goals and protect minority rights.	liberal

ACP	Recognizing Hate Crimes as a Public Health Issue	2017	The text you've provided generally aligns with a liberal viewpoint. Here's a breakdown of why:<br><br>1. **Opposition to Prejudice and Discrimination**: Liberal ideologies typically emphasize the importance of equality and non-discrimination for all individuals, regardless of their identity. The statement's strong stance against prejudice and discrimination based on a wide range of characteristics aligns with this principle.<br><br>2. **Hate Crimes as a Public Health Issue**: Recognizing hate crimes as a public health issue reflects a liberal perspective, which often considers the broader societal and health implications of discrimination and violence. It suggests a holistic approach to policy-making that includes social well-being.<br><br>3. **Opposition to Discriminatory Legislation**: Liberals generally oppose legislation that discriminates against individuals for any reason. The text's opposition to all legislation with discriminatory intent is consistent with a liberal commitment to equal rights and protection under the law for all citizens.<br><br>4. **Support for Anti-discrimination and Hate Crime Laws**: Advocating for laws that prevent discrimination and hate crimes is typical of liberal policies. Liberals often support proactive measures to protect minorities and marginalized groups from violence and discrimination.<br><br>5. **Support for Research and Data Collection**: The call for more research and statistics on hate crimes and their impact on public health is in line with a liberal viewpoint that favors informed policy decisions based on empirical evidence and the pursuit of knowledge to better understand and solve social issues.<br><br>While these positions align more closely with contemporary liberal thought, it's important to note that not all individuals or groups within a political ideology will agree on every issue. Additionally, perspectives and definitions of "liberal" and "conservative" can vary widely depending on geographical and cultural context.	liberal
ACP	Racism and Health in the United States		The text provided aligns with a liberal viewpoint. It strongly condemns racism, advocates for systemic change, and promotes policies aimed at achieving equity and justice, particularly within the healthcare system and law enforcement practices. The emphasis on addressing systemic racism, protecting marginalized communities, and calling for transparency and accountability in law enforcement are consistent with views typically associated with liberal ideology. This perspective focuses on social justice, equity, and the role of institutions in perpetuating or resolving disparities and injustices, which are key concerns in liberal thought.	liberal
ACP	Core Principles on Health Disparities and Disease Prevention	2022	The text suggests providing incentives to encourage individuals to take responsibility for their own health and engage in preventive care and health promotion activities. This viewpoint aligns with principles that can be appreciated by both liberal and conservative ideologies but for different reasons.<br><br>**Liberal Viewpoint**: Liberals typically support proactive health measures and might agree with incentivizing preventive care and personal responsibility in healthcare to improve public health outcomes and reduce overall healthcare costs. They often advocate for government involvement in healthcare to ensure everyone has access to preventive services and to address public health issues.<br><br>**Conservative Viewpoint**: Conservatives often emphasize personal responsibility and might support the idea of incentives as a way to encourage individuals to take charge of their own health, which aligns with their broader preference for limited government intervention. They might see this approach as a way to reduce reliance on government-funded healthcare systems and encourage more market-based solutions.<br><br>Overall, the statement can resonate with both ideologies, albeit for different underlying reasons. Liberals might focus on the collective benefits and equality of access, while conservatives might appreciate the emphasis on individual responsibility and reduced government involvement.	neutral
ACP	LGBT Health Disparities	2015	The text generally aligns with a liberal viewpoint. Liberal perspectives often emphasize inclusivity, anti-discrimination, and support for minority groups, including the LGBTQ+ community. The statements from the American College of Physicians advocate for inclusive and nondiscriminatory policies in medical settings, support for transgender health care, the recognition of diverse family structures, visitation rights regardless of sexual orientation or gender identity, civil marriage rights for same-sex couples, and opposition to conversion therapy. These positions align with liberal values that prioritize equality, civil rights, and the recognition and support of diverse identities and lifestyles. Conservative viewpoints, on the other hand, might emphasize traditional values and may be less supportive of some of these policies, particularly those related to transgender rights, same-sex marriage, and broader definitions of family.	liberal

ACP	Social Determinants of Health	2018	The text you've provided outlines policy positions from the American College of Physicians focused on addressing socioeconomic inequalities in health, integrating understanding of social determinants into medical education, encouraging collaborative care models, supporting adequate funding for health initiatives, promoting research into social determinants of health, and integrating health considerations into various policies and practices. These stances generally align with a liberal viewpoint for several reasons:<br><br>1. **Emphasis on Social Determinants of Health**: The focus on socioeconomic factors and social determinants of health as key elements in health disparities aligns with liberal perspectives that stress the role of social structures in individual outcomes and advocate for systemic change.<br><br>2. **Support for Public Interventions and Funding**: The call for increased public policy interventions, funding for federal and local agencies, and the integration of health into all policies reflects a belief in the government's role in addressing societal issues, which is more commonly associated with liberal ideologies.<br><br>3. **Team-Based, Collaborative Care Models**: Encouraging interprofessional communication and collaborative models aligns with a more community-oriented and holistic approach to healthcare, which is often more emphasized in liberal health policy.<br><br>4. **Research and Education**: Advocating for increased research into social determinants and integrating this knowledge into medical education suggests a progressive stance on adapting and evolving healthcare practices based on emerging evidence and understanding, which is a hallmark of more liberal thinking.<br><br>5. **Health Equity and Inclusivity**: The focus on reducing health disparities and encouraging health equity is a key component of liberal health policy, aiming to ensure that all individuals, regardless of their socioeconomic status, have access to the care and opportunities they need for good health.<br><br>While these stances predominantly align with liberal viewpoints, it's important to note that health and healthcare are complex issues that can transcend traditional political boundaries. Some conservatives might also support certain aspects of these policies, especially those that could lead to more efficient care delivery, improved health outcomes, and potentially reduced long-term costs. However, the overall emphasis on systemic change, social determinants, and government intervention is more typically associated with liberal ideologies.	liberal
ACP	Supporting the Health and Wellbeing of Indigenous Communities	2022	The text from the ACP (presumably a policy-oriented organization, though not explicitly identified here) aligns more with a liberal viewpoint. The focus on community-driven public policy, addressing historical injustices, prioritizing health and wellness promotion, and advocating for comprehensive, integrated, and culturally appropriate care are typical of liberal perspectives, which often emphasize social justice, equity, and community-based solutions. The emphasis on collaboration with Indigenous peoples, respect for traditional values, and the call for increased funding and resources for health care and infrastructure also align with liberal priorities of inclusivity and government intervention to address disparities and promote public welfare.<br><br>Moreover, the specific support for policies that address the social determinants of health, such as poverty, education, and environmental justice, reflects a holistic approach to health care and community well-being commonly found in liberal ideologies. The advocacy for evidence-based and community-driven approaches to addressing health disparities, mental health, substance use disorders, and violence in Indigenous communities also indicates a preference for policies that are informed by research and tailored to the needs of specific populations, aligning with liberal values of inclusivity and evidence-based policymaking.<br><br>Overall, the emphasis on equity, collaboration, respect for cultural values, and comprehensive, community-focused solutions is more characteristic of a liberal viewpoint, which typically prioritizes social justice, equity, and government intervention to address systemic issues and promote the welfare of marginalized communities.	liberal

ACP	Decreasing Barriers to Prescribe Buprenorphine for Opioid Use Disorder	2022	The statement about the American College of Physicians (ACP) supporting the elimination of the waiver requirement for physicians to administer, dispense, and prescribe buprenorphine for opioid use disorder treatment aligns more closely with a liberal viewpoint. Generally, liberal perspectives tend to favor public health measures that emphasize treatment and access to care for individuals with addiction, viewing it as a medical issue rather than strictly a legal one. They often support policies aimed at reducing barriers to treatment, believing that increasing access to care like medication-assisted treatment (MAT) for opioid use disorder can help address the public health crisis.<br><br>Conservative viewpoints might vary on this issue. Some conservatives might support strict regulatory approaches to substance use and prioritize law enforcement measures. However, it's important to note that views on drug policy have become less strictly divided along partisan lines in recent years, with some conservatives also supporting measures to improve treatment access as part of a broader approach to addressing the opioid crisis.<br><br>In any case, it's critical to understand that these are generalizations and individuals' views on such policies can vary widely within each political ideology.	liberal
ACP	Harm Reduction Strategies for Substance Use Disorders including Overdose Prevention Sites and Fentanyl Test Strips		The text you've provided outlines positions from the American College of Physicians (ACP) regarding harm reduction strategies for drug use. These positions generally align with what is typically considered a liberal viewpoint. Here's why:<br><br>1. **Support for Harm Reduction**: Liberal viewpoints often emphasize harm reduction strategies over punitive measures when it comes to drug use. The ACP's support for evidence-based harm reduction, safe use protocols, and connection to medical care reflects a focus on public health and safety over criminalization.<br><br>2. **Federal Funding for Syringe Support Programs**: Liberals generally support the use of federal funds for health initiatives, including those that help marginalized communities, like people who use drugs. Syringe support programs are seen as a way to reduce harm and prevent the spread of infectious diseases.<br><br>3. **Decriminalization of Drug Equipment**: Decriminalization is a common liberal stance, aiming to reduce the negative societal and individual impacts of drug laws. By recommending the decriminalization of items like syringe support program equipment and drug checking supplies, the ACP is aligning with a viewpoint that seeks to reduce the stigma and legal consequences associated with drug use.<br><br>4. **Support for Overdose Prevention Sites (OPS)**: OPS are a controversial topic, with liberal perspectives typically in favor due to their potential to save lives and provide access to health services. The call for legal protection for OPS operators and engagement with the community also reflects a liberal inclination towards inclusive, community-based approaches.<br><br>5. **Focus on Public Health and Safety**: The overall focus on public health, safety, and the well-being of people who use drugs reflects a liberal viewpoint that prioritizes individual health and societal benefit over punitive or moralistic approaches.<br><br>While these positions align more closely with liberal viewpoints, it's important to note that there's a range of perspectives within both liberal and conservative camps, and some conservatives may support certain harm reduction strategies as part of a broader approach to drug policy.	liberal

ACP	Prescription Drug Abuse	2023	The text provided outlines a policy stance from the American College of Physicians (ACP) on substance abuse, prescription drug abuse, and related medical practices. It doesn't neatly align with traditional liberal or conservative viewpoints as it touches on various aspects that could resonate with both sides. Here's a breakdown:<br><br>**Liberal-leaning aspects:**<br>- **Support for comprehensive education and prevention programs:** Generally, a focus on education and prevention over punitive measures is more aligned with liberal ideology.<br>- **Support for research on addiction and its treatment:** Emphasizing medical and scientific research is often associated with liberal stances.<br>- **Support for a national Prescription Drug Monitoring Program (PDMP):** Implementing national programs for health and safety can be seen as more government intervention, which is typically more accepted by liberal viewpoints.<br><br>**Conservative-leaning aspects:**<br>- **Balanced approach to controlled substances:** The call for a balanced approach that allows for medical treatment while also addressing drug abuse might resonate with conservatives who value personal responsibility and limited regulation in medical practice.<br>- **Recognition of individual clinical discretion:** The acknowledgment that maximum dosage and therapy duration limitations are not universally applicable might appeal to conservatives' preference for individualized, not government-mandated, healthcare solutions.<br>- **Concerns about administrative burdens:** The stance against imposing excessive administrative burdens aligns with conservative principles favoring smaller government and less regulation.<br><br>**Neutral/Both:**<br>- **Efforts to educate about the dangers of prescription drugs:** This could be seen as a non-partisan issue, as both sides generally agree on the importance of public health and safety.<br>- **Patient consent and financial aspects of drug testing:** Both sides might see merit in protecting patient rights and ensuring fair financial responsibility.<br><br>Overall, the ACP's position seems to strive for a balanced approach, blending elements of education, regulation, and individual discretion. It doesn't strictly fit into a liberal or conservative box but instead addresses the complexity of the issue with a multifaceted strategy. This reflects a more pragmatic and nuanced approach that's focused on specific outcomes rather than ideological purity.	neutral
ACP	Prevention and Treatment of Substance Use Disorders	2017	The recommendations presented align more closely with a liberal viewpoint. They focus on treating substance use disorders as medical issues rather than criminal ones, advocating for treatment over incarceration, decriminalization, and comprehensive public health strategies. This approach emphasizes the health aspects of substance use, seeking to reduce stigma and improve access to care. Such perspectives are generally associated with liberal ideologies, which often prioritize public health, harm reduction, and the reformation of criminal justice policies related to drug offenses. Conservative viewpoints might focus more on law enforcement, criminalization, and personal responsibility in addressing substance issues. However, it's important to note that views on substance use disorders can vary widely within both liberal and conservative circles, and there's a growing recognition across the political spectrum of the need for effective treatment and health-based approaches.	liberal

ACP	Policy Recommendations to Promote Prescription Drug Competition	2020	The text outlines policy positions on drug development, market exclusivity, patent systems, and advertising practices. Here's how they might align with liberal or conservative viewpoints:<br><br>1. **Support for legislative reforms to the Orphan Drug Act (ODA):**<br>- **Liberal**: Generally, liberals might favor this as it aims to increase innovation in rare disease drug development, possibly improving access to healthcare and supporting public health initiatives.<br>- **Conservative**: Conservatives might be cautious about reforms that could impose more regulations or hinder free-market principles but could support reforms that streamline development and reduce unnecessary government intervention.<br><br>2. **Reducing the period of data and market exclusivity for biologic drugs from 12 years to 7 years:**<br>- **Liberal**: Liberals typically support such measures as they can lead to increased competition and lower drug prices, making healthcare more accessible.<br>- **Conservative**: Conservatives might oppose reducing exclusivity periods as they often prioritize property rights and the incentives for innovation that longer exclusivity can provide.<br><br>3. **Opposition to anticompetitive pay-for-delay arrangements:**<br>- **Liberal**: Generally supported by liberals as it aims to combat market manipulation and promote access to lower-cost drugs.<br>- **Conservative**: While conservatives support free-market competition, some might see pay-for-delay as a legitimate business strategy, although others could oppose it if viewed as hindering true market competition.<br><br>4. **Elimination of tax deductions for direct-to-consumer product claim advertisements:**<br>- **Liberal**: Likely supported by liberals as it's a move against big pharma's influence on consumer behavior and could reduce medication overuse.<br>- **Conservative**: Conservatives might oppose this on grounds of free speech and opposition to increasing taxes or removing tax deductions.<br><br>Overall, the positions largely align with a **liberal viewpoint**, emphasizing reform, market competition, and access to healthcare, though aspects of these policies could be supported or contested by individuals from either side depending on their specific beliefs about government intervention, market regulation, and healthcare rights.	liberal
ACP	Medicare Prescription Drug Coverage	2022	The text presents a series of policy positions regarding Medicare Part D, focusing on sustainable financing, prioritization of low-income beneficiaries, cost control, support for generic and biosimilar drugs, opposition to restrictive formularies, and various consumer protections. These positions generally align with a liberal viewpoint, emphasizing:<br><br>1. **Sustainable Financing**: Advocating for stable and predictable funding sources for Medicare Part D without reducing other benefits, which reflects a liberal preference for comprehensive and well-funded social programs.<br><br>2. **Priority for Low-Income Beneficiaries**: Prioritizing assistance for low-income individuals is a hallmark of liberal policies, focusing on equity and access to healthcare.<br><br>3. **Cost Control Measures**: Supporting measures to control prescription drug costs and promote generics and biosimilars aligns with liberal views on healthcare affordability and access.<br><br>4. **Consumer Protections**: Advocating for regulations and disclosures, and opposing policies that might restrict access or increase costs for consumers, are typical of liberal positions emphasizing patient rights and protections.<br><br>5. **Opposition to Over-The-Counter Switch and Privatization**: Opposing the switch to over-the-counter status for certain drugs and the conversion of Medicare to a defined contribution program are stances that align with liberal priorities for maintaining comprehensive, regulated healthcare coverage.<br><br>In summary, the text's emphasis on sustainable funding, prioritization of vulnerable populations, comprehensive coverage, cost control, and consumer protections is more aligned with liberal ideologies, which typically advocate for robust, regulated, and equitable healthcare systems.	liberal

ACP	Improving FDA Regulation of Prescription Drugs	2022	The text suggests various reforms to strengthen the FDA's ability to approve, monitor, and regulate prescription drugs. It leans more towards a liberal viewpoint for several reasons:<br><br>1. **Increased Government Role and Funding**: Calling for increased funding and an expanded role for the FDA reflects a liberal perspective that favors a strong regulatory government to ensure public safety and welfare.<br>2. **Regulation of Drugs Manufactured Overseas**: Advocating for greater control over international manufacturing aligns with a desire for comprehensive regulation, typical of liberal policies.<br>3. **Emphasis on Safety and Transparency**: Prioritizing drug safety, efficacy, and public reporting of clinical trials suggests a precautionary approach and a demand for transparency, often associated with liberal values.<br>4. **Limitations on Market Practices**: Proposals to prohibit bundling of drugs and limit direct-to-consumer advertising are in line with liberal ideals of protecting consumers from potentially predatory market practices.<br>5. **Innovation in Approval Processes**: Encouraging the use of advanced scientific tools and a more thorough review process reflects a trust in scientific expertise and a proactive approach to public health, common in liberal thought.<br><br>While some conservatives might support aspects of this approach, especially those related to improving the safety and efficacy of drugs, the overall expansion of regulatory authority and government involvement generally aligns more with liberal viewpoints. It's worth noting that political perspectives can vary widely, and these classifications might not apply uniformly to all individuals or contexts.	probably liberal
ACP	FDA Regulation of Drugs and Medical Devices	2019	The statement reflects a viewpoint that emphasizes the importance of maintaining rigorous standards and oversight in drug and medical device approval, specifically supporting the authority of the FDA to demand thorough evaluations based on scientific and medical evidence. This perspective aligns more closely with a liberal viewpoint, which generally advocates for stronger regulatory measures to ensure public safety and uphold scientific standards. Conservatives might be more inclined to support less regulation and more market freedom, potentially viewing such strong regulatory support as restrictive to industry innovation and growth. However, it's essential to note that views on specific issues like FDA authority can vary within each ideological group and may not strictly adhere to the general liberal-conservative spectrum.	liberal
ACP	Removal of Drugs by the Food and Drug Administration (FDA)	2016	The text from the American College of Physicians (ACP) appears to advocate for a balanced approach to drug regulation, emphasizing the importance of communication with the medical profession and considering the experiences of physicians before withdrawing drugs from the market. This stance doesn't neatly align with traditional liberal or conservative viewpoints as it combines elements that could be appealing to both sides:<br><br>1. **Conservative aspects**: The text might resonate with conservative values by emphasizing caution and respect for the established practices and opinions of professionals in the field. Conservatives often prioritize individual expertise and caution in regulatory changes, advocating for less government intervention in professional and business practices.<br><br>2. **Liberal aspects**: On the other hand, the emphasis on ensuring drugs' safety and efficacy before they are marketed or continued to be available might align with more liberal views, which often advocate for stronger regulation and oversight to protect public health and safety.<br><br>In essence, the ACP's statement reflects a professional, rather than a politically ideological, perspective. It prioritizes the safety and effectiveness of medical treatments and the practical experiences of medical professionals while ensuring that any regulatory actions are taken with due consideration and not hastily. This approach is more about effective and responsible policy-making in healthcare than about liberal or conservative ideology.	neutral
ACP	Office Compounding of Allergen Extracts and Other Drugs	2016	The text you've provided is from a resolution supporting the 2008 USP Chapter 797 sterile compounding rules, particularly as they apply to allergen extracts. It also encourages contacting the FDA to ensure regulations prioritize patient safety while maintaining access to specialty care through in-office compounding. This text doesn't inherently align with a strictly liberal or conservative viewpoint. Instead, it focuses on healthcare policy, patient safety, and access to care.<br><br>Both liberals and conservatives might have various opinions on such regulations. Liberals might support the emphasis on patient safety and ensuring access to healthcare, while conservatives might appreciate the call for balanced regulation that doesn't overly restrict medical practices or the market. Ultimately, the text seems to aim for a balanced approach, prioritizing patient safety and access without explicitly endorsing a partisan viewpoint.	neutral

ACP	Statement of the American Pharmaceutical Association (APA) and ASIM on Prescriptions	2016	The text you've provided, outlining guidelines for pharmacists regarding prescription labels and communication with patients, doesn't inherently align with a liberal or conservative viewpoint. It is a set of professional guidelines aimed at ensuring patient safety and effective healthcare practices. These guidelines focus on practical, nonpartisan issues like clear communication, patient understanding, and cooperation between medical professionals. Both liberal and conservative ideologies generally support the overarching goals of public health and safety, although they might differ in their approaches to healthcare policy. However, this specific text is neutral and focuses solely on professional standards and practices within the pharmacy field.	neutral
ACP	Stemming the Escalating Cost of Prescription Drugs	2016	The text outlines a series of policy recommendations from the American College of Physicians (ACP) concerning pharmaceutical pricing, drug cost transparency, market competition, and patient access to medications. It's difficult to categorize the entire text as strictly liberal or conservative because it touches on aspects that could be supported by both sides, depending on the context and specific policy goals.<br><br>1. **Transparency in pricing and costs**:<br>- **Liberal Viewpoint**: Generally aligns with liberal priorities for greater transparency and regulation in the healthcare industry to protect consumers and control costs.<br>- **Conservative Viewpoint**: Conservatives might support transparency as a means to promote market efficiency and informed consumer choice, although they might be less enthusiastic about aggressive regulatory interventions.<br><br>2. **Elimination of restrictions on QALYs**:<br>- **Liberal Viewpoint**: Could be seen as supporting a more comprehensive and holistic approach to healthcare outcomes, aligning with a liberal emphasis on healthcare quality.<br>- **Conservative Viewpoint**: Might be supported if seen as removing unnecessary regulations and allowing for more market-driven solutions in healthcare research.<br><br>3. **Approaches to address medication costs**:<br>- **Liberal Viewpoint**: Proposals like negotiating drug prices and reimporting drugs align with liberal calls for stronger government intervention to control healthcare costs.<br>- **Conservative Viewpoint**: While traditionally less supportive of government negotiation in markets, some conservatives might support aspects like increasing competition as a way to reduce costs.<br><br>4. **Opposition to extending market exclusivities**:<br>- **Liberal Viewpoint**: Typically, liberals would strongly support this stance as it promotes competition and aims to reduce drug prices.<br>- **Conservative Viewpoint**: Conservatives who prioritize free-market solutions might also agree, as it could be seen as reducing artificial market barriers, though some might support protections for pharmaceutical companies' intellectual property as an incentive for innovation.<br><br>5. **Support for research into novel approaches**:<br>- **Liberal Viewpoint**: Supporting research and considering value-based decision-making is in line with liberal ideals of evidence-based policymaking and ensuring healthcare affordability.<br>- **Conservative Viewpoint**: Some aspects, like indication-specific pricing, could be seen as market-friendly and might gain support from conservatives interested in innovative, market-based solutions.<br><br>6. **Ensuring affordable access to specialty drugs**:<br>- **Liberal Viewpoint**: Emphasizes protecting vulnerable populations and ensuring access to necessary medications, a core liberal value.<br>- **Conservative Viewpoint**: While conservatives might support the idea of market-based health plans, they might be less inclined to support mandates on how private plans set their cost-sharing structures.<br><br>7. **Biosimilar drug policy**:<br>- **Liberal Viewpoint**: Encouraging the integration of biosimilars aligns with liberal goals of increasing competition and reducing costs.<br>- **Conservative Viewpoint**: Ensuring limited patient confusion and promoting safe use could be seen as a conservative approach to maintaining high-quality standards in healthcare.	probably liberal

			Overall, while some elements of the ACP's positions might find support across the political spectrum, the emphasis on regulation, transparency, and ensuring access to affordable healthcare align more closely with traditional liberal viewpoints. However, the focus on market competition and innovation in some areas could also resonate with conservative values. It's a nuanced mixture of policies that doesn't strictly fit into one ideological box.	

ACP	Prescription Drug Recalls	2020	The text you've provided outlines various proposals to enhance the safety and regulation of pharmaceutical products. Here's a breakdown of how these points might align with traditional liberal or conservative viewpoints:<br><br>1. **National recall notification network**: This idea leans towards a liberal viewpoint as it advocates for a more robust governmental role in ensuring public health and safety. Liberals generally support increased government intervention, especially in matters concerning public welfare.<br><br>2. **Thorough electronic tracking**: This proposal could be seen as technocratic and could garner support from both sides. Conservatives might support it as a means of ensuring free market safety and efficiency, while liberals might see it as a necessary regulation to protect consumers.<br><br>3. **Increased regulatory authority**: This point strongly aligns with a liberal viewpoint. Liberals often advocate for more government oversight and regulation, particularly in industries that have a significant impact on public health and safety, like pharmaceuticals.<br><br>4. **Process for expedited formulary exceptions**: This proposal might be more mixed. Conservatives typically support market-based solutions and might argue this interferes with the market, while liberals might support it as a means of ensuring access to necessary medications, especially during crises.<br><br>In general, the text promotes increased regulation and oversight, principles more commonly associated with liberal ideology. However, the specific context of healthcare and pharmaceuticals can blur traditional ideological lines, with both sides recognizing the need for some level of regulation to ensure safety and efficiency in the market.	liberal
ACP	Policy Recommendations for Pharmacy Benefit Managers to Stem the Escalating Costs of Prescription Drugs	2019	The positions outlined in the text generally align with a liberal viewpoint. Here's why:<br><br>1. **Regulation of Pharmacy Benefit Managers (PBMs)**: Advocating for improved transparency, standards, and regulation of PBMs, as well as a ban on gag clauses, reflects a liberal perspective that favors regulatory measures to protect consumers' interests and promote transparency in healthcare pricing.<br><br>2. **Access to Information**: Supporting the availability of accurate and actionable information on prescription medication pricing for both physicians and patients aligns with a liberal emphasis on informed decision-making and consumer rights. This viewpoint typically favors initiatives that enhance transparency and accessibility in healthcare.<br><br>3. **Reporting and Public Disclosure**: Urging health plans, PBMs, and manufacturers to report pricing details and make them publicly available, albeit with considerations for confidentiality and market impact, also leans liberal. It reflects a belief in the government's role in ensuring transparency and accountability in the healthcare market.<br><br>In summary, the support for increased regulation, transparency, and consumer protection in the context of healthcare and prescription medication pricing is more characteristic of liberal ideology, which often emphasizes the role of government in regulating markets and protecting consumers.	liberal

ACP	Policy Recommendations for Public Health Plans to Stem the Escalating Costs of Prescription Drugs:	2019	The text you've provided outlines a series of positions from the American College of Physicians (ACP) regarding modifications to Medicare Part D and other healthcare policies, particularly focusing on reducing costs for beneficiaries and the government. These positions generally align with a liberal viewpoint for several reasons:<br><br>1. **Advocacy for Lower Costs and Access**: The support for reducing or eliminating costs for low-income beneficiaries, implementing out-of-pocket spending caps, and adopting negotiation models to lower prescription drug prices reflects a concern for affordability and access to healthcare. These are typically liberal priorities, focusing on expanding access and reducing barriers to healthcare.<br><br>2. **Government Intervention and Regulation**: The endorsement of more direct government intervention in drug pricing (such as allowing the Secretary of Health and Human Services to negotiate drug prices and supporting a public Medicare Part D plan) is more in line with liberal ideologies, which often favor a stronger regulatory role for the government in healthcare to ensure fairness and protect consumers.<br><br>3. **Comprehensive Approach to Healthcare Reform**: The call for studies of payment models and efforts to minimize financial impacts on government programs indicates a holistic approach to healthcare reform. This tends to align with liberal perspectives that advocate for systematic changes to improve efficiency and equity in healthcare.<br><br>4. **Support for Generic and Biosimilar Drugs**: Encouraging the use of lower-cost alternatives to brand-name drugs can be seen as a way to make healthcare more affordable and accessible, aligning with liberal values of equity and support for underprivileged populations.<br><br>However, it's important to note that healthcare policy can transcend traditional liberal-conservative divides, and there may be conservatives who also support some of these measures, especially if they are seen as ways to reduce government spending in the long run or improve market efficiencies. Additionally, positions on healthcare are often complex and multifaceted, and individuals or groups might hold a mix of liberal and conservative views depending on the specific issue.	liberal
ACP	Direct-to-Consumer Prescription Drug Advertising	2019	The positions presented in the text generally align with a liberal viewpoint. Here's a breakdown of how each aligns:<br><br>1. **Position 1 - Opposition to Direct-to-Consumer (DTC) Advertising**: This stance is typically associated with a liberal viewpoint, emphasizing the protection of the patient-physician relationship and concern over commercial interests potentially misleading or confusing patients. The focus is on the well-being and informed consent of the patient rather than the marketing rights of pharmaceutical companies.<br><br>2. **Position 2 - Stronger Regulation and Oversight by the FDA**: Advocating for increased government intervention and regulation in the healthcare and pharmaceutical sectors is a hallmark of liberal policy. The call for Congress to empower the FDA with more authority, resources, and regulatory tools to control and monitor DTC advertising reflects a belief in the positive role of government in safeguarding public health and ensuring truthful and helpful information is disseminated.<br><br>3. **Position 3 - Support for Patient Education and Collaboration**: While this position is more moderate and could garner bipartisan support, its emphasis on evaluating and defining the impact of DTC ads, as well as ensuring complete and non-confusing health information, aligns with a liberal approach to healthcare. It supports an active role for government and professional bodies in managing and guiding healthcare information.<br><br>In summary, these positions focus on increased regulation, skepticism of commercial interests in healthcare, and a proactive government role in protecting patients—principles generally associated with liberal ideology.	liberal
ACP	Prescription Drug Importation as a Policy Option to Lower the Cost of Medications in the U.S.	2019	The text primarily aligns with a liberal viewpoint. It emphasizes the importance of affordable healthcare and prescription drugs, a common concern in liberal policies, focusing on safety and cost reduction while ensuring quality. It advocates for regulatory measures, oversight, and pilot programs, reflecting a trust in government intervention to solve systemic issues. The text also suggests expanding public health insurance coverage and scrutinizing pharmaceutical companies' practices, which are typically liberal stances concerned with public welfare and cautious of corporate influence. However, it's important to note that some aspects, like encouraging competition and drug importation, could be seen as market-friendly approaches, which might resonate with some conservative principles focused on market solutions. The detailed recommendations for safe drug importation and a balanced approach to cost, safety, and quality reflect a nuanced perspective that doesn't strictly adhere to ideological extremes.	liberal

ACP	Pharmacy Labeling	2016	The statement you've provided calls for a policy where both the generic and brand names of drugs are included on labels to reduce patient confusion and potential therapeutic errors. This policy doesn't strictly align with either a liberal or conservative viewpoint as defined in the typical American political context. Instead, it seems focused on patient safety and clarity in healthcare, which are concerns that can cross political lines.<br><br>In the U.S., liberal viewpoints generally emphasize the need for regulatory measures to protect consumers and public health, while conservative viewpoints often prioritize free market solutions and minimal regulation. However, both sides might support or oppose this policy for various reasons:<br><br>- **Liberals might support it** because it promotes consumer protection and informed decision-making in healthcare.<br>- **Conservatives might support it** as it could also be seen as a way to promote transparency and empower consumer choice in the market.<br><br>Ultimately, this policy is more about practical healthcare administration and patient safety than about broader ideological divides. It's best understood in the context of healthcare policy debates rather than the liberal-conservative spectrum.	neutral
ACP	Quality Assurance and Labeling	2016	The statement from the ACP (American College of Physicians) about requiring pharmaceutical manufacturers to perform ongoing quality assurance studies of their products through federal regulations or laws is generally aligned with a liberal viewpoint. This alignment is due to the advocacy for increased government oversight and regulation in the healthcare sector, which is more commonly associated with liberal ideologies. Liberals tend to support the idea that government intervention is necessary to protect consumers, ensure public health, and maintain high standards in industries like pharmaceuticals. They argue that without regulation, companies might prioritize profits over safety and efficacy.<br><br>On the other hand, conservative viewpoints typically emphasize less government intervention in business and more market freedom. Conservatives might argue that too much regulation can stifle innovation, increase costs, and burden businesses, potentially leading to higher prices for consumers and less economic efficiency.<br><br>It's important to note, however, that while these general trends exist, there can be a wide range of opinions within each political ideology, and some conservatives might support certain regulations for public safety reasons, just as some liberals might oppose certain regulations they see as overreaching.	liberal
ACP	Drug Formularies and Pharmacy Benefit Management	2022	The positions outlined in the formularies from the American College of Physicians (ACP) tend to align more closely with a liberal viewpoint, primarily focusing on patient care, physician autonomy, and the effective, safe, and equitable delivery of healthcare. Here's how the points generally match liberal principles:<br><br>1. **Emphasis on Patient Care Over Cost**: Liberal viewpoints often prioritize individual well-being and equitable access to resources over strict cost control. Opposing formularies developed primarily to control costs aligns with this view.<br>2. **Decision-Making Based on Effectiveness and Safety**: This reflects a liberal inclination towards evidence-based policy and healthcare, emphasizing the quality and outcomes of care rather than just efficiency or market-driven choices.<br>3. **Physician Autonomy and Ethical Considerations**: Liberal policies typically support professional autonomy and ethical considerations in healthcare, advocating for decisions that prioritize patient well-being over financial incentives.<br>4. **Transparency and Patient Education**: The emphasis on informing patients and involving them in understanding their care options reflects a liberal focus on individual rights and informed consent.<br>5. **Opposition to Conflicts of Interest**: This is in line with a liberal stance on ensuring that healthcare decisions are made in the best interest of patients, without undue influence from financial or other external pressures.<br>6. **Regulatory Oversight and Government Involvement**: Recommendations for government and industry studies, as well as the development of report cards and technology to improve formularies, align with a liberal trust in the role of regulatory bodies to oversee and improve healthcare systems.<br><br>Conservative viewpoints, on the other hand, might emphasize more market-driven approaches, cost containment, and less regulatory oversight. While conservatives do care about patient well-being and effective treatments, they might prioritize different mechanisms to achieve these goals, such as competition and free-market solutions.<br><br>It's important to note, however, that these distinctions aren't absolute, and there can be significant overlap in the desire from both sides to ensure effective, safe, and patient-centered healthcare. The delineation often comes down to the methods and mechanisms each side believes are most effective in achieving these goals.	liberal

ACP	Pharmacy Benefit Management	2023	The text you've provided outlines policy positions from the American College of Physicians (ACP) regarding Pharmacy Benefit Managers (PBMs). Here's a breakdown of how these points align with liberal or conservative viewpoints:<br><br>1. **Support for government regulation and industry self-regulation of PBMs**: This tends to align more with a liberal viewpoint, as liberals generally favor more government intervention and regulation in healthcare to ensure fairness and transparency.<br><br>2. **Support for the disclosure of financial relationships**: This point could align with both liberal and conservative viewpoints. Liberals might support it as a means of ensuring transparency and accountability, while conservatives might see it as a way to promote free market principles by providing more information to consumers.<br><br>3. **Requiring PBM requests to alter medication regimes to be based on peer-reviewed data**: This is a quality and safety issue that doesn't neatly fall into a liberal or conservative category. Both sides might agree on the importance of evidence-based practice, although the mechanism for enforcing it (government oversight vs. professional self-regulation) might be a point of contention.<br><br>4. **Requiring PBMs to provide medication history to physicians with patient consent**: This aligns with principles of patient privacy and informed consent, which are generally universal healthcare values. However, the emphasis on patient control over their information might be more appealing to conservatives who prioritize individual rights and freedoms.<br><br>Overall, these points reflect a nuanced approach to healthcare policy that incorporates elements of both liberal (e.g., support for regulation) and conservative (e.g., emphasis on transparency and individual rights) ideologies. They focus on practical measures to improve transparency, quality, and safety in healthcare, which are concerns that cross ideological lines. The ACP's stance seems to prioritize patient care and the integrity of the healthcare system over a strictly ideological approach.	neutral
ACP	Internet Prescribing	2022	The statement you provided from the American College of Physicians (ACP) emphasizes the importance of maintaining a direct physician-patient relationship and suggests that internet prescribing should only be used within the context of an established relationship. This viewpoint doesn't align strictly with traditional liberal or conservative ideologies, as it primarily focuses on medical ethics and patient care standards.<br><br>Both liberal and conservative viewpoints might find aspects of this statement to agree with. Liberals might support the emphasis on the physician-patient relationship and patient rights, while conservatives might agree with the caution around new technologies like internet prescribing. In general, this statement seems to prioritize professional medical ethics and patient care over political ideology.	neutral
ACP	Misuse of DEA Numbers	2022	The statement from the American College of Physicians (ACP) supporting the American Medical Association's (AMA) policy to eliminate the requirement for physicians' DEA numbers on prescriptions for non-controlled drugs seems primarily focused on practical and administrative concerns rather than ideological ones. However, the stance can be interpreted through both liberal and conservative lenses:<br><br>**Liberal Viewpoint:**<br>- **Reducing Bureaucracy:** Liberals might favor this position as it seeks to reduce administrative burdens, potentially making healthcare more efficient and accessible.<br>- **Privacy Concerns:** They might also support the notion of protecting confidentiality, which aligns with broader concerns about personal privacy and rights.<br><br>**Conservative Viewpoint:**<br>- **Reducing Government Regulation:** Conservatives might agree with the idea of eliminating what they could see as unnecessary regulatory requirements, aligning with a preference for smaller government and less intervention in professional practices.<br>- **Autonomy in Healthcare:** They might also view this policy as a way to grant more autonomy to physicians and reduce the influence of third parties like pharmacies and insurance companies.<br><br>Both sides might argue that their approach ultimately benefits patient care and the efficiency of the healthcare system. However, this statement doesn't neatly fit into a purely liberal or conservative viewpoint; it's more about practicality and the specifics of medical administration rather than ideological principles.	neutral

ACP	Mail Order Pharmacy Confidentiality	2016	The statement "ACP opposes the use of confidential prescribing data by third parties to directly contact patients for any purposes" doesn't inherently align with a traditional liberal or conservative viewpoint as it focuses on patient privacy and the ethical use of medical data. Both liberals and conservatives might have views on this issue, but they would likely be informed by broader considerations about patient rights, privacy, and the role of government and corporations in healthcare.<br><br>Liberals might support the statement as it aligns with protecting individual privacy rights and potentially limiting corporate overreach. Conservatives might also agree, seeing it as a defense of personal liberties and a stance against potential government or corporate intrusion. However, perspectives can vary widely within each ideology, depending on how the issue intersects with other beliefs about regulation, healthcare, and privacy. It's more a matter of specific policy perspective than a broad ideological viewpoint.	neutral
ACP	Negative Formularies	2023	The statement about encouraging the deletion of drugs from Negative Drug Formularies in favor of FDA A-rated generic substitutes doesn't inherently align with a traditional liberal or conservative viewpoint. It's more of a healthcare policy issue focused on drug regulation and cost management. Here's how both sides might view it:<br><br>- **Liberal Viewpoint**: Liberals might support this resolution as it could lead to reduced healthcare costs and increased access to medications for people. They generally advocate for measures that aim to make healthcare more affordable and accessible.<br><br>- **Conservative Viewpoint**: Conservatives might also support this resolution, as it can be seen as a market-driven approach to healthcare, reducing regulations and encouraging competition among drug manufacturers. This could lead to lower prices and more efficient healthcare spending, aligning with conservative values of free-market solutions and reduced government intervention.<br><br>However, the actual stance can vary widely depending on specific beliefs about pharmaceutical regulation, healthcare economics, and the role of government in healthcare. Additionally, both liberals and conservatives might have concerns about the safety and efficacy of generic drugs, or the implications for pharmaceutical innovation and industry. Therefore, this statement doesn't neatly align with one political ideology over another.	neutral
ACP	Proper Use of Accepted Drugs	2016	The text expresses a viewpoint advocating for physician autonomy and clinical judgment in determining the proper usage of approved drugs, rather than strict legislative or administrative controls. This perspective aligns more with principles typically associated with a liberal viewpoint, which often emphasizes individual professional judgment and less restrictive government intervention in professional practices. However, some aspects of this stance could be embraced by conservatives who support limited government regulation in professional affairs. Ultimately, the alignment might vary based on the broader context of healthcare policies and the specific beliefs of individuals within each ideological group.	neutral
ACP	Physician Drug Dispensing	2019	The text from the American College of Physicians (ACP) regarding patient rights and physician dispensing of medications doesn't align neatly with a traditional liberal or conservative viewpoint. It touches on various principles that could be appealing to both sides:<br><br>1. **Patient Rights and Choice**: The emphasis on informing patients of their rights and allowing them to choose where to fill their prescriptions aligns with liberal ideals of individual autonomy and consumer rights. This aspect also resonates with conservative values of personal freedom and market choice.<br><br>2. **Physician Dispensing**: The allowance for physicians to dispense medications, especially when beneficial to the patient, could be seen as a conservative endorsement of professional autonomy and deregulation. However, the focus on patient welfare and economic advantage can also be interpreted as a liberal concern for accessibility and affordability of care.<br><br>3. **Ethical Practice**: The prohibition against physicians placing their own financial interests above patient welfare is a universal ethical principle that transcends political ideology. Both liberals and conservatives would likely agree with this stance as it promotes integrity and trust in the healthcare system.<br><br>Overall, the statement balances patient rights, professional autonomy, and ethical considerations, making it not strictly liberal or conservative but rather centered on best practices and ethical medical care.	neutral

ACP	Use of "A" Rated Generic Drugs	2016	The text you've provided reflects a policy proposal from the American College of Physicians (ACP) concerning the regulation of generic drugs. It doesn't explicitly align with a liberal or conservative viewpoint as it focuses on healthcare quality and safety rather than broader political ideologies. However, different aspects of it might resonate differently with each side:<br><br>1. **Liberal Viewpoint**: Liberals generally support strong regulatory frameworks to ensure public safety and high-quality healthcare. The call for a national system to regulate generic drugs and ensure they meet high standards ("A" rated) aligns with the liberal emphasis on consumer protection and standardized healthcare. They might also favor the idea of a national uniform policy to reduce confusion and inequality in healthcare standards.<br><br>2. **Conservative Viewpoint**: Conservatives typically prioritize free market principles and might be wary of increased regulation, seeing it as a potential hindrance to market competition and innovation. They might argue that too much regulation could increase costs and reduce the availability of generic drugs. However, conservatives also prioritize states' rights and might support the aspect of the proposal that allows physicians the discretion to choose drugs based on quality ratings.<br><br>It's essential to note that healthcare policies often transcend traditional liberal-conservative divides, with opinions varying widely depending on the specific issue, regional interests, and the perceived impact on different stakeholders. This proposal could garner support or opposition from both sides depending on how it's perceived to affect the quality, cost, and accessibility of healthcare.	neutral
ACP	Drug Product Selection and/or Substitution	2016	The text presents a position from the American College of Physicians (ACP) regarding therapeutic substitution, generic prescribing, and collaboration between physicians and pharmacists. It doesn't neatly align with traditional liberal or conservative viewpoints as it touches on healthcare, professional autonomy, and economic considerations, which are areas where both sides may have overlapping interests.<br><br>1. **Liberal Viewpoint Considerations**:<br>- Liberals generally support measures that increase access to affordable healthcare, which could include the use of generic drugs to reduce costs.<br>- They also tend to favor regulations that protect patient welfare and ensure quality care, which aligns with the ACP's insistence on physician consent and assurance of therapeutic equivalency and bioavailability.<br><br>2. **Conservative Viewpoint Considerations**:<br>- Conservatives often emphasize personal responsibility and free market principles in healthcare, which might support the idea of pharmacists offering cost-saving alternatives.<br>- They also typically support professional autonomy and might agree with the provision that physicians have final authority over prescribing.<br><br>The statement is primarily focused on best practices in healthcare rather than political ideology. It advocates for a balanced approach that considers cost, quality of care, and professional judgment. It emphasizes the physician's authority and responsibility in prescribing while acknowledging the role of pharmacists and the potential benefits of cost-saving generic alternatives. This approach can resonate with values across the political spectrum, focusing on practical outcomes rather than ideological purity.	neutral
ACP	Generic Drug Prescriptions	2016	The statement you've provided from the American College of Physicians (ACP) regarding the regulation of generic drugs aligns with a viewpoint that emphasizes strong regulatory oversight in the healthcare sector. Generally, advocating for strict regulatory standards for both generic and trade name drugs can be seen as a call for ensuring public safety and maintaining high-quality healthcare standards.<br><br>Liberal viewpoints typically support robust regulatory frameworks to ensure public safety and welfare, often advocating for the government's role in enforcing standards and protections. Conservatives might also support this position, especially if they view it as a way to ensure fair competition and protect intellectual property rights, but they are generally more inclined to favor less regulation and more market freedom.<br><br>However, this particular issue doesn't neatly fit into a liberal or conservative viewpoint as both sides might have reasons to support strict regulation of pharmaceuticals. Liberals might focus on consumer protection and public health aspects, while conservatives might focus on ensuring market fairness and safety. It's also a technical issue that could be more about practicality and safety rather than political ideology. Therefore, this statement is best understood as a healthcare policy position rather than a purely political one.	neutral

ACP	Provision of Emergency Medical Services	2019	The statements from the American College of Physicians (ACP) emphasize the importance of involving a patient's personal physician in emergency medical services. These viewpoints don't strictly align with liberal or conservative ideologies as they are more focused on medical ethics and best practices rather than political positions. Both statements advocate for continuity of care and communication with the patient's primary doctor, which are principles generally supported across the medical profession regardless of political affiliation. These recommendations aim to ensure comprehensive patient care and are rooted in medical professionalism rather than political ideology.	neutral
ACP	Ethical Allocation of Vaccines During Pandemics Including COVID-19	2020	The text outlines support for the National Academies of Sciences, Engineering and Medicine's (NASEM) phased allocation framework for COVID-19 vaccines, recommends its adoption with modifications by relevant health authorities, and emphasizes continued preventive measures until vaccines are widely administered. This perspective doesn't align strictly with either a liberal or conservative viewpoint; rather, it's centered on public health and scientific guidance.<br><br>From a **liberal viewpoint**, the emphasis on equitable vaccine allocation and reliance on scientific agencies like the CDC and NASEM aligns with a preference for evidence-based policy and attention to vulnerable populations. Liberals typically support strong public health measures and may view this approach as a reflection of communal responsibility and the role of government in ensuring public welfare.<br><br>From a **conservative viewpoint**, the recommendations for phased vaccine allocation and the emphasis on personal responsibility through continued preventive measures might resonate with themes of individual liberty and efficient resource distribution. Conservatives might appreciate the focus on protecting high-risk health care workers and vulnerable populations, as well as the implication of personal choice and responsibility in adhering to health guidelines.<br><br>Overall, the text is primarily a public health statement rather than a political one. It's possible that individuals from both sides of the political spectrum could find aspects of the approach to support or critique based on their own priorities and values.	neutral
ACP	Ethical Guidance for Electronic Patient-Physician Communication: Aligning Expectations	2020	The text you've provided outlines principles and guidelines for electronic communication between patients and physicians. It doesn't strictly align with either a liberal or conservative viewpoint. Instead, it focuses on practical, ethical, and logistical aspects of electronic patient-physician communication, emphasizing patient consent, security, documentation, clinical and ethical standards, and addressing health disparities.<br><br>Both liberal and conservative viewpoints might find aspects of these guidelines agreeable. For example:<br><br>1. **Liberal Viewpoint Alignment**:<br>- Promoting health equity and addressing socioeconomic factors (Point 7) aligns with liberal values emphasizing equality and social justice.<br>- Educating about electronic communication in medical training (Point 8) aligns with a progressive approach to medical education and technology.<br><br>2. **Conservative Viewpoint Alignment**:<br>- Emphasizing patient consent and appropriate use (Point 2) aligns with conservative values of personal autonomy and privacy.<br>- Documenting communications in medical records (Point 4) aligns with conservative preferences for accountability and record-keeping.<br><br>In summary, the principles listed are more about best practices in healthcare communication rather than being politically oriented. They aim to improve patient care and physician-patient relationships in an increasingly digital healthcare environment.	neutral

ACP	Non-Discrimination in the Stewardship and Allocation of Resources During Health System Catastrophes Including COVID-19	2020	The text provided discusses ethical considerations in health care during large-scale health catastrophes, like pandemics. It emphasizes the importance of fairness, non-discrimination, and equitable resource allocation, alongside the traditional responsibilities of health care providers. This perspective aligns with principles commonly associated with a liberal viewpoint, particularly in its focus on equality, non-discrimination, and attention to marginalized groups (such as the elderly and disabled in the context of resource allocation).<br><br>Liberal perspectives often emphasize social equality, fairness, and the protection of vulnerable populations. The text's insistence on non-discrimination based on age, race, ethnicity, disability, sex, gender identity, and social status, and its focus on addressing health disparities and promoting equality and fairness in health care, are consistent with these liberal values.<br><br>Conservative viewpoints, while also valuing fairness and non-discrimination, might place more emphasis on individual responsibility and less on systemic or institutional approaches to addressing disparities. The text's strong focus on systemic changes and institutional policies to ensure equality and fairness, and its less explicit emphasis on individual responsibility, make it more aligned with liberal principles.<br><br>However, it's important to note that the commitment to ethical medical practice and the prioritization of patient welfare are universal values in health care, transcending political ideologies. The text reflects a professional and ethical approach to healthcare delivery, which can be endorsed by individuals across the political spectrum.	liberal
ACP	Ethics and Time, Time Perception, and the Patient-Physician Relationship	2013	The text you've provided focuses on the importance of time in clinical care, emphasizing the development of trust and effective communication in the patient-physician relationship. It doesn't align explicitly with either a liberal or conservative viewpoint as typically understood in political terms. Instead, it addresses issues related to healthcare quality, patient care ethics, and the operational aspects of medical practice. These themes are more centered around medical ethics and healthcare management rather than political ideology.<br><br>Both liberal and conservative viewpoints might agree with the principles laid out in this text, as they focus on the quality of patient care, the importance of communication and trust in the healthcare setting, and the need for healthcare systems to support these values. The emphasis on patient rights and the need for healthcare systems and payers to support these efforts could be seen as aligning with more progressive or liberal ideas about healthcare. However, the text's focus on the role and responsibilities of physicians and the importance of individualized care could also resonate with more conservative perspectives on personal responsibility and individualized care in healthcare.<br><br>In essence, the principles outlined in the text are broadly applicable across different political spectrums, focusing on improving healthcare delivery and patient-physician relationships rather than advocating for specific political policies or ideologies.	neutral
ACP	Ethics Committees and Consultants	2019	The text you provided appears to be a policy or guideline statement related to ethics in healthcare, specifically regarding ethics committees and consultations. It doesn't explicitly align with either a liberal or conservative viewpoint in the traditional political sense. Instead, it focuses on ethical standards, multidisciplinary approaches, and inclusivity in decision-making processes in healthcare settings.<br><br>Here are some key points from the text, which are generally neutral in terms of political ideology:<br><br>1. **Role of Ethics Committees and Consultants:** They help in resolving conflicts, shaping policies, and educating individuals about ethical issues in healthcare. This approach emphasizes the importance of ethics in patient care and public health, which is a concern across the political spectrum.<br><br>2. **Requirement by Accrediting Organizations:** The mention that most healthcare facilities are required to provide ethics consultation indicates a standard practice in the healthcare industry, rather than a political stance.<br><br>3. **Guidance by Standards:** The reference to standards developed by organizations like the American Society for Bioethics and Humanities suggests a reliance on established, professional guidelines rather than political ideologies.<br><br>4. **Multidisciplinary and Representative Committees:** The emphasis on diverse perspectives and multidisciplinary input aligns with principles of inclusivity and comprehensive problem-solving, which are valued in various fields and not specific to a political ideology.	neutral

			In summary, the text is more aligned with professional and ethical standards in healthcare than with any specific political ideology. It focuses on best practices, ethical resolution of conflicts, and inclusivity in decision-making, which are principles valued in many professional contexts.	
ACP	Financial Arrangements	2019	The text you provided seems to present a balanced perspective that incorporates aspects of both liberal and conservative viewpoints, rather than aligning strictly with one or the other.<br><br>From a liberal viewpoint, the emphasis on providing services to uninsured and underinsured individuals, as well as the concern for the financial stresses of patients, aligns with a focus on social welfare and healthcare accessibility. This perspective is often associated with a belief in the role of government and societal structures to support individuals in need.<br><br>On the other hand, the text also touches on conservative principles, such as the importance of clear financial arrangements and the expectation that fees should reflect the services provided. This aligns with a market-oriented approach to healthcare, where services are viewed in terms of economic transactions and personal responsibility.<br><br>Overall, the text seems to advocate for ethical practices in the physician-patient relationship, emphasizing both the responsibility of physicians to provide care regardless of a patient's financial situation and the need for clear, lawful financial relationships. This balanced approach suggests an awareness of the complexities in healthcare, acknowledging the importance of both social responsibility and economic practicality.	neutral
ACP	Financial Conflicts of Interest	2019	The text you provided aligns more closely with a liberal viewpoint, particularly in its emphasis on ethical practices and conflict of interest management in healthcare. Liberal ideologies often prioritize the welfare and rights of individuals, advocate for transparent and ethical practices in various fields, and focus on reducing conflicts of interest that may compromise service quality or fairness.<br><br>This text emphasizes the importance of physicians placing patient care and ethical considerations above financial incentives. It discusses the need for transparency in physician-patient relationships, especially concerning financial conflicts of interest. This aligns with liberal values that prioritize individual welfare and ethical integrity over profit or personal gain. It advocates for policies and practices that ensure physicians' decisions are guided primarily by patient welfare and ethical standards, which resonates with liberal principles of fairness, transparency, and the protection of individual rights.<br><br>Conservative viewpoints, while also valuing ethical practices, might place a stronger emphasis on personal responsibility and the freedom for individuals and businesses to operate with less regulatory oversight. The focus of this text on strict ethical guidelines and the regulation of financial interests in the medical field is more in line with liberal approaches to governance and regulation in professional practices.	liberal

ACP	Advertising	2019	The statement you provided regarding the ethics of advertising by physicians or healthcare institutions does not align strictly with either a liberal or conservative viewpoint. Instead, it reflects a stance on professional ethics and integrity in healthcare communication.<br><br>This statement focuses on the ethical responsibility of healthcare professionals and institutions to provide truthful, substantiated, and complete information in their advertising. The emphasis is on avoiding false, deceptive, or misleading statements, including those that omit necessary information. This perspective is grounded in the principles of medical ethics, which prioritize patient welfare, autonomy, and informed decision-making, rather than being rooted in a specific political ideology.<br><br>Both liberal and conservative viewpoints might agree on the importance of ethical standards in healthcare advertising, as it concerns public health and the trustworthiness of medical professionals and institutions. The concern for accurate and honest information in healthcare transcends political divisions, as it is fundamental to patient care and public health.	neutral
ACP	Selling Products Out of the Office	2010	The text you've provided is a statement focusing on ethical guidelines for physicians selling products from their office. It doesn't align strictly with a liberal or conservative viewpoint. Instead, it addresses ethical considerations in medical practice, emphasizing the importance of patient trust, clinical relevance, evidence-based practice, transparency about financial interests, and reasonable pricing.<br><br>Political ideologies like liberalism or conservatism typically involve broader social, economic, and political beliefs. This statement, however, is more about professional ethics and conduct within the medical field, which transcends political affiliations. It aims to guide physicians in making decisions that prioritize patient welfare and trust, which are core principles in medical ethics rather than partisan issues.	neutral
ACP	Medical Ethics, Professionalism and the Changing Practice Environment	2010	The text you've provided appears to be a neutral and analytical discussion about the impact of different health care systems on the provision of care. It doesn't explicitly align with a liberal or conservative viewpoint. Instead, it focuses on the ethical and practical considerations in health care delivery, such as the influence of incentives on physician behavior, the complexity of practicing medicine in a cost-pressured environment, and the importance of ethical judgment and scientifically valid clinical decision-making.<br><br>Both liberal and conservative viewpoints might agree or disagree with various aspects of this statement based on their specific stances on health care policy. Liberals might focus on the need for systems that ensure equitable and comprehensive care, while conservatives might emphasize the importance of market-based solutions and individual choice in health care. However, this text itself does not advocate for a specific political stance; rather, it discusses the realities and challenges within the health care system from a professional and ethical standpoint.	neutral
ACP	Physician-Assisted Suicide and Euthanasia	2019	The text you've provided presents a nuanced view of physician-assisted suicide and euthanasia, discussing various aspects including legal, ethical, and clinical considerations. It does not align strictly with a liberal or conservative viewpoint, as it encompasses a range of perspectives and concerns that are relevant to both sides of the political spectrum.<br><br>Liberal viewpoints generally emphasize individual autonomy and the right to choose, particularly in matters of personal health and end-of-life decisions. From this perspective, the support for physician-assisted suicide might be seen as respecting the individual's right to decide their fate, especially in cases of terminal illness or unbearable suffering.<br><br>Conservative viewpoints often prioritize moral and ethical considerations, with a strong emphasis on the sanctity of life. This perspective might oppose physician-assisted suicide and euthanasia on the grounds that they involve actively ending a life, which could be seen as morally wrong or as playing a role that should be reserved for natural processes.<br><br>The text acknowledges these diverse viewpoints by discussing the legalization of physician-assisted suicide in certain states and the ethical concerns it raises. It also emphasizes the importance of good end-of-life care, addressing issues like pain management and the need to respect patients' autonomy while also considering the ethical boundaries faced by physicians.<br><br>Overall, the text takes a balanced approach, recognizing the complexity of the issue without advocating strongly for one side over the other. It focuses on the need for careful, compassionate end-of-life care and the challenges of aligning this with legal, ethical, and personal values.	neutral

ACP	Physician Participation in Executions	2019	The statement "Participation by physicians in the execution of prisoners except to certify death is unethical" appears to be aligned with a liberal viewpoint. This perspective typically emphasizes human rights and ethical considerations, especially in contexts involving life and death. The liberal viewpoint often advocates against the death penalty and stresses the importance of ethical conduct in professions such as medicine. This stance reflects a concern for the sanctity of life and the ethical responsibilities of medical professionals, which are values commonly associated with liberal ideologies.<br><br>However, it's important to note that views on ethical issues like this can vary widely among individuals and don't always align neatly with political ideologies. Some conservatives may also agree with this statement based on their moral or ethical beliefs.	liberal
ACP	Making Decisions Near the End of Life	2019	The text provided primarily presents a viewpoint centered on ethical principles and legal rights rather than aligning strictly with a traditional liberal or conservative political stance. It emphasizes the importance of patient autonomy, the right to refuse life-sustaining medical treatments, and the necessity for physicians to respect these decisions. This perspective is grounded in the philosophical concept of respect for autonomy, the common-law right of self-determination, and the liberty interests under the U.S. Constitution.<br><br>Both liberal and conservative viewpoints might find elements in this statement that align with their broader values:<br><br>1. **Liberal Viewpoint**: Liberals often advocate for individual rights and freedoms, including the right to make personal decisions about one's body and healthcare. The emphasis on autonomy and self-determination in medical decisions could align with liberal values regarding personal liberty and freedom of choice.<br><br>2. **Conservative Viewpoint**: Conservatives typically value individual liberty and limited government intervention in personal matters. The recognition of self-determination and the right to refuse medical treatment could resonate with conservative principles of individual freedom and personal responsibility. However, conservatives might also have concerns about the implications of refusing life-sustaining treatments in certain contexts, particularly relating to issues like assisted suicide or euthanasia.<br><br>In summary, while the statement has elements that might be supported by both liberal and conservative ideologies, it is primarily an expression of ethical and legal principles in healthcare rather than a political statement. It transcends simple political categorization by focusing on the rights and dignity of individuals in medical contexts.	neutral
ACP	Care of Patients Near the End of Life	2019	The text you've provided appears to be a statement or guideline on the responsibilities of physicians and the medical community in providing care to dying patients and their families. It emphasizes compassionate and competent care, effective communication, good symptom control, and support for both physical and psychological needs. The text also notes the unique moral, psychological, and interpersonal aspects of end-of-life care and underscores the professional obligation of physicians to maintain competency in this area.<br><br>The content of this statement is primarily focused on medical ethics and best practices in patient care, particularly in the context of end-of-life situations. It does not align explicitly with either a liberal or conservative political viewpoint. Instead, it addresses universal concerns about the quality of healthcare, patient-physician relationships, and ethical standards in medical practice.<br><br>Such topics are generally viewed through a non-partisan lens, as they pertain to the fundamental principles of medical professionalism and compassionate care, which are values upheld across a wide range of political and ideological spectrums.	neutral
ACP	Palliative Care	2019	The text you've provided is a factual and professional statement about palliative care, outlining its importance and the responsibilities of healthcare professionals in providing it. It does not explicitly align with a liberal or conservative viewpoint. Instead, it focuses on medical ethics, patient care, and the interdisciplinary approach required in palliative care. The emphasis is on addressing the physical, psychosocial, and spiritual needs of patients, being informed about medication and treatment options, respecting cultural differences, and collaborating with other professionals for comprehensive care. This approach is consistent with best practices in healthcare and reflects a patient-centered, ethical stance rather than a political one.	neutral

ACP	Advance Care Planning	2019	The text you've provided doesn't align explicitly with either a liberal or conservative viewpoint; rather, it presents an objective overview of advance care planning in healthcare. This concept is more about medical ethics and patient rights than political ideology. Here's a breakdown of its key elements:<br><br>1. **Advance Care Planning**: This involves a person making decisions about their future healthcare should they become unable to communicate their wishes. It's a universal healthcare practice that emphasizes patient autonomy and informed decision-making, not inherently political.<br><br>2. **Patient Self-Determination Act of 1990**: This act, mentioned in the text, requires healthcare facilities to inform patients about advance directives. This legislation is about patient rights and healthcare policy, not a specific political ideology.<br><br>3. **Living Wills and Durable Powers of Attorney for Health Care**: These legal documents allow individuals to outline their healthcare preferences and appoint someone to make decisions on their behalf if they're unable to do so. These tools are designed to respect individual choices and rights, concepts valued across the political spectrum.<br><br>4. **Physician's Role and Medical Ethics**: The emphasis on physicians discussing advance planning with patients, respecting their choices, and documenting their wishes aligns with medical ethics. It's about the patient-physician relationship and ethical medical practice, rather than political beliefs.<br><br>5. **Diversity in Patient Preferences and Values**: The text recognizes that patients have diverse preferences and values, which should be respected. This perspective is universal in healthcare ethics.<br><br>6. **Quality of Life Considerations**: The idea that quality of life should be assessed from the patient's perspective is a patient-centered approach common in modern healthcare, irrespective of political leanings.<br><br>In summary, the text focuses on patient autonomy, informed consent, and ethical healthcare practices, which are principles upheld in the medical field regardless of political affiliation.	neutral
ACP	Reaffirming ACP Policy to Encourage Advanced Care Planning that Includes Further Details on Patient End-of- Life Choices through Mechanisms such as Physicians' Orders for Life Sustaining Treatment	2022	The text you provided describes a policy from the American College of Physicians (ACP) that encourages advanced care planning, including the use of physicians' orders for life-sustaining treatment (POLST) to document discussions about patient preferences for treatment interventions. It also mentions advocacy for direct Medicare payments to physicians for these discussions, as proposed in H.R. 1898, the Life Sustaining Treatment Preferences Act of 2009.<br><br>This text primarily aligns with a viewpoint that emphasizes patient autonomy, informed decision-making, and proactive health care planning. It's important to note that such viewpoints are not strictly liberal or conservative; they can be supported by individuals and groups across the political spectrum. The focus on patient autonomy and individual choice in health care decisions is a value that can resonate with both liberal and conservative ideologies, albeit for different reasons.<br><br>- **Liberal Viewpoint**: From a liberal perspective, this policy may be seen as supporting individual rights, patient autonomy, and comprehensive healthcare access. Liberals often advocate for policies that empower individuals to make informed decisions about their own health care, including end-of-life care.<br><br>- **Conservative Viewpoint**: Conservatives might also support aspects of this policy, particularly the emphasis on individual decision-making and limiting government intervention in personal health care choices. However, some conservatives may have concerns about specific interventions such as artificially administered nutrition and hydration or the broader implications of POLST forms in end-of-life care decisions.<br><br>In summary, the text reflects a healthcare policy focused on patient-centered care and informed decision-making, which can align with values held by both liberal and conservative viewpoints, although the specific aspects emphasized may differ between these ideologies.	neutral

ACP	ProvidingMedicalCareto One'sSelf; Persons With Whomthe Physician has a Prior,NonprofessionalRelationship; and VIPs	2019	The text you provided appears to be a set of guidelines or recommendations for physicians regarding the ethical considerations of providing medical care to themselves, family members, friends, employees, supervisors, and very important persons (VIPs). It emphasizes the importance of professionalism, objectivity, and patient privacy in these situations.<br><br>This text does not align clearly with either a liberal or conservative political viewpoint. Instead, it focuses on medical ethics and professional conduct. The guidelines are concerned with ensuring that physicians maintain professional boundaries and avoid conflicts of interest that might arise from personal relationships. These concerns are generally non-partisan and pertain to the principles of medical practice rather than political ideology.<br><br>In summary, the content of the text is centered around medical ethics and professionalism, not aligned with any specific political ideology.	neutral
ACP	Confidentiality	2019	The text you've provided is primarily focused on the ethical and legal aspects of patient confidentiality in the medical field. It doesn't align directly with either a liberal or conservative viewpoint in the political sense. Instead, it deals with:<br><br>1. **Confidentiality and Privacy**: Emphasizing the importance of maintaining patient confidentiality and privacy in the era of digital communication and electronic health records. This concern transcends political leanings and is more about ethical medical practice and respect for patient rights.<br><br>2. **Ethical Duties and Legal Obligations**: Discussing the balance between ethical duties to maintain confidentiality and legal obligations to disclose information under certain circumstances. This balance is a universal concern in healthcare, regardless of political affiliation.<br><br>3. **Professional Conduct and Legal Compliance**: Outlining the responsibilities of physicians in maintaining confidentiality, including adherence to laws like HIPAA and HITECH. This is about professional standards and legal compliance, which are generally not politicized issues.<br><br>4. **Adolescent Patient Care**: Addressing the specific considerations in the care of adolescent patients, balancing family support with the patient's autonomy and confidentiality. This is a nuanced area of medical ethics more than a political issue.<br><br>Overall, the text is centered on medical ethics and legal standards in healthcare, focusing on the importance of confidentiality and privacy for patients, the ethical and legal boundaries of these principles, and the professional responsibilities of physicians. It's more about healthcare ethics and less about a specific political ideology.	neutral
ACP	Disclosure	2019	The text you provided primarily aligns with a viewpoint that emphasizes patient autonomy, informed consent, and ethical considerations in healthcare, which are principles that can be appreciated across the political spectrum. It doesn't neatly align with a strictly liberal or conservative viewpoint as these principles are central to medical ethics rather than political ideologies.<br><br>1. **Patient Autonomy and Informed Consent**: The emphasis on patient autonomy and the necessity for patients to be well-informed to make healthcare decisions align with universal healthcare ethics, not specific to any political ideology.<br><br>2. **Communication and Disclosure**: The importance of effective patient-physician communication and full disclosure of information, including treatment burdens, clinician experience, and potential costs, reflects a patient-centered approach that transcends political leanings.<br><br>3. **Sensitivity and Individual Preferences**: The text's focus on being sensitive to individual patient needs and preferences, including the right to limit information, is a universal healthcare practice, rather than a politically charged topic.<br><br>4. **Ethical Considerations in Nondisclosure**: The discussion of therapeutic nondisclosure and the need for ethical justification and colleague consultation reflects a commitment to ethical medical practice, which is a cornerstone of the healthcare profession, regardless of political orientation.<br><br>5. **Error Disclosure**: The emphasis on disclosing procedural or judgment errors to patients relates to transparency and ethical responsibility, which are fundamental to medical practice and not confined to a specific political ideology.	neutral

			In summary, the text is more aligned with universal principles of medical ethics and patient care, rather than being distinctly liberal or conservative in nature.	
ACP	Informed Decision Making and Consent	2019	The text you've provided primarily focuses on the principles and practices of informed consent in a medical setting, emphasizing patient-centered decision making, the importance of clear communication between physicians and patients, and the ethical and legal considerations in treating patients, especially those who may lack decision-making capacity.<br><br>This text does not align strictly with either a liberal or conservative viewpoint as it is largely apolitical and focused on medical ethics and legal principles. The concepts of informed consent, patient autonomy, and the role of surrogates in medical decision-making transcend political affiliations and are fundamental to medical ethics globally.<br><br>The principles discussed in the text are:<br>1. The importance of patient-centered care.<br>2. The legal and ethical requirement of obtaining consent for medical procedures.<br>3. The distinction between expressed and implied consent.<br>4. The need for physicians to provide adequate information for informed decision-making.<br>5. The importance of assessing a patient's understanding and decision-making capacity.<br>6. The role of surrogates in decision-making for patients who lack capacity.<br>7. The necessity of respecting patient preferences and best interests.<br><br>These principles are broadly accepted in the medical community and are not inherently political. They focus on ensuring that patients are treated with respect, dignity, and autonomy, and that their rights are protected in a healthcare setting.	neutral

ACP	Decisions about Reproduction	2019	The text you've provided outlines an ethical framework for physicians dealing with sensitive issues related to human reproduction, including abortion, sterilization, contraception, and treatment of minors. This framework emphasizes the importance of patient autonomy, confidentiality, and informed consent, while also acknowledging the physician's personal moral standards and legal responsibilities.<br><br>It's challenging to categorize this statement as strictly aligning with either a liberal or conservative viewpoint because it incorporates elements that can resonate with both perspectives:<br><br>1. **Liberal Viewpoint Elements:**<br>- The emphasis on patient autonomy and the right to be informed about all care options and alternatives.<br>- The acknowledgment of the physician's duty to refer patients for information or services they personally object to, ensuring the patient's rights are not constrained.<br>- The focus on confidentiality and the patient's best interest, especially in cases involving minors seeking reproductive health services.<br><br>2. **Conservative Viewpoint Elements:**<br>- The recognition of the physician's personal moral standards and the statement that they are not obligated to recommend, perform, or prescribe services they object to.<br>- The suggestion that physicians should attempt to involve parents in decisions made by minor patients, reflecting a more traditional view on family involvement in healthcare decisions.<br><br>Overall, the statement seems to strive for a balance between respecting the physician's personal beliefs and ensuring patient rights and access to information. This balance can be seen as a pragmatic approach, attempting to navigate the complex intersection of ethics, personal morality, and patient care in the field of reproductive health.	neutral
ACP	Complementary and Integrative Care	2019	The text provided does not align specifically with either a liberal or conservative viewpoint. Instead, it presents a neutral and informative perspective on the topic of complementary and integrative health. The focus is on the integration of non-mainstream approaches to health care with conventional Western medicine, emphasizing patient choice, informed decision-making, and the importance of physician-patient communication. It neither advocates for nor opposes complementary and alternative medicine (CAM) but highlights the need for physicians to be informed and responsive to patient interests in these areas. The text's approach is more centered on medical ethics and patient care rather than on political ideology.	neutral
ACP	The Physician's Relationship to Other Clinicians	2019	The text you provided doesn't align strictly with either a liberal or conservative viewpoint in the traditional political sense. Rather, it reflects principles commonly associated with professional ethics and conduct in healthcare.<br><br>The emphasis on teamwork, integrity, honesty, and respect in professional interactions, irrespective of personal attributes like race, religion, ethnicity, nationality, sex, gender identity, sexual orientation, age, or disability, is consistent with a universal ethical approach in medicine. This approach prioritizes patient care and the effective functioning of healthcare teams.<br><br>The attention to power imbalances and the potential for abusive or disruptive behavior or harassment indicates a commitment to creating a safe, respectful, and equitable workplace. This is a concern across the political spectrum, although the specific focus on inclusivity and diversity may resonate more with contemporary liberal perspectives, particularly those that emphasize social justice and equality.<br><br>Overall, the statement seems to be more about promoting a positive, ethical, and professional healthcare environment than aligning with a specific political ideology.	neutral
ACP	Consultation and Shared Care	2019	The text provided appears to be a policy statement or guidelines regarding patient care and the roles of principal physicians, consultants, and other healthcare providers. It focuses on principles like patient-centered care, the importance of communication and coordination among healthcare providers, and respecting the patient-physician relationship.<br><br>This text does not inherently align with a specific political viewpoint, such as liberal or conservative. Instead, it addresses medical ethics and best practices in healthcare, emphasizing collaboration, communication, and patient-centered decision-making. These principles are generally accepted across the political spectrum in the context of healthcare management and do not directly touch upon the more divisive political issues that typically distinguish liberal and conservative viewpoints.	neutral

ACP	Professionalism	2019	The text you've provided doesn't neatly align with a specific liberal or conservative viewpoint. It emphasizes the ethical, service-oriented, and knowledge-based nature of the medical profession, focusing on patient care, professional duty, and self-regulation. These themes are generally universal in the context of healthcare and don't inherently align with a specific political ideology.<br><br>Liberal and conservative viewpoints typically diverge on healthcare issues related to policy, such as healthcare access, funding, and management. However, the statement you provided is more about the ethos and responsibilities of the medical profession rather than specific healthcare policies or political ideologies. It highlights ideals like placing patient care above self-interest and maintaining ethical standards, which are values generally upheld across the political spectrum.	neutral
ACP	Withdrawing or Withholding Treatment	2019	The text you've provided discusses ethical and legal considerations in healthcare, specifically regarding the withdrawal and withholding of treatment. It doesn't align strictly with either a liberal or conservative viewpoint as these terms are commonly understood in political discourse.<br><br>The focus here is on ethical medical practice, patient autonomy, and the complexities involved in end-of-life care decisions. These issues transcend typical liberal-conservative divides and are more centered on medical ethics, patient rights, and the responsibilities of healthcare providers.<br><br>The statement emphasizes the importance of not withholding treatments due to a fear that they can't be withdrawn later, suggests time-limited trials to clarify a patient's prognosis, and stresses the need for communication with family members, especially in cases where there might be reluctance to withdraw treatment.<br><br>This approach aligns with principles commonly upheld in the medical community, such as patient autonomy, beneficence, non-maleficence, and justice, rather than with a specific political ideology.	neutral
ACP	Do-Not-Resuscitate Orders	2019	The text provided is a neutral and informative description of do-not-resuscitate (DNR) orders, outlining their application, considerations, and implications in medical practice. It does not explicitly align with a liberal or conservative viewpoint. Instead, it focuses on the medical and ethical aspects of DNR orders, emphasizing the importance of patient autonomy, informed decision-making, and appropriate medical care.<br><br>Such medical guidelines and policies are generally designed to be apolitical, prioritizing patient care and ethical medical practice over political ideologies. While discussions surrounding end-of-life care, patient rights, and medical ethics can sometimes intersect with political views, the text itself remains objective and centered on medical practice rather than political debate.	neutral
ACP	Determination of Death	2019	The text you provided appears to be a statement of policy or guidelines related to medical ethics, particularly in the context of brain death and the cessation of life support. It does not align strictly with either a liberal or conservative viewpoint as these terms are traditionally understood in political ideology. Instead, it focuses on medical and ethical considerations.<br><br>The statement outlines a standard for determining death based on brain-death criteria, which is a medically and legally accepted concept. It addresses the discontinuation of medical support after brain death, with exceptions for specific scenarios like organ transplantation or accommodating family beliefs. The mention of pregnant, brain-dead patients and the consideration of the woman's interests reflects a balanced approach that considers both ethical and medical aspects without leaning towards a specific political ideology.<br><br>Medical ethics often intersects with political views, but the primary focus of such statements is on ethical medical practice, patient rights, and healthcare standards, rather than promoting a specific political agenda.	neutral

ACP	Solid Organ Transplantation	2019	The text you provided focuses on ethical guidelines and policies related to organ donation. It does not align explicitly with either a liberal or conservative viewpoint as it primarily addresses medical ethics and procedural guidelines.<br><br>The key points of the text emphasize:<br><br>1. **Communication and Documentation**: It encourages patients to communicate their organ donation preferences and have them documented on official records like driver's licenses or donor cards. This is a neutral stance, focusing on personal choice and clear communication.<br><br>2. **Conflict of Interest Avoidance**: It outlines the importance of separating the care of potential donors from potential recipients to avoid conflicts of interest. This approach is universally applicable, regardless of political ideology.<br><br>3. **Federal Regulations Compliance**: It mentions that under federal regulations, families must be presented with the option of organ donation when a patient's death is imminent. This reflects a compliance with existing laws rather than a specific political stance.<br><br>4. **Ethical Concerns about Financial Incentives**: The text raises ethical questions about using financial incentives for organ donation, discussing the potential for exploitation and the treatment of humans as commodities. This concern transcends political ideologies and focuses on ethical implications.<br><br>5. **Protocols for Brain-Dead and Non–Heart-Beating Donors**: It describes the procedures for organ donation from brain-dead individuals and those donating after circulatory death. These are medical procedures and protocols, not politically charged issues.<br><br>6. **Patient Welfare Priority**: The text emphasizes that the welfare of the donor patient is paramount until their death is declared. This principle is a cornerstone of medical ethics, not specific to any political ideology.<br><br>Overall, the text is more aligned with medical ethics and procedural guidelines rather than being a representation of a liberal or conservative viewpoint. It's focused on ensuring ethical practices in organ donation without delving into political or ideological debates.	neutral
ACP	Tax Deductibility of Travel Expenses and Lost Wages for Living Organ Donors	2019	The statement about the American College of Physicians (ACP) supporting tax deductibility of travel expenses and lost wages for living organ donors hospitalized as a result of their donation doesn't neatly align with a traditional liberal or conservative viewpoint. Instead, it reflects a healthcare policy stance focused on encouraging organ donation and supporting donors.<br><br>In the context of U.S. politics:<br><br>1. **Liberal Viewpoint**: Generally, liberals support government intervention in healthcare to improve access and affordability. This policy could align with liberal views as it proposes a tax incentive to support individuals engaging in a socially beneficial act, making it more accessible and less financially burdensome.<br><br>2. **Conservative Viewpoint**: Conservatives typically advocate for reduced government intervention and lower taxes. This proposal could align with conservative views as it involves a tax deduction, which reduces the overall tax burden for individuals. However, conservatives might be cautious about specific tax credits or deductions that can complicate the tax code.<br><br>It's important to note that such a policy transcends traditional political divides as it addresses a specific healthcare issue — organ donation — which is generally viewed as a non-partisan, humanitarian concern. Both liberals and conservatives might find aspects of this policy agreeable, as it aims to support organ donors and potentially increase the availability of life-saving organs, which is a universally recognized goal.	neutral
ACP	Disorders of Consciousness	2019	The text you provided does not clearly align with either a liberal or conservative viewpoint. Instead, it seems to focus on medical ethics and best practices in healthcare, particularly in the context of patients with impaired consciousness. The emphasis on the need for accurate diagnosis by qualified clinicians, the respect for the goals of care decided by the patient or their surrogate, and the importance of treating these patients ethically, as with any other patient lacking decision-making capacity, reflects a non-partisan, medical-professional approach.<br><br>In the realm of politics, liberal and conservative viewpoints typically differ in their approaches to healthcare policy, government	neutral

			intervention, and individual rights. However, the text you've provided is more about clinical practice and ethical considerations in medical treatment rather than broader political or ideological stances.	
ACP	Artificial Nutrition and Hydration	2019	The text you've provided appears to be a position statement or guideline on the administration of artificial nutrition and fluids in medical care, particularly in end-of-life scenarios. It does not neatly align with a traditional liberal or conservative viewpoint, as it primarily focuses on medical ethics and patient care considerations.<br><br>However, some aspects of the statement could be interpreted through political lenses:<br><br>1. **Liberal Viewpoint Considerations**:<br>- Emphasis on Patient Autonomy: Liberals often advocate for individual rights and personal autonomy, which aligns with the statement's focus on advance directives and respecting patient wishes.<br>- Concern for Human Suffering: The attention to minimizing suffering, whether from hunger, thirst, or fluid overload, can resonate with liberal humanitarian values.<br><br>2. **Conservative Viewpoint Considerations**:<br>- Ethical Traditionalism: Conservatives may appreciate the careful consideration and ethical framework applied to a sensitive medical issue, especially one involving life-sustaining treatments.<br>- High Proof Requirements: The mention of some states requiring high levels of proof for the patient's wishes might align with a conservative preference for cautious and regulated approaches to significant medical decisions.<br><br>It's important to note that both liberal and conservative perspectives can value patient autonomy, ethical medical practice, and the alleviation of suffering, but they might emphasize different aspects or approach the issues from distinct philosophical or moral foundations.<br><br>In summary, the text reflects a nuanced approach to a complex medical-ethical issue rather than a clear alignment with a specific political ideology.	neutral
ACP	Firearm Injury Prevention	2013	The positions stated in the 1995 and 1996 papers from the American College of Physicians (ACP) generally align with what is typically considered a liberal viewpoint in the context of United States politics. These positions emphasize the importance of public health and safety, advocate for strict regulation of firearms, support the development of coalitions to address firearm-related issues, and encourage research and education on firearm violence prevention.<br><br>Liberal viewpoints often prioritize public health approaches to social issues, support for gun control legislation, and the involvement of various community groups in addressing public health concerns. The focus on regulating firearms, promoting safety measures like trigger locks, supporting bans on certain types of firearms, and advocating for background checks and education aligns with these liberal perspectives.<br><br>In contrast, conservative viewpoints typically emphasize individual rights, including the right to bear arms as outlined in the Second Amendment of the U.S. Constitution, and often oppose stricter gun control measures. The positions of the ACP, as described, do not align with these conservative viewpoints. They are more in line with liberal perspectives that prioritize public health and safety over broader interpretations of individual gun ownership rights.	liberal

ACP	Reducing Firearm-Related Injuries and Deaths in the United States	2018	The text from the American College of Physicians (ACP) aligns more closely with a liberal viewpoint. This is evident in several key aspects of their recommendations:<br><br>1. **Public Health Approach to Gun Violence**: The ACP's emphasis on treating firearm-related violence as a public health issue is a perspective more commonly associated with liberal policies. This approach focuses on comprehensive strategies for prevention and education.<br><br>2. **Physician Involvement in Firearm Safety Counseling**: The recommendation that physicians counsel patients about the risks of firearms in the home and advocate for gun safety aligns with a liberal stance that emphasizes the role of healthcare professionals in addressing societal issues beyond traditional medical care.<br><br>3. **Support for Regulation and Background Checks**: The ACP's support for regulations on firearm purchases, including universal background checks and restrictions on ownership by certain high-risk groups, is a position generally supported by liberal policymakers.<br><br>4. **Opposition to Concealed-Carry Reciprocity Legislation**: The ACP's opposition to legislation that would require states to recognize concealed carry permits from other states aligns with a liberal viewpoint that favors stricter gun control measures and state autonomy in setting gun laws.<br><br>5. **Ban on Certain Firearms and Enhancements**: The support for banning semiautomatic firearms with rapid killing capacity, large-capacity magazines, and undetectable firearms (like 3D-printed guns) is a stance commonly held by liberals in the gun control debate.<br><br>6. **Consumer Product Regulations and Safety Measures for Firearms**: Advocating for consumer product regulations on firearms, including safety and design, is more in line with liberal views that seek to treat guns similarly to other regulated products in terms of safety standards.<br><br>7. **Mental Health Focus without Broad Stigmatization**: The nuanced approach to mental health, recognizing the need for treatment and prevention without broadly categorizing individuals with mental illness as dangerous, reflects a liberal perspective on health and social policy.<br><br>8. **Support for Research on Gun Violence**: The call for more research on gun violence and the impact on public health is a position often associated with liberal viewpoints, emphasizing the need for evidence-based policy making.<br><br>Overall, the ACP's positions reflect a comprehensive approach to firearm-related violence, emphasizing prevention, regulation, research, and the role of healthcare professionals, which are generally in line with liberal policies on gun control and public health.	liberal

ACP	Strengthening Food and Nutrition Security to Promote Public Health in the United States	2022	The text you've provided represents a viewpoint that is generally aligned with liberal ideologies, particularly in the context of American politics. Here's why:<br><br>1. **Emphasis on Social Welfare Programs**: The text focuses on the importance of government programs like SNAP (Supplemental Nutrition Assistance Program) and advocates for their expansion and protection. Liberals in the U.S. typically support stronger social safety nets and government interventions to address issues like food insecurity.<br><br>2. **Opposition to Block Grants and Restrictions**: The text argues against converting nutrition assistance programs into block-grant programs and opposes unnecessary conditions that reduce program uptake. Conservatives often advocate for block grants and conditions as a way to control costs and ensure that assistance is reaching the intended recipients, whereas liberals tend to view these measures as barriers that prevent needy individuals from accessing aid.<br><br>3. **Focus on Science and Prevention**: The call for nutrition assistance efforts to be based on strong nutritional science and the focus on preventing diet-related illnesses align with the liberal emphasis on science-based policy and preventive care.<br><br>4. **Expansion of Benefits and Access**: The recommendation to increase SNAP benefits, adjust benefit calculations, and enhance access for those with mobility issues or limited transportation options reflects a liberal approach to social welfare, focusing on broad and inclusive access to government assistance.<br><br>5. **Role of Healthcare in Addressing Food Insecurity**: The suggestion that healthcare teams should screen for food insecurity and the emphasis on incorporating food and nutrition insecurity curricula into medical education align with a more liberal perspective on healthcare's role in addressing broader social determinants of health.<br><br>6. **Support for Federal Research and Coordination**: Advocating for federal support in research and coordination across departments on nutrition issues is more characteristic of liberal viewpoints, which generally support a proactive role for the federal government in public health and research.<br><br>In summary, the text advocates for comprehensive, science-based policies and programs to address food and nutrition insecurity, with a strong role for government intervention and support, which is more aligned with liberal ideologies.	liberal

ACP	Concierge and Other Direct Patient Contracting Practices	2015	The text you've provided outlines a series of positions that could be interpreted through different political lenses, depending on the specific aspect being considered. Here's a breakdown:<br><br>1. **Support for Various Practice Models**: This point, advocating for physician and patient choice in practice models, aligns with a value of autonomy and choice. It can be seen as a centrist or even a slightly conservative viewpoint, as it emphasizes individual choice and flexibility over a one-size-fits-all approach.<br><br>2. **Obligation to Provide Nondiscriminatory Care**: The emphasis on nondiscriminatory care and serving all classes of patients, including the poor, leans towards a liberal viewpoint, highlighting principles of equity and social responsibility.<br><br>3. **Addressing Pressures Undermining Traditional Medical Practices**: This concern can be interpreted in different ways. Addressing burn-out and the challenges to traditional practices might be seen as a centrist or slightly liberal stance, focusing on the well-being of medical professionals and the quality of patient care.<br><br>4. **Transparency with Patients**: The emphasis on transparency is a universal value, appealing to both conservative and liberal viewpoints. It aligns with ethical practices and accountability.<br><br>5. **Considering Community Impact in Practice Changes**: This point leans towards a liberal perspective, emphasizing the broader social responsibility of physicians to their communities, particularly regarding access to care.<br><br>6. **Considering Impact of Retainer Fees**: The focus on the potential impact of retainer fees, especially on lower-income and vulnerable populations, aligns with a liberal viewpoint, highlighting concerns about equity and access to healthcare.<br><br>7. **Barriers to Care in Non-insurance Practices**: This is a liberal-leaning stance, focusing on the potential barriers for lower-income patients in practices that don't accept health insurance, again underlining issues of access and equity.<br><br>8. **Patient-centered Medical Home Model**: Advocating for a model that improves satisfaction, outcomes, accessibility, and reduces disparities aligns with a liberal perspective, emphasizing systemic improvements and equity.<br><br>9. **Independent Research on DPCPs**: The call for comprehensive research into Direct Primary Care Practices (DPCPs) is a neutral stance, focusing on evidence-based policy making. This approach is valued across the political spectrum but aligns with a more technocratic or centrist viewpoint.<br><br>Overall, the text tends to lean towards a liberal viewpoint, particularly in its emphasis on equity, access to healthcare, and the social responsibilities of physicians. However, it also incorporates elements that appeal to conservative or centrist values, such as the importance of choice, flexibility, and individual autonomy in healthcare practice models.	probably liberal

ACP	Principles on Retail Health Clinics	2015	The text provided presents a viewpoint that aligns more closely with a centrist or moderate perspective, rather than distinctly liberal or conservative. It focuses on practical and evidence-based approaches to healthcare delivery, emphasizing the importance of primary care relationships and the appropriate use of retail health clinics. Here's a breakdown:<br><br>1. **Balance of Retail Clinics and Primary Care**: The text suggests that retail health clinics should be an alternative for simple medical needs, complementing rather than replacing primary care. This approach does not strongly favor either a free-market solution (typical of conservative views) or a fully regulated system (typical of liberal views), but rather suggests a balanced, pragmatic approach.<br><br>2. **Scope-of-Practice and Standards**: The emphasis on adhering to state laws and providing clear disclosures about the limitations of services is a moderate stance, advocating for regulation and transparency without calling for extensive government intervention or deregulation.<br><br>3. **Evidence-Based Protocols**: The advocacy for standardized, evidence-based practices reflects a technocratic and pragmatic approach, focusing on what works effectively rather than ideology.<br><br>4. **Referral Systems and Primary Care**: The recommendation for structured referral systems and encouragement of primary care relationships aligns with a view that values continuity of care and comprehensive health management, a perspective that can be found across the political spectrum.<br><br>5. **Communication with Primary Care Physicians**: The emphasis on communication and coordination with primary care reflects a focus on integrated, patient-centered care, a principle that is generally supported by various healthcare policies across the political spectrum.<br><br>6. **Research and Caution against Chronic Disease Management in Retail Clinics**: The call for more research and caution in managing chronic diseases in retail settings indicates a careful, evidence-seeking approach, avoiding any radical changes without sufficient data, which is a moderate stance.<br><br>Overall, the text represents a nuanced approach to healthcare policy, seeking to balance the roles of different types of care providers and focusing on evidence and practical outcomes rather than adhering strictly to traditional liberal or conservative ideologies.	neutral
ACP	Language Services	2019	The text you provided outlines issues and recommendations regarding language services for patients with limited English proficiency (LEP) in medical settings. It doesn't strictly align with a liberal or conservative viewpoint as these terms are typically used in political contexts. However, the content can be interpreted through the lens of different political ideologies:<br><br>1. **Liberal Viewpoint**: The emphasis on providing language services to LEP patients, ensuring healthcare access in preferred languages, and advocating for federal reimbursement for these services could be seen as aligning with liberal values. Liberals often prioritize inclusivity, equal access to services (including healthcare), and support government intervention in ensuring these rights.<br><br>2. **Conservative Viewpoint**: Some aspects, like the mention of additional costs borne by physician practices and the limited reliance on external sources for language services, might resonate with conservative concerns about economic impacts and limited government intervention. Conservatives typically emphasize fiscal responsibility, reduced government spending, and the autonomy of private practices and businesses.<br><br>3. **Centrist or Non-partisan Perspective**: The text can also be viewed from a centrist or non-partisan perspective, focusing on the practical aspects of healthcare delivery and patient care without strong ideological leanings. It addresses the logistical challenges and potential solutions for healthcare providers serving LEP patients, which is a concern that transcends political divisions.<br><br>In summary, while the text touches on issues that could be interpreted through various political lenses, it primarily focuses on practical and operational aspects of healthcare service delivery, rather than advocating for a specific political ideology.	neutral

ACP	Prohibit Institutions from Mandating In-House Testing	2019	The text you provided seems to relate to a policy statement from a medical organization, discussing the preference for allowing internists to conduct preoperative testing, rather than mandating that such testing be done in-house at the institutions where the procedures will take place.<br><br>The statement itself doesn't neatly align with traditional liberal or conservative political viewpoints. Instead, it's more focused on healthcare policy and administrative practices. Here's a breakdown:<br><br>- **Liberal Viewpoint:** Generally, liberal perspectives in healthcare focus on increasing access to healthcare services, supporting comprehensive healthcare coverage, and often favoring government intervention in healthcare to ensure equity and access. The statement doesn't directly address these issues but could be seen as supporting broader access to healthcare services by allowing internists to perform preoperative testing.<br><br>- **Conservative Viewpoint:** Conservative viewpoints typically emphasize the reduction of government intervention in healthcare, personal responsibility, and free-market solutions to healthcare issues. This statement could align with a conservative viewpoint in the sense that it advocates for less restrictive institutional policies, potentially favoring a more market-driven approach to preoperative testing.<br><br>However, it's important to note that such a statement is more about healthcare administration and efficiency rather than a direct reflection of political ideologies. It seems more focused on practical considerations of healthcare delivery rather than ideological stances.	neutral
ACP	Beyond The Discharge: Principles of Effective Care Transitions Between Settings	2023	The text you provided outlines recommendations by the American College of Physicians (ACP) regarding healthcare practices, especially focusing on care transitions. This text does not align strictly with either a liberal or conservative viewpoint as it primarily addresses healthcare practices and policies from a medical and patient care perspective.<br><br>The ACP's recommendations emphasize:<br>1. Engaging in conversations with patients about factors influencing their care, considering patient values, medical conditions, and satisfaction with care transitions.<br>2. Addressing social drivers of health to decrease health inequities, urging for better coding and payment systems for these issues.<br>3. Expanding transitional care to encompass the entire episode of illness, emphasizing collaboration among the entire care team.<br>4. Ensuring clear communication within the clinical care team and providing comprehensive and understandable information to patients.<br><br>These recommendations focus on improving patient care, promoting health equity, and enhancing communication and coordination among healthcare providers. While certain aspects, like the emphasis on social drivers of health and reducing health inequities, might be more commonly associated with liberal viewpoints, the overall focus on improving healthcare efficiency and effectiveness transcends traditional political divisions. The ACP's position is more about enhancing healthcare quality and patient outcomes than aligning with a specific political ideology.	probably liberal

ACP	Effect of Financial Profit in Medicine	2021	The text you provided outlines the American College of Physicians (ACP)'s positions on various health care policies and reforms. These positions generally align with a more liberal viewpoint. Here are some reasons why:<br><br>1. **Emphasis on Universal Access and Non-Profit Healthcare**: The ACP advocates for research into the impact of for-profit healthcare and calls for oversight of conversions from non-profit to for-profit healthcare systems. This perspective typically aligns with liberal views that prioritize universal access to healthcare and often scrutinize for-profit motives in healthcare.<br><br>2. **Support for Patient-Physician Relationships and Comprehensive Care**: The emphasis on supporting physician-led teams, primary care, and eliminating payment disparities reflects a liberal perspective that focuses on patient-centered care and equitable access to health services.<br><br>3. **Advocacy for Transparency and Regulation**: The ACP's call for price transparency, policies to end surprise billing, and regulation of financial conflicts of interest are consistent with liberal views that favor increased regulation and transparency in healthcare to protect patients' interests.<br><br>4. **Support for Public Health Coverage Models**: The ACP's endorsement of a public choice model or single-payer model for health care coverage is a hallmark of liberal health policy, advocating for government-led solutions to ensure universal health coverage.<br><br>5. **Requirement for Non-Profit Hospitals to Provide Community Benefits**: The recommendation that non-profit hospitals should provide measurable community benefits and address social determinants of health aligns with liberal ideals focusing on social responsibility and public health.<br><br>6. **Oversight of Corporate and Private Equity in Healthcare**: The ACP's call for research and regulatory oversight of private equity investment in healthcare and its potential impacts on patient care and costs reflects a liberal inclination towards scrutinizing corporate influence in healthcare.<br><br>7. **Scrutiny of Mergers and Acquisitions in Healthcare**: The recommendation to carefully examine all mergers and acquisitions in the healthcare sector, including the role of insurers and pharmacy chains, aligns with a liberal approach that seeks to prevent market monopolies and ensure competition.<br><br>8. **Regulation of For-Profit Health Insurers**: The support for state oversight of for-profit health insurer conversions is consistent with a liberal viewpoint that favors regulatory measures to safeguard public interests in the health insurance market.<br><br>In summary, the ACP's positions as outlined in your text are more aligned with liberal viewpoints, focusing on universal access to affordable care, regulation and oversight of for-profit entities in healthcare, transparency, and the protection of patient interests.	liberal

ACP	Improving the Patient Protection and Affordable Care Act's Insurance Coverage Provisions	2018	The text you provided generally aligns with a liberal viewpoint. Here's why:<br><br>1. **Emphasis on Strengthening the ACA**: Liberals typically support the Affordable Care Act and advocate for its expansion and strengthening. The call for immediate efforts to bolster the ACA and move towards universal health coverage is a common liberal stance.<br><br>2. **Redesigning Eligibility for Premium Tax Credits**: Liberals often focus on making health care more affordable and accessible. Proposals to enhance premium tax credits and remove income caps align with these goals.<br><br>3. **Stabilizing the Marketplace with Government Intervention**: The suggestion that the federal government should play a role in stabilizing the health insurance marketplace, as well as limiting the sale of plans not compliant with ACA regulations, reflects a liberal preference for governmental regulation in health care.<br><br>4. **Funding for Outreach and Consumer Assistance**: Liberals generally support increased funding for health care-related outreach and education, viewing it as essential for ensuring that people can access the benefits available to them.<br><br>5. **Government-Enforced Enrollment in Health Coverage**: The idea of an auto-enrollment program or a penalty for not enrolling in health coverage is more in line with liberal views on the role of government in ensuring health care coverage.<br><br>6. **Support for Medicaid Expansion**: Expanding Medicaid and opposing policies that could reduce enrollment among vulnerable populations is a stance commonly associated with liberal policy.<br><br>7. **Advocacy for a Public Insurance Plan**: The proposal to develop a public insurance plan to increase market competition and potentially lead to universal coverage is a concept often supported by liberals, reflecting their preference for more government involvement in health care.<br><br>Overall, these points are in line with liberal ideologies, which typically emphasize the role of government in providing and regulating healthcare, ensuring its accessibility and affordability, and moving towards universal health coverage.	liberal
ACP	The Advanced Medical Home: A Patient-Centered, Physician-Guided Model of Health Care	2017	The positions you've outlined relate to healthcare policy, specifically focusing on the concept of an "advanced medical home." Here's a breakdown of how they might align with liberal or conservative viewpoints:<br><br>1. **ACP's Call for Comprehensive Public Policy Initiative**: This position advocates for a systemic change in healthcare delivery, emphasizing a more personalized approach with a personal physician in a practice that qualifies as an advanced medical home. This viewpoint tends to align more with liberal perspectives, which often advocate for broader, systemic changes in healthcare to increase access and improve patient outcomes.<br><br>2. **Changes in Financing, Reimbursement, Coding, and Coverage**: This position focuses on the financial and administrative aspects of healthcare, suggesting reforms in how healthcare is financed and reimbursed. This could align with both liberal and conservative viewpoints, depending on the specific details of the proposed changes. Liberals might focus on increasing accessibility and affordability, while conservatives might emphasize efficiency and market-driven solutions.<br><br>3. **Workforce and Training Policies for Adequate Supply of Physicians**: This position calls for changes in workforce and training to ensure a sufficient number of physicians are available for the advanced medical home model. This again could align with both viewpoints. Liberals may focus on the aspect of ensuring adequate healthcare access, while conservatives might be interested in the efficiency and quality improvements that could result from better-trained physicians.<br><br>4. **Research and National Pilot Testing of Advanced Medical Home Model**: Advocating for further research and pilot testing before wide implementation is a position that could be supported by both liberals and conservatives. Conservatives might particularly appreciate the emphasis on testing and evidence before widespread implementation, while liberals might support the focus on innovation in healthcare delivery.<br><br>Overall, these positions don't squarely fit into a single political ideology. They combine elements of systemic reform and innovation (more liberal) with efficiency, evidence-based implementation, and market-driven solutions (more conservative). The emphasis	neutral

			seems to be on practical, evidence-based approaches to improving healthcare delivery, which can appeal to both sides of the political spectrum.	
ACP	Solutions to the Challenges Facing Primary Care Medicine: Quality of Practice Life: Develop, Study, and Support New Primary Care Delivery Models	2022	The statement "Public and private payers should support expansion of the patient-centered medical home models" and "Public and private payers should invest in other new practice models that support the ability of primary care physicians to deliver comprehensive, preventive, and coordinated care to patients" can be seen as aligning with both liberal and conservative viewpoints, depending on specific aspects:<br><br>1. **Liberal Viewpoint**:<br>- **Emphasis on Comprehensive Healthcare**: Liberals generally advocate for comprehensive healthcare coverage and access for all individuals. The emphasis on expanding patient-centered medical home models and investing in primary care aligns with this principle.<br>- **Preventive Care**: The focus on preventive care is also a hallmark of more liberal healthcare policies, which often prioritize preventive measures to reduce long-term healthcare costs and improve public health outcomes.<br><br>2. **Conservative Viewpoint**:<br>- **Private Sector Involvement**: The statement includes "public and private payers," which suggests a role for the private sector in healthcare. Conservatives often favor private sector involvement over purely government-run solutions.<br>- **Innovation in Healthcare Delivery**: The call for new practice models to improve efficiency and coordination in healthcare can resonate with conservative ideals of innovation and efficiency in service delivery.<br><br>In summary, these statements encompass aspects that can be supported by both political ideologies, highlighting a focus on improving healthcare delivery and accessibility, with a role for both public and private entities.	neutral

ACP	Achieving Affordable Health Insurance Coverage for All	2019	The text provided aligns more closely with a liberal viewpoint, particularly in the context of American politics. Here are the key aspects that signify its liberal orientation:<br><br>1. **Support for the Affordable Care Act (ACA):** The text's emphasis on preserving and improving the ACA, a hallmark of liberal healthcare policy in the United States, indicates a liberal stance. Conservatives typically oppose the ACA and advocate for its repeal or significant modification.<br><br>2. **Expansion of Coverage and Protections for Preexisting Conditions:** Advocating for expanded coverage, especially for those with preexisting conditions, is a core principle of liberal health policy. Conservatives often support less regulation in healthcare, focusing more on market-based solutions.<br><br>3. **Federal Funding for State Health Care Redesign:** The call for continued federal support for state initiatives to expand health insurance coverage and improve healthcare delivery aligns with a liberal viewpoint, which often supports more active federal involvement in healthcare.<br><br>4. **Medicaid Expansion and Integration with CHIP:** Proposing Medicaid expansion and the integration with the Children's Health Insurance Program (CHIP) reflects a liberal perspective. Conservatives typically favor more limited government involvement in healthcare and have often opposed Medicaid expansion.<br><br>5. **Tax Credits and Subsidies for Uninsured Working Americans:** The advocacy for advance, refundable, and sliding scale tax credits for uninsured working Americans, and the suggestion to remove income caps for subsidies, align with liberal policies aimed at making healthcare more accessible and affordable.<br><br>6. **Mandates for Coverage Participation:** The suggestion of individual mandates, employer mandates, or automatic enrollment in publicly funded plans is a distinctly liberal approach, reflecting a belief in the government's role in ensuring universal healthcare coverage.<br><br>7. **Requirement for Core Set of Benefits in All Health Plans:** The demand that all health plans include a core set of evidence-based benefits is in line with liberal healthcare policies that emphasize comprehensive coverage.<br><br>Overall, the text's emphasis on government intervention, expansion of healthcare coverage, and support for the ACA and its improvements reflect a liberal viewpoint on healthcare policy.	liberal

ACP	Achieving A High Performance Health Care System with Universal Access	2019	The text you've provided aligns more closely with a liberal viewpoint. Key aspects of this perspective include:<br><br>1. **Universal Health Insurance Coverage**: Advocating for universal health insurance coverage is typically associated with liberal policies. The emphasis on equitable access to healthcare without financial barriers reflects a commitment to social welfare and equity, which are core principles of liberal ideology.<br><br>2. **Single-Payer and Pluralistic Systems**: The discussion of single-payer systems, which involve government as the sole payer of healthcare costs, is a hallmark of liberal health policy. This approach prioritizes equitable access and lower administrative costs, often at the expense of higher taxes and potential limitations on individual choice, which are trade-offs more commonly accepted in liberal thought. The mention of pluralistic systems, involving both government and private entities, also aligns with more progressive, liberal approaches that seek to balance universal coverage with individual choice.<br><br>3. **Focus on Primary Care and Prevention**: Emphasizing primary care services, preventive services, and management of chronic illnesses is consistent with liberal health policy, which often focuses on holistic, long-term health outcomes and preventive care to reduce overall healthcare costs.<br><br>4. **Encouragement of State Innovation and Federal Support**: The recommendation for federal funds to support state-based programs aligns with liberal principles of using government intervention to achieve social goals, in this case, universal healthcare coverage.<br><br>5. **Patient-Centered Health Care and Workforce Policy**: Advocating for patient-centered care and addressing the workforce needs in healthcare, especially in primary care, reflects a liberal approach that emphasizes both the quality of healthcare and the systemic needs of the healthcare industry.<br><br>6. **Reducing Administrative Costs and Supporting Health Information Technology (HIT)**: These recommendations are in line with liberal policies that seek to make healthcare more efficient and accessible, using technology and streamlined processes to improve patient care and reduce burdens.<br><br>7. **Investments in Medical Research**: Encouraging public and private investments in medical research, including comparative effectiveness research, is a position typically associated with liberal health policy, emphasizing innovation and evidence-based improvements in healthcare.<br><br>In summary, these recommendations focus on universal coverage, equitable access, government involvement in healthcare, and a comprehensive approach to health services, all of which are more commonly associated with liberal rather than conservative viewpoints.	liberal
ACP	Insurance of Unemployed and High-Risk	2015	The statement "ACP continues to support appropriate legislative and private sector approaches to provide health insurance coverage to patients who have difficulty obtaining such insurance because of unemployment or health status" generally aligns more with a liberal viewpoint. This is based on a few key aspects:<br><br>1. **Healthcare Accessibility**: Liberal ideologies typically emphasize the role of government in ensuring access to healthcare for all, including those who are unemployed or have pre-existing health conditions. This statement advocates for legislative and private sector solutions to expand health insurance coverage, which aligns with the liberal emphasis on healthcare as a right.<br><br>2. **Support for Vulnerable Populations**: The focus on helping individuals who have difficulty obtaining insurance due to unemployment or health status reflects a liberal concern for social welfare and support for vulnerable groups in society.<br><br>3. **Role of Government**: The mention of legislative approaches suggests a belief in the government's role in addressing healthcare issues, which is more characteristic of liberal than conservative viewpoints. Conservatives typically advocate for less government intervention in the market, including the healthcare sector.<br><br>However, it's important to note that such positions can sometimes find support across the political spectrum, especially when they involve partnerships between the public and private sectors. The specifics of the proposed solutions and their implementation can also shift the alignment more towards one end of the political spectrum or the other.	liberal

ACP	Participation in Managed Care Programs	2015	The statement, "ACP reaffirms its support for legislation allowing patients access to their physician of choice and physician due process for application to and retention within any health care plan," does not align strictly with either a liberal or conservative viewpoint. Instead, it touches on aspects that could be supported by both sides:<br><br>1. **Patient Access to Physicians**: Advocating for patients' rights to choose their physicians aligns with a patient-centered approach to healthcare. This perspective is generally supported across the political spectrum, as it emphasizes patient autonomy and choice.<br><br>2. **Physician Due Process**: Ensuring due process for physicians in healthcare plans relates to fair treatment and job security for healthcare professionals. This concept might be more closely associated with labor rights, which are often championed by liberal viewpoints, but it also aligns with professional autonomy and free market principles, which conservatives might support.<br><br>3. **Healthcare Legislation**: The support for legislation in healthcare can be interpreted in different ways. Liberals often advocate for more government involvement in healthcare to ensure accessibility and fairness, while conservatives typically support market-based solutions and personal choice.<br><br>In summary, this statement reflects principles that are important to both liberal and conservative viewpoints, focusing on patient choice and fairness for healthcare professionals without explicitly endorsing a political ideology.	neutral
ACP	Point-of-Service Legislation	2015	The statement "Legislation should be enacted which mandates a point-of-service option for all those insured under health insurance plans" generally aligns more with a liberal viewpoint. This perspective often supports the idea of government intervention in healthcare to ensure broader access and options for consumers. Liberals typically advocate for more inclusive health care policies and regulations that mandate certain features in health insurance plans to improve access and affordability.<br><br>Conservatives, on the other hand, often prefer less government involvement in healthcare and might view such mandates as overregulation that could increase costs or limit market freedom. They generally advocate for market-based solutions and may oppose mandates that they perceive as limiting consumer choice or increasing government control over healthcare.<br><br>It's important to note, however, that these are general tendencies and there can be a wide range of opinions within each political spectrum. Additionally, specific contexts or details of the legislation could also influence its support among different political groups.	liberal
ACP	Support for the Health Care Infrastructure	2016	The text you provided reflects a viewpoint more aligned with liberal ideologies. This perspective is characterized by an emphasis on government intervention and funding to address disparities in healthcare access, particularly for underserved populations in inner-city and rural areas. The support for federal funding for health programs and the focus on equity in healthcare distribution are hallmarks of liberal health policy principles. Conservatives, on the other hand, typically favor less government involvement in healthcare, emphasizing market-based solutions and individual responsibility over federal funding and mandates. The text's emphasis on national legislation, continuous financial support from the government, and focus on underserved communities distinguishes it from conservative viewpoints, which would likely advocate for more privatized solutions and less federal involvement.	liberal

ACP	Prioritization of Health Care	2016	The text you provided reflects a viewpoint that can align with elements of both liberal and conservative ideologies, depending on the context and specific policies being discussed. Here are some key points from the text and how they might align with different political viewpoints:<br><br>1. **Need to Set Priorities for Health Services**: Both liberals and conservatives recognize the need to prioritize health services due to limited resources. However, they may differ in their approaches to how these priorities should be set. Liberals might emphasize equal access and social equity in setting these priorities, while conservatives might focus more on market-driven solutions and individual choice.<br><br>2. **Broad Participation in Decision Making**: The call for broad participation of all affected sectors of society in establishing health care priorities is a principle that can be endorsed by both ideologies. Liberals typically support inclusive and democratic processes, while conservatives may also see value in involving various stakeholders, particularly if it includes private sector participation.<br><br>3. **Physicians' Leading Role**: Advocating for a leading role of physicians in the health care decision-making process can be seen as a professional stance rather than a strictly political one. It reflects a belief in the importance of expert opinion and could be supported by both sides, albeit with different nuances.<br><br>4. **Evaluation of Various Methods for Priority Setting**: The ongoing evaluation of methods for establishing health care priorities suggests a pragmatic approach, which is not strictly ideological. Both liberals and conservatives might agree on the necessity of such evaluations, though they may prefer different methods based on their underlying principles.<br><br>In summary, the text seems to advocate for a thoughtful, inclusive, and expert-driven approach to health care policy, which can find resonance in both liberal and conservative circles, depending on the specific details and implementations of the policies being discussed.	neutral
ACP	Self Inflicted Illness	2016	The text you provided seems to be a statement from the American College of Physicians (ACP) that supports health care reform legislation. This statement focuses on providing incentives to discourage self-inflicted avoidable illness and promoting health and cost-effective behavior beyond typical preventive measures.<br><br>Evaluating whether this aligns with a liberal or conservative viewpoint can be complex, as health care policy perspectives vary within each political spectrum. However, the emphasis on health care reform and providing incentives for health-promoting behaviors could be seen as aligning more with liberal viewpoints, which generally support more proactive government involvement in health care and preventative measures.<br><br>Conservative viewpoints, on the other hand, often prioritize personal responsibility and limited government intervention in health care, focusing more on market-based solutions and individual choice.<br><br>It's important to note that these are broad generalizations, and there can be considerable variation within each political ideology. Additionally, health care policies often integrate aspects that can appeal to both liberal and conservative perspectives.	probably liberal

ACP	ERISA Preemption	2015	The text you provided outlines policy positions related to the Employee Retirement Income Security Act (ERISA), advocating for:<br><br>1. Self-insured plans to be subject to state-imposed premium taxes funding state risk pool arrangements.<br>2. Self-insured plans to meet state standards restricting capricious and unfair changes in benefit packages.<br>3. Self-insured plans to be subject to state oversight, including penalties, for improper claims processing.<br><br>This text aligns more closely with a liberal viewpoint for several reasons:<br><br>1. **Emphasis on Regulation and Oversight**: The push for more state-level regulation and oversight of self-insured plans is typically associated with liberal ideology, which often advocates for increased government involvement in healthcare and employee benefits to ensure fairness and protect consumers.<br><br>2. **Support for State-Imposed Taxes to Fund Risk Pools**: The idea of using taxes to fund risk pools, which are mechanisms to provide coverage to high-risk individuals, aligns with liberal principles of social welfare and redistribution.<br><br>3. **Focus on Consumer Protection**: The emphasis on preventing "capricious and unfair changes in benefit packages" and ensuring proper claims processing reflects a liberal concern for protecting individuals from potential abuses by insurance companies or employers.<br><br>Conservative viewpoints, in contrast, tend to favor less government intervention in the market, lower taxes, and more individual or private sector control over healthcare and employee benefits. The proposals mentioned in your text would likely be seen by conservatives as adding more government bureaucracy and reducing the flexibility of private companies in managing their self-insured plans.	liberal
ACP	Insurance for Small Employers-Managed Care Programs	2017	The text you've provided outlines a viewpoint that could be seen as aligning more with a liberal perspective, primarily due to its emphasis on federal funding for healthcare and the regulation of managed care organizations. Here are a few key points:<br><br>1. **Support for Federal Funding**: The idea of providing federal funding for states to establish programs that help small employers purchase private health insurance at more affordable rates is more aligned with liberal policies. Liberals generally advocate for more government involvement in healthcare to increase accessibility and affordability.<br><br>2. **Managed Care Organizations for Medicaid**: The acceptance of managed care organizations as a method for delivering medical care to Medicaid recipients can also be seen as a liberal approach. This is because it involves a more regulated and structured approach to healthcare provision, especially for low-income groups.<br><br>3. **National Standards for Quality Assurance**: Advocating for the development of consistent national standards for quality assurance in managed care programs reflects a liberal viewpoint. It suggests a preference for centralized, standardized regulations over a more laissez-faire or market-driven approach typically favored by conservatives.<br><br>4. **Physician Oversight and State Requirements**: The emphasis on physician oversight, especially for programs serving Medicaid recipients, and the requirement for states to adhere to these standards, aligns with a liberal perspective that favors regulatory measures to ensure quality and equity in healthcare services.<br><br>5. **Context of the Organization (ACP)**: The American College of Physicians (ACP), which is referenced in your text, is known for advocating policies that increase access to care and improve public health, aligning with more liberal healthcare policy perspectives.<br><br>It's important to note, however, that healthcare policy can encompass a wide range of views and the delineation between 'liberal' and 'conservative' can sometimes be blurred, depending on specific policy details and the broader political context.	liberal

ACP	Non-Exemption of Government Employees from Health Care Reforms	2015	The statement from the American College of Physicians (ACP) advocating for any changes in the health care delivery system to include all federal civilian government employees, including Congress and the Administration, as well as all government facilities, does not clearly align with a traditional liberal or conservative viewpoint. This position appears more focused on inclusivity and uniformity in health care policy, advocating for a standard that applies equally to all federal employees.<br><br>In the U.S. political landscape:<br><br>- **Liberal Viewpoint**: Generally, liberals advocate for more comprehensive government involvement in healthcare, emphasizing universal coverage and equitable access. They often support policies that expand government-provided or government-funded healthcare.<br><br>- **Conservative Viewpoint**: Conservatives typically favor less government involvement in healthcare, stressing free-market solutions and personal responsibility. They often oppose large-scale government-funded healthcare programs, preferring private sector solutions.<br><br>The ACP's statement, in its emphasis on inclusivity and equal treatment within government health policies, shares aspects with liberal ideals of comprehensive coverage and equality. However, it doesn't explicitly advocate for broader healthcare reforms like universal healthcare or significant government expansion in healthcare, which are commonly associated with liberal policies. The statement could be interpreted as a call for fairness and consistency within the existing system, which might resonate with some conservative perspectives that emphasize equitable application of policies.<br><br>It's important to note that healthcare policy is a complex issue that can transcend traditional political boundaries, and positions on specific aspects of healthcare can vary within each political ideology.	probably liberal
ACP	Provider-Specific Taxes	2015	The text you've provided seems to align more closely with a conservative viewpoint. It expresses opposition to the idea of levying taxes on professional physician services, whether for funding specific healthcare programs or for general revenue enhancement. Generally, conservative perspectives favor lower taxes and less government intervention in business and professional activities. In contrast, liberal viewpoints often support the use of taxation as a means to fund public services, including healthcare. However, it's important to note that political beliefs can be complex and multifaceted, and such a statement might not fully encapsulate the entire ideology of an individual or organization.	conservative
ACP	Cost Containment Measures	2015	The text you've provided outlines the American College of Physicians' (ACP) support for various health care policies and reforms. It's important to note that the ACP is a professional organization focused on internal medicine, and its positions are primarily aimed at improving healthcare outcomes and systems rather than aligning with specific political ideologies.<br><br>However, if we attempt to categorize these views according to traditional liberal or conservative viewpoints in the United States:<br><br>1. **Support for transparency in health care premiums and costs**: This could be seen as aligning more with a liberal viewpoint, as liberals generally advocate for greater transparency and regulation in healthcare to ensure fairness and equity.<br><br>2. **Development of scientific data on managed care techniques**: This is a more technocratic approach, focusing on evidence-based policy, which doesn't neatly align with either liberal or conservative ideologies. Both sides might support this, albeit with different emphases on the role of government versus the market.<br><br>3. **Efforts to reduce health care costs associated with fraud, abuse, and incompetence**: Both liberals and conservatives might agree with this principle, but they might differ on the methods to achieve it. Liberals might focus on regulatory approaches, while conservatives might emphasize market-based solutions and personal accountability.<br><br>4. **Encouragement of wellness care in benefit packages**: This could align more with a liberal perspective, emphasizing preventive care and holistic approaches to health, although wellness programs can also be supported by conservatives for their potential to reduce long-term healthcare costs.<br><br>5. **Development of a Medicare Prospective Payment System (PPS) for hospital capital costs**: This is about efficiency and cost control in Medicare, which could align with conservative ideals about efficient government spending. However, the emphasis on maintaining high-quality care could resonate more with liberal priorities in healthcare.	neutral

			Overall, while there are elements that might resonate more with one political ideology than the other, the ACP's positions are largely centered on improving healthcare efficiency and effectiveness, which transcends simple liberal-conservative categorization.	
ACP	Managed Care in Health Care Reform	2015	The text you've provided outlines the American College of Physicians' (ACP) stance on managed care plans and health insurance legislation. It's not straightforward to label these views as strictly liberal or conservative, as they encompass aspects that might appeal to both political viewpoints.<br><br>1. **Emphasis on Individual Choice and Market Access**: The ACP's support for an individual's right to choose a non-managed care plan and the requirement for insurance carriers to offer managed care plans to both large and small employers suggests a market-oriented approach. This could align with conservative views that typically favor market freedom and individual choice.<br><br>2. **Regulation of Managed Care Plans**: The call for legislation that ensures managed care plans have a sufficient number of providers, provide benefits for out-of-network services in certain cases, and do not penalize primary care physicians with severely ill patients, indicates a desire for regulation in healthcare. This aspect leans more towards a liberal viewpoint, which often advocates for stronger regulation to ensure fairness and accessibility in healthcare.<br><br>3. **Opposition to Unfair Penalties and Taxes**: The opposition to unfair penalties on subscribers who use out-of-plan physicians and the opposition to tax penalties on employers not enrolling in a managed care plan could be seen as a conservative stance, emphasizing less government intervention and financial imposition on individuals and businesses.<br><br>Overall, the ACP's position blends elements that could be seen as both liberal (in terms of advocating for regulatory oversight to protect consumers and ensure accessibility) and conservative (emphasizing individual choice and market freedom). This reflects a more nuanced approach that focuses on practical solutions in healthcare policy rather than strictly adhering to a single political ideology.	neutral

ACP	Payment Issues	2015	The text you provided represents a viewpoint that is generally aligned with conservative principles, particularly in the context of healthcare policy. This stance emphasizes the autonomy of private entities and professionals in setting their fees, opposing government intervention or influence in these matters. Here's a breakdown:<br><br>1. **Opposition to Government Mandates on Private Payers**: The text's opposition to legislative proposals that would require private payers to align their payment structures with public programs like Medicare and Medicaid reflects a conservative viewpoint. Conservatism typically advocates for limited government intervention in business and private sector affairs.<br><br>2. **Physician Autonomy in Setting Fees**: The emphasis on physicians' rights to set their charges and enter into contracts with private individuals or payers without restrictions aligns with conservative values of individual freedom and free-market principles.<br><br>3. **Resistance to Public Program Influence**: The text's resistance to aligning private fee schedules with those of public programs indicates a preference for a healthcare system that is more market-driven, a common conservative stance. Conservatives often argue that government-run or influenced systems can lead to inefficiencies and reduced quality of care.<br><br>4. **Context of the ACP (American College of Physicians)**: The ACP is a professional organization representing internists. Its positions may not always neatly align with political ideologies but are focused on the interests and autonomy of its members. In this case, the position aligns more closely with conservative viewpoints on healthcare policy.<br><br>It's important to note that the application of liberal or conservative labels can sometimes oversimplify complex policy positions. While this stance aligns more with conservative principles, healthcare policy is multifaceted, and organizations like the ACP may adopt positions based on a variety of factors, including the interests of their members, healthcare outcomes, and economic considerations.	conservative
ACP	Reforming the Small Group Insurance	2015	The text you've provided appears to align more closely with a liberal viewpoint. The principles outlined in the statement emphasize the regulation of insurance companies, advocating for community rating, prohibiting medical underwriting, and promoting transparency in insurance rating and renewal practices. These ideas generally align with liberal ideologies that favor increased government regulation and oversight, especially in the healthcare sector, to ensure fairness and protect consumers.<br><br>Conservative viewpoints, on the other hand, tend to favor less government intervention in the market, emphasizing free-market solutions and personal responsibility. The emphasis on state flexibility in developing reinsurance mechanisms could be seen as a nod to conservative principles of state rights and decentralized government, but overall, the text leans more towards liberal policies in its approach to healthcare and insurance regulation.	liberal
ACP	Containing Health Care Costs	2023	The text from the ACP (American College of Physicians) in the FILED HoD Report V expresses a mix of viewpoints that don't align strictly with traditional liberal or conservative ideologies in the United States. The ACP's support for funding outcomes research, development of practice guidelines, medical liability reform, eliminating administrative inefficiencies, and implementing physician payment reform incorporates aspects of both conservative and liberal perspectives.<br><br>Key aspects aligning with a liberal viewpoint include:<br>1. Emphasis on patient access and affordability, especially for low-income patients and those with chronic diseases.<br>2. Support for evidence-based medical care and opposition to using technology assessment to limit new technology development.<br>3. Advocacy for cost sharing modifications to eliminate barriers to essential care.<br><br>Key aspects aligning with a conservative viewpoint include:<br>1. Selective contracting for high-cost, non-emergency procedures as a cost-containment measure.<br>2. Consideration of quality alongside cost in contracting decisions.<br>3. Medical liability reform, which is typically a conservative stance aimed at reducing the burden of malpractice suits on the healthcare system.<br><br>Overall, the ACP's stance seems to balance cost-efficiency and quality of care with patient access and evidence-based practices, indicating a blended approach rather than a clear-cut alignment with either a liberal or conservative viewpoint.	neutral

ACP	How Can Our Nation Conserve and Distribute Health Care Resources Effectively and Efficiently?	2022	The text you've provided outlines principles and guidelines for health resource allocation decisions, emphasizing evidence-based approaches, transparency, public involvement, and ethical considerations. It's not straightforwardly aligned with a traditional liberal or conservative viewpoint as understood in U.S. politics. Instead, it reflects a blend of values and principles that can be found across the political spectrum.<br><br>1. **Evidence-Based Decision-Making**: Both liberals and conservatives might agree on the importance of using clinical and cost-effectiveness data for health care decisions. This approach is consistent with a rational, data-driven perspective that transcends political leanings.<br><br>2. **Transparency and Public Involvement**: The emphasis on a transparent process and public participation aligns with democratic principles valued by both sides. Liberals might particularly appreciate the inclusiveness and focus on public input, while conservatives might support the transparency and accountability aspects.<br><br>3. **Non-Discrimination and Equity**: The principles of non-discrimination and addressing disparities may resonate more with liberal viewpoints, which often emphasize social justice and equity. However, the focus on not discriminating against any class or category of patients is a universal value that conservatives also endorse.<br><br>4. **Fiscal Responsibility and Societal Priorities**: The mention of fiscal responsibility and societal priorities can appeal to conservative values of efficiency and prudent use of resources. Liberals, on the other hand, might focus on the aspects of equitable access and quality of life improvements.<br><br>5. **Flexibility and Regional Variation**: The call for flexibility and accommodation of regional differences could align with conservative preferences for localized decision-making and skepticism of one-size-fits-all approaches.<br><br>6. **Periodic Review and Adaptation**: The idea of periodically reviewing and adapting the allocation process to reflect changing values and evidence is a pragmatic approach that doesn't squarely fit into a liberal or conservative box but instead suggests a willingness to evolve and adapt, a principle that can be appreciated across the political spectrum.<br><br>Overall, this text appears to blend various principles that are important to both liberal and conservative perspectives, focusing on pragmatic, ethical, and evidence-based approaches to health resource allocation.	neutral
ACP	Improving Health Care Efficacy and Efficiency Through Increased Transparency	2017	The text presented aligns more closely with a liberal viewpoint. The emphasis on transparency in healthcare pricing, patient protections, and regulatory interventions to manage healthcare costs and quality suggests a preference for more government involvement and regulation in the healthcare sector. Key aspects of this perspective include:<br><br>1. **Support for Transparency and Consumer Information**: Advocating for clear and accessible information about healthcare costs and quality aligns with liberal ideals that emphasize informed consumer choices and protection against hidden or unexpected costs.<br><br>2. **Legislative Actions for Healthcare Data**: The support for state-level legislation requiring health plans to submit data to a standardized database reflects a liberal inclination towards governmental oversight in healthcare.<br><br>3. **Protection Against Surprise Billing**: The focus on protecting patients from unexpected out-of-network healthcare costs, especially in emergencies, is consistent with liberal priorities of consumer protection and reducing the financial burden of healthcare on individuals.<br><br>4. **Regulatory Measures for Network Adequacy**: Advocating for regulatory oversight to ensure network adequacy in all fields, including emergency care, aligns with the liberal view that government has a role in ensuring the fairness and adequacy of health services.<br><br>5. **Federal and State Efforts to Manage Healthcare Costs**: The text's emphasis on both federal and state-level efforts to manage surprise billing and promote transparency indicates a preference for a multi-level governmental approach, a typical liberal standpoint.<br><br>In summary, the text's focus on government intervention, consumer protection, transparency, and regulatory measures in	liberal

			healthcare is more in line with liberal ideologies, which tend to support a larger role for government in managing and regulating various sectors, including healthcare.	
ACP	Envisioning a Better U.S. Health Care System for All: Coverage and Cost of Care – Universal Coverage	2019	The text you provided outlines recommendations from the American College of Physicians regarding healthcare coverage and policy in the United States. These recommendations align more closely with a liberal viewpoint for several reasons:<br><br>1. **Advocacy for Universal Coverage**: The emphasis on universal healthcare coverage, regardless of a person's place of residence, employment, health status, or income, is a principle more commonly associated with liberal policies. Liberals generally advocate for inclusive healthcare systems accessible to all individuals.<br><br>2. **Single-Payer or Public Choice Models**: The suggestion of either a single-payer system or a publicly financed coverage option alongside private insurance is a hallmark of liberal health policy. Conservatives typically favor market-based approaches and less government involvement in healthcare.<br><br>3. **Elimination of Cost Barriers for High-Value Care**: The focus on removing cost barriers, particularly for low-income patients and those with chronic diseases, resonates with liberal views on healthcare equity and access.<br><br>4. **Income-Adjusted Cost Sharing and Premiums**: The idea of income-adjusted cost-sharing and premium capping is more aligned with liberal ideologies, which often emphasize the need for progressive financial policies in healthcare.<br><br>5. **Standardized Payments to Ensure Access**: The recommendation that payment rates to healthcare providers must be sufficient to ensure access and not perpetuate disparities aligns with liberal concerns about healthcare access and equity.<br><br>6. **Mandatory Coverage and Employer Contribution**: The belief in automatic and mandatory enrollment in healthcare coverage, with requirements for employer contributions, is more typical of liberal policy frameworks.<br><br>7. **Progressive Financing of Healthcare**: The recommendation for financing healthcare through government spending, progressive taxes, and reallocating savings from reduced administrative costs aligns with liberal economic policies.<br><br>8. **Investment in Primary and Comprehensive Care**: The support for greater investment in primary care and preventive health services is a principle that is often emphasized in liberal healthcare policies.<br><br>Overall, these recommendations emphasize universal access, government involvement in ensuring equitable healthcare, progressive financing mechanisms, and the reduction of financial barriers for patients. These are key tenets of liberal healthcare policy as opposed to conservative viewpoints, which typically favor more market-driven approaches, less government intervention, and individual responsibility in healthcare financing.	liberal

ACP	Envisioning a Better U.S. Health Care System for All: Health Care Delivery and Payment System Reforms	2019	The text from "Building the Bridge to More Complete Value-Based Transformation" by the American College of Physicians (ACP) does not squarely align with either a liberal or conservative political viewpoint. Instead, it focuses on healthcare policy recommendations that transcend typical political divisions. Here's a breakdown of how the text aligns with various policy perspectives:<br><br>1. **Physician-led, Team-based Care Models**: This recommendation emphasizes the importance of effective, patient- and family-centered care, a principle that generally receives support across the political spectrum, although the specifics of implementation might vary.<br><br>2. **Variety of Approaches for Delivery and Payment Systems**: Advocating for a range of reform approaches reflects a pragmatic rather than ideologically driven stance. Both conservative and liberal viewpoints can agree on the need for flexibility, though they may differ on the preferred methods.<br><br>3. **Inclusion of Underserved Populations in Value-based Payment Models**: This recommendation aligns more closely with liberal policies that often emphasize inclusivity and addressing disparities in healthcare access. However, the focus on value-based models can also resonate with more fiscally conservative perspectives that favor efficiency and effectiveness in healthcare spending.<br><br>4. **Increased Payments for Primary Care**: The call for increased payments for primary care and reducing disparities in payment levels can be seen as a non-partisan issue, addressing systemic inefficiencies in healthcare.<br><br>5. **Reducing Administrative Complexity**: Both conservative and liberal viewpoints might support reducing administrative burdens in healthcare, though for different reasons. Conservatives might see this as a way to reduce government or bureaucratic influence, while liberals might view it as a means to improve healthcare delivery and access.<br><br>6. **Improving Quality Measurement Accuracy**: The focus on performance measures and methodologies, including patient-centered and evidence-based measures, is generally a healthcare management issue rather than a partisan one.<br><br>Overall, the ACP's recommendations focus on improving healthcare delivery and efficiency, addressing disparities, and promoting evidence-based practices. These goals can be shared by individuals across the political spectrum, though the specifics of how to achieve them might be subject to differing ideological perspectives.	neutral
ACP	Understanding the Fraud and Abuse Laws: Guidance for Internists	2022	The text you've provided appears to be more aligned with a conservative viewpoint, particularly in its emphasis on reducing regulatory burdens and its concern over the perceived overreach of fraud and abuse laws.<br><br>Conservative viewpoints typically favor less government intervention in business and professional practices, arguing that excessive regulation can be counterproductive, stifle innovation, and create unnecessary burdens. The text's focus on the idea that fraud and abuse laws are an "onerous burden" on practicing internists and create a climate of suspicion aligns with this conservative perspective.<br><br>Moreover, the concern that inadvertent billing and coding errors are being treated as fraud suggests a viewpoint that favors protecting professionals from what is seen as overzealous enforcement of regulations. This too is more in line with conservative views that often advocate for a more limited scope of government oversight in professional matters.<br><br>In contrast, a liberal viewpoint might be more inclined to emphasize the importance of strict fraud and abuse laws to protect public interest and might focus more on the benefits of regulation for ensuring ethical practices in healthcare.<br><br>It's important to note, however, that these are broad generalizations, and viewpoints on specific issues like fraud and abuse laws can vary within political ideologies.	conservative

ACP	Safe Harbors and the Stark Ban	2019	The text you've provided from the American College of Physicians (ACP) reflects a position that's primarily focused on ethical practices in medicine and healthcare policy, rather than aligning strictly with a liberal or conservative political viewpoint. The emphasis on educating physicians about ethical responsibilities, monitoring and evaluating the effects of bans on self-referral to clinical laboratories, and ensuring compliance with ethical standards in physician referrals are issues that can be of interest to both liberal and conservative perspectives in different ways.<br><br>Liberals might focus on aspects such as ensuring equitable access to healthcare services and preventing abuses that could harm patient care, while conservatives might be more interested in the aspects related to regulation, ethical business practices, and the autonomy of healthcare providers.<br><br>Overall, the statement seems more aligned with professional ethics in healthcare and the interests of physicians and patients, rather than being distinctly political in nature.	neutral
ACP	Referrals to Facilities in Which Physicians Have a Financial Interest	2015	The text provided doesn't neatly align with a traditional liberal or conservative viewpoint as it primarily addresses ethical standards and regulations in the medical field. However, its emphasis on transparency, regulation, and opposition to conflicts of interest could be interpreted through the lens of both political ideologies.<br><br>From a liberal perspective, the call for strict regulations and oversight on physician referrals and financial interests may resonate with a general preference for regulatory measures in professions to ensure fairness and prevent exploitation. Liberals often support policies that aim to protect consumers and maintain ethical standards in various industries.<br><br>On the other hand, conservatives might also find agreement with certain aspects of this text, especially those that emphasize personal responsibility and ethical conduct in the medical profession. However, they may have reservations about the extent of legislation and regulation proposed, as conservatives generally prefer less government intervention in professional practices and business operations, advocating for market-driven solutions.<br><br>In summary, while this text doesn't squarely fit into a liberal or conservative box, elements of it can be interpreted in ways that resonate with both ideologies, depending on the focus—whether it's on the regulatory aspect (more liberal) or on the personal responsibility and ethical conduct in business (more conservative).	neutral
ACP	The In-Office Ancillary Services Exception	2014	The text provided presents a nuanced perspective that includes elements aligning with both liberal and conservative viewpoints, rather than clearly favoring one over the other.<br><br>From a liberal standpoint, the support for the In-Office Ancillary Services (IOAS) exception under the Stark Self-Referral laws, with safeguards, aligns with a more regulatory and patient-centered approach. The emphasis on ensuring patient adherence, providing convenient access to healthcare services, and promoting the principles of Patient-Centered Medical Homes reflects a focus on comprehensive healthcare access and quality, a priority often associated with liberal viewpoints.<br><br>Conversely, the text also addresses concerns typically found in conservative viewpoints, such as the need to monitor and prevent unnecessary utilization and the potential overuse of services due to physician ownership interests. The call for minimizing administrative burdens on practices and supporting transitions to payment models that align with the value of care (like bundled payments or capitation) can be seen as aligning with conservative preferences for efficiency and cost-effectiveness in healthcare, as well as a tendency to favor market-driven solutions.<br><br>Overall, the text balances regulatory oversight and patient-centered care with concerns about efficiency, cost, and preventing overutilization, reflecting a blended approach that incorporates aspects of both liberal and conservative ideologies in healthcare policy.	neutral

ACP	Health Information Privacy, Protection, and Use in the Expanding Digital Health Ecosystem	2021	The principles outlined by the American College of Physicians (ACP) in this text do not align strictly with either a liberal or conservative viewpoint as traditionally defined in U.S. politics. Instead, they reflect a focus on ethical standards, patient privacy, security of health information, transparency, and accountability in the healthcare sector. These principles are more centered around healthcare policy and ethics rather than broader political ideologies.<br><br>1. **Protection of Personal Health Information**: Emphasizing the privacy and security of personal health information is a concern that crosses political boundaries. Both liberal and conservative viewpoints generally agree on the importance of protecting individual health data, though they might differ on the methods and extent of regulation.<br><br>2. **Transparency and Consent**: Advocating for increased transparency and consent regarding the use of personal health information is a principle that can resonate with both political ideologies. Liberals might focus on individual rights and empowerment, while conservatives might emphasize personal responsibility and informed consent.<br><br>3. **Confidentiality in Medical Care**: The principle that confidentiality is fundamental to medical care is widely accepted across the political spectrum. It aligns with the ethical standards of medical practice rather than with a specific political ideology.<br><br>4. **Incorporation of Privacy in Health IT**: The belief in integrating privacy and security principles in health IT and digital technologies is a forward-looking approach focused on technological ethics and patient safety, transcending traditional political divisions.<br><br>5. **Oversight and Enforcement Outside HIPAA**: Supporting oversight and enforcement for entities not covered by HIPAA reflects a concern for comprehensive regulation in health information privacy. This might align more closely with a liberal perspective favoring regulation, but it also touches on conservative values of accountability.<br><br>6. **Testing New Privacy and Security Measures**: Advocating for the testing and reevaluation of new privacy and security measures in healthcare settings is a principle emphasizing pragmatic and evidence-based approaches, which does not neatly fit into a liberal or conservative category.<br><br>Overall, these principles are more closely related to healthcare policy, ethics, and the responsible use of technology in healthcare rather than being distinctly liberal or conservative in nature.	neutral

ACP	Electronic Health Information Exchange	2019	The principles outlined by the American College of Physicians in this text are centered around health information exchange (HIE), the use of health information technology (HIT), and ensuring security and privacy in accordance with HIPAA. These principles do not neatly align with a purely liberal or conservative viewpoint as they are focused on the technical, legal, and practical aspects of health information management rather than broader political ideologies.<br><br>1. **Support for Electronic Health Information Exchange (HIE)**: This support for safe and secure electronic HIE is a practical approach to healthcare management and technology, transcending traditional political divisions.<br><br>2. **Development of Clear Guidelines and Understanding Legal Implications**: Advocating for clear guidelines and understanding the legal, financial, and workflow implications of HIE is a detail-oriented, administrative approach that doesn't align strictly with a political ideology.<br><br>3. **Technical Specifications for Data Exchange**: The emphasis on consensus for technical specifications, adherence to formal standards, and the use of standardized terminology for data exchange is a technologically progressive stance that prioritizes efficiency and interoperability in healthcare systems.<br><br>4. **Legal Compliance and Advocacy for Uniform Legislation**: The call for legal compliance and advocacy for uniform federal legislation suggests a regulatory approach that could be seen as aligning with a liberal perspective favoring regulation, but it's primarily about standardization and clarity in legal frameworks.<br><br>5. **Practice Redesign for Enhanced Coordination of Care**: Encouraging the development of infrastructure for information management and sharing aligns with modern healthcare models like the patient-centered medical home, focusing on improving quality of care and patient engagement.<br><br>6. **Security & Privacy**: Emphasizing the importance of security and privacy in HIE, with specific attention to areas such as user identification, encryption, and adherence to HIPAA, is a universal concern in the digital age, reflecting a commitment to patient confidentiality and data security.<br><br>In summary, the text represents a policy-oriented, practical approach to health information management, emphasizing technological advancement, legal clarity, and ethical standards in healthcare. These principles are more about effective healthcare administration and less about traditional liberal or conservative political ideologies.	neutral
ACP	E-Health	2019	The text you've provided outlines the general recommendations from the American College of Physicians (ACP) regarding e-Health activities. It's important to note that these recommendations are primarily focused on healthcare policy and practice, rather than aligning with a specific political ideology like liberalism or conservatism.<br><br>However, certain elements of these recommendations can be interpreted through the lens of liberal or conservative viewpoints:<br><br>1. **Liberal Viewpoint Alignment:**<br>- **Emphasis on Accessibility and Equity:** The recommendations focus on ensuring e-Health activities are accessible to all patients, including financially disadvantaged populations and those with low literacy or computer literacy. This aligns with liberal values that prioritize inclusivity and equal access to healthcare.<br>- **Support for Reform and Regulation:** The call for reform of payment policy to compensate physicians for e-Health services, and the emphasis on developing standards for Health IT, may align with liberal tendencies towards regulatory approaches and systemic reform for greater public good.<br><br>2. **Conservative Viewpoint Alignment:**<br>- **Efficiency and Cost-Effectiveness:** Recommendations that highlight improving the efficiency of healthcare and lowering costs through e-Health could align with conservative values that emphasize fiscal responsibility and efficient use of resources.<br>- **Support for Physician Autonomy:** The focus on aiding physician workflows and not burdening physicians with unnecessary regulations could be seen as aligning with conservative values that favor professional autonomy and less government intervention.<br><br>In summary, while the ACP's recommendations primarily address practical aspects of healthcare and e-Health implementation, aspects of these recommendations can be interpreted to align with both liberal and conservative values, depending on the specific focus (e.g., inclusivity and reform vs. efficiency and professional autonomy).	neutral

ACP	Recommendations to Guide the Use of Telemedicine	2015	The text you've provided outlines the American College of Physicians' (ACP) stance on telemedicine. While it is primarily focused on healthcare policy and not explicitly aligned with political ideologies, elements of it can be interpreted through the lens of liberal or conservative viewpoints.<br><br>1. **Liberal Viewpoint Alignment:**<br>- **Emphasis on Accessibility and Equity:** The ACP's support for telemedicine as a way to improve access to care for patients who lack medical expertise in their geographic area and for financially disadvantaged populations aligns with liberal values of inclusivity and equal access to healthcare.<br>- **Support for Federal Funding and Regulation:** The advocacy for ongoing federal funds to support telemedicine infrastructure and the call for telemedicine to adhere to the same standards of practice as in-person visits align with liberal tendencies towards government involvement in ensuring quality and accessibility in healthcare.<br><br>2. **Conservative Viewpoint Alignment:**<br>- **Efficiency and Cost Reduction:** The viewpoint that telemedicine can reduce medical costs and improve health outcomes reflects a conservative perspective valuing efficiency and fiscal responsibility in healthcare.<br>- **Professional Autonomy:** The belief that physicians should use their judgment about the appropriateness of telemedicine, and the recommendation for physicians to ensure telemedicine is secure and compliant with regulations, could resonate with conservative values emphasizing professional autonomy and minimal regulatory overreach.<br><br>In summary, while the ACP's position is primarily focused on practical aspects of telemedicine in healthcare, its various elements can be seen as aligning with both liberal (through its emphasis on accessibility, equity, and government support) and conservative (through its focus on efficiency, cost-effectiveness, and professional autonomy) viewpoints, depending on the specific focus of the recommendations.	neutral
ACP	Controlling Health Care Costs: Enhance Use of Health Information Technology	2023	The text you've provided seems to reflect a viewpoint that could be aligned with either liberal or conservative ideologies, depending on specific interpretations and contexts. Here's a breakdown:<br><br>1. **Incentivizing Health Information Technologies**: This point advocates for payment policies that incentivize the use of health information technologies in medical care. Both liberal and conservative viewpoints can support this, but the emphasis might differ:<br>- **Liberal Viewpoint**: Might focus on how such technologies can improve overall healthcare quality and accessibility, particularly for underserved communities.<br>- **Conservative Viewpoint**: Might emphasize the efficiency and cost-effectiveness of such technologies, potentially reducing overall healthcare spending.<br><br>2. **Support for Primary Care Practices**: The suggestion of providing technical support, training, and funding to primary care practices, especially smaller ones, to acquire health information technologies aligns with a more proactive government role in healthcare.<br>- **Liberal Viewpoint**: Generally favors government intervention and support in healthcare, viewing it as a way to ensure equity and quality in healthcare services.<br>- **Conservative Viewpoint**: While traditionally conservative views might be wary of increased government spending and intervention, there is also a conservative argument for supporting primary care as a means of reducing long-term healthcare costs and improving efficiency.<br><br>The mention of Patient-Centered Medical Homes (PCMHs) is indicative of a focus on quality and patient-centered care, a principle that can be embraced by both ideologies.<br><br>In summary, while the text does have elements that could be more readily associated with a liberal viewpoint, such as the emphasis on government support and intervention in healthcare, there are aspects that could be appealing to conservative perspectives as well, especially in terms of efficiency and cost-effectiveness. Political viewpoints often overlap in areas like healthcare, where the end goals (improved care, efficiency, accessibility) can be shared, even if the means of achieving them are debated.	neutral

ACP	Availability of Insurance Coverage Information to Patients	2022	The policy statement from the American College of Physicians (ACP) regarding the requirement for health insurance providers and third-party administrators to maintain a 24-hour communication line for information about coverage and benefits doesn't inherently align with a traditional liberal or conservative viewpoint. Instead, it focuses on improving healthcare access and transparency, which can be a concern across the political spectrum. Here's how it might be perceived from different viewpoints:<br><br>- **Liberal viewpoint:** Those with a liberal viewpoint might support this policy as it aligns with principles of increased healthcare access, patient rights, and transparency. Liberals generally advocate for stronger healthcare protections and might see this as a step towards a more informed and empowered patient base.<br><br>- **Conservative viewpoint:** Conservatives might have mixed views. Some might appreciate the focus on patient empowerment and market-based solutions to healthcare issues, viewing the policy as a way to improve competition and customer service among insurance providers. Others might oppose it if they see it as additional regulation on businesses or an overstep of government authority in private industry.<br><br>Ultimately, this policy is more about practical healthcare administration and patient access to information than it is about broader political ideologies. It could potentially garner support from both sides for different reasons, or face scrutiny based on how it's implemented and the perceived impact on insurance companies and healthcare providers.	neutral
ACP	Consumer-Directed Health Care and Health Savings Accounts (HSAs)	2015	The text provided aligns more with a liberal viewpoint, particularly evident in its emphasis on universal healthcare access, the protection of low-income individuals, and the call for comprehensive reforms in health care. It suggests a cautious approach to Health Savings Accounts (HSAs), recognizing them as one component in a broader strategy for health care reform rather than a standalone solution. The recommendations reflect a liberal perspective by emphasizing the need for:<br><br>1. **Universal Health Care Access**: Asserting that HSAs alone won't achieve universal access to health care is a stance often associated with liberal ideology, which typically supports more comprehensive health care reforms.<br><br>2. **Protective Measures for Low-Income Patients**: The call for safeguards for those with lower incomes aligns with liberal priorities of equity and support for vulnerable populations.<br><br>3. **Monitoring and Regulation**: The emphasis on continued monitoring, safety nets, and consumer protection is consistent with a liberal viewpoint that values regulation and oversight to ensure fairness and prevent harm to disadvantaged groups.<br><br>4. **Expansion of Public Programs**: The support for expanding Medicaid, S-CHIP, and other public programs reflects a liberal inclination towards government involvement in providing a safety net for the poor.<br><br>5. **Tax Credits and Incentives**: Proposing advanced refundable tax credits for lower-income uninsured Americans is a policy often supported by liberals as a way to make health care more accessible and affordable for all.<br><br>6. **Employer-based Coverage**: The emphasis on not eroding existing employer-based coverage and making HSAs attractive to small employers suggests a balance between market-based solutions and maintaining traditional insurance models, which is a nuanced position often found in liberal policies.<br><br>7. **Informed Consumer Decision-making**: Advocating for valid, reliable information and decision-support tools aligns with a liberal viewpoint on the importance of consumer protection and empowerment.<br><br>While the text does not outright reject conservative ideas like HSAs and consumer-directed health care, it frames them within a broader context of reforms and safeguards typically advocated by liberal policymakers. It suggests a multifaceted approach to health care reform that includes elements of market-based solutions while emphasizing the need for universal access, protection for low-income individuals, and comprehensive reforms, all of which are more traditionally aligned with liberal views.	liberal

ACP	Timely Payment on Claims	2017	The statement regarding the American College of Physicians (ACP) supporting legislation that requires prompt payment of physicians' clean claims within thirty days doesn't inherently align with a strictly liberal or conservative viewpoint. Both ideologies might find aspects to support or contest based on broader contexts:<br><br>- **Liberal Viewpoint**: Liberals might support this policy as part of a broader agenda for healthcare reform, emphasizing fair compensation, efficiency, and the rights of healthcare workers. They may view it as a step towards improving healthcare systems and ensuring that medical professionals are supported and incentivized to provide quality care.<br><br>- **Conservative Viewpoint**: Conservatives might appreciate the emphasis on efficient, prompt business practices and the potential reduction of bureaucratic hurdles. They may view it as a measure that supports small medical practices and promotes free-market principles by ensuring contracts and payments are honored promptly.<br><br>Both sides might also have concerns:<br><br>- **Liberals** might worry if such policies favor large healthcare providers or insurers disproportionately or lead to unforeseen complexities in healthcare delivery.<br><br>- **Conservatives** might be cautious about the potential for increased government regulation in private transactions or if it might lead to unnecessary bureaucratic oversight.<br><br>Ultimately, the alignment of this statement with liberal or conservative viewpoints can vary significantly based on individual beliefs, the specific context of the healthcare system in question, and how the policy interacts with other aspects of healthcare and economic regulation.	neutral
ACP	Voluntary Purchasing Pools: A Market Model for Improving Access, Quality, and Cost in Health Care	2017	The position paper from the American College of Physicians (ACP) on voluntary purchasing pools in the health care sector doesn't align strictly with a liberal or conservative viewpoint. Instead, it combines elements from both ideologies.<br><br>From a liberal perspective, the paper emphasizes increasing access to health care for small groups and individual persons, reducing administrative costs, and maintaining quality in a market dominated by corporate managed care. These goals reflect a liberal inclination towards universal health care coverage and addressing disparities in health care access. The support for standardizing benefit packages and community rating rules, as well as making purchasing pools available to low-income and underserved persons, aligns with a liberal agenda of ensuring equity and comprehensive coverage in health care.<br><br>From a conservative viewpoint, the recommendations support the concept of market-based solutions to health care issues by advocating for voluntary purchasing pools as a mechanism to increase competition and choice in the health care market. It suggests limiting the authority of purchasing groups to negotiate price unless a broad choice of health plans is offered, which resonates with conservative ideals of free market principles and limited government intervention. The call for performance-based contracting and minimizing political appointments to the boards of state-operated purchasing pools also reflects a conservative preference for efficiency and reduced government size.<br><br>In summary, the paper incorporates a blend of ideas from both liberal and conservative ideologies, aiming to create a market-based health care solution that also addresses concerns of access, cost, and quality. It's a pragmatic approach that seeks to balance competition and choice with the need for standardization and access to health care for all.	neutral

ACP	Small Business Pooling Arrangements and Association Health Plans (AHPs)	2015	The text presents policy recommendations from the American College of Physicians (ACP) regarding healthcare and insurance regulation. These recommendations include support for small business group purchasing, consumer protections, federal regulatory structures, state authority in insurance regulation, broad membership in purchasing pools, and alternative proposals to association health plans.<br><br>This text doesn't neatly align with a traditional liberal or conservative viewpoint as defined in U.S. politics. Instead, it incorporates elements that might be favored by both sides. For instance:<br><br>1. **Liberal Viewpoint Elements:**<br>- Advocacy for strong consumer protections and a comprehensive federal patient bill of rights.<br>- Support for essential health benefits and coverage for preventive and primary care.<br>- The suggestion to allow small businesses to buy into Medicaid or CHIP for employee coverage, which reflects a preference for expanding public health options.<br><br>2. **Conservative Viewpoint Elements:**<br>- The emphasis on market-based solutions and group purchasing to provide more options and potentially lower costs.<br>- Support for state authority in regulating insurance, traditionally a conservative preference for state over federal control.<br><br>The recommendations appear to seek a middle ground, advocating for robust consumer protections (often a liberal priority) while also promoting market-based solutions and state authority (commonly conservative stances). The ACP's position seems to reflect a pragmatic approach focused on practical outcomes and improvements in healthcare access and quality, rather than a strictly ideological one.	neutral
ACP	Concurrent Care	2016	The text you've provided appears to be a statement from the American College of Physicians (ACP) concerning healthcare policy, specifically regarding the recognition of medical subspecialties and the classification of internal medicine physicians in relation to family practice and general practice physicians within Medicare and Medicaid services.<br><br>This statement doesn't neatly align with a traditional liberal or conservative viewpoint as it primarily focuses on medical administration and specialist recognition. However, perspectives on healthcare policy can be influenced by broader political ideologies:<br><br>- **Liberal Viewpoint:** Generally, liberals advocate for a more extensive role of government in healthcare to ensure access and equity. They might support the differentiation of specialties to ensure quality care and proper reimbursement. Liberals might view the ACP's stance as a move toward more comprehensive and accurate healthcare services.<br><br>- **Conservative Viewpoint:** Conservatives typically favor less government intervention in healthcare, emphasizing market-driven solutions. They might support the distinction between specialties if it leads to more efficient and cost-effective healthcare delivery. However, they might be cautious about potential increases in administrative complexity and costs.<br><br>Ultimately, the ACP's statement is more professional and technical than political. It's concerned with the practicalities of medical service delivery rather than broader ideological debates. Both liberals and conservatives might agree or disagree with the specifics based on their views on healthcare policy, efficiency, and the role of government in medicine.	neutral

ACP	Principles on Preadmission Review Programs	2017	The text you've provided outlines principles for preadmission review programs as endorsed by the American College of Physicians (ACP) with modifications to the American Medical Association (AMA) principles. It's not strictly aligned with a liberal or conservative viewpoint as it focuses on medical policy and patient care procedures. However, the principles can be interpreted through different political lenses:<br><br>1. **Liberal Viewpoint**: A liberal perspective might appreciate the emphasis on immediate hospitalization without prior authorization in urgent cases, suggesting a patient-first approach and advocating for fewer bureaucratic hurdles in emergency care. The call for physician oversight and the appeal process might also be seen as promoting quality care and physician autonomy, aligning with a liberal emphasis on individual rights and protections.<br><br>2. **Conservative Viewpoint**: A conservative might focus on the aspects related to preventing government or payer mandates that do not improve care quality, suggesting a stance against overregulation. The mention of charging for physician time and effort might also resonate with conservative values of fair compensation and market-based solutions to healthcare.<br><br>Ultimately, the text seems to strive for a balance between efficient healthcare management and patient care quality, which can align with values across the political spectrum. The specific alignment might depend on how individual elements are emphasized or interpreted within the broader context of healthcare policy debates.	neutral
ACP	Preadmission Testing	2022	The text outlines a stance from the American College of Physicians (ACP) regarding the use of preadmission testing (PAT) to reduce inpatient hospital costs and encourages acceptance of this practice by hospitals and insurers. This viewpoint doesn't neatly align with traditional liberal or conservative ideologies as it encompasses aspects relevant to both:<br><br>1. **Cost Reduction and Efficiency (Common Conservative Traits)**: Conservatives generally favor market-driven solutions, efficiency, and cost reduction in healthcare. The advocacy for preadmission testing to reduce inpatient costs aligns with the conservative principle of making healthcare more efficient and affordable.<br><br>2. **Quality and Accessibility of Healthcare (Common Liberal Traits)**: Liberals tend to focus on improving the quality and accessibility of healthcare. By promoting preadmission testing by qualified practitioners in out-of-hospital settings, the ACP is advocating for broader access to healthcare services and potentially improved patient outcomes, which are concerns often emphasized in liberal healthcare policies.<br><br>However, the proposal doesn't inherently favor one ideology over the other. It's more focused on pragmatic healthcare policy improvements rather than ideological principles. Both liberals and conservatives might find aspects of the policy agreeable, depending on their specific priorities and beliefs about how to best achieve cost-effective, quality healthcare.	neutral
ACP	Core Principles on Financing	2022	The text provided outlines principles and recommendations for healthcare financing, focusing on ensuring adequate and predictable financing for critical access institutions, fair reimbursement for services, and progressive financing for public health insurance programs. This perspective aligns more closely with a liberal viewpoint for several reasons:<br><br>1. **Emphasis on Equity and Access**: The text prioritizes eliminating barriers to care and ensuring adequate funding for institutions serving underserved areas. This focus on healthcare equity and accessibility is a common theme in liberal ideologies, which often advocate for stronger safety nets and support systems for marginalized communities.<br><br>2. **Progressive Financing**: The suggestion that financing for public programs should be progressive, where individuals with higher incomes contribute more, is a hallmark of liberal economic policy. It reflects a commitment to reducing income inequality and ensuring that social services are funded in a way that is equitable and reflects the ability to pay.<br><br>3. **Opposition to Means-Testing**: The text's discouragement of explicit means-testing for access to programs aligns with a liberal perspective that advocates for universal access to essential services like healthcare. Liberals often argue that means-testing can create unnecessary barriers and stigmatize recipients, preferring universal or broadly accessible programs instead.<br><br>While these viewpoints generally align with liberal ideologies, it's important to note that the terms "liberal" and "conservative" can vary significantly in meaning based on the country and specific context. Moreover, some conservatives may also support aspects of these policies, especially if they are seen as solutions to improve efficiency or if they align with certain conservative values like local control and community support. However, based on the primary focus on equity, progressive financing, and broad access, the text leans more towards a liberal viewpoint.	liberal

ACP	Core Principles on Patient Rights, System Accountability, and Professionalism	2022	The text you've provided outlines a series of health reform proposals focusing on accountability, quality, evidence-based decision-making, innovation, patient rights, and professional responsibility. It doesn't align strictly with a liberal or conservative viewpoint as traditionally defined in U.S. politics. Instead, it blends elements that might be supported by both sides.<br><br>1. **Liberal Viewpoints**:<br>- **Emphasis on Patient Rights and Protections**: The focus on patient rights, such as the right to appeal denials of coverage and to have confidential health information protected, aligns with liberal priorities of consumer protection and individual rights in healthcare.<br>- **Accountability and Quality**: Liberals often support reforms that increase the quality of care and ensure that health care providers are held accountable for the care they provide, which is reflected in the proposal.<br><br>2. **Conservative Viewpoints**:<br>- **Evidence-Based Decision Making**: The emphasis on using clinical effectiveness as a basis for medical decisions and insurance coverage might resonate with conservatives who prefer market-based, efficient solutions and often advocate for reforms that are based on performance and results.<br>- **Innovation and Improvement**: Encouraging innovation, especially in health information technologies, aligns with a conservative preference for market-driven solutions and technological advancement to improve efficiency and reduce costs.<br><br>3. **Bipartisan or Neutral Elements**:<br>- **Professional Responsibility**: Calling for the medical profession to embrace reforms and uphold high ethical standards is a universally appealing principle that doesn't necessarily align with one political ideology.<br>- **Partnership in Designing Reforms**: Advocating for collaboration among government, business, and other stakeholders is a more neutral or bipartisan approach, recognizing the multifaceted nature of healthcare reform and the need for varied perspectives.<br><br>In summary, the text presents a nuanced approach to health reform, incorporating principles of accountability, patient rights, evidence-based practice, and professional responsibility. While certain aspects might be more favored by one political ideology or the other, the overall proposal doesn't neatly fit into a liberal or conservative box but rather reflects a comprehensive approach to healthcare improvement.	neutral
ACP	Federally Qualified Health Centers	2022	The statement about the American College of Physicians (ACP) studying the promotion of expanding Federally Qualified Health Centers (FQHCs) to decrease healthcare disparities and improve access for the medically underserved aligns more closely with liberal viewpoints. Generally, liberal ideologies tend to support increased government intervention and funding in healthcare to ensure access and equity. The focus on expanding FQHCs, which are often funded by the federal government to serve underserved areas and populations, fits within this framework by aiming to reduce disparities and improve healthcare access. Conservative viewpoints typically emphasize less government involvement and more market-based solutions, so while some conservatives might support FQHCs for their community-based approach and potential cost-efficiency, the idea of expanding them as a federal initiative might not align as closely with conservative priorities.	liberal
ACP	Health Insurance Consolidation	2015	The statement you've provided from the American College of Physicians (ACP) expresses opposition to the consolidation of health insurance companies, citing concerns about increased costs, reduced choice, and negative impacts on access to healthcare and physician negotiation. This viewpoint aligns more closely with liberal perspectives in several ways:<br><br>1. **Focus on Regulation and Oversight**: Liberals generally advocate for more regulation and oversight of industries to protect consumers. The ACP's statement against consolidation due to its negative effects on cost and choice reflects a desire for regulatory measures to prevent monopolistic practices, a stance more typical of liberal ideology.<br><br>2. **Emphasis on Access and Affordability**: Liberals often prioritize ensuring access to healthcare and maintaining its affordability. The ACP's concerns about increased costs and decreased choices for patients and employers echo liberal priorities of keeping healthcare accessible and affordable for all.<br><br>3. **Support for Collective Negotiation**: The mention of preserving physicians' ability to negotiate over health services suggests support for collective bargaining, which is traditionally more aligned with liberal values that emphasize labor rights and protections against large corporate entities.<br><br>It's important to note that while these points suggest a more liberal alignment, healthcare policy is complex, and individuals or organizations may hold views that don't neatly fit into a liberal or conservative box. Additionally, both liberals and conservatives	liberal

			might share concerns about the negative impacts of market consolidation in healthcare, albeit for different reasons and with different proposed solutions.	
ACP	Individual Mandate	2022	The text you've provided outlines a series of recommendations for health care reform, focusing on individual mandates, benefit packages, employer contributions, enforcement mechanisms, insurance market reforms, and strengthening the primary care workforce. The viewpoints expressed in these recommendations do not align strictly with traditional liberal or conservative ideologies, but rather incorporate elements from both, reflecting a more centrist or pragmatic approach to health care policy.<br><br>Here's a breakdown:<br><br>1. **Liberal Viewpoints:**<br>- The emphasis on ensuring that every legal resident has access to affordable and quality health care aligns with liberal ideologies, which often advocate for broader access to health care as a right.<br>- The recommendation for sufficient federal funding to subsidize private health insurance or public programs is also a common liberal stance, as it involves government intervention and support to ensure health care access.<br>- The call for reforms to the insurance market, including no exclusions for pre-existing conditions and modified community rate setting, reflects liberal priorities for a more regulated and equitable insurance system.<br><br>2. **Conservative Viewpoints:**<br>- The focus on individual mandates could be seen as aligning with conservative principles of personal responsibility, where individuals are required to obtain health insurance rather than depending solely on government-provided care.<br>- The idea that employers should provide health insurance coverage or contribute to a fund rather than having a government-only approach might resonate with conservative views favoring private sector solutions.<br><br>3. **Centrist or Pragmatic Elements:**<br>- The recommendations seek a balanced approach, incorporating both market-based solutions (like individual mandates and employer contributions) and government interventions (like subsidies and insurance reforms).<br>- The call for an expert advisory panel, including primary care physicians, to determine a core set of benefits suggests a pragmatic approach to defining essential health services based on expert advice rather than purely ideological grounds.<br><br>In summary, these recommendations represent a blend of liberal and conservative ideas, aiming for a comprehensive and balanced approach to health care reform. They reflect a pragmatic understanding that effective health care policy often requires elements from across the ideological spectrum to ensure access, affordability, quality, and sustainability.	neutral

ACP	Public Plan Option	2022	The text you've provided outlines specific policy recommendations regarding a public health insurance option within a health insurance exchange, focusing on equal treatment of public and private plans, reforms to improve care quality and efficiency, and financial safeguards. It doesn't explicitly align with a traditional "liberal" or "conservative" viewpoint as these terms are broadly understood in U.S. politics. However, it can be analyzed for alignment based on the policy content:<br><br>### Liberal Viewpoint Indicators:<br>- **Support for Public Health Option**: Generally, liberals in the U.S. are more supportive of government intervention in healthcare. The text's support for a public plan option aligns with this.<br>- **Comprehensive Reforms**: The emphasis on comprehensive reforms, including income-related subsidies and an essential health benefits package, aligns with liberal priorities of broad access and equity in healthcare.<br>- **Regulatory Oversight**: The desire for regulatory oversight and rules to ensure the public plan competes fairly with private plans is a stance often associated with liberal governance philosophies.<br><br>### Conservative Viewpoint Indicators:<br>- **Marketplace Solutions**: While conservatives typically prefer less government involvement, the text's support for a health insurance exchange (marketplace) can resonate with conservative ideals of market-based solutions and competition.<br>- **Voluntary Participation**: The emphasis on voluntary participation for individuals and providers and competitive physician payments might appeal to conservatives who favor less compulsory government involvement and more free-market principles.<br><br>### Neutral or Bipartisan Elements:<br>- **Quality and Efficiency**: Focus on care coordination, positive health outcomes, and cost-effectiveness are broadly appealing and can be seen as technocratic or pragmatic rather than inherently political.<br>- **Innovative Payment Models**: Encouraging innovation through patient-centered medical homes and value-based care is a goal that might find support across the political spectrum.<br><br>### Conclusion:<br>The text presents a nuanced approach that doesn't neatly fit into a liberal or conservative box. It contains elements that might be supported by both sides, such as market-based solutions and quality improvements, while also advocating for a public option and comprehensive reforms often associated with liberal politics. The exact alignment might depend on the specific priorities and beliefs of individuals within the liberal-conservative spectrum.	neutral
ACP	Reforming the Tax Exclusion for Health Insurance	2022	The text appears to align with a moderate to liberal viewpoint. It emphasizes comprehensive and portable health coverage for all Americans, a key liberal value focusing on universal healthcare access and equity. The suggestion to cap the income tax exclusion for employer-sponsored health insurance (ESI) indicates a desire to control costs and promote fairness, aligning with liberal ideals of progressive taxation and equitable resource distribution.<br><br>The recommendations aim to balance cost containment with the protection of low- and moderate-income workers and to ensure that changes do not incentivize employers to drop coverage, reflecting a nuanced approach to reform. This blend of cost control and protection for vulnerable groups is characteristic of a center-left policy stance, seeking to reform the system in a way that maintains coverage while also addressing inefficiencies and inequities.<br><br>However, the focus on incentives for individuals to choose cost-effective plans and the consideration of variations in health status and regional costs may also resonate with more conservative principles of market-driven solutions and individual responsibility. The text doesn't strictly adhere to a single ideological viewpoint but rather incorporates elements from both liberal and conservative perspectives, aiming for a balanced and comprehensive approach to health care reform.	neutral

ACP	Community Rating for Health Insurance	2022	The text you've provided outlines a position from the American College of Physicians (ACP) supporting community rating for health insurance and opposing experience-rating. Community rating means that insurers charge the same rate to all enrollees in a given area, regardless of their health status, age, or other factors. Experience-rating, on the other hand, allows insurers to set premiums based on an individual's or group's health status, age, and other risk factors.<br><br>Generally, the support for community rating over experience-rating aligns more closely with liberal viewpoints. Liberals often advocate for policies that promote equal access to healthcare, emphasizing the need for affordability and inclusivity. Community rating is seen as a way to ensure that everyone, regardless of their health status or age, has access to health insurance at a reasonable cost. This approach is in line with the liberal emphasis on social equity and support for regulations that prevent discrimination based on health status.<br><br>Conservatives, on the other hand, tend to favor less regulation and more market-based solutions. They might argue that experience-rating allows for more competition and efficiency, as it enables insurers to price policies according to risk, potentially lowering costs for healthier individuals. This stance is often grounded in a belief in individual responsibility and the idea that people should have the freedom to choose from a variety of insurance options tailored to their specific needs and risk profiles.<br><br>However, it's essential to note that these are general trends and not absolute. Individuals and organizations may hold a mix of views, and positions can vary based on specific policy details, regional preferences, and other factors.	liberal
ACP	Employer Opt-Out of Benefit Requirements	2014	The text aligns more closely with a liberal viewpoint. It emphasizes the importance of universal coverage and access to preventive healthcare services, advocating for requirements that all insurance plans cover a comprehensive, evidence-based health benefit package. This perspective is commonly associated with liberal ideology, which often prioritizes broader access to healthcare and government involvement in ensuring that access. The text also highlights concerns about under-insurance, health disparities, public health, and the potential for discriminatory practices, which are issues often emphasized in liberal discourse. It advocates against allowing employers to opt out of providing certain health benefits, which could lead to inequalities in healthcare access. This emphasis on equality and comprehensive coverage is more aligned with liberal principles, which tend to favor stronger regulations to promote social welfare and public health.	liberal
ACP	Insurers to Cover Hepatitis B Immunization	2008	The statement about the American College of Physicians (ACP) supporting federal legislation mandating insurance coverage for medically appropriate Hepatitis B immunization aligns more closely with a liberal viewpoint. Liberal ideologies generally advocate for more government involvement in healthcare to ensure broader access and coverage. They tend to support mandates and regulations that require insurance companies to cover various medical treatments and preventative measures, like vaccinations.<br><br>On the other hand, conservative viewpoints typically emphasize less government intervention in healthcare and more market-based solutions. Conservatives might argue against mandates on insurance companies, preferring individual choice and market competition to determine coverage options.<br><br>It's important to note, however, that viewpoints on specific issues like healthcare can vary within each ideological group, and some conservatives might support this measure for public health reasons. Similarly, some liberals might oppose it based on concerns about government overreach or other factors. Therefore, while the statement generally aligns more with liberal ideologies, it doesn't exclusively represent all individuals within that group.	liberal
ACP	Number of Medical Opinions	2015	The statement advocates for managed care and insurance programs to not limit the number of medical opinions a patient may obtain, suggesting that coverage should be based on medical necessity and appropriateness, judged individually. This perspective aligns with principles commonly associated with both liberal and conservative viewpoints in different ways:<br><br>1. **Liberal Viewpoint**: Typically, liberals advocate for comprehensive healthcare access and might support the idea that patients should have the freedom to seek multiple medical opinions to ensure quality care. They often emphasize individual healthcare rights and might agree with the notion that medical necessity and appropriateness should determine access to services rather than arbitrary limitations.<br><br>2. **Conservative Viewpoint**: Conservatives might also agree with the statement, but from the perspective of limiting government or insurer control over healthcare decisions. They often support personal responsibility and freedom in healthcare choices and might see the avoidance of arbitrary restrictions as a way to promote efficiency and individual choice in the healthcare market.<br><br>However, the statement is quite nuanced and doesn't strictly fall into a partisan viewpoint. It advocates for a balanced approach, recognizing the need for some regulation (not allowing arbitrary restrictions) while also emphasizing individualized assessment	neutral

			(medical necessity and appropriateness), which could resonate with a broader spectrum of beliefs. The specific stance one takes might depend on how they interpret and prioritize the values of freedom, responsibility, and the role of regulation in healthcare.	
ACP	Emergency Circumstance Fee	2015	The text from the American College of Physicians (ACP) suggests advocating for broader insurance coverage for medical services outside of standard hours, especially under emergency circumstances. This viewpoint doesn't align strictly with a liberal or conservative ideology as it focuses on healthcare access and insurance policy rather than broader political ideologies.<br><br>Liberal perspectives typically emphasize broad access to healthcare, advocating for comprehensive coverage and support for various medical services. They might support this statement as it pushes for more inclusive insurance coverage, ensuring patients receive care when needed, regardless of the time.<br><br>Conservative perspectives generally emphasize personal responsibility, market-driven solutions, and limited government intervention in healthcare. Some conservatives might agree with the statement if they believe it encourages efficiency and responsiveness in the healthcare system. Others might view it as potential overreach or unnecessary regulation.<br><br>In essence, the statement can resonate with values across the political spectrum, depending on how it's interpreted and the broader context of the healthcare debate. It's more about practical healthcare delivery and ensuring coverage during emergencies than about ideological divisions.	neutral
ACP	Core Principles on Health Insurance Coverage	2022	The text presents viewpoints that align more closely with a liberal perspective. Here's a breakdown of how the key points fit within the broader liberal vs. conservative spectrum:<br><br>1. **Universal Health Coverage and Single-Payer or Publicly Financed Systems**: Advocating for universal health coverage and considering a single-payer system or a public option alongside private insurance are typical liberal positions. Liberals often emphasize the right to healthcare regardless of one's circumstances and advocate for the government's role in ensuring access.<br><br>2. **Mandatory Enrollment and Incentives for Participation**: The idea of automatic and mandatory enrollment in health insurance programs, along with incentives to ensure participation, aligns with liberal policies favoring broader healthcare access and the belief that healthcare is a right. Conservatives typically prefer less government intervention and more personal choice in healthcare.<br><br>3. **State Flexibility with National Standards**: Allowing states to explore different healthcare solutions while adhering to national standards reflects a liberal inclination towards innovation and diversity in policy implementation, but with a federal safety net to ensure certain rights and access standards are met nationwide.<br><br>4. **Making Prescription Drugs More Affordable**: Advocating for measures to reduce prescription drug costs and opposing barriers that prevent access to necessary medications is a stance commonly seen in liberal policy. This viewpoint emphasizes the need for regulation and intervention to control costs and ensure access, contrasting with the conservative preference for market-based solutions and less regulation.<br><br>In summary, the text's emphasis on universal coverage, government involvement in ensuring access and affordability, and regulation of the healthcare industry are typically associated with liberal ideologies. Conservative viewpoints, on the other hand, would likely emphasize market solutions, personal responsibility, and limited government intervention in healthcare.	liberal

ACP	Establishing Benchmarks for Reasonable Health Insurance Administrative Costs	2022	The statement about establishing benchmarks for health insurance administrative costs and guidelines for spending on patient care aligns with principles that can be found in both liberal and conservative ideologies, but it's often more associated with a liberal viewpoint. Here's why:<br><br>1. **Liberal Viewpoint**: Liberals generally advocate for more regulation and oversight of the healthcare industry to ensure that funds are used effectively for patient care and to control costs. They often support measures that aim to increase transparency and efficiency within the system. The idea of establishing benchmarks and guidelines for administrative costs and patient care spending reflects a desire to regulate and optimize the industry for the benefit of the consumers, a common theme in liberal healthcare policies.<br><br>2. **Conservative Viewpoint**: Conservatives typically favor a free-market approach to healthcare, arguing that less government intervention leads to more competition and innovation, ultimately benefiting patients. They might support benchmarks and guidelines if they believe these measures will lead to greater efficiency without imposing excessive regulatory burdens. However, conservatives might be wary if they feel such measures are a step toward too much government control over the private sector.<br><br>The context and details of how the benchmarks and guidelines are to be implemented would further determine the alignment. If the approach is heavy on government regulation and oversight, it's more likely to be seen as liberal. If it's more about providing tools and frameworks for the industry to self-regulate and enhance market efficiency, it might be more palatable to conservative ideologies.	neutral
ACP	Requiring Healthcare Bills to be Uniform and Written so that Patients with Average Health Literacy Can<br>Understand Them	2022	The text you've provided represents a viewpoint advocating for regulation and legislation to ensure clarity and understandability in healthcare billing. This viewpoint aligns with principles that are generally considered liberal. Liberal ideologies often support the role of government in regulating industries to protect consumers and ensure fairness. In this case, advocating for legislation to make healthcare billing more transparent and understandable reflects a desire for government intervention to protect patients' rights and ensure they are well-informed. Conservatives might support the idea of transparency but are generally less supportive of increased regulation and government intervention in private industries, including healthcare. Therefore, while the specific alignment can vary based on broader context and individual perspectives, the statement primarily aligns with a liberal viewpoint due to its advocacy for regulation and legislation for consumer protection in healthcare.	liberal
ACP	Disclosure of Denials	2022	The text you've provided suggests a policy proposal aimed at increasing transparency in healthcare, specifically regarding the preauthorization review process conducted by health plans or related entities. This policy would require these entities to publicly disclose information about the denial of healthcare services, denial of payment, and rates of reversal on appeal.<br><br>**Liberal Viewpoint Alignment:**<br>1. **Transparency and Accountability**: The emphasis on transparency and public access to information aligns with liberal principles, which often advocate for increased oversight and transparency in various sectors, including healthcare.<br>2. **Consumer Protection**: By mandating the publication of denial rates and appeals, the policy appears to advocate for protecting consumers (patients) from potentially unfair practices, which is a common theme in liberal policies.<br>3. **Healthcare as a Right**: Liberals typically view healthcare as a fundamental right and may support measures that they believe will improve access and fairness in the healthcare system.<br><br>**Conservative Viewpoint Alignment:**<br>1. **Market Efficiency**: Conservatives might argue that transparency leads to more efficient markets by empowering consumers with information, although they might prefer market-based solutions over regulatory mandates.<br>2. **Limited Government Intervention**: While conservatives generally favor less government intervention, some might support this policy if they see it as a way to hold private entities accountable and prevent fraud or abuse.<br><br>**Conclusion:**<br>The policy appears to align more closely with a liberal viewpoint due to its emphasis on transparency, consumer protection, and possibly viewing healthcare as a right. It focuses on regulatory measures to ensure that health plans are accountable to the public and to physicians, which is more in line with liberal approaches to healthcare reform. However, aspects of the policy might also resonate with some conservative ideals related to market efficiency and accountability. As with many policies, individual interpretations can vary widely based on broader beliefs about the role of government, the healthcare system, and the market.	probably liberal

ACP	Evaluating the Impact of Preauthorization Programs for "Advanced Medical Imaging"	2019	The text you've provided seems to align with a viewpoint focused on practicality and evidence-based decision-making in healthcare rather than strictly liberal or conservative ideologies. It advocates for:<br><br>1. **Scientific Evaluation**: Calling for a careful and scientific evaluation of preauthorization programs suggests a desire for policies based on evidence and research. This is a common standpoint in many ideological frameworks, but it's often associated with a more technocratic or pragmatic approach to policy-making.<br><br>2. **Cost Savings and Patient Satisfaction**: The concern for cost savings might be seen as more conservative, as conservatives often prioritize fiscal responsibility. However, the combined focus on patient satisfaction indicates a balanced approach, considering both efficiency and the quality of patient care, which could be appealing across the spectrum.<br><br>3. **Compensation for Clinicians**: Encouraging compensation for clinicians involved in preauthorization processes might resonate more with liberal viewpoints, emphasizing fair compensation and worker rights. However, it can also be seen as a measure to ensure better healthcare services, which is a universal concern.<br><br>Overall, the text seems to advocate for policies that are efficient, evidence-based, and considerate of all stakeholders (patients, physicians, and health plans). This balanced approach might find supporters in both liberal and conservative circles, depending on how they weigh the various aspects of the proposal. It's more about pragmatic healthcare management than ideological leanings.	neutral
ACP	Evaluating the Impact of Pharmaceutical Preauthorization Programs	2019	The text reflects a perspective that aligns with a more liberal viewpoint. It emphasizes the need for careful and scientific evaluation of pharmaceutical preauthorization programs, considering various factors like cost, patient satisfaction, medical outcomes, and the workload of physician offices. The advocacy for a non-partisan entity to evaluate the impact on patient care and potential adverse outcomes for those who can't purchase prescribed medications suggests a concern for comprehensive healthcare access and quality, which is typically associated with liberal values. The call for lobbying Congress to mandate such evaluations indicates a desire for governmental intervention in healthcare oversight, another hallmark of liberal perspectives.	liberal
ACP	Advocating for Compensation for Completion of Preauthorization Program Applications for Pharmaceuticals	2019	The statement from the American College of Physicians (ACP) advocating for health plans to fairly compensate providers for the costs associated with completing preauthorizations for pharmaceuticals doesn't align strictly with either a liberal or conservative viewpoint. It's more focused on healthcare policy and the economics of medical practice rather than broader political ideologies. However, perspectives on it might vary:<br><br>- **Liberal Viewpoint**: Generally, liberals might support this statement as it advocates for fair compensation and could be seen as supporting healthcare providers and potentially improving patient access to necessary medications. It aligns with the broader liberal focus on workers' rights and fair compensation.<br><br>- **Conservative Viewpoint**: Conservatives might have mixed views. Some might agree with the principle of fair compensation and the reduction of bureaucratic obstacles in healthcare. Others might be wary of any mandates on private businesses, including health plans, preferring market-based solutions.<br><br>It's important to note that such issues are often complex and don't neatly fit into "liberal" or "conservative" categories. Different individuals within each group might have varying opinions based on their interpretations of fairness, market freedom, and the role of regulation in healthcare.	neutral
ACP	Publicizing Misleading or Fraudulent Representation by Health Insurers	2022	This text from the American College of Physicians (ACP) focuses on advocating for transparency and against misleading or fraudulent insurance practices. It doesn't strictly align with a liberal or conservative viewpoint as both sides might agree on the importance of transparency and ethical practices in healthcare insurance. The emphasis is on professional ethics and consumer protection rather than partisan politics. The goal is to inform members of potential dangers and collaborate with other societies to address these issues, which is more about advocacy for standards in the healthcare industry than a specific political stance.	neutral

ACP	Payment for Providing Information to Third Party Payers	2015	The text seems to be discussing policy recommendations from the American College of Physicians (ACP) regarding the regulation of third-party payers in the healthcare system. Here's a breakdown of the viewpoints it may align with:<br><br>### Liberal Viewpoint Alignment:<br>- **Regulation Support**: Liberals generally favor more regulation in healthcare to ensure fairness and equity. The ACP's call for regulations requiring third-party payers to cover additional information-sharing costs aligns with a desire for comprehensive, transparent healthcare processes.<br>- **National Regulation**: Advocating for national regulations over a patchwork of state laws can also be seen as a liberal stance, aiming for uniformity and broader protections across states.<br><br>### Conservative Viewpoint Alignment:<br>- **Costs and Efficiency**: Conservatives might support the aspect of ensuring third-party payers cover costs for providing comprehensive billing information if it's seen as a way to make billing more efficient and transparent, thus potentially reducing overall healthcare costs.<br>- **State vs. National Regulation**: While the ACP encourages national regulations, some conservatives might prefer state-level control over healthcare regulation. This detail might be more contentious among conservative audiences.<br><br>### Neutral Aspects:<br>- The specifics of who should pay for additional information (like detailed billing) can be viewed through both liberal and conservative lenses, depending on the broader context of healthcare reform and regulation philosophy.<br>- The text doesn't explicitly endorse a public vs. private healthcare approach, which is often a major dividing line between liberal and conservative healthcare policies.<br><br>In conclusion, the text aligns with certain principles from both liberal and conservative viewpoints. It leans toward a regulatory, structured approach favored by liberals, especially with its emphasis on national regulations. However, aspects of it could also be framed to align with conservative priorities around efficiency and potentially state-level autonomy. The exact alignment might depend on the broader context of the ACP's proposals and the specific healthcare beliefs of the individuals interpreting the text.	neutral
ACP	Medical Paperwork	2015	The text you've provided describes a policy recommendation from the American College of Physicians (ACP) regarding third-party payers and physician workload. It advocates for transparency, communication, and fair compensation when new policies increase the workload for physicians. It doesn't neatly align with a traditional liberal or conservative viewpoint as it touches on aspects that both sides might support or oppose:<br><br>1. **Liberal Viewpoint**: Liberals generally advocate for comprehensive healthcare policies and might support the idea of transparency and fair compensation for physicians as a means to ensure quality care and maintain a robust healthcare system. They might also agree with the involvement of professional societies in policy discussions, emphasizing collaboration and community input.<br><br>2. **Conservative Viewpoint**: Conservatives typically favor free-market solutions in healthcare and might support the notion of fair compensation as a matter of contractual justice and market efficiency. They might also appreciate the call for transparency and reduced administrative burdens as a way to streamline healthcare and reduce unnecessary costs.<br><br>This policy recommendation mainly focuses on practical, administrative aspects of healthcare delivery rather than broader ideological principles. It emphasizes fair compensation, transparency, and consultation, which are concerns that can transcend political lines. As such, it might find support across the political spectrum for different reasons.	neutral

ACP	Adopting a Single Definition of Medical Necessity	2022	The text you've provided refers to the American College of Physicians (ACP) adopting the American Medical Association's (AMA) single definition of medical necessity and advocating for all health plans in the United States to use this definition. This stance doesn't neatly align with traditional liberal or conservative viewpoints as it focuses more on standardizing healthcare practices and ensuring a unified approach to defining medical necessity.<br><br>In the U.S., healthcare policy can be a partisan issue, but the specific idea of adopting a uniform definition of medical necessity transcends typical liberal or conservative categorizations. Both sides might see different advantages in this approach:<br><br>- **Liberal Perspective**: From a liberal viewpoint, having a single definition of medical necessity might be seen as a step towards more equitable healthcare, ensuring that all patients have access to the same standard of care and reducing variability and potential inequality in treatment decisions. Liberals might also argue that this could lead to better healthcare outcomes and more patient-centric care.<br><br>- **Conservative Perspective**: Conservatives might appreciate the emphasis on standardization and efficiency that a single definition of medical necessity could bring. It could be seen as a way to reduce bureaucratic red tape and make the healthcare system more straightforward and less costly to navigate. There might also be an argument that it allows for clearer regulations and less government intrusion in deciding what constitutes necessary care.<br><br>Ultimately, the position is more about administrative consistency and less about the broader ideological beliefs typically associated with liberalism or conservatism in healthcare. It's more pragmatic than ideological, aiming to simplify and unify healthcare administration rather than pushing a particular political agenda.	neutral
ACP	Addressing the Increasing Burden of Health Insurance Cost Sharing	2016	The text you've provided appears to align more with a liberal viewpoint. It emphasizes reducing healthcare costs through systemic reforms rather than shifting costs to individuals, promoting value-based care, increasing transparency, and making care more accessible and affordable, especially for lower-income individuals. It advocates for government interventions like expanding tax credits, subsidies, and promoting health insurance literacy. These approaches typically resonate with liberal principles that prioritize equity, government involvement in ensuring access to healthcare, and a focus on preventative and value-based care systems. Conservative viewpoints, on the other hand, might emphasize less government involvement, more market-based solutions, and personal responsibility for healthcare costs.	liberal
ACP	Using Market Reform to Encourage Physician Primary Care	2016	The text you've provided outlines a position from the American College of Physicians (ACP) on physician workforce policy. It supports policies based on sound studies, discourages arbitrary targets, advocates for adequate payment to primary care physicians, and states that any workforce policy should only affect funding, not accreditation.<br><br>This stance doesn't neatly align with traditional liberal or conservative viewpoints. It's more focused on practical, profession-specific issues within healthcare. Liberal perspectives generally emphasize access to healthcare and support for healthcare professionals as part of broader social support systems. Conservative viewpoints often prioritize free-market solutions and may resist government intervention in setting targets or policies for healthcare.<br><br>The ACP's stance seems to blend elements from both sides: it supports using evidence-based research to inform policy (which can be more of a technocratic or liberal approach) and opposes arbitrary targets (which could align with conservative views favoring less regulation). The call for adequate payment to primary care physicians reflects a desire to ensure a robust healthcare system, a concern that crosses ideological lines.<br><br>In summary, the ACP's statement reflects a professional and pragmatic approach to healthcare policy rather than a strictly ideological one, focusing on what they believe will best support physicians and, by extension, patient care.	neutral

ACP	Physician Workforce Legislation	2016	The text primarily aligns with a liberal viewpoint. It advocates for increased federal involvement and regulation in the healthcare sector, particularly in training and distribution of physicians to ensure a higher number and proportion of primary care physicians. This includes supporting federal legislation to develop a national workforce policy, calling for the creation of a national commission to oversee this policy, and suggesting significant changes in funding and payment structures from Medicare and other payers to align with these goals.<br><br>Liberal perspectives generally favor a more active role for the government in providing and regulating services, especially in areas like healthcare, to ensure equitable access and distribution of resources. The emphasis on increasing the number of physicians in primary care and ensuring their distribution to underserved areas reflects a concern for social equity and access to healthcare services, which are typical of liberal ideologies. Additionally, the text's support for changing the economic and regulatory environment to favor primary care and its focus on training and mentoring through funded programs are in line with liberal priorities of education, training, and investment in public services.<br><br>Conservative viewpoints, in contrast, might focus more on market-driven solutions, less government intervention, and concerns about the efficiency and effectiveness of large-scale government programs. The text’s approach is more interventionist and regulatory than a conservative perspective would typically endorse, particularly in its advocacy for a detailed, nationally coordinated policy and significant changes to funding mechanisms.	liberal
ACP	Generating More Generalists: An Agenda of Renewal for Internal Medicine	2017	The text from the Federated Council for Internal Medicine (FCIM) and the American College of Physicians (ACP) doesn't strictly align with a traditional liberal or conservative viewpoint as understood in the political sense. Instead, it focuses on policy recommendations to improve the general internal medicine field and healthcare delivery, addressing issues like medical education, residency training, research funding, reimbursement, regulatory burdens, and care in underserved areas.<br><br>However, certain aspects of the text may resonate differently with liberal or conservative ideologies:<br><br>1. **Liberal Leanings**:<br>- **Promotion of Generalist Medicine**: The emphasis on enhancing primary care and generalist medicine aligns with more liberal health policy views, which often prioritize accessible, comprehensive healthcare for all.<br>- **Government and Institutional Resources**: Calling for leveraging government resources and creating incentives to attract physicians to underserved areas is a perspective more commonly associated with liberal policies, which generally support a stronger role for government in ensuring healthcare equity.<br><br>2. **Conservative Leanings**:<br>- **Efficiency and Market Incentives**: The suggestions to improve the practice environment through better reimbursement and reduced administrative burdens may appeal to conservative views favoring market efficiency and less regulatory interference.<br>- **Physician Extenders**: Exploring the use of physician extenders to deliver patient care more efficiently might resonate with conservative preferences for innovation and cost-saving measures in healthcare.<br><br>It's important to note that healthcare policy can be complex and multifaceted, and many ideas can garner support across the political spectrum. The proposals in this document seem primarily focused on practical solutions to healthcare challenges rather than promoting an overtly political ideology. They suggest a comprehensive approach to improving internal medicine through education, training, research, and policy changes, which could be supported by individuals with a variety of political beliefs.	neutral
ACP	Solutions to the Challenges Facing Primary Care Medicine	2022	The text advocating for a National Health Care Workforce Policy aligns more closely with liberal viewpoints, typically associated with the Democratic Party in the United States. Key aspects of liberal ideology include the belief in a strong, active role for the federal government in ensuring social justice, healthcare access, and the welfare of its citizens. This text's emphasis on federal involvement in healthcare workforce development, education incentives, and targeted support for underserved areas reflects these priorities. It suggests a centralized approach to addressing healthcare needs, focusing on expanding access to care, particularly in primary care, and ensuring an adequately trained workforce through federal support and regulation.<br><br>The call for increased federal spending on education, training, and incentives, as well as the establishment of a permanent national commission on the health care workforce, is consistent with liberal priorities of government intervention and investment in public services to promote equality and access. Conservatives, on the other hand, generally favor less government intervention, more market-based solutions, and local rather than federal control over healthcare and education, which contrasts with the proposals in the text.	liberal

ACP	Policy on Physician Reentry to the Workforce	2019	The text you've provided seems to focus on policies related to the reentry of physicians into the workforce and the support of federal funding for such programs. It doesn't explicitly align with a traditional liberal or conservative viewpoint as it addresses specific policy proposals rather than broader ideological principles. Both liberal and conservative viewpoints can support the idea of reentry programs for physicians, albeit for different reasons:<br><br>1. **Liberal Viewpoint**: Might emphasize the importance of reentry programs as a way to ensure a robust healthcare system and improve access to medical care for underserved communities. Liberals might also support the use of federal funding as a way to invest in public health infrastructure and workforce development.<br><br>2. **Conservative Viewpoint**: Might focus on the potential for reentry programs to alleviate physician shortages and improve the efficiency of the healthcare system. Conservatives might support such initiatives if they believe they will lead to a more competent workforce and potentially reduce reliance on foreign-trained physicians.<br><br>The statement about ensuring that funding for these programs does not divert funds from other important areas could be seen as a fiscally responsible position, which might align more closely with conservative priorities concerning budget allocation. However, the overall support for federal involvement could align with a liberal perspective on the role of government in healthcare.<br><br>In conclusion, while the text doesn't neatly fit into a liberal or conservative viewpoint, elements of it could be supported by both sides for different reasons. It's important to note that individuals and political groups might have nuanced positions that don't strictly adhere to broad ideological categories.	neutral
ACP	Principles on Dynamic Clinical Care Teams	2013	The text you've provided outlines principles and policies related to the organization and function of clinical care teams, licensure, reimbursement, and research in healthcare. These policies emphasize patient-centered care, multidisciplinary collaboration, flexibility in roles and responsibilities, the importance of licensure for public safety, and the need for reimbursement systems that incentivize quality care and teamwork.<br><br>This text does not align strictly with a traditional liberal or conservative viewpoint. Instead, it represents a professional and pragmatic approach to healthcare delivery, focusing on improving patient outcomes, ensuring safety, and optimizing team collaboration. Here's how some of the points might generally resonate with broader liberal or conservative perspectives:<br><br>**Liberal Viewpoint Tendencies**:<br>- Liberals might appreciate the emphasis on patient-centered care, the collaborative approach, and the flexibility in roles, seeing these as ways to ensure equitable and comprehensive care.<br>- The acknowledgment of the need for a diverse team of healthcare professionals might align with liberal values of inclusivity and utilizing a broad range of expertise.<br><br>**Conservative Viewpoint Tendencies**:<br>- Conservatives might appreciate the call for licensure to ensure public health and safety, as it aligns with a desire for order and standards in professional fields.<br>- The idea that reimbursement should reflect the complexity of the care provided might resonate with conservative views on merit-based compensation and fiscal responsibility.<br><br>However, the text is primarily professional in nature, aimed at outlining best practices and policies for effective healthcare delivery rather than promoting a political ideology. It's more about providing a framework for quality and safety in healthcare teams, which is a concern that transcends political lines. Both liberals and conservatives might find aspects of the text agreeable, as it focuses on improving healthcare outcomes, which is a universally appreciated goal.	neutral

ACP	Reducing Administrative Tasks in Health Care	2017	The positions outlined in the 2017 paper from the American College of Physicians (ACP) focus on reducing the administrative burden in healthcare, aiming to improve care quality, efficiency, and patient outcomes. This isn't strictly aligned with a liberal or conservative viewpoint as both sides of the political spectrum can find aspects to support:<br><br>- **Liberal Viewpoint**: Liberals might support these recommendations as they align with improving patient care and outcomes, focusing on evidence-based practices, and aiming for a more efficient healthcare system. The call for collaboration among stakeholders, including public entities, and the emphasis on transparency and patient-centeredness are also themes often supported by liberal ideologies. They often advocate for streamlined, efficient, and accessible healthcare, emphasizing the role of oversight and public involvement to ensure quality and fairness.<br><br>- **Conservative Viewpoint**: Conservatives might also find agreement with aspects of these recommendations, particularly the focus on reducing unnecessary administrative tasks, which can be seen as reducing bureaucracy and inefficiency. The emphasis on rigorous research and the potential for cost-saving through reduced administrative burdens align with conservative values of efficiency, reduced government (or external) interference, and fiscally responsible healthcare solutions. Additionally, the call for making better use of existing technologies and innovative approaches can resonate with conservative support for free-market solutions and technological innovation.<br><br>In essence, these recommendations center on pragmatic healthcare reform aimed at reducing inefficiencies and improving outcomes, which can transcend traditional liberal-conservative divides. They reflect a technocratic approach, focusing on practical measures and evidence-based strategies to improve the healthcare system. While the emphasis on certain aspects might resonate more with one political ideology than the other, the overall goals of improving quality, reducing unnecessary burdens, and focusing on patient outcomes are widely appealing across the political spectrum.	neutral
ACP	Compensation Equity and Transparency in the Field of Medicine	2017	The text you've provided outlines a position focused on equity, transparency, and research into disparities in physician compensation. It doesn't strictly align with traditional liberal or conservative viewpoints as defined in American politics but contains elements that both sides might support or critique differently:<br><br>1. **Liberal Viewpoint**:<br>- **Support for Equity and Anti-Discrimination**: Liberals generally advocate for policies promoting equity and opposing discrimination based on personal identity. The emphasis on equitable compensation regardless of race, gender, religion, nationality, sexual orientation, and gender identity aligns with liberal priorities for social justice and equality.<br>- **Transparency and Research**: Liberals often support transparency in various sectors and might favor the call for more research and data collection to understand and address disparities.<br><br>2. **Conservative Viewpoint**:<br>- **Merit-based Compensation**: Conservatives might agree with the idea that compensation should be based on skills, knowledge, and competencies. They often support meritocratic principles where rewards are based on individual effort and achievement.<br>- **Concerns over Regulation and Privacy**: Some conservatives might be wary of increased transparency and data collection, viewing it as potential overreach or a threat to personal privacy. They might prefer less regulatory intervention and more market-based solutions.<br><br>3. **Points of Contention**:<br>- **Role of Identity in Compensation**: While the statement's emphasis on not discriminating based on personal identity characteristics might be universally applauded, conservatives might question the extent to which such characteristics currently affect physician compensation or argue against what they see as an overemphasis on identity factors.<br>- **Method of Addressing Disparities**: Liberals might support proactive measures and interventions to address disparities, while conservatives might prefer less interventionist approaches and question the role of government or organizations in mandating transparency and data collection.<br><br>In conclusion, the text reflects a nuanced position advocating for fairness, knowledge, and transparency in physician compensation. Different aspects of it might resonate with or be critiqued by both liberals and conservatives, depending on their views on equity, regulation, and the role of personal identity in professional settings.	neutral

ACP	Achieving Gender Equity in Physician Compensation and Career Advancement	2018	The text you've provided reflects viewpoints typically aligned with a liberal or progressive ideology. Here are a few reasons why:<br><br>1. **Equity and Equality**: The emphasis on equitable compensation and opposition to discrimination based on personal identity characteristics, including gender, aligns with liberal values that focus on social justice and equal rights.<br><br>2. **Transparency and Accountability**: The support for transparency in compensation and routine assessment for equity is consistent with progressive calls for organizational accountability and fairness.<br><br>3. **Universal Access to Family and Medical Leave**: Advocating for universal access to paid family and medical leave is a stance more commonly associated with liberal ideologies, which often prioritize worker protections and benefits.<br><br>4. **Opposition to Discrimination**: The strong stance against discrimination, harassment, and retaliation based on personal identity characteristics is aligned with liberal principles of inclusivity and human rights.<br><br>5. **Support for Leadership and Career Development**: Encouraging mentorship, leadership development, and flexible career paths, especially for women, reflects a progressive approach to addressing systemic barriers and promoting diversity in the workplace.<br><br>6. **Implicit Bias Training**: The call for implicit bias training is a recognition of systemic biases and a commitment to addressing them, which is a key component of progressive social policies.<br><br>7. **Research and Actions to Address Gender Pay Inequity**: Recognizing and seeking to address gender pay inequity and barriers to career advancement for women is aligned with feminist and progressive movements that advocate for gender equality in the workplace.<br><br>8. **Comprehensive and Inclusive Policies**: The overall approach of advocating for comprehensive, inclusive, and flexible policies to support all physicians reflects a liberal orientation towards inclusivity and support for diverse needs.<br><br>While some conservatives might support certain aspects of these policies, the comprehensive approach to equity, diversity, and inclusion, as well as the emphasis on government and organizational intervention to secure these rights and benefits, is more commonly associated with liberal or progressive ideologies.	liberal
ACP	Physician Ordering of Durable Medical Equipment and Home Health Services	2017	The text presented aligns more with a liberal viewpoint. It emphasizes the importance of access to medical services, supports the role of federal oversight in preventing fraud and abuse, and stresses the need for patient-centered, medically necessary care. These positions generally reflect liberal ideals which prioritize accessible healthcare and government involvement in ensuring fairness and quality in the healthcare system. On the other hand, conservative viewpoints typically emphasize reduced government involvement in healthcare, more market-based solutions, and might oppose increased regulation or oversight as suggested in the text for fear it might lead to inefficiencies or reduced freedom for providers. However, the text does advocate for cost-sharing and against unnecessary expansion of services, which can align with conservative thoughts on personal responsibility and cost control in healthcare. Thus, while the text primarily reflects a liberal perspective, it also incorporates some elements that could be seen as fiscally conservative.	probably liberal
ACP	Home-Bound Care	2015	The statement from the American College of Physicians (ACP) regarding the belief that physicians should be compensated for their charges for allied health personnel, as well as reasonably reimbursed for the care and supervision of their home-bound patients, doesn't align strictly with either a liberal or conservative viewpoint in a traditional political sense. Instead, it addresses a specific professional and economic stance related to healthcare reimbursement.<br><br>In the U.S., views on healthcare and compensation can vary widely within both liberal and conservative circles. Generally, liberals might support comprehensive compensation for healthcare providers as part of a broader agenda for universal or expanded healthcare coverage. Conservatives might support the idea of fair compensation for services rendered as it aligns with free-market principles and the value of professional work.<br><br>However, both sides might have different ideas about the implementation, funding, and regulation of such payments. Liberals might prefer structured reimbursement rates within a regulated system, while conservatives might advocate for market-driven rates. Ultimately, the statement is more about professional standards and fair compensation in healthcare rather than a clear-cut political ideology.	neutral

ACP	Unnecessary Recertification Forms	2015	The text you've provided expresses a policy position related to healthcare administration and physician authority, specifically urging the Centers for Medicare & Medicaid Services (CMS) to modify recertification requirements for certain health conditions. It doesn't neatly align with a liberal or conservative viewpoint in a traditional political sense. Instead, it focuses on healthcare policy and administrative efficiency.<br><br>Liberal viewpoints generally support more comprehensive healthcare access and might favor reducing bureaucratic hurdles to care. They might support this position if they see it as a way to make healthcare more accessible and patient-centered.<br><br>Conservative viewpoints often emphasize reducing government involvement and might support this position if they see it as a way to decrease regulatory burdens and enhance personal freedom in healthcare choices.<br><br>However, both liberals and conservatives might have concerns about the implications for quality of care, oversight, and potential for abuse. Therefore, without further context on the broader political and social views of the American College of Physicians (ACP) or how this policy fits into larger healthcare debates, it's difficult to categorize this statement strictly as liberal or conservative. It's a specific healthcare policy recommendation that could have supporters and detractors on both sides of the aisle, depending on their priorities and how they view the role of regulation in healthcare.	neutral
ACP	Hospitals to Provide All Services on a Seven Day a Week Basis	2021	The text you've provided seems to be a policy statement from a medical organization (ACP—American College of Physicians, perhaps) emphasizing the importance of hospitals providing continuous services to meet patient needs. This statement focuses on ensuring that patients receive timely evaluation, treatment, and safe discharge around the clock. It doesn't explicitly align with a liberal or conservative viewpoint as it's primarily a healthcare policy recommendation rather than a political statement.<br><br>Both liberal and conservative individuals and groups might agree on the importance of continuous and accessible healthcare services, as this is a general public health and welfare issue. The specifics of how this is implemented and funded might be where liberal and conservative viewpoints diverge, with potential debates around government involvement, funding, and healthcare management. However, without additional context or information on these specifics, the statement itself remains neutral and focused on healthcare best practices rather than political ideology.	neutral
ACP	Controlling Health Care Costs: Certificate of Need Laws and Health Planning	2022	The text you've provided outlines perspectives on health planning and the evaluation of Certificate of Need (CON) programs. It does not explicitly align with a traditionally liberal or conservative viewpoint, as it focuses on practical health care management and resource allocation issues. However, elements can be interpreted as leaning towards one side or the other based on broader ideological frameworks:<br><br>1. **Liberal Viewpoint**:<br>- The emphasis on public engagement and ensuring resources meet health care needs might resonate with liberal ideals, which often prioritize community involvement and equitable distribution of resources.<br>- The call for research and regulation, such as through CON programs, to prevent maldistribution and redundancy could align with a more interventionist and regulatory stance typically associated with liberal politics.<br><br>2. **Conservative Viewpoint**:<br>- The focus on the effectiveness and impact of regulatory programs like CON could also align with conservative perspectives if the outcome of such research suggests that less regulation and more market-driven solutions are more effective.<br>- Conservatives might support the idea of ensuring resources are not wasted and that health care is efficiently allocated, but they may prefer different methods, such as increased competition and reduced government involvement, to achieve these ends.<br><br>Ultimately, the text appears to advocate for informed and efficient health care planning and resource allocation, emphasizing research and public engagement. The interpretation of whether it's more liberal or conservative could depend on the specific outcomes of the proposed research and planning and the ideological beliefs about the best ways to manage and allocate health care resources.	neutral

ACP	Inpatient Admission Criteria	2023	The text you've provided outlines a policy position from a medical college regarding the admission of patients into inpatient hospital settings and the role of payers in this process. It doesn't explicitly align with a liberal or conservative viewpoint as it primarily focuses on medical and procedural recommendations for patient admissions and interactions with healthcare payers. Here's how each viewpoint might interpret it:<br><br>- **Liberal Viewpoint**: Might support these recommendations as they emphasize evidence-based decision-making and advocate for transparency and fairness in healthcare processes, aligning with a general liberal emphasis on patient rights and equitable access to healthcare.<br><br>- **Conservative Viewpoint**: Might also agree with the emphasis on clear and transparent criteria and the protection of physician's medical judgment, which aligns with a conservative preference for less bureaucratic intervention and respect for professional expertise.<br><br>In essence, the statement is more about ensuring ethical and evidence-based medical practices rather than pushing a particular political ideology. It might be seen favorably by both sides for different reasons, depending on how it's interpreted in the context of broader healthcare debates.	neutral
ACP	Internist Hospital Privileges	2015	The statement provided reflects a viewpoint focused on professional standards, competence, and governance within the medical field, specifically concerning hospital privileges and the role of medical staff. It doesn't neatly align with traditional liberal or conservative ideologies as it focuses on medical governance rather than broader political or social issues.<br><br>Liberal viewpoints generally emphasize progressive change, equality, and government intervention in addressing social issues, while conservative viewpoints typically prioritize tradition, personal responsibility, and limited government intervention. This text, however, centers on the importance of training, competence, and professional governance within hospitals, which are concerns that both liberal and conservative individuals in the medical field might agree on, given their shared interest in maintaining high standards of patient care and professional integrity.<br><br>It's more about ensuring that those who have the necessary expertise and experience are making informed decisions about medical care and hospital operations. As such, it doesn't inherently support or oppose either political ideology but rather promotes a professional and standardized approach to hospital practice and governance.	neutral
ACP	Admission to a Hospital Medical Staff	2015	The text presents a viewpoint focused on maintaining standards and fairness in the admission of physicians to hospital medical staffs, advocating for an impartial review of qualifications over mere membership in specific groups or organizations. This perspective emphasizes the importance of individual merit and qualifications, which doesn't align strictly with a liberal or conservative viewpoint. Instead, it reflects a professional and ethical stance concerned with maintaining the quality of medical care and institutional integrity. Both liberal and conservative ideologies might support these principles for different reasons: liberals might focus on the fairness and equality aspects, while conservatives might appreciate the emphasis on merit and standards. Overall, the statement is more professional and ethical in nature rather than political.	neutral
ACP	Limitation or Cancellation of Hospital Privileges Based on Age	2023	The text you've provided outlines the American College of Physicians' (ACP) stance on the evaluation of physician competency and privileges, emphasizing peer evaluations and opposing age-based restrictions. This stance doesn't neatly align with traditional liberal or conservative viewpoints as it primarily concerns professional standards and medical ethics rather than broader political ideologies.<br><br>However, it might intersect with various aspects of both liberal and conservative thought. Liberals might appreciate the emphasis on individual evaluation and the fight against age discrimination, reflecting a commitment to individual rights and fairness. Conservatives might value the emphasis on professional self-regulation and skepticism toward arbitrary rules, which aligns with a preference for limited government intervention.<br><br>Overall, the text is more focused on professional policy and ethics in medicine than on political ideology. It reflects a nuanced position that prioritizes individual competence and peer evaluation over broad, imposed regulations, which can be appreciated across the political spectrum for different reasons.	neutral

ACP	Privileges in Clinical Departments of Hospitals	2015	The text you've provided seems to outline a policy stance from the American College of Physicians (ACP) regarding the delineation of privileges in a clinical department of a hospital. It emphasizes that this delineation is primarily a professional function of the physicians and medical staff, with the governing board of the hospital affirming the process's existence and implementation.<br><br>This stance doesn't inherently align strictly with a liberal or conservative viewpoint as traditionally defined in political terms. Instead, it focuses on the governance and administration of medical facilities. In general, conservative viewpoints tend to emphasize less regulation and more autonomy for institutions, while liberal viewpoints might advocate for more structured oversight and standards. The ACP's stance could be seen as balancing professional autonomy (more conservative) with institutional oversight (more liberal), but it primarily reflects a professional and administrative perspective rather than a partisan one.<br><br>It's important to note that issues of healthcare governance and policy can be complex and are often influenced by a range of factors, including ethical considerations, economic models, and practical experiences, which do not always align neatly with the political spectrum.	neutral
ACP	Establishment of Separate Subspecialty Departments Distinct from Departments of Medicine	2017	The statement from the American College of Physicians (ACP) regarding the structure and integrity of departments of internal medicine doesn't inherently align with a traditional political spectrum of liberal or conservative. It focuses on the organizational and structural aspects of medical departments rather than broader political or ideological issues. The ACP's stance is more about maintaining a cohesive, unified approach to internal medicine and discouraging the separation of subspecialties into distinct departments. This perspective is primarily concerned with medical education, quality of patient care, and administrative efficiency, rather than political ideology. It would be more relevant to discussions among healthcare professionals about the best ways to organize and manage medical institutions and has implications for healthcare delivery, professional education, and possibly healthcare costs, but it doesn't neatly fit into a liberal or conservative viewpoint.	neutral
ACP	Hospital-Employed Physicians on Hospital Medical Staffs	2015	The text you've provided is a statement from the American College of Physicians (ACP) regarding hospital governance and physician oversight. It doesn't directly align with traditional liberal or conservative viewpoints as it's more focused on medical professional standards and hospital operations than on broader political ideologies.<br><br>In the U.S. political context:<br>- **Liberal** viewpoints typically emphasize progressive change, social equality, and government intervention to address social inequities.<br>- **Conservative** viewpoints often emphasize tradition, limited government intervention, personal responsibility, and free market principles.<br><br>The ACP statement is about ensuring that physicians participating in patient care are members of the organized medical staff and adhere to hospital bylaws. This is more about professional governance and quality of care in medical institutions than about the liberal-conservative spectrum. Both political sides might agree on the importance of competent, regulated healthcare provision, though they might differ on methods and the degree of government involvement.<br><br>It's essential to consider that many policies and positions can have aspects that appeal to or affect both sides, and the perception of an issue as liberal or conservative can depend on the specific context and the broader values and policies it's associated with.	neutral
ACP	Hospital Governing Boards	2015	The text suggests that the American College of Physicians (ACP) believes in the importance of physicians having a direct say in the governance of hospitals by being voting members of the hospital board, and it advocates making this a requirement for accreditation. This viewpoint aligns more with principles typically associated with liberal ideologies, which emphasize inclusive participation and shared decision-making in institutions. Liberals often advocate for stakeholders, like workers or, in this case, physicians, to have a say in the management and decisions of the organizations they are part of. This is based on the belief that those directly involved in the work have valuable insights and should have a voice in governance.<br><br>However, it's important to note that the distinction isn't strictly partisan. Some conservatives might also support this idea, especially if they see it as a way to improve healthcare outcomes or as a matter of professional autonomy and rights. Healthcare governance and accreditation are complex issues that can cross typical ideological lines, with support or opposition coming from different perspectives based on broader beliefs about the best ways to manage and improve healthcare systems.	probably liberal

ACP	Opposing the Requirement that Hospitals Screen Patients for Methicillin-resistant Staphylococcus aureus (MRSA)	2022	The statement about the American College of Physicians (ACP) opposing legislative requirements for hospitals to screen patients for Methicillin-resistant Staphylococcus aureus (MRSA) doesn't neatly align with a traditional liberal or conservative viewpoint. Instead, it reflects a stance on a specific healthcare policy issue. Here's how both sides might view it:<br><br>- **Liberal Viewpoint:** Liberals generally advocate for stronger healthcare regulations to ensure patient safety and public health. They might support MRSA screening to prevent outbreaks and ensure hospitals are taking proactive steps to combat antibiotic resistance. However, some liberals might agree with the ACP if they believe that the decision should be left to medical professionals rather than mandated by the government.<br><br>- **Conservative Viewpoint:** Conservatives typically prefer fewer government regulations, arguing that they can be burdensome and limit professional autonomy. They might agree with the ACP's opposition to legislative requirements for MRSA screening, seeing it as an issue best left to individual hospitals and healthcare providers to decide based on their circumstances.<br><br>In essence, the ACP's position might be seen as aligning more with a conservative viewpoint on regulatory issues, advocating for professional discretion over government mandates. However, it's important to note that many factors influence stances on healthcare policies, including scientific evidence, ethical considerations, and practical implications, not just political ideologies.	probably conservative
ACP	Increasing Access to Healthcare for Non-Detained Asylum Seekers Living in the United States	2022	The text provided outlines policy recommendations and support measures for asylum seekers' healthcare. It's focused on providing medical care, ensuring culturally appropriate services, and advocating for research into their health needs. This perspective aligns more closely with a liberal viewpoint. Liberal ideologies typically emphasize the protection and expansion of individual rights and equality, including for marginalized communities such as immigrants and asylum seekers. The emphasis on comprehensive healthcare access, trauma-informed care, and the need for research and support reflects a concern for social justice and equity, hallmarks of liberal thought. Additionally, the focus on providing support through community health centers and safety-net hospitals, as well as the call for translation services and returning medical belongings, indicates a priority for accessible, inclusive healthcare services, which is also a common liberal stance.	liberal
ACP	Immigration	2017	The text generally aligns with a liberal viewpoint. Here's a breakdown of why:<br><br>1. **Support for Refugees and Opposition to Broad Entry Bans**: The ACP (American College of Physicians) supports expanding U.S. legal residency to vulnerable refugees and opposes denying refugee status based on country of origin. It also opposes broad entry bans based on country of origin or religion. These stances align with liberal views that emphasize humanitarian responsibilities and oppose broad, discriminatory bans.<br><br>2. **Opposition to Discrimination**: The ACP opposes discrimination based on religion, race, gender, gender identity, or sexual orientation. This inclusive approach is typically associated with liberal ideologies, which tend to emphasize equal rights and non-discrimination.<br><br>3. **Concern for Family Unity and Health Consequences**: Expressing concern about policies that split up families and the health consequences of such policies aligns with a liberal perspective that often prioritizes family unity and the broader social determinants of health.<br><br>4. **Support for "Dreamers" and DACA**: The ACP's support for undocumented individuals who came to the U.S. as children (often referred to as "Dreamers") and its call for preserving the Deferred Action for Childhood Arrivals (DACA) program are positions commonly held by liberals. They advocate for pathways to legal status for these individuals.<br><br>5. **Call for National Immigration Policy Consistent with Health Care Access**: The call for an immigration policy that ensures access to health care aligns with liberal priorities on health care access for all, including immigrants.<br><br>While these positions might not exclusively belong to a liberal ideology and can be supported by individuals with a range of political views, they generally fit within a liberal framework that emphasizes human rights, non-discrimination, and a comprehensive and humane approach to immigration and health care policy.	liberal
ACP	The Health Impact of Family Detentions in Immigration Cases	2018	The text presents a viewpoint that aligns more closely with liberal ideologies. It emphasizes the importance of family unity, the health and well-being of children, and a compassionate approach to immigration policies. These stances generally align with liberal perspectives, which often prioritize humanitarian concerns, the rights of immigrants, and the welfare of vulnerable populations. The text's focus on the adverse health impacts of family separation and detention, along with the call for community-based alternatives to detention and prioritizing the best interests of the child, are in line with liberal advocacy for comprehensive and humane	liberal

			immigration reforms. Conservative viewpoints might focus more on immigration law enforcement, border security, and the potential costs of liberal immigration policies, which are not addressed in this text.	
ACP	Genetic Testing and Reuniting Families	2018	The text you've provided presents a balanced viewpoint that emphasizes the importance of family reunification at the U.S. border while also highlighting the ethical, privacy, and practical considerations associated with the use of medical testing, particularly genetic testing, in this process. It doesn't strictly align with a traditional liberal or conservative viewpoint but rather merges concerns typically associated with both sides:<br><br>1. **Liberal Viewpoint Tendencies**:<br>- Emphasis on reunifying families, a priority often associated with more liberal immigration policies.<br>- Concern over privacy and the ethical implications of genetic testing, reflecting a focus on individual rights and protections.<br><br>2. **Conservative Viewpoint Tendencies**:<br>- The suggestion of using less intrusive means and the idea of testing as a last resort can resonate with a conservative preference for limited government intrusion.<br>- Emphasis on not storing samples or information beyond what is necessary could align with conservative values regarding privacy and limited government scope.<br><br>Overall, the text seems to take a centrist or pragmatic approach, acknowledging the importance of family reunification while also considering the ethical, privacy, and practical implications of the methods used to achieve this goal. It seeks a balanced solution that addresses the humanitarian concern of family separation and the logistical and ethical challenges of using medical data for identification.	neutral
ACP	National Immigration Policy and Access to Health Care	2022	The text provided outlines a series of positions and recommendations regarding access to health care for immigrants in the United States. These points generally align with a liberal viewpoint for several reasons:<br><br>1. **Emphasis on Universal Access**: The text argues for health care access for all residents, regardless of immigration status. This is a common liberal standpoint, focusing on universal health care and the right to health as a fundamental human right.<br><br>2. **National vs. State Solutions**: It advocates for a national policy rather than fragmented state-level solutions. Liberals often support federal-level solutions for systemic issues, believing they can provide more comprehensive and equitable outcomes.<br><br>3. **Healthcare as a Public Health Issue**: The argument that access to health care for undocumented individuals is a public health issue reflects a liberal perspective that prioritizes community health and preventative care over punitive or restrictive measures.<br><br>4. **Ethical Obligations and Non-Discrimination**: The emphasis on ethical obligations for healthcare professionals to treat all individuals and against discrimination in healthcare access aligns with liberal values of equality, justice, and human rights.<br><br>5. **Recognition of Economic and Public Health Impact**: The acknowledgment of the broader economic and public health ramifications of denying care to undocumented immigrants reflects a holistic view of healthcare, considering societal and long-term impacts rather than just individual or immediate costs.<br><br>However, it's important to note that the terms "liberal" and "conservative" can vary significantly in meaning depending on the country and context. In the U.S., the views expressed in the text would be more closely aligned with contemporary liberal or progressive stances, particularly those related to health care and immigration policy.	liberal

ACP	The Evolving Role of the Internal Medicine Specialist	2016	The text you've provided outlines the envisioned role of the Internal Medicine Specialist according to the American College of Physicians (ACP). It doesn't inherently align with a liberal or conservative political viewpoint. Instead, it focuses on the professional and clinical aspects of an Internal Medicine Specialist's role, emphasizing comprehensive care, evidence-based practice, patient advocacy, and health care system navigation.<br><br>Here's a breakdown of why it's not aligned with a specific political viewpoint:<br><br>1. **Comprehensive Care and Evidence-Based Practice**: Advocating for comprehensive and continuous care, along with an emphasis on evidence-based medicine, is a principle focused on improving patient outcomes and healthcare quality. These are goals shared across the political spectrum and pertain more to medical best practices than to political ideology.<br><br>2. **Healthcare System Navigation and Patient Advocacy**: While the text mentions guiding patients through a complex healthcare environment, it doesn't specify a stance on how the healthcare system should be structured or funded, which are common areas of political debate.<br><br>3. **Role Diversification**: The varied roles of an Internal Medicine Specialist, such as being a primary care provider, consultant, administrator, and educator, focus on the functional and professional aspects of healthcare rather than political ideologies.<br><br>4. **Quality Improvement and Cost-Effectiveness**: While there are debates on how to achieve quality improvement and cost-effectiveness in healthcare, the text doesn't delve into specific policies or reforms, which are typically where liberal and conservative viewpoints diverge.<br><br>In summary, the text is professional and clinical in nature, focusing on the roles and responsibilities of Internal Medicine Specialists according to the ACP. It does not advocate for specific political policies or ideologies, making it neutral rather than distinctly liberal or conservative.	neutral
ACP	Resolution Recognizing Geriatrics as a Primary Care Discipline	2016	The text you've provided is a policy recommendation from the American College of Physicians (ACP) that aligns with the American Geriatrics Society and the American Medical Association (AMA) House of Delegates. It advocates for recognizing geriatric medicine as a primary care discipline and aims to enhance training, education, and participation in delivering primary care services to older adults.<br><br>This statement doesn't neatly align with traditional liberal or conservative political viewpoints as it primarily focuses on healthcare policy and professional standards within the medical community. It reflects a consensus among medical professionals about the importance of specialized care for the elderly and does not delve into broader political or economic implications.<br><br>Both liberals and conservatives might support this policy for different reasons. Liberals might view it as a step toward improved healthcare access and quality, especially for a vulnerable population, aligning with a general emphasis on social welfare and public health. Conservatives might support it due to the focus on professional standards and improving healthcare outcomes, which can align with values around efficiency and quality in services.<br><br>In general, healthcare policies like this one often have supporters and detractors on both sides of the political spectrum, and their support or opposition can depend on broader healthcare system perspectives, fiscal considerations, and views on the role of government and professional bodies in regulating healthcare.	neutral

ACP	Promoting Internal Medicine	2016	The text from the American College of Physicians (ACP) outlines encouragement for internists to engage in community activities to promote the specialty of internal medicine, particularly in primary care. It suggests mentorships, counseling, and acting as a spokesperson as means of promotion.<br><br>This statement doesn't align strictly with either a liberal or conservative viewpoint as it primarily focuses on medical education and community involvement rather than political or ideological issues. Both liberals and conservatives might support such initiatives for various reasons:<br><br>1. **Liberal Viewpoint**: Liberals might appreciate this approach as it focuses on education, community engagement, and mentorship. They often support comprehensive healthcare education and initiatives that aim to provide equal opportunities for all individuals to pursue careers in healthcare.<br><br>2. **Conservative Viewpoint**: Conservatives might support this statement as well, seeing the value in promoting specialties that are seen as crucial to a functioning healthcare system. They might also appreciate the focus on individual responsibility and the private sector's role in educating and inspiring the next generation of physicians.<br><br>Overall, the statement is more professional and educational in nature rather than political. It's about encouraging participation in activities that promote a specific medical specialty and does not inherently lean towards a liberal or conservative viewpoint. It's more about the advancement of a profession and its benefits to the community, which can be supported across the political spectrum.	neutral
ACP	Physician Office Laboratories	2019	The text regarding the support and promotion of physician office laboratories for timely, efficient, accurate, and cost-effective patient testing doesn't clearly align with a traditional liberal or conservative viewpoint. It focuses on healthcare efficiency and quality, which are concerns across the political spectrum. Liberals might emphasize the access and quality of care aspects, while conservatives might focus on cost-effectiveness and efficiency. However, without more context or explicit mention of broader healthcare policies, it's not distinctly aligned with one ideology over the other.	neutral
ACP	Proficiency Testing in Physician Office	2019	The statement you provided from the American College of Physicians (ACP) focuses on encouraging members to use appropriate quality control measures and proficiency testing in laboratories. This directive aims at ensuring accurate and reproducible results in medical laboratories. This statement is primarily technical and professional rather than political, focusing on best practices in medical testing and quality assurance. It does not align directly with either a liberal or conservative viewpoint as it addresses professional standards and ethics in medical practice rather than political ideology. Both liberals and conservatives would likely agree on the importance of accuracy and reliability in medical testing, making this statement broadly acceptable across the political spectrum. It's more about professional responsibility and commitment to high-quality healthcare than about political ideology.	neutral
ACP	Reimbursement for Lab Services	2019	The text reflects viewpoints related to healthcare policy and reform, particularly focusing on reducing burdensome documentation for physicians and advocating for changes in Medicare's laboratory fee schedule. Here's how the text might align with different political viewpoints:<br><br>1. **Liberal Viewpoint**: A liberal perspective might appreciate the emphasis on reducing unnecessary burdens on physicians, as it could lead to more efficient healthcare delivery and potentially better care for patients. They might also support the idea of moving to a resource-based system for Medicare’s laboratory fee schedule, as it could represent a more equitable and efficient way of allocating healthcare resources.<br><br>2. **Conservative Viewpoint**: A conservative viewpoint might appreciate the call for regulatory and legislative changes that could lead to more streamlined operations and potentially reduce government overhead. They might also support the idea of limited test-sites for new coding methods, as this could be seen as a cautious and financially prudent approach to healthcare reform.<br><br>Overall, the text doesn't neatly align with a strictly liberal or conservative viewpoint. Instead, it suggests practical changes to healthcare administration that could, in theory, receive bipartisan support. The focus is more on improving efficiency and effectiveness in healthcare delivery rather than pushing a specific ideological agenda.	neutral

ACP	Laboratory Personnel Certification Under CLIA	2015	The text describes a stance taken by the American College of Physicians (ACP) regarding the categorization and qualification of personnel performing clinical tests. This issue primarily revolves around the regulation and oversight of healthcare professionals and practices. While it doesn't neatly align with traditional liberal or conservative viewpoints, it does touch upon themes relevant to both:<br><br>1. **Liberal Viewpoint Considerations**: Liberals generally advocate for more robust regulation and oversight in healthcare to ensure safety and quality. The idea of recategorizing certain tests to reflect their actual complexity might align with this as it suggests a nuanced and potentially stricter approach to regulation. However, the support for a "grandfather" clause, which would exempt certain personnel from new educational requirements, might be seen as a move to reduce regulatory burdens, which isn't typically a liberal stance.<br><br>2. **Conservative Viewpoint Considerations**: Conservatives typically prefer less government intervention and regulation, favoring market-based solutions and individual responsibility. The "grandfather" clause can be seen as a conservative approach, reducing the immediate impact of new regulations on current workers. However, the overall notion of categorizing and regulating medical tests does involve a level of oversight that some conservatives might find excessive.<br><br>In essence, the text represents a position focused more on practical implications within the medical field rather than a clear-cut ideological stance. It blends concerns for quality and safety in healthcare (more liberal) with respect for existing professionals and a desire to limit the disruptive impact of new regulations (more conservative). Therefore, it doesn't align exclusively with either a liberal or conservative viewpoint but instead reflects a profession-specific policy stance.	neutral
ACP	Self-Referral Legislation	2015	The text you've provided describes a policy stance from the American College of Physicians (ACP) regarding an exception to the Stark II ban on self-referrals. This policy aims to allow physicians who are not members of the same group practice but share the same building to share clinical laboratories and other in-office diagnostic facilities.<br><br>This stance doesn't align strictly with liberal or conservative viewpoints as it's primarily a healthcare policy issue. However, interpretations may vary:<br><br>1. **Liberal viewpoint**: Might support this as it could improve access to healthcare services, increase collaboration among healthcare providers, and potentially lower costs through shared resources.<br><br>2. **Conservative viewpoint**: Might also support this as it encourages free-market principles within healthcare, allowing physicians more autonomy and reducing regulatory burdens.<br><br>It's important to note that both liberals and conservatives could have objections based on concerns about the quality of care, potential for increased healthcare costs, or increased potential for conflicts of interest. The alignment with political ideologies often depends on how the policy is framed in terms of healthcare access, regulation, and market freedom.	neutral

ACP	Physician Office Labs in Medicare Risk Products	2019	The text outlines the American College of Physicians' (ACP) positions on specific healthcare policies related to Medicare Part B lab services and physician office labs. Here's a breakdown of how the viewpoints might align with traditional liberal or conservative perspectives:<br><br>1. **Opposition to awarding regional contracts to reference labs for all Medicare Part B lab services**: This position suggests a preference for maintaining a more diverse market of lab service providers rather than consolidating services under a few large reference labs. Conservatives might favor the free market aspect, while liberals might support it if they believe it leads to better healthcare outcomes.<br><br>2. **Guidance of a CMS-established body with adequate physician representation**: Calling for oversight and input from a body with physician representation may align more with a liberal viewpoint, emphasizing regulation and the importance of expert input in policy-making. However, conservatives might also support this if they see it as a way to ensure that industry professionals, rather than bureaucrats, guide decisions.<br><br>3. **Working with CMS to preserve the physician office lab**: This position indicates a desire to maintain the status quo and ensure that small, physician-operated labs remain viable. Conservatives might appreciate the support for small businesses and the push against potential overreach by large entities. Liberals might support it for ensuring access and local control over healthcare services.<br><br>4. **Ensuring appropriate reimbursement for services**: Advocating for fair compensation aligns with both liberal and conservative values but for different reasons. Liberals might see it as ensuring access to quality healthcare and fair pay for services, while conservatives might view it as a market issue where providers should be adequately compensated for their services.<br><br>Overall, the text reflects a nuanced position that doesn't neatly fit into a strictly liberal or conservative viewpoint. It combines elements of market competition, regulation, and professional autonomy, which are values that can be found across the political spectrum. The emphasis seems to be on practical outcomes and maintaining a balance between regulation and market forces, rather than ideological purity.	neutral
ACP	Shared Office Labs	2015	The statement about supporting an exception from the Stark II ban on self-referral to allow a shared office lab to be housed separately and bill Medicare seems to align more with a pragmatic or specific policy-oriented viewpoint rather than a strictly liberal or conservative one. Here's why:<br><br>1. **Liberal Viewpoint**: Generally, liberals in the U.S. political spectrum support more government intervention and regulation in healthcare to ensure fairness, access, and to control costs. They might support such exceptions if they believe it will improve healthcare access or efficiency.<br><br>2. **Conservative Viewpoint**: Conservatives typically advocate for less government intervention and more market-based solutions in healthcare. They might support this exception as it could be seen as reducing regulatory burdens and potentially lowering costs.<br><br>However, this statement is very specific and relates to a particular aspect of healthcare policy known as the "Stark Law" or "Stark II," which prohibits physician self-referral, especially where Medicare and Medicaid payments are concerned. The support for an exception seems to be more about practical healthcare delivery and efficiency rather than a broad ideological stance. It's about how to best implement an existing regulation, which can be a concern of both liberals and conservatives depending on the perceived outcomes.<br><br>In this case, both liberals and conservatives could potentially support this exception if they believe it leads to better patient care, more efficient service delivery, or cost savings without increasing fraud or abuse. Therefore, it's more indicative of a specific, pragmatic approach to healthcare policy rather than clearly aligning with a broad liberal or conservative ideology.	neutral

ACP	CLIA Regulations	2015	The statement about including simple, basic microscopic and non-microscopic tests under the waived category of the Clinical Laboratory Improvement Amendments (CLIA) doesn't inherently align with a specific liberal or conservative viewpoint. Instead, it addresses a technical aspect of healthcare regulation concerning the categorization of laboratory tests.<br><br>Liberal and conservative viewpoints typically diverge on broader healthcare policy issues such as the extent of government involvement, funding, and individual versus collective responsibility. However, the classification of laboratory tests under CLIA tends to be a more technical and specialized area that does not directly invoke ideological debates.<br><br>This statement likely reflects a consensus among medical professionals (as indicated by the references to the American College of Physicians (ACP) and the American Medical Association (AMA)) about the practicalities of laboratory testing regulations rather than a partisan political stance. It's more about ensuring that certain tests are appropriately categorized to reflect their complexity and the level of oversight required to ensure accuracy and safety.	neutral
ACP	Elimination of Fee for CLIA Certificate of Waiver	2015	The text refers to the American College of Physicians (ACP) working with the U.S. Department of Health and Human Services to ensure that the fee for the CLIA (Clinical Laboratory Improvement Amendments) Certificate of Waiver is limited to the actual cost of issuing the certificate. This statement seems to align more with a pragmatic or technocratic approach rather than fitting neatly into a traditional liberal or conservative viewpoint.<br><br>Here's why:<br><br>- **Liberal Viewpoint**: Generally, liberals might support such a measure if they see it as ensuring access to necessary services, promoting transparency, and preventing overcharging. They often advocate for government intervention to protect consumers and ensure fairness in healthcare.<br><br>- **Conservative Viewpoint**: Conservatives might also agree with this statement if they interpret it as a measure to prevent unnecessary government spending and overregulation. They typically favor limited government intervention and might see limiting fees to the actual cost as a way to prevent bloating in administrative costs.<br><br>In essence, the text reflects a policy-focused, practical approach aimed at ensuring efficiency and fairness in the administration of healthcare services. Both liberals and conservatives might find aspects to agree with, depending on their perspectives on government regulation, healthcare, and fiscal responsibility. The statement is more about good governance and less about ideological positions.	neutral
ACP	Payment for Handling or Conveyance of Specimen	2015	The statement about third-party reimbursement for specimen collection appears to be more of a technical or administrative concern rather than a strictly ideological one. However, its implications can be interpreted in various ways depending on liberal or conservative viewpoints:<br><br>1. **Liberal Viewpoint**: Liberals might focus on the aspect of regulatory compliance, such as adherence to OSHA regulations, as a necessary measure to ensure safety and quality in healthcare. They may argue that sufficient reimbursement is crucial for maintaining high standards of patient care and worker safety. Liberals generally support regulatory measures as a means to protect public health and safety.<br><br>2. **Conservative Viewpoint**: Conservatives might focus on the potential for increased costs and bureaucracy. They may argue that while safety and quality are important, excessive regulation and the need for higher reimbursements to cover these costs can lead to inefficiencies, increased healthcare costs, and may burden providers. Conservatives generally favor less regulation and might be skeptical of measures that increase operational costs for businesses or healthcare providers.<br><br>It's important to note that these interpretations can vary widely among individuals within each political ideology. Some may see it purely as a practical matter of ensuring healthcare providers are adequately compensated for their costs, while others may interpret it through a more ideological lens regarding regulation and market dynamics.	neutral

ACP	Physicians Performing Radiographs and Electrocardiograms in Offices	2015	The statement from the American College of Physicians (ACP) advocates for internists to be permitted to perform certain laboratory tests and procedures in their own offices and to receive fair reimbursement. This stance doesn't neatly align with traditional liberal or conservative viewpoints as it primarily addresses professional practice and healthcare efficiency rather than broader political ideologies.<br><br>On one hand, those with a conservative viewpoint might support this stance as it promotes individual autonomy for physicians, potentially reduces bureaucracy, and could enhance free-market principles within healthcare by allowing more providers to offer these services. It can be seen as a move toward decentralizing healthcare services and reducing reliance on larger hospital systems, which aligns with conservative values of smaller government and individual responsibility.<br><br>On the other hand, those with a liberal viewpoint might also support this policy as it could improve access to healthcare by making it more readily available and potentially more affordable if services are provided directly by internists rather than through larger, more expensive institutions. This could be seen as aligning with liberal values of increasing access to healthcare and making it more equitable.<br><br>However, the specifics of how this policy is implemented could sway the alignment. For example, if the policy led to increased costs for patients or compromised the quality of care, it might be opposed by those with a liberal viewpoint who prioritize healthcare accessibility and quality. Conversely, if it significantly increased private sector profits at the expense of patient care, it might be opposed by those with a conservative viewpoint who emphasize efficient and ethical healthcare delivery.<br><br>In conclusion, the statement reflects professional and healthcare management issues that cross over typical ideological lines, and support or opposition could vary among individuals with liberal or conservative viewpoints depending on how they prioritize different aspects of healthcare policy.	neutral
ACP	Relicensure--State Legislation	2016	The statement you've provided reflects a viewpoint on physician relicensure, emphasizing self-regulation and peer evaluation within the medical profession. It suggests that physicians are best evaluated by their peers through medical societies in cooperation with state authorities and seeks to explore methodologies beyond continuing education and examinations for relicensure.<br><br>This perspective doesn't neatly align with traditional liberal or conservative ideologies as it focuses more on professional self-governance and regulatory specifics within the medical field. However, elements of the statement can resonate differently with various political ideologies:<br><br>1. **Conservative Viewpoint**: Conservatives might appreciate the emphasis on self-regulation and the minimized role of external or governmental bodies in professional oversight. The idea of professional societies taking a lead role might be seen as a way to reduce bureaucratic control and maintain standards internally within the profession.<br><br>2. **Liberal Viewpoint**: Liberals might support the idea of continuous exploration of new methodologies for evaluating physician competence, indicating a progressive approach to medical licensure. However, they might also be concerned about ensuring that such self-regulation adequately protects patient rights and public health, and might prefer more external oversight to ensure transparency and accountability.<br><br>It's important to note that these are generalizations and actual opinions can vary widely within each ideological group. Additionally, the specifics of physician relicensure and the best methods for evaluating competence are complex issues that can transcend simple political categorization.	neutral

ACP	Regulation of Credentialing and Licensure	2017	The text you've provided outlines positions on physician accountability, maintenance of certification, and how these should be considered in various professional contexts. It doesn't explicitly align with a traditionally liberal or conservative viewpoint. Instead, it focuses on medical ethics, professional standards, and regulatory considerations.<br><br>1. The text emphasizes the importance of a variety of factors in physician credentialing and opposes making maintenance of certification an absolute requirement. This perspective values individual performance and continuous professional development, which can be seen as a more nuanced approach rather than a strictly liberal or conservative stance.<br><br>2. It argues against using maintenance of certification as the sole criterion for credentialing and reimbursement. This suggests a balance between recognizing standardized measures of competence and other individual attributes, which doesn't neatly fit into a political spectrum.<br><br>3. The caution against state regulation of medical credentialing could be interpreted as a conservative viewpoint favoring less government intervention. However, the concern is more about potential unintended consequences and maintaining professional standards rather than a broader ideological stance.<br><br>4. The call for states to ensure fair and transparent processes and to avoid overregulating professional standards might resonate with both conservative ideals of limited government and liberal ideals of fair treatment and transparency.<br><br>Overall, the text presents a position more focused on professional ethics and standards within the medical field rather than a clear-cut liberal or conservative political ideology.	neutral
ACP	ACP Professional Accountability Principles		The text you've provided outlines various aspects of accountability and professionalism, particularly in the context of the medical profession. It doesn't align specifically with a liberal or conservative viewpoint but rather discusses broader concepts applicable across various political and ideological spectrums.<br><br>- **Accountability:** The description of accountability focuses on the obligations and responsibilities of parties (in this case, physicians) to justify their actions and be held responsible. This concept is fundamental to both liberal and conservative ideologies, as both advocate for accountability in different contexts and to varying degrees.<br><br>- **Professionalism and Internal Professional Accountability:** These concepts emphasize a commitment to ethical standards and values within a professional community. This idea is generally supported across the political spectrum as it promotes integrity and responsibility in professions.<br><br>- **External Professional Accountability:** This includes public, regulatory, and market perspectives. Each perspective reflects a different aspect of accountability:<br>- **Public Perspective:** This suggests a social contract between the profession and society, a concept that can resonate with both liberal and conservative ideals, albeit in different ways.<br>- **Regulatory Perspective:** This involves adherence to governmental or healthcare entity standards. Conservatives might emphasize less regulation and more market-driven solutions, while liberals might focus on the importance of regulation to ensure public good.<br>- **Market Perspective:** This emphasizes accountability to the market and consumer/patient expectations. Conservatives generally favor market-based solutions and might support this perspective more strongly, while liberals might advocate for a balanced approach where the market is one of several mechanisms ensuring accountability.<br><br>While the text doesn't overtly favor one political ideology over another, individuals with different political beliefs might emphasize different aspects of accountability and professionalism based on their own values and priorities. For instance, a conservative might focus more on market accountability and less on regulatory aspects, while a liberal might emphasize the role of government and regulations in ensuring accountability and upholding the social contract.	neutral

ACP	Principles Regarding ACP and Professional Accountability		The text you've provided outlines standards and values for professional accountability, continuous self-scrutiny, and regulation within a medical college, specifically the American College of Physicians (ACP). It doesn't explicitly align with a liberal or conservative viewpoint as it primarily focuses on professional ethics, certification, and standards within a specific field rather than broader political or social ideologies.<br><br>However, certain aspects can be interpreted as leaning towards one viewpoint or another based on how they resonate with broader liberal or conservative values:<br><br>1. **Emphasis on self-regulation and professional accountability**: This could align with conservative values that emphasize personal responsibility and self-regulation rather than external or governmental oversight.<br><br>2. **Non-profit, transparent, and inclusive certification**: The emphasis on non-profit status, transparency, and accommodation could resonate with liberal values, particularly the focus on inclusivity and transparency.<br><br>3. **Continuous self-scrutiny and professional development**: This could be seen as aligning with liberal ideals of continual improvement and progressive development, or with conservative ideals of personal responsibility and self-improvement.<br><br>4. **Conflict-of-interest protections and quality control**: These could align with both liberal and conservative values, as both sides generally advocate for ethical standards and integrity, though the methods and emphasis might differ.<br><br>In summary, the text is professional and technical, primarily concerned with ethical and professional standards within a specific field. While it doesn't directly align with a political ideology, different aspects of it might resonate more with one side based on broader values and priorities.	neutral
ACP	Principles Guiding External Regulatory and Market Accountability		The text you've provided outlines principles and guidelines for regulatory or market entities holding physicians accountable. It focuses on transparency, meaningful engagement, flexibility, and non-burdensomeness in evaluation processes, as well as emphasizing the importance of quality control, appeals processes, public reporting, and comprehensive criteria for licensure and credentialing.<br><br>This text doesn't align strictly with a liberal or conservative viewpoint but incorporates elements that might be emphasized differently by each side:<br><br>1. **Liberal Viewpoint Affinities**:<br>- Emphasis on transparency and accountability could resonate with liberals, who often advocate for clear and open governance.<br>- The focus on diverse assessment methods and non-redundancy might align with liberal values of inclusiveness and efficiency in systems.<br>- The push for patient education and informed healthcare choices aligns with liberal tendencies to support consumer rights and empowerment.<br><br>2. **Conservative Viewpoint Affinities**:<br>- The text's emphasis on non-burdensomeness and balancing cost and time sensitivities could appeal to conservatives, who often prioritize efficiency and cost-effectiveness in regulations.<br>- The idea of avoiding absolute prerequisites for licensure and credentialing might resonate with a conservative preference for less rigid regulations and more individual assessment.<br>- The call for a standardized set of performance measures and data collection methodology could align with conservative values of consistency and predictability in regulatory processes.<br><br>In summary, the text seems to strive for a balanced approach that ensures rigorous, robust, and fair accountability for physicians while also being mindful of practicality and efficiency. It does not strictly adhere to liberal or conservative ideologies but rather incorporates aspects that both sides might find valuable in the context of healthcare regulation and physician accountability.	neutral

ACP	Principles underlying the efforts of the Federation of State Medical Boards (FSMB) to establish a Maintenance of<br>Licensure (MOL) process focused on the assuring of continuous physician competence		The text you've provided outlines principles and considerations for maintaining licensure for physicians, emphasizing lifelong learning, administrative feasibility, collaboration, state authority, patient care, flexibility, and a balance between transparency and privacy. Here's how these points might align with liberal or conservative viewpoints:<br><br>1. **Lifelong Learning and Improvement**: Both liberals and conservatives might agree on the importance of ongoing education and improvement in the medical profession. This principle is generally non-partisan, focusing on the betterment of medical services.<br><br>2. **Administrative Feasibility and Collaboration**: Conservatives might favor this point more as it suggests a streamlined, less bureaucratic approach. However, liberals might also support it if the collaboration includes a wide range of stakeholders, ensuring comprehensive and inclusive policymaking.<br><br>3. **State Authority**: This point might resonate more with conservative ideologies, which often emphasize states' rights and local governance over federal oversight. Liberals might support state authority too but could be more cautious to ensure uniformity and equity across states.<br><br>4. **Non-Compromise on Patient Care and Physician Practice**: Both sides would likely agree that patient care should not be compromised. However, conservatives might focus more on the autonomy and flexibility for physicians, while liberals might stress ensuring quality and safety standards.<br><br>5. **Flexible Infrastructure and Choice**: This principle might appeal more to conservatives due to its emphasis on flexibility and choice, aligning with free-market principles and individual autonomy. Liberals might support it too, provided the choices are equitable and accessible to all physicians.<br><br>6. **Balance of Transparency and Privacy**: Both liberals and conservatives value transparency and privacy, though the emphasis might differ. Conservatives might focus more on the rights of individual physicians (privacy), while liberals might emphasize the need for public accountability and transparency.<br><br>In summary, while some points might slightly lean towards one ideology, the principles outlined are largely non-partisan and focused on improving the medical licensure system in ways that could be supported by both liberal and conservative viewpoints. The emphasis is on practical, equitable, and quality-focused reforms rather than ideological divisions.	neutral

ACP	Long-Term Services and Supports for Older Adults		The text presents a series of recommendations from the American College of Physicians (ACP) on long-term services and supports (LTSS). It doesn't strictly align with a liberal or conservative viewpoint exclusively, as the suggestions incorporate elements that might resonate with both sides. Here's a breakdown:<br><br>1. **Liberal Viewpoints**:<br>- Support for a publicly funded, universal catastrophic LTSS insurance program suggests a more liberal stance, advocating for a significant role of the government in providing a safety net for all.<br>- Policies to promote expanded Home and Community-Based Services (HCBS) through Medicaid and other programs also align with more liberal policies that focus on expanding government-funded healthcare services.<br>- Emphasis on comprehensive training, pay increases, and benefit packages for LTSS sector workers reflects a liberal focus on worker rights and support.<br><br>2. **Conservative Viewpoints**:<br>- Encouraging private Long-Term Care Insurance (LTCI) through market-friendly policies like standardizing insurance policies and allowing the use of retirement funds for LTCI could resonate more with conservative ideals of private sector solutions and personal responsibility.<br>- Support for research into the effect of ownership status on the LTSS sector, including quality and cost, might align with conservative interests in transparency, efficiency, and the role of private equity.<br><br>3. **Bipartisan/Neutral Aspects**:<br>- Evidence-based interventions to assure and improve the quality of LTSS, like robust monitoring and the development of quality measures, are generally non-partisan and focus on practical outcomes.<br>- Support for emergency preparedness plans and coordination with public health departments is a broadly agreeable point that emphasizes safety and readiness, crossing ideological lines.<br><br>In summary, the text proposes a multipronged approach that includes both government-funded programs and support for private insurance options, as well as a strong emphasis on quality and safety. This blend of strategies suggests an effort to incorporate a wide range of ideas that might attract support from across the political spectrum. However, individual readers' interpretations might vary based on their own political perspectives and how they weigh the various components of the proposal.	neutral
ACP	Long Term Care	2019	The text reflects a viewpoint that aligns more with liberal ideologies, typically associated with the Democratic Party in the United States. It emphasizes the expansion of federal projects and the removal of administrative barriers to facilitate state experimentation in delivering integrated long-term care. Liberals generally advocate for increased government involvement in healthcare and support programs that provide services to vulnerable populations, including the elderly and disabled. The call for expanding federal initiatives and promoting integration of services is consistent with liberal priorities of ensuring access to healthcare and supporting social safety nets. However, it's worth noting that healthcare reform and support for the elderly and disabled can be bipartisan issues, with variations in approach between conservatives and liberals.	liberal
ACP	Regulatory Oversight of Boarding Care Facilities	2019	The statement provided doesn't align explicitly with a liberal or conservative viewpoint. It's primarily a policy stance by a medical organization (ACP - American College of Physicians, presumably) focusing on improving regulatory oversight of boarding care facilities. Both liberal and conservative ideologies might agree on the need for quality healthcare facilities but could differ on how to achieve it. Liberals might favor more government intervention and regulation, while conservatives might prefer less government involvement and more market-based solutions. However, this statement focuses on the support and dissemination of information rather than advocating for a specific governmental approach, making it more neutral and broadly focused on health care improvement.	neutral
ACP	Supervision of Care of Patients in Extended Care Facilities	2019	The text you've provided seems to be a policy or statement regarding the administration of care in extended care facilities. It mandates that all patient care in facilities like Skilled Nursing Facilities, Intermediate Care Facilities, and Residential Facilities be conducted only under the orders of an attending physician or their designee. This statement doesn't explicitly align with a liberal or conservative viewpoint as it primarily focuses on procedural standards for healthcare delivery rather than broader ideological issues.<br><br>Both liberals and conservatives might agree on the importance of professional medical oversight in patient care, although they might differ on other healthcare policy aspects such as funding, access, and the role of government. Liberals might emphasize the need for accessible, comprehensive healthcare, possibly supporting more government involvement, while conservatives might focus on personal responsibility and market-based solutions. However, this particular text is neutral and focused on ensuring medical professionalism and safety in extended care settings, without delving into the more divisive, ideological aspects of healthcare policy.	neutral

ACP	Physicians Visits to Nursing Home Patients	2015	The statement from the American College of Physicians (ACP) regarding the belief that medical necessity should dictate the frequency of physician visits to nursing home patients doesn't inherently align with a specific liberal or conservative viewpoint. It's a policy position centered on healthcare practice and patient needs rather than political ideology.<br><br>Liberal perspectives generally emphasize comprehensive healthcare access and might support this policy if it's seen as ensuring necessary care without unnecessary restrictions. Conservatives might also agree if they view the policy as a way to reduce regulatory burdens and promote efficiency in healthcare.<br><br>However, the interpretation could vary among individuals within each group, depending on how they balance concerns of healthcare quality, cost, government regulation, and individual autonomy. The statement focuses on practical healthcare delivery, so it's more about medical ethics and efficiency than political ideology.	neutral
ACP	"Swing Bed" Concept	2015	The text you've provided discusses the American College of Physicians (ACP) endorsement of the "swing bed" concept as a solution to the shortage of skilled nursing facility beds. This concept allows hospitals to use their beds to provide either acute or post-acute care as needed. It's important to understand that policies or endorsements like this don't neatly align with a liberal or conservative viewpoint in a straightforward manner. Instead, they reflect practical considerations and solutions to healthcare issues.<br><br>From a policy standpoint, conservatives typically emphasize reducing government spending and regulations, while liberals often focus on expanding access to healthcare and government involvement in ensuring healthcare coverage. The "swing bed" concept can be seen as a way to make more efficient use of existing resources (which could align with a conservative preference for efficiency and reduced spending) and as a means to increase access to necessary care (aligning with a liberal emphasis on access to healthcare). However, it's not explicitly partisan and likely has aspects that both liberal and conservative viewpoints might find appealing for different reasons.<br><br>In the context of healthcare policy, it's more productive to consider the specific goals and outcomes of a policy rather than trying to label it broadly as liberal or conservative. This approach allows for a more nuanced understanding of how various policies might align with different political philosophies or pragmatic healthcare needs.	neutral
ACP	Financing Long Term Care Benefits	2019	The text provided aligns more closely with a liberal viewpoint. Key indicators include:<br><br>1. **Support for Subsidies and Sliding Scale Benefits**: The advocacy for minimizing out-of-pocket expenses and providing a sliding scale subsidy for low-income beneficiaries reflects a liberal stance on social welfare and economic equality. Liberals typically support policies that aim to reduce economic disparities and provide assistance to those in lower income brackets.<br><br>2. **Additional Funding Mechanisms Beyond Beneficiaries**: Suggesting that the responsibility for financing Medicare long-term care benefits should extend beyond the beneficiary community, through measures like increased excise taxes on alcohol and tobacco, is indicative of a liberal approach. This perspective often entails a broader view of shared societal responsibility for healthcare and welfare programs.<br><br>3. **Encouraging Private Sector Solutions with Public Support**: While encouraging private sector solutions like long-term care asset protection insurance, the text also advocates for public programs that assist individuals in preserving their assets from being depleted due to healthcare costs. This balance of private and public sector involvement is a hallmark of many liberal policy approaches, seeking to blend market mechanisms with social safety nets.<br><br>4. **Expansion of Medicare and Medicaid**: The reference to expanding Medicare to include long-term care benefits and adjusting Medicaid eligibility to protect individual assets further aligns with liberal policies. These programs are traditionally supported and sought to be expanded by liberals who view government as a key player in ensuring healthcare access for all, especially the vulnerable populations.<br><br>In summary, the focus on reducing economic barriers to healthcare, expanding public healthcare programs, and introducing progressive funding mechanisms are all indicative of a liberal policy viewpoint.	liberal

ACP	Position Paper on Long Term Supports for Older Adults	2022	The text provided outlines the American College of Physicians' (ACP) recommendations for reforming long-term services and supports (LTSS). It doesn't explicitly align with a liberal or conservative viewpoint as it focuses on pragmatic solutions and improvements in healthcare. However, the policies suggested can be interpreted through both liberal and conservative lenses:<br><br>1. **Liberal Viewpoint:**<br>- **Support for Public Programs:** The call for a publicly funded, universal catastrophic LTSS insurance program aligns with liberal ideals of expanding public sector solutions and safety nets for healthcare.<br>- **Emphasis on Regulation and Standards:** Proposals for standardizing insurance policies and increasing funding for home and community-based services are typically supported by liberals who favor regulatory approaches to ensure quality and accessibility.<br>- **Workforce Support and Rights:** Policies aimed at improving training, pay, and benefits for workers in the LTSS sector resonate with liberal values concerning labor rights and worker protections.<br><br>2. **Conservative Viewpoint:**<br>- **Private Sector Involvement:** The emphasis on complementing public insurance with private long-term care insurance and promoting private sector solutions, such as hybrid insurance policies, can appeal to conservative values of market-based solutions and limited government.<br>- **Personal Responsibility and Preparedness:** Encouraging individuals to prepare for future LTSS needs and use personal funds (like retirement accounts) for private insurance aligns with the conservative emphasis on personal responsibility and self-reliance.<br>- **Research and Quality Improvements:** Support for research, quality improvements, and transparency in ownership and costs might be seen as efforts to enhance efficiency and effectiveness, principles often valued in conservative thought.<br><br>Overall, the ACP's recommendations represent a balanced approach, incorporating elements that can be supported by both liberal and conservative viewpoints. The focus on a mix of public and private sector solutions, emphasis on quality and efficiency, and support for workforce and consumer protections reflect a comprehensive strategy aimed at improving LTSS for all, rather than a strictly partisan stance.	neutral
ACP	Patient Protection Legislation	2022	The text from the American College of Physicians (ACP) outlines principles for patient protection legislation. It doesn't neatly align with traditional liberal or conservative viewpoints as these terms are commonly understood in the U.S. political context, but it does reflect certain values and priorities that can be associated with broader political ideologies:<br><br>1. **Patient-Centered Care**: The emphasis on physician over health plan decision-making and evidence-based definitions of medical necessity aligns with a viewpoint that prioritizes patient welfare and medical professionalism. This can be seen as a progressive stance, advocating for the rights and well-being of patients in the healthcare system.<br><br>2. **Access and Accountability**: Advocating for timely access to review processes, independent reviews, and a point-of-service option suggests a commitment to ensuring that patients have choices and recourse in their healthcare. This might resonate more with liberal ideologies that emphasize individual rights and protections.<br><br>3. **Regulatory Oversight**: The call for holding all health plans accountable, including those exempt from state regulation, suggests a belief in the need for regulatory oversight to protect patients. This could be seen as aligning more with a liberal perspective that supports the role of government in ensuring individual welfare and safety.<br><br>4. **Universal Application**: The principle that the legislation should apply to all insured Americans reflects a universalist approach, which is more commonly associated with liberal ideologies that focus on inclusivity and comprehensive protections.<br><br>However, it's important to note that these positions do not exclusively belong to any single political ideology. There are conservatives who might support these measures out of a concern for patient rights and market transparency, and liberals who might oppose some elements if they believe the measures don't go far enough or have other concerns. Health policy often transcends simple left-right politics, with different stakeholders (patients, providers, insurers, employers) having varied and sometimes overlapping interests.	probably liberal

ACP	Medical/Surgical and Psychiatric Service Integration and Reimbursement	2022	The text you've provided outlines the American College of Physicians' (ACP) stance on healthcare policies, advocating for integrated medical and psychiatric care, as well as medically necessary treatment for patients regardless of their condition. This viewpoint doesn't neatly align with traditional liberal or conservative ideologies, as it focuses on healthcare access and quality, which can be points of interest for both sides.<br><br>Liberal viewpoints typically emphasize the importance of accessible healthcare for all and might support the ACP's stance as it advocates for comprehensive care and access. On the other hand, conservatives often prioritize personal responsibility and free-market solutions in healthcare but might also agree with the need for efficient, quality care that the ACP advocates for, especially if it can be shown to reduce overall healthcare costs and improve outcomes.<br><br>Thus, the ACP's position might find support across the political spectrum, focusing on patient care and healthcare standards rather than fitting into a strict liberal or conservative viewpoint. It's more about the practical and ethical aspects of healthcare rather than a political ideology.	neutral
ACP	Appealing Managed Care Plans' Denials of Medical Care	2022	The text outlines policy recommendations from the American College of Physicians regarding the regulation of managed care plans. It emphasizes prompt and qualified decision-making, specialty relevance, timely appeals, and protection against retrospective payment denial. This perspective leans towards a liberal viewpoint in its advocacy for robust federal regulation to ensure patient care quality and physician support. It suggests a more active government role in healthcare, aligning with liberal principles of regulatory oversight and consumer protection in healthcare. However, some conservatives might also support aspects of these proposals that aim to improve healthcare efficiency and accountability. The text represents a specific stance within the broader debate on healthcare management and regulation.	probably liberal
ACP	Patient Choice of Health Plans and Physicians	2015	The text you've provided focuses on patient choice and transparency in healthcare. These principles don't exclusively align with either a liberal or conservative viewpoint as they encompass values that can be appreciated across the political spectrum.<br><br>1. **Patient Choice of Health Plans**: Advocating for patient choice in selecting health plans is a principle that resonates with both liberal and conservative ideologies. Liberals may support this as part of ensuring access to a range of healthcare options, including government and private plans. Conservatives might appreciate the emphasis on personal freedom and market dynamics in allowing patients to choose among competitors.<br><br>2. **Freedom to Choose Physicians**: The idea that patients should not be "locked-in" to one physician and can choose their care providers aligns with a universal value of personal freedom in healthcare. Conservatives might particularly appreciate this stance as it reflects free-market principles and individual autonomy. Liberals might also support this view in the context of ensuring patient rights and access to the best possible care.<br><br>3. **Informed of Restrictions**: The emphasis on patients being informed about restrictions in access to specialists is a principle of transparency that's widely valued. Conservatives might see this as a way to empower consumers in a healthcare market, while liberals might view it as a necessary protection for patient rights and informed decision-making.<br><br>In summary, the text promotes patient autonomy, choice, and transparency in healthcare—values that can be endorsed by individuals across the political spectrum for different reasons. It doesn't neatly fit into a "liberal" or "conservative" box but instead reflects a blend of principles that are important to many people, regardless of political affiliation.	neutral

ACP	Internists’ Role in a Managed Care Setting	2019	The text you've provided appears to be a series of policy statements from a medical organization (presumably the American College of Physicians (ACP)) regarding the roles of internists in managed care, the importance of physician-led patient care management, and the support for scope of practice based on training and expertise. Here's how the content might align with traditional liberal or conservative viewpoints:<br><br>1. **Support for Managed Care and Reimbursement Methods**: This point emphasizes the role of internists in managed care and the importance of recognizing and reimbursing primary and consultative services. **Liberal** perspectives generally favor more structured approaches to healthcare delivery and financing, which includes support for managed care systems that aim to improve quality and reduce costs. However, conservatives might also agree with the aspect of proper recognition and reimbursement, as it aligns with free-market principles if it promotes competition and quality.<br><br>2. **Physicians as Patient Care Managers**: Advocating for physicians, particularly internists, as leaders in patient care management could resonate with both viewpoints. **Conservatives** might favor this approach as it emphasizes professional autonomy and could be seen as a counter to bureaucratic healthcare systems. **Liberals** might support it as well, especially if it's part of a broader plan to improve healthcare access and outcomes through coordinated care.<br><br>3. **Scope of Practice Based on Training and Expertise**: This point advocates for scope of practice legislation that reflects physicians’ training and expertise. Both **liberals** and **conservatives** might agree with this principle, but for different reasons. **Liberals** might see it as a way to ensure high-quality care and patient safety, while **conservatives** might view it as a means to maintain professional standards and reduce unnecessary regulatory interference.<br><br>Overall, while the statements focus on healthcare policy and physician roles, they don't neatly fit into a strictly liberal or conservative viewpoint. They combine elements of professional autonomy, quality care, and efficient healthcare delivery that could be supported by individuals across the political spectrum depending on the context and implementation. The text seems more focused on professional standards and healthcare quality than on broader political ideologies.	neutral
ACP	Definition of Principal Care Services	2017	The text provided describes a model of healthcare known as the "Advanced Medical Home," emphasizing personalized, coordinated care primarily led by a primary care physician or, alternatively, a medical specialist. It doesn't explicitly align with a liberal or conservative viewpoint as it focuses more on a healthcare delivery model rather than a political ideology.<br><br>In the U.S. political context, liberals generally advocate for more comprehensive healthcare reforms, often supporting universal coverage or expanded government involvement to ensure access and equity. Conservatives typically favor market-based solutions, emphasizing private sector roles and personal choice.<br><br>The Advanced Medical Home model can potentially find support across the political spectrum. Conservatives might appreciate the emphasis on personal choice and the flexibility for specialists to act as primary care providers. At the same time, liberals might support the model's focus on comprehensive, coordinated care, which aligns with broader goals of improving healthcare access and outcomes.<br><br>Ultimately, the alignment with political ideologies would depend on the specific policies and implementations surrounding this model, such as how it's funded, the role of insurance, and regulations guiding the care. Without explicit details on these aspects, the text remains more of a healthcare management approach than a strictly political statement.	neutral
ACP	Definition of Primary Care Services	2019	The text outlines the American College of Physicians' (ACP) definition and guidelines for primary care services. It does not directly align with a liberal or conservative political viewpoint. Instead, it focuses on the medical and practical aspects of primary care, emphasizing comprehensive, accessible, and continuous healthcare. The principles listed are generally accepted professional standards in the medical community, advocating for quality patient care, preventive services, and coordination of treatment.<br><br>Political viewpoints might come into play more in discussions about how to implement these principles, such as in debates over healthcare policy, funding, and access, rather than in the medical definitions and standards themselves. Liberals might focus on broad access and support for these services through public funding or regulation, while conservatives might emphasize market-based solutions and individual choice. However, the text you've provided is strictly professional and neutral, outlining standards for care rather than a political approach to healthcare.	neutral

ACP	Physician Credentialing	2022	The text provided outlines policies and suggestions for streamlining the credentialing process for physicians within managed care plans. It doesn't strictly align with either a liberal or conservative viewpoint as it focuses on healthcare management efficiency and quality rather than broader ideological issues.<br><br>However, some aspects could be interpreted through different political lenses:<br><br>1. **Liberal Viewpoint**: Liberals might appreciate the emphasis on standardized and fair processes, ensuring that all physicians are evaluated equitably and have access to a fair appeals process. The involvement of physicians in developing these processes could also be seen as a positive, democratic approach to healthcare reform.<br><br>2. **Conservative Viewpoint**: Conservatives might value the efficiency and potential cost-saving measures of a uniform credentialing process. The focus on education, training, experience, and demonstrated competence aligns with merit-based evaluations, a concept often supported in conservative circles. The idea of reducing redundancy and bureaucracy in healthcare could also appeal to conservative ideals of smaller, more efficient government.<br><br>Ultimately, the text is more technical and administrative rather than ideological, aiming to improve healthcare management rather than propose a shift in healthcare policy or philosophy. Therefore, while certain elements might resonate more with one political ideology, the text as a whole doesn't squarely fit into a liberal or conservative viewpoint.	neutral
ACP	Physician Contracting	2015	The text outlines the American College of Physicians' (ACP) position on federal preemption of state laws concerning health plans and physician participation. It's not strictly aligned with either a liberal or conservative viewpoint; rather, it intersects with various aspects of both.<br><br>On one hand, the support for federal preemption over state laws could be seen as favoring a more centralized approach to regulation, which is typically associated with liberal politics. The emphasis on protecting the interests of patients and ensuring fair and transparent criteria for physician participation also aligns with liberal values of equity and consumer protection.<br><br>On the other hand, the focus on professional competence, quality of care, economic considerations, and the right of physicians to appeal decisions, engage in multiple plans, and be protected from termination without cause can resonate with conservative values. These points emphasize market principles, individual rights, and limiting regulatory overreach, which conservatives often advocate for.<br><br>Overall, the position reflects a blend of priorities: patient protection and quality of care (more liberal) with an emphasis on professional autonomy and market mechanisms (more conservative). It's a nuanced stance that seeks to balance regulatory oversight with individual rights and market dynamics.	neutral
ACP	Prohibition on Gag Clauses	2019	The text reflects the viewpoint of the American College of Physicians (ACP) on patient-physician communication. It advocates for the physician's right to share all pertinent medical care or treatment options with the patient, emphasizing the patient's best medical interest. This stance doesn't align strictly with liberal or conservative ideologies. Instead, it focuses on medical ethics and patient rights. Both liberal and conservative individuals might agree with this principle, as it relates to ensuring patients are fully informed about their treatment options and supports the physician's autonomy to communicate openly. The primary aim here is to enhance patient care and support informed decision-making, transcending political categorization to focus on healthcare principles.	neutral
ACP	Availability of Physician Payment Information	2019	The text provided outlines policies regarding transparency in health insurance plans and the disclosure of payment methodologies to physicians and patients. It doesn't explicitly align with a conservative or liberal viewpoint as it focuses on transparency and accountability in healthcare compensation. Both liberal and conservative ideologies might support these measures for different reasons:<br><br>- **Liberal Viewpoint**: Liberals generally advocate for transparency in healthcare to empower patients and ensure fair practices. They might support these policies as a step toward more comprehensive healthcare reform, arguing that knowledge of compensation arrangements can lead to better patient choices and more equitable healthcare.<br><br>- **Conservative Viewpoint**: Conservatives often prioritize market efficiency and informed consumer choices. They might favor these policies under the belief that transparency leads to competition and efficiency in the healthcare market, ensuring that services are priced fairly and providers are accountable.	neutral

			The text seems to be more about pragmatic healthcare policy than ideological alignment. It could be supported by either side for different reasons related to their broader goals for healthcare reform and market regulation.	
ACP	Assuring Physician Reimbursement, Incentives, and Financial Risk Sharing Do Not Compromise Patient Care	2019	The text you've provided appears to focus on detailed policy recommendations for managing financial risk in health care, particularly around capitation arrangements in managed care plans. It doesn't align strictly with a liberal or conservative viewpoint as understood in the general political sense. Instead, it's more technical and specific to health care policy, focusing on ensuring quality patient care, fair compensation for physicians, and transparency in health plans.<br><br>Here's how different aspects of the text might be perceived through a liberal or conservative lens:<br><br>1. **Liberal Perspective**:<br>- **Pro-Patient Care**: Advocating for quality care and protection against financial risk might align with a liberal emphasis on patient rights and access to comprehensive health care.<br>- **Regulation**: Calls for more regulation and oversight of health plans might resonate with liberal views favoring stronger consumer protections.<br><br>2. **Conservative Perspective**:<br>- **Market Solutions**: While the text calls for regulation, it also discusses restructuring financial arrangements and offering various plans, which could align with a conservative preference for market-based solutions and choice in health care.<br>- **Physician Autonomy**: Emphasizing fair compensation and protection for physicians might resonate with conservative values of professional autonomy and minimizing government interference.<br><br>However, these points are nuanced. Both liberals and conservatives are concerned with efficient, effective health care, but they often differ on the methods to achieve it. This text seems to aim for a middle ground, focusing on pragmatic solutions to improve managed care rather than espousing a clear ideological stance. It's more about practical, technical reforms within the existing health care system, which could garner support across the political spectrum depending on the specific context and framing.	neutral
ACP	Physician and Health Plan Liability	2022	The text you've provided outlines policies and positions regarding healthcare management, liability, and patient rights. Here's how it aligns with typical liberal and conservative viewpoints:<br><br>1. **Managed care organizations' responsibility and liability**: This point leans more toward a liberal viewpoint. Liberals generally advocate for strong consumer protections and hold organizations accountable for their services. Ensuring quality healthcare and holding organizations liable for negligence align with the liberal emphasis on regulation and protection in healthcare.<br><br>2. **Modifying ERISA laws and allowing claims in state courts**: This also aligns more with a liberal perspective. Liberals often support the idea of modifying existing laws to provide more avenues for individuals to seek redress and justice, particularly in healthcare. Allowing personal injury and wrongful death cases against health plans in state courts is an expansion of accountability and legal recourse, which is typically more favored by liberals who seek comprehensive protections for individuals.<br><br>3. **Opposition to physician liability when restricted by managed care plans**: This point could be seen as somewhat bipartisan but still generally aligns with a liberal viewpoint. Liberals tend to support protecting individual workers and professionals from undue consequences, especially when their decisions are restricted by larger organizations or systems. However, conservatives might also agree with this point to some extent, as it reduces the burden of liability on individual physicians, aligning with a more deregulatory approach.	liberal

			In summary, the text predominantly aligns with a liberal viewpoint due to its emphasis on regulation, consumer protections, and expanding legal recourse for individuals against large organizations. However, certain aspects, like opposing physician liability, may find some cross-over appeal with conservative viewpoints focused on reducing individual burdens and opposing expansive litigation.	
ACP	Health Plan Marketing Standards	2015	The text you've provided outlines positions on health insurance sales practices and standards for marketing health benefits plans. The viewpoints focus on advocating for legislation to prevent deceptive sales tactics and ensure accurate information in marketing health benefits plans. Here's how these align with general liberal or conservative viewpoints:<br><br>1. **Liberal Viewpoint**: Generally, liberal ideology tends to support increased regulation and oversight, especially in areas like healthcare and consumer protection. The call for legislation to prevent fraudulent and deceptive practices aligns with a liberal approach to government intervention to protect consumers and ensure fair practices in the market.<br><br>2. **Conservative Viewpoint**: Conservatives typically advocate for less government intervention in business and a free-market approach. However, conservative ideology also supports the rule of law and protecting citizens from fraud and deception. Therefore, while some conservatives might be wary of increasing regulation, many would also support measures to prevent outright fraud and abuse.<br><br>The specifics in these statements, such as the emphasis on transparency and penalties for deceptive practices, do not strictly align with one ideology. They reflect a pragmatic approach to healthcare marketing, focusing on consumer protection and market transparency, which can resonate with both liberal and conservative values to varying degrees. The balance between regulation and free market principles is often a point of debate between the two ideologies, with these positions potentially finding support across the spectrum depending on the broader context and specific legislative proposals.	neutral
ACP	For-profit Conversion of Health Care Organizations	2022	The text outlines recommendations from the American College of Physicians (ACP) regarding the conversion of nonprofit health care organizations to for-profit entities. The viewpoint expressed doesn't neatly align with traditional liberal or conservative ideologies but contains elements that both sides might support or critique.<br><br>From a **liberal perspective**:<br>- **Supportive Elements**: Liberals generally advocate for stronger government oversight and public involvement in health care to ensure equity and prevent profiteering. The emphasis on state oversight, public hearings, and preventing private inurement aligns with liberal views favoring regulation and transparency to protect public interests.<br>- **Critiques**: Some liberals might argue that the recommendations don't go far enough in preventing the privatization of healthcare or ensuring universal access.<br><br>From a **conservative perspective**:<br>- **Supportive Elements**: Conservatives might appreciate the recommendations for independent appraisals and the establishment of charitable foundations, as these can promote fiscal responsibility and philanthropy, which align with conservative values of efficiency and private sector solutions.<br>- **Critiques**: Conservatives typically favor less government intervention and might view the extensive oversight and regulations as burdensome and a hindrance to free market solutions in healthcare.	neutral

			**Overall**, the text promotes transparency, accountability, and community involvement in the conversion process of health care organizations, which are principles that can transcend political ideologies. The balance between regulation and encouraging private sector solutions might receive varying degrees of support from both sides, depending on the broader context of their health care and economic philosophies.	
ACP	Accountability of Medical Director	2022	The text you've provided seems to advocate for accountability and fairness in healthcare decision-making, particularly within managed care plans. It suggests that final decisions on the denial of services based on medical necessity should be made or reviewed by a licensed medical director and that instances of poor judgment should be reportable to state licensing boards. Here's how it aligns with liberal or conservative viewpoints:<br><br>1. **Liberal Viewpoint**: This text may resonate with liberal ideologies which often emphasize the protection of individual rights, the need for accountability in healthcare, and the importance of access to quality care. The focus on holding medical directors accountable and ensuring decisions are made by qualified professionals aligns with a liberal emphasis on regulation and oversight to protect consumers and patients.<br><br>2. **Conservative Viewpoint**: On the other hand, conservatives might appreciate the emphasis on professional accountability and the use of state licensing boards to oversee medical practice. This aligns with conservative principles of professionalism, individual responsibility, and limited, but effective, regulatory measures to ensure quality and fairness.<br><br>Overall, the text doesn't neatly fit into a purely liberal or conservative viewpoint. It combines elements that both sides might agree on: the importance of professional accountability and the need for fair and medically necessary healthcare services. It reflects a pragmatic approach to healthcare management that focuses on patient safety, professional accountability, and the quality of care, principles that can transcend political divides.	neutral

ACP	Utilization Review (UR) and Utilization Management (UM)	2019	The text provided outlines various policies and guidelines for managed care plans and utilization review/management (UR/UM) processes. It focuses on ensuring patient welfare, advocating for evidence-based medical practices, demanding transparency from managed care plans, and safeguarding the rights and roles of physicians in the healthcare system.<br><br>This text doesn't neatly align with traditional liberal or conservative viewpoints as understood in a political context. Instead, it's more closely aligned with healthcare policy reform and medical ethics. Here's how elements of both liberal and conservative viewpoints might interpret different aspects of this text:<br><br>### Liberal Viewpoint Alignment:<br>- **Patient Welfare:** Liberals often advocate for policies that prioritize individual welfare and rights, including in healthcare. The emphasis on never placing physician financial incentives over patient welfare aligns with a more progressive view of healthcare as a right.<br>- **Transparency and Regulation:** The call for transparency in UR/UM criteria and the requirement for managed care plans to disclose information are in line with liberal advocacy for consumer rights and informed decision-making in healthcare.<br><br>### Conservative Viewpoint Alignment:<br>- **Efficiency and Cost-Effectiveness:** The points about ensuring that preauthorization is only required when it is proven to reduce medically unnecessary services and doesn't cost more than it saves could resonate with conservative ideals of efficiency and cost containment in healthcare.<br>- **Standardization and Reducing Overutilization:** The focus on targeting medical procedures that are consistently overutilized and the call for standardization may align with conservative preferences for reducing unnecessary government or administrative bloat in healthcare.<br><br>### Neutral/Professional Stance:<br>- Many points, such as advocating for due process in appeals, ensuring UR/UM processes are educational, and not basing compensation on care denials, reflect professional ethical standards and best practices in medicine rather than a political ideology.<br><br>In conclusion, while certain aspects of the text might resonate more with one political ideology over another, the overall content is primarily centered around improving healthcare management, transparency, and ethics, transcending a simple liberal vs. conservative classification. It represents a nuanced approach to healthcare policy that incorporates elements valued by both sides of the political spectrum.	neutral
ACP	Concurrent Review of Inpatient Care	2015	The text you've provided outlines principles regarding the utilization review in managed care plans, focusing on issues like credentialing of reviewers, use of evidence-based medical protocols, transparency, and communication between review entities and healthcare providers. It doesn't explicitly align with a liberal or conservative viewpoint as it mainly addresses healthcare management and quality assurance practices. Both liberal and conservative perspectives might agree on the importance of such measures for different reasons:<br><br>- **Liberal Viewpoint**: Might support these principles as they emphasize evidence-based practice, transparency, and accountability in healthcare. Liberals typically advocate for strong patient protections and might see these guidelines as a way to ensure quality care and prevent unnecessary or inappropriate treatments.<br><br>- **Conservative Viewpoint**: Might appreciate the focus on efficient healthcare management and the potential for cost-saving through proper utilization review. Conservatives often emphasize the need for market-based solutions in healthcare and might view these principles as a way to maintain quality while controlling costs.<br><br>In the complex field of healthcare policy, ideas about management and quality assurance can cross ideological lines. Both sides might find aspects of these principles to align with their broader goals for the healthcare system, even if they approach them from different perspectives.	neutral

ACP	Physician Run Health Plans, Professional Accountability, and Anti-Trust Considerations	2019	The text you've provided outlines a stance by the American College of Physicians (ACP) on physician-led integration in healthcare, emphasizing the importance of professional values, physician leadership, quality, and accountability. It doesn't align strictly with a liberal or conservative viewpoint; rather, it addresses healthcare management and physician roles in a manner that could be supported by individuals across the political spectrum. Here's a breakdown of how different elements might be perceived:<br><br>1. **Physician-led integration and professional values**: This aspect is more aligned with a perspective that values individual professional judgment and leadership in healthcare, which can be a principle supported by both liberals and conservatives. It emphasizes the role of medical professionals rather than government or commercial entities in leading healthcare decisions.<br><br>2. **Quality and accountability**: The focus on quality of care and accountability is a universal concern, transcending political lines. The idea of integrating practices to improve quality and efficiency could appeal to conservative ideals of efficiency and liberal ideals of improved healthcare outcomes.<br><br>3. **Transparency and patient rights**: Calling for full disclosure and patient rights is a principle that both sides might agree on, as it emphasizes transparency and individual empowerment.<br><br>4. **Conflict between economic self-interest and professional commitment**: Highlighting this conflict may resonate more with liberal viewpoints concerned with the commercialization and corporatization of healthcare, but the call for a balance also fits with conservative values of professional autonomy and responsibility.<br><br>5. **Education and research**: The emphasis on education and research in medical schools and the support for this by various organizations might be seen as a more progressive stance, focusing on evidence-based practice and long-term investments in healthcare quality.<br><br>6. **Support for physicians and professional societies**: The call for support networks and resources for physicians could be seen as a professional-centric approach, aligning with conservative values of professional autonomy, but the method and the purpose of integration might also resonate with liberals focused on collaborative and quality-driven healthcare.<br><br>In summary, while certain elements might lean slightly towards one political ideology or another, the text as a whole presents a multifaceted approach to healthcare that incorporates values important to both liberal and conservative viewpoints. It's more focused on professional and practical aspects of healthcare delivery than on ideological divides.	neutral
ACP	Establishing Strategy that Uses Anti-Trust Laws to Prevent Insurance Market Domination by One or Few Carriers	2015	The statement from the American College of Physicians (ACP) advocating for changes to anti-trust laws to prevent market domination by a few insurers seems to align more closely with a liberal viewpoint. This perspective often emphasizes the need for regulatory measures to prevent monopolistic practices and to protect consumer and worker rights, which is consistent with the ACP's concern about ensuring patients' freedom to choose insurers and preventing unfair cost increases. However, it's essential to recognize that healthcare policy perspectives can vary widely within both liberal and conservative spectrums and that some conservatives might also support certain antitrust measures for different reasons. The statement focuses on healthcare market fairness and competition, which can be a concern across the political spectrum, though the approach and rationale might differ.	liberal

ACP	Establishing Strategy to Allow Physicians to Collectively Negotiate with Insurers	2016	The statement about the American College of Physicians supporting legislation allowing physicians to jointly negotiate with insurers doesn't inherently align with a traditional liberal or conservative viewpoint. Here's how it might be seen from different perspectives:<br><br>1. **Liberal Viewpoint**: From a liberal perspective, this statement might be seen favorably as it supports collective negotiation, which could be viewed as a way to level the playing field between individual physicians and larger insurance companies. It might be seen as a push for fairer working conditions and greater bargaining power for healthcare providers, which aligns with liberal values of workers' rights and equity.<br><br>2. **Conservative Viewpoint**: From a conservative standpoint, the emphasis might be on the freedom and autonomy of physicians to make decisions and negotiate terms that are best for their practices and patients. They might support this as a measure to promote free-market principles within healthcare, allowing physicians more control and less interference from government entities. However, some conservatives might be wary of any form of collective bargaining if they see it as potentially leading to increased healthcare costs or as a step towards more regulated healthcare systems.<br><br>3. **Non-partisan or Bipartisan Viewpoint**: Some may view this statement as non-partisan, focusing on practical, pragmatic solutions to improve the healthcare system's efficiency and fairness. The idea could be seen as an attempt to improve negotiation dynamics in the healthcare industry, which could benefit patients, providers, and insurers alike, without falling neatly into a liberal or conservative category.<br><br>It's essential to recognize that individuals within each political group might have varying opinions on this issue based on their personal beliefs, values, and interpretations of how this policy would affect the healthcare system. The statement's alignment can depend greatly on the specific context, the details of the legislation, and the broader healthcare policies being discussed.	neutral
ACP	Supporting the Use of Physician Office Labs (POLs) in a Managed Care Setting	2019	The text you've provided outlines various policies and suggestions for managed care plans, focusing on laboratory services within a healthcare setting. These suggestions aim to improve patient access to laboratory tests, ensure quality and cost-effectiveness, and enhance overall patient satisfaction. Here's a breakdown of how the text might align with liberal or conservative viewpoints:<br><br>1. **Liberal Viewpoint Tendencies:**<br>- **Emphasis on Patient Access and Convenience:** Advocating for in-office laboratory tests and against requiring patients to travel for tests aligns with a liberal perspective focusing on patient rights and access to healthcare.<br>- **Quality and Patient Satisfaction Surveys:** Suggesting that managed care plans survey enrollees and adjust based on feedback aligns with a liberal emphasis on patient-centered care and responsiveness to individual needs.<br>- **Regulatory Measures for Quality:** Recommending proficiency testing and accreditation for labs may resonate with liberals who often support regulatory measures to ensure quality and safety in healthcare.<br><br>2. **Conservative Viewpoint Tendencies:**<br>- **Cost-Effectiveness and Competition:** The suggestion to compare costs and allow for competitive rates may appeal to conservative values emphasizing market-driven solutions and cost containment in healthcare.<br>- **Financial Risk for Physicians:** Proposing that physicians be placed at financial risk for lab tests to address over-utilization could align with conservative preferences for personal responsibility and limiting unnecessary healthcare spending.<br><br>3. **Neutral/Pragmatic Aspects:**<br>- The text also contains elements that are fairly neutral or pragmatic, focusing on efficiency, quality, and appropriate access, which are concerns common to both sides.<br><br>It's important to note that healthcare policy is complex, and individuals or groups within the liberal or conservative spectrum might have varying opinions on these issues. Some conservatives might support patient access improvements, and some liberals might agree with cost-effective measures. The alignment with a particular viewpoint can also depend on how these suggestions are implemented and the broader context of the healthcare system they are a part of.	neutral

ACP	Statement on Arbitrary Classifications that Restrict the Practice of Internal Medicine	2022	The text provided outlines a position on physician practice and healthcare delivery by the American College of Physicians (ACP). It focuses on advocating for physician autonomy and competence, opposing restrictions imposed by specialty designations and insurance requirements. This stance doesn't neatly align with traditional liberal or conservative viewpoints as it centers on professional standards and healthcare policy rather than broader political ideologies.<br><br>Liberal perspectives typically emphasize access to healthcare and may support reducing barriers for physicians to offer a wide range of services. Conservatives might focus on reducing regulatory oversight and supporting professional autonomy, which aligns with the text's opposition to external restrictions.<br><br>However, both sides might find aspects to agree with or contest based on their broader healthcare policies and beliefs about regulation, professional self-governance, and patient access. The statement is professional and policy-oriented, making it less about partisan politics and more about healthcare delivery and physician practice.	neutral
ACP	Use of Board Certification	2015	The statement about board certification not being used to exclude or include physicians from participation in healthcare plans, employment opportunities, or hospital privileges doesn't neatly align with a liberal or conservative viewpoint. It's more focused on the professional standards and policies within the medical field, specifically about the criteria for physician qualifications and employment.<br><br>From a liberal perspective, the emphasis might be on ensuring equal opportunities and preventing discrimination based on certification status, thereby promoting a more inclusive healthcare system. Liberals might support this stance as it aligns with broader goals of equal access and fairness in employment.<br><br>Conservatives might focus on the freedom of healthcare organizations to set their own standards and the importance of maintaining high professional standards, possibly arguing that board certification is a valid measure of competence and quality. They might see this as a way to ensure that patients receive care from highly qualified professionals.<br><br>However, both viewpoints could conceivably agree or disagree with the statement for different reasons. For example, both might agree on the importance of high standards in healthcare but disagree on how to achieve it. It's important to recognize that positions on specific policy issues like this can vary within each ideological group and can be influenced by broader values, the specific context of the discussion, and other policy goals.	neutral
ACP	Managed Behavioral Health Organizations (MBHOs)	2022	The text you've provided discusses the sharing of disease management protocols between Managed Behavioral Health Organizations (MBHOs) and primary care physicians, emphasizing coordination and optimal patient care. This text doesn't align strictly with a liberal or conservative viewpoint; instead, it focuses on healthcare management and patient care coordination.<br><br>1. **Liberal Viewpoint**: Liberals typically advocate for comprehensive healthcare access and may support integrated care models that emphasize coordination between different healthcare providers to ensure patients receive holistic care. This view might support the idea of MBHOs sharing information with primary care physicians as it could lead to more comprehensive, patient-centered care.<br><br>2. **Conservative Viewpoint**: Conservatives might focus on the efficiency, privacy, and autonomy of healthcare providers. They may support the idea if it's seen as a way to improve care efficiency and reduce costs without unnecessary government intervention. However, they might also have concerns about privacy and the regulatory aspects of mandating such information sharing.<br><br>In conclusion, the text touches on aspects that both liberals and conservatives might find agreeable or concerning, depending on their perspectives on healthcare management, patient privacy, and the role of government in regulating healthcare practices. The emphasis here is on improving healthcare outcomes through collaboration, which is a goal that can transcend political boundaries.	neutral

ACP	Monitoring	2019	The statement provided seems to align with a viewpoint that is concerned with the structured, responsible transformation of Medicaid into state-managed care plans, emphasizing access, quality, and financial stability. It doesn't strictly align with liberal or conservative viewpoints as traditionally defined; rather, it focuses on practical and administrative aspects of healthcare policy.<br><br>Liberal perspectives generally support comprehensive, government-run or regulated healthcare to ensure access for all, often favoring expansion of programs like Medicaid. They might advocate for strong oversight and standards in the transformation of healthcare systems to protect underserved populations.<br><br>Conservative perspectives typically favor smaller government involvement, market-driven solutions, and state-level autonomy over healthcare. They might support the idea of states having the flexibility to manage Medicaid but also seek to ensure that such programs are financially sustainable and not overly burdensome on state budgets.<br><br>This statement, with its focus on uniform criteria, phased implementation, sound financial underpinnings, and standards of quality, seems to blend concerns that might be shared by both sides: ensuring effective, quality care (a more liberal concern) and emphasizing financial soundness and structured implementation (a more conservative concern). It suggests a careful, measured approach to policy change, likely intended to appeal to a broad range of stakeholders by addressing key concerns of access, quality, and sustainability.	neutral
ACP	Medicaid Waivers for Managed Care Demonstration Projects	2015	The text provided reflects a stance on Medicaid Section 1115 waivers, which are used for demonstration projects that experiment with new ways of providing and paying for healthcare services. The viewpoint expressed here doesn't align strictly with traditional liberal or conservative perspectives but contains elements that could be interpreted differently:<br><br>1. **Liberal Viewpoint:**<br>- **Promoting Access to Quality Medical Care:** Generally, liberals support expanding access to healthcare and improving quality. The emphasis on promoting Medicaid's objective to improve access might resonate with liberal ideals about universal healthcare access.<br>- **Flexibility and Sufficient Funding:** Liberals often advocate for adequate funding in social programs and may support flexible, long-term planning to ensure these programs' effectiveness and stability.<br><br>2. **Conservative Viewpoint:**<br>- **Support for Section 1115 Waivers:** Conservatives typically favor state-level innovation and flexibility over federal mandates. Section 1115 waivers allow states to experiment and potentially find more efficient, locally-tailored solutions to healthcare, which aligns with conservative preferences for smaller federal government and more state autonomy.<br>- **Concerns Over Long-term Planning and Predictability:** Fiscal responsibility and predictability in funding are often conservative concerns. Urging for renewal requirements that allow for long-range planning might align with conservative views on efficient and predictable government spending.<br><br>Overall, the statement supports using Section 1115 waivers under certain conditions and with an emphasis on flexibility and sufficient funding. It straddles both liberal and conservative viewpoints, aligning with liberal ideals about expanding access and improving healthcare and conservative principles of state autonomy and fiscal predictability. It's a nuanced position that emphasizes effective implementation of healthcare policy rather than adhering strictly to an ideological spectrum.	neutral

ACP	State Medicaid Managed Care Programs	2019	The text primarily outlines policy recommendations for state governments managing Medicaid and seems to focus on ensuring quality, accountability, and fairness in managed care programs. It doesn't align strictly with a liberal or conservative viewpoint as it blends various elements that could be appealing to both sides. For example:<br><br>1. **Liberal Viewpoints**:<br>- Emphasis on regulation and oversight (e.g., establishing grievance systems, imposing fines for non-compliance, requiring background checks).<br>- Focus on consumer protection and education (e.g., outreach programs, prohibiting certain marketing practices).<br>- Ensuring access and fairness in healthcare (e.g., rules against considering health status in enrollment).<br><br>2. **Conservative Viewpoints**:<br>- Emphasis on state authority and responsibility over federal (e.g., states should demonstrate organizational capacity, have the authority to impose fines).<br>- Promoting efficient management and accountability (e.g., requiring a certain percentage of payments to be spent on health care services, reporting on executive salaries).<br>- Encouraging private sector involvement and standards (e.g., independent enrollment brokers, applying similar standards to Medicaid and commercial plans).<br><br>The recommendations aim to improve Medicaid managed care's efficiency and effectiveness, a goal that both liberals and conservatives might support, albeit for different reasons. Liberals might focus on the aspects that promote equitable access and protection for beneficiaries, while conservatives might appreciate the emphasis on state authority, market mechanisms, and fiscal accountability. The text is a blend of regulatory and market-oriented approaches, reflecting a pragmatic focus on improving healthcare delivery within the Medicaid system rather than a strict ideological stance.	neutral
ACP	Physician Contacts with Medicare-HMO Intermediaries	2022	The text from the American College of Physicians (ACP) is policy-oriented and focuses on the ethics and regulations surrounding physician contracts with Medicare Advantage. Specifically, it opposes clauses that restrict physicians from billing other insurance carriers. This stance doesn't neatly align with traditional liberal or conservative viewpoints, as it touches on healthcare policy, business practices, and physician autonomy—all areas with complex and varied perspectives within each political ideology.<br><br>Liberals might support this stance as it advocates for physician autonomy and could potentially lead to broader access to healthcare services. It also pushes back against private insurance practices that might limit patient choices or physician decision-making. Conservatives might appreciate the emphasis on individual freedom and market competition, as it argues against restrictive contracts and could support more business opportunities for various insurance providers.<br><br>However, both sides might have concerns as well: liberals about the potential complexities and costs in a multi-payer system and conservatives about potential regulatory implications. In essence, the statement is more professional and pragmatic than political, aiming to navigate the complex healthcare landscape rather than championing a strictly liberal or conservative cause.	neutral
ACP	Disclosure of Information to Beneficiaries/Enrollees	2022	The text provided outlines detailed requirements and policies for managed care plans, focusing on transparency, beneficiary rights, and access to information. It doesn't explicitly align with either a liberal or conservative viewpoint as it combines elements that can be associated with both ideologies.<br><br>On one hand, the emphasis on detailed disclosures, patient rights, and access to information about treatment options and plan specifics could align with liberal ideologies, which often prioritize consumer protections, comprehensive healthcare access, and transparency in services. The focus on ensuring that patients understand their options, the push for quality and satisfaction metrics, and the requirements for reporting and accountability might resonate with liberal priorities of patient empowerment and informed choice in healthcare.<br><br>On the other hand, the text also emphasizes informed consumer choice, competition among plans, and the disclosure of financial aspects like medical expense ratios and physician incentive plans. These elements might align with conservative viewpoints, which often favor market-based solutions to healthcare, informed consumers making choices in a competitive environment, and transparency as a means to drive efficiency and quality in healthcare.<br><br>Overall, while some aspects of the text might resonate more with one ideology than the other, the text as a whole seems to aim for a comprehensive approach to managed care that incorporates transparency, accountability, and patient empowerment, aspects that are valued across the political spectrum. It's important to note that in the complex field of healthcare policy, many proposals contain	neutral

			elements that can be supported by multiple political perspectives, depending on the specific context and how they are implemented.	
ACP	Choice of Physicians in Medicare Managed Care Plans	2022	The text you've provided outlines a policy proposal for Medicare Managed Care plans, focusing on ensuring patient choice, network flexibility, and cost-sharing considerations. It doesn't explicitly align with a single political viewpoint as elements of it could be supported by both liberals and conservatives, but certain aspects may resonate more with one side:<br><br>1. **Liberal Viewpoint Tendencies:**<br>- The emphasis on patient choice and access to a broad range of physicians is consistent with liberal priorities of comprehensive healthcare access.<br>- The call for a Point-of-Service (POS) option and regulated cost-sharing, especially for lower-income enrollees, aligns with liberal values of social equity and support for the economically disadvantaged.<br>- Liberals might favor the government's role in setting cost-sharing limits and ensuring that healthcare is affordable for lower-income individuals.<br><br>2. **Conservative Viewpoint Tendencies:**<br>- The inclusion of a supplemental premium for additional services (POS rider) might be seen as supportive of market-based solutions and personal responsibility, aligning with conservative principles.<br>- Conservatives might appreciate the aspect of choice and the ability to opt into a wider network if the enrollee is willing to pay more, reflecting a preference for personal freedom and less restrictive networks.<br><br>Ultimately, the text seems to be attempting a balanced approach, trying to blend access and choice (often more liberal aims) with market-based solutions and individual responsibility (often more conservative aims). The real-world alignment would depend on the broader context of the healthcare debate at the time and how various groups perceive the details of these proposals.	neutral
ACP	Provision of Care to Enrollees with Chronic Conditions and Special Needs	2022	The text you've provided outlines proposals for enhancing accessibility and quality of care in Medicare Managed Care plans, with a focus on ensuring services for beneficiaries, especially those with chronic conditions and special needs. This doesn't neatly align with traditional liberal or conservative viewpoints as both sides of the political spectrum might have varied opinions on the specific measures and their implementation.<br><br>From a **liberal perspective**, there might be support for the emphasis on accessibility, quality of care, and protections for those with special health care needs. Liberals typically advocate for robust healthcare protections and might favor the comprehensive approach to ensuring that needs are met, particularly for vulnerable populations.<br><br>From a **conservative viewpoint**, while there might be support for improving efficiency and ensuring quality of care, there could be concerns about the potential for increased regulation and mandates on private plans. Conservatives often prioritize market-based solutions and might prefer less prescriptive methods for achieving these goals.<br><br>In general, the text reflects a policy-oriented, pragmatic approach to healthcare management, focusing on outcomes and accessibility rather than ideologically driven solutions. It's essential to consider that within both political spectrums, there's a range of opinions, and different individuals or groups might have their interpretations or preferred approaches to the issues addressed in the text.	neutral

ACP	Enrollees’ Access to Urgent and Emergency Care Services	2022	The text provided outlines policies for Medicare Managed Care plans to ensure beneficiaries have immediate access to urgent and emergency care. This approach aligns with a generally liberal viewpoint, which often emphasizes the importance of accessible healthcare and patient rights. The focus on using a prudent layperson's assessment for emergencies and ensuring timely decisions on preauthorization reflects a patient-centered approach, advocating for protections and access to care regardless of the final diagnosis, which is more aligned with liberal healthcare policies. Conservative viewpoints might focus more on cost containment, personal responsibility, and less government involvement in healthcare, potentially advocating for stricter criteria for emergency care coverage to prevent misuse and control costs. However, it's important to note that healthcare policies can vary widely within each political spectrum, and there are conservative perspectives that also value access to emergency care and patient protections.	liberal
ACP	Enrollees' Grievance and Appeals Rights and Procedures	2019	The text outlines specific criteria for appeals and grievances in Medicare Managed Care plans. This text does not explicitly align with a traditional liberal or conservative viewpoint, as it focuses on policy specifics rather than broader ideological principles. However, its emphasis on regulation, consumer protection, and accountability could be interpreted as aligning with typical liberal values, which often prioritize government oversight and protection of individual rights in healthcare. On the other hand, the emphasis on efficiency, transparency, and specific standards could also be appealing to some conservative perspectives that value streamlined government intervention and accountability. Ultimately, the text combines elements that could be supported by a range of political perspectives, focusing on practical improvements and protections for enrollees rather than ideological doctrine.	neutral
ACP	Handling of Reconsidered Appeals Determinations	2019	The text you've provided outlines procedural guidelines and regulations for handling cases in a Medicare managed care context, specifically concerning reconsiderations and hearings. It doesn't explicitly advocate for a liberal or conservative viewpoint but rather focuses on administrative procedures and rights for enrollees and physicians.<br><br>However, the underlying principles can be seen as aligning with broader ideological perspectives:<br><br>1. **Liberal Viewpoint**: Liberals generally advocate for strong consumer protections and a robust social safety net. The detailed process for reconsideration and the rights granted to enrollees and physicians can be seen as aligning with this viewpoint, as it emphasizes transparency, due process, and protections for individuals in the healthcare system.<br><br>2. **Conservative Viewpoint**: Conservatives typically emphasize efficient government operations and may support clear rules and regulations that prevent unnecessary bureaucracy and ensure quick resolution of issues. The specific time frames and the finality of decisions might appeal to conservative desires for efficiency and finality in government operations.<br><br>In essence, while the text is procedural and doesn't overtly side with a political ideology, aspects of it can be interpreted to align with values from both liberal and conservative perspectives, depending on how one emphasizes the importance of consumer protections versus efficient administration.	neutral
ACP	Quality Improvement Organizations (QIOs) Review of Disputed Inpatient Lengths of Stay	2019	The text you've provided outlines policies related to Quality Improvement Organizations (QIOs) and their role in reviewing disputes over the length of inpatient stays in Medicare. This topic intersects with broader debates about healthcare management, patient rights, and the role of government in health care.<br><br>From a **liberal viewpoint**, the emphasis might be on maintaining robust patient protections and ensuring that individuals have the right to appeal decisions made by their managed care plans. Liberals generally support strong oversight and regulation to ensure that patients receive the care they need and are not subject to premature discharge due to cost-cutting measures by insurance companies. The policy outlined could be seen as a way to protect Medicare enrollees from potentially harmful decisions made by managed care plans and to ensure a fair review process.<br><br>From a **conservative viewpoint**, there might be concerns about the efficiency and bureaucracy of such processes. Conservatives typically advocate for less government intervention and more market-based solutions. They might argue that while patient protection is important, the immediate review requirement could lead to unnecessary bureaucracy and hinder the efficiency of managed care plans. They might prefer solutions that increase competition and choice in the market, potentially leading to better care and more efficient dispute resolution.<br><br>However, it's important to note that healthcare is a complex issue, and individuals within each ideological group might have varying opinions on this specific policy. Some conservatives might support strong patient protections, while some liberals might have concerns about the efficiency of the process. The perspectives provided are generalizations and might not represent all individuals within each ideological group.	neutral

ACP	Standards for CMS Appeals Contractors	2019	The text outlines proposed standards and processes for contractors reviewing appeals and grievances in Medicare managed care plans. It doesn't explicitly align with a liberal or conservative viewpoint as it focuses on procedural and operational improvements rather than broader ideological principles. However, elements could be interpreted through different ideological lenses:<br><br>1. **Liberal Viewpoint:**<br>- **Emphasis on Transparency and Accountability:** Requiring contractors to disclose decision-making criteria and involve advisory committees may align with liberal values of transparency and fairness in healthcare.<br>- **Inclusive Decision-Making:** Involving a diverse range of medical professionals and requiring reports to medical associations could be seen as promoting a more inclusive and comprehensive healthcare system.<br><br>2. **Conservative Viewpoint:**<br>- **Efficiency and Quality:** The focus on performance standards and timely notifications may resonate with conservative values of efficiency and high-quality service in the private sector.<br>- **Local and State Involvement:** The requirement for state or regional advisory committees might appeal to conservatives preferring local or state-level governance over federal control.<br><br>Overall, the text aims to improve the quality and fairness of Medicare managed care reviews. It might find support across the political spectrum for its potential to enhance healthcare services' efficiency, transparency, and responsiveness. However, interpretations can vary based on individual or partisan perspectives on government involvement and private sector regulation in healthcare.	neutral
ACP	Utilization Review (UR) Requirements for Medicare managed care plans	2019	The text outlines specific recommendations for Medicare Managed care plans to ensure transparency, accountability, evidence-based practices, and ethical standards. It doesn't align strictly with a liberal or conservative viewpoint as both sides of the political spectrum could find aspects to support or critique:<br><br>1. **Liberal Viewpoint**: Liberals might favor these suggestions because they emphasize transparency, accountability, and patient protection. The focus on evidence-based criteria and the prohibition of compensation based on denial rates aligns with liberal values of fairness and evidence-based policy. The requirement for licensed professionals in review determinations and the call for timely access to review personnel also reflect a liberal emphasis on quality healthcare and patient rights.<br><br>2. **Conservative Viewpoint**: Conservatives might appreciate the emphasis on sound scientific principles and the most recent medical evidence as a basis for utilization review (UR) criteria. This can be seen as a way to ensure efficient and effective use of resources, which aligns with conservative values of efficiency and fiscal responsibility. They might also support the idea of making the process more transparent and accountable as a means of reducing unnecessary bureaucratic interference and promoting market efficiencies.<br><br>Both viewpoints might have concerns as well. Liberals might worry that despite these recommendations, the actual implementation could still prioritize cost savings over patient care. Conservatives might be concerned about the potential for increased regulation and oversight leading to higher costs and reduced flexibility for managed care plans.<br><br>Ultimately, the text proposes a balanced approach aimed at improving the quality and accountability of Medicare Managed care plans, and while it has elements that might appeal to both liberal and conservative perspectives, it doesn't squarely fit into one ideological category. It represents a nuanced approach to healthcare policy that emphasizes transparency, evidence, and patient care, areas that can, in some contexts, find bipartisan support.	neutral
ACP	Physician Reimbursement, Financial Incentives, Risk-Sharing, and Avoidance of Adverse Selection	2019	The text provided outlines policy recommendations and regulatory changes for managing Medicare and Medicaid payments and risk within managed care plans. It doesn't align strictly with a liberal or conservative viewpoint as it deals with the technical and administrative aspects of healthcare management, which can be advocated for by members of either political spectrum. However, the focus on adjusting payments to reflect patient risk, requiring methodologies to prevent discrimination against sicker patients, and providing adequate stop-loss coverage might be more closely associated with a liberal viewpoint as it emphasizes regulation, consumer protection, and equity in healthcare access. Conservatives might support aspects of these proposals that promote efficiency and proper allocation of resources but might be less inclined towards increased regulation. Ultimately, the text is more about pragmatic healthcare management than ideological positioning.	neutral

ACP	Medicare Risk Contracting	2019	The statements from the American College of Physicians (ACP) regarding improvements in the Medicare risk contracting program don't align squarely with traditional liberal or conservative viewpoints. They represent a mix of ideas that could appeal to different sides for various reasons. Here's a breakdown of how each point might be perceived:<br><br>1. **Revising the method of designating payment in Medicare risk contracts**: This could be seen as a technocratic approach to healthcare reform, appealing to both sides. Conservatives might appreciate the focus on efficiency and cost-effectiveness, while liberals might support efforts to ensure fair compensation for healthcare providers.<br><br>2. **Use of risk adjustments such as history of serious illnesses in setting payments to risk contracting plans**: This is a method to make pricing fairer and more reflective of actual needs, which could garner support from liberals for its focus on equity. However, conservatives might also see it as a way to make the system more market-efficient and financially sustainable.<br><br>3. **Requiring that beneficiaries be provided comparative information about all health plan choices available to them**: This promotes transparency and informed decision-making, which is a principle that can resonate with both ideologies. Liberals might see it as empowering consumers, while conservatives might appreciate the market-based approach to making informed choices.<br><br>4. **Requiring that beneficiaries stay with a health plan until the next annual enrollment period**: This might be seen as limiting consumer freedom, which could be opposed by liberals, but conservatives might support it for its potential to bring stability and predictability to the insurance market.<br><br>5. **Requiring reasonable, non-punitive increases in premiums and other cost-sharing for beneficiaries who choose to remain in the traditional Medicare fee-for-service system**: Conservatives might favor this as it encourages personal responsibility and could potentially reduce government spending. Liberals, however, might be concerned about the impact on low-income beneficiaries.<br><br>6. **Requiring that beneficiaries be provided comparative information concerning all Medicare risk contracting plans that are available to them**: Similar to point 3, this is about ensuring informed choices and could be supported by both sides for promoting transparency and consumer empowerment.<br><br>Overall, the ACP's statements reflect a nuanced approach to healthcare reform, incorporating elements of market efficiency, consumer choice, and equitable access. These ideas transcend simple liberal/conservative dichotomies and represent a blend of principles that might find support across the political spectrum.	neutral
ACP	Assessing Physician Performance in a Medicare Managed Care Plan Setting	2008	The text provided outlines principles for managed care plans in the context of Medicare, focusing on transparency, provider involvement, objective standards, and protection of physicians' rights. This approach doesn't align strictly with liberal or conservative viewpoints as traditionally defined, but instead touches on aspects that might resonate with both sides.<br><br>From a liberal perspective, the emphasis on quality, access, and involvement of healthcare professionals in decision-making aligns with a focus on individual rights and protections, as well as a collective responsibility to ensure quality healthcare. Liberals might appreciate the emphasis on transparency and the measures to protect against arbitrary actions by managed care plans.<br><br>From a conservative viewpoint, the insistence on objective standards and economic considerations could be seen as aligning with principles of efficiency and market-driven solutions. The focus on not compromising access and quality might also resonate with a conservative emphasis on personal responsibility and maintaining high standards in healthcare.<br><br>Overall, the text seems to strive for a balanced approach that incorporates accountability, quality, and fairness within the managed care system. It does not advocate for a specific political ideology but rather focuses on practical, ethical, and efficient management of healthcare services.	neutral

ACP	Medicare Managed Care Plan Reimbursement for Medical Education, Training, and Research	2019	The statement "Medicare payments to capitated medical plans should accurately reflect expenses for medical education, training, and research" seems to primarily focus on ensuring that Medicare payments are reflective and supportive of the broader costs associated with providing medical education and maintaining a skilled physician workforce. This is a policy-focused statement and doesn't explicitly align with a liberal or conservative viewpoint as both sides might agree on the principle of accurate and fair compensation for healthcare services, including the indirect costs of education and training.<br><br>However, the underlying values and solutions proposed to address this issue could be interpreted differently by liberal and conservative perspectives:<br><br>1. **Liberal Viewpoint**: Might emphasize the importance of comprehensive support for medical education and training as part of a broader commitment to accessible and high-quality healthcare for all. Liberals might argue for increased government spending or regulation to ensure that these educational costs are covered adequately.<br><br>2. **Conservative Viewpoint**: Might focus on market-based solutions and personal responsibility. Conservatives might argue that while accurate reflection of expenses is important, it's also crucial to keep government spending under control and ensure that the healthcare market operates efficiently without excessive regulation.<br><br>It's essential to recognize that these are generalizations and that there's a wide spectrum of beliefs within each political ideology. Additionally, healthcare policy is complex, and individuals might have nuanced views that don't fit neatly into a liberal or conservative box. Ultimately, the alignment of this statement with a particular viewpoint might depend on the broader context of the proposed policy and the values and priorities of the individual interpreting it.	neutral
ACP	Dual Eligibles	2016	The text you've provided outlines positions from the American College of Physicians (ACP) regarding various aspects of Medicare and Medicaid, particularly focusing on the Medicare Modernization Act, Medicare Part D, and issues affecting Qualified Medicare Beneficiaries (QMBs). These positions reflect concerns about the affordability and accessibility of prescription drugs for low-income individuals and suggest adjustments to reduce financial burdens and improve clarity and assistance in the healthcare system.<br><br>These viewpoints tend to align more closely with liberal perspectives, which typically emphasize the need for government intervention and support in healthcare to ensure that it is accessible and affordable for all, especially the underserved and economically disadvantaged populations. The focus on revising "clawback" provisions, advocating for clear communication from prescription drug plans, revising appeal thresholds, and adjusting co-payments to be more affordable are all indicative of a desire to strengthen and expand the safety net for vulnerable populations, a common theme in liberal healthcare policies.<br><br>However, it's important to note that healthcare policy can transcend traditional political boundaries, and there are conservatives who may also support certain reforms to improve efficiency and care outcomes in the system. The specifics of policy proposals and the reasoning behind them can vary widely within both liberal and conservative circles.	liberal

ACP	Medicaid and Health Reform	2022	The text provided outlines a comprehensive approach to Medicaid reform, with a focus on expanding coverage, improving access and quality of care, and ensuring financial sustainability. This perspective aligns more closely with a liberal viewpoint for several reasons:<br><br>1. **Expansion of Coverage and Uniform Eligibility:** The call for Medicaid to serve as a foundational source of coverage for all low-income individuals, with uniform national eligibility standards and federal subsidization, is typically aligned with liberal ideologies. Conservatives might favor more state control and less federal involvement.<br><br>2. **Adequate Payment Rates:** Advocating for increased payment rates to ensure provider participation and access to services is often associated with liberal perspectives that prioritize access to healthcare and equitable compensation for providers.<br><br>3. **Emphasis on Evidence-Based Care and Fraud Reduction:** While both liberals and conservatives are concerned with efficiency and reducing fraud, the emphasis on ensuring these efforts do not burden legal providers might be more aligned with a liberal viewpoint that often stresses the protection of rights and fair treatment.<br><br>4. **Flexibility and Choice in Long-Term Care:** The suggestion to offer more flexible long-term care options aligns with a liberal preference for individual choice and comprehensive care options.<br><br>5. **Consumer-Driven Reforms and Comprehensive Benefits:** Cautioning against consumer-driven reforms that might harm vulnerable populations and advocating for a core set of comprehensive benefits is typically associated with a liberal stance that prioritizes protection and comprehensive coverage for all, especially the disadvantaged.<br><br>6. **Collaboration and Streamlining Processes:** The call for federal and state collaboration and streamlined processes may be endorsed by both viewpoints, but the context here is toward expanding and improving access, which is more often a liberal objective.<br><br>7. **Accountability and Quality Improvement:** Focusing on quality of care, reducing disparities, and managing chronic diseases aligns with liberal priorities of equitable, high-quality healthcare for all.<br><br>8. **Counter-Cyclical Funding Mechanism:** Proposing a funding mechanism similar to unemployment insurance to increase federal dollars during economic downturns reflects a liberal approach to providing a safety net and stabilizing funding.<br><br>9. **Reduced Barriers to Enrollment and Outreach:** Efforts to ease enrollment and provide culturally competent outreach are typically liberal strategies aimed at increasing access and equity.<br><br>10. **Improving Physician and Patient Experience:** While both ideologies might support this, the specific mention of financial assistance for health technology and reducing administrative barriers may more closely align with liberal priorities of supporting providers and improving care quality.<br><br>11. **Innovative Delivery System Reforms:** Advocating for reforms like the patient-centered medical home reflects a liberal preference for innovative, coordinated, and comprehensive care models.<br><br>12. **Alternative Financing Structures:** Considering alternative financing to ensure solvency and high-quality care while alleviating financial pressure on states suggests a liberal approach to reforming and sustaining Medicaid in a way that maintains or expands its services.<br><br>Overall, the text emphasizes expanding access, ensuring quality and comprehensive coverage, and maintaining robust federal support and oversight—principles more commonly associated with liberal healthcare policies. Conservative viewpoints might differ by emphasizing state autonomy, market-driven solutions, and reduced federal spending and involvement.	liberal

ACP	Medicaid Standards for State Waivers	2017	The text presents viewpoints that align with principles often associated with both liberal and conservative ideologies, making it not strictly one or the other.<br><br>Liberal alignment is seen in the emphasis on standards, oversight, and public involvement to ensure quality care and protection of Medicaid beneficiaries. The call for meaningful public comments, thorough compliance, and uniform quality of care standards reflects a liberal inclination toward regulatory oversight and ensuring equitable access to healthcare services. Additionally, the insistence on revealing utilization review criteria to physicians and patients, and involving physicians in review decisions, aligns with liberal values of transparency and patient rights.<br><br>Conservative alignment can be discerned in the focus on managed care as a means to improve quality and reduce costs. The idea of managed care, capitated payments, and actuarial analysis are often embraced by conservatives as methods to increase efficiency and reduce government spending in healthcare. The focus on sound financial underpinnings before waiver approval also aligns with conservative principles of fiscal responsibility and ensuring that government programs are financially sustainable.<br><br>The text represents a balanced approach, advocating for reforms that increase efficiency and cost-effectiveness while ensuring quality and oversight, reflecting aspects of both liberal and conservative viewpoints.	neutral
ACP	Medicaid Expansion: Premium Assistance and Other Options	2016	The text presents a viewpoint that aligns more closely with liberal policy perspectives. Here's a breakdown of why:<br><br>1. **Emphasis on Information Accessibility and Assistance**: The call for clear dissemination of information, assistance for those with disabilities, and support for non-English speakers typically aligns with liberal priorities, which often focus on inclusivity and ensuring equal access to resources.<br><br>2. **Expansion of Coverage**: The insistence on providing a comprehensive health benefit package, including mental health parity and other benefits, reflects a liberal stance. Liberals generally advocate for broader healthcare coverage and access to a wide range of services.<br><br>3. **Cost Structure to Encourage Enrollment**: Arguing against high premiums and cost-sharing that could discourage enrollment or cause individuals to forgo care aligns with a liberal viewpoint, which often emphasizes the importance of making healthcare accessible and affordable to all, especially the low-income population.<br><br>4. **Opposition to Work Requirements**: The stance against work-related conditions for Medicaid eligibility is a distinctly liberal position. Conservatives often argue for such requirements as a way to promote self-sufficiency, while liberals argue that healthcare should be a right, not contingent upon employment status.<br><br>5. **Wellness Programs and Incentives**: The emphasis on positive incentives rather than punitive measures for health improvement and the specific reference to ethical considerations in patient incentives align with liberal views on healthcare, focusing on support and empowerment rather than penalties.<br><br>Overall, the text advocates for expansive, accessible, and supportive Medicaid programs without imposing strict or punitive measures on enrollees. These principles are more commonly associated with liberal ideologies, which prioritize social support systems and government intervention to ensure equitable access to healthcare.	liberal
ACP	Fellowship Start Date	2016	The text you've provided doesn't inherently align with a liberal or conservative viewpoint. It's a policy position from the American College of Physicians regarding the scheduling between residency completion and fellowship initiation. This seems to be more of an administrative or organizational stance rather than one based on political ideology. Both liberal and conservative individuals in the medical field might have various reasons to agree or disagree with this policy based on practical, educational, or institutional considerations rather than ideological ones.	neutral

ACP	United States Medical Licensure Exam Step II Clinical Skills Exam and the Comprehensive Osteopathic Medical Licensure Exam Part II Clinical Skills Exam	2016	The text you've provided doesn't inherently align with a specific liberal or conservative viewpoint. It's a policy recommendation from the American College of Physicians (ACP) regarding the adjustment of student financial aid budgets to cover the costs of licensure exams for medical students. This statement is primarily focused on the practical and financial aspects of medical education rather than expressing an ideological stance.<br><br>Liberal and conservative views typically differ on issues like the role of government, social policies, and economic regulations. While conservatives might emphasize individual responsibility and minimal government intervention in education and healthcare, liberals might advocate for increased government support and funding to ensure access and equity. However, this particular statement is more about educational and professional standards within the medical field and does not directly address broader ideological debates. It seems to aim for a pragmatic approach to financial aid for students facing high costs for required exams, which could be supported by people across the political spectrum who are concerned with the accessibility and affordability of medical education.	neutral
ACP	Geriatrics	2016	The text from the American College of Physicians (ACP) regarding the treatment of the elderly and the inclusion of geriatrics and clinical gerontology in internal medicine doesn't align distinctly with either a liberal or conservative viewpoint. It focuses on medical education, patient care, and the recognition of geriatrics as a crucial aspect of internal medicine. Both liberal and conservative ideologies generally support the advancement of medical care and education, albeit sometimes with different approaches to healthcare policy. This statement is more about professional standards and medical education than it is about political ideology.	neutral
ACP	Clinical Faculty of Medical Schools	2016	The text you've provided is a policy statement from the American College of Physicians (ACP) encouraging the involvement of non-salaried clinical faculty in educational and research programs, as well as curriculum development and administrative policies. It doesn't inherently align with a liberal or conservative viewpoint as it's primarily focused on educational and institutional policy rather than political ideology. Both liberal and conservative individuals or groups might support such measures for various reasons, such as improving medical education, fostering collaboration, or enhancing the quality of healthcare. The emphasis is on organizational governance and academic involvement rather than political or ideological issues.	neutral
ACP	Elimination of Federal Financial Assistance to Those Attending Unaccredited Medical Schools	2019	The statement about the American College of Physicians (ACP) supporting the elimination of federal financial assistance to U.S. students attending unaccredited medical schools doesn't neatly align with traditional liberal or conservative viewpoints as defined in American politics. Here's a brief analysis of how both sides might view it:<br><br>**Liberal viewpoint:**<br>- Liberals generally favor educational opportunities and support for students, but they also emphasize the importance of quality education and the protection of students from potentially predatory institutions. In this context, they might support the elimination of federal aid to unaccredited schools to ensure students attend institutions that meet certain quality standards.<br>- They may view this policy as a way to protect students from debt and poor educational outcomes.<br><br>**Conservative viewpoint:**<br>- Conservatives typically advocate for reduced government spending and might support the elimination of financial aid for unaccredited schools as a way to cut costs and prevent wasteful spending on institutions that don't meet certain standards.<br>- They might also argue for the importance of maintaining high educational standards and see this policy as a way to uphold these standards.<br><br>Both viewpoints could potentially agree on the elimination of aid if the rationale aligns with their broader principles, such as protecting students and ensuring the quality of education (a liberal perspective) or reducing unnecessary government spending and maintaining high standards (a conservative perspective). However, the specific stance would depend on the broader context of the policy and the priorities of the individuals or groups interpreting it.	neutral

ACP	Fair Contribution by Payers for Medical Education, Research and Indigent Care	2019	The text you've provided outlines a position from the American College of Physicians (ACP) that supports an all-payer approach to subsidize medical education, postgraduate training, and clinical research, as well as alternatives for subsidizing indigent care. This position does not align neatly with traditional liberal or conservative viewpoints as understood in a broad political context. Instead, it reflects specific policy preferences in the healthcare sector.<br><br>1. **All-Payer Approach to Medical Funding**: This approach suggests a system where all payers (government, private insurers, etc.) pay similar rates for medical services. This can be seen as a middle ground between a single-payer system (often associated with liberal viewpoints) and a market-driven approach (often associated with conservative viewpoints). The focus on equitable funding for medical education and research may be more aligned with liberal perspectives that emphasize equal access and support for public goods.<br><br>2. **Support for Subsidizing Indigent Care**: Providing financial support for indigent (low-income) care is a policy often supported by liberals who advocate for social safety nets and healthcare access for all, regardless of income. However, the text does not specify the method of subsidizing, which could range from government programs (more liberal) to private charity or market-based solutions (more conservative).<br><br>In summary, the ACP's position seems to blend elements that could be supported by both liberal and conservative viewpoints, focusing specifically on pragmatic solutions in the healthcare field rather than fitting neatly into a broad political ideology.	neutral
ACP	Revitalization of Internal Medicine: Overview of the Problem and Recommendations on Reducing Medical Student Debt	2023	The text you've provided outlines policies and viewpoints related to financing medical education, particularly in the field of primary care. These points do not align strictly with either a liberal or conservative viewpoint, as they encompass aspects that could be endorsed by individuals across the political spectrum. However, they do have certain tendencies that might be more closely associated with one viewpoint over the other:<br><br>1. **Advocating for Increased Financing and Improvements in Primary Care Service**: This point, focusing on enhancing the accessibility and effectiveness of primary care service through financial aid, could be seen as aligning more with liberal viewpoints. Liberals often advocate for increased funding in public health sectors and education, emphasizing the role of government in providing support and resources.<br><br>2. **Expanded Funding for Federal Loan Programs**: The call for expanded funding and eligibility for federal loan programs, especially those supporting primary care, again leans towards a liberal perspective. This is because it involves government intervention in education financing and supports the idea of making higher education more accessible, a principle often associated with liberal politics.<br><br>3. **Financial Aid, Debt Counseling, and Publicizing Opportunities**: Offering comprehensive financial aid and counseling, as well as better publicizing scholarship and loan-forgiveness opportunities, could be seen as a neutral or bipartisan approach. However, the emphasis on broad accessibility and support might slightly lean towards a liberal viewpoint, due to its focus on governmental support and resource allocation.<br><br>In summary, while these points do not neatly fit into a singular political category, they generally align more with liberal viewpoints due to their emphasis on increased funding, government involvement in education, and the accessibility of medical training and resources. Conservative viewpoints, in contrast, often emphasize reduced government spending and intervention, personal responsibility, and market-driven solutions.	probably liberal
ACP	Affiliation with LCME Approved Medical Schools	2019	The text you provided does not align explicitly with either a liberal or conservative viewpoint in the traditional political sense. It is a policy statement from the American College of Physicians (ACP) regarding the affiliation of teaching hospitals with medical schools accredited by the Liaison Committee on Medical Education (LCME) and schools associated with the American Association of Colleges of Osteopathic Medicine (AACOM).<br><br>This statement focuses on medical education and the organization of teaching hospitals, which are generally considered administrative and educational issues rather than partisan political ones. Such topics typically don't fall squarely within the liberal-conservative spectrum as they are more about the practicalities of medical education and healthcare administration.<br><br>Both liberal and conservative viewpoints might support the idea of improving medical education and training, although the reasons and methods supported might vary based on political ideology. However, this specific statement is more about the best practices in medical education rather than a political ideology.	neutral

ACP	The Case for Graduate Medical Education as a Public Good	2022	The text provided presents a viewpoint that aligns more closely with liberal principles, particularly in its emphasis on broad access to education, diversity, and societal responsibility. Here are the key aspects:<br><br>1. **Public Good and Societal Benefit**: The argument that graduate medical education is a public good benefiting all of society aligns with liberal viewpoints, which often emphasize the role of education as a societal responsibility and a means to promote the general welfare.<br><br>2. **Financial Support from All Payers**: The idea that all who pay for healthcare services should contribute to funding graduate medical education reflects a liberal perspective on shared societal responsibility and the redistribution of resources for the common good.<br><br>3. **Access for Lower and Middle-Income Families**: The emphasis on maintaining opportunities for students from lower and middle-income backgrounds, and the concern that without support, access will be limited to the affluent, mirrors liberal concerns about inequality and the importance of maintaining social mobility.<br><br>4. **Ethnic and Racial Diversity**: Efforts to increase ethnic and racial diversity in the medical profession are typically associated with liberal values, which prioritize inclusivity and equal opportunities for underrepresented groups.<br><br>5. **Quality of Patient Care Services**: The focus on maintaining high standards of quality in patient care and the training of resident physicians, while not exclusively liberal, complements the liberal emphasis on the importance of public services and welfare.<br><br>6. **Opportunities for the Best Qualified Candidates**: The argument that opportunities in the medical profession should be available to the best qualified, regardless of their financial background, aligns with the liberal ideal of meritocracy tempered by social equity.<br><br>In summary, the text emphasizes equitable access to education, societal responsibility, diversity, and quality in public services, which are key elements typically associated with liberal viewpoints.	liberal
ACP	Internal Medicine Training	2019	The text you've provided is primarily focused on advocating for the importance and preservation of traditional broad-based training in internal medicine. It emphasizes the role of internists in primary care, the value of internal medicine in the healthcare system, and the impact of internists in various training and research roles.<br><br>This text doesn't align strictly with a traditional liberal or conservative political viewpoint. Instead, it represents a professional stance regarding medical education and training within the field of internal medicine. The emphasis is on the importance of comprehensive training for internists and the objection to reducing internal medicine training in favor of other primary care training programs.<br><br>Issues of medical education and training often transcend typical political divisions and are more focused on healthcare policy, educational standards, and the quality of medical care. The perspective here is more about maintaining a high standard in medical education and ensuring that internists are well-prepared for their roles in healthcare, rather than aligning with a specific political ideology.	neutral

ACP	Graduate Medical Education (GME) Funding/Physician Workforce Policy	2017	The text provided appears to align more closely with liberal viewpoints, particularly in the context of American politics. Here are the reasons why:<br><br>1. **Support for International Physicians**: The idea of continuing to provide Graduate Medical Education (GME) opportunities for non-US citizens who have graduated from non-U.S. medical schools under the J-1 Exchange Visitor Program is in line with liberal perspectives on immigration and diversity. Liberals tend to support policies that encourage cultural and professional diversity and international exchange.<br><br>2. **Focus on Underserved Communities**: The emphasis on federal funding to support medical schools' efforts in expanding opportunities in underserved communities, increasing diversity, and encouraging careers in generalist medicine is consistent with liberal priorities. These priorities include addressing healthcare disparities, promoting social justice, and ensuring equitable access to healthcare.<br><br>3. **Establishment of a National All-Payer Fund for GME**: Proposing a national all-payer fund for the direct costs of GME indicates a preference for a more centralized, government-involved approach to healthcare funding. This approach is typically more aligned with liberal ideologies that favor greater government involvement in healthcare to ensure access and equity.<br><br>4. **Creation of a National Physician Workforce Advisory Body**: The suggestion to establish a national body to assess the physician workforce aligns with liberal tendencies towards regulatory oversight and planning to address systemic issues in healthcare.<br><br>5. **Evaluation of Consortia and Voucher System for Workforce Policy**: Evaluating various approaches for implementing workforce policy goals and controlling GME funds suggests a methodical, systemic approach to healthcare policy, which is a characteristic of liberal perspectives.<br><br>6. **Anti-Discrimination Policies in Training Programs**: Advocating for strict anti-discrimination policies in GME training programs is consistent with liberal values of equality, inclusivity, and non-discrimination.<br><br>7. **Balance of Ambulatory and Inpatient Experiences in Training**: Supporting a balanced approach in medical training, including the unification of primary care and internal medicine residency, reflects a focus on comprehensive healthcare, a principle more commonly associated with liberal healthcare policies.<br><br>Overall, these points indicate a viewpoint that values diversity, equity, systemic reform, and government involvement in healthcare, which are key aspects of liberal ideology in the United States.	liberal

ACP	Aligning GME Policy with the Nation’s Healthcare Workforce Needs	2022	The statements provided reflect a nuanced approach to healthcare and medical education policy, and while they may not align squarely with traditional liberal or conservative viewpoints, elements of both can be identified:<br><br>1. **Liberal Viewpoint Elements:**<br>- **Emphasis on Government Funding and Support:** The call for increased Medicare funding, government support for medical education, and the idea of a performance-based payment system for Graduate Medical Education (GME) align with liberal principles favoring government involvement in ensuring public welfare and education.<br>- **Focus on Primary Care and Workforce Needs:** The emphasis on training more primary care physicians and addressing healthcare workforce shortages mirrors liberal concerns about ensuring equitable access to healthcare services for all populations.<br><br>2. **Conservative Viewpoint Elements:**<br>- **Medical Educators Leading Curriculum Development:** The idea that medical educators, rather than governments, should lead in improving GME curricula aligns with conservative values of limited government intervention in professional and academic matters.<br>- **Pilot Projects and Innovation:** The encouragement of pilot projects and innovation in GME, along with the concept of a Center for Medical Education Innovation and Research, can be seen as aligning with conservative principles that favor market-driven solutions and experimentation in the private sector over government mandates.<br><br>3. **Balanced Aspects:**<br>- **Performance-based Payment System:** While the proposal of a performance-based GME payment system could be seen as a market-oriented approach (more conservative), the careful development and consideration of such a system to avoid destabilizing physician training also reflects a more cautious, potentially liberal approach to policy implementation.<br>- **All Payers Contributing to Financing Pool:** This idea suggests a more collective approach to funding medical education, which might be seen as a liberal principle, but it also involves spreading responsibility across all stakeholders, which can be interpreted as a fiscally responsible, conservative idea.<br><br>In summary, these statements represent a blend of both liberal and conservative ideas, emphasizing the role of government in supporting medical education and healthcare workforce development while also advocating for innovation, limited government intervention in curriculum development, and collective financial responsibility. This blend reflects the complexity and multifaceted nature of healthcare policy, which often transcends simple political categorization.	neutral

ACP	Core Principles on Physician Workforce and Graduate Medical Education	2023	The points you've provided do not neatly align with a strictly liberal or conservative viewpoint, as they incorporate elements that might be endorsed by both sides of the political spectrum. However, they can be analyzed in the context of typical liberal and conservative perspectives:<br><br>1. **Adjusting Medical School Class Size and Graduates:** This aligns more with a liberal perspective, focusing on planned, needs-based education and workforce development, a concept often favored by liberals who advocate for proactive government involvement in such planning.<br><br>2. **Shared Costs for Graduate Medical Education:** This principle, suggesting collective responsibility for funding education, leans towards a liberal viewpoint, emphasizing communal support for public goods like healthcare education.<br><br>3. **Balanced Mix of Generalists and Specialists:** This could be seen as neutral, appealing to both sides. Conservatives might appreciate the focus on efficient allocation of resources, while liberals might appreciate the emphasis on meeting national healthcare needs.<br><br>4. **Considering Non-Physician Health Care Professionals in Workforce Planning:** This is a more technocratic point and doesn't strongly align with a specific political ideology. It reflects a practical approach to healthcare workforce planning.<br><br>5. **Improving Geographic Distribution of Physicians:** The focus on addressing disparities and underserved populations is typically more aligned with liberal policies, which often emphasize social equity and targeted assistance to marginalized groups.<br><br>6. **Non-Discrimination in Medicine:** The strong emphasis on diversity and inclusion aligns with liberal values, particularly the focus on multiple dimensions of identity and non-discrimination.<br><br>7. **Funding for Graduate Medical Education:** The emphasis on stable, sufficient funding for healthcare education and support for indigent patients aligns more with liberal policies, which often advocate for robust public funding in healthcare and support for underprivileged populations.<br><br>Overall, while some points have a more neutral or technocratic tone, the overall emphasis on planned, equitable, and inclusive healthcare education and workforce development leans more towards a liberal perspective. However, it's important to note that these are broad generalizations and there can be considerable overlap and variation within each political ideology.	neutral

ACP	Financing U.S. Graduate Medical Education	2016	The text provided aligns more closely with a liberal viewpoint. This assessment is based on the emphasis on government involvement and regulation in graduate medical education (GME), a typical characteristic of liberal perspectives. Here's a breakdown of the key points that align with a liberal viewpoint:<br><br>1. **Federal Government Commitment to GME**: Advocating for continued government involvement in GME funding aligns with liberal views favoring government roles in public welfare and education.<br><br>2. **Financing Pool from All Payers**: The idea that all payers should contribute to a financing pool for residencies aligns with liberal principles of shared responsibility and communal funding for public goods.<br><br>3. **Evaluation Before Distribution of Funds**: Calling for a thorough evaluation before allocating funds is in line with a regulatory approach typical of liberal policies, which often emphasize the need for oversight and informed decision-making in public programs.<br><br>4. **Combining GME and IME into a Single Payment Program**: This suggests a streamlined, government-regulated approach, aligning with liberal preferences for organized, systemic solutions to public issues.<br><br>5. **Transparency in GME Funding**: Emphasizing transparency and appropriate allocation of government funds reflects liberal priorities for accountability in public spending.<br><br>6. **Lifting GME Caps**: Proposing to lift caps on GME to train more physicians, especially in primary care and other shortage areas, resonates with liberal views on expanding access to healthcare and addressing public health needs.<br><br>7. **Performance-Based GME Payment System**: While the concept of performance-based systems can be found across the political spectrum, the emphasis here on thoughtful development, stakeholder involvement, and alignment with educational goals is more characteristic of a liberal approach, which often stresses inclusivity, evidence-based policies, and cautious implementation of new systems.<br><br>8. **Pilot Projects for GME Funding**: Suggesting pilot projects to innovate in GME aligns with a liberal inclination towards experimentation and progressive reform in public sectors.<br><br>9. **Primary Care Training in Diverse Settings**: Advocating for primary care training in various settings, including non-hospital environments, and promoting innovation in training aligns with liberal values of diversity, innovation, and comprehensive education.<br><br>Overall, the emphasis on government involvement, regulation, transparency, inclusivity, and innovation in these points aligns more closely with liberal political ideologies.	liberal
ACP	Implementing Universal State and Federal J-1 Visa Application Processes	2022	The text you've provided outlines policy positions regarding the J-1 visa application process and the Conrad 30 program. These policies are focused on simplifying visa processes, ensuring fair distribution of physicians in underserved areas, and providing grace periods for physicians.<br><br>This approach tends to align more closely with a liberal viewpoint, particularly in its emphasis on facilitating immigration processes for medical professionals and focusing on equitable distribution of resources to underserved areas. Liberal perspectives often prioritize inclusive immigration policies and addressing inequalities in healthcare access.<br><br>Conservative viewpoints, on the other hand, might emphasize stricter immigration controls and less government intervention in determining the distribution of healthcare resources. They might be more inclined to maintain existing structures or to emphasize state-level decision-making over federal involvement.<br><br>It's important to note that the distinction between liberal and conservative viewpoints can be complex and vary depending on specific issues, contexts, and interpretations. This assessment is a generalization and may not fully capture the nuances of all liberal or conservative positions on these matters.	liberal

ACP	The Role of International Medical Graduates in the U.S. Physician Workforce	2019	The text you've provided reflects a viewpoint that aligns more with liberal policies, particularly in the context of U.S. political ideologies. The key aspects that suggest this alignment are:<br><br>1. **Support for International Medical Graduates**: The statement supports easing the immigration process for international medical graduates, which aligns with more liberal immigration policies.<br><br>2. **Streamlining Visa Processes**: The advocacy for streamlining J-1 and H1B visa processes for non-U.S. citizen international medical graduates is consistent with liberal viewpoints that generally favor more open and efficient immigration systems.<br><br>3. **Expansion of Visa Waiver Programs**: The support for expanding programs like Conrad 30, aimed at addressing physician shortages in underserved areas, reflects a commitment to healthcare accessibility, a priority often associated with liberal policies.<br><br>4. **Exemption from Visa Caps**: The position to exempt physicians in shortage specialties from annual H-1B visa caps and per-country limitations for employment-based immigrants indicates a liberal stance on immigration and healthcare workforce policies.<br><br>5. **Global Collaboration and Health Corps Development**: Encouraging collaboration between U.S. and international medical schools and supporting the creation of a Global Health Corps align with a more globalist and cooperative approach often found in liberal ideologies.<br><br>In summary, the text promotes liberal viewpoints, emphasizing open immigration policies for medical professionals, global collaboration in healthcare, and addressing healthcare accessibility, particularly in underserved areas.	liberal
ACP	Position Statement on Residency Program Closure Processes and Prevention of Hardship for Trainees	2022	The text you provided appears to be a policy statement or position from the American College of Physicians (ACP) concerning the management of residency or fellowship training programs in the event of program closures. These statements are primarily concerned with ensuring the well-being, rights, and educational continuity of medical trainees (residents and fellows) affected by such closures. Here's a breakdown of the alignment of these points with liberal or conservative viewpoints:<br><br>1. **Emphasis on Orderly Transfer and Communication**: This point emphasizes the importance of maintaining professional standards and clear communication in the transfer of trainees. This approach is generally aligned with both liberal and conservative values, as it emphasizes systematic responsibility and the importance of maintaining educational standards.<br><br>2. **Support for Residency Closure Policy**: The ACP's support for existing policies enforced by the Accreditation Council for Graduate Medical Education (ACGME) suggests a respect for established procedures and regulatory frameworks. This can be seen as aligning with conservative values that emphasize respect for established systems and institutions.<br><br>3. **Mitigating Immigration Hardships**: The support for mitigating immigration hardships for residents and fellows on training-related visas leans more towards a liberal viewpoint, which typically emphasizes compassion and support for individuals facing immigration challenges.<br><br>4. **Transfer of Funding for Displaced Trainees**: The stance on funding transfer and financial responsibility of the closing hospital aligns with a sense of fairness and responsibility towards the trainees, a view that can be seen as more liberal, focusing on the rights and welfare of individuals.<br><br>5. **Acknowledgment of Additional Hardships and Ethical Responsibilities**: The acknowledgment of various hardships faced by trainees and the urge for institutions to act responsibly and compassionately aligns with liberal values that emphasize empathy, support for individuals in challenging situations, and ethical responsibility.<br><br>Overall, while some aspects of these points align more closely with liberal values, particularly those related to immigration and individual welfare, the overall emphasis on maintaining professional standards and respecting established systems can also resonate with conservative values. The text primarily reflects a professional and ethical standpoint focused on the welfare of medical trainees, transcending a simple liberal-conservative dichotomy.	probably liberal

ACP	Investigating Possible Work-Related Abuses for Physicians Working Under the Conrad-30 Program	2023	The text you've provided appears to be a policy statement from a medical organization, specifically the American College of Physicians (ACP), focusing on creating a mechanism for members to report job-related abuses without fear of retribution. This statement is primarily oriented towards workplace fairness and advocacy for medical professionals.<br><br>Determining whether this text aligns with a liberal or conservative viewpoint can be complex because it touches on issues relevant to both. On one hand, the focus on protecting workers from abuses and advocating for fair employment practices could be seen as aligning with liberal viewpoints, which often emphasize worker rights and protections. On the other hand, the text doesn't necessarily align with partisan politics and could be seen as a professional organization's effort to ensure a fair and safe working environment for its members, a concern that can transcend political leanings.<br><br>In summary, while aspects of this statement might resonate more with liberal perspectives on worker rights, it's primarily a professional, rather than a political, statement focused on workplace fairness and safety in the medical field. It's important to consider that many organizational policies, especially in professional fields like medicine, are often designed to address specific functional or ethical issues rather than align with a particular political ideology.	probably liberal
ACP	Outpatient Residency Training	2019	The statement "ACP supports changes in the Centers for Medicare & Medicaid Services rules and regulations that would facilitate training of hospital-funded residents in non-hospital outpatient facilities" is primarily aligned with a liberal viewpoint. This stance is indicative of a preference for expanded access to healthcare and training, a position commonly associated with liberal ideologies. Liberal perspectives often emphasize the importance of healthcare access and the expansion of training and resources in the healthcare system.<br><br>Conservatives, on the other hand, might be more cautious about changes that could increase government spending or regulations. However, it's important to note that the issue of healthcare training and regulations can cross ideological lines, and there may be conservatives who support such changes for reasons such as improving medical education or efficiency in healthcare delivery.<br><br>In summary, while the statement leans more towards a liberal viewpoint due to its emphasis on expanding healthcare training and access, it's not strictly limited to one political ideology and can find support across the spectrum depending on the specific context and rationale.	probably liberal
ACP	Physician Workforce and Residency Training	2019	The text from the American College of Physicians (ACP) appears to align more with a liberal viewpoint, primarily due to its emphasis on diversity and the development of objective measures for quality assessment in medical training. Liberal perspectives often prioritize diversity and inclusivity in various sectors, including education and healthcare. The support for a variety of training programs to prepare residents for diverse practice environments also reflects a liberal inclination towards adaptability and inclusiveness in professional training.<br><br>Conservative viewpoints, on the other hand, might focus more on traditional methods of education and evaluation, potentially less emphasizing the diversity of backgrounds or training environments to the same degree.<br><br>However, it's important to note that the concepts of diversity and quality in education and training can be valued across the political spectrum, and the distinction between liberal and conservative viewpoints can sometimes blur, especially in contexts like medical education that prioritize both excellence and inclusivity.	liberal
ACP	Funding for Combined Residency Training Programs	2019	The statement about Medicare payments for graduate medical education appears to be primarily focused on policy specifics rather than aligning clearly with a traditional liberal or conservative viewpoint. It deals with the funding and structure of medical education, particularly for residents in combined primary care training programs.<br><br>Liberal and conservative viewpoints in the United States generally differ on matters like the size and role of government, social issues, and economic policies. However, when it comes to specific technical policies like this one, the alignment is not always clear-cut. This statement could be interpreted in different ways depending on the broader context:<br><br>1. **Liberal Viewpoint**: If seen as advocating for increased funding or support for medical education, it could align with liberal values that often support greater investment in public services and education.<br><br>2. **Conservative Viewpoint**: On the other hand, if this policy is seen as a way to make medical education more efficient or to control government spending in this area, it could be interpreted as aligning with conservative values, which often emphasize fiscal responsibility and efficiency in government spending.	neutral

			Overall, the statement seems to be more about the efficient allocation of resources and ensuring quality in medical education rather than aligning with a particular ideological stance. It's also important to consider that many policy positions do not neatly fit into a liberal or conservative framework, especially those that are highly technical or specialized, like this one.	
ACP	Attending Physicians and Physicians in Training	2016	The text you've provided primarily outlines the ethical and professional responsibilities of physicians, particularly in the context of teaching and patient care. It doesn't align specifically with either a liberal or conservative viewpoint in the political sense. Instead, it focuses on medical ethics, the duties of physicians in a teaching environment, and the importance of proper supervision and delegation of responsibilities in patient care.<br><br>This text emphasizes the importance of knowledge sharing, mentorship, and ethical conduct in the medical profession. It addresses the responsibilities of attending physicians in teaching and supervising medical trainees, ensuring patient welfare, and maintaining high standards of care. It also highlights the ethical obligation not to delegate patient care to unqualified individuals.<br><br>Overall, the text is more aligned with professional and ethical guidelines in the field of medicine rather than any political ideology.	neutral
ACP	Recommendations on Reform of Residency Training	2019	The text you've provided appears to be a set of recommendations from the American College of Physicians (ACP) regarding graduate medical education, specifically focusing on residency training. These recommendations do not explicitly align with a traditional liberal or conservative viewpoint. Instead, they focus on various aspects of medical training and practice, including community service, preventive care, humanistic values, work-life balance, exposure to diverse patient populations, procedural training, research, and benefits for residents.<br><br>The topics addressed in these recommendations, such as emphasis on public service, preventive care, humanistic medicine, and resident welfare, could be interpreted as aligning with values that are often associated with a more progressive or liberal approach to healthcare and education. These values include a focus on holistic care, community involvement, and the well-being of healthcare providers.<br><br>However, it's important to note that such initiatives and recommendations in the field of medicine often transcend traditional political boundaries. The primary aim of these recommendations is to improve the quality of medical education and healthcare delivery, which is a goal shared across the political spectrum. The emphasis on comprehensive training, exposure to diverse medical conditions, and the importance of research and procedural skills are elements that could find support regardless of political affiliation.<br><br>In summary, while some aspects of the ACP's recommendations might resonate more with certain political ideologies, the recommendations themselves are focused on improving medical education and healthcare delivery, which is a universally important objective.	neutral

ACP	Residency Work Hours and Compensation	2016	The text you've provided, which states that the American College of Physicians (ACP) believes that reductions in resident compensation to fund changes in graduate medical education is inappropriate, does not align strictly with either a liberal or conservative viewpoint in the traditional political sense. Instead, it reflects a professional and ethical stance regarding the funding of graduate medical education and the compensation of medical residents.<br><br>In the context of U.S. politics, "liberal" and "conservative" viewpoints often relate to broader economic and social policies rather than specific professional practices or standards within a field like medicine. The ACP's statement focuses on the fair compensation of medical residents and the integrity of funding for medical education, issues that transcend typical political divides and are more about professional standards and ethics in the medical field.<br><br>However, if we were to analyze it in a broader political context, one might argue that the statement aligns somewhat more with liberal principles, which generally emphasize fair labor practices and adequate funding for education. Conservatives might be more inclined to support market-driven solutions and might be less opposed to cost-cutting measures. But again, this is a very broad and not entirely accurate way to categorize a position that is primarily about professional standards in medicine.	neutral
ACP	Universal Hepatitis B Vaccination	2016	The statement "ACP recommends that medical schools and residency programs offer hepatitis B vaccine free of charge to its physicians-in-training and medical students" reflects a viewpoint that focuses on public health and preventative care. It doesn't align strictly with traditional conservative or liberal viewpoints as it addresses a health care issue rather than a political ideology.<br><br>In general:<br><br>1. **Liberal Viewpoint**: Liberals typically advocate for accessible healthcare for all, including preventive measures. They may support such a recommendation as it aligns with their broader goals of public health and ensuring that healthcare professionals are protected and healthy.<br><br>2. **Conservative Viewpoint**: Conservatives often prioritize individual responsibility and might argue that medical professionals should manage their own healthcare needs. However, they might also agree with this recommendation as it addresses the practical need to protect healthcare workers from a public health perspective.<br><br>The recommendation by the ACP (American College of Physicians) seems to be more about ensuring the health and safety of medical professionals, which transcends typical political divisions. It's a practical stance rather than a purely ideological one.	neutral
ACP	Private Patients in the Teaching Setting	2019	The text you provided outlines policies and procedures for teaching hospitals, emphasizing the role and responsibilities of attending physicians, particularly in relation to house officers (or residents). It stresses the importance of clear guidelines, adequate supervision, and the ultimate responsibility of the attending physician in patient care.<br><br>This text does not neatly align with a traditional liberal or conservative viewpoint. Instead, it focuses on administrative and ethical considerations in a medical teaching environment. Political ideologies like liberalism or conservatism typically address broader societal and governmental issues, whereas this text is specific to medical governance and education practices.<br><br>Liberal viewpoints generally advocate for progressive changes in social policies, increased governmental intervention in healthcare, and support for public welfare programs. Conservative viewpoints often emphasize personal responsibility, limited government intervention, especially in economic affairs, and traditional values.<br><br>The content of this text, however, centers on the operational and ethical management of medical training and does not directly address broader political or ideological issues that typically distinguish liberal and conservative viewpoints. It's more aligned with professional medical standards and educational practices than with any particular political ideology.	neutral

ACP	Teaching of Socioeconomics in Medical Schools and Residency Programs	2016	The statement you've provided reflects a perspective that emphasizes the importance of incorporating socioeconomics into medical education. This viewpoint is generally aligned with liberal ideologies, which often prioritize the integration of social factors, like socioeconomics, into various professional fields, including medicine. Liberal perspectives typically advocate for a broader understanding of the factors that affect individual and community health, beyond just biological aspects.<br><br>Conservative viewpoints, on the other hand, might be more inclined to focus on traditional medical education centered around biological and clinical sciences, potentially arguing that socioeconomics falls outside the primary scope of medical training.<br><br>However, it's important to note that the distinction isn't always clear-cut. There can be overlap, and individuals or groups within both liberal and conservative circles might support or oppose such initiatives based on various factors beyond a simple liberal-conservative divide. The complexity of healthcare issues often transcends traditional political lines.	liberal
ACP	Underprivileged Students	2016	The text from the American College of Physicians (ACP) emphasizes the role of medical practitioners in guiding and supporting underprivileged students throughout their education and career. This perspective aligns more closely with a liberal viewpoint for several reasons:<br><br>1. **Focus on Social Responsibility and Equality**: The statement highlights the importance of social responsibilities, a common theme in liberal ideologies. Liberals often advocate for policies and actions that support equal opportunities and address social inequalities.<br><br>2. **Support for Underprivileged Groups**: The specific mention of helping underprivileged students is a hallmark of liberal thinking, which tends to emphasize the need to assist marginalized or disadvantaged groups to achieve fairness and social justice.<br><br>3. **Emphasis on Education and Training**: Liberals generally prioritize education and believe in providing resources and support to ensure that all individuals, regardless of their background, have access to quality education and training.<br><br>4. **Long-Term Involvement in Social Welfare**: The text suggests a long-term commitment to guiding students from pre-medical training through to their professional practice. This approach is consistent with liberal perspectives that advocate for sustained support and intervention to address social issues.<br><br>While this viewpoint is more aligned with liberal ideologies, it's important to note that support for education and social responsibility can be found across the political spectrum, and the interpretation may vary depending on individual perspectives and contexts.	liberal
ACP	The Medical Record	2019	The text you provided doesn't neatly align with either a liberal or conservative viewpoint. Instead, it focuses on ethical and legal guidelines for maintaining and handling medical records, which is a matter of professional conduct in healthcare rather than a political issue.<br><br>The principles outlined in the text emphasize the importance of accuracy, completeness, and confidentiality in medical records. It stresses that patients have both an ethical and legal right to access their medical records, and that physicians must comply with this, irrespective of circumstances like nonpayment of medical bills. Furthermore, it discusses the use of electronic health records (EHRs), highlighting the need for these tools to enhance patient care while also maintaining privacy and security standards.<br><br>Overall, the focus of this text is on best practices in medical record-keeping and patient confidentiality, which are critical aspects of healthcare management. These topics are generally considered necessary professional standards in medicine and are not typically associated with a particular political ideology.	neutral

ACP	Health Information Technology and Privacy	2022	The text you've provided primarily aligns with a liberal viewpoint, particularly in its emphasis on privacy, informed consent, and patient rights within the healthcare system. Key elements that suggest a liberal perspective include:<br><br>1. **Emphasis on Individual Privacy and Consent**: The strong focus on safeguarding patient privacy, requiring informed consent for the use of Protected Health Information (PHI), and advocating for the use of deidentified or anonymized data aligns with liberal values that prioritize individual rights and privacy.<br><br>2. **Regulatory Framework for PHI**: The support for comprehensive federal privacy and security legislation, as well as the detailed guidelines for the use and sharing of PHI, reflects a liberal inclination towards regulatory oversight and the protection of individual rights within the healthcare system.<br><br>3. **Patient Empowerment**: The text advocates for patient empowerment, such as the right to access their PHI, make informed decisions, and be educated about their privacy rights. This approach aligns with liberal values that emphasize the empowerment and autonomy of individuals in healthcare decisions.<br><br>4. **Focus on Ethical Standards and Public Health**: The balance between the use of PHI for public health purposes and the protection of individual privacy rights is a hallmark of liberal health policy, which often seeks to balance individual rights with broader societal benefits.<br><br>5. **Advocacy for Inclusivity and Accessibility**: The call for consent models that account for literacy levels and preferred language, and the emphasis on making health information technology (HIT) accessible and equitable, are indicative of a liberal perspective that emphasizes inclusivity and equality in healthcare access.<br><br>While some aspects, like the call for a regulatory framework and the emphasis on societal benefit, could find resonance in conservative viewpoints, the overall emphasis on individual rights, privacy, and a strong regulatory approach to protect these rights is more aligned with liberal ideologies.	liberal

ACP	Confidentiality of Electronic Medical Records	2019	The text you provided outlines principles regarding patient privacy, access to medical records, informed consent, and the use of patient data in research. These principles do not align neatly with either a liberal or conservative viewpoint as traditionally defined in political terms. Instead, they are more closely related to ethical standards in healthcare and medical practice.<br><br>1. **Patient Privacy and Medical Record Confidentiality**: This principle emphasizes the protection of patient privacy, a concern that transcends political ideologies. Both liberal and conservative viewpoints generally agree on the importance of privacy, though the emphasis and approach might differ.<br><br>2. **Purpose of Patient Medical Records**: This is a procedural statement about the use of medical records, largely neutral in political terms.<br><br>3. **Restricted Access to Medical Information**: This principle, advocating for limited access to sensitive medical information, is consistent with broader concerns about privacy and confidentiality. It doesn't inherently favor a liberal or conservative stance.<br><br>4. **Patient Rights to Access and Amend Records**: This is about empowering patients and ensuring transparency, which can be seen as aligning with broader liberal values of individual rights but also fits within conservative views on personal responsibility and autonomy.<br><br>5. **Informed Consent for Disclosure**: Requiring informed consent aligns with ethical standards in healthcare, emphasizing patient autonomy and decision-making, values that are important across the political spectrum.<br><br>6. **Restrictions on Disclosures for Non-Healthcare Needs**: This suggests a cautious approach to sharing medical information, potentially aligning more with conservative views on law enforcement and legal procedures, but it also reflects a commitment to privacy valued by liberals.<br><br>7. **Use of De-identified Data in Research**: This aligns with ethical research practices and doesn't inherently favor a political ideology.<br><br>8. **Consent for Use of De-identified Data for New Purposes**: This principle reinforces the importance of informed consent, a concept valued across the political spectrum.<br><br>9. **Disclosure for Approved Research Only**: This is in line with ethical research standards and doesn't neatly fit into a liberal or conservative framework.<br><br>In summary, these principles are more about ethical healthcare practices and patient rights than about political ideology. They encompass values like privacy, autonomy, and responsible use of information that are important to both liberal and conservative perspectives in different ways.	neutral
ACP	Data Needs of Medical Research	2019	The text you provided appears to represent a viewpoint that seeks a balanced approach to privacy and access to medical records. It doesn't align strictly with a traditional liberal or conservative viewpoint but rather reflects a pragmatic approach often found in the healthcare policy domain.<br><br>1. **Liberal Viewpoint**: Typically, liberal perspectives emphasize the importance of protecting individual privacy and rights, including the confidentiality of medical records. This viewpoint would strongly support the aspect of the statement that calls for safeguards to prevent breaches of patient confidentiality.<br><br>2. **Conservative Viewpoint**: Conservative viewpoints often prioritize efficiency and minimizing regulatory burdens in healthcare. The part of the statement that mentions avoiding "unduly restrictive barriers" that could impede access to data for medical or public health research might resonate more with conservative values.<br><br>The statement you've provided seeks to balance these two priorities: protecting patient confidentiality while ensuring that necessary data is accessible for research and public health purposes. This kind of balanced approach is typical in healthcare policy discussions, where the need to protect individual rights must be weighed against the benefits of research and public health monitoring.	neutral

			Regarding federal standards or legislation, there are ongoing debates and discussions about the best ways to protect patient privacy while also allowing for the efficient use of data in medical research and public health. The specific reference to "electronic transmissions" of medical records suggests a recognition of the growing importance of digital health data and the unique challenges it presents in terms of privacy and access.	
ACP	EHR-Based Quality Measurement and Reporting - Critical for Meaningful Use and Health Care Improvement	2022	Determining whether these positions align more with a liberal or conservative viewpoint is complex because they pertain to health information technology (HIT) and Electronic Health Records (EHR), which are more technical and administrative aspects of healthcare rather than ideological. However, I can provide some insights into how these positions might be viewed through a political lens:<br><br>1. **Focus on Cost-Effective Healthcare**: This is a universal goal, but conservatives might be more vocal about cost-effectiveness, as it aligns with principles of fiscal responsibility.<br><br>2. **Engagement of All Stakeholders**: This principle could align with both liberal and conservative viewpoints. Liberals might emphasize the inclusion and fairness aspect, while conservatives might focus on the efficiency and effectiveness.<br><br>3. **Data Collection in Clinical Care**: This position is technical and not inherently political. Both sides might support this for different reasons: liberals for comprehensive care and conservatives for efficiency and reduced bureaucracy.<br><br>4. **Real-time Data Collection for Clinical Decision Support**: This is a technologically advanced approach that isn't specifically ideological. It might be supported across the political spectrum for its potential to improve healthcare delivery.<br><br>5. **Avoiding Increased Administrative Work and Costs**: Conservatives might particularly emphasize this point due to their focus on reducing regulatory burdens and costs. Liberals might also support it if it means more resources for patient care.<br><br>6. **Standardization of Data Elements**: This position is about efficiency and interoperability in healthcare IT, which is a generally bipartisan issue, though the level of regulation involved might be more scrutinized by conservatives.<br><br>7. **Support for Unified Standards Development**: This position, while technical, could be seen as more liberal if it's perceived as advocating for more regulation and standardization in healthcare. However, it also has a practical aspect that could be supported by conservatives for its potential to streamline and improve healthcare processes.<br><br>In summary, these positions mostly focus on improving healthcare efficiency and effectiveness through technology and standardization, which can be supported by both liberal and conservative viewpoints, although the emphasis and reasons for support might differ.	neutral

ACP	Clinical Documentation	2014	The text provided appears to focus on best practices for clinical documentation, emphasizing the role of technology and professional standards in improving patient care and outcomes. It doesn't align directly with a traditional liberal or conservative viewpoint as understood in political terms. Instead, it deals with healthcare policy and administration, areas where both liberal and conservative viewpoints may converge or diverge depending on the specific issue.<br><br>1. **Supporting Patient Care and Clinical Outcomes**: This is a universal goal in healthcare, irrespective of political affiliation. Enhancing communication for better patient care is a principle that transcends political boundaries.<br><br>2. **Professional Standards and Collaboration**: The call for physicians to work with organizations to define standards can be seen as a non-partisan issue. It relates more to professional governance and quality control in healthcare.<br><br>3. **Patient Access and Engagement**: Encouraging patient access to medical records aligns with broader goals of transparency and patient empowerment, which can be supported across the political spectrum.<br><br>4. **Use of Electronic Health Records (EHR)**: The emphasis on the effective use of EHRs and the concern about potential misuse for efficiency at the expense of accuracy and safety is a practical concern rather than a politically charged one.<br><br>5. **Value-Based Care and Accountable Care Models**: While the approach to healthcare financing and delivery models might be influenced by political views, the focus here is on improving outcomes through seamless patient care, a goal shared by many healthcare professionals.<br><br>6. **Coding and Documentation for Third Parties**: The opposition to unnecessary administrative burdens could resonate with both conservative principles of reducing bureaucracy and liberal principles of focusing resources on patient care.<br><br>7. **Research and Education in Health Information Technology**: This is about improving healthcare delivery and education, which are generally considered non-partisan objectives.<br><br>Overall, the text seems to be more about effective healthcare administration and less about ideological positions. It's more focused on practical, professional, and ethical considerations in healthcare documentation and technology use.	neutral
ACP	EHR System Design to Support 21st Century Clinical Documentation:	2014	The statements you've provided pertain to the optimization and functionality of Electronic Health Records (EHR) systems. They are largely technical and operational in nature, focusing on improving the efficiency, accuracy, and user-friendliness of EHR systems for both clinicians and patients. Here's a brief analysis of how each statement aligns with political viewpoints:<br><br>1. **Optimizing EHR for Team-Based Care:** This is a technologically progressive idea, focusing on enhancing healthcare delivery. It doesn't neatly align with either liberal or conservative viewpoints, as it is more about healthcare efficiency and patient care quality.<br><br>2. **Supporting Clinicians' Cognitive Processes:** This is about making EHR systems more intuitive and user-friendly for clinicians, which is a technological and practical matter rather than a politically charged one.<br><br>3. **"Write Once – Reuse Many Times" Principle:** This aims at improving data efficiency and accuracy in healthcare records. It's a principle of data management and technology optimization, not inherently political.<br><br>4. **Reducing Redundancy in EHR Systems:** This statement advocates for a more efficient system that avoids unnecessary steps. It's about streamlining processes, which is a general efficiency goal rather than a political statement.<br><br>5. **Integration of Patient-Generated Data:** This highlights the importance of patient involvement and data accuracy in EHRs. It leans towards patient empowerment and could be seen as aligning with a more progressive view of patient care, but it's still primarily a technological and healthcare quality issue.<br><br>Overall, these statements are more aligned with healthcare technology and management principles rather than with specific liberal or conservative political ideologies. They reflect a desire to improve the functionality and efficiency of healthcare systems, which is a universal concern across the political spectrum.	neutral

ACP	Application of Utilization Review Standards	2017	The statement from the American College of Physicians (ACP) regarding the application of basic quality standards set by state or federal government across all entities in the healthcare marketplace doesn't align strictly with either a liberal or conservative viewpoint. Instead, it reflects a position focused on healthcare policy and quality standards.<br><br>In the U.S. political context, liberals generally advocate for more government involvement in healthcare, including regulation and oversight to ensure quality and access. Conservatives tend to prefer less government intervention, emphasizing market-driven solutions and competition to improve quality and efficiency.<br><br>The ACP's stance on applying uniform quality standards to all entities in the healthcare market, including Independent Physician Associations (IPAs), medical groups, and other physician or hospital-directed organizations, is primarily a healthcare policy position. It advocates for standardization and quality assurance, which can be seen as aligning with the liberal viewpoint of ensuring government oversight for quality healthcare. However, it doesn't explicitly call for increased government intervention in the healthcare market, which might be more characteristic of a conservative approach to healthcare policy.<br><br>Thus, while the statement might find some support from both sides, it is more centered on healthcare policy and quality assurance rather than fitting neatly into a liberal or conservative political ideology.	neutral
ACP	Medical Appropriateness	2017	The text you've provided outlines a position from the American College of Physicians (ACP) regarding the appropriateness of medical tests, procedures, or investigations. It states that these are considered medically appropriate if they would significantly influence the diagnosis, course of treatment, or prognosis of a patient's illness, disease, or disability.<br><br>This statement is primarily clinical and evidence-based in nature, focusing on the practical and medical aspects of patient care. It doesn't align clearly with either a liberal or conservative political viewpoint. Instead, it reflects a medical and ethical perspective on patient care, emphasizing the importance of evidence and effectiveness in medical decision-making.<br><br>In the context of political ideologies, liberals typically advocate for wider access to healthcare and may support measures that ensure all treatments and tests necessary for patient care are accessible. Conservatives, on the other hand, might emphasize the cost-effectiveness and efficiency of healthcare services, potentially supporting this viewpoint if it leads to more efficient use of medical resources.<br><br>However, the statement itself is neutral and focused on medical best practices rather than political ideology. It's an example of a professional medical guideline aimed at ensuring high-quality patient care based on evidence and clinical relevance.	neutral
ACP	Medical Necessity and Insurance Coverage	2017	The text you provided appears to be a policy or guideline statement related to the evaluation of appropriateness in a professional context, particularly in reference to post-payment reviews. It emphasizes the reliance on scientifically acceptable data and professional consensus, as well as the transparency and accountability in the review process.<br><br>This statement does not align specifically with either a liberal or conservative viewpoint in the traditional political sense. Instead, it focuses on principles of evidence-based practice, professional standards, and transparency, which are values that can be embraced by individuals across a broad political spectrum.<br><br>In the context of professional practice, especially in fields like healthcare, finance, or law, such guidelines are typically designed to ensure fairness, accuracy, and objectivity in review processes. They advocate for decision-making based on empirical data and established professional norms, rather than subjective or biased judgments. This approach is generally considered a best practice in many professional fields, irrespective of political leanings.	neutral

ACP	Independent Review of Third Party Payers	2015	The statement "ACP supports the concept of an independent review entity with binding authority to adjudicate claims/disputes" doesn't align strictly with either a liberal or conservative viewpoint as traditionally defined in U.S. politics. It's more related to administrative and procedural policy rather than ideological stances.<br><br>1. **Liberal Viewpoint**: Generally, liberals tend to support regulatory bodies and oversight mechanisms to ensure fairness, justice, and protection of rights. An independent review entity could be seen as a means to provide unbiased adjudication, which aligns with these principles.<br><br>2. **Conservative Viewpoint**: Conservatives typically emphasize limited government intervention and may be skeptical of additional regulatory bodies. However, they also value the rule of law and fair dispute resolution. If such an entity is seen as a way to streamline processes and ensure fair outcomes without excessive government control, it could align with conservative values too.<br><br>In essence, the support for an independent review entity could be seen as a pragmatic approach to dispute resolution, rather than a strictly ideological one. It might be embraced by both liberals and conservatives depending on the specific context, the perceived effectiveness, and the way it aligns with broader principles of justice and efficiency.	neutral
ACP	Disclosure and Claims Review Requirements	2015	The text you've provided appears to be a policy statement related to healthcare, specifically focusing on the standardization and regulation of Utilization Review (UR) processes by health plans and private firms. It advocates for:<br><br>1. Standardizing training standards for those performing UR.<br>2. Regulating the criteria and parameters used by private UR firms.<br>3. Making the mechanisms of private UR firms more transparent.<br>4. Ensuring access to inquiries and appeals mechanisms offered by private UR firms.<br><br>This statement aligns more closely with a liberal viewpoint. Liberal policies typically emphasize the need for regulation and standardization in various sectors, including healthcare, to ensure fairness, transparency, and accountability. The emphasis on regulation, transparency, and access to appeals mechanisms reflects a concern for patient rights and oversight of private entities, which are common themes in liberal political philosophy.<br><br>However, it's important to note that healthcare policy can cross traditional political lines, and conservatives may also support certain regulations in the healthcare industry, particularly if they are seen as promoting efficiency, accountability, or quality of care. Therefore, while the statement leans more towards a liberal viewpoint, it's not exclusively so and could find some support across the political spectrum.	liberal
ACP	Utilization Review Committees	2015	The statement from the American College of Physicians (ACP) about members serving on society and hospital utilization committees to help control the use of beds, diagnostic agents, and therapeutic measures doesn't neatly align with a traditional liberal or conservative viewpoint. It focuses more on the practical aspects of healthcare management rather than ideological stances.<br><br>- **Healthcare Efficiency and Management**: This statement reflects a concern for efficient resource management in healthcare. It's about ensuring that medical resources like hospital beds and diagnostic tools are used effectively. This concern is shared across the political spectrum, though the methods of achieving it might vary.<br><br>- **Liberal Viewpoint Considerations**: Liberals generally support the efficient use of healthcare resources, but they tend to emphasize universal access to healthcare and may advocate for more government involvement in healthcare management.<br><br>- **Conservative Viewpoint Considerations**: Conservatives might agree with the need for efficient resource management in healthcare. However, they often prefer solutions that involve less government intervention, focusing more on market-driven approaches.<br><br>- **Contextual and Non-Political Nature**: The ACP's statement seems more focused on professional standards and practices within the healthcare industry. It's more about how physicians can contribute to the effective operation of healthcare facilities rather than a statement on broader healthcare policy.<br><br>In summary, while aspects of this statement might resonate more with one political group or another depending on their specific healthcare policies, the statement itself is primarily professional and managerial, rather than overtly political.	neutral

ACP	Medicaid Improvements for the Underserved	2017	The text you provided outlines several health care policy proposals, each with elements that can align with both liberal and conservative viewpoints, although they predominantly align with liberal perspectives. Here's a breakdown:<br><br>1. **Requirement for Managed Care Organizations to Provide Special Services in Inner-City Environments**: This proposal emphasizes accessible primary care, cultural and linguistic competence, coordination with social services, and capitation rates reflecting these services. This aligns more closely with liberal viewpoints, which often advocate for increased access to healthcare, especially for underserved communities, and an emphasis on social determinants of health.<br><br>2. **Restrict Direct Marketing and Use Independent Brokers for Health Plan Enrollment**: This suggestion aims to prevent "cherry-picking" (selecting only the healthiest individuals for insurance plans) and to provide objective information for enrollees. This could be seen as a liberal approach, focusing on regulation and fairness in the healthcare system. However, the emphasis on independent brokers and objective information can also align with conservative values of market-based solutions and consumer choice.<br><br>3. **Case Management for Persons with Serious Illnesses**: Providing case management for serious illnesses is a policy that could align with both viewpoints. Liberals might support it as a way to ensure comprehensive care and support for vulnerable populations. Conservatives might view it as a means to improve efficiency and reduce costs in healthcare by preventing unnecessary hospitalizations or emergency care.<br><br>4. **Include Risk-Adjustment Mechanisms for Costly Diseases**: This proposal is about protecting insurance plans from the financial risks of enrolling patients with expensive medical conditions. It aligns more with liberal perspectives that advocate for protections for individuals with pre-existing conditions and high medical costs. However, the concept of risk adjustment is also compatible with market-based insurance models, which can appeal to conservative viewpoints focused on financial sustainability.<br><br>In summary, while there are elements that both liberals and conservatives might support, the overall emphasis on accessibility, equity, and comprehensive care in these proposals leans more towards liberal viewpoints.	probably liberal
ACP	Developing a Medicare Buy-in Program	2016	The text presents viewpoints that align more closely with liberal ideologies, particularly those found in the United States. Here's an analysis of each point:<br><br>1. **Separate Financing for a Medicare Buy-in Program**: Advocating for a separate financing structure for a new Medicare Buy-in Program suggests a desire to expand healthcare coverage without overburdening the existing Medicare system. This aligns with liberal ideologies that support the expansion of government-run healthcare programs.<br><br>2. **Subsidies for Lower-Income Beneficiaries**: The idea of providing subsidies for lower-income individuals is a hallmark of liberal policies. It reflects a commitment to economic equity and social welfare, typical of liberal viewpoints.<br><br>3. **Eligibility for Ages 55-64 Regardless of Insurance Status**: Proposing to extend eligibility to a broader age range, regardless of current insurance status, indicates a move towards more inclusive healthcare coverage. This is consistent with liberal perspectives that often advocate for more comprehensive and accessible healthcare.<br><br>4. **Optional Enrollment with Full Benefits**: The emphasis on optional enrollment coupled with the provision of full Medicare benefits demonstrates a balance between offering expanded healthcare options and respecting individual choice. This approach, while practical, is more in line with liberal policies that focus on extending healthcare access.<br><br>Overall, these points suggest a liberal viewpoint, emphasizing healthcare expansion, inclusivity, and financial support for lower-income groups.	liberal

ACP	Promoting Transparency and Alignment in Medicare Advantage	2017	The text you provided reflects a perspective that is generally aligned with principles often associated with both liberal and conservative viewpoints in different aspects. Here's a breakdown:<br><br>1. **Emphasis on Funding and Beneficiary Care (Liberal)**: The support for ensuring that Medicare Advantage (MA) plans are funded at the level of the traditional Medicare program and that a significant portion of funding goes to actual beneficiary care aligns with liberal principles. Liberals typically advocate for strong funding of public health programs and ensuring that funds are used effectively for patient care.<br><br>2. **Transparency and Collaboration (Both)**: The call for transparency in the processes, policies, and procedures of Medicare Advantage Organizations (MAOs) and collaboration with stakeholders can be seen as a bipartisan issue. Both liberals and conservatives often support transparency and accountability in healthcare administration, although the reasons and approaches may differ.<br><br>3. **Administrative Simplification and Efficiency (Conservative)**: The focus on reducing administrative burdens, standardizing processes, and promoting efficiency in MA plans can align with conservative principles. Conservatives generally advocate for streamlined, efficient government programs and reduction of bureaucratic overhead.<br><br>4. **Quality Measurement and High-Quality Care (Both)**: The emphasis on aligning quality measurement systems and promoting high-quality care is a common goal across the political spectrum. Both liberals and conservatives value effective healthcare delivery, though they may prioritize different aspects.<br><br>5. **Value-Based Payment and System Reforms (Conservative)**: The advocacy for value-based payment models and delivery system reforms can be more closely aligned with conservative viewpoints, which often emphasize market-based solutions and reforms to improve efficiency and reduce costs in healthcare.<br><br>6. **Accountability and Investigation of Fraud (Both)**: Calling for investigations into potentially fraudulent activity and accountability for misuse in MA plans is a bipartisan issue. Both liberals and conservatives support measures to prevent fraud and misuse in healthcare programs.<br><br>7. **Beneficiary Choice and Understanding (Liberal)**: Ensuring that beneficiaries have clear information to compare benefits and options, and the stance against automatic enrollment in MA plans, aligns with principles of consumer protection and informed choice, which are often emphasized in liberal policies.<br><br>8. **Research on Federal Payments and Payment Models (Both)**: The call for more research into federal payments and payment models used by MAOs is a neutral stance, seeking data-driven insights to inform policy, a principle that transcends political lines.<br><br>Overall, the text incorporates elements that resonate with both liberal and conservative ideals, focusing on efficiency, transparency, beneficiary care, and accountability in healthcare administration.	neutral

ACP	Medicare Reform and Modernization	2023	The text you've provided appears to present a viewpoint that is generally aligned with a liberal perspective, particularly in the context of U.S. politics. Here's why:<br><br>1. **Support for Expanded Medicare Services**: The text advocates for the expansion of Medicare services, such as physician-directed Annual Wellness Visits and disease and case management. This is typically a liberal viewpoint, as liberals often support the expansion of government-provided healthcare services.<br><br>2. **Role of Subspecialists in Patient Care**: The statement that internal medicine subspecialists should be allowed to manage care for patients aligns with a more inclusive and comprehensive approach to healthcare, a stance commonly associated with liberal policies.<br><br>3. **Advocacy for New Funding Streams**: The suggestion that covered services should be adequately funded through new funding streams, rather than redirecting current funds, can be seen as a liberal stance. Liberals often advocate for increased funding for healthcare services, potentially through increased government spending or new sources of revenue.<br><br>4. **Reducing Paperwork for Physicians**: The point about avoiding over-burdensome paperwork for physicians is a bit more neutral and could be advocated by either liberals or conservatives. However, in the context of this statement, it seems to be more about ensuring that the expansion of services doesn't inadvertently create inefficiencies, which aligns with a focus on effective healthcare delivery, a concern of both liberals and conservatives, but presented here in a context that suggests a more liberal viewpoint.<br><br>It's important to note that such categorizations can be somewhat subjective and depend on the specific context and the broader spectrum of political beliefs in a given country.	liberal
ACP	ACP Support of Private Contracting Under Medicare	2022	The text you've shared reflects a blend of principles that could align with both liberal and conservative viewpoints in the United States, though it leans more towards a liberal perspective in certain aspects. Here's a breakdown:<br><br>1. **Support for the Doctor-Patient Relationship and Private Contracting**: The emphasis on the primacy of the doctor-patient relationship and the right to privately contract for care resonates with a conservative viewpoint, which often values individual choice and freedom in healthcare decisions.<br><br>2. **Patient Protections and Disclosures**: The requirement for physicians to disclose fees in advance and the stipulation that patients should be held harmless for charges exceeding the agreed amount align with liberal principles that focus on consumer protections and transparency in healthcare.<br><br>3. **Prohibitions on Private Contracting in Certain Cases**: The restrictions on private contracting in scenarios like when a physician is the sole community provider or when patients can't freely choose their physician, and the prohibition for dual Medicare-Medicaid eligible patients, align with a liberal viewpoint. These measures are designed to protect vulnerable populations and ensure equitable access to healthcare.<br><br>4. **Emergency and Life-Threatening Illness Care**: The requirement that private contracts must not reduce access to emergency or life-threatening illness care reflects a liberal perspective, emphasizing the importance of safeguarding access to essential health services.<br><br>5. **Monitoring and Reporting by Government Agencies**: The role of government agencies like the Centers for Medicare & Medicaid Services and the Medicare Payment Advisory Commission in monitoring healthcare access and reporting to Congress is a principle more commonly associated with liberal policies, which often support a more active role for government in healthcare.<br><br>In summary, while the support for private contracting and the emphasis on the doctor-patient relationship has conservative elements, the detailed patient protections, restrictions on private contracting in certain scenarios, and the involvement of government agencies in monitoring and reporting align more closely with liberal viewpoints.	liberal

ACP	Documentation of Evaluation & Management Visits	2017	The text provided doesn't neatly align with a strictly liberal or conservative viewpoint. Instead, it addresses operational and bureaucratic aspects of healthcare administration, specifically focusing on documentation requirements, utilization review, and compliance with Medicare guidelines. These issues are more about the efficiency and practicalities of healthcare delivery rather than ideological stances typically associated with liberal or conservative viewpoints.<br><br>In the U.S. political context:<br>- **Liberal (or progressive) viewpoints** often emphasize expanding access to healthcare, supporting government-run or heavily regulated healthcare systems, and prioritizing public health initiatives.<br>- **Conservative viewpoints** tend to emphasize free-market solutions, reduced government intervention in healthcare, and personal responsibility in health choices.<br><br>The text you've shared deals with:<br>1. **Reducing excessive documentation**: This could be seen as a practical measure that both liberal and conservative healthcare administrators might support, as it aims to streamline processes and reduce administrative burdens.<br>2. **Addressing post payment utilization review and downcoding issues**: This is again a practical concern, focusing on the fairness and efficiency of billing and payment processes, which doesn't inherently align with a specific ideological stance.<br>3. **Facilitating compliance with Medicare's documentation guidelines**: This suggests an effort to work within the existing framework of a government program (Medicare), which is a neutral action in the context of political ideologies.<br><br>In summary, these points are more about administrative efficiency and compliance in healthcare, and do not directly reflect liberal or conservative ideologies.	neutral
ACP	Solutions to the Challenges Facing Primary Care Medicine: Reimbursement: Provide Payment That Is Commensurate with the Value of Primary Care	2022	The text you've provided outlines specific policy recommendations regarding Medicare payments and the support for primary care physicians. Here's an analysis of its alignment with traditional liberal or conservative viewpoints:<br><br>1. **Advocating for Increased Medicare Payments and Support for Primary Care Physicians**: This aligns more closely with liberal viewpoints, which typically emphasize the expansion and strengthening of public healthcare systems, including Medicare. Liberals often advocate for increased government spending on healthcare to improve access and quality. The emphasis on raising compensation for primary care physicians and improving payment accuracy reflects a liberal approach to ensuring fair compensation and resource allocation in healthcare.<br><br>2. **Dedicated Federal Funding for Medicare Payments**: Proposing dedicated federal funding for Medicare payments also aligns with liberal perspectives. Liberals generally support the idea of federal involvement and investment in healthcare, viewing it as a public good that requires government support.<br><br>3. **Support for Patient-Centered Medical Home (PCMH) and Innovative Models**: This recommendation is somewhat bipartisan but leans towards liberal ideology. Liberals typically support innovative healthcare delivery models that emphasize patient-centered care, quality, and efficiency. The PCMH model, which focuses on coordinated care and long-term patient relationships, aligns with these principles.<br><br>4. **Encouraging Public and Private Payers to Support New Payment Models**: This point also shows a blend of ideologies but leans liberal. It suggests a role for both government and private entities in healthcare innovation, reflecting a liberal inclination towards collaborative approaches between public and private sectors to improve healthcare quality and efficiency.<br><br>Overall, the text aligns more with liberal viewpoints, particularly in its emphasis on government intervention and support for public healthcare systems, equitable compensation for healthcare providers, and innovative, quality-focused healthcare delivery models. However, the specific focus on market competitiveness and collaboration with private payers also incorporates elements that could be appealing across the political spectrum.	liberal

ACP	Advocating for Medicare Payment Rates for Internal Medicine Subspecialists Providing Primary Care	2022	The text you've provided outlines the American College of Physicians' (ACP) advocacy for the recognition and fair payment of services provided by primary care internal medicine specialists and subspecialists. This includes advocating for payment reforms that address inequities in payments for primary care and increasing Medicare payments for certain healthcare professionals.<br><br>This viewpoint aligns more closely with a liberal perspective, particularly in its emphasis on reforming healthcare payment systems to address inequities and ensure fair compensation for medical services. The focus on primary care and the equitable distribution of healthcare resources is consistent with liberal ideologies that prioritize social equality and support for systems that provide broader access to healthcare services.<br><br>However, it's important to note that while this viewpoint aligns with certain liberal principles, healthcare policy can be complex and multifaceted, and different aspects of it may be supported by individuals across the political spectrum for various reasons. Additionally, the ACP, as a professional organization, focuses primarily on the interests and needs of its members and the patients they serve, which may not neatly align with a specific political ideology.	liberal
ACP	Reforming Medicare: Adapting a Successful Program to Meet New Challenges	2017	The text you provided discusses various healthcare policies and strategies, particularly in the context of Medicare. It focuses on capitated or risk-sharing approaches, direct contracting with physician-run delivery systems, case management, bundled payment models, coordinated care, preventive care, private sector management approaches, Medicare coverage decisions for new technology, quality assurance, and payment rate revisions for Medicare HMOs.<br><br>This text does not align strictly with either a liberal or conservative viewpoint in the traditional sense of American politics. Instead, it addresses healthcare system efficiency and quality of care, emphasizing cost-effectiveness, direct physician engagement, comprehensive care for chronic and terminally ill patients, preventive care, and quality improvement. The proposals aim to improve Medicare by integrating more efficient payment systems (like capitated payments and bundled payments), fostering direct contracting, enhancing care coordination, and focusing on outcomes and quality standards.<br><br>Both liberal and conservative viewpoints might find elements they agree with in this text. Liberals might support the emphasis on comprehensive care, preventive services, and quality standards, which align with broader access and improved healthcare quality goals. Conservatives might appreciate the focus on cost-effectiveness, competitive bidding, and efficiency, which align with reducing government spending and increasing market-based solutions.<br><br>Overall, the text represents a pragmatic approach to healthcare reform, focusing on improving the Medicare system's efficiency and effectiveness, which can be seen as transcending traditional political divides.	neutral
ACP	Flu Vaccine	2017	The text regarding the American College of Physicians (ACP) petitioning the Centers for Medicare & Medicaid Services (CMS) to reimburse for the flu vaccine as clinically indicated or medically appropriate, rather than only every 12 months, doesn't strictly align with a traditional liberal or conservative viewpoint. Health policy, especially specifics like vaccination reimbursement policies, often transcends simple political categorizations.<br><br>Liberal viewpoints generally support more comprehensive healthcare coverage and may advocate for policies that increase access to healthcare services, including vaccinations. In this context, a liberal stance might favor broader reimbursement policies to ensure more people get vaccinated without facing financial barriers.<br><br>Conservative viewpoints typically emphasize personal responsibility and limited government intervention in healthcare. However, they also recognize the importance of public health measures. A conservative perspective might support this policy if it's seen as a cost-effective way to improve public health, but could be skeptical if it appears to increase government spending or bureaucracy.<br><br>In summary, the policy could be supported by both sides for different reasons: liberals for increasing healthcare access and conservatives for its potential public health benefits and cost-effectiveness. It's more about practical health policy than a clear ideological stance.	neutral

ACP	Medicare Carrier Contracts with CMS	2015	The text you've provided relates to a policy position regarding Medicare carrier contracts and the American College of Physicians (ACP). It's not straightforward to classify this text as either strictly liberal or conservative, as the context of healthcare policy often encompasses aspects that can be interpreted in different ways by different political ideologies.<br><br>In general, liberal viewpoints in the United States tend to support more government involvement in healthcare, aiming to ensure broader access to healthcare services. This includes supporting programs like Medicare and Medicaid. Conservatives, on the other hand, often advocate for less government intervention and more market-based solutions, focusing on personal responsibility and private sector options.<br><br>The specific policy position mentioned in your text — working to change Medicare carrier contracts to remove provisions that protect individual carriers from consequences of their actions — could be seen as aligning with liberal principles of holding corporations accountable and ensuring that government-run programs like Medicare are operated in the best interest of the beneficiaries. However, depending on the broader context and the specific details of what these changes entail, there could also be conservative arguments in favor, particularly if these changes are seen as a way to reduce bureaucratic inefficiencies or to improve market functioning within the Medicare system.<br><br>In conclusion, without more context about the motivations and implications of this policy position, it's challenging to definitively categorize it as liberal or conservative. Health policy often intersects with both ideological perspectives in various ways.	neutral
ACP	Timely Notification of Medicare Changes	2015	The text you've provided appears to be a policy statement from the American College of Physicians (ACP), urging the Centers for Medicare & Medicaid Services (CMS) to establish proper notice and comment periods for regulations and policies, and to notify practitioners about policy changes, especially when these changes might negatively affect coverage or payment.<br><br>This statement aligns with a viewpoint that emphasizes structured governance, transparency, and consideration for the impact of policy changes on healthcare practitioners. It doesn't neatly align with a strictly liberal or conservative viewpoint as these terms are commonly understood in the political context. Instead, it reflects concerns that are pertinent to the medical community and healthcare policy, which can be important to people across the political spectrum.<br><br>The focus on thorough regulatory processes and the impact of policy changes on professional practice can be seen as a non-partisan, professional stance rather than one rooted in a specific political ideology. It's more about advocating for the interests of healthcare practitioners and ensuring that changes in healthcare policy are made with adequate input and consideration of their potential impact.	neutral
ACP	Carrier Screens and Denial of Payment for Medically Necessary Visits	2015	The text you've provided seems to relate to a policy recommendation from the American College of Physicians (ACP) regarding Medicare payments. It suggests that screening should be used to flag cases for further review rather than automatically denying payments for covered services.<br><br>This statement doesn't neatly align with a traditional liberal or conservative viewpoint. Instead, it addresses a specific operational aspect of Medicare, which is a government-run health insurance program for the elderly and disabled in the United States.<br><br>- **Liberal Viewpoint**: Generally, liberals support a strong role for government in healthcare, advocating for comprehensive coverage and access to healthcare for all. They often favor measures that ensure fewer denials of coverage and support mechanisms that ensure equitable access to healthcare services.<br><br>- **Conservative Viewpoint**: Conservatives typically advocate for limited government intervention in healthcare and prefer market-based solutions. They might support measures that control costs and prevent abuse of the system, which can include strict criteria for coverage.<br><br>The ACP's recommendation aims to balance the need for oversight (to prevent misuse of Medicare funds) with the need to ensure that patients have access to covered services. This approach could be seen as trying to find a middle ground between ensuring fiscal responsibility (a concern more typically associated with conservative views) and providing comprehensive healthcare access (a concern more typically associated with liberal views).<br><br>In summary, this statement reflects a practical approach to healthcare policy that doesn't strictly adhere to a clear-cut liberal or conservative viewpoint. It's more focused on improving the functionality and fairness of an existing healthcare program.	neutral

ACP	Durable Goods Fraud	2015	The text you've provided, which states that the American College of Physicians (ACP) supports the certification of all Medicare/Medicaid durable medical equipment providers and the existence of a mechanism to report potential abuses in the marketing of these goods, doesn't neatly align with traditional liberal or conservative viewpoints in a straightforward manner. It touches on aspects of healthcare policy, particularly related to government-operated programs like Medicare and Medicaid.<br><br>1. **Liberal Viewpoint**: Generally, liberals in the United States tend to support stronger government regulation and oversight of healthcare and healthcare providers. The call for certification of providers and mechanisms to report abuses could be seen as aligning with this perspective, as it suggests a preference for structured oversight and regulation to ensure fairness and prevent abuse.<br><br>2. **Conservative Viewpoint**: On the other hand, conservatives typically advocate for less government intervention in business and healthcare, emphasizing free market solutions. However, conservatives also often support measures to prevent fraud and abuse in government programs. The focus on preventing abuses in the marketing of medical goods could be seen as a way to ensure that government funds are used effectively and efficiently, which aligns with conservative principles of fiscal responsibility.<br><br>In summary, the statement could be interpreted as aligning with certain aspects of both liberal and conservative viewpoints. It suggests regulation and oversight (more liberal) but within the context of ensuring efficiency and preventing abuse in government spending (which can also be a conservative concern).	neutral
ACP	Medicare Physician Time Survey	2015	The text you've provided appears to be a policy statement from the American College of Physicians (ACP) regarding the evaluation of time spent by physicians in Medicare Part A funded activities. It advocates for a modification in the process to make it less time and labor-intensive, thereby allowing physicians to spend more time on patient care while still providing reliable data to CMS (Centers for Medicare & Medicaid Services) and its intermediaries.<br><br>This statement doesn't align strictly with either a liberal or conservative viewpoint in the traditional sense. Instead, it focuses on practical and administrative aspects of healthcare delivery. The emphasis is on streamlining bureaucratic processes to improve efficiency and patient care, which could be supported by individuals across the political spectrum.<br><br>However, one might interpret elements of this statement through various political lenses:<br><br>1. **Liberal Viewpoint**: From a liberal perspective, this statement could be seen as advocating for improved healthcare efficiency and quality, aligning with a general emphasis on healthcare reform and accessibility.<br><br>2. **Conservative Viewpoint**: From a conservative viewpoint, the focus on reducing bureaucratic burden and potentially cutting down on government-related administrative overhead might be seen as a positive step towards efficiency and reduced government intervention in healthcare.<br><br>Ultimately, the interpretation can vary based on individual beliefs and the broader context of healthcare policies and politics.	neutral

ACP	Equity in Post-Payment Utilization Reviews	2015	The text you've provided outlines the American College of Physicians' (ACP) stance on specific policies related to post-payment review procedures in healthcare, particularly concerning Medicare carriers and physicians. To assess whether it aligns with a liberal or conservative viewpoint, we can analyze the key elements:<br><br>1. **Due Process and Right to Review Audit Sample**: Advocating for due process and the right for physicians to review audit samples aligns with principles of fairness and transparency in administrative processes. Both liberal and conservative viewpoints generally support due process rights, although the context might influence their emphasis.<br><br>2. **Publication of Regulations**: The support for the publication of regulations enforced by Medicare review personnel is a call for transparency and accountability in government operations. This stance can be seen in both liberal and conservative viewpoints, as transparency in government operations is a widely supported principle.<br><br>3. **Legislation to Limit Repayment Until Appeal Exhaustion**: This point advocates for the rights of physicians in the face of bureaucratic processes. Conservatives often emphasize the protection of individuals and businesses from what they perceive as overreach by government or large institutions. Liberals also support fair appeal processes but might focus more on the balance between individual rights and public welfare.<br><br>4. **Limitations on Annual Interest Rate and Reciprocal Interest Payment**: The proposal to limit the interest rate charged to physicians and to require reciprocal interest payments by carriers for errors suggests a focus on fairness and protection against potential financial exploitation. Both liberal and conservative viewpoints might support this, albeit for different reasons. Liberals might see it as a protection against corporate or institutional power, while conservatives might view it as a safeguard for individual financial rights.<br><br>Overall, the ACP's stance as outlined in your text does not strictly align with a liberal or conservative viewpoint. Instead, it combines elements of fairness, transparency, and protection of individual rights against institutional powers, which are values that can be found across the political spectrum. The focus here seems more on professional ethics and fair administrative practices within the healthcare system rather than on broader political ideologies.	neutral
ACP	Medicare Payment for Injectables	2015	The text regarding the American College of Physicians (ACP) supporting a fair and reasonable Medicare reimbursement policy for injectables aligns more closely with a liberal viewpoint. This stance emphasizes the importance of ensuring that healthcare providers are adequately compensated for their services, which includes covering actual costs and providing a reasonable administration fee. This perspective typically aligns with liberal values that prioritize equitable access to healthcare and support for the medical profession. Conservatives might support the idea of fair reimbursement but may have different views on the specifics of government involvement in setting these policies.	liberal
ACP	Prohibit States from Tampering with Federally Funded Health Programs in Which States Have Not Contributed	2015	The text you provided refers to the American College of Physicians' (ACP) support for Congressional legislation that would prohibit states from mandating Medicare assignment and reverse existing state laws mandating it. Analyzing the political alignment of this position can be complex, as it touches on several aspects of healthcare policy, state rights, and federal regulation.<br><br>1. **Liberal Viewpoint**: Generally, liberals in the United States advocate for increased government involvement in healthcare, including more robust federal programs like Medicare. However, the liberal viewpoint also often supports state rights in implementing healthcare policies. The ACP's stance against mandatory Medicare assignment by states might not align neatly with traditional liberal views, as it advocates for less state control over Medicare policies.<br><br>2. **Conservative Viewpoint**: Conservatives typically favor less government intervention in healthcare and more market-driven solutions. They also often support states' rights over federal control. In this context, the ACP's position might align more with conservative principles, as it opposes state mandates regarding Medicare assignment, advocating for less government intervention at the state level.<br><br>However, it's important to note that healthcare policy is a complex area where liberal and conservative viewpoints can overlap or diverge in unexpected ways. The ACP's stance might be driven more by practical considerations within healthcare policy than by a straightforward alignment with a traditional political ideology.	probably conservative

ACP	Explanation of Benefits	2015	The statement "ACP urges that Medicare carriers be required to publish accurate and updated lists of participating physicians at least annually" seems to be more aligned with a liberal viewpoint, primarily because it advocates for increased transparency and regulation in the healthcare system. Liberal ideologies often support the idea of government intervention to ensure fairness, accessibility, and transparency in healthcare.<br><br>Conservatives, on the other hand, typically favor less government involvement in healthcare, preferring market-driven solutions and reduced regulation. Thus, a conservative viewpoint might argue against such mandates on private Medicare carriers, seeing them as unnecessary government interference in the healthcare market.<br><br>However, it's important to note that healthcare policies can sometimes cross traditional political lines, and there could be conservatives who support such measures for reasons of efficiency or patient care standards. Similarly, some liberals might oppose it if they believe the measure doesn't go far enough in reforming healthcare.	liberal
ACP	Separation of Physician Services from Other Part B Medicare Services	2019	The text about the American College of Physicians (ACP) supporting the policy of separating physician services from other Part B expenditures for Medicare accounting purposes doesn't align explicitly with either a liberal or conservative viewpoint. It's more a statement of policy preference regarding the administration of Medicare, a U.S. federal health insurance program.<br><br>Medicare policy discussions often cross traditional political lines. Liberals generally advocate for expanded government involvement in healthcare, while conservatives typically support more market-based approaches. However, this particular policy stance by ACP focuses on the internal accounting and administrative practices within Medicare. It appears to be more about efficiency and clarity in financial management rather than a broader ideological stance on healthcare policy.<br><br>The policy could have implications that either side might support or oppose based on broader goals. For example, conservatives might favor this separation if it leads to more transparent and potentially reduced government spending. Liberals might support it if it results in more effective allocation of funds towards patient care. However, without additional context about the implications or goals of this policy, it's difficult to categorize it strictly as liberal or conservative.	neutral
ACP	Medical Review Programs	2015	The text provided represents a viewpoint that could be aligned with either liberal or conservative ideologies, depending on the specific aspects emphasized. It doesn't neatly fit into a strict political categorization because it addresses healthcare policy in a way that incorporates concerns common to both political spectra.<br><br>Key aspects of the text that might align with liberal viewpoints include:<br><br>1. **Emphasis on Collaboration with the Medical Profession:** Liberals often advocate for inclusive policy-making processes that involve stakeholders. The call for substantial input by the medical profession in developing utilization and quality review screens aligns with this approach.<br><br>2. **Focus on Quality and Necessity of Care:** Liberals tend to support measures that ensure high-quality healthcare services, as evidenced by the advocacy for medical review criteria that reflect broad medical consensus and allow for independent medical judgment.<br><br>Aspects that might resonate with conservative viewpoints include:<br><br>1. **Targeted Medical Review Programs:** Conservatives generally favor efficient government spending and might support the idea of targeted reviews to prevent waste in healthcare spending.<br><br>2. **Decreased Intrusion into Medical Practice:** The emphasis on decreasing unnecessary government intrusion into the practice of medicine aligns with the conservative principle of limited government intervention in private sectors, including healthcare.<br><br>Overall, the text seems to strive for a balanced approach, advocating for efficient, quality healthcare delivery with input from medical professionals, which could find support across the political spectrum.	neutral

ACP	Physician Diagnostic Related Groups (DRGs)	2015	The text you've provided expresses a viewpoint on healthcare policy, specifically related to physician payment systems in the context of Medicare. It does not neatly align with traditional liberal or conservative viewpoints as understood in a broad political sense. Instead, it reflects specific policy preferences and concerns within the healthcare industry.<br><br>The text argues against a physician Diagnosis-Related Group (DRG) system for Medicare, voicing concerns that it might undermine the quality of care, create conflicts within medical staff, and lead to adversarial relationships between physicians and hospitals. It also opposes mandatory assignment for physicians and favors a resource cost relative value scale (RVS) as a means to establish prices for physician services.<br><br>These positions are more about the specifics of healthcare administration and payment models than about broader political ideologies. They reflect a preference for a certain type of payment structure within the healthcare system, rather than a clear liberal or conservative stance. In healthcare policy, both liberal and conservative viewpoints can encompass a range of opinions on payment models, with concerns varying based on the impacts on quality of care, efficiency, and cost-control.<br><br>Therefore, it's more accurate to say that this text represents a specific stance within healthcare policy debates rather than a broader political ideology.	neutral
ACP	Quality Care	2015	The text you provided expresses a viewpoint that aligns more closely with liberal ideologies. The emphasis on preserving Medicare as an entitlement program, especially for vulnerable populations like the elderly, disabled, and individuals with end-stage renal disease, and the focus on providing cost-effective, quality health care, are positions typically supported by liberal viewpoints. Liberals generally advocate for the role of government in ensuring access to healthcare for all, especially the most vulnerable, and are supportive of programs like Medicare.<br><br>In contrast, conservative viewpoints often emphasize reduced government intervention in healthcare, favoring market-driven solutions and personal responsibility. While conservatives may support certain aspects of Medicare, they often advocate for reforms to reduce government spending and involvement in healthcare.<br><br>It's important to note, however, that these are general trends and there can be a wide range of opinions within each ideological spectrum.	liberal
ACP	Update of Geographic Practice Cost Index	2022	The statement about working with other parties to sponsor legislation that updates the Medicare Geographic Practice Cost Index (GPCI) and allows for future updates to occur on a regularly scheduled basis appears to be focused on healthcare policy rather than explicitly aligning with a liberal or conservative viewpoint. This approach suggests a pragmatic stance towards healthcare administration, aiming to improve and modernize the system.<br><br>Liberal and conservative viewpoints often differ in their approaches to healthcare, with liberals typically advocating for more government involvement and conservatives favoring less. However, the specific proposal to update the GPCI for Medicare reflects a more technical and administrative aspect of healthcare policy. It doesn't inherently lean towards a traditionally liberal or conservative stance but rather indicates an interest in ensuring the healthcare system operates effectively and fairly, potentially benefitting healthcare providers and recipients in various geographic areas.<br><br>This perspective is more about administrative efficiency and fairness in healthcare delivery rather than a broader ideological stance.	neutral

ACP	Legislate Coverage for Preventive Benefits	2022	The text you provided outlines a series of policy proposals related to Medicare, focusing on preventive health benefits, the "Welcome to Medicare" examination, and the roles of various healthcare providers. This text can be analyzed from a political viewpoint to determine if it aligns more closely with liberal or conservative ideologies.<br><br>1. **Emphasis on Preventive Healthcare**: The focus on preventive healthcare, including improvements to the "Welcome to Medicare" examination and promoting separate payments for Medicare-covered preventive services, aligns more with liberal viewpoints. Liberals typically advocate for comprehensive healthcare policies that emphasize prevention and access to a broad range of healthcare services.<br><br>2. **Role of Government in Healthcare**: The proposals suggest an active role for government in healthcare, specifically in Medicare, which is more consistent with liberal ideologies. Liberals generally support a stronger government role in ensuring healthcare access and regulating healthcare services.<br><br>3. **Support for Non-Physician Healthcare Providers**: The emphasis on the roles of nurse practitioners and physician assistants and exploring payment for their services also aligns with a more liberal perspective. This viewpoint often includes support for a diverse range of healthcare professionals and an integrated approach to healthcare delivery.<br><br>4. **Medicare Regulations and Reimbursement**: The focus on clarifying Medicare rules and advocating for adequate reimbursement levels reflects concerns about the effectiveness and efficiency of government healthcare programs, a common theme in liberal policy discussions.<br><br>However, it's important to note that these issues can also be viewed through a conservative lens, especially when it comes to efficient use of government resources and the specifics of how these policies are implemented. While the overall emphasis on preventive healthcare and the role of government in healthcare aligns more closely with liberal viewpoints, certain aspects of the proposals, like the focus on clear regulations and efficient use of Medicare funds, can also resonate with conservative values of efficient government spending and clear, streamlined regulations.<br><br>Therefore, while the text generally aligns more with liberal viewpoints due to its emphasis on preventive healthcare and an active government role in healthcare, elements of it can also be interpreted in a way that aligns with conservative principles.	liberal

ACP	Reforming Medicare in the Age of Deficit Reduction	2023	The text you provided outlines various policy recommendations concerning the Medicare program in the United States. Analyzing its alignment with liberal or conservative viewpoints requires understanding the core principles of these ideologies:<br><br>1. **Liberal Viewpoint**: Generally, liberals advocate for government intervention in healthcare to ensure universal access and affordability. They often support policies that expand public healthcare programs and regulate healthcare costs.<br><br>2. **Conservative Viewpoint**: Conservatives typically favor less government involvement in healthcare, arguing for market-based solutions and personal responsibility. They often oppose extensive government control over healthcare, preferring private sector solutions.<br><br>Now, let's analyze the provided text in this context:<br><br>1. **Promotion of New Care Models, Patient-Centered Care, and Technology**: Advocating for innovation in healthcare and promoting patient-centered models aligns more with a liberal viewpoint, as it involves active government role in shaping healthcare delivery.<br><br>2. **Support for Medical Home Models and Bundled Payments**: These models, which focus on coordinated care and efficiency, align with both liberal and conservative principles. Liberals may support them for their potential to improve care and efficiency, while conservatives may see them as a way to control costs and improve market efficiency.<br><br>3. **Opposition to Premium Support Model Conversion**: This opposition aligns with a liberal viewpoint, as liberals typically support the existing structure of Medicare and are cautious about shifting to models that might resemble privatization.<br><br>4. **Support for Adjusting Medicare Advantage Funding and Increasing Eligibility Age**: These points reflect a blend. Adjusting Medicare Advantage funding is more liberal, focusing on parity with traditional Medicare. Increasing the eligibility age could be seen as a conservative approach to fiscal sustainability.<br><br>5. **Incremental Increase in Premiums for Wealthier Beneficiaries**: This progressive approach to funding Medicare is more in line with liberal ideology, which often supports higher contributions from wealthier individuals.<br><br>6. **Redesigning Benefits Based on Value and Clinical Effectiveness**: This idea could appeal to both sides. Liberals might support it for ensuring value and effectiveness in healthcare spending, while conservatives might see it as a way to make healthcare delivery more efficient and cost-effective.<br><br>7. **Combining Medicare Parts A and B with Protections for Beneficiaries**: This proposal could align with both ideologies but leans more towards liberal principles due to its focus on beneficiary protections and evidence-based exemptions.<br><br>8. **Changes to Medigap and Support for Palliative and Hospice Care**: These recommendations are more aligned with liberal views, focusing on comprehensive care and protecting beneficiaries from high costs.<br><br>9. **Reducing Costs in Medicare Part D**: The proposal for the federal government to act as a negotiator for drug prices is a classic liberal policy stance, advocating for government intervention to control costs.<br><br>In summary, the text predominantly aligns with liberal viewpoints due to its emphasis on government intervention, protection of beneficiaries, and focus on value and efficiency within a government-led healthcare framework. However, some elements, like bundled payments and market efficiency measures, could also resonate with conservative principles.	liberal

ACP	Extrapolation Technique in Postpayment Review	2015	The text you've provided doesn't straightforwardly align with either a liberal or conservative viewpoint in the traditional sense of U.S. politics. It addresses a specific policy issue related to Medicare, specifically urging the Centers for Medicare & Medicaid Services (CMS) and Congress to mandate that Medicare carriers provide data to justify the statistical validity of their extrapolation findings before requesting the return of payments made to physicians.<br><br>This statement appears to be more focused on healthcare policy, transparency, and fairness in the Medicare system, rather than aligning with broader ideological principles typically associated with liberalism or conservatism. Both liberals and conservatives might support or oppose this position based on their views on healthcare regulation, government oversight, and fiscal responsibility.<br><br>Liberal viewpoints generally favor more government involvement in healthcare and might support such measures for ensuring fairness and transparency. Conversely, conservatives typically advocate for less government intervention but might support this policy as a measure for fiscal accountability and preventing potential misuse of funds. However, without more context about the underlying motivations or implications of this policy, it's challenging to definitively classify it as liberal or conservative.	neutral
ACP	Medicare Contractor Reviews of Medical Necessity for Hospital Inpatient Admissions Supported by Evidence- Based Medicine	2022	The text you've provided appears to be a policy statement from the American College of Physicians (ACP). It emphasizes the importance of evidence-based medicine and physician judgment in Medicare Contractor reviews for hospital inpatient admissions. The statement advocates for transparency in the processes used by Medicare Contractors for determinations or denials of medical necessity and insists on physician reviews for denied admissions based on commercial criteria.<br><br>This text doesn't align strictly with either a liberal or conservative viewpoint in the traditional political sense. Instead, it reflects a professional and ethical stance focused on medical practice and healthcare policy. The principles of evidence-based medicine, transparency, and physician involvement in healthcare decisions are not inherently political but are more related to ensuring quality and fairness in healthcare practices.<br><br>This perspective is more about advocating for best practices in healthcare administration rather than supporting a particular political ideology. It underlines the importance of medical expertise and evidence in decision-making processes, which is a common viewpoint across various healthcare organizations and professionals, regardless of their political leanings.	neutral
ACP	Peer Review and Expunging Remote Peer Citations	2017	The text you've provided relates to a policy request by the American College of Physicians (ACP) regarding the handling of quality citations for physicians. It requests that the Centers for Medicare & Medicaid Services (CMS) mandate Quality Improvement Organizations (QIOs) to have a uniform policy allowing physicians to expunge quality citations over five years old if no subsequent citations have occurred.<br><br>This request doesn't neatly align with traditional liberal or conservative viewpoints in politics. Instead, it focuses on healthcare administration and physician management. Such policy recommendations often transcend simple political divides and are more concerned with practical, administrative, or professional aspects of healthcare services.<br><br>Liberal and conservative viewpoints in the U.S. typically differ on broader healthcare issues like the extent of government involvement in healthcare, healthcare financing, and access to healthcare services. However, the specifics of administrative policies like the one mentioned are usually more technical and less ideological. They can be supported or opposed by individuals across the political spectrum depending on their views on healthcare administration, professional autonomy, and regulatory oversight.	neutral

ACP	Physician Involvement in Quality Improvement Organizations (QIOs)	2017	The text you provided, which discusses the American College of Physicians (ACP) encouraging internists to become actively involved with Quality Improvement Organizations (QIOs) and supporting physician-directed QIOs under the Centers for Medicare & Medicaid Services, doesn't align distinctly with either a liberal or conservative viewpoint in the traditional political sense.<br><br>Healthcare policy, especially around quality improvement and Medicare, often intersects with both liberal and conservative agendas, but in different ways. For instance:<br><br>- **Liberal Viewpoint**: Generally, liberals support more government involvement in healthcare, advocating for broader access and quality improvement through governmental programs. In this context, ACP's encouragement for physician involvement in QIOs could be seen as aligning with liberal values of improving healthcare quality and efficacy through structured, government-facilitated programs.<br><br>- **Conservative Viewpoint**: On the other hand, conservatives often support the idea of physician-led initiatives and less bureaucratic interference in healthcare. The call for "physician-directed QIOs" might align with conservative values emphasizing professional autonomy and less direct government control over healthcare practices.<br><br>However, this statement is primarily professional and technical, focusing on the involvement of medical professionals in quality improvement processes. It's more about the operational aspect of healthcare management rather than a direct endorsement of a specific political ideology. It reflects the ACP's stance on how quality improvement should be managed in the healthcare system, which can intersect with broader political views but is not explicitly political in itself.	neutral
ACP	Quality Improvement Organization (QIO) Accountability	2015	The statement provided appears to align more closely with a viewpoint that emphasizes transparency, accountability, and effective use of public funds. This perspective can be associated with both liberal and conservative ideologies, depending on the context.<br><br>1. **Advocacy for Transparency and Accountability**: The call for state-by-state review of costs and results to be a matter of public record is a stance that advocates for transparency. This is a principle that can be embraced by both liberal and conservative viewpoints, as it deals with ensuring that governmental actions and expenditures are open to public scrutiny.<br><br>2. **Demand for Evidence of Effectiveness**: The requirement for the Centers for Medicare & Medicaid Services (CMS) to document ongoing effectiveness of Quality Improvement Organizations (QIOs) before receiving additional budget funding suggests a demand for evidence-based decision-making. This approach is often supported by both political spectrums, although the specific areas of focus might vary.<br><br>3. **Fiscal Responsibility**: The emphasis on reviewing and justifying budget allocations before providing additional funding is a principle commonly associated with fiscal conservatism. However, it can also align with liberal perspectives that prioritize efficient and effective use of public funds, especially in healthcare.<br><br>4. **Healthcare Context**: The reference to healthcare, specifically the CMS and QIOs, adds a layer of complexity. Healthcare policy views vary widely within both liberal and conservative circles, with different groups emphasizing various aspects like universal coverage, cost containment, quality of care, and private versus public roles in healthcare.<br><br>In summary, the statement reflects principles of transparency, accountability, and evidence-based policy making, which are values that can be found across the political spectrum. The specific alignment might depend on the broader context of the healthcare policies and the ideologies of the groups involved.	neutral

ACP	Quality Improvement Organization (QIO) Intervention to be Educational	2015	The text you provided suggests a viewpoint that aligns more with liberal perspectives, particularly in the context of healthcare and professional regulation. This viewpoint emphasizes education and improvement in professional conduct as primary responses to issues in clinical practice, rather than immediate punitive measures.<br><br>Liberal viewpoints often prioritize rehabilitation, education, and systemic improvements over punitive actions. The idea of first resorting to educational intervention aligns with these principles, suggesting an approach that seeks to improve and correct rather than immediately punish.<br><br>Conservative viewpoints, on the other hand, might lean more towards immediate accountability and may be more supportive of direct punitive measures like fiscal sanctions or licensing actions in response to professional misconduct or incompetence.<br><br>It's important to note, however, that these categorizations can be overly simplistic. Views on such specific policy matters can vary widely within political ideologies, and individuals or groups may hold positions that do not neatly align with broad ideological labels.	liberal
ACP	Quality Improvement Organization (QIO) Demerit Program	2015	The text you've provided seems to align more with a viewpoint focused on healthcare quality and transparency, rather than fitting neatly into a typical liberal or conservative political stance. It discusses the American College of Physicians' (ACP) beliefs regarding the responsibilities of Quality Improvement Organizations (QIOs) and Peer Review Organizations (PROs) in notifying physicians about quality problems and disseminating information while maintaining confidentiality.<br><br>This viewpoint emphasizes accountability and transparency in healthcare, which can be supported by individuals across the political spectrum. The focus is on improving healthcare quality and ensuring that physicians are informed and engaged in addressing quality issues. This approach is more about practical healthcare management and less about the broader, more divisive issues that typically distinguish liberal and conservative viewpoints.	neutral
ACP	Quality Improvement Organization (QIO) Guidelines	2015	The text you've provided seems to be a policy statement from the American College of Physicians (ACP). It emphasizes that any adverse decision from a Quality Improvement Organization (QIO) should be based on objective evidence, possibly including references to standard medical and surgical literature.<br><br>This statement aligns more with a principle of evidence-based practice in medicine rather than a specific political ideology. Both liberal and conservative viewpoints generally support the use of evidence-based approaches in healthcare, though they might differ in their approaches to healthcare policy overall.<br><br>The emphasis on objective evidence and standard medical literature is a hallmark of modern medical practice, which strives to base clinical decisions on the best available evidence. This approach is neutral and doesn't inherently align with either liberal or conservative ideologies, as it's primarily focused on ensuring the best patient outcomes based on empirical evidence.	neutral
ACP	Quality Improvement Organization (QIO) Reimbursement for Physicians	2015	The statement "ACP recommends that, under QIO reimbursement principles, physicians should be adequately compensated for medical review and administrative services" doesn't distinctly align with either a liberal or conservative viewpoint in a straightforward manner. The American College of Physicians (ACP) is advocating for adequate compensation for physicians for their medical review and administrative services, which is more of a professional stance than a political one.<br><br>However, the way this issue is interpreted can vary based on political viewpoints:<br><br>1. **Liberal Viewpoint**: Liberals might support this statement as part of a broader agenda for fair labor practices and ensuring that professionals are compensated appropriately for their work. They might also view it as a step towards improving healthcare systems and ensuring that medical professionals are not overburdened or underpaid.<br><br>2. **Conservative Viewpoint**: Conservatives might agree with this statement from the perspective of market-driven compensation and the importance of valuing professional work. They might also see it as a matter of individual rights where professionals should be compensated fairly for their services.<br><br>Overall, the statement is more aligned with professional ethics and healthcare administration principles rather than being distinctly political. Both liberals and conservatives might find aspects to agree with, depending on their broader views on healthcare, labor rights, and market economics.	neutral

ACP	National Practitioner Data Bank	2015	The text you've provided outlines the American College of Physicians' (ACP) stance on certain legislative matters related to the National Practitioner Data Bank. Classifying this stance as strictly liberal or conservative is challenging because it involves nuanced positions on medical regulation, physician privacy, and legal reporting standards, which don't neatly align with a traditional political spectrum.<br><br>- **Physician Privacy and Due Process**: ACP advocates for due process and privacy rights for physicians, requiring court action or medical licensing jurisdiction intervention before a physician is reported to the National Practitioner Data Bank. This could be seen as a conservative viewpoint insofar as it emphasizes individual rights and protections against potentially overreaching regulations.<br><br>- **Opposition to Unauthorized Data Sharing**: ACP's opposition to unauthorized agencies requiring physicians to turn over their Data Bank reports could be interpreted as a stance for privacy and against regulatory overreach, aligning with more conservative principles of limited government intervention.<br><br>- **Support for Thresholds on Reporting**: Advocating for a minimum threshold on reporting settlements or liability awards against physicians suggests a desire to protect professionals from potentially frivolous or minor claims. This stance might resonate more with conservative views that often emphasize the need to protect professionals from excessive legal burdens.<br><br>- **Evaluation of Data Bank's Effectiveness and Confidentiality**: This position indicates a desire for oversight and efficiency in government operations, a concern that can be found across the political spectrum.<br><br>- **Opposition to Additional Data Reporting Requirements**: Opposing additional reporting requirements could be seen as a conservative viewpoint favoring less regulation.<br><br>However, these positions also reflect a focus on professional standards, oversight, and accountability, concerns that are not exclusive to any single political ideology. In contemporary politics, issues of privacy, regulatory oversight, and professional standards cross traditional liberal-conservative boundaries, making a straightforward classification difficult.	neutral
ACP	National Data Bank Reporting	2015	The statement regarding the American College of Physicians' (ACP) support for the American Medical Association's (AMA) proposed $30,000 minimum floor for reporting medical malpractice settlements to the National Practitioner Data Bank does not clearly align with a strictly liberal or conservative viewpoint. The stance on this issue is more related to healthcare policy and professional practice standards than to the traditional liberal-conservative political spectrum.<br><br>In the context of U.S. politics:<br><br>- **Liberal Viewpoint**: Generally focuses on advocating for consumer protections, access to healthcare, and sometimes greater regulation of industries, including the healthcare sector.<br><br>- **Conservative Viewpoint**: Often emphasizes free market principles, personal responsibility, and less government intervention in business, including in the healthcare industry.<br><br>The proposal in question relates to the threshold for reporting medical malpractice settlements. This is a specific issue within the healthcare sector that can be interpreted differently depending on one's viewpoint on healthcare regulation, professional accountability, and patient rights. It doesn't neatly fit into the liberal-conservative spectrum as it involves considerations of both professional self-regulation (more a conservative approach) and transparency in healthcare practices (which can align with liberal principles of consumer protection).<br><br>It's important to understand that many policy issues, especially those involving professional standards and industry-specific regulations, don't always align clearly with broad political ideologies.	neutral

ACP	Public Access to the National Practitioner Data Bank	2015	The positions outlined in the American College of Physicians (ACP) statement you provided don't align strictly with either a traditional liberal or conservative viewpoint. Instead, they reflect a nuanced approach to healthcare policy, balancing the need for transparency, patient protection, and the rights of healthcare professionals. Here's a breakdown:<br><br>1. **Opposition to Opening Malpractice Claims Information**: This stance can be seen as more conservative, as it aligns with the protection of physician privacy and the avoidance of public scrutiny based on possibly misleading or incomplete data.<br><br>2. **Support for Access to Information on Finalized Adverse Actions**: This is a more liberal stance, advocating for transparency and consumer protection in healthcare.<br><br>3. **Evaluating Public Release of Physician Information**: This position indicates a balanced approach, seeking to protect both consumers and healthcare providers' reputations. It doesn't clearly align with a traditional political stance but rather reflects a pragmatic, patient-centered approach.<br><br>4. **Support for Tort Reform Legislation**: The support for tort reform is typically a conservative viewpoint, as it often involves limiting litigation against healthcare providers and can be seen as a way to control healthcare costs and protect practitioners.<br><br>Overall, the ACP's positions reflect a complex interplay of concerns related to patient rights, healthcare transparency, and the protection of medical practitioners. They seem to prioritize a balanced approach over strictly adhering to traditional liberal or conservative doctrines.	neutral
ACP	Statement of Principles on the Role of Governments in Regulating the Patient-Physician Relationship.	2012	The text you provided reflects principles recommended by the American College of Physicians (ACP) regarding the roles of federal and state governments in health care and the patient-physician relationship. It does not align strictly with either a liberal or conservative viewpoint as traditionally defined in U.S. politics, but rather focuses on ethical and practical considerations in medical practice and healthcare policy.<br><br>Here are some key aspects of the text and their potential alignment with broader political viewpoints:<br><br>1. **Emphasis on the Patient-Physician Relationship**: The text stresses the importance of the patient-physician relationship and ethical obligations like beneficence and confidentiality. This perspective aligns with a patient-centered approach to healthcare, which is a principle that can be supported by both liberal and conservative viewpoints, albeit with different approaches to implementation.<br><br>2. **Opposition to Overregulation in Healthcare**: The caution against laws or regulations that excessively dictate the content of physician-patient interactions or mandate specific medical treatments aligns more with a conservative viewpoint, which typically favors less government intervention in private matters, including healthcare.<br><br>3. **Flexibility in Medical Practice**: The emphasis on the need for medical practice to be guided by current scientific evidence and the flexibility to adapt to individual patient circumstances can be seen as a centrist approach, valuing both the expertise of medical professionals (a more liberal value) and the limitation of government mandates (a more conservative value).<br><br>4. **Inclusivity in Treatment**: The stance against legislation that prevents physicians from treating certain types of patients (e.g., based on immigration status, race, or sexual orientation) aligns with liberal values that emphasize inclusivity and equality.<br><br>5. **Evidence-Based Decision Making**: The focus on evidence-based medical practice and the role of clinical guidelines reflects a technocratic approach, where decisions are based on scientific evidence rather than political ideology. This is a principle that can be endorsed by both sides but is more often associated with liberal perspectives that emphasize the role of science in policymaking.<br><br>6. **Adaptability of Laws and Regulations**: The recommendation that laws governing medical practice must be revised as needed and the call for a process for timely appeal reflects a pragmatic approach, valuing adaptability and responsiveness in healthcare policy.<br><br>In summary, the text presents a nuanced viewpoint that incorporates elements typically associated with both liberal and conservative perspectives, focusing on patient-centered care, evidence-based practice, and a balanced approach to regulation. It doesn't neatly fit into a single political category but instead reflects a more multidimensional approach to healthcare policy.	neutral

ACP	Sexual Contact between Physician and Patient	2019	The text you provided appears to be a statement or guideline regarding the ethics of physician-patient relationships, specifically addressing the issues of sexual relationships between physicians and their current or former patients. It does not directly align with a liberal or conservative viewpoint in the traditional political sense. Instead, it is focused on ethical considerations in the medical profession.<br><br>The statement emphasizes the importance of maintaining professional boundaries in the physician-patient relationship due to the inherent power imbalance and the patient's vulnerability. It outlines the ethical concerns with both current and former patient-physician relationships, focusing on issues like dependency, trust, transference, and the exploitation of these elements in a sexual context.<br><br>In general, discussions about professional ethics and boundaries in healthcare are more about maintaining high standards of conduct within the profession and ensuring patient safety and trust, rather than being about political ideology.	neutral
ACP	Medical Risk to Physician and Patient	2019	The text you've provided outlines the ethical obligations of physicians in treating infectious diseases and managing risks associated with their profession. It doesn't align directly with either a liberal or conservative viewpoint in the political sense. Instead, it focuses on medical ethics and professional responsibilities.<br><br>This text emphasizes the duty of physicians to treat all patients in need, regardless of the risks to themselves, and to take appropriate precautions to minimize the spread of infections. It also discusses the obligations of physicians to be tested and to quarantine themselves if necessary, to disclose their condition if there's a significant risk to patients, and to comply with public health policies.<br><br>The principles outlined in this text are grounded in the ethics of the medical profession rather than in political ideology. They reflect a commitment to patient care, public health, and professional responsibility, aspects that are central to medical ethics irrespective of political alignment.	neutral
ACP	The Physician and the Patient	2019	The text you provided reflects a viewpoint focused on medical ethics and professionalism, emphasizing the physician's duty to prioritize patient welfare and interests. This perspective transcends traditional liberal or conservative political ideologies, as it is rooted in the principles of medical ethics and patient care.<br><br>Such principles are generally accepted across the political spectrum, as they emphasize the moral and professional obligations of physicians to serve their patients' best interests, regardless of external factors like financial arrangements or patient characteristics. This viewpoint aligns with the universal values of medical practice, which prioritize patient welfare, confidentiality, and the responsible use of medical expertise.<br><br>In summary, the text aligns more with universally accepted medical ethical principles rather than being distinctly liberal or conservative in nature.	neutral
ACP	Initiating and Discontinuing the Patient-Physician Relationship	2019	The text you provided does not align strictly with either a liberal or conservative viewpoint as it primarily focuses on the ethical and professional standards of the patient-physician relationship. It emphasizes mutual understanding, effective communication, respect for patient autonomy, and the importance of professional competence and responsibility in medical care. These principles are generally accepted across the political spectrum and are fundamental to medical ethics, rather than being indicative of a particular political ideology.<br><br>Key aspects include:<br><br>- The importance of understanding the patient's health problems, values, and expectations.<br>- Mutual obligations in the patient-physician relationship.<br>- The necessity of professional competence, responsible conduct, and compassion in medical practice.<br>- The role of effective communication and the need to overcome barriers like health literacy issues.<br>- Ethical considerations in telemedicine and the establishment of a patient-physician relationship.<br>- The moral obligation of physicians to provide care for ill persons and not to discriminate.<br>- Recognition of different cultural beliefs and values in medical care.<br>- The physician's responsibility to serve the best interests of the patient and the ethical considerations in terminating the physician-patient relationship.	neutral

			These principles are foundational to medical practice and ethics, transcending political divisions by focusing on the welfare and rights of patients and the responsibilities of healthcare professionals.	
ACP	Patient-Physician Covenant	2017	The text you've provided expresses a viewpoint centered around the ethical and moral responsibilities of physicians and the medical profession. It doesn't align strictly with a liberal or conservative viewpoint as it focuses on the principles of medical ethics and professionalism. These principles, such as the commitment to patient welfare, competence, advocacy, and ethical medical care, are universal in the field of medicine and transcend political ideologies. Instead, they represent a consensus within the medical community about the foundational values and commitments that guide medical practice. Both liberal and conservative perspectives might agree on the importance of these ethical standards, although they might differ in their approach to healthcare policy and funding.	neutral
ACP	Controlling Health Care Costs: Encourage Cost-Consciousness and Patient Involvement in Shared Decision- Making	2022	The text you've provided outlines principles and recommendations for health care practices and policies. It doesn't align strictly with either a liberal or conservative viewpoint as it incorporates elements that could be appealing to both sides. Here's a breakdown:<br><br>1. **Encouraging patient cost-consciousness and responsibility**: This may resonate more with conservative ideals, which often emphasize individual responsibility and cost efficiency in healthcare.<br><br>2. **Price transparency from providers**: This is a cross-partisan issue that has support from both liberals and conservatives, as it promotes transparency and can lead to more informed consumer choices.<br><br>3. **Shared decision-making and informed patient choices**: This principle is generally supported across the political spectrum. It aligns with liberal values of patient rights and empowerment, and conservative values of individual autonomy.<br><br>4. **Payment for shared decision-making**: Advocating for additional payments to physicians for time spent on shared decision-making could be seen as supportive of healthcare providers, a stance that doesn't align strictly with traditional liberal or conservative views.<br><br>5. **Medicare demonstration projects for shared decision-making**: The focus on Medicare might lean more liberal, as it suggests an active role for a government program in healthcare innovation, but the concept of testing and implementing decision-making tools can appeal to conservatives interested in healthcare efficiency and innovation.<br><br>6. **Advance planning and value-based care**: Both sides generally support the idea of care that respects a patient's values and wishes, though the mention of not withholding care due to costs may align more with liberal healthcare principles which emphasize access and equity.<br><br>7. **Research and incentives for quality of life and palliative care**: This point may have broad appeal, as improving quality of life and support for terminally ill patients is a universal concern, though the method of providing incentives could be a point of debate between more market-driven (conservative) and government-supported (liberal) approaches.<br><br>In summary, the text represents a blend of principles that encourage personal responsibility, transparency, patient empowerment,	neutral

			and quality care. These ideas cross traditional political boundaries and could be supported by individuals across the political spectrum for different reasons. It reflects a nuanced approach to healthcare policy that doesn't neatly fit into a single ideological category.	
ACP	Unsolicited Communications	2016	The text describes the American College of Physicians' (ACP) opposition to unsolicited communications or "cold calling" related to pharmaceuticals, medical equipment, and healthcare services. It expresses concerns about inappropriate treatment, interference with patient-clinician relationships, increased healthcare costs, and legal issues. This position aligns more closely with a liberal viewpoint, which typically emphasizes consumer protection, the regulation of business practices to safeguard public health, and the importance of maintaining the integrity of the patient-clinician relationship. Liberals often advocate for measures that prevent companies from engaging in practices that might prioritize profit over patient welfare. Conservatives, on the other hand, might emphasize market freedom and be more likely to support fewer restrictions on business communications. However, views on specific healthcare policies can vary widely within each political spectrum, and some conservatives might also support such regulations due to concerns about ethical medical practices and patient rights.	liberal
ACP	Second Opinions	2017	The text you've provided seems to be a position statement from the American College of Physicians (ACP) regarding internists' roles in second opinion programs. It doesn't explicitly align with a liberal or conservative viewpoint as it's primarily focused on healthcare policy and professional standards rather than broader political ideologies. Support for the involvement of internists in second opinion programs can be seen as a stance on improving healthcare quality and patient care, which are concerns that cut across political lines. Both liberals and conservatives might agree or disagree with this position based on their views on healthcare administration, professional autonomy, and the role of specialists in medical decision-making. Therefore, it's more of a professional or industry-specific stance rather than a strictly political one.	neutral

ACP	Voluntary Physician-Directed Peer Review	2015	The text from the American College of Physicians (ACP) emphasizes the importance of voluntary, physician-directed peer review programs aimed at maintaining high-quality medical care. It supports local autonomy in these programs while prioritizing the quality of care over cost considerations. The ACP encourages using quality assurance activities and professional education to achieve high-quality, cost-effective medical care, acknowledging the limited ability of such programs to contain costs alone.<br><br>This statement aligns with aspects of both liberal and conservative viewpoints:<br><br>1. **Liberal Viewpoint**:<br>- **Emphasis on Quality of Care**: Liberals often prioritize access to high-quality healthcare and might support the emphasis on maintaining high standards over cost-cutting measures.<br>- **Support for Professional Autonomy and Education**: The liberal perspective might appreciate the focus on professional education and autonomy, seeing it as a means to ensure that healthcare decisions are made by those with the most expertise rather than being driven solely by economic considerations.<br><br>2. **Conservative Viewpoint**:<br>- **Local Autonomy**: Conservatives typically favor local control and might agree with the idea that peer review programs should maintain local autonomy, resisting centralized, national criteria that could be seen as overreach.<br>- **Voluntary Participation**: The conservative viewpoint often emphasizes personal responsibility and might favor the voluntary nature of these programs, as opposed to mandatory government-led initiatives.<br><br>Overall, the statement seems to blend principles that can be appreciated from both sides of the political spectrum. It doesn't strictly align with one over the other but rather incorporates values like quality, professional autonomy, and local control, which are points of importance to both liberals and conservatives in different contexts.	neutral
ACP	The Role of Performance Assessment in a Reformed Health Care System	2019	The text primarily aligns with a liberal viewpoint, emphasizing comprehensive healthcare reform, quality improvement, and equitable access to care. It advocates for payment and delivery system reforms that promote high-value care, patient-centered approaches, and system-based collaboration, aligning with liberal ideals of comprehensive healthcare reform and social equity. The focus on integrating reforms into patient-centered medical homes, engaging physicians in program development, and emphasizing preventive health care reflects liberal values of participatory governance and proactive healthcare management.<br><br>The text also supports financial incentives and penalties linked to performance to drive behavior change, reflecting a liberal approach to using policy and economic tools for societal benefit. The emphasis on fair compensation for physicians, attention to reducing health disparities, and the inclusion of socioeconomic adjustments in performance measures further align with liberal concerns for social justice and equity.<br><br>Moreover, the call for transparency, stakeholder collaboration, and continuous improvement in healthcare quality and delivery demonstrates a progressive stance on healthcare reform, focusing on adaptability, inclusivity, and evidence-based practices. Overall, the text's comprehensive approach to healthcare reform, focus on equity and quality, and emphasis on collaborative and evidence-based solutions are more closely aligned with liberal ideologies.	liberal

ACP	A System in Need of Change: Restructuring Payment Policies to Support Patient-Centered Care	2017	The positions outlined focus on health care reform, emphasizing patient-centered care, quality improvement, efficiency, and comprehensive payment restructuring. They don't align strictly with traditional liberal or conservative viewpoints as they encompass aspects that both sides might support or contest. Here's a breakdown:<br><br>1. **Liberal Viewpoint Tendencies**:<br>- Emphasis on comprehensive, coordinated care and patient-centered approaches.<br>- Support for increased payments and resources for primary care, which could be seen as expanding access and improving care quality.<br>- Advocacy for risk-adjusted, prospective payments might appeal to those favoring reforms aimed at equality and comprehensive care.<br><br>2. **Conservative Viewpoint Tendencies**:<br>- Recommendations for reforming payment structures to incentivize quality and efficiency can resonate with market-oriented approaches to healthcare.<br>- The call for pilot projects and incremental implementation might align with a conservative preference for cautious, tested policy changes rather than broad, sweeping reforms.<br><br>3. **Overlap and Ambiguity**:<br>- Both liberals and conservatives might agree on the importance of improving healthcare quality and efficiency, though they might disagree on methods.<br>- The focus on technology and innovation can appeal across the political spectrum.<br>- Both sides might support or critique the specific financial incentives and payment models based on broader fiscal and regulatory philosophies.<br><br>In summary, these positions blend elements that could be supported by both liberals and conservatives, depending on the broader context of their healthcare and fiscal policies. The focus on improving care quality, efficiency, and patient-centered approaches, alongside detailed payment restructuring, reflects a nuanced approach that doesn't neatly fit into a single ideological category.	neutral
ACP	Reform of the Dysfunctional Healthcare Payment and Delivery System	2017	The text you've provided is a set of recommendations and positions focused on the valuation of physician services and Medicare reforms. It doesn't explicitly align with a liberal or conservative viewpoint as it primarily addresses technical and administrative aspects of healthcare policy rather than ideological principles. However, the underlying values and implications of these recommendations can be interpreted through different ideological lenses:<br><br>### Liberal Viewpoint Considerations:<br>- **Focus on Quality and Accessibility:** Liberals often advocate for improved healthcare quality and accessibility, which aligns with the text's recommendations for coordinated care and technology use.<br>- **Regulatory Oversight:** The call for more structured and evidence-based valuation of physician services might resonate with a liberal preference for regulatory oversight to ensure fairness and efficiency in healthcare.<br><br>### Conservative Viewpoint Considerations:<br>- **Market-Based Reforms:** Conservatives might support the ideas of re-examining methodologies and ensuring that payments reflect true resource costs, which can be seen as a push for more market-based approaches.<br>- **Efficiency and Accountability:** Calls for better processes, review mechanisms, and modifying payment systems to reward quality and efficiency might appeal to conservative ideals of accountability and fiscal responsibility.<br><br>### Neutral Aspects:<br>- Many of the positions focus on technical reforms to improve the efficiency and accuracy of Medicare payments, which is a non-partisan administrative goal.<br>- The emphasis on evidence-based practice and technology integration is broadly supported across the political spectrum for improving healthcare outcomes.<br><br>### Conclusion:<br>The text is more about practical healthcare policy reforms than ideological arguments. While different aspects might resonate more with one political leaning over another, the document as a whole doesn't clearly align with a singular liberal or conservative	neutral

			viewpoint. It's focused on pragmatic solutions to improve Medicare and healthcare delivery, which can be a concern for people across the political spectrum.	
ACP	Controlling Health Care Costs: Ensure Accurate Pricing of Services	2023	The text discusses ensuring the accuracy of relative value determinations in Medicare, focusing on identifying potentially undervalued and overvalued services and recommending revisions. This is a policy-oriented statement rather than an ideological one. However, it can be interpreted through different political lenses:<br><br>1. **Liberal viewpoint**: Liberals might support this statement as it aligns with the idea of improving and expanding healthcare services, ensuring fairness, and making healthcare accessible and equitable. They often advocate for stronger regulations and oversight to ensure that healthcare systems work effectively and fairly.<br><br>2. **Conservative viewpoint**: Conservatives might agree with the aspect of ensuring accuracy and efficiency in government spending on healthcare, as they generally favor efficient use of resources and might support measures that prevent wasteful spending. However, they might be skeptical of increased government intervention in healthcare markets, preferring market-based solutions.<br><br>Ultimately, the statement is more about good governance and efficient healthcare policy than about liberal or conservative ideologies. Both sides might find aspects to agree with, depending on their priorities and how they interpret the role of government in healthcare.	neutral

ACP	Composition of the Relative Value Update Committee	2019	The text you've provided seems to be a policy statement related to the composition of the Relative Value Update Committee (RUC), advocating for better representation for physicians who provide primarily cognitive services. This statement doesn't neatly align with traditional liberal or conservative viewpoints as it's more focused on healthcare policy and professional representation rather than broader political ideologies.<br><br>In the U.S. political context:<br>- **Liberal viewpoints** generally advocate for broader access to healthcare, increased regulation of healthcare industries, and support for public healthcare options.<br>- **Conservative viewpoints** often emphasize market-based solutions, less regulation, and private healthcare options.<br><br>This statement is about representation within a specific committee, which could be supported by either side for different reasons:<br>- **A liberal** might support this as part of broader efforts to improve healthcare outcomes and ensure diverse professional perspectives are considered in policy-making, possibly believing that better representation leads to more equitable and comprehensive healthcare policies.<br>- **A conservative** might agree with this statement from a standpoint of professional autonomy, fairness, and efficiency, or a belief in the importance of specialized expertise in decision-making processes.<br><br>Ultimately, without more context about the implications of the RUC's composition or the specific concerns of cognitive service physicians, it's difficult to categorize the statement definitively as liberal or conservative. It reflects concerns specific to the medical profession and healthcare policy, areas where there is a wide range of opinions within both ideological spectrums.	neutral
ACP	CMS Contracts with Professional Review Organizations	2022	The statement from the American College of Physicians (ACP) regarding opposition to performance-based contract requirements creating inappropriate incentives for payment error identification is generally neutral and focused on professional standards and practices within healthcare. It doesn't align strictly with a liberal or conservative viewpoint as it is primarily concerned with the ethics and efficacy of medical billing practices. Both liberal and conservative perspectives might have different reasons to support or oppose such policies based on broader considerations of healthcare management, financial incentives, and patient care standards. Therefore, without additional context or related policy proposals, it's not appropriate to categorize this statement as strictly liberal or conservative. It's more aligned with professional healthcare policy and ethics.	neutral
ACP	Billing for Contracted Diagnostic Services	2022	The statement you've provided reflects a policy position on healthcare billing, particularly around the idea that billing for contracted diagnostic services should be limited to the actual service cost plus a reasonable fee for additional services. This concept might align with various political viewpoints depending on the broader context and the reasons for supporting it.<br><br>**Liberal Viewpoint:**<br>- Liberals might support this statement as it can be seen as a way to prevent excessive healthcare costs and ensure fair billing practices. It aligns with the liberal focus on consumer protection and access to affordable healthcare.<br>- They might argue that it helps to regulate the healthcare market, preventing providers from charging exorbitant prices and thereby making healthcare more accessible.<br><br>**Conservative Viewpoint:**<br>- Conservatives might also agree with this statement, especially if they view it as a measure that promotes transparency and fair competition in the healthcare market.<br>- They might support the idea of limiting billing to reasonable costs as a way to encourage efficiency and cost-effectiveness in healthcare, principles that align with fiscal conservatism.<br><br>However, the stance any individual or group might take on this policy can vary widely based on their specific beliefs and priorities about healthcare, government regulation, market dynamics, and personal responsibility. It's also possible that some liberals might oppose it if they believe it doesn't go far enough in regulating healthcare costs, while some conservatives might oppose it if they see it as too much government intervention in the market. Therefore, while the statement can resonate with both liberal and conservative values in different ways, it doesn't neatly fit into one category without more context.	neutral

ACP	Principles on Payment for Physician Services	2023	The text you've provided outlines the American College of Physicians' (ACP) policies and positions on various healthcare issues, focusing on utilization guidelines, peer review, data development, discussion of fees, compensation for services, and patient cost-sharing among other topics. It doesn't explicitly align with a liberal or conservative viewpoint as these terms are broadly defined and can vary greatly depending on specific issues and contexts. However, the content can be analyzed for themes that might resonate more with one political ideology or another.<br><br>**Liberal Viewpoints:**<br>- The emphasis on developing valid utilization guidelines and data systems for improving healthcare efficiency and effectiveness might align with a more liberal approach, focusing on evidence-based policies to improve quality and access to care.<br>- Advocating for physicians to provide services to uninsured and underinsured persons and to participate in pluralistic payment systems could be seen as aligning with liberal values of inclusivity and a broad approach to healthcare access.<br><br>**Conservative Viewpoints:**<br>- The emphasis on peer review and professional autonomy in setting guidelines and reviewing care may resonate with conservative values of limited government intervention and respect for professional expertise.<br>- Encouraging patient cost-sharing and opposing payment through hospital administration could be seen as supporting individual responsibility and market-based solutions, which are often conservative principles.<br><br>Overall, the document seems to strive for a balance between ensuring quality, professional autonomy, and efficiency in healthcare services, which can be points of interest for both liberal and conservative ideologies. It doesn't strictly adhere to one side but rather presents a nuanced approach that reflects the complexity of healthcare policy.	neutral
ACP	Physician Billing for Services Related to Lab Work	2015	The text you've provided outlines a stance focused on promoting fair compensation for work related to laboratory tests, specimen collection, and handling, as well as seeking better reimbursement for test interpretation and related evaluation and management services. Here's a breakdown of how different political ideologies might view this text:<br><br>### Liberal Viewpoint:<br>- **Agreement with Fair Compensation:** Liberals generally support measures that ensure workers are fairly compensated for their labor. The focus on true overhead costs and fair reimbursement aligns with liberal ideals of worker rights and fair pay.<br>- **Support for Healthcare Workers:** Liberals often advocate for the rights and fair compensation of healthcare workers, seeing it as a part of broader healthcare reform and social justice issues.<br><br>### Conservative Viewpoint:<br>- **Market-Driven Solutions:** Conservatives might agree with the idea of fair compensation but prefer market-driven solutions over regulated or mandated increases in reimbursement. They might support reforms if they believe these lead to more efficient and cost-effective healthcare.<br>- **Small Government Approach:** Some conservatives might be wary of any approach that appears to increase government involvement in private industries, including healthcare. They might prefer less regulation and more competition as the means to achieve fair compensation.<br><br>### Neutral Aspects:<br>- The text is primarily technical and focuses on specific changes without advocating for a broad political ideology.<br>- Both liberals and conservatives might agree on the importance of fair compensation but differ on how it should be achieved.<br><br>### Conclusion:<br>The statement doesn't neatly align with a strictly liberal or conservative viewpoint. It could be seen as aligning with liberal ideals regarding fair worker compensation and healthcare reform but also fits into conservative principles of fair market compensation and efficiency in healthcare. The political alignment might depend more on the broader context of how these improvements are proposed to be implemented and the specific beliefs of individuals within the liberal and conservative spectrums.	neutral

ACP	Principles of Payment for Physicians Services	2015	The text you've provided outlines principles for transparency and communication between physicians and patients regarding fees and insurance participation. This viewpoint doesn't neatly align with a traditional liberal or conservative perspective as it touches on aspects valued by both sides:<br><br>1. **Transparency and Patient Empowerment:** The emphasis on physicians volunteering fee information and discussing costs upfront aligns with a broader desire for transparency in healthcare, a principle that can be endorsed by both liberal and conservative viewpoints. Liberals might appreciate the empowerment this gives patients in making informed healthcare decisions, while conservatives might value the market-like transparency and the potential for informed choice to drive competition and efficiency.<br><br>2. **Personal Responsibility and Free Market Principles:** The idea that patients should be informed about whether a physician participates in their insurance plan before elective procedures could be seen as supporting personal responsibility and free market principles, which are typically more conservative values. It suggests that patients have a role in managing their healthcare costs and choices, akin to making informed decisions in a market.<br><br>3. **Consideration for Financial Hardship:** The mention of physicians making arrangements in cases of financial need may resonate more with liberal principles, which often emphasize the need for equity and support for those in challenging circumstances.<br><br>Ultimately, the text seems to advocate for ethical practices and patient-physician communication rather than pushing a specific political agenda. It represents a blend of principles like transparency, personal responsibility, and compassion that are valued to varying degrees across the political spectrum.	neutral
ACP	Principles on Which Reimbursement Plans Should be Based	2015	The text you've provided outlines principles believed by the American College of Physicians (ACP) regarding physician reimbursement. It doesn't neatly align with a traditional liberal or conservative viewpoint as it touches on aspects that could be interpreted as aligning with both sides.<br><br>From a conservative perspective, the emphasis on physicians' rights to set their own fees, participate or decline in insurance plans, and the use of peer review might resonate with values of individual autonomy, free-market principles, and less government intervention in business practices.<br><br>On the other hand, the call for third-party payers to recognize the value of cognitive services and the involvement of physicians in developing fee allowances could align with a more liberal viewpoint that advocates for fair compensation and a more collaborative, regulated approach to healthcare pricing.<br><br>It's also important to note that healthcare policy is complex and often transcends simple political categorization. Both liberals and conservatives might find aspects of the ACP's principles that they agree or disagree with based on their broader views on healthcare economics, the role of government, and the rights of individual practitioners and patients.	neutral
ACP	Resource Costs as the Basis for Determining Charges and Allowances	2016	The statement from the American College of Physicians (ACP) regarding allowances for cognitive, procedural, and technical services being based on resource costs reflects a viewpoint that is primarily pragmatic and focused on fairness and efficiency in healthcare compensation. It doesn't neatly align with traditional liberal or conservative ideologies. Instead, it emphasizes rational, cost-based approaches to compensation for medical services.<br><br>Liberals might appreciate the emphasis on ensuring that healthcare providers are fairly compensated for their time and resources, potentially leading to better overall healthcare quality. This view might align with broader liberal values of ensuring fairness and support for professionals in public services.<br><br>Conservatives might appreciate the market-based approach to compensation, where payments are based on the specific resources and costs involved in providing services. This can be seen as a way to ensure efficiency and prevent overpayment, aligning with conservative values of fiscal responsibility and limited intervention.<br><br>In summary, the statement is primarily focused on practical, sector-specific policy rather than broader ideological principles, and it incorporates values that can be appreciated by both liberal and conservative viewpoints.	neutral

ACP	Increasing RVU for E/M Codes	2016	The text you've provided is from a policy statement by the American College of Physicians (ACP). It doesn't inherently align with a liberal or conservative viewpoint as it focuses on practical aspects of healthcare reimbursement rather than ideological ones. The statement advocates for recognizing and reimbursing medical services provided outside of regular hours, which is a matter of healthcare policy and economics.<br><br>Liberal and conservative perspectives might differ in their approach to healthcare reform and payment models, but the statement itself is more about ensuring fair compensation for physicians. A liberal viewpoint might focus on how this impacts access to healthcare and fair wages, while a conservative viewpoint might consider the effects on business operations and overall healthcare costs. The text is primarily professional and technical, aiming to address practical challenges in healthcare service delivery without an evident political stance.	neutral
ACP	Technical Procedures--Third Party Coverage	2015	The text you've provided expresses a viewpoint on healthcare policy, particularly regarding third-party coverage and reimbursement for technical medical procedures. It advocates for non-discriminatory coverage based on the setting and the specialty of the physician, as long as the setting is medically appropriate. Additionally, it calls for equal reimbursement for procedures performed in ambulatory or outpatient settings compared to inpatient settings.<br><br>This viewpoint doesn't neatly align with traditional liberal or conservative ideologies as it touches on aspects that might be supported or contested by both sides. Here's a brief breakdown of how each side might view it:<br><br>- **Liberal Viewpoint**: Liberals generally advocate for comprehensive healthcare coverage and might support the idea of non-discriminatory coverage and equal reimbursement as a means to ensure that all individuals have access to necessary medical procedures, regardless of where they are performed. They might view these policies as steps toward a more equitable healthcare system.<br><br>- **Conservative Viewpoint**: Conservatives typically emphasize the importance of market forces and personal responsibility in healthcare. They might support the idea of non-discriminatory coverage insofar as it promotes competition and efficiency among providers. However, some conservatives might be wary of mandates on reimbursement rates, viewing them as government overreach into the healthcare market.<br><br>It's important to note that these are generalizations and the actual viewpoints can vary widely among individuals and specific groups within each ideology. Additionally, healthcare policy is complex and can intersect with other issues, leading to a variety of stances within both liberal and conservative circles.	neutral
ACP	Monthly Capitated Payment for Dialysis Services	2015	The text provided appears to advocate for policy changes to better reflect the costs of providing dialysis services. It doesn't align strictly with liberal or conservative viewpoints as both sides might support or oppose such measures based on different ideologies. Liberals might support it as part of healthcare reform to ensure fair pricing and access, while conservatives might back it if they see it as a way to make healthcare more market-efficient and reduce unnecessary spending. The alignment really depends on the broader context of the policy and the specific arguments each side presents.	neutral

ACP	Smoking Cessation Counseling	2015	The text you've provided appears to be advocating for policy changes related to healthcare reimbursement, particularly concerning smoking cessation counseling and the recognition of tobacco abuse as a medically necessary diagnosis. Here's how it might align with typical liberal or conservative viewpoints:<br><br>### Liberal Viewpoint:<br>- **Support for Healthcare Reform:** Liberals often advocate for comprehensive healthcare reforms, including increased coverage for various treatments and preventive measures. The push for recognizing diagnostic codes for tobacco abuse and ensuring payment for smoking cessation counseling aligns with the liberal emphasis on preventive care and broader healthcare access.<br>- **Regulation and Oversight:** Liberals generally support more substantial regulation and oversight in healthcare, including measures that ensure patients receive necessary care without prohibitive costs. Advocating for third-party payers to reimburse physicians for smoking cessation efforts fits within this perspective.<br><br>### Conservative Viewpoint:<br>- **Market-Driven Solutions:** Conservatives typically favor market-driven solutions and may be skeptical of increased regulation in healthcare. They might support the idea of smoking cessation counseling but prefer solutions that don't involve mandates on third-party payers or expanded definitions of medical necessity.<br>- **Limited Government Intervention:** Conservatives often advocate for limited government intervention in healthcare. They might argue that while helping patients stop smoking is important, it should not be mandated or regulated extensively by government entities.<br><br>### Conclusion:<br>The text leans more towards a liberal viewpoint due to its emphasis on regulatory advocacy and ensuring healthcare coverage for specific treatments. It reflects a desire for systemic change in how healthcare providers and third-party payers approach smoking cessation, which is more in line with liberal healthcare reform ideals. However, perspectives on these issues can vary widely within each political ideology, and some conservatives might also support these measures as part of a public health initiative.	liberal
ACP	Guidelines on Appropriate Use of the Telephone for Diagnosis and Treatment of Patients	2019	The text seems to align with a pragmatic approach rather than strictly liberal or conservative viewpoints. It focuses on the efficiency and practicality of telephone-based professional services in healthcare, advocating for their recognition and reimbursement by insurance providers, including Medicare and Medicaid. This stance emphasizes the importance of accessibility and efficiency in healthcare services, which can resonate with both liberal ideals of expanded access to healthcare and conservative principles of cost-effective care and individual choice.<br><br>The text argues for uniform billing codes and reimbursement policies, suggesting a systematic approach to healthcare services delivered over the phone. This idea might be seen as appealing to liberals for its potential to improve access to care and to conservatives for its emphasis on fair compensation for medical professionals and efficient healthcare delivery. Overall, the text presents a balanced view, prioritizing effective healthcare delivery and fair compensation rather than aligning with a specific political ideology.	neutral
ACP	Reimbursement for Two Procedures Performed on the Same Day	2015	The text you've provided reflects a policy position from the American College of Physicians (ACP) regarding the reimbursement of medical evaluations and related services. It does not inherently align with a liberal or conservative viewpoint, as it is a specific stance on healthcare billing practices. The liberal-conservative spectrum typically involves broader ideologies and policies. However, perspectives on healthcare reimbursement can sometimes intersect with these ideologies. For example:<br><br>- **Liberal Viewpoint:** Might emphasize the need for comprehensive healthcare coverage and might support policies that ensure services are adequately reimbursed, advocating for patient access over cost-saving measures.<br>- **Conservative Viewpoint:** Could focus on cost containment, fiscal responsibility, and the role of private markets in healthcare, possibly favoring policies that reduce federal spending.<br><br>The ACP's position seems focused on ensuring that physicians are fairly compensated for their services, a stance that doesn't neatly fit into a liberal or conservative framework but can be supported or contested by either side based on broader healthcare policy views. Interpretation would depend on the broader context of healthcare policy and fiscal responsibility discussions within these ideologies.	neutral

ACP	Case Management Services	2015	The statement suggests that the American College of Physicians (ACP) believes in recognizing and reimbursing physicians for case management services by Congress, the Centers for Medicare & Medicaid Services (CMS), and the insurance industry. This viewpoint can align with aspects of both liberal and conservative ideologies, depending on the context and the broader beliefs of each group regarding healthcare.<br><br>1. **Liberal Viewpoint**: Liberals generally support increased government involvement in healthcare and may favor policies that ensure fair compensation for medical professionals, improve patient care, and expand access to healthcare services. From a liberal perspective, advocating for reimbursement for case management could be seen as a step toward more comprehensive and accessible healthcare.<br><br>2. **Conservative Viewpoint**: Conservatives typically advocate for limited government intervention and might be skeptical of increasing government spending or mandates. However, they might support reimbursement for case management if they believe it will lead to more efficient healthcare delivery, reduce overall costs, or improve the quality of care without expanding government control.<br><br>In essence, the alignment of this statement with liberal or conservative viewpoints depends on the specific arguments and values each side emphasizes regarding healthcare policy. Some liberals might see it as a way to improve healthcare quality and access, while some conservatives might view it as a method to enhance efficiency and reduce unnecessary expenses in the healthcare system.	neutral
ACP	Prayer Fees Reimbursed as a Medical Expense	2015	The text you've provided appears to be a position statement from a professional medical organization, the American College of Physicians (ACP), regarding the use of prayer as a form of therapy. This statement expresses a view that prayer should not delay access to traditional medical care, should not be reimbursable by third parties as a medical therapy, and should not be considered a tax-deductible medical expense.<br><br>This position does not neatly align with traditional liberal or conservative viewpoints as understood in the context of U.S. politics. Instead, it reflects a medical and ethical stance based on the principles of evidence-based medicine and the separation of religious practices from medical treatment. Here's how different sides might view it:<br><br>- **Liberal Viewpoint:** Liberals might agree with this statement as it aligns with a secular, evidence-based approach to healthcare and supports the idea that medical treatment should be based on scientific evidence and best practices.<br><br>- **Conservative Viewpoint:** Conservatives might have mixed views. Some might agree with the emphasis on traditional medical care and the concern about third-party reimbursements. Others, particularly those with strong religious convictions, might view prayer as a vital component of health and well-being and disagree with its exclusion from recognized medical therapy and reimbursement.<br><br>It's important to note that within both liberal and conservative circles, there's a wide range of beliefs about the role of religion in healthcare and the extent to which healthcare should be regulated, which can affect how this statement is perceived. Therefore, while the statement reflects a specific ethical and professional stance, its alignment with political ideologies can vary greatly among individuals.	neutral

ACP	Computerized Electrocardiograms (EKGs)	2015	The text you've provided appears to be a policy or guideline statement on the appropriate billing practices for physicians interpreting electrocardiograms (EKGs), whether those are computer-assisted or not. It doesn't explicitly align with a liberal or conservative viewpoint as it's more focused on professional standards and ethics within the medical community. It suggests that physicians should charge fairly for their services based on their expertise and the usual charges within their community, emphasizing the physician's ultimate responsibility for patient care.<br><br>In the context of political viewpoints:<br><br>- **Liberal Viewpoint**: A liberal perspective might focus on the accessibility and affordability of healthcare, advocating for regulations to ensure that charges for medical services are fair and not prohibitive for patients. They might also support the use of technology to improve healthcare outcomes but would insist on equitable access to such technology.<br><br>- **Conservative Viewpoint**: A conservative perspective might emphasize the free market's role in determining prices, including the right of professionals to set their fees based on their skills and experience. They might also stress individual responsibility and the importance of minimal regulatory interference in professional practices.<br><br>The statement seems to balance these perspectives by acknowledging the physician's right to be compensated for their expertise while also implying a need for those charges to be fair and in line with community standards. It doesn't strongly advocate for a specific political ideology but rather focuses on professional and ethical considerations.	neutral
ACP	Disability Determination Reports	2015	The text describes a policy stance from the American College of Physicians (ACP) regarding the expenses incurred by physicians when providing reports on a patient's physical status for disability determination by governmental agencies. It suggests that physicians may charge patients for these reports if the requesting agency doesn't provide payment.<br><br>This position doesn't align strictly with liberal or conservative viewpoints as traditionally defined. Instead, it addresses a practical aspect of healthcare administration and physician compensation. However, different aspects might resonate differently with various political ideologies:<br><br>- **Liberal viewpoint**: Might focus on the patient's right to healthcare and disability support, emphasizing the need for government or insurance to cover such costs to prevent additional financial burdens on patients.<br><br>- **Conservative viewpoint**: Might appreciate the emphasis on personal responsibility and the discretion given to physicians to manage their financial affairs, aligning with free-market principles and less government intervention in business practices.<br><br>In reality, views on such specific policy matters can vary widely within each ideological group, often influenced by broader beliefs about healthcare, government's role, and market regulation. Therefore, while the text doesn't strictly align with one political ideology, different aspects of it might be supported or opposed by individuals across the political spectrum.	neutral
ACP	Patient Counseling	2015	The statement "ACP supports greater recognition and adequate reimbursement for extended and complex counseling" seems to align with a viewpoint that emphasizes healthcare access and provider support. This position could be associated with either liberal or conservative viewpoints, depending on the broader context and specific policies proposed to achieve these goals.<br><br>Liberal viewpoints typically emphasize expanding access to healthcare, improving quality, and supporting healthcare providers through adequate reimbursement and recognition. They often support government intervention and policies that aim to ensure everyone has access to comprehensive healthcare services, including mental health and counseling.<br><br>Conservative viewpoints, on the other hand, might also support adequate reimbursement and recognition for healthcare providers but often emphasize market-based solutions, reduced government intervention, and personal responsibility in healthcare. They might advocate for these changes within the context of broader healthcare system reform that reduces regulatory burdens and increases competition.<br><br>In the absence of more context about the specific policies ACP (presumably the American College of Physicians) is advocating, it's challenging to definitively categorize the statement as strictly liberal or conservative. It's essential to consider the broader policy framework and goals associated with the statement to understand its alignment fully.	neutral

ACP	Patient Education	2019	The text you've provided appears to be a policy statement from the American College of Physicians (ACP) emphasizing the importance of patient education in medical care. This statement doesn't align strictly with a liberal or conservative viewpoint but rather addresses healthcare quality and patient engagement, which are concerns across the political spectrum. Here are some considerations:<br><br>1. **Education and Patient Empowerment**: Emphasizing patient education and behavioral change is generally a non-partisan issue, focusing on improving health outcomes and patient autonomy. Both liberal and conservative perspectives might agree on the importance of educating patients, though they might differ on implementation strategies or funding.<br><br>2. **Role of Physicians and Supplementary Methods**: The text suggests a team approach to healthcare, involving physicians and possibly other healthcare providers in patient education. This collaborative approach is a standard practice in medical care, not inherently political.<br><br>3. **Record-keeping and Compensation**: The mention of recording educational exposures in medical records and potentially recognizing patient education as a compensable part of services could be viewed through a political lens. Conservatives might focus on the aspects of personal responsibility and the efficient use of resources, while liberals might emphasize the right to comprehensive healthcare, including education. However, the statement itself doesn't advocate for a specific political solution.<br><br>4. **Peer Review**: The call for local peer review when the appropriateness of charges is questioned suggests a measure to ensure accountability and quality in healthcare services. This concept could be supported by both ends of the political spectrum, as it emphasizes professional standards and accountability.<br><br>In summary, the statement focuses on quality care and the role of patient education, topics that are generally viewed as essential across the political spectrum. While different political groups might have varying opinions on how best to implement and fund such education, the statement itself does not strongly advocate for a liberal or conservative viewpoint.	neutral
ACP	Payment for Services Not Requested by Attending Physician	2015	The statement you provided focuses on the ethical practice of billing in healthcare, specifically stating that physicians, including hospital-based specialists, should not bill patients for consultative or other medical services unless those services were requested by the attending physician. This viewpoint primarily centers on medical ethics and the appropriate conduct of billing practices rather than a direct liberal or conservative political stance. It emphasizes the protection of patients from potentially unnecessary or unauthorized charges, promoting transparency and fairness in healthcare.<br><br>In the broader context, discussions about healthcare practices and billing can be influenced by liberal or conservative viewpoints, with liberals typically advocating for more comprehensive healthcare coverage and regulations to protect patients, while conservatives might focus on free market solutions and personal responsibility. However, this specific statement is more about ethical standards and professional conduct within the medical community rather than a direct expression of political ideology. It's a stance that individuals from either side of the political spectrum might agree on due to its focus on fairness and patient protection.	neutral
ACP	Solo Practitioners--Payment Schedules	2015	The statement from the American College of Physicians (ACP) urging insurance carriers and the Centers for Medicare & Medicaid Services not to discriminate against solo practitioners in payment schedules doesn't neatly align with a traditional liberal or conservative viewpoint. Instead, it focuses on healthcare policy and fairness within the medical profession. Both liberals and conservatives might support or oppose this statement based on their broader views on healthcare regulation, the role of government in healthcare, market dynamics, and support for small practices versus larger healthcare entities. It's more specific to the healthcare industry's regulatory and payment systems rather than being inherently political.	neutral
ACP	Comprehensive Payment Reform: Reforming Physician Paymentsto Achieve Greater Value in Health Care Spending	2022	The text doesn't align strictly with a traditional liberal or conservative viewpoint as it focuses on healthcare policy and reform, specifically regarding payment models for physician services. The approach advocates for efficiency, effectiveness, and patient-centered care, emphasizing the value of primary care and the need for a system that rewards quality and coordination over volume. Such ideas might find support across the political spectrum, as they address concerns about healthcare costs, quality, and access, which are universal issues. However, the emphasis on regulated reforms and structured payment models might be more aligned with liberal views that generally favor structured government interventions in healthcare to ensure fairness and efficiency. At the same time, the focus on incentives, market sensitivity, and the need for sustainable and politically viable solutions could also resonate with conservative principles that emphasize market-based solutions and fiscal responsibility. Therefore, this text represents a blend of ideas that could potentially appeal to or be contested by both sides, depending on specific ideological stances and how the proposals are framed within broader healthcare debates.	neutral

ACP	Mandating Reimbursements for Periodic Health Promotion Visits	2019	The text outlines support for third-party payer recognition and payment for health promotion visits, coverage of tests ordered during such visits, and an educational program on coding for these visits. This perspective tends to align more with a liberal viewpoint for several reasons:<br><br>1. **Advocacy for Comprehensive Coverage**: The emphasis on ensuring third-party payers (like insurance companies) recognize and cover periodic health promotion visits and associated tests reflects a liberal viewpoint, which often advocates for comprehensive healthcare coverage and preventive care measures to improve overall health outcomes and reduce long-term costs.<br><br>2. **Support for Regulation and Legislation**: The call for legislation and regulation to ensure coverage and payment is typical of liberal perspectives that support a more active governmental role in healthcare to ensure that individuals receive necessary medical services without financial barriers.<br><br>3. **Focus on Prevention and Education**: The focus on age-appropriate screening, prevention, and counseling, as well as the implementation of an educational program, aligns with liberal views that prioritize preventive care and informed healthcare decisions as means to improve public health and reduce healthcare expenses over time.<br><br>However, it's important to note that while these points lean more towards a liberal perspective, healthcare policy is complex, and there are conservatives who also support various forms of preventive care and healthcare education. The distinction often lies in the mechanisms for implementation and the extent of government involvement preferred.	liberal
ACP	The Patient Centered Medical Home Neighbor	2022	The text you've provided primarily focuses on the guidelines and principles for effective collaboration and coordination between primary care practices and specialty/subspecialty practices within the Patient-Centered Medical Home (PCMH) and Patient-Centered Medical Home Neighbor (PCMH-N) models. It does not explicitly align with a liberal or conservative viewpoint as it's largely technical and policy-focused, dealing with healthcare management and quality improvement rather than broader ideological issues.<br><br>However, various aspects of this text might resonate differently with liberal and conservative viewpoints:<br><br>1. **Liberal Viewpoint**: Liberals generally support comprehensive, coordinated, and accessible healthcare and might view the emphasis on patient-centered care, coordination, and quality as positive steps toward a more equitable and effective healthcare system. The focus on collaboration and integrated care might align with liberal values of community-oriented solutions and comprehensive welfare.<br><br>2. **Conservative Viewpoint**: Conservatives might appreciate the text's emphasis on improving efficiency and effectiveness in healthcare delivery. The focus on clear agreements, accountability, and the potential exploration of nonfinancial and financial incentives might resonate with conservative values of personal responsibility, market-driven solutions, and smaller government involvement.<br><br>The text itself is neutral and technical, aiming to improve healthcare delivery rather than promote a political ideology. It's more about the practicalities of healthcare management and less about the broader philosophical or political debates surrounding healthcare policy. As such, interpretations of its alignment with political viewpoints might vary based on individual perspectives and priorities.	neutral
ACP	Gender Pay Gap within the Field of Medicine	2016	The text you've provided appears to advocate for equal pay for physicians regardless of gender and calls for transparency and further study on gender pay inequity. These points generally align with a liberal viewpoint, which typically emphasizes equality, fairness, and the need to address systemic issues such as gender pay gaps. Liberal ideologies often advocate for policies that promote equal opportunities and seek to understand and rectify societal inequalities.<br><br>Conservatives might support the idea of equal pay for equal work as a matter of fairness and meritocracy but may have different views on the mechanisms for achieving this, such as the degree of government intervention or regulation needed. They might also question the extent of the pay gap issue, its causes, or the best methods to study and address it.<br><br>It's important to note that while these views tend to align with general liberal or conservative ideologies, there is a wide range of beliefs within each group, and individuals may hold different or nuanced positions on these issues.	neutral

ACP	Reimbursement for Concurrent Care	2015	The statement you've provided reflects a perspective on healthcare policy and reimbursement, specifically regarding concurrent care provided by medical subspecialists and its reimbursement by Medicare. Here's how it might align with liberal or conservative viewpoints:<br><br>### Liberal Viewpoint:<br>- **Support for Regulation and Oversight**: Liberals typically advocate for regulated and equitable healthcare systems. The call for clear definitions and guidelines by a national body (CMS) aligns with liberal ideals of ensuring fairness and consistency in healthcare.<br>- **Advocacy for Comprehensive Care**: The emphasis on medically necessary concurrent care suggests a focus on comprehensive patient care, a common liberal priority.<br><br>### Conservative Viewpoint:<br>- **Concerns Over Government Involvement**: Conservatives might be wary of increased government intervention in healthcare, as suggested by the call for CMS to direct Medicare carriers. They often prefer market-based solutions and local control.<br>- **Efficiency and Cost**: However, the desire for a "nationally uniform reimbursement definition" could resonate with conservatives seeking efficiency and cost containment in healthcare.<br><br>### Neutral Aspects:<br>- The statement is primarily technical and focused on specific policy measures rather than broader ideological principles.<br>- Both sides might agree on the importance of clear guidelines and appropriate care but differ on the methods to achieve this.<br><br>### Conclusion:<br>The statement embodies principles that could be supported by both sides for different reasons: liberals for the equitable and comprehensive care aspects, and conservatives for the potential for efficiency and standardized practices. It doesn't strongly align with one viewpoint over the other but rather focuses on practical measures in healthcare policy. The true alignment might depend more on the broader context of these policies and how they're implemented.	neutral
ACP	Reimbursement for New Physicians	2015	The text you've provided states that the American College of Physicians (ACP) opposes reimbursement models based on the number of years a physician has been in practice. This viewpoint doesn't neatly align with a traditional liberal or conservative ideology. Instead, it's more related to healthcare policy and professional standards. Both liberal and conservative viewpoints can have varied perspectives on this issue depending on their broader beliefs about healthcare systems, professional compensation, and quality of care. Liberals might support it if they see it as promoting equality and focusing on quality and outcomes, while conservatives might support it if they see it as promoting efficiency and merit-based systems. However, these are generalizations and the actual stance would depend on the specific arguments and context within the broader healthcare debate.	neutral
ACP	Reimbursement Policy on Long Term Care Patients	2015	The text from the American College of Physicians (ACP) aligns with principles that can be appreciated across the political spectrum but generally leans more towards a liberal viewpoint. Here's how:<br><br>1. **Focus on Equity and Quality of Care:** The emphasis on ensuring that patients receive care based on their medical needs rather than payment type or other unrelated parameters resonates with liberal values of fairness and equity in healthcare.<br><br>2. **Opposition to Overbearing Documentation:** Arguing that documentation should not deter from providing quality care reflects a stance against bureaucratic obstacles, which can align with both liberal and conservative viewpoints but leans liberal when connected with healthcare access and equity.<br><br>3. **Resource-Based Reimbursement:** Advocating for reimbursement reflecting resource costs, regardless of the service location, suggests a desire for a more standardized and fair healthcare system, a common theme in liberal healthcare policies.<br><br>4. **Intent of Medicare's Policy:** Aiming to increase the level of service and ensure it's medically necessary and of high quality aligns with liberal objectives of expanding and improving public healthcare programs like Medicare.<br><br>However, the text doesn't strictly adhere to all liberal policies and might find some support among conservatives who advocate for sensible healthcare reforms, efficient resource use, and quality medical care. The focus on not allowing documentation to hinder care and ensuring reimbursement reflects actual costs might resonate with conservative viewpoints favoring efficiency and reduced bureaucracy.	liberal

			In all, while the text mostly aligns with liberal healthcare principles, it also includes elements that can be supported by a broad range of political ideologies, reflecting a nuanced approach to healthcare policy.	
ACP	Reimbursement for Physicians That Complete All Medicare Claims	2015	The text appears to reflect a viewpoint that is not strictly liberal or conservative but rather focused on practical aspects of healthcare administration. It advocates for the American College of Physicians (ACP) position on ensuring that administrative costs associated with complying with Medicare regulations, including mandatory claims submission, are adequately recognized in reimbursement formulas. This perspective emphasizes the financial and operational realities of healthcare providers dealing with complex regulations, which is a concern that can cross ideological lines. Both liberal and conservative viewpoints might agree on the need for efficient and fair compensation for healthcare providers, although they might differ on broader healthcare policy issues. This text focuses specifically on the administrative and operational aspects rather than broader ideological healthcare debates.	neutral
ACP	Payment For Services Provided by Covering Physicians	2015	The text describes an effort by the American College of Physicians (ACP) to work with the Centers for Medicare & Medicaid Services (CMS) to streamline billing processes while ensuring compliance and fraud prevention. It doesn't explicitly align with a liberal or conservative viewpoint; instead, it focuses on practical, administrative solutions within the healthcare system. Both liberals and conservatives might agree on the importance of efficient billing processes and fraud prevention, though they might disagree on how to implement such measures. The statement is primarily professional and administrative, aiming for a balance between ease of billing and regulatory compliance.	neutral
ACP	CMS Enacted Reduction in DXA Reimbursement	2022	The text presents policy positions related to Medicare payment for dual-energy x-ray absorptiometry (DXA) services and the implementation of a specific Medicare Improvement for Patients and Providers Act (MIPS) measure. These positions are from the American College of Physicians (ACP). The support for a government-commissioned study and reliance on clinical recommendations from the United States Preventive Services Task Force (USPSTF) to inform healthcare measures indicates a preference for evidence-based policy and potentially more government involvement in healthcare.<br><br>In the U.S. political spectrum:<br>- **Liberal viewpoint:** Generally favors more government involvement in healthcare to ensure access and quality, supports evidence-based policy, and values interventions like preventive services. This text aligns with these aspects, suggesting a tilt towards a liberal viewpoint.<br>- **Conservative viewpoint:** Often emphasizes less government intervention, more market-based solutions, and may prioritize cost-saving measures. While conservative viewpoints might support efficient healthcare measures, the emphasis on government-commissioned studies and specific regulatory measures might be less aligned with traditional conservative preferences for less regulation and more market-driven solutions.<br><br>However, healthcare policy can cross ideological lines, and both conservatives and liberals might find aspects to agree with in the pursuit of efficient, effective healthcare. It's essential to recognize that individual positions on such specific issues can vary widely within each ideological group.	neutral

ACP	Resource Based Relative Value Scale (RBRVS) Use in Productivity and Compensation Systems	2017	The text primarily discusses technical policy recommendations related to the Centers for Medicare & Medicaid Services (CMS) and the Resource-Based Relative Value Scale (RBRVS). It's a request from the American College of Physicians (ACP) for more transparency and accurate reflection of resource values in medical billing, specifically concerning work, practice expense, and professional liability.<br><br>This text doesn't neatly align with a traditional liberal or conservative viewpoint as it is more focused on healthcare policy and administrative procedures rather than broader political ideologies. However, the implications of these policies can be interpreted differently depending on one's political perspective:<br><br>- **Liberal viewpoint**: From a liberal perspective, the call for transparency and accurate reflection of resource values in CMS's RBRVS might be seen as a step towards ensuring fairness and accountability in healthcare. Liberals typically support measures that they believe improve the equity and accessibility of healthcare services.<br><br>- **Conservative viewpoint**: Conservatives might view these recommendations as a way to reduce bureaucratic inefficiency and promote market-based solutions by accurately reflecting the cost of services without governmental adjustments. They generally favor less government intervention and more transparency in pricing to allow for informed consumer decisions and competition.<br><br>Both sides might agree on the need for transparency and accurate data, but their reasons and what they wish to achieve with it might differ. It's essential to understand that many policy issues, especially those as complex as healthcare finance, transcend simple left-right politics and involve a variety of stakeholder perspectives and interests.	neutral
ACP	RBRVS Terminology	2016	The text you provided pertains to healthcare policy and reimbursement, specifically mentioning the American College of Physicians (ACP), the American Medical Association (AMA), the Relative Value Scale Update Committee (RUC), and the Resource-Based Relative Value Scale (RBRVS). The ACP is urging the AMA to publish recommendations for work relative value units (RVUs) that the Centers for Medicare & Medicaid Services (CMS) does not accept, and it distinguishes between Medicare reimbursement schedules and the RBRVS.<br><br>This statement does not neatly align with a traditional liberal or conservative viewpoint as it's focused on healthcare administration and policy rather than broader ideological issues. Both liberals and conservatives have varied and nuanced views on healthcare policy, often shaped by broader principles like market freedom, government intervention, and individual rights.<br><br>- **Liberal viewpoint**: Might support transparency and accountability in healthcare administration, advocating for policies that ensure equitable access to healthcare. They might view the publication of RVU recommendations as a step toward more informed decision-making and fairness in reimbursement rates.<br><br>- **Conservative viewpoint**: Might emphasize market-based solutions and limited government intervention in healthcare. They could support the publication of RVU recommendations if they believe it would lead to more efficient and cost-effective healthcare by fostering competition and informed choices.<br><br>In summary, the text doesn't inherently align with one political ideology over the other. It's more about the specifics of healthcare administration and transparency, and opinions on it could vary within both liberal and conservative circles depending on their broader healthcare policy views.	neutral

ACP	Refinement and Implementation of the Medicare Fee Schedule	2019	The text you've provided seems to discuss a stance on healthcare policy, specifically addressing changes to the Medicare Fee Schedule and the "Budget Neutrality" provision. It doesn't explicitly align with a liberal or conservative viewpoint as it focuses on a specific policy issue within the American healthcare system. However, the stance on budget neutrality and legislative changes can hint at broader ideological leanings depending on the context and the specifics of the proposed changes.<br><br>In general:<br><br>- **Liberal Viewpoint**: May support changes to healthcare policies that increase spending on Medicare or expand services, often with a focus on increasing access and coverage. They might oppose strict budget neutrality if it limits the ability to expand or enhance Medicare services.<br><br>- **Conservative Viewpoint**: Likely to emphasize fiscal responsibility, possibly supporting budget neutrality to prevent increased spending. Conservatives might focus on maintaining current spending levels or seeking efficiency within existing budgets.<br><br>The text's call for "aggressive" action to obtain legislative changes to prevent distortion of Relative Values might suggest a proactive stance on reforming healthcare financing, but without more context, it's hard to definitively categorize it as liberal or conservative. It's also important to note that healthcare policy can transcend traditional political labels, with individuals and groups across the spectrum holding various nuanced positions on how best to manage and finance healthcare.	neutral
ACP	Preferential Update in RVUs	2016	The text you've provided expresses opposition to a preferential update in Relative Value Units (RVUs) for surgeons at the expense of the rest of the medical profession, particularly primary care physicians. This stance aligns more closely with a liberal viewpoint for several reasons:<br><br>1. **Emphasis on Equality:** Liberal ideologies often emphasize fairness and equality, advocating for policies that distribute resources and opportunities more evenly across different groups. In this case, opposing preferential treatment for surgeons in favor of a more balanced approach that considers the needs of all medical professionals, including primary care physicians, aligns with these liberal principles.<br><br>2. **Support for Primary Care:** Liberals tend to support robust public health systems and may prioritize primary care as a foundation for preventive medicine and accessible healthcare. Arguing against policies that might undermine primary care physicians could reflect a liberal viewpoint that values widespread access to basic healthcare services.<br><br>3. **Concern for Professional Equity:** The statement reflects a concern for equity among different medical professionals. Liberal perspectives often advocate for policies that prevent excessive disparities and promote fairness within systems, including healthcare.<br><br>It's important to note that terms like "liberal" and "conservative" can vary significantly in meaning depending on the context, especially across different countries and political systems. Additionally, individuals and organizations might hold a mix of views that don't neatly fit into one category. In the context of American politics, the statement's emphasis on fairness and support for primary care aligns more with typical liberal positions, but someone with conservative views might also agree with the statement for different reasons, such as a concern for fiscal responsibility or maintaining a high standard of patient care.	liberal

ACP	RBRVS and Private Insurers	2016	The statement "ACP urges all third-party payers to adopt RBRVS (Resource-Based Relative Value Scale) principles, but not CMS's (Centers for Medicare & Medicaid Services) implementation methodology" reflects a nuanced stance that doesn't strictly align with traditional liberal or conservative viewpoints. Here's a breakdown of how both sides might view it:<br><br>**Liberal Viewpoint:**<br>- **Generally Supportive of RBRVS:** Liberals might support the use of RBRVS because it aims to allocate medical resources more fairly and can potentially reduce healthcare costs, a common goal in liberal healthcare policy.<br>- **Critique of CMS Implementation:** They may agree with the critique of the CMS's implementation if they believe it doesn't go far enough in ensuring equitable access to healthcare or if it's seen as inefficient.<br><br>**Conservative Viewpoint:**<br>- **Varied Opinions on RBRVS:** Conservatives might be skeptical of RBRVS if they see it as a government overreach into the healthcare market. However, some conservatives may support it if it's viewed as a means to make healthcare more market-efficient and reduce unnecessary spending.<br>- **Criticism of Government Administration:** The critique of CMS's methodology might resonate with conservatives who generally favor less government intervention and more market-based solutions.<br><br>**Non-Partisan Professional View:**<br>- The American College of Physicians (ACP), as a professional organization, tends to focus on what's best for patient care and the medical profession. Their stance likely comes from a perspective of practicality and effectiveness rather than political ideology. They might advocate for RBRVS principles because they see them as a fair way to determine payment for medical services, but have specific concerns about how CMS is implementing those principles.<br><br>In summary, the statement reflects a specific policy stance that is more about the effectiveness and fairness of a particular healthcare payment system than about broader ideological principles. Both liberals and conservatives might find aspects to agree with or critique, depending on their views on healthcare policy and government involvement in the healthcare system.	neutral
ACP	Pharmacist Scope of Practice		The text provided outlines positions from the American College of Physicians (ACP) regarding physician-pharmacist collaborative practice agreements and the role of pharmacists in patient care. This perspective doesn't neatly align with traditional liberal or conservative viewpoints, as it spans issues related to healthcare quality, professional collaboration, and regulation, which are concerns for both sides of the political spectrum. Here's how the positions might be viewed:<br><br>**Liberal Viewpoints:**<br>- **Support for Collaborative Practice**: Liberals generally favor team-based healthcare approaches that can improve access and outcomes. The emphasis on dynamic clinical care teams and ensuring that team members act in the patient's best interests aligns with a focus on comprehensive and accessible care.<br>- **Role of Pharmacists in Immunization**: The support for pharmacists as immunizers, especially during public health emergencies, aligns with liberal priorities for broadened healthcare access and public health initiatives.<br><br>**Conservative Viewpoints:**<br>- **Opposition to Independent Prescriptive Privileges**: Conservatives might appreciate the emphasis on physician oversight and the opposition to independent pharmacist prescriptive privileges as it maintains a more traditional, regulated approach to healthcare delivery and could be seen as upholding high standards of patient care.<br>- **Concerns Over Cost and Compensation**: The focus on not solely basing expanded roles on cost savings, and ensuring compensation for collaborative services, might resonate with conservative views on market-driven healthcare and fair compensation for professional services.<br><br>**Both Viewpoints:**<br>- **Quality and Safety**: Both liberals and conservatives would likely agree on the importance of quality care, patient safety, and ensuring that professionals are well-trained and acting within their scope of practice. The text emphasizes structured referrals, training requirements, and coordination with primary care, which are issues of universal concern.<br><br>**Conclusion:**<br>This text primarily reflects a professional and pragmatic standpoint focused on the best outcomes for patients and the healthcare system rather than a strictly ideological one. It combines an appreciation for collaborative, team-based care with a respect for	neutral

			professional boundaries and quality standards, elements that can resonate across the political spectrum. The positions aim to balance improved access and innovation in healthcare delivery with safeguards to maintain quality and safety, reflecting concerns common to both liberal and conservative perspectives.	
ACP	Use of New Techniques	2019	The text you've provided seems to advocate for the use of new diagnostic instruments and techniques by physicians across various specialties, emphasizing the importance of clinical competence over specialty designation. This perspective doesn't align strictly with a liberal or conservative viewpoint in the traditional political sense. Instead, it focuses on medical policy and practice, advocating for flexibility and competency in the healthcare system to improve patient care and reduce costs.<br><br>Generally, a liberal viewpoint might support such measures if they are seen as ways to improve access to healthcare and encourage innovation, while a conservative viewpoint might focus on the importance of maintaining high standards and possibly the autonomy of medical professionals to manage their practices. However, both sides might find agreeable elements in this policy, such as the focus on improving patient care and preventing excessive costs. It's more a question of healthcare policy and professional standards than a straightforward political issue.	neutral
ACP	Input from Practicing Internists to the Practice Management Center (PMC)	2019	The statement from the American College of Physicians (ACP) about devising a formal mechanism for providing input from practicing internists to the Department of Medical Practice (DMP) seems neutral and doesn't inherently align with a liberal or conservative viewpoint. It's focused on organizational and procedural aspects of medical practice rather than ideological or political issues. Both liberal and conservative individuals in the medical field might support the idea of ensuring that practicing physicians have a voice in the departments that impact their work, as it's more about effective governance and representation within the profession than about political ideology.	neutral

ACP	Appropriate Utilization of Endoscopy	2015	The text describes the American College of Physicians' (ACP) support for various initiatives related to the practice and regulation of endoscopic procedures. It doesn't align strictly with a liberal or conservative viewpoint; rather, it focuses on healthcare quality, safety, and efficiency. The key points include:<br><br>1. **Development of practice guidelines**: This aims to ensure quality and appropriate use of endoscopic procedures. Both conservative and liberal viewpoints might support this, as it pertains to improving healthcare quality and safety.<br><br>2. **Linking reimbursement to appropriate utilization**: This could be seen as a cost-containment measure, which might align more with a conservative view focused on efficient healthcare spending. However, liberals might also support it if they believe it leads to more equitable and rational healthcare delivery.<br><br>3. **Limiting payment to appropriately trained practitioners**: This is about ensuring that those performing procedures are qualified, which is a non-partisan issue generally supported across the spectrum for patient safety and quality care.<br><br>4. **Credentialing based on training and competence, not membership or designation**: This suggests a merit-based system, which both sides might agree on as it promotes fairness and quality in healthcare provision.<br><br>5. **Encouraging the use of the ACPNET network for developing guidelines**: This is about leveraging technology and networks to improve healthcare guidelines, which is generally a non-partisan issue.<br><br>Overall, the text focuses on promoting quality, safety, and efficiency in healthcare through specific standards and practices for endoscopic procedures. Both liberals and conservatives might find aspects to agree with, depending on their specific priorities and beliefs about the role of regulation, training, and efficiency in healthcare. It's more of a professional and procedural standpoint than a politically charged one.	neutral
ACP	Medical Screening Programs	2019	The text provided outlines a stance on medical screening programs, emphasizing cost-effectiveness and the importance of evaluation by a qualified physician prior to high-risk procedures. This viewpoint doesn't neatly align with traditional liberal or conservative ideologies as it focuses more on practical medical ethics and procedures rather than broader political or economic policies.<br><br>However, one might infer potential alignments based on how different ideologies view healthcare:<br><br>1. **Conservative Viewpoint Considerations**: Conservatives might agree with the emphasis on cost-effectiveness, as it aligns with a general preference for fiscal responsibility. However, they might have varied opinions on the extent of medical regulation or the role of physicians in making healthcare decisions.<br><br>2. **Liberal Viewpoint Considerations**: Liberals might appreciate the focus on thorough patient evaluation and quality care, aligning with a general preference for comprehensive healthcare access and regulation. However, they might have different views on how to achieve cost-effectiveness and the role of government in healthcare.<br><br>It's important to note that these are generalizations and real-world political beliefs can be more nuanced. Moreover, medical ethics and practices often transcend political lines, focusing on patient welfare, efficacy, and scientific evidence rather than ideology.	neutral

ACP	Controlling Health Care Costs: Wellness, Prevention, and Chronic Disease Management	2022	The text you've provided seems to blend elements from both liberal and conservative viewpoints, making it a mix rather than strictly aligning with one side. Here's a breakdown:<br><br>1. **Encouraging personal responsibility for health**: This is more of a conservative principle, emphasizing individual responsibility and personal choices in health and wellness.<br><br>2. **Increasing federal and state funding for health promotion**: This typically aligns with liberal viewpoints, advocating for more government intervention and support in public health initiatives.<br><br>3. **Support for public policy in health and wellness**: The call for community planning and promotion of healthy lifestyles can be seen in both liberal and conservative ideologies. Conservatives might support it from a personal responsibility and community engagement perspective, while liberals might focus on the need for government to facilitate healthy environments.<br><br>4. **Employers and health plans funding programs for wellness**: This is somewhat of a mixed approach. Conservatives might like the idea of private entities taking initiative, reducing the need for government programs. Liberals might appreciate the focus on preventive care and wellness, which are often part of more progressive health care reform ideas.<br><br>In summary, the text advocates for a combination of personal responsibility, government funding, community planning, and private sector involvement in health promotion. This blend of ideas does not neatly fit into a single ideological category but rather borrows from both liberal and conservative perspectives to create a comprehensive approach to health and wellness.	neutral
ACP	Disability Certification	2011	The text doesn't explicitly align with a liberal or conservative viewpoint. It focuses on the ethical considerations physicians must navigate when determining disability status for patients. It emphasizes honesty and factual reporting in disability evaluations, acknowledging the physician's role as an advocate while cautioning against misrepresentation. Both liberals and conservatives might agree on the importance of ethical standards in medical practice. However, the underlying concepts of social support and disability might be interpreted differently based on one's political viewpoint:<br><br>- **Liberal Perspective:** Liberals might focus on the text's acknowledgment of societal support for individuals with chronic illnesses and the physician's role in advocating for patients who need financial support. They may emphasize the importance of a compassionate approach to healthcare and social safety nets.<br><br>- **Conservative Perspective:** Conservatives might appreciate the emphasis on honesty and not distorting medical information to obtain benefits. They might focus on the importance of trustworthiness and maintaining strict criteria for disability to prevent system abuse.<br><br>Overall, the text presents a balanced view on ethical medical practice, with potential interpretations varying based on one's political leanings and emphasis on different parts of the text.	neutral
ACP	Equitable Risk Classification in Medical Liability Premiums	2015	The statement from the ACP (American College of Physicians) supports the idea that medical liability insurance premiums should be based on the actual cost and risk associated with providing insurance to each specific group or category. This perspective can align with elements from both liberal and conservative viewpoints.<br><br>Conservatives might favor this approach as it aligns with free-market principles, emphasizing that prices and premiums should reflect the actual risk and cost, promoting efficiency and personal responsibility. It's a stance that resonates with a more individualistic approach to insurance, where premiums are tailored to the specific risks and costs of an individual or a group.<br><br>Liberals, on the other hand, might appreciate the emphasis on fairness and equity, ensuring that premiums are not arbitrarily set but are reflective of the actual costs and risks involved. This can be seen as a way to prevent undue burden on certain groups and ensure that insurance is accessible and fair.<br><br>In summary, the statement touches on principles that are important to both ideologies: market-based solutions and fairness. The alignment might vary based on the broader context of the policy and how it's implemented.	neutral

ACP	Liability Coverage for Physician Members of Hospital Committees	2019	The statement "ACP believes that all hospitals should hold harmless or provide liability insurance for all physicians who participate in hospital committee work" doesn't neatly align with a traditional liberal or conservative viewpoint. It's more related to healthcare policy and physician welfare. Both liberal and conservative perspectives can have varied stances on such specific policy matters, depending on the broader context of healthcare system beliefs, fiscal responsibility, and individual rights.<br><br>Liberals might support this statement as part of a broader agenda for comprehensive healthcare and worker protections, viewing it as a measure to ensure that healthcare providers can serve without fear of personal financial ruin. On the other hand, conservatives might also support it from a standpoint of personal responsibility and protecting physicians who contribute to hospital governance, but they might also be wary of the potential for increased costs and implications for the healthcare market.<br><br>The American College of Physicians (ACP), which is referred to in the statement, is a non-partisan organization focused on enhancing the quality and effectiveness of healthcare. ACP's advocacy positions generally focus on improving healthcare and the practice environment for physicians, which doesn't necessarily align with a specific political ideology.	neutral
ACP	Medical Liability Reform	2014	The text you've provided outlines various recommendations for reforming the medical liability system and improving patient safety. The recommendations seem to blend both liberal and conservative viewpoints:<br><br>1. **Liberal Aspects**:<br>- The emphasis on improving patient safety, promoting a culture of quality improvement, and training physicians aligns with a more liberal viewpoint that focuses on systemic improvements and comprehensive approaches to healthcare.<br>- Supporting the development of safe harbor protections and advocating for communication and resolution programs also reflect a desire for systemic changes that are often associated with liberal perspectives.<br><br>2. **Conservative Aspects**:<br>- The recommendation for caps on noneconomic damages and tort system reforms like those in the California Medical Injury Compensation Reform Act (MICRA) aligns with conservative viewpoints that often seek to limit lawsuit potential and reduce the impact on the medical industry.<br>- Advocating for the examination of the insurance industry's financing operations and the establishment of minimum standards for expert witnesses can also reflect a conservative approach focusing on regulation and standards to curb excessive or unwarranted claims.<br><br>3. **Neutral/Blended Aspects**:<br>- Recommendations for research into the effect of team-based care, pilot projects for health courts, and alternative dispute resolution models do not distinctly align with one political ideology. They represent a more neutral approach focused on exploring various solutions to complex problems.<br><br>In summary, while some aspects of the text align more with conservative viewpoints (like caps on damages and tort reforms) and others align more with liberal viewpoints (like emphasizing patient safety and systemic improvements), the overall document seems to aim for a balanced approach that incorporates elements from both ideologies. It's a comprehensive set of recommendations that seeks to address the issue from multiple angles, reflecting a pragmatic approach to reforming the medical liability system.	neutral

ACP	ERISA	2017	The text you've provided outlines the American College of Physicians' (ACP) stance on certain health policy reforms. It doesn't neatly align with a traditional liberal or conservative viewpoint because both viewpoints have complex and sometimes overlapping positions on healthcare reform. However, we can analyze the components:<br><br>1. **Alternatives to traditional tort reforms**: Conservatives generally favor traditional tort reforms to limit litigation and reduce costs. Liberals are more likely to support exploring alternatives that might offer broader protections to patients.<br><br>2. **Enterprise liability, no-fault approaches, and privately contracted mediations**: These are innovative approaches to resolving disputes and could be seen as attempts to find a middle ground. They might appeal to liberals wanting more patient protections and conservatives interested in efficiency and cost containment.<br><br>3. **Seeking liability reforms in a managed care environment**: Conservatives might favor this as it could lead to more market-driven solutions and potentially lower costs. Liberals might support it if it leads to better patient protections and outcomes.<br><br>4. **Legislation to change ERISA**: The Employee Retirement Income Security Act (ERISA) is complex, and positions on it can vary. Generally, advocating for changes that would hold healthcare plans legally liable for patient injuries suggests a focus on patient rights and protections, which is more traditionally a liberal stance.<br><br>In summary, the ACP's positions seem to blend elements that could be appealing to both sides, with a perhaps slight lean towards traditionally liberal values of patient protection and rights. However, the realignment of political ideologies, especially around healthcare, makes it difficult to categorize such positions strictly as "liberal" or "conservative."	probably liberal
ACP	Principles on the Role of Governments in Regulating the Patient-Physician Relationship	2012	The text provided reflects principles recommended by the American College of Physicians (ACP) concerning the roles of government in healthcare and the patient-physician relationship. It doesn't align strictly with a liberal or conservative viewpoint but has elements that might be supported or critiqued by both sides.<br><br>1. **Support for Patient-Physician Relationship and Ethics (Point 1):** Both liberals and conservatives generally agree on the importance of the patient-physician relationship and medical ethics. The emphasis on beneficence, honesty, and privacy is universally appealing, though the extent and means of government involvement might be a point of contention.<br><br>2. **Opposition to Restrictive Laws (Point 2 & 3a):** The stance against laws restricting physician-patient discussions might align more with a liberal viewpoint that emphasizes personal freedoms and patient rights. However, conservatives who advocate for limited government interference in personal lives might also support this.<br><br>3. **Non-discrimination in Treating Patients (Point 3b):** Advocating for the treatment of all patients regardless of status aligns with a liberal perspective on equality and anti-discrimination. While many conservatives might agree with the ethical standpoint, they might disagree on legal and regulatory mechanisms to enforce it.<br><br>4. **Governmental Regulation and Public Health (Point 3c, 3v, and 5):** The caution against overregulation could resonate with conservative ideals of limited government, but the acknowledgment of public health objectives and the need for evidence-based policy aligns with a liberal trust in scientific expertise and structured interventions.<br><br>5. **Emphasis on Individual Patient Values (Point 4):** This might appeal to conservatives' respect for individual rights and autonomy. Liberals might also agree, emphasizing the patient's right to control their healthcare decisions.<br><br>6. **Need for Adaptive Laws and Avoidance of Undue Burdens (Point 6 & 7):** The call for flexible, evidence-based guidelines and regulations that don't overburden physicians might find support across the spectrum, though the approach and rationale might differ between liberal and conservative perspectives.<br><br>In summary, the text contains principles that could be endorsed by both liberal and conservative viewpoints for different reasons. It advocates for patient rights, ethical medical practice, and evidence-based regulation, with a cautious approach to government intervention—themes that resonate to varying degrees with both ideologies.	neutral

ACP	Principles Regarding Professional Accountability	2018	The text you provided outlines the principles and guidelines for professional accountability, particularly focusing on the clinical and ethical standards within the American College of Physicians (ACP). It emphasizes continual self-scrutiny, self-regulation, certification, and transparency in evaluation processes, among other things. This text is primarily professional and procedural in nature and doesn't explicitly align with a traditional liberal or conservative political viewpoint. Instead, it focuses on the ethical and professional standards expected of ACP members, promoting continual improvement, accountability, and transparency which are values that can be endorsed by individuals across the political spectrum. The text emphasizes the importance of ethical conduct, professional development, and maintaining standards, which are concerns central to the professional field it addresses rather than to a particular political ideology.	neutral
ACP	Definition of Internal Medicine Physicians	2023	The text you've provided is a definition of internal medicine physicians from the American College of Physicians (ACP). It doesn't explicitly align with a liberal or conservative viewpoint. Rather, it provides a professional and clinical description of what internal medicine physicians do. It focuses on the application of scientific knowledge and clinical expertise in the care of adults, which is a neutral and factual description of the profession. This kind of definition is typically intended to be informative and non-partisan, aimed at providing clarity about the role and responsibilities of internal medicine physicians.	neutral
ACP	The Physician and Society	2019	The text you've provided outlines a viewpoint on the professional and ethical responsibilities of physicians towards their patients and society. It doesn't align explicitly with either a liberal or conservative viewpoint as it focuses on professional ethics and standards in medicine rather than broader political ideologies.<br><br>Liberal and conservative viewpoints typically diverge on issues like government intervention, individual liberties, economic policies, and social issues. This text, however, focuses on the mutual expectations and responsibilities between physicians and society, emphasizing accountability, competence, and consideration. These are principles that can be endorsed by individuals across the political spectrum as they relate to professional conduct and ethics in healthcare, rather than specific political or ideological beliefs.	neutral
ACP	Obligations of the Physician to Society	2022	The text provided does not explicitly align with a liberal or conservative viewpoint as it primarily focuses on the ethical and professional responsibilities of physicians towards their patients and society. It emphasizes the importance of health and human rights, advocating for community health, addressing social and environmental causes of disease, and ensuring access to healthcare for all, regardless of personal characteristics like race. These themes are generally centered around the professional ethics and responsibilities of physicians and do not delve into the ideological debates often associated with liberal or conservative politics. However, some might interpret the emphasis on community health, human rights, and addressing disparities as aligning more closely with liberal values, which typically focus on social equity and collective welfare. At the same time, the call for individual responsibility and professionalism can be seen as compatible with conservative values, which often emphasize personal responsibility and individual ethics. It's important to note that such interpretations can vary widely, and the text primarily focuses on the ethical obligations of physicians rather than promoting a specific political ideology.	neutral
ACP	The Changing Practice Environment	2019	The text presents a nuanced view that doesn't strictly align with a single political ideology, liberal or conservative. It emphasizes ethical medical practice, patient welfare, efficient resource use, and the need for open dialogue among all stakeholders in the healthcare system. The text advocates for physicians to prioritize patient care above financial incentives and for transparent, fair processes in healthcare decision-making. It also acknowledges the complexities of the healthcare system, including the impact of insurance and the need for attention to underserved populations.<br><br>This perspective aligns with liberal values in its emphasis on equitable access to healthcare and attention to the needs of the uninsured or underinsured. It also reflects conservative values in its focus on efficient resource use and personal responsibility in understanding insurance rules.<br><br>Overall, the text represents a balanced viewpoint focused on ethical medical practice and patient-centered care, transcending simple political categorization by addressing concerns common to both liberal and conservative perspectives. It highlights the multifaceted nature of healthcare decision-making and the importance of balancing various interests and obligations within the system.	neutral
ACP	Expert Witnesses	2019	The text you've provided is primarily a statement on the ethical duties and responsibilities of physicians when involved in legal proceedings as expert witnesses. It doesn't directly align with a liberal or conservative viewpoint as it focuses on professional ethics in medicine rather than political ideology. It emphasizes the importance of physicians' participation in judicial processes to ensure informed decisions and just outcomes, advocating for ethical conduct, objectivity, and appropriate compensation for their expertise. Both liberal and conservative perspectives might agree on the necessity of expert testimony in legal proceedings and the ethical standards professionals should uphold, making this text more about professional ethics than political ideology.	neutral

ACP	Strikes and Other Joint Actions by Physicians	2019	The text presents a viewpoint that seems to align with both liberal and conservative principles in different ways. The focus on high-quality patient care, the exercise of independent clinical judgment, and advocacy for system change resonate with liberal values of ensuring fairness and access to healthcare. Liberals typically support reformative actions to improve systems for the betterment of society.<br><br>On the other hand, the text's opposition to strikes, work stoppages, and other forms of protest that could disrupt patient care aligns more closely with conservative values, which emphasize law and order, respect for tradition, and individual responsibility. The emphasis on not engaging in anticompetitive behavior and seeking alternative advocacy methods also reflects a conservative viewpoint that values market principles and orderly conduct.<br><br>Overall, the text seems to strive for a balanced approach, advocating for change and improvement in the healthcare system (a more liberal viewpoint) while also emphasizing the importance of maintaining patient care and order (a more conservative viewpoint). It suggests a nuanced position that doesn't fully align with one side but rather integrates aspects of both perspectives.	neutral
ACP	The Impaired Physician	2019	The text provided doesn't align explicitly with a liberal or conservative viewpoint; instead, it discusses ethical and professional standards in the medical field. It focuses on the responsibility of physicians to refrain from patient care when impaired for any reason, including substance use or mental health issues, and the duty of their peers to assist and report if necessary. It emphasizes patient safety, professional accountability, and the need for confidentiality and proper monitoring. These topics are generally considered non-partisan and relate more to medical ethics and professional conduct than to a specific political ideology.	neutral
ACP	Peer Review	2019	The text you've provided outlines ethical guidelines and responsibilities for physicians, particularly regarding peer review and professional conduct. It doesn't neatly align with a liberal or conservative viewpoint as it's more focused on professional ethics and standards within the medical community. The emphasis is on maintaining trust, ensuring accountability, and protecting patients, which are principles that both liberals and conservatives might agree on. The text underscores the importance of self-regulation, fairness, and the duty to report misconduct, which are values transcending political ideology. It's more about upholding the integrity and trustworthiness of the medical profession than about political leanings.	neutral
ACP	Conflicts among Members of a Health Care Team	2019	The text provided outlines principles of teamwork, mutual respect, and ethical decision-making in healthcare. It doesn't inherently align with a strictly liberal or conservative viewpoint. Instead, it focuses on best practices for healthcare professionals to collaborate effectively and ethically. The emphasis on teamwork, mutual respect, and the use of ethics committees to resolve disputes are principles that can be valued across the political spectrum. While individuals with different political beliefs might interpret or prioritize these aspects differently, the text itself is centered on professional and ethical standards rather than political ideology.	neutral
ACP	Physician-Driven Integration: A Response to the Corporatization of Medicine	2017	The text appears to align more with a liberal viewpoint. It emphasizes the importance of physician-led integration, professional values, collaborative practice, and the role of physician leadership in quality and utilization management. These principles generally align with a liberal perspective on healthcare, focusing on collaborative, quality-driven care and prioritizing the well-being of patients and healthcare providers. The text advocates for professional commitment, altruism, research, and self-improvement in healthcare, and supports the involvement of professional societies, medical schools, and government in research and education. These concepts tend to reflect a liberal ideology that values collective action, regulation, and the role of government and professional organizations in supporting healthcare systems and ensuring quality and equitable care.	liberal
ACP	Promoting the Leadership Role of Physicians in the Health Care Team	2022	The text from the American College of Physicians (ACP) seems to align more closely with a conservative viewpoint in the context of health care policy. It emphasizes the distinct roles and leadership of physicians in healthcare teams, suggesting a preference for traditional, hierarchical models of care delivery. This stance might resonate with conservative values that emphasize respect for established structures and roles. However, political viewpoints are complex and can vary widely among individuals, so this interpretation might not hold for every liberal or conservative. Some liberals might agree with the emphasis on physician leadership due to a focus on expertise and quality care, while some conservatives might prefer more market-based or diversified models of care. Ultimately, determining the exact alignment requires considering a wider range of perspectives and policies.	conservative
ACP	Volunteers in Medicine	2017	The text expresses support for organized efforts involving volunteer healthcare professionals in responding to public health emergencies and delivering healthcare to displaced, indigent, and uninsured individuals. This viewpoint aligns more closely with a liberal perspective, which often emphasizes the importance of collective action and social responsibility, particularly in providing care and support for underprivileged populations. Conservative viewpoints, on the other hand, tend to prioritize individual responsibility and limited government intervention. However, it's important to note that there can be a wide range of beliefs within each political ideology, and some conservatives might also support such initiatives based on values of community support and volunteerism.	liberal
ACP	Corporate Medical Practice	2019	The text you've provided from the American College of Physicians (ACP) regarding the disclosure of ownership in medical practices doesn't align strictly with either liberal or conservative viewpoints as traditionally defined in politics. Instead, it's focused on ethical and professional standards within the medical field. The suggestion that physicians disclose their employment status and the ownership details of their practice is rooted in transparency and patient trust, which are principles valued across the political	neutral

			spectrum. The ACP's call for co-adoption of this policy by the American Medical Association (AMA) indicates a desire for broader professional consensus rather than a political stance.	
ACP	Physician-Run Health Plans and Antitrust	2017	The text appears to present a viewpoint that could resonate with aspects of both liberal and conservative ideologies, making it difficult to classify strictly as one or the other.<br><br>From a **liberal perspective**, it might align due to:<br>1. **Advocacy for reform and protection**: Liberals often support reformative measures in healthcare to ensure fairness and equity, as well as protections for providers and patients. The text's emphasis on due-process protections and reform to empower physicians aligns with this.<br>2. **Empowerment of individual providers**: Liberals typically advocate for empowering smaller entities and individuals against larger, possibly oppressive systems. The encouragement for physicians to form their own networks and plans could be seen as a move towards decentralization and empowerment at a more personal level.<br><br>From a **conservative perspective**, it might resonate due to:<br>1. **Market-based solutions**: Conservatives generally prefer market-driven solutions over government intervention. The text's encouragement for physicians to create and operate their own health plans suggests a move towards more private, market-based solutions.<br>2. **Skepticism towards large entities**: While often associated with supporting big businesses, conservatives also express concern over large, controlling entities, especially in the context of personal freedom and autonomy. The concern over large managed-care entities and the push for more autonomy for individual physicians could align with this viewpoint.<br><br>In conclusion, the text doesn't clearly favor one ideology over the other. Instead, it focuses on specific issues and solutions that could be supported by individuals across the political spectrum depending on their views on healthcare, market regulation, and professional autonomy.	neutral
ACP	Continuing to Assess and Provide New Information on Non-Traditional Care Models	2023	The text you've provided from the American College of Physicians (ACP) seems to focus on supporting internal medicine physicians in patient-centered practice models. It emphasizes accessibility, ethics, viability, and strengthening the patient-physician relationship. This stance doesn't align strictly with a liberal or conservative viewpoint as it primarily focuses on professional standards and patient care within the medical community. Both liberals and conservatives might support such a statement because it centers on quality healthcare and professional ethics rather than a specific political ideology.	neutral
ACP	Supporting Legislation that Requires Nationwide Criminal Background Checks for Health Care Workers	2022	The text reflects support for provisions in the federal Patient Protection and Affordable Care Act of 2010, specifically the requirement for nationwide criminal background checks on applicants for positions involving direct care of vulnerable patients. This viewpoint aligns more closely with a liberal perspective. Generally, liberal viewpoints tend to favor regulatory measures in healthcare to ensure patient safety and uphold quality of care. They often support reforms and policies aimed at expanding access to healthcare services and improving patient safety, which this provision aims to address by ensuring that caregivers for vulnerable patients have undergone background checks. Conservative viewpoints, on the other hand, might emphasize less regulation and more market-driven solutions in healthcare, although they also value patient safety and quality of care.	liberal

ACP	The Health Care Response to Pandemic Influenza	2022	The text you've provided outlines various positions taken by the American College of Physicians (ACP) regarding the preparation and response to pandemic influenza. It doesn't align strictly with liberal or conservative viewpoints as typically defined in American politics. Instead, it focuses on practical, medical, and ethical considerations that span across the political spectrum. Here's a breakdown:<br><br>- **Public Health and Preparedness (Positions 1, 2, 6, 7):** These positions emphasize the importance of preparedness, coordination, and resource availability. Both conservatives and liberals generally agree on the necessity of preparedness, though they might differ on the methods and the level of government involvement.<br><br>- **Surveillance and Confidentiality (Positions 3, 4):** These positions balance the need for effective public health monitoring with individual rights to privacy, a concern that resonates across the political spectrum. Conservatives might emphasize individual rights more, while liberals might focus on the collective good, but the positions here strive to balance both perspectives.<br><br>- **Infection Control and Physician Autonomy (Position 5):** Advocating for clear, fair, and minimal restrictions, as well as protections for physicians, is a stance that doesn't neatly fit into liberal or conservative boxes. Both sides might agree on the importance of clear guidelines and due process.<br><br>- **Care Provision (Position 8):** The idea of utilizing non-hospital settings to provide care during a pandemic is pragmatic and can be supported by both sides for different reasons: conservatives for the potential to reduce government spending and liberals for the increased accessibility of care.<br><br>- **Provider Safety (Position 9):** Ensuring the safety of healthcare providers is a non-partisan issue that is universally important across political beliefs.<br><br>In summary, the positions taken by the ACP in this text are focused on effective, ethical, and equitable healthcare delivery during a pandemic. These goals transcend traditional political divisions and are more aligned with public health and medical ethics than with any specific political ideology.	neutral
ACP	Recognizing Critical Disaster Preparedness Programs	2022	The text you've provided is from the American College of Physicians (ACP) and outlines support for disaster preparedness training programs. It doesn't inherently align with a liberal or conservative viewpoint. Generally, disaster preparedness and response are considered non-partisan issues, focusing on public safety and health rather than political ideology. Both liberals and conservatives might agree on the importance of such training for mitigating the effects of disasters. However, the level of government involvement, funding, and specific approaches to disaster preparedness might vary and could be points of debate between different political ideologies.	neutral
ACP	Drug-Resistant Tuberculosis	2016	The text reflects a stance focused on public health and safety regarding drug-resistant tuberculosis. It doesn't neatly align with a traditional liberal or conservative viewpoint. Both political spectrums might agree on the importance of addressing public health issues, but they might differ on methods and funding. The focus here is on:<br><br>1. **Recognizing the seriousness of drug-resistant tuberculosis**: This suggests a priority on healthcare and public safety, a concern across the political spectrum.<br><br>2. **Regulations to decrease risks of exposure**: Conservatives generally prefer less regulation, while liberals might support more regulatory measures for public health. However, both might agree on the necessity in this specific context of healthcare.<br><br>3. **Support for research and outpatient treatment**: Liberals typically advocate for more funding in healthcare and research, but conservatives might also support this when it's about addressing a significant threat to the population.<br><br>It's more about practical health policy than political ideology. The statement from the American College of Physicians (ACP) is likely driven by medical and scientific concerns rather than a particular political agenda.	neutral

ACP	Supporting Restrictions on Tanning Establishments	2022	The text presents a viewpoint advocating for restrictions on tanning devices, particularly for minors, and for clear warnings about the potential harms of ultraviolet radiation. This perspective aligns more closely with a liberal viewpoint, which typically emphasizes the role of regulation in protecting public health and safety. Liberals often support measures that restrict activities or industries when they pose health risks, especially to vulnerable populations like minors. The focus on strict labeling and advertising guidelines also reflects a liberal inclination toward consumer protection and informed decision-making.<br><br>Conservatives, on the other hand, might be more inclined to resist such regulations, favoring less government intervention in personal choices and business operations. They might argue that individuals should have the freedom to assess risks and make their own decisions about tanning, and that businesses shouldn't be burdened with excessive regulations.<br><br>However, it's important to note that public health issues like this can sometimes garner bipartisan support, especially when the health risks are well-documented and involve protecting minors. So while the viewpoint in the text leans more liberal, it's not exclusively so.	liberal
ACP	Working with CMS to Identify Fair and Equitable Compensation for Formulas for Vaccines	2022	The text you've provided outlines a policy approach from the American College of Physicians (ACP) aiming to work with the Centers for Medicare and Medicaid Services (CMS) to develop compensation formulas for vaccine distribution. It's focused on ensuring fair and equitable compensation for vaccine acquisition and administration, aiming for widespread immunization in various practice settings.<br><br>This statement doesn't neatly align with traditional conservative or liberal viewpoints. Both conservatives and liberals might support the idea of widespread immunization and fair compensation for healthcare providers, but they might disagree on the methods to achieve these goals.<br><br>Conservatives might be wary of too much government intervention in healthcare and prefer market-based solutions, while liberals typically support more direct government involvement to ensure equitable access to healthcare. The focus on "fair and equitable compensation" and working with a government agency like CMS might be more aligned with liberal ideas of ensuring equitable access to healthcare services. However, the mention of considering "wholesale/retail cost differentials" might appeal to more market-oriented, conservative viewpoints that favor understanding and leveraging market dynamics.<br><br>It's important to note that individuals' opinions can vary widely within these broad political categories, and many people hold nuanced views that don't perfectly align with one side or the other.	neutral
ACP	Sodium Intake	2022	The statement you've provided from the American College of Physicians (ACP) seems to advocate for public health measures aimed at reducing sodium intake among American consumers and supports the Centers for Disease Control and Prevention (CDC) in its efforts toward this goal. This type of statement generally aligns with a public health perspective rather than a strictly political one.<br><br>However, perspectives on public health initiatives can sometimes align with broader political ideologies. Generally, liberal viewpoints tend to support proactive public health measures and may advocate for regulatory or educational initiatives to promote healthier lifestyles. This includes support for organizations like the CDC and policies aimed at reducing the consumption of substances that could lead to health issues, such as excessive sodium.<br><br>On the other hand, conservative viewpoints might emphasize personal responsibility and freedom of choice, potentially viewing such measures as overreach by governmental bodies. Conservatives might prefer less regulation and more individual choice, even in matters of health.<br><br>It's important to note, though, that public health is a complex field and doesn't always neatly align with political ideologies. Individuals and organizations across the political spectrum might support or oppose such measures based on various factors, including economic considerations, beliefs about the role of government, and interpretations of scientific evidence. Additionally, positions on specific issues can vary within ideological groups.	probably liberal
ACP	Excessive Heat Exposure	2020	The text appears to align with a liberal viewpoint. It emphasizes public health, worker safety, and the role of government and regulatory bodies in ensuring the well-being of individuals against environmental risks like heat exposure. The call for federal and state government intervention, research, and development of public health interventions, as well as the recommendation for regulatory standards from OSHA, are typically associated with a liberal perspective that advocates for a proactive role of the government in healthcare and worker protection. Conservative viewpoints might focus more on individual responsibility and less government intervention in business operations. However, concern for public health and safety is a non-partisan issue, and individuals across the political spectrum might support various aspects of these statements.	liberal

ACP	Opposing the Use of Antimicrobials for Agricultural Purposes	2011	The statement from the American College of Physicians (ACP) regarding the opposition to the use of antimicrobials in agriculture for growth promotion and/or prophylaxis, as well as advocating for the phasing out of antimicrobials for nontherapeutic uses, aligns more closely with a liberal viewpoint. This perspective typically emphasizes environmental protection, public health, and regulation of practices that could harm these areas. Liberals generally support stricter regulations on agricultural practices to protect public health and the environment, which includes concerns about antimicrobial resistance stemming from the overuse of antibiotics in livestock.<br><br>On the other hand, conservative viewpoints might prioritize less regulation on businesses and agriculture, advocating for the rights of farmers and industries to make their own decisions about production practices. They might argue that such regulations could increase costs for farmers and consumers or be skeptical about the extent of the impact these practices have on public health and the environment.<br><br>It's important to note, however, that the issue isn't strictly partisan, and there can be a range of opinions within each political spectrum. Concerns about antibiotic resistance and its impact on human health can transcend political divides, leading to some level of bipartisan agreement on certain regulatory measures.	liberal
ACP	Modernizing the United States' Public Health Infrastructure	2023	The text provided primarily aligns with a liberal viewpoint, typically characterized by an emphasis on systemic change, public investment, and government intervention to address public and social issues. Here's how:<br><br>1. **Support for Public Infrastructure and Coordination**: Liberal perspectives often advocate for a strong role of government in public health and coordination at various levels. The call for a designated official to guide and oversee public health efforts reflects a belief in organized, systemic solutions, a common liberal stance.<br><br>2. **Government Funding and Flexibility**: The emphasis on sustained, stable funding for public health from government sources aligns with liberal views on public investment for societal welfare. Exploring private sources to supplement government spending is also in line with more progressive ideas about diverse funding streams.<br><br>3. **Workforce Support and Protection**: Advocating for competitive salaries, educational support, and addressing workplace challenges like burnout and violence are consistent with liberal priorities around labor rights and worker support.<br><br>4. **Combatting Misinformation**: The focus on fighting dis- and misinformation, particularly through media literacy and partnerships, reflects a liberal concern for evidence-based governance and public discourse.<br><br>5. **Data Infrastructure and Privacy**: The emphasis on a modern public health data infrastructure with privacy protections and interoperability standards reflects liberal values around both innovation and individual rights.<br><br>6. **Collaboration and Community Health**: Encouraging collaboration between primary care and public health and focusing on health equity and community-based approaches are hallmarks of a more liberal, holistic view of health care.<br><br>While some aspects, like exploring private funding sources and promoting interoperability, might find support across the political spectrum, the overall emphasis on comprehensive, government-led, and systemic interventions is more aligned with a liberal viewpoint. Conservative perspectives might favor more limited government roles, market-based solutions, and individual responsibility over the collective approaches outlined in the text.	liberal

ACP	Position Statement on Immunization of Health Care Workers	2021	The text from the American College of Physicians (ACP) primarily aligns with a liberal viewpoint. It emphasizes the importance of vaccinations for healthcare workers to ensure public safety and reduce the transmission of infectious diseases. This is a stance generally associated with a more liberal or progressive viewpoint, which tends to support science-based public health measures and interventions.<br><br>The text also advocates for comprehensive strategies to increase immunization rates, including education to counter misinformation and making vaccines more accessible. This approach aligns with liberal values of addressing public health through broad, inclusive measures and education.<br><br>Additionally, the text's emphasis on health equity and the acknowledgment of historical and ongoing mistrust among marginalized communities reflect a liberal perspective that seeks to understand and address systemic inequalities and the impacts of discrimination and racism in healthcare.<br><br>However, it's important to note that public health and safety are concerns that cross political lines, and there are conservatives who also support widespread vaccination and public health measures. The viewpoint expressed is more about a commitment to public health and safety than strictly political ideology.	liberal
ACP	Preparing for Future Pandemics and Public Health Emergencies	2023	The text you've provided is primarily aligned with a liberal viewpoint. It emphasizes a strong role for the federal government in public health policy, including developing and funding comprehensive pandemic preparedness and response plans, prioritizing health equity, and supporting public–private partnerships in vaccine development. It also advocates for significant governmental intervention in health care supply chains, workforce training, and the protection of health care workers. These positions generally align more closely with contemporary liberal ideologies, which tend to support more extensive government involvement in health care and social welfare programs, as well as a focus on reducing health disparities and ensuring universal access to essential services. Conservative viewpoints, in contrast, typically emphasize smaller government, individual responsibility, and private sector solutions to public health and economic challenges. However, it's important to note that public health can be a bipartisan issue, with support for certain measures across the political spectrum.	liberal
ACP	Position Statement on Immunization Policies to Protect the Public	2021	The text from the American College of Physicians (ACP) primarily aligns with a liberal viewpoint, particularly in its strong advocacy for widespread immunization and the elimination of non-medical exemptions for vaccines. It supports state laws promoting immunization and endorses the need for employers and schools to require proof of vaccination for certain diseases. The text also acknowledges the necessity of federal and state regulations to expand vaccination rates, especially during public health emergencies.<br><br>The ACP's position on eliminating non-medical exemptions and advocating for employer and school vaccine mandates is more commonly associated with liberal viewpoints, which tend to emphasize the role of government and collective action in public health. The acknowledgment of historical mistrust in healthcare institutions and the emphasis on outreach and education to marginalized populations also reflect a liberal perspective focused on social justice and equity.<br><br>However, it's essential to note that public health and safety issues like immunization can transcend traditional liberal-conservative divides, especially in contexts where the focus is on evidence-based interventions to protect community health. While this statement leans towards a liberal viewpoint due to its emphasis on regulation and collective responsibility, public health policies can find support across the political spectrum depending on the context and specific proposals.	liberal

ACP	Envisioning a Better U.S. Health Care System for All: Reducing Barriers to Care and Addressing Social Determinants of Health	2019	The text from the American College of Physicians outlines a series of policy positions that can generally be aligned with a more liberal or progressive viewpoint. These positions emphasize:<br><br>1. **Ensuring an Adequate Supply of Physicians:** Focusing on the distribution of healthcare providers to underserved areas aligns with a liberal perspective that prioritizes equitable access to healthcare.<br><br>2. **Investment in Public Health Infrastructure:** The call for greater investment in public health, including addressing social determinants of health, is a hallmark of progressive health policy.<br><br>3. **Environmental Health and Climate Change:** Emphasizing the need to address environmental health issues and the health impacts of climate change is more commonly associated with liberal viewpoints, as it involves proactive government intervention in these areas.<br><br>4. **Critical Public Health Objectives:** The specific objectives listed, such as reducing smoking and tobacco use, addressing substance use disorders, tackling maternal mortality (particularly among African American women), reducing firearm-related injuries and deaths, and improving access to nutritious food, are all issues typically emphasized in liberal policy agendas.<br><br>These positions focus on proactive government intervention in healthcare, public health, and environmental issues, which are more aligned with liberal ideologies that advocate for a strong role of government in addressing societal and health-related issues. Conservative viewpoints, in contrast, might emphasize more limited government intervention, individual responsibility, and private sector solutions to these challenges.	liberal
ACP	Performance Measurement Appeals	2019	The text you've provided outlines guidelines for a fair and transparent process in performance measurement programs, particularly for physicians. It focuses on ensuring accuracy in performance ratings, providing a clear explanation of the measurement criteria, allowing for an appeals process, and minimizing administrative burden on physicians. This text does not align neatly with traditional liberal or conservative viewpoints as understood in the political sense.<br><br>The focus on fairness, transparency, and the rights of physicians to challenge and understand their evaluations could be seen as aligning with liberal values, which often emphasize fairness and transparency in processes. On the other hand, the emphasis on detailed procedural guidelines and the potential for reducing bureaucratic hurdles could resonate with conservative values, which often favor efficiency and clarity in processes, especially in professional or business contexts.<br><br>Overall, this text appears to be more focused on professional ethics and good governance practices within the context of healthcare performance measurement, rather than on broader political ideologies. Its primary concern seems to be with the fairness and accuracy of the evaluation process for healthcare professionals, which is a concern that can transcend typical liberal-conservative divides.	neutral
ACP	Coverage of Obesity Treatment	2016	The text you've provided seems to be a policy position from the American College of Physicians (ACP). This position advocates for the analysis of obesity programs in terms of payment methods (individual versus insurance coverage) and their effectiveness in weight loss management. Additionally, it suggests that if insurance coverage for obesity programs is found to be cost-effective, the ACP will advocate for expanded coverage.<br><br>This position aligns more closely with a liberal viewpoint for several reasons:<br><br>1. **Emphasis on Public Health and Preventive Care:** The focus on obesity programs and the consideration of their effectiveness and cost-effectiveness is a hallmark of liberal healthcare policies, which often prioritize public health and preventive care.<br><br>2. **Advocacy for Insurance Coverage:** The suggestion that insurance should cover obesity programs if they are cost-effective reflects a liberal perspective on healthcare. This viewpoint often supports wider insurance coverage for various health services, especially those that contribute to long-term health outcomes.<br><br>3. **Role of Research and Evidence in Policy Making:** The call for analysis and evidence-based decision-making is consistent with a liberal approach to policy, which typically values research and scientific evidence as a basis for healthcare decisions.<br><br>It's important to note that while these aspects lean towards a liberal viewpoint, healthcare policy is complex and can't always be	liberal

			neatly categorized into 'liberal' or 'conservative'. Different aspects of healthcare policy can be supported by individuals across the political spectrum.	
ACP	Unbundling of Preventive and Problem Related Office Visits	2019	The text you provided from the American College of Physicians (ACP) reflects a stance on healthcare policy, specifically regarding payment for preventive and problem-related office visits. It doesn't align strictly with a traditional liberal or conservative viewpoint, as these terms can be broadly interpreted and depend on specific contexts. However, the statement can be analyzed within the framework of common liberal and conservative perspectives on healthcare:<br><br>1. **Liberal Viewpoint**: Liberals generally advocate for broader access to healthcare and support government involvement in ensuring that access. The ACP's advocacy for payers to cover multiple types of primary care services without bundling or rejection of claims aligns with liberal principles of increasing healthcare accessibility and reducing patient costs.<br><br>2. **Conservative Viewpoint**: Conservatives typically emphasize free market solutions in healthcare and might be wary of increased government involvement. However, conservatives also support efficient healthcare delivery. The ACP's stance could align with conservative views if seen as promoting efficiency and reducing bureaucratic hurdles in healthcare delivery.<br><br>In summary, while the statement from the ACP doesn't neatly fit into a strictly liberal or conservative box, aspects of it could be supported by both viewpoints, depending on how the principles of healthcare accessibility, government involvement, and market efficiency are prioritized.	neutral
ACP	Alternative Health Care	2017	The text you've provided seems to focus on a position regarding medical practice, emphasizing the importance of evidence-based therapies and holding all medical practitioners, including those practicing alternative medicine, to the same standards. This stance does not inherently align with a traditional liberal or conservative viewpoint as it primarily concerns medical ethics and standards of practice rather than political ideology.<br><br>The emphasis on evidence-based medicine and uniform standards across medical practices reflects a commitment to scientific rigor and consistency in patient care, values that are generally upheld across various political spectrums. Both liberal and conservative viewpoints might agree on the importance of evidence-based practices in medicine, although their reasons for support might differ based on broader ideological beliefs.<br><br>In summary, this statement is more closely aligned with medical ethics and professionalism than with a specific political ideology. It represents a consensus view in the medical community about the importance of evidence-based practice and equal standards for all medical practitioners.	neutral
ACP	Promotion of the Involvement of Practicing Physicians in NCQA Accreditation Mechanisms	2015	The statement "ACP promotes the involvement of practicing physicians in the development of accreditation measures" does not strongly align with either a traditional liberal or conservative viewpoint. It is primarily a professional stance focused on healthcare and medical accreditation standards. The American College of Physicians (ACP), as a medical organization, advocates for the involvement of medical professionals in creating and maintaining accreditation standards, which is more of a professional and industry-specific perspective rather than a political one.<br><br>Such a stance is based on the belief that those who are directly involved in the practice of medicine are best suited to contribute to	neutral

			the development of standards that govern their profession. This approach is rooted in principles of professional expertise and quality assurance in healthcare, rather than being driven by a specific political ideology.	
ACP	Continuity of Medical Care	2016	The text you've shared appears to be a policy or statement from a medical organization, encouraging its members to ensure the continuity of quality medical care for patients, including those who are home-bound or in nursing homes. This statement doesn't explicitly align with a liberal or conservative viewpoint in the typical political sense. Instead, it reflects a commitment to medical care and patient welfare, which are central to the healthcare profession.<br><br>Both liberal and conservative ideologies generally support the concept of providing quality medical care, although they may differ in their approaches to healthcare policy and funding. This statement seems to be more about professional medical ethics and standards rather than a political stance.	neutral
ACP	Definition of Medical Care	2019	The text you provided discusses the American College of Physicians' (ACP) definition of "medical care" and "health care." This distinction highlights a nuanced understanding of healthcare, where "medical care" is defined as the part under the direct control of physicians, and "health care" encompasses broader social, economic, and environmental factors that influence health but are beyond the direct control of medicine.<br><br>This perspective can align with various political viewpoints, and it's not strictly liberal or conservative. It might resonate more with liberal viewpoints in its acknowledgment of broader social determinants of health, which are often emphasized in progressive health policy discussions. Liberals generally advocate for policies that address these broader determinants, recognizing that health outcomes are influenced by a range of factors beyond just medical treatment.<br><br>On the other hand, the emphasis on the role of physicians and the distinction between direct medical care and broader health influences could also be compatible with conservative perspectives, which often emphasize personal responsibility and the importance of individual medical decisions.<br><br>In summary, this text reflects a multifaceted view of healthcare that acknowledges both the clinical aspects and the broader social determinants. It can be interpreted in ways that align with both liberal and conservative viewpoints, depending on the specific aspects one focuses on.	neutral

ACP	Comparative Effectiveness	2019	The positions outlined here from the American College of Physicians (ACP) generally align more with a liberal viewpoint. This assessment is based on several key aspects of the recommendations:<br><br>1. **Government Involvement and Funding**: The emphasis on federal government support for the comparative effectiveness entity reflects a liberal perspective, which often advocates for stronger government involvement in healthcare and public health initiatives.<br><br>2. **Evidence-based and Scientific Approach**: The focus on strict scientific standards and evidence-based practices is a hallmark of liberal health policy, emphasizing the role of science and research in guiding healthcare decisions.<br><br>3. **Transparency and Stakeholder Involvement**: The call for transparent proceedings and the involvement of various stakeholders, including payers, scientists, providers, and industry representatives, aligns with liberal values of inclusivity and comprehensive policymaking.<br><br>4. **Patient-Centered Care and Equitable Resource Distribution**: The recommendations stress patient-centered care and equitable distribution of healthcare resources. These principles are often emphasized in liberal healthcare policies, which prioritize equity and access for all individuals.<br><br>5. **Cost-Effectiveness Analysis in Healthcare Decisions**: The use of cost-effectiveness as a tool (but not the sole criterion) in healthcare decisions reflects a balance between efficiency and quality of care, which is a common theme in liberal health policy.<br><br>6. **Education and Public Discussion**: The emphasis on educating the public and promoting discussion about healthcare resource allocation reflects a liberal viewpoint of engaging and informing the populace in health policy.<br><br>It's important to note that these positions are focused on improving healthcare quality, efficiency, and accessibility, which are common goals across the political spectrum. However, the methods and principles advocated in these positions are more aligned with liberal viewpoints, especially regarding the role of government, emphasis on evidence and science, and commitment to equitable and patient-centered care.	liberal
ACP	Controlling Health Care Costs: Comparative Effectiveness Research	2022	The text you've provided outlines a perspective focused on the systematic development of comparative clinical and cost-effectiveness evidence for medical services, emphasizing the role of a national entity and the federal government in this process. It also highlights the importance of considering cost alongside clinical effectiveness and suggests that various stakeholders, including health care payers, professionals, patients, employers, and health plans, should use this information in decision-making.<br><br>This perspective aligns more closely with a liberal viewpoint for several reasons:<br><br>1. **Role of Government**: The emphasis on a significant role for the federal government in funding, implementing, and maintaining a comparative effectiveness entity aligns with liberal ideologies that often advocate for a stronger governmental role in healthcare and public welfare.<br><br>2. **Comprehensive Evaluation of Health Services**: The focus on both clinical and cost-effectiveness reflects a liberal inclination towards holistic and inclusive healthcare policies, considering both economic and clinical outcomes.<br><br>3. **Equal Access to Information**: Advocating for improved access to information for all stakeholders, including patients, healthcare providers, and payers, is in line with liberal values of equity and transparency in healthcare.<br><br>4. **Value-based Benefit Design**: The suggestion for employers and health plans to adopt value-based programs based on independent research reflects a liberal approach to health policy, focusing on evidence-based, equitable access to care.<br><br>5. **Cost as a Factor, Not the Sole Criterion**: Acknowledging cost as an important factor but not the sole criterion for evaluating clinical interventions aligns with liberal views that prioritize patient care and outcomes over purely economic considerations.<br><br>However, it's important to recognize that healthcare policy can have elements that appeal to a broad range of political ideologies, and some aspects of this text could be endorsed by individuals with conservative viewpoints as well, especially where it concerns	liberal

			efficiency and cost-effectiveness. Political ideologies are not always clear-cut, and there can be overlaps and nuances in specific policy proposals.	
ACP	Patient Safety in the Office-Based Practice Setting	2017	The recommendations outlined here don't neatly align with either a liberal or conservative viewpoint in the traditional political sense. These recommendations are focused on improving patient safety, physician well-being, and healthcare system efficiency, which are generally seen as non-partisan, professional concerns in the field of healthcare.<br><br>1. **Emphasis on Patient Safety and Medical Error Reporting**: The first recommendation emphasizes the importance of a culture of patient safety in healthcare settings. This includes nonpunitive reporting of medical errors, which is a widely accepted best practice in healthcare management, irrespective of political leanings.<br><br>2. **Addressing Physician Stress and Burnout**: The second recommendation focuses on research and actions to mitigate physician stress and burnout. This is a growing concern in healthcare, acknowledging the human element in providing medical care, and is not particularly political.<br><br>3. **Patient and Family Education**: The third recommendation's focus on patient and family education and health literacy is about empowering patients, a goal that transcends political boundaries.<br><br>4. **Research and Development in Patient Safety**: The fourth recommendation advocates for continued research in patient safety, especially in primary care and ambulatory settings. This is more about scientific and clinical advancement rather than a political issue.<br><br>5. **Team-based Care Models**: The fifth recommendation supports team-based care models like the patient-centered medical home. This approach to care is focused on improving outcomes and efficiency, which can be supported across the political spectrum.<br><br>6. **Health Information Technology Systems**: The sixth recommendation's focus on tailoring health IT systems for patient safety is a technologically oriented goal, not inherently political.<br><br>7. **National Effort to Prevent Patient Harm**: The seventh recommendation proposes a national effort to prevent patient harm. While the idea of a national entity could be seen as aligning with more centralized approaches to healthcare, the goal itself—patient safety—is broadly supported.	neutral

			In summary, these recommendations are aligned more with professional and healthcare quality improvement goals rather than being specifically liberal or conservative in nature. They reflect common themes in contemporary healthcare discussions, such as patient safety, physician well-being, and the effectiveness of healthcare delivery systems.	
ACP	Primary Care in High Quality-Low Cost Areas	2022	The text stating that the American College of Physicians (ACP) supports federal legislation to fund research reflecting the value and cost-effectiveness of primary care appears to be more aligned with a liberal viewpoint. This alignment is suggested by the emphasis on federal funding for healthcare research, a position typically associated with liberal policies. Liberals often advocate for greater government involvement in healthcare, including funding for research to improve the efficiency and effectiveness of medical care. This approach contrasts with conservative viewpoints, which generally favor less government intervention in healthcare and more market-based solutions. However, it's important to note that viewpoints on healthcare can vary within both liberal and conservative circles, and this assessment is a generalization.	liberal
ACP	Developing Methods and Resources for Small Practices To Fairly Negotiate with Accountable Care Organizations	2022	The text you provided seems to align more closely with a liberal viewpoint. It discusses the support for the development of methods and resources to assist small practices in negotiating with Accountable Care Organizations (ACOs). This advocacy for smaller practices in the healthcare system suggests a focus on equity and support for smaller entities, which is more commonly associated with liberal ideologies. Additionally, the call for implementation of these methods with the Centers for Medicare and Medicaid Services and other insurers indicates a preference for active government involvement in healthcare, a stance typically associated with liberal views. However, it's important to note that such issues can be complex and might not align perfectly with traditional political labels.	liberal

ACP	Joint Principles for Accountable Care Organizations	2022	The text you've provided outlines the structure and payment policies of Accountable Care Organizations (ACOs). It's focused on healthcare delivery, quality, efficiency, and payment models within these organizations.<br><br>The content of this text can align with both liberal and conservative viewpoints, albeit in different aspects:<br><br>1. **Liberal Viewpoint Alignment**:<br>- Emphasis on **accessible, effective, team-based integrated care** and **cultural competence** is often aligned with liberal priorities in healthcare, which focus on inclusivity and comprehensive care.<br>- The focus on **community engagement and addressing community needs** (e.g., interfacing with state programs and public education entities) aligns with the liberal approach of community-based healthcare solutions.<br>- The **voluntary nature of participation** and the emphasis on **patient and family engagement** in decision-making processes reflect a patient-centric approach that is often championed in liberal healthcare policies.<br><br>2. **Conservative Viewpoint Alignment**:<br>- The **administrative structure and efficiency**, including **transparent processes for payment and quality management**, can resonate with conservative ideals of accountability and efficiency in healthcare.<br>- The mention of **protecting ACOs from existing antitrust and gain-sharing laws** might align with the conservative viewpoint favoring less government regulation in business and healthcare operations.<br>- The emphasis on **variety in payment approaches**, including fee-for-service and shared savings, and the protection against “insurance risk” align with conservative principles of market-driven solutions and financial responsibility in healthcare.<br><br>In summary, the text combines elements of patient-centered care, community engagement, efficiency, transparency, and flexible payment models, which can resonate with both liberal and conservative ideals in different ways. It reflects a blended approach, aiming to address a wide range of priorities in the healthcare sector.	neutral
ACP	Development of the Accountable Care Organization Model	2022	The text you provided outlines principles and guidelines for Accountable Care Organizations (ACOs) and seems to focus on patient-centered care, efficiency, quality improvement, and transparent administration in healthcare. It emphasizes the importance of physician involvement in policy decisions, patient input, technological integration, and fair payment models.<br><br>This text doesn't align clearly with a traditional liberal or conservative viewpoint, as it combines elements that both sides might agree or disagree with:<br><br>1. **Liberal Viewpoints**:<br>- Emphasis on patient-centered care and quality improvement aligns with liberal ideals of comprehensive and accessible healthcare.<br>- The call for transparency and public involvement in decision-making reflects a liberal preference for openness and accountability in institutions.<br>- Encouraging the use of health information technology aligns with progressive attitudes towards embracing technology for societal benefit.<br><br>2. **Conservative Viewpoints**:<br>- The voluntary nature of physician participation and recognition of the importance of small practices might appeal to conservative ideals of individual choice and skepticism towards large, centralized systems.<br>- Emphasizing the protection of practices from undue influence by larger entities could align with conservative principles of limiting the power of large institutions.<br>- The focus on efficient use of resources and minimizing administrative burdens can resonate with conservative priorities of fiscal responsibility.<br><br>3. **Neutral/General Healthcare Management Principles**:<br>- Many points, like promoting efficient and coordinated care, are general healthcare management principles that aren't inherently political.<br><br>In summary, the text represents a blend of healthcare management principles with aspects that could be appealing to both liberal and conservative perspectives, depending on individual priorities and interpretations within the broader context of healthcare policy.	neutral

ACP	Research	2019	The text you provided does not align exclusively with either a liberal or conservative viewpoint. Instead, it focuses on ethical standards and best practices in scientific and medical research. The principles outlined, such as the importance of honesty, integrity, rigorous peer review, transparency in reporting, and the need to prioritize patient outcomes and generalizable knowledge over personal recognition or financial gain, are broadly accepted across the scientific community regardless of political affiliation.<br><br>This approach to research ethics emphasizes universal values in scientific inquiry, such as the importance of honesty in reporting results, the integrity of the research process, ethical considerations involving human subjects, and the responsibility of scientists to maintain objectivity and avoid conflicts of interest. These principles are foundational to the scientific method and are generally endorsed across the political spectrum, as they focus on the integrity and effectiveness of scientific research rather than on ideological or political considerations.	neutral
ACP	Protection of Human Subjects	2019	The text provided does not neatly align with a strictly liberal or conservative viewpoint. Instead, it focuses on ethical principles and practices in medical research. Key elements include:<br><br>1. **Responsibility for Valid and Ethical Research**: Emphasizing the need for medical professionals and researchers to ensure research validity, ethical conduct, and significant value.<br><br>2. **Minimizing Risk and Maximizing Safety**: The importance of planning research to reduce subject risk, increase safety, and achieve a beneficial risk-benefit ratio.<br><br>3. **Fair Distribution of Benefits and Risks**: Stating that research benefits and risks must be fairly distributed, with special attention to avoiding exploitation of vulnerable populations.<br><br>4. **Conflict of Interest in Research**: Addressing the potential conflicts between a researcher's role and patient care responsibilities.<br><br>5. **Voluntary Participation and Informed Consent**: Highlighting the necessity for voluntary participation in research, informed consent, and the right to withdraw at any time.<br><br>6. **Protection of Vulnerable Groups**: Special measures for groups more vulnerable to coercion or undue influence, like children or economically disadvantaged persons.<br><br>7. **Research Involving Cognitively Impaired Individuals**: Discussing ethical considerations and consent processes for research involving individuals with impaired decision-making capacities.<br><br>8. **Ethical Oversight and Independent Review**: The role of Institutional Review Boards (IRBs) in ensuring ethical standards, protecting human subjects, and maintaining appropriate benefit-risk ratios.<br><br>9. **Physician Responsibility in Research**: Physicians' obligations to avoid conflicts of interest, maintain ethical standards, and ensure patient safety in research contexts.<br><br>10. **Privacy and Confidentiality**: Compliance with privacy laws and guidelines, like the HIPAA Privacy Rule, in the context of research.<br><br>The focus on ethical considerations, fairness, subject safety, and informed consent transcends traditional political alignments. It aligns more with universal principles of medical ethics and human rights, emphasizing the importance of ethical conduct in medical research, regardless of political ideology.	neutral
ACP	Innovative Medical Therapies	2019	The text you've provided appears to take a neutral, scientific approach towards the topic of innovative medical therapies, rather than aligning explicitly with a liberal or conservative viewpoint. It focuses on the ethical considerations, safety, and regulatory aspects of medical innovation, emphasizing the importance of careful assessment, informed consent, and adherence to regulatory standards. This approach is typical of scientific and medical discourse, which prioritizes patient safety, evidence-based practice, and ethical considerations over political ideologies.	neutral

ACP	Scientific Publication	2019	The text you've provided appears to be a set of ethical guidelines or standards for authorship and research integrity, likely in a medical or scientific context. It focuses on the responsibilities of authors in research, including honesty in reporting, disclosure of conflicts of interest, ethical conduct in publication, and the importance of transparency in clinical trials.<br><br>This text does not align specifically with a liberal or conservative political viewpoint. Instead, it is centered on principles of scientific integrity and ethical conduct in research. These principles are universally recognized in the scientific community and are not typically associated with a particular political ideology. The emphasis is on ensuring the accuracy and integrity of scientific research, which is a foundational aspect of the scientific method and scholarly communication, transcending political affiliations or ideologies.	neutral
ACP	Public Announcement of Research Discoveries	2019	The text you've provided doesn't align distinctly with either a liberal or conservative viewpoint. Instead, it focuses on the ethics and responsibilities of researchers and institutions in communicating scientific findings to the public and media. The emphasis is on caution, precision, and responsibility in public announcements to avoid misinterpretation and misinformation. This approach is consistent with general principles of scientific communication and integrity, transcending specific political ideologies.	neutral
ACP	Financial Support of Medical Research	2016	The text you provided appears to advocate for ongoing research with adequate financial support, caution against sudden changes in this support, and suggest a merit-based allocation of governmental medical research funds. This viewpoint does not align squarely with traditional liberal or conservative viewpoints as it addresses specific policies related to medical research funding rather than broader political ideologies.<br><br>In general:<br><br>- **Liberal Viewpoint**: Tends to support increased government involvement in various sectors, including healthcare and research. Liberals might advocate for robust funding for medical research and equitable distribution of resources.<br><br>- **Conservative Viewpoint**: Typically favors limited government intervention and might be more cautious about government spending, including in areas like medical research. However, conservatives also value the efficient use of resources and might support merit-based allocation of funds.<br><br>This statement from the American College of Physicians (ACP) focuses on the practical aspects of research funding, emphasizing the need for stable support, merit-based allocation, and broad distribution of funds. It doesn't strictly adhere to a particular political ideology but rather addresses the operational and ethical considerations in medical research funding.<br><br>Political alignment can be complex, and specific policy positions like this one can find support across the political spectrum depending on the context and the underlying principles being emphasized (e.g., efficiency, equity, innovation).	neutral
ACP	Supporting Research Into the Therapeutic Roles of Marijuana	2019	The positions outlined here generally align more with a liberal viewpoint, particularly in the context of U.S. politics.<br><br>1. **Support for Scientific Evaluation and Research of Marijuana**: Liberal viewpoints often emphasize the importance of scientific research and evidence-based policy, especially in areas like drug policy. The call for rigorous scientific evaluation and increased research (Positions 1, 1a, and 1b) to understand the therapeutic benefits of marijuana fits within this framework.<br><br>2. **Encouragement of Nonsmoked Forms of THC**: This stance (Position 2), focusing on the therapeutic value of nonsmoked forms of THC, aligns with liberal views prioritizing public health and safety while recognizing the potential medical benefits of cannabis derivatives.<br><br>3. **Support for the Current Process of Obtaining Research-Grade Cannabis**: Position 3's support for the current federal process is a nuanced stance. It could be seen as a conservative approach in maintaining existing structures, but in the context of advocating for further research, it aligns more with liberal perspectives.<br><br>4. **Evidence-Based Review of Marijuana's Legal Status**: The call for an evidence-based review of marijuana's classification as a Schedule I controlled substance (Position 4) aligns with liberal positions favoring drug policy reform based on scientific findings and public health considerations.<br><br>5. **Support for Exemption from Prosecution for Medical Use**: Position 5's strong support for protecting physicians and patients involved in the medical use of marijuana under state laws is a distinctly liberal stance. It emphasizes patient rights, medical autonomy, and the decriminalization aspects of marijuana use, all of which are more commonly associated with liberal viewpoints.	liberal

			These positions collectively indicate a focus on scientific evidence, public health, and reform of drug laws, particularly in relation to marijuana, which are more characteristic of liberal rather than conservative views. However, it's important to note that the distinction between liberal and conservative viewpoints can vary depending on regional and cultural contexts.	
ACP	Steroids	2016	The statement "ACP opposes the use of anabolic steroids to enhance athletic performance" does not inherently align with a specific liberal or conservative viewpoint. It appears to be a position based on health or ethical considerations rather than political ideology.<br><br>The American College of Physicians (ACP) is a professional organization for internal medicine physicians. Their stance against the use of anabolic steroids for enhancing athletic performance is likely grounded in medical concerns about the health risks associated with steroid use, as well as the principles of fair play in sports.<br><br>Both liberal and conservative viewpoints generally support fair competition in sports and are concerned about public health. However, the reasons for their support might differ based on their broader political and social perspectives. Liberals might focus more on the public health aspect and the importance of government regulation to protect individuals, while conservatives might emphasize personal responsibility and the integrity of sport.<br><br>Overall, this statement is more about medical ethics and health policy than about aligning with a specific political ideology.	neutral
ACP	Genetic Information	2019	The positions listed regarding genetic discrimination generally align with a liberal viewpoint. This viewpoint emphasizes the protection of individual privacy and non-discrimination, particularly in areas like health insurance and employment. Key aspects of this perspective include:<br><br>1. Prohibiting insurance providers from using genetic information to deny or limit health coverage, affect eligibility, or determine premiums.<br>2. Preventing employers from using genetic information in employment decisions.<br>3. Restricting insurers and employers from requiring genetic testing.<br>4. Requiring informed consent for any disclosure of genetic information.<br>5. Advocating for comprehensive federal protection against genetic discrimination.<br><br>These positions reflect a liberal approach, focusing on safeguarding individual rights and privacy, promoting equality, and advocating for federal-level solutions to address disparities and inconsistencies in state laws. This contrasts with a conservative viewpoint, which might emphasize more limited government intervention in private business practices and a greater reliance on state-level legislation.	liberal

ACP	Assessment of Health Care Technology	2015	The text you've provided presents a viewpoint that aligns with a mix of both liberal and conservative perspectives, reflecting a pragmatic approach to healthcare technology and policy.<br><br>From a liberal viewpoint:<br>1. The emphasis on rigorous assessment of new technologies and pharmaceuticals for safety and effectiveness aligns with a liberal perspective that prioritizes evidence-based approaches and public health safety.<br>2. The support for federal funding for technology assessment programs suggests a belief in the role of government in healthcare, a common liberal stance.<br>3. The focus on benefits to patients over cost considerations reflects a patient-centric approach often found in liberal healthcare policies.<br><br>From a conservative viewpoint:<br>1. The insistence on evaluations being unassociated from reimbursement decisions resonates with conservative values of market-driven healthcare and limiting government interference in pricing.<br>2. The support for the use of technology assessments by third-party payers, Medicare, and Medicaid in making coverage and reimbursement decisions suggests a blend of public and private sector roles, which is a more conservative approach.<br>3. The call for cost-effectiveness assessments, while balanced against patient benefits, also reflects a conservative perspective on fiscal responsibility and efficiency in healthcare spending.<br><br>Overall, the text represents a balanced approach, advocating for evidence-based, patient-centered healthcare while recognizing the importance of cost-effectiveness and the roles of both public and private sectors in healthcare.	neutral
ACP	Support Food and Drug Administration's (FDA) Attempt to Regulate Tobacco	2019	The text stating that the ACP (American College of Physicians) strongly supports the Commissioner of the FDA in having nicotine declared an addictive substance aligns more closely with a liberal viewpoint. This perspective typically emphasizes public health initiatives, government intervention in health matters, and strict regulations on substances that can harm health, like nicotine. Liberals often advocate for policies that protect public health and support regulatory agencies like the FDA in their efforts to control harmful substances.<br><br>In contrast, a conservative viewpoint generally favors less government intervention in personal choices and business practices, and might be more inclined to oppose increased regulation on substances like nicotine, seeing it as an overreach of government power.<br><br>It's important to note, however, that public health issues can sometimes transcend typical political divisions, and there can be a wide range of opinions within each political ideology.	liberal
ACP	Inpatient Use of Pharmaceutical Aids of Smoking Cessation	2019	The statement "Hospitals should be encouraged to approve pharmaceutical aids to smoking cessation for inpatient use" does not explicitly align with a specific political viewpoint, either liberal or conservative. It focuses on healthcare policy, particularly on the promotion of smoking cessation aids in hospitals.<br><br>In the context of U.S. politics:<br><br>1. **Liberal Viewpoint**: Generally, liberals tend to support proactive healthcare measures, including the use of pharmaceutical aids for smoking cessation. They often advocate for comprehensive healthcare services and might view such measures as beneficial for public health.<br><br>2. **Conservative Viewpoint**: Conservatives might support this statement as well, especially if they see it as a cost-effective healthcare strategy or as a means of reducing long-term healthcare costs associated with smoking-related illnesses. However, some conservatives might be cautious about endorsing specific pharmaceutical solutions if they perceive it as regulatory overreach or as an undue influence of pharmaceutical companies in healthcare decisions.<br><br>Overall, the statement can find support across the political spectrum, as it addresses a public health issue rather than a strictly partisan one. It reflects a focus on improving health outcomes and can be seen as a pragmatic approach to healthcare.	neutral

ACP	Tobacco-Divestiture	2015	The statement "Health related industries should consider divesting themselves of investments in companies which provide major support for the promotion of tobacco use" generally aligns more closely with a liberal viewpoint.<br><br>Liberal perspectives typically emphasize public health and welfare, advocating for policies that protect individuals from harmful substances like tobacco. Divestment from tobacco companies is seen as a way to reduce the influence and reach of these companies, thus potentially decreasing tobacco use and improving public health. This aligns with the liberal focus on regulatory measures for public health.<br><br>On the other hand, conservative viewpoints often prioritize free-market principles and individual responsibility. From a conservative perspective, businesses, including those in health-related industries, should be free to invest as they see fit without external pressures to divest from certain industries. The emphasis here is more on individual choice and market freedom, rather than on top-down regulatory or policy-driven approaches.<br><br>It's important to note that these are generalizations and that views can vary widely within each political spectrum. Additionally, public health issues like tobacco use can sometimes transcend traditional political boundaries, with both conservatives and liberals finding common ground on certain aspects of such issues.	liberal
ACP	Contribution to Death Certificates	2015	The text you provided seems to focus on public health policy related to tobacco use, particularly in the context of death certificates. This type of policy initiative is generally associated with public health advocacy and may align more closely with a liberal viewpoint. Liberal perspectives often emphasize the role of government and public institutions in addressing health issues, promoting public awareness, and implementing regulations for public welfare.<br><br>Conservative viewpoints, on the other hand, might be less inclined to support such measures if they perceive them as overly regulatory or as an expansion of government intervention in personal or business practices. However, it's important to note that public health issues can sometimes transcend traditional political divides, especially when they pertain to widespread concerns like the impact of tobacco use.<br><br>The American College of Physicians (ACP), as mentioned in your text, is a professional organization that typically focuses on medical practice and public health issues. Their encouragement of tracking tobacco use in death certificates is likely motivated by a desire to better understand and mitigate the health impacts of tobacco.<br><br>In summary, while this text might align more closely with a liberal perspective due to its focus on public health and regulatory measures, the issue of tobacco use and its health consequences can be of concern across the political spectrum.	liberal

ACP	Tobacco Control and Prevention	2022	The text you've provided aligns more closely with a liberal viewpoint. It emphasizes the role of government intervention and regulation in public health issues, particularly in the area of tobacco control. Key aspects of this viewpoint include:<br><br>1. **Support for Government Intervention**: Advocating for comprehensive state and federal efforts to control tobacco use, especially among young people, is a hallmark of liberal policy, which often emphasizes the role of government in addressing public health concerns.<br><br>2. **Healthcare and Insurance Involvement**: Calling for public and private insurers to provide comprehensive tobacco cessation benefits reflects a liberal approach to healthcare, where there is an emphasis on broad access to healthcare services, including preventive measures.<br><br>3. **Funding for Public Health Initiatives**: The proposal that states commit to funding tobacco control at specific levels and that tobacco-generated revenue be used for tobacco control efforts aligns with liberal perspectives on public health funding.<br><br>4. **Educational and Prevention Efforts**: The focus on youth education, prevention, and enhanced antismoking campaigns is consistent with liberal policies that prioritize education and preventative measures in public health.<br><br>5. **Regulatory Measures**: Advocating for the FDA to ban certain tobacco product flavorings and to regulate electronic nicotine delivery systems is aligned with a liberal approach that favors regulation for public health benefits.<br><br>6. **Smoke-Free Laws**: The call for comprehensive smoke-free laws in non-residential and residential areas is an example of a liberal approach to public health, where government regulations are used to protect public welfare.<br><br>7. **Media Responsibility**: Urging the media industry to discourage tobacco use and highlight its dangers, particularly to youth, reflects a liberal perspective on the role of media in public health and societal issues.<br><br>Overall, these points emphasize a proactive government role in healthcare, regulation for public welfare, and comprehensive public health initiatives, which are more commonly associated with liberal rather than conservative viewpoints. Conservative viewpoints, in contrast, often emphasize limited government intervention, individual responsibility, and private sector solutions over public sector interventions.	liberal
ACP	Electronic Nicotine Delivery Systems	2014	The text you provided outlines various recommendations from the American College of Physicians (ACP) regarding electronic nicotine delivery systems (ENDS), commonly known as e-cigarettes. These recommendations include extending regulatory authority, banning flavored tobacco products, supporting taxes on tobacco products, restricting promotion and advertising, extending clean air laws to ENDS, and funding research on the health effects of ENDS.<br><br>This text generally aligns more with a liberal viewpoint for several reasons:<br><br>1. **Regulation and Government Intervention:** The recommendations advocate for increased government regulation and intervention in the tobacco and ENDS industries. This is typically more aligned with liberal ideologies, which often support more government involvement in regulating industries to protect public health and safety.<br><br>2. **Public Health Emphasis:** The focus on public health, especially concerning the youth and the environment (like extending clean air laws), is a common feature of liberal policies. Liberals often prioritize public health measures and preventive care.<br><br>3. **Taxation as a Deterrent:** The suggestion to use taxation as a means to discourage use among children and adolescents is in line with liberal approaches of using fiscal policies to drive social outcomes.<br><br>4. **Research and Evidence-Based Policy:** The emphasis on funding research to understand the health effects and creating policies based on scientific evidence is characteristic of a liberal approach, which typically values scientific research in policy-making.<br><br>However, it's important to note that public health issues often transcend simple political categorizations, and there may be conservatives who also support these measures due to concerns about public health and the welfare of children and adolescents. Additionally, some liberal viewpoints might oppose certain aspects of these recommendations, especially if they view them as	liberal

			infringing on personal freedoms. Therefore, while these recommendations mostly align with liberal viewpoints, they can have cross-partisan support or opposition depending on the specific context and framing of the issues.	
ACP	Minimum Legal Age to Purchase Tobacco	2018	The statement about the American College of Physicians (ACP) recommending raising the minimum legal sale age to purchase all tobacco products to 21 years aligns more closely with a public health perspective rather than being distinctly liberal or conservative. Public health policies often focus on the well-being and health of the general population and can be supported by individuals across the political spectrum.<br><br>In the context of American politics:<br><br>1. **Liberal Viewpoint**: Generally, liberals in the U.S. tend to support regulations aimed at promoting public health and safety. This includes support for measures that restrict the sale of potentially harmful products like tobacco to minors. They often view such regulations as necessary for the protection of young people and the broader community.<br><br>2. **Conservative Viewpoint**: Conservatives traditionally favor less government intervention in business and personal choices. However, when it comes to issues of public health and especially the protection of minors, there can be significant support among conservatives for regulations like raising the legal age for tobacco sales. The focus here is often on protecting young people from making decisions that could have long-term negative health impacts.<br><br>In this case, the policy recommended by the ACP could potentially garner support from both sides, as it focuses on public health and protecting young people, goals that can resonate across the political spectrum. It's important to recognize that such issues can transcend simple liberal-conservative dichotomies, especially when they focus on widely acknowledged public health concerns.	neutral

ACP	Labeling	2015	The text you've provided expresses a viewpoint that supports stronger package labeling on all tobacco products to inform people of the health hazards associated with smoking, and suggests updating this labeling as new scientific evidence becomes available.<br><br>This viewpoint doesn't neatly align with traditional liberal or conservative ideologies in a strict sense. Both liberals and conservatives might support such measures for different reasons:<br><br>- **Liberal Viewpoint**: Liberals generally advocate for strong public health measures and may support such labeling as part of broader efforts to promote public health and safety. They often favor government intervention in public health issues and might see this as a way to reduce smoking rates and associated healthcare costs.<br><br>- **Conservative Viewpoint**: Some conservatives might also support such measures, especially if they view it as a means of promoting personal responsibility and informed consumer choice. However, other conservatives might oppose it if they see it as excessive government regulation interfering with business.<br><br>In summary, the viewpoint expressed in the text can potentially find support across the political spectrum, depending on the specific arguments and values emphasized. It's more a public health issue than a strictly partisan one.	neutral
ACP	Tobacco Marketing and Promotion	2017	The text you've provided, detailing the American College of Physicians' (ACP) stance on tobacco marketing and promotion, aligns more closely with a liberal viewpoint. This alignment can be deduced from several key aspects:<br><br>1. **Emphasis on Public Health and Prevention**: The focus on educating youth about the dangers of tobacco and discouraging its use aligns with liberal perspectives that prioritize public health initiatives and preventive care.<br><br>2. **Concern for Vulnerable Groups**: The explicit mention of protecting minorities and women from targeted tobacco advertising reflects a liberal concern for the welfare of groups that are often marginalized or disproportionately affected by harmful practices.<br><br>3. **Regulatory Stance on Corporate Practices**: The opposition to medical societies providing mailing lists to companies that advertise tobacco in their magazines indicates a regulatory approach to corporate practices, which is more typical of liberal ideologies.<br><br>In contrast, conservative viewpoints might emphasize personal responsibility and less government or societal intervention in private industries like tobacco. They might also be less inclined to support regulations that limit corporate advertising practices, viewing such measures as overreach.<br><br>However, it's important to note that public health issues like tobacco use can sometimes transcend traditional political divides, with both liberals and conservatives recognizing the health risks associated with tobacco.	liberal
ACP	Congress to End Subsidies Related to Tobacco Production and Distribution	2019	The statement, "ACP believes that Congress should pass legislation ending all subsidies to the tobacco industry," generally aligns with a liberal viewpoint. Liberal ideology often emphasizes public health and welfare, and tends to support government intervention in industries that are perceived as harmful to health, such as the tobacco industry. Ending subsidies to the tobacco industry is consistent with these principles, as it would represent a move to discourage tobacco use and promote public health.<br><br>Conservative viewpoints, on the other hand, often prioritize free market principles and limited government intervention in business. Conservatives might argue that ending subsidies interferes with the free market and could be seen as overreach by the government.<br><br>It's important to note that these are general trends and there can be exceptions. Political beliefs can be complex, and individuals or groups may hold a mix of liberal and conservative views on different issues.	liberal

ACP	Support FDA Regulation	2017	The text you provided reflects a viewpoint that aligns more closely with liberal ideologies. Liberal perspectives often emphasize public health initiatives, government regulation of potentially harmful substances, and actions to protect vulnerable populations (like underage individuals) from health risks. In this case, the support for the Food and Drug Administration's regulation of tobacco, advocating for restrictions on access to tobacco products by underage people, and the call for the elimination of government subsidies for tobacco products are consistent with such liberal viewpoints.<br><br>These positions also align with public health and preventive care principles, focusing on reducing the health risks associated with tobacco use. The advocacy for higher excise taxes and restrictions on smoking in workplaces further supports public health goals, which are typically more emphasized in liberal policies.<br><br>Conservative viewpoints, on the other hand, tend to emphasize less government intervention in businesses, including regulations, and might be more inclined to support individual freedoms regarding lifestyle choices, including the use of tobacco products. However, it's important to note that public health issues like tobacco use can sometimes transcend traditional liberal-conservative divides, with members of both groups recognizing the health risks involved.	liberal
ACP	Increasing Federal Excise Taxes on Alcohol and Tobacco	2017	The statement about increasing the federal excise tax on alcohol and tobacco to fund tobacco cessation and alcohol abstinence programs generally aligns with a liberal viewpoint. Liberal perspectives typically advocate for higher taxes, particularly on products deemed harmful, to fund public health initiatives. This approach reflects a belief in proactive government intervention to address health issues and the social determinants of health.<br><br>In contrast, conservative viewpoints usually favor lower taxes and limited government intervention in the market. Conservatives might argue that such tax increases unfairly burden consumers or businesses and prefer individual responsibility over government-led health initiatives.<br><br>However, it's important to note that views on specific issues like this can vary within each political spectrum, and not all liberals or conservatives may align perfectly with these general tendencies.	liberal
ACP	Smoking	2017	The statement "ACP urges all physicians to stop smoking and to prohibit smoking in their offices" does not inherently align with a specific political viewpoint, be it liberal or conservative. It is a health-oriented directive aimed at reducing smoking and its associated health risks. The American College of Physicians (ACP) is focused on improving health and healthcare, which transcends political ideologies. Health recommendations like these are generally based on medical evidence and public health concerns rather than political beliefs.	neutral
ACP	Tobacco-Youth	2015	The text you've provided doesn't neatly align with a traditional liberal or conservative viewpoint, as it encompasses issues that can be interpreted differently across the political spectrum.<br><br>The opposition to legislation that would criminalize sting operations to identify illegal cigarette sales to minors might be seen as a conservative viewpoint, as conservatives often support law enforcement tactics and methods. However, it could also be seen as liberal, as liberals generally advocate for measures to protect children and reduce the influence of tobacco.<br><br>The encouragement of Parent-Teacher Associations and teachers' unions to make schools tobacco-free could be interpreted as a liberal stance, given the focus on public health and education, areas typically emphasized by liberals. However, it could also align with conservative values that prioritize the well-being of children and community standards.<br><br>In summary, the text reflects a blend of concerns and values that can be found in both liberal and conservative ideologies, focusing primarily on public health, child welfare, and education.	neutral

ACP	Exporting Tobacco Products	2015	The statements you've provided from the American College of Physicians (ACP) predominantly align with a liberal viewpoint. Here's a breakdown of why:<br><br>1. **Advocating for Trade Policy Consistent with Health Policy**: This perspective often aligns with liberal ideology, which tends to emphasize public health and welfare over commercial interests, especially in matters like tobacco exports that have a significant impact on global health.<br><br>2. **Support for Federal Legislation on Health Warning Labels**: This aligns with a liberal viewpoint that advocates for government intervention and regulation in public health matters. The emphasis on labeling in native languages and adherence to U.S. standards when exporting reflects a concern for global health standards, a typical liberal stance.<br><br>3. **Restrictions on Foreign Advertising by U.S. Tobacco Producers**: This position, which seeks to limit advertising and promote public health over commercial interests abroad, aligns with liberal ideologies that favor regulation and public welfare.<br><br>4. **Opposition to U.S. Government Efforts to Relax Tobacco Regulations in Other Countries**: This stance is consistent with a liberal viewpoint, which often opposes deregulation in areas that affect public health and welfare.<br><br>5. **Opposition to the Importation and Exportation of Tobacco Products**: This position, which supports making U.S. foreign export policy more consistent with domestic health policy, aligns with liberal views favoring regulation and public health over free trade in harmful products.<br><br>In general, these points reflect a liberal perspective, focusing on public health, regulation, and global welfare, often at the expense of free trade and commercial interests.	liberal
ACP	Position Paper on Health Care for Our Nation's Veterans	2021	The recommendations from the American College of Physicians (ACP) regarding the Veterans Health Administration (VHA) don't align neatly with a strictly liberal or conservative viewpoint. Rather, they reflect a blend of principles that can be found across the political spectrum. Here's a breakdown:<br><br>1. **Support for the VHA's Mission and Funding**: This aligns with a generally liberal viewpoint that emphasizes the role of government in providing healthcare. However, conservatives who prioritize veterans' issues may also support this.<br><br>2. **Veterans Community Care Program as a Supplement**: This recommendation balances liberal support for the VHA with a conservative preference for private healthcare options, suggesting a middle-ground approach.<br><br>3. **Seamless Care Coordination and Voluntary Participation**: These points emphasize efficiency and choice, which are often conservative values, but they're situated within a largely government-run system, which aligns more with liberal views.<br><br>4. **Full Coverage for Higher-Income Veterans and Reduced Copayments**: This is a liberal-leaning recommendation, emphasizing broader coverage and reduced costs for individuals.<br><br>5. **Collaboration to Reduce Administrative Burnout**: This point could appeal across the political spectrum, as it addresses efficiency and the well-being of healthcare professionals.<br><br>6. **Recruitment and Retention Best Practices**: This leans towards a liberal viewpoint in terms of advocating for increased government spending and resources in healthcare.<br><br>7. **Support for Education and Research**: The emphasis on education and research is generally a liberal viewpoint, though the specific focus on veterans might also find support among conservatives.<br><br>8. **Patient-Aligned Care and Research Funding**: This aligns with a liberal approach to healthcare, focusing on patient-centered care and government-funded research.<br><br>Overall, these recommendations embody a nuanced, pragmatic approach, recognizing the importance of both government and private sector roles in providing healthcare to veterans. They don't squarely fit into a single political ideology, illustrating how veterans' healthcare can be a unifying, bipartisan issue.	neutral

ACP	Veterans Community Care Program	2023	The text you provided outlines two positions related to healthcare for veterans, specifically focusing on the integration and access of non-Veterans Affairs (VA) physicians and services into the Veterans Health Administration (VHA) system. These positions are from the American College of Physicians (ACP).<br><br>Position 3 advocates for allowing non-VA physicians to prescribe medications for veterans eligible for non-VHA care, with those prescriptions being filled either within the VHA system or outside in urgent situations. It also suggests that non-VA physicians should have access to and utilize VHA formularies, and have a process to petition for the use of non-formulary drugs for certain patients.<br><br>Position 4 advocates for allowing non-VHA physicians to order laboratory and radiologic tests, seek subspecialty consultations, and treatment at VHA facilities for veterans receiving non-VHA care. It also emphasizes the importance of informing veterans about the circumstances under which the VHA will cover services performed outside the VHA system.<br><br>Both positions focus on improving access to healthcare for veterans, particularly in terms of integrating non-VHA resources and ensuring veterans receive comprehensive care. These positions do not align clearly with a traditional liberal or conservative viewpoint as they primarily address healthcare administration and veterans' access to services, rather than broader ideological issues.<br><br>Healthcare policy can be viewed through various political lenses, but these specific proposals are more centered on practical healthcare management and veterans' welfare, areas that often see bipartisan support. The emphasis is on effective, accessible healthcare for veterans, a goal that both liberal and conservative groups might agree with, although they may differ in their approaches to achieving it.	neutral
ACP	Position Statement on Promoting Policy Standards for Workplace Violence Prevention and Management	2021	The text provided primarily aligns with a viewpoint focused on workplace safety and the well-being of health care workers, rather than fitting neatly into a traditional liberal or conservative political viewpoint. Here are some key aspects:<br><br>1. **Emphasis on Safety and Health**: The focus on reducing workplace violence and ensuring a safe working environment for physicians and other health care workers is a concern that transcends political lines. Both liberal and conservative viewpoints generally support the health and safety of workers, though they may differ in their approaches to regulation and implementation.<br><br>2. **Support for Legislative Efforts and Regulations**: The support for legislative efforts to reduce workplace violence and the call for action by federal agencies like the Occupational Safety and Health Administration (OSHA) can be seen as aligning with more regulatory or interventionist approaches typically associated with liberal viewpoints. However, conservatives may also support such measures, particularly when they address specific and pressing issues like workplace violence in healthcare.<br><br>3. **Call for Research and Data-Driven Approaches**: The emphasis on research and data to understand and address workplace violence is a pragmatic approach that can appeal across the political spectrum. It suggests a reliance on evidence and scientific inquiry, which is a neutral stance politically.<br><br>4. **Commitment to Worker Rights and Institutional Responsibility**: The belief that healthcare workers have a right to work in safe conditions and that institutions have a duty to ensure safety can resonate with both liberal and conservative values. Liberals might emphasize the rights of workers and the responsibility of institutions, while conservatives might focus on the importance of maintaining a productive and safe work environment.<br><br>Overall, the text seems to prioritize practical solutions and safety measures in the healthcare workplace, which is a concern that both liberals and conservatives might find important, albeit potentially for different reasons or with different proposed solutions.	neutral

ACP	Family Violence	2016	The text you provided is a statement from the American College of Physicians (ACP) supporting the American Medical Association's (AMA) national campaign against family violence. It outlines a series of steps that internists are encouraged to take in order to address and reduce the prevalence of family violence. This includes becoming knowledgeable about diagnosis and treatment, understanding legal aspects, participating in community prevention activities, and engaging in research on family violence.<br><br>This statement aligns more closely with a socially progressive or liberal viewpoint, as it emphasizes proactive community engagement, legal awareness, and social responsibility in addressing a significant public health and social issue. Liberal perspectives often prioritize social justice, public health initiatives, and community support networks. The focus on research, education, and community involvement in tackling a social problem like family violence aligns well with these principles.<br><br>However, it's important to note that the issue of family violence and its prevention can transcend traditional political boundaries. Efforts to combat family violence may find support across a wide political spectrum, as it's a concern that affects individuals and communities regardless of political affiliation. While the approach outlined in the statement may resonate more with liberal values, the fundamental goal of reducing family violence is broadly relevant and important across the political spectrum.	probably liberal
ACP	Preparation for Chemical and Biological Terrorism	2023	The text you provided from the American College of Physicians (ACP) does not align strictly with either a liberal or conservative viewpoint in the traditional political spectrum. It focuses on public health policy, particularly in the context of preparing for and responding to biological and chemical attacks. The key elements of the text include:<br><br>1. Emphasizing the importance of education and training for physicians in dealing with biological and chemical attacks.<br>2. Advocating for the development of public health structures to handle such disasters.<br>3. Stating that funding for combating biological or chemical attacks should be part of defense efforts and not detract from other medical research.<br>4. Calling for Congress and various departments to work together in developing crisis management plans and ensuring community preparedness.<br>5. Stressing the need for sufficient funding in various areas, including public health departments, hospital training, food and water safety, and the Centers for Disease Control and Prevention (CDC).<br><br>These points focus on preparedness, public health infrastructure, and funding allocation. They represent a pragmatic approach to a specific set of issues rather than a clear-cut ideological stance. Such policies could be supported by individuals across the political spectrum, as they deal with public safety and health, areas often considered as non-partisan or cross-partisan concerns. The emphasis on government funding and coordination might be more aligned with a liberal perspective that often advocates for proactive government involvement in public health issues. However, the focus on national defense and security through preparedness for biological and chemical attacks could also resonate with conservative viewpoints that prioritize national security.	neutral
ACP	Nuclear Weapons and Other Weapons of Mass and Indiscriminate Destruction	2017	The text you provided, advocating for the elimination of nuclear weapons and other weapons of mass and indiscriminate destruction, generally aligns more closely with a liberal viewpoint. Liberal perspectives often prioritize disarmament, international cooperation, and the use of non-military means for conflict resolution. The emphasis on dissemination through organizations like the World Health Organization also reflects a liberal inclination towards multilateralism and global governance structures.<br><br>However, it's important to note that such issues can sometimes transcend traditional political boundaries, and there can be support for disarmament and non-proliferation across the political spectrum, albeit for different reasons. Conservatives might also support such measures, particularly if they are framed in the context of national security, stability, and fiscal responsibility.<br><br>The reference to the American College of Physicians (ACP) and its policy positions indicates a focus on health implications, which is a common perspective in liberal policy circles, where there is a greater emphasis on public health and its intersection with global policy issues.	liberal

ACP	Resolution on the International Campaign to Ban Antipersonnel Landmines	2016	The text you've provided appears to be a policy statement from the American College of Physicians (ACP). It expresses support for an international campaign to ban antipersonnel mines and emphasizes the need for education and advocacy about their impact on public health, medical, and social infrastructures.<br><br>This statement doesn't neatly align with traditional liberal or conservative viewpoints as defined in the U.S. political spectrum. It focuses more on humanitarian and public health concerns rather than ideological positions.<br><br>Issues like the ban on antipersonnel mines often transcend typical political boundaries, receiving support from a wide range of political viewpoints due to their focus on human rights, public health, and international law. It's not uncommon for both liberal and conservative groups to support such measures, albeit sometimes for different underlying reasons. Liberals might focus on the humanitarian and global health aspects, while conservatives might emphasize national security and international stability.<br><br>In summary, this statement from the ACP is more aligned with humanitarian and public health advocacy than with specific political ideologies.	neutral
ACP	Nuclear Weapons	2016	The text you provided seems to align with a viewpoint that is generally more liberal in nature. It emphasizes the recognition of the threat posed by nuclear weapons and advocates for diplomatic efforts to limit, reduce, and ultimately eliminate these weapons. This perspective tends to align with liberal values, which often prioritize diplomacy and global cooperation in addressing international security issues, including nuclear disarmament.<br><br>However, it's important to note that the issue of nuclear disarmament can also find support across different political spectra, and the approach to it might vary depending on the context and specific policies involved. In some cases, conservative viewpoints may also support disarmament efforts, especially if they align with national security interests or international stability goals.	liberal
ACP	Reproductive Health Policy in the United States	2023	The text provided aligns more closely with a liberal viewpoint. Liberal ideologies typically emphasize individual rights, personal freedoms, and support for social policies that offer greater access to health care, including reproductive services. The statements from the American College of Physicians (ACP) reflect these liberal values through their emphasis on:<br><br>1. The right of individuals to make decisions about their reproductive health, opposing government restrictions that limit access to reproductive healthcare, including contraception and abortion.<br><br>2. Opposition to restrictive laws that penalize healthcare providers and patients for accessing or providing reproductive health care services.<br><br>3. The belief that third-party private citizens should not enforce state laws restricting reproductive health care services.<br><br>4. Opposition to the use of personal health information to prosecute individuals seeking reproductive health care.<br><br>5. Advocacy for equitable access to high-quality health care for all individuals, supporting the shipment and delivery of legally prescribed drugs and the freedom for patients to travel across state lines for healthcare services.<br><br>These positions are typically associated with liberal viewpoints as they advocate for personal choice in healthcare, oppose restrictive governmental interference, and emphasize equitable access to healthcare services.	liberal

ACP	Funding for Women’s Health Clinics	2017	The text you've shared from the American College of Physicians (ACP) appears to be aligned more with a liberal viewpoint. This alignment is based on the support for comprehensive women's health services, including contraception and sexually transmitted infection testing and treatment, as well as opposition to legislative or regulatory restrictions that would limit funding to clinics providing these services.<br><br>In general, liberal viewpoints in the United States often emphasize the protection of individual rights and freedoms, including reproductive rights and access to a full range of healthcare services. The emphasis on evidence-based services and opposition to restrictions on funding for women's health clinics reflects these liberal values of individual choice and access to healthcare.<br><br>On the other hand, conservative viewpoints typically place a greater emphasis on traditional values and may support more restrictions on reproductive healthcare, particularly regarding issues like contraception and abortion. Conservatives might be more likely to support legislation that restricts funding to clinics providing certain reproductive health services.<br><br>It's important to note that these are general trends and there can be a wide range of opinions within each political ideology. Also, the interpretation of whether a position is liberal or conservative can vary depending on cultural and regional contexts.	liberal
ACP	Principles on Women’s Health	2017	The text you've provided outlines principles endorsed by the American College of Physicians (ACP) in conjunction with other medical organizations such as the American College of Obstetricians and Gynecologists (ACOG), the American Academy of Pediatrics (AAP), and the American Academy of Family Physicians (AAFP). These principles focus on women's health issues and advocate for:<br><br>1. Unencumbered access to affordable, evidence-based health care for women throughout their lifespan.<br>2. Opposition to political interference in the patient-provider relationship.<br>3. Protection of current benefits and coverage for women, including preventive care and the ban on gender rating.<br>4. Protection of Medicaid coverage and financing, ensuring consistent treatment of qualified providers.<br><br>These principles generally align more with a liberal viewpoint. Liberal perspectives often emphasize the importance of accessible healthcare, reproductive rights, and opposition to political interference in healthcare decisions. This stance is evident in the emphasis on "evidence-based health care", "opposing political interference", and "protecting Medicaid coverage".<br><br>However, it's important to note that healthcare can be a complex and nuanced topic, with different individuals and groups within both liberal and conservative circles holding varied opinions on specific issues. The principles mentioned here focus on healthcare access and equity, which are typically more emphasized in liberal agendas.	liberal
ACP	Women’s Health Care	2017	The text you've provided appears to be policy recommendations regarding women's health care from the American College of Physicians (ACP). These recommendations emphasize the importance of comprehensive and integrated health care for women, the need for improved education and training in women's health for internists, and the requirement for competency in managing common conditions in women.<br><br>These recommendations do not align strictly with a liberal or conservative viewpoint in the traditional political sense. Instead, they focus on medical education, health care quality, and patient care standards. The emphasis on comprehensive health care and education in women's health might be more associated with progressive or liberal viewpoints that generally advocate for broad healthcare access and improvements. However, the focus on improving standards and competencies in medical practice is a non-partisan issue that aligns with the overall goal of enhancing healthcare quality, which can be endorsed by individuals across the political spectrum.<br><br>It's important to note that in the context of healthcare and medical practice, discussions about improving care quality and medical education are typically centered around patient outcomes and medical ethics, rather than political ideology.	neutral

ACP	Teenage Pregnancy	2017	The text you've provided seems to align more closely with a liberal viewpoint. Liberal perspectives often emphasize the importance of community-based programs, addressing social and economic consequences of issues like teenage pregnancy, and supporting family-planning initiatives. This approach typically involves advocating for increased funding for educational and clinical services related to family planning, which is consistent with the text.<br><br>Conservative viewpoints, on the other hand, might focus more on personal responsibility, limited government intervention in social issues, and might be less inclined to support increased federal funding for family-planning programs.<br><br>However, it's important to note that these alignments are not absolute, and there can be a wide range of opinions within each political spectrum. Additionally, some aspects of this statement could be supported by individuals from various political backgrounds, especially when focusing on the well-being of underserved communities.	liberal
ACP	Women’s Health Policy in the United States	2018	The text you provided outlines the viewpoints of the American College of Physicians (ACP) on various issues related to women's health care. To determine whether these viewpoints align more with a liberal or conservative perspective, it's important to consider the general ideologies associated with these political spectrums:<br><br>1. **Emphasis on Women's Health Care in Internal Medicine**: This point focuses on the importance of training in women's health issues across different medical specialties. This approach is generally non-partisan, but the emphasis on comprehensive healthcare training could be more in line with liberal ideologies that often prioritize inclusive and wide-ranging healthcare education.<br><br>2. **Access to Affordable, Comprehensive Health Care**: Advocating for non-discriminatory, affordable healthcare coverage, including for women, is typically aligned with liberal viewpoints. Conservatives may support affordable healthcare as well, but often emphasize market-based solutions and personal responsibility over government intervention.<br><br>3. **Reproductive Rights and Opposition to Government Restrictions**: The support for reproductive rights, including access to contraception and abortion, and opposition to government restrictions in these areas, strongly aligns with liberal viewpoints. Conservative perspectives traditionally favor more restrictions on abortion and may emphasize alternative approaches to family planning.<br><br>4. **Opposition to Restrictions on Reproductive Health Care**: Similar to point 3, opposing medically unnecessary restrictions on reproductive healthcare professionals or facilities aligns with liberal ideologies, which often advocate for fewer barriers to accessing healthcare services, including reproductive services.<br><br>5. **Support for Universal Access to Family and Medical Leave**: Advocating for paid family and medical leave policies is typically associated with liberal policies, as they often support stronger worker protections and benefits.<br><br>6. **Increased Support for Survivors of Violence**: The emphasis on screening, education, and resources for survivors of intimate partner or sexual violence is generally a bipartisan issue, but the approach outlined here is more comprehensive and systemic, which can be more aligned with liberal ideologies.<br><br>7. **Representation of Women in Clinical Research**: The focus on improving women's representation in clinical research and addressing specific health issues is a progressive stance, often associated with liberal viewpoints, emphasizing inclusivity and diversity in medical research.<br><br>Overall, while some aspects of these viewpoints could be supported by individuals across the political spectrum, the comprehensive, inclusive, and rights-focused approach to women's healthcare and reproductive rights aligns more closely with liberal ideologies.	liberal

ACP	Maternal Mortality Review Committees	2018	The text you've provided outlines the American College of Physicians' (ACP) support for establishing maternal mortality review committees (MMRCs) and related programs to improve maternal health outcomes in the United States. This stance focuses on data collection, analysis, and implementation of strategies to prevent maternal deaths and enhance maternal health.<br><br>Regarding its alignment with liberal or conservative viewpoints:<br><br>1. **Liberal Viewpoint**: The emphasis on systematic data collection, cross-jurisdictional cooperation, and implementation of best practices for maternal health aligns with typical liberal viewpoints, which often prioritize public health initiatives, evidence-based policy, and government intervention in healthcare to improve outcomes.<br><br>2. **Conservative Viewpoint**: While conservatives generally advocate for limited government intervention, they might support this initiative if it is seen as a means to improve health outcomes without excessive regulation or government overreach. Some conservative perspectives might favor such initiatives if they are implemented at a state or local level, respecting the principle of decentralized governance.<br><br>However, it's important to note that issues like maternal health can transcend traditional political divides. Both liberals and conservatives can find common ground in efforts to improve health outcomes, although their approaches and the extent of government involvement might differ. The ACP's position, as described, seems primarily focused on health outcomes and best practices, which can be supported across the political spectrum.	neutral
ACP	Support for Lactating Medical Students, Post-Graduate Trainees, and Physicians	2023	The text provided appears to be a policy statement from the American College of Physicians (ACP) regarding support for breastfeeding among medical students, post-graduate trainees, and physicians. This statement emphasizes the importance of lactation accommodation and support in healthcare institutions.<br><br>The viewpoint expressed in this text does not neatly align with traditional liberal or conservative political ideologies. Instead, it focuses on workplace accommodations and support for breastfeeding, which is a public health and employee welfare issue. Such policies can be supported by individuals across the political spectrum for various reasons:<br><br>1. **Public Health Perspective**: Emphasizing the health benefits of breastfeeding aligns with a public health viewpoint, which can be endorsed by both liberals and conservatives.<br><br>2. **Employee Rights and Welfare**: Advocating for workplace accommodations, like lactation spaces and paid time for lactation, aligns with a broader concern for employee rights and welfare. This is often more associated with liberal policies that focus on workers' rights, but it can also be supported by conservatives under family values and individual welfare.<br><br>3. **Family Values**: The support for breastfeeding and providing accommodations for it can also be viewed through the lens of family values, which is a key aspect often emphasized in conservative viewpoints.<br><br>In summary, while some aspects of this policy could be more traditionally aligned with liberal viewpoints, particularly those related to employee rights and welfare, the overall focus on supporting family health and well-being through breastfeeding accommodation can resonate with values across the political spectrum.	neutral

AAFP	AAFP CME Credit System Eligibility Requirements	10/2023	The text you've provided outlines criteria for Continuing Medical Education (CME) activities set by the American Academy of Family Physicians (AAFP). These criteria are designed to ensure that CME activities are relevant, evidence-based, and appropriate for family medicine practitioners.<br><br>This content does not directly align with a traditional liberal or conservative political viewpoint. Instead, it focuses on professional standards and educational guidelines within the field of family medicine. The emphasis is on ensuring quality, relevance, and scientific basis in medical education, which are principles that transcend political ideologies.<br><br>Key elements of this text include:<br><br>1. **Relevance to Family Medicine**: Emphasizing that CME activities must be pertinent to the scope of family medicine, ensuring that physicians receive education that is directly applicable to their field.<br><br>2. **Evidence-Based and Educationally Appropriate Content**: The focus on evidence-based or customary and generally accepted medical practices aligns with a scientific and professional approach to medical education, regardless of political leaning.<br><br>3. **Educational Needs and Professional Practice Gaps**: Addressing the educational needs based on professional practice gaps is a standard approach in professional development, aimed at improving the quality of patient care.<br><br>4. **Compliance with ACCME Standards**: Ensuring integrity and independence in accredited continuing education is a matter of maintaining professional and ethical standards in medical education.<br><br>5. **Functional Medicine**: The guidelines for functional medicine education focus on providing an overview for educational purposes rather than practical training in these techniques.<br><br>Overall, these guidelines reflect a commitment to high-quality, relevant, and professional medical education rather than a specific political ideology. They are centered around the best practices and standards in medical education and are likely to be supported by medical professionals regardless of their personal political views.	neutral
AAFP	AAFP Credit System Policy	12/2023	The text you've provided outlines policies and procedures related to Continuing Medical Education (CME) credit as administered by the American Academy of Family Physicians (AAFP). This document is primarily administrative and procedural in nature and does not inherently align with either a liberal or conservative viewpoint.<br><br>The policies discussed focus on maintaining standards for CME providers, ensuring transparency and professionalism, and updating content in line with new scientific developments. These practices are common in many professional and educational organizations and are generally aimed at maintaining high standards of education and ethics. They do not explicitly endorse or oppose any political ideology.<br><br>It's important to recognize that the administration of educational credits and the establishment of professional standards, while they can be influenced by broader societal and political contexts, are typically neutral with respect to partisan politics. The primary goal of such policies is usually to ensure quality and integrity in professional education, rather than to advance any specific political agenda.	neutral
AAFP	AAFP Definitions for Policy Statement, Position Paper and Discussion Paper		The text you've provided is a neutral description of the processes and definitions related to policy formulation in an academy setting. It does not align specifically with either a liberal or conservative viewpoint. The text focuses on explaining the mechanisms by which policy is articulated and the distinctions between various types of documents (Policy Statements, Position Papers, Discussion Papers) used in that process. It emphasizes procedural aspects rather than ideological content, making it a neutral, informative description suitable for an educational or organizational context.	neutral

AAFP	AAFP Guiding Principles for Value-Based Payment.	9/2022	The text you've provided seems to focus on policy recommendations for the U.S. healthcare system, particularly in the context of primary care and family physicians. It advocates for a shift from fee-for-service models to value-based payment (VBP) models, emphasizing the importance of primary care in improving health outcomes, reducing inequities, and controlling healthcare spending.<br><br>This text does not neatly align with a traditional liberal or conservative viewpoint as understood in the typical political spectrum. Instead, it presents a perspective focused on healthcare policy and management. The principles outlined are more technical and administrative rather than ideological.<br><br>- Advocacy for value-based payment models: This aligns with a general healthcare reform perspective, which can be seen across the political spectrum. It suggests an interest in improving efficiency and effectiveness in healthcare delivery, which could resonate with both conservative and liberal views.<br><br>- Emphasis on primary care and reducing healthcare disparities: These points might be more closely aligned with a liberal viewpoint, which often emphasizes equitable access to healthcare and addressing social determinants of health.<br><br>- Focus on reducing administrative burdens and improving efficiency: This aspect might appeal to conservative viewpoints, which often emphasize efficiency and reduced bureaucracy in healthcare.<br><br>- Collaboration between various stakeholders: This is a pragmatic approach that does not strictly fall into a liberal or conservative category but indicates a willingness to work across different sectors (like government agencies, unions, and healthcare providers).<br><br>In summary, while the text contains elements that might resonate more with one political ideology than another, it predominantly presents a healthcare-focused perspective that encompasses concerns typically shared across the political spectrum.	neutral
AAFP	AAFP Mailing List Policy	7/2023	The text you've provided outlines policies regarding the rental and use of the American Academy of Family Physicians (AAFP) mailing list for the promotion of Continuing Medical Education (CME) activities, other promotional mailings, and survey/research projects. This text primarily focuses on operational and ethical guidelines for using the AAFP mailing list and does not explicitly align with a liberal or conservative viewpoint.<br><br>It is more about maintaining professional standards, ensuring the relevance and accuracy of information sent to AAFP members, and protecting the integrity and privacy of the AAFP's mailing list. These policies are geared towards ensuring that any promotions or communications sent to their members are reputable, factually accurate, and relevant to their practice or education in family medicine.<br><br>Such policies are common in professional organizations and are typically designed to uphold the quality and relevance of information shared with their members, rather than to promote a specific political viewpoint.	neutral
AAFP	AAFP Mission Statement	12/2023	The statement from the American Academy of Family Physicians about improving the health of patients, families, and communities through serving the needs of members with professionalism and creativity doesn't inherently align with a specific political viewpoint, either liberal or conservative. It focuses on the mission of a medical organization, which is primarily concerned with healthcare and professional standards. This kind of mission statement generally aims to be apolitical, concentrating on health and service rather than political ideologies.	neutral
AAFP	AAFP Promotions: Print Advertorials	2016	The text you've provided appears to be a policy statement or guideline from the American Academy of Family Physicians (AAFP) regarding their participation in advertorials or sponsored content in various media outlets. This statement outlines their commitment to transparency and ethical advertising practices.<br><br>Determining whether this text aligns with a liberal or conservative viewpoint isn't straightforward. The content focuses on ethical advertising practices, transparency, and the separation of editorial and commercial content. These are issues that generally transcend typical political liberal-conservative divides.<br><br>In broader terms, issues of media transparency and ethical advertising can be of interest to both liberal and conservative viewpoints, although the specific concerns or emphasis might differ. Liberals might focus more on the social responsibility and the potential impact of advertising on public health and misinformation. Conservatives might emphasize the importance of free market principles, including the freedom for businesses to advertise, and the responsibility of the media to be transparent and unbiased.<br><br>However, this text primarily addresses professional and ethical standards in the context of medical information and advertising. It	neutral

			does not explicitly advocate for political or ideological positions typically associated with liberal or conservative viewpoints. Instead, it reflects a professional and ethical stance on how to handle sponsored content in a way that maintains trust and clarity for readers.	
AAFP	AAFP-ACOG Joint Statement on Cooperative Practice and Hospital Privileges	2019	The text provided appears to be a policy or position statement related to maternity care, focusing on the importance of collaborative relationships among various health professionals in the United States. It emphasizes the role of cooperative work environments, shared decision making, and consistent standards of care across specialties, especially in perinatal services. This includes the involvement of family physicians, obstetricians, nurse midwives, and others in providing comprehensive care to pregnant women.<br><br>This statement does not align explicitly with either a liberal or conservative viewpoint as typically defined in political terms. Instead, it addresses professional standards and practices in healthcare, focusing on the quality of patient care and the collaboration among different medical specialties. Such topics generally transcend traditional political divides, focusing more on medical best practices, patient safety, and healthcare administration.<br><br>In the broader context of healthcare policy, aspects of this statement could be supported by individuals across the political spectrum. For example, emphasis on collaboration, high standards of care, and the inclusion of various healthcare professionals might align with more progressive or liberal views on healthcare accessibility and quality. On the other hand, the emphasis on professional standards and individual responsibility among healthcare providers might resonate with conservative values related to personal accountability and professional autonomy.<br><br>Overall, the statement is more closely aligned with professional healthcare standards and practices than with specific political ideologies.	neutral
AAFP	AAFP-SHM Joint Statement on Hospitalists Trained in Family Medicine	1/2022	The text you've provided is primarily informational and professional in nature, focusing on the role, training, and recognition of hospitalists, particularly those trained in family medicine. It does not explicitly align with a liberal or conservative viewpoint as it is centered on medical practice and education rather than political ideology.<br><br>The content discusses the qualifications, training, and contributions of hospitalists, especially those with a background in family medicine. It outlines the various aspects of their training, the demand for their services in the United States, and the recognition they can receive from professional medical organizations.<br><br>Such information is generally neutral and aimed at informing about a specific medical profession rather than advocating for a particular political stance. It focuses on professional standards, educational requirements, and the contribution of these professionals to public health, which are topics typically outside the direct scope of liberal or conservative ideologies.	neutral

AAFP	Abolish Corporal Punishment in Schools	1/2022	The text you provided from the American Academy of Family Physicians (AAFP) regarding the abolishment of corporal punishment in schools doesn't strictly align with either a liberal or conservative viewpoint in a traditional political sense. It is more a position grounded in medical, educational, and psychological perspectives.<br><br>The AAFP's stance is based on evidence and research suggesting that corporal punishment is less effective than other behavioral management techniques and may exacerbate behavioral issues. This viewpoint is centered around concerns for child welfare, educational efficacy, and psychological well-being, rather than political ideology.<br><br>However, in the broader socio-political context, the opposition to corporal punishment might be more commonly associated with liberal viewpoints, which tend to emphasize non-violent methods of discipline, focus on the psychological well-being of students, and advocate for progressive educational practices. Conservative viewpoints, traditionally, might be more inclined to support traditional disciplinary methods, including corporal punishment, under the purview of maintaining order and discipline.<br><br>It's important to note that these associations can vary significantly based on geographical, cultural, and individual factors, and there are certainly liberals who might support corporal punishment and conservatives who oppose it. The issue transcends simple political categorization and delves into deeper debates about child development, education, and societal values.	probably liberal
AAFP	Adolescent Health Care, Confidentiality	2020	The text provided does not align strictly with either a liberal or conservative viewpoint in the political sense. Instead, it focuses on medical ethics and best practices regarding confidentiality in adolescent healthcare. The points mentioned are more centered on medical professionalism, patient rights, and legal standards rather than political ideologies.<br><br>This approach emphasizes the importance of confidentiality in healthcare for adolescents, recognizing their legal rights while also acknowledging the beneficial role of parental involvement. It navigates through the complexities of healthcare delivery to adolescents, balancing legal requirements, ethical considerations, and the medical well-being of the patient.<br><br>The text also acknowledges the variations in state laws and the necessity for physicians to be aware of these differences. It underscores the importance of informed consent, the role of the physician in maintaining confidentiality, and the exceptions to confidentiality, such as imminent danger or legal requirements to report certain conditions.<br><br>Overall, the emphasis is on the responsibility of healthcare professionals to provide confidential, respectful, and appropriate care to adolescent patients, considering both legal and ethical dimensions. This approach transcends simple political categorizations, focusing instead on patient-centered care and professional responsibility.	neutral
AAFP	Adolescent Health Care, Role of the Family Physician	1/2022	The text you've provided presents a viewpoint that emphasizes the role of family physicians in addressing the comprehensive health care needs of adolescents. It advocates for the involvement of family physicians in a range of areas, including physical health, reproductive health, gender identity and expression, substance use, and mental health. It also underscores the importance of confidentiality, science-based health education, and safety in schools.<br><br>This viewpoint can be considered aligned more with liberal perspectives, particularly in its emphasis on inclusive and comprehensive health care, focus on reproductive rights, and attention to gender identity and expression. The call for family physicians to be actively involved in community and legislative advocacy for these issues is also more characteristic of a liberal stance, which often involves proactive engagement in policy and community health matters.<br><br>However, it's important to note that the provision of comprehensive health care and the emphasis on the role of family physicians in adolescent health can be supported across the political spectrum. The text does not explicitly align with strictly partisan viewpoints but rather focuses on broad healthcare principles and the role of family physicians in community health.	liberal

AAFP	Adolescent Health Care, Sexuality and Contraception	2020	The text from the American Academy of Family Physicians (AAFP) largely aligns with what is generally considered a liberal viewpoint. This assessment is based on several key aspects:<br><br>1. **Emphasis on Comprehensive Sex Education:** The liberal viewpoint typically supports comprehensive sex education in schools, which includes topics like consent, intimate relationships, human sexual anatomy, sexual reproduction, sexually transmitted infection prevention, sexual activity, sexual orientation, gender identity, abstinence, contraception, and reproductive rights. The text reflects this approach.<br><br>2. **Opposition to Abstinence-Only Education:** The text states that the AAFP does not support abstinence-only sexual education. This stance is more aligned with liberal perspectives, which often critique abstinence-only programs for lacking comprehensive sexual health information.<br><br>3. **Focus on Evidence-Based Information:** The advocacy for medically accurate, evidence-based information is a hallmark of liberal approaches to education, particularly in areas of science and health.<br><br>4. **Inclusion of Discussions on Sexual Orientation and Gender Identity:** The text's emphasis on the inclusion of sexual orientation and gender identity in education is more commonly associated with liberal viewpoints, which tend to advocate for LGBTQ+ inclusivity and rights.<br><br>5. **Attention to Reproductive Rights and Confidential Care:** The mention of reproductive rights and the provision of confidential care to adolescents seeking family planning services aligns with liberal views on reproductive health and privacy.<br><br>6. **Engagement in Community Efforts for Sex Education:** The encouragement of community involvement in sex education reflects a more progressive approach to public health and education.<br><br>7. **Sensitivity to Adolescent Sexual Exploration:** The text's approach to adolescent sexual exploration, including open-ended questions about sexual orientation and gender identity in a sensitive atmosphere, is in line with a more liberal perspective that emphasizes understanding and acceptance of diverse sexual identities.<br><br>It's important to note that these categorizations are not absolute and there can be overlap in viewpoints. Additionally, terms like "liberal" and "conservative" can vary in meaning depending on the cultural and political context.	liberal
AAFP	Advancing Health Equity by Addressing the Social Determinants of Health in Family Medicine (Position Paper)	4/2019	The text you provided outlines a position paper on the social determinants of health (SDoH) and their impact on health inequities, as well as the role of family physicians and policy interventions in addressing these issues. It's a comprehensive and detailed exposition of how various social factors like socioeconomic status, race, ethnicity, sexual orientation, and geographic location impact health outcomes.<br><br>Regarding its alignment with a liberal or conservative viewpoint, it's important to note that concepts of health equity and social determinants of health tend to be more prominently emphasized in liberal or progressive ideologies. This viewpoint often advocates for systemic change and policy interventions to address inequalities and improve social conditions, which aligns with the approaches and recommendations in the text.<br><br>Conservative viewpoints generally emphasize personal responsibility, limited government intervention, and market-based solutions. However, this doesn't mean that conservatives are unconcerned with health disparities; rather, the approaches and solutions they advocate might differ, often focusing more on individual choice and market-driven solutions.<br><br>This paper heavily focuses on systemic factors and the role of policy in addressing health inequities, which is more aligned with liberal perspectives. It discusses the importance of addressing structural determinants of health through government and policy interventions, emphasizes the impact of societal structures on individual health outcomes, and advocates for broad systemic changes, all of which are typically associated with liberal policy approaches.	liberal

AAFP	Advancing Health Equity: Principles to Address the Social Determinants of Health in Alternative Payment Models	4/2018	The text you've provided primarily focuses on the implementation and impact of value-based healthcare, particularly with respect to social determinants of health (SDoH). It discusses various principles and approaches to integrating SDoH into healthcare delivery and payment systems, emphasizing the importance of addressing these determinants to improve health outcomes and reduce disparities.<br><br>This text does not explicitly align with a traditional liberal or conservative viewpoint as understood in the context of U.S. politics. Rather, it approaches the subject from a healthcare policy and administration perspective, discussing practical and systemic ways to improve healthcare outcomes. The emphasis on SDoH, value-based care, and addressing healthcare disparities can be associated with broader principles often emphasized in progressive or liberal policy discussions, such as a focus on equity, comprehensive care, and the social factors influencing health. However, the text also touches on efficiency, evidence-based approaches, and resource allocation, which are concerns often highlighted in conservative viewpoints.<br><br>It's important to note that healthcare policy can transcend traditional political divisions, with different aspects appealing to different viewpoints. The integration of SDoH into healthcare delivery is a complex issue that involves considerations of public health, economics, social justice, and medical practice, which can be viewed through various ideological lenses.	neutral
AAFP	Adverse Childhood Experiences	10/2023	The text you've provided presents a factual and research-based approach to understanding and addressing Adverse Childhood Experiences (ACEs). It doesn't neatly align with either a liberal or conservative viewpoint in a traditional political sense. Instead, it focuses on the scientific and medical aspects of ACEs, as well as the public health initiatives recommended by the American Academy of Family Physicians (AAFP).<br><br>Key points in the text, such as acknowledging the significant impact of ACEs on health, advocating for prevention and treatment programs, and supporting research and policy measures, are generally aligned with a public health perspective. This approach emphasizes understanding social, environmental, and psychological factors that contribute to health and well-being, and it advocates for comprehensive strategies to address these issues.<br><br>In the political context, both liberal and conservative viewpoints might intersect with these issues in different ways:<br><br>1. **Liberal Viewpoint**: Might focus on the societal and systemic factors contributing to ACEs and advocate for broad social and health policies to address these root causes. This could include support for mental health services, social safety nets, and educational programs.<br><br>2. **Conservative Viewpoint**: Might emphasize individual responsibility, family values, and community-based solutions. Conservatives might support interventions that strengthen family structures and local community support systems.<br><br>However, the text itself remains largely neutral, focusing on the medical and public health aspects rather than political ideologies. It presents ACEs as a significant health concern that requires attention from the medical community, policymakers, and society at large.	neutral
AAFP	Advertising: Youth Products	10/2023	The text you've provided, stating that the American Academy of Family Physicians (AAFP) believes advertising campaigns should promote healthy lifestyles and opposes targeting youth with advertising that relies on sexually suggestive or violent themes, doesn't align strictly with either a liberal or conservative viewpoint in a traditional political sense. Instead, it reflects a health-focused perspective.<br><br>Public health and welfare concerns, like promoting healthy lifestyles and protecting youth from potentially harmful influences, are generally non-partisan issues that can be supported by individuals across the political spectrum. The stance against sexually suggestive or violent themes in advertising to youth could be seen as aligning with concerns about public morality and child welfare, which are sometimes more emphasized in conservative discourse. However, the emphasis on health promotion and preventive care can also be found in liberal approaches to public health policy.<br><br>It's important to recognize that many organizations, like the AAFP, operate on principles that transcend simple political categorization, focusing instead on their specific areas of expertise and concern, in this case, family health and welfare.	neutral

AAFP	Aging	10/2023	The text you provided seems to be a policy statement from the American Academy of Family Physicians (AAFP) regarding their support for various aspects of geriatric care. This statement, dating from October 2023, emphasizes the organization's commitment to research, faculty development, continuing medical education, residency training, and advocacy resources in the field of aging and care for the elderly.<br><br>The content of this statement doesn't inherently align with a strictly liberal or conservative viewpoint. Instead, it reflects a professional and organizational commitment to improving healthcare for the elderly. Both liberal and conservative viewpoints can support such initiatives, albeit potentially for different reasons or with varying approaches.<br><br>- **Liberal Viewpoint:** Might focus on the aspect of providing comprehensive care and resources to an often underserved population, emphasizing the role of government and public policy in supporting such initiatives.<br><br>- **Conservative Viewpoint:** Might appreciate the focus on education and training, seeing it as a way to improve efficiency and effectiveness in healthcare delivery, potentially reducing long-term costs.<br><br>However, the interpretation of such a policy statement can vary based on the broader context of an individual's beliefs and the specific political and social issues of the time. It's important to consider that such statements from professional organizations are often designed to be broadly agreeable and focused on professional standards and improvements rather than political ideologies.	neutral
AAFP	Alcohol Advertising and Underage Alcohol Usage	9/2023	The text you've provided presents a viewpoint that is primarily focused on public health concerns related to underage alcohol consumption and the potential impact of alcohol advertising on youth, especially in socioeconomically disadvantaged communities. This perspective aligns with a focus on social responsibility, public health, and the regulation of industries to protect vulnerable populations.<br><br>In the context of American politics, these themes can be associated with both liberal and conservative viewpoints, although the emphasis can differ:<br><br>1. **Liberal Viewpoint**: Liberals often emphasize the role of government in regulating industries to protect public health and the welfare of vulnerable populations, including children and socioeconomically disadvantaged communities. The call for regulation of alcohol advertisements and concern over public health issues can align with liberal perspectives on government intervention for social welfare.<br><br>2. **Conservative Viewpoint**: Conservatives may also be concerned about public health issues, especially when it relates to family values and the well-being of youth. The focus on family physicians being aware of and addressing these issues can resonate with conservative values emphasizing family and community. However, conservatives typically prefer less government regulation of private industries, which might contrast with the call for stricter regulation of alcohol advertisements.<br><br>In summary, while the text's focus on public health and regulation may lean more towards a liberal viewpoint, its emphasis on family and community health can also align with conservative values. The viewpoint is not strictly partisan and can be seen as a cross-cutting issue that resonates with broader concerns in both liberal and conservative circles.	neutral
AAFP	Anti-Harassment Policy	1/2019	The text you've provided is a policy statement from the American Academy of Family Physicians (AAFP) concerning harassment and professional conduct. It focuses on creating an environment free from harassment and offensive treatment, emphasizing respect, professionalism, and adherence to generally accepted social behaviors.<br><br>This statement aligns more with principles that are often associated with a liberal viewpoint, particularly in its emphasis on inclusivity and protection against discrimination based on a wide range of characteristics such as age, race, gender, sexual orientation, and others. Liberal perspectives typically prioritize diversity, inclusivity, and the protection of individual rights, especially for marginalized groups.<br><br>However, it's important to note that the commitment to a harassment-free environment and the promotion of professional conduct are values that can be endorsed by individuals across the political spectrum. The focus on professionalism and respect in the workplace is a universal principle that transcends political ideologies. While the explicit mention of specific groups and the comprehensive nature of the policy might be more closely aligned with liberal values, the fundamental objective of ensuring a respectful and professional environment is a broadly shared goal.	liberal

AAFP	Antibiotic Resistance, Food Production and Human Health	4/2020	The text you've provided appears to be focused on public health policy, particularly regarding the use of antibiotics in farm animals. This issue intersects with several broader topics such as healthcare, environmental policy, and regulation of the food industry.<br><br>From a political perspective, the viewpoints expressed in this text could align with either liberal or conservative values, but the emphasis is slightly more towards a liberal viewpoint. Here's why:<br><br>1. **Public Health Emphasis:** The concern for human health consequences of non-therapeutic antibiotic use reflects a liberal tendency to prioritize public health and safety, often through regulatory measures.<br><br>2. **Regulatory Approach:** The advocacy for restricting antibiotic use and requiring proof of efficacy and a positive cost/benefit analysis aligns with a more regulatory, interventionist approach typically associated with liberal politics.<br><br>3. **Support for Federal Legislation:** The support for Federal legislation to regulate the use of antibiotics in the food industry is indicative of a belief in the role of government in regulating industries for public good, a stance more commonly associated with liberal politics.<br><br>However, it's important to note that concern about public health and the responsible use of antibiotics in food production can transcend traditional political boundaries. Conservatives might also support such measures if they are seen as essential for public health and safety, or if they align with a conservative emphasis on ethical stewardship and responsible management of resources.<br><br>In summary, while the text leans slightly more towards a liberal viewpoint due to its emphasis on regulation and public health, the issue itself can find support across the political spectrum depending on the context and framing.	probably liberal
AAFP	Antibiotics	7/1905	The text you provided doesn't clearly align with a liberal or conservative viewpoint. It focuses on the medical and public health perspective regarding the use of antibiotics. The statement emphasizes the importance of responsible antibiotic prescribing based on evidence and guidelines, which is a matter of medical best practice rather than political ideology.<br><br>The American Academy of Family Physicians (AAFP) is a professional medical organization, and their position as stated here is centered on ensuring patient safety and public health, which are universal concerns across the political spectrum. The reference to using evidence-based guidelines, like those from the Choosing Wisely Campaign, reinforces this non-partisan, medically-focused approach.	neutral
AAFP	Antitrust Policy	1/2019	The text you've provided is a statement from the American Academy of Family Physicians (AAFP) focusing on adherence to U.S. antitrust laws. It outlines policies and practices to ensure compliance with federal and state antitrust laws and avoid unlawful restraint of trade. This statement primarily addresses legal and ethical standards concerning competition and market practices.<br><br>This text does not align explicitly with either a liberal or conservative viewpoint in the traditional political sense. It is more focused on legal compliance and ethical business practices, which are concerns that cut across political lines. Both liberal and conservative groups generally agree on the importance of adhering to laws and maintaining ethical standards in professional organizations, although they might differ in their approaches to regulation and market competition.<br><br>The content here is centered on ensuring that the AAFP's actions are lawful and ethical, and it does not delve into political or ideological issues typically associated with liberal or conservative viewpoints.	neutral

AAFP	Area Health Education Centers	7/2023	The statement regarding Area Health Education Centers (AHECs) does not explicitly align with a strictly liberal or conservative viewpoint. It primarily focuses on healthcare education and resource distribution, particularly in areas with a scarcity of health personnel. The goals mentioned—improving distribution and quality of health personnel, supporting family medicine, and promoting continuing medical education—tend to be more broadly related to public health and education rather than being distinctly partisan issues.<br><br>However, the support for such programs can be seen through different political lenses:<br><br>1. **Liberal Viewpoint**: A liberal perspective might emphasize the role of AHECs in addressing healthcare disparities and ensuring equitable access to healthcare services, especially in underserved areas. The focus on education, community health, and government-supported initiatives for health personnel could align with liberal priorities around social equity and government intervention in healthcare.<br><br>2. **Conservative Viewpoint**: A conservative perspective might appreciate the emphasis on local and regional solutions to healthcare challenges, as well as the potential for AHECs to foster more efficient use of resources and support for family medicine, which could be seen as aligning with conservative values of family, community, and limited government intervention.<br><br>Overall, the statement is more centered on healthcare policy and education, which can be supported across the political spectrum depending on the specific aspects emphasized and the approach to implementation.	neutral
AAFP	Athletic Performance Enhancing Drugs	7/1905	The statement from the American Academy of Family Physicians (AAFP) regarding the use of drugs or blood products for medical purposes and not for the enhancement of athletic performance is primarily a medical or ethical stance, rather than a political one. It doesn't align specifically with either liberal or conservative viewpoints as it is focused on medical ethics and the appropriate use of medical treatments and substances.<br><br>Both liberal and conservative viewpoints typically agree on the importance of ethical standards in medicine, though they may differ in other areas of healthcare policy. The stance against using performance-enhancing drugs in sports is generally supported across the political spectrum as it relates to fairness in competition, health risks associated with drug misuse, and the integrity of athletic performances.	neutral
AAFP	Athletic Trainers for High School Athletes	9/2023	The text from the American Academy of Family Physicians (AAFP) focuses on recommendations for enhancing the well-being of student athletes in schools with interscholastic sports programs. The key points include emergency action planning, accessibility of automated external defibrillators, training coaches in first aid and CPR, recognizing concussion symptoms, and involving board-certified athletic trainers in the care team. Additionally, the AAFP suggests state boards of education and health departments collaborate with schools to develop and implement these policies.<br><br>This text primarily aligns with a non-partisan, health and safety-focused viewpoint. It emphasizes the importance of preparedness and care in the context of school sports programs, advocating for measures to ensure the safety and well-being of student athletes. These recommendations are grounded in public health and safety principles rather than political ideology, making them broadly applicable and not specifically aligned with either liberal or conservative viewpoints. The focus is on practical measures to enhance student safety in sports, a concern that transcends political affiliations.	neutral

AAFP	Blood, Tissue, and Biological Material Donation	10/2023	The text you've provided doesn't strictly align with either a liberal or conservative viewpoint in a traditional political sense. Instead, it represents a position based on medical ethics and public health policy.<br><br>The statement emphasizes the importance of voluntary donations of biological materials (like tissue, blood, and human milk) for a functioning healthcare system, which is a view generally supported across various political spectrums. The American Academy of Family Physicians (AAFP) encourages family physicians to discuss the importance of becoming a donor, which aligns with promoting public health and awareness.<br><br>The AAFP's opposition to lifetime donor bans and arbitrary time-based deferrals, especially those that stigmatize gender and sexual minorities, suggests a focus on inclusivity and evidence-based medicine. This perspective aligns more closely with contemporary liberal viewpoints, which often advocate for the rights and fair treatment of marginalized groups and evidence-based policy decisions.<br><br>However, the emphasis on evidence-based decisions and individual risk assessments over broad-based deferrals can resonate with certain conservative perspectives that favor individual assessment over group categorization.<br><br>Overall, the statement appears to be more aligned with public health and medical ethics rather than a strictly political ideology. It reflects a commitment to inclusivity, evidence-based practices, and the importance of voluntary donations in healthcare, which can find support across different political beliefs.	neutral
AAFP	Bloodborne Pathogens	7/1905	The text you provided, regarding the American Academy of Family Physicians' (AAFP) stance on the prevention and management of blood-borne infections, does not align explicitly with either a liberal or conservative viewpoint in the traditional political sense. It focuses on public health strategies and medical practices rather than political ideologies.<br><br>However, the specific recommendations might be received differently across the political spectrum:<br><br>1. **Immunizations, Safe Blood Banking, and Universal Precautions**: These are generally non-controversial public health measures supported across the political spectrum, although there may be varying degrees of emphasis or support for mandatory policies.<br><br>2. **Avoiding Use of Contaminated Needles and Needle Exchange Programs**: Needle exchange programs are typically more supported by liberals, who often view them as harm reduction strategies. Conservatives might be less supportive, viewing them as potentially enabling drug use.<br><br>3. **Providing Pre- and Post-Exposure Prophylaxis, and Access to Treatment**: Access to treatment is a public health issue that generally garners wide support, but the methods of funding and implementing such access can be politically divisive. Liberals often support broader access through public funding, while conservatives may favor more market-based solutions.<br><br>It's important to note that these are generalizations and there can be a wide range of views within each political ideology. Public health issues often transcend traditional political boundaries, as they focus on the well-being of the community as a whole.	neutral

AAFP	Breastfeeding (Policy Statement)	10/2023	The text you've provided seems to reflect a mix of viewpoints that align with both liberal and conservative perspectives, focusing primarily on the health and wellbeing aspects of breastfeeding, which can be viewed as universally important across political ideologies.<br><br>1. **Breastfeeding as a Norm and Health Benefits**: The emphasis on breastfeeding as the physiological norm and the detailed discussion of its medical and psychological benefits is a topic that can be embraced by both liberal and conservative viewpoints. The emphasis on natural processes and the importance of mother-child bonding can resonate with conservative values that emphasize traditional family structures and natural health practices. Simultaneously, liberals, who often prioritize science-based approaches to health and child rearing, would also support these aspects.<br><br>2. **Inclusive Language and Recognition of Diverse Family Structures**: The acknowledgement of gender diversity in parenting and the use of inclusive language (like "chestfeeding") aligns more closely with liberal values. Liberals typically emphasize inclusivity, diversity, and the recognition of various gender identities and family structures. This part of the text shows sensitivity to transgender and non-binary individuals, which is a key concern in liberal ideologies.<br><br>3. **Support and Education from Family Physicians**: The call for family physicians to be knowledgeable and supportive of breastfeeding, and to use inclusive language, is a healthcare-oriented approach that doesn't inherently align with a specific political ideology. Both liberals and conservatives value the role of healthcare professionals in providing appropriate care, although the reasons and contexts may differ.<br><br>Overall, the text combines elements that are important to both liberal and conservative viewpoints, with a strong emphasis on health and inclusivity. The focus is on promoting the best practices for child-rearing and supporting diverse family structures, which transcends traditional political divides.	neutral
AAFP	Breastfeeding and Lactation for Medical Trainees	7/1905	The text provided is primarily informational and educational, focusing on the benefits of breastfeeding and the need for support systems for breastfeeding medical trainees. It aligns with healthcare and educational policies rather than a specific political viewpoint. The emphasis is on the health benefits of breastfeeding for infants and mothers, the importance of support in the workplace, and the need for appropriate facilities and policies for breastfeeding medical trainees.<br><br>This text doesn't explicitly align with either a liberal or conservative viewpoint. It is more aligned with public health recommendations and best practices in workplace policy for supporting breastfeeding mothers. The recommendations and policies it advocates for are based on scientific research and medical consensus, rather than political ideology.	neutral
AAFP	Breastfeeding, Family Physicians Supporting (Position Paper)	1/2022	The text you've provided seems to be a comprehensive and evidence-based overview on breastfeeding, its benefits, historical context, and current practices and recommendations by health authorities. It does not explicitly align with either a liberal or conservative viewpoint. Instead, it focuses on medical and health-related facts, historical trends, and social considerations related to breastfeeding.<br><br>The document discusses the historical evolution of breastfeeding practices, the shift towards formula feeding, and the re-emergence of breastfeeding as a recommended practice due to its health benefits. It addresses public health recommendations, disparities in breastfeeding rates among different racial and ethnic groups, and the need for better training and support for breastfeeding in medical settings.<br><br>It also touches upon social and cultural aspects, such as the exploitation of wet nurses and body image issues, and recognizes the diverse family structures and gender identities involved in parenting and breastfeeding.<br><br>Overall, the text appears to be a well-rounded, medically-informed discussion on breastfeeding, rather than a piece advocating for a specific political ideology. It seems more focused on public health, medical education, and social awareness rather than aligning with a specific political viewpoint.	neutral
AAFP	Business Principles, Undergraduate Medical Education	9/2022	The text from the American Academy of Family Physicians (AAFP) about promoting health policy and health system management education in undergraduate medical training doesn't align strictly with a liberal or conservative viewpoint. It focuses on educational content in medical training, emphasizing the importance of understanding health policy, health system management, evolving payment models, and sustainable practice opportunities in primary care.<br><br>Such educational initiatives are generally viewed as practical measures to improve healthcare delivery and are not necessarily partisan in nature. They aim to equip future medical professionals with a comprehensive understanding of the healthcare system,	neutral

			which can lead to more informed and effective practice. This focus on education and practical skills development transcends traditional political divisions.	
AAFP	Cancer Care	1/2022	The text you provided is a policy statement from the American Academy of Family Physicians (AAFP) regarding comprehensive cancer care. It focuses on the importance of integrated healthcare teams at all stages of cancer care, emphasizing patient access to screening, diagnosis, specialty care, survivorship management, palliative care, and hospice care.<br><br>This statement doesn't align explicitly with a liberal or conservative viewpoint in the typical political sense. Instead, it reflects a medical and healthcare-oriented perspective. The emphasis on accessibility, evidence-based treatment, patient understanding, and integrated care approaches is consistent with general principles in modern healthcare, which prioritize patient-centered care, evidence-based practice, and interdisciplinary collaboration.<br><br>These principles are generally supported across a wide range of political views, as they focus on improving healthcare outcomes and quality of care for patients. The statement's emphasis on accessibility and affordability may resonate more with political viewpoints that prioritize healthcare reform and access, but these are not inherently liberal or conservative positions.<br><br>It's important to note that in healthcare policy, the focus is often on patient outcomes and evidence-based practices rather than political ideologies.	neutral
AAFP	Capitation, Primary Care	1/2022	The text you've provided outlines the American Academy of Family Physicians' (AAFP) policy on primary care capitation, which is a payment arrangement for healthcare services. This approach involves paying healthcare providers a set amount for each person attributed to them per period, regardless of the actual volume of services provided. The text doesn't explicitly align with a traditional liberal or conservative viewpoint as it's primarily focused on healthcare policy specifics rather than broader political ideologies.<br><br>However, the concept of capitation and its implementation can be viewed through different political lenses:<br><br>1. **Liberal Viewpoint**: Liberals might support capitation as it can promote more efficient and preventative care, potentially reducing overall healthcare costs and ensuring that resources are allocated based on patient needs rather than the volume of services rendered. The focus on risk adjustment factors like social determinants of health aligns with a liberal emphasis on addressing broader socio-economic factors in healthcare.<br><br>2. **Conservative Viewpoint**: Conservatives might have reservations about capitation. They generally favor market-driven solutions and might view capitation as a step towards managed care and away from fee-for-service models, which they often prefer. However, conservatives might support aspects of capitation that could lead to cost savings and reduced government spending in healthcare.<br><br>It's important to note that these are broad generalizations and the actual viewpoints can vary widely among individuals within each political spectrum. The focus of the text on practical aspects of healthcare payment systems, such as risk adjustment, scope of practice, and payment methodologies, suggests an approach aimed more at pragmatic healthcare management rather than ideological alignment.	neutral
AAFP	Care for the Transgender and Gender Nonbinary Patient	12/2023	The text provided aligns more closely with a liberal viewpoint. Liberal perspectives typically emphasize inclusivity, diversity, and the importance of acknowledging and supporting various gender identities. The statement from the American Academy of Family Physicians (AAFP) emphasizes the normality of gender diversity, the necessity of gender-affirming care, and the importance of education on these topics in medical training. It also advocates for legal protection and access to gender-affirming healthcare services, which are positions more commonly associated with liberal viewpoints.<br><br>Conservative viewpoints, on the other hand, might emphasize more traditional or biologically-based views of gender and could be less supportive of gender-affirming care, particularly for children and adolescents. They may also prioritize parental rights and involvement in healthcare decisions for minors over the informed consent model mentioned in the statement.	liberal

			It's important to note that these are general tendencies and there can be a wide range of opinions within both liberal and conservative spectrums.	
AAFP	Care Management Fees	1/2022	The text you've provided outlines a perspective on care management in healthcare, focusing on its definition, payment models, and recommendations for how it should be funded. This perspective aligns more closely with a liberal viewpoint, although it's important to note that issues in healthcare can be complex and may not fit neatly into a single ideological category. Here's why it leans towards a liberal viewpoint:<br><br>1. **Emphasis on Comprehensive Patient Care:** The focus on facilitating appropriate patient care, increasing patient satisfaction, and improving outcomes reflects a holistic approach to healthcare. Liberals often advocate for healthcare policies that prioritize comprehensive care and patient outcomes.<br><br>2. **Support for Non-Traditional Payment Models:** The text criticizes traditional fee-for-service payment models and suggests alternative payment methods, such as prospective payments. Liberals typically support moving away from fee-for-service models, which they often view as contributing to higher healthcare costs without improving care quality.<br><br>3. **Advocacy for Predictable Funding, Especially for Vulnerable Practices:** The suggestion that care management should be funded predictably and adequately, with special mention of rural and small, independent practices, reflects a concern for equity in healthcare access. This concern is more aligned with liberal values, which often emphasize support for underserved or vulnerable populations.<br><br>4. **Separate Compensation for Care Management Efforts:** The argument that expenses and outcomes/savings from care management efforts should be compensated separately can be seen as an endorsement of value-based care, a concept more commonly supported by liberal healthcare policies.<br><br>5. **Autonomy for Practices in Using Funds:** The idea that practices should have autonomy in how they use care management fees aligns with a liberal perspective on allowing healthcare providers the flexibility to address patient needs as they see fit.<br><br>However, it's essential to recognize that many aspects of healthcare policy, including care management, are subject to a wide range of viewpoints and the liberal-conservative spectrum may not fully capture the nuances of these discussions.	liberal
AAFP	Certificates of Added Qualification (CAQ)	1/2022	The text you've provided seems to be a policy statement from the American Academy of Family Physicians (AAFP) regarding the American Board of Family Medicine (ABFM) Certificate of Added Qualifications (CAQs). This statement focuses on the recognition of additional training in specific disciplines for family physicians and the criteria for credentialing.<br><br>In terms of political alignment, this statement does not clearly align with a traditional liberal or conservative viewpoint. It is primarily focused on professional standards and practices within the medical field. Such statements typically reflect the consensus or position of professionals in the field regarding best practices and standards, rather than a political ideology.<br><br>Liberal and conservative viewpoints often differ in areas such as government involvement in healthcare, social issues, economic policies, and environmental regulations. This statement, however, is more about professional guidelines and standards within the medical community, which generally transcends partisan political divides.<br><br>It's important to recognize that not all statements or policies can be neatly categorized into 'liberal' or 'conservative' viewpoints, especially those that are technical or professional in nature, like this one.	neutral

AAFP	Cesarean Delivery in Family Medicine (Position Paper)	7/2021	The text you've shared appears to be a comprehensive and detailed overview of the role of family physicians in obstetric care, particularly focusing on cesarean delivery. It addresses various aspects such as the scope of practice for family physicians, training methodologies, demonstration of proficiency, credentialing and privileges, and public health implications.<br><br>This text does not align distinctly with either a liberal or conservative viewpoint in the traditional political sense. Instead, it is a professional, medical document that focuses on the practical aspects of healthcare delivery, particularly in rural and underserved areas. It discusses the importance of training family physicians in cesarean deliveries to ensure adequate healthcare access in areas that might not have obstetricians/gynecologists available.<br><br>The text emphasizes the importance of equal access to healthcare, quality of care, and addressing disparities in healthcare outcomes (e.g., higher maternal mortality rates among Black individuals, individuals with low income, and those living in rural areas). These concerns can be seen as aligning with broader social justice and public health principles, which are sometimes more emphasized in liberal ideologies. However, the primary focus of the document is on practical and educational aspects of medical training and service provision, making it more of a professional guideline than a political statement.	neutral
AAFP	Chelation Therapy	5/2023	The text you've provided appears to be a statement on medical treatment, specifically regarding the use of chelation therapy. It's important to note that medical recommendations, such as this, are typically based on scientific evidence and clinical studies, rather than political ideologies like liberalism or conservatism.<br><br>In the text, the use of chelation therapy is recommended for heavy metal intoxication based on validated testing. However, it suggests that the use of chelation therapy for other conditions, such as atherosclerotic vascular or neuro-degenerative disease, is investigational and not recommended. This stance reflects a scientific, evidence-based approach to medicine, where treatments are recommended based on proven effectiveness and safety.<br><br>It's not accurate to categorize such a statement as aligning with a liberal or conservative viewpoint. Instead, it aligns with a medical and scientific perspective, emphasizing evidence-based practice. Political ideologies typically do not dictate medical treatments, as these are based on clinical evidence and guidelines set by medical professionals and organizations.	neutral
AAFP	Child Abuse	7/1905	The text you've provided does not inherently align with either a liberal or conservative viewpoint. It is a statement focused on the recognition, prevention, and treatment of child abuse, emphasizing the role of family physicians in these processes. The text highlights the importance of understanding child development, recognizing signs of abuse, and the necessity of reporting suspected abuse to the appropriate authorities. It also underscores the value of community resources for support and prioritizes the safety and well-being of the child.<br><br>This approach to child abuse is broadly professional and clinical, concerned with medical and social welfare practices. It doesn't explicitly endorse political ideologies but rather focuses on best practices in healthcare and child welfare. Child abuse is a concern across the political spectrum, and the measures described in the text are generally supported by a wide range of viewpoints for their focus on health and safety.	neutral
AAFP	Children's Health	9/2022	The text you provided appears to align more with a liberal viewpoint. This assessment is based on the emphasis on inclusivity, non-discrimination, and the specific mention of support for various groups such as those based on sexual orientation and gender identity. These themes are often associated with liberal or progressive ideologies, which typically advocate for the rights and protections of diverse groups, including LGBTQ+ individuals, and emphasize the importance of comprehensive healthcare access for all.<br><br>Conservative viewpoints, in contrast, might not place as much emphasis on these specific issues or may approach them differently, often prioritizing traditional values or different interpretations of rights and freedoms.<br><br>It's important to note that the classification of viewpoints as "liberal" or "conservative" can vary depending on the context and the specific issues at hand. Additionally, individuals and organizations may hold a mix of views that don't neatly fit into one category.	liberal

AAFP	Chronic Pain Management and Opioid Misuse: A Public Health Concern (Position Paper)	7/1905	The executive summary you provided does not neatly align with a strictly liberal or conservative viewpoint. Instead, it presents a complex and multifaceted approach to managing chronic pain and opioid misuse, which encompasses elements that could be supported by individuals across the political spectrum. Here are some key points:<br><br>1. **Focus on Patient-Centered Care**: The emphasis on patient-centered, compassionate care aligns with a more holistic and empathetic approach to healthcare, which can be a value shared across political lines.<br><br>2. **Emphasis on Multidisciplinary Care and Collaboration**: The call for collaboration among healthcare professionals and the integration of various therapeutic modalities, such as medication-assisted treatment (MAT), and community resources, reflects a comprehensive approach to healthcare that transcends simple political categorizations.<br><br>3. **Recognition of Opioid Misuse as a Public Health Issue**: The document acknowledges the complexity of opioid misuse and chronic pain as interrelated public health crises. This recognition is important to both liberal and conservative viewpoints, as it addresses the need for systemic change (more liberal) while also recognizing individual and community responsibility (more conservative).<br><br>4. **Advocacy for Research and Evidence-Based Practice**: The call for more research and evidence-based guidelines could be seen as a scientifically grounded approach that appeals to a more progressive or liberal standpoint. However, it's also pragmatic and can appeal to more conservative views that emphasize effective and accountable healthcare practices.<br><br>5. **Policy and Education Recommendations**: The recommendations for changes in education, policy, and advocacy, such as adjusting payment models and expanding insurance coverage for MAT, can be seen as progressive. However, they are also rooted in practical considerations of healthcare effectiveness and resource management, which can align with more conservative fiscal perspectives.<br><br>6. **Balance of Pain Management and Opioid Risk**: The emphasis on balancing effective pain management with the risks of opioid misuse reflects a nuanced understanding of healthcare, avoiding overly simplistic solutions that might be more typical of partisan viewpoints.<br><br>Overall, the document represents a balanced, comprehensive, and pragmatic approach to a complex healthcare issue, incorporating elements that could be supported by both liberal and conservative viewpoints. It underscores the need for collaborative, evidence-based, and compassionate healthcare strategies that address both individual needs and broader public health concerns.	neutral
AAFP	Civil Marriage Equality	10/2023	The statement from the American Academy of Family Physicians (AAFP) supports the right to civil marriage for all couples regardless of gender, sex, race, ethnicity, or national origin, with the aim of contributing to overall health, longevity, and improved family stability. This perspective generally aligns with a liberal viewpoint. Liberal ideology typically emphasizes equality, individual rights, and support for social policies that promote inclusivity and diversity. The support for marriage equality regardless of gender, sex, race, or ethnicity is consistent with liberal values that advocate for equal rights and non-discrimination.	liberal
AAFP	Clinical Outcomes, Disclosing Unanticipated: A Resource Guide for Family Physicians (Position Paper)	10/2023	The text you've provided doesn't align strictly with either a liberal or conservative viewpoint as it primarily discusses the ethical, legal, and professional aspects of physician-patient communication, particularly in the context of disclosing adverse events. The focus is on the importance of transparency, patient safety, and effective communication in healthcare, which are concerns shared across the political spectrum.<br><br>The American Academy of Family Physicians (AAFP) and the CANDOR toolkit advocate for open communication between physicians and patients, emphasizing ethical responsibility and patient rights, which can be seen as universally important regardless of political ideology. The mention of apology laws and CANDOR legislation shows a blend of legal and ethical considerations, highlighting the complex interplay between healthcare, law, and policy. These issues transcend simple political categorization as they involve ethical considerations and practical implications that are relevant to both conservatives and liberals, each perhaps appreciating different aspects of the approach based on their values and beliefs.	neutral

AAFP	Clinical Practice Guidelines Policy	5/2023	The text from the American Academy of Family Physicians (AAFP) doesn't inherently align with a traditional liberal or conservative viewpoint. It focuses on advocating for evidence-based clinical practice guidelines, emphasizing patient-centered care, transparency, and minimizing bias. These principles are grounded in scientific best practices and quality care, rather than political ideology.<br><br>Here's why it doesn't clearly align with one side:<br><br>1. **Liberal Viewpoints:**<br>- Liberals generally advocate for evidence-based policymaking, healthcare equity, and comprehensive patient care, which aligns with the AAFP's advocacy for patient-centered, evidence-based guidelines.<br>- The emphasis on minimizing financial and intellectual bias and involving patient representatives might resonate with liberal ideologies that focus on inclusivity and reducing corporate influence in healthcare.<br><br>2. **Conservative Viewpoints:**<br>- Conservatives often emphasize personal responsibility, cost-effectiveness, and minimizing government intervention in healthcare, which can align with the AAFP's focus on feasible, measurable, and achievable guidelines.<br>- The stress on clinician's judgment and competency over specialty might appeal to conservative values of individualism and professional autonomy.<br><br>In summary, the statement is more about promoting scientifically sound, unbiased, and effective healthcare practices than advocating for a specific political ideology. It's more aligned with professional and ethical standards in medicine than with the political spectrum.	neutral
AAFP	Clinical Procedural Skills, Support	1/2022	The text you provided seems to be a policy or position statement from the American Academy of Family Physicians (AAFP). It doesn't align strictly with a liberal or conservative viewpoint as it focuses on procedural skills courses for family physicians, collaboration with other specialties, and the roles of family physicians in hospitals and healthcare organizations.<br><br>However, the themes it touches upon could be interpreted differently depending on one's political viewpoint:<br><br>1. **Liberal Viewpoint:** Liberals might focus on the collaborative approach and support for comprehensive training as a means to ensure high-quality healthcare for all. They might also appreciate the emphasis on continued education and credentialing as it aligns with a general preference for regulated and standardized practices in healthcare.<br><br>2. **Conservative Viewpoint:** Conservatives might appreciate the focus on individual physician's rights and responsibilities, emphasizing the role of family physicians in healthcare organizations. The mention of "joint political action" could be seen as a call for professional autonomy and less government intervention, which often aligns with conservative values.<br><br>Overall, the statement is professional and focused on healthcare standards rather than political ideology. Different aspects might resonate differently with individuals across the political spectrum, but it doesn't strictly advocate for a liberal or conservative viewpoint. It's more centered around professional best practices and collaboration within the healthcare industry.	neutral
AAFP	Clinical Proctoring	7/1905	The text from the American Academy of Family Physicians (AAFP) regarding clinical proctoring doesn't inherently align with a specific liberal or conservative viewpoint. It's a position statement focused on ensuring quality and competence in the healthcare field, particularly among physicians seeking hospital privileges. The emphasis is on peer review, equal application of requirements, and structured evaluation methods, which are professional and practical matters rather than ideological or political ones.<br><br>Both liberal and conservative individuals or groups might support these measures as they relate to improving healthcare quality, ensuring patient safety, and maintaining high standards of professional practice. Liberals might appreciate the emphasis on equality and thoroughness in the evaluation process, while conservatives might value the focus on professional competence and accountability. Ultimately, the text is centered around best practices in a medical context rather than political ideology.	neutral

AAFP	Clinical Skills Assessment for Learners	1/2022	The text you've provided is a policy statement from the American Academy of Family Physicians (AAFP) concerning the assessment of clinical skills in medical students. It doesn't explicitly align with a liberal or conservative viewpoint. Instead, it focuses on:<br><br>1. **Educational Standards and Competency**: Emphasizing the importance of thorough and standardized clinical skills training and assessment in medical schools.<br>2. **Access and Affordability**: Advocating for affordable and accessible clinical skills examinations by national boards like the NBME and NBOME.<br>3. **Fairness and Transparency**: Calling for transparent and fair testing practices, standards, and feedback for students.<br>4. **Regulatory Recommendations**: Suggesting that without proper national assessments, state licensing boards should reconsider their examination requirements.<br><br>Such a statement is primarily centered on educational policy, professionalism, and ensuring the quality of medical education and practice. It's more focused on practical, educational, and ethical considerations rather than ideological ones. Both liberal and conservative viewpoints might find aspects to agree with, such as the emphasis on high standards in medical training (a conservative angle on maintaining traditions of rigor and excellence) or the focus on accessibility and fairness (a liberal angle on reducing barriers and ensuring equality). However, it's important to note that educational and professional standards often transcend simple political categorization.	neutral
AAFP	CME Credit, AAFP/AMA Equivalency Agreement	4/2020	The text you've provided is a factual statement about the reciprocal recognition of credit between the American Medical Association (AMA) and the American Academy of Family Physicians (AAFP). It outlines the specifics of how credits from one organization are accepted by the other and the appropriate way to report these credits. This information is procedural and administrative in nature and does not inherently align with a liberal or conservative viewpoint. It's focused on the logistics of professional recognition and continuing education for physicians, which is a non-partisan issue. It doesn't advocate for policy or express opinions on issues that typically divide liberal and conservative ideologies.	neutral
AAFP	CME Credit, AAFP/CFPC Reciprocal Agreement	7/1905	The text you've provided outlines a bilateral reciprocal certification agreement between the American Academy of Family Physicians (AAFP) and the College of Family Physicians of Canada (CFPC) regarding Continuing Medical Education (CME) and Continuing Professional Development (CPD) activities. It describes the procedural details for certifying activities based on the primary target audience's nationality and the conversion of credits between the two organizations.<br><br>This text is primarily administrative and procedural, focusing on the specifics of how CME/CPD activities are certified and credited between the AAFP and CFPC. It doesn't inherently align with a liberal or conservative viewpoint as it's a neutral, factual description of an agreement between two professional medical organizations. It doesn't advocate for any political or social policies, nor does it touch upon any typically partisan issues. Instead, it aims to provide clarity and guidelines for medical professionals seeking certification and credit for their continuing education, a goal that is generally non-partisan and focused on professional development and standards rather than political ideology.	neutral
AAFP	CME Credits, AAFP/AOA Equivalency Agreement	7/1905	The text you've provided is a factual statement about the interchangeability and recognition of continuing medical education (CME) credits between the American Osteopathic Association (AOA) and the American Academy of Family Physicians (AAFP). It outlines the types of credits available and the conditions under which they are recognized by each association.<br><br>This information is administrative in nature and does not inherently align with a liberal or conservative viewpoint. It's focused on professional development and accreditation within the medical community, which is typically a non-partisan topic. Both liberal and conservative physicians, as well as those with other political beliefs, would be equally subject to these rules and guidelines in their professional practice.	neutral
AAFP	CME Credits, AAFP/DHP Equivalency Agreement	2020	The text you've provided doesn't inherently align with either a liberal or conservative viewpoint. It primarily discusses the mutual recognition of continuing medical education credits between the American Academy of Family Physicians (AAFP) and the Department of Healthcare Professions (DHP), Ministry of Public Health - Qatar. This is a procedural and administrative matter concerning the accreditation and reporting of professional education credits. It doesn't touch upon any ideological issues or policy debates typically associated with liberal or conservative politics. Instead, it focuses on professional standards and procedures within the medical community.	neutral

AAFP	CME Mandatory for Relicensure	10/2023	The text primarily reflects a moderate or centrist viewpoint, leaning towards a professional and pragmatic approach to medical education and licensure rather than a distinctly liberal or conservative political ideology. Here's how:<br><br>1. **Support for Lifelong Learning and CME**: The support for lifelong learning and Continuing Medical Education (CME) as a required component of relicensure aligns with a broader, non-partisan understanding of the importance of ongoing education in maintaining high standards in the medical profession.<br><br>2. **State Licensure and Public Protection**: The acknowledgment of state licensure as mandatory and aligned with public protection and safety is a pragmatic stance, recognizing the role of regulation in ensuring quality and safety in healthcare. This is not inherently liberal or conservative but rather a professional standard.<br><br>3. **Against Mandated Topic-Specific CME**: The opposition to mandated topic-specific CME for relicensure suggests a preference for physician autonomy and professional judgment. This could be seen as leaning slightly towards a conservative viewpoint favoring less prescriptive regulation, but it's primarily rooted in professional autonomy rather than a broader political ideology.<br><br>4. **Encouragement of Involvement in Boards and Committees**: The encouragement for representatives to be involved in boards or committees that mandate CME reflects a proactive and participatory approach to professional governance. This is more about ensuring that those affected by policies have a say in them, which is a democratic principle rather than a strictly liberal or conservative one.<br><br>Overall, the text is more aligned with a professional and industry-specific perspective, focusing on the balance between regulation, quality of care, and professional autonomy, rather than a clear-cut liberal or conservative political stance.	neutral
AAFP	CME Mission Statement	4/2022	The text provided outlines the goals and principles of the American Academy of Family Physicians (AAFP) regarding its Continuing Medical Education (CME) program. The text is primarily educational and professional in nature, focusing on the delivery of high-quality, unbiased, evidence-based educational activities for family physicians and healthcare professionals. The emphasis is on adult learning principles, a variety of formats, and the practical application of learning to improve medical practice.<br><br>The content doesn't inherently align with a liberal or conservative viewpoint as it's focused on medical education and professional development rather than political ideology. Its emphasis on evidence-based practice and unbiased information is a cornerstone of scientific and medical communities, which typically prioritize data and outcomes over political leanings.<br><br>Both liberal and conservative individuals in the healthcare field might support the idea of continuous learning and improvement in medical practice based on the latest evidence and best practices. As such, the text seems to be neutral and professional, designed to appeal to a wide audience of medical practitioners regardless of their personal political beliefs.	neutral
AAFP	CME Requirements for AAFP Membership	9/2021	The text provided outlines continuing medical education (CME) requirements for active members of a professional association, with specifics on the types and sources of credits needed. It doesn't inherently align with a liberal or conservative viewpoint. Issues of education, professional development, and organizational requirements are typically non-partisan and focus on maintaining standards within a profession rather than reflecting political ideologies. Both liberal and conservative viewpoints might support the idea of continued education and professional standards as a means to ensure quality and competence in the field.	neutral

AAFP	Co-Payments	7/1905	The text provided outlines the American Academy of Family Physicians (AAFP)'s position on co-payments within medical practice settings. It doesn't explicitly align with a liberal or conservative viewpoint. Instead, it focuses on policy suggestions and guidelines for the application of co-payments in healthcare.<br><br>Liberal viewpoints generally emphasize access to healthcare as a right and might support measures to reduce out-of-pocket costs for patients. They might favor the idea of waiving co-payments in cases of financial hardship and encouraging preventive care through single co-payments for multiple services.<br><br>Conservative viewpoints typically stress personal responsibility and market-driven solutions in healthcare. They might support co-payments as a way to encourage responsible use of medical services and to help control healthcare costs.<br><br>The text touches on aspects that could resonate with both viewpoints:<br>1. **Differential Co-Payments**: Encouraging patients to maintain a medical home could be seen as promoting personal responsibility (a conservative angle) while also aiming to ensure better health outcomes and potentially lower overall costs (a liberal angle).<br>2. **Multiple Co-Payments**: This could be seen as a conservative approach by ensuring that each service is accounted for, potentially discouraging unnecessary use of services.<br>3. **Waivers**: The ability to waive co-payments for financial hardship aligns more with a liberal viewpoint emphasizing access and equity in healthcare.<br><br>Ultimately, the text seems to aim for a practical approach to managing co-payments in a way that balances cost control with access to necessary medical services, reflecting elements that could be supported by both liberal and conservative perspectives.	neutral
AAFP	Coding and Payment	10/2023	The text you've provided outlines the American Academy of Family Physicians (AAFP)'s stance on procedural coding and payment systems in healthcare. It primarily focuses on administrative practices, standardization, and fair payment processes. This text doesn't inherently align with a strictly liberal or conservative viewpoint, as it's more focused on practical and operational aspects of healthcare administration rather than ideological positions.<br><br>Both liberals and conservatives might find aspects to agree with. For instance, conservatives might appreciate the emphasis on standardized procedures and the potential for reducing bureaucratic inefficiency. On the other hand, liberals might support the call for fair payment practices and ensuring physicians are adequately compensated for the full scope of their services. Both sides might agree on the importance of clear, consistent coding practices for efficiency and transparency in billing.<br><br>It's also worth noting that healthcare policy is a complex area with a wide range of perspectives within both liberal and conservative camps, so individuals might align with different aspects of the statement based on their personal beliefs and experiences within the healthcare system.	neutral
AAFP	Collecting Racial, Ethnic, Sexual Orientation, and Gender Identity Data in Surveys	2019	The text from the American Academy of Family Physicians (AAFP) doesn't align strictly with a liberal or conservative viewpoint as it focuses on public health policy and practice. However, the emphasis on collecting detailed information on race, ethnicity, sexual orientation, and gender identity to address health disparities and ensure equitable distribution of resources is generally more aligned with liberal viewpoints. Liberals tend to advocate for policies that recognize and address systemic inequalities and support marginalized communities. The conservative viewpoint traditionally emphasizes individual responsibility and might be less focused on the collection of such specific demographic data, arguing for more limited government intervention. However, perspectives within each political ideology can vary widely, and public health is a field where bipartisan agreement can sometimes be found on specific issues.	probably liberal
AAFP	Collective Bargaining as an Integral Part of Single Payer	7/1905	The statement "The AAFP unequivocally supports the right of physicians to organize and bargain collectively" aligns more with a liberal viewpoint. Generally, liberal ideologies in the United States tend to support labor rights, including the right to organize and collective bargaining. These principles are often associated with protecting workers' rights and promoting fair labor standards, which are typically endorsed by liberal groups and labor unions. Conservative viewpoints, on the other hand, might emphasize free market principles and may be more critical of unions and collective bargaining, viewing them as potentially disruptive to business and economic growth. However, it's important to note that individuals' views can vary, and not all liberals or conservatives might align perfectly with these generalizations.	liberal

AAFP	Collective Negotiation	1/2022	The text you've provided generally aligns more with a liberal viewpoint. The emphasis on improving access to the healthcare system, reducing costs, and advocating for primary care physicians to negotiate with health insurance companies reflects a liberal stance that often prioritizes increased regulation and support for public welfare initiatives. Conservatives typically favor less government intervention in business and might argue that such exemptions could lead to reduced competition and higher costs. However, viewpoints can vary within each political ideology, and some conservatives might support certain aspects of the proposal if they believe it could lead to a more efficient and cost-effective healthcare system. It's also important to note that these are broad generalizations and individuals' views may differ.	liberal
AAFP	Colonoscopy (Position Paper)	7/2020	The text provided doesn't inherently align with a liberal or conservative viewpoint. It's a medical document focusing on the practice, benefits, and training surrounding colonoscopy, particularly in the context of family physicians. The content discusses the procedure's importance in colorectal cancer prevention and detection, training requirements for family physicians, and the potential community benefits of increased access to this service.<br><br>However, the implications or interpretations of the text could be viewed through different ideological lenses:<br><br>1. **Liberal Viewpoint**: From a liberal perspective, one might focus on the potential for increased access to healthcare and preventive services. The idea that family physicians in rural and underserved areas could provide colonoscopy services might be seen as a way to reduce healthcare disparities and improve public health outcomes.<br><br>2. **Conservative Viewpoint**: A conservative viewpoint might appreciate the emphasis on individual physician discretion and flexibility in scope of practice. The focus on community implications and allowing family physicians to expand their services could be seen as a way to support local healthcare solutions and reduce reliance on specialized and potentially more costly services.<br><br>Overall, the text is more clinical and educational in nature, primarily focused on medical practice rather than political ideology. How it's interpreted might depend more on the reader's existing viewpoints and how they believe healthcare should be managed and provided.0	neutral
AAFP	Colposcopy (Position Paper)	7/2020	The text you've provided is predominantly factual and medical in nature, focusing on the prevalence, diagnosis, screening, and management of cervical cancer, along with recommendations and guidelines for healthcare providers. It doesn't inherently align with a liberal or conservative viewpoint as it mainly presents medical facts, statistics, and recommendations based on scientific research and healthcare policies.<br><br>Political views typically become relevant when discussing the implementation of healthcare policies, funding for medical research, or access to healthcare services, including cancer screening and prevention programs. Liberals and conservatives might have differing opinions on how to fund and manage these services, but the text itself is neutral, focusing on medical information rather than policy or political ideology. It's more about medical practice and public health than about politics.	neutral
AAFP	Commission Meetings, Officer Attendance At	4/2022	The text you've provided appears to be a procedural guideline regarding the attendance and prioritization of participants in cluster meetings, specifying a limit on the number of officers and giving priority to certain positions. This does not inherently align with a liberal or conservative viewpoint as it's administrative in nature and doesn't touch on any ideological issues typically associated with these political spectrums. It's more about governance and operational procedures within an organization rather than a statement on broader political or social policies.	neutral
AAFP	Community and Migrant Health Centers	7/1905	The text you've provided reflects a viewpoint that aligns more closely with a liberal perspective, typically characterized by a focus on increasing access to healthcare and support for underserved communities. It emphasizes the importance of community and migrant health center programs, which are often supported by policies that aim to provide more equitable healthcare access. Such positions are usually associated with liberal or progressive ideologies that prioritize social welfare and public health initiatives. However, it's important to note that the desire to improve healthcare access can cross ideological lines, and there may be conservatives who also support such measures for various reasons.	liberal
AAFP	Comprehensive Care, Access to	10/2023	The text from the American Academy of Family Physicians (AAFP) regarding access to essential health care for all, regardless of social and economic status, typically aligns more closely with a liberal viewpoint. This is because liberal ideologies generally emphasize the importance of equal access to healthcare as a fundamental right and support the idea of governmental involvement in providing and ensuring this access. The mention of identifying appropriate funding for these services also aligns with liberal perspectives that often advocate for public funding and support of healthcare systems to ensure broad, equitable access. However, it's important to note that the concept of universal healthcare access can also find support among some conservatives, especially those who focus on the pragmatic aspects of healthcare efficiency and preventive care. The viewpoints can vary widely within each political spectrum, and not all liberals or conservatives might fully agree with the statement.	liberal

AAFP	Comprehensive Care, Definition of	7/2023	The text you've provided outlines a healthcare approach focused on comprehensive care over time, considering various aspects of a patient's life. This approach doesn't inherently align with a liberal or conservative viewpoint as it is a healthcare model rather than a political statement. Both political sides might interpret and support it differently based on their broader healthcare policies and philosophies:<br><br>1. **Liberal Viewpoint**: Liberals might support this as it aligns with a holistic approach to healthcare, emphasizing preventative care, mental health, and environmental factors. They may view it as a way to ensure equitable access to comprehensive healthcare services for all, which is in line with liberal values of inclusivity and social welfare.<br><br>2. **Conservative Viewpoint**: Conservatives might appreciate the focus on preventative care, which can be seen as a way to reduce long-term healthcare costs, a common conservative concern. They might also support the individualized aspect of the care, which aligns with conservative values of personal responsibility and individual choice.<br><br>However, the exact alignment can vary based on how this practice is implemented, funded, and its implications on issues like government involvement in healthcare, individual freedom, and economic impact. Therefore, the text itself is neutral, but its interpretation can skew liberal or conservative based on broader contexts.	neutral
AAFP	Confidentiality, Patient/Physician	9/2022	The text primarily aligns with a liberal viewpoint due to its emphasis on individual rights, patient confidentiality, and comprehensive access to healthcare information. The principles outlined reflect a liberal stance on privacy and healthcare, advocating for patient empowerment, protection of personal data, and the importance of a trusting relationship between physician and patient. It supports individual rights and access to personal medical records while also recognizing the need for certain regulations and standards in the sharing of medical information. The focus on safeguarding patient rights and advocating for standardized practices in medical information handling reflects liberal values of personal freedom, privacy, and the importance of regulatory frameworks to protect these rights.	liberal
AAFP	Considerations for Pregnancy, Breastfeeding, and COVID-19	1/2022	The text provided outlines a medically-informed and public health-oriented viewpoint rather than a strictly liberal or conservative one. It discusses the potential risks and benefits of COVID-19 vaccination for pregnant or lactating patients, based on available scientific evidence and public health recommendations. The focus is on patient safety, informed decision-making, and the inclusion of pregnant and lactating individuals in vaccine trials and health discussions. The stance of encouraging discussion between patients and physicians, promoting breastfeeding while taking precautions against COVID-19, and supporting ongoing scientific research doesn't neatly align with a particular political ideology but rather reflects a healthcare perspective prioritizing patient care and public health.<br><br>Both liberal and conservative viewpoints may find aspects of this text to agree with or contest based on their broader perspectives on healthcare, public health policy, and individual rights. For instance, liberals might support the emphasis on inclusive healthcare and informed patient choice, while conservatives might appreciate the focus on personal medical discussions between patients and physicians. However, the text itself is primarily medical and public health-focused rather than politically driven.	neutral
AAFP	Consultations	10/2023	The text provided doesn't neatly align with a traditional liberal or conservative viewpoint as it primarily addresses professional standards and ethics in the medical field. It emphasizes the importance of consultation among medical staff to maintain high standards of care, ensure timely assistance, and avoid discrimination based on specialty or department. Both liberal and conservative ideologies might support these principles for different reasons.<br><br>Liberals might agree with the emphasis on collaboration and equal access to consultation as a means to ensure equitable and high-quality healthcare. They may view it as a way to uphold patient rights and support comprehensive care.<br><br>Conservatives might agree with the principles outlined as they emphasize professional autonomy, individual capabilities, and potentially resist overregulation by external entities. They may see the guidelines as a way to maintain high standards of practice and ensure that physicians' judgment is respected.<br><br>Overall, the text focuses on ethical and professional standards in medicine rather than political ideology. Its principles could be supported by individuals across the political spectrum who value high-quality healthcare, professional integrity, and patient well-being.	neutral

AAFP	Consultations, Referrals, and Transfers of Care	10/2023	The text you've provided is primarily factual and informative, focused on defining medical terms and procedures rather than advocating for a specific ideological viewpoint. It outlines the definitions and distinctions between "consultation," "referral," and "transfer of care" as understood by the American Academy of Family Physicians (AAFP). This content is more aligned with professional medical guidelines and practices rather than a liberal or conservative political stance.<br><br>However, if one were to infer any ideological leanings based on the content, it might be through the lens of how each ideology views healthcare management:<br><br>1. **Liberal Viewpoint**: Liberals generally advocate for comprehensive healthcare access and might support the detailed delineation of responsibilities and communications between physicians as a way to ensure high-quality patient care and autonomy. The emphasis on clear communication, patient preferences, and collaboration could resonate with liberal values of inclusivity and patient rights.<br><br>2. **Conservative Viewpoint**: Conservatives might appreciate the emphasis on professional judgment and the structured approach to healthcare management, aligning with conservative values of individual responsibility (in this case, of the physician) and efficient systems. The clear definitions and expectations could be seen as a way to maintain high standards and accountability in healthcare.<br><br>Overall, the text is neutral and aims to clarify professional medical terms rather than promote a political ideology. Any alignment with liberal or conservative viewpoints would be a matter of interpretation based on broader values and beliefs about healthcare management.	neutral
AAFP	Continuing Medical Education (CME), Definition	6/2023	The text you've provided is a neutral description of Continuing Medical Education (CME) and doesn't inherently align with a liberal or conservative viewpoint. It focuses on the educational process and objectives for healthcare professionals to maintain and enhance their skills. It doesn't touch on political or ideological issues that would typically characterize a liberal or conservative stance. Instead, it's a factual statement about a professional development practice in the healthcare field.	neutral
AAFP	Continuing Medical Education, AAFP Activities and Industry Funding	7/1905	The text outlines policies and guidelines regarding continuing medical education (CME) from the American Academy of Family Physicians (AAFP). It doesn't explicitly align with a liberal or conservative viewpoint. Instead, it focuses on maintaining responsibility, objectivity, balance, and independence in CME activities, especially regarding financial support and relationships with proprietary entities. It emphasizes adherence to ethical guidelines and opposes government enforcement of these guidelines, suggesting a preference for self-regulation within the medical profession. This might resonate more with conservative views favoring less government intervention. However, it also supports transparency and ethical standards, which can align with liberal values of accountability and integrity. Overall, the text is more professional and regulatory than politically partisan.	neutral
AAFP	Continuity and Coordination of Care, Long-Term and Subacute Facilities	10/2023	The text from the American Academy of Family Physicians (AAFP) regarding the role of family physicians in long-term and extended care facilities is primarily professional and healthcare-oriented, rather than political. It doesn't directly align with a liberal or conservative viewpoint as it focuses on advocating for patient care, promoting quality healthcare, and coordinating care in various facilities. Both liberal and conservative individuals might support these principles as part of a broader commitment to quality healthcare, albeit potentially with different approaches to healthcare policy and funding. Therefore, it's more accurate to say this text reflects a healthcare professional perspective rather than a political one.	neutral
AAFP	Continuity of Care, Definition of	2020	The text provided doesn't align strictly with either a liberal or conservative viewpoint as it's primarily focused on healthcare policy and practice rather than political ideology. However, the emphasis on continuity of care, cost-effectiveness, and a physician-led, team-based approach to health care might be interpreted differently depending on individual political perspectives.<br><br>Those with a more conservative viewpoint might appreciate the focus on cost-effectiveness and the role of personal responsibility, as the text emphasizes the patient and physician working cooperatively and the physician’s role in early recognition of problems, potentially reducing the need for more extensive, expensive treatments.<br><br>On the other hand, liberals might appreciate the emphasis on quality care, patient safety, and the idea of a medical home, which aligns with a more collective approach to healthcare, ensuring that all individuals have access to consistent and comprehensive care.<br><br>Overall, the text is more professional and neutral, focusing on best practices in family medicine rather than advocating for a particular political stance. It aims to present a standard in medical care that's beneficial for all patients, which is a goal that both liberals and conservatives might find valuable in their own ways.	neutral

AAFP	Coverage Equity for Drugs, Testing, Procedure, Preventive Services, and Reproductive Technologies	10/2023	The text reflects a liberal viewpoint. It emphasizes non-discrimination and inclusive health coverage based on gender identity, sexual orientation, and marital status. The support for comprehensive coverage of reproductive services, elective sterilization, and especially transition-related care for transgender individuals aligns with liberal perspectives that advocate for expanded healthcare rights and recognition of diverse identities. The emphasis on patient-centered care and evidence-based standards also reflects liberal values of individual autonomy and science-based policy. Conservatives might be more hesitant about the broad inclusion of services and the specific focus on gender identity and reproductive rights, as conservative viewpoints often prioritize traditional family structures and may have concerns about the extent and nature of healthcare coverage.	liberal
AAFP	Coverage of Self-Measured Blood Pressure Devices	10/2023	The statement from the American Academy of Family Physicians (AAFP) regarding the coverage of validated, at-home self-measured blood pressure monitoring devices under public and private benefit plans aligns with neither a strictly liberal nor conservative viewpoint exclusively. It focuses on a healthcare policy that emphasizes evidence-based practice and patient support. Generally, advocating for expanded healthcare coverage and emphasizing evidence-based medicine can be seen as aligning with more progressive or liberal healthcare policies. However, the emphasis on clinical validation and the potential for reducing long-term healthcare costs through better early management can also resonate with more conservative viewpoints that focus on efficient healthcare spending and reducing unnecessary interventions. Therefore, this statement can be seen as a healthcare policy position that transcends traditional partisan lines, focusing instead on practical, evidence-based patient care and healthcare efficiency.	neutral
AAFP	Coverage of Vitamin D Supplementation for Infants	7/1905	This text primarily focuses on health recommendations regarding vitamin D supplementation for infants and doesn't inherently align with a liberal or conservative viewpoint. It discusses the medical perspective and guidelines issued by the American Academy of Family Physicians (AAFP) concerning vitamin D deficiency and its implications for infants. The mention of third-party payer coverage is a healthcare policy issue, which might be interpreted differently based on individual political views, but the text itself is neutral and focused on health guidelines rather than political ideology. It's important to note that healthcare topics can become politicized, but the fundamental concern of this statement is the well-being of infants, not promoting a particular political stance.	neutral
AAFP	Coverage, Patient Education, and Counseling for Family Planning, Contraceptive Methods, and Sterilization Procedures	7/1905	The text from the American Academy of Family Physicians (AAFP) primarily aligns with a liberal viewpoint. It advocates for comprehensive coverage and access to various contraceptive methods, sterilization procedures, and education on sexual health without cost-sharing. It emphasizes the importance of Long-Acting Reversible Contraception (LARC) as a first-line method and supports ensuring adequate payment for these services. Additionally, it focuses on the importance of sexual health education for both men and women, aiming to reduce unwanted pregnancies and inform about sexually transmitted diseases.<br><br>In the U.S. political context, advocating for broad access to contraception and education on sexual health, especially without cost, is generally associated with liberal or progressive ideologies. These viewpoints tend to emphasize reproductive rights, healthcare access, and comprehensive sexual education as part of individual freedoms and public health. Conservative viewpoints, on the other hand, might focus more on abstinence education, have reservations about certain types of contraception, or oppose government-mandated healthcare provisions.	liberal
AAFP	COVID-19 Therapeutics	9/2022	The text from the American Academy of Family Physicians (AAFP) primarily aligns with a viewpoint that values scientific evidence and cautious, ethical medical practice over any particular political ideology. However, how it's interpreted could vary between liberal and conservative perspectives.<br><br>**Liberal Viewpoint:**<br>- Liberals generally support strong regulatory agencies like the FDA and value the role of government in ensuring public health and safety. The AAFP's emphasis on FDA approval and evidence-based treatment aligns with this perspective.<br>- The call for transparent and rapid evaluation of potential treatments might also resonate with liberal values of transparency and accountability in both the healthcare system and government.<br><br>**Conservative Viewpoint:**<br>- Conservatives often emphasize individual choice and might support the right of physicians and patients to try unapproved drugs, especially in life-threatening situations. Some might view the AAFP's caution as a limitation on personal freedom or a slow bureaucratic approach.<br>- However, many conservatives also value prudent use of resources and might agree with the AAFP's stance on not stockpiling or widely prescribing unproven treatments, as it aligns with fiscally conservative principles.<br><br>In essence, the statement is professional and scientific, focusing on evidence-based medicine and ethical considerations. It doesn't inherently align with a political ideology but could be supported or criticized by individuals across the political spectrum depending on their values and beliefs about healthcare, government regulation, and individual rights.	neutral

AAFP	Credentialing and Privileging Decisions of Family Physicians by Non-Physician Practitioners	2020	The text reflects a conservative viewpoint towards healthcare professional roles and scope of practice. It emphasizes a traditional hierarchy in medical practice, advocating for the authority and expertise of residency-trained, board-certified family physicians over non-physician practitioners. This stance is typical of conservative perspectives that favor established structures and roles, especially in professional fields. The text underscores a resistance to change in the healthcare system and supports maintaining a clear distinction and hierarchy between different levels of medical practitioners.	conservative
AAFP	Criminalization of the Medical Practice	12/2023	The text you provided aligns more with a liberal viewpoint. It emphasizes the protection of clinicians and patients in the context of reproductive, abortion, and gender-affirming healthcare, advocating against the criminalization of these services. This stance generally aligns with liberal ideologies, which tend to support the right to access comprehensive healthcare, including abortion and gender-affirming treatments, and emphasize evidence-based medical practices over legal or criminal restrictions. Conservatives, on the other hand, might focus more on regulating or restricting access to abortion and might not prioritize or support gender-affirming care to the same extent. They often emphasize the potential moral and ethical implications of these healthcare services rather than solely focusing on the evidence-based medical standards.	liberal
AAFP	Criminalization of the Provision of Medical Care to Undocumented Individuals	7/2023	The text provided aligns more closely with a liberal viewpoint. Liberal perspectives often emphasize healthcare access as a fundamental right for all individuals, regardless of their legal status or nationality. The emphasis on the physician-patient relationship and opposition to criminalizing medical care for undocumented individuals reflects a commitment to universal healthcare principles and the protection of human rights. Conservative viewpoints, in contrast, might prioritize legal status and national borders more strongly and may support more restrictive policies regarding the provision of services to undocumented immigrants. The stance taken by the American Academy of Family Physicians (AAFP) in this text is consistent with broader liberal values that prioritize inclusivity and access to healthcare for all individuals.	liberal
AAFP	Cultural Sensitivity: The Importance of Cultural Sensitivity in Providing Effective Care for Diverse Populations (Position Paper)	6/1905	The text from the position paper of the American Academy of Family Physicians (AAFP) primarily aligns with viewpoints that are often associated with liberal ideologies, particularly in its emphasis on health equity, cultural sensitivity, and the importance of addressing disparities in health outcomes for diverse populations. Several key aspects of the paper point towards this alignment:<br><br>1. **Health Equity and Social Justice:** The document's focus on eliminating health disparities and achieving optimal health for everyone regardless of cultural, racial, ethnic, or linguistic differences reflects a commitment to social justice and equity, principles often emphasized in liberal ideologies.<br><br>2. **Cultural and Linguistic Appropriateness:** The endorsement of culturally and linguistically appropriate services and the integration of cultural sensitivity into healthcare delivery are aligned with a liberal viewpoint that values diversity, inclusivity, and the recognition of systemic inequalities in healthcare.<br><br>3. **Support for Federal and Public Initiatives:** The AAFP's support for legislative, regulatory, and accreditation mandates, along with its endorsement of public and private payer initiatives to facilitate access to language and cultural services, suggests a belief in the role of government and policy in improving healthcare outcomes. This is consistent with liberal perspectives that advocate for government intervention to address social inequities.<br><br>4. **Comprehensive View of Culture:** The broad definition of culture used by the AAFP and the acknowledgment of a wide range of cultural characteristics indicate an inclusive approach to healthcare that seeks to understand and address the needs of a diverse population, a stance more commonly associated with liberal viewpoints.<br><br>5. **Advocacy and Policy Making:** The AAFP's involvement in advocacy, policy-making, and governance to integrate cultural sensitivity and promote health equity aligns with liberal ideologies' focus on policy reform as a mechanism to drive social change and address disparities.<br><br>While the document does not explicitly align itself with any political ideology, the emphasis on equity, diversity, inclusion, and the role of policy and governance in improving healthcare outcomes for all individuals are themes that are more typically associated with liberal rather than conservative viewpoints. Conservative viewpoints might focus more on individual responsibility, market-driven solutions, and less government intervention in healthcare, which are not the primary focuses of this position paper.	liberal

AAFP	Culturally Proficient, Health Care	10/2023	The text reflects a viewpoint that emphasizes the importance of cultural competency and sensitivity within the healthcare system, particularly among family physicians. This perspective aligns more closely with liberal viewpoints, which typically prioritize diversity, equity, and inclusivity. Liberal ideologies often emphasize the need for systems and policies that acknowledge and address systemic inequalities, including those found in healthcare. The focus on understanding and respecting cultural and ethnic backgrounds, addressing language barriers, and ensuring equitable access to healthcare services reflects a commitment to reducing disparities and improving healthcare outcomes for all patients, regardless of their cultural or ethnic background.<br><br>Conservative viewpoints, while also valuing the importance of patient care and the physician-patient relationship, may place a stronger emphasis on individual responsibility and less on systemic changes to address cultural and linguistic differences. Conservatives might support the idea of cultural competency but could be more cautious about the extent of regulatory mandates or the implementation of standardized programs across all healthcare settings, favoring more localized or individual-led initiatives.<br><br>The text's advocacy for education on cultural and ethnic differences in medical training, adherence to federal mandates for interpretive services, and the use of national standards for culturally and linguistically appropriate services are indicative of a policy approach that seeks to systematically address and integrate cultural sensitivity into healthcare practices. This approach is more aligned with liberal principles that advocate for systemic changes to promote inclusivity and equity.	liberal
AAFP	Culturally Sensitive Interpretive Services - AAFP Legislative Stance	10/2023	The text from the American Academy of Family Physicians (AAFP) regarding support for legislation to make funding available for culturally sensitive interpretive services for those with limited English proficiency, or who are deaf, or otherwise language impaired, generally aligns more with a liberal viewpoint. Liberal ideologies often prioritize inclusivity, diversity, and equity in healthcare and other social services. They advocate for policies that support marginalized and disadvantaged groups, including non-English speakers and those with disabilities, to ensure they have equal access to services and opportunities.<br><br>This position reflects a commitment to reducing healthcare disparities and increasing accessibility for all individuals, regardless of language proficiency or sensory impairments. It emphasizes the importance of culturally and linguistically appropriate services as part of a broader approach to social justice and equity, which are core values typically associated with liberal perspectives.<br><br>Conservative viewpoints, while also potentially supportive of improving healthcare access, might focus more on individual responsibility and less on government-funded interventions. They might prioritize different methods for addressing healthcare access and equity issues, possibly favoring private sector solutions over government-funded programs.<br><br>It's important to note, however, that support for such initiatives can cross political lines, and there may be conservatives who support this type of legislation as part of broader healthcare reforms aimed at improving efficiency and outcomes. The distinction often comes down to the approach and the extent of government involvement rather than the ultimate goal of improving healthcare access and quality.	liberal
AAFP	Data Stewardship	7/1905	The text you've provided outlines guidelines for the stewardship of health data, focusing on the ethical collection, storage, transmission, analysis, and reporting of digital health information. It emphasizes protecting the privacy and confidentiality of patients and physicians, ensuring data quality, and managing consent for data use. This approach to health data management does not squarely align with traditional liberal or conservative viewpoints as these guidelines are primarily concerned with practical, ethical, and legal considerations regarding personal health information.<br><br>However, aspects of this text can be seen as reflecting broader values that might be emphasized differently by liberal or conservative perspectives:<br><br>- **Privacy and Consent**: The strong emphasis on privacy, consent, and the right of physician practices to control data submission aligns with values that both political viewpoints might support, albeit for different reasons. Liberals might focus on the protection of individual rights and autonomy, especially regarding personal information. Conservatives might also value privacy but focus on the freedom of practices to operate without undue external interference.<br>- **Regulatory Compliance**: The mention of compliance with HIPAA and similar laws reflects a recognition of the role of government in setting standards for privacy and data protection, a principle that can be supported across the political spectrum. Liberals might more strongly emphasize the role of government regulation in protecting individuals, while conservatives might focus on the need for such regulations to be balanced with freedoms and not overly burdensome to businesses.<br>- **Data Quality and Safety**: The focus on data quality and safety is a non-partisan issue, as both sides generally agree on the importance of accurate and reliable health information for patient care.	neutral

			Overall, the text promotes principles of good data governance and stewardship in healthcare, which are essential to both protecting individuals' rights and ensuring the effectiveness of the healthcare system. These principles can be supported by both liberal and conservative viewpoints, though the reasons for their support may differ based on underlying values regarding the role of government, individual rights, and the operation of the healthcare system.	
AAFP	Direct Contracting with Businesses by Family Physicians (Discussion Paper)	7/1905	The text describes a healthcare payment model known as "direct contracting," which involves employers contracting directly with physicians or physician groups to provide primary care services to their employees and families. The American Academy of Family Physicians (AAFP) supports this model as it allows for physician and patient choice in healthcare delivery and promotes comprehensive and preventive care services while aiming to reduce healthcare costs and administrative burdens.<br><br>Labeling this text as aligning with either a liberal or conservative viewpoint oversimplifies the complex landscape of healthcare policy. Both liberal and conservative perspectives can find elements in the direct contracting model to support or critique based on their broader healthcare goals and principles:<br><br>- **Liberal viewpoint:** Liberals generally advocate for accessible, affordable healthcare for all and might support aspects of direct contracting that increase access to comprehensive primary care services and emphasize preventive care. However, they might be concerned about the potential for such models to exclude vulnerable populations or to create a two-tiered healthcare system where only certain employees have access to these services.<br><br>- **Conservative viewpoint:** Conservatives often emphasize market-driven solutions to healthcare and personal choice, making them likely to support the direct contracting model for its potential to reduce costs, decrease administrative burdens, and increase efficiency through competition. They might also appreciate the model's emphasis on direct payment and reduced reliance on traditional insurance mechanisms, viewing it as a step towards more privatized healthcare provision.<br><br>The direct contracting model, as described, incorporates elements that could be seen as beneficial from both perspectives: promoting patient and provider choice, aiming to reduce healthcare costs, and focusing on preventive and comprehensive care. However, the success of such models in addressing broader healthcare challenges, such as coverage for all population segments and managing overall healthcare spending, would likely influence how it is viewed from each ideological perspective.<br><br>In summary, the text outlines a healthcare delivery model that transcends simple ideological categorization by incorporating elements that could be endorsed by both liberal and conservative viewpoints, depending on the specific aspects emphasized and the outcomes achieved.	neutral

AAFP	Direct Primary Care	5/2023	The text describing the Direct Primary Care (DPC) model does not neatly align with traditional liberal or conservative viewpoints as understood in the context of U.S. politics. Instead, it touches on aspects that could be appealing to both ideological spectrums for different reasons:<br><br>1. **Appeal to Conservative Viewpoints:**<br>- **Market-Driven Health Care:** Conservatives generally favor market-driven solutions over government intervention in health care. The DPC model's emphasis on direct payment to physicians, bypassing traditional insurance and reducing regulatory burdens, aligns with the conservative preference for free market principles within healthcare.<br>- **Patient Choice and Physician Autonomy:** The model promotes patient choice and physician autonomy, principles often advocated by conservatives. It allows patients and doctors to enter into agreements without the involvement of third parties, potentially increasing efficiency and satisfaction.<br><br>2. **Appeal to Liberal Viewpoints:**<br>- **Access to Healthcare:** Liberals typically prioritize access to healthcare for all. The DPC model, by potentially lowering costs and improving the physician-patient relationship, could be seen as a way to enhance access to primary care services for those who can afford the model's fees.<br>- **Preventive and Comprehensive Care:** The emphasis on preventive care, wellness, and chronic care management aligns with liberal views on healthcare, where the focus is on comprehensive care that can prevent more costly interventions later.<br><br>However, the DPC model also presents areas of concern for both sides:<br>- **Conservatives** might worry about the sustainability of such models without broader market reforms and how it fits into the larger health insurance ecosystem.<br>- **Liberals** might be concerned about the model's affordability and accessibility for lower-income individuals, as well as how it integrates with efforts to achieve universal healthcare coverage.<br><br>The DPC model, by focusing on the patient-physician relationship, reducing bureaucracy, and potentially lowering costs, transcends simple political categorization. Its support from the American Academy of Family Physicians (AAFP) suggests a focus on improving healthcare delivery and outcomes rather than aligning with a specific political ideology. In discussing healthcare models like DPC, the emphasis tends to be more on practical outcomes such as access to care, cost, and quality of service rather than on fitting neatly into a liberal or conservative agenda.	neutral
AAFP	Direct to Consumer Genetic Testing	2019	The text from the American Academy of Family Physicians (AAFP) regarding direct to consumer (DTC) genetic testing does not neatly align with a traditional liberal or conservative viewpoint in the political sense. Instead, it reflects a professional and ethical stance on the need for regulation, oversight, informed consent, and privacy protections in the context of genetic testing. These concerns cross the spectrum of political ideologies, focusing on patient safety, the quality of medical advice, and the protection of personal data.<br><br>Here's how the text aligns with broader policy perspectives that might be associated with liberal or conservative viewpoints:<br><br>- **Consumer Protection and Regulation:** The call for the Food and Drug Administration (FDA) to define standards for informed consent and counseling, as well as to assess and oversee the claims made by companies, could be seen as aligning with a more regulatory or liberal perspective that emphasizes the role of government in ensuring consumer protection and the integrity of medical practices.<br><br>- **Privacy Protections:** The emphasis on expanding privacy and nondiscrimination protections concerning genetic information is a concern that transcends traditional political divides but could be more closely associated with liberal perspectives that prioritize individual rights and protections from discrimination.<br><br>- **Professional Oversight:** The insistence on involving healthcare professionals in the process of genetic testing and counseling is a position that emphasizes the importance of expert guidance and quality care, a concern that is broadly shared across the political spectrum.<br><br>- **Ethical Standards for Minors:** The call for more stringent standards for obtaining consent from minors reflects a concern for vulnerable populations, a focus that is generally emphasized across both liberal and conservative perspectives, though the approaches to addressing it may vary.	neutral

			In summary, the AAFP’s position is primarily focused on ethical, professional, and patient safety concerns rather than on political ideology. It advocates for responsible genetic testing practices that involve healthcare professionals, informed consent, privacy protections, and regulatory oversight to ensure accuracy and prevent discrimination. This approach aims to balance the benefits of direct to consumer genetic testing with the need to protect consumers and patients from potential risks and ethical issues.	
AAFP	Direct-to-Consumer Advertising of Infant Formula	7/1905	The text you've provided relates to the American Academy of Family Physicians' (AAFP) stance on breastfeeding and the promotion of infant formula. This viewpoint emphasizes the importance of breastfeeding as the primary and optimal method of infant nutrition, whenever it is possible and safe, and suggests restrictions on advertising or promoting infant formula to the public. It also advises hospitals and clinicians to avoid distributing formula samples and related advertising or literature.<br><br>This stance is not inherently aligned with a specific liberal or conservative political viewpoint. Instead, it reflects a public health perspective based on medical and nutritional evidence regarding the benefits of breastfeeding for both infants and mothers. Public health policies and recommendations like these are typically grounded in scientific research and are aimed at promoting the overall health and well-being of the population.<br><br>Policies or positions that focus on public health issues such as infant nutrition can be supported or criticized by individuals across the political spectrum, depending on how these policies intersect with broader political beliefs about government intervention, personal freedom, and public welfare. For instance:<br><br>- **Liberal Viewpoint**: Those with liberal viewpoints might support such policies as part of broader efforts to promote public health and well-being, emphasizing the role of government and healthcare providers in supporting healthy choices through education and regulation.<br><br>- **Conservative Viewpoint**: Those with conservative viewpoints might support the emphasis on family and parental decision-making in infant nutrition but could have concerns about government or institutional restrictions on advertising, advocating for more personal choice and less regulatory intervention.<br><br>In summary, while the AAFP's stance is grounded in a health and scientific perspective rather than a political one, the support for or opposition to such policies can vary based on how individuals or groups view the role of government and institutions in personal and public health decisions.	neutral

AAFP	Direct-to-Consumer Advertising of Prescription Pharmaceuticals, Nonprescription Medications, Health Care Devices, and Health-Related Products and Services	1/2022	The text provided outlines the American Academy of Family Physicians' (AAFP) stance on direct-to-consumer (DTC) advertising of pharmaceuticals and health-related products. It doesn't neatly align with traditional conservative or liberal viewpoints in a political sense but rather addresses public health policy and ethical considerations in advertising practices. Here's a breakdown in relation to potential viewpoints:<br><br>- **Liberal Viewpoints**: The text could align with liberal perspectives insofar as it advocates for regulation and oversight (e.g., adherence to FDA and FTC guidelines), transparency (e.g., accurate and complete information), consumer protection (e.g., mentioning negative or adverse reactions), and ethical considerations (e.g., preventing conflict of interest in referrals). Liberals often support the idea of regulatory frameworks that protect consumers and ensure that companies are transparent and accountable.<br><br>- **Conservative Viewpoints**: Parts of the text could resonate with conservative principles, especially the emphasis on information accuracy, balanced presentation, and the idea that advertisements should not promote unhealthy or unsafe practices. Conservatives often advocate for personal responsibility and might support the idea that consumers should have access to information that allows them to make informed decisions. However, conservatives might have mixed views on the degree of regulation suggested, as they generally favor less government intervention in business.<br><br>The AAFP's stance seems to prioritize ethical advertising practices that inform and protect consumers without explicitly endorsing or opposing the practice of DTC advertising itself. It focuses on ensuring that such advertising is done responsibly, with a strong emphasis on patient education and safety. This approach is more about public health ethics and professional standards rather than fitting neatly into a political liberal or conservative framework.	neutral
AAFP	Disaster Planning	10/2023	The text you provided doesn't strictly align with either a liberal or conservative viewpoint in a traditional, political sense. Rather, it emphasizes the importance of disaster preparedness, community protection, and healthcare readiness, which are issues of broad concern across the political spectrum. However, aspects of the text could be interpreted as aligning more closely with certain values or priorities that are sometimes associated with one political viewpoint or another, depending on the context:<br><br>1. **Emphasis on Community and Public Health:** The focus on planning and preparedness for disasters, protecting the community from environmental dangers, and the importance of healthcare settings in disaster response could be seen as aligning with liberal values that prioritize collective action, public health, and government involvement in ensuring the welfare of the community.<br><br>2. **Attention to Marginalized Communities:** The explicit mention of understanding how marginalized communities are disproportionately affected by disasters due to socioeconomic status, racial disparities, disability, housing stability, and access to healthcare reflects a concern for social justice and equity. This focus is often associated with liberal viewpoints that emphasize the need to address systemic inequalities and ensure that all community members, especially the most vulnerable, have the support and resources they need.<br><br>3. **Partnership with Public Agencies:** The call for family physicians to collaborate with local and national public health organizations to create detailed action plans could be seen as advocating for a cooperative approach between the government and healthcare professionals. This approach is more typically aligned with liberal perspectives that support a role for government in managing public health issues and disaster response.<br><br>4. **Preemptive Planning and Education:** The emphasis on preemptive planning, education, and empowerment for disaster preparedness is a principle that transcends political boundaries, appealing to the broader notion of responsibility and foresight in healthcare and community safety.<br><br>In general, the text promotes values of preparedness, community care, and health equity, which can resonate with a wide audience regardless of political affiliation. The emphasis on collaboration, equity, and proactive planning in healthcare reflects a comprehensive approach to disaster management that prioritizes the well-being and safety of all community members, especially those who are most vulnerable.	probably liberal

AAFP	Discipline in Schools	10/2023	The text presents a viewpoint that aligns more closely with liberal ideologies than conservative ones. This alignment is evident in several key aspects:<br><br>1. **Opposition to Zero-Tolerance Policies:** The text criticizes zero-tolerance policies in schools, which are often associated with a more conservative, punitive approach to discipline. The criticism is based on the argument that such policies are inflexible and fail to consider the nuances of individual situations.<br><br>2. **Emphasis on Social Determinants of Health:** The text frames education as a social determinant of health and highlights the disproportionate impact of punitive disciplinary policies on marginalized populations, including gender and racial disparities. This perspective is consistent with liberal viewpoints that stress the importance of addressing systemic inequalities and the social factors that influence health outcomes.<br><br>3. **Advocacy for Alternatives to Punishment:** The call for investigating and implementing alternative, evidence-based disciplinary methods suggests a preference for rehabilitative over punitive measures. This approach aligns with liberal ideologies that focus on rehabilitation and the underlying causes of behavior rather than solely on punishment.<br><br>4. **Concern Over the School-to-Prison Pipeline:** The text directly mentions the school-to-prison pipeline, a concept often discussed in liberal circles, which refers to the policies and practices that push students, particularly those from marginalized groups, out of schools and into the criminal justice system. Highlighting this issue reflects a concern for systemic injustice and the long-term consequences of punitive disciplinary policies.<br><br>While the text does not explicitly identify itself with a political ideology, its focus on equity, systemic reform, and the social context of education and health disparities are themes commonly associated with liberal viewpoints.	liberal
AAFP	Disclosure of Corporate Ties Affecting Formulary Choices and Drug Substitution	7/1905	The text from the American Academy of Family Physicians (AAFP) outlines a position advocating for transparency in the relationships between healthcare providers, pharmaceutical companies, and pharmacy-related entities. It also emphasizes the importance of making medication-related decisions based on evidence-based therapeutic and quality of care considerations rather than economic factors. This stance aligns with principles that could be supported by both liberal and conservative viewpoints for different reasons:<br><br>- **Liberal Viewpoint**: The call for full disclosure of corporate ties and financial relationships aligns with liberal principles of transparency, consumer protection, and the prioritization of patient care over profit. Liberals often advocate for policies that ensure healthcare decisions are made in the best interest of patients and support measures that reduce the influence of corporations in healthcare. The emphasis on evidence-based therapeutic and quality of care considerations over economic considerations also reflects a liberal inclination towards science-based policies and patient-centric healthcare.<br><br>- **Conservative Viewpoint**: While conservatives typically support free market principles and might favor less regulation in business, many also advocate for transparency and ethical practices in all sectors, including healthcare. The emphasis on not making formulary decisions and drug switching primarily for economic reasons could resonate with conservative values of individual choice and the importance of quality care. Additionally, conservatives may see the push for transparency as a way to foster competition and efficiency in the market, as it empowers consumers (in this case, patients and physicians) with information to make informed decisions.<br><br>The text embodies a cross-partisan approach that focuses on ethical standards, patient care quality, and evidence-based decision-making. It doesn't neatly align with a purely liberal or conservative viewpoint but rather addresses concerns relevant to a broad spectrum of political beliefs. The focus on transparency and evidence-based care transcends traditional partisan divides, highlighting shared values in the pursuit of optimal healthcare outcomes.	neutral

AAFP	Discrimination, Family Medicine Residency Graduates	7/1905	The text you've shared focuses on a policy regarding the credentialing and payment of family medicine residency graduates who have not yet taken their board exams. Specifically, it suggests that health care organizations should not discriminate against these individuals within one year of graduation. This policy is more about fairness and equity in the professional credentialing process rather than a clear-cut issue that aligns neatly with either liberal or conservative political viewpoints.<br><br>However, analyzing the underlying principles can give us some insights:<br><br>- **Liberal Viewpoint Alignment:** From a liberal perspective, this policy could be seen as promoting equal opportunities and reducing barriers for new graduates entering the workforce. Liberals often advocate for policies that ensure fairness, equal treatment, and support for individuals as they enter or re-enter the workforce. By advocating for a policy that prevents discrimination against new graduates based on their certification status, it aligns with the liberal emphasis on inclusivity and equality.<br><br>- **Conservative Viewpoint Considerations:** Conservative viewpoints typically emphasize meritocracy, personal responsibility, and the importance of standards and qualifications. Some conservatives might argue that board certification is an important standard that ensures quality and competence in healthcare. However, this policy does not eliminate the need for board certification; it merely provides a grace period for new graduates, which could be seen as a reasonable accommodation that does not compromise on quality or standards.<br><br>In summary, the text seems to straddle a pragmatic approach that could garner support from both sides for different reasons. It addresses fairness and opportunity (more aligned with liberal values) while also potentially acknowledging the importance of standards in the medical profession (a concern for conservatives), albeit with a temporary grace period. The emphasis is on practicality and fairness in professional advancement rather than on ideological grounds.	neutral
AAFP	Discrimination, Patient	2020	The text you've provided outlines a stance against discrimination in all its forms, emphasizing inclusivity and equality across various personal and social dimensions such as race, gender, sexual orientation, and economic status. This viewpoint aligns more closely with liberal ideologies, which often prioritize social justice, equality, and the protection of individual rights across diverse communities. Liberal perspectives typically advocate for policies and positions that aim to address inequalities and support marginalized groups, seeking to ensure equal opportunities and treatment for all individuals regardless of their background or identity.<br><br>Conservative viewpoints, while also valuing equality and individual rights, might emphasize different approaches to achieving these goals, often focusing on tradition, personal responsibility, and limited government intervention in social issues. Thus, the explicit and broad opposition to discrimination stated in the text is more reflective of values commonly associated with liberalism, especially in its emphasis on a wide range of identities and conditions as grounds for anti-discrimination efforts.	liberal
AAFP	Discrimination, Physician	2020	The text from the American Academy of Family Physicians (AAFP) supports the idea that employment, credentialing, and privileging decisions for physicians should be based on professional qualifications and advocates for diversity in the physician workforce to improve culturally competent care. This stance aligns more closely with principles often associated with a liberal viewpoint for several reasons:<br><br>1. **Emphasis on Diversity**: The support for diversity in the physician workforce and the belief that this diversity can enhance the delivery of culturally competent care align with liberal values, which often prioritize inclusivity, equality, and the recognition of diverse backgrounds as a strength in various fields, including healthcare.<br><br>2. **Equity in Professional Opportunities**: The insistence that decisions related to hiring, credentialing, and privileging be based solely on verifiable professional criteria suggests an opposition to discrimination or bias based on non-professional factors. This aligns with liberal principles that advocate for equality and fair treatment for all individuals regardless of their background.<br><br>3. **Healthcare as a Vehicle for Social Justice**: The underlying suggestion that healthcare systems and physician groups can address disparities in care through diversity reflects a liberal perspective on using systemic changes to achieve broader social goals, such as justice and equity in healthcare access and quality.<br><br>While these principles might be supported by individuals across the political spectrum to varying degrees, the explicit focus on diversity and equity tends to be more closely associated with liberal viewpoints. Conservative viewpoints might also support merit-	liberal

			based professional decisions but may place less emphasis on the active promotion of diversity as a criterion for enhancing healthcare delivery.	
AAFP	Discriminatory Policing	9/2022	The text provided reflects viewpoints that align more closely with liberal ideologies, particularly those concerned with social justice, equality, and police reform. Here's a breakdown of why this text leans towards a liberal viewpoint:<br><br>1. **Emphasis on Accountability and Transparency in Policing**: The call for consistent communication, transparency, and accountability in police interactions with the public is a stance often associated with liberal perspectives. These principles are advocated to ensure that policing practices are fair and do not infringe upon the rights of individuals.<br><br>2. **Opposition to Discriminatory Policing and Excessive Force**: The text's focus on the hazards of discriminatory policing and the use of excessive force, especially against people of color and other minority groups, aligns with liberal concerns about systemic racism and social inequality within the criminal justice system.<br><br>3. **Support for Recommendations from President Obama's Task Force**: The endorsement of the recommendations from President Obama's 2015 Final Report of the President's Task Force on 21st Century Policing further aligns the text with liberal viewpoints. These recommendations aim to reform policing practices to make them more community-oriented, fair, and less discriminatory.<br><br>4. **Advocacy for Policies Prohibiting Profiling and Discrimination**: The specific support for policies that prohibit profiling and discrimination based on a wide range of characteristics (race, ethnicity, national origin, etc.) reflects a liberal commitment to equality and protection of civil rights for all individuals, regardless of their background or identity.<br><br>5. **Support for De-escalation Techniques and Minimal Use of Force**: The call for the adoption of evidence-based de-escalation techniques and the minimal use of force is consistent with liberal approaches to policing. These approaches prioritize non-violent methods and the preservation of life and safety over aggressive or militarized responses.<br><br>While the text presents a balanced view by initially recognizing the effectiveness of policing in reducing crime and promoting safety, its focus on reforming policing practices to address issues of discrimination, excessive force, and the importance of equality and civil rights strongly aligns with liberal ideologies. Conservative viewpoints might also share concerns over accountability and effective policing but may differ in emphasis, particularly regarding the extent of reforms and the methods proposed to address issues within the policing system.	liberal

AAFP	Diversity in the Workforce	9/2022	The text aligns more closely with a liberal viewpoint. It emphasizes the importance of diversity, equity, and inclusion (DEI) in the healthcare workforce as strategies to address health disparities, systemic racism, and inequalities in healthcare access and quality. Liberal perspectives typically advocate for social justice, equity, systemic reform, and the acknowledgment of systemic racism as factors affecting health outcomes. The focus on expanding opportunities for underrepresented groups in medicine, addressing implicit bias, and advocating for systemic changes inside and outside the healthcare setting are hallmarks of liberal approaches to social and health policy.<br><br>Conservative viewpoints might also recognize the importance of diversity in healthcare but may emphasize different strategies for achieving it, such as merit-based systems or less focus on systemic racism as a primary cause of health disparities. Conservatives might also be more cautious about the extent of government or institutional intervention in promoting diversity and equity, preferring market-based or individual choice-driven solutions.<br><br>The actions supported by the AAFP (American Academy of Family Physicians) outlined in the text, such as leading efforts to develop a diverse workforce, encouraging DEI initiatives, diversifying medical education, and advocating for justice-oriented changes, align with liberal ideals of actively addressing and rectifying inequalities through systemic reforms and targeted actions.	liberal
AAFP	Domestic Partner Benefits	10/2023	The statement aligns more closely with a liberal viewpoint. The American Academy of Family Physicians (AAFP) advocating for the legal recognition of domestic partnership benefits to address healthcare inequities reflects principles often associated with liberal ideologies, particularly in regard to supporting LGBTQ+ rights, promoting equality, and addressing disparities in access to healthcare. Liberal viewpoints typically emphasize social equity, support for diverse family structures, and the expansion of civil rights to include marginalized communities. This position focuses on inclusivity and equal treatment for married and unmarried families, including those within the LGBTQ+ community, which are key issues often championed by liberal perspectives.<br><br>Conservative viewpoints, on the other hand, might prioritize traditional family structures and may have differing views on government involvement in recognizing domestic partnerships and extending benefits. However, it's important to note that perspectives on these issues can vary widely within both liberal and conservative circles, and individuals or groups may hold nuanced positions that do not fully align with the generalizations of either ideology.	liberal
AAFP	Driver Education	10/2023	The text you've provided outlines the American Academy of Family Physicians (AAFP)'s recommendations regarding driver education. These recommendations focus on safety and education, advocating for driver education to be required for all new drivers and available for drivers who experience events that might impact their driving abilities. The emphasis is on evidence-based and age-appropriate curricula that include rules of the road, handling emergency situations, and maintaining vehicle safety.<br><br>This stance does not align strictly with a liberal or conservative viewpoint as traditionally defined in political terms. Instead, it represents a public health and safety perspective. The focus on education, evidence-based practices, and preventive measures to enhance driver safety transcends political ideologies, aiming to reduce accidents and improve outcomes for all drivers.<br><br>Issues of public health and safety, such as driver education, can sometimes be politicized, but the recommendations themselves are based on concerns for safety and efficacy rather than political ideology. Therefore, while different political groups might have varying opinions on the implementation of such education programs, including their scope, funding, and requirements, the recommendations as stated are neutral and aimed at improving public safety.	neutral

AAFP	Drug Enforcement Administration (DEA) - AAFP Legislative Stance	7/1905	The text outlines a position that doesn't neatly align with traditional liberal or conservative viewpoints as understood in a broad political context. Its focus is on practical reforms and improvements in the regulation and administration of controlled substances within the healthcare system. Here's a breakdown of how the text might be perceived from both viewpoints:<br><br>- **Liberal Viewpoint Alignment:**<br>- **Support for Reforms and Collaboration:** The call for reforms to improve the DEA's authority and the emphasis on partnerships with various stakeholders might align with a more liberal viewpoint that often values comprehensive, community-based approaches to solving systemic issues. The focus on improving public health and simplifying administration could also resonate with liberal priorities on healthcare access and efficiency.<br>- **Opposition to Third-Party Access to DEA Numbers:** Liberals might view the opposition to third-party access to DEA registration numbers as a stance that protects patient privacy and rights, aspects highly valued in liberal ideologies.<br><br>- **Conservative Viewpoint Alignment:**<br>- **Support for Law Enforcement and National Security:** The mention of enhancing the DEA's role in law enforcement and national security could align with conservative values that prioritize strong measures in these areas. Conservatives might also appreciate the focus on anti-fraud activities and the careful regulation of controlled substances, reflecting concerns about drug abuse and illegal distribution.<br>- **Concerns Over Regulatory Overreach:** The opposition to the use of DEA numbers by third parties, particularly for insurance claims, might resonate with conservative concerns over regulatory overreach and the imposition of bureaucratic requirements on healthcare providers.<br><br>However, the statement is primarily focused on technical and administrative aspects of drug enforcement and healthcare regulation. It seeks to balance the needs of law enforcement and public health without taking a clear stance on broader ideological issues. The position is more about pragmatic healthcare policy than about aligning with a specific political ideology. This reflects how professional medical associations like the AAFP often prioritize the interests of patients and healthcare providers in their policy positions, focusing on practical outcomes rather than political ideology.	neutral
AAFP	Drug Pricing - AAFP Legislative Stance	7/1905	The text from the American Academy of Family Physicians (AAFP) concerning support for legislation to ensure the availability of effective, safe, and affordable medications through policies that include promoting competition and availability, transparency, patient-centric pricing, drug price negotiation, and review, reflects principles that are generally associated more with a liberal viewpoint. Here's why:<br><br>1. **Promotion of Governmental Authority**: The emphasis on utilizing governmental authority to regulate and promote competition, transparency, and affordability in the pharmaceutical market aligns with a liberal perspective, which often advocates for a more active government role in healthcare to ensure fairness and accessibility for all citizens.<br><br>2. **Transparency and Patient-Centric Pricing**: Advocating for transparency in drug pricing and policies that focus on patient-centric pricing models align with liberal views on healthcare, emphasizing the importance of making healthcare accessible and affordable for patients rather than prioritizing profit margins for pharmaceutical companies.<br><br>3. **Drug Price Negotiation and Review**: The support for drug price negotiation and review is a stance that typically aligns with liberal policies, favoring regulatory mechanisms to control drug prices. This approach is based on the belief that healthcare should be a right rather than a privilege, and that excessive drug prices can limit access to essential medications for many people.<br><br>It's important to note that while these policies might align more closely with liberal viewpoints, healthcare affordability and access are issues that can transcend traditional political boundaries. Both liberal and conservative individuals and groups may support efforts to improve the healthcare system, though their approaches and specific policy solutions might differ. The AAFP's position, as stated, focuses on broad principles of affordability, transparency, and government intervention to ensure medication availability, which are typically more aligned with liberal healthcare policy priorities.	liberal

AAFP	Drug Switching Notices	2019	The text provided outlines the American Academy of Family Physicians (AAFP)'s stance on patient-centered formularies and the conditions under which drug switching notices might be considered appropriate. This position does not neatly align with traditional liberal or conservative viewpoints in a political sense but rather focuses on healthcare practices that prioritize patient welfare, evidence-based management, privacy compliance, transparency, and professional consultation.<br><br>The emphasis on patient-centric care, evidence-based guidelines, and transparency could be appealing to those with a liberal viewpoint, as these elements often align with a preference for healthcare policies that prioritize individual patient needs and rights, as well as the use of scientific evidence in decision-making. The support for generic or therapeutically equivalent drugs as a cost-saving measure could also resonate with liberal viewpoints that favor accessible and affordable healthcare.<br><br>Conversely, the text's focus on reducing healthcare costs through the use of generics and ensuring that changes are HIPAA-compliant and transparent about financial impacts could find some support from conservative viewpoints, especially those that value efficient healthcare spending, patient privacy, and the reduction of unnecessary healthcare costs.<br><br>Ultimately, the position described is best seen as a healthcare policy stance that transcends simple political categorization. It reflects a comprehensive approach to patient care that incorporates elements of cost management, patient autonomy, and evidence-based practice—concerns that cut across the political spectrum.	neutral
AAFP	Drugs - Identification	9/2019	The statement regarding the American Academy of Family Physicians (AAFP) supporting the use of a uniform identification code for individual drug capsules and tablets, as well as comprehensive labeling of drug containers, does not clearly align with traditional political viewpoints such as liberal or conservative. Instead, it reflects a position on public health policy and patient safety.<br><br>Both liberal and conservative perspectives can support the idea of improving drug safety and transparency, although the reasons and emphasis may differ. Liberals might focus on consumer rights and protections, ensuring that patients have access to necessary information for making informed healthcare decisions. Conservatives might support this from the angle of improving market efficiency and reducing regulatory burdens that complicate the identification and use of medications, as long as it does not introduce excessive government intervention.<br><br>Therefore, this statement is more about healthcare policy and patient safety than it is about political ideology. It reflects a commitment to improving the clarity, safety, and efficiency of medication use, goals that can be supported across the political spectrum for different reasons.	neutral

AAFP	Drugs - Therapeutic Substitution	7/2023	The text reflects concerns and positions that might align with both liberal and conservative viewpoints, depending on how one interprets the underlying values and priorities. Here's a breakdown of how it might align with each perspective:<br><br>### Liberal Viewpoint Alignment<br>- **Healthcare Access and Quality:** Liberals often advocate for policies that ensure access to high-quality healthcare and patient rights. The opposition to therapeutic substitution without consulting physicians could align with a liberal viewpoint that prioritizes individualized patient care and the protection of patient-doctor decision-making authority.<br>- **Regulation to Protect Public Interest:** The call for maintaining strict anti-substitution laws and regulations can also resonate with liberal perspectives that support the role of government in regulating industries, including healthcare, to protect public interests and ensure safety and efficacy.<br><br>### Conservative Viewpoint Alignment<br>- **Opposition to Overregulation:** Conservatives might support the stance against therapeutic substitution if they view it as a means to prevent overregulation and interference in the doctor-patient relationship, emphasizing personal freedom and professional autonomy.<br>- **Cost Containment Concerns:** While the text criticizes policies like step therapy and drug formularies designed for cost containment, a nuanced conservative viewpoint might focus on the need for such policies to keep healthcare costs down but argue for implementing them in a way that doesn't compromise patient care.<br><br>### Cross-Ideological Concerns<br>- **Doctor-Patient Relationship:** Both liberals and conservatives can agree on the importance of preserving the integrity of the doctor-patient relationship, which is central to the text's argument against therapeutic substitution without consultation.<br>- **Quality of Care vs. Cost Containment:** The debate over maintaining quality care while managing healthcare costs is a concern across the political spectrum. The text's criticism of policies prioritizing cost over care can resonate with individuals regardless of their political alignment, though the solutions proposed might differ.<br><br>In summary, the text reflects concerns that intersect with values held by both liberal and conservative viewpoints, focusing on the quality of patient care, the autonomy of healthcare providers, and the complexities of healthcare regulation and cost containment. The alignment with a particular viewpoint can depend significantly on how one prioritizes these concerns and the proposed solutions.	neutral
AAFP	Drugs, Generic	9/2022	The text primarily focuses on healthcare policy, specifically on the use of generic medications, advocating for patient access to affordable medication, and addressing the administrative burdens that can impact the doctor-patient relationship. This stance does not neatly align with the traditional liberal-conservative spectrum as understood in a political context, since it deals more directly with healthcare practices and policies rather than broader political ideologies.<br><br>However, the emphasis on patient advocacy, reducing healthcare costs through the use of generic medications, opposing mandatory generic substitution to ensure physician and patient choice, and the call to eliminate administrative hurdles like prior authorizations could be interpreted through various political lenses:<br><br>- **Liberal Viewpoint Alignment**: The text could align with liberal viewpoints in its emphasis on making healthcare more affordable and accessible to all, reducing administrative burdens that could hinder access to care, and advocating for patient rights in the healthcare system. Liberals often prioritize healthcare reform that expands access and reduces costs for patients, and the positions outlined in the text support such objectives.<br><br>- **Conservative Viewpoint Considerations**: While the text does not directly advocate for conservative principles like reduced government involvement, it does emphasize the importance of choice (opposing mandatory generic substitution) and could be seen as supporting market-driven solutions (such as the development and use of generic drugs to lower costs). However, the opposition to mandatory policies might also resonate with conservative values of individual choice and skepticism of overregulation.<br><br>In essence, the text represents a healthcare policy perspective focused on patient care, affordability, and the reduction of administrative burdens. It crosses typical political divisions by advocating for positions that could be supported by individuals across the political spectrum, depending on their views on healthcare reform, regulation, and the role of government in managing healthcare costs and practices.	neutral

AAFP	Drugs, Opposition to Mandatory Education for Drug Prescribing	1/2022	The text reflects a nuanced stance on drug prescription and monitoring policies, aligning with principles that could be supported by both liberal and conservative viewpoints, depending on the context and underlying values being emphasized.<br><br>- Opposition to mandatory education for prescribing specific drugs, such as opioids, might resonate more with conservative views that prioritize minimizing government mandates and regulations on professional practices. This stance aligns with a preference for less government intervention in the operations of medical professionals and businesses.<br><br>- Support for programs providing funding to develop prescription drug monitoring programs (PDMPs) and facilitating interstate exchange of monitoring information can be seen as a proactive public health measure, which might align more closely with liberal viewpoints that emphasize the role of government in ensuring public safety and health through regulation and oversight.<br><br>Both viewpoints, however, share common ground in addressing the opioid crisis and ensuring the responsible prescription of drugs, showing that such policy positions can transcend simple political categorization. The focus on both limiting overregulation (a typically conservative concern) and enhancing public health measures through support for PDMPs (often a liberal priority) demonstrates a balanced approach that seeks to address the complex issue of opioid prescription and abuse without fully aligning with the ideology of one political spectrum over the other.	neutral
AAFP	Drugs, Physician Dispensing	7/1905	The text provided outlines a position from the American Academy of Family Physicians (AAFP) regarding the rights of physicians to diagnose, prescribe, and dispense medications and therapeutic products. It emphasizes the autonomy of medical professionals while also advocating for high standards of practice. This stance does not neatly align with traditional liberal or conservative viewpoints as it touches on aspects that both sides of the political spectrum might support or contest, depending on the context. Here's how:<br><br>1. **Liberal Viewpoint Considerations:**<br>- **Autonomy in Healthcare:** Liberals often support individual rights and autonomy, including in healthcare. The emphasis on the physician's right to diagnose, prescribe, and dispense medication without undue regulation could resonate with liberal ideals of personal freedom and professional autonomy.<br>- **Regulation for Safety and Equality:** However, liberals also tend to support regulations that ensure safety, quality, and equality. The call for high standards among dispensing physicians aligns with the liberal emphasis on regulatory measures to protect public health and ensure equitable access to high-quality care.<br><br>2. **Conservative Viewpoint Considerations:**<br>- **Limited Regulation:** Conservatives generally advocate for limited government intervention in professional and business practices. The statement that "no regulation or laws should infringe upon that right" aligns with conservative principles of minimizing regulatory burdens on professionals and businesses.<br>- **Professional Autonomy and Responsibility:** The emphasis on professional autonomy and the expectation that physicians be held to high standards reflects conservative values of personal responsibility and the belief in the professionalism of healthcare providers to make the best decisions without excessive government oversight.<br><br>In summary, the text from the AAFP reflects a nuanced position that champions both professional autonomy and accountability. It highlights a balance between the right of physicians to practice medicine without undue interference and the importance of maintaining high standards of care. This balance can appeal to aspects of both liberal and conservative ideologies, depending on the specific emphasis (autonomy vs. regulation for quality and safety). Thus, categorizing this statement as purely liberal or conservative might oversimplify its broader appeal and the complexity of healthcare policy debates.	neutral

AAFP	Drugs, Prescribing	7/1905	The text provided outlines the position of the American Academy of Family Physicians (AAFP) on several issues related to the prescription of pharmaceuticals and the roles of healthcare professionals in this process. The stance does not neatly align with traditional liberal or conservative viewpoints as understood in a political sense, but rather reflects professional and healthcare policy perspectives.<br><br>The opposition to actions that limit patients' access to pharmaceuticals prescribed by a physician and the stance against allowing non-physicians to alter prescriptions can be seen as prioritizing patient care and the authority of physicians in the healthcare process. These positions emphasize the importance of medical expertise and autonomy in clinical decision-making, which can appeal to values across the political spectrum.<br><br>However, some elements of the AAFP's position might resonate differently with liberal and conservative audiences:<br><br>1. **Liberal Viewpoint Alignment:** Liberals often advocate for accessible healthcare services and might support the AAFP's opposition to limitations on patient access to prescribed pharmaceuticals. The emphasis on ensuring broad access to medications and opposing unnecessary restrictions by insurers or other entities aligns with liberal values of healthcare equity and patient rights.<br><br>2. **Conservative Viewpoint Considerations:** Conservatives might appreciate the AAFP's emphasis on the role of professional judgment and expertise in healthcare, reflecting a preference for minimizing external interference in the physician-patient relationship. The stance that only licensed doctors should have the statutory authority to prescribe drugs could resonate with conservative views favoring established professional hierarchies and traditional roles within healthcare.<br><br>The text mainly addresses professional standards, healthcare access, and the roles of different healthcare providers in the prescription process. It advocates for policies that ensure physicians maintain control over prescribing practices, which is a stance focused on healthcare quality and professional autonomy rather than a direct reflection of political ideology. Thus, while aspects of the statement might find support or opposition within both liberal and conservative circles, its primary alignment is with professional healthcare policy rather than a clear-cut political ideology.	neutral
AAFP	Durable Medical Equipment, Unsolicited Vendor Requests	1/2022	The text outlines a policy stance from the American Academy of Family Physicians (AAFP) regarding unsolicited requests for durable medical equipment (DME) or supplies, which is not inherently aligned with a strictly liberal or conservative political viewpoint. Instead, it addresses a professional and ethical issue within healthcare practice, focusing on the physician-patient relationship, medical necessity, and the influence of direct-to-consumer marketing.<br><br>This policy emphasizes the importance of medical necessity over market-driven demands, suggesting that physicians should prioritize clinical judgment and patient education over responding to unsolicited vendor requests. It supports the idea that medical decisions should be grounded in evidence-based practice and the clinical needs of the patient, rather than external marketing pressures.<br><br>In the broader context of healthcare policy debates, liberals and conservatives may interpret or leverage such a stance differently, depending on their views on healthcare regulation, the role of market forces in healthcare, consumer rights, and the autonomy of medical professionals. For example:<br><br>- **Liberal viewpoint**: Individuals with a liberal viewpoint might support this policy as it protects patients from potentially unnecessary medical expenses and emphasizes the need for healthcare decisions to be based on medical necessity rather than commercial interests. They may see it as a step toward safeguarding patient welfare and promoting evidence-based medicine, aligning with broader goals of healthcare reform that prioritize patient care over profit.<br><br>- **Conservative viewpoint**: Individuals with a conservative viewpoint might appreciate the policy's implicit support for physician autonomy and discretion in making medical decisions. They could argue that it helps prevent regulatory overreach into the physician-patient relationship and keeps decision-making in the hands of medical professionals rather than government or commercial entities.<br><br>Overall, the policy itself is centered on clinical ethics and professional standards, which transcends simple political categorization. It reflects concerns common to many healthcare providers about the influence of external factors on clinical decision-making and the integrity of the patient-care provider relationship.	neutral

AAFP	Education, Physician Retraining	10/2020	The text you've provided outlines the qualifications and training standards for family physicians as endorsed by the American Academy of Family Physicians (AAFP). It specifies the criteria for someone to be recognized as a family physician, focusing on the educational and certification requirements necessary for this medical specialty. This text is primarily professional and educational in nature, rather than political. It does not explicitly align with either a liberal or conservative viewpoint as it discusses standards for medical education and certification in the field of family medicine, rather than political or ideological issues.<br><br>In general, discussions around education, certification standards, and professional qualifications within a specific field like medicine are not inherently political. They are typically focused on ensuring that practitioners have the necessary knowledge, skills, and competencies to provide high-quality care to patients. While healthcare policy itself can be a deeply political and partisan topic in the United States, with debates over healthcare access, insurance, and regulation often breaking down along liberal and conservative lines, the specific content of this text does not engage with those debates. It simply outlines what is required to be considered a qualified family physician according to a particular professional organization.	neutral
AAFP	EGD, Training and Credentialing of Family Physicians In (Position Paper)	9/2022	The text you've provided is a detailed and technical overview of Esophagogastroduodenoscopy (EGD) procedures, training, indications, and policy recommendations from a medical and procedural standpoint. It focuses on the scope of practice for family physicians, clinical indications for EGD, training methodology, proficiency testing and documentation, credentialing and privileges, and miscellaneous issues including public health implications and current research agendas.<br><br>This content is primarily medical and procedural, devoid of any explicit political or ideological viewpoints that would align it distinctly with liberal or conservative ideologies. It is centered on the medical practice, safety guidelines, and the expansion of family physicians' roles in providing comprehensive care, including EGD procedures. The emphasis is on improving patient access to care, especially in underserved areas, ensuring safety and efficacy in medical procedures, and outlining the process for obtaining necessary training and credentials.<br><br>Liberal versus conservative viewpoints typically relate to political, social, or economic issues rather than medical procedures and practices. While discussions about healthcare access, funding, and policy can have political dimensions (with liberals generally supporting broader government involvement in healthcare and conservatives often advocating for limited government and more private sector solutions), the text itself is neutral, focusing on medical best practices rather than political or ideological debates.	neutral
AAFP	Elder Mistreatment	7/1905	The text you've provided primarily focuses on the issue of elder mistreatment, outlining its forms, consequences, and the roles family physicians can play in prevention, detection, and intervention. It advocates for a comprehensive approach to elder care, emphasizing the importance of awareness, education, and legal adherence to protect older adults from abuse and neglect.<br><br>This content does not neatly align with traditional political divides such as liberal or conservative viewpoints in a direct manner. Instead, it addresses elder mistreatment as a public health and social justice issue. The focus on prevention, education, and multidisciplinary approaches to safeguarding the well-being of older adults is consistent with principles that transcend political ideologies. It emphasizes human rights, the welfare of vulnerable populations, and the responsibility of healthcare providers and the community in supporting these individuals.<br><br>However, one could argue that the emphasis on systemic solutions, community involvement, and the need for regulatory compliance may resonate more with viewpoints typically associated with liberal ideologies, which often stress the role of societal structures and policies in addressing social issues. Liberals tend to support robust social safety nets and regulatory frameworks to protect vulnerable populations.<br><br>Conversely, conservatives might focus on the importance of family, individual responsibility, and limited government intervention, though they would also likely agree on the importance of preventing elder mistreatment. The call for community and family involvement in preventing elder abuse could align with conservative values related to community cohesion and personal responsibility.<br><br>Ultimately, the text champions a cause that is of universal concern, aiming for the well-being of older adults, which is a value shared across the political spectrum. It advocates for a proactive, informed, and compassionate approach to healthcare that seeks to protect one of the most vulnerable segments of society from harm.	neutral

AAFP	Electronic Health Records	5/2023	The text provided outlines a position from the American Academy of Family Physicians (AAFP) regarding the adoption of health information technology, including electronic health records, to support medical homes, care coordination, continuity, patient-centeredness, safety, and quality of care. This position doesn't neatly align with traditional liberal or conservative political viewpoints as it focuses on healthcare quality improvement through technology, which is generally a bipartisan issue.<br><br>Liberal viewpoints often emphasize the importance of accessible, equitable healthcare and might support the integration of technology to improve healthcare delivery and patient outcomes. Conservatives may also support technological advancements in healthcare, especially if such advancements can lead to more efficient, cost-effective care, and reduced governmental intrusion in healthcare decisions.<br><br>The statement is more aligned with healthcare policy and innovation rather than a partisan viewpoint. It emphasizes the potential benefits of technology in improving healthcare outcomes, which can be a point of agreement across the political spectrum. The focus is on practical, technological solutions to healthcare challenges, rather than ideological or political arguments.	neutral
AAFP	Electronic Nicotine Delivery Systems (ENDS)	2019	The text you've provided outlines a position on Electronic Nicotine Delivery Systems (ENDS) that is focused on public health concerns, particularly regarding youth and the need for more research on the safety and efficacy of ENDS as a cessation device. This stance, especially the emphasis on regulation, research, and public health advocacy, aligns more closely with viewpoints typically associated with liberal ideologies. Liberal perspectives often prioritize public health measures, regulatory oversight for safety, and the protection of vulnerable populations, such as children and young adults, from potentially harmful products.<br><br>The call for banning marketing and advertising of ENDS to children and youth, advocating for research into the safety and efficacy of these products, and recommending cessation interventions aligns with a preventive approach to health care and a desire for regulatory frameworks that protect public health. These are hallmarks of a more liberal approach to public policy and health care.<br><br>However, it's important to note that public health is a broad field that can transcend political boundaries, and there may be conservatives who also support these measures out of concern for public health and safety. The classification of such positions as strictly liberal or conservative can be somewhat fluid, depending on the broader context of the discussion and the specific aspects of ENDS regulation and public health policy being debated.	liberal
AAFP	Electronic Prescribing	1/2022	The text you've provided outlines a policy position by the American Academy of Family Physicians (AAFP) regarding electronic prescribing and seems to focus on pragmatic solutions to healthcare delivery challenges rather than aligning clearly with ideological views typically associated with liberal or conservative viewpoints. The support for electronic prescribing to promote quality patient care is a stance that emphasizes technological advancement and efficiency in healthcare, a principle that can be appreciated across the political spectrum.<br><br>The acknowledgment of exceptions, such as public health emergencies or limited broadband access, reflects a nuanced understanding of real-world barriers to implementing technology-based solutions. This approach indicates a flexibility and consideration of varying circumstances that affect healthcare delivery, which is not inherently political but rather practical and patient-centered.<br><br>However, discussions around healthcare policy can sometimes reflect broader political ideologies in terms of how actively the government should be involved in providing solutions for public health and technological access. Liberals might support the statement due to its emphasis on using technology to improve healthcare outcomes and acknowledging systemic barriers (like broadband access), which often align with a liberal focus on equity and government intervention to address disparities. Conservatives might appreciate the pragmatic approach to not overly rely on technology when it's not feasible and support for exceptions that respect individual circumstances and local conditions, aligning with conservative values of flexibility and limited government overreach.<br><br>Ultimately, this text primarily aligns with a healthcare policy perspective focused on effectiveness, adaptability, and patient care rather than a clear political ideology. It reflects a balanced approach to integrating technology in healthcare while recognizing and adjusting to the limitations and barriers that exist in real-world settings.	neutral

AAFP	Emergency Department Call for Family Physicians (Position Statement)	2020	The text you've provided presents a viewpoint that could be interpreted as aligning with principles that are both liberal and conservative, depending on how one defines these terms and focuses on different aspects of the argument. However, the primary focus is on equitable treatment, responsibility, and fairness within the medical profession, rather than explicitly political ideologies. Here's a breakdown of how aspects of this text might align with liberal or conservative viewpoints:<br><br>### Aspects That Could Align with Liberal Viewpoints:<br>- **Emphasis on Equity and Fairness:** The text's focus on ensuring equitable treatment for family physicians, who may be disproportionately burdened by on-call duties, aligns with liberal values that prioritize fairness and equal treatment for all individuals.<br>- **Support for Worker Rights:** Advocating for proper compensation and fair work conditions for physicians who are asked to take on more than the average share of on-call responsibilities echoes liberal emphasis on workers' rights and protections.<br>- **Community Responsibility:** The acknowledgment of hospitals' obligations to meet community needs and legal obligations for emergency medical care can be seen as aligning with liberal views that emphasize the importance of social responsibility and the role of institutions in supporting community welfare.<br><br>### Aspects That Could Align with Conservative Viewpoints:<br>- **Professional Responsibility:** The emphasis on the obligation of medical staff members to provide on-call coverage as part of their professional duties can resonate with conservative values that stress personal responsibility and the fulfillment of one's duties.<br>- **Individual Accountability:** The call for family physicians to share in on-call requirements similarly to their colleagues in other specialties reflects a conservative principle of individual accountability and the expectation that each person fulfills their role within a system.<br><br>### Neutral or Non-partisan Aspects:<br>- **Practical Solutions for Systemic Issues:** The proposal for hospitals to establish fair baselines of participation for medical staff and to ensure equitable treatment across specialties is a practical approach to addressing systemic issues within healthcare. This focus on systemic reform and fair practices transcends traditional political alignments and is more centered on professional ethics and healthcare management principles.<br><br>In summary, the text does not align strictly with either liberal or conservative viewpoints but rather addresses professional ethics, equity, and responsibility in the healthcare system. Its emphasis on fairness, equitable treatment, and community responsibility intersects with values that can be appreciated across the political spectrum.	neutral
AAFP	Emergency Medical Care	7/1905	The statement, "All people should have access to emergency medical care," and the subsequent details on the roles and responsibilities of family physicians in emergency care, align more closely with viewpoints that are typically associated with liberal ideologies, especially in the context of healthcare. Liberal viewpoints often emphasize the importance of universal access to healthcare services, including emergency medical care, as a fundamental right. The emphasis on ensuring that all individuals, regardless of their location or the specific challenges they face, have access to necessary medical services reflects a commitment to equity and access in healthcare, principles often advocated by liberal ideologies.<br><br>However, it's important to note that the principle of providing access to emergency medical care can also be supported by individuals with conservative viewpoints, especially when the focus is on practical measures to ensure efficient and effective healthcare delivery. The details provided about the training of family physicians and the encouragement for them to develop protocols and have the necessary equipment to handle emergencies can be seen as emphasizing personal responsibility and local solutions, which are values often associated with conservative ideologies.<br><br>In the broader political and social discourse, the debate around healthcare often centers on how best to achieve universal access and the role of government versus the private sector in healthcare provision. While the statement and details provided do not explicitly endorse a specific policy solution, the emphasis on universal access aligns more closely with liberal viewpoints on healthcare.	neutral

AAFP	Emergency Medicine, Family Physicians in	9/2022	The text you've provided focuses on principles related to the provision of emergency medical care, emphasizing the importance of quality patient care, the role of family physicians in emergency settings, and the criteria for emergency department credentialing. It does not explicitly align with a strictly liberal or conservative viewpoint in the political sense. Instead, it addresses professional and operational standards within the healthcare system.<br><br>However, one could interpret elements of this text through the lens of broader political ideologies:<br><br>1. **Liberal Viewpoint Considerations:**<br>- Emphasizing the provision of comprehensive emergency medical services to a diverse population and the importance of quality care could resonate with liberal values that prioritize inclusive and equitable access to healthcare.<br>- The call for cooperative relationships among a variety of health professionals may also align with liberal inclinations towards collaborative and integrated approaches to healthcare.<br><br>2. **Conservative Viewpoint Considerations:**<br>- The emphasis on the role of training, experience, and current competence in determining who should provide emergency care can be seen as aligning with conservative values that stress merit and qualifications over other factors.<br>- The argument against specialty certification as the sole criterion for the ability to practice in emergency settings might also find agreement among conservatives who value practical experience and proven competence.<br><br>In essence, the text primarily addresses healthcare professionalism and standards rather than engaging directly with political ideologies. Yet, its focus on quality care, inclusivity, and merit-based credentialing can find interpretations within both liberal and conservative perspectives depending on how these themes are viewed in relation to broader healthcare policy debates.	neutral
AAFP	End Ableism in Family Medicine	10/2023	The text reflects a viewpoint that prioritizes inclusivity and the recognition of ableism (discrimination in favor of able-bodied people) within the medical community. This perspective aligns more closely with principles often associated with liberal viewpoints, which tend to emphasize equality, inclusivity, and social justice across various sectors, including healthcare. Liberal perspectives typically advocate for the recognition and addressing of systemic inequalities and biases, such as ableism, to ensure equal opportunities and treatment for all individuals, regardless of their physical or mental abilities.<br><br>Conservative viewpoints, on the other hand, might not prioritize the explicit addressing of ableism in the same way, focusing instead on traditional approaches to medicine and healthcare. However, it's important to note that the desire to improve access and equity in healthcare can cross political lines, and there may be conservatives who support efforts to combat ableism in the medical field.<br><br>The text specifically mentioning the American Academy of Family Physicians' (AAFP) commitment to promoting policies and educational curricula inclusive of people with all abilities indicates a proactive approach to diversity and inclusion. This approach is more commonly associated with liberal ideologies that advocate for systemic changes to address inequalities.	liberal
AAFP	End Corporal Punishment of Children	7/1905	The statement that the AAFP (American Academy of Family Physicians) discourages the use of corporal punishment of children tends to align more closely with a liberal viewpoint. Liberal perspectives generally emphasize the protection of individual rights and well-being, including the rights of children, and often advocate for non-violent methods of discipline that focus on understanding, communication, and positive reinforcement. Liberals are more likely to support policies and practices that aim to prevent harm and promote the psychological and physical health of children, which includes opposition to corporal punishment due to its potential negative impacts on child development and behavior.<br><br>Conservative viewpoints, on the other hand, might place a higher emphasis on traditional methods of discipline, including the use of corporal punishment, under the belief that it can be an effective way to maintain order and teach respect for authority. However, it's important to note that views on corporal punishment can vary widely within both conservative and liberal communities, and opposition to or support for such practices can also be found across a broad spectrum of political and personal beliefs.<br><br>Discussions around corporal punishment are often centered on the best ways to ensure the safety, health, and well-being of children, with various professional organizations, including those in the fields of medicine, psychology, and education, advising against its use based on research indicating potential long-term harm.	liberal

AAFP	Environmental Health and Climate Change	7/1905	The text aligns more closely with a liberal viewpoint. It emphasizes the importance of addressing climate change, reducing pollution, and protecting public access to environmental health research. These priorities are typically associated with liberal or progressive political ideologies, which often prioritize environmental protection, public health, and the regulation of pollutants and greenhouse gases. The opposition to actions that reduce public access to environmental health data or weaken protections for air and water also aligns with a liberal perspective, as it reflects a commitment to transparency, public welfare, and environmental justice. Additionally, the focus on the disproportionate impact of environmental issues on vulnerable populations is a hallmark of liberal approaches, which emphasize equity and the need to protect all segments of society, especially those most at risk.	liberal
AAFP	Equal Opportunity	2019	The statement "The AAFP (American Academy of Family Physicians) supports equal social, economic, and professional opportunity for all members," reflects a principle that can be interpreted as aligning with liberal viewpoints, particularly in its emphasis on equality and inclusivity. Liberal ideology often focuses on promoting equal opportunities, reducing inequalities, and ensuring that everyone, regardless of their background, has the same rights and opportunities in society and professionally.<br><br>However, it's important to note that the concept of supporting equal opportunities can also be embraced by some conservatives, especially if it aligns with principles of meritocracy and equal treatment under the law. The distinction often comes down to the methods proposed to achieve these equal opportunities and the extent of intervention considered appropriate by the government or institutions.<br><br>Given the broad appeal of the principle of equality, without further context on how the AAFP intends to support equal opportunities (such as specific policies or programs), it's difficult to categorize the statement strictly within a liberal or conservative viewpoint. It represents a value that could be supported across the political spectrum, albeit for different reasons and with different approaches to implementation.	liberal
AAFP	Equal Representation of Women in Family Medicine	2020	The text outlines policies from the American Academy of Family Physicians (AAFP) that aim to encourage and support the participation and leadership of women in the field of family medicine. These policies focus on encouraging women to enter the specialty, actively participate in programs and activities, and take on leadership roles, with an aim for proportionate representation in leadership.<br><br>These policies align more closely with viewpoints traditionally associated with liberal ideologies, which often emphasize equality, diversity, and inclusivity in all sectors of society, including the workplace. Liberal viewpoints typically advocate for policies that support gender equality, the breaking down of barriers to entry and advancement for underrepresented groups, and the creation of more inclusive environments.<br><br>Conservative viewpoints, on the other hand, may also support the principle of equality but might prioritize meritocracy and tradition in the approach to leadership and participation in professional fields. Conservatives might not emphasize proactive measures to ensure representation but rather focus on individual qualifications without specific initiatives aimed at promoting a particular group.<br><br>It's important to note, however, that supporting women in medicine can be seen as a broadly accepted goal across the political spectrum, and the emphasis on gender equality and representation has been increasingly recognized and supported by various organizations, regardless of their political orientation. The specific approach and emphasis on proactive policies for inclusion and representation, as mentioned in the text, tend to align more closely with liberal ideologies.	liberal
AAFP	Equality for All Families	9/2022	The text you've provided aligns more closely with a liberal viewpoint. Liberal perspectives generally advocate for equal rights and protections for all individuals, regardless of gender or sexual orientation. This includes support for legal equality for gender and sexual minorities, which encompasses not only individual rights but also those affecting families and relationships. The statement from the American Academy of Family Physicians (AAFP) emphasizes the importance of legal equality for gender and sexual minority families in contributing to overall health, family stability, and the well-being of their children, which are priorities often emphasized within liberal ideologies.<br><br>Conservative viewpoints, on the other hand, might prioritize traditional family structures and may have reservations about extending certain legal protections or recognitions to gender and sexual minority families. It's important to note, however, that there is a wide range of beliefs within both liberal and conservative communities, and perspectives can vary widely among individuals and organizations within those groups.	liberal

AAFP	Equity for Non-binary Service Members	10/2023	The text aligns more closely with a liberal viewpoint. This is evident in its support for transgender and gender-diverse individuals, particularly within the context of the military and legal documentation. Liberal perspectives generally advocate for inclusivity, diversity, and the recognition of a wide spectrum of gender identities beyond the traditional binary of male and female. The emphasis on providing gender-inclusive identity options on legal and government documentation, including for uniformed service members, reflects a commitment to these principles.<br><br>Conservative viewpoints, on the other hand, tend to emphasize traditional values and may be less supportive of policies that recognize gender identities beyond the binary of male and female, especially within institutions like the military. Therefore, the stance taken by the American Academy of Family Physicians (AAFP) as described in your text would more likely be associated with liberal ideologies.	liberal
AAFP	Ethical Application of Artificial Intelligence in Family Medicine	10/2023	The text outlines a perspective on the integration of artificial intelligence (AI) and machine learning (ML) into family medicine, emphasizing the enhancement of patient care, transparency, the addressing of implicit bias, the importance of training data diversity, privacy respect, system integration, accountability, and trustworthiness. These principles reflect a viewpoint that is more aligned with a liberal perspective, particularly in its emphasis on equity, inclusiveness, transparency, and accountability.<br><br>Liberal viewpoints often prioritize inclusivity and equity, addressing systemic issues such as racism and health inequity, and the comprehensive care approach mentioned for all genders, races, ethnicities, sexual orientations, and ages aligns with this. The focus on mitigating health inequities, including systemic racism, by collaborating with community stakeholders to effect positive change is also indicative of a liberal stance, which typically emphasizes social justice and community engagement.<br><br>The stress on transparency, addressing implicit bias, and ensuring privacy and data protection align with liberal values regarding individual rights and protections, as well as with a general liberal emphasis on ethical considerations in technology and healthcare. The insistence on diverse and representative training data for AI/ML systems to avoid perpetuating existing biases or creating new health inequities reflects a commitment to fairness and equality, key tenets of liberal ideology.<br><br>Moreover, the call for AI/ML solutions to be evaluated with rigor, integrated into systems thoughtfully, and designed for trustworthiness and safety aligns with a proactive, regulatory approach to innovation, which is more characteristic of liberal viewpoints that advocate for strong oversight to protect individual and public interests.<br><br>In contrast, conservative viewpoints might focus more on traditional healthcare models, less on systemic inequalities, and might have a different emphasis regarding the role and regulation of technology in healthcare. While there is crossover between liberals and conservatives on the importance of healthcare quality and innovation, the specific focuses and approaches outlined in this text are more closely aligned with liberal values.	liberal
AAFP	Ethics and Advance Planning for End-of-Life Care	9/2022	The text you've provided outlines a comprehensive view on advance directives, end-of-life care, and the ethical considerations surrounding medical decisions at the end of life. It emphasizes respect for patient autonomy, the importance of culturally sensitive and compassionate care, and the role of family physicians in ensuring that patients' wishes are understood and respected. These principles focus on patient rights, informed decision-making, access to palliative and hospice care, and the option for medical aid in dying under certain circumstances.<br><br>This perspective does not align strictly with traditional liberal or conservative viewpoints as they are commonly understood in the political sense. Instead, the focus is on medical ethics, patient rights, and the provision of compassionate care, which are values that can be endorsed across a broad spectrum of political and ethical beliefs.<br><br>However, the emphasis on patient autonomy, the right to choose or refuse treatment, and the support for medical aid in dying could be more closely associated with liberal viewpoints, which generally prioritize individual rights and freedoms, including in medical and personal decisions. The support for evidence-based care, cultural sensitivity, and the incorporation of nontraditional treatments also align with a more progressive approach to healthcare.<br><br>Conversely, the respect for life, emphasis on palliative care, and the conditions under which medical aid in dying is considered could find resonance within conservative circles, especially those that value the sanctity of life but also recognize the importance of reducing suffering and respecting individual wishes at the end of life.<br><br>In summary, while the text embodies principles that might find broader acceptance across the political spectrum, its focus on autonomy, informed consent, and individual choice in healthcare decisions leans towards principles often associated with liberal	probably liberal

			viewpoints. Nonetheless, it is essential to recognize that issues of healthcare, patient rights, and end-of-life care transcend simple political categorizations and are deeply personal, ethical, and complex in nature.	
AAFP	Evaluation of Family Medicine Specialty Certifying Board, Guiding Principles for the	7/1905	The text you've provided outlines principles for specialty-certifying boards in the context of medical practice, focusing on transparency, relevance, representation, non-burdensomeness, and appropriate use of board certification. These principles aim to ensure that certification processes are fair, relevant to practice, and not unduly burdensome to physicians, while also being transparent and providing for physician input and accountability.<br><br>These principles do not neatly align with a strictly liberal or conservative viewpoint in the traditional political sense. Instead, they reflect a professional and ethical stance that seeks to balance the interests of various stakeholders in the healthcare system, including physicians, patients, and the institutions that govern medical practice. Here's why:<br><br>- **Transparency** and **nonprofit organizational structure** can be appealing to both liberal viewpoints, which often emphasize accountability and equality, and conservative viewpoints, which value efficiency and fiscal responsibility.<br>- **Relevance of board certification to professional practice** and **defined opportunities for representation of family physician interests** suggest a desire to ensure that certification remains closely tied to the practical and evolving needs of healthcare providers and patients, a principle that transcends traditional political divides.<br>- **Certification procedures are not burdensome to physicians** and the emphasis on **appropriate use of board certification** reflect a pragmatic approach to regulation, one that seeks to minimize unnecessary barriers to practice while maintaining high standards of care.<br><br>This document seems more focused on good governance, ethical standards, and practical concerns within the medical profession rather than pushing a specific political agenda. It advocates for practices that ensure the certification process is relevant, fair, and not overly burdensome, which could be supported by individuals across the political spectrum who are concerned with improving healthcare quality and accessibility.	neutral

AAFP	Expansion of Residency Training Programs of Federally Qualified Community Health Centers (FQHCs) and Teaching Health Centers (THCs)	9/2022	The text you've provided outlines a position supporting the expansion of residency training programs at Federally Qualified Health Centers (FQHCs) and the permanence of the Teaching Health Center (THC) Graduate Medical Education program, with specific conditions related to funding, equitable distribution, and commitment to education. These views are generally aligned with policies that focus on expanding access to healthcare education and services, improving healthcare workforce distribution, and ensuring sustainability in funding. Such positions often find more favor within liberal viewpoints, especially those that emphasize public investment in healthcare infrastructure, education, and access to services for underserved populations.<br><br>Liberal viewpoints typically advocate for increased government involvement in healthcare to ensure that it is accessible and equitable for all citizens, including support for programs that aim to address disparities in healthcare access and education. The emphasis on sustainable funding, equitable distribution, and organizational commitment to education aligns with these liberal principles of equity, access, and public responsibility in healthcare.<br><br>Conservative viewpoints, on the other hand, might emphasize less government intervention in healthcare, preferring market-driven solutions and minimizing public expenditure on healthcare programs. However, there can be conservative support for specific initiatives that align with goals of efficiency, effectiveness, and sustainability in healthcare education and delivery, especially if they also support community health needs and can be shown to be cost-effective in the long run.<br><br>The text reflects a policy stance rather than a purely political viewpoint, aiming to improve medical education infrastructure and access to quality healthcare services. While it may resonate more with liberal policy priorities, aspects of it could also gain support from conservatives who see value in its goals for healthcare system improvement and efficiency.	probably liberal
AAFP	Expectations of Family Medicine Residency Graduates	1/2022	The text you've provided outlines the competencies expected of family medicine residency graduates, focusing on comprehensive, equitable, and community-oriented care. It emphasizes evidence-based practice, cultural competence, preventive care, lifelong learning, teamwork, patient advocacy, and addressing health disparities.<br><br>This document does not explicitly align with a liberal or conservative viewpoint in the traditional political sense. Instead, it reflects principles of medical education and practice that prioritize patient-centered care, health equity, and the social determinants of health. These principles are broadly accepted within the medical community as essential to the practice of modern medicine.<br><br>However, discussions on topics such as health equity, access to affordable care, and addressing social determinants of health can intersect with political viewpoints:<br><br>- **Liberal viewpoints** might emphasize the importance of addressing health disparities, ensuring universal access to healthcare, and the role of social determinants in health outcomes. The focus on equitable, affordable care and community health could resonate with liberal perspectives on health care as a right and the need for systemic interventions to achieve health equity.<br><br>- **Conservative viewpoints** might focus on individual responsibility for health, the role of private sector in healthcare innovation and delivery, and concerns about the extent of government involvement in healthcare. However, conservatives also value the importance of family and community support systems in providing care, which could align with the emphasis on family and community-oriented care outlined in the document.<br><br>Ultimately, the principles outlined in the text are foundational to the practice of family medicine and aim to ensure that physicians are equipped to meet the diverse needs of their patients and communities. They transcend simple political categorization, reflecting a commitment to improving health outcomes for all individuals through high-quality, comprehensive care.	neutral

AAFP	Expedited Partner Therapy	9/2022	The text you've provided appears to be a neutral statement regarding a specific healthcare policy supported by the American Academy of Family Physicians (AAFP) concerning expedited partner therapy (EPT) and its alignment with the Centers for Disease Control and Prevention (CDC) recommendations. This statement primarily focuses on a medical practice and public health guideline rather than explicitly aligning with a liberal or conservative viewpoint.<br><br>Expedited partner therapy (EPT) is a treatment strategy for sexual partners of individuals diagnosed with sexually transmitted infections (STIs), such as chlamydia or gonorrhea, without the need for the partner to first see a healthcare provider. The goal of EPT is to prevent the spread of STIs and reduce reinfection rates.<br><br>Support for EPT from a medical and public health perspective is based on evidence showing its effectiveness in controlling STI transmission. The mention of checking state law requirements suggests an acknowledgment of the legal and regulatory framework within which healthcare providers must operate, further emphasizing the practical, rather than ideological, nature of the statement.<br><br>While healthcare policies can be influenced by broader political or ideological beliefs, the specific support for EPT by the AAFP, as outlined in your text, is more about implementing evidence-based practices to improve public health outcomes. Political or ideological perspectives might influence opinions on such healthcare practices, but the text itself is focused on medical recommendations and legal compliance rather than partisan viewpoints.	neutral
AAFP	Fairness in Federal Programs for All U.S. Citizens	1/2022	The statement "All U.S. citizens, including citizens of the territories, should be treated fairly in federal programs" aligns more closely with a liberal viewpoint, particularly when considering the political context of the United States. Liberal ideologies often emphasize equality, fairness, and the extension of rights and benefits to all individuals, including marginalized or less represented groups. The mention of citizens of the territories highlights a focus on inclusivity and equity, which are key principles in liberal thought.<br><br>Conservative viewpoints, while also valuing fairness and the rule of law, may prioritize different aspects of policy, such as states' rights, limited federal government intervention, and a more traditional interpretation of the Constitution that could affect how territories and their citizens are viewed in relation to federal programs.<br><br>However, it's important to note that the desire for fair treatment in federal programs can cross ideological lines, and there may be conservatives who support this view as well, especially if it aligns with principles of justice and efficiency. The distinction between liberal and conservative viewpoints often depends on the broader context of the policies in question, including how rights and fairness are defined and implemented.	liberal
AAFP	Family Medicine Academic Department	7/1905	The text you've provided outlines standards and operational guidelines for departments of family medicine within U.S. medical schools, focusing on their structure, mission, and functional requirements. It doesn't explicitly align with traditional liberal or conservative viewpoints as it focuses on educational and institutional guidelines rather than political or social ideologies.<br><br>The emphasis on education, research, and clinical service as the core missions of family medicine departments is broadly aligned with a universal approach to medical education, valuing a comprehensive and interdisciplinary approach to healthcare education and delivery. The call for adequate resources, representation, and involvement in scholarly activities reflects a commitment to quality and excellence in medical education, which transcends political divisions.<br><br>However, one might argue that the text indirectly touches upon values that can be associated with broader political or ideological perspectives:<br><br>1. **Emphasis on Community and Preventive Medicine:** A focus on community health and preventive medicine could be seen as aligning more closely with liberal viewpoints that prioritize public health initiatives, preventive care, and holistic approaches to healthcare. These are often associated with policies aimed at reducing healthcare disparities and improving access to healthcare services for all community members.<br><br>2. **Interdisciplinary and Collaborative Approach:** The call for collaboration among departments to achieve institutional objectives might resonate with a more progressive approach to education and healthcare, emphasizing teamwork, interprofessional education, and integrated care models. This approach can be seen as part of a broader liberal agenda to foster inclusivity, diversity, and a comprehensive healthcare system that addresses various determinants of health.<br><br>3. **Resource Allocation:** The insistence on comparable representation, funding, space, and educational venues can reflect a commitment to equity and fairness in the distribution of resources. While this is a universal principle, debates over resource	probably liberal

			allocation can mirror larger political discussions about funding priorities and investment in public goods, including education and healthcare, which are often more emphasized in liberal policy agendas.<br><br>Despite these potential indirect associations, the text primarily presents a neutral stance focused on the operational and educational standards of family medicine departments in medical schools. It aims to ensure that these departments are well-equipped to fulfill their mission, without delving into the broader political implications of healthcare policy and education.	
AAFP	Family Medicine Departments	10/2023	The text you've provided outlines a policy or position regarding the structure and authority of departments within hospitals, particularly emphasizing the importance and autonomy of departments of family medicine. This policy seeks to ensure that family medicine departments have rights, duties, and responsibilities on par with other specialty departments and can directly recommend privileges within their scope without needing approval from other departments. The final decision-making power rests with the hospital's governing body or board of trustees.<br><br>This text does not neatly align with traditional political labels like "liberal" or "conservative" as it focuses on hospital administration and medical staff organization rather than broader political or social issues. However, its emphasis on the equality and autonomy of the family medicine department can be interpreted through various lenses:<br><br>1. **Liberal Viewpoint Alignment**:<br>- Advocating for equality and equitable treatment across different medical departments could be seen as aligning with liberal values, which often emphasize fairness, equity, and the dismantling of hierarchical structures that might limit participation or recognition.<br>- Promoting comprehensive care through family medicine, which often serves a wide range of patients, including underserved populations, aligns with a focus on accessible and equitable healthcare, a priority for many liberal perspectives.<br><br>2. **Conservative Viewpoint Considerations**:<br>- The emphasis on departmental autonomy and the reduction of bureaucratic oversight (in terms of not requiring assent or approval from other departments) could resonate with conservative values that favor decentralized decision-making and less regulation.<br>- The principle that the ultimate responsibility lies with the hospital governing body or board of trustees might align with conservative views on the importance of traditional structures and hierarchies in maintaining order and accountability.<br><br>Ultimately, the text seems to focus more on improving the functionality and governance within hospitals rather than advocating for a specific political ideology. It proposes a model where departments of family medicine are given autonomy and respect similar to other specialties, aiming to enhance the hospital's overall service quality and patient care. The alignment with liberal or conservative viewpoints might depend more on how individuals interpret the implications of these organizational changes rather than the content itself explicitly advocating for one perspective over another.	neutral

AAFP	Family Medicine Faculty	2019	The text provided outlines a stance from the American Academy of Family Physicians (AAFP) regarding the qualifications of family medicine department chairs, directors of residency programs, and faculty involved in teaching family medicine. It emphasizes the importance of documented training, experience, demonstrated abilities, and current competence, which may include certification by relevant medical boards, for those in teaching and leadership positions within family medicine. It also advocates for qualifications that meet current accreditation requirements for other faculty who are not family physicians but work within these departments or programs.<br><br>This stance is primarily professional and educational in nature, focusing on standards for faculty qualifications in medical education. It does not directly align with traditional political divides such as liberal or conservative viewpoints. Instead, it reflects a commitment to maintaining high standards of medical education and ensuring that those who teach and lead in the field of family medicine are well-qualified. This focus on professional qualifications and accreditation standards is a matter of educational policy and professional ethics rather than a partisan political issue.<br><br>However, indirectly, one might argue that any emphasis on rigorous standards for medical education and the endorsement of continuous certification could be seen in a broader political or ideological context. For example:<br><br>- **Liberal viewpoint**: Might appreciate the emphasis on high standards and continuous improvement in medical education, as it aligns with values of progress, quality healthcare for all, and the importance of evidence-based practices.<br><br>- **Conservative viewpoint**: Might support the emphasis on merit-based qualifications and standards as reflecting values of personal responsibility, excellence, and maintaining high professional standards.<br><br>In summary, the text is primarily about professional standards and does not directly align with a liberal or conservative viewpoint. Its focus is on the qualifications of medical educators, which is a concern that transcends political divides, aiming instead to ensure the quality of medical education and healthcare outcomes.	neutral
AAFP	Family Medicine Interest Groups	7/1905	The text you've provided outlines a recommendation from the American Academy of Family Physicians (AAFP) regarding family medicine interest groups (FMIGs). It advocates for the support and participation of medical students in FMIGs and calls on various entities within the medical community to provide structural and financial support. This position is centered on educational policy and professional development within the field of family medicine rather than a specific political ideology.<br><br>However, the emphasis on community involvement, educational support, and collaborative efforts between different stakeholders could align with principles that are often emphasized in liberal viewpoints, such as the importance of community support systems, education, and collective efforts for the common good. Liberals typically advocate for strong support systems in education and healthcare, emphasizing the role of community and government in fostering opportunities for all.<br><br>Conversely, conservative viewpoints generally prioritize individual responsibility, limited government intervention, and private sector solutions to social and economic issues. From a conservative perspective, the recommendation for support might be viewed through the lens of how best to achieve educational and professional development goals without overly relying on government or public funds, if at all.<br><br>Nevertheless, the text primarily reflects professional and educational goals within the medical community, and any alignment with liberal or conservative viewpoints would be indirect and based on broader interpretations of the principles behind the support for FMIGs. It is more accurately described as a policy recommendation within the field of healthcare education rather than a political statement.	probably liberal

AAFP	Family Medicine's Role in Undergraduate Medical Education	10/2023	The text outlines recommendations from the American Academy of Family Physicians (AAFP) regarding the integration of family medicine into medical school curricula. These recommendations focus on ensuring medical students understand the role of family medicine in healthcare, advocating for an increase in student choice towards family medicine, and producing a diverse workforce to meet the health needs of underserved areas. The emphasis is on early exposure, innovative teaching methods, longitudinal clinical experiences, role models, and the institutional recognition of family medicine's value.<br><br>This text does not inherently align strictly with either a liberal or conservative viewpoint in the political sense. Instead, it addresses educational and professional standards within the medical field, focusing on improving medical education, healthcare delivery, and addressing health equity and underserved communities. These goals can be seen as aligned with broader societal and healthcare improvement objectives that are supported across the political spectrum.<br><br>However, certain aspects of the recommendations, such as the focus on health equity and serving underserved areas, might resonate more with those holding liberal viewpoints, as these are often associated with a focus on social justice and equal access to healthcare. Similarly, the emphasis on comprehensive care and preventive medicine aligns with healthcare approaches often advocated by those on the liberal end of the spectrum.<br><br>On the other hand, conservatives might support aspects of the recommendations that emphasize the importance of choice (in this case, student choice of family medicine) and the practical aspects of healthcare delivery, such as in-office procedures and the use of emerging technologies. The focus on creating a diverse workforce could also be seen as a recognition of the need for a healthcare system that serves all parts of society effectively, which can be a point of agreement across political lines.<br><br>In summary, while the AAFP's recommendations focus on medical education and practice rather than political ideology, different aspects of these recommendations may resonate differently across the political spectrum, depending on how they align with broader views on healthcare, education, and societal needs.	neutral
AAFP	Family Medicine, Definition of	2019	The text you've provided is a factual description of the field of family medicine and does not inherently align with a liberal or conservative viewpoint. It outlines the broad scope and comprehensive nature of family medicine as a medical specialty, focusing on the integration of various sciences to provide care for individuals and families across all ages, genders, organ systems, and diseases.<br><br>In political or ideological discussions, the terms "liberal" and "conservative" typically refer to positions or viewpoints on social, economic, and political issues. Descriptions of medical specialties, such as the one provided, are neutral and pertain to the practice and principles of medicine rather than political ideologies.<br><br>Therefore, this text is best understood as an educational or informational statement about the field of family medicine, rather than as an expression of a political or ideological stance.	neutral

AAFP	Family Medicine, Quality Health Care in	1/2022	The text you've provided outlines a viewpoint on healthcare that emphasizes several key principles: accessibility, safety, cost-effectiveness, equity, evidence-based practice, responsiveness to patient needs and preferences, and respect for personal values and beliefs. These principles can be found across the political spectrum but are often more closely associated with liberal viewpoints in the context of the ongoing healthcare debate in many countries, including the United States.<br><br>1. **Accessibility**: Advocating for healthcare to be accessible to all individuals aligns with liberal viewpoints that emphasize the right to healthcare and the reduction of barriers to accessing care.<br><br>2. **Cost-effectiveness**: While both liberal and conservative viewpoints might agree on the importance of cost-effectiveness, the manner in which it is achieved (e.g., through government intervention vs. market-based solutions) can be a point of contention.<br><br>3. **Equity**: The focus on equity in healthcare is strongly aligned with liberal ideologies that prioritize reducing disparities in health outcomes across different populations.<br><br>4. **Evidence-based care**: This principle is widely supported across the healthcare profession and, while not inherently political, its application can be influenced by policy decisions that reflect liberal or conservative values.<br><br>5. **Responsiveness and respect for patients' values**: Emphasizing patient-centered care and respect for personal values aligns with a more holistic approach to healthcare, which is often associated with liberal perspectives that prioritize individual rights and autonomy in healthcare decisions.<br><br>While these principles are broadly aligned with liberal views on healthcare reform, particularly in the emphasis on equity and accessibility, it's important to recognize that there can be significant overlap in what individuals and policymakers from both liberal and conservative backgrounds might consider important in achieving quality healthcare. The distinction often lies in the approaches and policies proposed to realize these principles.	liberal
AAFP	Family Medicine, Specialist in	3/2023	The text you've shared is factual and neutral, focusing on the criteria for defining a "specialist" in family medicine according to the American Academy of Family Physicians (AAFP). It outlines the qualifications required for physicians to be considered specialists in family medicine, including completing an approved residency program, maintaining eligibility for active membership in the AAFP, or obtaining certification by a recognized certifying board in the specialty.<br><br>This information is apolitical and does not inherently align with either liberal or conservative viewpoints. It is a professional standard set by a medical organization to ensure the quality and competency of family medicine physicians. Political ideologies typically concern themselves with matters of public policy, governance, social issues, and economic theories, rather than the specific credentialing criteria within professional fields such as medicine.	neutral
AAFP	Family Physician Burnout, Well-Being, and Professional Satisfaction (Position Paper)	10/2023	The text provided primarily aligns with perspectives and approaches that can be found across the political spectrum, but it does contain elements that might be more closely associated with liberal viewpoints, particularly in its emphasis on systemic and organizational solutions to physician burnout, the focus on diversity and inclusivity, and the concern for work-life balance and well-being. Liberal viewpoints often prioritize systemic change to address societal issues, emphasize the importance of diversity and equity, and advocate for policies that support the well-being of individuals within systems. The text's focus on addressing the root causes of physician burnout through systemic change, its acknowledgment of the unique challenges faced by physicians of various demographics (including race, ethnicity, gender, sexual orientation), and its advocacy for a culture that supports physician well-being and professional satisfaction all resonate with these liberal values.<br><br>However, it's important to note that the concern for physician burnout and the pursuit of systemic solutions to improve health care quality and provider well-being are not exclusively liberal issues. They are broadly recognized as critical aspects of healthcare reform and quality improvement by professionals across the political spectrum. The emphasis on systemic and organizational change, rather than solely individual resilience or personal responsibility, might lean more towards a liberal approach, but the overall goal of improving healthcare outcomes and provider well-being is universally valued.<br><br>Conservative viewpoints might also support aspects of the text, especially those related to improving the efficiency and effectiveness of healthcare delivery, reducing regulatory burdens that contribute to burnout, and fostering professional satisfaction through autonomy and decision-making. The differences between liberal and conservative perspectives might be more about the means and emphasis rather than the fundamental goals of healthcare improvement.	probably liberal

			In summary, while the text's approach to addressing physician burnout through systemic and organizational change aligns more closely with liberal values of systemic reform and inclusivity, its overarching goals of improving healthcare outcomes and provider well-being are shared across the political spectrum.	
AAFP	Family Physician Workforce and Residency Education	1/2022	The text provided outlines policy recommendations and actions for the American Academy of Family Physicians (AAFP) focusing on the education, support, and practice of family medicine. It emphasizes the importance of continuously monitoring the need for family physicians, enhancing teaching skills, supporting residency programs, and ensuring that family physicians can meet public health needs. Additionally, it highlights the role of family physicians in delivering comprehensive and patient-focused care.<br><br>This text does not neatly align with traditional liberal or conservative viewpoints as understood in a political sense. Instead, it represents a professional, organizational perspective focused on improving medical education and healthcare delivery within the field of family medicine. The emphasis is on education, quality of care, and the adaptability of the healthcare workforce to meet community needs, which are concerns that transcend political affiliations. However, interpretations can vary based on the elements highlighted:<br><br>1. **Liberal Viewpoint Considerations**: A liberal perspective might appreciate the focus on comprehensive healthcare, community needs, and the support for educational programs aimed at improving quality of care. Liberals often emphasize the importance of accessible, high-quality healthcare and might view the proactive approach to education and support for family medicine as aligned with broader goals of improving healthcare access and outcomes.<br><br>2. **Conservative Viewpoint Considerations**: A conservative perspective might value the emphasis on the primary role of family physicians in healthcare delivery, potentially seeing it as a way to maintain traditional healthcare relationships and structures. Conservatives might also support the focus on improving the efficiency and effectiveness of medical education and training, seeing it as a way to ensure high-quality care without necessarily expanding government involvement in healthcare.<br><br>The proposals focus on internal improvements within the field of family medicine rather than suggesting broad policy changes at the national level, which makes the text more about professional practice and less about partisan policy debates. Therefore, while individual elements might resonate more with one political perspective or another, the text as a whole is best understood as a professional stance on improving the field of family medicine rather than as a statement aligned with a specific political ideology.	neutral

AAFP	Family Physician, Definition	7/1905	The text you've provided outlines the role and qualifications of family physicians, emphasizing their comprehensive training and ability to provide care to all family members regardless of various factors. This description focuses on the professional scope and responsibilities of family physicians, detailing their ability to serve as patient advocates and coordinate care effectively.<br><br>The content of this statement is primarily factual and educational, describing the broad and integrative role of family physicians in healthcare. It does not explicitly align with a liberal or conservative viewpoint, as it focuses on the medical profession's standards and expectations rather than political or ideological beliefs.<br><br>Both liberal and conservative perspectives might find value in the emphasis on comprehensive and preventive healthcare, as well as the focus on patient advocacy and the efficient use of healthcare resources. Liberals might appreciate the inclusive and holistic approach to healthcare, emphasizing preventive services and the social determinants of health. Conservatives might support the focus on family and the efficient use of resources through the appropriate use of consultants and community resources.<br><br>Therefore, the text is more aligned with professional medical standards and practices rather than being inherently political or ideological in nature.	neutral
AAFP	Family Physicians as Role Models for Underrepresented Students	7/1905	The text provided reflects a viewpoint more commonly associated with liberal or progressive ideologies, especially in its focus on addressing underrepresentation and inequity in the medical workforce. The emphasis on diversity, equity, and inclusion aligns with liberal values that prioritize social justice, equal opportunity, and efforts to reduce disparities in various sectors, including healthcare. The call for family physicians to act as role models, advocate for the profession, and take an active interest in the educational aspirations of underrepresented populations, along with encouraging residency programs to engage with these communities, underscores a commitment to community engagement and social responsibility. These themes are often more pronounced in liberal viewpoints, which tend to support systemic change to address inequalities.<br><br>Conservative viewpoints, while also concerned with the quality of healthcare and education, might focus more on individual responsibility, merit-based achievement, and less on systemic or structural factors contributing to underrepresentation. They may advocate for improving healthcare and education but might prioritize different methods, such as private sector solutions or less government intervention.<br><br>However, it's important to note that addressing healthcare inequity and promoting diversity in the medical field can cross ideological lines, with individuals from various political backgrounds recognizing the importance of a diverse medical workforce in providing equitable care. The specific framing and solutions proposed for addressing these issues might differ, reflecting broader ideological beliefs.	liberal

AAFP	Family Physicians Delivering Emergency Medical and Urgent Care	10/2023	The text you've provided outlines a series of principles supporting the role of family physicians in emergency departments (EDs), urgent care settings, and rural emergency hospitals, emphasizing their broad scope of training, versatility, and importance in providing comprehensive, cost-effective care. These arguments are largely centered on healthcare accessibility, quality, and efficiency, rather than aligning directly with traditional political ideologies such as liberal or conservative.<br><br>However, the principles touch on issues that can be interpreted through both liberal and conservative lenses, depending on the aspects one chooses to emphasize:<br><br>1. **Liberal Viewpoint Considerations**:<br>- Emphasizing the importance of accessible, community-based healthcare and the role of family physicians in ensuring that care reaches underserved or rural areas might resonate with liberal ideologies focused on equity and healthcare access for all.<br>- The support for comprehensive training and evidence-based practice aligns with a preference for rigorously tested, scientifically backed approaches to healthcare, a stance often associated with liberal perspectives.<br>- Highlighting the need for physician-led teams and the limitations of substituting less trained professionals could be seen as advocating for high standards of care and patient safety, themes common in liberal healthcare discussions.<br><br>2. **Conservative Viewpoint Considerations**:<br>- The emphasis on cost-effectiveness and efficiency, particularly in the context of leveraging family physicians to address healthcare delivery challenges, can align with conservative views that prioritize fiscal responsibility and efficiency in healthcare.<br>- Advocating for the role of family physicians in emergency and urgent care settings based on their broad training and ability to provide care across a wide range of conditions might appeal to conservative ideals of practicality and resourcefulness in addressing healthcare needs.<br>- The principles also touch on the importance of individual training and competence over specialty certification alone, which can resonate with conservative values of meritocracy and individual achievement.<br><br>Ultimately, the text promotes a pragmatic approach to healthcare delivery that emphasizes the skills and training of family physicians as essential to addressing current healthcare challenges, especially in emergency and rural settings. This approach can find support across the political spectrum, depending on the specific aspects one focuses on, such as improving healthcare access, enhancing quality of care, or ensuring efficient use of healthcare resources.	neutral
AAFP	Family, Definition of	2019	The text you've provided presents a definition and role of the family in society that doesn't neatly align with a strictly liberal or conservative viewpoint as commonly understood in political terms, especially within the context of American politics. Instead, it outlines a functional perspective on the family, emphasizing its importance in economic support and protection, particularly for vulnerable individuals.<br><br>Both liberal and conservative viewpoints might find elements of this description agreeable for different reasons:<br><br>- **Conservative Viewpoint:** Conservatives often emphasize the importance of traditional family structures as essential to societal stability and the welfare of individuals. They might agree with the text's emphasis on the family's role in providing economic and protective needs, viewing it as supportive of their advocacy for family values and the traditional family as the backbone of society.<br><br>- **Liberal Viewpoint:** Liberals might focus on the inclusion of "genetic and/or emotional relationship" to define family, which can be interpreted as inclusive of various family structures beyond the traditional nuclear family. This broader definition could align with liberal values that advocate for recognition and support of diverse family configurations, including single-parent households, LGBTQ+ families, and other non-traditional arrangements.<br><br>The text's emphasis on the family's role in society, particularly regarding support and protection of vulnerable members, is a point that can cross ideological lines. Both political viewpoints recognize the importance of the family, though they may differ on what family structures should be supported or prioritized.<br><br>This analysis is based on a general understanding of liberal and conservative viewpoints and may not capture all nuances or the diversity of opinions within each ideological camp. The text itself seems more focused on describing a sociological function of the family rather than promoting a specific ideological stance.	neutral

AAFP	Fee-splitting	7/1905	The text you've provided outlines a position from the American Academy of Family Physicians (AAFP) regarding fee-splitting, specifically opposing the practice due to ethical concerns and the potential impact on patient care. This stance doesn't neatly align with traditional liberal or conservative political viewpoints because it primarily addresses professional and ethical standards within the medical field rather than broader political or economic ideologies.<br><br>Liberal and conservative viewpoints typically differ on issues like government intervention in healthcare, healthcare funding, and access to healthcare services. Liberals often advocate for more government involvement in healthcare to ensure equitable access for all, while conservatives typically support a more market-driven approach, emphasizing private sector solutions and individual choice.<br><br>The AAFP's opposition to fee-splitting is based on ethical considerations regarding transparency, patient autonomy, and the integrity of the physician-patient relationship. It focuses on ensuring that medical decisions are made in the best interest of the patient without undue influence from financial arrangements between healthcare providers. This stance is more about upholding medical ethics and less about a specific political ideology, demonstrating the organization's commitment to patient-centered care.<br><br>In summary, the AAFP's position on fee-splitting transcends traditional political divisions, focusing instead on ethical standards and the quality of patient care, which are priorities shared across the healthcare profession regardless of individual political beliefs.	neutral
AAFP	Fees to Physicians for Referrals to Other Health Care Providers	1/2022	The text you've shared appears to be a statement on ethical guidelines regarding physician payments and business inducements. It emphasizes the inappropriateness of physicians receiving payments or other forms of value from entities in exchange for generating business for those entities. It also clarifies that this stance does not conflict with existing legal frameworks and exceptions, such as safe harbors provided under Federal Anti-Kickback legislation, Stark Law, and other similar arrangements that are part of the U.S. healthcare system.<br><br>This statement does not neatly align with a purely liberal or conservative viewpoint in the traditional political sense. Instead, it reflects ethical considerations in the medical profession and compliance with legal standards to prevent conflicts of interest and ensure the integrity of healthcare practices. The text is more about legal and ethical standards in healthcare rather than a political ideology.<br><br>Both liberal and conservative viewpoints might support the principles outlined in the text, as both sides generally agree on the need for ethical standards in healthcare, although they might disagree on the specifics of healthcare policy and regulation. Liberals might emphasize the protection of patient interests and equitable access to care, while conservatives might focus on the importance of market-driven solutions and the reduction of regulatory burdens that interfere with physician practices. However, the ethical stance against kickbacks and for transparency in healthcare transactions is a bipartisan concern, aligned with broader interests in maintaining trust in healthcare systems and preventing fraud or abuse.	neutral
AAFP	Fees, Global Surgical	2019	The text you provided outlines the American Academy of Family Physicians (AAFP)'s definition and stance on global surgical fees and their belief in a zero-day global period for surgical services. This topic primarily relates to healthcare policy and payment systems rather than directly aligning with traditional liberal or conservative viewpoints. However, the implications of such policies can resonate differently across the political spectrum, depending on broader healthcare policy beliefs.<br><br>Liberal viewpoints generally support more comprehensive healthcare coverage and may advocate for policies that ensure broader access to care, including post-operative. They might support the idea of revaluing surgical services to ensure that payments are fair and cover necessary post-operative care, seeing it as a way to improve healthcare access and quality.<br><br>Conservative viewpoints often emphasize cost control, efficiency in healthcare, and minimizing governmental or insurance-based intervention. From this perspective, there might be concerns about the potential for increased healthcare costs if surgical services are revalued to include more post-operative care outside of a global fee, unless it is shown to also improve efficiency or outcomes in a cost-effective manner.<br><br>The AAFP's stance focuses on the technical aspects of billing and compensation for surgical services, aiming for a system that they believe would more accurately reflect the work done by physicians. This policy suggestion is rooted in a desire for fairness and accuracy in physician compensation, rather than a clear-cut liberal or conservative ideology. However, the broader implications of such policy changes on healthcare costs, access, and quality can be interpreted differently depending on one's political or ideological leanings.	neutral

			Therefore, while the text itself is neutral and technical, focusing on healthcare policy specifics, its support or opposition might align with broader liberal or conservative ideologies based on how it is perceived to affect healthcare access, quality, and costs.	
AAFP	Fellowship, Definition	1/2022	The text you've provided describes a factual aspect of medical education, specifically about fellowships after family medicine residency. It discusses the structured training that leads to additional knowledge and expertise in a particular area and mentions the possibility of earning a Certificate of Added Qualification. This information is neutral and educational, focusing on the process and outcomes of post-residency training in medicine. It does not align with or promote a specific political ideology, liberal or conservative. It is purely informational, related to medical education, and does not engage with political viewpoints or ideologies.	neutral
AAFP	Female Genital Mutilation	1/2022	The text provided outlines a medical and human rights perspective on Female Genital Mutilation or Cutting (FGM/C), rather than aligning explicitly with a liberal or conservative political viewpoint. It presents the stance of the American Academy of Family Physicians (AAFP) on FGM/C, emphasizing the organization's opposition to the practice based on health, ethical, and human rights grounds. The AAFP's position includes a call for efforts to end FGM/C, education for physicians about the practice and its management, and the provision of culturally sensitive support for affected individuals.<br><br>FGM/C is widely recognized as a violation of human rights by international organizations and is opposed across a broad spectrum of political and cultural contexts. Opposition to FGM/C is generally based on concerns about physical harm, violation of bodily autonomy, and the negative impact on women's health, rather than being tied to a specific political ideology. The focus on medical evidence, human rights, and the provision of sensitive and informed healthcare support reflects a universal approach to addressing the issue that transcends traditional political divisions.	neutral
AAFP	First Dollar Coverage for Preventive Care	9/2022	The text provided outlines a policy recommendation from the American Academy of Family Physicians (AAFP) advocating for health insurance plans, including high-deductible health plans (HDHPs), to offer coverage for preventive services without the need for a deductible or co-insurance. This recommendation is centered around the idea that ensuring access to preventive care without additional out-of-pocket costs can improve public health outcomes and potentially reduce overall healthcare costs by catching health issues early.<br><br>The viewpoint aligns more closely with liberal or progressive ideologies in the context of U.S. politics. Liberal viewpoints generally emphasize the importance of accessible healthcare for all and often support policies that aim to reduce barriers to receiving care, including financial barriers. The emphasis on first-dollar coverage for preventive services reflects a belief in proactive healthcare measures and a prioritization of public health.<br><br>Conservative viewpoints, on the other hand, often prioritize market-based solutions to healthcare and may favor policies that encourage consumer-driven healthcare choices, such as high-deductible health plans that ostensibly incentivize consumers to make more cost-conscious healthcare decisions. From this perspective, policies that mandate coverage requirements for insurance plans, such as requiring first-dollar coverage for preventive services, might be viewed as limiting consumer choice or increasing the cost of insurance premiums.<br><br>It's important to note, however, that the distinction between liberal and conservative viewpoints on healthcare is not always clear-cut, and there may be individuals and groups within each political orientation that hold varying opinions on this issue.	liberal

AAFP	Genital Surgeries in Intersex Children	10/2023	The text you've shared reflects viewpoints that might be associated more closely with liberal perspectives, especially in the context of healthcare and human rights discussions. Here's why:<br><br>1. **Emphasis on Individual Autonomy and Consent**: The focus on delaying genital surgeries until intersex children can consent aligns with liberal values that prioritize individual rights and autonomy. Liberals often advocate for policies that protect the individual's ability to make informed decisions about their bodies and healthcare.<br><br>2. **Opposition to Medically Unnecessary Procedures**: The opposition to medically unnecessary genital surgeries on intersex children without their consent aligns with a liberal emphasis on bodily autonomy and the ethical principle of "do no harm." This perspective challenges traditional medical practices and norms, advocating for a more patient-centered approach.<br><br>3. **Focus on Evidence-Based Medicine**: The call for decisions about surgeries to be based on scientific evidence and the well-being of the patient, rather than societal or cultural norms, reflects a liberal inclination towards evidence-based policy and healthcare.<br><br>4. **Consideration of Mental Health and Patient Satisfaction**: The concern for the potential irreversible effects of surgery, including mental health conditions and patient dissatisfaction, aligns with liberal perspectives that prioritize holistic wellbeing, including both physical and mental health outcomes.<br><br>5. **Promotion of Self-Determination and Self-Advocacy**: The emphasis on promoting self-determination and patient-centered information is consistent with liberal values that stress the importance of empowering individuals to make informed decisions about their lives and healthcare.<br><br>However, it's important to note that the support for the rights and well-being of intersex individuals might also find resonance among some conservatives who prioritize individual rights and autonomy, or who are persuaded by the medical and ethical arguments against unnecessary surgeries. The distinction between liberal and conservative viewpoints can sometimes be fluid, depending on the specific issues and values at stake. The text primarily reflects a human rights and patient-centered approach to healthcare, which transcends simple political categorization but may align more closely with principles typically advocated by liberals in the context of gender, sexuality, and healthcare rights.	liberal
AAFP	Good Samaritan Law	7/2023	The text describes a policy position from the American Academy of Family Physicians (AAFP) regarding legislation that would grant immunity from civil actions for alleged negligence to doctors who provide emergency care without compensation. This stance is primarily centered around legal and healthcare policy rather than aligning clearly with a traditional liberal or conservative viewpoint.<br><br>However, analyzing the policy's potential implications and motivations might suggest a nuanced alignment:<br><br>1. **Conservative Viewpoint**: From a conservative perspective, such legislation could be seen as reducing litigation and potentially lowering healthcare costs by protecting physicians from legal consequences when voluntarily aiding in emergencies. This could align with conservative values of personal responsibility, limiting government intervention (in terms of lawsuits), and fostering volunteerism.<br><br>2. **Liberal Viewpoint**: From a liberal perspective, the focus might be on ensuring that all individuals receive high-quality emergency healthcare without the barrier of potential legal action deterring volunteer medical assistance. This could align with liberal values of comprehensive healthcare access and protection for both patients and healthcare providers in emergency contexts.<br><br>The policy bridges interests that could be supported by both sides for different reasons: conservatives might emphasize the reduction of litigation and regulatory burdens, while liberals might focus on ensuring healthcare access and protections. However, it's important to note that such policy positions can also attract criticism from both sides—liberals might be concerned about accountability and the potential for negligence, while conservatives might be wary of any legislation that could be seen as expanding government influence in healthcare.<br><br>Thus, while the text does not align neatly with either a liberal or conservative viewpoint in a traditional sense, it touches on interests and concerns that are relevant to both political spectrums. The focus is more on practical healthcare policy and legal protection for medical professionals in specific circumstances rather than a broader ideological stance.	neutral

AAFP	Graduate Medical Education Financing Policy	10/2023	The text you've provided outlines a series of principles aimed at reforming Graduate Medical Education (GME) financing and policy to better address the needs of the U.S. healthcare system, particularly in the area of primary care. These principles focus on increasing the number of family medicine residency positions, establishing accountability for federal GME payments, creating new funding collaborations, increasing funding to specific programs like the Teaching Health Center Graduate Medical Education (THCGME) program, modernizing GME financing, and supporting the growth of family medicine residencies through restructuring of funding priorities.<br><br>This text does not strictly align with a single political viewpoint (liberal or conservative) but rather addresses systemic issues in healthcare education and workforce distribution that concern both sides of the political spectrum. However, certain aspects of these principles can be associated more closely with liberal or conservative perspectives:<br><br>1. **Liberal Viewpoint Alignment**:<br>- The emphasis on increasing the number of family medicine residency positions and ensuring a balanced physician workforce aligns with liberal priorities on expanding access to healthcare and primary care services.<br>- Establishing accountability for federal GME payments and advocating for new funding collaborations to address health disparities, social determinants of health, and ensuring equitable healthcare access resonate with liberal values focused on social justice and equity.<br>- Increasing funding to programs like the THCGME and advocating for permanent funding structures for such programs reflect a liberal inclination towards investing in community health and supporting underserved populations.<br><br>2. **Conservative Viewpoint Considerations**:<br>- Some conservatives might support aspects of these principles that emphasize accountability and the efficient use of federal funds, as well as the potential for private sector involvement in funding GME.<br>- The idea of modernizing GME financing to be more evidence-based and transparent can also find support from conservatives who favor reforming government programs to increase efficiency and effectiveness.<br><br>In general, these principles represent a comprehensive approach to improving healthcare workforce training and distribution that transcends simple liberal-conservative dichotomies. They reflect a blend of priorities that include efficiency, accountability, equity, and the strategic use of resources to meet national healthcare needs. The focus on primary care and addressing the maldistribution of healthcare resources can be seen as addressing both conservative interests in efficient and effective use of resources and liberal interests in equity and access to healthcare.	neutral
AAFP	Graduated Driver's License Programs	9/2022	The text you provided outlines a policy position from the American Academy of Family Physicians (AAFP) supporting graduated driver's licensing programs to reduce motor vehicle-related injuries and fatalities. This position does not strictly align with traditional liberal or conservative viewpoints, as it primarily concerns public health and safety measures. However, aspects of the policy could be interpreted through the lens of both political ideologies in the following ways:<br><br>1. **Liberal Viewpoint**:<br>- **Public Safety and Welfare**: The emphasis on graduated licensing, mandatory education, and strict regulations for young drivers could align with liberal principles of using government intervention to ensure public safety and welfare.<br>- **Addressing Discrimination and Accommodations**: The mention of implementing accommodations for drivers with adaptive needs to address discrimination against ableism could resonate with liberal values of inclusivity and equality.<br><br>2. **Conservative Viewpoint**:<br>- **State Variance and Individual Responsibility**: While the policy advocates for a uniform set of minimum requirements, it recognizes the need for variances by state, which could appeal to conservative preferences for state rights and localized control. The emphasis on individual responsibility (e.g., maintaining a clean driving record, zero tolerance for alcohol and drug use) might also find support in conservative circles that value personal accountability.<br><br>In general, policies related to public safety, especially those aiming to protect young drivers, can garner bipartisan support. The specifics of the policy, such as the strictness of the regulations and the balance between state and federal oversight, might be areas where opinions diverge along more ideological lines. However, the focus of this text is on practical measures to improve safety rather than promoting a particular political ideology.	neutral

AAFP	Guidelines on the Supervision of Non-Physician Clinicians (NPCs)	10/2023	The text provided outlines guidelines and perspectives on the role and supervision of non-physician clinicians (NPCs) like physician assistants, nurse practitioners, and nurse midwives in healthcare settings, particularly in relation to family physician practices. This text doesn't neatly align with a liberal or conservative viewpoint in a political sense, as it primarily addresses healthcare practice, management, and regulatory compliance issues.<br><br>However, certain elements of the discussion could be interpreted differently based on one's political viewpoint:<br><br>1. **Support for Team-Based Care and NPCs in Underserved Areas**: Advocating for increased access to healthcare services in underserved communities through innovative staffing models, including NPCs, could be seen as aligning with more progressive or liberal healthcare policies that emphasize healthcare access and equity.<br><br>2. **Emphasis on Physician Supervision and Regulation Compliance**: The insistence on NPCs functioning under the direction and responsible supervision of licensed physicians, even in states where NPCs have independent practice authority, might be viewed as a more conservative approach. This perspective prioritizes traditional medical hierarchy and the role of physicians at the top, possibly reflecting a more cautious approach to expanding healthcare practice roles and scopes.<br><br>3. **Focus on Quality of Care and Patient Safety**: The emphasis on ensuring high-quality care and patient safety through detailed supervision, protocols, and review processes is a universal concern across the healthcare sector, transcending political ideologies. It reflects a commitment to professional standards and patient welfare.<br><br>4. **Legal and Regulatory Compliance**: The detailed discussion on complying with federal, state, and local laws and regulations, as well as the call for transparency in patient care practices (including the role of NPCs), is a pragmatic approach that is necessary for any healthcare practice, regardless of political leanings.<br><br>In summary, while the text addresses issues relevant to healthcare policy and practice, its focus on operational, regulatory, and professional standards makes it more of a practical guide than a political statement. The perspectives on healthcare access, team-based care, and regulatory compliance can intersect with broader political debates on healthcare policy, but the text itself is not explicitly partisan.	neutral
AAFP	Gun Violence, Prevention of (Position Paper)	7/1905	The text provided aligns more closely with a liberal viewpoint for several reasons:<br><br>1. **Public Health Approach**: It advocates for treating gun violence as a national public health epidemic, emphasizing the need for research and evidence-based strategies to reduce gun-related morbidity and mortality. This approach is typically more associated with liberal perspectives that focus on systemic and preventive measures rather than punitive or deregulatory approaches favored by conservative viewpoints.<br><br>2. **Research and Funding**: The text calls for federal funding for research into gun violence and effective prevention strategies. Historically, liberal policymakers have been more likely to support the idea of using government resources to study and address gun violence, whereas conservative viewpoints often resist increased government spending on issues they see as linked to individual rights or freedoms, such as gun ownership.<br><br>3. **Regulation and Legislation**: The text advocates for constitutionally appropriate restrictions on firearms, including the manufacturing and sale of large-capacity magazines and firearms with features designed to increase their killing capacity. It also suggests implementing policies like background checks and safe storage laws. Such regulatory measures are more commonly supported by liberal viewpoints, which prioritize the reduction of gun violence over broader interpretations of the Second Amendment rights emphasized by conservative perspectives.<br><br>4. **Multi-disciplinary Collaboration**: The emphasis on a multi-disciplinary approach that includes healthcare providers, community programs, and legislative action reflects a holistic view of public health that is more aligned with liberal philosophies. These philosophies often advocate for comprehensive solutions involving various stakeholders to address societal issues.<br><br>5. **Constitutional Rights Respect**: While it supports gun control measures, the text also acknowledges the importance of respecting the Constitutional right to bear arms. This nuance is characteristic of liberal positions that seek to balance individual freedoms with public safety and health concerns.	liberal

			In summary, the text's focus on research, preventive measures, regulation, and a public health approach to gun violence, along with its advocacy for specific policy actions, aligns more closely with liberal viewpoints that prioritize societal health and safety within the framework of respecting constitutional rights.	
AAFP	Hate Crimes	7/1905	The text reflects a viewpoint that aligns more closely with liberal ideologies, particularly in its emphasis on protecting vulnerable groups, advocating for anti-discrimination and hate crime laws, and focusing on the health needs of hate crime survivors. Liberal viewpoints often prioritize social justice, equality, and the protection of civil rights for all individuals, including those in protected classes such as race, color, religion, gender, sexual orientation, and disability status.<br><br>The support for the development and implementation of anti-discrimination and hate crime laws is consistent with a liberal approach to ensuring that individuals are protected from violence and discrimination based on their identity. Additionally, the focus on research and educational programs aimed at preventing hate crimes and promoting interventions to address the health needs of survivors indicates a proactive approach to social issues, which is a hallmark of liberal ideologies.<br><br>Conservative viewpoints, on the other hand, may also support the rule of law and the protection of individuals from crime but might place a different emphasis on how to address the issues of hate crimes and discrimination, often focusing more on individual responsibility and less on systemic changes or expansive government intervention.<br><br>It's important to note that the categorization of viewpoints as strictly "liberal" or "conservative" can sometimes oversimplify complex positions that individuals or organizations may hold. However, based on the emphasis on anti-discrimination laws, protection of vulnerable groups, and a proactive approach to social justice, the text you've provided aligns more closely with a liberal viewpoint.	liberal
AAFP	Health Benefits	7/2023	The statement provided aligns more closely with a liberal viewpoint, as it emphasizes equality and inclusivity in health benefits for all individuals, reflecting liberal values of social equality, inclusiveness, and support for equal rights across different groups within society. Liberal perspectives often advocate for policies and practices that aim to ensure equitable access to resources and rights, including healthcare, regardless of family structure or other social determinants.<br><br>Conservative viewpoints, on the other hand, might emphasize traditional family structures and personal responsibility in accessing healthcare, potentially focusing less on systemic approaches to equality and inclusivity. However, it's important to note that views on healthcare access can vary widely within both liberal and conservative spectrums, and this assessment is a generalization rather than a definitive classification of the statement.	liberal

AAFP	Health Care Costs, Methods for Reducing	2019	The text presents a viewpoint that combines elements typically associated with both liberal and conservative perspectives in the context of healthcare policy. Here's a breakdown of how different parts align with each ideology:<br><br>1. **Liberal Viewpoint Elements:**<br>- **Emphasis on Health Promotion and Disease Prevention:** Advocating for the training of family physicians who focus on health promotion and disease prevention to avoid costly hospital care aligns with liberal viewpoints. Liberals often support preventative care as a means to improve public health outcomes and reduce overall healthcare costs.<br>- **Public Education on Health Responsibilities:** The focus on educating the public about the importance of exercise, nutrition, and safety, as well as the risks of unhealthy behaviors, reflects a proactive approach to health that is generally supported across the political spectrum but aligns well with liberal emphasis on public health initiatives.<br><br>2. **Conservative Viewpoint Elements:**<br>- **Concern Over Medical Liability and Lawsuits:** The focus on medical liability risk management and the effort to reduce medical liability suits is often associated with conservative viewpoints. Conservatives typically advocate for tort reform as a way to lower healthcare costs by reducing the burden of malpractice insurance and litigation on healthcare providers.<br>- **Emphasis on Individual Responsibility for Health and Costs:** The call for public education that emphasizes the individual's responsibility for personal health and healthcare costs aligns with conservative values that prioritize personal accountability and self-reliance.<br>- **Quality Control Over Cost Containment:** Advocating for quality control in healthcare regulation rather than purely focusing on cost containment can be seen as aligning with a conservative approach that values the quality of care and the autonomy of healthcare providers over government-imposed cost controls.<br><br>3. **Bipartisan or Neutral Elements:**<br>- **Advocacy for the Patient:** The commitment to representing and advocating for patients transcends political ideologies and is a core principle of medical ethics.<br>- **Self-Imposed Cost Containment by Physicians:** Encouraging physicians to practice cost-effective care while maintaining high quality can be seen as a pragmatic approach that appeals across the political spectrum.<br><br>Overall, the text integrates approaches that focus on individual responsibility and market-driven reforms (more conservative) with those that emphasize preventative care and public health education (more liberal). This blend suggests a comprehensive approach to healthcare policy that seeks to balance quality, cost-efficiency, and individual accountability.	neutral
AAFP	Health Care Facility Visitation Rights of Patients	1/2022	The text you've shared outlines a policy position by the American Academy of Family Physicians (AAFP) that supports the rights of patients to full and equal visitation rights, emphasizing that these rights should be consistent with the patient's wishes, supported by advance healthcare directives, and not more restrictive than those afforded to immediate family members. It also mentions the importance of considering potential intimate partner violence or other forms of abuse.<br><br>This position aligns more closely with a liberal viewpoint for several reasons:<br><br>1. **Support for Patient Autonomy and Rights**: Liberal perspectives often emphasize individual rights, autonomy, and the importance of ensuring equal access to rights and services for all individuals. By advocating for full and equal visitation rights for patients, the AAFP's position reflects a commitment to these principles.<br><br>2. **Inclusivity**: The stance on visitation rights not being more restrictive than those of immediate family members can also be seen as an effort to be inclusive of diverse family structures and relationships, which is a value more commonly associated with liberal ideologies. This inclusivity is important in contexts where non-traditional relationships (such as those involving unmarried partners, same-sex partners, or close friends) may not always be recognized or given equal consideration in healthcare settings.<br><br>3. **Consideration of Vulnerable Populations**: The mention of taking consideration if there is a suspicion of intimate partner violence or other forms of abuse demonstrates a concern for the protection of vulnerable populations. Liberal viewpoints often prioritize the protection and support of individuals in vulnerable situations, advocating for policies that seek to identify and mitigate risks to their well-being.<br><br>It's important to note, however, that support for patient rights and protections against abuse can also be found across the political spectrum. The focus on patient autonomy, inclusivity, and protection may resonate with individuals regardless of their political	liberal

			affiliation, even if the approach or emphasis might differ.<br><br>The AAFP's position as described reflects a principled stance on healthcare rights and patient care that prioritizes individual wishes, inclusivity, and safety, which are values often emphasized in liberal viewpoints.	
AAFP	Health Care for All: A Framework for Moving to a Primary Care-Based Health Care System in the United States	2018	The text provided aligns more closely with a liberal viewpoint, primarily due to its emphasis on universal health care coverage, the right to health as a basic human right, and the focus on comprehensive and longitudinal primary care. It advocates for a variety of health system reform options, including single-payer systems, public options, and expansions of existing public programs like Medicare and Medicaid, which are typically supported by liberal or progressive political ideologies. The document also underscores the importance of addressing social determinants of health, insurance reforms for consumer protections, and increasing investments in primary care—all of which are often associated with liberal policy positions focused on equity, access to care, and public investment in health services.<br><br>The advocacy for universal health care coverage based on the principle that health is a basic human right, along with the call for significant reform to the U.S. health care system to ensure that everyone has access to necessary health services, reflects a vision for a more inclusive, equitable health care system. This vision contrasts with conservative viewpoints, which often emphasize market-based solutions, limited government intervention in health care, and personal responsibility for health care decisions and costs.<br><br>Moreover, the document's support for policy changes such as insurance reforms, increased primary care investment, and addressing the social determinants of health aligns with liberal approaches to health care reform that prioritize comprehensive care, prevention, and the overall well-being of the population. These perspectives are generally supportive of more active government roles in health care to ensure coverage, affordability, and quality of care for all individuals.	liberal

AAFP	Health Care is a Right	1/2022	The text you've provided generally aligns more closely with a liberal viewpoint, particularly in the context of American politics. Here are the reasons why:<br><br>1. **Health as a Basic Human Right**: The statement that health is a basic human right for every person, regardless of their background or status, is a principle more commonly associated with liberal or progressive ideologies. Conservatives might also support the idea of healthcare for all, but the emphasis on it as a "right" is more typical of liberal perspectives.<br><br>2. **Universal Access**: The call for universal access to timely, high-quality, and affordable healthcare services is a hallmark of liberal healthcare policy objectives. This approach often involves a significant role for government in ensuring access to healthcare, which aligns with liberal views on the role of government in addressing social issues.<br><br>3. **Support for the Uninsured**: The encouragement for healthcare providers to offer care without charge or at reduced charges to uninsured patients reflects a commitment to healthcare equity, a priority more commonly found in liberal ideologies. This approach suggests an active effort to reduce healthcare disparities, which is a key issue for many liberals.<br><br>4. **Inclusivity**: The explicit inclusion of diverse groups, such as those of different races, religions, genders, or sexual orientations, in the statement of rights to health reflects a liberal emphasis on inclusivity and social justice.<br><br>While these points align more closely with liberal viewpoints, it's important to recognize that there can be crossover in some areas, and not all conservatives or liberals may fully agree with every aspect as outlined. Political beliefs are complex and can vary widely among individuals within the same general ideological category.	liberal
AAFP	Health Care Professionals, Family Physician Training With	9/2019	The text provided outlines an educational approach for family medicine graduates that emphasizes the importance of interprofessional collaboration to achieve the Quadruple Aim in healthcare: improving population health, enhancing patient care experience, reducing healthcare costs, and improving the work life of healthcare providers. This approach is centered on training residents to work effectively within interprofessional teams, including a wide range of healthcare professionals, to deliver patient- and population-centered care that is safe, timely, efficient, effective, and equitable.<br><br>This perspective generally aligns more closely with a liberal viewpoint, particularly in its emphasis on comprehensive, collaborative, and equitable healthcare delivery. The focus on interprofessional education, team-based care, and the Quadruple Aim reflects values often associated with liberal perspectives, such as inclusivity, holistic and preventative care, and the goal of reducing healthcare disparities. Additionally, the emphasis on improving the work life of clinicians and staff, and ensuring that care delivery is patient-centered and equitable, aligns with liberal values that prioritize worker well-being and equal access to quality healthcare.<br><br>However, it's important to note that the principles of interprofessional education and collaborative practice are not inherently political and can be supported across the political spectrum. The focus on efficiency, effectiveness, and reducing healthcare costs can also appeal to conservative viewpoints that prioritize fiscal responsibility and efficiency in healthcare delivery. The emphasis on specific roles and qualifications, such as the stipulation that only physicians who have completed specific residency programs may use the designation of family physician, might resonate with conservative values regarding professional standards and accountability.<br><br>In summary, while the text leans towards a liberal viewpoint due to its emphasis on collaboration, equity, and comprehensive care, the principles of improving healthcare delivery through interprofessional education and practice can find support across the political spectrum.	liberal
AAFP	Health Education	5/2023	The text provided appears to be a statement about the importance of health education and its role in disease prevention and health management, rather than a political or ideological viewpoint. It doesn't explicitly align with either liberal or conservative viewpoints as it focuses on the role of healthcare professionals in educating their patients and the public about health. The emphasis on education and empowerment for better health outcomes is a principle that can be supported across a broad spectrum of political and ideological beliefs.<br><br>The American Academy of Family Physicians (AAFP) is a professional association that represents family physicians, family medicine residents, and medical students. The statement underscores the organization's commitment to health education as a critical component of healthcare, aiming to improve patient outcomes and public health. This perspective is generally in line with the healthcare community's consensus on the importance of patient education for disease prevention and health management, rather than being tied to a specific political ideology.	neutral

AAFP	Health Education in Schools	1/2022	The text outlines a comprehensive approach to health education from grades K through 12, emphasizing the importance of providing accurate information on a wide range of health topics, developing critical thinking and decision-making skills, understanding the impact of family health history, and promoting personal and social responsibility towards health. This perspective aligns more closely with a liberal viewpoint for several reasons:<br><br>1. **Emphasis on Comprehensive Health Education:** The focus on a broad spectrum of health topics, including sensitive subjects such as sexual health, mental health, and substance abuse, reflects a liberal inclination towards education that is inclusive, comprehensive, and aimed at preparing students to navigate complex societal issues.<br><br>2. **Promotion of Critical Thinking and Personal Responsibility:** The emphasis on developing critical thinking, decision-making skills, and personal responsibility aligns with liberal values of empowerment and individual autonomy, encouraging students to make informed choices about their health and well-being.<br><br>3. **Social Responsibility:** The call for students to develop a sense of social responsibility and participate in the promotion of health education to their peers, family, and community suggests a community-oriented perspective that is often associated with liberal ideologies, which value collective well-being and support for communal health initiatives.<br><br>4. **Inclusion and Accessibility:** The support for continued health education in the community through adult education programs indicates a commitment to lifelong learning and ensuring that health education is accessible to all members of the community, reflecting liberal values of inclusivity and equity.<br><br>Overall, while health education itself can be a non-partisan issue, the comprehensive and inclusive approach outlined in the text, along with its focus on empowerment, social responsibility, and accessibility, tends to align more closely with liberal viewpoints, which prioritize these values in education and public policy.	liberal
AAFP	Health Equity	7/1905	The text provided does not explicitly align with a traditional "liberal" or "conservative" viewpoint as those terms are often used in a political context. Instead, it focuses on public health principles and the promotion of health equity, which involves addressing social determinants of health, and working towards the highest level of health for all people. These principles are concerned with improving healthcare access, addressing disparities, and considering a broad range of factors that impact health, which can be supported by individuals across the political spectrum.<br><br>However, the emphasis on health equity, addressing social determinants of health, and collaboration with various entities, including government and social service providers, to affect positive change might be more closely associated with viewpoints that prioritize social justice, public health, and comprehensive healthcare policies. These are often (but not exclusively) associated with more progressive or liberal policy approaches, which tend to emphasize the role of societal structures in individual and community health outcomes and support interventionist policies to address inequalities and disparities.<br><br>It's important to recognize that public health and healthcare policy can transcend traditional political divides, with individuals and organizations across the political spectrum recognizing the importance of addressing health disparities and promoting the well-being of all community members. The focus of the American Academy of Family Physicians (AAFP) as described in the text is on the broad and inclusive goal of achieving the highest possible level of health for everyone, which is a universal concern that can be approached from various policy perspectives.	probably liberal
AAFP	Health Impacts of Immigration	7/1905	The text provided aligns more closely with a liberal viewpoint. Liberal perspectives generally emphasize the importance of human rights, equality, and access to essential services for all individuals, regardless of their immigration status. The emphasis on ensuring that immigrant and refugee persons, whether documented or undocumented, have access to vital health care services without compromising their rights reflects a commitment to these principles. The support for privacy protections, timely healthcare access in detention facilities, measures to reduce stress associated with detention and deportation threats, and appropriate payment for physicians caring for immigrant persons and their families are all positions that typically align with liberal values of inclusivity, health care as a human right, and the protection of vulnerable populations. Conservative viewpoints might focus more on the enforcement of immigration laws and may prioritize the rights of citizens and legal residents in discussions about resource allocation and access to services.	liberal

AAFP	Health in All Policies	1/2022	The text you've provided aligns more closely with a liberal viewpoint. It emphasizes the importance of integrating health considerations into all areas of policy to address social determinants of health (SDoH), promote health equity, and ensure that health outcomes are considered in the legislative process. This approach reflects a focus on systemic change, inclusivity, and the use of scientific evidence and community input to inform policy decisions, which are principles often associated with liberal perspectives.<br><br>The Health in All Policies (HiAP) approach, as described, advocates for collaborative and cross-sectoral efforts to improve population health and sustainability. It emphasizes the need for an inclusive policymaking process that considers the impacts of various policies on health outcomes, especially for communities experiencing disparities. This comprehensive and preventive approach to public health is typically more aligned with liberal ideologies, which prioritize social justice, equity, and the role of government in addressing societal issues.<br><br>Conservative viewpoints, on the other hand, might prioritize individual responsibility for health, limited government intervention in private sectors, and might be more skeptical of broad, cross-sectoral government approaches to public health. Conservatives may support health policies that focus on market-based solutions, personal choice, and less regulatory oversight.<br><br>It's important to note that these generalizations do not apply to all individuals or to all policy positions within either liberal or conservative camps. Political beliefs are complex and can vary widely among individuals.	liberal
AAFP	Health Literacy	2020	The text you provided focuses on the concept of health literacy and emphasizes the importance of improving the capacity of individuals to understand health information and make informed health care decisions. The advocacy for health literacy by the American Academy of Family Physicians, including strategic approaches to healthcare system design, research, quality improvement, and education of medical professionals, does not align strictly with a conservative or liberal viewpoint in the political sense.<br><br>The focus on health literacy is fundamentally a public health issue, aiming to improve patient outcomes, enhance the quality of care, and ensure equitable access to health information and services. Both conservative and liberal perspectives may support the goal of health literacy, but they might differ in their approaches to achieving it. For example:<br><br>- **Liberal Viewpoint**: Might emphasize the role of government and public health programs in promoting health literacy, advocating for policies that ensure access to education and health services for all, especially underserved populations.<br><br>- **Conservative Viewpoint**: Might focus on individual responsibility and the role of private sector initiatives in promoting health literacy, potentially advocating for market-based solutions to improve healthcare quality and access.<br><br>However, the text itself is neutral and aimed at improving healthcare outcomes through education and system improvements. It does not explicitly advocate for policy changes or government interventions that are typically associated with political ideologies. Instead, it underscores the importance of health literacy as a critical component of patient care and healthcare system effectiveness, which can be a point of consensus across the political spectrum.	neutral

AAFP	Healthy Foods	10/2023	The text provided aligns more closely with a liberal viewpoint for several reasons:<br><br>1. **Support for Social Welfare Programs**: The text advocates for the continued development of systems that promote equitable access to affordable and nutritious foods, particularly emphasizing support for populations vulnerable to food insecurity. The endorsement of federal programs like SNAP (Supplemental Nutrition Assistance Program) and WIC (Special Supplemental Nutritional Program for Women, Infants, and Children), as well as child nutrition programs like the Summer Food Service Program (SFSP) and the National School Lunch/Breakfast Programs, reflects a liberal perspective that often prioritizes government intervention and support for disadvantaged populations.<br><br>2. **Focus on Eliminating Disparities**: The call for sustained funding for evidence-based policies and programs aimed at eliminating disparities in healthy food access underscores a commitment to social equity, a principle commonly associated with liberal ideologies. This focus on reducing inequalities aligns with liberal priorities of addressing social determinants of health and ensuring that all individuals, regardless of their socio-economic status, have access to the resources needed for a healthy life.<br><br>3. **Emphasis on Evidence-based, Culturally Competent Approaches**: The advocacy for nutrition guidelines that are evidence-based and culturally competent reflects a liberal inclination towards science-based policy making and inclusivity. This perspective values diversity and the importance of tailoring health initiatives to be respectful and relevant to various cultural backgrounds.<br><br>4. **Holistic View of Health**: The recognition of the role family physicians play in counseling patients on nutrition and connecting individuals to community resources indicates a holistic approach to healthcare. This approach, which considers a wide variety of foods for a healthful diet and the potential of healthy foods to manage chronic health conditions, aligns with liberal viewpoints that often emphasize preventive care and the broader determinants of health.<br><br>Overall, the text's emphasis on equitable access to resources, support for vulnerable populations, and reliance on evidence-based, inclusive policies is more characteristic of liberal ideologies, which prioritize social welfare, equity, and the role of government in addressing societal challenges.	liberal
AAFP	Healthy Nutrition in Health Care Facilities and Other Workplaces	10/2023	The text provided aligns more closely with viewpoints that are often associated with liberal or progressive ideologies, rather than conservative ones, based on its emphasis on the following aspects:<br><br>1. **Public Health and Preventive Care**: The focus on promoting healthy behaviors, affordable access to healthy foods, and education on nutrition aligns with a liberal perspective that prioritizes public health initiatives and preventive care as means to improve overall health outcomes and reduce healthcare costs.<br><br>2. **Environmental Considerations**: The mention of using local, environmentally friendly food and packaging reflects a concern for sustainability and environmental health, which is more commonly associated with liberal viewpoints that prioritize environmental protection and sustainability efforts.<br><br>3. **Workplace Wellness Programs**: The emphasis on extending high-quality nutrition to all workplaces and supporting policies to provide healthy food options can be seen as part of a broader liberal agenda that seeks to improve worker welfare and public health through institutional policies.<br><br>Conservative viewpoints, on the other hand, might prioritize individual choice and responsibility over institutional policies promoting specific health behaviors, and might be more cautious about the potential costs and regulatory implications of such comprehensive health and environmental initiatives. However, it's important to note that public health and wellness can be areas of bipartisan agreement, and the emphasis on practical, preventive care measures can also resonate across the political spectrum, albeit with different approaches to implementation.<br><br>This interpretation is generalized and may not apply universally, as individuals and groups within each political ideology can hold a wide range of views on specific issues.	liberal
AAFP	Hearing Loss, Deafness, and the Hard-of-Hearing	10/2023	The text provided does not align specifically with a liberal or conservative viewpoint in the context of American political ideologies. Instead, it reflects a professional and inclusive approach to healthcare, focusing on accessibility and accommodation for patients with deafness and hearing loss. The recommendations are aimed at improving healthcare delivery and patient care for a specific group of patients by ensuring they have equal access to communication and services. Such measures are generally in line with principles of accessibility, inclusiveness, and healthcare equity, which can be supported across a broad spectrum of political and	neutral

			social beliefs. These recommendations prioritize the needs of patients with hearing impairments, advocating for practices that ensure their effective participation in their healthcare.	
AAFP	Helmet Laws	7/1905	The text you've provided is a policy position from the American Academy of Family Physicians (AAFP) advocating for the use of helmets across various modes of transportation and recreational activities, and for legislation that supports helmet use. It also acknowledges the benefits of bike and scooter share programs but notes the issue of helmets not being readily available through these services.<br><br>This position doesn't neatly align with traditional liberal or conservative viewpoints as defined in a political sense. Instead, it reflects a public health perspective focused on injury prevention and safety. Public health recommendations like these are generally based on evidence and research aimed at reducing harm and preventing injuries, rather than being motivated by political ideologies.<br><br>However, the support for legislation requiring helmet use could be interpreted differently depending on one's political viewpoint:<br>- **From a liberal perspective**, advocating for mandatory helmet laws might be seen as a government's responsibility to protect public health and safety, which aligns with a more regulatory approach to governance.<br>- **From a conservative perspective**, some might view such mandates as an overreach of government authority, preferring personal responsibility and freedom of choice over compulsory regulations.<br><br>The emphasis on public health and safety, along with support for legislative measures to enforce helmet use, might be more readily associated with policy approaches that prioritize communal well-being and regulatory interventions. However, the primary intent here is to enhance safety and prevent injuries, transcending simple liberal or conservative categorization.	neutral
AAFP	Home Diagnostic Test Kits	7/1905	The text you provided is a neutral statement from the American Academy of Family Physicians (AAFP) regarding home diagnostic test kits. It neither explicitly aligns with a liberal nor conservative viewpoint. The statement focuses on the recognition of the increase in home diagnostic tests for various diseases and conditions and emphasizes the importance of consulting with physicians for the selection, use, and interpretation of these tests. This guidance aims at ensuring patients use these tests effectively and safely, and it reflects a concern for patient care and the physician-patient relationship rather than a political stance.	neutral
AAFP	Home Health Care	2020	The text you've provided appears to be a neutral description of home health care and its importance, rather than aligning explicitly with a liberal or conservative viewpoint. It focuses on the role of family physicians in providing and managing home health care services, the necessity of their involvement from the initial decision-making to the subsequent planning and provision of these services, and the importance of adequate compensation for physicians to ensure ongoing access to home health care.<br><br>There are elements within the text that could be supported by both liberal and conservative perspectives, depending on the broader context:<br><br>- **Liberal viewpoint**: Might focus on the importance of accessible and comprehensive healthcare for all, including the availability of home health care services as part of a broader healthcare system that aims to be inclusive and supportive of patients' needs in various settings. Advocates from a liberal perspective might emphasize the role of government and insurance programs in providing adequate compensation to ensure these services are accessible to everyone, reflecting a commitment to healthcare as a right.<br><br>- **Conservative viewpoint**: Might appreciate the emphasis on family physicians and the continuity of care within the family context, reflecting values of personal responsibility and the importance of family in healthcare decisions. Conservatives might also support the idea of adequate compensation for physicians as a market-driven solution to ensure the quality and availability of home health care services, emphasizing the role of private insurance and market mechanisms in healthcare.<br><br>The text itself does not advocate for a specific political or economic model for the provision and funding of home health care services, which makes it more of a neutral description of the importance of family physicians in home health care. It emphasizes professional qualifications, the continuity of care, and the need for appropriate compensation without delving into the political debates over how healthcare should be structured, funded, or accessed.	neutral

AAFP	Homelessness	10/2023	The text aligns more closely with a liberal viewpoint. It advocates for the Housing First approach, which is a progressive policy aimed at addressing homelessness by providing immediate access to housing without preconditions, such as sobriety or participation in treatment programs. This model emphasizes the importance of stable housing as a fundamental right and a crucial element of healthcare, recognizing that access to safe and affordable housing significantly impacts one's health outcomes. The focus on social determinants of health, integrated healthcare services, and support for individuals experiencing homelessness reflects a holistic and compassionate approach to public health and social welfare issues, which are hallmarks of liberal perspectives.<br><br>Conservative viewpoints, on the other hand, might emphasize personal responsibility, conditional support, and a more limited role for government in addressing social issues, potentially favoring approaches that include preconditions for receiving housing or prioritizing economic incentives for private sector solutions to homelessness.<br><br>The American Academy of Family Physicians (AAFP)'s support for Housing First programs and the emphasis on the intersection of housing and healthcare demonstrate a commitment to addressing systemic issues through comprehensive, integrated support services, aligning with principles typically associated with liberal policies.	liberal
AAFP	Hospice Care	1/2022	The text you've provided outlines a definition and perspective on hospice care, emphasizing its multidisciplinary, comprehensive nature and the pivotal role of family physicians in leading hospice care teams. This perspective focuses on the medical, emotional, and spiritual support provided to patients at the end of their lives, as well as the importance of continuous patient relationships.<br><br>This viewpoint does not neatly align with traditional political labels such as "liberal" or "conservative." Instead, it addresses healthcare delivery, particularly end-of-life care, in a way that transcends simple political categorization. It advocates for a holistic, patient-centered approach to palliative care, which is a principle that can be supported across the political spectrum.<br><br>Discussions about healthcare, including hospice care, can indeed involve political beliefs, particularly regarding funding, access, and the extent of government involvement in healthcare. However, the specific focus on multidisciplinary care and the role of family physicians in hospice care is more closely related to medical best practices and patient care philosophies than to partisan politics.<br><br>In the broader context, individuals or groups may interpret and advocate for aspects of hospice care in ways that reflect their political ideologies, such as debates over healthcare policy, insurance coverage, and the role of government in healthcare. However, the text you've provided is centered around the structure and philosophy of care rather than explicit political arguments or policy prescriptions.	neutral
AAFP	Hospital Medical Staff and Other Health Care Organizations, Board Recertification	7/1905	The text you've provided focuses on a policy regarding the requirements for medical staff membership in terms of board certification and its uniform application across different specialties. This policy does not explicitly align with a liberal or conservative viewpoint as it is more concerned with professional standards and equity within the medical field rather than broader political ideologies.<br><br>Liberal and conservative viewpoints typically diverge on issues related to government intervention, individual freedoms, economic policies, and social values. The content of this text, however, addresses an administrative and professional practice within healthcare organizations, aiming for fairness and consistency in the qualifications of medical staff. It emphasizes the importance of applying the same certification standards to all medical professionals, regardless of their specialty, which is a matter of professional governance rather than political ideology.<br><br>Thus, the statement is more about ensuring a high standard of care and professional equity rather than reflecting a specific political or ideological stance.	neutral
AAFP	Hospital Medical Staff, Board Certification for Membership	7/1905	Medical staff membership and hospital privileges should be granted on the basis of the individual physician's documented training and/or experience, demonstrated abilities, and current competence. All medical staff members should recognize that overlap occurs among specialties and that no one department “owns” or has exclusive rights to any particular privileges.<br><br>The American Academy of Family Physicians is opposed to the use of specialty board certification as the sole or an exclusionary criterion in determining medical staff membership. (1990) (2019 COD)	neutral

AAFP	Hospital Medical Staff, Liaison Between Governing Boards and	10/2023	The text you've shared reflects a professional and organizational standpoint rather than a direct alignment with liberal or conservative political viewpoints. The American Academy of Family Physicians (AAFP) is advocating for the involvement of physicians in hospital governance, emphasizing the importance of having medical professionals on the boards of hospitals to serve as effective liaisons between the medical staff and the governing bodies of hospitals. This stance is based on the belief that physicians, due to their medical expertise and understanding of patient care needs, can contribute significantly to decision-making processes that affect hospital operations and patient care.<br><br>This position is more about advocating for the representation of medical professionals in hospital governance and ensuring that medical staff have a voice in decisions that affect patient care and hospital operations. It does not directly address or align with the broader social, economic, or political ideologies typically associated with liberal or conservative viewpoints. Instead, it focuses on professional representation, healthcare management, and the improvement of healthcare delivery, which are concerns that cross political boundaries and can be supported by individuals across the political spectrum.	neutral
AAFP	Hospital Use of Infant Formula in Breastfeeding or Chestfeeding Infants	2022	The text provided outlines a position from the American Academy of Family Physicians (AAFP) regarding breastfeeding, chestfeeding, and the use of formula for infants. This stance emphasizes support for breastfeeding/chestfeeding as the preferred method of infant feeding and suggests limiting the promotion and distribution of formula in healthcare settings to encourage this practice. The AAFP also advocates for the use of donor human milk as a supplement when necessary and for providing resources for lactation assistance to parents who choose to breastfeed/chestfeed exclusively.<br><br>This position aligns more closely with viewpoints typically associated with liberal ideologies, which often emphasize public health policies that support natural and holistic approaches to healthcare, including breastfeeding. Liberal perspectives generally advocate for policies that aim to provide comprehensive support for child-rearing practices that are considered beneficial for the health and well-being of both the child and the mother. The emphasis on breastfeeding and chestfeeding, and the caution against the indiscriminate distribution of formula, reflect a preference for evidence-based practices in maternal and child health, which is a common feature of liberal health policy.<br><br>Conservative viewpoints, on the other hand, might focus more on individual choice and the role of free markets in healthcare, including the availability and marketing of infant formula. While not necessarily opposed to breastfeeding, conservative policies might be less prescriptive about infant feeding practices and more supportive of parental autonomy in making decisions about whether to breastfeed, chestfeed, or use formula.<br><br>It's important to note, however, that opinions on infant feeding practices can vary widely within both liberal and conservative circles, and the issue does not neatly fit into the traditional political spectrum. The support for breastfeeding and chestfeeding is widespread across various political and social groups, driven by the consensus on its health benefits for most infants and mothers. The AAFP's position is primarily rooted in health considerations rather than political ideology, reflecting the medical consensus on the benefits of breastfeeding/chestfeeding and the appropriate use of formula as a supplement when necessary.	liberal
AAFP	Hospitalists Trained in Family Medicine	7/1905	The text provided appears to focus on the qualifications, training, and roles of hospitalists and family physicians within the healthcare system, specifically regarding the care of hospitalized patients. It emphasizes the importance of education, competence, and a comprehensive approach to medical care that includes coordination across different care settings, quality improvement, and addressing patients' psychosocial needs.<br><br>This text does not directly align with a distinctly liberal or conservative political viewpoint as it primarily addresses medical education, professional standards, and patient care practices within the healthcare field. It advocates for inclusivity and competence in the hospitalist role, emphasizing the broad and comprehensive training of family physicians and their capability to provide care in hospital settings. These are professional and educational standards in medicine rather than politically charged issues.<br><br>The focus on inclusivity, comprehensive care, and interdisciplinary teamwork is consistent with a general ethos of ensuring quality healthcare and could be supported by individuals across a wide political spectrum. The emphasis on education and training aligns with a universal principle in healthcare professionalism: ensuring that all practitioners are well-qualified to provide the best possible care to their patients.<br><br>Thus, the text is more about professional standards and healthcare quality than about aligning with a specific political ideology.	neutral

AAFP	Hospitals, Transfer of Patients	9/2022	The text presents a viewpoint that emphasizes patient welfare, the importance of physician and patient (or their family) consultation in medical decisions, and the minimization of financial barriers in the transfer of patients between institutions. It also highlights the necessity of adherence to federal law and the importance of communication and information sharing through technology.<br><br>This perspective aligns more closely with **liberal** viewpoints, particularly in its emphasis on reducing financial barriers to healthcare and ensuring that patient care decisions are made based on health needs rather than economic factors. Liberals often advocate for policies that aim to make healthcare accessible and affordable for all, regardless of financial status, and support the involvement of patients and their families in healthcare decisions. The stress on compliance with federal laws and the use of current technology for information sharing also reflects a progressive approach to healthcare, prioritizing transparency, patient rights, and the effective use of technology.<br><br>Conservative viewpoints, on the other hand, might focus more on market-driven solutions to healthcare, personal responsibility for health care decisions, and less government involvement in healthcare transactions. While conservatives also value patient care, their approach might emphasize different mechanisms for achieving healthcare goals, such as private health insurance markets and minimizing regulatory burdens on providers.<br><br>However, it's important to note that healthcare is a complex issue that can transcend traditional liberal-conservative dichotomies, and individuals with conservative views might also support aspects of the approach outlined in the text, especially those related to physician autonomy and patient-physician decision-making.	liberal
AAFP	Human Trafficking	10/2023	The text provided outlines a position on human trafficking that emphasizes the importance of awareness, education, and a multi-disciplinary approach to identify, manage, and prevent human trafficking. This stance is largely humanitarian and focuses on the health and welfare of individuals affected by human trafficking. It advocates for training health professionals to recognize and assist victims, calls for research, and encourages collaboration among various agencies and organizations to combat human trafficking.<br><br>This viewpoint does not align strictly with either a liberal or conservative political ideology in the traditional sense. Instead, it addresses human trafficking as a serious human rights and public health issue. The emphasis on compassionate care, education, and the involvement of healthcare professionals in identifying and helping victims can be seen as aligning with broader social justice and public health perspectives, which are often associated with liberal ideologies. However, the call for collaborative efforts and the recognition of human trafficking as a critical issue affecting individuals across the globe and within the U.S. can resonate with values across the political spectrum, including those held by conservatives who prioritize law and order and the protection of vulnerable populations.<br><br>In summary, the text's focus on human trafficking transcends simple political categorization, embodying a humanitarian perspective that advocates for the dignity, health, and welfare of individuals, which are concerns that can and do cross political lines.	neutral

AAFP	Hydraulic Fracturing (Fracking): Health Effects and Disclosure of Proprietary Information	1/2022	The text provided presents a viewpoint that aligns more closely with liberal perspectives, especially in the context of environmental policy and public health. Here are the key reasons why it aligns with a liberal viewpoint:<br><br>1. **Emphasis on Environmental and Public Health Risks:** The text highlights the negative impacts of hydraulic fracturing (fracking) on the environment and public health, including the contamination of water sources and soil, and the association with serious health effects like asthma and cancer. Liberal perspectives often prioritize environmental protection and public health over the interests of industry.<br><br>2. **Concern for Marginalized Communities:** The text notes the disproportionate impact of fracking on marginalized communities, communities of color, and communities with lower incomes. This focus on social justice and equity is a hallmark of liberal ideologies, which advocate for policies that protect vulnerable populations from environmental and economic harms.<br><br>3. **Support for Transparency and Regulation:** The text opposes legislation that protects proprietary information about the chemicals used in fracking and limits the ability of communities to regulate or restrict fracking. Liberals generally support greater transparency in corporate practices and the regulation of activities that pose risks to the environment and public health.<br><br>4. **Advocacy for Independent Research:** The call for rigorous independent research into the effects of fracking and opposition to legislation that restricts the disclosure of chemical information align with a liberal viewpoint that values science and evidence-based policy-making.<br><br>5. **Opposition to Preemption Legislation:** The opposition to preemption in state law that limits local communities' ability to regulate and restrict fracking reflects a liberal perspective that supports local control and decision-making, particularly in matters affecting local environments and health.<br><br>Overall, the text's focus on environmental protection, public health, social justice, transparency, and regulation is more aligned with liberal values and policy preferences. Conservative viewpoints, in contrast, might emphasize the economic benefits of fracking, energy independence, and less regulatory oversight, arguing for the importance of industry and market freedom over the concerns highlighted in this text.	liberal
AAFP	Hygiene, Personal Hygiene in School Settings	7/1905	The text provided advocates for policies ensuring the dignity, well-being, and health of school students through access to personal hygiene items, facilities consistent with their gender identity, and hygiene education. This perspective typically aligns more closely with liberal viewpoints for several reasons:<br><br>1. **Access to Personal Hygiene Products:** Advocating for access to personal hygiene products in schools, including menstrual products, reflects a concern for equity and the well-being of all students. It acknowledges socioeconomic disparities that may prevent some students from having these essential items. This concern for equity and support for social welfare programs is generally associated with liberal ideologies.<br><br>2. **Restroom Access Consistent with Gender Identity:** The emphasis on allowing students access to restrooms consistent with their gender identity aligns with liberal views on LGBTQ+ rights and inclusion. This standpoint reflects a broader liberal emphasis on protecting the rights and dignity of LGBTQ+ individuals.<br><br>3. **Hygiene Education:** The call for schools to provide hygiene education to supplement parental guidance can also be seen as a liberal stance. It underscores the role of public education in promoting public health and individual well-being, principles that are often emphasized in liberal thought.<br><br>4. **Public Health and Welfare:** The overall focus on ensuring health, dignity, and well-being through accessible hygiene practices and education in schools reflects a commitment to public welfare and preventive care. These are key aspects of liberal policies that prioritize public health initiatives and access to care and services for all individuals, especially vulnerable populations.<br><br>While these viewpoints are broadly categorized as liberal based on their emphasis on inclusivity, equity, and public welfare, it's important to note that there can be a range of opinions within both liberal and conservative circles. Some conservatives may also support aspects of these policies, especially if framed around public health and safety. However, the specific mention of gender identity and the strong emphasis on equity and inclusivity are more typically associated with liberal positions.	liberal

AAFP	Immunization Exemptions	7/1905	The text you provided outlines a position on immunization that emphasizes public health, vaccine safety, and adherence to established scientific and medical guidelines. It does not directly align with a political ideology on its own, as public health recommendations are based on scientific evidence rather than political beliefs. However, the way different political groups might view this statement can vary, influenced by their stance on issues like government intervention, individual freedoms, and public health policies.<br><br>- **Liberal Viewpoint**: Generally, liberals might support this position because it emphasizes the collective responsibility to public health and welfare, supports science-based policy, and advocates for vaccination as a means to protect community health. Liberals tend to favor policies that promote public health and safety, even if it means imposing certain requirements on individuals for the greater good.<br><br>- **Conservative Viewpoint**: Conservatives might have mixed views on this statement. Some conservatives who prioritize individual rights and have concerns about government mandates might be wary of strict vaccination requirements without room for personal or religious exemptions. However, other conservatives, especially those who prioritize public health and science-based policies, may support these recommendations. The conservative perspective can vary greatly depending on how an individual balances issues of personal freedom, public health, and the role of government in regulating health practices.<br><br>It's important to note that views on vaccination can cross traditional political lines, with individuals from both sides of the political spectrum having varying opinions on the matter. The stance presented in the text is more aligned with a public health perspective that may be supported by individuals across the political spectrum who prioritize scientific evidence and the welfare of the community.	neutral
AAFP	Immunizations	10/2023	The text provided outlines positions on vaccination policies and practices that are generally aligned with a viewpoint favoring public health initiatives, equitable access to healthcare, and a proactive role for government in healthcare provision. This perspective typically aligns more closely with liberal viewpoints, particularly in the context of the United States.<br><br>Liberal viewpoints often emphasize the role of government in ensuring public health, advocating for universal healthcare access, and supporting policies that aim to reduce disparities in healthcare access and outcomes. The support for universal immunizations regardless of socioeconomic or insurance status, advocacy for government programs to subsidize vaccine costs, and the call for insurance to cover immunizations without copayments or deductibles reflect a commitment to making healthcare services accessible to all. These positions also advocate for reducing the financial and administrative burdens on healthcare providers, ensuring they can focus on patient care rather than navigating complex insurance requirements or managing financial risks associated with vaccine supplies.<br><br>In contrast, conservative viewpoints might emphasize a more limited role for government in healthcare, favoring market-driven solutions and personal responsibility over government-funded programs. While there is bipartisan support for vaccination as a critical component of public health, the extent and mechanisms of government involvement in funding, distribution, and mandating vaccines can be points of divergence between liberal and conservative perspectives.<br><br>The text emphasizes the importance of:<br>- Universal access to vaccines recommended by authoritative bodies like the AAFP and ACIP.<br>- Government and insurance coverage of vaccination without patient cost-sharing.<br>- Simplifying the vaccine supply and distribution process for healthcare providers.<br>- Ensuring timely and fair payment to providers for vaccination services.<br><br>These positions are aligned with a philosophy that sees healthcare access, including vaccination, as a public good that should be supported by public policy and funding. This approach is typically more aligned with liberal ideologies that prioritize equity in healthcare access and the role of government in facilitating that access.	liberal

AAFP	Impaired and Clinically Deficient Physicians	12/2020	The text you've provided outlines a professional and clinical approach to addressing physician illness and impairment, focusing on definitions, the continuum of illness and impairment, and policies for support and management within the context of the American Academy of Family Physicians (AAFP). It does not directly align with a liberal or conservative viewpoint in the political sense, as it primarily concerns medical professionalism, standards, and support systems for practitioners facing health challenges.<br><br>The discourse on physician health and support for those with impairments is more related to medical ethics, professional standards, and healthcare policy rather than partisan political ideologies. While discussions surrounding healthcare provision, support for medical professionals, and approaches to professional standards and ethics can have political dimensions—especially in debates over healthcare funding, access, and regulation—the specific focus here is on ensuring competent medical care and supporting physicians' health, which is a concern across the medical profession irrespective of political affiliation.<br><br>In broader contexts, approaches to healthcare policy and support for professionals within the system can reflect underlying political philosophies, with more progressive or liberal views typically emphasizing systemic support, access to care, and inclusive policies, while conservative viewpoints might focus on personal responsibility, limited intervention, and the role of private sectors in providing solutions. However, this text specifically addresses professional conduct and support within the medical community, which transcends simple political categorization.	neutral
AAFP	Implicit Bias	10/2023	The text you've provided aligns more closely with a liberal viewpoint, particularly in its focus on addressing implicit bias, promoting health equity, and reducing health disparities through education and systemic changes. Liberal ideologies often emphasize the importance of recognizing and addressing systemic inequalities, including those within healthcare, to ensure equal treatment and outcomes for all individuals, regardless of their background. The emphasis on education about implicit bias, evidence-based mitigation strategies, and the call for broader institutional and structural policy changes to achieve greater health equity are in line with liberal approaches to social justice and healthcare reform.<br><br>Conservative viewpoints, while also concerned with effective healthcare, may prioritize different approaches, focusing more on individual responsibility, the reduction of government intervention in healthcare, and the preservation of traditional values and systems. While conservatives may recognize the existence of health disparities, the solutions they advocate for might differ, emphasizing market-based solutions, personal choice, and less regulatory oversight as means to improve healthcare outcomes.<br><br>The text's advocacy for systemic change, acknowledgment of implicit bias, and the focus on culturally appropriate, patient-centered care as strategies to combat health disparities are indicative of a more progressive stance on healthcare reform and social justice issues.	liberal
AAFP	Incarceration and Health: A Family Medicine Perspective (Position Paper)	1/2022	The text presents viewpoints and policies that are more aligned with liberal perspectives. It emphasizes the importance of healthcare access, reducing disparities, and advocating for humane treatment and supportive reentry into society for incarcerated and detained individuals, including those seeking asylum. The support for reducing sentences for nonviolent and drug possession offenders, opposing detention for asylum seekers, advocating for comprehensive healthcare access (including reproductive health care, mental health services, and substance use treatment), and addressing the socio-economic and racial disparities inherent in the justice system are typically associated with liberal ideologies. These positions focus on rehabilitation, health equity, and social justice, aiming to address the root causes of incarceration and improve public health outcomes for all, especially the most vulnerable populations.	liberal
AAFP	Inclusion of Intersectional Education Regarding LGBTQIA+ Patients for Medical Students, Residents and Physicians	10/2023	The text provided aligns more closely with a liberal viewpoint. Liberal ideologies generally emphasize equality, social justice, and the protection of marginalized communities. The focus on addressing health disparities, promoting inclusivity, and supporting comprehensive LGBTQIA+ training within medical education reflects these principles. Additionally, the emphasis on intersectionality and the opposition to funding restrictions for education or care related to LGBTQIA+ communities are consistent with liberal priorities of ensuring equal rights and access to healthcare for all, regardless of sexual orientation, gender identity, or racial background.<br><br>Conservative viewpoints, on the other hand, tend to emphasize traditional values and may be more cautious about changes to existing systems, including the integration of specific content related to LGBTQIA+ communities in education. While there is diversity within conservative perspectives, and not all conservatives would necessarily oppose the initiatives outlined in the text, the proactive approach to addressing health disparities and advocating for inclusivity detailed here is more typically associated with liberal positions.	liberal

AAFP	Independent Physician Associations (IPAs) Definition	12/2019	The text provided outlines the principles and strategic considerations involved in forming and operating an Independent Physician Association (IPA). It does not neatly align with traditional liberal or conservative political viewpoints, as it primarily focuses on healthcare management and business strategies aimed at improving healthcare delivery and efficiency. However, elements of the text can be interpreted as aligning with aspects of both liberal and conservative perspectives on healthcare, depending on the specific focus.<br><br>From a **liberal perspective**, the emphasis on improving healthcare delivery, enhancing access to comprehensive services, and focusing on preventive health and the management of chronic conditions could be seen as aligning with liberal values of ensuring access to quality healthcare for all. The notion of physicians working together to negotiate better contracts and provide more comprehensive care could also be interpreted as an effort to balance the power dynamics in healthcare, ensuring that patient care remains a priority over profit. The focus on preserving the doctor/patient relationship and ensuring clinical autonomy could align with liberal values of individual rights and protections within the healthcare system.<br><br>From a **conservative perspective**, the emphasis on efficiency, autonomy, and financial incentives for physicians could resonate with conservative values of free market principles and individual responsibility. The formation of IPAs as a means to reduce overhead, pursue business ventures, and negotiate favorable contracts with other entities reflects an entrepreneurial approach to healthcare. The concept of risk-sharing and capitation models, where IPAs assume financial risk for patient care, can also align with conservative preferences for market-driven solutions to healthcare costs and the reduction of regulatory burdens to foster innovation and competition.<br><br>Overall, the text presents a model of healthcare organization that seeks to balance efficiency, quality of care, and financial viability through collaborative business strategies. It advocates for a system that allows physicians to maintain autonomy and leverage their expertise to improve patient outcomes, which could find support across a broad political spectrum, depending on how individual elements are emphasized or implemented.	neutral
AAFP	Independent Practice	9/2022	The text provided outlines the stance of the American Academy of Family Physicians (AAFP) on the versatility and support needs of family medicine physicians, particularly highlighting the organization's commitment to physician and patient choice, as well as its recognition of the unique challenges faced by physicians in small or independent practice environments. It also mentions the AAFP's advocacy for policies that support independent practice.<br><br>This text doesn't neatly align with traditional liberal or conservative viewpoints as understood in a political context. Instead, it focuses on professional and practice-related issues within the healthcare sector, advocating for support and flexibility in the practice models of family medicine. The emphasis on physician autonomy, patient choice, and the support for small or independent practices can resonate with values often appreciated across the political spectrum:<br><br>- **Liberal viewpoints** might appreciate the emphasis on patient choice and the call for resources and support to ensure access to care, seeing it as an endorsement of diversity in healthcare provision and an effort to maintain high standards of care across different settings.<br><br>- **Conservative viewpoints** might appreciate the support for independent practices and the emphasis on physician choice and autonomy, viewing it as a defense of small businesses and an argument against overregulation and consolidation in healthcare, which they may see as detrimental to patient care and professional independence.<br><br>In summary, the text reflects professional values and priorities within the field of family medicine rather than a clear-cut political ideology. It speaks to a broad consensus on the importance of supporting physicians in various practice environments to ensure effective patient care.	neutral

AAFP	Information Technology Used in Health Care	9/2022	The text presents viewpoints that align with principles commonly associated with both liberal and conservative perspectives, although it may more closely align with liberal viewpoints due to its emphasis on equity, access to care for underserved populations, and the role of government and policy in healthcare. Here's how it aligns with each viewpoint:<br><br>### Liberal Viewpoints:<br>- **Support for Underserved Populations**: The emphasis on prioritizing financial and technical support for practices caring for historically underserved and marginalized populations aligns with liberal priorities of equity and social justice.<br>- **Federal Incentives and Policies**: The call for federal incentives to support uniform standards and interoperability, as well as the development of policies that consider the health and emotional wellbeing of patients, reflects a belief in the positive role of government intervention in healthcare to ensure fair access and standards.<br>- **Privacy Protections for All**: Advocating for privacy protections for all parties handling personal health information extends regulatory protections beyond current standards, aligning with liberal views on privacy and consumer protection.<br><br>### Conservative Viewpoints:<br>- **Efficiency and Reduced Administrative Burden**: The focus on creating an efficient health data ecosystem that does not impose undue financial or administrative burdens on physician practices can resonate with conservative values of free market efficiency and reduced regulation.<br>- **Interoperability and Reduced Government Burden**: Advocating for a system of interoperability that connects patients with their care teams through technology rather than through more direct government intervention could be seen as a conservative approach to healthcare reform, emphasizing market-driven solutions.<br><br>### Common Ground:<br>- **Interoperability and Information Sharing**: Both liberal and conservative viewpoints might agree on the benefits of interoperability and efficient information sharing in healthcare, as these can lead to improved patient outcomes and more efficient care delivery.<br>- **Value-Based Payment Arrangements**: Support for value-based payment arrangements that include financial incentives for quality and cost-effective care might also find bipartisan support, as they aim to improve healthcare outcomes while controlling costs.<br><br>In summary, while the text supports initiatives that could be seen as liberal due to their focus on equity, comprehensive privacy protections, and the use of federal incentives to achieve healthcare goals, it also includes elements that could gain support from conservative viewpoints, particularly in its emphasis on efficiency and reduced regulatory burden. The blend of these approaches reflects a comprehensive view of healthcare reform that seeks to balance efficiency, equity, and privacy in the health data ecosystem.	neutral
AAFP	Infringement on Patient Physician Relationship	1/2022	The text you've provided emphasizes the importance of a confidential relationship between patient and physician for effective medical care, opposes legislation that infringes on the patient-physician communication, and advocates for physicians' freedom to discuss all aspects of health and safety openly with their patients. This stance aligns more closely with viewpoints typically associated with liberal ideologies, especially in the context of healthcare policy.<br><br>Liberal viewpoints often prioritize individual rights and freedoms, including the right to privacy and autonomy in healthcare decisions. The opposition to legislation that limits the scope of communication between patients and physicians reflects a liberal stance on protecting patient rights and ensuring comprehensive healthcare access without governmental or legislative interference. This perspective is rooted in the belief that medical decisions should be made based on the best interests of the patient, guided by medical professionals rather than restricted or dictated by external laws that may not take into account individual circumstances or the nuances of medical practice.<br><br>Moreover, the focus on the potential harm to individual, family, and community health due to legislative restrictions on medical communication underscores a liberal emphasis on public health and the broader social implications of healthcare policy. It reflects a viewpoint that healthcare policies should be informed by medical expertise and evidence-based practices rather than political or ideological agendas.<br><br>However, it's important to recognize that healthcare issues can cross traditional ideological lines, and there may be conservatives who also support the principles outlined in the text, particularly those who advocate for doctor-patient confidentiality and	liberal

			autonomy in medical decision-making. The delineation between liberal and conservative viewpoints on specific issues like this can sometimes be fluid, with individuals and groups within each ideology holding a range of opinions.	
AAFP	Institutional Racism in the Health Care System	7/2019	The text you've provided aligns more closely with a liberal viewpoint. Liberal ideologies often emphasize the importance of recognizing and addressing systemic issues, including racism, and advocate for policies and practices aimed at promoting equality, diversity, and inclusiveness. The emphasis on acknowledging racism as a systemic issue, the historical context of racial and ethnic discrimination in healthcare, and the call for active measures to dismantle racist practices and policies are characteristic of liberal perspectives on social justice and equity.<br><br>Conservative viewpoints, on the other hand, might focus more on individual responsibility, limited government intervention, and may prioritize personal liberty over systemic analyses of social issues. While there are conservatives who recognize the importance of addressing racism, the systemic and institutional focus, as well as the advocacy for comprehensive policy changes and organizational reforms mentioned in the text, are more typically associated with liberal approaches to social reform.<br><br>The American Academy of Family Physicians (AAFP)'s stance as described in the text reflects a proactive and systemic approach to combating racism within healthcare, which includes recognizing the historical and ongoing impact of racism, advocating for anti-racist policies, and calling for significant organizational and societal changes. This approach is aligned with liberal principles of equity, social justice, and the role of institutions in addressing systemic inequalities.	liberal
AAFP	Insurance Coverage for Acupuncture Treatment for Chronic Pain	4/2020	The statement about the American Academy of Family Physicians (AAFP) supporting insurance coverage of acupuncture for pain control aligns more closely with a liberal viewpoint, particularly in the context of healthcare policy. Liberal viewpoints often emphasize the expansion of healthcare services and coverage, including alternative treatments like acupuncture, as part of a broader approach to patient care and wellness. This perspective advocates for increased access to a variety of treatment options within the healthcare system, reflecting a belief in the importance of comprehensive and inclusive healthcare policies.<br><br>Conservative viewpoints, in contrast, may prioritize traditional medical treatments and could be more cautious about the inclusion and insurance coverage of alternative therapies. They might focus more on cost-control measures, the role of private insurance, and less government intervention in healthcare choices, potentially leading to a more selective approach to what treatments should be covered by insurance.<br><br>However, it's important to note that opinions on specific healthcare policies like acupuncture coverage can vary widely within both liberal and conservative circles. Some conservatives may support such measures if they are seen to reduce overall healthcare costs or are supported by evidence of effectiveness. Similarly, some liberals might be skeptical of alternative treatments' efficacy or relevance in a healthcare coverage model. The distinction is not always clear-cut and can depend on broader perspectives on healthcare access, evidence-based medicine, and the role of government in regulating healthcare options.	liberal

AAFP	Integration of Primary Care and Public Health (Position Paper)	1/2022	The text provided generally aligns with a liberal viewpoint, particularly in its emphasis on social determinants of health, health equity, the integration of primary care and public health, and the advocacy for systemic changes to improve health outcomes. The focus on upstream factors, such as governance, culture, and societal values, and the call for actions that address social inequities and promote health equity, are consistent with liberal perspectives on health care. These viewpoints typically advocate for broad, systemic reforms to address inequalities and emphasize the role of social and environmental factors in health outcomes.<br><br>Liberal perspectives often support the idea that government and public policies play a vital role in ensuring equitable health care access and improving public health. The text's emphasis on collaboration between family physicians, public health entities, and community organizations, as well as the call for adjustments in payment models to facilitate integration and improve population health, aligns with these ideals.<br><br>In contrast, conservative viewpoints might focus more on individual responsibility for health, market-driven solutions to health care, and less government intervention in health care systems. While there is overlap in the recognition of the importance of health care quality and outcomes, the approaches and solutions proposed can differ significantly between conservative and liberal perspectives.<br><br>The text's approach to addressing the health care system's challenges by integrating primary care and public health, focusing on social determinants of health, and advocating for systemic changes to promote health equity is more aligned with liberal viewpoints on health care reform.	liberal
AAFP	Integrative Medicine	7/1905	The text provided outlines a viewpoint from the American Academy of Family Physicians (AAFP) regarding the integration of complementary and alternative medicine (CAM) or integrative medicine into conventional medical practice. It emphasizes the importance of evidence-based evaluation of these practices using scientific methods, including both quantitative and qualitative research to assess their efficacy and effectiveness. It also highlights the importance of considering cultural perspectives in the research process and suggests that family physicians educate themselves about non-conventional methods of healing to better serve their patients.<br><br>This viewpoint can be seen as aligning more closely with a liberal perspective, especially in the context of healthcare and medicine. Liberal viewpoints often prioritize inclusivity, diversity, and the consideration of a wide range of perspectives and approaches. The text's emphasis on evidence-based evaluations, cultural sensitivity, and the incorporation of alternative medicine into patient care reflects a willingness to explore and validate diverse healthcare practices beyond conventional medicine. This openness to integrating a variety of healing traditions and the focus on holistic patient care are characteristics often associated with more progressive or liberal approaches to health and wellness.<br><br>However, it's important to note that the acceptance and integration of CAM into conventional medical practice is not strictly a partisan issue and may find support across the political spectrum, particularly when such practices are evidence-based and shown to be effective. The emphasis on rigorous scientific evaluation and the potential benefits to patient care can appeal to values held by individuals with a range of viewpoints.<br><br>In summary, while the AAFP's stance on integrative medicine may align more closely with values typically associated with liberal perspectives—such as openness to diverse methodologies and a holistic approach to patient care—it is fundamentally focused on improving healthcare outcomes through evidence-based practices, a goal that transcends political ideology.	probably liberal

AAFP	Integrative Medicine, Credit for CME Activities	7/1905	The text provided outlines the American Academy of Family Physicians (AAFP)'s stance on the inclusion of integrative medicine in Continuing Medical Education (CME) and the standards and requirements for such inclusion. This stance is more aligned with a pragmatic approach to healthcare education rather than strictly fitting into liberal or conservative viewpoints. Here's why:<br><br>1. **Focus on Evidence-based Practice:** The insistence on evidence-based or customary and generally accepted medical practice, as well as the caution against practices that are not proven effective or could be dangerous, reflects a commitment to scientific rigor and patient safety. This approach is generally supported across the political spectrum but may resonate more with those who emphasize evidence-based policy and healthcare decisions, which can be characteristic of both liberal and conservative viewpoints depending on the context.<br><br>2. **Openness to Integrative Medicine:** The acknowledgment of integrative medicine and the importance of educating physicians about it can be seen as a more progressive or liberal stance, as it involves openness to a wider range of healing practices beyond conventional Western medicine. This inclusivity reflects a holistic approach to healthcare, recognizing the value of alternative therapies when they are supported by evidence and can safely complement conventional treatments.<br><br>3. **Regulatory Oversight and Professional Standards:** The text emphasizes the role of regulatory oversight (by the COCPD) in ensuring that CME content meets high standards of medical education and practice. This concern for regulation and quality control can align with more conservative values of maintaining professional standards and protecting patients from unproven or harmful treatments. However, it also fits with liberal values that prioritize patient safety and evidence-based healthcare.<br><br>4. **Balancing Innovation with Caution:** The approach taken by the AAFP balances openness to new medical practices (innovation) with a cautionary stance that prioritizes patient safety and evidence-based outcomes. This balance is a hallmark of pragmatic healthcare policy, which seeks to integrate the best of traditional and alternative medicine while filtering out ineffective or dangerous treatments.<br><br>In conclusion, the text represents a pragmatic approach to healthcare education that values evidence-based practice, patient safety, and the inclusion of effective integrative medicine. It does not neatly fit into a strictly liberal or conservative viewpoint but rather reflects a balanced, patient-centered approach to medical education and practice.	neutral
AAFP	Intimate Partner Violence	2019	The text you've provided focuses on the issue of intimate partner violence (IPV) and outlines the role that family physicians can play in identifying, preventing, and addressing it. This focus is primarily on healthcare, public health, and social welfare, topics that are often emphasized in liberal viewpoints due to their focus on social justice, healthcare accessibility, and the protection of vulnerable populations. Liberal perspectives typically advocate for comprehensive healthcare policies, support for survivors of violence, and the implementation of preventive measures to address the root causes of social issues, which aligns with the text's emphasis on screening, intervention, and education.<br><br>However, it's important to note that addressing IPV transcends traditional political divisions, and the recognition of IPV as a critical issue, along with the need for intervention and support for affected individuals, can be supported across the political spectrum. The text does not explicitly endorse or reject political ideologies but focuses on practical, evidence-based approaches to health care and IPV prevention, which can be supported by conservatives as well, especially when framed in the context of family values and community responsibility.<br><br>In summary, while the approach and solutions proposed in the text might align more closely with traditional liberal values of social welfare and public health, the issue of IPV itself and the call for action can resonate with individuals across the liberal-conservative spectrum, depending on how the strategies are framed and implemented.	probably liberal

AAFP	Issues Related to the Incarceration and Detention of Minors	2020	The text you've provided aligns more closely with a liberal viewpoint for several reasons:<br><br>1. **Focus on Rehabilitation Over Punishment**: The emphasis on rehabilitative treatment and evidence-based programs for minors, instead of incarceration, is a principle more commonly associated with liberal ideologies. Liberals often advocate for addressing the root causes of criminal behavior through social and psychological support, rather than solely relying on punitive measures.<br><br>2. **Protection of Minors' Rights**: The stance against trying minors as adults, holding them in adult facilities, giving them life sentences, and using solitary confinement aligns with liberal views on juvenile justice. These positions emphasize the need for a justice system that recognizes the developmental differences between minors and adults and seeks to protect minors' rights and well-being.<br><br>3. **Access to Comprehensive Health Care**: The call for minors to have access to comprehensive health care, including behavioral health care and reproductive health care, reflects liberal priorities regarding health care access and the holistic treatment of individuals.<br><br>4. **Support for Immigrant Minors**: The statements advocating for the non-detention of unaccompanied minors who are undocumented immigrants, refugees, and asylum seekers, and the opposition to family separation at immigration and detention facilities, are consistent with liberal views on immigration. Liberals often advocate for more compassionate immigration policies that respect human rights and the needs of vulnerable populations.<br><br>5. **Emphasis on Education and Mental Health**: The insistence on access to age-appropriate educational programming and settings that meet basic standards for physical and mental health underscores a liberal perspective on the importance of education and mental health support as fundamental rights and as part of rehabilitation.<br><br>Overall, the positions outlined in the text prioritize the health, well-being, and rights of minors within the justice and immigration systems, reflecting principles more commonly associated with liberal viewpoints. These stances advocate for systemic changes that focus on rehabilitation, health care access, and the protection of vulnerable populations, aligning with liberal values of equity, compassion, and support for the rights of individuals, especially minors.	liberal
AAFP	Laboratories Sharing Data	9/2022	The text you've provided appears to be focused on the policy recommendation or stance of the American Academy of Family Physicians (AAFP) regarding the exchange of laboratory data within healthcare settings. It advocates for seamless exchange of laboratory data between the laboratory and any member of the care team upon request, emphasizing that this should occur through the practice's preferred method without incurring additional costs to the practice.<br><br>This stance is primarily centered on improving healthcare delivery and efficiency rather than aligning directly with traditional liberal or conservative political viewpoints. The focus is on operational efficiency, patient care, data accessibility, and cost containment within the healthcare system. Such issues often receive bipartisan support, as they address universal concerns in healthcare like data interoperability, privacy, and cost management.<br><br>However, the broader context of healthcare policy debates can sometimes frame similar stances within a liberal or conservative viewpoint based on how they intersect with issues like government regulation, healthcare access, and the role of private enterprise in healthcare services. For example:<br><br>- **Liberal Viewpoint**: Might support this stance as part of broader efforts to ensure universal healthcare access, favoring regulations that make healthcare more efficient and accessible to all, and seeing the seamless exchange of data as a way to reduce costs and improve patient outcomes.<br><br>- **Conservative Viewpoint**: Might also support the efficient exchange of healthcare data if seen as a way to reduce unnecessary regulatory burdens on healthcare providers, improve market efficiency, and maintain patient choice without imposing new costs on healthcare practices.<br><br>In summary, the text itself is neutral and focuses on practical improvements in healthcare practice rather than ideological positions. Its alignment could be interpreted differently depending on the broader context of healthcare policy discussions and the specific aspects emphasized by liberal or conservative viewpoints.	neutral

AAFP	Laboratories, Physician Office	5/2023	The text you've provided outlines a position of the American Academy of Family Physicians (AAFP) regarding the payment and regulation of clinical laboratory procedures performed in physician offices. Specifically, it supports the idea that timely diagnostics in physician offices can improve patient care and advocates for appropriate payment for these services. Additionally, it emphasizes the importance of these laboratories participating in recognized accreditation and proficiency testing programs to ensure the accuracy of diagnostic tests and laboratory results.<br><br>This position does not straightforwardly align with traditional liberal or conservative political viewpoints as it focuses more on healthcare practice management and quality assurance than on broader political or ideological issues. Both liberal and conservative viewpoints may find aspects to support in this statement:<br><br>- **Liberal Viewpoint**: Liberals might support this position due to its emphasis on improving patient care through timely diagnostics and ensuring the quality and accuracy of laboratory tests, which aligns with broader liberal priorities of enhancing healthcare access and quality for all.<br><br>- **Conservative Viewpoint**: Conservatives might also support this position, particularly due to its implications for healthcare market efficiency and the potential for reducing regulatory burdens if it implies streamlining processes for physician offices. Moreover, conservatives may view the support for appropriate payment and accreditation as a means to uphold high standards in private healthcare practice.<br><br>In essence, the text is more about practical healthcare policy and ensuring quality and efficient patient care than it is about aligning with a specific political ideology. It reflects a professional and operational stance rather than a politically motivated one.	neutral
AAFP	Laetrile	5/2023	The statement "Scientific evidence has shown that Laetrile has no place in a treatment regimen for cancer patients" reflects a viewpoint that prioritizes scientific evidence and medical consensus over alternative or unproven treatments. It doesn't directly align with a political ideology such as liberal or conservative in the traditional sense. Instead, it emphasizes the importance of evidence-based medicine.<br><br>In general, discussions about the effectiveness of medical treatments based on scientific evidence tend to cross political lines, with individuals from both liberal and conservative backgrounds supporting treatments that have been proven effective through rigorous scientific research. However, attitudes towards alternative treatments can sometimes reflect broader political or ideological beliefs about regulation, medical freedom, and trust in traditional medical institutions, which can vary within and across political spectra.<br><br>It's important to note that the political implications of a statement like this can depend on the context in which it is made and the broader conversation about healthcare and medical treatment in which it is situated.	neutral
AAFP	Leadership Development	1/2022	The text provided does not explicitly align with a liberal or conservative viewpoint as it focuses on the role and training of family physicians, emphasizing their potential for leadership in healthcare improvement. The emphasis is on communication skills, teamwork, problem-solving, and advocacy for healthcare solutions, which are professional attributes rather than politically partisan positions.<br><br>However, the advocacy for health care solutions at various levels and the focus on improving patient care quality, safety, and access can be interpreted through different political lenses. For example:<br><br>- **Liberal Viewpoint**: Advocates might see the emphasis on improving access to healthcare and establishing standards of excellence as aligning with liberal values, which often prioritize healthcare reform, access to care for all, and the reduction of healthcare disparities. The focus on advocacy at local, state, national, and international levels can also resonate with liberal priorities on healthcare as a fundamental right.<br><br>- **Conservative Viewpoint**: Some aspects, such as the emphasis on leadership and the development of problem-solving skills, might be seen as aligning with conservative values that prioritize individual responsibility, innovation, and solutions that can include private sector initiatives. However, the conservative perspective might diverge on how to achieve healthcare goals, focusing more on market-driven solutions and less government intervention.<br><br>In summary, the text is more centered on professional development and roles within the healthcare system rather than political ideology. Its alignment with liberal or conservative viewpoints would largely depend on how its principles are applied in practice and interpreted through the lens of broader healthcare policy debates.	neutral

AAFP	Learner Access to Mental Health Services	1/2022	The text you provided primarily focuses on the importance of mental health services, wellness interventions, and support systems for medical students, residents, and physicians. It emphasizes the need for accessible, stigma-free mental health care within educational and professional healthcare settings. This approach aligns with a perspective that values comprehensive healthcare, inclusivity, and support for mental health, which can be associated with both liberal and conservative viewpoints in different contexts.<br><br>**Liberal Viewpoint Alignment:**<br>- The emphasis on mental health services, reducing stigma, and ensuring accessibility aligns with a liberal viewpoint that prioritizes healthcare access, mental health awareness, and the removal of barriers to care.<br>- The focus on institutional responsibility to foster a supportive culture around mental health could be seen as aligning with liberal ideals about the role of institutions in promoting public health and welfare.<br>- The advocacy for systemic changes, such as protected time for mental health care, opt-out systems for mental health services, and ensuring confidentiality and support for individuals experiencing mental health challenges, reflects a progressive approach to healthcare and education.<br><br>**Conservative Viewpoint Considerations:**<br>- Conservative viewpoints might also support elements of the text, especially those emphasizing personal responsibility (e.g., physician self-care) and the importance of mental health in maintaining professional competence and quality patient care.<br>- The idea that healthcare professionals need to manage their mental health to provide high-quality care could resonate with conservative values that prioritize individual responsibility and the efficiency of healthcare services.<br>- However, the detailed institutional and systemic interventions proposed might be viewed as excessive by some conservative perspectives, which often emphasize smaller government and less regulatory involvement in private sectors, including education and healthcare.<br><br>In summary, while the proposals and values outlined in the text have broad relevance and could find support across the political spectrum, the emphasis on systemic change, accessibility, and institutional responsibility for mental health care aligns more closely with liberal viewpoints. However, the focus on quality of care and individual responsibility within the professional context might also find resonance with conservative values.	probably liberal
AAFP	Legislative and Regulatory Activities	10/2023	The text you've provided outlines the American Academy of Family Physicians' (AAFP) approach to legislative and regulatory activities without explicitly aligning with a liberal or conservative viewpoint. The focus is on advocating for patients, promoting family medicine, and supporting evidence-based healthcare. These goals and methods can be seen as non-partisan and aim to advance the interests of family physicians, their patients, and communities through informed policy engagement.<br><br>The emphasis on evidence-based healthcare is a principle that transcends political affiliations, focusing instead on what research shows is effective in patient care. Advocating for policies that support the healthcare profession and patient care does not inherently align with a specific political ideology but rather seeks to improve health outcomes and the practice environment for healthcare providers.<br><br>The structured approach to analyzing legislation, communicating with lawmakers, and encouraging member involvement suggests a pragmatic and organized strategy to influence healthcare policy. While certain healthcare policies may be more supported by one political party than another, the text itself does not advocate for partisan positions but rather for actions that support the profession and patient care based on evidence and advocacy.	neutral

AAFP	Licensure	10/2023	The statement that the American Academy of Family Physicians (AAFP) supports the concept of licensure and relicensure, dating back to 1976 and referenced again in October 2023, does not inherently align with a strictly liberal or conservative viewpoint on its own. This stance is primarily a professional and regulatory position related to ensuring that physicians meet certain standards of education, skill, and ethics to practice medicine.<br><br>Support for professional licensure and relicensure is a matter of public safety and professional competence, aimed at ensuring that healthcare providers are qualified and maintain their qualifications over time. Both liberal and conservative viewpoints might agree on the importance of such measures, although they might differ on the specifics of implementation, regulatory oversight, and the extent of regulation required.<br><br>Liberal viewpoints might emphasize the role of government in regulating professions to protect public health and safety, advocating for strong oversight and comprehensive standards. Conservative viewpoints might focus on the importance of professional self-regulation, minimizing government intervention while still recognizing the need for some level of licensure to ensure competence and protect the public.<br><br>In summary, the AAFP's support for licensure and relicensure is a position that transcends simple political categorization, focusing instead on professional standards and public health.	neutral
AAFP	Licensure, Restricting Physician Licensure	1/2022	The statement that the American Academy of Family Physicians (AAFP) opposes making participation in a health plan a condition of physician licensure reflects a position on healthcare policy that doesn't neatly align with traditional liberal or conservative viewpoints in a straightforward manner. Instead, it touches upon issues related to healthcare access, physician autonomy, and regulatory policies in the healthcare sector. Here's a brief overview of how it might align with broader political viewpoints:<br><br>- **Liberal Viewpoint:** Liberals generally advocate for increased government involvement in healthcare to ensure access and equity. They might support policies that ensure a wide range of providers are available within health plans to maximize patient choice and access. However, opposing mandatory participation in a health plan could be seen as supporting physician autonomy and freedom, allowing them to choose how they practice. This specific position doesn't squarely fit the liberal agenda but aligns with the broader goal of ensuring access to care.<br><br>- **Conservative Viewpoint:** Conservatives typically support less government regulation and intervention in the market, valuing individual choice and competition. From this perspective, opposing mandatory participation in a health plan aligns with conservative values by resisting additional regulation on physicians' practices. It emphasizes professional autonomy and the principle that the government should not unduly interfere with the operation of healthcare markets or the practice of medicine.<br><br>The AAFP's position is more closely related to the principles of professional autonomy and the regulatory environment of healthcare practice rather than the broader ideological divides of liberal vs. conservative healthcare policies. It reflects concerns about the implications of tying licensure to health plan participation, which could affect physicians' ability to practice based on their professional judgment and preferences, potentially impacting healthcare delivery and access.	neutral

AAFP	Licensure/Relicensure, Definitions	7/2023	The text you've provided outlines the concepts of licensure and relicensure in the context of medical practice, specifically how they are mechanisms by which a state grants and renews permission for individuals to practice medicine. This text, in itself, is neutral and factual, focusing on the procedural aspects of medical licensure without delving into ideological or policy debates.<br><br>However, discussions about licensure can align with liberal or conservative viewpoints depending on the broader context or specific arguments being made. For example:<br><br>- **Liberal viewpoints** might focus on the importance of licensure as a means to ensure public safety, maintain high standards in the medical profession, and protect consumers from unqualified practitioners. They might also advocate for the regulatory framework to be flexible enough to not unduly limit access to the medical profession, particularly for underrepresented groups.<br><br>- **Conservative viewpoints** might emphasize the role of licensure as necessary for upholding professional standards but could also argue that overly stringent licensure requirements can create barriers to entry in the profession, limit competition, and potentially drive up healthcare costs. Conservatives might advocate for reducing unnecessary regulatory burdens to facilitate greater market freedom and efficiency.<br><br>The text you've provided does not explicitly endorse or oppose licensure; it simply describes the mechanisms of licensure and relicensure. Thus, without additional context or commentary, it does not align clearly with either a liberal or conservative viewpoint. It's important to consider the broader discussion or the specific policy proposals being made to accurately gauge the ideological alignment.	neutral
AAFP	Long-Term Care	7/2023	The text presents viewpoints that can be associated with both liberal and conservative ideologies, but it leans more towards a liberal perspective, especially in the context of American politics. Here's a breakdown of how the different elements align with each viewpoint:<br><br>1. **Support for Federal Policy on Long-Term Care**: Advocating for a comprehensive federal policy to address long-term care, including various forms of support (respite care, nursing home care, home health care), is generally more aligned with liberal ideologies. Liberals tend to support stronger government intervention in healthcare to ensure access and quality of care for all citizens.<br><br>2. **Verification by a Physician and Physician-Directed Evaluation**: The emphasis on professional medical evaluation and the role of physicians in directing care reflects a focus on quality and expertise in healthcare provision. This approach is not strictly partisan but leans towards a more regulated healthcare system, which is more in line with liberal views on healthcare.<br><br>3. **Case Management System and Coordination of Care**: Advocating for a case management system with opportunities for family physicians to coordinate or provide care aligns with both viewpoints but leans more towards a liberal perspective in emphasizing organized, accessible healthcare systems that ensure continuous and comprehensive care.<br><br>4. **Quality Assurance through Peer Review**: The call for Peer Review Organizations or state utilization review organizations to review medical care for quality assurance reflects a commitment to high-quality healthcare standards, which is a priority across the political spectrum but fits within the liberal advocacy for oversight and regulation in healthcare.<br><br>5. **Addressing Spousal Impoverishment**: Including provisions to address spousal impoverishment directly reflects a liberal concern for the financial protection and well-being of families affected by long-term care needs.<br><br>6. **Eligibility Based on Functional/Cognitive Capacity**: Basing eligibility on functional or cognitive capacity rather than diagnosis suggests a more inclusive approach to determining need for assistance, which aligns more with liberal principles of accessibility and equity in healthcare.<br><br>7. **Public and Private Financing**: Advocating for a mix of public and private financing for long-term care reflects a pragmatic approach that can appeal to both sides but is more characteristic of liberal strategies for achieving universal coverage and accessibility.<br><br>8. **Payment for Physician Visits Based on Service Appropriateness**: This point suggests a flexible, needs-based approach to healthcare financing, which is consistent with liberal views on ensuring healthcare is patient-centered and accessible.	liberal

			Overall, the emphasis on government involvement, quality assurance, inclusivity, and financial protection for individuals aligns more closely with liberal ideologies that advocate for comprehensive, accessible, and equitable healthcare systems. However, the proposal also includes elements (like mixed public-private financing) that could find support among conservatives who favor a role for private enterprise in healthcare.	
AAFP	Managed Care Reform	9/2022	The text outlines a position that aligns more closely with liberal viewpoints, particularly in the context of health care reform in the United States. The emphasis on ensuring access to high-quality health care, improving transparency, protecting patients' rights, and advocating for the role of primary care physicians as patient advocates reflect principles commonly associated with liberal or progressive health care policies. These policies prioritize patient care, equitable access, and comprehensive health care reform aimed at enhancing the quality and affordability of health care.<br><br>Liberal viewpoints typically advocate for reforms that increase oversight of insurance companies and managed care organizations (MCOs), ensure fairness in patient treatment, and promote accessibility to necessary medical services without undue financial burden on patients. The specific provisions mentioned, such as limiting prior authorization, ensuring a just and equitable appeals process, and requiring MCOs to make decisions based on medical necessity by knowledgeable physicians, are consistent with these goals.<br><br>Conservative viewpoints, on the other hand, might emphasize less regulation, more market-driven solutions to health care, and a reduced role for government in managing health care policies and systems. The text's focus on regulatory measures for MCOs and the emphasis on standardized, patient-centered care reforms suggest a more liberal approach to health care reform, aiming to balance the interests of patients, physicians, and health care providers within a regulated framework that prioritizes quality and equitable access to care.	liberal

AAFP	Marijuana and Cannabinoids: Health, Research and Regulatory Considerations (Position Paper)	7/2019	The text primarily aligns with a liberal viewpoint, particularly in its nuanced approach to marijuana and cannabinoid policy, emphasizing decriminalization, medical research, and public health considerations over punitive measures. Several key aspects highlight its liberal orientation:<br><br>1. **Support for Decriminalization**: The document advocates for the decriminalization of marijuana possession for personal use, emphasizing treatment and intervention over incarceration. This approach is more commonly associated with liberal policies, which often prioritize public health and harm reduction strategies over criminal penalties.<br><br>2. **Advocacy for Medical Research**: The text calls for decreased regulatory barriers to facilitate clinical and public health research on cannabis, including its reclassification from a Schedule I controlled substance. This stance reflects a liberal inclination towards evidence-based policy making and the acknowledgment of potential therapeutic benefits of marijuana and cannabinoids.<br><br>3. **Emphasis on Public Health and Patient-Centered Care**: The document outlines a comprehensive approach to addressing substance misuse, focusing on diagnosis, treatment, prevention, and patient and public health outcomes. This holistic and patient-centered approach is more aligned with liberal views on healthcare, which typically advocate for inclusive and accessible health services.<br><br>4. **Recognition of Potential Therapeutic Benefits**: While acknowledging the need for more research, the text highlights preliminary evidence of marijuana and cannabinoids' potential therapeutic benefits. This recognition of potential positive aspects, alongside the call for balanced research on negative impacts, indicates a liberal perspective that values scientific inquiry and nuanced understanding over outright prohibition or dismissal.<br><br>5. **Opposition to Recreational Use with a Focus on Regulation and Safety**: Despite opposing the recreational use of marijuana, the document supports robust regulation, safe usage, and further research into the effects of recreational use. This balanced stance, advocating for safety and informed policy rather than outright prohibition, aligns with liberal approaches to drug policy, which often seek to mitigate harm while considering individual freedoms and public health implications.<br><br>In summary, the text's focus on decriminalization, medical research, public health, and nuanced regulatory approaches reflects a liberal viewpoint on marijuana and cannabinoid policy. It emphasizes evidence-based practices, harm reduction, and the potential benefits of marijuana and cannabinoids while recognizing the need for appropriate regulations and safety measures.	liberal
AAFP	Marijuana Possession for Personal Use	7/1905	The text presents a nuanced viewpoint that incorporates elements from both liberal and conservative perspectives, making it not strictly aligned with one over the other. Here's how it reflects aspects of both:<br><br>### Liberal Viewpoint Elements:<br>- **Support for Decriminalization**: The support for decriminalization of marijuana possession for personal use aligns more closely with liberal viewpoints, which often emphasize reforming drug laws to reduce incarceration rates and address drug use as a public health issue rather than a criminal one.<br>- **Advocacy for Research and Treatment**: The emphasis on the need for intervention and treatment over incarceration for marijuana use and the call for more research into the effects of recreational marijuana use are consistent with a public health approach to drug policy, which is more often associated with liberal positions.<br><br>### Conservative Viewpoint Elements:<br>- **Opposition to Recreational Use**: The opposition to the recreational use of marijuana can be seen as aligning with more traditional conservative values, which often prioritize law and order and may view drug use as morally or socially harmful.<br>- **Acknowledgment of State Laws**: Recognizing the existence of state laws that have approved recreational use or possession indicates a respect for states' rights, a principle that is often emphasized in conservative thought. However, this acknowledgment does not necessarily endorse these laws, fitting into a more cautious conservative approach to changing drug policies.<br><br>### Overall:<br>The text demonstrates a balanced approach by opposing recreational use (a more traditionally conservative stance) while supporting decriminalization and advocating for research and treatment (more liberal approaches). This blend of viewpoints suggests an effort to find a middle ground that addresses public health concerns, supports evidence-based policy, and respects individual rights and state autonomy. It reflects a pragmatic stance focused on minimizing harm and promoting well-being, without fully aligning with the ideological extremes of either political spectrum.	neutral

AAFP	Medicaid Services	10/2023	The text from the American Academy of Family Physicians (AAFP) presents viewpoints that generally align more closely with liberal ideologies, particularly in the context of healthcare policy. Here's a breakdown of why it aligns more with a liberal viewpoint:<br><br>1. **Advocacy for comprehensive healthcare coverage for all Medicaid recipients**: The call for a uniform range of mandatory services and state-approved optional services for all Medicaid recipients reflects a commitment to ensuring that all individuals, regardless of their economic status, have access to a broad spectrum of healthcare services. This is a principle more commonly associated with liberal policies, which often emphasize the importance of universal healthcare access and the government's role in providing it.<br><br>2. **Support for fair and adequate Medicaid payment rates**: The insistence that Medicaid payment for services should be at least at Medicare rates to comply with the "equal access" provision of the Medicaid statute suggests an emphasis on equity and fairness in healthcare compensation. This standpoint aligns with liberal perspectives that advocate for equitable payment structures to ensure that healthcare providers are incentivized to serve Medicaid recipients.<br><br>3. **Continuous eligibility and coverage for vulnerable populations**: The emphasis on providing continuous eligibility for at least twelve months and including provisions for the homeless and medically uninsurable speaks to a commitment to protect and support the most vulnerable populations. Expanding Medicaid to avoid coverage gaps further aligns with liberal ideals of expanding government programs to ensure comprehensive coverage for those in need.<br><br>4. **Endorsement of peer and utilization review systems**: The endorsement of peer review systems and utilization review systems to promote uniform quality of care aligns with the liberal value of ensuring high standards of healthcare for all, regardless of socioeconomic status. This suggests a belief in the role of oversight and regulation in maintaining quality within public health programs.<br><br>While these points reflect principles commonly associated with liberal ideologies, it's important to note that healthcare policy can transcend traditional political boundaries. Advocacy for comprehensive and equitable healthcare coverage can find support across the political spectrum, driven by a shared recognition of the importance of healthcare access. However, the specific emphasis on expansion, equity, and government involvement in healthcare provision is more closely aligned with liberal viewpoints.	liberal

AAFP	Medicaid, Core Principles	2020	The text outlines principles and recommendations for the Medicaid program that align with a perspective typically associated with liberal or progressive political viewpoints. Here's a breakdown of how the text aligns with a liberal viewpoint:<br><br>1. **Increase in Federal Share for Medicaid**: Advocating for an increased federal share if Medicaid enrollment rises due to federal legislation reflects a liberal stance on expanding government support for healthcare, ensuring that states are not overburdened financially by expansions in Medicaid enrollment.<br><br>2. **Equal Payment for Primary Care Services**: The call for payment for primary care services to be at least equal to Medicare's rates indicates a desire to improve the compensation for primary care physicians, which can lead to better access to care for Medicaid recipients. This is consistent with liberal priorities of improving healthcare access and quality.<br><br>3. **Patient-Centered Medical Home Model**: Promoting the patient-centered medical home model, with appropriate payment for case management and chronic care coordination, aligns with liberal values of comprehensive, coordinated, and accessible healthcare services.<br><br>4. **First-Dollar Coverage of Preventive Services**: Requiring first-dollar coverage for preventive services emphasizes the importance of preventive care, a principle often supported by liberals as a means to reduce long-term healthcare costs and improve health outcomes.<br><br>5. **Evidence-Based Cost-Containment**: The focus on evidence-based research for cost-containment and defining medical necessity is consistent with a liberal viewpoint that policy decisions should be informed by scientific evidence and research.<br><br>6. **Support for Health Information Exchange**: Advocating for Medicaid to support health information exchange and electronic medical records through adequate payment aligns with liberal goals of modernizing healthcare infrastructure to improve care coordination and efficiency.<br><br>7. **Comprehensive Coverage for Tobacco Cessation, Substance Abuse, and Mental Health**: The emphasis on mandatory coverage for tobacco cessation, substance abuse treatment, and mental health services reflects liberal priorities for comprehensive healthcare that addresses all aspects of an individual's health.<br><br>8. **Equity in Territorial Assistance and Continuous Eligibility**: Calling for equitable federal financial participation in territorial assistance programs and continuous eligibility for at least twelve months supports a liberal agenda of ensuring fair access to healthcare services and reducing barriers to coverage.<br><br>9. **Accountability for Medicaid Managed Care Organizations**: The demand for accountability in Medicaid Managed Care Organizations, particularly regarding the adequacy of care networks and beneficiary access to services, aligns with liberal values of transparency, accountability, and access to healthcare.<br><br>Overall, the text promotes expanded access to healthcare, emphasizes the role of federal support, advocates for evidence-based policy decisions, and prioritizes comprehensive and preventive care services. These principles are closely aligned with liberal or progressive ideologies that focus on expanding healthcare access and improving healthcare quality through government intervention and support.	liberal

AAFP	Medical Home	9/2022	The text describing the Patient-Centered Medical Home (PCMH) model doesn't strictly align with a liberal or conservative viewpoint in the traditional sense of political ideologies. Instead, it outlines a healthcare delivery model focused on improving access, quality, and coordination of care. This model is based on principles that are generally aimed at enhancing patient care through a more organized, efficient, and patient-focused system.<br><br>However, different aspects of the PCMH model might be supported or criticized by individuals across the political spectrum for various reasons:<br><br>- **Liberal Viewpoint**: Individuals with liberal viewpoints might support the PCMH because it emphasizes equitable access to care, patient and caregiver engagement, and comprehensive healthcare services, including mental health. Liberals often advocate for reforms that increase access to healthcare services, improve quality of care, and ensure that healthcare systems are responsive to the needs of patients, especially underserved populations. The focus on continuous quality improvement and the optimal use of health information technology aligns with liberal priorities for healthcare reform.<br><br>- **Conservative Viewpoint**: Conservatives might appreciate the aspects of the PCMH that focus on efficiency, such as the use of data for continuous quality improvement and the emphasis on care coordination to prevent unnecessary services and reduce costs. The model's emphasis on accountability and potentially more efficient use of resources could appeal to conservative priorities for reducing government spending and increasing the efficiency of healthcare delivery. However, conservatives might be skeptical of the need for enhanced and prospective accountable payments and the degree of emphasis on comprehensive care, depending on their views on government involvement in healthcare.<br><br>In summary, the PCMH model incorporates elements that could be supported by both liberal and conservative viewpoints, depending on how the principles are implemented and the outcomes they achieve. The model's primary focus is on improving the healthcare delivery system for patients, which is a goal that transcends political ideologies.	neutral
AAFP	Medical Identification	10/2023	The statement you've presented focuses on a practical approach to healthcare, advocating for individuals to carry medical identification and information that could be critical in emergency situations. This viewpoint emphasizes the importance of accessibility to personal medical history for emergency personnel to provide safer and more appropriate care. It is based on a recommendation by the American Academy of Family Physicians.<br><br>The content of this statement does not inherently align with a strictly liberal or conservative viewpoint. Instead, it addresses a public health and safety issue with a solution aimed at improving emergency medical outcomes. Both liberal and conservative perspectives generally support the idea of improving healthcare outcomes and ensuring that emergency medical services are provided with the best possible information to make informed decisions.<br><br>In political discourse, the distinction between liberal and conservative viewpoints often involves debates on healthcare policy, funding, management, and the role of government in healthcare. However, the recommendation for individuals to carry medical identification to improve emergency care transcends these political divides, focusing on practical measures to enhance patient care. This recommendation is more about best practices in healthcare rather than a political stance on how healthcare should be managed or provided.	neutral
AAFP	Medical Learner Safety and Wellness During Pandemic Crisis	1/2022	The text provided outlines principles aimed at ensuring the safety, wellness, and educational integrity of medical learners, including medical students, early graduates, and residents, especially in the context of clinical service provision and during crises such as a pandemic. These principles advocate for voluntary participation, proper personal protective equipment (PPE), inclusion in vaccine distribution, adequate supervision, insurance and compensation, involvement in decision-making, compliance with work hours and education requirements, and the maintenance of patient safety and quality care.<br><br>This text does not explicitly align with traditional political viewpoints such as liberal or conservative as it primarily focuses on health and education policy within the medical community. Instead, it emphasizes safety, well-being, educational opportunities, and fair treatment of medical learners, which are concerns that cross political lines. However, its emphasis on inclusion, protection, and fair compensation could resonate more with viewpoints that prioritize workers' rights, public health, and education, themes often associated with liberal perspectives. Conversely, the focus on regulation, adherence to established guidelines, and ensuring quality care and patient safety can also align with conservative values that emphasize personal responsibility, the importance of tradition and established systems, and high-quality healthcare delivery.<br><br>In summary, while the text's principles can find support across the political spectrum, the emphasis on safety, rights, and equitable	neutral

			treatment of learners might be more closely aligned with liberal values, particularly in their focus on comprehensive protections and inclusivity. However, it's important to recognize that these principles reflect a professional and ethical stance within the medical education community that transcends simple political categorization.	
AAFP	Medical Necessity for the Hospitalization of the Abused and Neglected Child	7/1905	The text primarily outlines a position on how to handle cases of child abuse and neglect, focusing on the responsibilities of family physicians, the healthcare system, and third-party payers in ensuring the safety and well-being of children. It does not neatly align with traditional liberal or conservative political viewpoints as defined in the context of American politics or similar political divides in other countries. Instead, it addresses professional, ethical, and legal standards in the medical field concerning child welfare.<br><br>However, certain aspects of the statement could be interpreted through a political lens, depending on one's viewpoint:<br><br>- **Liberal Viewpoint Alignment:** The emphasis on the necessity of healthcare access for vulnerable populations, such as children suspected of abuse or neglect, and the assertion that financial concerns must not impede medical judgment, align with principles often associated with liberal ideologies. These include the belief in a strong role for government and other institutions in providing social services and ensuring public welfare. The call for third-party payers (usually insurance companies) to have an ethical responsibility to cover admissions might also resonate with liberal perspectives on healthcare access and equity.<br><br>- **Conservative Viewpoint Considerations:** While conservative viewpoints typically emphasize limited government intervention, personal responsibility, and free-market solutions to social issues, the text's focus on the ethical and legal obligations of medical professionals and institutions to protect children could find common ground with conservative values of family integrity and child welfare. However, the call for third-party payers to cover such admissions, regardless of financial concerns, might be seen as at odds with more market-oriented conservative views on healthcare funding and insurance.<br><br>It's important to note that issues of child welfare and protection from abuse transcend political boundaries, and there is often bipartisan support for policies and measures that aim to safeguard children. The text reflects a consensus within the medical community about best practices for the treatment and protection of children suspected of being abused or neglected, which can be supported by individuals across the political spectrum due to its focus on child safety and health rather than on political ideology.	neutral
AAFP	Medical Schools, Service to Minority, Vulnerable and Underserved Populations	7/1905	The text you provided aligns more closely with a liberal viewpoint. The focus on improving access to healthcare for disadvantaged, disenfranchised, minority, vulnerable, and underserved populations, along with the emphasis on inclusivity in medical education and targeted recruitment of medical students and residents from these communities, reflects principles often associated with liberal ideologies. Liberal perspectives typically prioritize social equity, access to essential services for all, especially marginalized groups, and the role of government and institutions in facilitating these objectives.<br><br>The support for expanding the National Health Service Corps (NHSC) as a strategy to improve healthcare in underserved areas, and the encouragement of government support for initiatives aimed at directing medical professionals towards careers in family medicine in underserved areas, also align with liberal priorities of using public policy and resources to address social disparities and healthcare access issues.<br><br>Conservative viewpoints, while also concerned with healthcare access and quality, might focus more on market-driven solutions, reduced government intervention, and personal responsibility in healthcare provision. The emphasis in this text on government-supported initiatives and targeted recruitment efforts in underserved areas is more characteristic of liberal approaches to addressing healthcare disparities.	liberal

AAFP	Medical Schools, Underrepresentation In Medicine	2020	The text reflects a viewpoint that aligns more closely with liberal ideologies, particularly in its emphasis on equity, diversity, and inclusivity within the medical profession. Liberal viewpoints often prioritize social justice, equal opportunity, and efforts to reduce disparities among different groups in society, including in education and professional fields.<br><br>The focus on equitable representation, leadership opportunities for those underrepresented in medicine, and the development of specific outreach programs to increase diversity in medical school applicants and matriculants are consistent with liberal efforts to address systemic inequalities and promote inclusiveness. Supporting leadership by family physicians who are underrepresented in medicine, and recommending commitment to the development of medical careers among those underrepresented or underpowered, also aligns with liberal values of diversity and equity.<br><br>Conservative viewpoints, on the other hand, might focus more on individual merit and qualifications without necessarily emphasizing programs specifically designed to increase representation of underrepresented groups. While conservatives may support diversity and equal opportunity, they might be more likely to question policies that they perceive as prioritizing identity over merit or that imply affirmative action.<br><br>Thus, the statement from the AAFP (American Academy of Family Physicians) reflects a commitment to addressing disparities and supporting diversity in the medical field, which aligns more closely with liberal values and policies aimed at achieving social equity and justice.	liberal
AAFP	Medical Student Debt	1/2022	The text you've provided reflects a viewpoint that leans more towards a liberal perspective, especially in the context of U.S. politics. Here are the reasons why:<br><br>1. **Emphasis on Access to Healthcare:** The statement emphasizes the expansion of the workforce to ensure that all Americans have access to a primary care patient-centered medical home. This focus on universal access to healthcare is a core principle of liberal ideologies, which often advocate for policies that aim to provide equal healthcare opportunities to all individuals, regardless of their economic status.<br><br>2. **Support for Federal Funding and Financial Aid:** The call for expanded funding for federal loan programs, specifically those that support family medicine and primary care, aligns with liberal viewpoints that support the role of government in providing financial assistance and resources to address social issues, in this case, the healthcare workforce shortage.<br><br>3. **Debt Relief for Medical Students:** The support for efforts to reduce the debt burden of medical students through the deferment of loan payments, tax-deductibility of interest, and the development of innovative debt relief programs reflects a liberal approach to education and professional training. Liberals often advocate for policies that reduce financial barriers to education and professional development, arguing that such barriers can prevent talented individuals from pursuing careers that are beneficial to society, like family medicine.<br><br>4. **Addressing Barriers to Choosing Family Medicine:** The recognition that debt incurred from medical training is a barrier to choosing family medicine as a profession, and the call for targeted support to address this issue, is consistent with liberal perspectives that seek to address systemic barriers and inequalities in professional fields.<br><br>It's important to note that while the text aligns more closely with liberal viewpoints, issues of healthcare access, education funding, and professional development can also be of concern to conservatives, though the methods and policies they support to address these issues may differ. Conservative viewpoints might focus more on market-driven solutions, private sector involvement, and minimizing government intervention, as opposed to direct government funding and support.	liberal

AAFP	Medical Student Participation with the Electronic Health Record	9/2022	The text provided outlines the American Academy of Family Physicians' (AAFP) stance on medical student participation in patient Electronic Health Records (EHRs). It emphasizes the importance of this access for the benefit of patient care and student education, advocating for no additional cost from the EHR vendor for such access. The text also stresses the need for appropriate supervision of medical students in this context and cautions against an over-reliance on student documentation for clinical operations.<br><br>This position primarily focuses on educational and operational aspects of medical training and healthcare delivery, rather than aligning directly with traditional liberal or conservative political viewpoints. The emphasis is on practical aspects of medical education, access to necessary tools for learning and patient care, and the operational efficiency of healthcare providers.<br><br>However, if we were to interpret the text in the context of broader political ideologies:<br><br>- **Liberal viewpoint alignment** might be seen in the advocacy for equal access to necessary educational resources (in this case, EHRs) without additional financial burdens on students. Liberals often prioritize access to education and healthcare resources as a means of leveling the playing field and ensuring equal opportunities for all.<br><br>- **Conservative viewpoint alignment** is less directly applicable here. However, conservatives might support the aspect of the statement that emphasizes the need for supervision and caution against over-reliance on students, reflecting a conservative approach to change and innovation that prioritizes caution and established practices.<br><br>In summary, the text is more closely aligned with practical and operational considerations in medical education and healthcare rather than fitting neatly into a liberal or conservative political framework. It addresses concerns relevant to both the quality of medical education and the efficiency and effectiveness of healthcare delivery, without making explicit political statements.	neutral
AAFP	Medical Waste Disposal in Non-Medical Locations	7/1905	The text provided outlines concerns and recommendations regarding the disposal of medical waste, emphasizing public health and environmental safety. It does not explicitly align with a liberal or conservative political viewpoint, as issues of public health and environmental safety can be of concern across the political spectrum. However, the focus on regulation, public education, and community-based programs for the safe disposal of medical waste could be seen as aligning more closely with typical liberal viewpoints, which often emphasize the role of government and community initiatives in addressing public health and environmental issues. Conservative viewpoints, on the other hand, might focus more on individual responsibility and private sector solutions to such problems, although this is not exclusive and there can be considerable overlap on issues of public health and safety.<br><br>The text specifically mentions the American Academy of Family Physicians (AAFP) and its support for:<br><br>1. Education about safe disposal of medical waste to the public,<br>2. Community-based disposal programs that are readily available and affordable, and<br>3. Policies to encourage and programs that provide safe community disposal of medical waste from non-medical settings.<br><br>These recommendations suggest a proactive approach to managing medical waste, aiming to mitigate health risks and environmental concerns. The emphasis on education, community involvement, and policy support reflects a comprehensive strategy that seeks to balance public health needs with environmental protection, which can be supported by individuals across the political spectrum who prioritize these issues.	probably liberal

AAFP	Medically Underserved	1/2022	The text reflects principles that are more commonly associated with liberal viewpoints, especially in the context of U.S. politics. Here's why:<br><br>1. **Emphasis on Reducing Health Disparities:** The focus on actively seeking to reduce health disparities experienced by marginalized communities, communities of color, and communities with lower average incomes aligns with liberal priorities of equality and social justice.<br><br>2. **Social, Environmental, and Political Advocacy:** The encouragement for members to engage in advocacy across social, environmental, and political realms to address health disparities is consistent with a liberal approach to health care, which often includes broader societal and systemic changes.<br><br>3. **Cooperation with Community Health Centers and Free Clinics:** The support for cooperation between family physicians, community health centers, and free clinics emphasizes a community-based approach to health care, which is more aligned with liberal values that prioritize accessible and community-oriented health solutions.<br><br>4. **Promotion of Fact-Based Health Education:** Advocating for fact-based health education in various community settings reflects a liberal stance on education and public health, emphasizing the importance of evidence-based policies and information dissemination.<br><br>5. **Sustained Funding for Evidence-Based Policies:** The call for sustained funding for policies and programs that promote health and disease prevention reflects a liberal perspective on public health, prioritizing long-term investment in health care solutions and preventive measures.<br><br>6. **Simplified Regulations and Improved Medical Payment Models:** While the desire for simplified regulations could be seen in both liberal and conservative viewpoints, in this context, it is coupled with the goal of encouraging the establishment of physician practices in underserved areas, aligning with liberal aims to increase healthcare accessibility.<br><br>7. **Development of Programs for Underserved Areas:** Finally, the focus on developing programs to encourage health care professionals to work in underserved areas aligns with liberal priorities around equity and access to health care for all, regardless of income or geographic location.<br><br>It's important to note, however, that health care can be a bipartisan issue, with both liberal and conservative groups advocating for reforms that address disparities and improve the health care system. The specific policies and approaches favored can vary widely within these broad categories.	liberal

AAFP	Medicare Payment	9/2022	The text outlines a series of positions taken by the American Academy of Family Physicians (AAFP) regarding Medicare payments to physicians, focusing on equity, transparency, and fairness in reimbursement practices. This stance encompasses a broad range of policy recommendations aimed at adjusting the Medicare payment system to better support family physicians and, by extension, their patients.<br><br>The positions advocate for:<br><br>- More equitable payment structures that reflect the actual costs and resources involved in providing care.<br>- Opposition to programs that limit physician payment and mandatory assignment, suggesting a preference for more autonomy for physicians in setting their fees.<br>- Support for transparency in Medicare payments and the elimination of language that might be misleading or vague to beneficiaries.<br>- A unified approach to calculating payments, except when deviations serve specific public policy objectives.<br>- Adjustments to payment formulas to more accurately represent the costs of delivering care, including support for models that value evaluation and management services.<br>- Initiatives aimed at enhancing primary care through additional payment models, such as care management fees for medical homes, and advocating for payment levels that support primary care in both traditional Medicare and Medicare Advantage plans.<br>- Encouragement of performance-based bonus payments and the development of alternative payment models that focus on primary care and health equity.<br><br>This text generally aligns with a policy perspective that emphasizes the value of primary care, equitable access to healthcare services, and the improvement of health outcomes through strategic adjustments in payment systems. Such perspectives often find more resonance within liberal policy frameworks, which tend to emphasize equity, access to healthcare, and the government's role in ensuring that access. However, the emphasis on fairness, transparency, and efficiency in payment, as well as the support for performance-based measures and the allowance for physician autonomy in fee setting, could also find some support among more conservative viewpoints that value market principles and individual choice within healthcare.<br><br>In essence, while the detailed policy positions of the AAFP could be seen as aligning more closely with liberal viewpoints due to their focus on equity and access, elements of these positions also reflect values typically associated with conservative perspectives, such as efficiency, transparency, and a degree of market-oriented flexibility. This blend of priorities reflects the complexity of healthcare policy, where initiatives often seek to balance equity and efficiency to serve the broadest possible range of interests.	probably liberal
AAFP	Medicare/Medicaid Abuses	7/1905	The text you've provided expresses a viewpoint from the American Academy of Family Physicians (AAFP) regarding the abuse of Medicare/Medicaid or any health assistance programs. It emphasizes the AAFP's condemnation of such abuses and its call for due process to be followed. This statement is more aligned with a principle of accountability and fairness in healthcare administration than with a specific political ideology such as liberal or conservative.<br><br>Liberals typically advocate for strong social safety nets, including robust health assistance programs, and emphasize the importance of protecting these programs from abuse while ensuring they serve those in need effectively. Conservatives might also agree with the need for accountability and due process in these programs, emphasizing efficient government spending and preventing fraud.<br><br>The focus of the AAFP's statement on due process and opposition to abuses in health assistance programs speaks to a shared concern across the political spectrum for integrity and effectiveness in healthcare administration. It doesn't neatly align with one political viewpoint but rather addresses a concern that transcends political divisions, focusing on the ethical and fair management of health assistance resources.	neutral

AAFP	Medication, Device, and Biologic Agents Testing and Selection	10/2023	The text you've shared focuses on principles of drug and device testing, representation in clinical research, "off label" use of medications, and shared decision-making in patient care. These principles broadly align with values that are not strictly limited to a liberal or conservative viewpoint but rather reflect a commitment to evidence-based medicine, patient safety, inclusivity in medical research, and patient-centered care.<br><br>- **Evidence-Based Medicine and Patient Safety**: The emphasis on rigorous pre- and post-market testing for safety and effectiveness is a hallmark of evidence-based medicine, which is a foundation of modern medical practice. Both liberal and conservative viewpoints generally support the use of scientific evidence to guide medical decision-making, though they may differ on the degree of regulation they deem necessary.<br><br>- **Inclusivity in Clinical Research**: Advocating for adequate representation of historically underrepresented populations in clinical research aligns with broader goals of equity and inclusivity. This principle is often more emphasized in liberal policies, which typically focus on addressing inequalities and promoting diversity, but the importance of diverse representation in clinical trials is widely recognized across the political spectrum for its role in improving healthcare outcomes for all.<br><br>- **"Off Label" Use of Medications**: Support for the scientifically backed "off label" use of medications indicates a flexible approach to patient care, acknowledging that the best treatment option might not always be one that has been officially approved for a specific indication. This flexibility in treatment choices transcends political divides, appealing to the practical aspects of patient care, though there might be differing opinions on the level of regulatory oversight required.<br><br>- **Patient-Centered Care and Shared Decision-Making**: The emphasis on considering the patient's values and preferences in treatment decisions reflects a commitment to patient autonomy and shared decision-making. This approach is a key aspect of patient-centered care, a concept widely endorsed across the healthcare field. It aligns with both liberal values of individual rights and autonomy and conservative values of personal responsibility and decision-making.<br><br>In summary, the principles outlined in the text are consistent with a patient-centered, evidence-based approach to healthcare that transcends simple liberal or conservative categorizations. They reflect a commitment to scientific rigor, inclusivity, patient safety, and respect for patient autonomy—all fundamental to ethical medical practice.	neutral
AAFP	Membership Designation	2019	The text you've provided appears to be guidelines or policies related to professional designations within the field of family medicine, specifically for members of the American Academy of Family Physicians (AAFP) and diplomates of the American Board of Family Medicine (ABFM). It outlines the proper and ethical ways to indicate one's earned degrees or certifications in professional contexts.<br><br>This text does not align directly with political viewpoints, liberal or conservative. It focuses on professional standards and ethics within a medical organization, which is a non-partisan issue. The guidelines are about how medical professionals should represent their qualifications and achievements, which is a matter of professional conduct rather than political ideology.	neutral
AAFP	Membership Evaluation, Discrimination in	7/1905	The text you've provided outlines a position against discrimination in various forms, emphasizing inclusivity and equality across a wide range of characteristics and identities. This stance, taken from a policy or statement by the American Academy of Family Physicians (AAFP), is more closely aligned with viewpoints typically associated with liberal ideology. Liberal perspectives generally advocate for protection against discrimination, support for diversity, and the promotion of equal rights for individuals regardless of their race, religion, gender, sexual orientation, and other characteristics.<br><br>Conservative viewpoints, while varying widely, traditionally emphasize individual responsibility, limited government intervention in personal lives, and may prioritize different aspects of policy and societal structure. While conservatives may also support anti-discrimination measures, the emphasis on a broad range of categories for protection and the explicit inclusion of gender expression, gender identity, and sexual orientation in anti-discrimination policies is more characteristic of liberal positions.<br><br>It's important to note, however, that terms like "liberal" and "conservative" can encompass a wide range of beliefs and positions within different contexts, and individuals or organizations might hold views that don't neatly fit into one category or another. Additionally, views on specific issues can evolve over time within both liberal and conservative circles.	liberal

AAFP	Menstrual Hygiene Products, Access to	2020	The text you've provided aligns more closely with a liberal viewpoint. The emphasis on recognizing menstrual hygiene products as medical necessities, advocating for their free or reasonably priced availability in public restrooms, and specifically calling for their provision at no charge to incarcerated or detained individuals, reflects concerns typically associated with liberal politics. These concerns include gender equality, public health, human rights, and addressing the needs of marginalized populations.<br><br>Liberal viewpoints often prioritize social equity, access to healthcare, and the protection of human rights, advocating for policies that aim to reduce inequalities and support vulnerable groups. The mention of menstrual hygiene products as a necessity rather than a luxury item and the focus on ensuring access to these products for all, including those in detention facilities, is indicative of a commitment to these principles.<br><br>Conservative viewpoints, while varied, tend to emphasize individual responsibility, limited government intervention in personal lives, and fiscal conservatism, which might lead to a more cautious approach to government-mandated provisions like those described in the text. However, it's important to note that perspectives on specific issues like menstrual hygiene can vary within both liberal and conservative circles, and there may be conservatives who support such measures on the grounds of public health and basic human dignity.	liberal
AAFP	Mental and Behavioral Health Care Services by Family Physicians (Position Paper)	12/2023	The text provided presents a comprehensive overview of mental health issues, emphasizing the need for improved access to care, the integration of mental health services into primary care, and addressing health inequities among various populations. While it does not explicitly align with a strictly liberal or conservative viewpoint, several elements of the text reflect concerns and policy positions more commonly associated with liberal perspectives:<br><br>1. **Emphasis on Health Equity and Access**: The text highlights disparities in mental health prevalence and access to care among different racial and ethnic groups, as well as specific challenges faced by marginalized populations (e.g., LGBTQ+ individuals, rural populations, incarcerated people). Addressing health inequities and advocating for inclusive healthcare practices are often more closely aligned with liberal viewpoints, which tend to emphasize social justice and equity.<br><br>2. **Advocacy for Comprehensive Healthcare Coverage**: The discussion about the need for improved payment for behavioral health services, equitable access to healthcare, and the role of Medicaid and private insurance in covering mental health treatment reflects a liberal inclination towards ensuring that healthcare is accessible and affordable for all, regardless of socioeconomic status.<br><br>3. **Support for Integrated Healthcare Models**: The call for the integration of mental and behavioral health services into primary care and the support for telemedicine and telepsychiatry suggest a progressive approach to healthcare delivery. These models aim to make healthcare more holistic, efficient, and accessible, aligning with liberal priorities for innovation in healthcare to meet diverse needs.<br><br>4. **Focus on Systemic Issues and Institutional Racism**: Acknowledging the impact of institutional racism on healthcare access and outcomes, and the need for culturally competent clinicians, is indicative of a liberal perspective that recognizes systemic inequalities and the importance of addressing them to improve healthcare for all.<br><br>However, it's important to note that mental health is increasingly recognized as a non-partisan issue, with growing awareness and advocacy across the political spectrum for improving mental health care access and addressing mental health challenges. While the specific approaches and solutions might vary, there is a broad consensus on the importance of addressing mental health as a critical component of public health.	liberal

AAFP	Mental Health, Parity in Coverage for Patients	10/2023	The text you provided outlines a stance on health insurance coverage, particularly focusing on mental and behavioral health. This stance advocates for:<br><br>- Parity in health insurance coverage and payment for all patients, irrespective of the nature of their health issues (mental or medical).<br>- Inclusion of mental and behavioral health coverage within the scope of services provided by family physicians, rather than restricting these services to specialized networks.<br>- Enforcement of mental health parity requirements by federal agencies.<br>- Integration of payment for licensed behavioral health providers into the medical benefits of patients, especially when these services are delivered under the supervision of a primary care physician within an integrated behavioral health model.<br><br>This viewpoint aligns more closely with liberal perspectives, particularly in its emphasis on regulatory oversight, the integration of mental health services with primary care, and the call for parity in health insurance coverage. Liberal ideologies often prioritize the expansion of healthcare access and the enforcement of policies that ensure equitable treatment across different types of healthcare, including mental health. The support for federal enforcement mechanisms and the integration of behavioral health into primary healthcare settings reflects a belief in the role of government and integrated healthcare systems in improving patient care and outcomes.<br><br>Conservative viewpoints, on the other hand, might emphasize less regulatory oversight, more market-driven solutions, and potentially less support for mandates on private health insurance providers. While there is a growing recognition across the political spectrum of the importance of mental health, the methods of achieving parity and integration of services can differ significantly between liberal and conservative ideologies.<br><br>The American Academy of Family Physicians (AAFP)'s position, as outlined in your text, reflects a comprehensive approach to healthcare that seeks to reduce disparities between mental and medical health treatments. This approach is more aligned with principles typically advocated by liberal policies, which focus on inclusivity, equitable access to healthcare services, and a strong role for government in regulating and ensuring the provision of healthcare services.	liberal
AAFP	Mental Health, Physician Responsibility	7/1905	The text provided outlines a perspective on family medicine that emphasizes a holistic approach to patient care, incorporating the treatment of mental health issues alongside physical health concerns within the context of family medicine. This approach focuses on treating the whole patient by recognizing the interconnectedness of mind, body, and spirit, and highlights the importance of diagnosing and treating mental illnesses within the family and individual context. It also mentions the expansion of treatment options, improvements in pharmacological treatments, and the development of care guidelines as factors making the treatment of mental illness in the family physician's office more practical, necessary, and appropriate.<br><br>This perspective is generally aligned with a viewpoint that values comprehensive healthcare, including the integration of mental health services into primary care. It emphasizes the importance of access to a broad range of healthcare services and the need for healthcare systems to adapt to provide holistic care.<br><br>In terms of political alignment, while healthcare approaches themselves are not inherently political, the emphasis on comprehensive, integrated care and the importance of treating mental health conditions as part of primary care can be associated with more liberal healthcare policies. These policies often prioritize the expansion of healthcare services, including mental health services, and advocate for a healthcare system that is accessible and inclusive of all types of care.<br><br>Conservative viewpoints might also support the integration of mental health into primary care but may emphasize different aspects, such as cost-effectiveness, personal responsibility, or the role of private sector solutions in healthcare innovation.<br><br>Thus, while the text outlines a healthcare philosophy rather than a political stance, its emphasis on comprehensive and integrated care aligns more closely with principles often advocated by those with liberal viewpoints on healthcare policy. However, it's essential to note that the importance of mental health in family medicine can be recognized across the political spectrum, with varying approaches to how it should be implemented and funded.	probably liberal

AAFP	Mercury in Food as a Human Health Hazard	9/2022	The text you've provided aligns more closely with viewpoints that are traditionally considered liberal rather than conservative, primarily due to its emphasis on public health, environmental regulation, and the proactive role of government agencies in safeguarding public health. Here's a breakdown of why it might be seen as aligning with a liberal viewpoint:<br><br>1. **Support for Regulation and Testing by Government Agencies**: The text supports continued testing and reporting of mercury levels in seafood by local, state, and national agencies. This is a stance that generally aligns with liberal viewpoints, which often advocate for regulatory measures to protect public health and the environment.<br><br>2. **Emphasis on Public Health and Safety**: Advocating for family physicians to inform their patients about the benefits and risks of seafood consumption, especially concerning vulnerable populations like developing fetuses and young children, reflects a prioritization of public health. Liberal perspectives typically emphasize the importance of public health initiatives and the role of healthcare providers in promoting well-being.<br><br>3. **Consumer Information and Protection**: The call for consumer-friendly guidelines from the Environmental Protection Agency (EPA) and the Food and Drug Administration (FDA) regarding seafood consumption suggests a belief in the right of consumers to be informed about the safety of their food. This is in line with liberal views that often support transparency and consumer rights, particularly in relation to environmental health issues.<br><br>4. **Environmental Concerns**: The reference to the safety of the water supply containing the fish ties into broader environmental concerns, suggesting an interest in not only the direct impact of mercury on health but also the overall quality of natural water sources. Liberal ideologies typically place a strong emphasis on environmental protection and sustainability.<br><br>However, it's important to note that concern for public health and safety, especially regarding food and environmental issues, can transcend traditional political divisions. Conservatives might also support such measures, especially when they align with interests in public safety, economic stability for the seafood industry, and local food sourcing. The distinction often comes down to the degree of regulatory intervention advocated for and the perceived roles of government versus market forces in ensuring safety and health.	liberal
AAFP	Migrant Health Care	2019	The text reflects a viewpoint that is generally more aligned with liberal perspectives, particularly in its emphasis on providing access to essential health care services regardless of immigration status. Liberal viewpoints often prioritize inclusivity, equity, and the provision of welfare services to all individuals, including marginalized and underserved populations. The focus on the health and well-being of migrant and seasonal workers, and the call for physicians and the public to be aware of and address their unique health care needs, aligns with these principles.<br><br>Conservative viewpoints, on the other hand, might emphasize a more restricted approach to health care access, often prioritizing citizens and legal residents, and may have concerns about the implications of providing services to individuals regardless of immigration status on public resources, legality, and immigration policy.<br><br>It's important to note that these are broad generalizations and that there is a wide range of opinions within both liberal and conservative circles. The specific stance of the American Academy of Family Physicians (AAFP) reflects a commitment to universal health care access and the health needs of all individuals living in the U.S., which is more closely associated with liberal values on health care and immigration.	liberal

AAFP	Military Service, Physicians' Draft	7/2023	The text from the American Academy of Family Physicians (AAFP) regarding their stance on a physician draft and the mobilization of citizens for defense purposes does not align strictly with a liberal or conservative viewpoint in the traditional sense of these terms within American political discourse. Instead, it reflects a pragmatic and nuanced approach to addressing the issue of military service and physician recruitment, which can be analyzed through several lenses:<br><br>1. **Opposition to a Separate Physician Draft**: This position might be seen as aligning with more progressive or liberal values insofar as it opposes compulsory service specifically targeted at a particular profession and advocates for exploring alternatives to conscription. Liberals often emphasize individual rights and might argue against policies that unduly burden specific groups.<br><br>2. **Support for Efficient and Dignified Use of Human Resources in a Draft**: This aspect of their position suggests a balanced approach to a complex issue. It recognizes the potential necessity of a draft while advocating for it to be conducted in a manner that respects individual dignity and efficiency. This could be seen as a centrist or moderate stance, valuing both the needs of national defense and the rights of individuals.<br><br>3. **Local Administration Under Federal Regulations**: Advocating for local administration guided by federal regulations might appeal to those across the political spectrum. Conservatives often favor local control and minimal federal intervention, whereas liberals might focus on the need for federal oversight to ensure fairness and respect for individual rights.<br><br>The AAFP's stance is primarily focused on the practical and ethical considerations of mobilizing human resources for national defense, rather than being driven by a specific political ideology. It emphasizes the importance of exploring all alternatives to conscription, ensuring any draft is administered fairly, and respecting individual dignity. This approach can resonate with values across the political spectrum, reflecting a concern for both the effectiveness of defense measures and the rights and well-being of individuals.	neutral
AAFP	Mistreatment of Learners in Medical Education	9/2022	The text presents viewpoints and recommendations that align more closely with liberal ideologies, particularly those focused on diversity, equity, inclusion (DEI), and anti-discrimination measures within the medical education system. Key aspects include:<br><br>1. **Promotion of an Inclusive and Equitable Learning Environment**: The text emphasizes creating a learning environment that is inclusive and equitable for everyone, condemning mistreatment and discrimination in any form. This approach aligns with liberal ideologies that prioritize social justice, equity, and the protection of rights for marginalized and underrepresented groups.<br><br>2. **Focus on Diversity and Representation**: The emphasis on increasing diversity and representation of underrepresented groups at all levels of medical education reflects liberal values. The belief is that a diverse medical workforce can enhance learning, decrease isolation, and lead to better patient outcomes, reflecting a commitment to social justice and equity.<br><br>3. **Anti-Oppressive, Anti-Harm Lens**: Viewing all aspects of education through an anti-oppressive, anti-harm lens is a hallmark of progressive thought. This approach seeks to dismantle systems of power and privilege that perpetuate discrimination and inequality.<br><br>4. **Equitable Learner Assessment and Evaluation**: Advocating for equitable assessment and evaluation processes to eliminate potential biases reflects a commitment to fairness and justice, ensuring that all learners are assessed on their merits without the influence of implicit or structural biases.<br><br>5. **Change Management Frameworks for Institutional Change**: The call for institutions to use change management frameworks to address ideological, institutional, interpersonal, and internalized oppression aligns with progressive strategies for systemic change. This approach seeks to overhaul existing systems to make them more equitable and just.<br><br>6. **Critique of Historical and Structural Inequities**: The text's acknowledgment of the historical and structural inequities within medical education, including the lasting impact of the Flexner Report on the representation of African American physicians, demonstrates a liberal inclination towards recognizing and rectifying past injustices to create a more equitable future.<br><br>In summary, the text's focus on promoting diversity, equity, and inclusion, addressing systemic inequities, and advocating for systemic changes to create a more inclusive and equitable medical education system aligns with liberal viewpoints. These priorities reflect a commitment to social justice, equity, and the rights of marginalized and underrepresented groups, hallmarks of liberal ideologies.	liberal

AAFP	Motorized Recreational Vehicles	7/1905	The text from the American Academy of Family Physicians (AAFP) regarding recommendations for family physicians on the use of motorized recreational vehicles and the support for certain laws related to their operation doesn't neatly align with a traditional liberal or conservative viewpoint in a political sense. Instead, it focuses on public health and safety considerations.<br><br>The recommendations advocate for physician awareness of the risks associated with motorized recreational vehicles, advice on their safe use based on an individual's development, and support for laws that aim to mitigate those risks (e.g., age restrictions, speed limits, and prohibitions on operating under the influence). These positions generally reflect a concern for public health and safety, which is a cross-cutting issue that can garner support from both liberal and conservative individuals, depending on the context and framing.<br><br>- **Liberal viewpoint considerations:** Liberals might support these recommendations due to the emphasis on regulation for safety and health, the concern for the welfare of children, and the support for legal measures to prevent harm. These align with broader liberal values focusing on the role of governance in protecting individuals, especially vulnerable populations, from preventable risks.<br><br>- **Conservative viewpoint considerations:** Conservatives might also support aspects of these recommendations, particularly those that align with traditional values of personal responsibility and protection of family and youth. The emphasis on state laws and the potential for local control over regulations can also appeal to conservative principles favoring decentralized governance. However, some conservatives might oppose what they see as excessive regulation, arguing for more personal freedom and less government intervention in recreational activities.<br><br>In summary, the text reflects a public health perspective that transcends simple liberal-conservative dichotomies, focusing instead on the wellbeing and safety of individuals, especially children. The support or opposition to these recommendations from either side of the political spectrum might vary based on broader views on regulation, governance, and personal freedom.	neutral
AAFP	Motorized Vehicle Occupant Protection	7/1905	The text provided primarily aligns with a public health and safety perspective rather than fitting neatly into a liberal or conservative political viewpoint. The focus is on promoting the safety of all vehicle occupants through the use of appropriate restraint systems and safety features, based on best practices and guidelines for child restraint systems (CRS) that consider the size and age of children. The American Academy of Family Physicians (AAFP)'s support for legislation mandating occupant restraint systems is a stance on improving public health outcomes rather than a position that can be clearly categorized as liberal or conservative.<br><br>Both liberal and conservative viewpoints might find common ground on the importance of public safety, especially when it comes to protecting children. Liberals might emphasize the role of government in ensuring public safety and setting standards for health and safety, including the support of legislation for mandatory use of safety restraints. Conservatives might also support such measures, focusing on the individual responsibility of parents and guardians to ensure the safety of their children, and the role of legislation in upholding societal standards of safety.<br><br>Thus, the text reflects a commitment to public health and safety, advocating for practices and legislation aimed at reducing injuries and fatalities in motor vehicle accidents. It is a professional and apolitical stance that emphasizes evidence-based recommendations for the safety of children and adults in vehicles.	neutral
AAFP	National Health Service Corps	7/2023	The text you've provided outlines a position that primarily aligns with a liberal viewpoint, particularly in the context of United States politics. The support for the National Health Service Corps (NHSC), advocacy for reauthorization and appropriate funding, emphasis on loan repayment programs, and the goal of eliminating disparities in healthcare due to race, class, income, geography, language, or immigration status are all elements that are typically associated with liberal or progressive policy positions. These positions emphasize the role of government in providing healthcare, reducing inequalities, and ensuring that all individuals have access to healthcare services regardless of their socio-economic status.<br><br>Liberal viewpoints often prioritize public health initiatives, equitable access to healthcare, and the reduction of disparities in health outcomes. The text's focus on supporting a government program designed to improve access to primary care in underserved communities, along with its emphasis on funding for training the health workforce and addressing disparities, reflects these priorities.<br><br>Conservative viewpoints, on the other hand, might emphasize less government intervention in healthcare, prioritize private sector solutions, or focus on reducing government spending in public health programs. However, it's important to note that there can be crossover in support for specific health initiatives across the political spectrum, and the support for programs like the NHSC might also find backing among some conservatives, especially if the programs are seen as a means to address healthcare provider	liberal

			shortages in a cost-effective manner.<br><br>In summary, the positions outlined in the text you provided are more closely aligned with liberal viewpoints due to their emphasis on government involvement in healthcare, focus on reducing healthcare disparities, and support for funding health workforce training programs.	
AAFP	National Minority Health Month	5/2023	The text you've shared, which mentions the American Academy of Family Physicians recognizing April as National Minority Health Month, seems to be aligned with a non-partisan, health-focused initiative rather than strictly liberal or conservative viewpoints. National Minority Health Month is aimed at promoting improved health outcomes in minority populations and encouraging interest in family medicine. This initiative reflects concerns with public health, health equity, and access to medical care for underserved communities. These issues can be of interest to and supported by people across the political spectrum, although the emphasis on health equity and minority health can sometimes be more closely associated with liberal viewpoints that prioritize social equity and justice. However, the goal of improving health outcomes and ensuring that all communities have access to quality healthcare is a broadly shared value that transcends simple political categorization.	probably liberal
AAFP	Naturopathic Practice	9/2022	The text presents a position focused on medical professionalism, scientific evidence, and patient safety rather than aligning directly with traditional political liberal or conservative viewpoints. It articulates the American Academy of Family Physicians (AAFP)'s stance on the licensure and practice of naturopaths, emphasizing concerns over the scientific basis of naturopathic theory and practice, the adequacy of naturopathic education, and the implications for patient health and safety.<br><br>This position could potentially find support across the political spectrum for different reasons:<br><br>- **Conservative viewpoints** might appreciate the emphasis on maintaining established medical standards and protecting patients from practices not supported by the scientific community, potentially viewing it as a stance against unnecessary expansion of healthcare professions that could increase healthcare costs or undermine traditional medical authority.<br><br>- **Liberal viewpoints** might support the focus on evidence-based practice and patient safety, advocating for rigorous standards in healthcare to ensure all practitioners are adequately trained and qualified, which aligns with a broader commitment to high-quality healthcare for everyone.<br><br>The focus of the AAFP's position is on ensuring that healthcare providers are well-trained and rely on scientifically validated methods, which transcends simple liberal-conservative dichotomies and speaks more to debates within healthcare policy regarding the role and recognition of alternative medicine. The argument is grounded in a concern for the health and safety of patients and the integrity of the healthcare system, rather than in political ideology.	neutral

AAFP	Needle Exchange Programs and Safe Injection Sites	7/1905	The text you provided aligns more closely with a liberal viewpoint for several reasons:<br><br>1. **Support for Harm Reduction Strategies**: Liberal ideologies often emphasize harm reduction approaches to public health issues, including substance use disorders. The focus is on reducing the negative consequences associated with drug use for individuals and communities, rather than solely on the use itself. This contrasts with more conservative viewpoints, which may prioritize abstinence-only approaches and stricter legal penalties for drug use.<br><br>2. **Advocacy for Needle Exchange Programs and Safe Injection Sites**: These programs are typically supported by liberals as part of a broader harm reduction strategy. They are seen as pragmatic solutions to reduce the spread of infectious diseases like HIV and Hepatitis among people who use drugs. Conservative perspectives might view these programs as enabling drug use and may oppose them on moral or legal grounds.<br><br>3. **Emphasis on Treatment and Engagement Over Punishment**: The statement highlights the importance of engaging individuals in treatment for substance use disorders and suggests that harm reduction strategies can increase the likelihood of individuals entering treatment programs. This approach is more aligned with liberal views on health care and drug policy, which often stress the need for compassionate, evidence-based interventions over punitive measures.<br><br>4. **Concern for Vulnerable Populations**: The text mentions providing health and preventive services to "vulnerable and high-risk populations," a focus that aligns with liberal priorities on social justice and equity in health care access. Liberals tend to advocate for policies that address the needs of marginalized groups, including those affected by substance use disorders.<br><br>5. **Legal and Policy Awareness**: The call for physicians to be knowledgeable about state statutes regarding harm reduction strategies suggests an advocacy for evidence-based policy making and a legal framework that supports public health goals. While this could be supported by individuals across the political spectrum, the specific mention of harm reduction aligns more closely with liberal ideologies.<br><br>It's important to note, however, that public health and harm reduction strategies can receive bipartisan support, especially when evidence strongly suggests their effectiveness in saving lives and reducing healthcare costs. The delineation between liberal and conservative viewpoints can sometimes blur in the context of pragmatic health policy solutions.	liberal
AAFP	Neonatal Circumcision	10/2023	The text provided presents a balanced perspective on neonatal circumcision, incorporating elements that could align with viewpoints across the political spectrum rather than strictly adhering to either a liberal or conservative viewpoint.<br><br>1. **Health Benefits and Medical Perspective**: The mention of potential health benefits, such as the prevention of urinary tract infections, reduced risk of penile cancers, and sexually transmitted infections, leans towards a factual, medical perspective that can be supported by both liberal and conservative viewpoints. Emphasizing medical benefits is a common argument used to justify the practice from a public health standpoint, which may not strictly align with political ideologies.<br><br>2. **Safety and Procedural Information**: The emphasis on the safety of the procedure when performed in the newborn period and the low risk of complications is a neutral stance, focusing on procedural aspects rather than cultural or ethical considerations. The mention of providing analgesia for pain management during the procedure is a compassionate consideration that is generally supported across the board.<br><br>3. **Informed Consent and Cultural Sensitivity**: The text highlights the importance of culturally and linguistically appropriate consent and care, which aligns more closely with liberal values that emphasize individual rights, informed consent, and cultural sensitivity. Advocating for informed consent and ensuring families are fully aware of the benefits and risks reflects a respect for autonomy and individual choice.<br><br>4. **Access to Medical Services**: The call for access to circumcision as a covered medical service suggests a viewpoint that supports healthcare accessibility and potentially government or insurance support for medical procedures, which is a stance more commonly associated with liberal ideologies focused on healthcare equity.<br><br>Overall, while the text includes elements that could be appreciated by both liberal and conservative perspectives, the emphasis on informed consent, cultural sensitivity, and healthcare accessibility leans slightly more towards liberal values. However, it's important	probably liberal

			to note that views on neonatal circumcision can vary widely within political groups, and individuals may hold differing opinions on the topic that do not strictly align with their broader political affiliations.	
AAFP	New Physician, Definition	2020	The text you've provided is a factual definition related to the classification of new physicians based on the time since completing their residency or extended training. It specifies that "new physicians are defined as 'those who completed residency or extended training immediately following residency seven years ago or less.'" This definition is procedural and pertains to the medical field, specifically regarding the classification of physicians based on their years of experience post-residency.<br><br>This type of definition does not inherently align with a political viewpoint, whether liberal or conservative. It is a neutral statement that establishes criteria for categorizing physicians based on their experience level. Political viewpoints, whether liberal or conservative, generally pertain to opinions or stances on policy, governance, social issues, and the like, rather than technical definitions within professional fields.<br><br>In summary, the provided text is apolitical and does not align with either a liberal or conservative viewpoint. It serves a specific, practical purpose within the medical community for classification and possibly for policy implementation within that context.	neutral
AAFP	Nuclear Waste Disposal	7/1905	The text you've provided outlines a position by the American Academy of Family Physicians (AAFP) regarding the safe handling, transportation, and storage of nuclear waste, as well as support for continued research into improving the safety and efficiency of nuclear reactors and minimizing exposure to nuclear waste. This statement does not strictly align with a traditional liberal or conservative viewpoint as it primarily focuses on public health and safety rather than broader political or ideological positions.<br><br>Environmental protection and public health safety are concerns that cross traditional political divides, and advocacy for the safe management of nuclear waste and the advancement of nuclear technology can be found within both liberal and conservative circles, albeit for different reasons. Liberals might support these measures due to environmental concerns and the pursuit of sustainable and clean energy alternatives, while conservatives might view advancements in nuclear technology as a means to achieve energy independence and economic growth.<br><br>The AAFP's stance is rooted in a professional commitment to safeguarding public health rather than advancing a specific political agenda. It reflects a pragmatic approach to dealing with the realities of nuclear energy and waste, emphasizing the importance of safety, research, and responsible management.	neutral

AAFP	Nuclear, Biological and Chemical (NBC) Warfare	2020	The text you provided expresses a viewpoint that aligns more closely with what is generally considered a liberal perspective, especially in the context of international relations and disarmament policies. This is because the emphasis on a worldwide moratorium and the elimination of nuclear, biological, and chemical weapons often aligns with principles of multilateralism, international cooperation, and peacekeeping, which are more commonly associated with liberal or progressive political ideologies.<br><br>Liberals tend to advocate for policies that prioritize global security through disarmament, diplomacy, and international treaties to reduce the threat of warfare and the spread of weapons of mass destruction. They often support international institutions and agreements that aim to achieve these goals, seeing them as essential tools for maintaining peace and security on a global scale.<br><br>Conservatives, on the other hand, might be more inclined to emphasize national security interests, military readiness, and the deterrence value of maintaining a strong defense, including the possession of such weapons as a means to prevent aggression from other nations. They may be more skeptical of international agreements that they perceive as potentially limiting a nation's sovereignty or its ability to defend itself.<br><br>However, it's important to note that issues of national and global security can sometimes transcend simple liberal-conservative divides, with individuals and groups within both ideologies sometimes supporting or opposing disarmament measures based on nuanced considerations. The context of the statement, being from the American Academy of Family Physicians, also indicates that the perspective is rooted in concerns for public health and the global implications of the use of such weapons, rather than a strictly political ideology.	liberal
AAFP	Nurse Midwives, Certified	2020	The text reflects a viewpoint that emphasizes regulation, oversight, and integration within the existing medical system for certified nurse midwives (CNMs). It advocates for specific training, certification following an RN license, and the functioning of CNMs under the supervision of licensed physicians. Furthermore, it supports quality assurance in training programs and advocates for payment for CNM services within an integrated practice arrangement.<br><br>This stance aligns more closely with a perspective that values structured oversight, professional standards, and integration within traditional healthcare frameworks, which can be seen as conservative in the context of healthcare policy. Conservative viewpoints often prioritize the maintenance of established professional hierarchies, regulatory standards, and cautious integration of practitioners into the healthcare system to ensure patient safety and maintain professional standards.<br><br>However, it's important to note that the terms "liberal" and "conservative" can vary widely in their application, especially when discussing healthcare policy. In some contexts, advocating for the inclusion and recognition of nurse midwives could be seen as a progressive stance, given that it promotes broader access to maternity care services and recognizes the role of non-physician healthcare providers. The emphasis on strict training and oversight, however, suggests a conservative approach to integrating CNMs into the healthcare system, ensuring they operate within established medical practices and under the supervision of physicians.<br><br>In healthcare debates, a more "liberal" viewpoint might advocate for greater autonomy for CNMs, including less restrictive practice environments and broader scope of practice without the need for physician supervision. This perspective might emphasize expanding access to care through more diverse healthcare provider roles and reducing barriers to practice for nurse midwives.<br><br>The American Academy of Family Physicians (AAFP) policy statement, therefore, represents a conservative approach within the spectrum of healthcare policy debates concerning the role, training, and integration of nurse midwives into the healthcare system. It advocates for a model that maintains traditional professional hierarchies and oversight mechanisms to ensure patient safety and care quality.	conservative

AAFP	Nurse Practitioners	2020	The text reflects a viewpoint that leans more towards what might be traditionally seen as conservative in the context of healthcare policy, specifically regarding the roles and independence of nurse practitioners (NPs) compared to physicians. This perspective emphasizes a structured hierarchy in healthcare, with physicians at the top and nurse practitioners working under their supervision, rather than advocating for the independent practice of NPs. Here are a few reasons why it might be seen as aligning with conservative principles:<br><br>1. **Emphasis on Traditional Roles and Supervision**: The text underscores the importance of maintaining traditional roles within the healthcare system, where nurse practitioners function under the "direction and responsible supervision of a practicing, licensed physician." This approach favors a more hierarchical structure within healthcare professions, which is often aligned with conservative views on professional regulation and roles.<br><br>2. **Caution on Independent Practice**: The statement explicitly opposes the idea of nurse practitioners functioning as independent health practitioners. Conservative viewpoints typically prioritize established norms and caution when it comes to expanding scopes of practice, especially in fields as critical as healthcare, out of concern for patient safety and the maintenance of quality standards.<br><br>3. **Quality Control and Training**: The emphasis on constantly monitoring training programs to assure quality and adjusting the number of graduates to reflect demonstrated needs suggests a careful and measured approach to healthcare education and workforce planning. This could align with conservative perspectives that favor oversight, regulation, and ensuring that changes in professional scopes are justified and based on clear needs.<br><br>However, it's important to note that the discussion around the roles, independence, and regulation of healthcare professionals like nurse practitioners can cross traditional political lines. Stakeholders within the healthcare system, including physicians, nurse practitioners, healthcare organizations, and patients, have diverse opinions on this issue, and their views do not always align neatly with political ideologies. The American Academy of Family Physicians (AAFP), which is represented in the text, focuses on the interests and perspectives of family physicians, which may not directly correspond to broader liberal or conservative political ideologies but rather to the specific professional and patient care standards they advocate for.	conservative
AAFP	Nursing Profession	5/2023	The text provided aligns with a viewpoint that emphasizes collaboration and mutual respect in the healthcare profession rather than a specific political ideology such as liberal or conservative. The statement from the American Academy of Family Physicians (AAFP) focuses on the value of interprofessional relationships between physicians and nurses, advocating for policies that promote cooperation and enhance the quality of patient care. This perspective is centered around healthcare quality and team dynamics rather than political or ideological stances.<br><br>It recognizes the importance of each role within the healthcare system, advocating for a model of care that is comprehensive and patient-centered. Such viewpoints are generally supported across the political spectrum, as they address universal goals of improving healthcare outcomes and fostering positive work environments in clinical settings.<br><br>This approach is in line with broader healthcare policy discussions that focus on improving patient care, healthcare delivery, and professional collaboration, issues that can be supported by individuals with a wide range of political beliefs. Therefore, it's more accurately described as a professional or sector-specific viewpoint rather than being strictly liberal or conservative.	neutral

AAFP	Obesity and Overweight	9/2022	The text you've provided outlines a healthcare policy recommendation focusing on the screening, prevention, and management of obesity through behavioral interventions, medication, and societal efforts to improve nutrition and physical activity. Its stance on involving healthcare providers in obesity management, advocating for insurance coverage of evidence-based interventions, and the inclusion of FDA-approved anti-obesity medications suggests a viewpoint that aligns more closely with liberal policies, particularly in the following aspects:<br><br>1. **Emphasis on Preventative Healthcare**: The focus on screening and preventing obesity-related morbidity and mortality aligns with liberal perspectives that prioritize preventative healthcare measures to improve public health outcomes and reduce long-term healthcare costs.<br><br>2. **Support for Comprehensive Insurance Coverage**: Advocating for public and private insurance plans to cover behavioral interventions and anti-obesity medications reflects a liberal viewpoint on expanding access to healthcare services and treatments. This is consistent with the idea that healthcare should be accessible and affordable for all, regardless of income.<br><br>3. **Regulatory Support for Medication Coverage**: The support for including all classes of FDA-approved anti-obesity medications in insurance formularies aligns with liberal views on the role of regulation in ensuring comprehensive healthcare coverage and the availability of necessary treatments.<br><br>4. **Public Health Initiatives**: Encouraging participation in efforts to improve nutrition and encourage physical activity aligns with liberal views on the importance of community and governmental involvement in public health initiatives.<br><br>While the text promotes healthcare strategies that could be supported across the political spectrum, its emphasis on preventative care, comprehensive insurance coverage, and regulatory support for treatment availability typically aligns more with liberal policy positions. Conservative viewpoints might focus more on personal responsibility for health, market-driven solutions for healthcare access, and limited government intervention in healthcare and insurance markets. However, it's important to note that opinions on healthcare policies can vary widely within both liberal and conservative circles, and the distinction may not always be clear-cut.	liberal
AAFP	Officers' Protocol	1/2022	The text you've provided does not inherently align with either a liberal or conservative viewpoint. It outlines the protocol for listing officers of the American Academy of Family Physicians (AAFP), including positions such as President, President-elect, Chair of the Board of Directors, Speaker of the Congress of Delegates, Vice Speaker of the Congress of Delegates, and Executive Vice President. This information pertains to organizational structure and procedural formalities rather than expressing ideological beliefs or political positions.<br><br>In general, the listing of officers or organizational structures is administrative and neutral in terms of political ideology. It is more about governance and the operational framework of the organization rather than a statement of political or ideological values.	neutral
AAFP	Opposition to Restrictive Covenants	10/2023	The text you've provided outlines a position on restrictive covenants in physician employment contracts, emphasizing the importance of maintaining the patient-physician relationship and patient access to care. It criticizes non-compete clauses that limit a patient's access to their physician and restricts patient choice due to geographic, time, and scope of practice constraints when a physician leaves a practice.<br><br>This perspective aligns more closely with principles often associated with a liberal viewpoint, especially in its emphasis on individual rights (in this case, patient rights) and access to services (healthcare, in particular). Liberal viewpoints typically prioritize ensuring equitable access to essential services, including healthcare, and may advocate for regulations or policies that protect consumer and individual rights against what are seen as overly restrictive business practices.<br><br>However, it's important to note that issues of healthcare access and the dynamics of patient-physician relationships can transcend simple political categorizations. Advocates from various political backgrounds might find common ground on the importance of maintaining continuity of care and ensuring patients have unimpeded access to their chosen healthcare providers. The focus on patient rights and access to care could be supported by individuals across a broad political spectrum who prioritize these values within the healthcare system.<br><br>Moreover, the stance against restrictive covenants might also appeal to those with a conservative viewpoint who value free market principles, arguing that such restrictions interfere with competition and the ability of professionals to move freely within the market.<br><br>In summary, while the text might more directly align with liberal priorities regarding healthcare access and patient rights, the	liberal

			underlying issues it addresses—professional freedom, patient access to care, and opposition to restrictive practices—can resonate across a broader political spectrum.	
AAFP	Oral Health	9/2022	The text provided outlines positions and recommendations by the American Academy of Family Physicians (AAFP) regarding dental services, emphasizing the importance of oral health, the interconnection between oral health and general health, the support for fluoridation of public water supplies, and the advocacy for addressing oral health disparities. These viewpoints focus on public health measures, preventive care, and the integration of healthcare services to improve health outcomes.<br><br>These perspectives align more closely with liberal viewpoints, especially in regard to public health policy and addressing health disparities. Liberal viewpoints typically emphasize the role of government and public policy in ensuring access to healthcare, preventive services, and addressing social determinants of health to reduce inequalities. The support for fluoridation of public water supplies as a preventive measure against dental caries and the emphasis on education and advocacy for equal access to dental services reflect a public health approach that aims to improve health outcomes at the population level, which is consistent with principles often associated with liberal policies.<br><br>Moreover, the focus on educating physicians and the public about oral health, the encouragement of collaboration between different healthcare professionals, and the advocacy for measures that address health disparities underscore a comprehensive approach to health care that seeks to improve access and quality of care for all, another hallmark of liberal health policy perspectives.<br><br>However, it's important to note that public health and preventive care can also receive support across the political spectrum, especially when the measures are proven to be cost-effective and have a significant impact on improving health outcomes. The emphasis on evidence-based practice, such as the systematic review on water fluoridation, shows a commitment to scientific evidence which transcends political ideologies.	liberal

AAFP	Organ Donation: Addressing the Shortage of Registered Organ Donors	9/2022	The text provided outlines a position from the American Academy of Family Physicians (AAFP) regarding the shortage of donor organs and suggests strategies to increase organ donation. This position does not neatly align with traditional liberal or conservative political viewpoints as it primarily addresses public health and ethical considerations rather than partisan issues.<br><br>However, certain elements of the AAFP's position may resonate differently across the political spectrum:<br><br>1. **Support for expanding organ donation through education and dialogue:** This approach emphasizes informed choice and respect for individual beliefs and preferences. It can be seen as a non-partisan or bipartisan approach, promoting public health without coercing individuals, a principle that can be appreciated across political lines.<br><br>2. **Advocacy for opt-out (presumed consent) programs and mandated choice policies:** These strategies may be more controversial, as they involve a degree of government intervention in personal decisions. Presumed consent and mandated choice policies could be seen as aligning more closely with liberal viewpoints that are generally more supportive of government involvement in promoting public health and welfare. Conservatives might be more skeptical of such approaches, emphasizing personal freedom and minimal government intervention.<br><br>3. **Attention to disparities affecting minorities:** The mention of the disproportionate impact of organ shortages on minorities and the call to address these disparities might align more with liberal perspectives, which often emphasize equity and social justice issues. Conservative viewpoints might also recognize the importance of addressing these disparities but may propose different methods for doing so, focusing more on individual choice and less on systemic interventions.<br><br>Overall, while the text addresses a public health issue that is not inherently political, aspects of the proposed solutions might resonate differently with individuals depending on their political beliefs. The focus on education, informed consent, and addressing disparities is generally consistent with a humanitarian perspective that transcends simple liberal-conservative categorizations.	neutral
AAFP	Over-the-Counter Oral Contraceptives	7/1905	The text provided aligns more closely with a liberal viewpoint. The emphasis on reducing barriers to access for contraception, including support for over-the-counter access to oral contraceptives and insurance coverage regardless of prescription status, reflects priorities typically associated with liberal perspectives on public health and women's reproductive rights. Liberal viewpoints often focus on increasing access to healthcare services and supporting preventative care measures, which include access to contraception to manage and reduce unintended pregnancies.<br><br>The support for over-the-counter access to oral contraceptives without a prescription and advocating for insurance coverage of these contraceptives align with efforts to make healthcare more accessible and equitable. These positions are consistent with liberal policies that emphasize individual rights and the importance of accessible healthcare options to improve public health outcomes.<br><br>Conservative viewpoints, on the other hand, may place a greater emphasis on regulatory oversight of healthcare services and might have more reservations about making prescription medications available over the counter without a prescription. Additionally, conservative positions might focus more on abstinence education and less on expanding access to contraceptives as a means of addressing unintended pregnancies.<br><br>The text's references to studies and global practices further support its argument for increasing access to contraceptives, showcasing an evidence-based approach to public health policy that is often characteristic of liberal viewpoints.	liberal
AAFP	Paid Sick Leave	5/2023	The text you provided advocates for the provision of paid sick leave by all employers for employees to use when they or a family member are unwell, along with protection for employees from retaliation for using sick leave. This viewpoint aligns more closely with liberal ideologies, which often emphasize workers' rights, social welfare policies, and protections for employees in the workplace. Liberal perspectives generally support the idea that the government or employers should provide benefits like paid sick leave to ensure that workers do not have to choose between their health (or their family's health) and their job.<br><br>Conservative ideologies, on the other hand, tend to prioritize free market principles, limited government intervention in business practices, and personal responsibility. From a conservative perspective, mandates on businesses, such as requiring paid sick leave, might be seen as overreach that could burden businesses, especially small ones, and stifle economic freedom and growth.<br><br>However, it's important to note that viewpoints on specific policies like paid sick leave can vary widely within both ideological spectrums, and there may be conservatives who support such measures under certain conditions, just as there may be liberals who have concerns about the implementation or impact of such policies.	liberal

AAFP	Parental Leave During Residency Training	9/2022	The text outlines a comprehensive approach to parental leave within family medicine residency programs, emphasizing the health and well-being of the parent and child, educational requirements, and uninterrupted patient care. It doesn't squarely align with traditional liberal or conservative viewpoints as understood in the political sense but incorporates elements that could be appreciated across the political spectrum.<br><br>From a **liberal viewpoint**, the emphasis on paid parental leave, equality in leave benefits for expectant and adoptive parents, and the provision of insurance benefits during leave align with a focus on worker rights, equality, and support for families. Liberals typically advocate for policies that support work-life balance, gender equality, and comprehensive benefits for workers, which this text supports by advocating for up to 12 weeks of paid parental leave and equal treatment for expectant and adoptive parents.<br><br>From a **conservative viewpoint**, the aspects of the text that ensure compliance with educational requirements and uninterrupted patient care might resonate more. Conservatives often emphasize personal responsibility and the importance of fulfilling one's duties. The text's focus on making sure residency education is not compromised and that patient care continues seamlessly during a resident's absence could align with these values. Additionally, the text's emphasis on compliance with certifying boards and laws like the Family and Medical Leave Act (FMLA) might also find support in conservative circles for its adherence to existing legal frameworks and standards.<br><br>Overall, the text represents a balanced approach, aiming to support the needs of residents during significant life events while ensuring the quality and continuity of medical education and patient care. Such an approach demonstrates a pragmatic blend of ensuring individual rights and meeting professional obligations, which transcends simple political categorization.	neutral
AAFP	Patient Care, Concurrent	7/1905	The text you provided describes a specific aspect of healthcare policy and practice, namely the provision and billing of concurrent care, without explicitly aligning with traditional liberal or conservative viewpoints. This text is primarily technical and procedural, focusing on the importance of ensuring that when multiple healthcare professionals provide necessary care to the same patient on the same day, this care should be appropriately recognized and reimbursed.<br><br>Liberal viewpoints in healthcare often emphasize the need for accessible, comprehensive healthcare services for all, focusing on the expansion of healthcare coverage and the protection of patient rights. Conservatives might stress the importance of minimizing government involvement in healthcare, advocating for market-driven solutions to healthcare provision and efficiency.<br><br>The statement about concurrent care does not explicitly advocate for a broad healthcare policy change or discuss the role of government versus market-driven solutions in healthcare. Instead, it argues from a standpoint of medical necessity and the operational aspect of healthcare provision—ensuring that when multiple professionals provide essential care, the system should support appropriate compensation for such services.<br><br>Therefore, while the call for appropriate payment for medically necessary concurrent care might resonate with broader healthcare principles both liberals and conservatives could agree on—such as efficient, high-quality patient care—the text does not directly advocate for or against the broader policy positions that typically distinguish liberal from conservative viewpoints. It focuses on best practices within the existing healthcare system rather than suggesting systemic reforms.	neutral
AAFP	Patient Education	1/2022	The text you've provided outlines a viewpoint on patient education and the role of family physicians in promoting public health through accurate, evidence-based, and culturally proficient education. This viewpoint emphasizes the importance of inclusivity and evidence-based practice in healthcare.<br><br>This perspective aligns more closely with principles often associated with liberal viewpoints, particularly in its emphasis on inclusivity, cultural proficiency, and evidence-based practices. Liberal perspectives typically advocate for policies and practices that ensure healthcare is accessible, equitable, and informed by the best available evidence to meet the diverse needs of the population. The mention of culturally proficient patient education reflects a commitment to addressing health disparities and ensuring that healthcare practices are inclusive and respectful of diverse cultural backgrounds, which is a priority often emphasized in liberal healthcare policies.<br><br>However, it's important to note that advocating for evidence-based, inclusive healthcare practices can also be supported by individuals with conservative viewpoints, especially when the focus is on improving health outcomes and ensuring the efficient use of resources. The distinction often lies in the approach and underlying values rather than the objective of improving health education and outcomes per se.	probably liberal

			In contemporary political discourse, the lines between liberal and conservative viewpoints on healthcare can sometimes blur, especially on issues where there is broad consensus on goals like improving health outcomes and efficiency, even if there are differences in preferred methods to achieve these goals. The specific emphasis on cultural proficiency and inclusivity in this text makes it more aligned with liberal viewpoints, but the fundamental goal of improving health through education is widely recognized across the political spectrum.	
AAFP	Patient Referrals	2020	The text provided aligns more closely with viewpoints that could be considered liberal rather than conservative, particularly in the context of healthcare policy debates in the United States. This alignment is due to several key factors:<br><br>1. **Emphasis on Patient Choice and Transparency**: The text highlights the importance of patients having the freedom to select their healthcare providers and settings, along with a strong call for transparency in coverage, network details, and costs. These principles are often associated with efforts to improve patient rights and access to healthcare, themes commonly found in liberal healthcare policy proposals.<br><br>2. **Support for Evidence-Based Care**: The mention of physicians having the freedom to provide care based on patient values, evidence-based care, quality, and value reflects a commitment to scientific standards and quality care over purely market-driven or cost-based considerations. This approach is more aligned with liberal perspectives that advocate for healthcare quality and accessibility as priorities.<br><br>3. **Responsibility of Healthcare Providers**: The text asserts that physicians receiving referrals have a responsibility for the patient care plan and the quality of care, emphasizing the ethical responsibilities of healthcare providers. This focus on accountability and ethical standards in healthcare aligns with liberal values of ensuring high-quality and responsible healthcare delivery.<br><br>4. **Acknowledgment of Special Circumstances**: Recognizing that there may be limitations on choice in certain situations but insisting that these be clearly explained and free from coercion suggests a nuanced understanding of healthcare access. This perspective aligns with liberal views that recognize the complexities of healthcare and the need for protections against undue influence or limitation on patient rights.<br><br>However, it's important to note that the concepts of patient choice, transparency, and quality care are not exclusively liberal and can be supported by conservatives under certain frameworks, especially those that emphasize market-driven solutions and individual freedoms. The distinction often lies in the mechanisms proposed to achieve these goals and the degree of regulation or government involvement deemed appropriate.<br><br>In the current polarized healthcare debate in the U.S., this statement from the AAFP would likely be more embraced by those with liberal viewpoints, given its emphasis on patient rights, transparency, and the ethical responsibilities of healthcare providers within a framework that supports access and informed choice.	liberal

AAFP	Patient-Centered Formularies	7/1905	The text provided outlines the American Academy of Family Physicians (AAFP)'s position on various aspects of pharmaceutical products, their prescription, and management within healthcare systems. It is not strictly aligned with a liberal or conservative viewpoint as defined in political terms. Instead, it addresses practical and ethical considerations relevant to medical practice, patient care, and the pharmaceutical industry. The stances taken reflect a balanced approach to ensuring effective, accessible, and cost-efficient healthcare, while also recognizing the importance of pharmaceutical innovation and the complexities of drug formulary management.<br><br>Key aspects such as supporting access to needed pharmaceutical products, recognizing the role of restrictive formularies in optimizing clinical outcomes while minimizing costs, concerns about direct-to-consumer advertising, and the emphasis on evidence-based, patient-centered formulary design transcend simple political categorizations. These positions aim to navigate the intricacies of healthcare provision, balancing cost, efficacy, and quality of care in a manner that could find support across a broad spectrum of political and ideological beliefs.<br><br>Moreover, the concerns over potential conflicts of interest and the emphasis on transparency, collaboration, and patient safety indicate a commitment to ethical practices and accountability in healthcare. Such principles are widely advocated across the political spectrum, although the specifics of implementation may be debated within different ideological frameworks.<br><br>In summary, the AAFP's positions as outlined are focused on healthcare quality, access, and ethics, areas that are of concern to both liberal and conservative perspectives, but they are addressed in a way that seeks to find practical and equitable solutions rather than aligning with a specific political ideology.	neutral
AAFP	Payer Acceptance of Supplemental Data	2019	The text provided outlines a policy position of the American Academy of Family Physicians (AAFP) regarding the operations of health plans, specifically in the context of value-based contracts and the submission of supplemental data by physicians. This position does not neatly align with traditional liberal or conservative viewpoints as understood in a political context, because it primarily addresses administrative and operational issues in healthcare delivery and payment systems rather than broader ideological or political themes.<br><br>However, some aspects of this position could be interpreted through the lens of broader healthcare policy debates, which often do have ideological underpinnings:<br><br>1. **Liberal Viewpoint Considerations**: From a liberal perspective, the emphasis on ensuring physicians have the ability to submit comprehensive data to health plans might be seen as aligning with a preference for thorough, equitable healthcare systems that prioritize patient care and provider fairness. Liberals often advocate for healthcare reforms that increase access and quality of care, and ensuring accurate payment to providers for value-based care could be viewed as part of this broader agenda.<br><br>2. **Conservative Viewpoint Considerations**: Conservatives might focus on the aspects of this position that relate to efficiency, transparency, and the proper functioning of market-based mechanisms within the healthcare system. The emphasis on allowing physicians to submit supplemental data can be seen as a way to ensure that value-based payment systems, which are designed to incentivize high-quality care, operate as intended without bureaucratic obstacles.<br><br>The text essentially advocates for practical improvements in the healthcare payment system to ensure it works as intended for both providers and patients. While the underlying value-based payment model can be debated on ideological grounds—with conservatives potentially favoring it for its market-driven approach to improving healthcare quality and liberals possibly supporting it for its focus on outcomes and patient care—the specific policy position outlined here is more technical and operational in nature, transcending simple liberal-conservative dichotomies.	neutral

AAFP	Payment for Non Face-to-Face Physician Services	2020	The text you've provided outlines a policy stance by the American Academy of Family Physicians (AAFP) regarding physician payment for electronic communications and evaluations, emphasizing that payment should be based on the medical necessity and appropriateness of the services rather than the technology used to deliver them. This viewpoint does not neatly align with traditional political categories of "liberal" or "conservative" as it focuses on healthcare policy and the economics of medical practice rather than broader political ideologies.<br><br>However, some aspects of this stance could be interpreted through the lens of larger political and policy debates:<br><br>1. **Support for Innovation in Healthcare Delivery**: The AAFP's support for payment for electronic communication and evaluations could be seen as endorsing innovation and flexibility in healthcare delivery, principles that can be supported by both liberal and conservative viewpoints, albeit for different reasons. Liberals might support this as part of broader access to healthcare services, while conservatives might see it as a way to improve efficiency and reduce costs.<br><br>2. **Emphasis on Medical Necessity over Technology**: The statement's focus on medical necessity and appropriateness, regardless of the delivery method, underscores a patient-centered approach to healthcare. This can resonate with liberal perspectives that prioritize healthcare access and equity. However, it also aligns with conservative views that emphasize the importance of reducing unnecessary spending and focusing on effective care.<br><br>3. **Healthcare Payment Reform**: The idea of changing how healthcare services are paid for, including supporting separate payments for electronic communications, touches on broader debates about healthcare reform. Liberals may view this as a step towards more comprehensive healthcare coverage and access, especially in underserved areas. Conservatives might support it if they see it as a market-driven solution that enhances competition and consumer choice.<br><br>In conclusion, while the AAFP's position is specific to healthcare policy and not overtly political, its implications can be interpreted in ways that resonate with both liberal and conservative perspectives, depending on one's focus—be it innovation, efficiency, patient-centered care, or healthcare access and equity. The stance is more about advocating for the evolution of payment models in healthcare to keep pace with technological advancements and changing medical practices, a concern that crosses traditional political boundaries.	neutral
AAFP	Payment, Lifestyle Medicine	2020	The text outlines the American Academy of Family Physicians (AAFP)'s recognition of the importance of lifestyle interventions in the prevention and management of common conditions seen in family medicine, and its support for payment for evidence-based lifestyle medicine interventions in physician practices. This position does not explicitly align with a traditional political spectrum of liberal or conservative viewpoints. Instead, it reflects a medical and health-focused perspective prioritizing evidence-based approaches to health care and prevention.<br><br>Healthcare policy and the emphasis on preventive medicine, including lifestyle interventions, can be supported across the political spectrum. Liberals might emphasize the role of healthcare systems in providing comprehensive care and preventive services as part of a broader social safety net, potentially supporting such interventions through public funding or insurance mandates. Conservatives might support the idea of preventive care to reduce long-term healthcare costs, but they might prefer solutions that involve less government intervention and more market-based approaches to healthcare.<br><br>The focus on "evidence-based" interventions is a hallmark of modern medical practice, emphasizing the importance of scientific research in guiding healthcare decisions, which is a principle that transcends political ideology. The support for payment for these interventions suggests an interest in integrating them into the healthcare payment system, which could resonate with both liberal and conservative viewpoints, depending on the proposed implementation method.<br><br>In summary, the text promotes a health-first perspective that advocates for the integration of scientifically supported lifestyle interventions into the healthcare system, a stance that can find support across different political ideologies depending on the context and proposed implementation strategies.	neutral

AAFP	Payment, Non-Physician Providers	2020	The text you've provided advocates for equal quality and reimbursement for services provided by non-physician providers under physician supervision as those directly provided by physicians. This viewpoint can be interpreted through both liberal and conservative lenses, depending on the context and underlying values being emphasized.<br><br>From a **liberal viewpoint**, this statement could align with principles of equality, access to care, and the importance of a diverse healthcare workforce. Liberals might argue that ensuring non-physician providers are reimbursed at the same level as physicians for comparable services could help address healthcare disparities, increase access to care, and recognize the valuable contributions of all healthcare providers. This approach could be seen as a way to support a more inclusive and equitable healthcare system.<br><br>From a **conservative viewpoint**, the emphasis might be placed on the quality of care and the efficiency of healthcare delivery. Conservatives might support the idea if it is seen as a way to maintain high standards of care while also potentially reducing costs, as long as it does not interfere with market principles or lead to increased government regulation. They might also emphasize the role of physician supervision in ensuring the quality of care provided by non-physician providers.<br><br>In summary, the statement can align with values from both political spectrums depending on the specific arguments and emphasis. It touches on issues of healthcare quality, efficiency, and equity, which are of concern to both liberals and conservatives, albeit for potentially different reasons.	neutral
AAFP	Payment, Physician	10/2023	The text provided outlines the position of the American Academy of Family Physicians (AAFP) on the principles that should guide a reliable payment system for healthcare. This position emphasizes quality care, access to care, the importance of the physician/patient relationship, prevention, health maintenance, early diagnosis and treatment, appropriate payment for cognitive services, and the need for flexibility and annual reevaluation of payment systems. It also stresses the need for payment systems to recognize the value of primary care, ensure equitable payment, and support continuous comprehensive patient care, including mental health services.<br><br>These principles generally align more with a liberal viewpoint in the context of American politics, for several reasons:<br><br>1. **Emphasis on Access and Quality of Care:** The focus on ensuring access to quality care for all, regardless of economic status or other factors, mirrors liberal priorities of inclusivity and healthcare as a right rather than a privilege.<br><br>2. **Support for Preventative Care and Comprehensive Services:** The emphasis on prevention, health maintenance, and comprehensive care reflects a public health approach that seeks to mitigate healthcare costs and improve health outcomes through early intervention, which is a stance often supported by liberal policies.<br><br>3. **Recognition of the Social Determinants of Health:** The acknowledgment of the need to adjust payment systems to cater to underserved needs or areas suggests an understanding of the social determinants of health, a concept widely acknowledged and advocated for in liberal health policy circles.<br><br>4. **Equitable Payment and Anti-Discrimination:** The call for equitable payment and non-discrimination in physician payment, including considerations for race, color, religion, gender identity, and other factors, aligns with liberal values of equity, diversity, and inclusion.<br><br>5. **Flexibility and Physician Autonomy:** While the desire for flexibility in payment systems and the autonomy for physicians to set their own charges may seem more market-oriented, the context here is about ensuring the system is responsive to varied medical needs and circumstances, which can be seen as ensuring a patient-centered approach, another key aspect of liberal healthcare perspectives.<br><br>However, it's important to note that healthcare policy is complex, and there can be overlap in the priorities and values of different political ideologies when it comes to specific policy proposals or principles. The focus on efficiency, effectiveness, and sustainability in healthcare delivery is a concern across the political spectrum, though the approaches to achieving these goals may differ.	liberal

AAFP	Peer Review	1/2022	The text you provided appears to be centered around the principles of medical ethics, quality of patient care, and the professional development of family physicians through peer review. It does not explicitly align with either a liberal or conservative political viewpoint. Instead, it focuses on the importance of evidence-based care, accountability, and continuous improvement in the healthcare sector.<br><br>Here are some reasons why the text does not align specifically with a liberal or conservative viewpoint:<br><br>1. **Evidence-Based Care and Quality Assurance**: The emphasis on evidence-based care, quality patient services, and peer review is a matter of professional practice in medicine. Both liberal and conservative viewpoints generally support the idea of improving healthcare quality, albeit they might differ on the methods to achieve it.<br><br>2. **Professional Development and Accountability**: The text's focus on the role of family physicians in leading and participating in peer review processes for the improvement of patient care is a professional standard. It advocates for accountability and professional development within the medical community, principles that are widely accepted across the political spectrum.<br><br>3. **Privacy and Transparency**: The call for confidentiality in peer review, alongside transparency in decision-making processes, reflects a balance between the privacy of medical professionals and the public's right to accountability in healthcare. This balance of interests is a common concern in both liberal and conservative discussions about healthcare, but the specific stance taken here is more about ethical medical practice than political ideology.<br><br>4. **Community Sensitivity and Cultural Competence**: Encouraging care that is sensitive to community needs, culture, and practices is a principle that transcends political divides, emphasizing the importance of personalized and culturally competent healthcare.<br><br>In summary, the text promotes ideals of professionalism, accountability, and continuous improvement in healthcare, which are broadly accepted across the political spectrum. It is more aligned with medical ethics and professional standards than with any specific political ideology.	neutral
AAFP	Performance Measures Criteria	7/1905	The text presents a viewpoint that is largely neutral regarding traditional liberal vs. conservative political divides, focusing instead on principles of healthcare quality improvement, evidence-based practice, patient-centered care, and the practical application of performance measures in healthcare settings. It emphasizes the importance of improving healthcare outcomes through scientifically grounded, equitable, and practical performance measures, which is a concern that spans across the political spectrum.<br><br>However, certain aspects of the text may align more closely with principles traditionally supported by one political ideology over another:<br><br>1. **Liberal Viewpoint Alignment**:<br>- The emphasis on equity, including adjustments for social determinants of health and not masking disparities due to race, ethnicity, socioeconomic status, or gender, aligns with liberal priorities around healthcare equity and addressing systemic inequalities.<br>- The focus on transparency, patient-centeredness, and inclusivity in healthcare measures reflects a liberal inclination towards ensuring healthcare systems are accountable to the needs and preferences of all patients, including marginalized and diverse populations.<br><br>2. **Conservative Viewpoint Considerations**:<br>- The text's emphasis on cost-effectiveness, reducing undue financial burdens, and ensuring that performance improvement efforts are justified by impacts on patient outcomes could resonate with conservative values focused on efficiency, fiscal responsibility, and minimizing regulatory or financial burdens on healthcare providers.<br>- The mention of harmonization across all payers and the practicality of measures might appeal to conservative preferences for streamlined, less bureaucratic systems that avoid redundancy and encourage private sector efficiency.<br><br>Overall, the document is more centered on advancing healthcare quality and efficiency, principles that both liberals and conservatives might find valuable, albeit for different reasons. The approach to healthcare improvement it advocates is comprehensive, considering the importance of evidence-based practices, equity, and practicality, which suggests a balanced	neutral

			perspective aimed at broad improvements in health outcomes and system performance rather than aligning with a specific political ideology.	
AAFP	Perinatal Ultrasound Examination	10/2023	The text provided primarily focuses on medical practices and education related to perinatal care and the use of ultrasound in family medicine. It outlines the importance of ultrasound examinations in various stages of pregnancy, the types of care provided, and the training and documentation recommended for family physicians. This information is presented in a neutral and factual manner, aimed at improving patient care and medical practice in the field of family medicine.<br><br>This text does not inherently align with a political viewpoint, liberal or conservative, as it discusses medical practices and standards without delving into political or ideological debates surrounding issues like reproductive rights or healthcare policy. It emphasizes the importance of training and competence in performing ultrasound examinations for pregnancy care, which is a matter of medical education and practice rather than political ideology.<br><br>The focus on enhancing diagnostic and therapeutic capabilities, the importance of training in family medicine residency programs, and the recommendations for documentation and demonstration of proficiency are all centered on improving patient care and outcomes. Such objectives transcend political affiliations, aiming instead to contribute to the field of family medicine and the care of pregnant individuals through evidence-based practices.<br><br>Therefore, while the discussion of healthcare practices can sometimes intersect with political discussions, particularly in areas such as access to care and healthcare policy, the text itself does not advocate for a specific political ideology but rather focuses on medical education and practice standards.	neutral

AAFP	Pharmacists	10/2023	The text you've provided outlines the American Academy of Family Physicians (AAFP)'s position on the role of pharmacists in healthcare, particularly in the context of collaborative practice agreements and the scope of pharmacists' authority in patient care and medication management. This position does not align neatly with a purely liberal or conservative viewpoint, as these terms are traditionally understood in the political spectrum. Instead, it reflects a professional and healthcare policy perspective that prioritizes integrated care teams, quality of healthcare delivery, and patient safety, while also delineating the professional boundaries between pharmacists and physicians.<br><br>However, one might interpret the AAFP's stance through the lens of broader healthcare policy debates that often divide liberal and conservative viewpoints:<br><br>1. **Support for Collaborative Practice Agreements**: The emphasis on integrated care teams and collaborative practice could be seen as aligning with more progressive or liberal views on healthcare, which often advocate for multidisciplinary approaches to healthcare delivery and comprehensive patient care. This perspective values the contribution of various healthcare professionals and supports systems that facilitate teamwork and shared expertise.<br><br>2. **Opposition to Independent Prescription Authority for Pharmacists**: The resistance to expanding pharmacists' scope to include independent prescription authority and opposition to dispensing medication beyond the expiration of the original prescription (except for emergencies) could be interpreted as a conservative approach, prioritizing the traditional roles and safeguards within the healthcare system. This viewpoint might emphasize the importance of maintaining established professional hierarchies and protocols to ensure patient safety and the integrity of medical practice.<br><br>3. **Healthcare Cost Considerations**: Advising patients about lower-cost alternatives for prescription medications reflects a concern for the affordability of care, a priority that crosses the political spectrum but is often more emphasized in liberal healthcare policy discussions. This focus aligns with efforts to make healthcare more accessible and affordable for patients.<br><br>Overall, the AAFP's position is best understood within the context of healthcare professionalism and policy, focusing on what is believed to best serve patient care and safety rather than fitting neatly into a political ideology. The distinctions made between the roles of healthcare providers reflect considerations of professional expertise, patient safety, and the effective delivery of healthcare services rather than a clear-cut alignment with liberal or conservative political ideologies.	neutral
AAFP	Pharmacists Dispensing Drugs - AAFP Legislative Stance	7/2023	The text you've shared mentions the American Academy of Family Physicians (AAFP) encouraging its state chapters to oppose legislation that would allow pharmacists to dispense medication beyond the expiration of the original prescription, except for emergencies. This stance by the AAFP can be interpreted in relation to liberal or conservative viewpoints, but it's important to consider that health policy issues often transcend simple political dichotomies.<br><br>- **Liberal Viewpoint**: From a liberal perspective, this stance might be seen as prioritizing patient safety and the importance of physician oversight in medication management. Liberals often advocate for strong regulatory frameworks to ensure public safety and may view the physician's role in prescribing medication as integral to patient care. Thus, opposing such legislation could align with a liberal viewpoint that emphasizes the need for healthcare regulations that protect patient health.<br><br>- **Conservative Viewpoint**: From a conservative perspective, this position might be interpreted differently. Conservatives generally favor less regulation and more personal freedom, which could include supporting policies that allow pharmacists more discretion in dispensing medications, especially in scenarios that could avoid treatment interruptions. However, conservatives also emphasize personal responsibility and the importance of professional judgment, which in this case, supports the idea that only medical professionals (e.g., physicians) should make decisions about extending prescriptions.<br><br>In reality, health policy issues like this one can find supporters and detractors across the political spectrum, depending on how the issues are framed and what values are prioritized. The AAFP's stance seems to prioritize patient safety and the preservation of the doctor-patient relationship in medication management, which could align with values across the political spectrum. It's also important to consider that professional medical organizations often base their positions on clinical evidence and patient safety considerations rather than on political ideologies.	neutral

AAFP	Pharmacists' Right of Conscientious Objection	10/2023	The text from the American Academy of Family Physicians (AAFP) outlines a balanced approach to handling pharmacists' conscientious objections to filling prescriptions, emphasizing the importance of patient access to legally prescribed and medically indicated treatments while also accommodating the personal beliefs of pharmacists. This position does not neatly align with traditional liberal or conservative viewpoints as it seeks to respect and protect the interests of both patients and healthcare providers.<br><br>- **Liberal Viewpoint Alignment**: The emphasis on ensuring patient access to treatment and the requirement for governmental policies to protect patients' rights to obtain legally prescribed medications align with liberal perspectives on healthcare accessibility and the prioritization of patient rights. Liberals typically advocate for robust healthcare policies that ensure individuals have access to necessary medical treatments without undue barriers.<br><br>- **Conservative Viewpoint Considerations**: The acknowledgment of pharmacists' right to conscientious objection and the effort to accommodate these personal beliefs could be seen as aligning with more conservative viewpoints that value individual conscience and religious freedom. Conservatives often emphasize the importance of protecting individual rights, including the right to act according to one's moral or religious beliefs, especially in the context of healthcare.<br><br>This stance by the AAFP attempts to navigate a middle ground that respects and addresses the concerns of both sides of the debate by:<br>1. Protecting the patient's right to access necessary medical treatments.<br>2. Recognizing the personal beliefs of healthcare providers like pharmacists.<br>3. Implementing a practical framework for addressing situations where these rights and beliefs intersect, ensuring transparency and timeliness in resolving any conflicts.<br><br>The text represents a nuanced position that acknowledges the complexities of healthcare delivery, emphasizing the need for policies that balance individual rights with the imperative of accessible, quality care for all patients.	neutral
AAFP	Physical Activity in Children	5/2023	The text provided presents a viewpoint focused on public health and wellness, particularly emphasizing the importance of regular physical activity for children and adolescents. It is more aligned with a non-partisan, evidence-based perspective rather than strictly liberal or conservative viewpoints. Both liberal and conservative perspectives can support the promotion of public health measures, though they may differ in their approaches to implementation and policy emphasis.<br><br>This text emphasizes the importance of:<br>- Regular physical activity for healthy growth and development.<br>- The correlation between physical activity and various health benefits.<br>- The role of family physicians in promoting physical activity within communities and beyond individual behavior-change strategies.<br>- The need to address environmental factors that influence opportunities for healthy activities.<br><br>Such a stance is grounded in public health research and advocacy, aiming to improve health outcomes across populations. It advocates for a collaborative approach involving healthcare professionals, families, and educational institutions to create environments conducive to physical activity. This approach can appeal to a broad spectrum of political beliefs, focusing on the tangible benefits of exercise for physical, cognitive, and psychological health.<br><br>While liberal viewpoints might emphasize the role of government and public policy in creating supportive environments for health, including infrastructure for physical activity and educational programs, conservative viewpoints might focus on individual responsibility and community-level initiatives. However, the text itself is centered on health promotion and does not explicitly align with the ideological foundations of either political spectrum, illustrating a universal concern for public health and wellness.	neutral

AAFP	Physician and Patient Relationships, Professional Responsibility	7/1905	The text you've shared expresses a viewpoint that emphasizes individual freedom and moral autonomy, particularly for the physician. It highlights the importance of a trusting relationship between physician and patient, and it asserts that physicians should not be compelled to act against their own judgment or moral principles. When it comes to categorizing this viewpoint as "liberal" or "conservative," it's a bit nuanced:<br><br>1. **Conservative Perspective**: The emphasis on individual moral principles and the right of the physician to withdraw from providing care that conflicts with those principles align with conservative values, especially regarding the protection of individual rights and moral autonomy. Conservatives often stress the importance of personal freedom and the protection of individual conscience, especially in the context of professional obligations and ethical standards.<br><br>2. **Liberal Perspective**: Liberals might support the idea of a mutually trusting and satisfactory relationship between physician and patient and might also agree with the principle that good medical care requires respect for both parties. However, liberals might be more inclined to emphasize the rights and access of patients to various forms of medical care, potentially advocating for policies that ensure no patient is denied necessary treatments based on the personal beliefs of individual healthcare providers.<br><br>The text primarily aligns with principles that could be appreciated by both sides for different reasons but leans more towards a conservative viewpoint in its emphasis on the physician's right to act according to personal judgment and moral principles, even if it means withdrawing from providing certain treatments. This aspect of the statement highlights a conservative inclination towards protecting individual liberties and moral autonomy over a broader mandate for universal access to all forms of medical care regardless of the provider's personal beliefs.	probably conservative
AAFP	Physician Assistants	7/1905	The text provided outlines the American Academy of Family Physicians' (AAFP) position on the role and integration of physician assistants (PAs) within the healthcare system. It emphasizes the importance of accreditation, certification, and supervised practice arrangements, as well as supporting payment for services rendered by PAs. This stance does not neatly align with traditional political ideologies such as liberal or conservative in a direct manner. Instead, it focuses on professional standards, patient care quality, and healthcare system efficiency.<br><br>However, aspects of the AAFP's position can be interpreted through the lens of broader healthcare policy debates, which often intersect with liberal and conservative viewpoints:<br><br>1. **Emphasis on Team-Based Care and Integration**: The support for integrated practice arrangements and the requirement for supervision by a licensed physician might be seen as a call for maintaining high standards of patient care and ensuring accountability within the healthcare system. This approach could be interpreted as conservative in the sense that it advocates for a structured hierarchy and traditional roles within the healthcare profession. However, it also aligns with more progressive or liberal views that advocate for comprehensive and accessible healthcare, recognizing the value of all healthcare professionals in delivering care.<br><br>2. **Support for Payment for PA Services**: The stance on patient and third-party payment for PA services indicates an acknowledgment of the valuable role PAs play in healthcare delivery and the need for their services to be compensated accordingly. This could be seen as aligning with more liberal views that support expanded access to healthcare services and the recognition of a broader range of healthcare providers. It advocates for the economic sustainability of healthcare professions that are essential for expanding access to care.<br><br>3. **Regulatory and Educational Standards**: The insistence on accreditation, certification, and appropriate delegation of duties reflects a commitment to maintaining high professional standards and ensuring patient safety. This approach could be seen as conservative in its emphasis on regulation and standards. However, it also reflects a liberal emphasis on the importance of education and certification in improving healthcare quality and safety.<br><br>In summary, while the AAFP's position on PAs involves considerations of professional standards, healthcare quality, and economic aspects of healthcare delivery, it does not squarely fit into a single political ideology. It reflects a blend of principles that prioritize patient care quality, professional integrity, and the efficient operation of the healthcare system, elements that can be valued across the political spectrum.	neutral

AAFP	Physician Compensation for Nurse Practitioner and Physician Assistant Oversight	2019	The text primarily focuses on healthcare management and the organization of care teams, emphasizing the importance of multidisciplinary teams led by physicians, appropriate compensation for physician oversight of nurse practitioners (NPs) and physician assistants (PAs), and factors influencing compensation. This perspective does not neatly align with traditional political divisions like liberal or conservative viewpoints in a direct way, as it addresses professional practice standards, compensation models, and the operational aspects of healthcare delivery rather than ideological or policy positions typically associated with these political spectrums.<br><br>However, the emphasis on physician-led teams and structured compensation for supervision might be seen in different lights depending on one's political or ideological viewpoint:<br><br>- **More Conservative Viewpoint:** Individuals with a conservative viewpoint on healthcare might appreciate the emphasis on physician leadership and structured compensation models as a means to maintain high standards of care and ensure that healthcare delivery is led by highly trained professionals. The call for clear, contract-based compensation agreements can also resonate with conservative values related to business practices and professional autonomy.<br><br>- **More Liberal Viewpoint:** Those with a liberal viewpoint might focus on the collaborative aspect of healthcare delivery mentioned in the text, valuing the integrated, multidisciplinary teams and the recognition of NPs and PAs as vital components of the healthcare system. They might also support the idea of fair compensation models that acknowledge the diverse contributions of different healthcare professionals. However, they may also advocate for broader scope of practice laws for NPs and PAs as a way to increase access to care, a topic that is related but not directly addressed in the given text.<br><br>Ultimately, the text deals with professional standards and practices in healthcare rather than overt political ideologies. Its focus on team-based care, leadership, and compensation models speaks more to the operational and administrative concerns within the healthcare sector than to broader political debates. It’s also important to recognize that healthcare policy can intersect with political ideologies in complex ways, and different aspects of the text might align with different political viewpoints depending on how they are interpreted and what aspects are emphasized.	neutral
AAFP	Physician Dispensing of Drug Samples	2019	The statement from the American Academy of Family Physicians (AAFP) regarding the practice of physicians providing sample medications at no charge to patients reflects a health policy position that doesn't neatly align with traditional liberal or conservative viewpoints as understood in a strict political sense. Instead, it touches upon aspects of healthcare accessibility and cost-effectiveness, which are concerns across the political spectrum. Here's a breakdown of how both viewpoints might perceive this policy:<br><br>- **Liberal Viewpoint**: Liberals often advocate for increased accessibility to healthcare services and medications, emphasizing the importance of making healthcare affordable and accessible to all, regardless of income. From this perspective, the AAFP's support for providing sample medications at no charge could be seen as aligned with liberal values, as it directly contributes to improving patient access to necessary medications. The emphasis on cost-effectiveness also aligns with the desire to manage healthcare resources responsibly to ensure broad access.<br><br>- **Conservative Viewpoint**: Conservatives typically emphasize the importance of individual choice and responsibility in healthcare, as well as the efficiency and effectiveness of private sector solutions over government intervention. The AAFP's policy could be viewed through a conservative lens as promoting physician autonomy and discretion, which aligns with conservative values around individual choice and decision-making in healthcare. Furthermore, encouraging members to consider the cost-effectiveness of samples may resonate with conservative priorities for fiscal responsibility and efficient use of resources.<br><br>Overall, the AAFP's position is more focused on practical healthcare delivery and ensuring that physicians have the flexibility to provide care in a manner that considers both the patients' needs and the cost implications of treatment options. It represents a policy approach aimed at improving healthcare access and efficiency, rather than a statement with clear-cut alignment along the traditional liberal-conservative spectrum.	neutral

AAFP	Physician Expert Witness in Medical Liability Suits	2019	The text you've provided outlines principles and guidelines for the conduct and qualifications of physician expert witnesses in medical liability cases. It emphasizes the importance of objective, impartial testimony, the ethical responsibilities of physicians in the justice system, and standards for expert witness qualifications and compensation. These guidelines are presented as measures to ensure fair and equitable outcomes in medical liability suits.<br><br>This text primarily reflects a non-partisan, professional standpoint focused on legal and medical ethics rather than aligning directly with traditional political ideologies like liberalism or conservatism. It concentrates on improving the integrity and reliability of medical expert testimony in the judicial process, which is a concern that transcends political affiliations.<br><br>However, if we were to analyze the underlying principles for alignment with broader ideological themes, several points emerge:<br><br>1. **Emphasis on Professional Ethics and Standards**: The call for objective, impartial testimony and the establishment of clear standards for expert witnesses could be seen as aligning with a liberal perspective insofar as it emphasizes systemic fairness and accountability. Liberals often advocate for regulations and standards to ensure fairness and protect individuals' rights.<br><br>2. **Support for Legislation to Establish Qualifications**: The support for legislative action to establish qualifications for physician expert witnesses might resonate with both liberal and conservative viewpoints, but for different reasons. Liberals might see it as a means to ensure fairness and protect individuals from malpractice or negligence. Conservatives might support it as a way to uphold professional standards and prevent frivolous lawsuits, which they often argue can burden the healthcare system and professionals.<br><br>3. **Concern Over Compensation and Influence**: The guidelines caution against accepting disproportionately high fees for expert testimony, which could be interpreted as influencing the testimony. This concern aligns with a broad ethical stance that transcends political ideology but could be particularly resonant with liberal concerns over the influence of money in legal and medical practices.<br><br>4. **Requirement for Active Clinical Experience**: The emphasis on having current, active clinical experience in the relevant medical specialty might align more closely with conservative preferences for practical, hands-on experience and credentials in professional fields, reflecting a merit-based approach to qualifications.<br><br>Overall, the text is best understood as a professional and ethical guideline rather than a political statement. Its focus is on improving the legal process in medical liability cases through specific standards and practices for expert witnesses, which is an issue that both liberal and conservative legal and medical professionals could find merit in, albeit for different reasons.	neutral
AAFP	Physician Reentry	2020	The text you've provided outlines a policy approach to the issue of physician reentry into clinical practice after a period of inactivity. This text does not inherently align with a strictly liberal or conservative viewpoint as it addresses a practical issue within the healthcare system focused on maintaining a skilled workforce and ensuring high-quality patient care. The emphasis on transparency, integration into existing procedures, and the focus on patient care quality are principles that can be supported across a wide political spectrum.<br><br>Issues related to healthcare policy can often become politicized, but the specific concerns addressed in this text are more about professional standards, regulatory processes, and workforce management. These are areas where there is potential for bipartisan agreement on the importance of clear, fair, and efficient processes to support the reintegration of qualified professionals into the healthcare system.<br><br>In the broader context of healthcare policy, liberals and conservatives might differ on how to achieve the goals of high-quality patient care, the role of government in regulating healthcare, and how to best manage healthcare workforces. However, the specific focus on making the reentry process for physicians clear, integrated with existing certification procedures, and centered on patient care is a practical matter that doesn't neatly fit into the ideological divides of liberal or conservative viewpoints.	neutral

AAFP	Physician Remediation Education	6/2023	The text you provided outlines a policy position from the American Academy of Family Physicians (AAFP) regarding the approach to address gaps in educational and clinical skills among physicians. This policy emphasizes the importance of prescriptive, remedial educational programs based on individual assessments and the documentation of competence upon completion.<br><br>This position is more aligned with a pragmatic approach to medical education and professionalism rather than fitting neatly into a liberal or conservative political viewpoint. It focuses on ensuring that physicians maintain high standards of competence and are adequately prepared to provide quality healthcare, which is a concern that transcends political affiliations.<br><br>In general, policies aimed at improving educational outcomes and professional standards in healthcare are supported across the political spectrum, although the methods of implementation and funding may sometimes be points of debate between liberal and conservative viewpoints. However, this specific text emphasizes quality and accountability in medical education, which are goals that are not inherently political or aligned with a specific ideology.	neutral
AAFP	Physician's Right Relative to Imposed Administrative Costs	2020	The text presents a viewpoint that emphasizes the financial autonomy and transparency of physicians in their dealings with administrative requirements imposed by public or private health plans, regulatory authorities, employers, or other entities. It advocates for physicians' right to charge and receive payment for administrative tasks not directly related to patient care, such as formulary compliance changes or completion of specific forms like those for the Family Medical Leave Act (FMLA).<br><br>This position can be interpreted through both liberal and conservative lenses, depending on the aspects emphasized:<br><br>- **Conservative Viewpoint**: From a conservative perspective, the emphasis on the autonomy of physicians to charge for their services aligns with free market principles. Conservatives generally advocate for minimal government intervention in business practices, including healthcare. The call for transparency and the ability to charge for services rendered, even if administrative, could be seen as supporting the efficiency and sustainability of healthcare practices from a business standpoint.<br><br>- **Liberal Viewpoint**: From a liberal perspective, the emphasis might be placed on the aspect of transparency and potentially on ensuring that administrative costs do not become prohibitive for patients. Liberals often focus on the accessibility and affordability of healthcare for all. While this text does not directly address affordability, the call for transparency could be interpreted as a step toward ensuring that patients are aware of and possibly protected from excessive healthcare costs.<br><br>However, the text primarily leans towards a viewpoint that could be more closely associated with conservative principles of free market operations within the healthcare system, focusing on the rights of healthcare providers to charge for their services in a transparent manner. It doesn't explicitly address broader healthcare system reform or the impact of these charges on access to care, issues that are often more prominently featured in liberal discussions about healthcare policy.<br><br>It's important to note that such interpretations can vary widely depending on individual perspectives, and the political context can influence how these viewpoints are received. The emphasis on professional autonomy, market principles, and transparency in billing practices aligns with broader conservative values regarding the operation of healthcare systems, though aspects of this position could also find support among liberals, particularly in terms of advocating for transparency.	probably conservative
AAFP	Point of Care Testing Policy	4/2020	The text you've shared appears to focus on healthcare efficiency, patient care improvement, and cost reduction through the use of point-of-care testing performed in physician offices. It does not explicitly align with a traditional liberal or conservative viewpoint as it primarily addresses healthcare practice improvements rather than broader political or ideological positions.<br><br>Liberal and conservative viewpoints on healthcare often diverge on the role of government in healthcare, healthcare access, and funding. Liberals typically advocate for broader government involvement in ensuring access to healthcare for all, possibly through universal healthcare systems or regulations ensuring comprehensive coverage. Conservatives may emphasize market-driven solutions, limited government intervention, and individual choice in healthcare providers and insurance.<br><br>The statement from the American Academy of Family Physicians (AAFP) centers on the practical aspects of healthcare delivery—specifically, the benefits of point-of-care testing. It discusses improving patient care, making healthcare delivery more efficient, and potentially reducing costs. These goals can be supported across a wide political spectrum, as they focus on outcomes and efficiency rather than the means to achieve universal healthcare access or the extent of government involvement.<br><br>This focus on improving healthcare outcomes through specific medical practices is more about healthcare policy and less about the broader ideological divides. Both liberal and conservative individuals could potentially support the principles outlined in the	neutral

			statement if they believe that point-of-care testing aligns with their broader goals for healthcare reform, whether those goals involve expanding access, reducing costs, or improving quality of care.	
AAFP	Policing Standards	1/2022	The text presented aligns more closely with a liberal viewpoint. It emphasizes the need for systemic reforms in policing practices, focusing on reducing harm to Black, Indigenous, and people of color (BIPOC) and other marginalized populations. The document advocates for addressing health inequities, systemic racism, and the negative impacts of policing on community health and well-being. It calls for a shift in policies and procedures within law enforcement agencies to prioritize public health and safety, particularly for BIPOC communities. The text also supports community-based alternatives to traditional policing and incarceration, highlighting the importance of mental health services, substance use treatment, and social support programs as part of a comprehensive approach to public safety and health.<br><br>Liberal viewpoints often prioritize social justice, equity, and reforms aimed at addressing systemic inequalities and promoting the welfare of all community members, especially those historically marginalized. The focus on health inequities, systemic racism, and the advocacy for substantial reforms in policing practices, as well as the call for increased funding for community support and mental health services, align with liberal principles of social equity and justice.	liberal
AAFP	Political Action	10/2023	The text you've shared outlines a position by the American Academy of Family Physicians (AAFP) regarding political engagement and advocacy for family medicine. It emphasizes the importance of being politically engaged at all levels of government, developing advocacy tactics, and building relationships with legislators. It also mentions working with political parties and candidates to educate them on the importance of family medicine.<br><br>This statement does not explicitly align with either a liberal or conservative viewpoint. Instead, it focuses on advocacy for family medicine, encouraging political engagement among its members, and educating legislators and political candidates about the importance of family medicine in healthcare. The emphasis is on bipartisan engagement to support the interests of family medicine, rather than advocating for specific partisan policies.<br><br>Political engagement and advocacy in healthcare can be supported by individuals across the political spectrum, as improving healthcare and supporting physicians can be seen as non-partisan goals. The approach outlined by the AAFP aims to elevate the role and needs of family medicine within the broader healthcare system, which is a goal that transcends traditional political divides.	neutral

AAFP	Poverty and Health - The Family Medicine Perspective (Position Paper)	1/2022	The text primarily aligns with viewpoints often associated with liberal or progressive ideologies, particularly in its emphasis on systemic factors contributing to poverty and health inequities, the importance of addressing social determinants of health (SDoH), and advocating for comprehensive, community-oriented primary care and public health integration. Key aspects that reflect a liberal viewpoint include:<br><br>1. **Systemic Understanding of Poverty and Health:** The text emphasizes that poverty and health outcomes are deeply influenced by systemic factors, a perspective often associated with liberal ideologies which focus on the role of social, economic, and environmental structures in shaping individual and community health.<br><br>2. **Focus on Social Determinants of Health (SDoH):** It underlines the importance of addressing the social determinants of health as a means to improve health outcomes. This approach is aligned with progressive views on health care, which advocate for a holistic understanding of health that includes social, economic, and environmental factors.<br><br>3. **Advocacy for Community and Public Health Integration:** The call for integrating primary care and public health efforts reflects a progressive approach to healthcare, emphasizing the need for systemic changes to achieve health equity and optimal health for everyone.<br><br>4. **Emphasis on Equity and Inclusion:** The text calls for culturally proficient care teams and addresses health disparities, advocating for equity and inclusion in healthcare. This aligns with liberal values of equality, diversity, and inclusivity.<br><br>5. **Support for Government and Policy Interventions:** The advocacy for regulatory frameworks, economic incentives, and policies such as Health in All Policies (HiAP) approach, aligns with progressive ideologies that support the role of government and policy in addressing public health issues and promoting health equity.<br><br>6. **Community Engagement and Leadership:** The emphasis on community leadership and the role of family physicians in advocating for and implementing community-responsive care and interventions reflects a progressive viewpoint on the importance of community engagement and empowerment in health care.<br><br>Overall, the text's focus on systemic factors, social determinants of health, community-oriented care, health equity, and the role of policy and government interventions in improving health outcomes is more aligned with liberal or progressive ideologies than conservative ones, which might emphasize individual responsibility, limited government intervention in health care, and market-driven solutions to health care and poverty.	liberal
AAFP	Pre- and Post-Operative Care	10/2023	The text you've provided discusses the role of family physicians in pre- and post-operative care, emphasizing the importance of a holistic approach to patient health that includes medical and social history, care coordination, and partnership between the family physician and the primary surgeon. It highlights the benefits of such an approach, including reduced risks associated with surgery, fewer postoperative complications, enhanced healthcare access, improved quality, and controlled costs.<br><br>This text aligns more closely with viewpoints that are typically considered neither strictly liberal nor conservative in the traditional political sense. Instead, it focuses on healthcare delivery and policy, advocating for a collaborative, integrated approach to patient care. Such perspectives are commonly supported across a broad spectrum of healthcare professionals and policymakers who are interested in improving patient outcomes, enhancing the efficiency of healthcare delivery, and managing healthcare costs.<br><br>In the context of healthcare policy discussions, both liberal and conservative viewpoints may find elements of this approach agreeable. Liberals might support the emphasis on comprehensive care, access to healthcare, and the potential to improve quality and control costs, aligning with a broader goal of healthcare for all. Conservatives might appreciate the focus on efficiency, cost control, and the role of professionals in managing care, which can align with principles of market-driven solutions and individualized care plans.<br><br>Therefore, the text reflects a professional and pragmatic approach to healthcare that transcends simple political categorization, focusing instead on practical outcomes and the well-being of patients. It is rooted in the principles of family medicine and integrated care, which are widely accepted practices in the medical community aimed at improving healthcare delivery and patient outcomes.	neutral

AAFP	Pre-Medical Student Shadowing	1/2022	The text describes the American Academy of Family Physicians' (AAFP) position on workforce expansion, shadowing opportunities, and educational resources related to family medicine. It advocates for increased access to primary care through a patient-centered medical home model, student shadowing opportunities at various educational levels, resources for family physicians on career information and HIPAA compliance, and the importance of patient confidentiality, including the right of patients to decline student shadows during medical encounters.<br><br>This statement aligns more closely with viewpoints typically associated with liberal or progressive ideologies, which often emphasize the expansion of healthcare access, educational opportunities in the medical field, and the protection of patient rights and privacy. These positions focus on inclusivity in medical education, workforce expansion to meet public health needs, and respect for patient autonomy—all principles that are generally supported by liberal perspectives on healthcare reform and education.<br><br>However, the statement also reflects pragmatic approaches to healthcare education and workforce development, without explicitly endorsing or opposing broader political or economic policies. Thus, while it leans towards principles often supported by liberal ideologies, it primarily focuses on practical measures to improve healthcare access and education, which can be supported across the political spectrum for their potential to enhance the quality and availability of healthcare services.	probably liberal
AAFP	Pre-payment and Post-payment Audits	7/1905	The text you've provided expresses a viewpoint on the administration and regulation of healthcare, specifically concerning the use of pre-payment and post-payment audits in physician offices. It criticizes the indiscriminate use of these audits for being disruptive and fostering a culture of mistrust, advocating for a more selective approach that is supported by concrete evidence of abuse. Furthermore, it suggests that focused medical reviews by peers in the same specialty are preferable to broad audits.<br><br>This viewpoint doesn't align strictly with traditional liberal or conservative ideologies in a straightforward manner. Instead, it reflects concerns over regulatory practices in healthcare, emphasizing efficiency, fairness, and professional autonomy.<br><br>However, if we were to analyze the underlying values and concerns, we might draw some connections:<br><br>- **Liberal Viewpoint Alignment:** There might be a slight alignment with liberal viewpoints in the emphasis on ensuring that regulatory mechanisms do not become overly burdensome or intrusive, potentially mirroring broader liberal concerns about the need for regulations to be fair, evidence-based, and not overly punitive. Liberals often advocate for policies that protect the rights and autonomy of individuals and groups, including in professional contexts.<br><br>- **Conservative Viewpoint Alignment:** On the other hand, the call for less onerous mechanisms and the criticism of what might be seen as government overreach or inefficiency could also resonate with conservative principles, which emphasize limited government intervention, fiscal responsibility, and the importance of individual and business autonomy.<br><br>In essence, the text's viewpoint might find some support across the political spectrum, depending on how its principles are interpreted in the context of broader ideological beliefs about government regulation and professional autonomy. It's more focused on practical and professional concerns within the healthcare system than on advancing a specific political ideology.	neutral
AAFP	Preceptorships	10/2023	The text you've shared seems to focus on educational policy and the promotion of family medicine within the medical community, as advocated by the American Academy of Family Physicians (AAFP). It outlines the AAFP's position on the importance of role modeling, mentorship, and clinical training for medical and premedical students, aiming to enhance the choice of family medicine as a career path. This stance emphasizes the value of high-quality, continuous, compassionate, and coordinated care in medical education and practice.<br><br>This perspective does not directly align with traditional political viewpoints such as liberal or conservative as it primarily addresses educational and professional standards within the medical field rather than political ideologies. The focus is on improving medical education and promoting family medicine, which are objectives that can be supported across the political spectrum.<br><br>In the broader context, issues related to healthcare education and practice can sometimes be politicized, particularly when discussions involve funding, access to care, or healthcare reform. However, the specific emphasis in this text on enhancing medical education and supporting family medicine preceptorships is more about professional development and healthcare quality than about partisan politics.	neutral

AAFP	Preconception Care (Position Paper)	9/2022	The text you've shared generally aligns more closely with viewpoints often associated with liberal perspectives, especially in the context of healthcare and social issues in the United States. This alignment can be seen in several key aspects of the text:<br><br>1. **Inclusivity and Gender Spectrum Consideration**: The explicit recognition of patients across the gender spectrum and the effort to remain gender-neutral in discussions about pregnancy and preconception care reflect a progressive stance on gender identity issues, which is more commonly associated with liberal viewpoints.<br><br>2. **Focus on Health Equity and Addressing Disparities**: The text emphasizes the need to reduce health disparities and achieve health equity, especially among racial and ethnic minorities. It mentions the impact of social determinants of health and institutional racism on health outcomes, which are concerns that are often highlighted by more liberal perspectives.<br><br>3. **Support for Comprehensive Reproductive Care**: The advocacy for comprehensive contraceptive services, including emergency contraception and long-acting reversible contraception (LARC), and the emphasis on the importance of access to these services for preventing unintended pregnancies align with more liberal policies on reproductive rights and health.<br><br>4. **Public Health Initiatives and Preventive Care**: The support for national health initiatives like Healthy People 2030, which focuses on reducing unintended pregnancies and improving preconception health through preventive care, aligns with a more liberal emphasis on public health interventions and preventive healthcare.<br><br>5. **Evidence-Based Health Policy**: The call for evidence-based recommendations and interventions to improve pregnancy-related and fetal outcomes, along with the critique of barriers to accessing care (such as fragmented healthcare systems and inadequate insurance coverage), reflects a liberal viewpoint on healthcare policy and reform.<br><br>6. **Consideration for Mental and Behavioral Health**: The comprehensive approach to addressing mental and behavioral health as part of preconception care, including screening for substance use and providing appropriate counseling and treatment, aligns with liberal perspectives on integrating mental health with overall healthcare.<br><br>It's important to note that while these aspects of the text may align more closely with liberal viewpoints, the focus on providing high-quality, evidence-based healthcare is a goal shared across the political spectrum. The emphasis on inclusivity, health equity, and comprehensive care, however, tends to be more pronounced in liberal policy discussions.	liberal
AAFP	Preferred Unit of Measurement for Liquid Medications	1/2022	The statement about the American Academy of Family Physicians (AAFP) supporting a standardized approach for the use of milliliters (mL) as the preferred unit of measurement for liquid medications to prevent unintended medication overdoses in children does not directly align with traditional liberal or conservative viewpoints as they are commonly understood in political terms. This statement reflects a position on public health and safety, specifically regarding medication dosing for children, rather than a political or ideological stance.<br><br>Public health and safety initiatives often transcend political boundaries, aiming for outcomes that benefit the general population, regardless of political affiliation. The emphasis on using milliliters (mL) for liquid medications is a practical measure intended to reduce confusion and the risk of dosing errors, which can lead to serious health consequences for children. Such measures are typically based on scientific research and evidence-based practices rather than political ideology.<br><br>Therefore, this text is best understood as aligning with a viewpoint focused on medical safety and public health, rather than fitting neatly into a liberal or conservative political framework.	neutral

AAFP	Pregnancy, Perinatal, and Newborn Care by Family Physicians	10/2023	The text provided focuses on the medical and healthcare aspects of pregnancy, perinatal, and newborn care within the field of Family Medicine. It emphasizes the importance of comprehensive care, the need for basic and advanced training for family medicine residents in these areas, and the advocacy for access to care regardless of geographical location or socioeconomic status. This text outlines policy positions and advocacy efforts aimed at improving healthcare delivery and reducing disparities in maternal and newborn health outcomes.<br><br>This content does not explicitly align with traditional liberal or conservative political viewpoints as it primarily addresses healthcare practice, training, and policy within the context of Family Medicine. The focus is on medical education, healthcare access, quality of care, and overcoming disparities in health outcomes, which are concerns that can be supported across the political spectrum. However, the emphasis on access to care for underserved populations, addressing healthcare disparities, and advocating for comprehensive reproductive healthcare services, including contraception and management of pregnancy complications, could be seen as aligning more closely with viewpoints traditionally associated with liberal ideologies. These ideologies often prioritize healthcare equity, access to comprehensive reproductive health services, and the reduction of disparities in healthcare outcomes.<br><br>It is important to note that healthcare and medical practice policies, especially those related to reproductive health, can intersect with broader political and social debates. However, the specific focus here on medical training, credentialing, and the provision of care based on competence rather than specialty designation is primarily a matter of healthcare policy and professional standards rather than a direct reflection of political ideology.	neutral
AAFP	Prevention and Management of Sexually Transmitted Infections	10/2023	The text provided outlines the American Academy of Family Physicians (AAFP)'s position on the prevention and management of sexually transmitted infections (STIs) and promotes a comprehensive, evidence-based, and inclusive approach to sexual health. This perspective tends to align more closely with liberal viewpoints for several reasons:<br><br>1. **Emphasis on Education and Awareness**: The focus on public and professional education about STIs, including prevention, diagnosis, treatment, and control, aligns with liberal perspectives that prioritize science-based education and public health initiatives.<br><br>2. **Inclusive Approach to Sexual Health**: Recognizing the diversity of human gender identity, relationships, and sexual practices reflects a progressive stance on LGBTQ+ issues. This inclusivity and the nonjudgmental obtaining of sexual histories are more commonly associated with liberal values, which tend to emphasize equality and nondiscrimination.<br><br>3. **Patient-Centered Care**: The emphasis on patient-centered physical examinations, individualized screening, and treatment recommendations, and safer sex counseling aligns with liberal viewpoints that prioritize individual rights and personalized healthcare.<br><br>4. **Opposition to Discrimination**: The AAFP's opposition to discrimination against patients receiving STI-specific therapies, such as PrEP and PEP, in obtaining health-related services and insurance, reflects a commitment to healthcare equity, a principle often associated with liberal ideologies.<br><br>5. **Prevention Strategies**: The endorsement of a variety of prevention strategies, including barrier methods, vaccinations, PrEP, and PEP, along with the promotion of open discussions about sexual health, abstinence, and monogamy, demonstrates a comprehensive approach to STI prevention that is more in line with liberal perspectives on public health and personal autonomy.<br><br>6. **Expedited Partner Therapy (EPT) and Patient-Delivered Partner Therapy (PDPT)**: The support for EPT and PDPT, which involve treating partners of infected individuals without direct medical evaluation, reflects a pragmatic and progressive approach to reducing STI transmission, aligning with liberal public health strategies.<br><br>7. **Screening Recommendations Based on Sex Designation at Birth and Current Anatomy**: The nuanced approach to screening recommendations, which considers sex designation at birth, current anatomy, and sexual practices rather than solely gender identity, seeks to provide equitable and effective healthcare. This evidence-based, inclusive approach is more characteristic of liberal perspectives, which advocate for healthcare policies that accommodate diversity and promote health equity.<br><br>Overall, the text reflects a liberal viewpoint through its comprehensive, inclusive, and evidence-based approach to sexual health and STI prevention, emphasizing education, patient-centered care, and opposition to discrimination, while promoting a broad range of prevention strategies and treatments.	liberal

AAFP	Prevention of Gun Violence (Policy Statement)	7/1905	The text provided reflects a viewpoint more commonly associated with liberal or progressive ideologies, particularly in the context of American politics. This assessment is based on several key points outlined in the text:<br><br>1. **Support for Primary Prevention Strategies**: The emphasis on primary prevention strategies to reduce injuries and deaths associated with gun ownership and violence aligns with a public health approach to gun control. This perspective prioritizes safety and well-being over unrestricted gun rights, which is a stance more often associated with liberal viewpoints.<br><br>2. **Advocacy for Federal and State Policies for Balance**: The call for policies that balance the right to own firearms with health, safety, and societal well-being suggests a preference for regulatory measures over a more libertarian or conservative approach that might prioritize individual gun ownership rights with fewer restrictions.<br><br>3. **Support for Gun Violence Research Funding**: Advocating for appropriate funding for gun violence research and public health surveillance is a stance that has faced opposition from conservative lawmakers, particularly due to restrictions like the Dickey Amendment in the past. Liberal and progressive groups have been more supportive of such research as a basis for informed policy-making.<br><br>4. **Physician Counseling on Injury Prevention**: The emphasis on the role of physicians in counseling patients about safe storage practices and injury prevention, including opposition to state “gag rule” bills, aligns with a public health approach to gun safety. This approach has faced opposition from some conservative groups who view it as an intrusion into individual rights.<br><br>5. **Expansion of Background Checks**: The call for expanding the National Instant Criminal Background Check System (NICS) to cover sales at gun shows, over the Internet, and in classified ads goes beyond current federal law. This expansion is a policy proposal more commonly supported by liberals and opposed by conservatives, who often argue that such measures infringe on Second Amendment rights.<br><br>6. **Support for Stronger Gun Trafficking Laws and Reinstating the Ban on Assault Weapons**: Advocacy for stronger laws against gun trafficking, straw purchases, and the reinstatement of the ban on the sale of assault weapons and high-capacity magazines is a position more commonly associated with the Democratic Party and liberal policy advocates.<br><br>In summary, the positions outlined in the text align more closely with a liberal or progressive viewpoint on gun control, emphasizing public health, safety, and regulation over a more unregulated approach to gun ownership.	liberal
AAFP	Prevention of Sexual and Gender-Based Harassment in Medical Education and Clinical Settings	7/1905	The text you've provided aligns more closely with viewpoints typically associated with liberal ideologies, particularly in its emphasis on acknowledging and addressing issues of sexual and gender-based harassment, advocating for inclusive policies, and protecting vulnerable groups. Liberal perspectives often prioritize social justice, equality, and the protection of individual rights, especially for marginalized or disadvantaged communities. The focus on developing policies that protect individuals from sexual and gender-based harassment, especially those who identify or are perceived as women, aligns with these liberal values by seeking to address and mitigate systemic issues within the medical field that impact the safety, well-being, and performance of those involved.<br><br>Conservative viewpoints, while also potentially supportive of efforts to combat harassment, may emphasize different approaches or priorities, such as focusing on individual responsibility or the autonomy of institutions to manage their affairs without external mandates. However, it's important to note that the desire to address sexual and gender-based harassment can cross ideological lines, and there might be conservatives who support the measures outlined in the text, albeit possibly with different emphases or proposed mechanisms.<br><br>The stance taken by the American Academy of Family Physicians (AAFP) reflects a broader commitment to creating safe, inclusive, and equitable environments within the medical profession, which is consistent with principles often advocated by those with liberal viewpoints.	liberal

AAFP	Preventive Medicine	9/2022	The text you've provided outlines a stance on health promotion and disease prevention as fundamental aspects of primary care and family medicine, specifically mentioning the American Academy of Family Physicians' (AAFP) support for evidence-based, cost-effective preventive medicine. This perspective emphasizes the importance of preventive measures in healthcare, advocating for policies and payment structures that support such services.<br><br>This viewpoint doesn't neatly align with traditional political labels like "liberal" or "conservative" because it focuses on medical best practices and healthcare policy rather than broader political ideologies. However, it can intersect with political viewpoints in the following ways:<br><br>1. **Liberal Viewpoint Alignment**: The emphasis on preventive care and the advocacy for policies and payment reforms to support such care can align with liberal viewpoints, especially those advocating for healthcare reform, increased access to healthcare services, and the belief in a strong role for government in regulating and supporting healthcare systems. Liberals often support the idea of preventive care as a way to improve overall health outcomes and reduce healthcare costs in the long term.<br><br>2. **Conservative Viewpoint Considerations**: Some conservative viewpoints might also support aspects of preventive care, especially the focus on cost-effectiveness and evidence-based practices. Conservatives typically advocate for efficient spending and might see preventive medicine as a way to reduce healthcare costs by preventing more expensive treatments later on. However, they might have different ideas about the role of government in healthcare, often favoring less regulation and more market-based solutions.<br><br>The text primarily reflects a healthcare policy perspective rather than a purely political one, aiming for the advancement of health outcomes through preventive care. It suggests a pragmatic approach to healthcare, which can be supported by individuals across the political spectrum, depending on how these goals are proposed to be achieved.	neutral
AAFP	Primary Care	1/2022	The text provided outlines definitions and principles related to primary care in the healthcare system, emphasizing integrated, accessible, person-centered, and equitable healthcare services. It does not align strictly with either a liberal or conservative viewpoint in a political sense, as the concepts of improving healthcare quality, accessibility, and efficiency can be supported across the political spectrum. However, certain aspects of the text may resonate more with specific ideological perspectives:<br><br>1. **Liberal Viewpoint Alignment**: The emphasis on equitable and accessible health care, patient advocacy, and the coordination of healthcare services to achieve cost-effective care align with liberal values of universal access to healthcare, reducing inequalities, and prioritizing patient-centered care. Liberals often advocate for healthcare reforms that increase access and equity, and the principles outlined in the text align with these goals.<br><br>2. **Conservative Viewpoint Considerations**: Conservatives typically emphasize the role of private healthcare providers, market-driven solutions to healthcare, and personal responsibility in health. While the text focuses on the structure and principles of primary care rather than the means of healthcare delivery, aspects such as the emphasis on efficiency and cost-effectiveness in healthcare could align with conservative interests in reducing wasteful spending and improving healthcare delivery efficiency.<br><br>The text primarily focuses on the operational, clinical, and ethical considerations of primary care rather than explicitly political or ideological issues. Thus, while certain principles may align more closely with the general values of one political ideology over another, the document itself is structured around healthcare professional consensus and standards for primary care. It advocates for a healthcare system that is responsive to patient needs, supports continuous and comprehensive care, and seeks to achieve better health outcomes at lower costs, which are goals that can be supported by individuals across the political spectrum.	neutral

AAFP	Primary Care Physician, Generic	7/1905	The statement from the American Academy of Family Physicians (AAFP) regarding the role of the family physician as the ideal primary care physician and their opposition to the creation of a "generic" primary care physician designation does not directly align with traditional political viewpoints such as liberal or conservative. This stance is more related to the organization's perspective on healthcare practice and policy, focusing on the importance of the family physician's role in providing comprehensive and continuous care to patients.<br><br>In the context of healthcare policy, "liberal" viewpoints generally support increased government intervention in healthcare to ensure access, affordability, and quality of care, while "conservative" viewpoints tend to favor less government involvement, emphasizing market-driven solutions and individual choice. The AAFP's statement is primarily a professional and policy stance regarding the qualifications, training, and role of primary care physicians within the healthcare system, rather than a position on the broader political spectrum of healthcare access or funding.<br><br>This statement could, however, indirectly intersect with broader political debates about healthcare system organization, primary care access, and quality of care. For instance, discussions on how to best organize primary care might touch upon issues of healthcare access and delivery models, which are indeed politicized. Yet, the statement itself focuses on professional standards and roles within the medical community rather than explicitly endorsing a political ideology regarding healthcare policy.	neutral
AAFP	Principles for Administrative Simplification (Position Paper)	10/2023	The text you've provided advocates for reducing administrative burdens on primary care physicians and improving the efficiency and effectiveness of healthcare delivery. It calls for simplification in areas such as prior authorization, step therapy, quality and performance measurement, medical supply coverage requirements, and medical record documentation. The American Academy of Family Physicians (AAFP) outlines specific principles aimed at reducing these burdens and ensuring timely patient access to treatment.<br><br>This perspective aligns more closely with viewpoints traditionally associated with liberal ideologies, which often emphasize the role of structured regulation and reform to improve healthcare accessibility, efficiency, and quality. The focus on administrative simplification, reducing costs, and improving both patient and provider experiences reflects a desire to make the healthcare system more patient-centered and equitable. Liberal viewpoints typically support the idea that government and policy reforms can play a significant role in addressing systemic issues in healthcare, including reducing bureaucracy and improving the interaction between public and private healthcare entities.<br><br>Conservative viewpoints, on the other hand, tend to emphasize market-driven solutions, reduced government intervention, and personal responsibility in healthcare. While there may be conservative support for reducing administrative burdens to improve efficiency and reduce costs, the emphasis might be more on deregulation and enhancing competition among private insurers and providers to achieve these goals.<br><br>However, it's important to note that the issues of administrative burden and healthcare efficiency can cross ideological lines. Both liberal and conservative healthcare professionals and policymakers may find common ground in seeking to reduce unnecessary administrative tasks, as these issues directly impact patient care and the sustainability of healthcare practices. The text, by focusing on pragmatic solutions to universally acknowledged problems, could appeal to a broad spectrum of political beliefs, even though its emphasis on standardization and cooperation among payers aligns more closely with liberal principles of healthcare reform.	liberal

AAFP	Principles to Optimize Family Physician Employment (Position Paper)	7/2023	The text you provided discusses several principles and practices aimed at enhancing the primary care and family medicine workforce, with a focus on equitable compensation, leadership, autonomy, and support for care delivery. These topics generally align with viewpoints that emphasize systemic improvements, equity, and the prioritization of patient care over productivity metrics. These concerns can be found across the political spectrum but are often more closely associated with liberal viewpoints due to the emphasis on equitable access to healthcare, the importance of primary care in reducing healthcare costs and improving outcomes, and the support for value-based care models over traditional fee-for-service models.<br><br>Liberal viewpoints typically prioritize healthcare reform that aims to increase access to care, reduce healthcare disparities, and promote models of care that are patient-centered and value-based. The emphasis on equity in employment for family physicians, transparency in compensation regardless of gender, gender identity, sexual orientation, race, and ethnicity, and the focus on leadership roles for family physicians in governance and management structures align with these liberal priorities. Additionally, the critique of employment practices that prioritize financial over clinical considerations, and the call for autonomy in clinical decision-making, reflect concerns with the current healthcare system that are often highlighted by those with a more progressive stance on healthcare reform.<br><br>Conservative viewpoints, while also concerned with the efficiency and effectiveness of healthcare delivery, may place a greater emphasis on market-driven solutions, the reduction of government involvement in healthcare, and may have a different approach to addressing healthcare workforce issues. However, it's important to note that there is a wide range of perspectives within both liberal and conservative circles on how to best achieve a high-performing healthcare system, and there can be overlap in support for certain policies, such as the importance of primary care.<br><br>In summary, while the principles outlined in the text align with general liberal values related to healthcare equity, access, and reform, it's important to recognize that there is diversity of opinion within political ideologies on how to address these issues. The focus on primary care as a means to improve healthcare outcomes and equity is a concern that transcends traditional political boundaries.	probably liberal
AAFP	Prior Authorization and Step Therapy (Position Paper)	10/2023	The text presents a viewpoint that leans more towards a liberal perspective, particularly in its emphasis on reducing barriers to patient care, advocating for evidence-based and patient-centered healthcare policies, and promoting the reduction of administrative burdens on physicians. Liberal viewpoints often prioritize access to healthcare, transparency, and efficiency in healthcare delivery, focusing on the well-being of patients and ensuring they receive timely and necessary treatment without undue interference from insurance companies or overly burdensome regulatory processes.<br><br>The text's criticism of prior authorization (PA) and step therapy protocols, which are seen as obstacles to timely and effective patient care, aligns with liberal perspectives on healthcare reform that advocate for reducing insurance company control over patient treatment plans. The call for exemptions for physicians with high approval rates and those in financial risk-sharing agreements suggests a desire to streamline processes for proven providers, emphasizing trust in the medical professionals' judgment over bureaucratic procedures.<br><br>Moreover, the demand for these processes to be evidence-based, transparent, and efficient, as well as the call for public availability of clinical criteria used for coverage decisions, reflects a liberal inclination towards accountability, patient rights, and the importance of science and evidence in policy-making.<br><br>Conservative viewpoints, on the other hand, might focus more on cost containment, the role of competition and market forces in improving healthcare quality and efficiency, and may be more supportive of PA and step therapy as tools to manage healthcare costs. However, it's important to note that there can be overlap in the desire for efficient, transparent healthcare processes across the political spectrum, but the emphasis on patient access and reducing administrative burdens is more typically associated with liberal policy priorities.	liberal

AAFP	Privilege Support Protocol	10/2023	The text from the American Academy of Family Physicians (AAFP) focuses on professional standards and practices within the medical field, specifically regarding the criteria and support for obtaining hospital privileges by physicians. This position emphasizes the importance of qualifications, training, experience, and competence over other factors, such as the specialty of the physician. It also mentions opposition to discrimination based on specialty, aligning with principles of fairness and meritocracy.<br><br>This stance is more aligned with principles that both liberal and conservative viewpoints might support, as it emphasizes professional competence, individual qualifications, and anti-discrimination. However, the specific emphasis on meritocracy and individual achievement might resonate more with conservative principles that focus on individual responsibility and qualifications. On the other hand, the opposition to discrimination and the focus on equitable treatment across specialties can also align with liberal values that prioritize equality and fairness.<br><br>Thus, the text itself does not neatly align with a strictly liberal or conservative viewpoint but rather reflects professional and ethical standards that could be endorsed by individuals across the political spectrum. It focuses on the medical profession's internal standards rather than broader political or ideological debates.	neutral
AAFP	Privileges, Colonoscopy	10/2023	The text provided does not neatly align with a liberal or conservative viewpoint in the traditional political sense. It focuses on the practical aspects of healthcare provision, particularly regarding colon cancer screening and the qualifications for physicians to perform colonoscopies. This text emphasizes the importance of having adequate numbers of qualified physicians to meet public health challenges and outlines a process for granting colonoscopy privileges based on training, experience, and demonstrated competence.<br><br>Issues such as healthcare access, quality of care, and the specifics of medical credentialing and privileging processes typically transcend simple liberal-conservative divides. Liberals and conservatives might have different opinions on how healthcare should be funded, managed, or regulated, but the specifics given here are more technical and administrative rather than ideological.<br><br>For example:<br><br>- **Liberals** might focus on ensuring that screening guidelines and privileges are set in a way that maximizes access for all communities, particularly underserved ones, and could emphasize the role of public institutions in overseeing these processes.<br>- **Conservatives** might emphasize the role of private healthcare facilities and professional autonomy in determining privileging criteria, potentially advocating for less government intervention in such decisions.<br><br>However, this text centers on operational and quality control measures within healthcare facilities, which are generally agreed upon principles across the healthcare industry, aimed at ensuring that patients receive safe and effective care. These are areas where there is often bipartisan agreement on the goals, if not always on the methods to achieve them.	neutral
AAFP	Privileges, Electrocardiogram Interpretation	10/2023	The text you've provided outlines a professional and procedural stance taken by the American Academy of Family Physicians (AAFP) regarding the qualifications and privileges of physicians, particularly in the context of interpreting electrocardiograms. This stance is based on the principles of fairness, equality, and competence in medical practice. It does not inherently align with traditional political labels such as "liberal" or "conservative" because it focuses on medical professionalism, education, and credentialing rather than political or social ideology.<br><br>The AAFP's statement emphasizes the importance of basing privileges on individual training, experience, and demonstrated competence, and it advocates for equal treatment and standards for all physicians, regardless of their specialty. This approach is rooted in the values of meritocracy and equal opportunity within the medical profession. While discussions about healthcare policies can sometimes reflect broader liberal or conservative viewpoints, particularly when it comes to issues of access, regulation, and the role of government, the specific position stated here is more closely related to professional standards and practices within the healthcare industry rather than to partisan political ideologies.	neutral

AAFP	Privileges, Emergency Care Services	10/2023	The text you've shared reflects a policy stance from the American Academy of Family Physicians (AAFP) rather than a strictly political viewpoint. It emphasizes the importance of qualifications, such as training, experience, and current competence, for family physicians to practice in emergency departments, suggesting that these factors should be more significant than the physician's specialty alone.<br><br>This stance is more about professional standards and healthcare quality than about aligning with traditional liberal or conservative viewpoints. In the context of healthcare policy, liberals typically advocate for broad access to healthcare services and may support measures that increase the pool of qualified emergency care providers. Conservatives may focus on the autonomy of medical institutions to set their own standards and the importance of maintaining high-quality care, potentially agreeing with the emphasis on competence and qualifications.<br><br>The statement aligns with a viewpoint that prioritizes professional competence and patient care quality over traditional specialty boundaries, which could be supported by both liberal and conservative perspectives for different reasons. It's a professional and pragmatic approach to healthcare delivery rather than a politically ideological one.	neutral
AAFP	Privileges, Special/Critical Care Unit	10/2023	The statement you've provided from the American Academy of Family Physicians (AAFP) focuses on the criteria for granting privileges to physicians in special or critical care units, emphasizing training, experience, and demonstrated current competence. This position does not inherently align strictly with either a liberal or conservative viewpoint as it addresses professional standards within the medical field rather than political or ideological issues.<br><br>Liberal and conservative viewpoints typically differ on social, economic, and political issues such as government intervention in healthcare, taxation, and social welfare programs. The AAFP's stance is more about ensuring quality healthcare and professional standards in hospitals, which is a goal that can be supported across the political spectrum. It speaks to the importance of competency and qualifications over other non-merit-based criteria, which is a principle that can find agreement in various political ideologies for different reasons.<br><br>In summary, the statement is centered on healthcare quality and professionalism rather than political ideology, making it not explicitly aligned with either liberal or conservative viewpoints.	neutral
AAFP	Privileges, Surgical Assistant	10/2023	The text you've provided appears to be a policy or position statement regarding the role and training of family medicine residents in surgical settings, the qualifications of family physicians to assist in surgeries, and guidelines for their involvement and compensation. This statement does not neatly align with traditional political labels like "liberal" or "conservative" because it primarily addresses medical education, professional standards, and healthcare practices rather than overt political issues or ideologies.<br><br>However, certain aspects of the statement could be interpreted in light of broader healthcare policy debates that do have political dimensions. For example:<br><br>1. **Advocacy for Comprehensive Care and Physician Involvement in Surgery**: The emphasis on comprehensive and continuous care, psychological support, and the coordination of care reflects a patient-centered approach to healthcare. Both liberals and conservatives might support this principle, but the emphasis on such holistic care might more closely align with liberal healthcare priorities, which often stress the importance of accessibility, continuity, and breadth of healthcare services.<br><br>2. **Fair Compensation for Physician Services**: The call for fair compensation for physician assistance in surgery touches on healthcare financing and reimbursement policies. While both liberal and conservative viewpoints might agree on the principle of fair compensation, the mechanisms for achieving this—such as through government programs, insurance reform, or market-driven solutions—can be politically contentious and align differently along the political spectrum.<br><br>3. **Regulations and Hospital Rules**: The statement's stance on hospital rules and the ability of family physicians to assist in surgeries despite surgical residency program rules might invoke broader debates on regulatory oversight versus professional autonomy in healthcare. Conservatives might emphasize professional autonomy and reduced regulatory interference, while liberals might focus on standards and oversight to ensure quality and equity in healthcare services.<br><br>4. **Non-Physician Surgical Assistance**: The text's cautious acceptance of non-physician surgical assistance in specific cases where physician assistance is unavailable could be seen through the lens of healthcare workforce debates. Liberals might view this as a pragmatic approach to addressing healthcare provider shortages and expanding access to care, while conservatives might focus on the importance of maintaining professional standards and the quality of care.	neutral

			Overall, while the statement itself is focused on healthcare practice standards rather than political ideology, different elements of it could resonate with or be supported by both liberal and conservative perspectives depending on how those elements connect to broader debates on healthcare policy, financing, and regulation.	
AAFP	Privileging, Family Medicine	10/2023	The text provided outlines the American Academy of Family Physicians (AAFP)'s stance on the training, education, privileging, and practice scope of family physicians. It emphasizes the comprehensive skill set of family physicians across various care settings, their eligibility for performing a wide range of medical procedures, and the criteria for granting clinical privileges based on competence and training rather than specialty or affiliations. It also addresses the importance of evidence-based criteria, continuity of care, and access to educational opportunities for all physicians. Furthermore, it opposes the idea of non-physician clinicians making independent privileging decisions for family physicians and advocates for fair and equal treatment of physicians across specialties in the privileging process.<br><br>This text does not neatly align with traditional political divides such as liberal or conservative viewpoints. Instead, it focuses on professional standards, training, and competency in the medical field, particularly within family medicine. The emphasis on evidence-based criteria, equal standards, and access to educational opportunities for all physicians reflect principles of professional excellence and equity in healthcare provision. These principles can be supported across the political spectrum as they pertain to improving healthcare quality, ensuring patient safety, and promoting the professional development of physicians. The opposition to non-physician clinicians making privileging decisions could be seen as a stance on maintaining professional standards and qualifications, which is a matter of professional governance rather than a political ideology.<br><br>Overall, the text is more aligned with professional and healthcare policy discussions rather than being distinctly liberal or conservative. It advocates for policies that ensure high-quality patient care, professional competency, and fair privileging processes within the healthcare system.	neutral

AAFP	Procedural Skills Training, Residency Criteria	9/2022	The text provided outlines a comprehensive framework for procedural training in a family medicine residency program. It focuses on the educational and operational aspects necessary to ensure that residents are well-prepared to perform medical procedures safely and effectively. This includes background knowledge, patient care, technical skills, documentation, and continuous evaluation and improvement of skills.<br><br>This content is primarily educational and operational in nature, rather than ideological or political. It does not explicitly align with either a liberal or conservative viewpoint as it pertains to the non-partisan issue of medical education and patient care standards. The emphasis is on ensuring that medical professionals are well-trained, informed, and competent in their procedures, which is a universal concern across the healthcare industry irrespective of political affiliations.<br><br>In the context of political discourse, "liberal" and "conservative" viewpoints generally refer to positions on government policy, individual freedoms, economic systems, and social values. This text, however, is focused on medical education and does not engage with these broader political debates. It aims at establishing a high standard of care and professionalism in family medicine, a goal that transcends political boundaries and is vital for the wellbeing of patients and the integrity of the medical profession.	neutral
AAFP	Procedural Skills, Scope of Training in Family Medicine Residencies	7/1905	The text presents a viewpoint centered on the educational standards and practices within family medicine residencies, advocating for a comprehensive and evolving curriculum that includes a wide range of procedures relevant to family medicine. This perspective primarily focuses on the improvement of medical education and healthcare delivery rather than aligning directly with traditional liberal or conservative political ideologies.<br><br>However, the emphasis on comprehensive training, including maternity care and the adaptation to new or emerging procedures, could be interpreted through various ideological lenses:<br><br>1. **Liberal Viewpoint:** From a liberal perspective, the emphasis on a broad and inclusive education that adapts to include new or emerging procedures could be seen as aligning with values of progressiveness, adaptability, and inclusiveness in healthcare. Liberals might also appreciate the focus on maternity care, viewing it as part of a comprehensive approach to reproductive health and rights.<br><br>2. **Conservative Viewpoint:** Conservatives might support the text's emphasis on maintaining high standards of medical education and ensuring that residents are prepared for a wide range of scenarios in their practice. The focus on inpatient and ambulatory settings could be seen as a commitment to traditional medical training and practice. However, they might have varied opinions on how much emphasis should be placed on new or emerging procedures, depending on their views on traditional vs. innovative practices in medicine.<br><br>Overall, the text's primary focus on medical education quality and scope within family medicine residencies transcends simple political categorization. It speaks to issues relevant to both viewpoints, such as the quality of healthcare education and the preparedness of medical professionals, without directly addressing the more divisive political issues typically associated with liberal or conservative ideologies.	neutral

AAFP	Professional Competence Evaluation	5/2023	The text you've shared focuses on the governance and oversight of medical licensure and specialty certification within the United States. Specifically, it suggests that the evaluation of competence in various medical specialties, including family medicine, should remain a responsibility of the individual specialty boards, and that medical licensure should be managed by state boards of medical examiners without being directly tied to specialty certification or recertification status.<br><br>This stance is more aligned with a viewpoint that emphasizes professional autonomy and the decentralized regulation of medical professions rather than a strictly liberal or conservative political viewpoint. It advocates for maintaining existing structures of professional self-regulation and state-level oversight rather than centralizing or federalizing these processes.<br><br>In the context of liberal versus conservative viewpoints in politics, this text doesn't neatly fit into either category because it deals with professional standards and regulatory practices rather than broader political or social ideologies. Both liberals and conservatives could find aspects of this stance to support, depending on their views on professional autonomy, state versus federal authority, and how best to ensure medical competence and protect patient safety.<br><br>- **From a liberal perspective**, there might be support for ensuring high standards of medical competence and consumer protection, possibly advocating for more uniform national standards or oversight to achieve these goals.<br><br>- **From a conservative perspective**, there might be support for the emphasis on state-level control and the autonomy of professional boards, consistent with a preference for decentralized government and the principle of subsidiarity.<br><br>Therefore, the text reflects professional and regulatory principles more than it does a clear political ideology.	neutral
AAFP	Professional Medical Liability	1/2022	The text you've provided outlines the goals and methods of the American Academy of Family Physicians (AAFP) concerning professional medical liability and insurance issues. It suggests reforms aimed at reducing the costs associated with professional liability insurance for physicians and ensuring more equitable compensation for patients harmed by medical negligence.<br><br>The viewpoint expressed in this text can be considered to align more closely with conservative political viewpoints for several reasons:<br><br>1. **Emphasis on Tort Reform**: The proposals for federal liability reforms, such as limiting payments on non-economic damages, limiting attorneys' fees, and reforming the joint and several liability rules, are commonly supported by conservative policymakers. These reforms are often advocated for under the premise that they will reduce the costs of malpractice insurance for healthcare providers and the overall costs of healthcare.<br><br>2. **Support for Non-Legislative and Legislative Solutions**: The text calls for a balance between non-legislative and legislative solutions, including tax relief, loan forgiveness, and incentives for serving in underserved areas. While advocating for government action might not exclusively be a conservative stance, the specific types of reforms proposed (e.g., tax relief) often resonate more with conservative principles of governance.<br><br>3. **Focus on Reducing Litigation**: The proposed measures aim to reduce the frequency and impact of litigation against healthcare providers by establishing higher thresholds for filing lawsuits, requiring affidavits from specialists, and encouraging alternative dispute resolution systems. These approaches are consistent with conservative views favoring less litigation and lower regulatory burdens on professionals.<br><br>4. **Advocacy for State and National Legislative Solutions**: The call for state and national legislative solutions to aid physicians reflects a belief in the need for legal and regulatory frameworks that support the medical profession and ensure its sustainability. This approach is typically associated with a conservative viewpoint that seeks to create a more favorable business environment for professionals, including healthcare providers.<br><br>However, it's important to note that the desire to ensure equitable compensation for patients and to support solutions that benefit both patients and physicians can find resonance across the political spectrum. The specific focus on reforming liability insurance and reducing litigation costs, however, leans more towards conservative policy preferences.	conservative

AAFP	Professional Medical Liability, Lawsuits	7/1905	The statement from the American Academy of Family Physicians (AAFP) denouncing the use of medical liability lawsuits as a means to pursue social and ethical policy does not neatly align with traditional liberal or conservative viewpoints as defined in American politics. The stance focuses on a specific issue within the medical and legal fields, and it addresses concerns about the implications of using the legal system to effect social or ethical changes in medicine.<br><br>Liberal viewpoints typically emphasize the use of government and legal mechanisms to protect individual rights and to promote social justice, which might include supporting the use of legal actions to address perceived injustices or unethical practices in various fields, including healthcare.<br><br>Conservative viewpoints often emphasize limited government intervention, personal responsibility, and the importance of maintaining traditional structures and practices. From this perspective, there might be support for the AAFP's stance as it advocates against using the legal system in ways that could be seen as overreach or as an inappropriate means to enact policy changes.<br><br>However, the AAFP's statement can be interpreted as focusing on the practical and professional concerns specific to the medical field, such as the impact of liability lawsuits on healthcare practices, rather than a broader ideological stance. It might be seen as advocating for the resolution of social and ethical issues within the healthcare system through means other than litigation, which could resonate with principles found across the political spectrum regarding the efficiency, effectiveness, and appropriateness of mechanisms for change.<br><br>In summary, while aspects of the statement might be more supported or criticized by one side or the other depending on how it's interpreted in the context of broader debates about the role of litigation in society, the statement itself primarily reflects a professional and sector-specific perspective rather than a straightforwardly liberal or conservative viewpoint.	neutral
AAFP	Professional Self-Regulation, Competence, and Certification of Physicians	10/2023	The text you've provided outlines the American Academy of Family Physicians' (AAFP) stance on continuing professional development, physician licensure, credentialing, and specialty board certification. It emphasizes the importance of ongoing education and self-regulation within the medical profession to ensure high-quality, cost-effective care. It also advocates for the use of a comprehensive set of criteria for physician assessment that includes, but is not limited to, specialty board certification.<br><br>This position does not align neatly with traditional political labels such as "liberal" or "conservative" because it focuses on professional standards and practices within the medical field rather than broader political or ideological issues. The viewpoints expressed relate to healthcare quality, professional development, and regulatory practices in medicine—areas that can be supported by individuals across the political spectrum for various reasons.<br><br>- **Support for Professional Development and Self-Regulation**: Advocating for continuous learning and self-regulation within a profession could be supported by both liberals and conservatives for different reasons. Liberals might focus on the aspect of ensuring high-quality care for all and the potential for reducing healthcare disparities, while conservatives might appreciate the emphasis on professional autonomy and self-regulation as opposed to heavy government regulation.<br><br>- **Position on Specialty Board Certification**: The nuanced stance on board certification—recognizing its value but opposing its use as an absolute requirement for licensure, employment, etc.—is a pragmatic approach that seeks to balance ensuring competence without unduly restricting the physician workforce. This balance might appeal to those across the political spectrum who are interested in practical solutions that ensure quality care without imposing excessive barriers.<br><br>In conclusion, the text presents a position focused on healthcare quality and professional ethics, areas that transcend simple liberal-conservative dichotomies. It reflects a professional organization's effort to navigate complex issues related to medical practice, education, and regulation in a way that promotes both quality care and professional integrity.	neutral

AAFP	Promoting Early Literacy Development	2019	The text you've provided focuses on recommendations for family physicians to promote early literacy development in children from six months to six years of age. These recommendations include advising parents and caregivers about the importance of reading aloud, counseling them on age-appropriate reading activities, and participating in early literacy programs.<br><br>This advice is primarily educational and developmental in nature, aimed at improving children's health and development outcomes. It does not explicitly align with a political ideology such as liberal or conservative viewpoints. Instead, it represents a position based on pediatric and educational research emphasizing the benefits of early literacy for children's cognitive and language development.<br><br>Both liberal and conservative viewpoints can support the promotion of early literacy, albeit for potentially different reasons. Liberals might emphasize the role of early literacy in leveling the playing field for children of all socio-economic backgrounds, seeing it as part of broader efforts to address inequalities. Conservatives might support early literacy initiatives as part of family values and personal responsibility, emphasizing the role of parents in their children's education.<br><br>The focus on early literacy as a healthcare intervention by physicians is more about applying evidence-based practices to support child development rather than promoting a specific political ideology. It suggests a multidisciplinary approach to child development that includes healthcare professionals, educators, and families working together to support the well-being and development of children.	neutral
AAFP	Protective Equipment for Recreational and Competitive Sports Activities	7/1905	The text provided aligns more closely with a pragmatic, safety-oriented viewpoint rather than a distinctly liberal or conservative political stance. It focuses on the American Academy of Family Physicians' (AAFP) recommendations for family physicians to advise patients on the use of protective equipment in recreational and competitive sports activities. The emphasis is on ensuring that the equipment has been rigorously tested and is effective in reducing the risk of injury, and on promoting access to low-cost protective equipment for youth sports activities.<br><br>This viewpoint transcends traditional political divides by focusing on public health and safety measures that are evidence-based. It advocates for responsible consumer behavior and awareness regarding the effectiveness and certification of protective sports equipment. The recommendation to provide low-cost options for protective equipment could be seen as aligning with a more inclusive, possibly progressive, approach to health and safety in sports, aiming to ensure that all children, regardless of their economic background, have access to safe sports practices. However, this is not explicitly political and is more about advocating for health and safety standards that benefit the community at large.	neutral
AAFP	Provider, Use of Term (Position Paper)	2023	The viewpoint expressed in the text regarding the American Academy of Family Physicians (AAFP) opposition to the use of the term "provider" as a substitute for "physician" does not neatly align with traditional liberal or conservative political viewpoints. Instead, it reflects a professional and terminological concern specific to the field of healthcare.<br><br>The opposition is based on the nuances of professional training, expertise, and the implications for patient care and perception. This concern transcends typical political divides and focuses on the accuracy of terminology, the recognition of professional qualifications, and the potential implications for healthcare delivery and patient understanding. The AAFP's stance is more about advocating for clarity in healthcare professional roles and maintaining the distinction between different levels of training and expertise.<br><br>However, discussions about healthcare terminology and the organization of healthcare services can sometimes intersect with broader political debates about healthcare policy, cost, access, and quality. In such contexts, different political groups might interpret or leverage the AAFP's stance in ways that align with their broader healthcare policy positions. For example, conservatives might support the distinction to emphasize the value of physician-led care and caution against cost-cutting measures that they perceive might compromise quality. Liberals might focus on the inclusivity of healthcare teams and ensuring access to a broad range of healthcare services, potentially seeing the term "provider" as a way to recognize the diverse contributions of healthcare professionals.<br><br>Yet, without more context, it's important to note that the AAFP's statement is primarily concerned with professional standards and patient care rather than being a direct commentary on broader political ideologies.	neutral

AAFP	Public Reporting of Physician Performance, Guiding Principles	2019	The text you've provided outlines a stance on public reporting of physician performance, emphasizing transparency, accountability, and improvement in healthcare quality and outcomes while also acknowledging the limitations and potential burdens of such reporting. This position does not neatly align with traditional liberal or conservative viewpoints as understood in a political context. Instead, it reflects a policy perspective focused on healthcare quality, patient empowerment, and physician accountability.<br><br>Liberal viewpoints typically support transparency and accountability in healthcare, arguing for patient empowerment and access to information that enables informed decision-making. They often advocate for measures that ensure quality care and equitable access to healthcare services. The emphasis on transparency, patient empowerment, and the importance of evidence-based criteria and the involvement of physicians in the development of performance measures could align with these liberal principles.<br><br>Conservative viewpoints, on the other hand, might also support aspects of this stance, especially the concerns over administrative burdens, the importance of physician-patient relationships, and the potential for misinterpretation or misuse of performance data. Conservatives often emphasize the autonomy of healthcare providers, caution against excessive government or third-party payer intervention, and highlight the potential for regulatory measures to impose undue burdens on healthcare professionals.<br><br>The text's balanced approach, advocating for the utility of performance reporting while cautioning against its limitations and potential misuses, reflects a nuanced perspective that seeks to improve healthcare outcomes without compromising the integrity of the physician-patient relationship or imposing excessive administrative burdens. It suggests a policy position aimed at improving healthcare quality through informed decision-making and accountability, rather than adhering strictly to a political ideology.	neutral
AAFP	Race Based Medicine	7/2020	The text you've provided aligns more closely with a liberal viewpoint, particularly in the context of how race and healthcare are discussed. Liberal perspectives often emphasize the social construction of race and the importance of considering social determinants of health, including how race as a category can impact health outcomes. The text's emphasis on the variability and poor definition of racial categories, as well as the opposition to using race as a proxy for biology or genetics in medical contexts, reflects a critique of essentialist and biological determinism perspectives that are more commonly critiqued or rejected by liberal viewpoints.<br><br>The statement from the American Academy of Family Physicians (AAFP) that opposes the use of race as a biological marker and encourages the search for alternative indicators for medical risk factors aligns with efforts to address health disparities through a more nuanced understanding of race, ethnicity, and the social factors that contribute to health outcomes. This approach is consistent with a liberal emphasis on equity and social justice in healthcare, aiming to improve health outcomes and reduce disparities by moving beyond simplistic and potentially misleading categorizations based on race.<br><br>Conservative viewpoints, while varying widely, might be more inclined to emphasize individual responsibility in health and may be more skeptical of systemic or structural explanations for health disparities, including the emphasis on race as a social construct. However, it's important to note that perspectives on race, healthcare, and the use of race in medical research can vary widely within both liberal and conservative circles, and the distinction is not always clear-cut.	liberal
AAFP	Radiology, Family Physician Interpretation of Outpatient Radiographs	10/2023	The text provided outlines a position regarding the use of diagnostic radiography in family medicine practice and does not inherently align with a liberal or conservative political viewpoint in the traditional sense. It focuses on healthcare practice, advocating for the integration of radiography in family medicine to improve patient care, access, and outcomes. The statement emphasizes that clinical privileges should be granted based on a physician's training, experience, and demonstrated competence rather than specialty designation alone. It also highlights the importance of appropriate payment for services rendered by family physicians using diagnostic radiography.<br><br>Such policy positions are more related to healthcare management and practice rather than directly reflecting political ideologies. Both liberal and conservative viewpoints can support the improvement of patient care and access, making it difficult to categorize the text strictly within a political spectrum. Discussions around healthcare often involve considerations of efficiency, quality of care, access, and cost, which can be valued across the political spectrum.<br><br>However, discussions on the allocation of resources, payment for services, and the extent of medical services provided can sometimes reflect broader political ideologies about government involvement in healthcare, individual autonomy, and market-driven versus state-driven healthcare systems. But without explicit mention of these broader issues, the text remains focused on healthcare practice improvement rather than political ideology.	neutral

AAFP	Referral, Unsolicited Laboratory	7/1905	The text you've provided outlines a policy statement from the American Academy of Family Physicians regarding the handling of unsolicited clinical test results by family physicians, particularly in situations where there is no established patient-physician relationship. This policy itself is neutral and is more focused on professional guidelines and ethical considerations in medical practice rather than aligning with a specific political or ideological viewpoint, such as liberal or conservative.<br><br>The statement is centered on the operational and ethical obligations of physicians when they encounter unsolicited test results, aiming to clarify the physician's responsibilities in these specific circumstances. It does not inherently lean towards liberal or conservative viewpoints, as it does not address or advocate for broader political, economic, or social policies that typically distinguish liberal from conservative ideologies.<br><br>Liberal and conservative viewpoints often differ on healthcare issues such as the extent of government involvement in healthcare, healthcare funding, and access to healthcare services. However, the specifics provided here are more about the practical and ethical considerations in the medical field rather than broader healthcare policies or ideological debates.	neutral
AAFP	Reparative or Conversion Therapy	1/2022	The text reflects a viewpoint that aligns more closely with liberal perspectives, especially in the context of contemporary American politics. Liberal viewpoints generally emphasize the importance of inclusivity, the protection of rights for sexual and gender minorities, and the promotion of mental health practices that do not seek to change an individual's sexual orientation or gender identity. The opposition to "reparative" or "conversion" therapy by the American Academy of Family Physicians (AAFP) is consistent with these liberal values, as such therapies have been widely discredited and condemned by major medical and psychological institutions for being unethical and harmful.<br><br>The AAFP's recommendation for patients and their families to seek services that provide accurate information on sexual orientation and gender identity, and that increase social support while reducing stigma, further underscores a commitment to supporting the well-being and rights of sexual and gender minority individuals. This approach is generally opposed by conservative viewpoints that might favor traditional or more rigid interpretations of gender and sexual orientation, and in some cases, might support the use of therapies aimed at changing these inherent aspects of a person.<br><br>It's important to note that while this text aligns with what is typically considered a liberal perspective, views on LGBTQ+ issues can vary widely among individuals, and political labels like "liberal" and "conservative" can sometimes oversimplify complex positions.	liberal
AAFP	Reporting on Residency Status of Patients	9/2022	The text you've provided reflects a viewpoint that aligns more closely with liberal ideologies. Liberal perspectives often emphasize the importance of healthcare access for all individuals, regardless of their legal residency status. They tend to support policies that protect patient confidentiality, promote inclusivity, and oppose measures that could deter vulnerable populations from seeking necessary medical care due to fear of legal consequences.<br><br>Opposition to regulations or legislation requiring health care workers to collect and report data on a patient's legal residency status is rooted in the belief that healthcare should be a universally accessible right and that such requirements could undermine public health efforts by discouraging undocumented immigrants from seeking care. This stance prioritizes patient privacy and the ethical obligation of healthcare providers to offer care without discrimination.<br><br>Conservative viewpoints, on the other hand, might support the collection of residency status information as part of broader efforts to enforce immigration laws and maintain national security. They may argue that such measures are necessary to ensure the legality of individuals within the country and to allocate resources effectively.<br><br>The American Academy of Family Physicians' (AAFP) position, as described, is indicative of a liberal approach to healthcare policy, focusing on the rights and well-being of all individuals, regardless of immigration status.	liberal

AAFP	Reproductive and Maternity Health Services	9/2022	The text you've provided outlines a position that aligns more closely with a liberal viewpoint, especially in the context of American politics. This alignment is based on several key aspects:<br><br>1. **Support for Comprehensive Reproductive Services**: The support for comprehensive pregnancy and reproductive health services, including access to abortion and contraception, is a position typically associated with liberal perspectives. Liberals often advocate for the right to choose and access to a full range of reproductive health services as part of individual rights and women's health care.<br><br>2. **Opposition to Non-evidence-based Restrictions**: The statement's opposition to "nonevidence-based restrictions" on medical care, particularly in the context of abortion and other reproductive health services, also aligns with liberal viewpoints. Liberals generally emphasize the importance of science and evidence in policy-making, especially regarding health care and women's reproductive rights.<br><br>3. **Belief in Health Care as a Right**: The assertion that pregnancy and reproductive health services are essential to general health care and should be covered under all insurance plans reflects a principle often found in liberal ideologies—that health care is a right and should be accessible to all, regardless of economic status.<br><br>4. **Infringement on Patient-Physician Relationship**: Although not detailed in your query, the reference to concerns about infringement on the patient-physician relationship typically pertains to opposition against legislative or external interference in medical decisions. This principle is often emphasized by liberals who argue that decisions about medical care, especially reproductive health care, should be made between a patient and their doctor without governmental restrictions.<br><br>In contrast, conservative viewpoints in the American political landscape tend to emphasize more restrictive policies on abortion, advocate for religious and moral considerations over scientific evidence in some cases, and may prioritize individual responsibility over the assertion of health care as a universal right. Thus, the position of the American Academy of Family Physicians (AAFP) as described would more closely align with liberal ideologies.	liberal
AAFP	Reproductive Decisions	9/2022	The text you've provided outlines a policy statement from the American Academy of Family Physicians (AAFP) regarding the approach family physicians should take in educating patients about contraceptive options, handling unintended pregnancies, and navigating situations where a physician's personal beliefs may conflict with providing certain types of reproductive health care.<br><br>This statement aligns more closely with a liberal viewpoint for several reasons:<br><br>1. **Emphasis on Comprehensive Reproductive Health Education:** The emphasis on providing patient education on contraceptive options and all available options in the event of an unintended pregnancy is consistent with liberal views on reproductive rights and the importance of access to comprehensive sexual and reproductive health information and services.<br><br>2. **Support for Patient Autonomy:** The guidance supports patient autonomy by encouraging physicians to educate patients about all options, including termination of pregnancy and adoption, and to facilitate access to these services if requested. This respect for patient choice is a hallmark of liberal perspectives on reproductive health.<br><br>3. **Requirement to Provide Care Despite Personal Beliefs:** The instruction for family physicians to refer patients to a colleague if their personal beliefs prevent them from providing the requested resources or education reflects a liberal stance on ensuring access to care regardless of individual provider beliefs. This prioritizes patient access to care over personal or religious objections, which is more aligned with liberal ideologies that emphasize the separation of personal beliefs from professional responsibilities in healthcare.<br><br>4. **Awareness of Legal Context:** The encouragement for physicians to stay informed of state and federal laws regarding reproductive health suggests a recognition of the evolving legal landscape surrounding abortion and reproductive rights, areas often subject to political and ideological battles between conservative and liberal viewpoints.<br><br>In contrast, a conservative viewpoint might emphasize more restrictions on abortion access, greater allowances for physicians to refuse to provide certain services based on personal or religious beliefs, and less emphasis on proactive patient education regarding contraceptive options and abortion.	liberal

			It's important to note that these terms, "liberal" and "conservative," can vary in meaning based on the context and region. However, within the context of U.S. politics and social policy, the AAFP's statement is more aligned with what is typically considered a liberal approach to reproductive health care and rights.	
AAFP	Reproductive Decisions, Coverage for	10/2023	The text you've provided aligns more closely with a liberal viewpoint, particularly in the context of United States political ideologies. Liberal positions often emphasize the importance of access to comprehensive healthcare services, including reproductive and pregnancy-related services, and advocate for the protection of individual rights to make decisions about one's own body. This includes support for the availability of abortion, contraception, fertility treatments, and other reproductive health services without undue restrictions.<br><br>The American Academy of Family Physicians (AAFP)'s statement supports access to a wide range of pregnancy and reproductive health services and opposes "nonevidence-based restrictions" on medical care. This stance is consistent with liberal views on reproductive rights, which argue against restrictive laws that limit access to abortion and other reproductive health services based on ideological or non-scientific grounds.<br><br>Conservative viewpoints, especially those that are more socially conservative, tend to emphasize more restrictive approaches to abortion and may prioritize policies that limit access to certain reproductive health services. Conservatives may support legislation that imposes restrictions on abortion access, often citing moral or ethical reasons, and may not prioritize the expansion of coverage for fertility treatments or contraception to the same extent as liberals.<br><br>Therefore, the AAFP's position as described would generally be seen as aligning with liberal ideologies, which prioritize individual rights to healthcare and reproductive choices, and advocate for evidence-based policies in healthcare provision.	liberal

AAFP	Reproductive Decisions, Promoting Access to Medication and Aspiration Terminations by Family Physicians	9/2022	The text you provided outlines a position of the American Academy of Family Physicians (AAFP) supporting family physicians in providing medication and aspiration terminations, which refers to abortion services. The stance aligns more closely with a liberal viewpoint for the following reasons:<br><br>1. **Support for abortion rights**: Generally, liberal or progressive political ideologies in the United States are more supportive of abortion rights and access to comprehensive reproductive health services, including abortion. They tend to advocate for the right of individuals to make decisions about their own bodies and reproductive health.<br><br>2. **Emphasis on medical autonomy and competence**: The statement emphasizes the role of trained and competent physicians in providing these services, which aligns with liberal views on ensuring access to safe and professional medical care for abortion, as opposed to restricting these services.<br><br>3. **Public health perspective**: Liberals often approach abortion from a public health perspective, advocating for access to safe and legal abortion services as a way to protect the health and well-being of individuals. The AAFP's support suggests a focus on ensuring that family physicians can provide comprehensive care, which is consistent with this view.<br><br>In contrast, conservative viewpoints in the U.S. tend to be more restrictive regarding abortion rights, often opposing or seeking to limit access to abortion services based on moral or religious grounds. Conservative positions may prioritize legislative or policy efforts to reduce the availability of abortion, emphasize alternative options like adoption, or advocate for judicial decisions that could overturn or diminish legal precedents like Roe v. Wade (though this was overturned in June 2022, which has since led to more state-level restrictions on abortion).<br><br>Therefore, the AAFP's position as described would more likely be supported by those with liberal viewpoints, reflecting a commitment to reproductive rights, medical autonomy, and the importance of access to comprehensive healthcare services.	liberal
AAFP	Reproductive Decisions, Training in	9/2022	The text you've provided outlines the American Academy of Family Physicians' (AAFP) position on training medical students and residents in a wide range of reproductive health services, including contraception, management of ectopic pregnancies, miscarriage management, and both the education and optional participation in abortion procedures. Additionally, it emphasizes the protection of healthcare professionals' moral and ethical beliefs while ensuring patients receive complete, accurate medical information and referrals for evidenced-based services.<br><br>This position can be seen as aligning more closely with a liberal viewpoint for several reasons:<br><br>1. **Comprehensive reproductive health education**: The emphasis on a broad education covering all aspects of reproductive health, including contraception and abortion, aligns with liberal views that support reproductive rights and the importance of providing comprehensive sexual and reproductive health education.<br><br>2. **Opt-in participation for abortion procedures**: The policy respects individual healthcare professionals' moral and ethical beliefs regarding abortion by making participation in such procedures opt-in. This approach attempts to balance the liberal emphasis on providing comprehensive reproductive health services with respecting individual healthcare professionals' beliefs.<br><br>3. **Obligation to provide accurate information and referrals**: The insistence on the ethical obligation to provide complete and accurate medical information and appropriate referrals, even if it involves services the physician may not provide directly (like abortion), aligns with liberal values of patient autonomy and the right to access a full range of reproductive health services.<br><br>However, the statement also reflects a sensitivity to conservative perspectives by recognizing and protecting healthcare providers' rights to opt-out of performing procedures that conflict with their moral or ethical beliefs. This aspect can be seen as a compromise aimed at respecting diverse beliefs within the medical community while still ensuring patients have access to comprehensive care.<br><br>In summary, while the AAFP's position incorporates elements designed to respect a range of beliefs among healthcare providers, its foundational support for comprehensive reproductive health education and ensuring patient access to a full spectrum of services leans more towards a liberal viewpoint.	liberal

AAFP	Research - Ethical Participation by Family Physicians	7/1905	The text provided outlines principles and standards for ethical conduct in medical research, focusing on the importance of scientific integrity, participant protection, transparency, and the management of conflicts of interest. It does not explicitly align with either a liberal or conservative viewpoint as traditionally understood in political terms. Instead, the text represents a professional and ethical stance that emphasizes the importance of rigorous scientific methodology, ethical considerations in research involving human subjects, transparency regarding sponsorship and funding, and the protection of participants' rights and well-being.<br><br>These principles are broadly accepted across the medical and scientific communities and are not typically associated with a specific political ideology. They reflect a consensus on best practices for ethical medical research, aiming to ensure that research is conducted responsibly, with respect for participants, and with the goal of advancing medical knowledge and patient care. Such guidelines are designed to uphold the integrity of the research process and protect individuals from potential harms, biases, or conflicts of interest that could undermine the scientific validity of the research or the welfare of participants.	neutral
AAFP	Research, Collaborative	5/2023	The text you've provided seems to focus on a non-partisan issue, emphasizing the importance of collaborative research among clinicians, including practice-based research networks, and researchers, with an encouragement towards the expansion of such collaboration at both national and state levels. This statement, from its content, doesn't inherently align with a liberal or conservative viewpoint as traditionally defined in political terms.<br><br>Liberal and conservative viewpoints typically differ on issues like government intervention in the economy, social welfare policies, environmental regulations, and individual liberties versus societal norms. The statement you've provided is related to the organization and promotion of scientific research, which is generally considered a neutral or bipartisan area, focusing on the improvement of healthcare outcomes through collaboration.<br><br>The emphasis on collaboration and expansion of research could be seen as aligning with broader principles of progress and innovation, which are values that can be endorsed by parties across the political spectrum. It suggests a preference for evidence-based practices and the sharing of knowledge to improve public health, rather than a stance on a specific political issue.<br><br>Thus, without additional context that might frame this endorsement in light of a broader political agenda, the statement itself does not clearly align with either a liberal or conservative viewpoint.	neutral
AAFP	Research, Family Medicine Journals	1/2022	The text you've provided appears to be focused on the policies and objectives of the American Academy of Family Physicians (AAFP) regarding the publication of medical research and evidence-based recommendations. It emphasizes the importance of maintaining strong publication venues for original research in family medicine and advocates for the inclusion of quantitative risk information in medical literature.<br><br>This text primarily aligns with professional and academic standards rather than political ideologies. It is centered on advancing scientific knowledge, improving research publication practices, and enhancing the quality of medical information available to practitioners and patients. These goals are generally viewed as non-partisan and are aimed at fostering evidence-based practice in medicine.<br><br>The focus on evidence-based recommendations, transparency in risk information, and support for scientific research does not directly align with traditional liberal or conservative political viewpoints. Instead, it reflects a commitment to scientific integrity, patient care, and the advancement of medical knowledge. Both liberal and conservative individuals within the medical community could support these principles as they transcend political affiliations and focus on the betterment of healthcare outcomes and the advancement of medical science.	neutral

AAFP	Residency Training Leading to Dual Board Certification	7/1905	The text provided does not neatly align with traditional liberal or conservative viewpoints as commonly understood in political terms. It primarily discusses the structure and policy recommendations for medical education, specifically regarding family medicine and combined residency programs. The focus is on ensuring quality education, maintaining the integrity of family medicine, addressing social needs, and regulating financial incentives in medical training. These concerns crosscut typical political lines and focus more on medical education standards, healthcare access, and professional training quality.<br><br>Political ideologies might intersect with these topics in broader discussions about healthcare policy, funding for medical education, and approaches to addressing healthcare needs in underserved areas. However, the text itself is more technical and professional in nature, aimed at outlining best practices and standards within medical education rather than advocating for a specific political or ideological stance on healthcare.<br><br>In some contexts, the emphasis on social needs and expanding access to care could resonate more with liberal viewpoints that prioritize healthcare equity and access. Meanwhile, the concern over financial incentives and ensuring efficient use of funding might align with conservative viewpoints that emphasize fiscal responsibility and efficiency. Yet, these connections are indirect, as the primary focus of the text is on educational standards and practices within the medical community.	neutral
AAFP	Resident and Student Education, Discrimination In	7/1905	The text reflects a viewpoint that is generally aligned with liberal ideologies, particularly in its emphasis on inclusivity and opposition to discrimination across a broad range of identities and statuses. Liberal perspectives typically prioritize social equality, advocate for the rights of marginalized communities, and support policies aimed at reducing discrimination and promoting diversity. The comprehensive list of categories for which discrimination is condemned, including but not limited to race, sexual orientation, gender expression/identity, and socioeconomic status, aligns with liberal values that emphasize equal rights and protections for all individuals, regardless of their background or identity.<br><br>Conservative viewpoints, on the other hand, can vary widely, but traditionally, they tend to emphasize individual responsibility, limited government intervention in personal lives, and may prioritize traditional values. While many conservatives also oppose discrimination, the extent and manner in which anti-discrimination policies are supported or opposed can differ from liberal approaches, particularly when it comes to government or institutional mandates.<br><br>It's important to note, however, that terms like "liberal" and "conservative" can encompass a wide range of beliefs and values within them, and individuals or organizations may not align perfectly with all aspects of a generalized ideological description. The American Academy of Family Physicians (AAFP), in this statement, is emphasizing its commitment to diversity and inclusion in the medical profession, which aligns with broader liberal principles of equality and anti-discrimination.	liberal

AAFP	Resident and Student Leadership	2019	The text provided reflects principles and values that could resonate with both liberal and conservative viewpoints in different ways, but it leans more towards principles often associated with a liberal viewpoint due to its emphasis on diversity, health equity, and community involvement. Here’s a breakdown of how these elements align with typical political viewpoints:<br><br>1. **Support for Professional Development and Leadership**: The commitment to the professional development of family medicine residents and medical students, including providing education, resources, leadership opportunities, and career planning, is a universally appealing principle that transcends political lines. Both liberal and conservative viewpoints generally support professional development in healthcare.<br><br>2. **Advocacy for Health and Health Equity**: The focus on advocating for health and health equity, directing initiatives, and shaping policy to improve health outcomes for all is often more closely aligned with liberal ideologies. Liberals tend to emphasize the importance of health equity and the role of social determinants in health, advocating for policies aimed at reducing health disparities.<br><br>3. **Diversity in the Workforce**: The emphasis on a diverse primary care workforce to meet the health needs of patients and the importance of engaging multiple, diverse student and resident voices are principles that are strongly supported within liberal circles. Diversity and inclusion are key liberal values, reflecting a belief in the strength of diverse perspectives in improving outcomes and fostering innovation.<br><br>4. **Community Collaboration and Catalyzing Change**: The text's emphasis on community collaboration and enabling residents and medical students to become catalysts for change in their communities is in line with a liberal emphasis on community engagement and grassroots activism as means to achieve social change and improve public health.<br><br>5. **Universal Access and Opportunity**: The call to provide opportunities for all residents and medical students to become champions for family medicine and to ensure a sufficient supply of family physicians for communities reflects a commitment to universal access to healthcare. This is a principle more commonly associated with liberal ideologies, which often prioritize expanding access to healthcare services and reducing barriers to care.<br><br>While the focus on education, professional development, and ensuring a qualified healthcare workforce can appeal to conservative values, especially regarding self-improvement and individual responsibility, the specific emphasis on diversity, equity, and community involvement aligns more closely with liberal principles. It's important to note that healthcare and education are complex fields where many values can overlap, and individuals or organizations might support these goals for a variety of reasons that transcend simple political categorization.	probably liberal

AAFP	Resident Work Hours	2020	The text provided aligns more closely with viewpoints that could be characterized as progressive, which often find a home within the broader liberal spectrum, rather than the conservative one. This assessment is based on several key factors inherent in the text:<br><br>1. **Emphasis on Worker Welfare and Safety**: The focus on reducing work hour burdens, ensuring safe working environments, and promoting the well-being of resident physicians aligns with liberal principles that prioritize worker rights and protections. Liberals often advocate for policies that protect employees from overwork, ensure safe working conditions, and promote work-life balance.<br><br>2. **Regulatory Support for Worker Protection**: The support for effective enforcement of work hour regulations and the development of mechanisms to promote physician well-being reflects a belief in the role of regulation and oversight in protecting workers. This stance is more commonly associated with liberal ideologies, which tend to support the use of government regulation to ensure fair treatment of workers and to protect their health and safety.<br><br>3. **Ethical Duty to Patients and the Profession**: The emphasis on ethical duty and the provision of safe, compassionate, quality medical care aligns with liberal values of ensuring high standards of care and the importance of healthcare as a right. The text suggests that fulfilling these duties depends on a supportive work environment, which includes limiting work hours to prevent fatigue and sleep deprivation.<br><br>4. **Structured Support and Guidelines**: The call for formal mechanisms for promoting well-being, structured support groups, and written guidelines for duty hours reflects a structured approach to addressing these issues. This approach is in line with liberal perspectives that advocate for systemic solutions and supports to address social and workplace issues.<br><br>5. **Rights to Confidential Reporting**: Advocating for residents' rights to confidentially report work hour violations is consistent with liberal values that emphasize individual rights and protections within the workplace, ensuring that workers can report abuses without fear of retribution.<br><br>While these issues can concern individuals across the political spectrum, the emphasis on regulation, worker protection, and systemic approaches to ensuring well-being and safety in the workplace are more closely aligned with liberal viewpoints. Conservative viewpoints, in contrast, might emphasize less regulatory intervention, more autonomy for programs and institutions, and a different balance between individual responsibility and systemic change.	liberal
AAFP	Residential Pool Safety	2019	The text provided outlines safety measures supported by the American Academy of Family Physicians (AAFP) regarding residential pool safety. These measures include fencing, adult training in CPR, emergency communication tools, safety equipment, constant supervision, swimming lessons, use of flotation devices, and drain safety. This text primarily represents a public health and safety perspective rather than aligning directly with a traditional political viewpoint such as liberal or conservative.<br><br>However, the emphasis on regulation and safety measures could be interpreted through the lens of political ideologies:<br><br>- **Liberal Viewpoint**: Liberals often advocate for regulations and measures that protect public health and safety, especially for children. The support for mandatory safety measures, such as fencing and the requirement for CPR training, aligns with the liberal emphasis on the role of oversight and regulation in preventing accidents and ensuring the well-being of the community. Thus, one could argue that the text aligns with a liberal viewpoint in its advocacy for preventative measures and regulations to ensure safety.<br><br>- **Conservative Viewpoint**: Conservatives typically prioritize individual freedoms and responsibilities, and may argue that while safety is important, mandatory regulations can be seen as an overreach of government authority. However, conservatives also value the protection of family and children, and may see the importance of such measures in preventing tragedies. The text does not explicitly call for government enforcement of these measures, which could make it more palatable from a conservative standpoint, as it could be seen as guidance rather than mandatory regulation.<br><br>In summary, while the text promotes safety measures that could be seen as aligning with a more regulatory or liberal approach to public health and safety, its primary focus is on the non-partisan issue of preventing accidents and ensuring the safety of children around residential pools. Therefore, it might be more accurate to say that the text aligns with a public safety perspective rather than a strictly political one.	probably liberal

AAFP	Restraints on Incarcerated Women During Labor, Use of	2020	The text presents a viewpoint that aligns more closely with liberal ideologies, particularly in its emphasis on human rights and the health and welfare of pregnant women who are incarcerated. Liberal perspectives typically prioritize individual freedoms, human rights, and social justice issues, and this statement from the American Academy of Family Physicians (AAFP) reflects concerns over the treatment of vulnerable populations and the negative impacts of certain practices on health outcomes.<br><br>The recommendation to limit the use of restraints on pregnant incarcerated women, citing the potential negative impacts on maternal and fetal health, and the call for policies that ensure the least restrictive approaches align with liberal values that prioritize compassion, dignity, and the health and safety of individuals, especially those in vulnerable conditions. The reference to the negative implications of shackling during pregnancy also suggests a critique of harsh penal practices, which is a stance more commonly associated with liberal rather than conservative viewpoints, as conservatives might focus more on law and order perspectives that prioritize security and discipline.<br><br>In contrast, conservative viewpoints might emphasize the importance of security measures to maintain order and safety within correctional facilities, potentially supporting the use of restraints as necessary for preventing harm or escape. However, the text advocates for a balance between security needs and the health and welfare of pregnant women, suggesting that restraints should only be used when there is an "imminent risk of harm to others or of escape."<br><br>Overall, the emphasis on humane treatment, health outcomes, and the call for policy changes to protect vulnerable populations indicate a perspective more aligned with liberal ideologies.	liberal
AAFP	Retail Clinics	2019	The text provided outlines the position of the American Academy of Family Physicians (AAFP) regarding the integration of retail clinics into the broader health care system, with a strong emphasis on maintaining a patient-centered, primary care-focused approach. This viewpoint can be analyzed through both liberal and conservative lenses, as it incorporates elements that resonate with both ideologies.<br><br>From a **liberal perspective**, the emphasis on patient-centered care, the importance of access to convenient and transparent health care, and the encouragement of evidence-based care management protocols align with a focus on comprehensive health care access for all. Liberals often advocate for health care systems that are inclusive, equitable, and that prioritize the health and well-being of patients over profit margins. The AAFP's stance on ensuring that retail clinics support a strong patient-physician relationship and do not fragment care could be seen as aligning with these values.<br><br>On the other hand, the **conservative viewpoint** might also find alignment with the text, especially in its endorsement of market-based developments in health care delivery and the support for patient choice. Conservatives typically support free-market solutions to health care, arguing that competition and consumer choice can lead to improvements in quality and efficiency. The text's emphasis on collaboration between primary care and retail clinics, as well as the requirement for physician-led care, could be interpreted as a nod to maintaining high standards of care within a market-driven environment.<br><br>However, the text does not strongly advocate for or against government intervention in health care, nor does it explicitly address health care financing, which are often key points of divergence between liberal and conservative health care policies. Instead, it focuses on clinical and operational standards for retail clinics to ensure they complement the broader goals of patient-centered, comprehensive care provided by primary care physicians and medical homes.<br><br>Therefore, the text could be seen as straddling both liberal and conservative perspectives, emphasizing elements like patient choice and market-based solutions (more conservative) while also advocating for coordinated, comprehensive care that ensures quality and access (more liberal). The primary focus is on how retail clinics can integrate into and support a health care system that prioritizes the patient's well-being and continuity of care, rather than pushing a specific political ideology.	neutral

AAFP	Rights, Protections, and Support for Survivors of Sexual Assault	2020	The text you've shared primarily focuses on the recognition of sexual violence as a serious public health issue and advocates for the rights and protections of survivors of sexual violence within the legal and healthcare systems. The stance taken by the American Academy of Family Physicians (AAFP) in supporting survivors of sexual violence, emphasizing compassionate treatment, and advocating for a legal framework that protects their rights, is generally aligned with views that prioritize individual rights, victim support, and public health measures. These views can be found across the political spectrum but are more commonly associated with liberal viewpoints, especially given the emphasis on comprehensive rights for survivors, legal protections, and the critique of re-victimization practices.<br><br>Liberal viewpoints often emphasize the importance of human rights, social justice, and the role of the government in protecting vulnerable populations and ensuring equitable treatment under the law. The text's emphasis on protecting survivors from re-victimization, supporting their well-being, and advocating for a legal framework to codify these protections aligns with these liberal principles. However, it's also worth noting that concern for victims of sexual violence and the desire for effective legal protections can transcend political divisions, with individuals from various political backgrounds recognizing the importance of addressing and preventing sexual violence.<br><br>The specific policies and approaches advocated for in the text, such as protection against re-victimization in legal proceedings and the prioritization of survivor well-being, are indicative of a progressive approach to addressing sexual violence. This approach not only seeks to punish perpetrators but also to provide comprehensive support and protections for survivors, suggesting a more holistic view of justice and recovery.<br><br>It's important to recognize that while these views might more closely align with what is typically considered liberal, the fight against sexual violence and support for survivors is a concern that cuts across political lines, with many conservatives also supporting measures to protect and support survivors. The framing and priorities might differ, but the core concern for justice and support can be shared across the spectrum.	liberal
AAFP	Role Definition of Family Medicine	2019	The text you've provided outlines the role and value of family physicians in the health care system. It emphasizes the comprehensive, personalized, and preventative nature of their care, as well as their role in managing health care systems and partnering with public health initiatives. This description focuses on the importance of accessible, continuous, and integrated health care, which are principles that can be supported by individuals across the political spectrum.<br><br>However, the emphasis on broad access to personalized health care, prevention, management of illness, and the integration of health care systems could align more closely with a liberal viewpoint in the context of health care policy. Liberal viewpoints often emphasize the importance of accessible and equitable health care for all, the role of preventative care, and the integration of health care services to improve health outcomes. They may also support the idea of health care systems being led by physicians who prioritize patient-centered care and public health partnerships.<br><br>Conversely, conservative viewpoints might focus more on market-driven solutions to health care, individual responsibility for health, and less government involvement in health care systems. However, the text does not directly address these aspects, and the value of family physicians as described can be appreciated by those with conservative views as well, especially in terms of efficiency, personalized care, and the use of data to improve health care delivery.<br><br>Therefore, while the text might resonate more with liberal viewpoints on health care accessibility and system integration, its focus on efficient, data-driven, and patient-centered care has broad appeal that transcends simple political categorization. It emphasizes values and practices that are essential to the effective delivery of health care, regardless of political beliefs.	probably liberal

AAFP	Rural Graduate Medical Education (GME) Policy	1/2022	The text outlines principles and supports for expanding and enhancing Graduate Medical Education (GME) in rural areas. This approach emphasizes the importance of training and retaining a medical workforce in rural communities to address healthcare access and equity. The principles focus on broadening the scope of GME training, engaging communities, advocating for targeted funding, building institutional support, maintaining educational standards with flexibility for rural settings, and supporting ongoing evaluation and evidence dissemination related to rural GME.<br><br>These principles could be aligned with both liberal and conservative viewpoints, depending on the context of their advocacy and implementation. However, they lean more towards a liberal viewpoint in several ways:<br><br>1. **Emphasis on Equity and Access:** The focus on healthcare equity and access to care in rural areas aligns with liberal principles that prioritize reducing disparities and ensuring equitable access to healthcare services for all populations, including underserved rural communities.<br><br>2. **Support for Federal and State Funding:** Advocating for federal and state GME funding to support rural programs is consistent with liberal viewpoints that support government intervention and funding as mechanisms to address public needs and societal inequalities, including in healthcare.<br><br>3. **Community Engagement and Public Health:** The emphasis on community engagement and addressing the social determinants of health reflects a liberal approach to healthcare, recognizing the role of social and community factors in health outcomes and the importance of public health initiatives.<br><br>4. **Flexibility and Accreditation Standards:** Supporting accreditation standards that provide flexibility for rural GME while maintaining educational standards demonstrates a balance between ensuring quality healthcare education and adapting to the unique needs of rural settings, which aligns with liberal values of innovation and adaptability in public services.<br><br>However, it's important to note that the principles also embody values that can resonate with conservative viewpoints, particularly in their emphasis on local community engagement and partnerships with existing healthcare institutions. The focus on practical solutions to healthcare workforce shortages in rural areas, through targeted training and support, can appeal to conservative principles of localized decision-making and community-based approaches to problem-solving.<br><br>Overall, while the text aligns more closely with liberal values of equity, government support, and comprehensive public health strategies, its pragmatic approach to addressing rural healthcare challenges through education, community engagement, and partnerships can find support across the political spectrum.	probably liberal

AAFP	Rural Health Care in Medical Education	2020	The text provided outlines policy recommendations and support for initiatives aimed at improving medical education and increasing the supply of physicians in rural areas. It advocates for:<br><br>1. Inclusion of rural medicine curriculum in medical education.<br>2. Federal and state funding for medical schools focused on graduating rural physicians.<br>3. Redesign of graduate medical education funding to support rural-based residency programs.<br>4. Increased flexibility in medical education curriculum design to meet the needs of rural healthcare.<br>5. Partnerships and financial incentives to encourage training of rural physicians.<br><br>This stance emphasizes the importance of accessible healthcare in rural areas, support for medical education reforms, and the role of government funding and incentives in achieving these goals.<br><br>**Liberal vs. Conservative Viewpoint:**<br><br>- **Liberal Perspective:** The recommendations align more closely with a liberal viewpoint, as they advocate for increased government involvement and funding in healthcare education, particularly in underserved rural areas. Liberals typically support the expansion of healthcare access and the use of federal and state resources to address disparities in healthcare availability. The emphasis on government-funded programs and partnerships to improve rural healthcare access reflects a belief in the positive role of government intervention to achieve social equity.<br><br>- **Conservative Perspective:** While conservatives also recognize the importance of healthcare access, they might have reservations about the methods proposed. Conservatives generally prefer solutions that rely more on private sector initiatives and less on government funding and intervention. They might support some aspects of the recommendations, such as the importance of rural healthcare, but could prefer alternative methods for achieving these goals, such as market-driven solutions or decentralized decision-making rather than federal mandates or funding.<br><br>The emphasis on federal and state funding, legislative initiatives, and flexibility in education accreditation standards signifies a policy approach that seeks to address systemic issues through coordinated efforts between government entities and educational institutions. This approach is typically more aligned with liberal policy perspectives, which advocate for the role of government in addressing societal needs and inequalities, especially in sectors like healthcare and education.	liberal
AAFP	Rural Health Care, "First Responder" Training	7/1905	The text provided suggests a viewpoint focused on enhancing community health and safety through "first responder" training, especially in rural areas. This proposal aims to empower community members with the skills needed to respond to emergencies, thereby potentially reducing the time it takes for professional medical help to arrive. The stance does not inherently align exclusively with either a liberal or conservative viewpoint, as the emphasis is on practical, community-based health initiatives, which can be supported across the political spectrum.<br><br>- **Liberal Viewpoint**: A liberal perspective might support this initiative as part of broader healthcare and community support programs. Liberals often advocate for comprehensive healthcare services and preventive measures to ensure communities are well-equipped to handle emergencies. The focus on community empowerment and health education aligns with liberal values of community support, accessibility, and inclusiveness in healthcare.<br><br>- **Conservative Viewpoint**: A conservative perspective might also support this initiative, emphasizing self-reliance, community responsibility, and local solutions to local problems. Conservatives often favor initiatives that empower individuals and communities to take care of themselves without relying heavily on government intervention. The proposal for "first responder" training in rural areas could be seen as a way to enhance community resilience and self-sufficiency, principles often valued by conservatives.<br><br>In summary, the proposal for "first responder" training in rural communities can appeal to both liberal and conservative viewpoints, though for different reasons. Liberals may focus on the aspect of enhancing healthcare accessibility and community welfare, while conservatives may see it as a means of promoting self-reliance and local problem-solving. This demonstrates how certain healthcare and safety initiatives can transcend traditional political divides by addressing universal concerns about community well-being and emergency preparedness.	neutral

AAFP	Rural Health Care, Access to	2020	The statement from the American Academy of Family Physicians (AAFP) about supporting programs and initiatives for financial stability and delivery system support for physicians in rural communities, aimed at eliminating disparities in access to quality care for all populations, aligns more closely with principles typically associated with a liberal viewpoint. This alignment is based on several core aspects:<br><br>1. **Emphasis on Equity in Healthcare:** The liberal viewpoint often prioritizes policies and initiatives that aim to reduce disparities and achieve equity in healthcare access. The AAFP's statement focuses on eliminating disparities in access to quality care, especially for rural communities, which is a goal closely aligned with liberal policies that emphasize social equity and justice.<br><br>2. **Support for Government or Community Interventions:** The statement implies support for programs and initiatives that could involve government action or community support to ensure financial stability for physicians in underserved areas. This approach is consistent with liberal perspectives that advocate for a proactive role of government in addressing social issues, including healthcare.<br><br>3. **Focus on Vulnerable Populations:** The liberal viewpoint typically highlights the need to support vulnerable and underserved populations. The AAFP's statement on supporting physicians in rural communities directly aims to improve healthcare access for populations that are often underserved or overlooked, aligning with liberal priorities of inclusiveness and support for vulnerable groups.<br><br>However, it's important to note that support for improving healthcare access and addressing disparities can also be found across the political spectrum, and there might be conservative viewpoints that also support such initiatives, albeit through different approaches or policies. The distinction often lies in the methods and extent of government involvement rather than the goal of improving healthcare access itself.	liberal
AAFP	Rural Practice, Keeping Physicians In (Position Paper)	2014	The text outlines a comprehensive overview of the challenges and solutions associated with recruiting and retaining physicians in rural areas. It advocates for increased recruitment of medical students from rural backgrounds, enhanced training in rural medical practices, and improved funding and support for rural medical education. The emphasis on equitable healthcare access, the focus on family medicine, and the call for government action to support rural physicians suggest an alignment with a more progressive or liberal viewpoint.<br><br>Liberal viewpoints often prioritize equal access to healthcare and may advocate for government intervention to address inequalities, such as the rural-urban healthcare gap. The text's focus on systemic changes in medical education, support for family medicine, and the critical role of government in ensuring equitable healthcare access align with liberal priorities of inclusivity, community support, and proactive government involvement in addressing societal issues.<br><br>However, it's important to note that the issue of rural healthcare access can transcend traditional liberal-conservative divides, appealing to a broad spectrum of political beliefs due to its focus on improving healthcare outcomes for underserved populations.	liberal
AAFP	Safe Driving	1/2022	The statement from the American Academy of Family Physicians (AAFP) focuses on public health and safety measures regarding driving and does not directly align with a traditionally liberal or conservative viewpoint in the political sense. It emphasizes the importance of evaluating and reducing motor vehicle fatalities and injuries through various means, including legislation to ban text messaging while driving and support for healthcare providers who report impairment concerns.<br><br>This perspective is rooted in a concern for public safety and health, which transcends political affiliations. Both liberal and conservative viewpoints can support such measures, although the approach to and emphasis on regulation and individual freedoms might vary between the two. Liberals might emphasize the role of government in ensuring public safety and health, advocating for stricter regulations to protect individuals. Conservatives might focus on personal responsibility and the importance of not overly burdening individuals or businesses with regulations, although they may also support measures that clearly benefit public safety.<br><br>The specifics of the AAFP's statement, such as supporting efforts to reduce driver distraction and impairment and encouraging family physicians to discuss safe driving, are generally non-partisan and align with the broader goal of enhancing public health and safety.	neutral

AAFP	School Commute and Transportation Safety	10/2023	The text you've shared articulates a viewpoint that aligns more closely with liberal ideologies, especially in its emphasis on equity, safety, and broad legislative involvement in the management of school transportation. Liberal perspectives often prioritize government intervention to ensure public safety, equitable access to services, and the protection of vulnerable populations. This viewpoint is reflected in the call for comprehensive research, regulations, and specific standards for all modes of school transportation, with a particular focus on equity for low-income students, students of color, students with disabilities, and those living in rural areas.<br><br>Conservative viewpoints, on the other hand, might emphasize limited government intervention, local control over education and transportation issues, and may prioritize cost-efficiency and the role of personal responsibility (e.g., parental choices about school transportation) over broad legislative mandates.<br><br>The mention of "legislative and educational efforts," "mandatory special licensing and training for school bus drivers," and ensuring "fair outcomes of all school transportation methods to ensure equity" are indicative of a desire for government oversight and action to address systemic issues, which is more aligned with liberal principles. The focus on equity and inclusivity across various demographics also highlights a liberal orientation towards policy-making that seeks to actively rectify disparities and promote social justice.	liberal
AAFP	School Nutrition: Healthy Eating Options in Schools	10/2023	The text provided reflects a viewpoint that emphasizes public health, nutrition education, and community involvement in school environments. These themes are often associated with a liberal viewpoint, especially in the context of advocating for policy changes and government involvement to ensure healthier living conditions for children and communities. Liberal perspectives typically support the idea of using government resources and regulations to promote public health initiatives, ensure access to healthy food in schools, and involve various stakeholders (students, parents, educators, etc.) in decision-making processes.<br><br>The focus on:<br><br>- Ensuring sound nutrition in schools<br>- Involvement of a broad group of stakeholders in planning and decision-making<br>- The call for adequate funding from local, state, and federal sources<br>- The integration of behavior-focused nutrition education throughout all grade levels<br>- Encouraging the incorporation of school gardens and locally grown foods<br>- Meeting diverse cultural and taste preferences<br>- Providing adequate space and pleasant surroundings for eating<br><br>These points align with a liberal approach to public health and education, advocating for systemic changes to support healthier lifestyles. Conservatives might also support aspects of healthy eating and nutrition education but may have different views on the extent of government involvement, funding sources, and the role of personal responsibility versus public regulation in achieving these goals.<br><br>It's important to note that the concepts of healthy eating and nutrition can be supported across the political spectrum, but the methods of implementation and the emphasis on government involvement versus market-based solutions or individual choices might vary between liberal and conservative viewpoints.	liberal

AAFP	School Safety and Student Mental Health	7/1905	The text from the American Academy of Family Physicians (AAFP) largely aligns with viewpoints that are typically considered liberal, particularly in the context of its emphasis on comprehensive school safety, mental health support, and the integration of schools within a broader community health framework. This perspective is characterized by a focus on preventive measures, the importance of addressing social and emotional learning, and the support for multi-disciplinary approaches to student wellness and safety.<br><br>Liberal viewpoints often prioritize the role of social support systems and the government in ensuring the welfare of individuals, especially children. The text's emphasis on the need for safe environments free from violence and substance abuse, the importance of mental health support, and the advocacy for collaborative efforts between healthcare providers and educational institutions reflect a commitment to these principles. Furthermore, the support for evidence-based intervention and the Whole School, Whole Community, Whole Child model by the CDC underlines a holistic approach to child welfare that goes beyond traditional academic learning to include physical and mental health, which is consistent with liberal values focused on comprehensive well-being and community involvement.<br><br>However, it's essential to note that issues of school safety and mental health support can transcend political divides, and there may be conservative viewpoints that also support various aspects of the AAFP's position, especially regarding the importance of safe learning environments. The distinction often comes in the methods and policies proposed to achieve these goals, with conservative approaches typically emphasizing different strategies or priorities.<br><br>The alignment with liberal or conservative viewpoints can also vary depending on specific policies or the political climate. What is crucial is the focus on child welfare, safety, and health as foundational to successful education and overall well-being.	liberal
AAFP	School-Based Health Clinics, Guidelines	7/1905	The text provided appears to lean more towards a liberal viewpoint rather than a conservative one. This assessment is based on several key aspects of the content and the policies it advocates for:<br><br>1. **Access to Comprehensive Healthcare**: The emphasis on ensuring all children and adolescents have access to high-quality, continuous, and comprehensive health care services aligns with liberal values that prioritize universal healthcare access and equity in healthcare services.<br><br>2. **School-Based Health Clinic Programs**: The support for school-based health clinic programs, especially in areas where healthcare needs are unmet, reflects a liberal approach to addressing social determinants of health and reducing healthcare disparities by integrating services into community settings.<br><br>3. **Impact on Academic Achievement**: Highlighting the positive impact of health services on academic achievement, high school graduation rates, and student engagement supports a holistic view of child development. This perspective is typically more associated with liberal ideologies that consider education and healthcare as interconnected facets of public welfare.<br><br>4. **Comprehensive Clinical Services**: The inclusion of services such as contraceptive care and sexually transmitted infection screenings within school-based health clinics is a stance more commonly supported by liberal viewpoints. These services are part of a broader liberal agenda to promote sexual and reproductive health and rights, especially among adolescents.<br><br>5. **Collaboration and Confidentiality**: The call for cooperation between school-based programs and medical homes, along with ensuring confidentiality for adolescents, reflects liberal values of privacy, individual rights, and the importance of integrated care models.<br><br>6. **Development of Policies by a Diverse Council**: Advocating for the development of health policies by a council that includes a wide range of community stakeholders aligns with liberal principles of inclusivity, community engagement, and shared decision-making.<br><br>While these positions might be supported by individuals across the political spectrum to varying degrees, the emphasis on universal access, comprehensive services, reproductive rights, and the integration of health services into educational settings are more closely aligned with liberal policies and ideologies. Conservative viewpoints might prioritize different approaches to healthcare, focusing more on parental rights, local control over education and health policies, and potentially more restrictive views on reproductive health services in schools.	liberal

AAFP	Screening	10/2023	The text provided outlines a medical and health policy perspective that emphasizes individualized care, evidence-based practice, and shared decision-making between patients and physicians. It does not explicitly align with traditional political ideologies such as liberal or conservative viewpoints. Instead, it focuses on medical best practices and the importance of considering a range of factors (including individual risk factors, evidence-based approaches, and the social determinants of health) when deciding on screening for asymptomatic diseases.<br><br>However, some elements of the text could be interpreted in a way that resonates with broader political or ideological themes:<br><br>1. **Emphasis on Evidence-Based Practices and Individualized Care**: The focus on evidence-based screening and the avoidance of mass or direct-to-consumer screenings that are not evidence-based can appeal to those who advocate for scientifically informed approaches to healthcare, a stance often associated with liberal viewpoints. However, the emphasis on individual choice and consultation with a physician also aligns with conservative values that prioritize personal freedom and individual decision-making.<br><br>2. **Caution Against Race-Based Approaches**: The caution against race-based approaches to screening, with an emphasis on the social and systemic determinants of health as drivers of health inequities, aligns with liberal viewpoints that focus on addressing systemic inequities and social determinants of health. This perspective recognizes the complex factors contributing to health disparities beyond biological differences, a stance that seeks to address broader social and systemic issues.<br><br>3. **Shared Decision-Making**: The advocacy for shared decision-making between patients and physicians reflects a patient-centered approach to healthcare, emphasizing the importance of respecting patients' values and preferences. This approach is generally supported across the political spectrum but can be particularly resonant with liberal ideologies that emphasize empowerment and patient rights.<br><br>In summary, while the text primarily presents a medical and health policy perspective centered on best practices and patient care, aspects of it can be interpreted in ways that align with broader ideological themes. The focus on evidence-based, individualized care, caution against race-based screening, and emphasis on addressing social determinants of health can resonate with liberal viewpoints, but the text itself is focused on medical practice rather than political ideology.	probably liberal
AAFP	Seal, AAFP	3/2023	The text provided appears to focus on the values, symbolism, and goals of the American Academy of Family Physicians (AAFP) rather than aligning directly with either a liberal or conservative political viewpoint. It emphasizes the importance of family medicine within the American health care system, highlighting aspects like enlightenment, healing, honor, and the foundational role of family physicians in providing quality, cost-effective health care.<br><br>The symbols mentioned (the torch, serpent encircled staff, and the tagline) and the narrative about the AAFP's commitment to strengthening the position of family physicians in healthcare reflect professional and institutional values. These are oriented towards enhancing the respect, awareness, and effectiveness of family medicine rather than promoting a specific political ideology.<br><br>The emphasis on cost-effective, quality health care and the positioning of family physicians as crucial to the health care system can resonate with broad, bipartisan values—affordable and effective health care is a priority across the political spectrum. However, how these goals are achieved can sometimes be viewed through ideological lenses, with different opinions on the role of government, private sector, and regulatory frameworks in achieving these outcomes.<br><br>In summary, the text is professional and institutional in nature, focusing on the mission and values of the AAFP rather than aligning explicitly with liberal or conservative viewpoints.	neutral
AAFP	Seal, AAFP, Use of	2/2018	The text you've provided appears to be a formal statement regarding the use of the American Academy of Family Physicians (AAFP) Seal, detailing policies on its usage, endorsement implications, and the process required for obtaining permission to use it. This content is administrative and procedural in nature, focusing on intellectual property rights and organizational governance rather than expressing or aligning with political or ideological viewpoints typically associated with liberal or conservative perspectives.<br><br>The statement outlines the conditions under which the AAFP Seal can be used, emphasizing the need for written permission and the process through which such requests are reviewed. This kind of text is neutral and does not engage with political or social issues that would categorize it as liberal or conservative. It is more about the internal governance and policy enforcement of an organization, in this case, the AAFP, than about broader political or ideological debates.	neutral

AAFP	Sensitivity to Diversity and Cultural Proficiency in AAFP Education	7/2020	The text you've provided describes a commitment by the American Academy of Family Physicians (AAFP) to improve family physicians' ability to provide high-quality care to all patient populations, with a focus on cultural proficiency, sensitivity to diverse populations, and addressing health disparities. This approach aligns more closely with principles commonly associated with liberal viewpoints, particularly the emphasis on inclusivity, equity, and addressing disparities among diverse populations. Liberal perspectives often prioritize social justice, equity in healthcare, and the recognition of systemic barriers that different groups may face.<br><br>Conservative viewpoints, while also valuing high-quality healthcare, may place a stronger emphasis on individual responsibility and may be less focused on systemic or structural factors contributing to health disparities. The conservative approach to healthcare reform often centers on market-driven solutions and personal choice within the healthcare system.<br><br>However, it's important to note that the commitment to improving healthcare quality and accessibility can be a non-partisan issue, supported by individuals across the political spectrum. The focus on cultural proficiency and addressing specific health disparities as mentioned in the text specifically aligns with broader goals of achieving equity in healthcare, which is a key aspect of many liberal healthcare policies.	liberal
AAFP	Separation of Families	5/2023	The text provided appears to align more closely with a liberal viewpoint. The focus on the psychological and emotional well-being of children, the opposition to forced separation of families at borders, and the emphasis on the potential long-term negative health consequences of such policies are topics generally more emphasized and supported by liberal ideologies. Liberal perspectives often prioritize humanitarian concerns, the protection of human rights, and the well-being of individuals, particularly vulnerable populations such as children. The opposition to practices that are seen as harmful to individuals' mental and physical health, such as the forced separation of children from their families, is consistent with these values.<br><br>Conservative viewpoints, on the other hand, might prioritize national security, immigration control, and the enforcement of existing laws, potentially viewing strict border policies as necessary measures, even if they result in family separations. However, it's important to note that there can be a wide range of opinions within any political ideology, and not all conservatives may support family separation policies.<br><br>The American Academy of Family Physicians (AAFP)'s statement emphasizes the health and well-being of children, a stance that is generally more associated with liberal policies that focus on the humanitarian aspects of immigration and the rights of individuals.	liberal
AAFP	Serious Reportable Events and Hospital Acquired Conditions	7/1905	The text primarily focuses on healthcare improvement, emphasizing evidence-based guidelines, the elimination of serious reportable events (SREs), and hospital-acquired conditions (HACs), and advocating for performance improvement measures in healthcare settings. This perspective aligns more closely with principles that are generally supported across the political spectrum in the context of healthcare quality and safety improvement. The emphasis on evidence-based practice, quality improvement, and patient safety are topics that find support among both liberal and conservative viewpoints, as they pertain to enhancing the effectiveness and efficiency of healthcare delivery, reducing wasteful spending, and improving patient outcomes.<br><br>However, there are a few nuances that might make it more appealing to one side or the other based on how they are implemented or discussed in the broader political context:<br><br>1. **Liberal Viewpoint Alignment**: The focus on systemic change and the call for further research to improve healthcare outcomes can resonate with liberal ideologies that emphasize the role of government and organized systems in promoting public health, ensuring patient safety, and supporting scientific research to inform policy and practice.<br><br>2. **Conservative Viewpoint Considerations**: Conservatives might also support these initiatives, especially if the approaches emphasize cost-effectiveness, reduction in wasteful spending, and improvements in healthcare quality that do not necessitate significant government intervention beyond what is necessary to ensure safety and efficacy. The support might be particularly strong if the strategies include market-driven solutions and private sector innovation.<br><br>In essence, while the text does not explicitly align with either a strictly liberal or conservative viewpoint, its emphasis on evidence-based healthcare improvement is broadly compatible with agendas that aim to enhance the quality, safety, and efficiency of healthcare, albeit through potentially different mechanisms or policy approaches. The specific alignment might then depend on the broader context of how these initiatives are proposed, funded, and implemented, including the roles of government, private sector, and healthcare professionals.	neutral

AAFP	Sexual Assault as a Public Health Issue	7/1905	The text you've provided outlines a viewpoint focused on public health and safety, particularly regarding sexual assault. It emphasizes the importance of education on sexual consent and support for survivors of sexual assault. This perspective is generally aligned with principles that prioritize individual rights, victim support, and public health awareness, which can be associated with liberal viewpoints. However, the acknowledgment of sexual assault as a serious issue and the emphasis on education and support for victims are concerns that cross political lines and could also find support among conservatives who prioritize public safety and justice for crimes.<br><br>In the context of American politics, liberal viewpoints often emphasize social justice, healthcare access, and comprehensive sex education, which aligns with the emphasis on educating patients about sexual consent and supporting survivors of sexual assault. Conservatives might also support efforts to combat sexual assault but may differ in approaches to education and public health initiatives.<br><br>Therefore, while the focus on public health, victim support, and education about consent aligns more closely with liberal viewpoints, the fundamental concern for addressing and preventing sexual assault transcends traditional liberal-conservative divides, reflecting a broader societal concern.	probably liberal
AAFP	Shared Medical Appointments/Group Visits	10/2023	The text provided outlines the concept of shared medical appointments or group visits, highlighting their benefits and providing recommendations for their implementation and billing. This approach to healthcare delivery does not inherently align exclusively with either a liberal or conservative viewpoint as it focuses on practical, healthcare delivery methods rather than political ideology.<br><br>However, aspects of the text can be interpreted to align with values typically associated with both political perspectives:<br><br>1. **Liberal Viewpoint Alignment**: The emphasis on collaborative healthcare, patient empowerment, and the holistic inclusion of various healthcare professionals (such as behaviorists, nutritionists, or health educators) reflects a more communal, inclusive approach to healthcare. This could resonate with liberal values that prioritize community-oriented solutions and comprehensive healthcare access. Furthermore, the suggestion that third-party payers (insurance companies) should cover these appointments and possibly waive out-of-pocket costs to incentivize participation aligns with liberal advocacy for reducing financial barriers to healthcare access.<br><br>2. **Conservative Viewpoint Considerations**: The text also touches upon efficiency in healthcare delivery, patient satisfaction, and improving outcomes, which can resonate with conservative values that emphasize efficient use of resources, personal responsibility (as seen in the encouragement of patient self-care), and the freedom for practices to utilize existing billing codes for reimbursement. The mention of fee-for-service coding and third-party payer coverage may also align with conservative preferences for market-based healthcare solutions and minimal regulatory interference in the billing process.<br><br>The mention of both enhancing patient self-care and the role of third-party payers could be seen as attempting to balance individual responsibility with systemic support, suggesting a pragmatic approach to healthcare that seeks to integrate beneficial practices within the existing framework.<br><br>In essence, while the text promotes a specific healthcare delivery model focused on shared medical appointments, its alignment with political ideologies depends on which aspects of the approach one emphasizes—community and accessibility (more liberal) or efficiency and market-based solutions (more conservative). It appears to aim for a middle ground, advocating for a healthcare innovation that could theoretically garner support from a broad spectrum of political viewpoints for its potential to improve patient care and satisfaction.	neutral

AAFP	Sign Language Translation Services	10/2023	The statement from the American Academy of Family Physicians (AAFP) regarding support for payment for all interpretation services, including sign language regardless of delivery format, and including coverage by public and private payers, aligns more closely with viewpoints often associated with liberal ideologies. This stance reflects principles of inclusivity, accessibility, and equity in healthcare, which are typically emphasized in liberal perspectives. Liberal ideologies often advocate for policies that ensure equal access to services for all individuals, including those with disabilities, and support the role of both public and private sectors in funding these services.<br><br>Conservative viewpoints, on the other hand, might emphasize less government intervention in private sectors, including healthcare, and could advocate for a more market-driven approach to service provision and payment. However, it's important to note that issues of accessibility and support for individuals with disabilities can cross traditional political lines, and there may be support for such measures from individuals and groups across the political spectrum.<br><br>The specific emphasis on comprehensive coverage by both public and private payers for interpretation services, including sign language, underscores a commitment to ensuring that all patients have equal access to healthcare services, which is a key aspect of healthcare equity—a principle widely supported in liberal ideologies.	liberal
AAFP	Specialty Hospitals	10/2023	The text you've provided outlines a position from the American Academy of Family Physicians (AAFP) that calls for clearer definitions and stricter enforcement by the Centers for Medicare and Medicaid Services (CMS) regarding the "whole hospital" exception to the prohibition of self-referrals by physicians. This statement is more focused on healthcare policy and regulation rather than directly aligning with traditional liberal or conservative viewpoints.<br><br>However, its implications can be interpreted in ways that resonate with broader political and ideological beliefs:<br><br>1. **Liberal viewpoint considerations**: Liberals often advocate for stricter regulations in healthcare to ensure fairness, prevent conflicts of interest, and protect patient care. They may support this statement as it calls for clearer definitions and stricter enforcement of regulations. The emphasis on regulation could be seen as aligning with a liberal viewpoint that seeks to ensure healthcare equity and prevent any form of exploitation or unethical practices.<br><br>2. **Conservative viewpoint considerations**: Conservatives typically favor less government intervention in business practices, including healthcare, and might be more skeptical of increased regulations. However, they also advocate for transparency and efficiency in healthcare to ensure it remains high-quality and accessible without unnecessary government overreach. The call for strict enforcement to prevent self-referrals might be seen as a measure to increase transparency and efficiency, which could align with conservative principles to some extent.<br><br>The statement's focus on specific regulatory practices within the healthcare system makes it more a matter of professional ethics and healthcare policy rather than a clear-cut issue along liberal or conservative lines. It reflects concerns about ethical practices, conflict of interest, and ensuring that healthcare services are provided in the best interest of patients, which are values that can cross ideological boundaries.	neutral
AAFP	Sports Medicine, Counseling About Risk of Contact/Collision Sports	7/1905	The text you've provided appears to focus on medical advice and safety recommendations from the American Academy of Family Physicians (AAFP) regarding participation in contact and collision sports. It emphasizes the importance of family physicians understanding and communicating the risks associated with these sports, including gender-based differences, and outlines a responsible approach to managing and minimizing these risks. The guidance focuses on ensuring patient safety and health through informed decisions and protocols for returning to play post-injury.<br><br>This text does not inherently align with a liberal or conservative viewpoint as it primarily addresses health and safety concerns in sports from a medical perspective. The advice aims to enhance awareness and understanding among physicians and patients about the potential risks involved in contact and collision sports and to promote safe practices. It reflects a commitment to patient care and injury prevention rather than a political or ideological stance.<br><br>Issues related to sports, health, and safety can become politicized in broader discussions, particularly when gender differences or protocols for dealing with injuries such as concussions are involved. However, the text itself is neutral, focusing on medical advice and best practices rather than engaging with political or ideological debates.	neutral

AAFP	Sports Medicine, Health and Fitness	9/2022	The text primarily presents a viewpoint focused on health, fitness, and the role of family physicians in providing comprehensive care, including sports medicine and preventive services. It does not neatly align with traditional liberal or conservative political viewpoints as it centers on medical practice and patient care rather than explicit political ideologies. However, some aspects of the text could be interpreted as aligning more closely with certain values typically associated with liberal viewpoints:<br><br>1. **Emphasis on Equity and Inclusion:** The encouragement of equity and inclusion in team sports and physical fitness activities, including for patients with disabilities and gender-diverse patients, aligns with liberal values that prioritize diversity, equity, and inclusion across all sectors of society, including healthcare.<br><br>2. **Comprehensive Healthcare Approach:** The focus on comprehensive medical management and preventive care, including the support for continued medical education and patient education on health, fitness, and nutrition, reflects a holistic approach to healthcare. This can be seen as aligning with more liberal policies that advocate for preventive care and comprehensive healthcare access.<br><br>3. **Support for Gender-Diverse Patients:** Specifically encouraging full and equal participation in affirmed gender by gender-diverse patients is consistent with liberal viewpoints that support the rights and recognition of LGBTQ+ individuals.<br><br>While these aspects might resonate more with liberal viewpoints due to the emphasis on inclusion, equity, and comprehensive healthcare, the core message of promoting health and fitness through family medicine is broadly applicable across the political spectrum. The text focuses on medical practice and patient care principles that can be endorsed by individuals with a wide range of political beliefs.	liberal
AAFP	Sports Medicine, Persons with Disabilities: Participation in Sports and Physical Activities	10/2023	The text aligns more closely with a viewpoint that transcends the traditional liberal-conservative spectrum, focusing instead on inclusivity, health, and accessibility for persons with disabilities. It emphasizes the importance of participation in sports and physical activities for individuals with disabilities, advocating for informed family physicians, adaptive equipment, and accessible facilities. This approach is rooted in principles of equal opportunity, improved health outcomes, and independence for people with disabilities, which are values that can be endorsed by a broad range of political and social perspectives.<br><br>In the context of political ideologies, both liberal and conservative viewpoints can support the inclusion and accommodation of individuals with disabilities, albeit possibly for different underlying reasons. Liberals might emphasize equality, social justice, and the role of government in ensuring access and rights for all individuals, including those with disabilities. Conservatives might support these measures as well, potentially focusing on individual liberties, the importance of community support, and private sector solutions to achieve similar goals.<br><br>Therefore, the statement by the AAFP (American Academy of Family Physicians) is more closely aligned with a universally humanistic perspective that prioritizes health, well-being, and inclusivity, rather than being easily categorized into a conventional political ideology.	neutral
AAFP	Stimulant Drinks and Products	7/1905	The text provided doesn't neatly align with traditional liberal or conservative viewpoints as commonly understood in U.S. politics. Instead, it focuses on public health concerns related to the consumption of energy drinks and related stimulant products, particularly among young people, and calls for regulatory action by the Food and Drug Administration (FDA). However, the emphasis on regulation, public health, and consumer protection can be interpreted through the lens of broader political ideologies:<br><br>- **Liberal Viewpoint Alignment:** The call for FDA regulation, standardization of labeling, ongoing monitoring of ingredients, and the concern for public health and safety, particularly for minors, aligns with principles often associated with liberal ideologies. Liberals generally support a more active role for government in regulating businesses to protect consumers, especially vulnerable populations like children and those with health conditions. The emphasis on evidence-based policy and consumer protection could be seen as consistent with liberal priorities.<br><br>- **Conservative Viewpoint Consideration:** While conservative ideologies typically emphasize free market principles and limited government intervention in business, there is also a conservative perspective that values individual responsibility and the protection of family and community health. Some conservatives may support regulations that they see as necessary to protect public health, especially when it concerns children and adolescents. However, the emphasis on new regulations might be viewed with skepticism by those who prioritize deregulation and believe that market forces should dictate product availability and consumer choice.<br><br>The content of this text primarily reflects concerns about public health and safety rather than economic or ideological principles,	neutral

			making it a matter more of public policy and health policy than of partisan politics. It suggests a stance that is proactive in the regulation of products that could pose health risks, a position that can find support across the political spectrum, depending on the context and framing of the issue.	
AAFP	Striving for Birth Equity	7/1905	The text presents viewpoints and recommendations that align more closely with liberal viewpoints, particularly in its focus on addressing systemic inequalities, institutional racism, and the disparities in health outcomes based on race, income, and geographical location. Liberal ideologies often emphasize the importance of social justice, equity, and the role of systemic factors in individual and community health outcomes. The emphasis on educational efforts to address inequities, the acknowledgment of institutional racism, and the commitment to birth equity and the conditions for optimal births for all people reflect principles commonly associated with liberal perspectives on health care and social policies.<br><br>Conservative viewpoints, while also concerned with health outcomes and the quality of care, might focus more on individual responsibility, market-driven solutions to healthcare issues, and less government intervention in addressing social inequities. The text’s emphasis on systemic and institutional factors and the call for a concerted effort to address racial and social inequalities in health care delivery diverges from these conservative principles.<br><br>The text’s approach is indicative of a broader public health perspective that recognizes the complex interplay of social determinants of health and the need for systemic changes to improve health outcomes for marginalized and underserved populations.	liberal
AAFP	Striving for Birth Equity: Family Medicine's Role in Overcoming Disparities in Maternal Morbidity and Mortality	7/2020	The text presents a viewpoint that aligns more closely with liberal ideologies, primarily due to its emphasis on systemic inequalities, the call for broad-based policy changes, the support for Medicaid expansion, and the focus on addressing racial, economic, and geographical disparities in maternal health outcomes. The emphasis on structural and institutional barriers, implicit bias in healthcare, and the need for federal support and policy interventions to improve maternal morbidity and mortality rates reflect concerns typically associated with liberal perspectives on healthcare and social justice.<br><br>Conservative viewpoints, by contrast, might focus more on individual responsibility, market-driven solutions, and limited government intervention in healthcare. The text's support for actionable solutions backed by policy changes, advocacy for Medicaid expansion, and emphasis on addressing social determinants of health diverge from these conservative principles.<br><br>Furthermore, the text highlights the importance of education on implicit bias and advocates for systemic changes to address health disparities, which aligns with liberal approaches to addressing inequalities in healthcare. The call for collaboration across various stakeholders to implement evidence-based strategies and the emphasis on social justice and equity in healthcare also reflect liberal values.<br><br>In summary, the text's focus on addressing systemic inequalities, advocating for policy changes, and emphasizing social determinants of health aligns more with liberal ideologies concerning healthcare and social equity.	liberal

AAFP	Student Choice of Family Medicine, Incentives for Increasing	1/2022	The text you provided leans more towards a liberal viewpoint, primarily due to its emphasis on equitable access to healthcare, diversity in medical education, and the role of government and other entities in supporting primary care. The call for holistic review in medical school admissions, focus on underserved populations, and advocacy for federal and state policies to support family medicine resonate with liberal values that prioritize social equity, access to services for all segments of the population, and an active role for government in addressing societal needs.<br><br>The liberal viewpoint often emphasizes the importance of social determinants of health, the need for diversity and inclusion in all sectors (including medicine), and the belief in government intervention to correct market failures or inequities. The text aligns with these principles through its:<br><br>- Advocacy for programs that encourage a diverse and equitable primary care workforce.<br>- Support for educational reforms that prioritize exposure to family medicine and primary care from an early age.<br>- Call for policy changes and financial incentives to make family medicine a more attractive and feasible career path.<br><br>Conservative viewpoints, while also supportive of effective healthcare, might focus more on market-driven solutions, reduced government intervention, and personal responsibility in healthcare decisions. The approach outlined in the text, with its emphasis on systemic reforms, educational initiatives, and government and institutional support, is more aligned with liberal approaches to healthcare reform.	liberal
AAFP	Student-Run Free Clinics	10/2023	The text provided reflects viewpoints that align more closely with liberal ideologies, particularly those related to healthcare policy. The key aspects of the statement that suggest a liberal orientation include:<br><br>1. **Support for Health as a Basic Human Right**: The assertion that health is a basic human right for every person is a principle more commonly associated with liberal or progressive ideologies. This viewpoint emphasizes the importance of equitable access to healthcare services for all individuals, regardless of their socio-economic status.<br><br>2. **Advocacy for Student-Run Free Clinics**: The support for student-run free clinics to provide essential healthcare services for uninsured and other underserved populations reflects a commitment to addressing inequalities in healthcare access. This approach is typically advocated by those on the liberal end of the political spectrum, who prioritize social justice and equity in healthcare.<br><br>3. **Inclusion of Family Physicians in Student-Run Free Clinics**: The emphasis on comprehensive, patient-centered care and the engagement of medical students with family medicine are indicative of a broader liberal viewpoint on education and healthcare delivery. It underscores the importance of holistic care, community engagement, and the nurturing of future healthcare professionals with a strong sense of social responsibility.<br><br>4. **Focus on Community and Comprehensive Care**: The text emphasizes delivering essential patient-centered care within the context of the community and providing comprehensive medical care for all age groups. This focus on community-based, holistic care approaches is more aligned with liberal perspectives that advocate for integrated, accessible healthcare systems designed to meet the needs of diverse populations.<br><br>In contrast, conservative viewpoints on healthcare might emphasize more market-driven approaches, personal responsibility for health and healthcare, and a more limited role for government in healthcare provision. While there is diversity within both liberal and conservative perspectives on healthcare, the specific aspects highlighted in the text—such as health as a human right, support for free clinics for underserved populations, and a focus on comprehensive, community-based care—tend to align more closely with liberal ideologies.	liberal

AAFP	Substance Use Disorder Education for Students and Residents	9/2022	The text outlines a comprehensive approach to substance use disorder (SUD) education for students and family medicine residents, emphasizing harm reduction, social equity, patient-centered care, and advocacy. This perspective aligns more closely with viewpoints typically associated with liberal ideologies. Liberal viewpoints often prioritize holistic, inclusive, and evidence-based approaches to healthcare, focusing on reducing inequalities and addressing the social determinants of health. The emphasis on harm reduction, addressing social inequities, breaking down stigma, and advocating for patient rights and voices in policy and program development reflects a progressive approach to drug policy and treatment.<br><br>Conservative viewpoints, on the other hand, might prioritize more traditional approaches to drug use and addiction, often focusing on law enforcement, abstinence-only treatment models, and personal responsibility over systemic factors contributing to SUD. While there's a growing recognition across the political spectrum of the need for evidence-based treatment and the failure of punitive drug policies, the comprehensive and inclusive approach outlined in the text, especially the focus on social inequities and harm reduction, is more aligned with liberal principles.<br><br>In summary, the text's approach to SUD education, with its emphasis on comprehensive, evidence-based, and inclusive strategies, aligns more with liberal viewpoints on healthcare and drug policy.	liberal
AAFP	Substance Use Disorders	7/1905	The text you've provided outlines a comprehensive approach to addressing substance use disorders (SUDs), including prevention, treatment, harm reduction, and advocacy for policy changes. It does not align strictly with traditional liberal or conservative viewpoints as it encompasses elements that both sides of the political spectrum might support or critique depending on their stance on public health, drug policy, and healthcare access.<br><br>**Liberal Viewpoint Alignment:**<br>- **Harm Reduction Strategies:** The support for harm reduction strategies such as needle exchange programs and naloxone distribution aligns with liberal policies that focus on reducing the immediate harms associated with drug use, rather than strictly enforcing drug abstinence.<br>- **Decriminalization and Treatment Over Incarceration:** The text's emphasis on decriminalization of marijuana possession and the focus on treatment over incarceration for drug use is more aligned with liberal viewpoints that prioritize public health approaches over criminal justice solutions to drug problems.<br>- **Advocacy for Healthcare Parity and Access:** The call for parity in substance use disorder treatment in health care plans and the removal of barriers to access medication-assisted treatment (MAT) reflects a liberal inclination towards ensuring equal access to healthcare services and reducing stigma associated with SUDs.<br><br>**Conservative Viewpoint Elements:**<br>- **Opposition to Recreational Marijuana Use:** The opposition to the recreational use of marijuana, while supporting decriminalization, might resonate with more conservative views that are cautious about fully legalizing substances without more research into their effects.<br>- **Support for Law Enforcement and Regulation:** The emphasis on reinforcing laws and strategies to limit substance misuse and advocating for legislation to facilitate prevention and treatment can align with conservative perspectives that value law and order as well as structured regulatory frameworks for managing public health issues.<br><br>However, it's important to note that the approach outlined in the text is multifaceted and does not neatly fit into a single political ideology. It reflects a public health perspective that integrates evidence-based practices for the comprehensive management of SUDs, including education, treatment, prevention, and support for affected individuals and communities. This approach is indicative of a growing consensus in many parts of the world that complex health issues like SUDs require nuanced, multidisciplinary strategies that transcend traditional political divisions.	neutral

AAFP	Sugar Sweetened Beverages	7/1905	The statement about the American Academy of Family Physicians (AAFP) supporting the taxation of sugar-sweetened beverages to reduce over-consumption, with the aim of improving public health and combating the obesity epidemic, and directing tax revenues towards public health improvement programs, tends to align more closely with liberal viewpoints for several reasons:<br><br>1. **Public Health Intervention**: Liberals are generally more supportive of government interventions in public health issues, especially if those interventions aim to address widespread health concerns like obesity. The idea of using taxation as a tool to modify consumer behavior for health benefits fits within this perspective.<br><br>2. **Governmental Role in Healthcare**: The suggestion that tax monies should be directed towards programs that improve public health reflects a belief in a significant role for government in healthcare. Liberals typically advocate for stronger government involvement in healthcare to ensure equitable access and address public health challenges.<br><br>3. **Regulatory Approach to Nutrition and Health**: Liberals often support regulatory approaches to address nutritional and health issues, including policies that discourage unhealthy consumption patterns through taxation or restrictions. The proposal to tax sugar-sweetened beverages as a method to combat obesity aligns with this approach.<br><br>Conversely, conservative viewpoints tend to emphasize personal responsibility over government intervention in matters of nutrition and health. Conservatives are more likely to oppose such taxes, viewing them as overreach by the government into individual choices and the free market. They might argue that education and personal choice are better methods to address health issues like obesity rather than imposing taxes.<br><br>It's important to note, however, that these alignments are not absolute, and there can be diversity of opinion within each political ideology. Furthermore, public health policies like this can sometimes gain support across the political spectrum, depending on the context and how the policies are framed.	liberal
AAFP	Sunscreen Usage in Schools	2019	The text outlines a position on public health and safety, specifically focusing on measures to prevent harmful sun exposure for students in a school setting. It advocates for education about sun safety, the provision of shaded areas, careful scheduling to avoid peak UV exposure times, encouragement of protective clothing, and policies allowing students to use sunscreen without unnecessary barriers.<br><br>This position does not neatly align with traditional liberal or conservative political viewpoints as they are commonly understood in the United States. Instead, it focuses on practical health considerations and safety measures for children. Both liberal and conservative viewpoints might support these measures, albeit for different underlying reasons:<br><br>- **Liberal viewpoint alignment** might be seen in the emphasis on proactive public health measures, educational initiatives, and removing bureaucratic obstacles to health care access (in this case, allowing students to use sunscreen without a doctor's note). Liberals often prioritize public health initiatives and might see this as part of broader efforts to ensure safety and well-being in schools.<br><br>- **Conservative viewpoint alignment** could be inferred in the focus on local control and parental permission for sunscreen application, reflecting a preference for individual choice and minimal regulatory hurdles. Conservatives might also support the idea of preventing health issues through personal responsibility (such as wearing sun-protective clothing) and community-based solutions (like creating shaded play areas).<br><br>However, the text primarily represents a non-partisan, health-focused stance rather than a political one. Public health and safety measures, especially those concerning children, often receive bipartisan support. The specifics—such as the emphasis on local decision-making versus federal guidelines, or the balance between individual freedoms and collective safety—might elicit varied responses along the political spectrum, but the text itself is centered on pragmatic health advice rather than ideological positions.	neutral

AAFP	Teaching, Physician Responsibility	2020	The text you provided focuses on the importance of developing a strong and diverse family medicine workforce and emphasizes the need for trained preceptors, support for physician education, and efforts to build a diverse workforce that addresses health disparities. This text aligns more closely with viewpoints typically associated with liberal ideologies rather than conservative ones, for several reasons:<br><br>1. **Emphasis on Diversity and Health Disparities**: The call to build a diverse workforce and address health disparities resonates with liberal viewpoints, which often prioritize equity, diversity, and the reduction of inequalities in healthcare.<br><br>2. **Support for Education and Training**: The emphasis on actively engaging in physician education and supporting efforts to enhance training resources aligns with liberal perspectives that support investment in education and training programs.<br><br>3. **Community Engagement and Broad Recruitment**: The text's focus on recruiting and retaining faculty and preceptors from a wide range of backgrounds to represent the "depth and breadth of family medicine" aligns with liberal values of inclusivity and community engagement.<br><br>However, it's important to note that supporting a strong healthcare workforce, emphasizing education, and aiming to reduce health disparities are issues that can also find support across the political spectrum. The focus on practical improvements in healthcare education and workforce development can resonate with conservative viewpoints as well, especially when these efforts are seen as ways to improve the quality and efficiency of healthcare delivery.<br><br>The distinction between liberal and conservative viewpoints often comes down to the methods and policies proposed to achieve these goals, such as the level of government involvement or the emphasis on private sector solutions. This text, however, focuses more on the goals and less on specific policies, making it primarily aligned with values typically supported by liberals but not exclusively so.	liberal
AAFP	Team-Based Care	10/2023	The text you've provided focuses on the importance of physician-led, integrated care teams in the primary care setting, emphasizing collaboration among health professionals to serve the best interest of patients. This perspective aligns more with a viewpoint that values evidence-based, collaborative approaches to healthcare delivery, rather than fitting neatly into traditional liberal or conservative political categories.<br><br>Liberal viewpoints on healthcare often stress the importance of accessible, universal healthcare coverage, focusing on the role of government in ensuring healthcare access for all citizens. Conservatives, on the other hand, might emphasize the role of private sector solutions and personal responsibility in healthcare, often advocating for less government involvement.<br><br>The text's emphasis on physician-led teams and multidisciplinary collaboration can be seen as a focus on improving healthcare quality and outcomes, a goal that can attract support from across the political spectrum. It advocates for utilizing the full extent of professionals' training and abilities within a structured team environment to provide high-value and accessible healthcare. This approach is consistent with initiatives to improve healthcare efficiency and effectiveness, which can be supported by both liberals and conservatives for different reasons.<br><br>Thus, while the text outlines a specific approach to healthcare delivery, it doesn't strictly align with liberal or conservative viewpoints but rather focuses on practical, outcomes-based healthcare delivery improvements.	neutral

AAFP	Telehealth and Telemedicine	1/2022	The text provided outlines a position that supports the expanded use of telehealth and telemedicine, emphasizing the importance of accessibility, standardization, patient safety, and physician responsibility. It advocates for policies that ensure continuity of care, enhance access to medical services across state lines, and support telemedicine through educational initiatives, payment model adjustments, and addressing socioeconomic disparities. This perspective aligns more closely with principles typically associated with a liberal viewpoint, particularly in its emphasis on:<br><br>1. **Healthcare Accessibility and Equity**: The text advocates for broadening access to healthcare services through telehealth, which is a priority often associated with liberal policies. The focus on ensuring that telemedicine does not widen disparities among vulnerable populations reflects a liberal concern for equity in healthcare access.<br><br>2. **Regulatory Adaptations for Improved Care**: The call for streamlined licensure processes and reciprocity compacts to facilitate cross-state telemedicine services reflects a willingness to adapt regulatory frameworks to improve healthcare delivery, a stance often found in liberal healthcare policy discussions.<br><br>3. **Support for Value-Based and Patient-Centered Care**: The emphasis on payment models that support patient choice and adequately reimburse telemedicine services aligns with liberal perspectives on healthcare reform, focusing on value-based care that prioritizes patient outcomes and choices.<br><br>4. **Educational and Training Initiatives**: Advocating for medical education that includes telemedicine reflects a forward-thinking approach to healthcare delivery, emphasizing the importance of preparing healthcare professionals for evolving practices, a principle consistent with progressive views on education and healthcare innovation.<br><br>5. **Addressing Social Determinants of Health**: Acknowledging the role of broadband access as a social determinant of health and advocating for universal access to support telehealth services mirrors liberal concerns for addressing broader socioeconomic factors affecting health.<br><br>It's important to note that while these positions align more closely with liberal viewpoints, the support for telehealth and telemedicine transcends traditional political divisions. Many aspects of telehealth policy, such as improving access and efficiency in healthcare, can also find support among conservatives, especially when focusing on reducing regulatory barriers and supporting innovation. However, the specific emphasis on equity, regulatory adaptation for accessibility, and comprehensive support systems for telehealth implementation as presented in the text are more characteristic of liberal policy priorities.	liberal
AAFP	Television and Digital Media, Ethics	1/2022	The text provided reflects a viewpoint that aligns more closely with liberal ideologies than conservative ones, primarily due to its emphasis on social equity, the portrayal of positive social behaviors, and concerns over public health issues. Here's a breakdown of how these elements fit within the liberal-conservative spectrum:<br><br>1. **Social Equity:** The mention of promoting social equity is a hallmark of liberal ideologies, which often focus on reducing inequalities and ensuring equal opportunities for all individuals, regardless of their background.<br><br>2. **Healthy Lifestyles and Public Health:** The emphasis on encouraging healthy lifestyles and the caution against modeling violence, the use of tobacco, vapes, e-cigarettes, illicit drugs, and the misuse of alcohol align with a public health perspective that is generally more emphasized in liberal politics. Liberals tend to support government intervention in public health to improve outcomes and reduce health disparities.<br><br>3. **Positive Social Behaviors:** The call for programming that promotes positive social behaviors indicates a preference for media that fosters a cooperative, inclusive society. This viewpoint often aligns with liberal perspectives, which emphasize community and social responsibility.<br><br>4. **Accurate Portrayal of Social and Political Issues:** The desire for accurate portrayals of social and political issues suggests an emphasis on truthfulness and awareness, values that are not exclusive to either liberal or conservative ideologies but can align with liberal emphasis on education and informed citizenship.<br><br>5. **Avoidance of Discriminatory Behaviors:** The stance against modeling discriminatory behaviors reflects liberal principles of equality and anti-discrimination. Liberals typically advocate for the rights of marginalized groups and the importance of representing all individuals fairly and without bias.	liberal

			While these positions align more closely with liberal viewpoints, it's essential to recognize that public health and the desire for positive social outcomes can transcend political boundaries. Conservative viewpoints may also support aspects of healthy living or accurate media portrayals but might differ in approaches to achieving these goals or in the emphasis on certain issues over others. The text's focus on social equity and explicit mention of avoiding discriminatory behaviors particularly highlights its alignment with liberal ideologies.	
AAFP	Third-Party Credentialing	10/2023	The text reflects a viewpoint that leans more towards a liberal perspective, particularly in the context of health care policy. The emphasis on administrative simplification, reducing barriers to physician credentialing, and ensuring fair compensation and transparency in the credentialing process align with liberal principles of increasing access to healthcare, reducing inefficiencies within the healthcare system, and promoting equity and fairness for healthcare providers.<br><br>Liberal perspectives often advocate for streamlined healthcare processes, increased government oversight or intervention to ensure equitable treatment, and policies that aim to reduce administrative burdens on healthcare providers. The suggestions made by the American Academy of Family Physicians (AAFP) for a single, nationally standardized credentialing application, along with specific recommendations for payer practices, reflect these liberal ideals by advocating for a more organized, efficient, and fair healthcare system that supports both providers and patients.<br><br>Conservative viewpoints, on the other hand, might emphasize less government involvement in healthcare, more market-driven solutions, and potentially less regulation around credentialing and payer practices, prioritizing the autonomy of insurance companies and healthcare institutions over a standardized approach.<br><br>The AAFP's stance seeks to balance the needs of healthcare providers with the operational realities of payers but does so with a clear emphasis on fairness, efficiency, and accessibility, which are more closely aligned with liberal values in the context of healthcare reform.	liberal

AAFP	Tiered and Narrowed Physician Networks	10/2023	The text reflects principles and viewpoints that align with a mix of values that could be appreciated by both liberal and conservative perspectives, making it more centrist or bipartisan in nature. However, the emphasis on ensuring access to healthcare, supporting the physician-patient relationship, focusing on improving patient care, and advocating for transparency and equity in physician performance measurement and patient steering could be seen as aligning more closely with principles often emphasized by liberal viewpoints. These principles include:<br><br>- **Access to Healthcare:** Advocating for sufficient access to healthcare for patients, which includes opposition to practices that might restrict access such as tiering, narrowing, or terminating family physicians from networks. This is a principle often associated with liberal views, which generally support wider access to healthcare for all.<br>- **Emphasis on Quality of Care:** The focus on improving patient care and ensuring that quality-of-care assessments are a prominent feature of steering programs aligns with a value placed on the welfare of the individual patient and the community, a stance often found in liberal healthcare policy.<br>- **Transparency and Equity:** Calling for transparency in data methodology used to tier, rate, designate, or exclude family physicians, and ensuring that all patient data used to evaluate a physician is adjusted for age, gender, severity, and socioeconomic factors. These principles reflect a liberal emphasis on fairness and equity in healthcare.<br><br>On the other hand, the text does not exclusively align with liberal viewpoints. It also emphasizes the importance of physician-patient relationships and the efficiency of healthcare delivery, which can resonate with conservative values that prioritize personal responsibility, the value of individual relationships over systemic approaches, and efficient operation of the healthcare system to prevent unnecessary expenditures.<br><br>Moreover, the call for mechanisms to ensure patient access to a primary care physician and for insurers to cover out-of-network services without additional cost to the patient could be seen as advocating for a balance between market-driven healthcare systems and the need for regulatory measures to protect patient interests, a balance that might appeal to moderate or centrist views within the political spectrum.<br><br>In essence, the text represents a healthcare policy perspective that prioritizes patient care, continuity of healthcare, and transparency, which transcends simple liberal-conservative categorizations by incorporating elements important to both sides of the political spectrum.	probably liberal
AAFP	Tobacco Advertising and Labeling	7/1905	The text outlines the American Academy of Family Physicians' (AAFP) stance on tobacco product advertisements, including Electronic Nicotine Delivery Systems (ENDS), and their advocacy for measures to reduce tobacco use and exposure to tobacco advertising. This position includes opposition to all forms of tobacco advertisement, support for removing corporate tax deductions for such advertising, urging prominently displayed health hazard warnings on tobacco products, and advocating for "plain pack" packaging to reduce brand appeal.<br><br>This viewpoint aligns more closely with liberal or progressive public health policies, which typically emphasize the role of regulation and public interventions in promoting health and safety. These policies often prioritize preventive measures to tackle public health issues, such as tobacco use, and seek to reduce the impact of industries that are seen as harmful to public health. The focus on restricting advertising, particularly to protect children, and the support for state and community efforts to limit tobacco exposure are indicative of a public health-oriented approach that values collective well-being and the role of government and organizations in safeguarding health.<br><br>Conservative viewpoints, in contrast, might prioritize individual freedom, including the freedom of businesses to advertise, and may be more inclined to resist government interventions that are seen as limiting personal or corporate liberties. However, it's important to note that public health issues, such as tobacco control, can sometimes garner bipartisan support, especially when the focus is on protecting children or reducing healthcare costs associated with smoking-related diseases.<br><br>Thus, while the AAFP's stance on tobacco advertising and packaging might more naturally align with liberal public health policy perspectives, public health issues like tobacco use prevention can transcend traditional liberal-conservative divides, depending on the specific context and framing of the issue.	liberal

AAFP	Tobacco: Preventing and Treating Nicotine Dependence and Tobacco Use (Position Paper)	1/2022	The text you've provided outlines a comprehensive and detailed perspective on the harmful effects of tobacco use, the importance of tobacco cessation, and a call for action to reduce smoking rates and protect individuals from secondhand smoke. It emphasizes the need for evidence-based policy changes, community and state advocacy, national advocacy, and the role of family physicians in tobacco cessation efforts. Additionally, it calls for increased regulation of tobacco and nicotine products, including e-cigarettes and ENDS (Electronic Nicotine Delivery Systems), and advocates for health care facilities to be tobacco-free.<br><br>This text can be seen as aligning with viewpoints commonly associated with both liberal and conservative perspectives, depending on the specific aspect being considered. However, the focus on public health, regulation of tobacco products, advocacy for government action to protect public health, and support for health care interventions are often more closely associated with liberal viewpoints. Liberal perspectives typically emphasize the role of government in regulating industries to protect public health and the environment, as well as the importance of public health initiatives and access to health care services.<br><br>Conservative viewpoints, on the other hand, often prioritize personal responsibility, free market principles, and limited government intervention in businesses. However, the issue of tobacco regulation has seen support from across the political spectrum due to the clear evidence of the harm tobacco use causes to individuals and society. Thus, while the emphasis on regulation and public health intervention may more closely align with liberal viewpoints, the universal concern for public health and the economic burden of tobacco-related diseases can also resonate with conservative values, especially when considering the long-term benefits of reducing health care costs and improving public health.<br><br>Therefore, while some elements of the text may align more closely with liberal viewpoints, particularly in terms of regulatory and public health perspectives, the overall concern for reducing the impact of tobacco use is a bipartisan issue that transcends traditional liberal-conservative divides.	probably liberal
AAFP	Transparency	10/2023	The text reflects a viewpoint that emphasizes transparency in health care, advocating for the sharing of information related to payment, coverage, and evaluation of health care services among various stakeholders, including public and private payers, hospital systems, and other care delivery organizations. This perspective advocates for the open disclosure of data, methodologies, and policies that affect patient care and the evaluation of healthcare providers.<br><br>This viewpoint aligns more closely with principles typically associated with a liberal perspective, particularly in its emphasis on transparency, accountability, and the equitable distribution of information. Liberals often advocate for policies that ensure the public has access to information that affects their lives and well-being, including in the healthcare sector. They may argue that such transparency is crucial for patient rights, enabling individuals to make informed decisions about their health care, and for ensuring fairness in how healthcare services are provided and evaluated.<br><br>However, it's important to note that the call for transparency in healthcare can also find support across the political spectrum. Many conservatives may also support transparency as a means to promote competition, improve quality, and reduce healthcare costs. The emphasis on transparency and accountability can be seen as a non-partisan issue that aims to improve the healthcare system for all stakeholders involved.<br><br>Thus, while the text aligns with principles often associated with liberal viewpoints, the call for transparency in healthcare transcends traditional political boundaries and can be supported by individuals with a wide range of political beliefs. The focus on transparency is fundamentally about improving the healthcare system, making it more accountable, and ensuring that individuals have the information they need to make informed decisions about their care.	probably liberal

AAFP	Trauma-Informed Care	1/2022	The text you've provided focuses on Trauma-Informed Care (TIC) and its principles, emphasizing a compassionate and comprehensive approach to healthcare that acknowledges and addresses the impact of trauma on individuals' physical and mental health. This approach is patient-centered and seeks to create an environment that is sensitive to the needs of those who have experienced trauma, without necessarily requiring them to disclose their traumatic experiences. The text also highlights the importance of incorporating TIC into the training of family physicians and the development of workplace policies that support the wellbeing of healthcare professionals and their patients.<br><br>This text does not neatly align with traditional political labels such as "liberal" or "conservative" in a direct sense because it focuses on healthcare practices rather than explicit political ideologies or policies. However, the emphasis on systemic factors (such as systemic racism) and the holistic approach to care could be seen as aligning more closely with viewpoints traditionally associated with liberal ideologies, which often prioritize social equity, the acknowledgment of systemic issues, and comprehensive welfare policies. Liberal viewpoints tend to emphasize the importance of addressing social determinants of health and might advocate for policies and practices that consider the broader context of individuals' lives, including their experiences of trauma.<br><br>On the other hand, conservative viewpoints typically emphasize personal responsibility, limited government intervention in healthcare, and may prioritize individualized approaches to care over systemic analyses. However, this does not mean that conservative viewpoints oppose compassionate care or the acknowledgment of trauma; rather, the emphasis and framing of solutions might differ.<br><br>It's important to note that the implementation of Trauma-Informed Care practices is primarily a healthcare issue rather than a partisan one. Healthcare professionals across the political spectrum can support TIC principles based on the evidence supporting their effectiveness in improving patient care and outcomes. The focus on trauma-informed care reflects a growing recognition within the healthcare field of the need to address the holistic needs of patients in a way that is sensitive to their experiences and backgrounds.	neutral
AAFP	Treatment of Survivors of Sexual Assault	7/1905	The text outlines a policy position from the American Academy of Family Physicians (AAFP) regarding the care of survivors of sexual assault. It emphasizes comprehensive medical care, including treatment of physical injuries, access to emergency contraception, forensic evidence collection, mental health support, and prophylactic treatment for sexually transmitted diseases. This position primarily aligns with what are generally considered liberal viewpoints for several reasons:<br><br>1. **Access to Emergency Contraception**: The emphasis on ensuring access to emergency contraception is a stance more commonly supported by liberal ideologies, which tend to advocate for reproductive rights and access to a full range of family planning services.<br><br>2. **Comprehensive Sexual Health Services**: The call for a comprehensive approach to sexual health, including prophylactic treatment for sexually transmitted diseases, reflects a public health perspective that prioritizes access to healthcare services without moral judgment, a position more typically associated with liberal viewpoints.<br><br>3. **Mental Health Support**: The recognition of the psychological impact of sexual assault, including the provision for the identification and management of posttraumatic stress disorder and prevention of revictimization, aligns with liberal priorities on mental health care and the understanding of the complex needs of survivors.<br><br>4. **Evidence Collection and Preservation**: While the forensic evidence collection can be a non-partisan issue, the context of ensuring it's done in a way that supports the survivor's rights and needs fits within a broader liberal framework of victim support and rights.<br><br>It's important to note, however, that these alignments are not absolute, and there may be individuals and groups within conservative circles who support these measures out of a commitment to healthcare, justice, or victims' rights. The political spectrum is broad, and positions on specific issues like these can sometimes transcend simple liberal-conservative divides. However, based on the general political and social values associated with each side, this text is more aligned with liberal viewpoints due to its emphasis on comprehensive healthcare services, reproductive rights, and victim support without imposing moral or restrictive judgments on the services provided.	liberal

AAFP	Types and Definitions of CME Activities and Credits Members May Earn for AAFP Membership	10/2023	The text you provided outlines the Continuing Medical Education (CME) requirements and activities as recognized by the American Academy of Family Physicians (AAFP). It details the types of activities that qualify for CME credit, differentiating between formal and informal CME, and outlines the credit designation and maximums for various activities. This information is factual and administrative in nature, focused on the educational standards and requirements for family physicians to maintain their membership in the AAFP and ensure their medical knowledge remains current.<br><br>The content does not explicitly align with a liberal or conservative viewpoint as it pertains to the structure and requirements of medical education for family physicians rather than political or ideological issues. It focuses on professional development and education in the medical field, which is generally considered a non-partisan topic. The emphasis is on the importance of ongoing learning and improvement in the medical profession to provide comprehensive and effective healthcare.<br><br>Thus, while healthcare and education can be topics of political debate, the specific details and approach outlined in this text are neutral and aimed at promoting professional standards and quality care in family medicine, rather than advocating for a particular political or ideological stance.	neutral
AAFP	Urban Training Program in Family Medicine	1/2022	The text you've provided outlines criteria for an "Urban Training Program in Family Medicine." It does not explicitly align with a liberal or conservative viewpoint in the political sense. Instead, it focuses on educational and training standards aimed at preparing medical professionals to serve in urban environments, particularly underserved communities. Here are some considerations:<br><br>1. **Focus on Underserved Communities**: The emphasis on serving urban underserved populations, along with the requirement for a mission statement dedicated to this cause, could be interpreted as aligning with more progressive or liberal values. This is because there is a focus on equity and access to healthcare, which are often emphasized in liberal policy discussions.<br><br>2. **Culturally Effective, Community-Responsive Care**: The criteria for training that is culturally effective and community-responsive, along with experiences in diverse settings like community health centers, homeless shelters, and HIV/AIDS clinics, suggest a holistic approach to healthcare. This aligns with a view that healthcare should be inclusive and tailored to meet the diverse needs of the population, which can also be seen as a more progressive stance.<br><br>3. **Comprehensive Healthcare Training**: The inclusion of structured educational experiences in areas such as occupational health, adolescent medicine, mental illness care, and substance abuse treatment indicates a comprehensive approach to healthcare education. This broad view of healthcare, emphasizing the need to address a wide range of health issues, might not strictly align with traditional conservative or liberal viewpoints but suggests a progressive approach to medical education.<br><br>4. **Urban Focus**: The definition of urban metropolitan areas and the flexibility in meeting the training criteria in smaller metropolitan areas do not inherently align with a political viewpoint but rather address practical considerations in healthcare education and delivery.<br><br>In summary, while the text itself is focused on educational criteria and does not explicitly endorse a political ideology, some of the values it promotes—such as a focus on underserved communities, inclusivity, and a comprehensive approach to healthcare—might resonate more with ideologies typically associated with progressive or liberal viewpoints. However, it's important to recognize that healthcare and education in serving underserved communities can and do cross political lines, with individuals from various political backgrounds supporting efforts to improve access to and quality of healthcare.	probably liberal

AAFP	Use of Face Masks During the COVID-19 Pandemic	9/2022	The text provided outlines a public health stance on the use of face masks to prevent the transmission of SARS-CoV-2, the virus responsible for COVID-19. It emphasizes the effectiveness of high-quality masks like KN95/KF94 and N95 respirators, while also acknowledging that cloth and surgical masks offer more protection than not wearing any mask at all. This stance is based on evidence demonstrating the role of masks in preventing the spread of respiratory diseases.<br><br>The viewpoint expressed in the text aligns more closely with what is generally considered a liberal perspective, particularly in the context of the United States' political and cultural discourse around COVID-19. During the pandemic, public health measures such as mask mandates and recommendations have often been more strongly advocated for by individuals and groups on the liberal side of the political spectrum. These measures have been part of a broader public health approach emphasizing collective action and compliance with scientific recommendations to manage the pandemic.<br><br>Conservative viewpoints have varied, with some conservatives supporting mask use and others opposing mandates or questioning the efficacy of masks, often emphasizing personal freedom, individual choice, and skepticism of government mandates or interventions.<br><br>It's important to note that public health recommendations are based on scientific evidence and are intended to protect public health regardless of political affiliations. The perception of these measures as aligning with liberal or conservative viewpoints is more reflective of the political climate and public discourse than of the scientific evidence itself.	liberal
AAFP	Value-Based Insurance Design	1/2022	The text describing Value-based Insurance Design (VBID) and the principles supported by the American Academy of Family Physicians (AAFP) does not strictly align with either a traditional liberal or conservative viewpoint as understood in the context of U.S. politics. Instead, it reflects a policy approach aimed at improving healthcare efficiency and quality based on evidence-based practices. However, different aspects of the text can be interpreted as aligning with values commonly associated with both political ideologies, depending on how one views the role of government, the market in healthcare, and the emphasis on evidence-based decision-making.<br><br>1. **Liberal Viewpoint Alignment**: The emphasis on reducing financial barriers to high-value healthcare services, ensuring transparency in coverage and cost-sharing policies, and the focus on evidence-based data to support healthcare decisions align with liberal principles that prioritize equitable access to healthcare and the government's role in regulating and ensuring quality in healthcare services. Liberals typically support policies that aim to make healthcare more accessible and affordable, especially for underprivileged populations, and might view VBID as a tool to achieve these goals.<br><br>2. **Conservative Viewpoint Considerations**: The principles of VBID also touch on elements that conservatives might support, such as the involvement of practicing physicians in the development of VBID programs, the emphasis on flexibility in the design and implementation of VBID programs, and the encouragement of innovations in Medicare Advantage and Medicaid managed care plans. These aspects can be seen as aligning with conservative values that emphasize the role of personal responsibility, market-based solutions to healthcare, and limiting the government's role in healthcare decision-making.<br><br>3. **Bipartisan Appeal**: VBID's focus on value, efficiency, and evidence-based care has the potential for bipartisan support. It aims to improve healthcare outcomes while also addressing cost-effectiveness, appealing to both those who prioritize healthcare access and quality (a concern often associated with liberal politics) and those who are focused on reducing wasteful spending and improving efficiency in healthcare delivery (a concern often associated with conservative politics).<br><br>In summary, while the VBID framework and the AAFP's principles on its implementation incorporate aspects that could be appealing to both liberal and conservative viewpoints, they are primarily grounded in healthcare policy and management principles aimed at improving quality, accessibility, and efficiency in healthcare. The approach is more about practical healthcare delivery improvements than fitting neatly into the ideological spectrum of liberal vs. conservative politics.	neutral

AAFP	Violence (Position Paper)	7/1905	The text provided focuses on a comprehensive understanding and approach to addressing violence and its health impacts, which aligns with themes commonly found in both liberal and conservative viewpoints, albeit in different aspects. Here's a breakdown of how different parts of the text might be seen through both lenses:<br><br>1. **Understanding and Addressing Violence Across All Groups:** The text emphasizes that violence affects individuals across all economic, racial, religious, or other groups, acknowledging the universal nature of this issue. This inclusive approach is more closely aligned with **liberal viewpoints**, which often emphasize equality, inclusivity, and the societal structures contributing to violence.<br><br>2. **Evidence-based Approaches to Violence:** The emphasis on using a growing body of evidence to link forms of violence with adverse health effects and on promoting trauma-informed care aligns with a **liberal perspective**, which typically values scientific evidence and comprehensive healthcare approaches.<br><br>3. **Social Determinants of Health:** The discussion about the ecological framework in which violence occurs, including social, economic, and cultural risk factors, reflects a **liberal viewpoint** that considers broader societal factors as key components of individual health outcomes.<br><br>4. **Empowerment and Harm Reduction:** Strategies aimed at empowering patients and promoting harm-reduction strategies can be seen in both liberal and conservative views. However, the focus on empowerment and addressing the root causes of violence, including providing resources for vulnerable populations (e.g., LGBTQ+, adolescents, immigrants), tends to be more strongly emphasized in **liberal policies**.<br><br>5. **Policy Engagement and Community Resources:** The encouragement for family physicians to engage in policy-making and to connect with community resources reflects a proactive, community-oriented approach to health and violence prevention. While both liberals and conservatives might support community engagement, the focus on policy influence and comprehensive community resources aligns more closely with a **liberal viewpoint** that advocates for systemic change to address social issues.<br><br>6. **Prevention and Education:** The focus on prevention, education about healthy relationships, and the role of family physicians in addressing violence through primary care settings can find support across the political spectrum. However, the comprehensive approach to prevention and the emphasis on education and societal involvement might lean towards a **liberal perspective**, which often advocates for preventive measures and education as tools for social change.<br><br>In summary, while the text contains elements that could be supported by both liberal and conservative viewpoints, its comprehensive approach to understanding violence, emphasis on societal and structural factors, focus on inclusivity and empowerment, and advocacy for policy engagement and community resources align more closely with liberal perspectives on addressing social issues and health disparities.	liberal
AAFP	Violence as a Public Health Concern	10/2023	The text you've provided outlines the American Academy of Family Physicians (AAFP)'s position on violence as a major public health concern and outlines their approach to addressing this issue through education, advocacy, and partnership with law enforcement and community organizations. This position, focusing on the social determinants of health, the disproportionate impact of violence on vulnerable populations, and the call for proactive involvement in violence prevention, aligns more closely with viewpoints traditionally associated with liberal ideologies.<br><br>Liberal viewpoints often emphasize the role of social, economic, and environmental factors in affecting individual health and well-being and advocate for comprehensive, inclusive approaches to addressing public health issues. The emphasis on the broad range of human relationships, complex interactions, and the specific mention of vulnerable populations such as women, children, and LGBTQ+ individuals, as well as the call for advocacy, education, and community partnership, are indicative of a holistic approach to health and well-being that is often associated with liberal perspectives.<br><br>Conservative viewpoints, while also concerned with public health, might focus more on individual responsibility, the role of family and traditional social structures, and may be less inclined to emphasize systemic or structural factors such as the influence of media, substance use, and availability of weapons in the same way. While conservatives certainly recognize violence as an issue, the approach to addressing it might differ, with possibly more emphasis on law enforcement and criminal justice solutions rather than broad-based public health interventions.<br><br>It's important to note that public health is a field that ideally transcends political ideologies, aiming to find effective solutions to	liberal

			health issues that affect all members of society. However, the strategies and emphases chosen can reflect broader ideological perspectives.	
AAFP	Violence in the Media and Entertainment (Position Paper)	1/2022	The text you've provided does not strictly align with either a liberal or conservative viewpoint in a direct political sense. Instead, it presents a comprehensive overview of concerns regarding the impact of media violence on public health, particularly among children and adolescents. These concerns are based on a broad range of studies and reports from reputable organizations like the World Health Organization, the U.S. Surgeon General, and the Children’s Defense Fund, among others.<br><br>However, the emphasis on public health implications of media violence, the call for action in terms of education, parental guidance, and regulation, and the focus on societal and community responsibilities might resonate more with viewpoints typically associated with liberal perspectives. This is because liberal viewpoints often prioritize public health initiatives, advocate for regulatory measures to protect vulnerable populations (especially children), and support the role of government and community organizations in addressing social issues.<br><br>Conversely, conservative viewpoints might be more focused on issues related to media censorship, the protection of Second Amendment rights in the context of discussions about gun violence, and a preference for less regulatory interference in media and entertainment industries. Yet, concerns about the impact of media violence on society and the emphasis on family values and parental responsibility in monitoring and guiding children's media consumption could find agreement across a broad political spectrum.<br><br>Ultimately, the text underscores a non-partisan issue – the impact of media violence on public health – that could be addressed through a variety of interventions, including education, advocacy, and policy reform, appealing to concerns common to both liberal and conservative perspectives in different ways.	neutral
AAFP	Violence, Harassment and Bullying	1/2022	The text you've provided outlines a stance against violence, harassment, and bullying, emphasizing the importance of protecting individuals across a wide range of identities. This stance advocates for inclusivity and the protection of diverse groups based on race, ethnicity, socioeconomic status, religion, sexual orientation, gender identity and expression, physical status or ability, political affiliation, immigration status, disability, or other personal characteristics.<br><br>This viewpoint aligns more closely with liberal or progressive ideologies, which generally prioritize social justice, equity, and the protection of individual rights, especially for marginalized and vulnerable groups. Liberal perspectives often advocate for policies and practices that seek to address and prevent discrimination and violence against individuals based on their identity markers.<br><br>Conservative viewpoints might also oppose violence, harassment, and bullying, but the emphasis on a broad range of identities and the explicit mention of categories like sexual orientation, gender identity and expression, and immigration status as protected characteristics is more characteristic of liberal ideologies. Conservative perspectives might focus more on traditional values or approaches to these issues and might not emphasize the protection of such a wide range of identities to the same extent.<br><br>However, it's important to note that opposition to violence, harassment, and bullying is a universal value that can be found across the political spectrum, even if the approaches to addressing and framing these issues might differ.	liberal

AAFP	Violence, Illegal Acts Against Health Care Professionals	10/2023	The text you provided outlines a stance on violence against health care professionals, emphasizing condemnation of violence, support for legal enforcement against such acts, and the importance of security and preparedness within health care facilities. This position primarily focuses on safety, legal enforcement, and professional-community relations rather than explicitly aligning with traditional liberal or conservative viewpoints. However, the emphasis on strict enforcement of laws against violence and the call for felony charges for crimes against health care workers can be interpreted through different political lenses:<br><br>- **Liberal viewpoint:** From a liberal perspective, the emphasis might be appreciated for its focus on protecting workers' rights and ensuring a safe working environment, which aligns with broader liberal values of worker protection and safety regulations. However, liberals might also focus on the root causes of violence and advocate for comprehensive strategies that include mental health support, community engagement, and preventive measures alongside law enforcement.<br><br>- **Conservative viewpoint:** Conservatives might focus on the call for prompt enforcement of laws and the classification of violent crimes as felonies, which aligns with conservative values of law and order and strict penalties for crimes. The emphasis on building relationships with law enforcement and ensuring security protocols are in place within health care facilities also resonates with conservative priorities around safety and crime prevention.<br><br>In essence, while the text does not neatly fit into a single political ideology, elements of it can be appreciated or critiqued from both liberal and conservative perspectives. It underscores a universally shared value across the political spectrum: the importance of protecting health care professionals and ensuring their safety in the workplace.	neutral
AAFP	Virtual e-Visits	5/2023	The text you've shared outlines the American Academy of Family Physicians (AAFP)'s support and guidelines for virtual e-visits between patients and healthcare providers. It emphasizes the importance of secure, online communication systems for these interactions, establishes criteria for when and how they should occur, and advocates for their recognition as a payable service by healthcare systems.<br><br>This text, in itself, is primarily focused on healthcare policy and the practical aspects of implementing telehealth solutions rather than aligning directly with traditional political ideologies such as liberal or conservative viewpoints. However, its implications can be interpreted differently depending on one's political or ideological perspective:<br><br>1. **Liberal Viewpoint Alignment**: From a liberal standpoint, the support for virtual e-visits could be seen as aligning with a desire for increased access to healthcare services, leveraging technology to make healthcare more accessible and efficient, and potentially reducing healthcare costs. Liberals often advocate for healthcare reform that expands access to care, and telehealth can be a component of such reform. The emphasis on privacy, security (e.g., HIPAA compliance), and the patient's consent may also resonate with liberal values related to individual rights and protections.<br><br>2. **Conservative Viewpoint Considerations**: From a conservative perspective, the emphasis on technology and innovation in healthcare delivery could be seen as positive, particularly if it leads to cost savings and increased efficiency within the healthcare system without expanding government control. Conservatives may appreciate the focus on maintaining the standard of care and the potential for telehealth to enhance the free market aspects of healthcare by providing more options for consumers. However, conservatives might be cautious about aspects that could lead to increased regulatory oversight or mandates on healthcare providers.<br><br>Ultimately, the text reflects a policy position focused on improving healthcare delivery through technology. Its alignment with liberal or conservative viewpoints might depend on the broader context of how it fits into healthcare reform discussions, individual rights and privacy concerns, and the balance between innovation, regulation, and market forces in healthcare.	neutral

AAFP	Visa (J-1) Fast Track	7/1905	The statement that the American Academy of Family Physicians (AAFP) supports a J-1 Visa fast track process similar to the Premium Processing service available to H-1B Visa applicants reflects a viewpoint that is more aligned with liberal immigration policies, which typically advocate for more accessible and efficient immigration processes. This position supports easing the process for medical professionals to work in the United States, which can be seen as part of broader efforts to address healthcare worker shortages and ensure access to medical care.<br><br>Liberal viewpoints generally favor more open immigration policies and the removal of unnecessary barriers to immigration, especially for individuals who can contribute to sectors with significant needs, such as healthcare. By advocating for a fast track process, the AAFP is emphasizing the importance of bringing in healthcare professionals to meet the demand for medical services, which aligns with liberal priorities of addressing public health needs and supporting diversity in the workforce.<br><br>Conservative viewpoints, on the other hand, may emphasize stricter immigration controls and more rigorous processes for entering the country. However, it's important to note that there can be bipartisan support for specific policies that address critical workforce shortages in areas like healthcare, making such positions not strictly partisan.<br><br>The AAFP's support for a streamlined visa process for medical professionals is a policy position focused on healthcare delivery and workforce issues rather than a clear-cut alignment with the broader ideological spectrum. However, in the context of immigration policy, it leans more towards a liberal viewpoint due to its emphasis on facilitating immigration for skilled workers.	liberal
AAFP	Visa (J-1) Waiver Program	5/2023	The text you've provided outlines a position from the American Academy of Family Physicians (AAFP) regarding the training and distribution of physicians both within the United States and from other countries, specifically through the use of J-1 visa waivers for physicians to serve in rural and underserved communities. This stance focuses on addressing healthcare disparities and ensuring adequate healthcare services in areas with less access to medical professionals.<br><br>This perspective can be seen as aligning more closely with liberal viewpoints for several reasons:<br><br>1. **Emphasis on Healthcare Accessibility**: Liberal ideologies often prioritize the expansion of healthcare services to ensure that all individuals, regardless of where they live or their economic status, have access to healthcare. The AAFP's statement supports the idea of increasing healthcare access in rural and underserved areas.<br><br>2. **International Cooperation and Inclusion**: The mention of providing training opportunities for physicians from other countries and recognizing the role of J-1 visa waivers highlights a more inclusive, global approach to healthcare. This aligns with liberal tendencies towards internationalism and the belief in the benefits of diversity and cross-cultural collaboration.<br><br>3. **Addressing Disparities**: The focus on improving healthcare disparities is a key concern of liberal policies, which often seek to address and reduce inequalities across various sectors, including healthcare. The AAFP's position underscores the importance of targeted efforts to improve health outcomes in communities that are traditionally underserved.<br><br>However, it's important to note that improving healthcare accessibility and addressing disparities can also be supported by individuals with conservative viewpoints, especially when such measures are seen as ways to enhance community well-being and efficiency in healthcare delivery. The distinction often lies in the methods and policies proposed to achieve these goals.<br><br>In summary, while the AAFP's position as described may align more closely with liberal viewpoints due to its emphasis on accessibility, inclusion, and addressing disparities, aspects of this stance can also resonate with conservative values that prioritize community health and efficient resource use. The interpretation can vary depending on the broader context of the policies and values of the individual assessing the statement.	liberal

AAFP	Vision and Principles of a Quality Measurement Strategy for Primary Care (Position Paper)	2019	The text provided outlines a comprehensive vision and principles for improving quality measurement in primary care, under the guidance of the American Academy of Family Physicians (AAFP). It does not strictly align with traditional liberal or conservative political viewpoints but rather focuses on health policy and quality improvement within the healthcare system. However, elements of the text could be interpreted through the lens of broader ideological perspectives commonly associated with these political orientations.<br><br>1. **Focus on Reducing Administrative Burden and Improving Patient Care**: The emphasis on reducing the administrative burden on physicians, especially primary care physicians, and improving patient care through more effective and meaningful quality measures aligns with a general liberal viewpoint that advocates for the well-being of healthcare providers and patients. This includes addressing social determinants of health (SDoH) and advancing health equity, which are topics often emphasized by liberal perspectives.<br><br>2. **Value-Based Payment Critique**: The critique of current value-based payment systems and the call for measures that truly reflect improvements in patient care over financial concerns might resonate more with liberal viewpoints, which often stress the importance of patient-centered care over profit. However, the acknowledgment of the need for value-based payment and performance measures could also align with conservative views on efficiency and accountability in healthcare spending.<br><br>3. **Technology and Data Use**: The advocacy for the principled redesign of health information technology to support quality improvement and the use of data for better healthcare outcomes can be seen as a technologically progressive stance that does not inherently align with a specific political ideology. Both liberal and conservative perspectives might support the integration of technology and data in healthcare, albeit with different emphasis on regulation, privacy concerns, and the role of government versus the private sector.<br><br>4. **System-Level Performance Measures and Shared Accountability**: The push for system-level performance measures and shared accountability in healthcare outcomes can be interpreted as a call for collective action and cooperation within the healthcare system, which is more closely aligned with liberal ideologies emphasizing community and systemic solutions. However, the focus on efficiency, cost reduction, and better health outcomes could also find support within conservative viewpoints that prioritize fiscal responsibility and the reduction of unnecessary governmental intervention.<br><br>In summary, the text primarily focuses on healthcare quality improvement and does not neatly fit into a liberal or conservative viewpoint. Instead, it presents a blend of ideas that emphasize patient care, efficiency, and the use of technology and data, which could garner support across the political spectrum, reflecting a more centrist or pragmatic approach to healthcare policy.	neutral
AAFP	Women's Health Care	7/1905	The statement from the American Academy of Family Physicians (AAFP) regarding the importance of a sufficient family physician workforce to meet public needs for appropriate women's healthcare doesn't inherently align with a strictly liberal or conservative viewpoint on its own. It expresses a position on healthcare provision, emphasizing the need for accessible and adequate healthcare services for women, which is a concern that crosses political boundaries.<br><br>However, perspectives on how to achieve adequate healthcare, the role of government in healthcare, and specific healthcare policies can vary significantly between liberal and conservative viewpoints. Liberals might interpret this statement as supportive of policies that ensure universal healthcare access, including comprehensive women's health services. Conservatives might agree with the necessity of adequate women's healthcare but may differ on the methods to achieve it, possibly advocating for market-driven solutions or limited government intervention.<br><br>The focus of the AAFP statement is on the importance of a capable workforce in family medicine to address women's health needs, which is a practical concern rather than a politically charged one. Yet, how this goal aligns with broader healthcare policy can be interpreted differently depending on one's political or ideological leanings.	neutral
AAFP	Women's Health Care, Family Physicians Providing	2020	The statement about family physicians being well-trained, qualified, and involved in providing comprehensive, continuing care of women throughout their lifecycle does not inherently align with a specific political viewpoint, whether liberal or conservative. It is a factual statement regarding the role and capabilities of family physicians in healthcare, particularly in the context of women's health.<br><br>Political viewpoints might diverge more on how healthcare is provided, funded, or accessed rather than on the qualifications of healthcare providers themselves. For example, debates between liberal and conservative perspectives might focus on issues like public vs. private healthcare funding, the extent of government involvement in healthcare, or specific healthcare policies such as	neutral

			reproductive rights. However, the acknowledgment of the professionalism and importance of family physicians in providing comprehensive care is a matter of professional consensus and medical practice standards rather than political ideology.	
AAFP	Women's Health Specialty	2020	The statement you've shared from the American Academy of Family Physicians (AAFP) regarding their support for excellence in healthcare for women, but opposition to the creation of a separate medical specialty or subspecialty in women's health, reflects a position primarily centered on healthcare policy and professional standards rather than a clear alignment with liberal or conservative political viewpoints. Here's why:<br><br>1. **Healthcare Policy and Professional Standards**: The AAFP's position is more about how healthcare should be organized and delivered to ensure the best outcomes for women. They support high standards in women's health but believe that creating a separate specialty may not be the best approach. This stance is grounded in considerations about healthcare delivery, professional training, and resource allocation within the medical field.<br><br>2. **Cross-partisan Relevance**: Issues of healthcare organization, efficiency, and effectiveness can be of concern to individuals across the political spectrum. Conservatives might agree with the stance if they view it as a means to prevent unnecessary expansion of medical bureaucracy or to maintain a holistic approach to family medicine. Liberals might agree if they see it as ensuring comprehensive care without segregating women's health from the broader healthcare system.<br><br>3. **Complexity of Healthcare Views**: Both liberal and conservative viewpoints can support a wide range of positions on healthcare organization and specialization. Some liberals might prefer specialized care for women to address specific health issues more effectively, while some conservatives might support it as a means of improving healthcare outcomes and efficiency. Conversely, members of both groups might oppose it for various reasons, including concerns about cost, accessibility, or the fragmentation of care.<br><br>The AAFP's position is primarily a professional and policy-oriented one, focusing on what they believe is the best approach to providing healthcare to women within the context of family medicine. While different political groups might find reasons to support or oppose this stance based on their broader healthcare philosophies, the position itself does not neatly align with a liberal or conservative viewpoint without considering additional context about those viewpoints' priorities and concerns in healthcare.	neutral

AAFP	Workforce Reform	2023	The text presents viewpoints that align more closely with liberal perspectives, especially in the context of health care reform in the United States. Key aspects that highlight this alignment include:<br><br>1. **Emphasis on Equity and Diversity**: The focus on recruiting physicians from underrepresented communities and ensuring that the medical workforce reflects the diversity of the population is a principle more commonly associated with liberal ideologies, which prioritize diversity and equity in all sectors, including healthcare.<br><br>2. **Reform in Medical Education and Training**: Advocating for significant reforms in medical education and training to address primary care shortages, especially in rural and underserved areas, aligns with liberal priorities on improving access to healthcare for all, regardless of socio-economic status.<br><br>3. **Financial Support and Debt Relief for Medical Students**: The support for policies that decrease the cost of medical education, reduce student debt, and address the pay disparity between primary care and other medical specialties echoes liberal views on making education more accessible and addressing economic barriers to career choices.<br><br>4. **Accountability for Medical Schools and Residency Programs**: The call for medical schools and residency programs to be accountable in addressing community health needs and health inequities reflects a liberal approach to healthcare reform, emphasizing the role of educational institutions in societal well-being.<br><br>5. **Broad and Adaptive Scope of Practice**: Supporting a broad and adaptive scope of practice for family physicians, protecting it from limiting systemic forces, and promoting equitable care for underserved groups align with liberal views on expanding healthcare access and services to meet community needs.<br><br>Overall, the text advocates for comprehensive healthcare reform with a strong emphasis on equity, diversity, and accessibility, principles that are more commonly associated with liberal ideologies. It suggests systemic changes to improve health outcomes, reduce inequities, and make healthcare more affordable, aligning with the broader liberal agenda of social justice and equal access to essential services.	liberal
