# Supplementary material for: Analysis of the Political Viewpoint of Policy Statements From Professional Medical Organizations Using ChatGPT With GPT-4: Cross-Sectional Study
Source: JMIR Form Res. 2025 Jun 13;9:e66204. doi: 10.2196/66204 (PMC12205260; doi:10.2196/66204)
Supplement: Multimedia Appendix 2 [file formative_v9i1e66204_app2.docx]

Examples of liberal policy statements with part of their ChatGPT output. The full output is available in Multimedia Appendix 2.

| Medical organization and policy statement title (date) | | Portion of ChatGPT output | Political category (topic) |
| --- | --- | --- | --- |
| **AAP^a^** | | |  |
|  | Global Climate Change and Children’s Health (November 1, 2015) | - “The text you’ve provided aligns more closely with a liberal viewpoint. It emphasizes the scientific consensus on human-driven climate change, highlights the vulnerability of children to climate change impacts, and advocates for substantial action to mitigate these effects.” | Liberal (climate and environment) |
|  | Detention of Immigrant Children (May 1, 2017) | - “The text you provided focuses on the treatment and care of immigrant children, particularly those who are unaccompanied or in family units, and emphasizes the importance of their health and well-being. This perspective aligns more closely with liberal viewpoints for several reasons.” | Liberal (immigration) |
|  | Firearm-Related Injuries Affecting the Pediatric Population (November 1, 2012) | - “The text you provided aligns more closely with a liberal viewpoint. It emphasizes the need for gun control, the dangers of firearm availability, particularly in homes with children and adolescents, and supports specific measures such as the regulation of firearms, a ban on semiautomatic assault weapons, and strong regulations on handguns for civilian use.” | Liberal (firearms) |
| **ACS^b^** | | | |
|  | Statement on Supreme Court Decision in Dobbs v. Jackson Women’s Health Case (June 24, 2022) | - “In summary, the ACS’s statement reflects a liberal viewpoint through its emphasis on personal and professional autonomy in healthcare, support for reproductive services, and advocacy for evidence-based, accessible healthcare for all.” | Liberal (reproductive rights) |
|  | Diversity (January 1, 2018) | - “1. **Emphasis on Diversity and Multiculturalism**: The text highlights a strong commitment to multiculturalism and diversity. Liberal viewpoints typically stress the importance of including people from diverse backgrounds and ensuring equal opportunities for all, regardless of their race, ethnicity, or other demographic characteristics.” - “Active Promotion of Inclusion in Leadership**: The mention of ensuring that leadership positions are held by individuals from diverse groups aligns with a liberal perspective, which often advocates for representation and inclusivity in decision-making roles.” - “Focus on Underrepresented Groups**: The acknowledgment of the need for specific recruitment and development of individuals from underrepresented groups is a hallmark of liberal approaches, which prioritize social justice and equity.” - “Merit-based Nominations with an Inclusive Approach**: The combination of basing nominations on individual qualifications and the active effort to include diverse backgrounds is reflective of a liberal viewpoint that seeks to balance meritocracy with inclusivity.” | Liberal (social justice) |
| **APA^c^** | | | |
|  | Position Statement on Police Brutality and Black Males (December 2018) | - “The text’s focus on systemic issues, the impact of police brutality on mental health, and the call for broad reforms and collaborative efforts between law enforcement and mental health professionals is more aligned with liberal ideologies that prioritize systemic change and address issues of racial inequality and justice.” | Liberal (criminal justice) |
|  | Position Statement on Affirmative Action (July 2017) | - “The position described in the text aligns more closely with a liberal viewpoint. It emphasizes affirmative action, the recruitment and training of minority psychiatrists, and the recognition of the special mental health needs of minority populations. These priorities are typically associated with liberal ideologies, which often prioritize diversity, equity, and inclusion in various sectors, including healthcare and education.” | Liberal (social justice) |
|  | Position Statement on Issues Related to Sexual Orientation and Gender Minority Status (July 2020) | - “The text provided aligns more closely with a liberal viewpoint. This assessment is based on the emphasis on inclusivity, support for diverse sexual orientations and gender identities, opposition to practices like conversion therapy, and advocacy for equal rights in various aspects of life such as employment, military service, and family rights.” | Liberal (social justice) |
| **ACOG^d^** | | | |
|  | Racism in Obstetrics and Gynecology (February 2022) | - “The text provided primarily aligns with a liberal viewpoint. It emphasizes the social construction of race, acknowledges systemic racism and its impact on health disparities, and calls for systemic changes to address these issues. This approach is generally associated with liberal or progressive ideologies, which tend to focus on social justice, systemic inequalities, and the need for active reform to address historical and ongoing injustices.” | Liberal (racial equity) |
|  | Opposition to Criminalization of Individuals During Pregnancy and the Postpartum Period (December 2020) | - “The text presents a viewpoint that aligns more closely with liberal principles. It emphasizes the importance of patient autonomy, bodily autonomy, and opposes criminalizing behaviors during pregnancy, which are typically associated with progressive or liberal stances on healthcare and reproductive rights. The stance against punitive measures for pregnant individuals, especially concerning substance use and refusal of care, reflects a focus on public health, social justice, and individual rights over strict legal enforcement, which are hallmarks of liberal ideology.” | Liberal (criminal justice) |
|  | Abortion Policy (May 2022) | - “The text you’ve provided aligns more closely with a liberal viewpoint. It emphasizes the importance of comprehensive, evidence based health care, which includes access to abortion services. The liberal perspective typically supports the idea of broad access to a range of healthcare services, including reproductive rights and the protection of patient-clinician relationships free from governmental or external interference. The text’s strong opposition to efforts that impede access to abortion, its advocacy for privacy and dignity in healthcare decisions, and its condemnation of stigma and violence against healthcare providers are all stances generally associated with liberal ideologies.” | Liberal (reproductive rights) |
| **ACP^e^** | | | |
|  | Racism and Health: A Position Statement from the American College of Physicians (June 8, 2020) | - “The text primarily aligns with a liberal viewpoint. It emphasizes the condemnation of systemic racism and discrimination, advocating for equitable access to healthcare, and calling for comprehensive reforms in law enforcement practices to ensure equality and reduce violence and discrimination, particularly against Black, indigenous, and other people of color. The focus on social justice, systemic change, and the protection of minority rights are themes commonly associated with liberal ideologies, which typically emphasize equality, civil rights, and the need for systemic reforms to address social inequalities.” | Liberal (racial equity) |
|  | Nuclear Weapons (2016) | - “The text you provided seems to align with a viewpoint that is generally more liberal in nature. It emphasizes the recognition of the threat posed by nuclear weapons and advocates for diplomatic efforts to limit, reduce, and ultimately eliminate these weapons.” | Liberal (foreign policy or national security) |
|  | Environmental Health (2022) | - “**Climate Change and Global Warming**: Acknowledging climate change as a crisis and advocating for immediate action to limit global temperature rise is a position more commonly associated with liberal viewpoints. Conservatives may acknowledge climate change but often differ in the urgency and type of response they support.” - “**Environmental Justice**: The focus on environmental justice and the specific mention of communities of color, low-income groups, and marginalized populations is a hallmark of liberal policies, which often emphasize social equity and addressing disparities.” | Liberal (climate and environment) |
| **AAFP^f^** | | | |
|  | Medical Schools, Underrepresentation In Medicine (2020) | - “The focus on equitable representation, leadership opportunities for those underrepresented in medicine, and the development of specific outreach programs to increase diversity in medical school applicants and matriculants are consistent with liberal efforts to address systemic inequalities and promote inclusiveness. Supporting leadership by family physicians who are underrepresented in medicine, and recommending commitment to the development of medical careers among those underrepresented or underpowered, also aligns with liberal values of diversity and equity.” | Liberal (social justice) |
|  | Policing Standards (January 2022) | - “The text presented aligns more closely with a liberal viewpoint. It emphasizes the need for systemic reforms in policing practices, focusing on reducing harm to Black, Indigenous, and people of color (BIPOC) and other marginalized populations. The document advocates for addressing health inequities, systemic racism, and the negative impacts of policing on community health and well-being. It calls for a shift in policies and procedures within law enforcement agencies to prioritize public health and safety, particularly for BIPOC communities.” | Liberal (crime and criminal justice) |
|  | Hydraulic Fracturing (Fracking): Health Effects and Disclosure of Proprietary Information (January 2022) | - “**Emphasis on Environmental and Public Health Risks:** The text highlights the negative impacts of hydraulic fracturing (fracking) on the environment and public health, including the contamination of water sources and soil, and the association with serious health effects like asthma and cancer. Liberal perspectives often prioritize environmental protection and public health over the interests of industry.” - “Concern for Marginalized Communities: The text notes the disproportionate impact of fracking on marginalized communities, communities of color, and communities with lower incomes. This focus on social justice and equity is a hallmark of liberal ideologies, which advocate for policies that protect vulnerable populations from environmental and economic harms.” | Liberal (climate and environment) |

^a^AAP: American Academy of Pediatrics.

^b^ACS: American College of Surgeons.

^c^APA: American Psychiatric Association.

^d^ACOG: American College of Obstetricians and Gynecologists.

^e^ACP: American College of Physicians.

^f^AAFP: American Academy of Family Physicians.

All 13 conservative or probably conservative policy statements with part of their ChatGPT output. The full output is available in Multimedia Appendix 2.

| Medical organization and policy statement title (date) | | Portion of ChatGPT output | Political category (topic) |
| --- | --- | --- | --- |
| **APA^a^** | | |  |
|  | Position Statement Against the Use of Cannabis for PTSD (July 2019) | “The text you’ve provided appears to align with a more conservative viewpoint. This assessment is based on the following observations:  1. Emphasis on Lack of Credible Studies: the statement highlights the absence of credible studies supporting the clinical effectiveness of medical cannabis for PTSD. This cautious approach to adopting new medical treatments without substantial evidence is often associated with conservative perspectives, which typically emphasize traditional methods and rigorous scientific validation.  2. Non-endorsement of Medical Cannabis: The American Psychiatric Association’s (APA) position of not endorsing medical cannabis for PTSD treatment reflects a conservative stance. Conservative viewpoints often express skepticism or caution regarding the legalization and medical use of cannabis, preferring established treatments and further research.” | Conservative (alternative treatments) |
| **ACP^b^** | | |  |
|  | Provider-Specific Taxes (2015) | “The text you’ve provided seems to align more closely with a conservative viewpoint. It expresses opposition to the idea of levying taxes on professional physician services, whether for funding specific healthcare programs or for general revenue enhancement. Generally, conservative perspectives favor lower taxes and less government intervention in business and professional activities.” | Conservative (service tax) |
|  | Payment Issues (2015) | “The text you provided represents a viewpoint that is generally aligned with conservative principles, particularly in the context of health care policy. This stance emphasizes the autonomy of private entities and professionals in setting their fees, opposing government intervention or influence in these matters. Here’s a breakdown:  1. **Opposition to Government Mandates on Private Payers**: The text’s opposition to legislative proposals that would require private payers to align their payment structures with public programs like Medicare and Medicaid reflects a conservative viewpoint. Conservatism typically advocates for limited government intervention in business and private sector affairs.  2. **Physician Autonomy in Setting Fees**: The emphasis on physicians’ rights to set their charges and enter into contracts with private individuals or payers without restrictions aligns with conservative values of individual freedom and free-market principles.” | Conservative (payment structures) |
|  | Understanding the Fraud and Abuse Laws: Guidance for Internists (2022) | “The text you’ve provided appears to be more aligned with a conservative viewpoint, particularly in its emphasis on reducing regulatory burdens and its concern over the perceived overreach of fraud and abuse laws.  Conservative viewpoints typically favor less government intervention in business and professional practices, arguing that excessive regulation can be counterproductive, stifle innovation, and create unnecessary burdens. The text’s focus on the idea that fraud and abuse laws are an “onerous burden” on practicing internists and create a climate of suspicion aligns with this conservative perspective.  Moreover, the concern that inadvertent billing and coding errors are being treated as fraud suggests a viewpoint that favors protecting professionals from what is seen as overzealous enforcement of regulations. This too is more in line with conservative views that often advocate for a more limited scope of government oversight in professional matters.” | Conservative (fraud and abuse laws) |
|  | Promoting the Leadership Role of Physicians in the Health Care Team (2022) | “The text from the American College of Physicians (ACP) seems to align more closely with a conservative viewpoint in the context of health care policy. It emphasizes the distinct roles and leadership of physicians in healthcare teams, suggesting a preference for traditional, hierarchical models of care delivery. This stance might resonate with conservative values that emphasize respect for established structures and roles.” | Conservative (leadership hierarchy) |
|  | Opposing the Requirement that Hospitals Screen Patients for Methicillin-resistant Staphylococcus aureus (MRSA) (2022) | “In essence, the ACP’s position might be seen as aligning more with a conservative viewpoint on regulatory issues, advocating for professional discretion over government mandates. However, it’s important to note that many factors influence stances on healthcare policies, including scientific evidence, ethical considerations, and practical implications, not just political ideologies.” | Probably conservative (health care regulation) |
|  | Prohibit States from Tampering with Federally Funded Health Programs in Which States Have Not Contributed (2015) | “2. **Conservative Viewpoint**: Conservatives typically favor less government intervention in healthcare and more market-driven solutions. They also often support states’ rights over federal control. In this context, the ACP’s position might align more with conservative principles, as it opposes state mandates regarding Medicare assignment, advocating for less government intervention at the state level.” | Probably conservative (states’ rights) |
| **AAFP^c^** | | |  |
|  | Credentialing and Privileging Decisions of Family Physicians by Non-Physician Practitioners (2020) | “The text reflects a conservative viewpoint towards healthcare professional roles and scope of practice. It emphasizes a traditional hierarchy in medical practice, advocating for the authority and expertise of residency-trained, board-certified family physicians over nonphysician practitioners.” | Conservative (leadership hierarchy) |
|  | Nurse Midwives, Certified (2020) | “The text reflects a viewpoint that emphasizes regulation, oversight, and integration within the existing medical system for certified nurse midwives (CNMs). It advocates for specific training, certification following an RN license, and the functioning of CNMs under the supervision of licensed physicians. Furthermore, it supports quality assurance in training programs and advocates for payment for CNM services within an integrated practice arrangement.  This stance aligns more closely with a perspective that values structured oversight, professional standards, and integration within traditional health care frameworks, which can be seen as conservative in the context of health care policy.” | Conservative (professional standards) |
|  | Nurse Practitioners (2020) | “The text reflects a viewpoint that leans more towards what might be traditionally seen as conservative in the context of healthcare policy, specifically regarding the roles and independence of nurse practitioners (NPs) compared to physicians. This perspective emphasizes a structured hierarchy in healthcare, with physicians at the top and nurse practitioners working under their supervision, rather than advocating for the independent practice of NPs.” | Conservative (leadership hierarchy) |
|  | Professional Medical Liability (January 2022) | “The text you’ve provided outlines the goals and methods of the American Academy of Family Physicians (AAFP) concerning professional medical liability and insurance issues. It suggests reforms aimed at reducing the costs associated with professional liability insurance for physicians and ensuring more equitable compensation for patients harmed by medical negligence. The viewpoint expressed in this text can be considered to align more closely with conservative political viewpoints for several reasons.” | Conservative (medical liability) |
|  | Physician and Patient Relationships, Professional Responsibility (2019) | “The text primarily aligns with principles that could be appreciated by both sides for different reasons but leans more towards a conservative viewpoint in its emphasis on the physician’s right to act according to personal judgment and moral principles, even if it means withdrawing from providing certain treatments. This aspect of the statement highlights a conservative inclination towards protecting individual liberties and moral autonomy over a broader mandate for universal access to all forms of medical care regardless of the provider’s personal beliefs.” | Probably conservative (physician autonomy) |
|  | Physician’s Right Relative to Imposed Administrative Costs (2020) | “However, the text primarily leans towards a viewpoint that could be more closely associated with conservative principles of free market operations within the healthcare system, focusing on the rights of healthcare providers to charge for their services in a transparent manner. It doesn’t explicitly address broader healthcare system reform or the impact of these charges on access to care, issues that are often more prominently featured in liberal discussions about healthcare policy.” | Probably conservative (billing autonomy) |

^a^APA: American Psychiatric Association.

^b^ACP: American College of Physicians.

^c^AAFP: American Academy of Family Physicians.
